# Distinct properties of human pathogenic *Candida* species revealed by systematic comparative phenotypic screening of clinical isolates

**DOI:** 10.1128/msystems.00786-25

**Published:** 2025-12-08

**Authors:** Reinhard Beyer, Isabella Zangl, Bernhard Seidl, Ildiko-Julia Pap, Michaela Lackner, Joseph Strauss, Birgit Willinger, Christoph Schüller

**Affiliations:** 1 Institute of Microbial Genetics, University of Natural Resources and Life Sciences, Vienna (BOKU), Tulln, Austria; 2 Core Facility Bioactive Molecules: Screening and Analysis (BMoSA), University of Natural Resources and Life Sciences, Vienna (BOKU), Vienna, Austria; 3 Institute for Hygiene and Microbiology, University Hospital of St. Pölten, St. Pölten, Austria; 4 Institute of Hygiene and Medical Microbiology, Medical University of Innsbruck, Innsbruck, Austria; 5 Bioactive Microbial Metabolites (BiMM), University of Natural Resources and Life Sciences, Vienna (BOKU), Tulln, Austria; 6 Division of Clinical Microbiology, Department of Laboratory Medicine, Medical University of Vienna 27271, Vienna, Austria; Tufts University, Medford, Massachusetts, USA

**Keywords:** *Candida*, fungal pathogens, phenotypes, stress response, population phenotyping

## Abstract

Fungi associated with humans include several *Candida* species that rely on phenotypic plasticity for persistence and pathogenicity. Key adaptive traits, such as adherence, stress resistance, and biofilm formation, enable survival in diverse host niches. However, the degree of intra- and interspecific phenotypic variation across human-associated *Candida* species has not been systematically characterized. We analyzed 1,366 clinical isolates representing 13 *Candida* species using high-throughput quantitative fitness profiling under controlled environmental stressors, antifungal exposure, and biofilm-inducing conditions. The resulting data set revealed both conserved and species-specific adaptive signatures. Isolates consistently segregated into three phenotypic archetypes: heat-resistant fast growers, osmo-sensitive strains, and slow growers. A robust inverse correlation was detected between basal growth rate and stress resistance, reflecting a fundamental physiological trade-off. In addition, distinct resistance profiles against antifungal agents and environmental stressors highlighted species-specific adaptive trajectories and ecological specialization. Despite genetic homogeneity, *C. parapsilosis* isolates displayed striking phenotypic heterogeneity. By contrast, the closely related *C. albicans* and *C. dubliniensis* exhibited divergent stress-response profiles. High-resolution fitness mapping of *C. glabrata* isolates revealed that temperature stress progressively disrupts multiple cellular functions, whereas osmotic stress exerts more discrete, pathway-specific effects. Our systematic phenotypic landscape analysis delineates conserved versus species-specific adaptive properties among human-associated *Candida* species, providing a comparative framework to interrogate evolutionary trends, ecological specialization, and pathogenic potential.

**IMPORTANCE:**

Human-associated fungi include multiple *Candida* species whose persistence relies on phenotypic plasticity enabling adherence, stress resistance, and biofilm formation. Yet, the extent of phenotypic variation within and across species remains poorly defined. We profiled 1,366 clinical isolates from 13 *Candida* species using high-throughput quantitative fitness assays under environmental stress, antifungal exposure, and biofilm-inducing conditions. The analysis uncovered both conserved and species-specific adaptive traits. Isolates segregated into three major phenotypic archetypes: heat-resistant fast growers, osmo-sensitive strains, and slow growers. A consistent inverse correlation emerged between basal growth rate and stress resistance, revealing a fundamental physiological trade-off. Species-specific resistance signatures further reflected ecological specialization and divergent adaptive trajectories. Our quantitative framework establishes, for the first time, a comparative phenotypic landscape across a multispecies collection of human-associated *Candida*, providing new insights into their ecological specialization and adaptive strategies.

## INTRODUCTION

An estimated million fungal species inhabit the biosphere and occupy nearly all ecological niches (1). In association with animal and plant hosts, they may exist as benign colonizers or as serious pathogens. Only a small subset infects humans, causing diseases that range from superficial to life-threatening (2, 3). Nevertheless, these infections impose a considerable burden on healthcare systems (4, 5). Because fungal pathogens are eukaryotes, only a few antifungal drugs target fungus-specific cellular processes (6–8). At least 30 *Candida* species are capable of infecting humans (9, 10), and up to 70% of healthy human adults are carriers (11). Notably, fungal colonization may also confer beneficial effects on mammalian immunity (12, 13). Adaptation to the human host and pathogenicity have arisen independently several times among *Candida* species, and phylogenetic relatedness is a poor predictor of pathogenic potential (14, 15).

*Candida* species colonize several niches of the human body (16) but may also persist in environmental reservoirs. For instance, the five major human-pathogenic *Candida* species have been isolated from natural samples across North America. The case of *Candida glabrata* (currently classified as *Nakaseomyces glabratus*), which appears to be only secondarily associated with humans, suggests that the host is not necessarily the primary ecological niche of fungal pathogens (17, 18). Similarly, *Cryptococcus neoformans* originates from the environment (19), and its pathogenicity is thought to have been shaped in the combat with predatory amoebae (20). Likewise, the emerging drug-resistant pathogenic species *Candidozyma auris* is linked to environmental reservoirs (21). Most species of *Candida* have been isolated from the vagina, with *C. albicans* being by far the most frequently isolated species. Vaginitis is also caused by *C. glabrata* (5%–20%) and less commonly by other non-albicans *Candida* species, such as *C. parapsilosis* complex, *C. tropicalis*, and *Pichia kudriavzevii* (*C. krusei*) (22). *C. albicans* and *C. glabrata* are generally more prevalent at mucosal sites. All these species are isolated from nails, skin, and blood at different frequencies with no strict anatomical site specificity for any single species. *C. dubliniensis* is most commonly associated with oral carriage (23). *C. glabrata* and *P. kudriavzevii* (*C. krusei*) occur significantly more often among the elderly population. *C. parapsilosis* has been associated with skin and intravenous catheters (24). Such diverse environments likely require a high phenotypic flexibility from human-associated *Candida* strains (25).

Phenotypic variation of these fungi is based on a flexible genomic structure, epigenetic regulation, genetic capacitors (like Hsp90), developmental stages, possibly prions, and other traits (3, 26, 27). Molecular typing approaches have revealed a high rate of genetic diversity within clinically important species, such as *C. albicans*, *C. glabrata*, *C. tropicalis*, and *C. lusitaniae* (28, 29). Ploidy changes, aneuploidy, and loss of heterozygosity are rapid and reversible mechanisms for generating genome diversity. They have a major impact on phenotypic properties and drug resistance (30, 31). Karyotypic alterations have been observed in *C. auris* exposed to various stresses (32). Genome rearrangements, including copy number variations, also occur dynamically and expand rapidly during adaptation to antifungal drugs (33).

Sexually reproducing populations maintain genetic and phenotypic diversity (34, 35). Although sexual reproduction may be rare or extremely rare in certain *Candida* species, the capacity for it is widespread among human-associated *Candida* (36, 37). For example, genomic footprints of recombination in *C. glabrata* provide compelling evidence for a sexual cycle (18). In *C. albicans*, genetic diversity arises through a parasexual mating cycle that is induced by the host environment and followed by a concerted loss of chromosomes. Nevertheless, such mating events are rare and depend on specific environmental conditions (38, 39). Genomic plasticity, together with largely clonal asexual reproduction, may enhance genetic drift.

A special case of phenotypic adaptation is the selection of *C. glabrata* mutants resistant to azole antifungals, which leads to the emergence of functional petites and strains that have either lost mitochondrial DNA or harbor a defective respiratory chain (40). Mitochondrial dysfunction, which may be transient, confers cross-resistance to both phagocytic killing and fluconazole (41). However, petite mutants are less virulent *in vivo* despite their selective advantage during azole exposure (42–44). Reversion of petite mutants lacking mitochondrial DNA (such as rho0) is unlikely in the absence of mating. Developmental programs also contribute to phenotypic diversity (45). White-opaque switching in *C. albicans*, *C. dubliniensis*, and *C. tropicalis* represents a programmed and reversible phenotypic transition triggered by environmental cues (46, 47). Interestingly, aged *C. glabrata* cells show increased resilience to neutrophil killing and oxidative stress and adhere more effectively to host cells (48). Thus, we propose that *Candida* species benefit from phenotypic flexibility, which is enabled by diverse mechanisms that generate genetic diversity.

Until now, phenotypic variation both within and between *Candida* species has not been explored systematically on a large scale. Most of the current data on *C. albicans*-host interactions are based on experimental studies with a limited number of strains, primarily on strain SC5314 (49). This strain, in contrast to many other wild isolates, carries a mutation in the RNAi pathway, which has an effect on gene expression in subtelomeric regions (50). *C. albicans* isolates have different pathogenicity properties, which were recognized earlier (51). Phenotyping of *Candida* isolates has either focused on individual species (52–54) or has investigated a few specific phenotypic traits (55), often focusing on antifungal resistance (56, 57). Moreover, methodologies vary between studies. Comparative studies of properties of individual isolates are prone to open new insights such as hidden functional genetic traits. Genetic differences between isolates such as loss of heterozygosity were correlated with fitness (58), adherence, filamentation, and virulence (59). The analysis of several isolates suggested a correlation between virulence and the expression level of immune evasion proteins (60). The natural diversity of *C. albicans* and its impact on host-fungal interactions has been discussed previously (25, 49).

We conducted a systematic and quantitative phenotypic analysis of more than 1,300 isolates representing diverse *Candida* species to assess their phenotypic potential and diversity under various stress conditions, antifungal drug exposure, and during biofilm formation. This work provides a comprehensive phenotypic characterization across a representative set of *Candida* species.

## MATERIALS AND METHODS

### Materials and strains

*C. glabrata* strains (*n* = 224) were obtained from the General Hospital in Vienna, Austria (AKH Vienna). A total of of 1,366 *Candida* strains (various species) were obtained from several different hospitals within Austria (61). All strains were obtained from anonymous patients, purified by streaking to single colonies and stored at −80°C in 30% glycerol.

### Phenotypic screening and growth analysis

For high throughput phenotypic testing, strains were grown on YPD (Yeast Extract, Peptone, 2% Dextrose; Lactan, Austria) agar plates, at 37°C for 1–3 days. A total of 5–10 colonies were picked from each plate and suspended in 1 mL sterile water. For the growth assay in standard 96-well plates (Costar 96 well, Corning Inc., USA), each well contained 180 µL YPD and 20 µL cell suspension to obtain a starting OD6_00nm_ of about 0.1. All growth assays reported here were performed at least in triplicate. Inoculated samples were then incubated at 37°C, except for low and high temperature assays. OD_600nm_ measurements were taken at regular intervals (1–2 h), and growth rate µ [h^−1^] and maximum cell density k [AU] were extracted from the resulting fitted growth curves using a custom R script and the R package “Growthcurver” (62). All growth parameters were referenced to growth performance in YPD at 37°C. Conditions for *C. glabrata* were osmotic stress (0.6, 0.675, 1, 1.5, and 1.9 M NaCl), nitrite (1 mM NaNO2, Merck), oxidative stress (10 mM H2O2, Merck), pH (3.6, 3.4), lactic acid (111 mM), fluconazole (32 mg/l, Avantar/VWR), biofilm, iron starvation (150 µM BPS, Merck), and temperature (15°C, 20°C, 30°C, 33.5°C, 37°C, 39.5°C, 42°C, 57°C, and 50°C).

### Antifungal fitness assay

Antifungal susceptibility testing (AFST) was performed in RPMI medium supplemented with 2% glucose and 0.165 mol*L^−1^ MOPS pH 7 (3-(N-morpholino)-propanesulfonic acid, Sigma), as described previously (63). Briefly, isolates were inoculated and grown at 37°C for 24 h in medium supplemented with eight different antifungals in a 10-step twofold dilution series. All antifungals were (Anidulafungin, Caspofungin, Micafungin, Merck, Germany) and prepared as 200× stock solutions in dimethyl sulfoxide (DMSO). OD_600nm_ measurement was recorded every hour, and growth curve fitting was performed as described above. Growth parameters (µ and k) were transformed into a DRC (dose-response curve), outlining growth performance at different drug concentrations. The area under the curve (AUC) of each dose response curve was extracted and referenced to the no-drug control to obtain the antifungal fitness parameters “µ(Antifungal)” and “k(Antifungal)”, depending on whether growth rate or maximum cell density was used to construct the DRC. AUC mirrors the performance under a broad concentration range on a continuous scale in contrast to the discrete MIC values, which only consider a distinct cut-off. All calculations and data handling steps were performed in R.

### Data analysis and statistics

The phenotypic parameters calculated with the R package “Growthcurver” (62), such as growth rate and maximum growth, were subjected to principal component analysis (PCA) to reduce the complexity of the multidimensional data set, using the packages FactoMineR and FactoShiny (64). Correlation analysis between individual parameters was performed using a Spearman’s rank-order test with a significance threshold of *P* < 0.001. Correlations were classified as weak (0.35 ≥ ρ > 0.2), moderate (0.5 ≥ ρ > 0.35), or strong (ρ > 0.5). To validate correlations, we confirmed that the *P*-value was <0.001, that they also appeared in the factor analysis of the PCA, that they were reflected by corresponding phenotypic clusters, and that observations were consistent within groups of variables. Conditions expected to elicit the same biological response (e.g., different concentrations of NaCl or derivatives of the same antifungal class) were grouped to avoid weighting artifacts. To quantify intraspecies phenotypic variation, we calculated the distances to the respective barycenter, representing the arithmetic mean position in phenotypic space for each group of isolates. PERMANOVA was performed using the *adonis2* function of the Vegan package (version 2.8-0) (65). Silhouette scores were calculated with the Silhouette package (66), and each cluster pair was tested by pairwise PERMANOVA to support the reported number of clusters. Results and values used to generate all figures are provided in Fig. S2 and Tables S1 to S8.

### Biofilm formation assay

For biofilm assays, we adapted a previously described method (67). Cultures were grown overnight in YPD and diluted 1:20 in sterile water. The cell suspension was then dispensed into 96-well round-bottom plates containing 100 µL YNB broth (1.7 g/L Yeast Nitrogen Base [MP Biomedicals, Germany], 2% dextrose) per well. Plates were incubated for 2 h at 37°C and washed twice with sterile PBS (8 g/L NaCl, 0.2 g/L KCl, 1.44 g/L Na_2_HPO_4_, 0.245 g/L KH_2_PO_4_) to remove any non-adhering yeast cells, and 100 µL of fresh YNB broth was added. The plate was sealed (Breathe-Easy membrane, Merck) to reduce evaporation and incubated at 37°C for 48 h. Previous reports showed that this time span is sufficient to evoke biofilm formation of *C. albicans, C. glabrata, C. krusei*, and *C. parapsilosis,* albeit not for all species optimal and thus leading to different biofilm levels (68). Nevertheless, we used one high-throughput protocol to find differences between the isolates. Plates were washed three times with sterile PBS and dried at room temperature. Crystal violet solution (0.5% w/v in distilled water, Merck, Germany) was added. After 1 h incubation at room temperature, excess dye was removed by five washing steps with PBS. After drying at room temperature, 125 µL 30% acetic acid solution was added for 30 min to dissolve the biofilm. The optical density of the supernatant was measured at 550 nm.

### Multi-locus sequence typing (MLST)

MLST is used for typing genetic relationships among microbial strains based on a set of housekeeping genes resulting in distinct allelic profiles designated as sequence type (ST). MLST primer sequences were published for *C. albicans*, *C. glabrata*, *C. krusei*, *C. tropicalis,* and *C. dubliniensis* (69–73). Single colonies grown on YPD were picked into 15 µL GoTaq Green Master Mix (Promega) with primers and amplified by PCR. Forward primers were used for initial sequencing. Single-end sequences of selected loci from 186 isolates were obtained. Uncertain base calls were inspected manually and adjusted according to observed peak heights using FinchTV software. A quality check was performed in R with a mean peak quality cutoff of 15, and samples were repeated if the quality was deemed unsatisfactory. Each sequence was matched to a respective allele published on pubmlst.org. A sequence identity of 100% was required; otherwise, colony PCR and sequencing were repeated using the reverse primer to check the results. Sequences from all loci were concatenated using FABox 1.5 and analyzed using the goeBURST algorithm provided by phyloviz open-source software (74) and MEGA-X (75). All sequences were submitted to the pubmlst.org repository via the respective curators. New alleles are listed in Table S7 and are marked with an asterisk alongside the specific novel SNP.

## RESULTS

### Heat stress reveals phenotypic differences among *C. glabrata* strains

**Fig 1:**
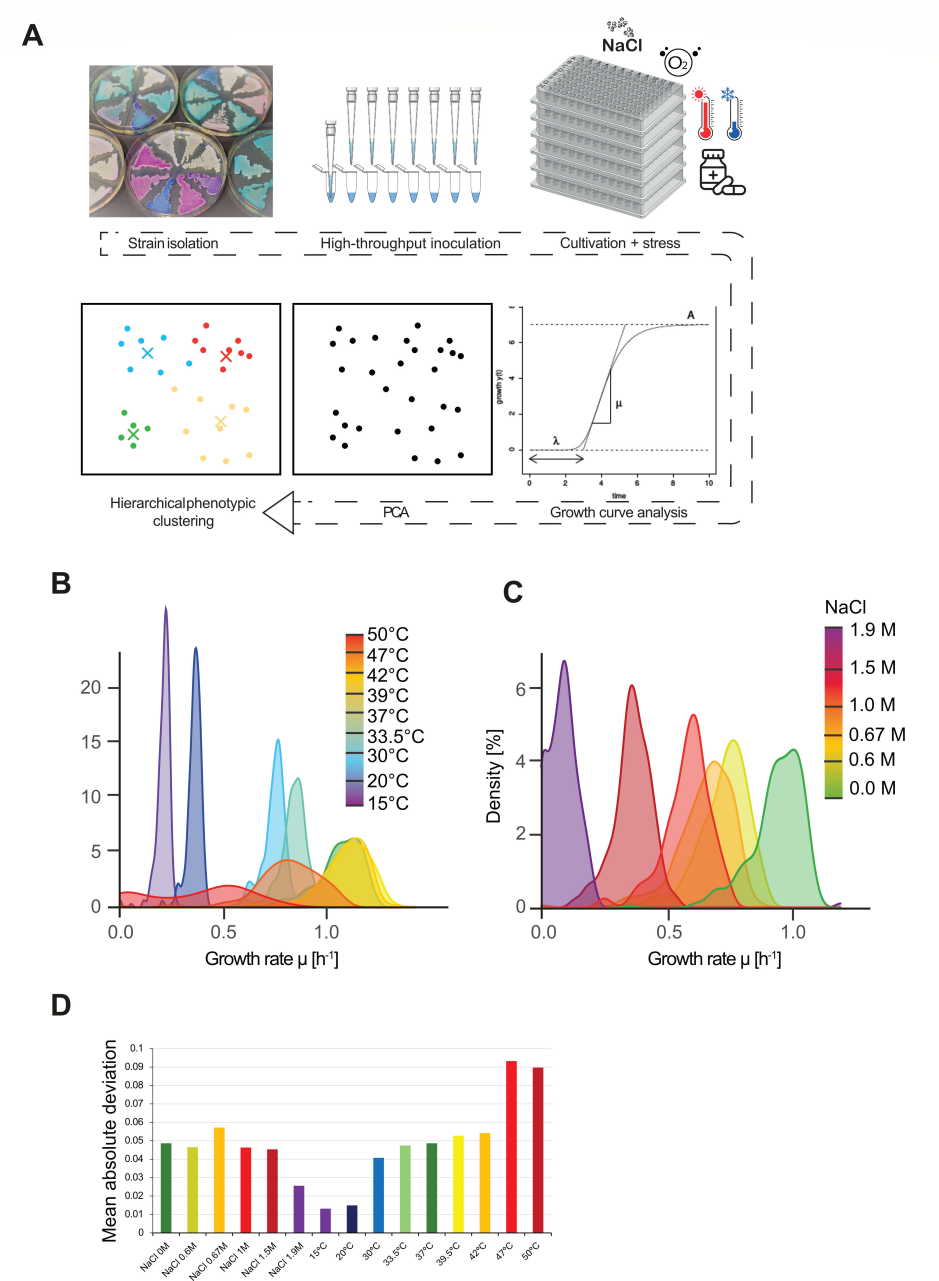
High-throughput phenotypic analysis. Screening steps (**A**). Pipetting steps were performed using a liquid handling robot. Strains were cultivated in 96-well microplates with optical density readings (600 nm) in regular intervals. Data analysis and curve fitting steps were performed in R (tidyverse, FactoMineR, growthcurver) and further with PCA and hierarchical clustering. Density plot of growth rates of clinical *C. glabrata* isolates under (**B**) different temperatures and (**C**) different levels of osmotic stress. Population spread was determined by calculating mean absolute deviation for each condition (**D**).

*C. glabrata* is exposed to both host-associated and environmental niches and proliferates mostly clonally (17). Adaptation to diverse environments may be facilitated by multiple mechanisms, including transient alterations of transcriptional patterns driven by both epigenetic and genetic variation. To examine the degree of phenotypic variation, a collection of clinical isolates was exposed to generic environmental stress to reveal strain-specific phenotypic properties and patterns of phenotypic divergence within the population. Fitness variation of *C. glabrata* isolates was determined with a quantitative method *in vitro* (Fig. 1A). We collected growth curves of each isolate at different incubation temperatures (15°C to 50°C) and a range of osmotic stress conditions using NaCl up to 1.9 M. For high-throughput, we used 96-well microplates and robotic liquid handling to set up and inoculate cultures. Growth parameters of *C. glabrata* in such microcultures (0.2 mL) resemble common batch cultivation in shaking flasks (e.g. µ_max_ ≈ 1 h^−1^) (76). To extract fitness parameters, growth curves were fitted to a logistic model based on periodic measurements of optical density (OD_600nm_) in liquid medium (Table S4). *C. glabrata* isolates proliferated most rapidly in the temperature range of 37°C to 42°C. At lower temperatures, the isolates grew more slowly, and the distribution of growth rates was narrow (Fig. 1B and D). At 15°C, the lowest tested temperature, growth rates were in the range of 0.16 to 0.23 h^−1^. A fraction of the tested *C. glabrata* isolates could grow substantially at temperatures above 45°C. At high temperatures, substantial phenotypic fitness differences between isolates emerged. Severe temperature stress at 47°C and 50°C lead to slower and more distributed growth rates (Fig. 1B and C). Surprisingly, high osmotic stress, although reducing growth rates effectively, produced a different pattern (Fig. 1C and D). Figure 1D shows the mean absolute deviation as a measure for the breadth of the distributions.

In conclusion, we observed that clinical *C. glabrata* isolates exhibit high phenotypic divergence under severe heat stress, whereas their responses remain largely uniform under osmotic challenge. This finding suggests that high osmotic stress does not induce additional nonspecific cellular stress, while elevated temperatures progressively trigger cellular dysfunction, resulting in a broader distribution of individual growth phenotypes.

### *C. glabrata* strains separate into distinct phenotypic clusters

**Fig 2:**
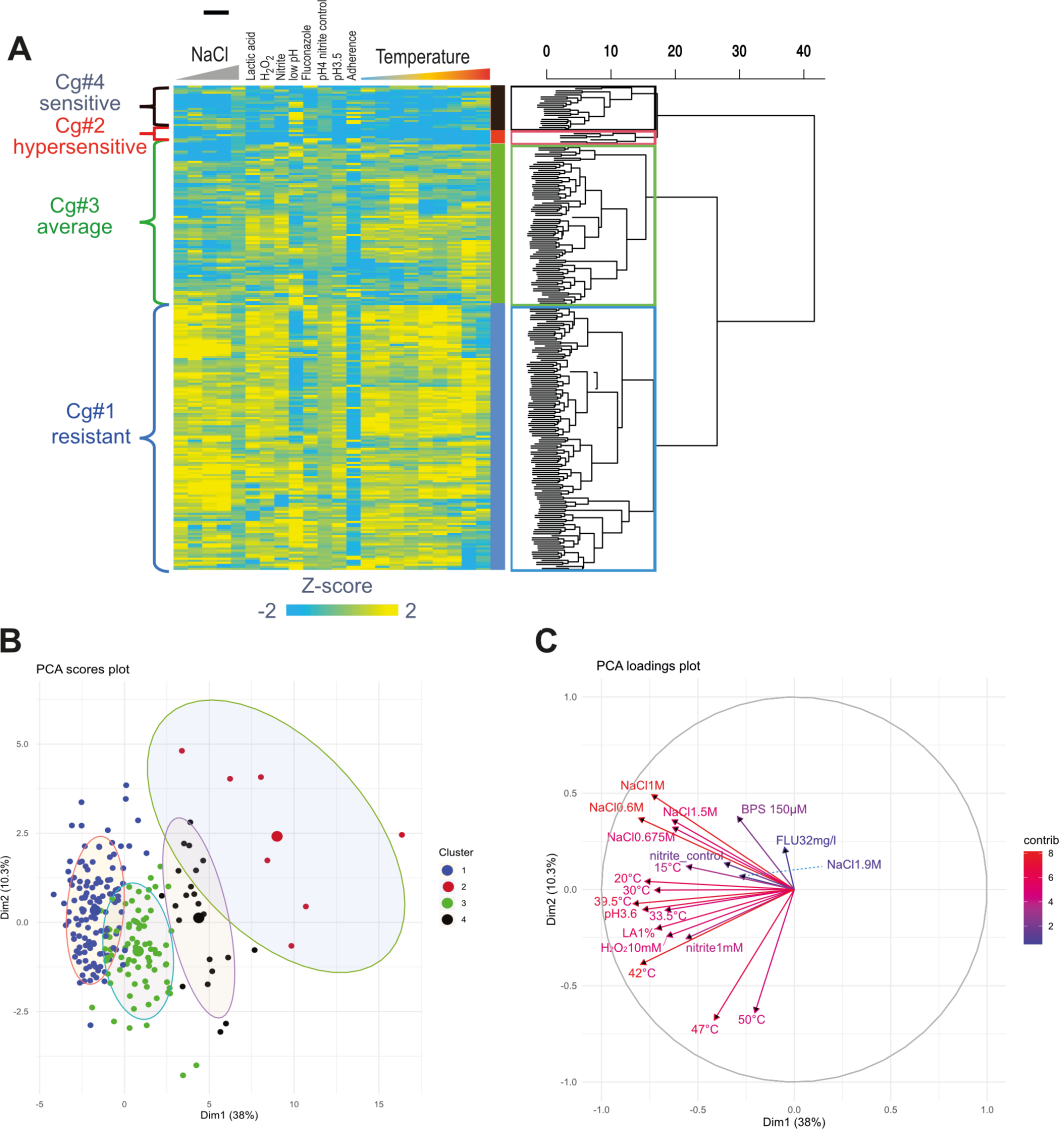
Phenotypic analysis of multiple parameters. (**A**) PCA and hierarchical clustering z-transformed data reveals four phenotypic clusters represented as a heat map. (**B**) *C. glabrata* isolates plotted against the first two principal components in a PCA scores plot. Cluster assignments are indicated by color. (**C**) PCA loading map showing the individual conditions (filled triangles) plotted versus the first two PCs. Variables contributing similarly to the PCA cluster together. Variables close to the edge contribute more to the respective dimension and are thus more influential. FLZ = fluconazole, LA = lactic acid.

The phenotypic properties of individual *C. glabrata* isolates may represent either discrete or continuous traits. We used a quantitative high-throughput approach to analyze the population under conditions encountered in the environment and potentially within the human host (16). For example, resistance to oxidative or nitrosative stress is important when facing protective immune cells like macrophages (77). High lactic acid concentrations and low pH environments occurring in the human vagina environment can inhibit *Candida* growth (78). Iron requirements were assessed with bathophenanthroline disulfonate (BPS), which depletes dissolved Fe(II) ions (79–81). Additionally, we quantified the ability to form biofilms on surfaces. Growth parameters, including growth rate, lag phase, and total growth, were determined for each condition (detailed in Materials and Methods), and the resulting data set (Table S4) was analyzed using principal component analysis (PCA), hierarchical clustering, and permutational multivariate analysis of variance (PERMANOVA). The combined phenotypic footprint of 238 *C*. *glabrata* isolates separated into four clusters, Cg#1 to Cg#4, is shown as a heatmap (Fig. 2A). Separation of the clusters was tested by permutation analysis and is reported as a dispersion blot (Fig. S2C). PERMANOVA showed a significant group effect (pseudo-F = 46.081, df = 3, 237, r2 = 0.37, *P* = 0.0005), indicating strong multivariate differentiation among groups. Cluster Cg#1 (*n* = 130) included isolates resistant to multiple stress types, whereas cluster Cg#2 (*n* = 7) comprised slow-growing and stress-sensitive isolates. Cluster Cg#3 (*n* = 79) represented isolates with intermediate fitness across all conditions, and cluster Cg#4 consisted of slow-growing isolates displaying resistance to various stresses. The relative spread of the individual principal component properties in the population is visualized with each individual plotted on the first two principal components (Fig. 2B). Strains of cluster Cg#2 separated farthest from others in the individual factor map of the PCA. The contribution of the particular conditions to the first two principal components is visualized as a PCA loading map (Fig. 2C). Osmotic stress treatments at different concentrations are located close to each other, suggesting a similar impact on the growth rate. Cold and heat stress are independent, as shown by their perpendicular position. We observed variation in the intrinsic fluconazole resistance of the *C. glabrata* isolates (82). Other more subtle correlations detected by Spearman Rank Order test are listed in Table S1. Taken together, using a quantitative setup for determination of growth parameters, we identified four distinct phenotype classes in a population of *C. glabrata* isolates and found that basal growth rate was inversely correlated with stress resistance.

### *Candida* species show unique phenotypic responses

**Fig 3:**
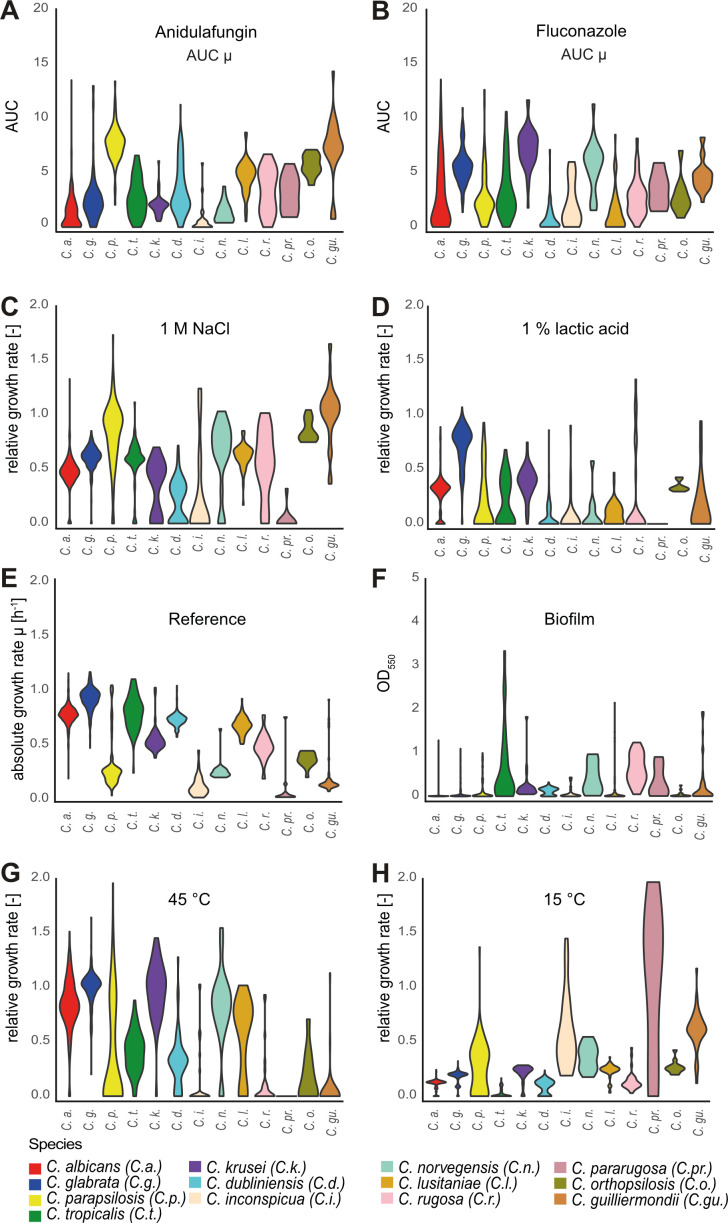
The spread of selected fitness parameters differs between species. Species (color-coded) and conditions are indicated within each violin plot. (**A and B**) AUC represents the area under the antifungal dose-response curve (constructed using growth rate µ). (**F**) Biofilm values (OD_550nm_) correspond to the amount of adherent cells remaining on a plastic surface after washing. Absolute growth rate (**E**) indicates growth rate per hour; relative growth rate (**C, D, G, and H**) is normalized to the absolute growth rate at 37°C (“Reference”).

We extended our analysis of phenotypic traits to other human-associated *Candida* species. We chose 1,366 isolates from 13 *Candida* species from Austrian hospitals (63). All isolates were exposed to stress conditions partly mimicking certain aspects of the human or mammalian host environment. Growth kinetics were measured and referenced to growth at 37°C as the control condition (Table S5). Fitness against antifungals was quantified using the AUC as described in Materials and Methods (83). The calculated fitness parameters were based on maximum growth rates and maximum cell densities (OD600) or, in the case of biofilm, on absolute optical density values. The robot-aided experimental setting enabled broad comparative analysis across multiple experiments. Results are visualized as violin plots (selected examples in Fig. 3, entire set in Data SD4). Dependent on the stress condition, we observed a different degree of phenotypic diversity between species. Some species, such as *C. glabrata* (Fig. 3A through H in blue), behaved more uniformly over many different stress conditions, while others, for example, *C. parapsilosis* (Fig. 3A through H in yellow) exhibited considerable phenotypic variation.

Regarding resistance to antifungals (Fig. 3A and B), we found that *C. parapsilosis* and *C. guilliermondii* exhibited high fitness, while *C. krusei*, *C. norvegensis,* and *C. glabrata* were the fittest species when challenged with azoles. We further found high osmotic stress resistance in *C. parapsilosis*, *C. norvegensis*, *C. lusitaniae*, *C. rugosa*, *C. orthopsilosis,* and *C. guilliermondii* isolates (Fig. 3C). *C. glabrata* tolerated lactic acid and low pH stress (Fig. 3D). With regard to basal growth (36), *C. glabrata* displayed the fastest growth rates of all investigated species, while *C. parapsilosis*, *C. inconspicua,* and especially *C. pararugosa* (µ < 0.1 h^−1^) were relatively slow (Fig. 3E). Interestingly, *C. tropicalis* (in green) showed high growth variation (±0.15 h^−1^) even in a controlled environment, while variance of growth rates in other species remained low. Biofilm formation (Fig. 3F) occurred, albeit in a few isolates of *C. tropicalis*, *C. rugosa*, *C. pararugosa*, *C. norvegensis*, *C. krusei*. Phenotypic variation increased at high temperature (45°C) and was diminished at low temperature (15°C) in most species (Fig. 3G and H). Interestingly, *C. parapsilosis*, *C. lusitaniae*, *C. pararugosa,* and *C. guilliermondii* isolates showed phenotypic diversification at lower temperatures (Fig. 3H). Taken together, we find substantial phenotypic variation between *Candida* species when exposed to an assortment of environmental conditions mimicking the human host. Moreover, we find that even closely related species may have different phenotypic properties.

### Human-associated *Candida* species segregate into several phenotypically distinct and species-enriched clusters

**Fig 4:**
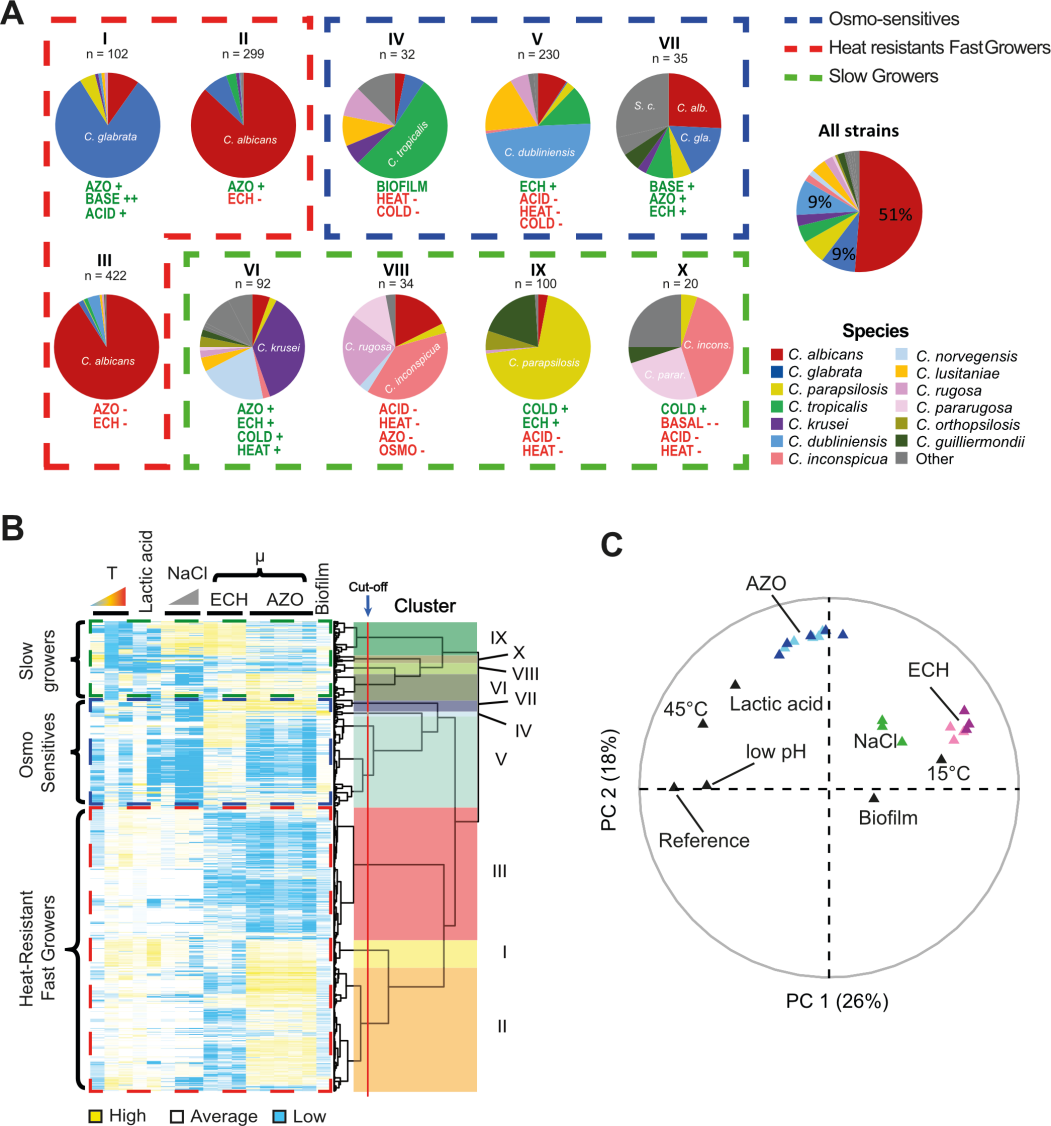
Phenotypic characterization of *Candida* species. (**A**) Species composition and phenotypic attributes of each phenotypic cluster and assignment to superclusters (dashed lines). The composition of the total set of isolates is displayed on the right. The dominating species are indicated within each pie chart. Differences between clusters compared to the overall average: “--” “-'' lower “++” +” higher performance. Clusters are labeled with roman numerals. In total, 1,366 isolates were included in the screen. (**B**) Factor map of the PCA showing the individual variables (filled triangles) plotted versus the first two PCs. Similar conditions are grouped and indicated by color. Variables not belonging to any groups are colored black. Variables that contribute similarly to the PCA cluster together. Variables close to the edge contribute more to the respective dimension and are thus more influential. (**C**) Heatmap displaying the hierarchical clustering of fitness of 1,366 *Candida* isolates. Blue color indicates low performance (sensitivity), while yellow indicates high performance. Clusters are indicated as dashed lines. Sub-clusters on the right. Abbreviations: BASE = Basal growth; COLD = 15°C; HEAT = 45°C; ACID = low pH or lactic acid stress; OSMO = osmotic stress. ECH = Echinocandins (Micafungin, Anidulafungin, and Caspofungin); AZO = Azoles (Fluconazole, Itraconazole, Voriconazole, Posaconazole, and Isavuconazole).

The measured phenotypic variation allowed us to search for inter- and intraspecies patterns. We normalized the growth parameter relative to the species-specific reference growth condition and reduced the dimensionality using PCA and hierarchical clustering. This revealed three overarching phenotypic patterns (designated clusters A, B, and C), which further separated into 10 subclusters designated by roman numerals I to X. We defined key phenotypic properties (Table S2) and analyzed species composition as illustrated by heat maps and pie charts (Fig. 4A and B). To assess differences in overall phenotypic profiles among groups, PERMANOVA was conducted based on Euclidean distances for 10 clusters (r2 = 0.65, *P* = 0.0005). Furthermore, clusters A, B, and C were likewise supported by PERMANOVA (r2 = 0.38, *P* = 0.0005) and pairwise PERMANOVA (Table S8). The number of 10 clusters chosen was supported by the adonis2 permutation test, and a pairwise PERMANOVA resulted in some support (r2 = 0.2 to 0.7; Table S8); however, the data structure including all strains is essentially characterized by a substantial overlap. Out of several tested numbers of clusters, the chosen 10 separates the different species according to their assumed biological properties.

Most *C. albicans* and *C. glabrata* isolates belong to the “Heat-resistant Fast Grower” cluster A (*n* = 823, Fig. 4A, red dashed line). They share a high growth rate at 37°C and 42°C. Within this cluster, most *C. glabrata* isolates group into the azole-resistant, fastest-growing cluster I (µ = 0.93 h^−1^ ± 0.12 h^−1^), while *C. albicans* isolates separate into azole-resistant (cluster II) and azole-sensitive (cluster III). The second largest cluster B contains the “Osmo-sensitives” (*n* = 297, Fig. 4A, dashed blue line). This cluster was divided into the biofilm-forming cluster IV (containing the majority of *C. tropicalis* isolates), echinocandin-resistant cluster V (dominated by *C. dubliniensis*), and multi-resistant cluster VII (containing fast-growing and multi-resistant *C. albicans*, *C. glabrata,* and *S. cerevisiae*). Compared with *C. albicans*, *C. dubliniensis* is more heat sensitive and more resistant to echinocandins (84). Additionally, we detected osmotic, cold, and acid sensitivity of *C. dubliniensis* (Fig. 3C and H).

Cluster C, “Slow Grower” (Fig. 4A, *n* = 246, green dashed line) divides it into four subclusters. Cluster IX (*n* = 100) harbors predominantly echinocandin and cold-resistant *C. parapsilosis* and *C. guilliermondii* isolates. Cluster VI (*n* = 92) contains heat and cold stress, azole and echinocandin-resistant *C. krusei* and *C. norvegensis* isolates. Cluster VIII (*n* = 34) comprised mostly of hyper-sensitive *C. inconspicua* and *C. rugosa* isolates. These were sensitive to all stress factors tested here. Finally, cluster X (*n* = 20) comprised isolates of *C. pararugosa* and *C. inconspicua* with a very low basal growth rate of just 0.06 ± 0.03 h^−1^ and elevated resistance to cold stress. The hierarchical clustering performance of each isolate can be visualized in the form of a heatmap (Fig. 4B). We evaluated correlations between stress conditions (Fig. 4C). Azole resistance and echinocandin resistance appear perpendicular to each other, indicating independence. Growth at 15°C and 37°C appears in opposite directions, indicating a negative correlation. Thus, isolates are either heat or cold adapted, while resistance to azoles is independent from resistance to echinocandins.

**Fig 5:**
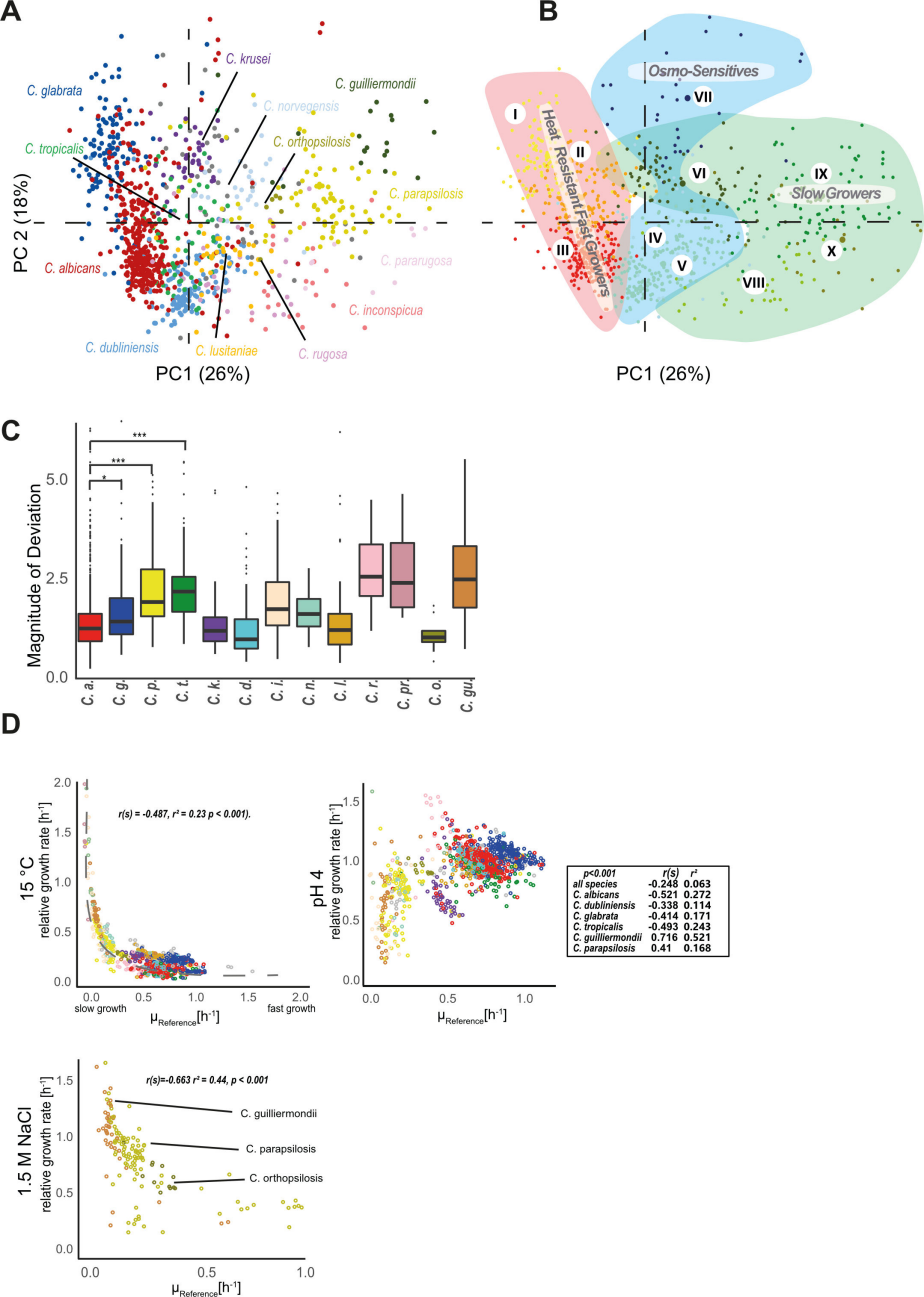
Phenotypic landscape. (**A**) Factor map of individuals with each dot representing an isolate. Isolates are color-coded according to species. Members of the same species group tend to agglomerate. (**B**) Factor map with isolates color-coded according to (super-)cluster assignment. Superclusters are indicated by shaded areas in the plot. Differently colored isolates within each supercluster indicate the individual sub-clusters, which group together if they behaved similarly in the phenotypic screen. (**C**) The magnitude of deviation is calculated as difference of weighted sum of squares for each species and indicates the phenotypic variance of each set of isolates when grouped according to species. High values indicate that representatives of this species are phenotypically different from each other. (**D**) Correlation analysis of selected combinations of variables. Spearman ranked order test results for correlation are indicated.

The phenotypic diversity within the *Candida* genus appears to be substantial. The resulting clusters were often dominated by a single species, and individual species were often restricted to a few clusters. We plotted the phenotypic landscape of the *Candida* species colored by species and clusters after PCA in two dimensions (Fig. 5A and B) and as 3D versions by including the third PC (Data SD1 and SD2). Most isolates cluster alongside species boundaries, albeit the degree of variability differs between species. *C. albicans* (red) occupies large swaths of the phenotypic landscape. A dense patch of *C. dubliniensis* isolates (light blue) is located near but separated from the closely related *C. albicans*. Factor analysis is also useful to determine the degree of variation between species and among the species isolates. The clusters are depicted as shaded areas in Fig. 5B. Each species occupies a distinct space in this phenotypic landscape. Notably, cluster boundaries largely coincide with species designation, which is evident by comparing Fig. 5A and B, which display the same data.

We were further interested in the degree of intra-species variation and found stark differences between species. For example, *C. dubliniensis* strains were comparatively uniform, while *C. parapsilosis* isolates showed greater dispersion. We quantified the phenotypic variability by calculating the distance between the phenotypic barycenter of the respective isolates (Fig. 5C), determined as the species-specific weighted sum of overall differences. Higher overall values correlate with high intra-species differences.

According to our panel of stress conditions, the analyzed strains can be grouped into several clusters based on their phenotypic properties. We observed clustering along species boundaries but also substantial intra-species variation.

### Genetic typing analysis by MLST does not support correlation to phenotypes

We were interested in to what extent general genetic distances could explain intraspecies phenotypic differences. We analyzed the occurrence of single nucleotide polymorphisms via MLST for *C. albicans*, *C. glabrata*, *C. tropicalis*, *C. dubliniensis,* and *C. krusei* in selected strains with high phenotypic differences. We assigned a sequence type (ST) to each isolate based on the combination of alleles of a set of commonly used genetic loci (pubmlst.org). For phylogenetic analysis, we used the concatenated sequences of the six to seven amplicons, depending on the species (Data SD3; Table S7). We found new alleles and new sequence types. Among the 39 *C*. *dubliniensis* isolates, 10 different STs were identified. New STs were found among the isolates of *C. tropicalis* (74%), *C. albicans* (69%), and *C. krusei* (49%). Phylogenetic distances were minimal between *C. dubliniensis* isolates and maximal between *C. tropicalis* isolates. We included the phenotypic cluster designations into the phylogenetic trees to correlate phenotypic diversity with genetic diversity. The phenotypic clusters appeared dispersed throughout the tree in all investigated species (not shown). Some isolates with identical STs displayed different phenotypes. This was especially pronounced in *C. dubliniensis* and *C. tropicalis*, with many isolates showing identical MLST profiles. Overall, we could not detect a correlation between fitness under individual stress conditions and ST genotypes, nor with branches within our constructed phylogenetic tree. We conclude that the phenotypes quantified here are not correlated with phylogenetic branches based on MLST data. Further analysis, including whole-genome sequencing and systematic gene expression studies, might provide deeper insights.

### Genus-wide correlation analysis suggests phenotypic stratification of *Candida* species

In addition to the multivariate approach of phenotypic analysis, we performed a pairwise correlation analysis of parameters using the Spearman rank-order test (Table S1). Selected correlations are visualized as dot plots color coded by species (Fig. 5D). All correlations of the tested conditions are provided in Data SD5. We observed a negative, nonlinear correlation between growth at 15°C and basal growth rate (Fig. 5D, panel 15°C; Spearman’s rank correlation test: r(s) = −0.487, r² = 0.23 *P* <0 .001). Isolates with slow basal growth rate showed similar growth rates at 15°C. Isolates from slow-growing species such as *C. parapsilosis* and *C. guilliermondii* were among those who performed best in cold conditions. Fast-growing isolates, such as most *C. glabrata* isolates, did not perform in the cold. This correlation followed a logarithmic trend throughout all tested isolates. Furthermore, factor analysis (Fig. 4D) placed the variable for 15°C opposite to reference growth (37°C), indicating an inverse relationship between the two parameters. Isolates of clusters VI, IX, and X perform well at 15°C but grew slowly at 37°C. Furthermore, we found for most species a negative correlation between low pH tolerance and basal growth (Fig. 5D, panel pH 4; Spearman’s rank correlation test indicated). Isolates of *C. parapsilosis, C. orthopsilosis,* and *C. guilliermondii* belonging to cluster IX showed a negative correlation between growth under osmotic stress and basal growth rate (Fig. 5D, panel 1.5 M NaCl; Spearman’s Rank correlation test r(s) = −0.663 r² = 0.44, *P* < 0.001). All associations between conditions are compiled in a Data SD5.

## DISCUSSION

### A quantitative phenotypic overview of human-associated *Candida* strains

We previously reported on the antifungal resistance of clinical isolates of *Candida* species (63). Here, we focus on individual phenotypic variations under conditions related to virulence or associated with survival in the host. Fitness in growth assays was quantified using a high-throughput method with high reproducibility. The tested conditions included osmotic stress, varying temperatures, low pH and lactic acid stress, biofilm formation, and resistance to antifungal drugs.

Hierarchical clustering of quantitative phenotypic data separated the isolates into three main groups (Heat-Resistant Fast Growers, Osmo-Sensitives, Slow Growers) and ten subclusters. Most species were enriched in a single cluster (Fig. 4A). *C. tropicalis*, *C. albicans,* and *C. inconspicua* were distributed across multiple clusters. Antifungal resistance was the primary distinguishing feature for these species, implying resistant subpopulations. *C. albicans* isolates separated into azole-resistant (cluster II) or azole-sensitive (cluster III) strains. *C. tropicalis* isolates diverged, among other features, in their echinocandin resistance, while *C. inconspicua* isolates separated into azole-sensitive (cluster VIII) and intermediate azole-resistant (cluster X).

The host environment may both limit and facilitate phenotypic diversification. There are many different niches within the human body (e.g., the skin, intestine, or vagina), posing unique challenges and, in turn, requiring different phenotypic adaptations for a commensal organism. The human immune system likely exerts a specific selective pressure that is independent of the *Candida* species (85). This notion is supported by observations showing that, in a vaginal epithelial cell model, the *in vitro* transcriptional response to four different *Candida* species is initially uniform during the early stages of infection but becomes species-specific once host cell damage occurs (80). The initial uniform host response might channel phenotypic diversification.

Different phenotypically defined isolates could help estimate the contribution of intraspecies diversity. Phenotypic clusters observed in our collection separate the strains along species boundaries, indicating their adaptation to specific niches. Phenotypic bifurcation was observed for a few species of our collection and is possibly subject to a selection bias in the collection through the host environment or prior antifungal treatment.

### Probing the genetic connection between phenotypes and genotypes

To assess the genetic relationships among isolates, we performed MLST analysis of the five most common *Candida* species. The MLST database (pubmlst.org) hosts sequence information from isolates collected worldwide (86). Our phenotypic clusters appeared evenly dispersed among the phylogenetic branches and thus did not correlate with the phylogenetic topology (Fig. S1). Thus, phenotypic adaptations appear to be more flexible than genetic changes. MLST data are based on a small proportion of the genome and the relevant changes might occur in other loci. Based on whole-genome data, *C. glabrata* phylogeny is geographically correlated and can be divided into seven clades (17, 87). MLST data from all publicly available *C. glabrata* isolates from the pubmlst.org database did not reveal such a correlation (data not shown), arguing for the need for broader genomic analysis. Moreover, progeny of genetically indistinguishable reference *C. glabrata* strains may show distinct phenotypic profiles (88). We found that isolates with identical MLST profiles occasionally showed distinct phenotypic behavior. In our *C. tropicalis* isolates, MLST analysis found no correlation between sequence types and azole resistance (89). For the five species investigated, we could not detect an association between MLST-based genetic markers and phenotype. We conclude that further investigation into genomic, epigenetic, or transcriptional profiling under different conditions would be necessary to identify the genetic basis of phenotypic variation.

### *Candida* species occupy phenotypic niches and adapt *ad hoc* to stress

*Candida* species exhibit highly diverse fitness levels under temperature stress (Fig. 3G and H). *C. glabrata and C. krusei* tolerate elevated temperatures, whereas *C. tropicalis* and *C. dubliniensis* are temperature-sensitive. Isolates growing rapidly at ambient temperature also do so at higher temperatures. This is reflected by the group of “Heat-resistant fast growers” and the proximity of the two parameters on the factor map (Fig. 4C). We observed phenotypic stratification of growth performance at 45°C relative to basal growth (Fig. 5D, “45°C”), even though correlation was moderate (ρ = 0.38). Here, isolates that grow well at 37°C also tend to grow well at 45°C. These temperature adaptations might represent one step toward host adaptation. The most prevalent pathogenic *Candida* species, *C. albicans* and *C. glabrata*, are heat-adapted, which confers a growth advantage in mammals. Heat tolerance is necessary but not sufficient for host adaptation (90). In contrast, species that cause fewer infections, such as *C. krusei*, *C. inconspicua,* and *C. pararugosa*, lack this adaptation.

Analogous to heat adaptation, a trade-off exists between cold adaptation and basal growth speed. We observed a negative correlation between growth at 15°C and basal growth rate (Fig. 5D, panel “15°C”). Adaptation to adverse conditions, such as cold stress and drug resistance, might involve a metabolic trade-off occurring across the *Candida* clade. Certain species, such as *C. norvegensis*, *C. inconspicua*, *C. pararugosa*, *C. guilliermondii,* and *C. parapsilosis,* appear to be adapted to cooler environments, yet exhibit slow overall growth. Cold adaptation is, at least in part, an active process. This process has been studied in *S. cerevisiae*, which transiently adapts to cold stress through transcriptional regulation and trehalose accumulation (91). Cold adaptation might be favorable for *C. parapsilosis* in colonizing the skin rather than internal body sites and thus might explain its environmental niche.

Regarding antifungal resistance patterns, we found a significant but small negative correlation between fitness under echinocandin treatment and basal growth rate, indicating a fitness cost associated with echinocandin resistance. Echinocandin-resistant species, such as *C. parapsilosis* and *C. guilliermondii*, have a low basal growth rate, whereas sensitive species grow rapidly. Our phenotypic clustering distinguished between echinocandin-sensitive fast growers (clusters II and III) and echinocandin-resistant slow growers (clusters VI and IX). The slower basal growth may also result from the metabolic costs of general and specific resistance mechanisms, such as overexpression of FKS genes (92). A growth deficit, determined under “ideal” conditions, rarely existing in highly challenging environments may be traded for stress resistance (93, 94).

We found substantial osmotic stress resistance among the investigated species (Fig. 3C, “1 M NaCl”). Many species, especially *C. glabrata*, *C. parapsilosis*, *C. guilliermondii*, and *C. albicans*, were able to grow in a medium supplemented with 1.9 M NaCl, corresponding to 3800 mOsmol/l. For comparison, physiological osmolalities in the human vagina or gut are around 400 mOsmol/L (95). Resistance to osmotic stress was distributed across many clusters and therefore not correlated with other adaptations. Among *C. parapsilosis* and *C. guilliermondii* isolates, resistance to osmotic stress coincided with slower basal growth (Fig. 5D, “1.5 M NaCl”). Altogether, the *Candida* genus was stratified into heat-adapted and cold-adapted clusters. Furthermore, reduced basal growth is associated with resistance to echinocandins, osmotic stress, and cold stress.

### *C. glabrata* phenotypes divide into four clusters

*C. glabrata* ranks second among *Candida* species causing human infections. SNPs and duplications are common in *C. glabrata*, especially in the subtelomeric regions and in adhesion-related genes such as the *EPA* genes (96). As a haploid organism that largely propagates clonally, *C. glabrata* expresses recessive mutations phenotypically, which could potentially promote variation under selective pressure. We identified four prevalent phenotypic patterns. The largest group (Cg#1) showed robust growth under both optimal and stress conditions, while a second group (Cg#4) displayed the opposite phenotype (Fig. S2D). Cluster Cg#3 included isolates with intermediate fitness across all conditions. Biofilm formation in *C. glabrata* is driven by the expansion of adhesin-coding *EPA* genes, which are key determinants of pathogenicity. These genes are located in variable, low-complexity subtelomeric regions (90, 97–99). Adhesion properties were spread among all clusters and thus appear not connected to specific stress resistance patterns. *C. glabrata* further displayed a high degree of population variance when challenged with elevated temperatures (≥42°C), whereas growth at 15°C was similar among all isolates tested (Fig. 1B). Osmotic and heat stress affected population fitness distributions differently (Fig. 1B and C). The distribution of fitness remained narrow under both high and low osmotic stress. The spread of fitness within the population suggests increasing cellular pathology with rising temperature. In contrast, higher osmotic stress does not appear to impose additional stress.

### *C. albicans and C. dubliniensis* population phenotypes reveal adaptive flexibility

*C. dubliniensis* is genetically much more uniform than *C. albicans* (72). In the regions analyzed by MLST, there are less than 1% of SNPs among *C. dubliniensis* isolates compared with more than 6% found among *C. albicans* strains. The low proportion of SNPs indicates a relatively recent speciation event (100). Genome-wide SNP frequency among *C. albicans* strains is close to 1,4% (101). *C. dubliniensis* strains of our collection appeared phenotypically uniform in one cluster (V, Fig. 4A). *C. albicans* strains divided between azole-resistant (Cluster III) and an azole-sensitive (Cluster II and III) in a 40:60 ratio. The isolates in the two clusters only differ in their azole phenotype, and the difference was gradual rather than occurring in discrete steps (Fig. 5B). This adaptation is perhaps caused by prior encounters with azole-based drugs. The bifurcation *of C. albicans* is unique among the species investigated here and might contribute to superior adaptivity to the human host. Azole resistance may be caused by an array of different mechanisms such as point mutations in certain genes (e.g. *ERG11*) or overexpression of drug efflux pumps (*CDR1* or *MDR1*) (92, 102). We also found no correlation with basal growth rate, indicating that azole resistance, unlike echinocandin resistance, does not come with a metabolic cost. *C. albicans* appears as a highly adaptive organism, which shows migration into phenotypic spaces supporting also its association to humans.

### Genetically uniform *C. parapsilosis* is phenotypically highly diverse

*C. parapsilosis* isolates in our collection appear exceptionally diverse in the phenotypic landscape (Fig. 3A, C, D, G and H), despite being genetically uniform as indicated by low frequency of SNPs between isolates (73). The propensity of *C. parapsilosis* to adhere to host cells varies between isolates and correlates with the expansion ratios for ALS genes (103). In our strains, we observed poor adhesion (Fig. 3F, “Biofilm”). C*. parapsilosis* isolates clustered largely with *C. orthopsilosis* in accordance with previous observations (73). Compared to other *Candida* species, *C. parapsilosis* occupies a larger phenotypic space and appears to be more diverse, especially at 15°C and 45°C (Fig. 4B). Given its innate resistance to echinocandins, the extent of phenotypic variation and thus its ability to adapt to challenging environments might be of further interest.

We show that phenotypic variation is substantial within and between human-associated *Candida* species. The method of quantitative phenotypic analysis opens a new view at characteristic overlaps and distinctions of such populations. The *Candida* strains analyzed here are phenotypically diverse and separate phenotypically alongside species boundaries. Intraspecies connections between phenotype and genotype were not resolved with MLST and await a more fine-grained deep genomic analysis. Despite largely clonal reproduction, most species occupy a relatively discrete space, presumably due to both host and environmental constraints.

## SUPPLEMENTAL MATERIAL

Data SD1 msystems.00786-25-s0001.gif Animated gif of 3D PCA.

Data SD2 msystems.00786-25-s0002.gif Animated gif of 3D PCA.

Data SD3 msystems.00786-25-s0003.txt MLST sequence information.

Data SD4 msystems.00786-25-s0004.pdf Violin blots of phenotypes.

Data SD5 msystems.00786-25-s0005.pdf All correlations.

Figure S1 msystems.00786-25-s0006.tiff Figure S1

Figure S2 msystems.00786-25-s0007.tiff Figure S2

Legends msystems.00786-25-s0008.docx Legends to supplemental files.

Supplemental Tables msystems.00786-25-s0009.xlsx Tables S1 to S8.

ASM does not own the copyrights to Supplemental Material that may be linked to, or accessed through, an article. The authors have granted ASM a non-exclusive, world-wide license to publish the Supplemental Material files. Please contact the corresponding author directly for reuse.
