# Supplementary figures and images for: Distinct properties of human pathogenic Candida species revealed by systematic comparative phenotypic screening of clinical isolates
Source: mSystems. 2025 Dec 8;11(1):e00786-25. doi: 10.1128/msystems.00786-25 (PMC12817934; doi:10.1128/msystems.00786-25)

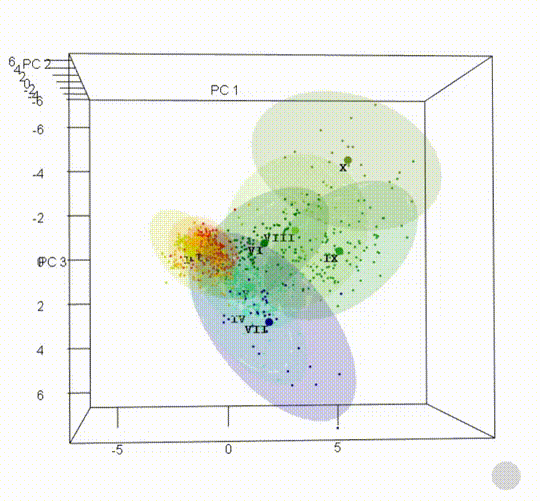

Supplement: Data SD1 — Animated gif of 3D PCA. [file msystems.00786-25-s0001.gif]

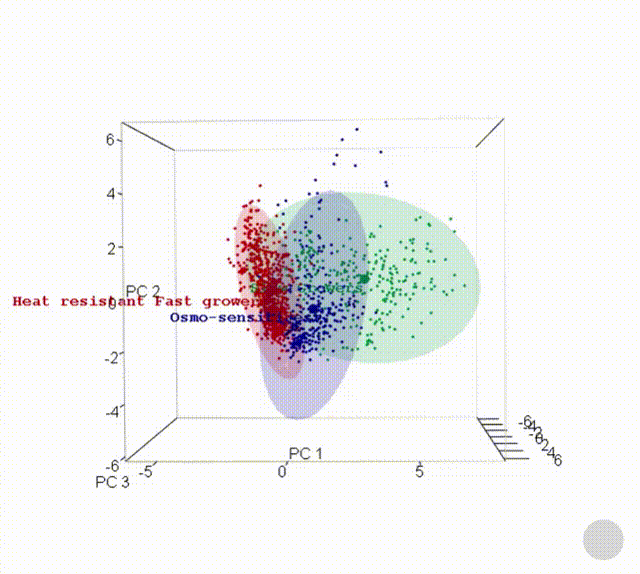

Supplement: Data SD2 — Animated gif of 3D PCA. [file msystems.00786-25-s0002.gif]

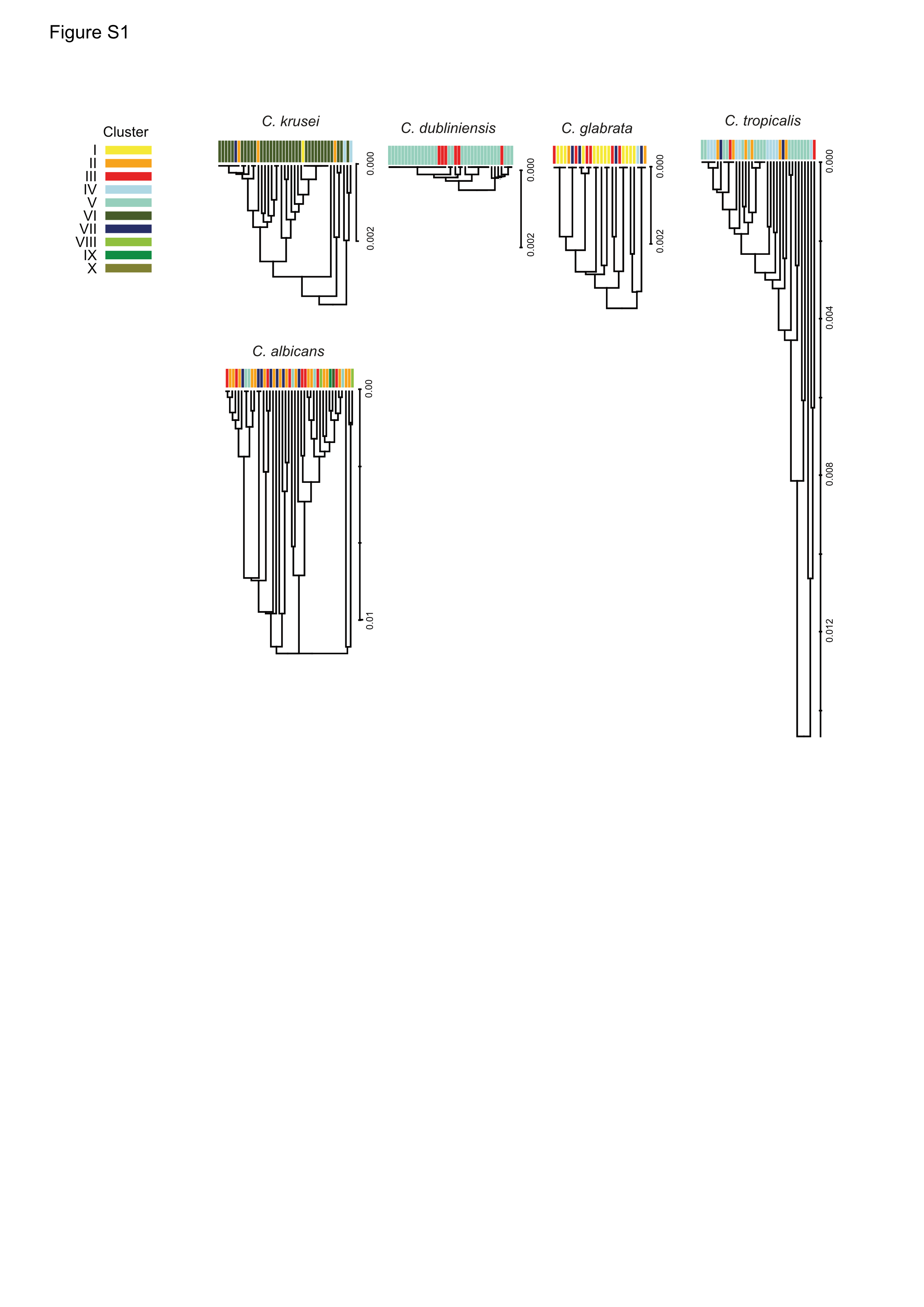

Supplement: Figure S1 [file msystems.00786-25-s0006.tiff]

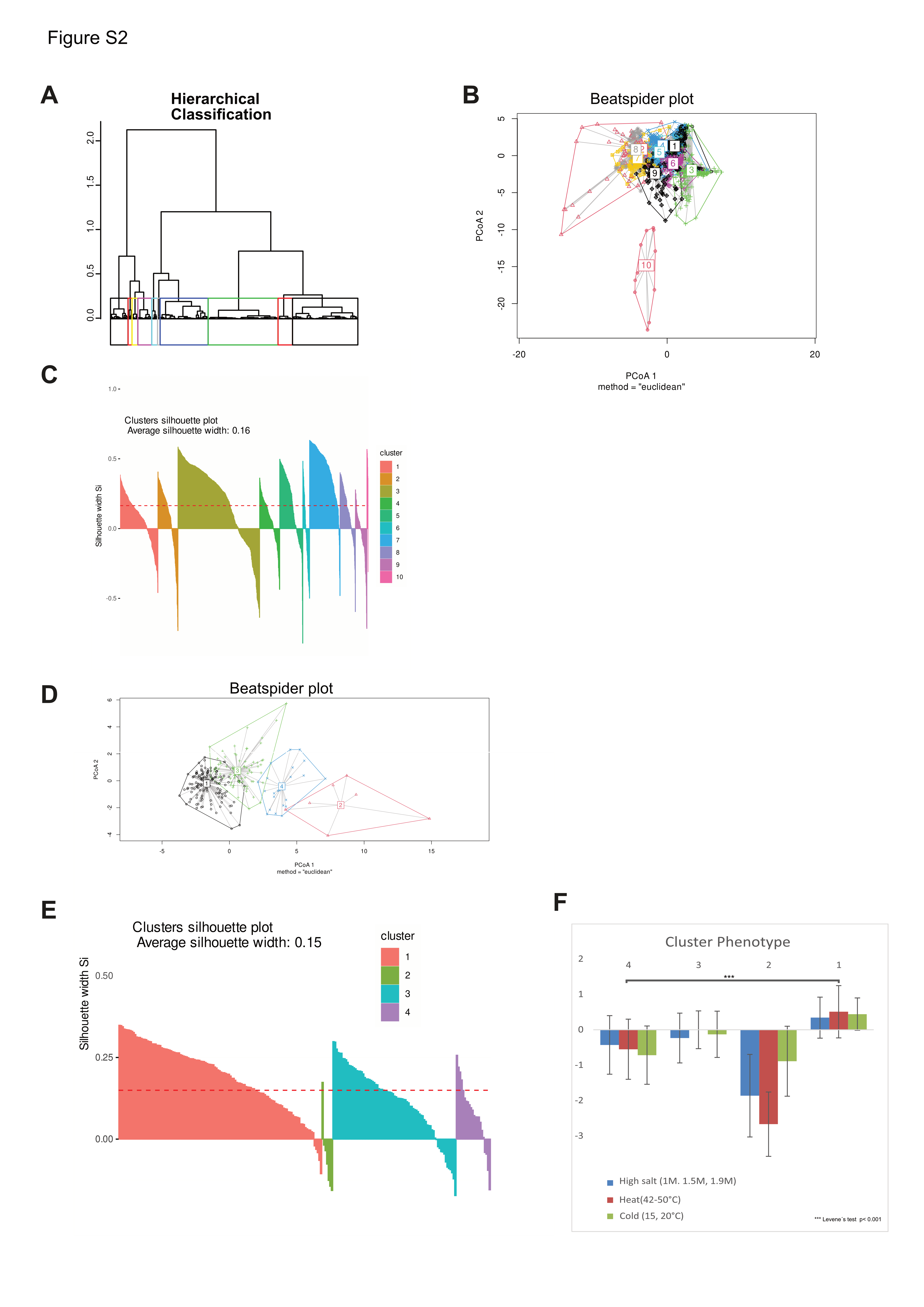

Supplement: Figure S2 [file msystems.00786-25-s0007.tiff]
