# Supplementary material for: Distinct properties of human pathogenic Candida species revealed by systematic comparative phenotypic screening of clinical isolates
Source: mSystems. 2025 Dec 8;11(1):e00786-25. doi: 10.1128/msystems.00786-25 (PMC12817934; doi:10.1128/msystems.00786-25)
Supplement: Data SD4 — Violin blots of phenotypes. [file msystems.00786-25-s0004.pdf]

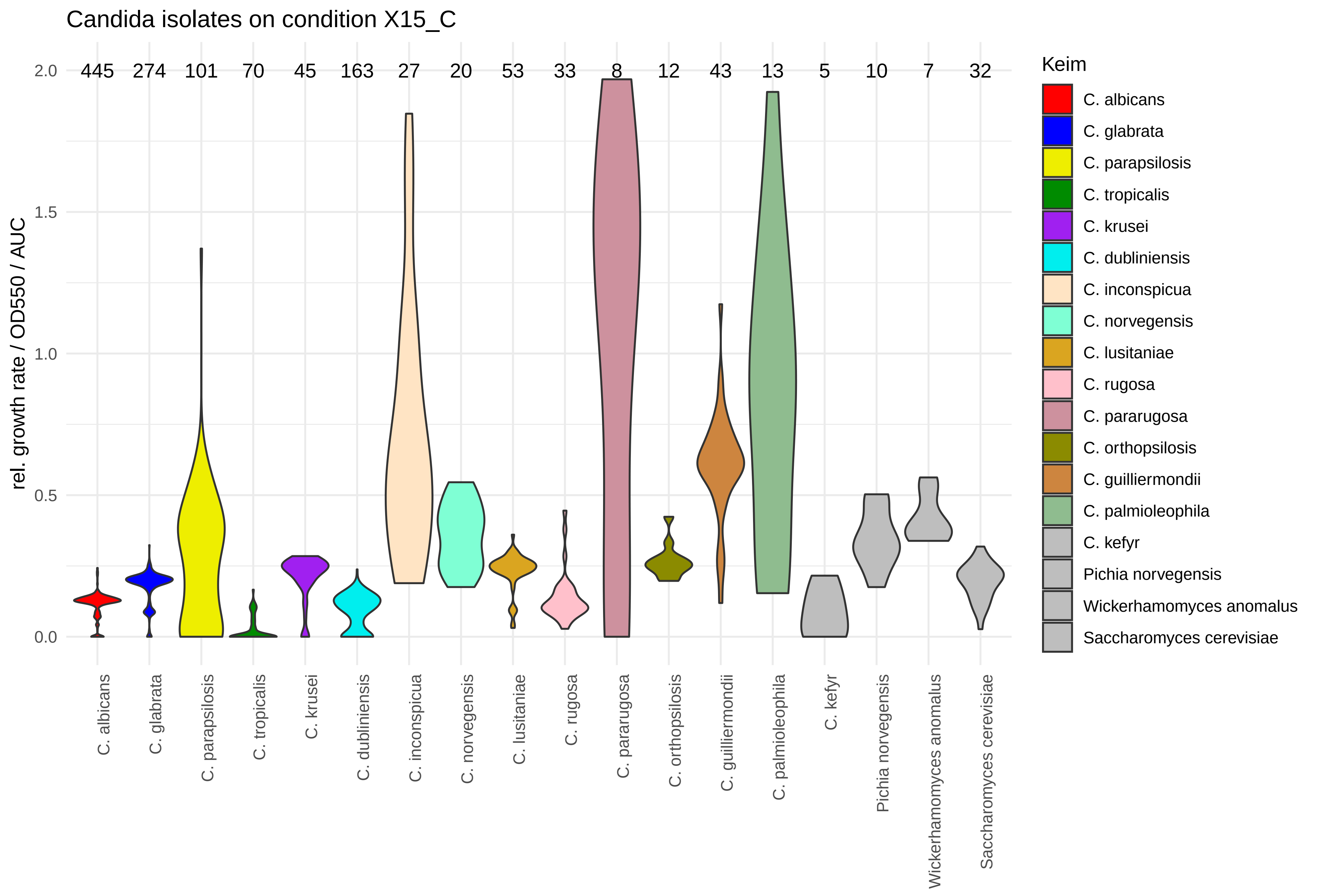

Candida isolates on condition X15_C
rel. growth rate / OD550 / AUC
0.0
0.5
1.0
1.5
2.0
445
274
101
70
45
163
27
20
53
33
8
12
43
13
5
10
7
32
C. albicans
C. glabrata
C. parapsilosis
C. tropicalis
C. krusei
C. dubliniensis
C. inconspicua
C. norvegensis
C. lusitaniae
C. rugosa
C. pararugosa
C. orthopsilosis
C. guilliermondii
C. palmioleophila
C. kefyr
Pichia norvegensis
Wickerhamomyces anomalus
Saccharomyces cerevisiae
Keim
C. albicans
C. glabrata
C. parapsilosis
C. tropicalis
C. krusei
C. dubliniensis
C. inconspicua
C. norvegensis
C. lusitaniae
C. rugosa
C. pararugosa
C. orthopsilosis
C. guilliermondii
C. palmioleophila
C. kefyr
Pichia norvegensis
Wickerhamomyces anomalus
Saccharomyces cerevisiae

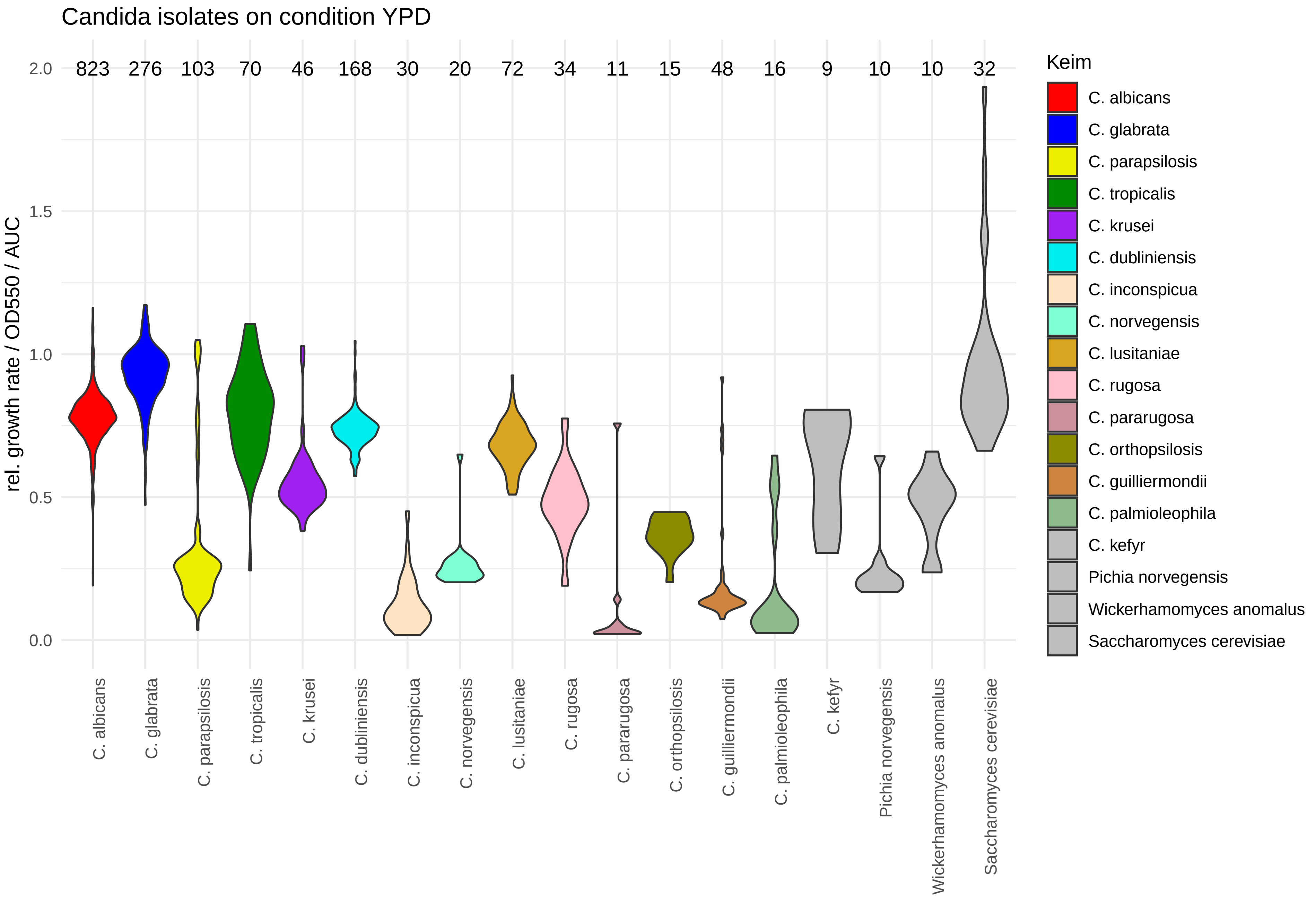

Candida isolates on condition YPD
rel. growth rate / OD550 / AUC
0.0
0.5
1.0
1.5
2.0
823
276
103
70
46
168
30
20
72
34
11
15
48
16
9
10
10
32
C. albicans
C. glabrata
C. parapsilosis
C. tropicalis
C. krusei
C. dubliniensis
C. inconspicua
C. norvegensis
C. lusitaniae
C. rugosa
C. pararugosa
C. orthopsilosis
C. guilliermondii
C. palmioleophila
C. kefyr
Pichia norvegensis
Wickerhamomyces anomalus
Saccharomyces cerevisiae
Keim

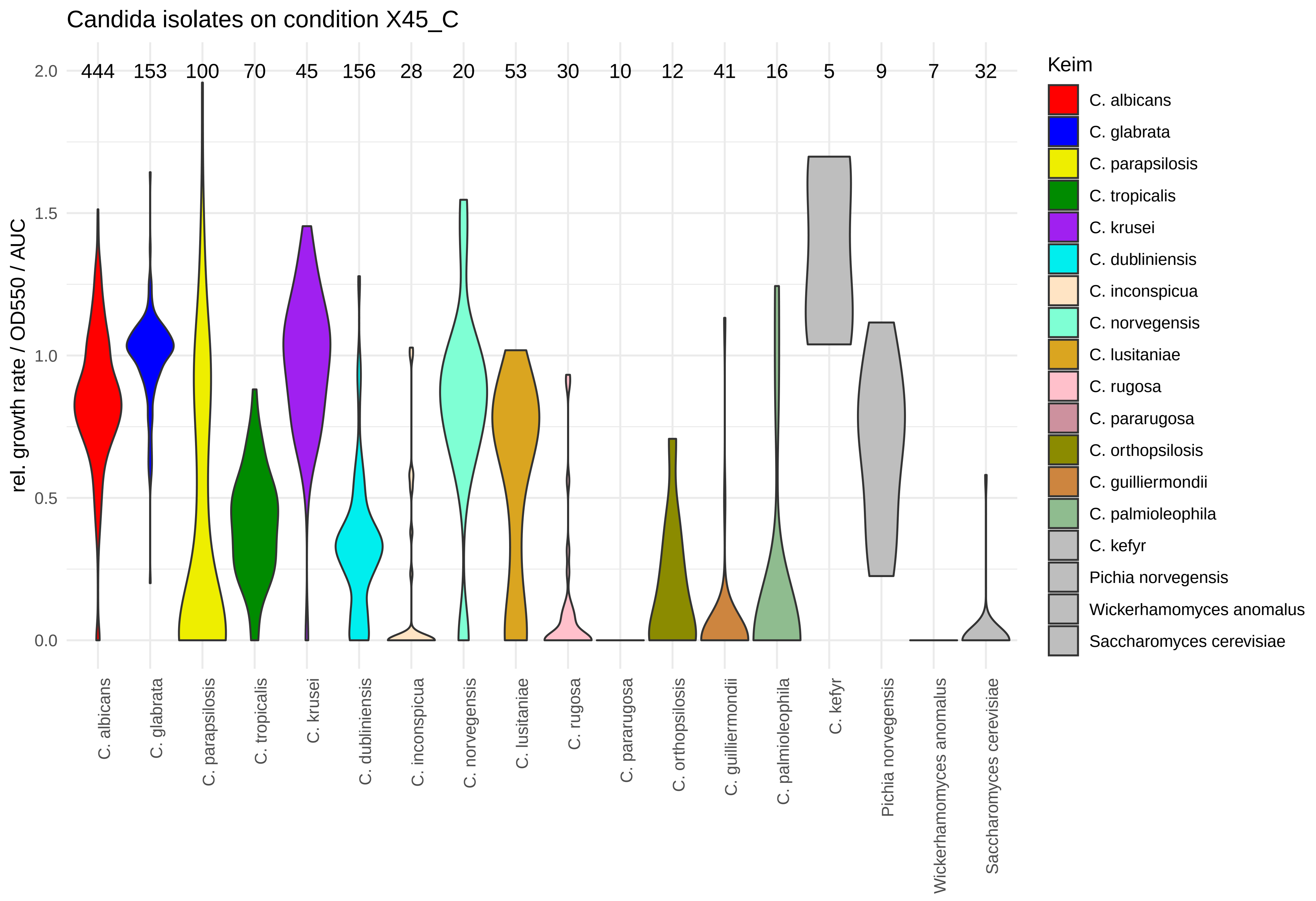
Candida isolates on condition X45_C
rel. growth rate / OD550 / AUC
2.0
1.5
1.0
0.5
0.0
444
153
100
70
45
156
28
20
53
30
10
12
41
16
5
9
7
32
C. albicans
C. glabrata
C. parapsilosis
C. tropicalis
C. krusei
C. dubliniensis
C. inconspicua
C. norvegensis
C. lusitaniae
C. rugosa
C. pararugosa
C. orthopsilosis
C. guilliermondii
C. palmioleophila
C. kefyr
Pichia norvegensis
Wickerhamomyces anomalus
Saccharomyces cerevisiae
Keim

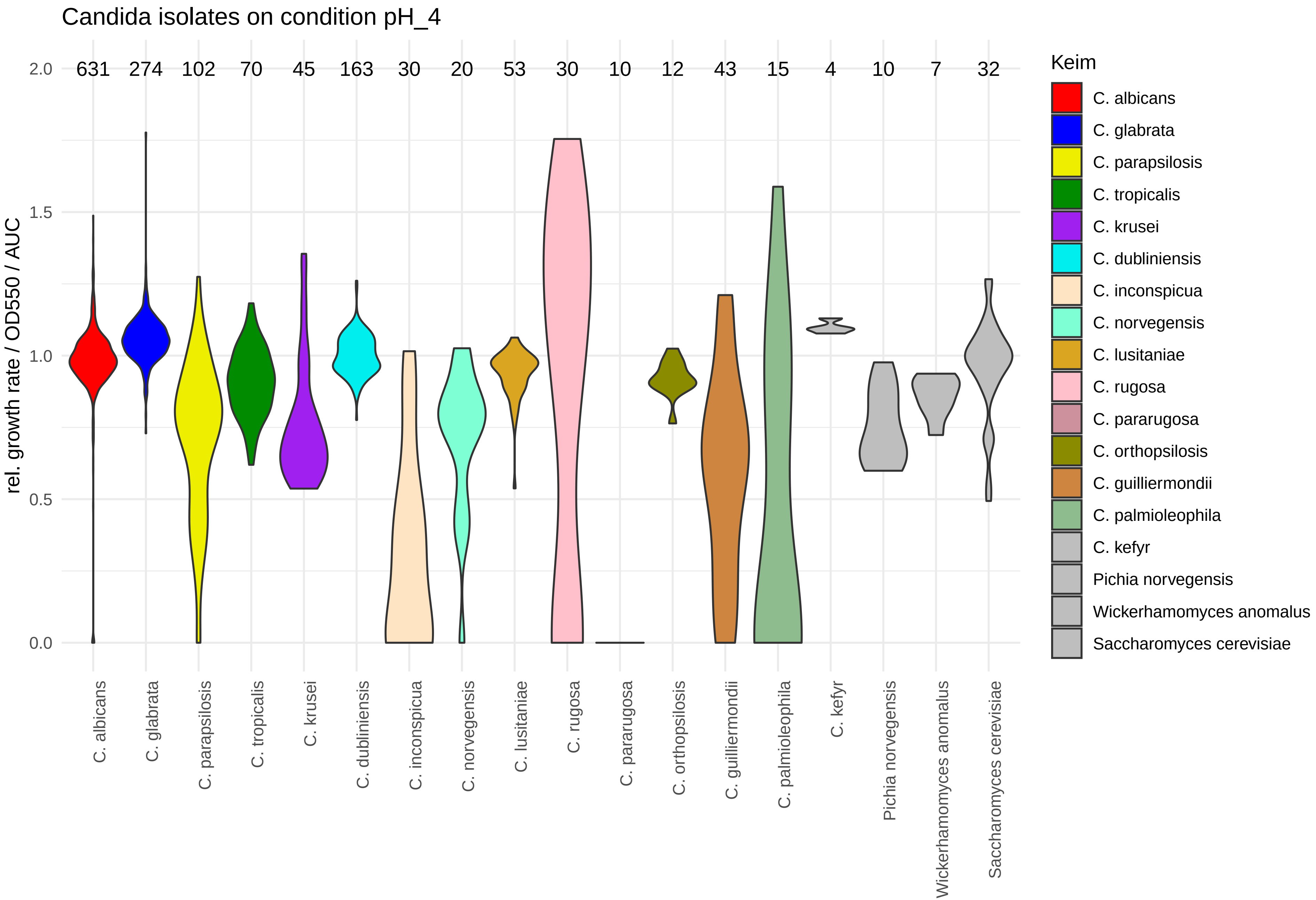

Candida isolates on condition pH_4
rel. growth rate / OD550 / AUC
2.0
1.5
1.0
0.5
0.0
631
274
102
70
45
163
30
20
53
30
10
12
43
15
4
10
7
32
C. albicans
C. glabrata
C. parapsilosis
C. tropicalis
C. krusei
C. dubliniensis
C. inconspicua
C. norvegensis
C. lusitaniae
C. rugosa
C. pararugosa
C. orthopsilosis
C. guilliermondii
C. palmioleophila
C. kefyr
Pichia norvegensis
Wickerhamomyces anomalus
Saccharomyces cerevisiae
Keim

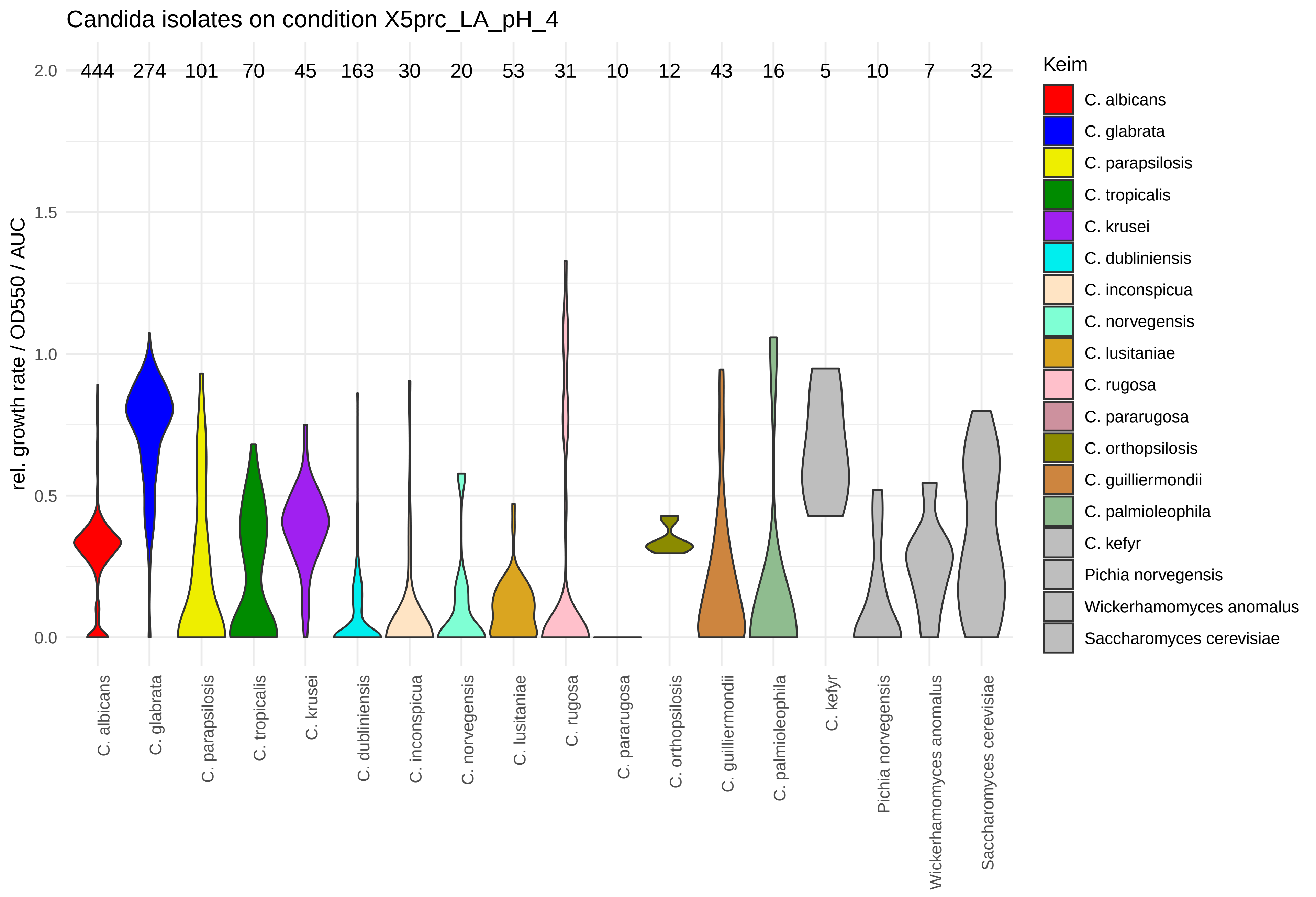

Candida isolates on condition X5prc_LA_pH_4
rel. growth rate / OD550 / AUC
2.0
1.5
1.0
0.5
0.0
444 274 101 70 45 163 30 20 53 31 10 12 43 16 5 10 7 32
C. albicans
C. glabrata
C. parapsilosis
C. tropicalis
C. krusei
C. dubliniensis
C. inconspicua
C. norvegensis
C. lusitaniae
C. rugosa
C. pararugosa
C. orthopsilosis
C. guilliermondii
C. palmioleophila
C. kefyr
Pichia norvegensis
Wickerhamomyces anomalus
Saccharomyces cerevisiae
Keim

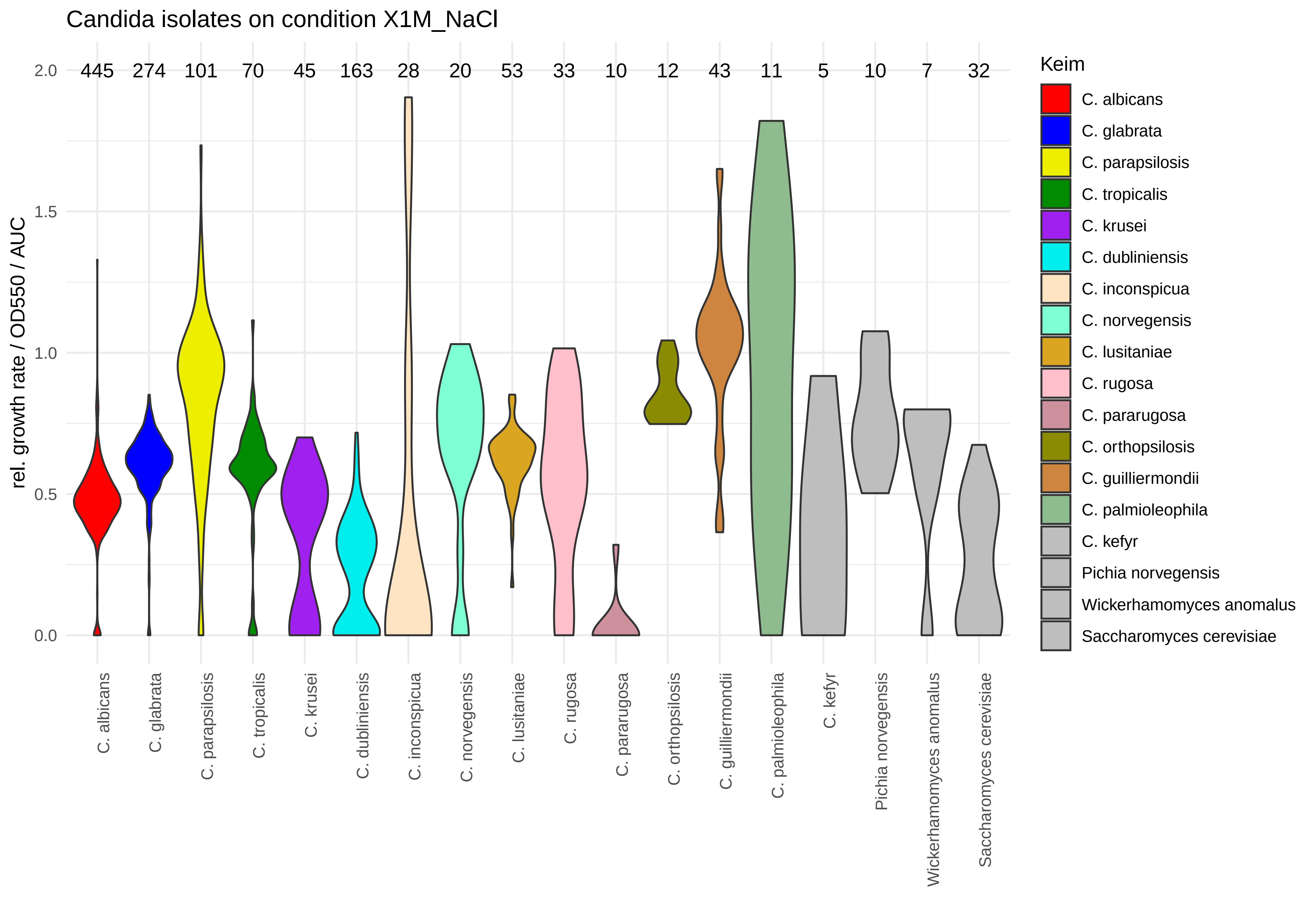

Candida isolates on condition X1M_NaCl
rel. growth rate / OD550 / AUC
2.0
1.5
1.0
0.5
0.0
445
274
101
70
45
163
28
20
53
33
10
12
43
11
5
10
7
32
C. albicans
C. glabrata
C. parapsilosis
C. tropicalis
C. krusei
C. dubliniensis
C. inconspicua
C. norvegensis
C. lusitaniae
C. rugosa
C. pararugosa
C. orthopsilosis
C. guilliermondii
C. palmioleophila
C. kefyr
Pichia norvegensis
Wickerhamomyces anomalus
Saccharomyces cerevisiae
Keim

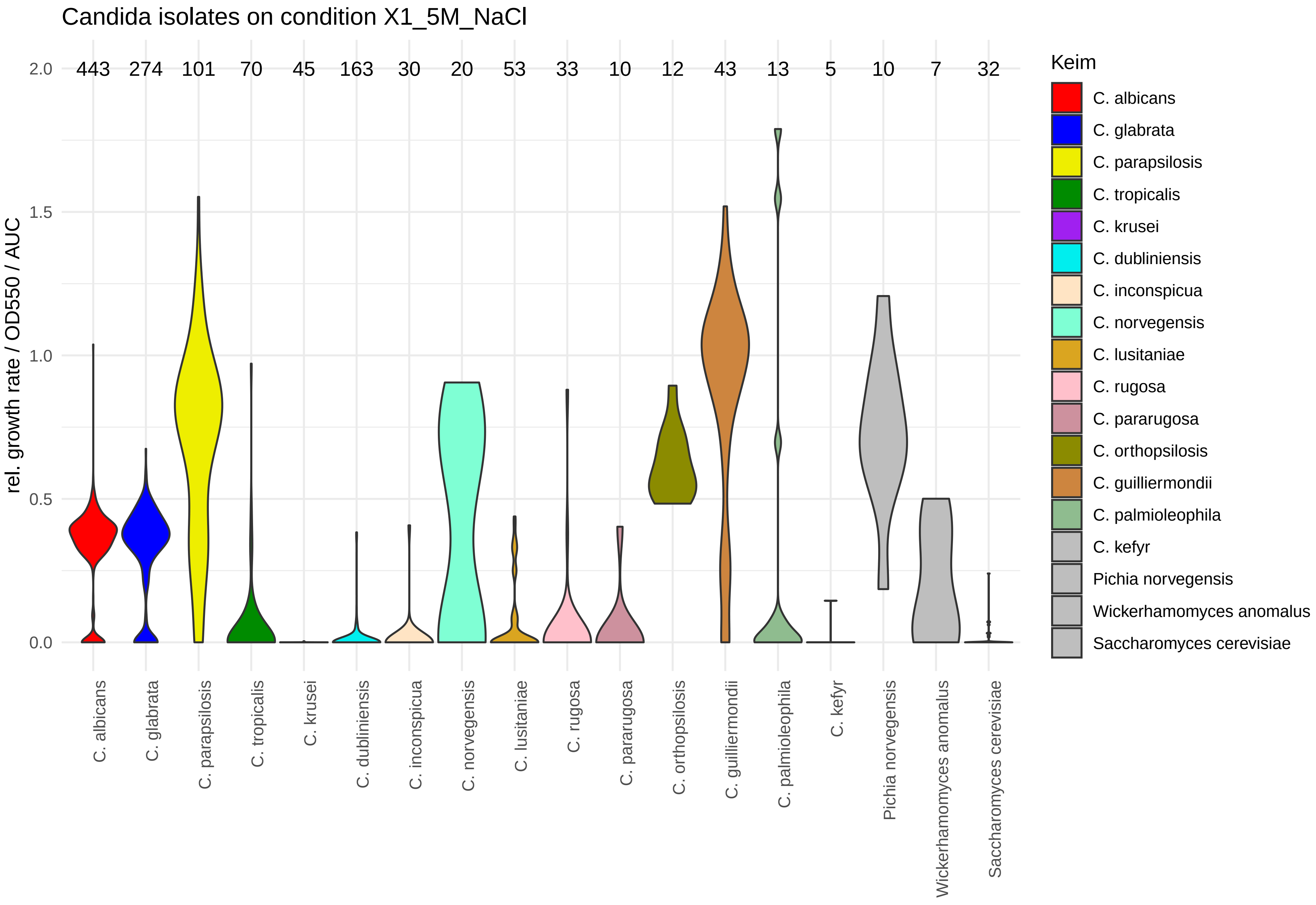

Candida isolates on condition X1_5M_NaCl
rel. growth rate / OD550 / AUC
0.0
0.5
1.0
1.5
2.0
443
274
101
70
45
163
30
20
53
33
10
12
43
13
5
10
7
32
C. albicans
C. glabrata
C. parapsilosis
C. tropicalis
C. krusei
C. dubliniensis
C. inconspicua
C. norvegensis
C. lusitaniae
C. rugosa
C. pararugosa
C. orthopsilosis
C. guilliermondii
C. palmioleophila
C. kefyr
Pichia norvegensis
Wickerhamomyces anomalus
Saccharomyces cerevisiae
Keim

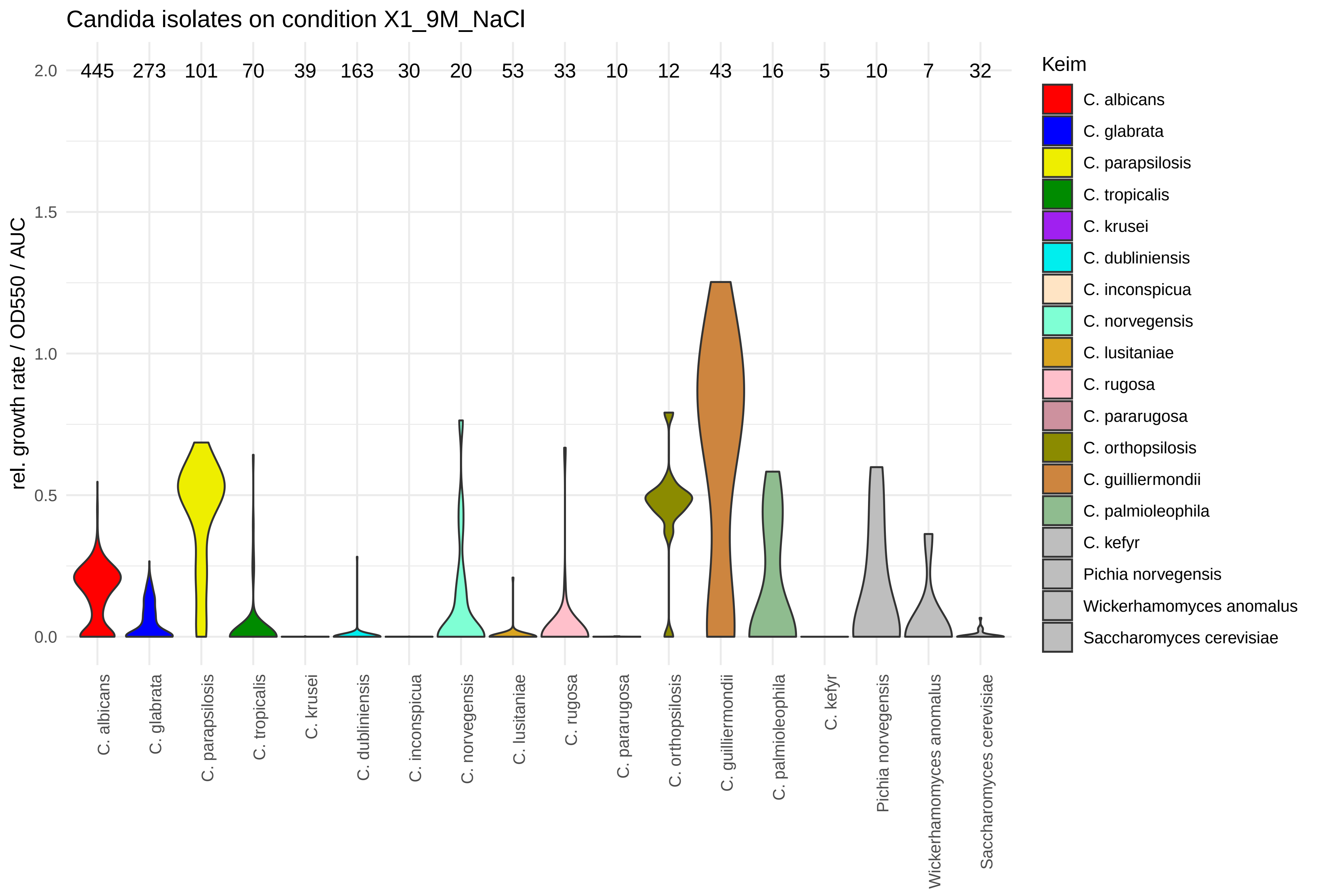
Candida isolates on condition X1_9M_NaCl
rel. growth rate / OD550 / AUC
2.0
1.5
1.0
0.5
0.0
445
273
101
70
39
163
30
20
53
33
10
12
43
16
5
10
7
32
C. albicans
C. glabrata
C. parapsilosis
C. tropicalis
C. krusei
C. dubliniensis
C. inconspicua
C. norvegensis
C. lusitaniae
C. rugosa
C. pararugosa
C. orthopsilosis
C. guilliermondii
C. palmioleophila
C. kefyr
Pichia norvegensis
Wickerhamomyces anomalus
Saccharomyces cerevisiae
Keim
C. albicans
C. glabrata
C. parapsilosis
C. tropicalis
C. krusei
C. dubliniensis
C. inconspicua
C. norvegensis
C. lusitaniae
C. rugosa
C. pararugosa
C. orthopsilosis
C. guilliermondii
C. palmioleophila
C. kefyr
Pichia norvegensis
Wickerhamomyces anomalus
Saccharomyces cerevisiae

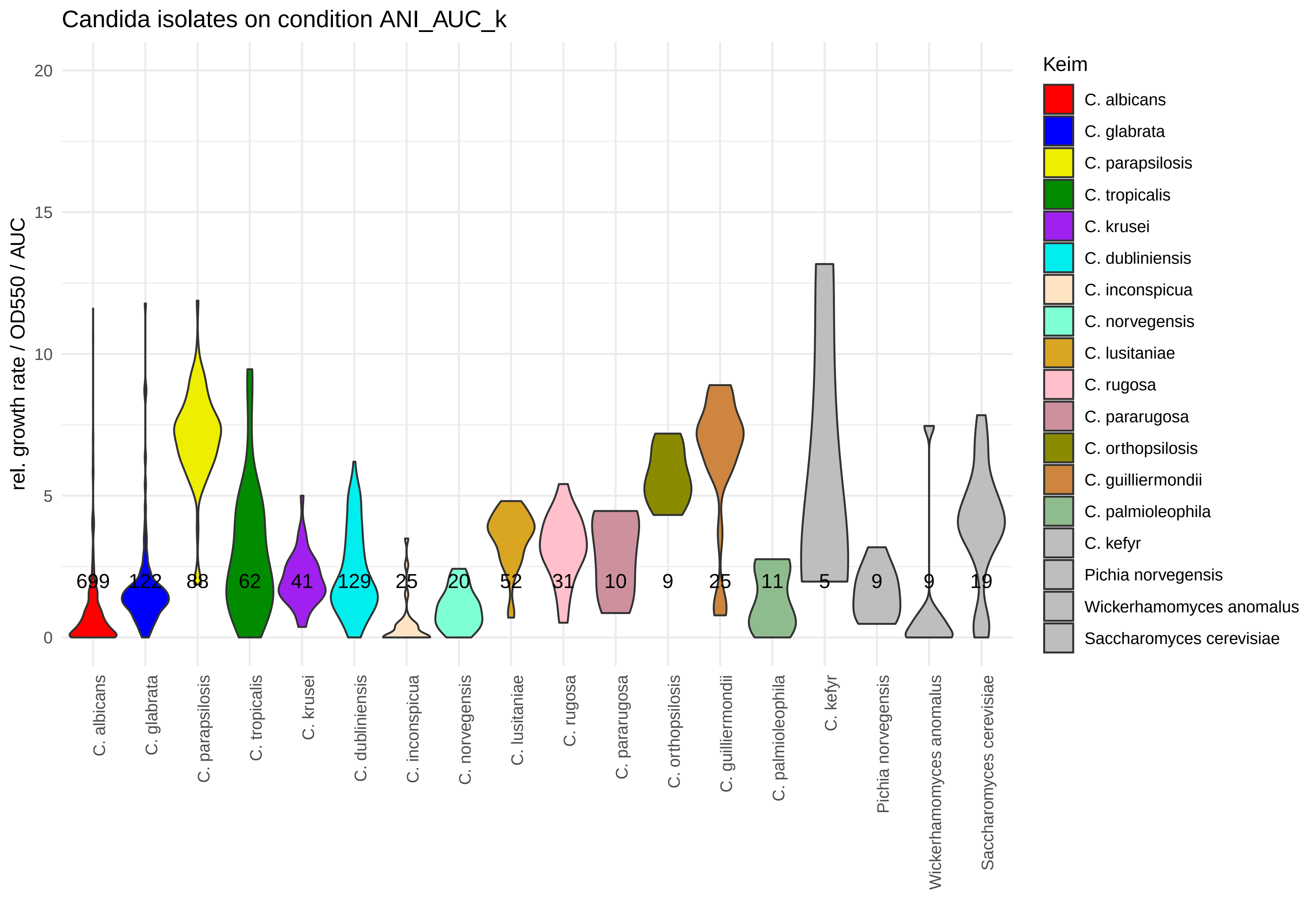
Candida isolates on condition ANI_AUC_k
rel. growth rate / OD550 / AUC
20
15
10
5
0
699
122
88
62
41
129
25
20
52
31
10
9
25
11
5
9
9
19
C. albicans
C. glabrata
C. parapsilosis
C. tropicalis
C. krusei
C. dubliniensis
C. inconspicua
C. norvegensis
C. lusitaniae
C. rugosa
C. pararugosa
C. orthopsilosis
C. guilliermondii
C. palmioleophila
C. kefyr
Pichia norvegensis
Wickerhamomyces anomalus
Saccharomyces cerevisiae
Keim
C. albicans
C. glabrata
C. parapsilosis
C. tropicalis
C. krusei
C. dubliniensis
C. inconspicua
C. norvegensis
C. lusitaniae
C. rugosa
C. pararugosa
C. orthopsilosis
C. guilliermondii
C. palmioleophila
C. kefyr
Pichia norvegensis
Wickerhamomyces anomalus
Saccharomyces cerevisiae

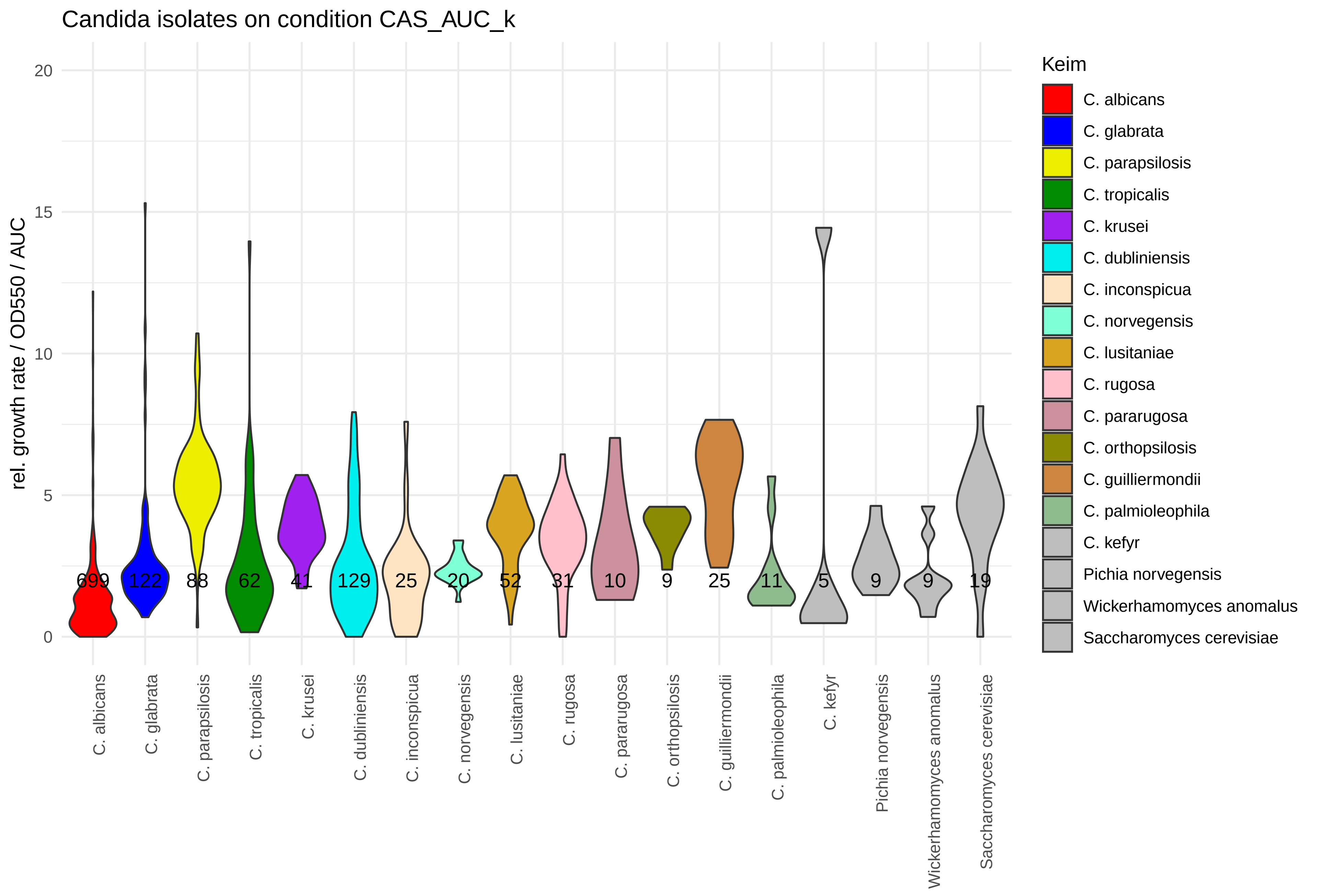

Candida isolates on condition CAS_AUC_k
rel. growth rate / OD550 / AUC
0
5
10
15
20
699
122
88
62
41
129
25
20
52
31
10
9
25
11
5
9
9
19
C. albicans
C. glabrata
C. parapsilosis
C. tropicalis
C. krusei
C. dubliniensis
C. inconspicua
C. norvegensis
C. lusitaniae
C. rugosa
C. pararugosa
C. orthopsilosis
C. guilliermondii
C. palmioleophila
C. kefyr
Pichia norvegensis
Wickerhamomyces anomalus
Saccharomyces cerevisiae
Keim
C. albicans
C. glabrata
C. parapsilosis
C. tropicalis
C. krusei
C. dubliniensis
C. inconspicua
C. norvegensis
C. lusitaniae
C. rugosa
C. pararugosa
C. orthopsilosis
C. guilliermondii
C. palmioleophila
C. kefyr
Pichia norvegensis
Wickerhamomyces anomalus
Saccharomyces cerevisiae

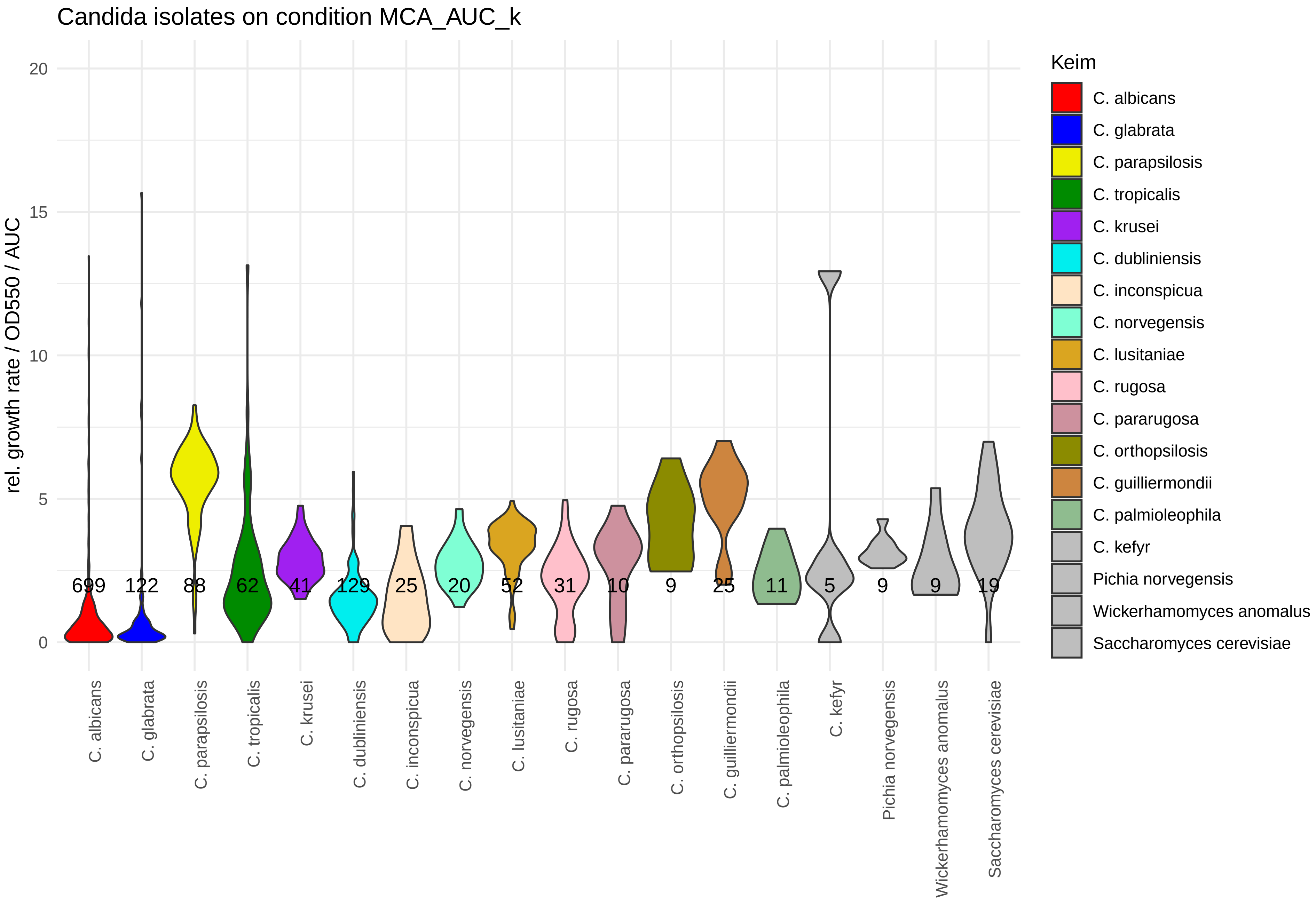
Candida isolates on condition MCA_AUC_k
rel. growth rate / OD550 / AUC
0
5
10
15
20
699
122
88
62
41
129
25
20
52
31
10
9
25
11
5
9
9
19
C. albicans
C. glabrata
C. parapsilosis
C. tropicalis
C. krusei
C. dubliniensis
C. inconspicua
C. norvegensis
C. lusitaniae
C. rugosa
C. pararugosa
C. orthopsilosis
C. guilliermondii
C. palmioleophila
C. kefyr
Pichia norvegensis
Wickerhamomyces anomalus
Saccharomyces cerevisiae
Keim

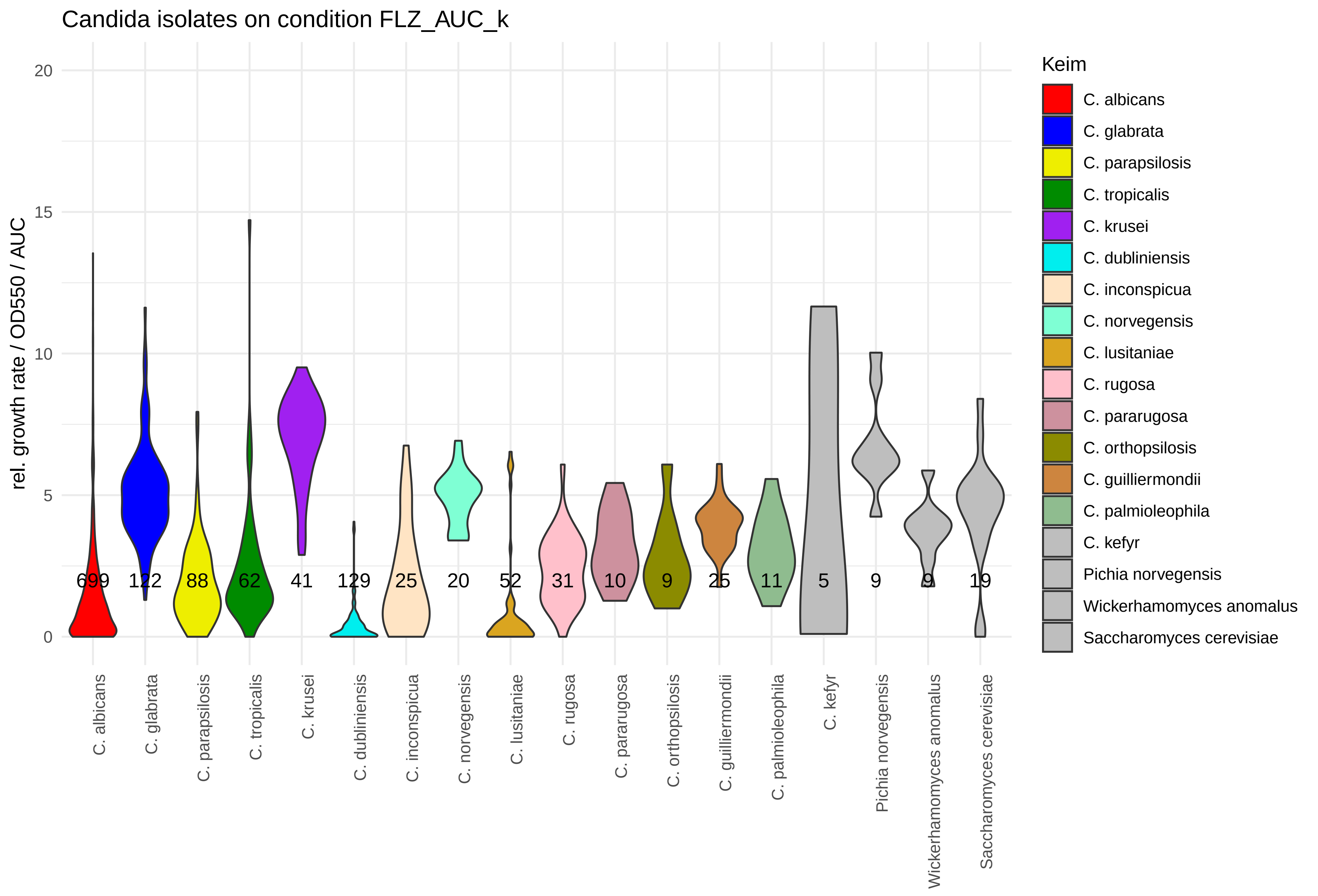
Candida isolates on condition FLZ_AUC_k
rel. growth rate / OD550 / AUC
0
5
10
15
20
699
122
88
62
41
129
25
20
52
31
10
9
25
11
5
9
9
19
C. albicans
C. glabrata
C. parapsilosis
C. tropicalis
C. krusei
C. dubliniensis
C. inconspicua
C. norvegensis
C. lusitaniae
C. rugosa
C. pararugosa
C. orthopsilosis
C. guilliermondii
C. palmioleophila
C. kefyr
Pichia norvegensis
Wickerhamomyces anomalus
Saccharomyces cerevisiae
Keim
C. albicans
C. glabrata
C. parapsilosis
C. tropicalis
C. krusei
C. dubliniensis
C. inconspicua
C. norvegensis
C. lusitaniae
C. rugosa
C. pararugosa
C. orthopsilosis
C. guilliermondii
C. palmioleophila
C. kefyr
Pichia norvegensis
Wickerhamomyces anomalus
Saccharomyces cerevisiae

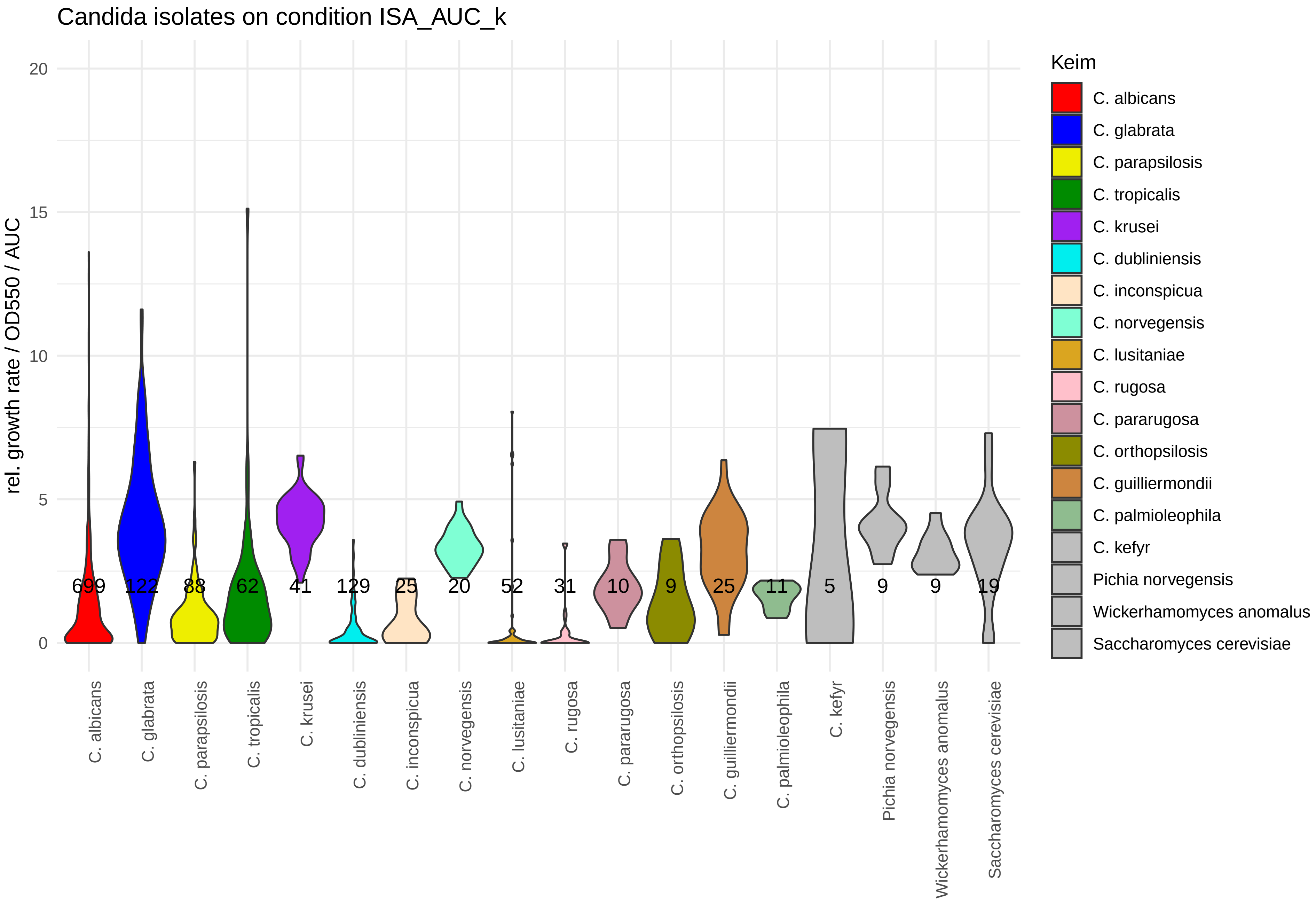

Candida isolates on condition ISA_AUC_k
rel. growth rate / OD550 / AUC
20
15
10
5
0
699
122
88
62
41
129
25
20
52
31
10
9
25
11
5
9
9
19
C. albicans
C. glabrata
C. parapsilosis
C. tropicalis
C. krusei
C. dubliniensis
C. inconspicua
C. norvegensis
C. lusitaniae
C. rugosa
C. pararugosa
C. orthopsilosis
C. guilliermondii
C. palmioleophila
C. kefyr
Pichia norvegensis
Wickerhamomyces anomalus
Saccharomyces cerevisiae
Keim
C. albicans
C. glabrata
C. parapsilosis
C. tropicalis
C. krusei
C. dubliniensis
C. inconspicua
C. norvegensis
C. lusitaniae
C. rugosa
C. pararugosa
C. orthopsilosis
C. guilliermondii
C. palmioleophila
C. kefyr
Pichia norvegensis
Wickerhamomyces anomalus
Saccharomyces cerevisiae

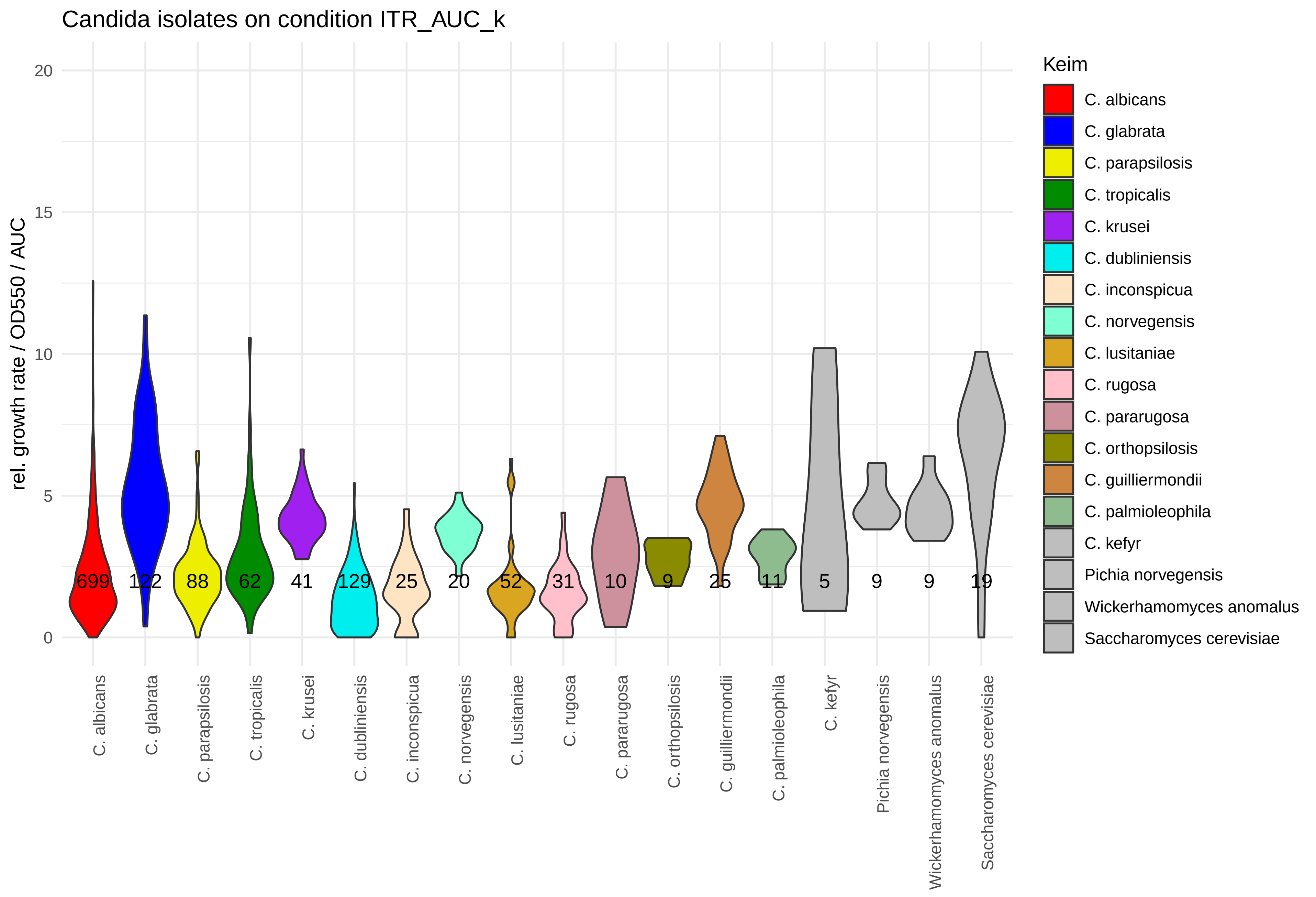

Candida isolates on condition ITR_AUC_k
rel. growth rate / OD550 / AUC
20
15
10
5
0
699
122
88
62
41
129
25
20
52
31
10
9
25
11
5
9
9
19
C. albicans
C. glabrata
C. parapsilosis
C. tropicalis
C. krusei
C. dubliniensis
C. inconspicua
C. norvegensis
C. lusitaniae
C. rugosa
C. pararugosa
C. orthopsilosis
C. guilliermondii
C. palmioleophila
C. kefyr
Pichia norvegensis
Wickerhamomyces anomalus
Saccharomyces cerevisiae
Keim
C. albicans
C. glabrata
C. parapsilosis
C. tropicalis
C. krusei
C. dubliniensis
C. inconspicua
C. norvegensis
C. lusitaniae
C. rugosa
C. pararugosa
C. orthopsilosis
C. guilliermondii
C. palmioleophila
C. kefyr
Pichia norvegensis
Wickerhamomyces anomalus
Saccharomyces cerevisiae

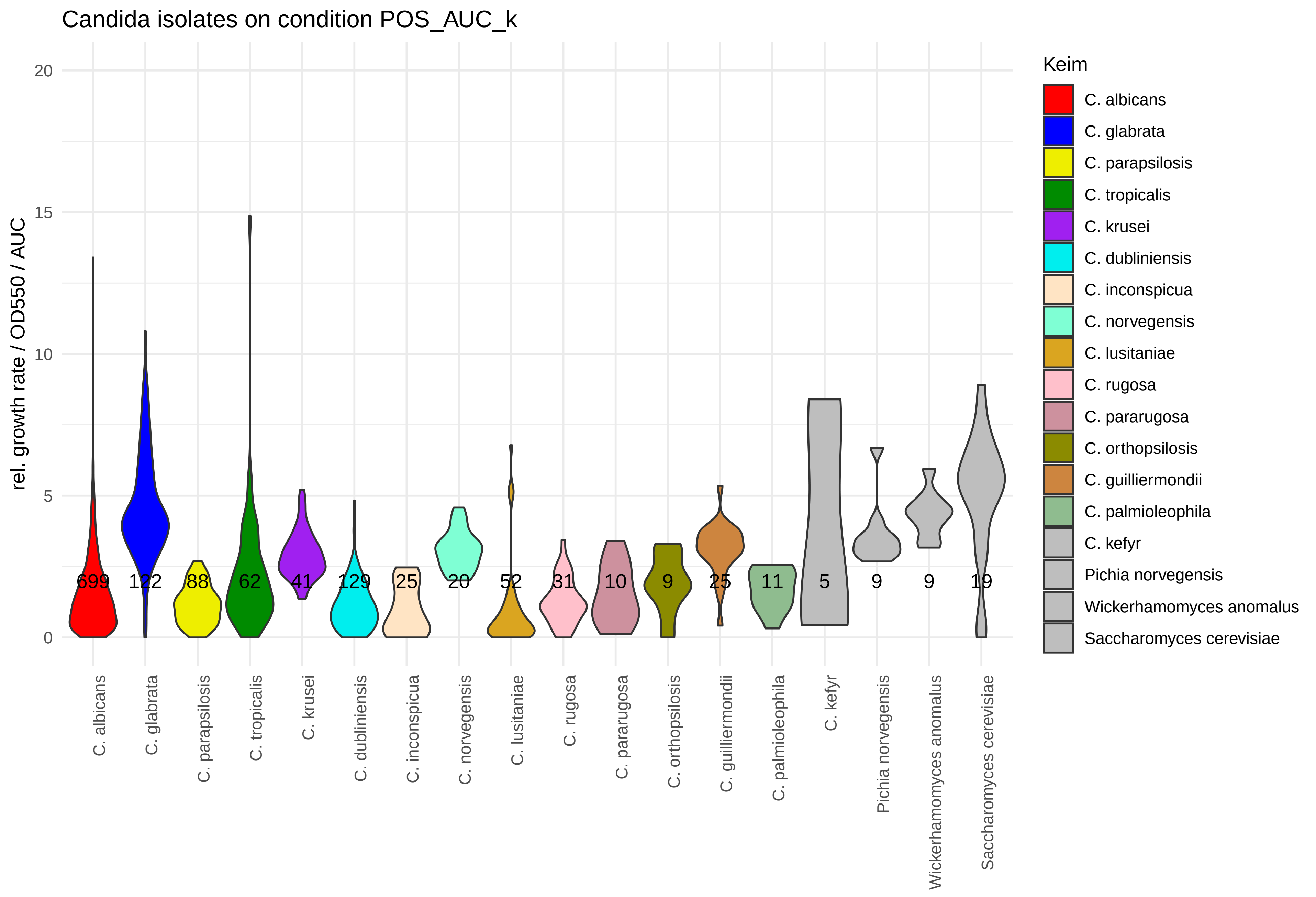
Candida isolates on condition POS_AUC_k
rel. growth rate / OD550 / AUC
20
15
10
5
0
699
122
88
62
41
129
25
20
52
31
10
9
25
11
5
9
9
19
C. albicans
C. glabrata
C. parapsilosis
C. tropicalis
C. krusei
C. dubliniensis
C. inconspicua
C. norvegensis
C. lusitaniae
C. rugosa
C. pararugosa
C. orthopsilosis
C. guilliermondii
C. palmioleophila
C. kefyr
Pichia norvegensis
Wickerhamomyces anomalus
Saccharomyces cerevisiae
Keim
C. albicans
C. glabrata
C. parapsilosis
C. tropicalis
C. krusei
C. dubliniensis
C. inconspicua
C. norvegensis
C. lusitaniae
C. rugosa
C. pararugosa
C. orthopsilosis
C. guilliermondii
C. palmioleophila
C. kefyr
Pichia norvegensis
Wickerhamomyces anomalus
Saccharomyces cerevisiae

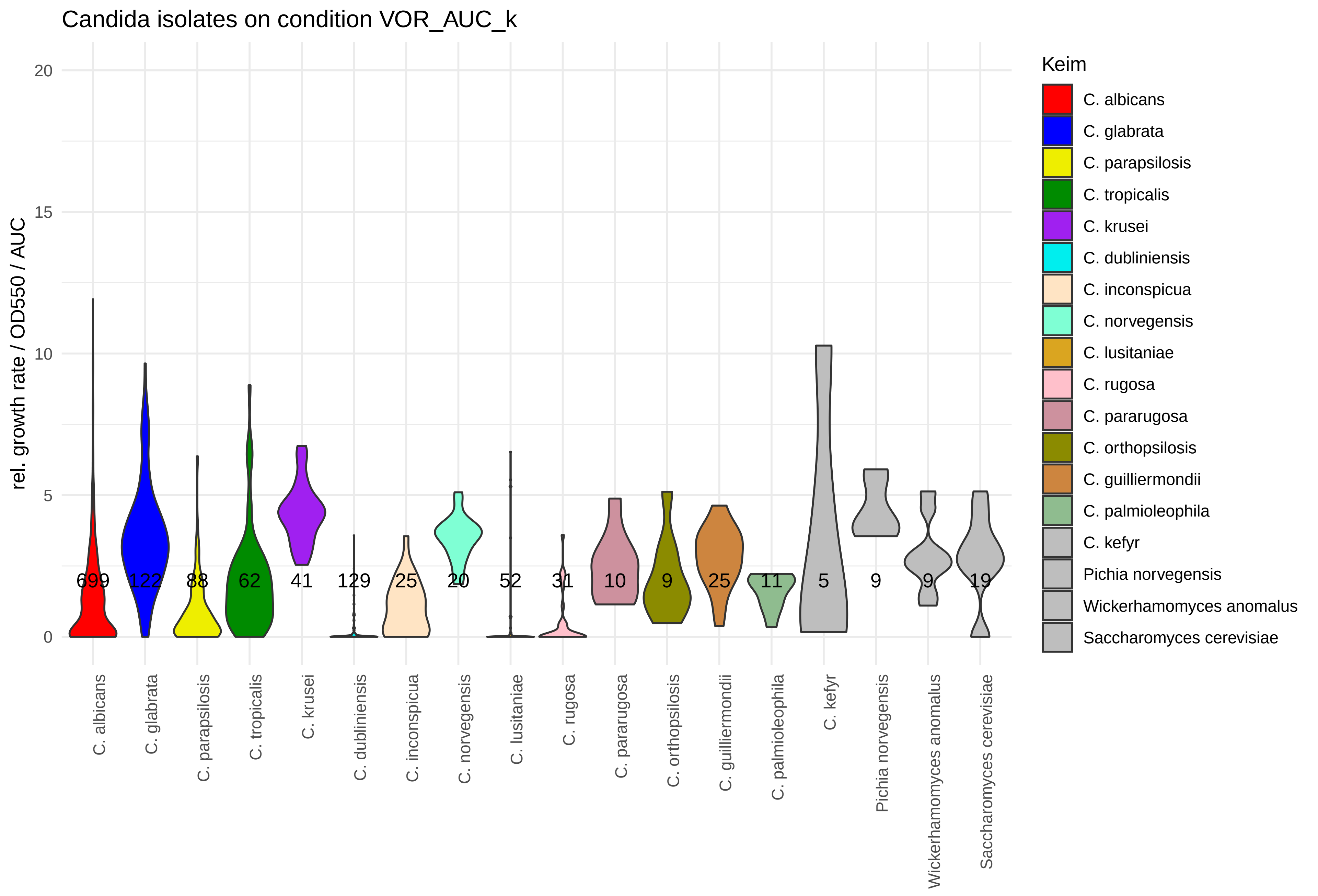
Candida isolates on condition VOR_AUC_k
rel. growth rate / OD550 / AUC
20
15
10
5
0
699
122
88
62
41
129
25
20
52
31
10
9
25
11
5
9
9
19
C. albicans
C. glabrata
C. parapsilosis
C. tropicalis
C. krusei
C. dubliniensis
C. inconspicua
C. norvegensis
C. lusitaniae
C. rugosa
C. pararugosa
C. orthopsilosis
C. guilliermondii
C. palmioleophila
C. kefyr
Pichia norvegensis
Wickerhamomyces anomalus
Saccharomyces cerevisiae
Keim
C. albicans
C. glabrata
C. parapsilosis
C. tropicalis
C. krusei
C. dubliniensis
C. inconspicua
C. norvegensis
C. lusitaniae
C. rugosa
C. pararugosa
C. orthopsilosis
C. guilliermondii
C. palmioleophila
C. kefyr
Pichia norvegensis
Wickerhamomyces anomalus
Saccharomyces cerevisiae

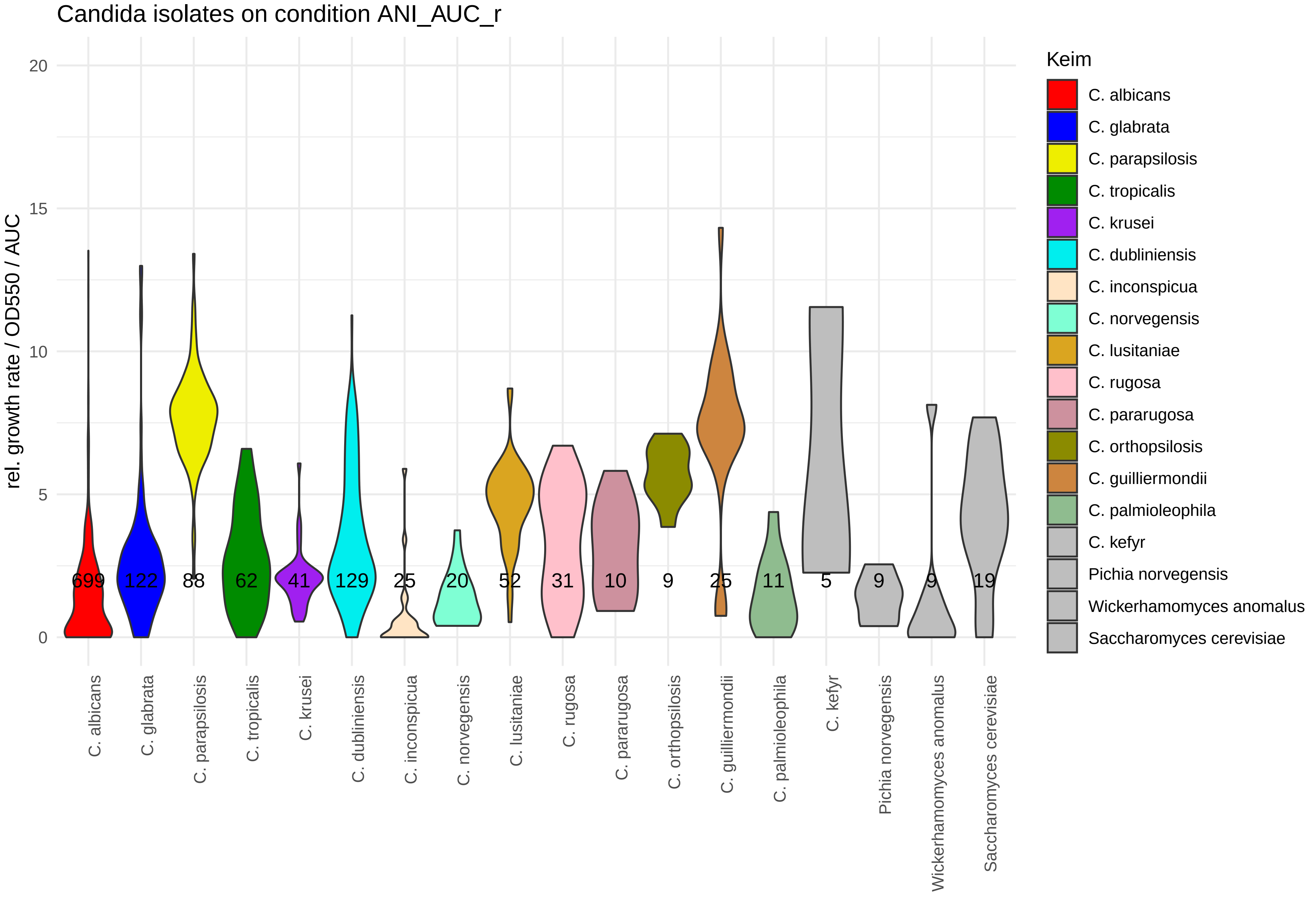

Candida isolates on condition ANI_AUC_r
rel. growth rate / OD550 / AUC
0
5
10
15
20
699
122
88
62
41
129
25
20
52
31
10
9
25
11
5
9
9
19
C. albicans
C. glabrata
C. parapsilosis
C. tropicalis
C. krusei
C. dubliniensis
C. inconspicua
C. norvegensis
C. lusitaniae
C. rugosa
C. pararugosa
C. orthopsilosis
C. guilliermondii
C. palmioleophila
C. kefyr
Pichia norvegensis
Wickerhamomyces anomalus
Saccharomyces cerevisiae
Keim
C. albicans
C. glabrata
C. parapsilosis
C. tropicalis
C. krusei
C. dubliniensis
C. inconspicua
C. norvegensis
C. lusitaniae
C. rugosa
C. pararugosa
C. orthopsilosis
C. guilliermondii
C. palmioleophila
C. kefyr
Pichia norvegensis
Wickerhamomyces anomalus
Saccharomyces cerevisiae

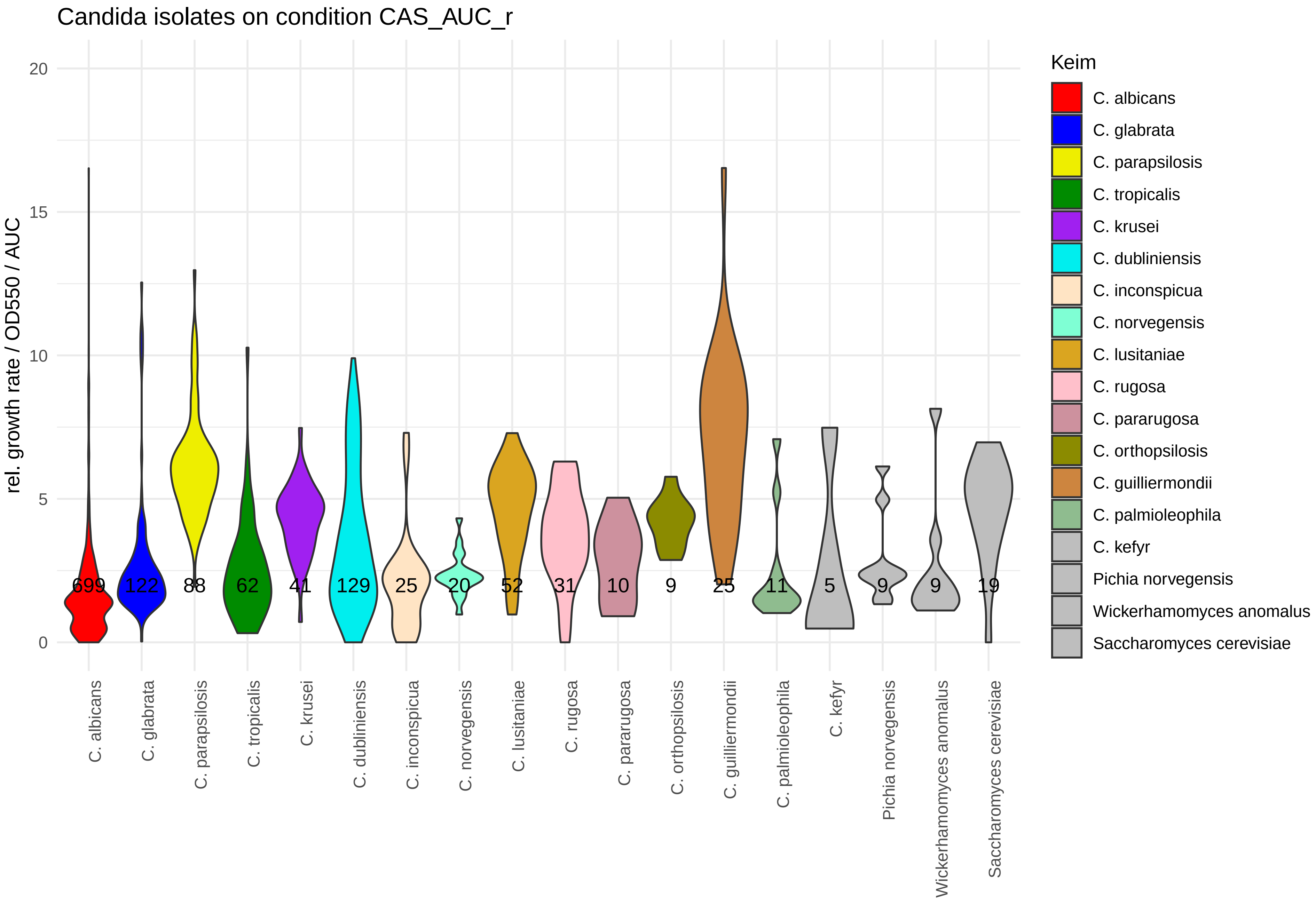

Candida isolates on condition CAS_AUC_r
rel. growth rate / OD550 / AUC
20
15
10
5
0
699
122
88
62
41
129
25
20
52
31
10
9
25
11
5
9
9
19
C. albicans
C. glabrata
C. parapsilosis
C. tropicalis
C. krusei
C. dubliniensis
C. inconspicua
C. norvegensis
C. lusitaniae
C. rugosa
C. pararugosa
C. orthopsilosis
C. guilliermondii
C. palmioleophila
C. kefyr
Pichia norvegensis
Wickerhamomyces anomalus
Saccharomyces cerevisiae
Keim
C. albicans
C. glabrata
C. parapsilosis
C. tropicalis
C. krusei
C. dubliniensis
C. inconspicua
C. norvegensis
C. lusitaniae
C. rugosa
C. pararugosa
C. orthopsilosis
C. guilliermondii
C. palmioleophila
C. kefyr
Pichia norvegensis
Wickerhamomyces anomalus
Saccharomyces cerevisiae

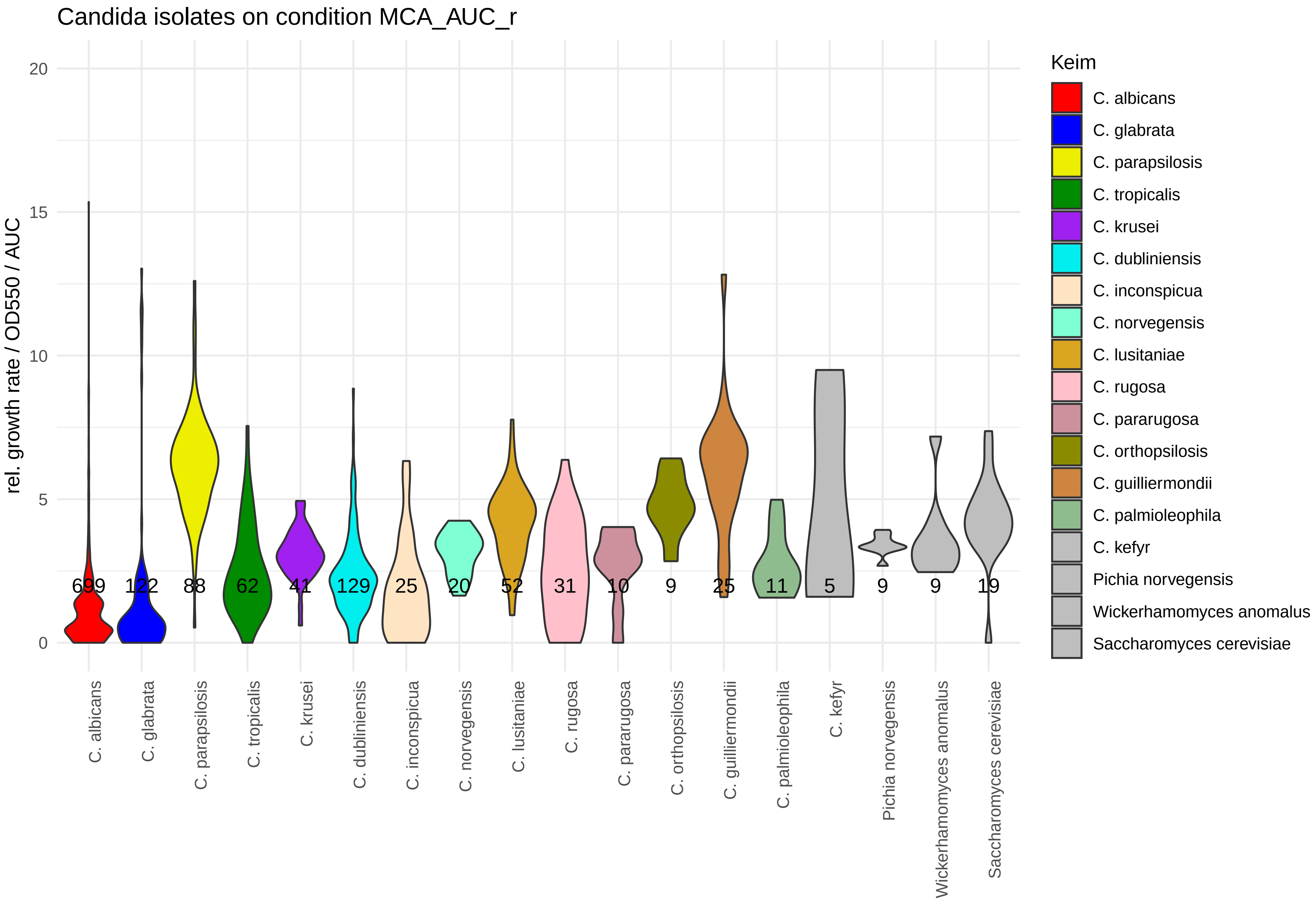
Candida isolates on condition MCA_AUC_r
rel. growth rate / OD550 / AUC
0
5
10
15
20
699
122
88
62
41
129
25
20
52
31
10
9
25
11
5
9
9
19
C. albicans
C. glabrata
C. parapsilosis
C. tropicalis
C. krusei
C. dubliniensis
C. inconspicua
C. norvegensis
C. lusitaniae
C. rugosa
C. pararugosa
C. orthopsilosis
C. guilliermondii
C. palmioleophila
C. kefyr
Pichia norvegensis
Wickerhamomyces anomalus
Saccharomyces cerevisiae
Keim
C. albicans
C. glabrata
C. parapsilosis
C. tropicalis
C. krusei
C. dubliniensis
C. inconspicua
C. norvegensis
C. lusitaniae
C. rugosa
C. pararugosa
C. orthopsilosis
C. guilliermondii
C. palmioleophila
C. kefyr
Pichia norvegensis
Wickerhamomyces anomalus
Saccharomyces cerevisiae

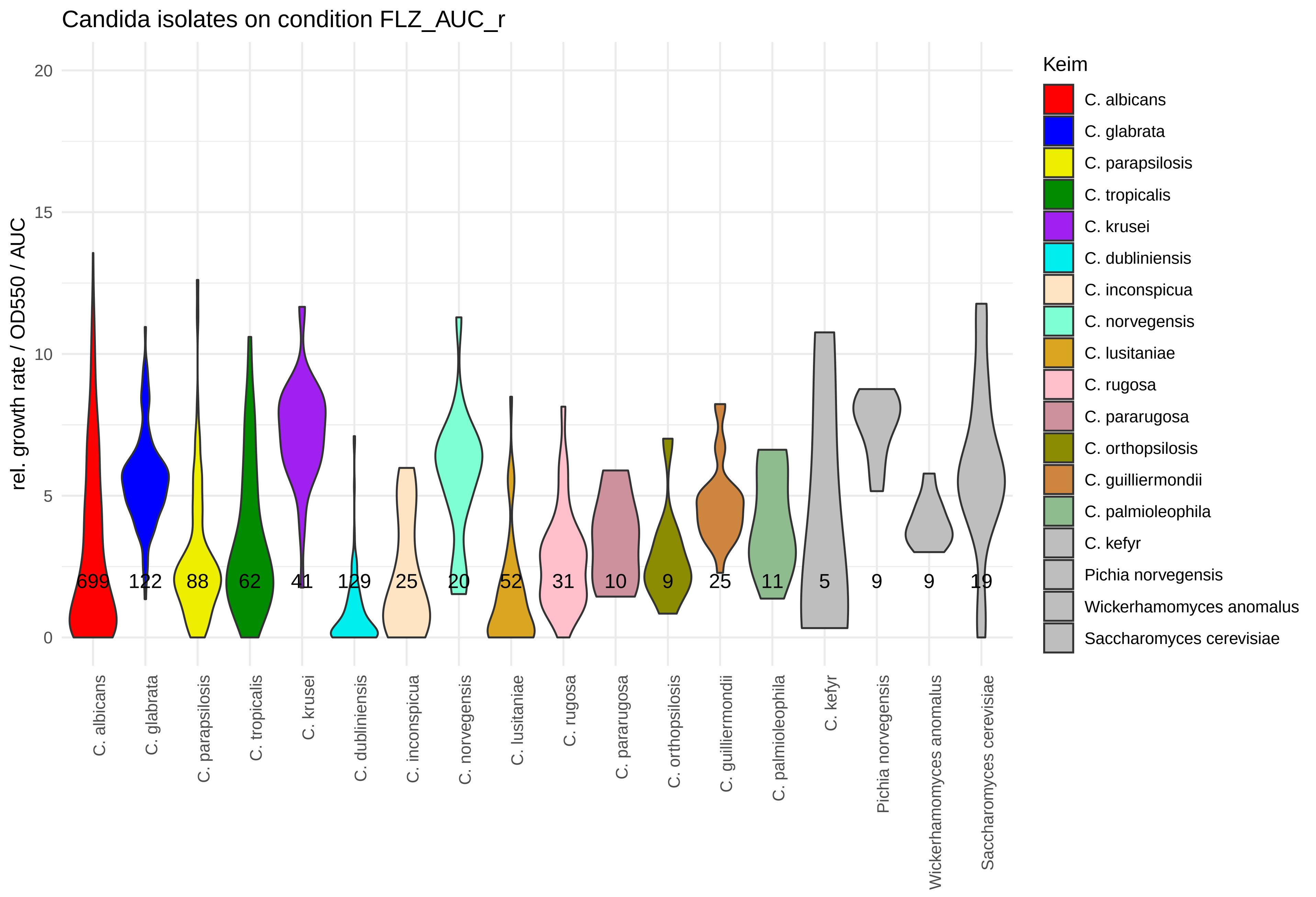

Candida isolates on condition FLZ_AUC_r
rel. growth rate / OD550 / AUC
20
15
10
5
0
699
122
88
62
41
129
25
20
52
31
10
9
25
11
5
9
9
19
C. albicans
C. glabrata
C. parapsilosis
C. tropicalis
C. krusei
C. dubliniensis
C. inconspicua
C. norvegensis
C. lusitaniae
C. rugosa
C. pararugosa
C. orthopsilosis
C. guilliermondii
C. palmioleophila
C. kefyr
Pichia norvegensis
Wickerhamomyces anomalus
Saccharomyces cerevisiae
Keim

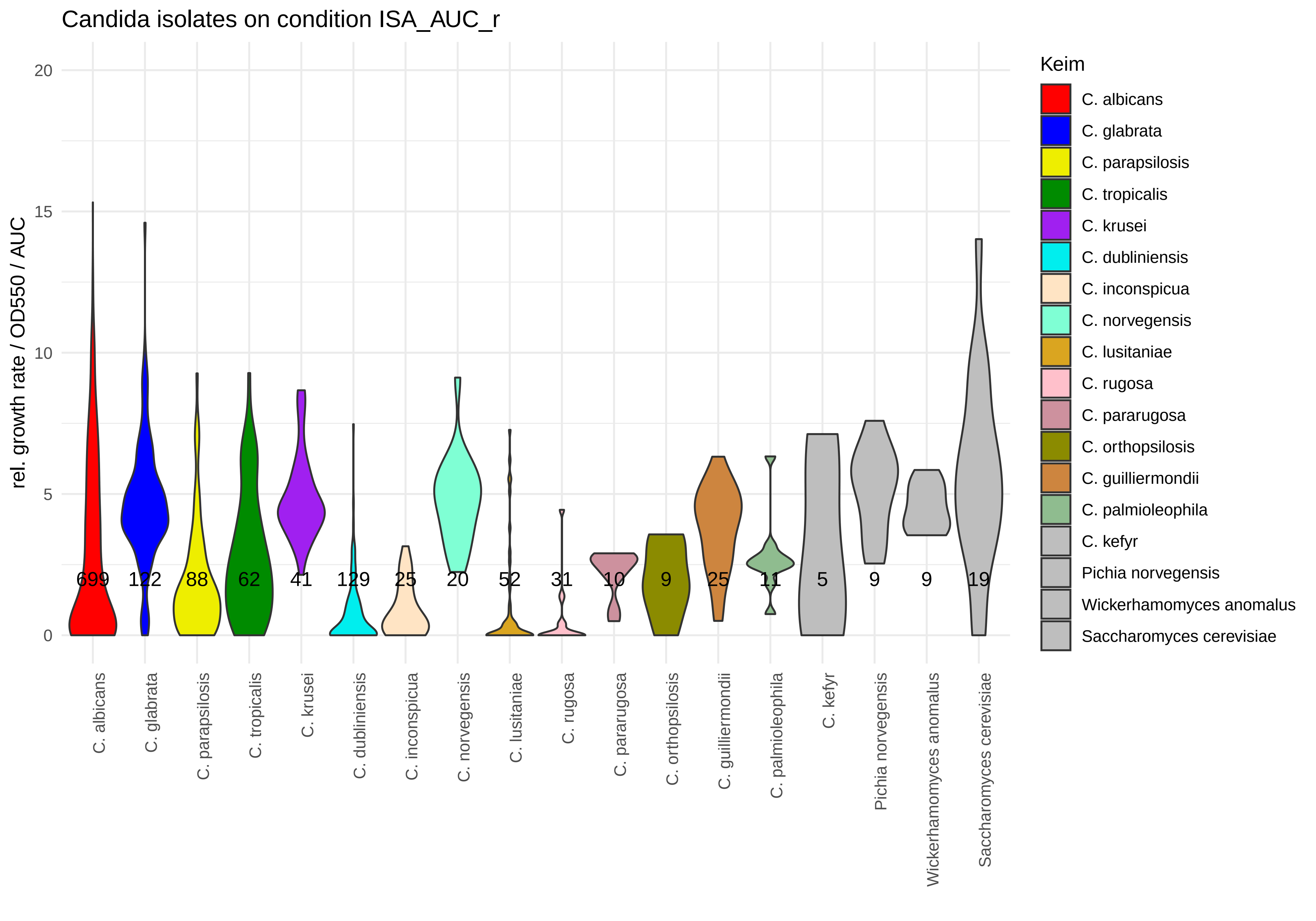

Candida isolates on condition ISA_AUC_r
rel. growth rate / OD550 / AUC
20
15
10
5
0
699
122
88
62
41
129
25
20
52
31
10
9
25
11
5
9
9
19
C. albicans
C. glabrata
C. parapsilosis
C. tropicalis
C. krusei
C. dubliniensis
C. inconspicua
C. norvegensis
C. lusitaniae
C. rugosa
C. pararugosa
C. orthopsilosis
C. guilliermondii
C. palmioleophila
C. kefyr
Pichia norvegensis
Wickerhamomyces anomalus
Saccharomyces cerevisiae
Keim
C. albicans
C. glabrata
C. parapsilosis
C. tropicalis
C. krusei
C. dubliniensis
C. inconspicua
C. norvegensis
C. lusitaniae
C. rugosa
C. pararugosa
C. orthopsilosis
C. guilliermondii
C. palmioleophila
C. kefyr
Pichia norvegensis
Wickerhamomyces anomalus
Saccharomyces cerevisiae

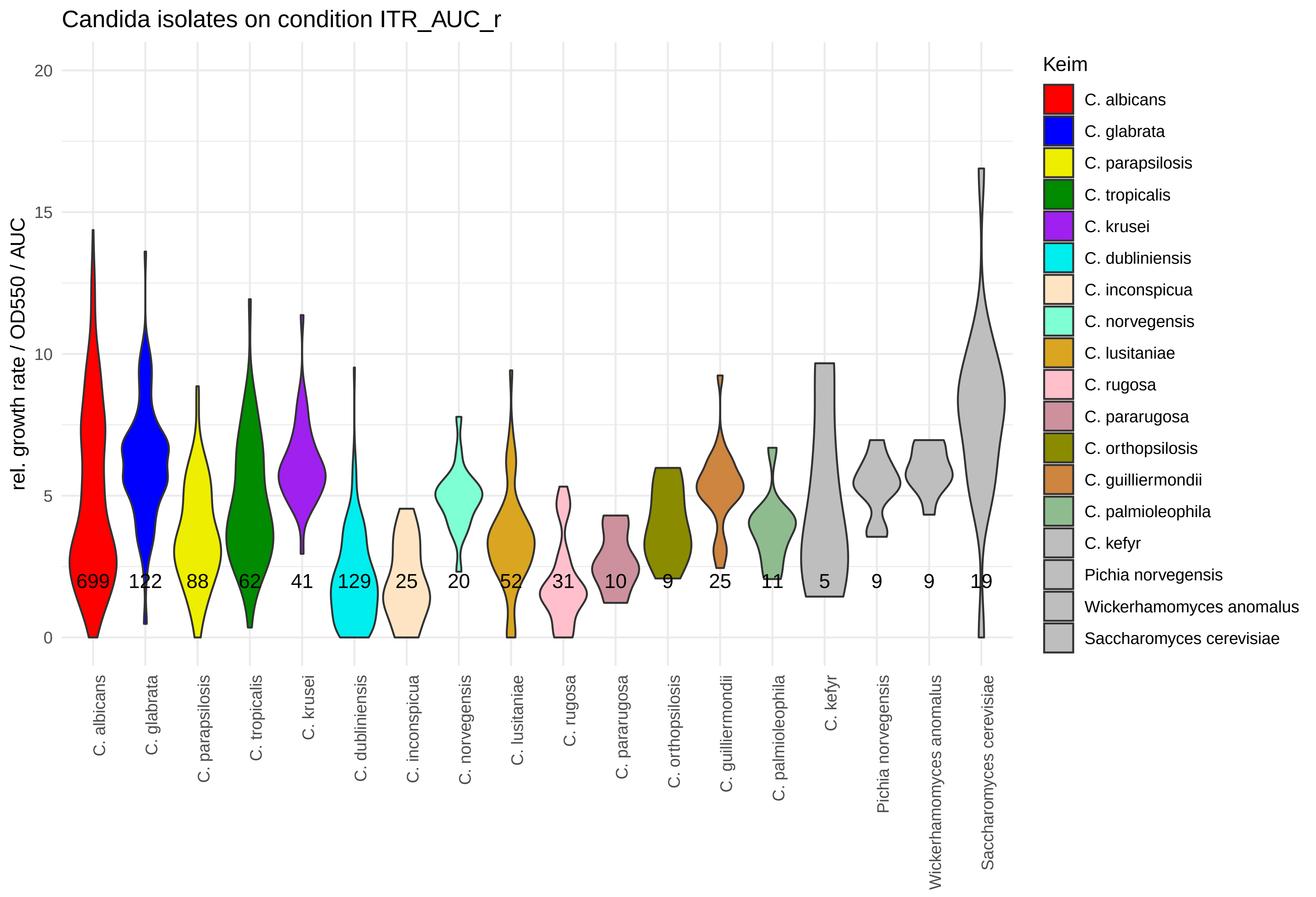

Candida isolates on condition ITR_AUC_r
rel. growth rate / OD550 / AUC
20
15
10
5
0
699
122
88
62
41
129
25
20
52
31
10
9
25
11
5
9
9
19
C. albicans
C. glabrata
C. parapsilosis
C. tropicalis
C. krusei
C. dubliniensis
C. inconspicua
C. norvegensis
C. lusitaniae
C. rugosa
C. pararugosa
C. orthopsilosis
C. guilliermondii
C. palmioleophila
C. kefyr
Pichia norvegensis
Wickerhamomyces anomalus
Saccharomyces cerevisiae
Keim
C. albicans
C. glabrata
C. parapsilosis
C. tropicalis
C. krusei
C. dubliniensis
C. inconspicua
C. norvegensis
C. lusitaniae
C. rugosa
C. pararugosa
C. orthopsilosis
C. guilliermondii
C. palmioleophila
C. kefyr
Pichia norvegensis
Wickerhamomyces anomalus
Saccharomyces cerevisiae

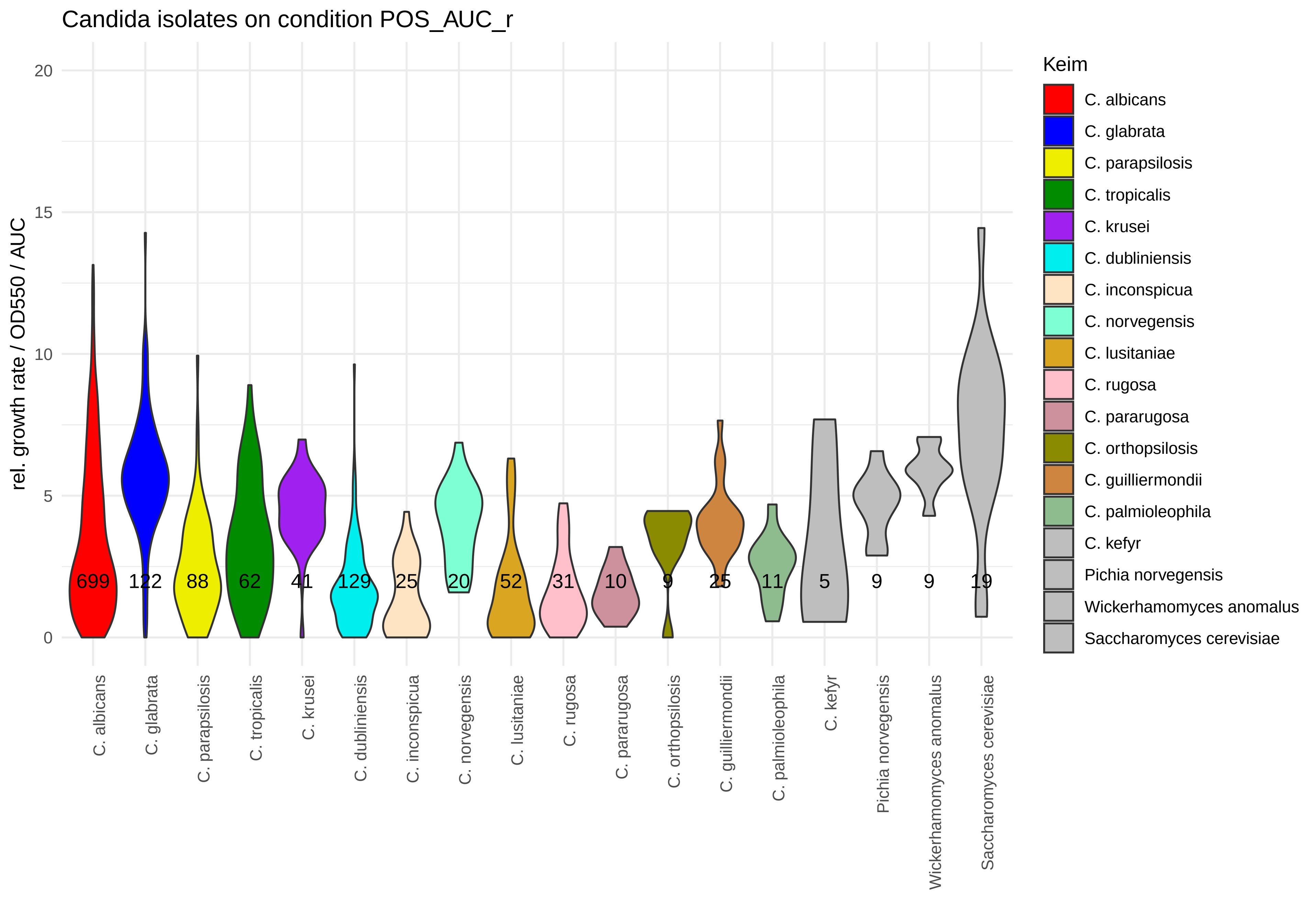

Candida isolates on condition POS_AUC_r
rel. growth rate / OD550 / AUC
20
15
10
5
0
699
122
88
62
41
129
25
20
52
31
10
9
25
11
5
9
9
19
C. albicans
C. glabrata
C. parapsilosis
C. tropicalis
C. krusei
C. dubliniensis
C. inconspicua
C. norvegensis
C. lusitaniae
C. rugosa
C. pararugosa
C. orthopsilosis
C. guilliermondii
C. palmioleophila
C. kefyr
Pichia norvegensis
Wickerhamomyces anomalus
Saccharomyces cerevisiae
Keim
C. albicans
C. glabrata
C. parapsilosis
C. tropicalis
C. krusei
C. dubliniensis
C. inconspicua
C. norvegensis
C. lusitaniae
C. rugosa
C. pararugosa
C. orthopsilosis
C. guilliermondii
C. palmioleophila
C. kefyr
Pichia norvegensis
Wickerhamomyces anomalus
Saccharomyces cerevisiae

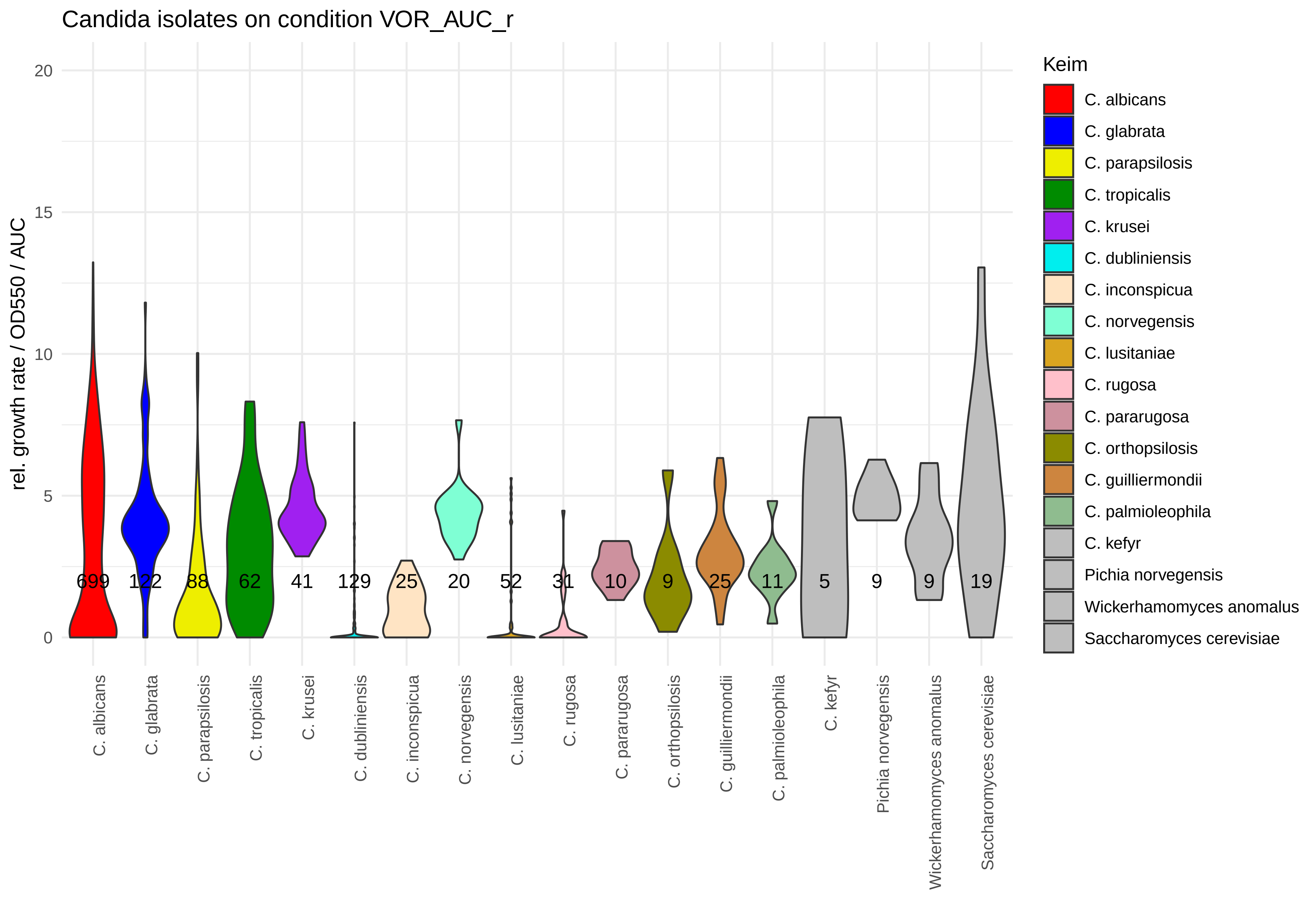

Candida isolates on condition VOR_AUC_r
rel. growth rate / OD550 / AUC
0
5
10
15
20
699
122
88
62
41
129
25
20
52
31
10
9
25
11
5
9
9
19
C. albicans
C. glabrata
C. parapsilosis
C. tropicalis
C. krusei
C. dubliniensis
C. inconspicua
C. norvegensis
C. lusitaniae
C. rugosa
C. pararugosa
C. orthopsilosis
C. guilliermondii
C. palmioleophila
C. kefyr
Pichia norvegensis
Wickerhamomyces anomalus
Saccharomyces cerevisiae
Keim

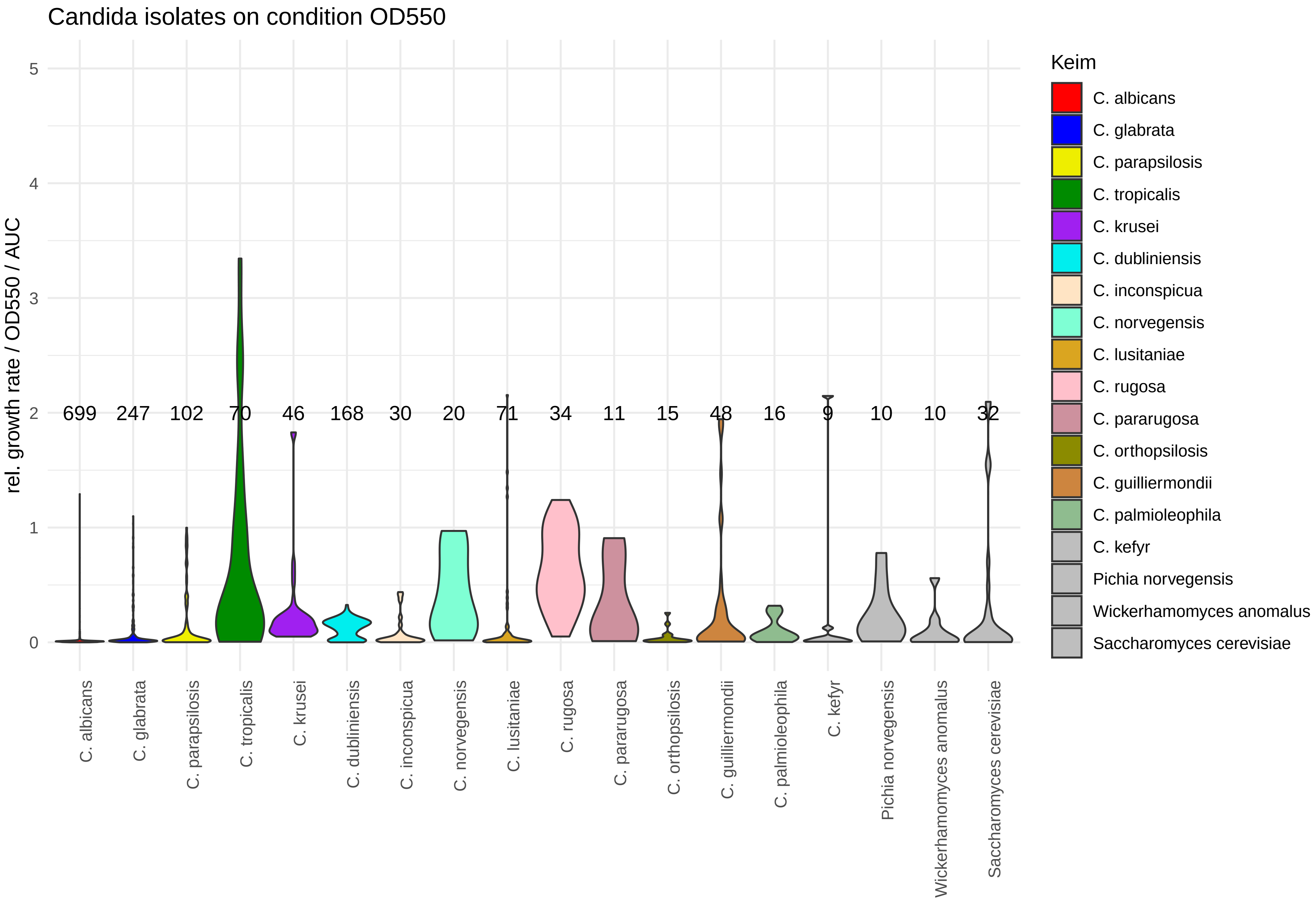

Candida isolates on condition OD550
rel. growth rate / OD550 / AUC
0
1
2
3
4
5
699
247
102
70
46
168
30
20
71
34
11
15
48
16
9
10
10
32
C. albicans
C. glabrata
C. parapsilosis
C. tropicalis
C. krusei
C. dubliniensis
C. inconspicua
C. norvegensis
C. lusitaniae
C. rugosa
C. pararugosa
C. orthopsilosis
C. guilliermondii
C. palmioleophila
C. kefyr
Pichia norvegensis
Wickerhamomyces anomalus
Saccharomyces cerevisiae
Keim
