# Supplementary material for: Distinct properties of human pathogenic Candida species revealed by systematic comparative phenotypic screening of clinical isolates
Source: mSystems. 2025 Dec 8;11(1):e00786-25. doi: 10.1128/msystems.00786-25 (PMC12817934; doi:10.1128/msystems.00786-25)
Supplement: Legends — to supplemental files. [file msystems.00786-25-s0008.docx]

**Supplementary Material Legends**

**Supplementary Figure S1:**

A phylogenetic tree from concatenated and aligned locus sequences. Phenotypic clusters are indicated by color. Branch lengths are drawn to scale. Isolates do not cluster according to phenotype. *C. dubliniensis* isolates showed very close genetic relatedness among isolates while *C. albicans* and *C. tropicalis* showed high diversity between isolates.

**Supplementary Figure S2:**

Determination of cut off for informative number of clusters. A) Dendrogram of the Hierarchical clustering with 10 indicated clusters. B) Betaspider plot of the full dataset with 10 clusters. C) Silhouette scores of 10 clusters. D) Betaspider plot of the *C. glabrata* dataset E) Silhouette scores of four *C. glabrata* clusters. F) Mean of high salt (1M, 1.5M, 1.9M) , Heat (42°C to 50 °C) and Cold (15°C and 20°C) of C. glabrata growth values for the four clusters . Levene's test significance indicated between groups

**Supplementary Tables:**

**Table_S1_S7:** Table S1 to S7 are sheets in one file

**Supplementary Table S1**: Spearman analysis of tested *C. glabrata* isolates and all isolates.

**Supplementary Table S2:** Phenotypic properties of the surveyed Candida isolates can be grouped in three superclusters and clusters (I-X).

**Supplementary Table S3:** Grouping of similar conditions.

**Supplementary Table S4:** Growth parameters used to phenotype 223 *C. glabrata* isolates.

**Supplemental Table S5:** Total set of isolates included in the final phenotypic analysis.

glabrata isolates under different conditions.

**Supplemental Table S6**: Result of Spearman Ranked-Order Test using filtered data according to species.

**Supplementary** Table **S7:** Result of Multi-Locus-Sequence Typing (MLST) from 186 Candida strains.

**Supplementary** Table **S8:** Pairwise PERMANOVA of the *C. glabrata*, and All Strains dataset: r2 values

**Supplementary. Data SD1:** Animated gif of the 3D phenotypic landscape using 1366 clinical Candida isolates. Each axis depicts a principal component. Isolates are grouped by cluster designation indicated by roman numerals.

**Supplementary Data SD2:** Animated gif of the phenotypic landscape using 1366 clinical Candida isolates. Each axis depicts a principal component. Isolates are grouped by cluster designation.

**Supplementary Data SD3:**  MEGAX datasets for constructing the phylogenetic trees. Genetic data was obtained using commonly used MLST probes for the different Candida species.

**Supplementary Data SD4:**  Distribution of all Candida isolates under all conditions tested.

**Supplementary Data SD5:**  Comparison of all conditions for all species
