# Supplementary material for: Distinct properties of human pathogenic Candida species revealed by systematic comparative phenotypic screening of clinical isolates
Source: mSystems. 2025 Dec 8;11(1):e00786-25. doi: 10.1128/msystems.00786-25 (PMC12817934; doi:10.1128/msystems.00786-25)
Supplement: Data SD5 — All correlations. [file msystems.00786-25-s0005.pdf]

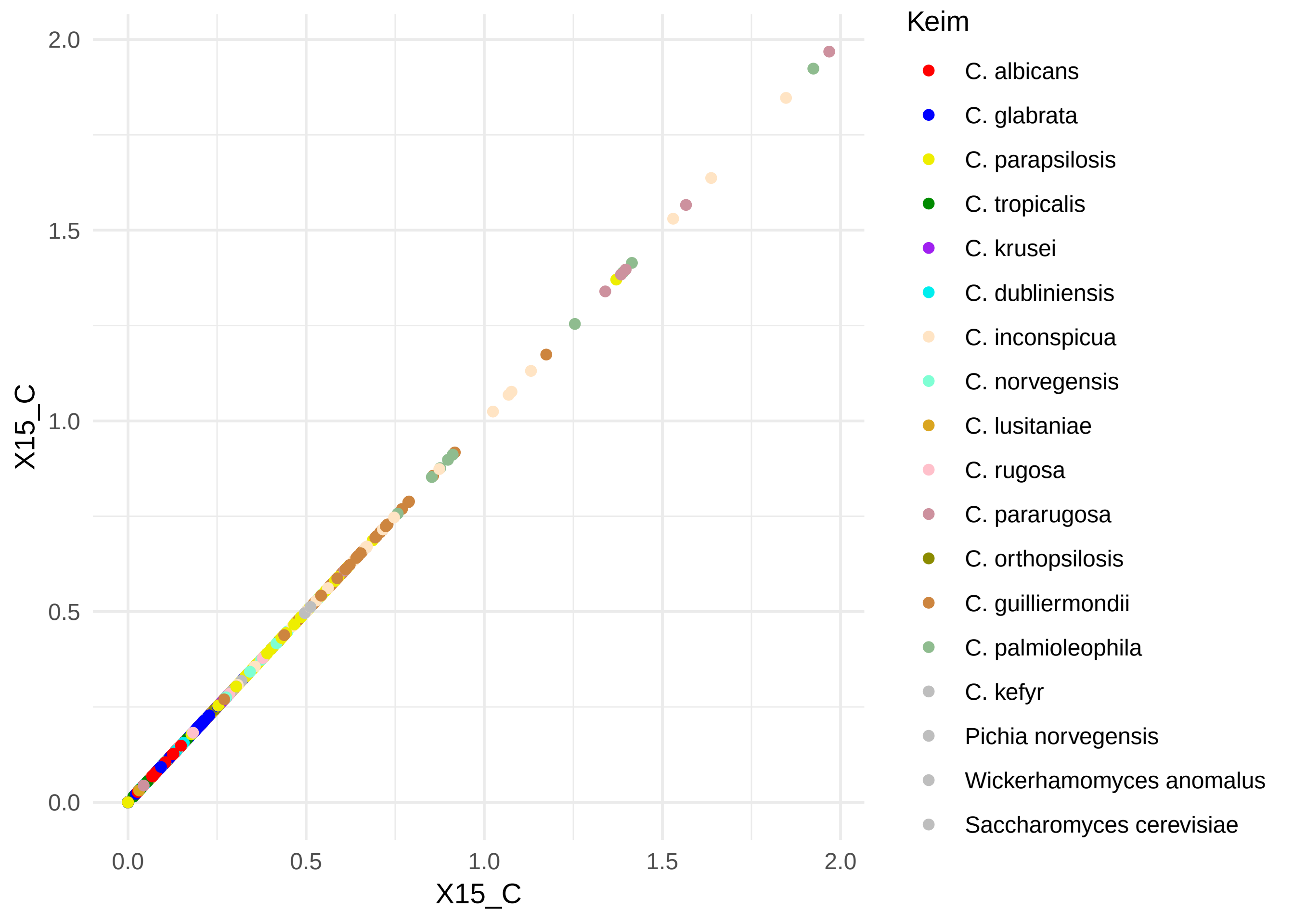
Keim
C. albicans
C. glabrata
C. parapsilosis
C. tropicalis
C. krusei
C. dubliniensis
C. inconspicua
C. norvegensis
C. lusitaniae
C. rugosa
C. pararugosa
C. orthopsilosis
C. guilliermondii
C. palmioleophila
C. kefyr
Pichia norvegensis
Wickerhamomyces anomalus
Saccharomyces cerevisiae
X15_C
X15_C
0.0
0.5
1.0
1.5
2.0
0.0
0.5
1.0
1.5
2.0

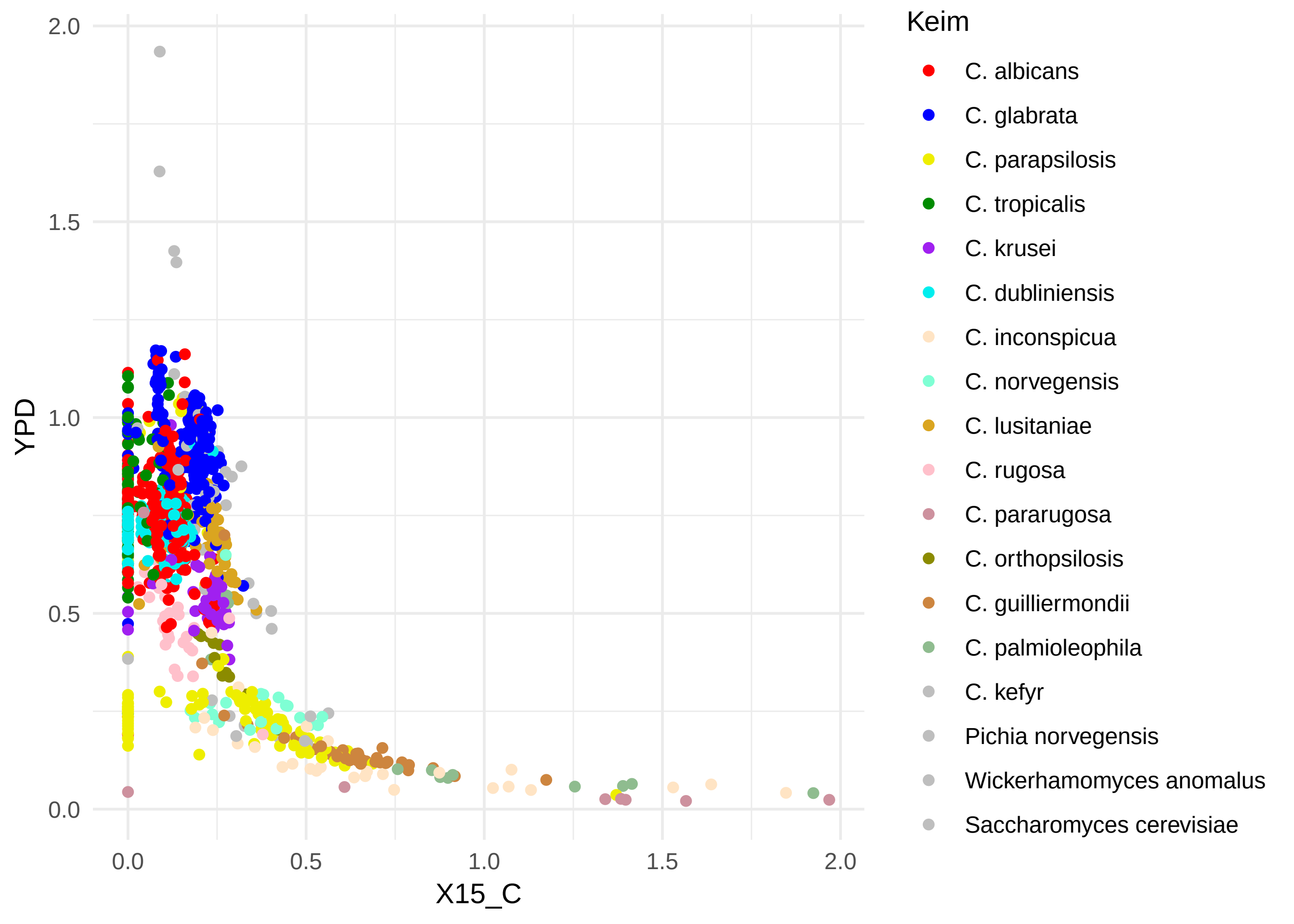

Keim
C. albicans
C. glabrata
C. parapsilosis
C. tropicalis
C. krusei
C. dubliniensis
C. inconspicua
C. norvegensis
C. lusitaniae
C. rugosa
C. pararugosa
C. orthopsilosis
C. guilliermondii
C. palmioleophila
C. kefyr
Pichia norvegensis
Wickerhamomyces anomalus
Saccharomyces cerevisiae
YPD
X15_C
0.0
0.5
1.0
1.5
2.0

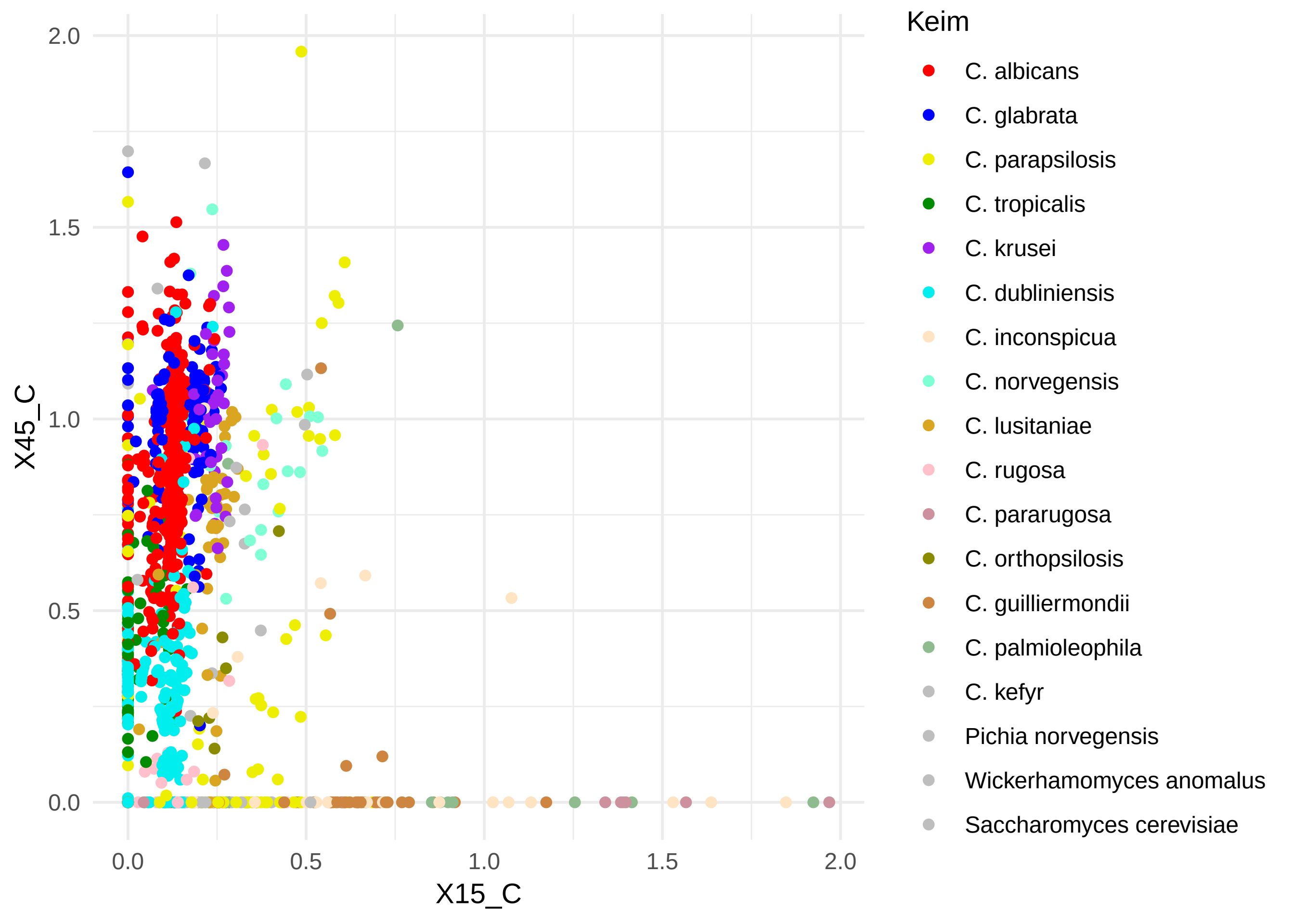
Keim
C. albicans
C. glabrata
C. parapsilosis
C. tropicalis
C. krusei
C. dubliniensis
C. inconspicua
C. norvegensis
C. lusitaniae
C. rugosa
C. pararugosa
C. orthopsilosis
C. guilliermondii
C. palmioleophila
C. kefyr
Pichia norvegensis
Wickerhamomyces anomalus
Saccharomyces cerevisiae
X45_C
X15_C
0.0
0.5
1.0
1.5
2.0

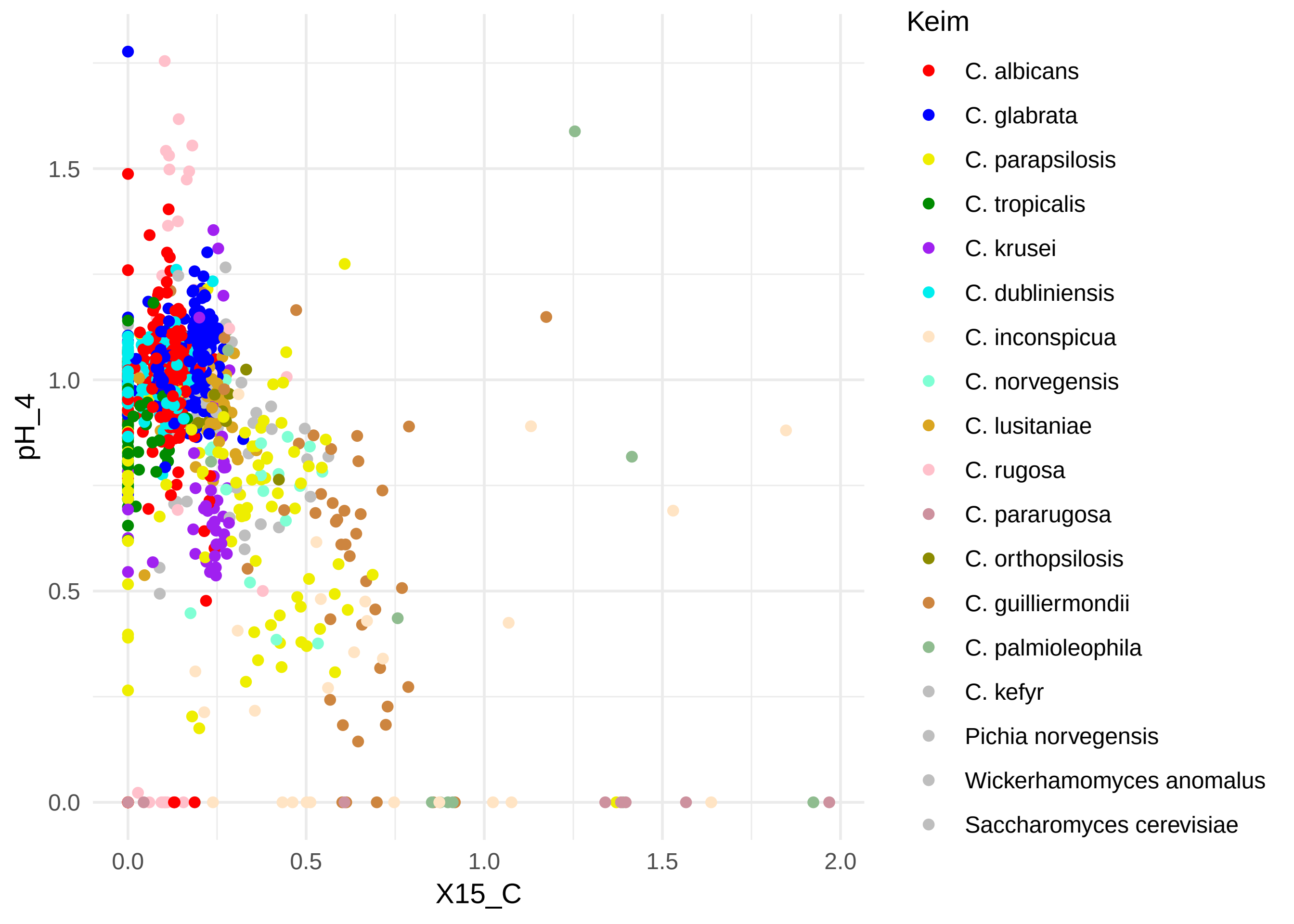
pH_4
X15_C
0.0
0.5
1.0
1.5
2.0
Keim
C. albicans
C. glabrata
C. parapsilosis
C. tropicalis
C. krusei
C. dubliniensis
C. inconspicua
C. norvegensis
C. lusitaniae
C. rugosa
C. pararugosa
C. orthopsilosis
C. guilliermondii
C. palmioleophila
C. kefyr
Pichia norvegensis
Wickerhamomyces anomalus
Saccharomyces cerevisiae

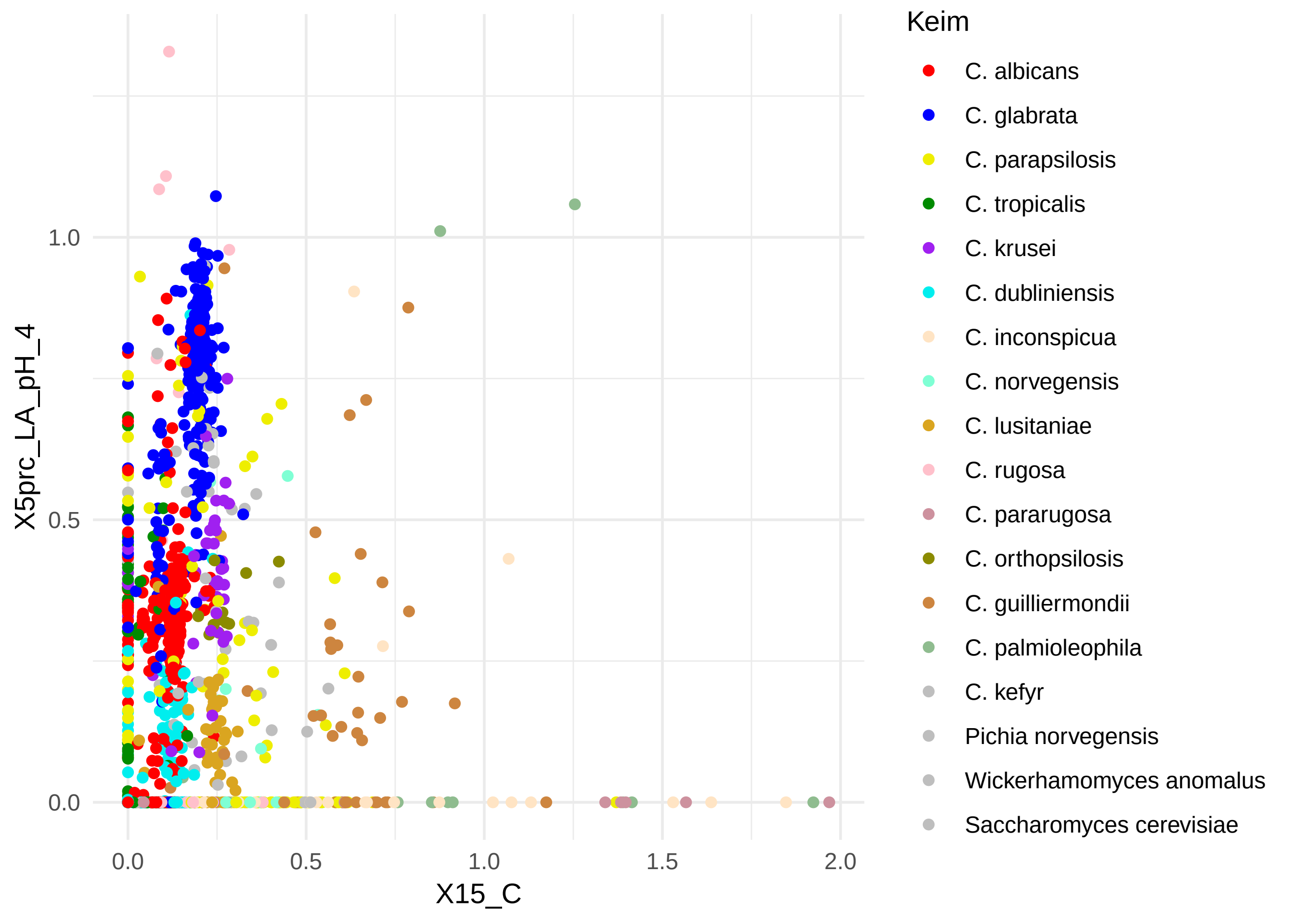
Keim
C. albicans
C. glabrata
C. parapsilosis
C. tropicalis
C. krusei
C. dubliniensis
C. inconspicua
C. norvegensis
C. lusitaniae
C. rugosa
C. pararugosa
C. orthopsilosis
C. guilliermondii
C. palmioleophila
C. kefyr
Pichia norvegensis
Wickerhamomyces anomalus
Saccharomyces cerevisiae
X5prc_LA_pH_4
1.0
0.5
0.0
0.0
0.5
1.0
1.5
2.0
X15_C

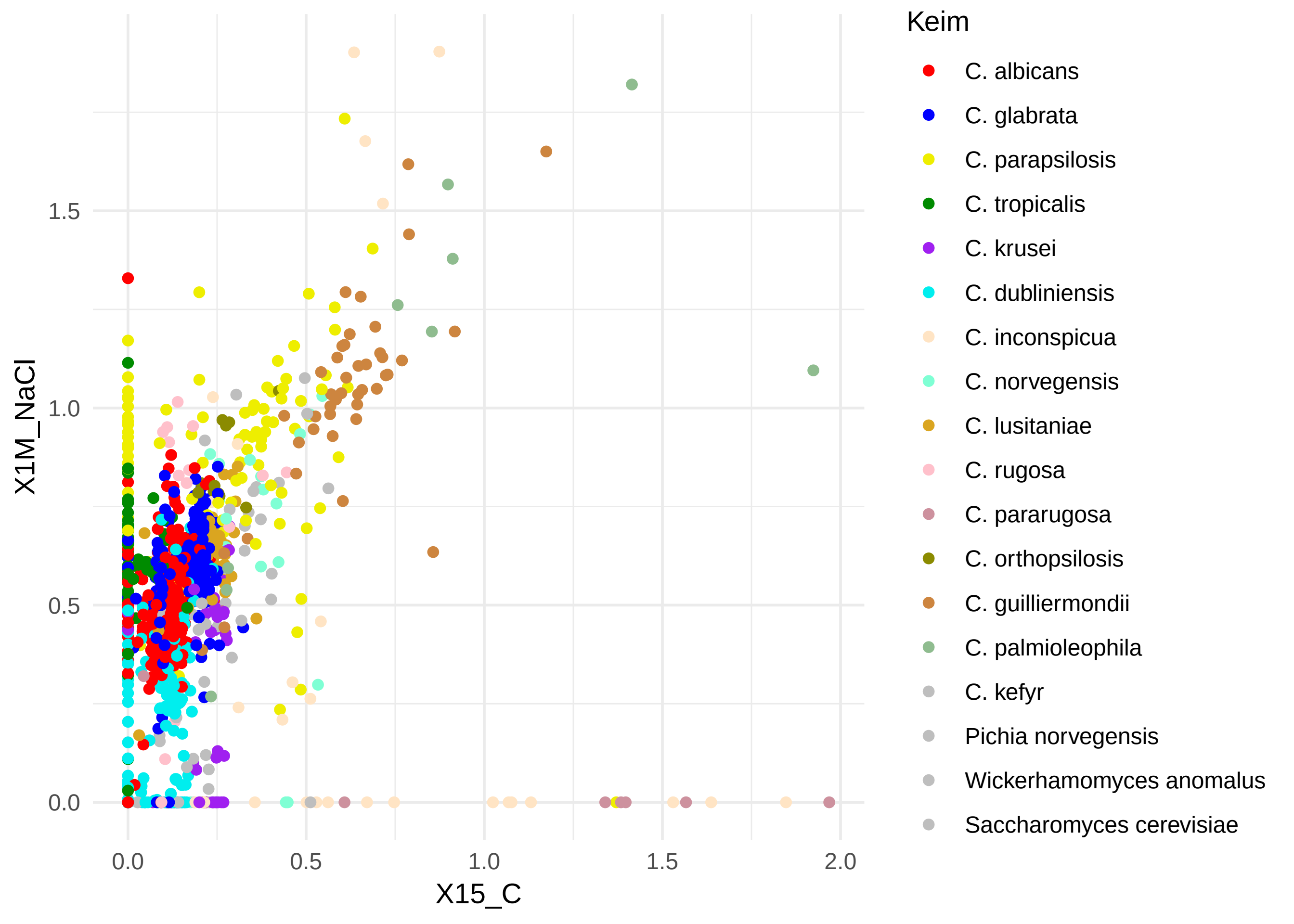

Keim
C. albicans
C. glabrata
C. parapsilosis
C. tropicalis
C. krusei
C. dubliniensis
C. inconspicua
C. norvegensis
C. lusitaniae
C. rugosa
C. pararugosa
C. orthopsilosis
C. guilliermondii
C. palmioleophila
C. kefyr
Pichia norvegensis
Wickerhamomyces anomalus
Saccharomyces cerevisiae
X1M_NaCl
X15_C
0.0
0.5
1.0
1.5
2.0

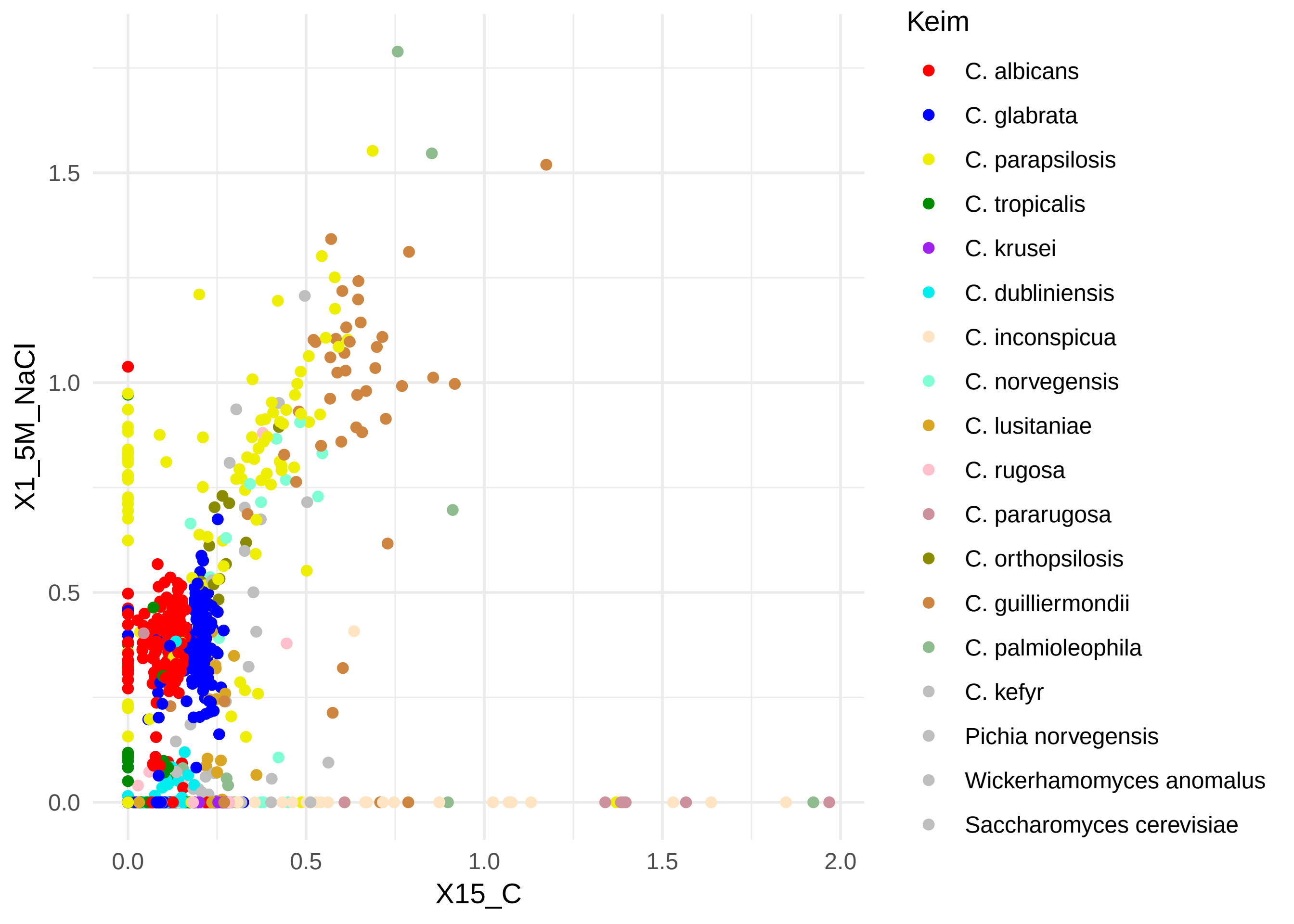
Keim
C. albicans
C. glabrata
C. parapsilosis
C. tropicalis
C. krusei
C. dubliniensis
C. inconspicua
C. norvegensis
C. lusitaniae
C. rugosa
C. pararugosa
C. orthopsilosis
C. guilliermondii
C. palmioleophila
C. kefyr
Pichia norvegensis
Wickerhamomyces anomalus
Saccharomyces cerevisiae
X1_5M_NaCl
X15_C
0.0
0.5
1.0
1.5
2.0

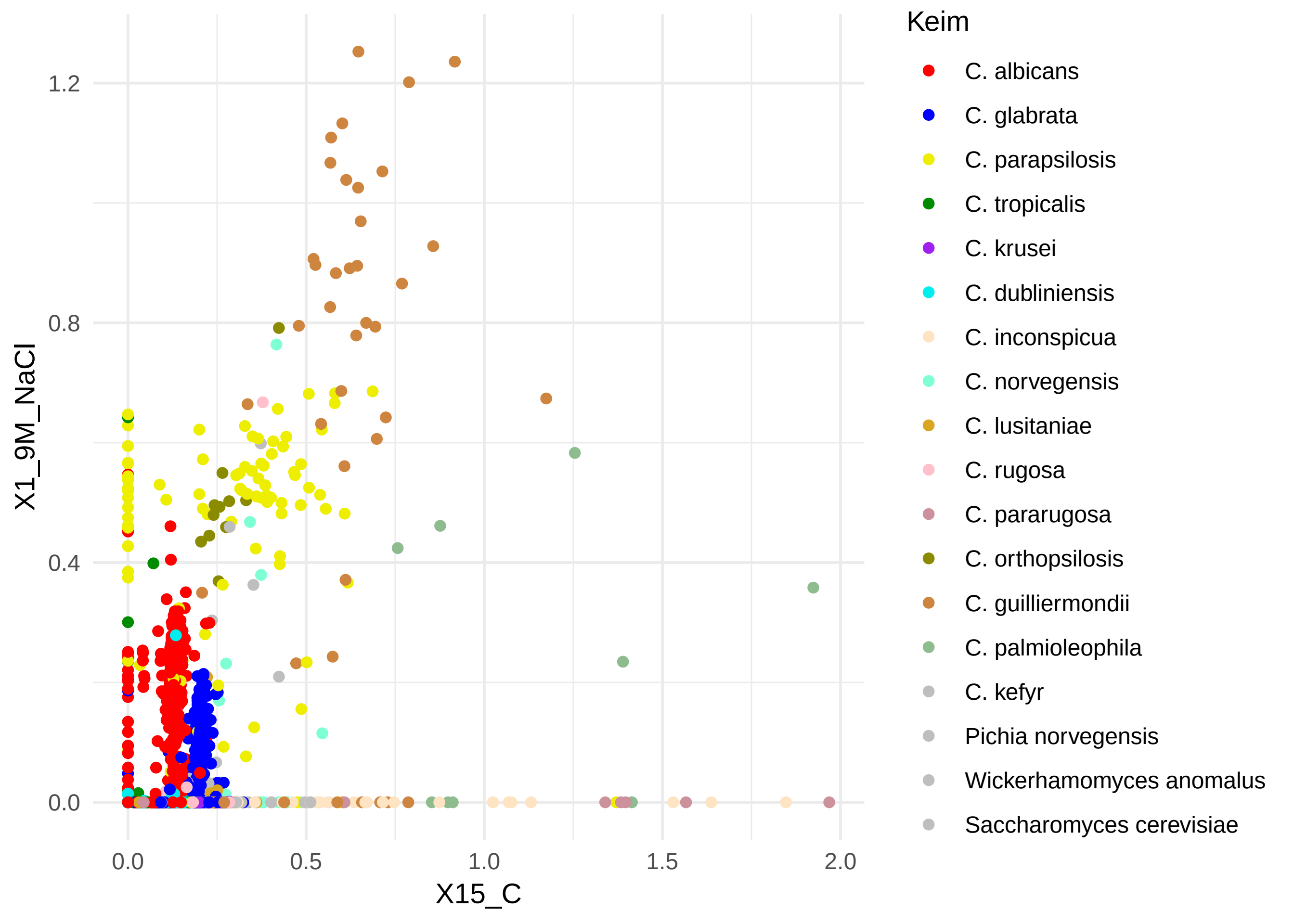
Keim
C. albicans
C. glabrata
C. parapsilosis
C. tropicalis
C. krusei
C. dubliniensis
C. inconspicua
C. norvegensis
C. lusitaniae
C. rugosa
C. pararugosa
C. orthopsilosis
C. guilliermondii
C. palmioleophila
C. kefyr
Pichia norvegensis
Wickerhamomyces anomalus
Saccharomyces cerevisiae
X1_9M_NaCl
1.2
0.8
0.4
0.0
0.0
0.5
1.0
1.5
2.0
X15_C

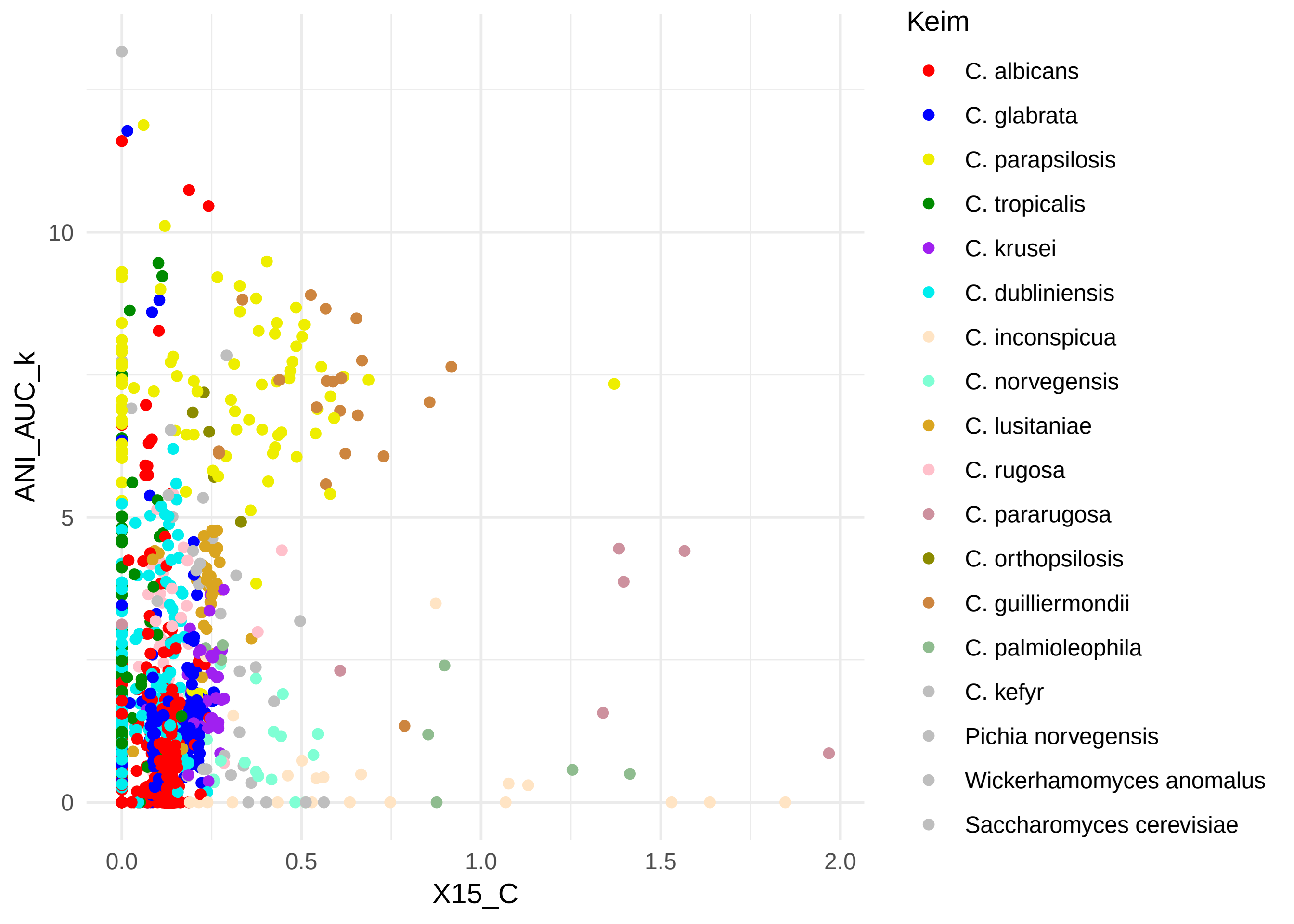
Keim
C. albicans
C. glabrata
C. parapsilosis
C. tropicalis
C. krusei
C. dubliniensis
C. inconspicua
C. norvegensis
C. lusitaniae
C. rugosa
C. pararugosa
C. orthopsilosis
C. guilliermondii
C. palmioleophila
C. kefyr
Pichia norvegensis
Wickerhamomyces anomalus
Saccharomyces cerevisiae
ANI_AUC_k
10
5
0
0.0
0.5
1.0
1.5
2.0
X15_C

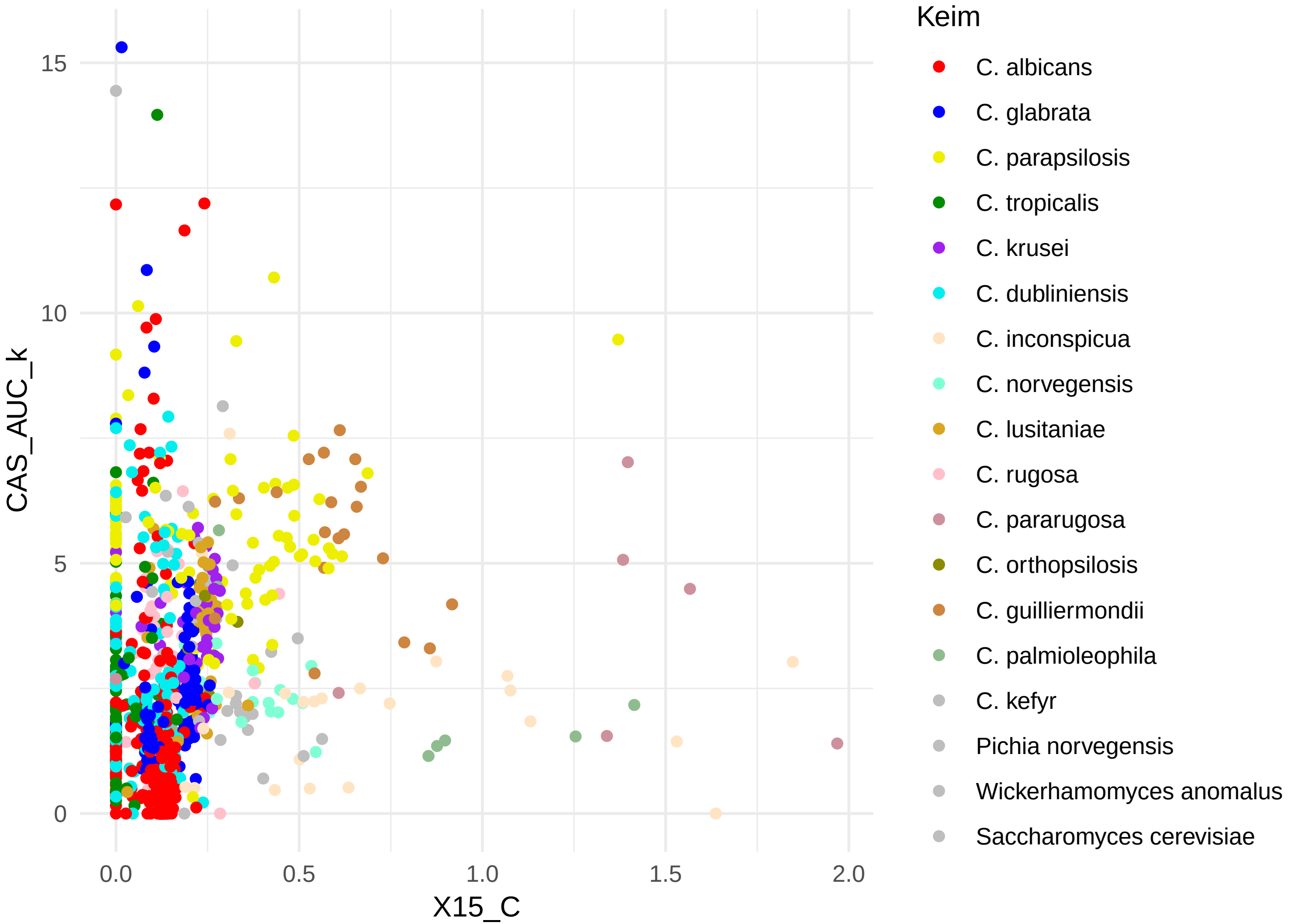
Keim
C. albicans
C. glabrata
C. parapsilosis
C. tropicalis
C. krusei
C. dubliniensis
C. inconspicua
C. norvegensis
C. lusitaniae
C. rugosa
C. pararugosa
C. orthopsilosis
C. guilliermondii
C. palmioleophila
C. kefyr
Pichia norvegensis
Wickerhamomyces anomalus
Saccharomyces cerevisiae
CAS_AUC_k
X15_C
0
5
10
15
0.0
0.5
1.0
1.5
2.0

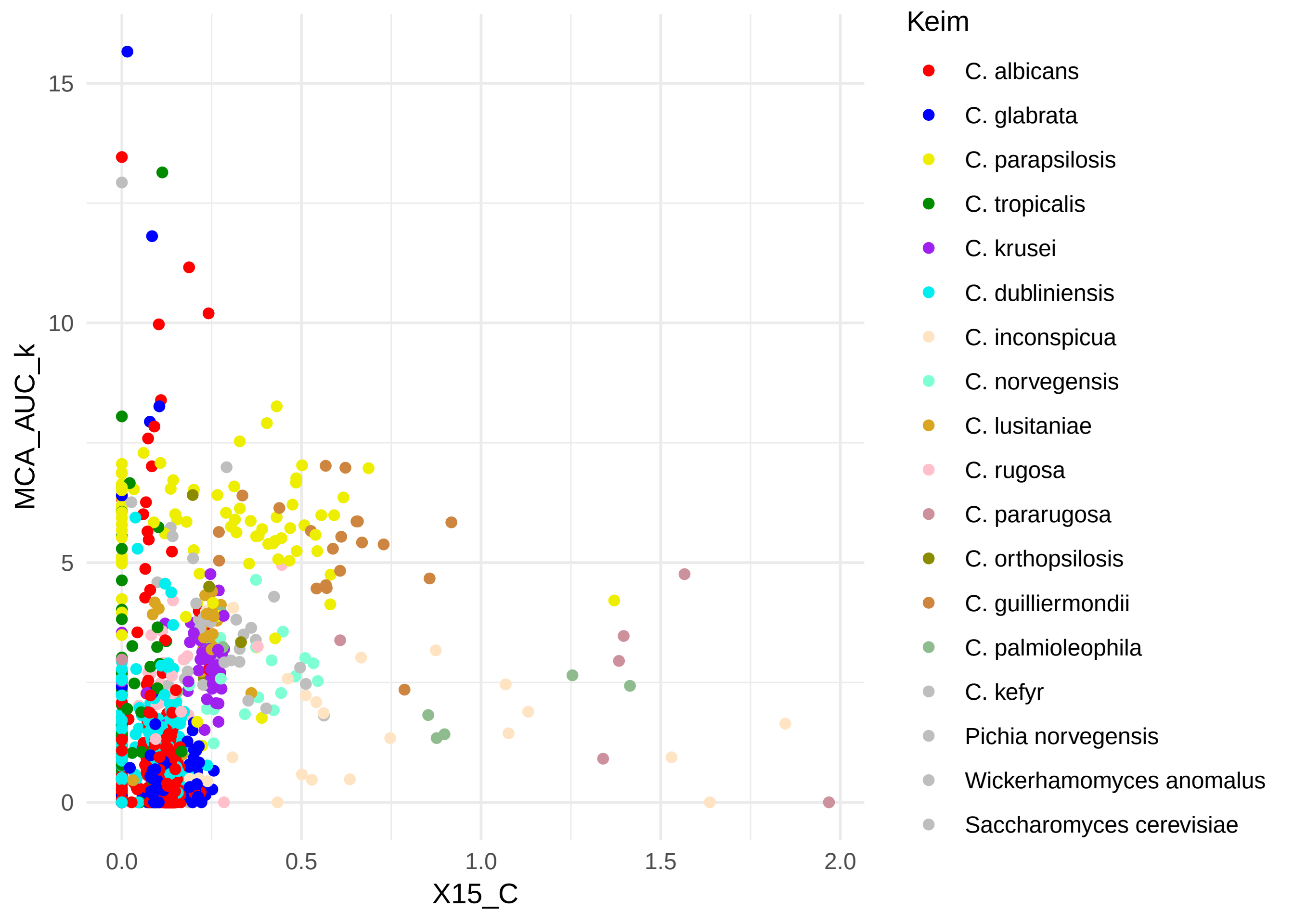

Keim
C. albicans
C. glabrata
C. parapsilosis
C. tropicalis
C. krusei
C. dubliniensis
C. inconspicua
C. norvegensis
C. lusitaniae
C. rugosa
C. pararugosa
C. orthopsilosis
C. guilliermondii
C. palmioleophila
C. kefyr
Pichia norvegensis
Wickerhamomyces anomalus
Saccharomyces cerevisiae
MCA_AUC_k
X15_C
0
5
10
15
0.0
0.5
1.0
1.5
2.0

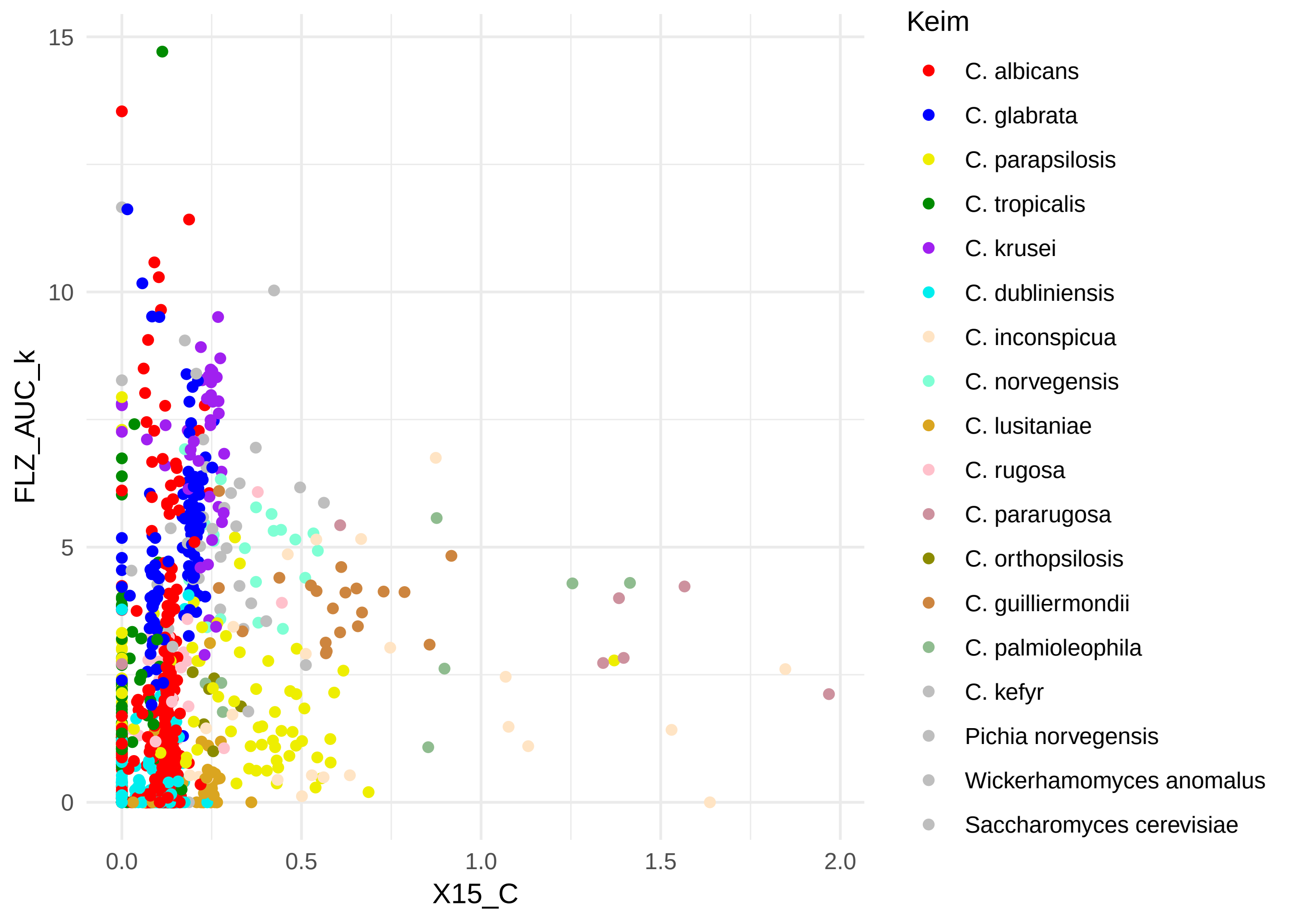

FLZ_AUC_k
15
10
5
0
0.0
0.5
1.0
1.5
2.0
X15_C
Keim
C. albicans
C. glabrata
C. parapsilosis
C. tropicalis
C. krusei
C. dubliniensis
C. inconspicua
C. norvegensis
C. lusitaniae
C. rugosa
C. pararugosa
C. orthopsilosis
C. guilliermondii
C. palmioleophila
C. kefyr
Pichia norvegensis
Wickerhamomyces anomalus
Saccharomyces cerevisiae

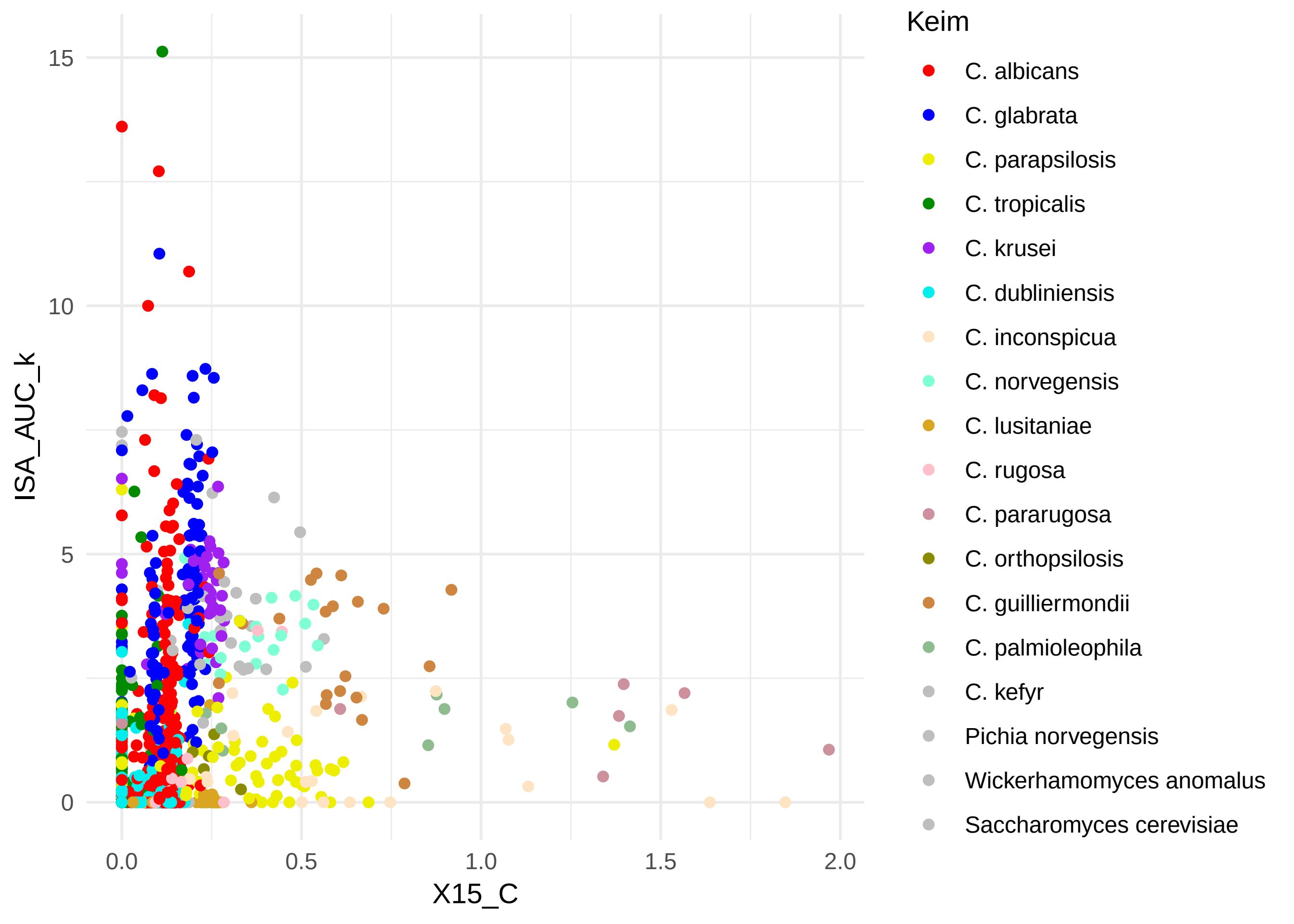

Keim
C. albicans
C. glabrata
C. parapsilosis
C. tropicalis
C. krusei
C. dubliniensis
C. inconspicua
C. norvegensis
C. lusitaniae
C. rugosa
C. pararugosa
C. orthopsilosis
C. guilliermondii
C. palmioleophila
C. kefyr
Pichia norvegensis
Wickerhamomyces anomalus
Saccharomyces cerevisiae
ISA_AUC_k
X15_C
15
10
5
0
0.0
0.5
1.0
1.5
2.0

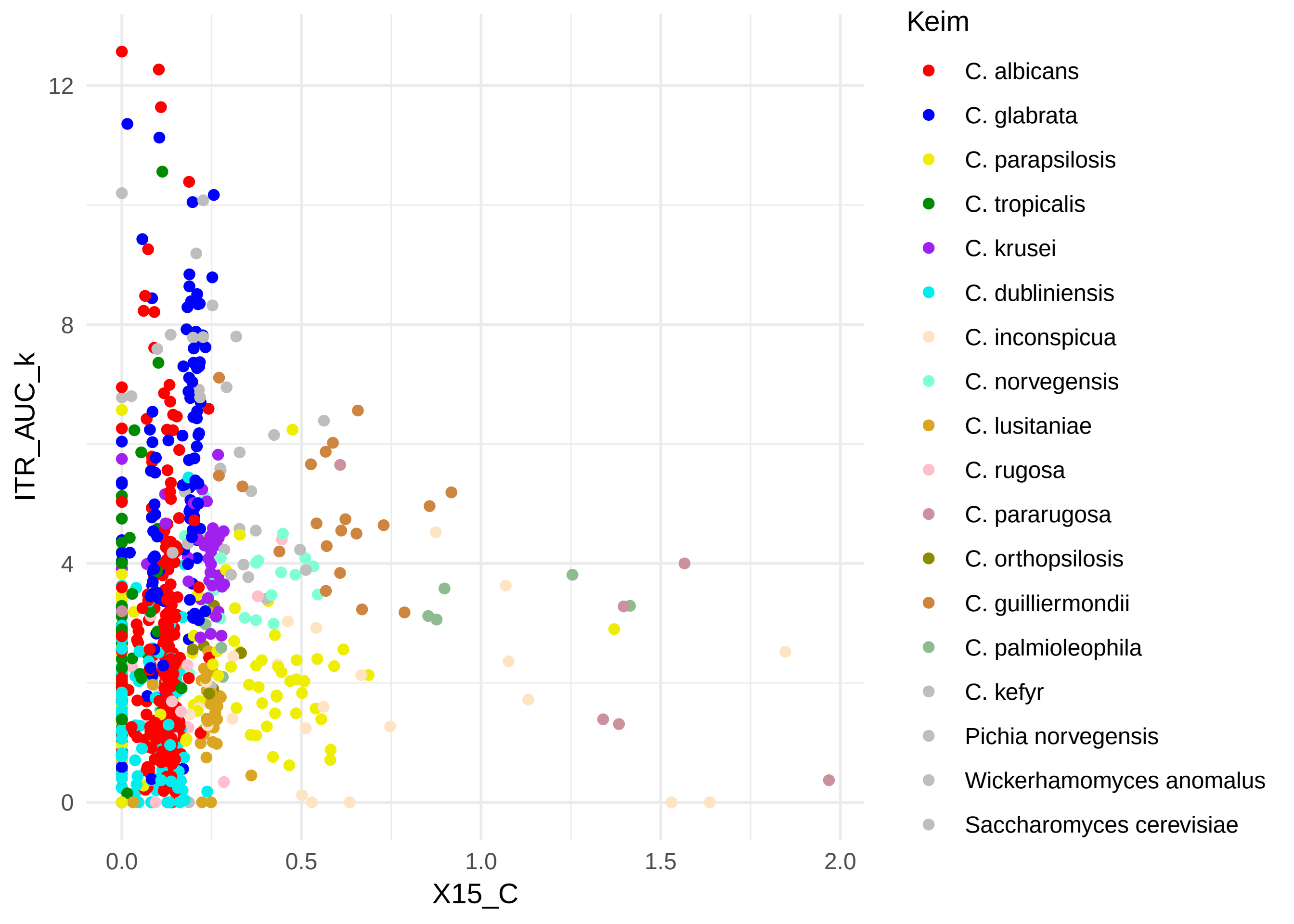
Keim
C. albicans
C. glabrata
C. parapsilosis
C. tropicalis
C. krusei
C. dubliniensis
C. inconspicua
C. norvegensis
C. lusitaniae
C. rugosa
C. pararugosa
C. orthopsilosis
C. guilliermondii
C. palmioleophila
C. kefyr
Pichia norvegensis
Wickerhamomyces anomalus
Saccharomyces cerevisiae
ITR_AUC_k
X15_C
0
4
8
12
0.0
0.5
1.0
1.5
2.0

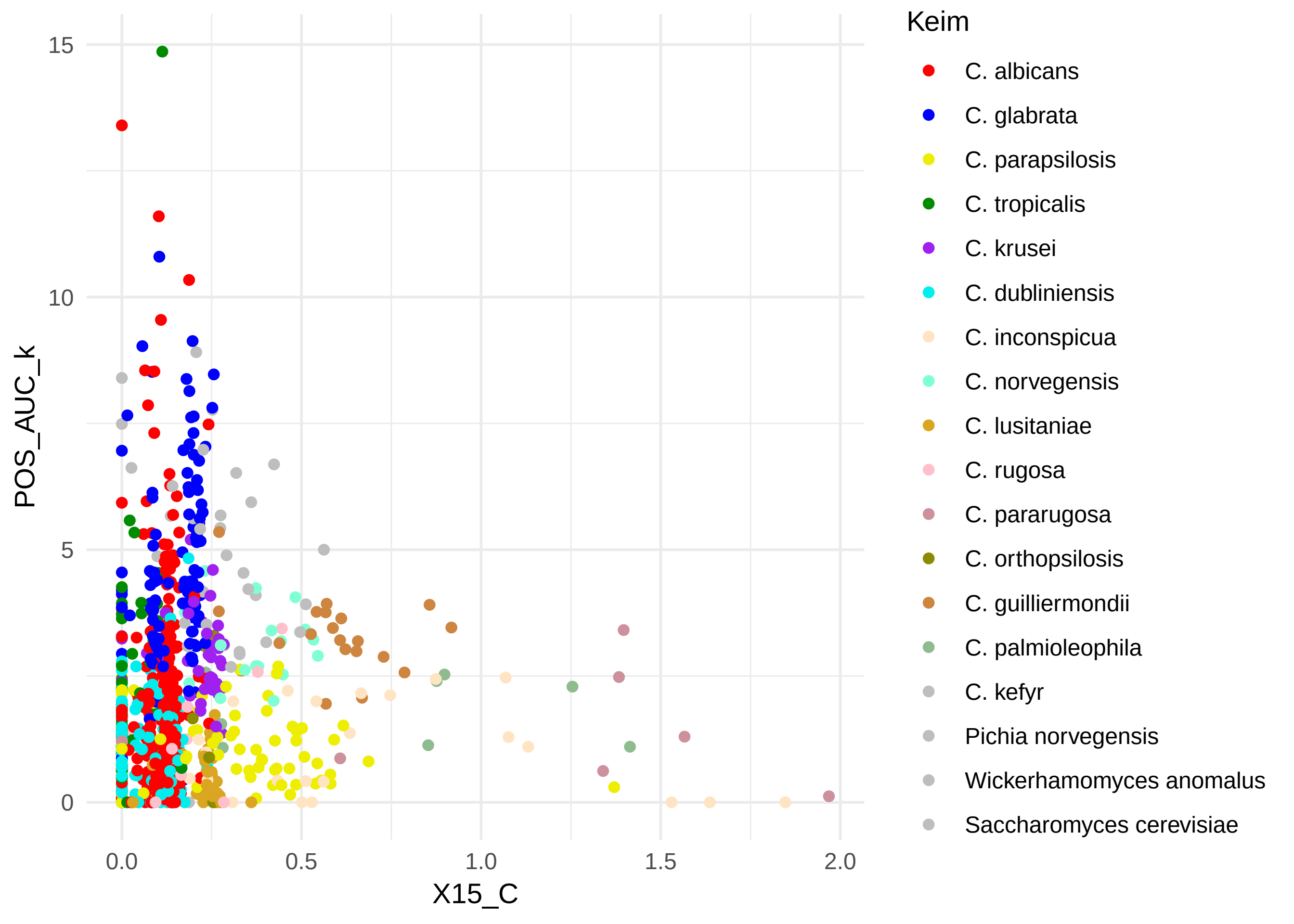

Keim
C. albicans
C. glabrata
C. parapsilosis
C. tropicalis
C. krusei
C. dubliniensis
C. inconspicua
C. norvegensis
C. lusitaniae
C. rugosa
C. pararugosa
C. orthopsilosis
C. guilliermondii
C. palmioleophila
C. kefyr
Pichia norvegensis
Wickerhamomyces anomalus
Saccharomyces cerevisiae
POS_AUC_k
X15_C
15
10
5
0
0.0
0.5
1.0
1.5
2.0

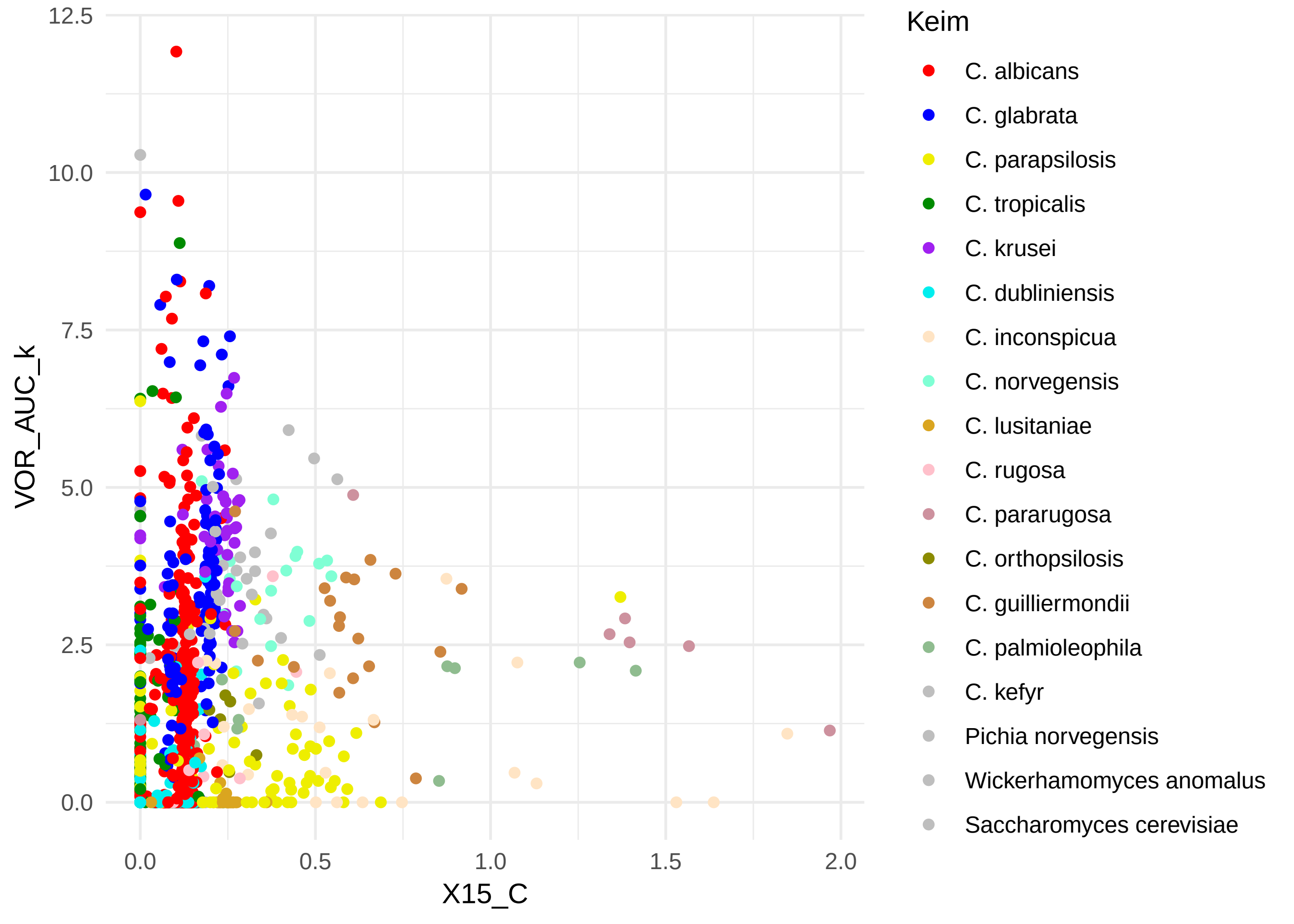

Keim
C. albicans
C. glabrata
C. parapsilosis
C. tropicalis
C. krusei
C. dubliniensis
C. inconspicua
C. norvegensis
C. lusitaniae
C. rugosa
C. pararugosa
C. orthopsilosis
C. guilliermondii
C. palmioleophila
C. kefyr
Pichia norvegensis
Wickerhamomyces anomalus
Saccharomyces cerevisiae
VOR_AUC_k
X15_C
0.0
2.5
5.0
7.5
10.0
12.5
0.0
0.5
1.0
1.5
2.0

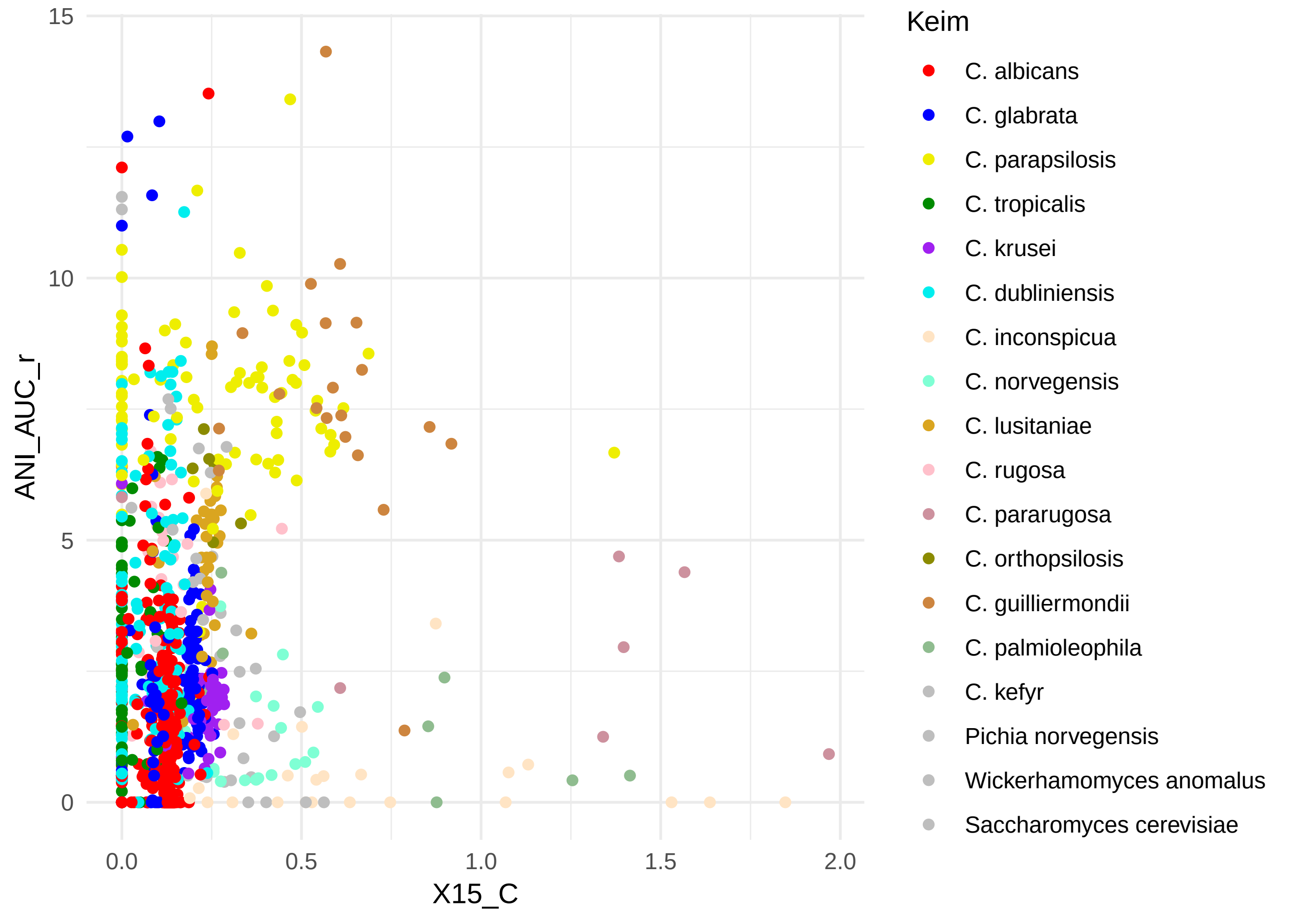
15
10
5
0
ANI_AUC_r
0.0
0.5
1.0
1.5
2.0
X15_C
Keim
C. albicans
C. glabrata
C. parapsilosis
C. tropicalis
C. krusei
C. dubliniensis
C. inconspicua
C. norvegensis
C. lusitaniae
C. rugosa
C. pararugosa
C. orthopsilosis
C. guilliermondii
C. palmioleophila
C. kefyr
Pichia norvegensis
Wickerhamomyces anomalus
Saccharomyces cerevisiae

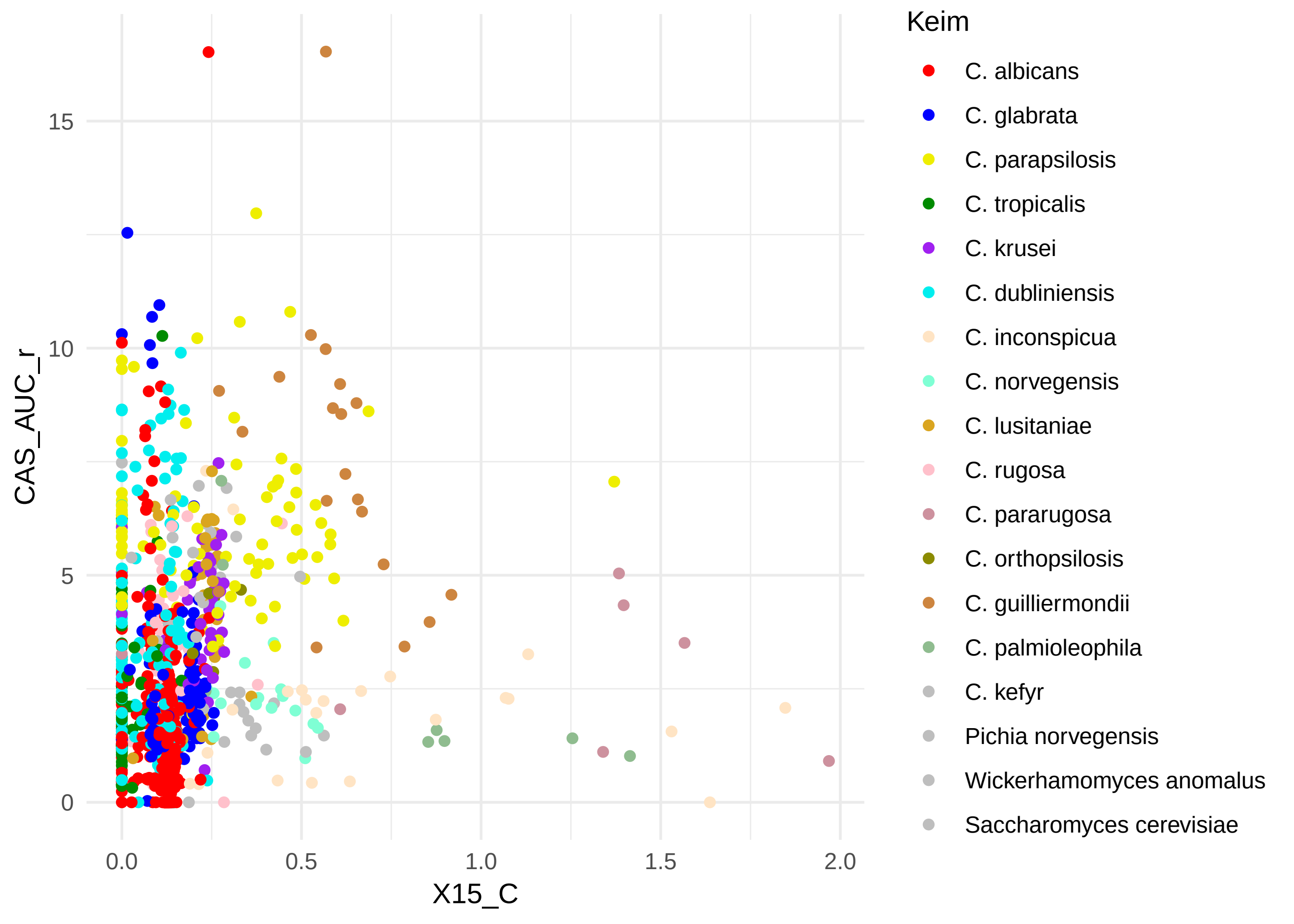

Keim
C. albicans
C. glabrata
C. parapsilosis
C. tropicalis
C. krusei
C. dubliniensis
C. inconspicua
C. norvegensis
C. lusitaniae
C. rugosa
C. pararugosa
C. orthopsilosis
C. guilliermondii
C. palmioleophila
C. kefyr
Pichia norvegensis
Wickerhamomyces anomalus
Saccharomyces cerevisiae
CAS_AUC_r
X15_C
0
5
10
15
0.0
0.5
1.0
1.5
2.0

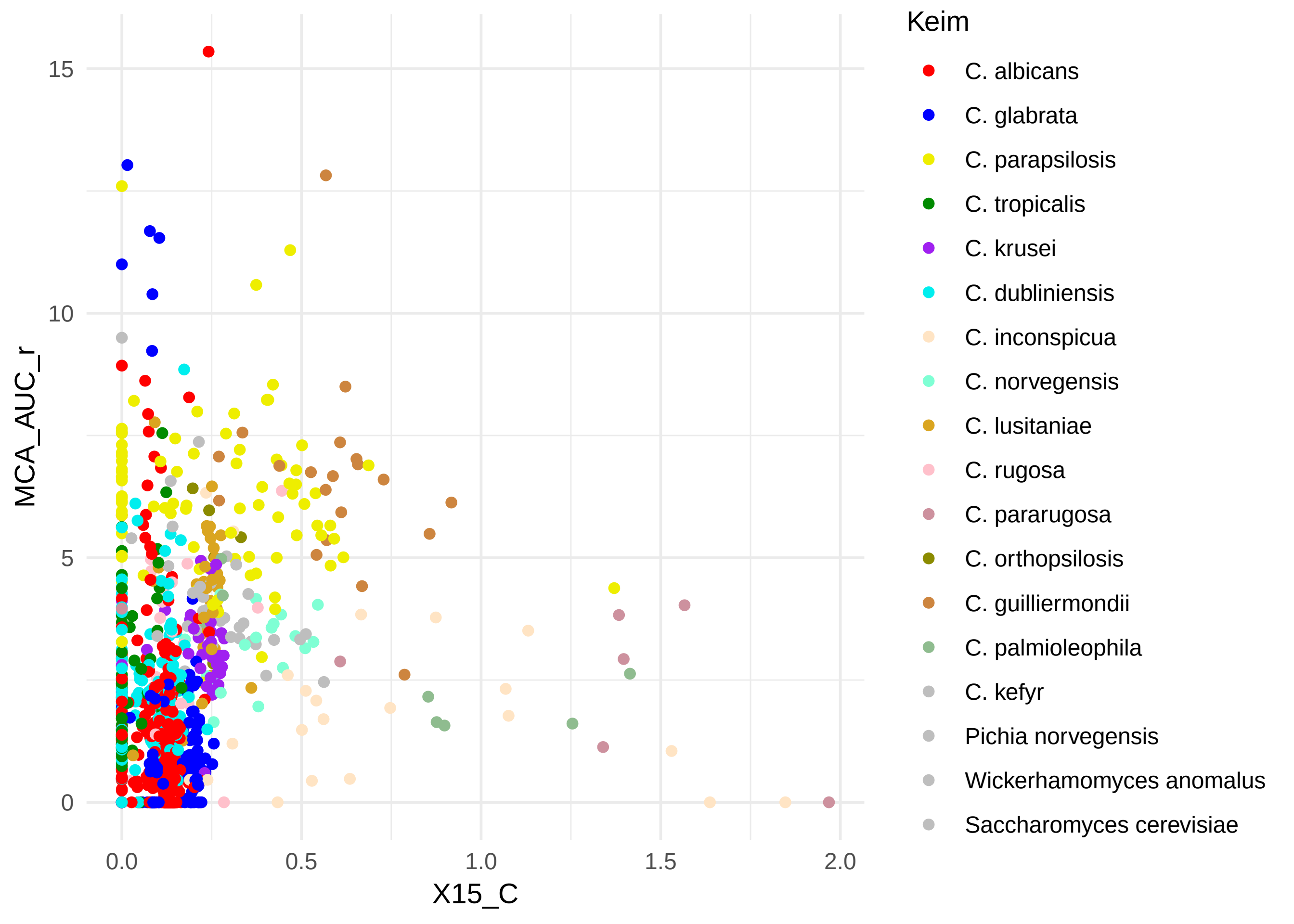

Keim
C. albicans
C. glabrata
C. parapsilosis
C. tropicalis
C. krusei
C. dubliniensis
C. inconspicua
C. norvegensis
C. lusitaniae
C. rugosa
C. pararugosa
C. orthopsilosis
C. guilliermondii
C. palmioleophila
C. kefyr
Pichia norvegensis
Wickerhamomyces anomalus
Saccharomyces cerevisiae
MCA_AUC_r
X15_C
0
5
10
15
0.0
0.5
1.0
1.5
2.0

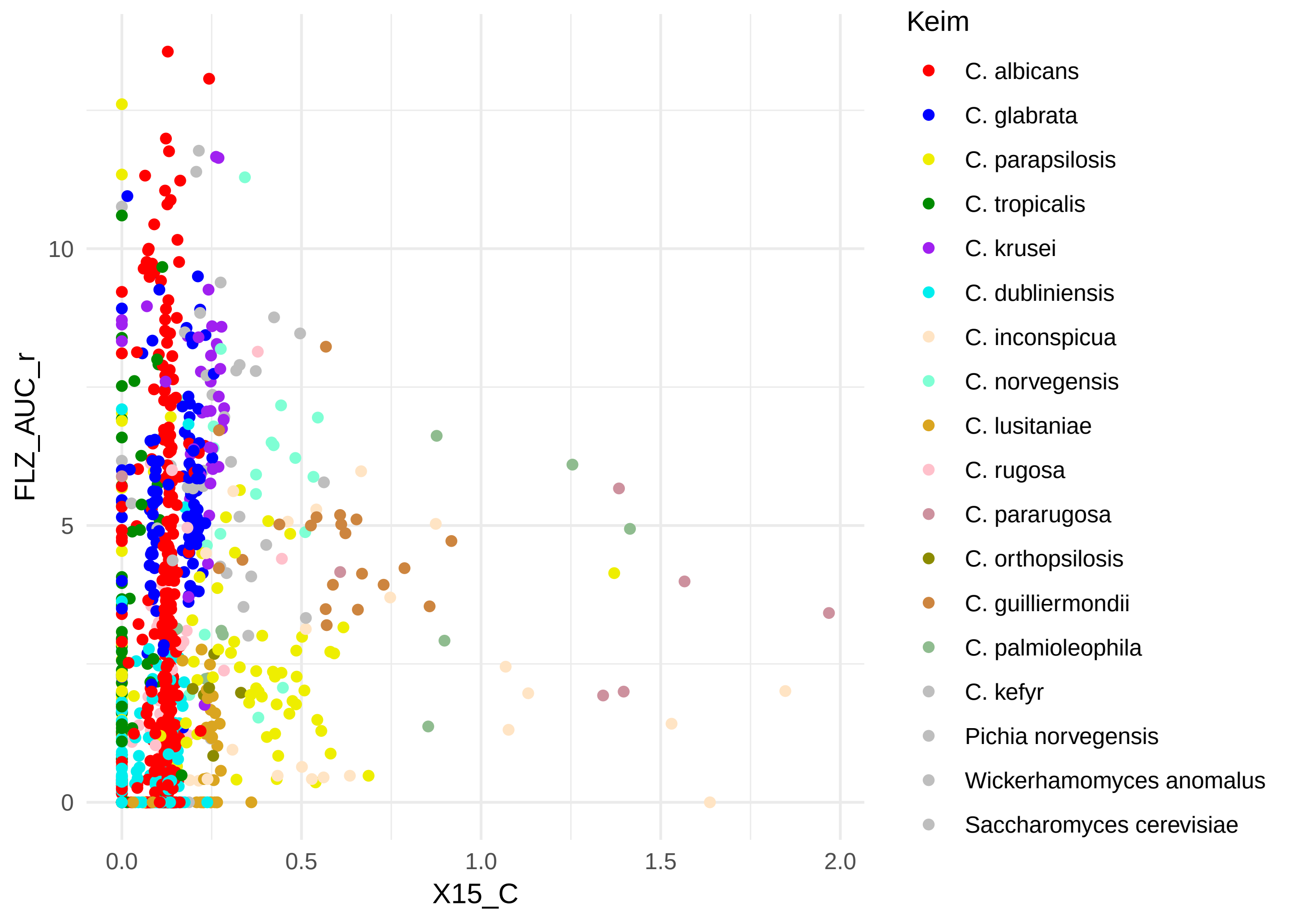
Keim
C. albicans
C. glabrata
C. parapsilosis
C. tropicalis
C. krusei
C. dubliniensis
C. inconspicua
C. norvegensis
C. lusitaniae
C. rugosa
C. pararugosa
C. orthopsilosis
C. guilliermondii
C. palmioleophila
C. kefyr
Pichia norvegensis
Wickerhamomyces anomalus
Saccharomyces cerevisiae
FLZ_AUC_r
10
5
0
0.0
0.5
1.0
1.5
2.0
X15_C

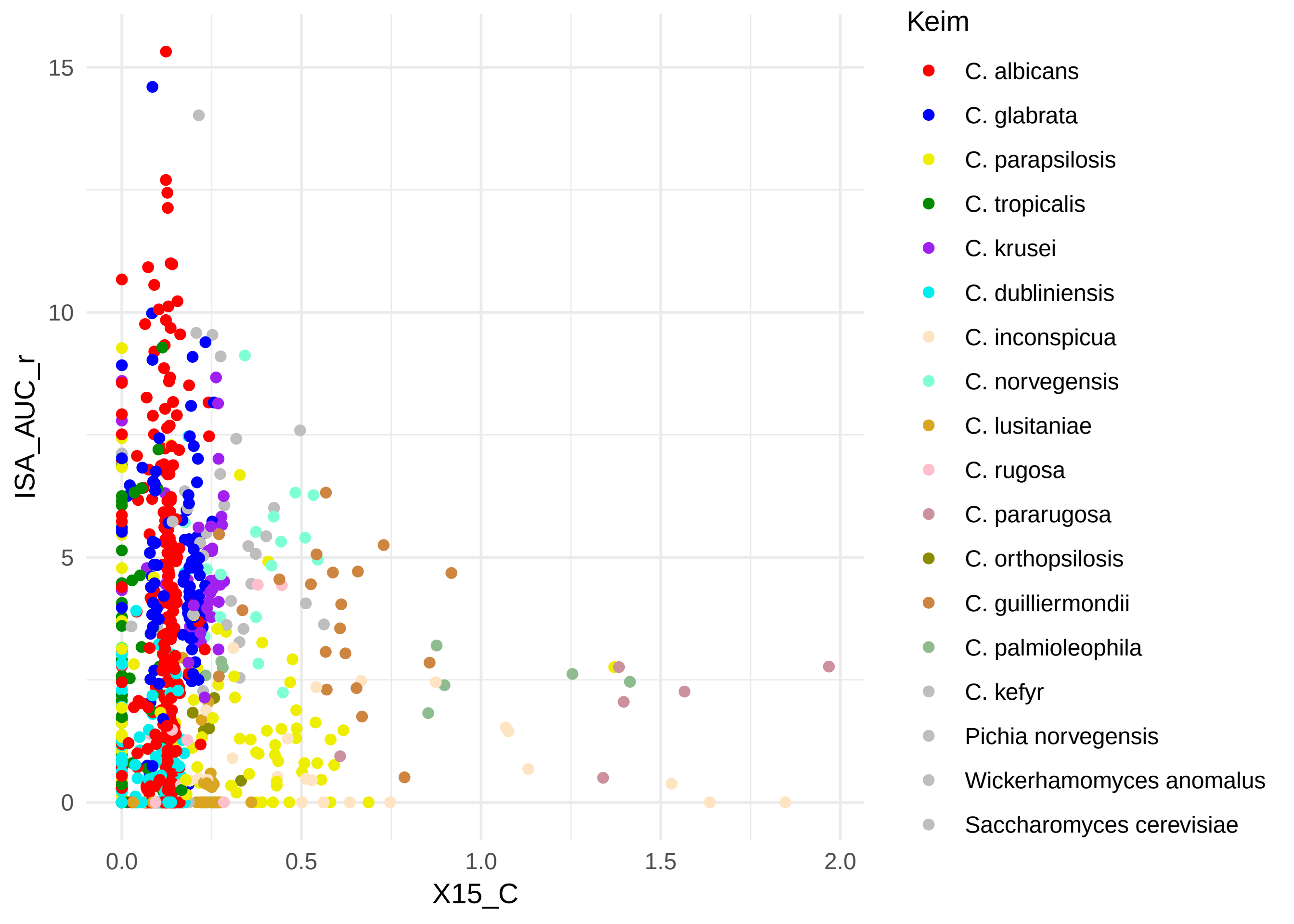
Keim
C. albicans
C. glabrata
C. parapsilosis
C. tropicalis
C. krusei
C. dubliniensis
C. inconspicua
C. norvegensis
C. lusitaniae
C. rugosa
C. pararugosa
C. orthopsilosis
C. guilliermondii
C. palmioleophila
C. kefyr
Pichia norvegensis
Wickerhamomyces anomalus
Saccharomyces cerevisiae
ISA_AUC_r
X15_C
0
5
10
15
0.0
0.5
1.0
1.5
2.0

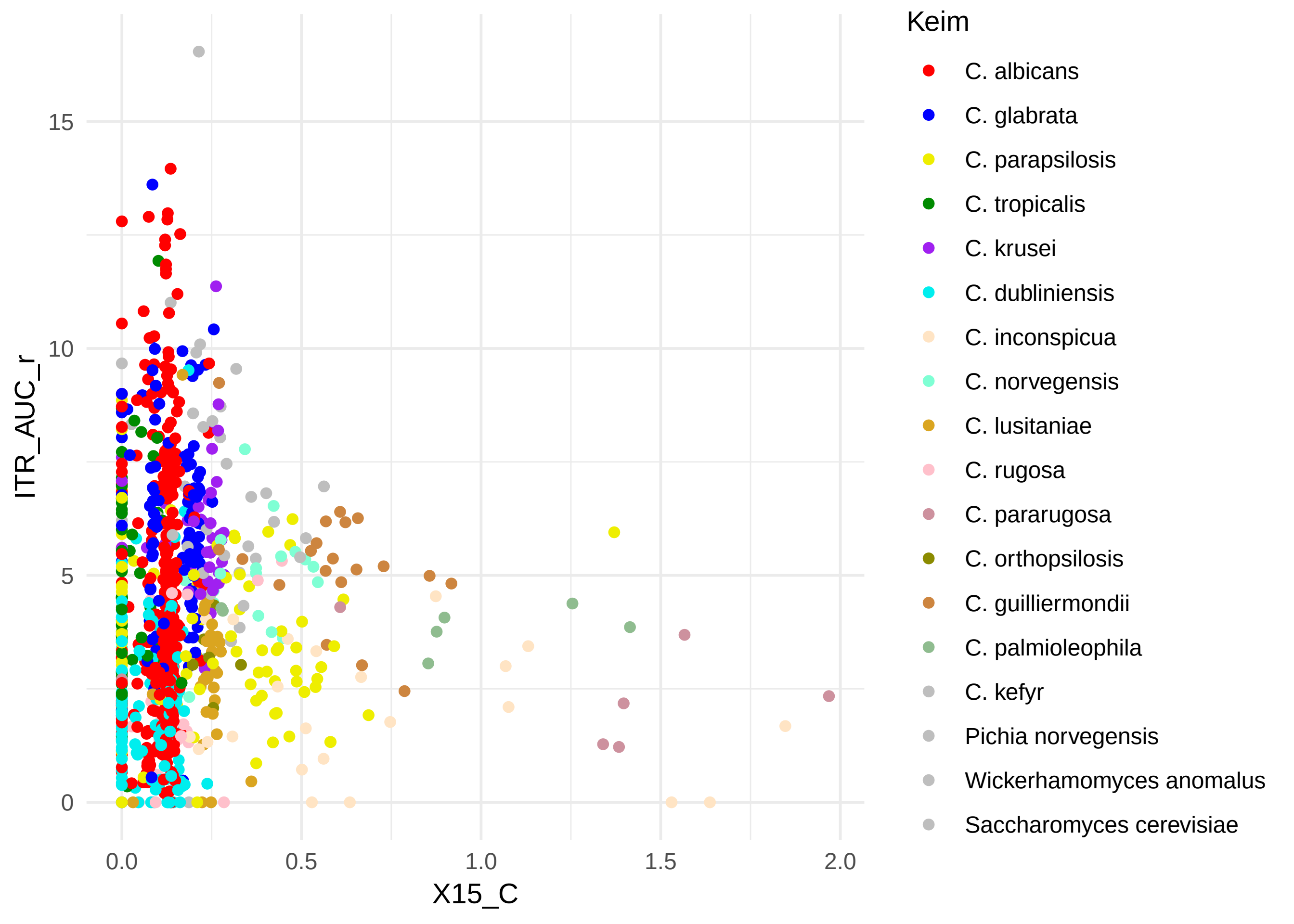
Keim
C. albicans
C. glabrata
C. parapsilosis
C. tropicalis
C. krusei
C. dubliniensis
C. inconspicua
C. norvegensis
C. lusitaniae
C. rugosa
C. pararugosa
C. orthopsilosis
C. guilliermondii
C. palmioleophila
C. kefyr
Pichia norvegensis
Wickerhamomyces anomalus
Saccharomyces cerevisiae
ITR_AUC_r
0
5
10
15
X15_C
0.0
0.5
1.0
1.5
2.0

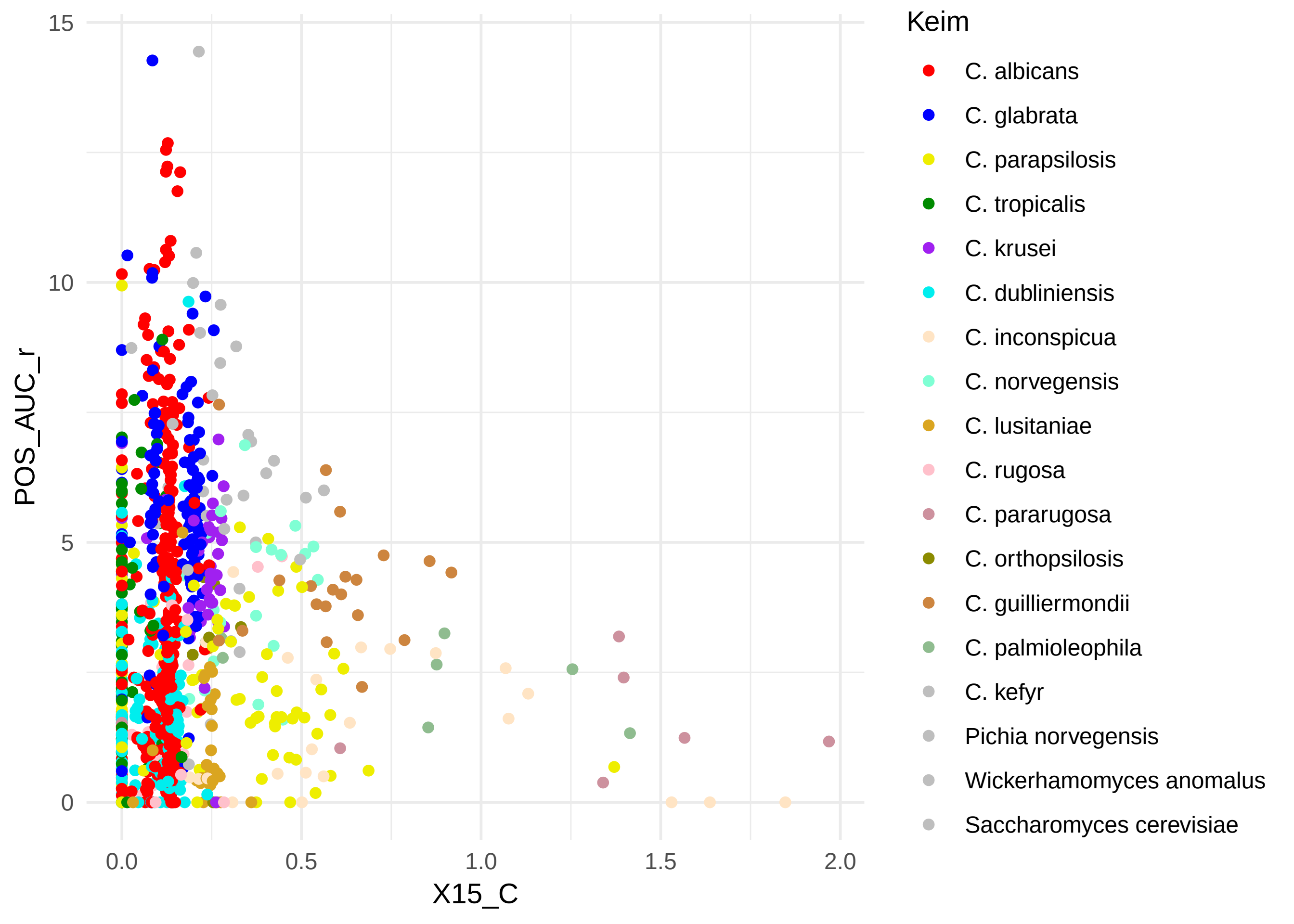

Keim
C. albicans
C. glabrata
C. parapsilosis
C. tropicalis
C. krusei
C. dubliniensis
C. inconspicua
C. norvegensis
C. lusitaniae
C. rugosa
C. pararugosa
C. orthopsilosis
C. guilliermondii
C. palmioleophila
C. kefyr
Pichia norvegensis
Wickerhamomyces anomalus
Saccharomyces cerevisiae
POS_AUC_r
X15_C
0
5
10
15
0.0
0.5
1.0
1.5
2.0

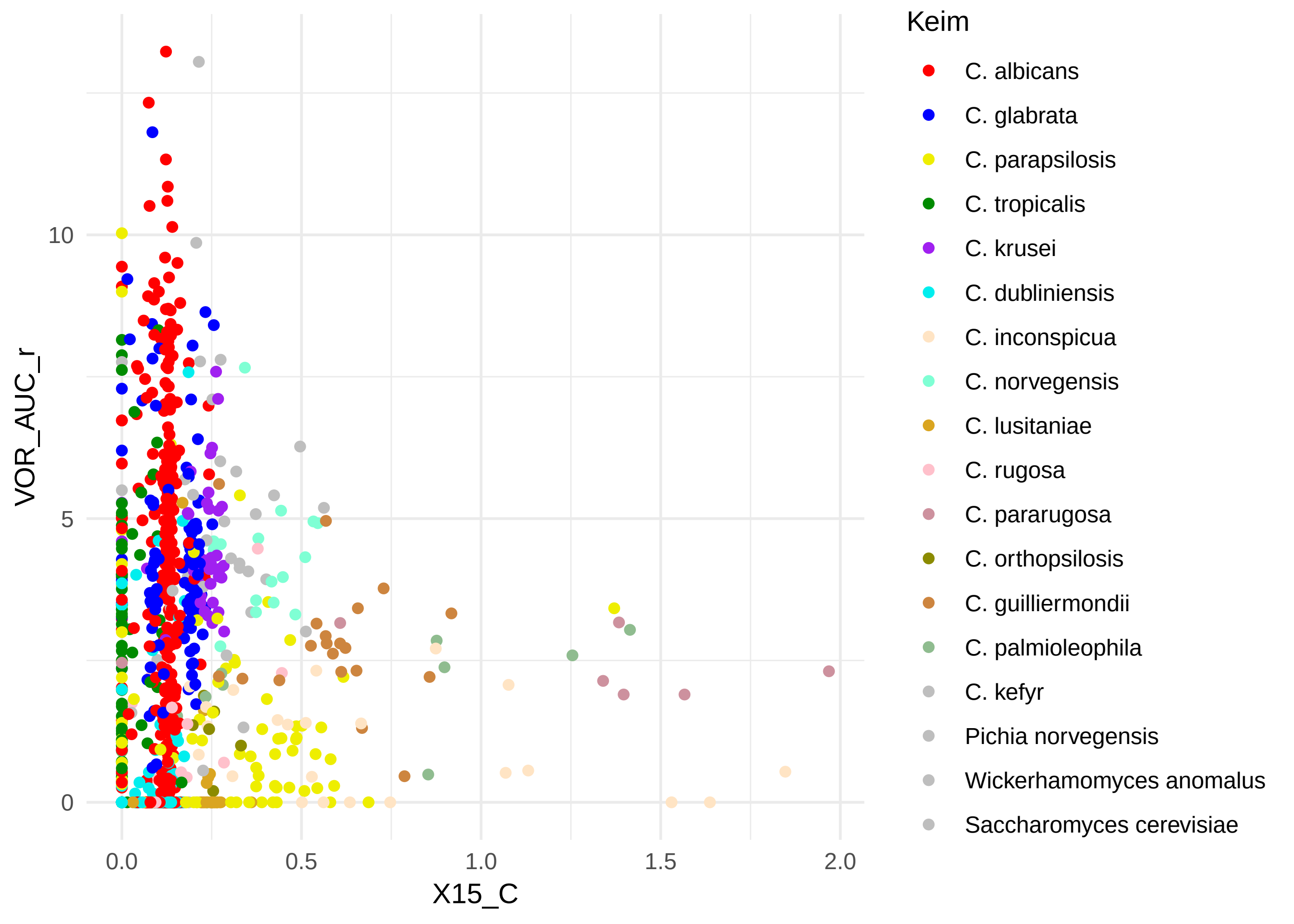
Keim
C. albicans
C. glabrata
C. parapsilosis
C. tropicalis
C. krusei
C. dubliniensis
C. inconspicua
C. norvegensis
C. lusitaniae
C. rugosa
C. pararugosa
C. orthopsilosis
C. guilliermondii
C. palmioleophila
C. kefyr
Pichia norvegensis
Wickerhamomyces anomalus
Saccharomyces cerevisiae
VOR_AUC_r
0
5
10
X15_C
0.0
0.5
1.0
1.5
2.0

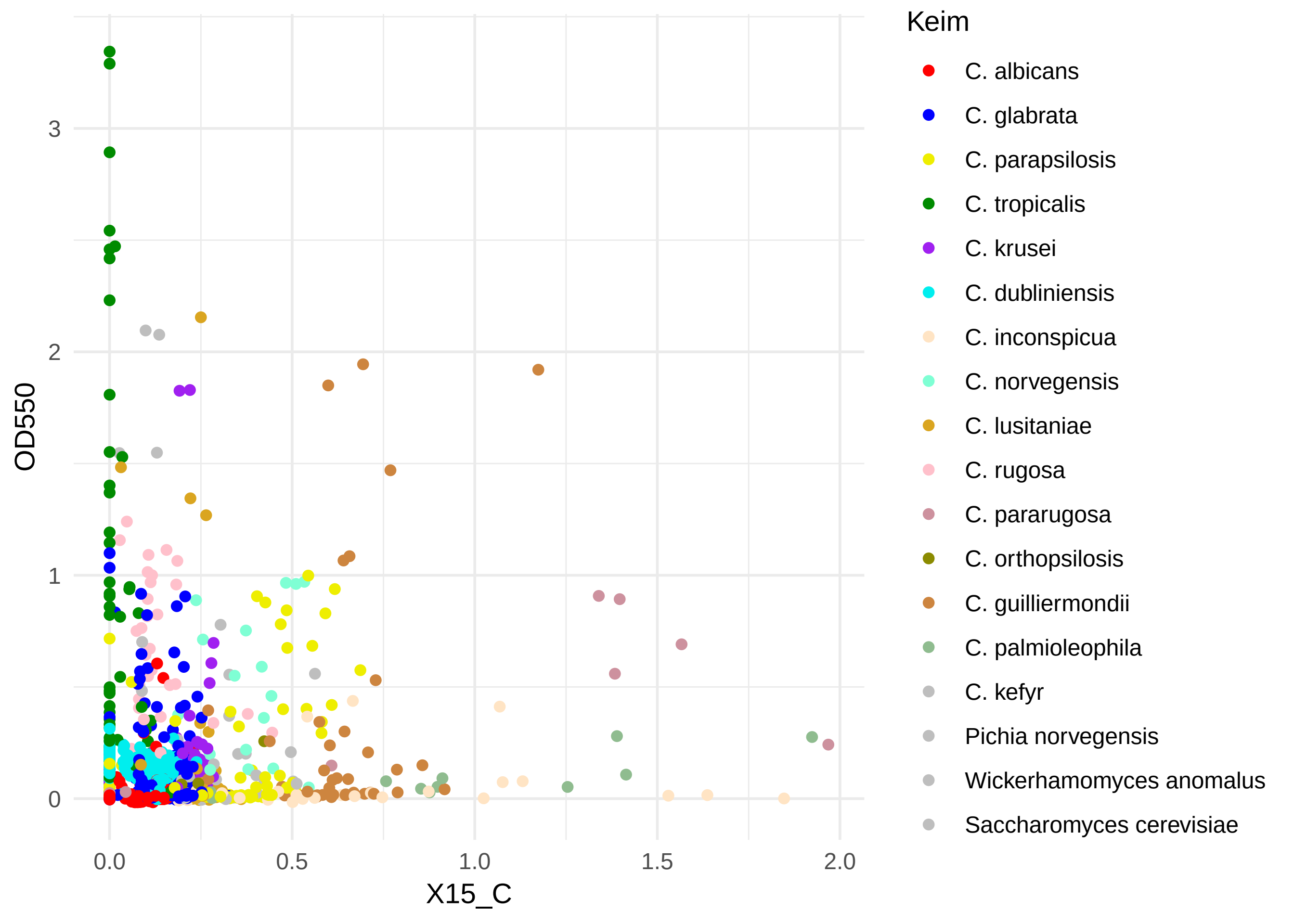

Keim
C. albicans
C. glabrata
C. parapsilosis
C. tropicalis
C. krusei
C. dubliniensis
C. inconspicua
C. norvegensis
C. lusitaniae
C. rugosa
C. pararugosa
C. orthopsilosis
C. guilliermondii
C. palmioleophila
C. kefyr
Pichia norvegensis
Wickerhamomyces anomalus
Saccharomyces cerevisiae
OD550
X15_C
0
1
2
3
0.0
0.5
1.0
1.5
2.0

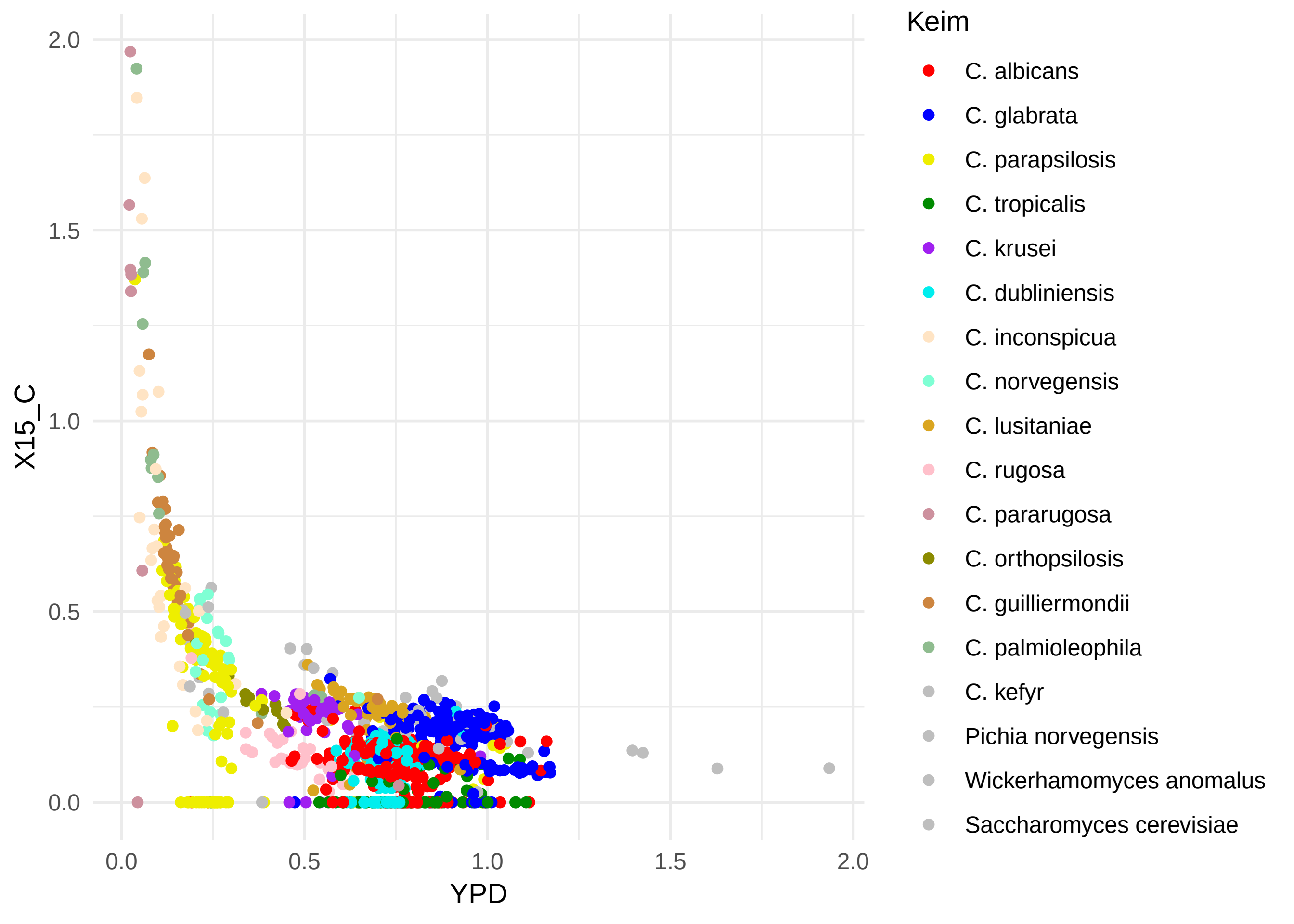

Keim
C. albicans
C. glabrata
C. parapsilosis
C. tropicalis
C. krusei
C. dubliniensis
C. inconspicua
C. norvegensis
C. lusitaniae
C. rugosa
C. pararugosa
C. orthopsilosis
C. guilliermondii
C. palmioleophila
C. kefyr
Pichia norvegensis
Wickerhamomyces anomalus
Saccharomyces cerevisiae
X15_C
YPD
0.0
0.5
1.0
1.5
2.0
0.0
0.5
1.0
1.5
2.0

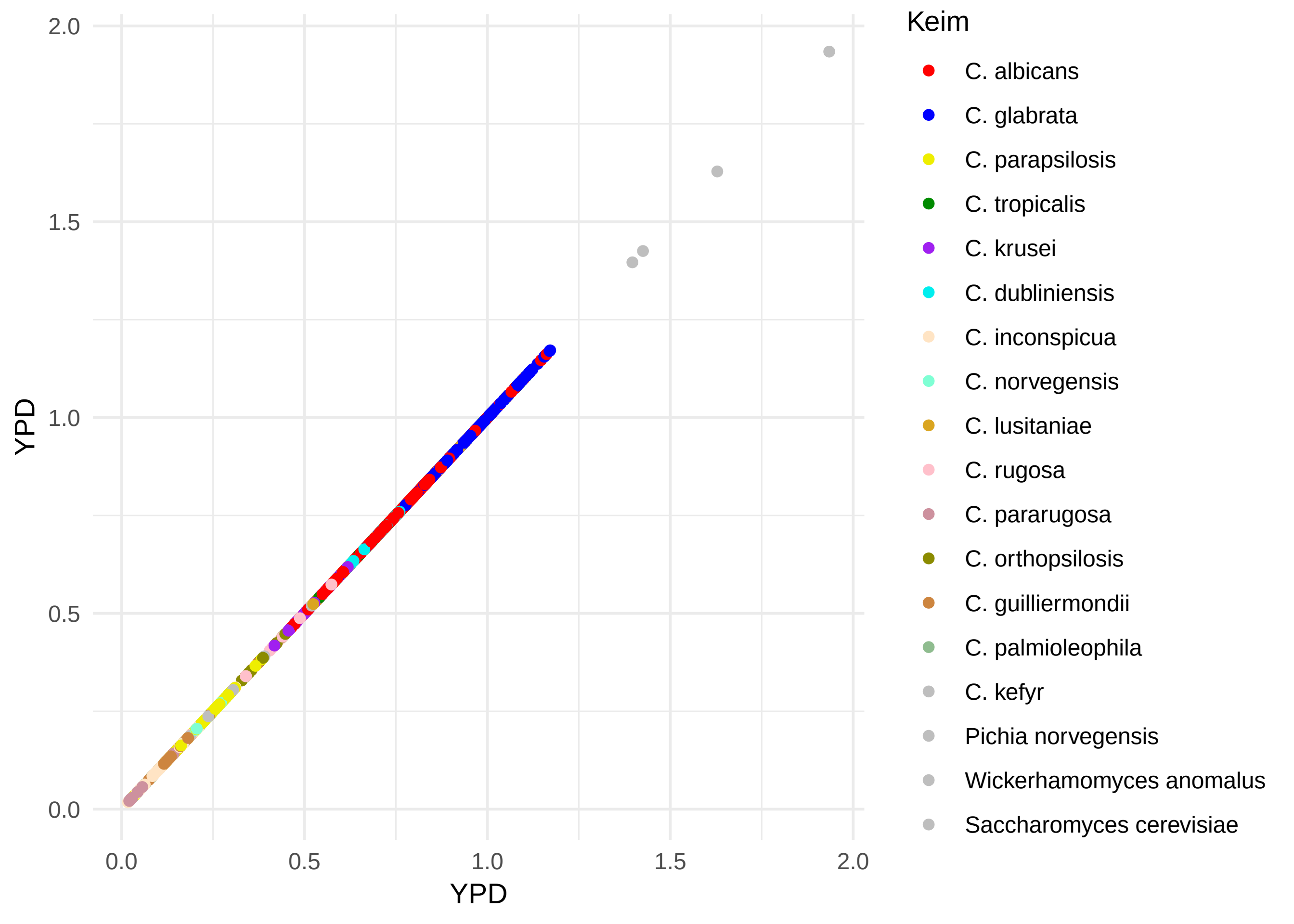
Keim
C. albicans
C. glabrata
C. parapsilosis
C. tropicalis
C. krusei
C. dubliniensis
C. inconspicua
C. norvegensis
C. lusitaniae
C. rugosa
C. pararugosa
C. orthopsilosis
C. guilliermondii
C. palmioleophila
C. kefyr
Pichia norvegensis
Wickerhamomyces anomalus
Saccharomyces cerevisiae
YPD
YPD
0.0
0.5
1.0
1.5
2.0

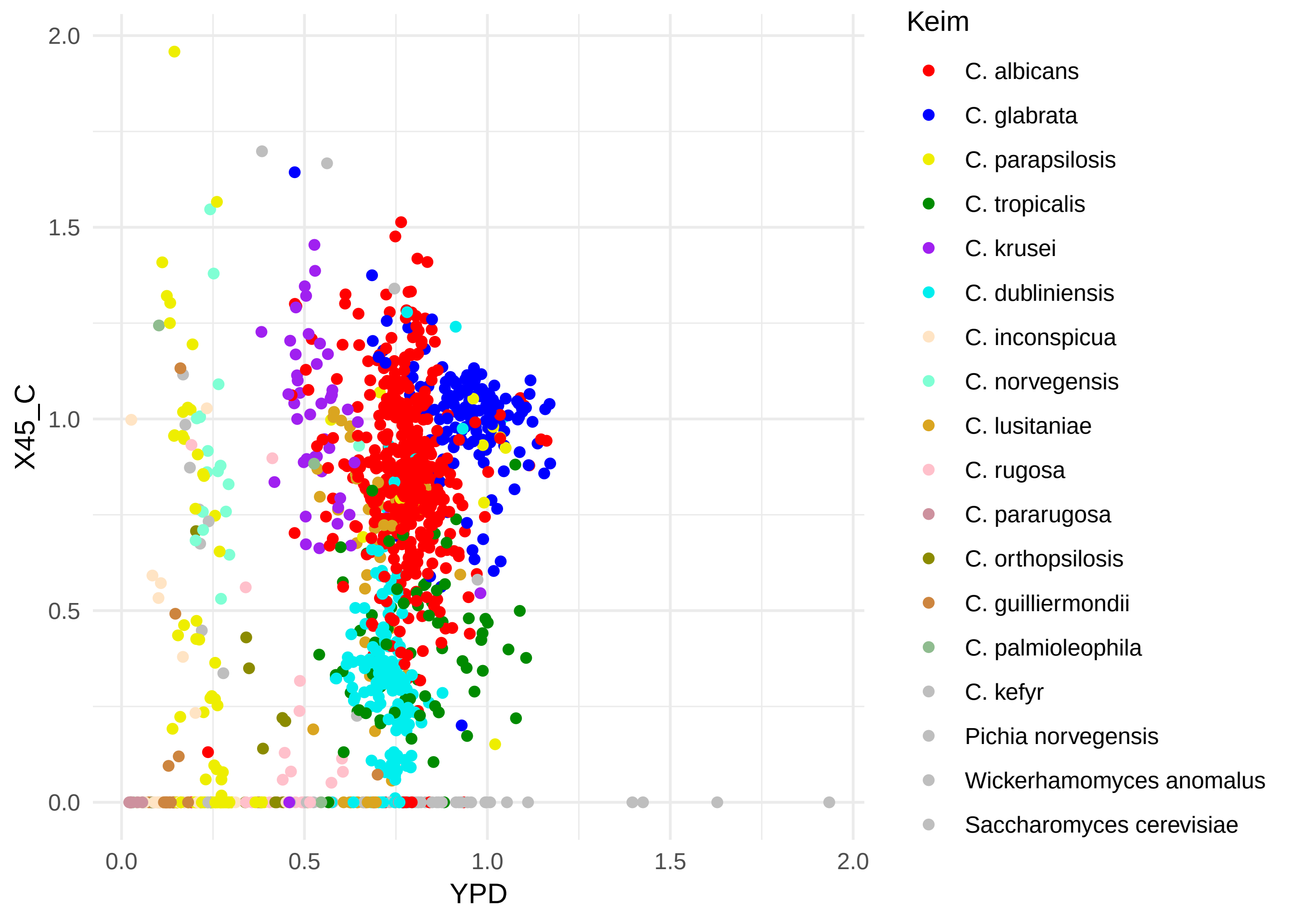
Keim
C. albicans
C. glabrata
C. parapsilosis
C. tropicalis
C. krusei
C. dubliniensis
C. inconspicua
C. norvegensis
C. lusitaniae
C. rugosa
C. pararugosa
C. orthopsilosis
C. guilliermondii
C. palmioleophila
C. kefyr
Pichia norvegensis
Wickerhamomyces anomalus
Saccharomyces cerevisiae
X45_C
YPD
0.0
0.5
1.0
1.5
2.0

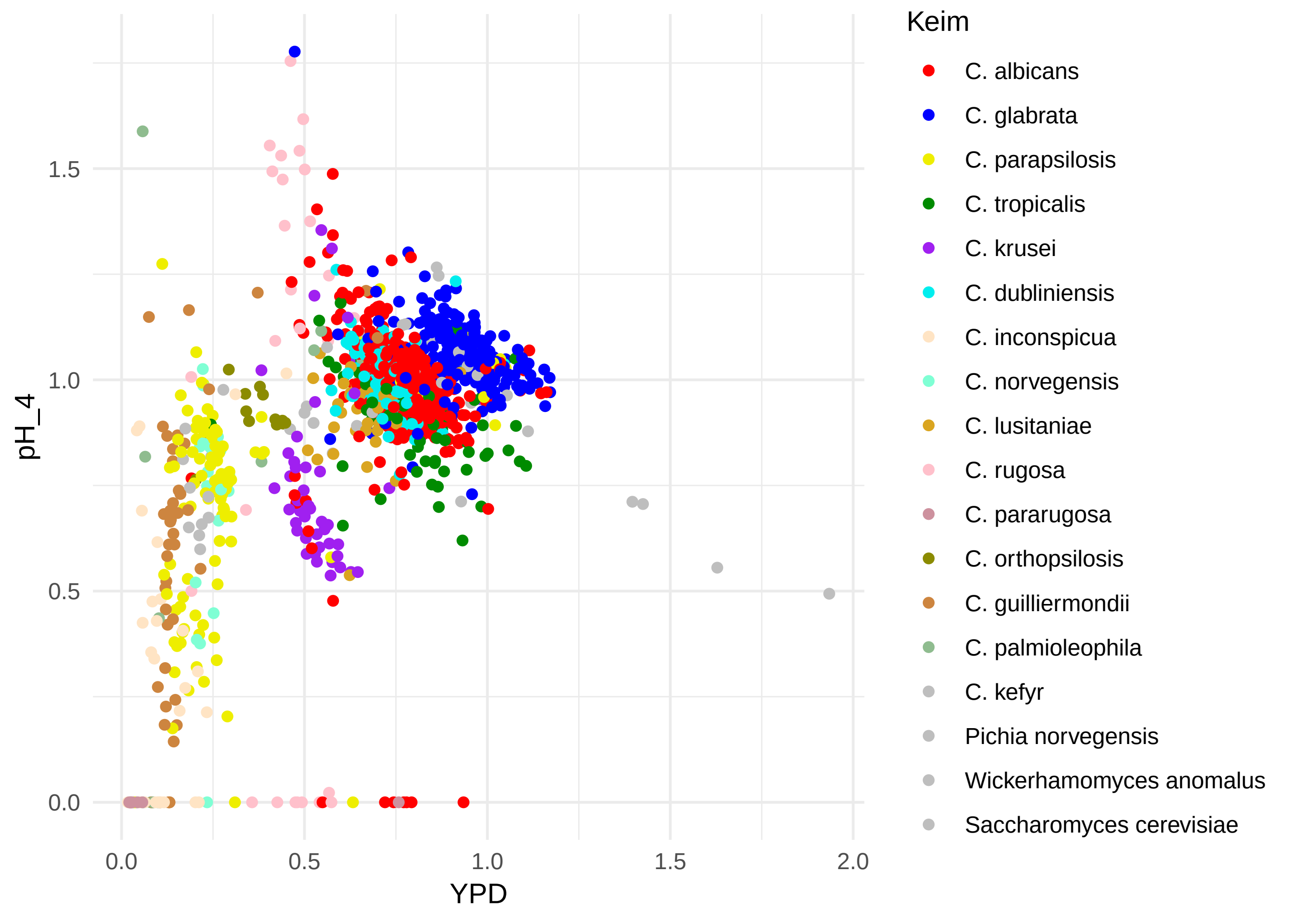
Keim
C. albicans
C. glabrata
C. parapsilosis
C. tropicalis
C. krusei
C. dubliniensis
C. inconspicua
C. norvegensis
C. lusitaniae
C. rugosa
C. pararugosa
C. orthopsilosis
C. guilliermondii
C. palmioleophila
C. kefyr
Pichia norvegensis
Wickerhamomyces anomalus
Saccharomyces cerevisiae
pH_4
YPD
0.0
0.5
1.0
1.5
2.0

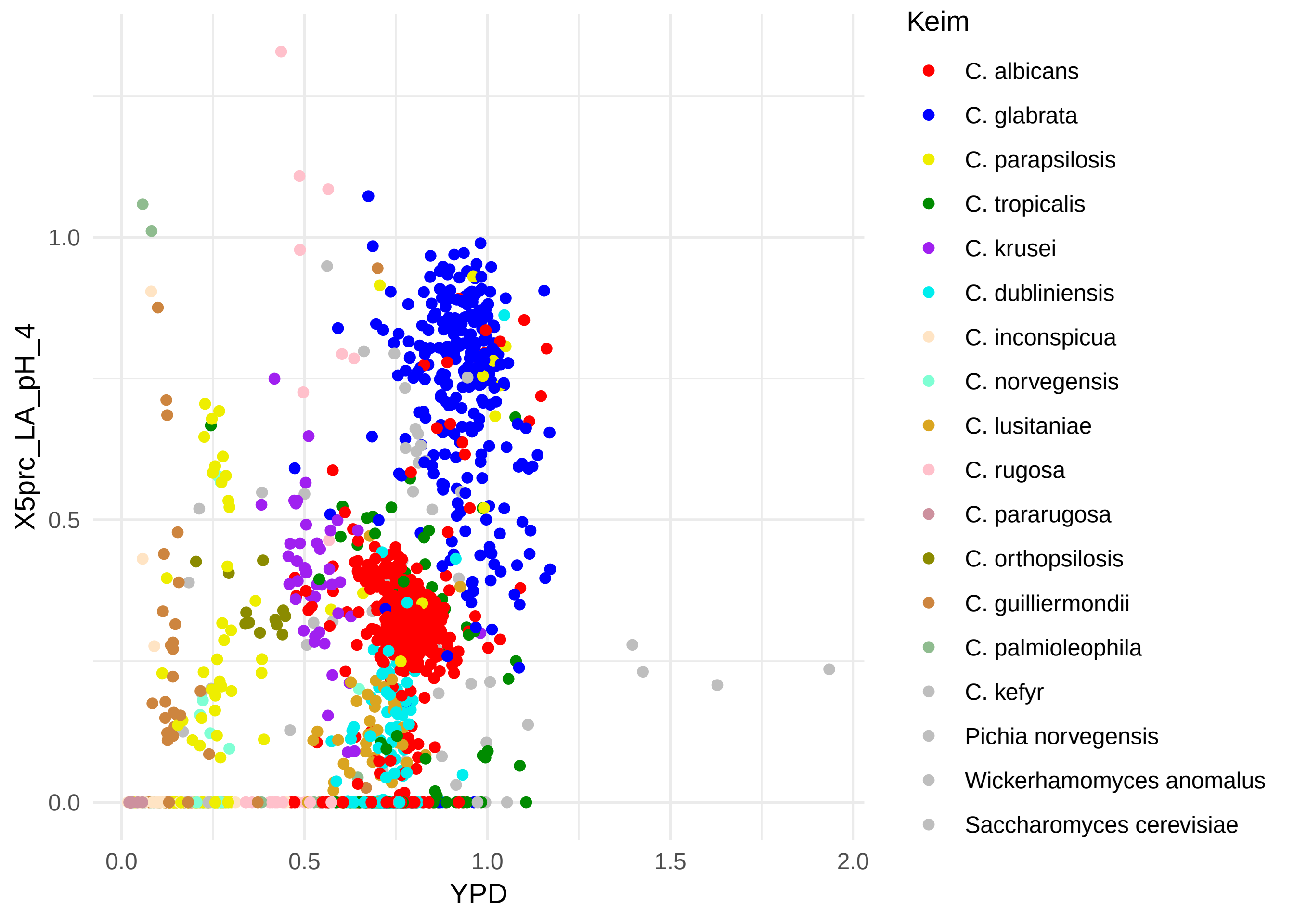
Keim
C. albicans
C. glabrata
C. parapsilosis
C. tropicalis
C. krusei
C. dubliniensis
C. inconspicua
C. norvegensis
C. lusitaniae
C. rugosa
C. pararugosa
C. orthopsilosis
C. guilliermondii
C. palmioleophila
C. kefyr
Pichia norvegensis
Wickerhamomyces anomalus
Saccharomyces cerevisiae
X5prc_LA_pH_4
YPD
0.0
0.5
1.0
0.0
0.5
1.0
1.5
2.0

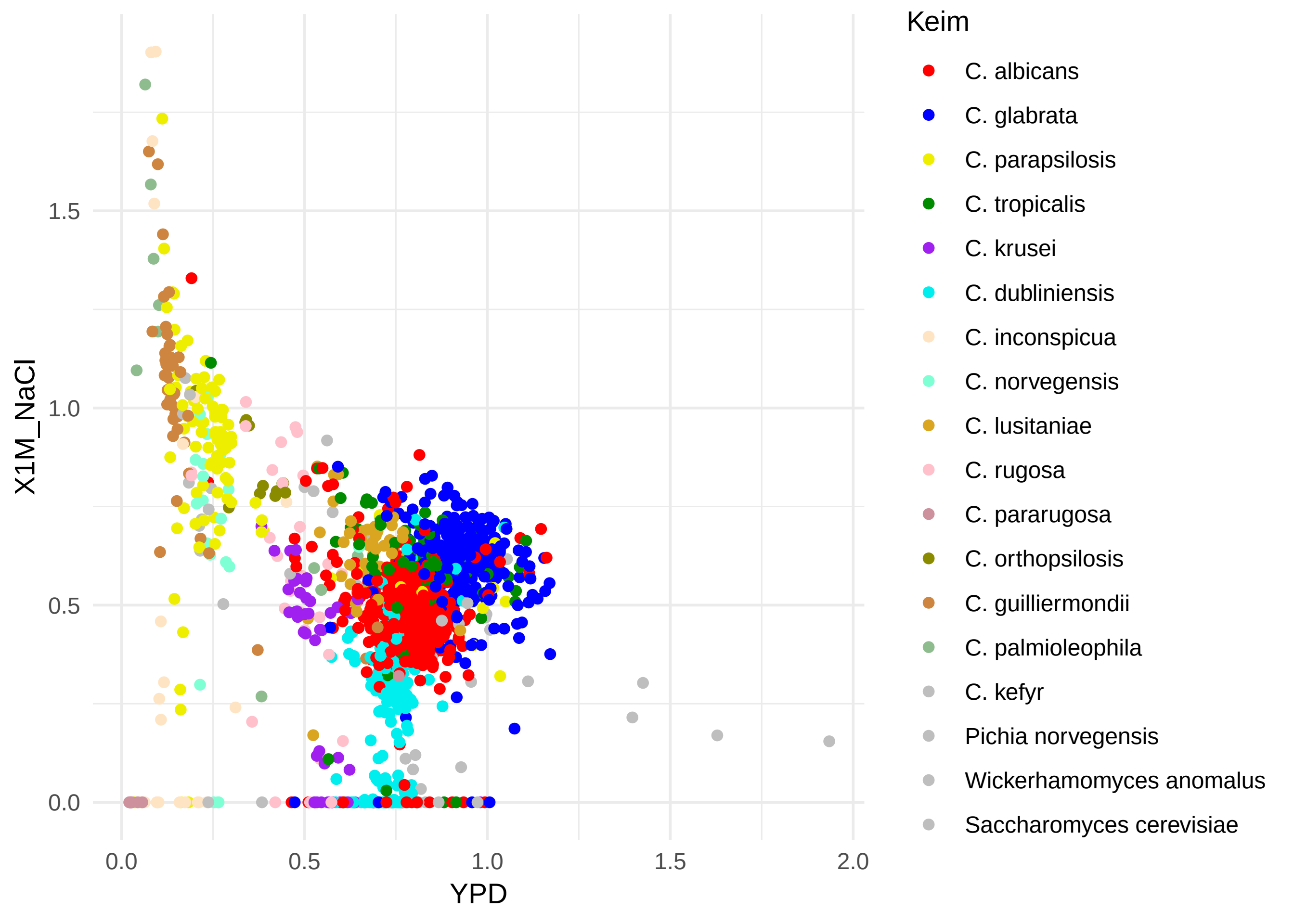

Keim
C. albicans
C. glabrata
C. parapsilosis
C. tropicalis
C. krusei
C. dubliniensis
C. inconspicua
C. norvegensis
C. lusitaniae
C. rugosa
C. pararugosa
C. orthopsilosis
C. guilliermondii
C. palmioleophila
C. kefyr
Pichia norvegensis
Wickerhamomyces anomalus
Saccharomyces cerevisiae
X1M_NaCl
YPD
0.0
0.5
1.0
1.5
2.0

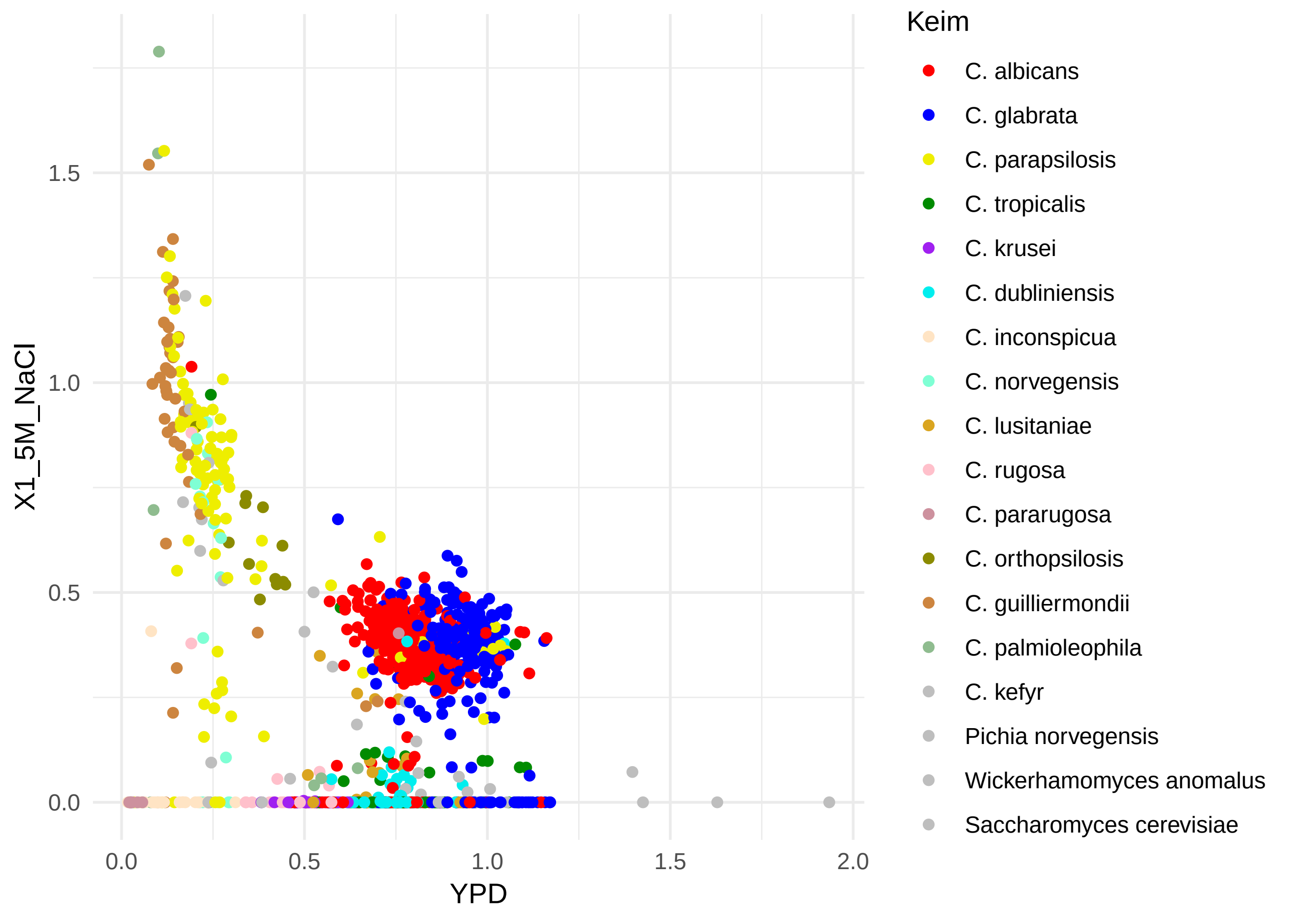
Keim
C. albicans
C. glabrata
C. parapsilosis
C. tropicalis
C. krusei
C. dubliniensis
C. inconspicua
C. norvegensis
C. lusitaniae
C. rugosa
C. pararugosa
C. orthopsilosis
C. guilliermondii
C. palmioleophila
C. kefyr
Pichia norvegensis
Wickerhamomyces anomalus
Saccharomyces cerevisiae
X1_5M_NaCl
0.0
0.5
1.0
1.5
YPD
0.0
0.5
1.0
1.5
2.0

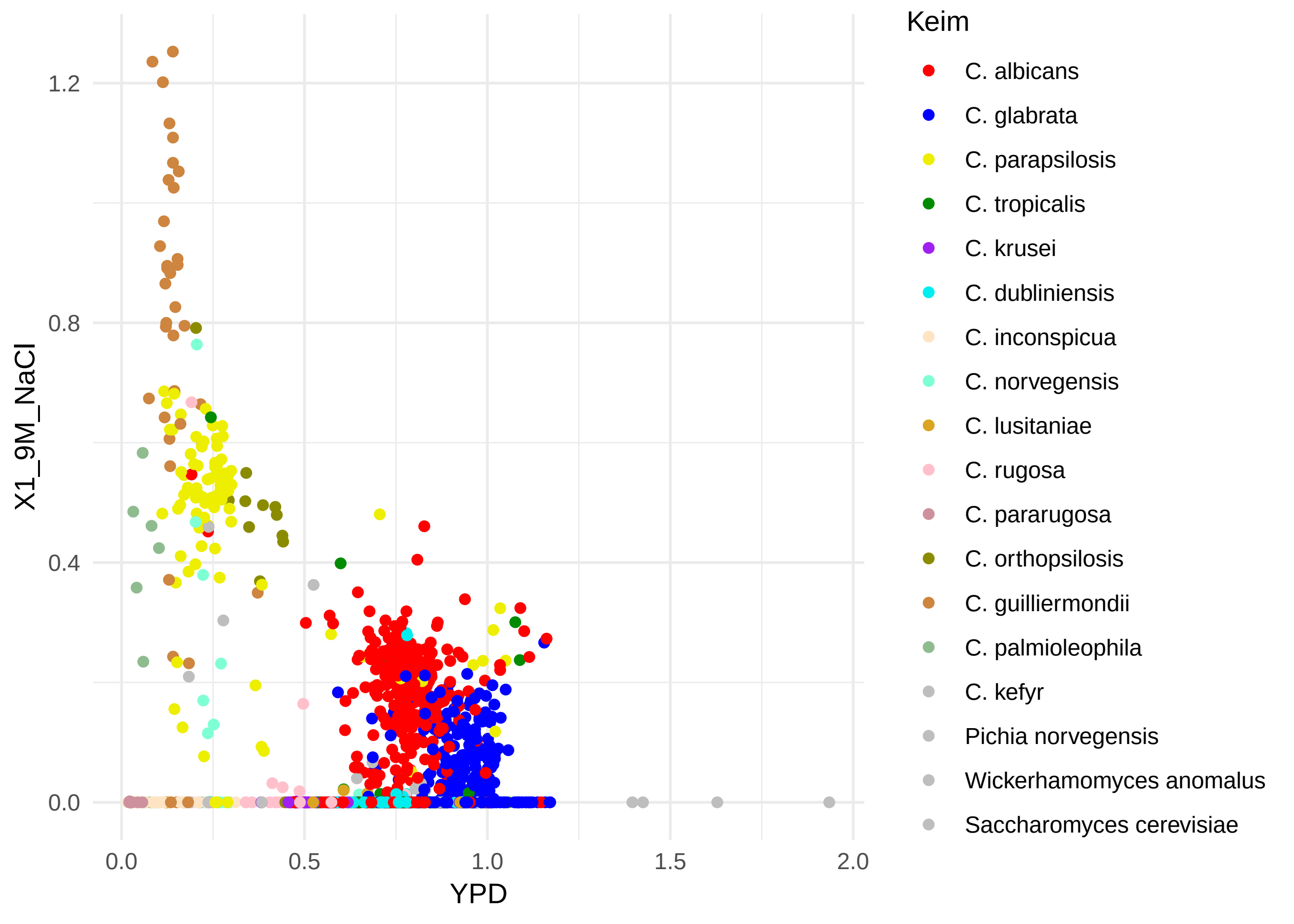
Keim
C. albicans
C. glabrata
C. parapsilosis
C. tropicalis
C. krusei
C. dubliniensis
C. inconspicua
C. norvegensis
C. lusitaniae
C. rugosa
C. pararugosa
C. orthopsilosis
C. guilliermondii
C. palmioleophila
C. kefyr
Pichia norvegensis
Wickerhamomyces anomalus
Saccharomyces cerevisiae
X1_9M_NaCl
YPD
0.0
0.4
0.8
1.2
0.0
0.5
1.0
1.5
2.0

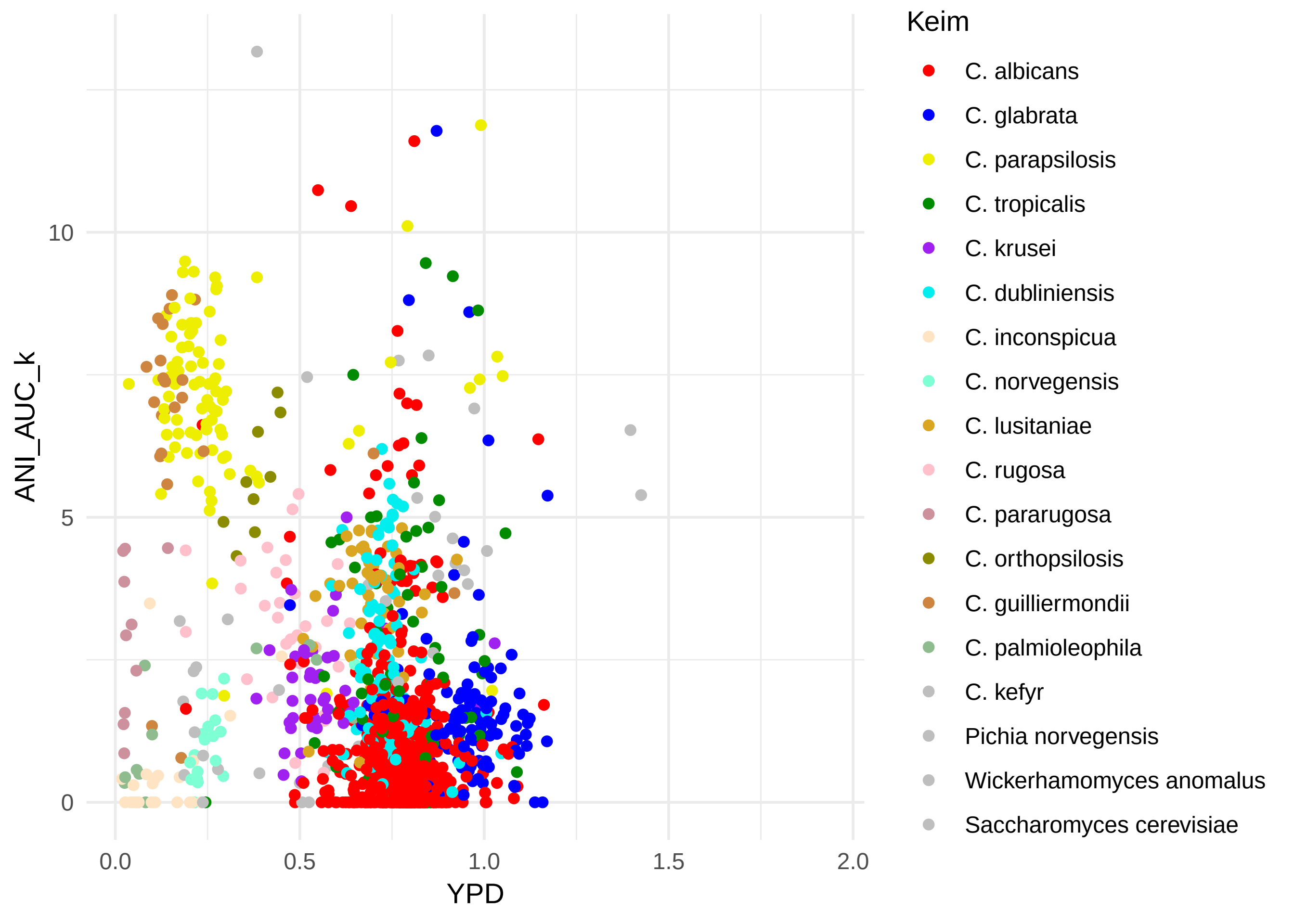

Keim
C. albicans
C. glabrata
C. parapsilosis
C. tropicalis
C. krusei
C. dubliniensis
C. inconspicua
C. norvegensis
C. lusitaniae
C. rugosa
C. pararugosa
C. orthopsilosis
C. guilliermondii
C. palmioleophila
C. kefyr
Pichia norvegensis
Wickerhamomyces anomalus
Saccharomyces cerevisiae
ANI_AUC_k
10
5
0
0.0
0.5
1.0
1.5
2.0
YPD

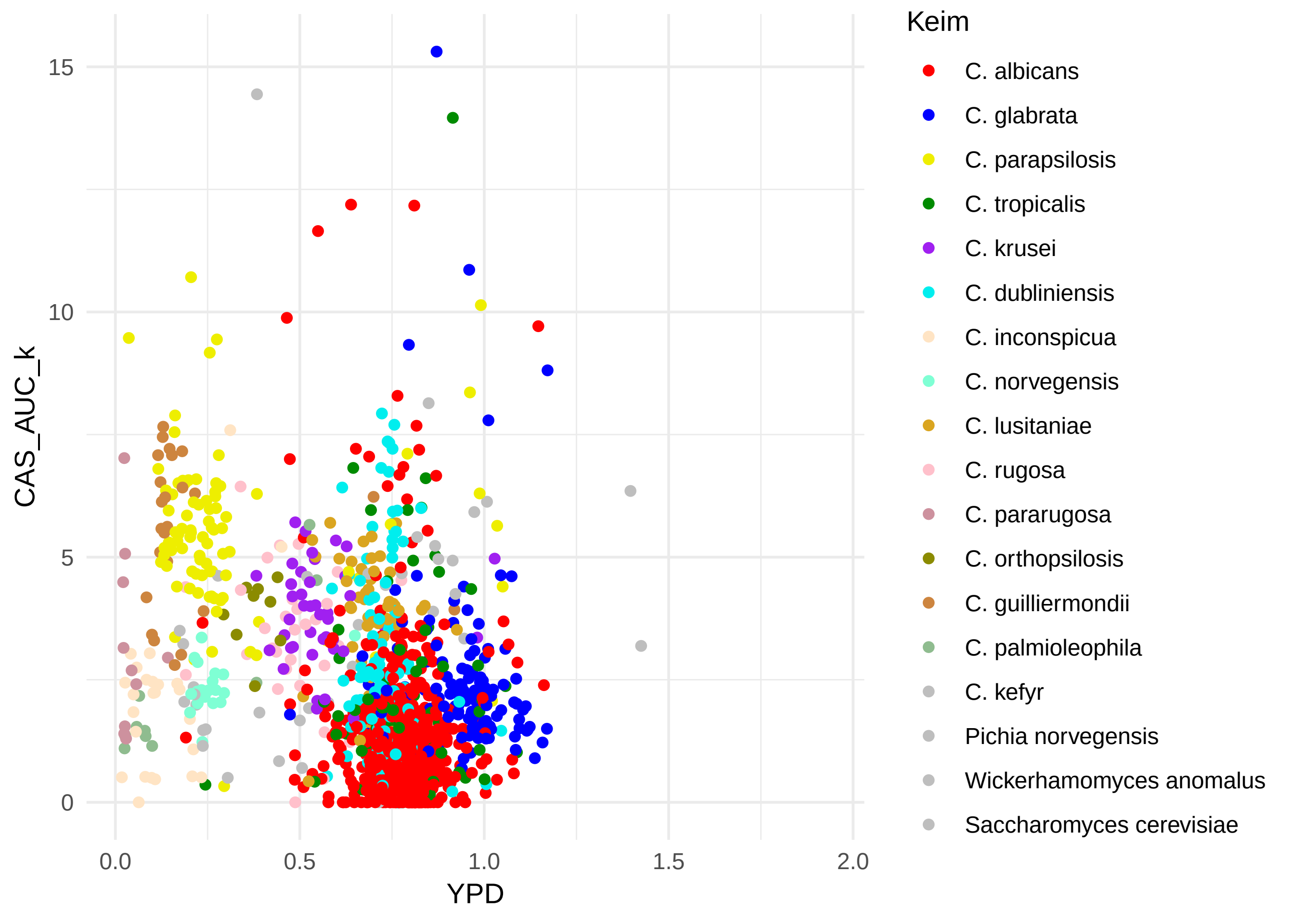

Keim
C. albicans
C. glabrata
C. parapsilosis
C. tropicalis
C. krusei
C. dubliniensis
C. inconspicua
C. norvegensis
C. lusitaniae
C. rugosa
C. pararugosa
C. orthopsilosis
C. guilliermondii
C. palmioleophila
C. kefyr
Pichia norvegensis
Wickerhamomyces anomalus
Saccharomyces cerevisiae
CAS_AUC_k
YPD
0
5
10
15
0.0
0.5
1.0
1.5
2.0

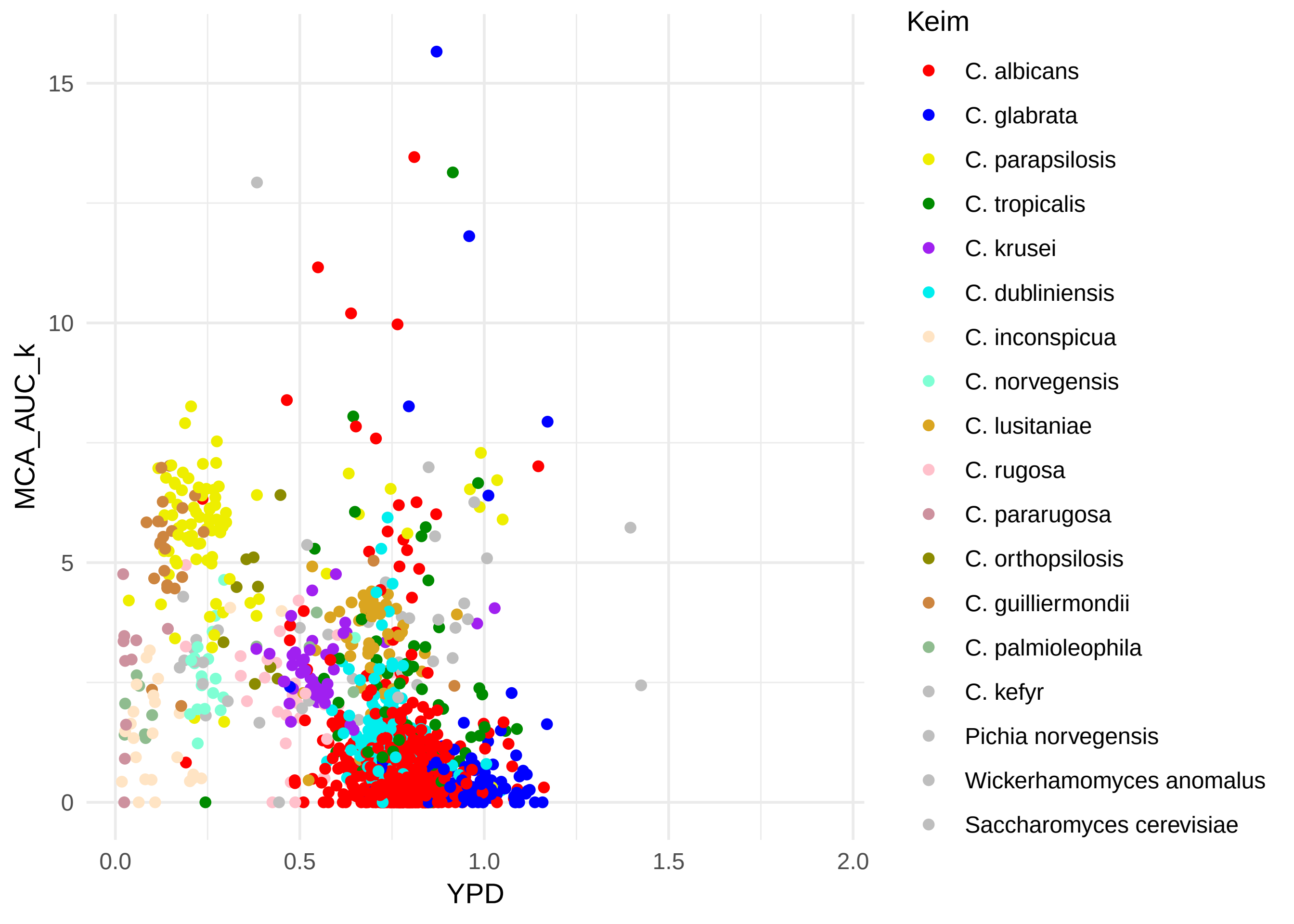
Keim
C. albicans
C. glabrata
C. parapsilosis
C. tropicalis
C. krusei
C. dubliniensis
C. inconspicua
C. norvegensis
C. lusitaniae
C. rugosa
C. pararugosa
C. orthopsilosis
C. guilliermondii
C. palmioleophila
C. kefyr
Pichia norvegensis
Wickerhamomyces anomalus
Saccharomyces cerevisiae
MCA_AUC_k
YPD
0
5
10
15
0.0
0.5
1.0
1.5
2.0

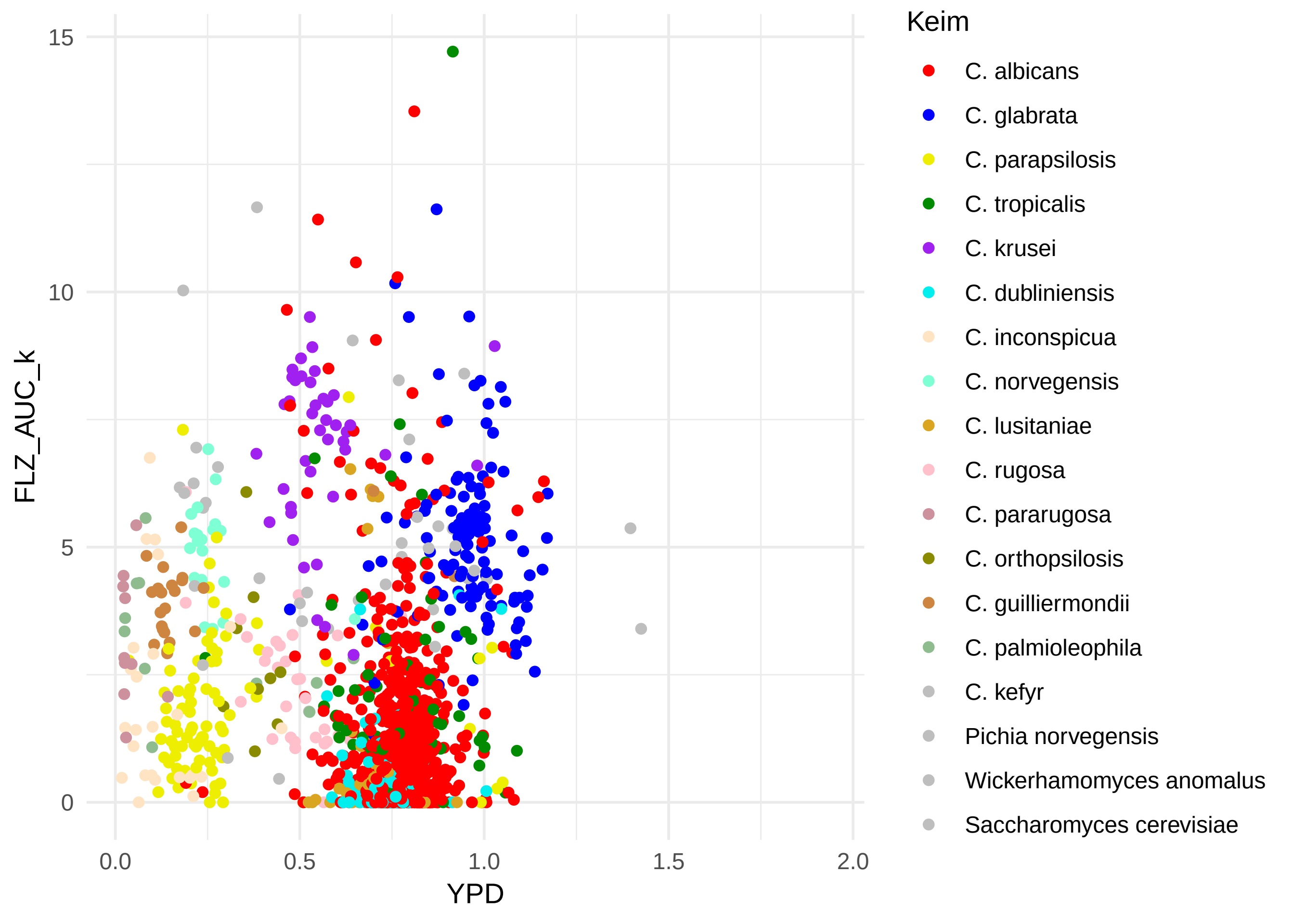

15
10
5
0
FLZ_AUC_k
0.0
0.5
1.0
1.5
2.0
YPD
Keim
C. albicans
C. glabrata
C. parapsilosis
C. tropicalis
C. krusei
C. dubliniensis
C. inconspicua
C. norvegensis
C. lusitaniae
C. rugosa
C. pararugosa
C. orthopsilosis
C. guilliermondii
C. palmioleophila
C. kefyr
Pichia norvegensis
Wickerhamomyces anomalus
Saccharomyces cerevisiae

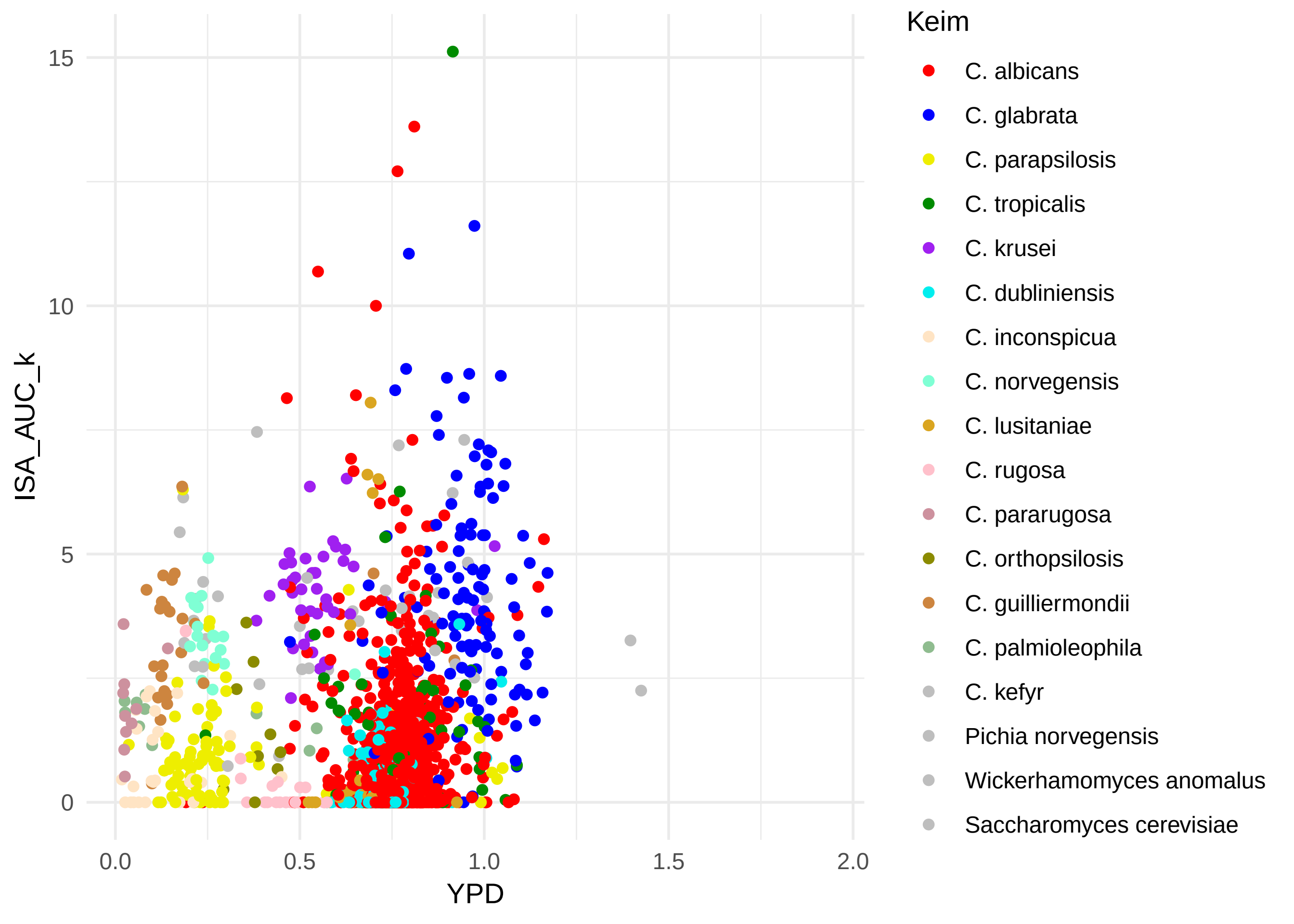

Keim
C. albicans
C. glabrata
C. parapsilosis
C. tropicalis
C. krusei
C. dubliniensis
C. inconspicua
C. norvegensis
C. lusitaniae
C. rugosa
C. pararugosa
C. orthopsilosis
C. guilliermondii
C. palmioleophila
C. kefyr
Pichia norvegensis
Wickerhamomyces anomalus
Saccharomyces cerevisiae
ISA_AUC_k
YPD
15
10
5
0
0.0
0.5
1.0
1.5
2.0

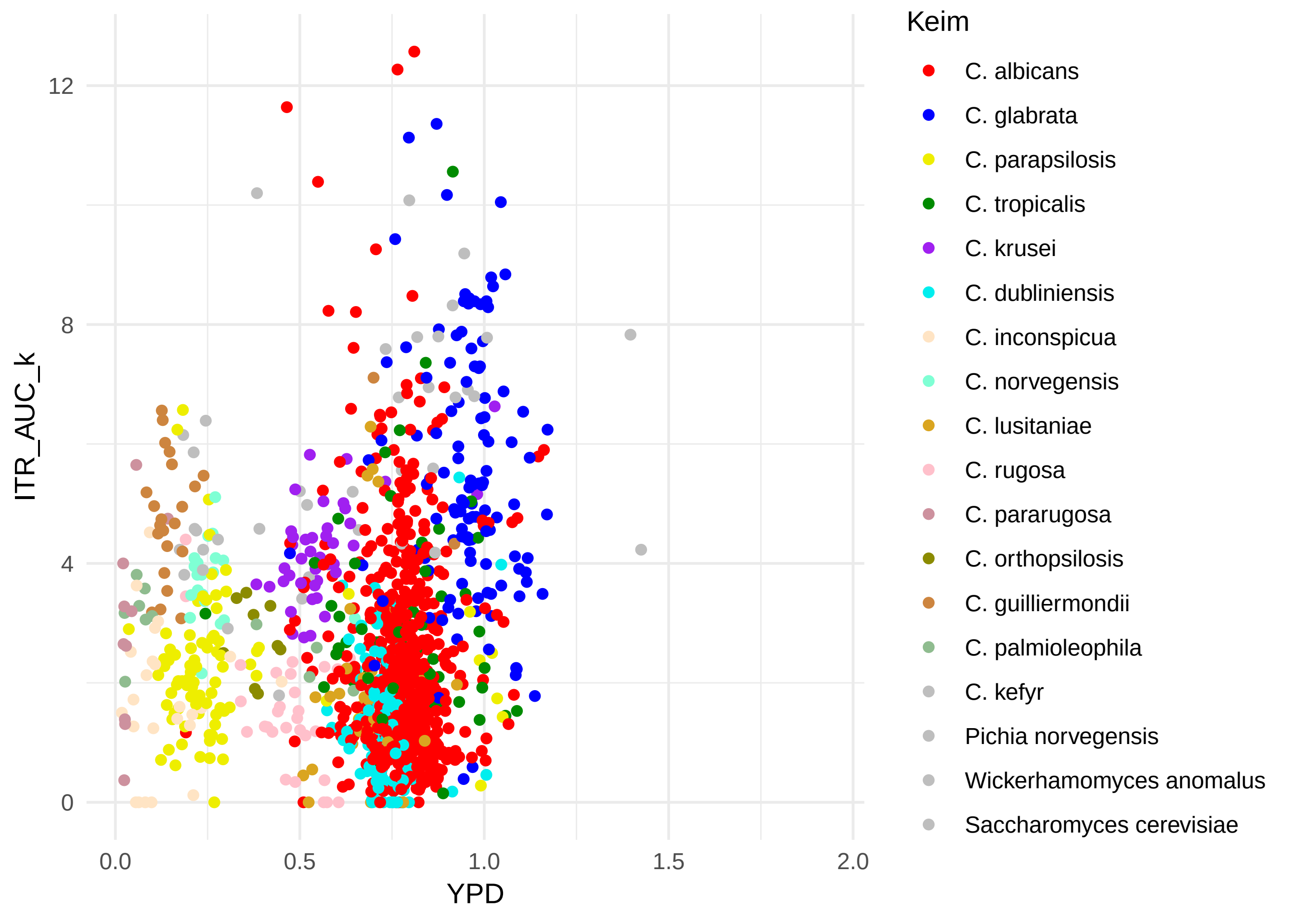
Keim
C. albicans
C. glabrata
C. parapsilosis
C. tropicalis
C. krusei
C. dubliniensis
C. inconspicua
C. norvegensis
C. lusitaniae
C. rugosa
C. pararugosa
C. orthopsilosis
C. guilliermondii
C. palmioleophila
C. kefyr
Pichia norvegensis
Wickerhamomyces anomalus
Saccharomyces cerevisiae
ITR_AUC_k
0
4
8
12
0.0
0.5
1.0
1.5
2.0
YPD

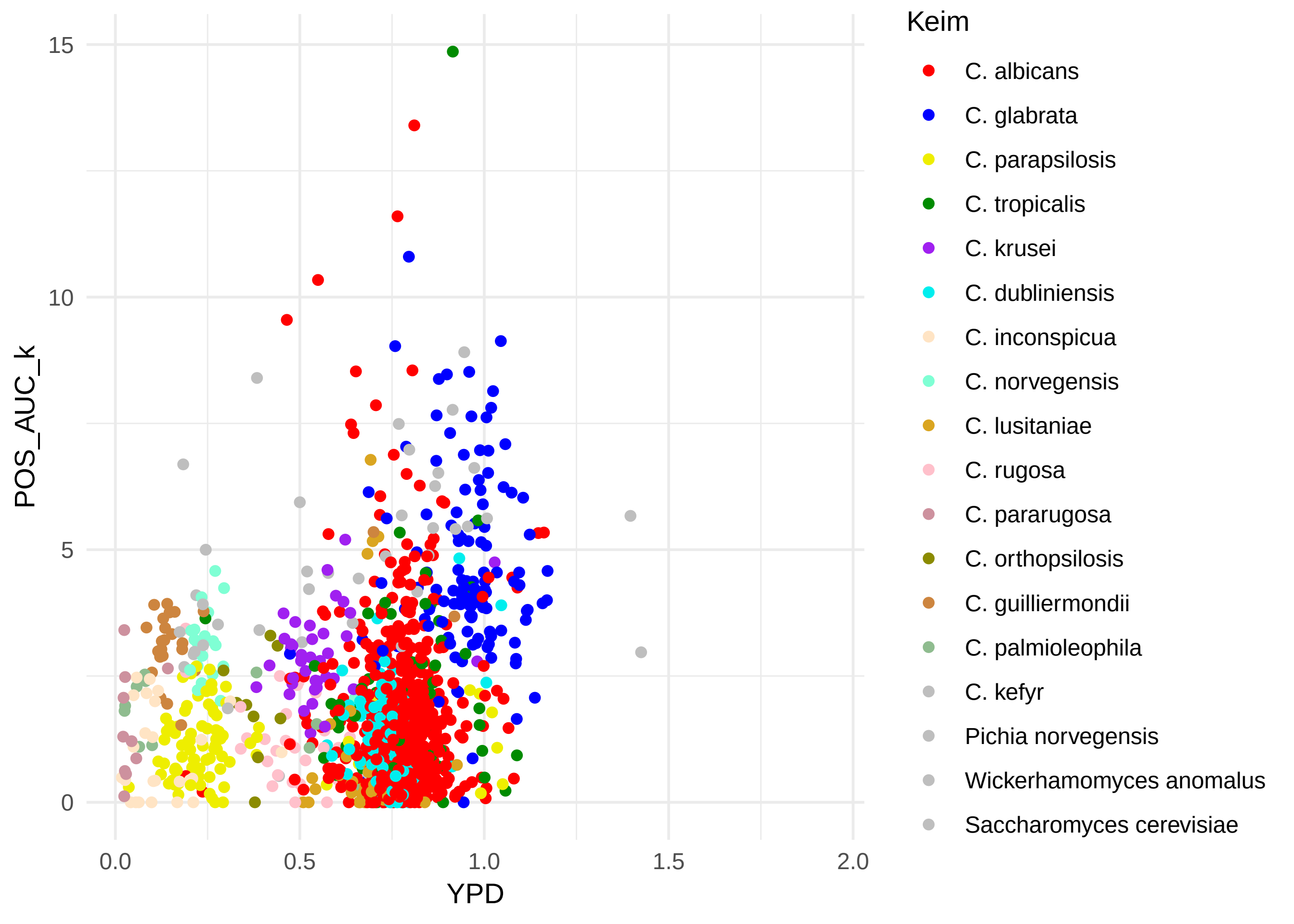

POS_AUC_k
15
10
5
0
0.0
0.5
1.0
1.5
2.0
YPD
Keim
C. albicans
C. glabrata
C. parapsilosis
C. tropicalis
C. krusei
C. dubliniensis
C. inconspicua
C. norvegensis
C. lusitaniae
C. rugosa
C. pararugosa
C. orthopsilosis
C. guilliermondii
C. palmioleophila
C. kefyr
Pichia norvegensis
Wickerhamomyces anomalus
Saccharomyces cerevisiae

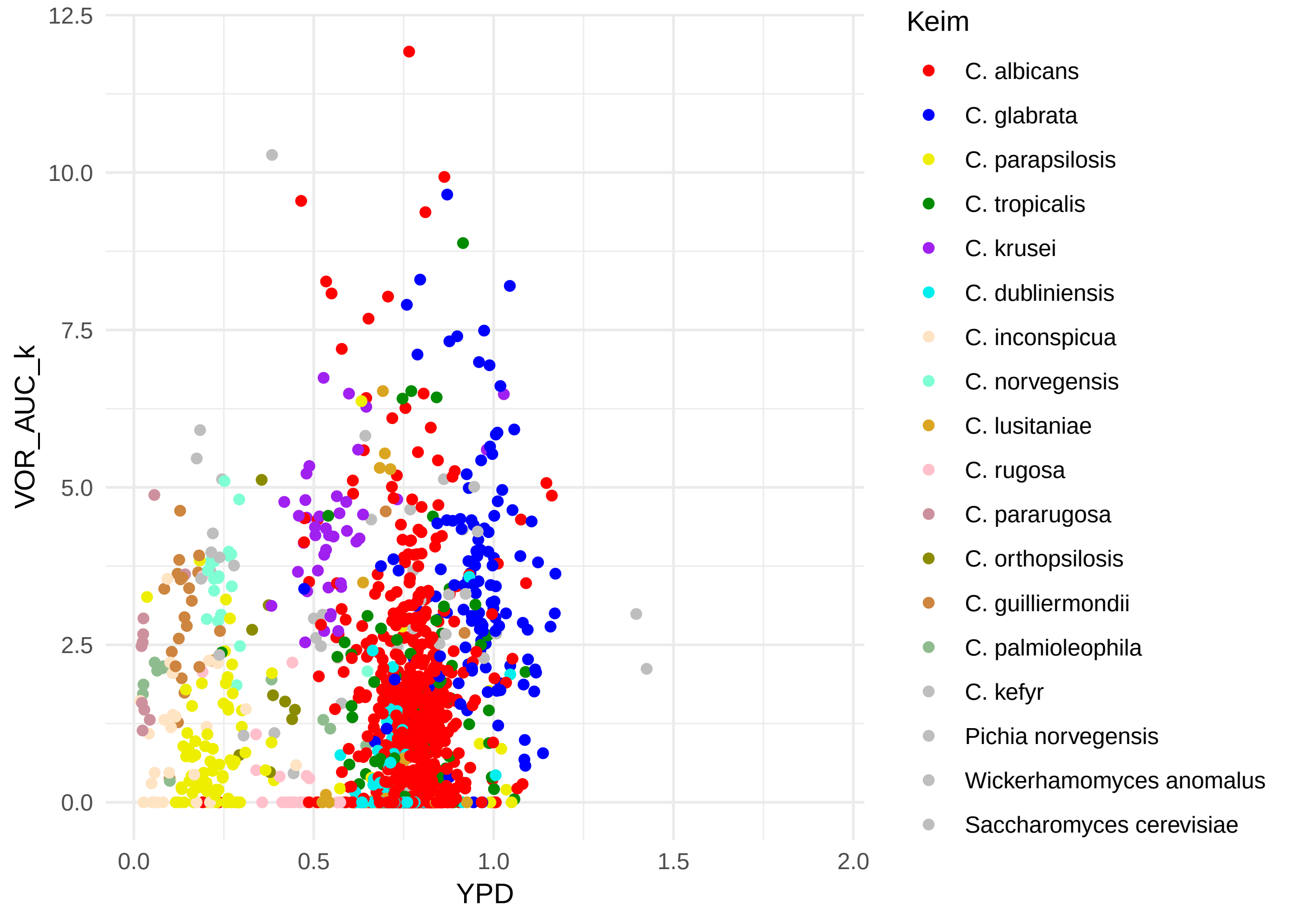
12.5
10.0
7.5
5.0
2.5
0.0
VOR_AUC_k
0.0
0.5
1.0
1.5
2.0
YPD
Keim
C. albicans
C. glabrata
C. parapsilosis
C. tropicalis
C. krusei
C. dubliniensis
C. inconspicua
C. norvegensis
C. lusitaniae
C. rugosa
C. pararugosa
C. orthopsilosis
C. guilliermondii
C. palmioleophila
C. kefyr
Pichia norvegensis
Wickerhamomyces anomalus
Saccharomyces cerevisiae

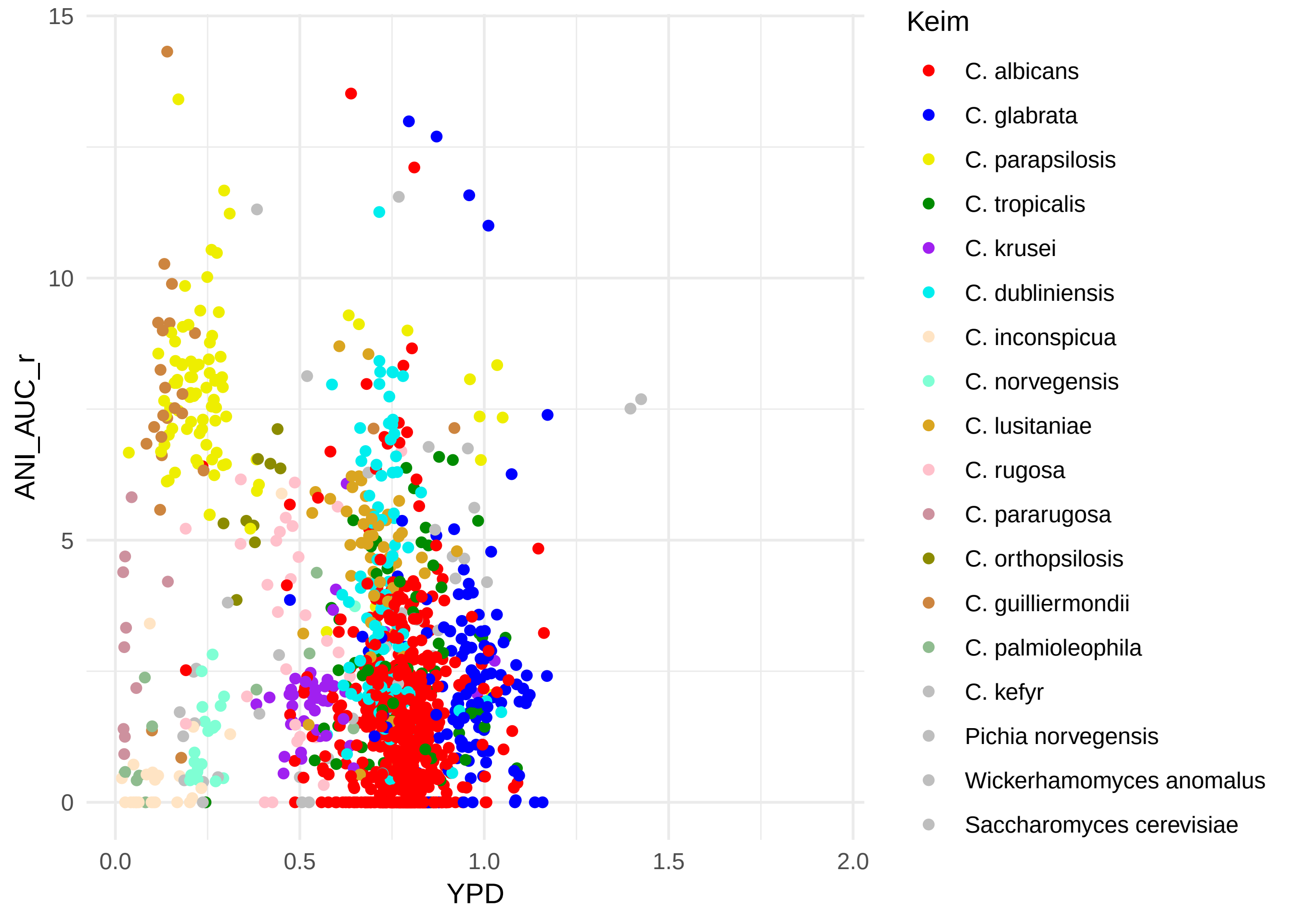

15
10
5
0
ANI_AUC_r
0.0
0.5
1.0
1.5
2.0
YPD
Keim
C. albicans
C. glabrata
C. parapsilosis
C. tropicalis
C. krusei
C. dubliniensis
C. inconspicua
C. norvegensis
C. lusitaniae
C. rugosa
C. pararugosa
C. orthopsilosis
C. guilliermondii
C. palmioleophila
C. kefyr
Pichia norvegensis
Wickerhamomyces anomalus
Saccharomyces cerevisiae

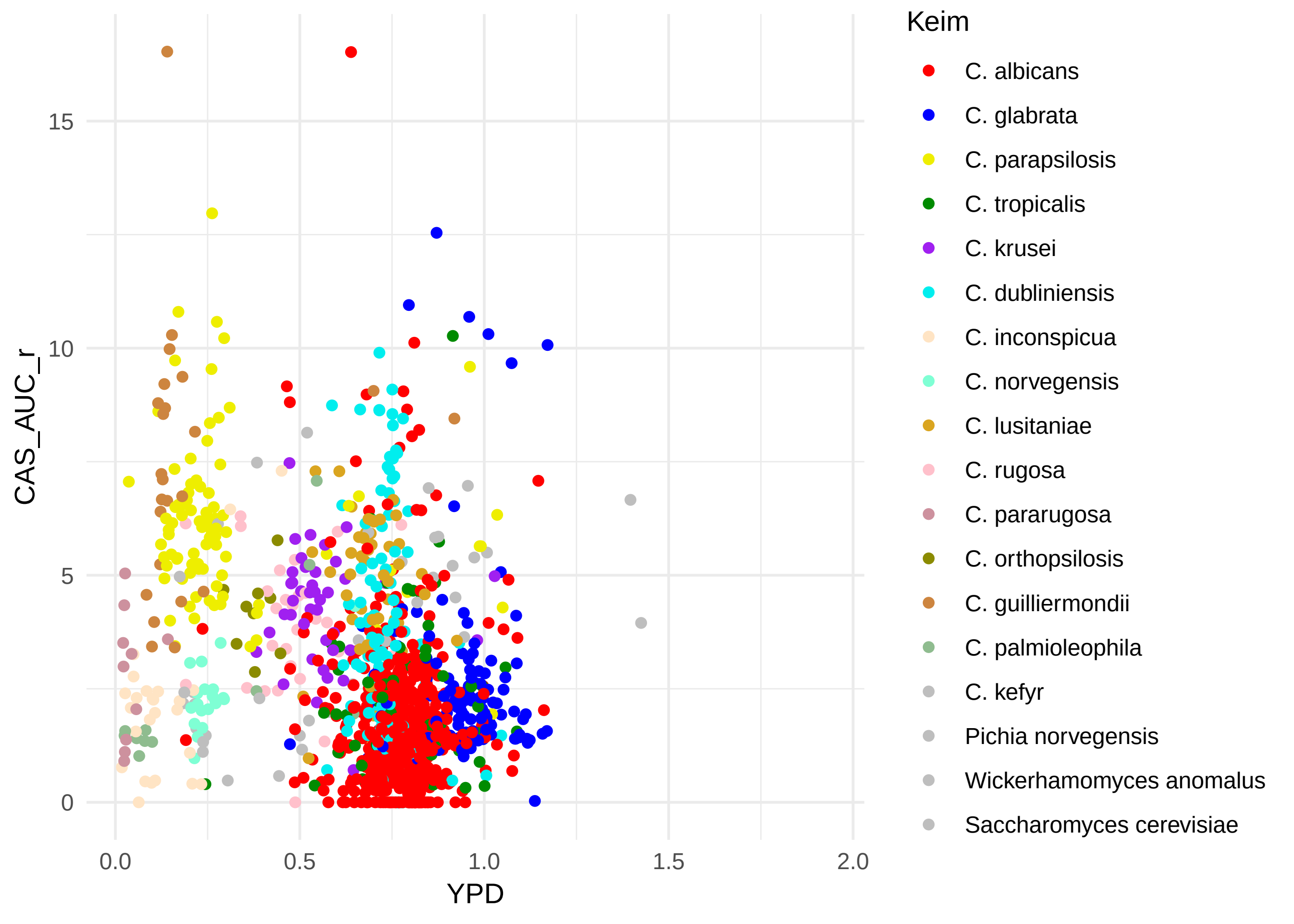

Keim
C. albicans
C. glabrata
C. parapsilosis
C. tropicalis
C. krusei
C. dubliniensis
C. inconspicua
C. norvegensis
C. lusitaniae
C. rugosa
C. pararugosa
C. orthopsilosis
C. guilliermondii
C. palmioleophila
C. kefyr
Pichia norvegensis
Wickerhamomyces anomalus
Saccharomyces cerevisiae
CAS_AUC_r
YPD
0
5
10
15
0.0
0.5
1.0
1.5
2.0

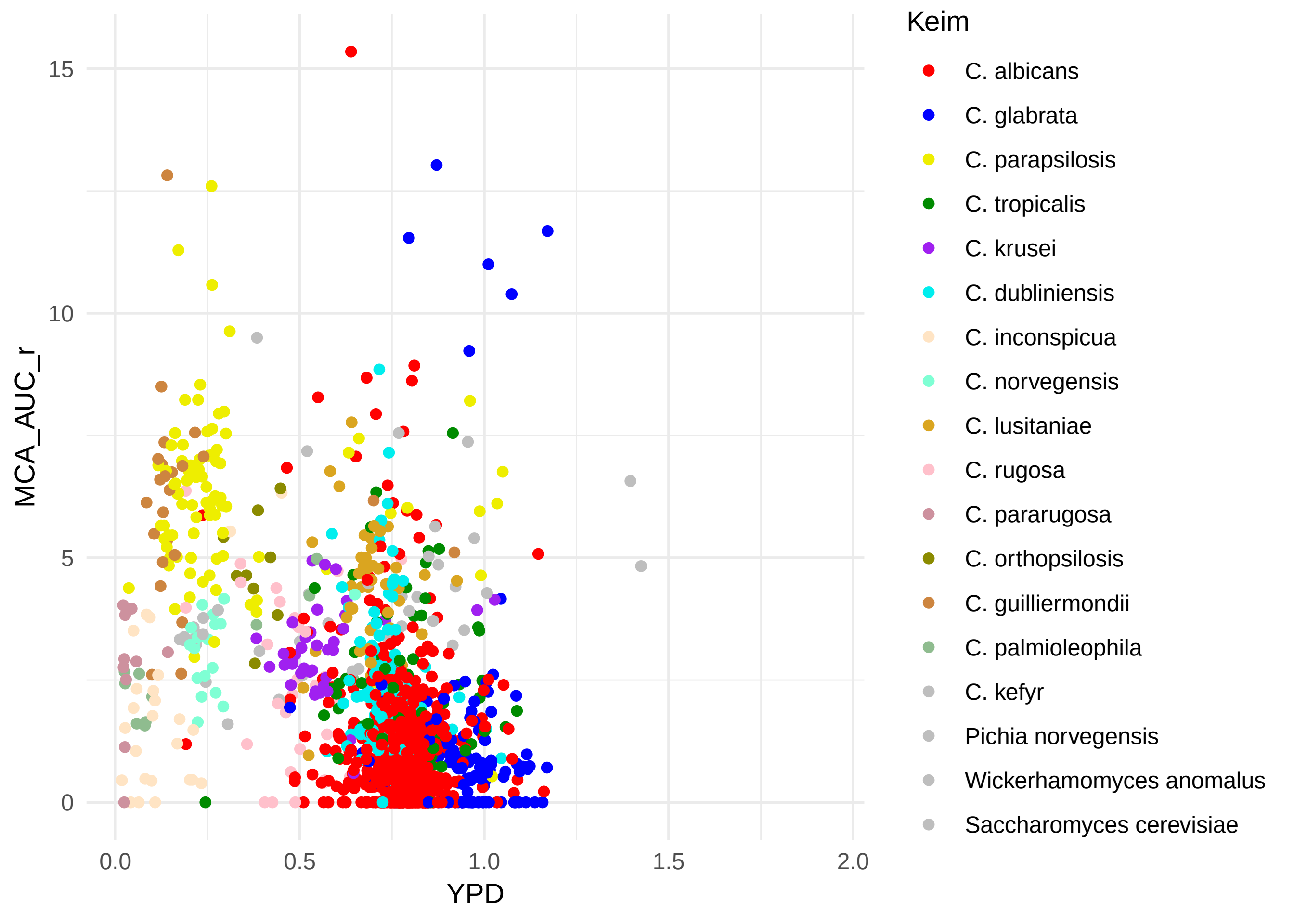

Keim
C. albicans
C. glabrata
C. parapsilosis
C. tropicalis
C. krusei
C. dubliniensis
C. inconspicua
C. norvegensis
C. lusitaniae
C. rugosa
C. pararugosa
C. orthopsilosis
C. guilliermondii
C. palmioleophila
C. kefyr
Pichia norvegensis
Wickerhamomyces anomalus
Saccharomyces cerevisiae
MCA_AUC_r
15
10
5
0
0.0
0.5
1.0
1.5
2.0
YPD

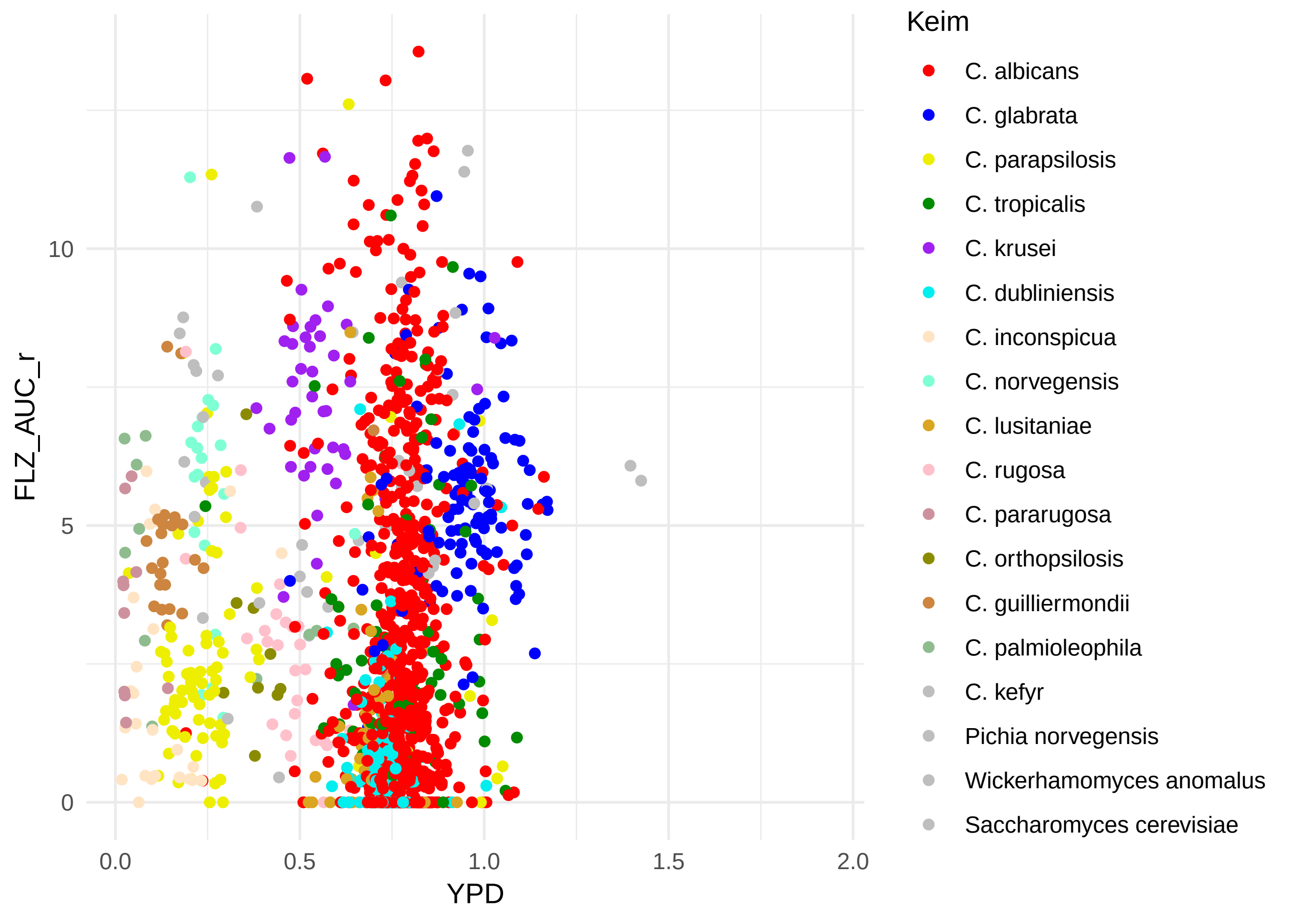
Keim
C. albicans
C. glabrata
C. parapsilosis
C. tropicalis
C. krusei
C. dubliniensis
C. inconspicua
C. norvegensis
C. lusitaniae
C. rugosa
C. pararugosa
C. orthopsilosis
C. guilliermondii
C. palmioleophila
C. kefyr
Pichia norvegensis
Wickerhamomyces anomalus
Saccharomyces cerevisiae
FLZ_AUC_r
10
5
0
0.0
0.5
1.0
1.5
2.0
YPD

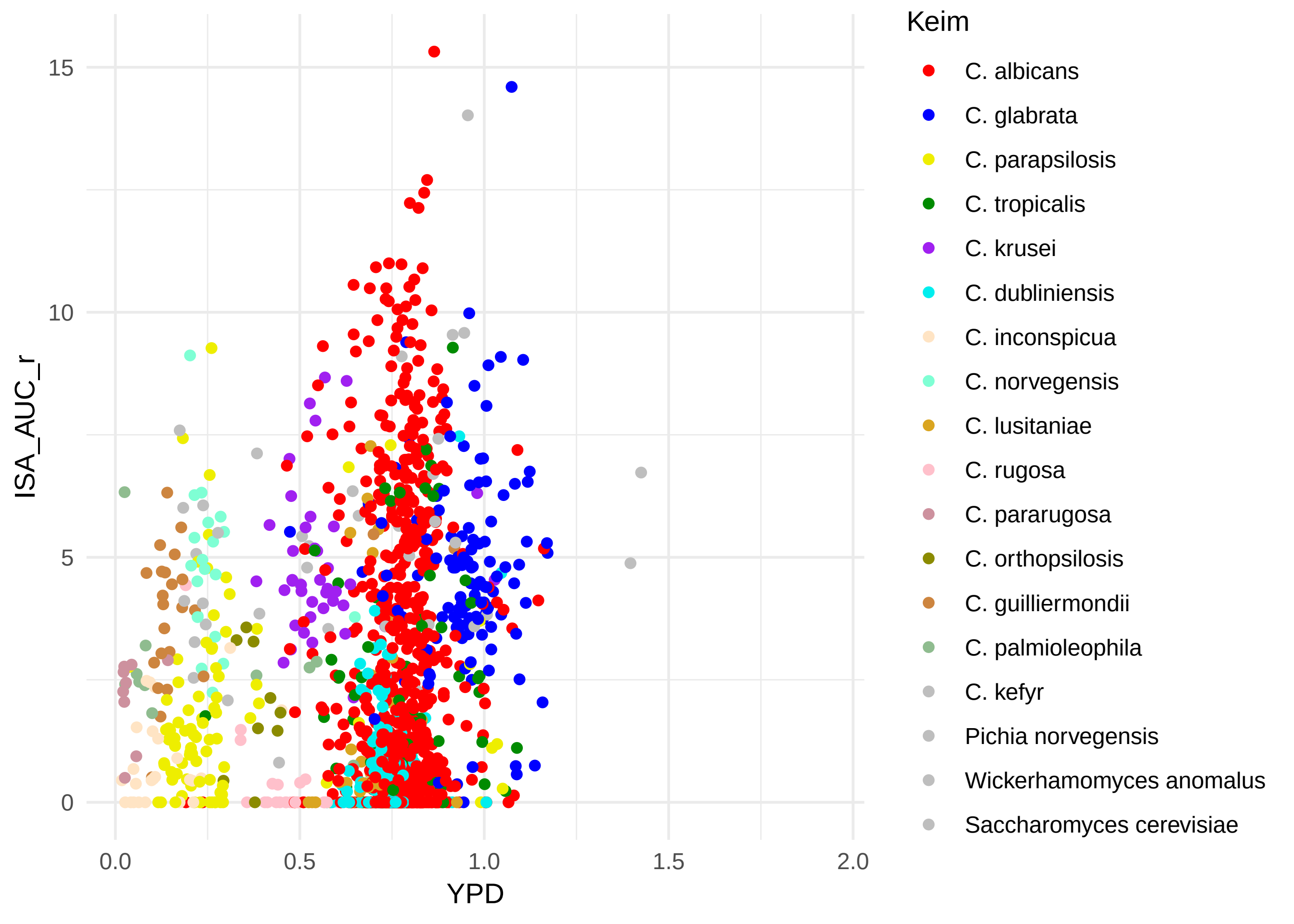

Keim
C. albicans
C. glabrata
C. parapsilosis
C. tropicalis
C. krusei
C. dubliniensis
C. inconspicua
C. norvegensis
C. lusitaniae
C. rugosa
C. pararugosa
C. orthopsilosis
C. guilliermondii
C. palmioleophila
C. kefyr
Pichia norvegensis
Wickerhamomyces anomalus
Saccharomyces cerevisiae
ISA_AUC_r
YPD
0
5
10
15
0.0
0.5
1.0
1.5
2.0

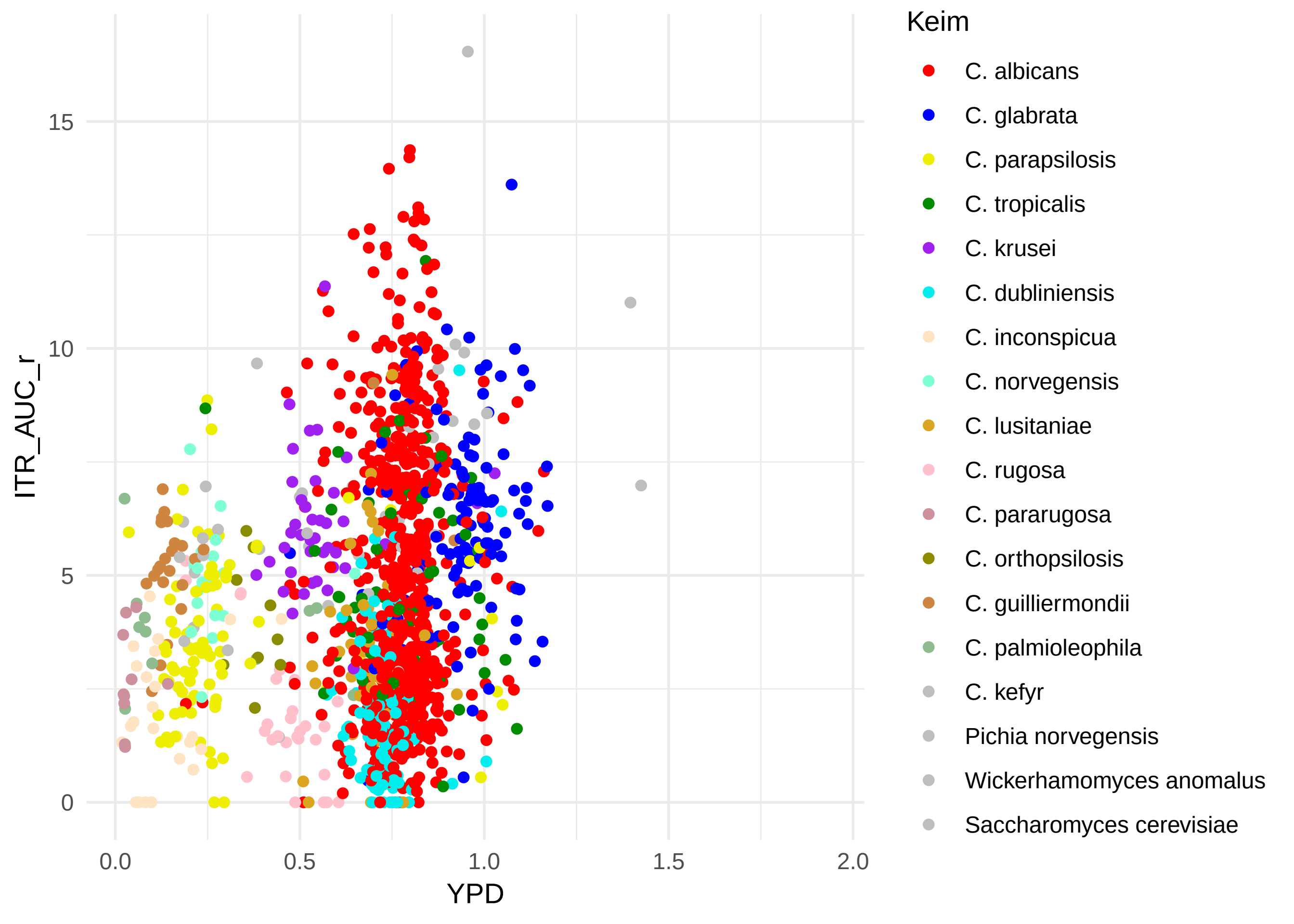

Keim
C. albicans
C. glabrata
C. parapsilosis
C. tropicalis
C. krusei
C. dubliniensis
C. inconspicua
C. norvegensis
C. lusitaniae
C. rugosa
C. pararugosa
C. orthopsilosis
C. guilliermondii
C. palmioleophila
C. kefyr
Pichia norvegensis
Wickerhamomyces anomalus
Saccharomyces cerevisiae
ITR_AUC_r
0
5
10
15
YPD
0.0
0.5
1.0
1.5
2.0

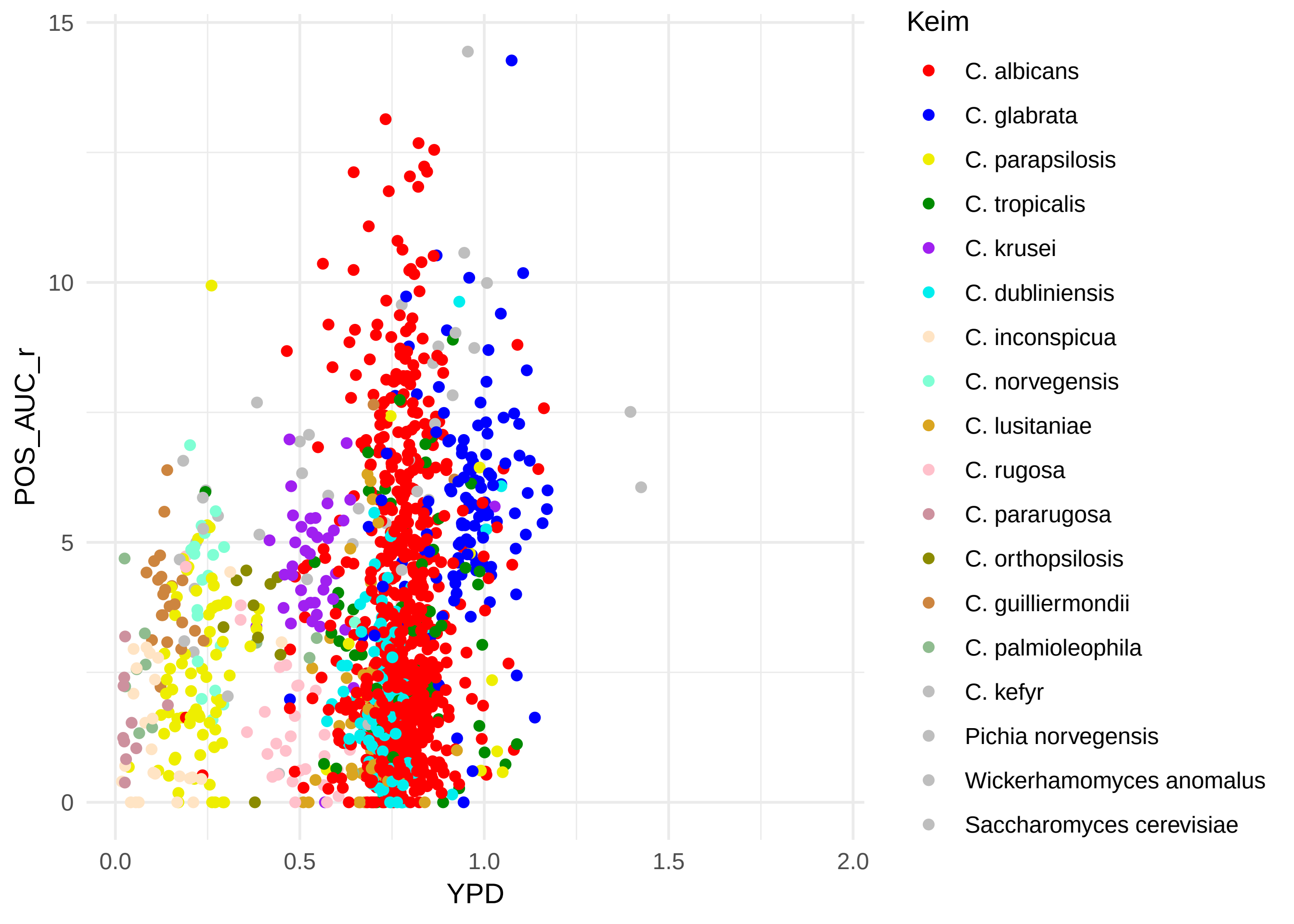

Keim
C. albicans
C. glabrata
C. parapsilosis
C. tropicalis
C. krusei
C. dubliniensis
C. inconspicua
C. norvegensis
C. lusitaniae
C. rugosa
C. pararugosa
C. orthopsilosis
C. guilliermondii
C. palmioleophila
C. kefyr
Pichia norvegensis
Wickerhamomyces anomalus
Saccharomyces cerevisiae
POS_AUC_r
YPD
0
5
10
15
0.0
0.5
1.0
1.5
2.0

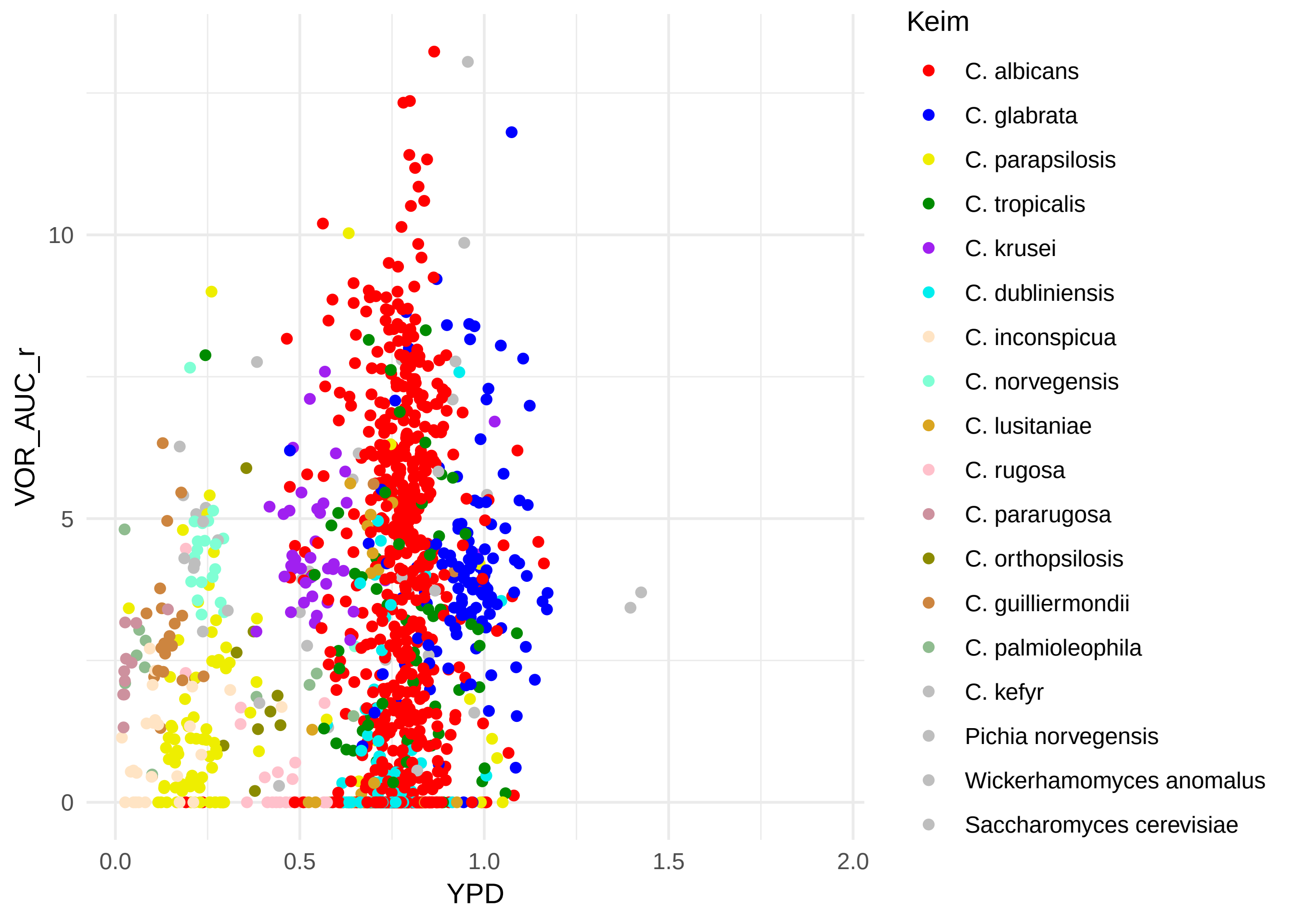
Keim
C. albicans
C. glabrata
C. parapsilosis
C. tropicalis
C. krusei
C. dubliniensis
C. inconspicua
C. norvegensis
C. lusitaniae
C. rugosa
C. pararugosa
C. orthopsilosis
C. guilliermondii
C. palmioleophila
C. kefyr
Pichia norvegensis
Wickerhamomyces anomalus
Saccharomyces cerevisiae
VOR_AUC_r
10
5
0
0.0
0.5
1.0
1.5
2.0
YPD

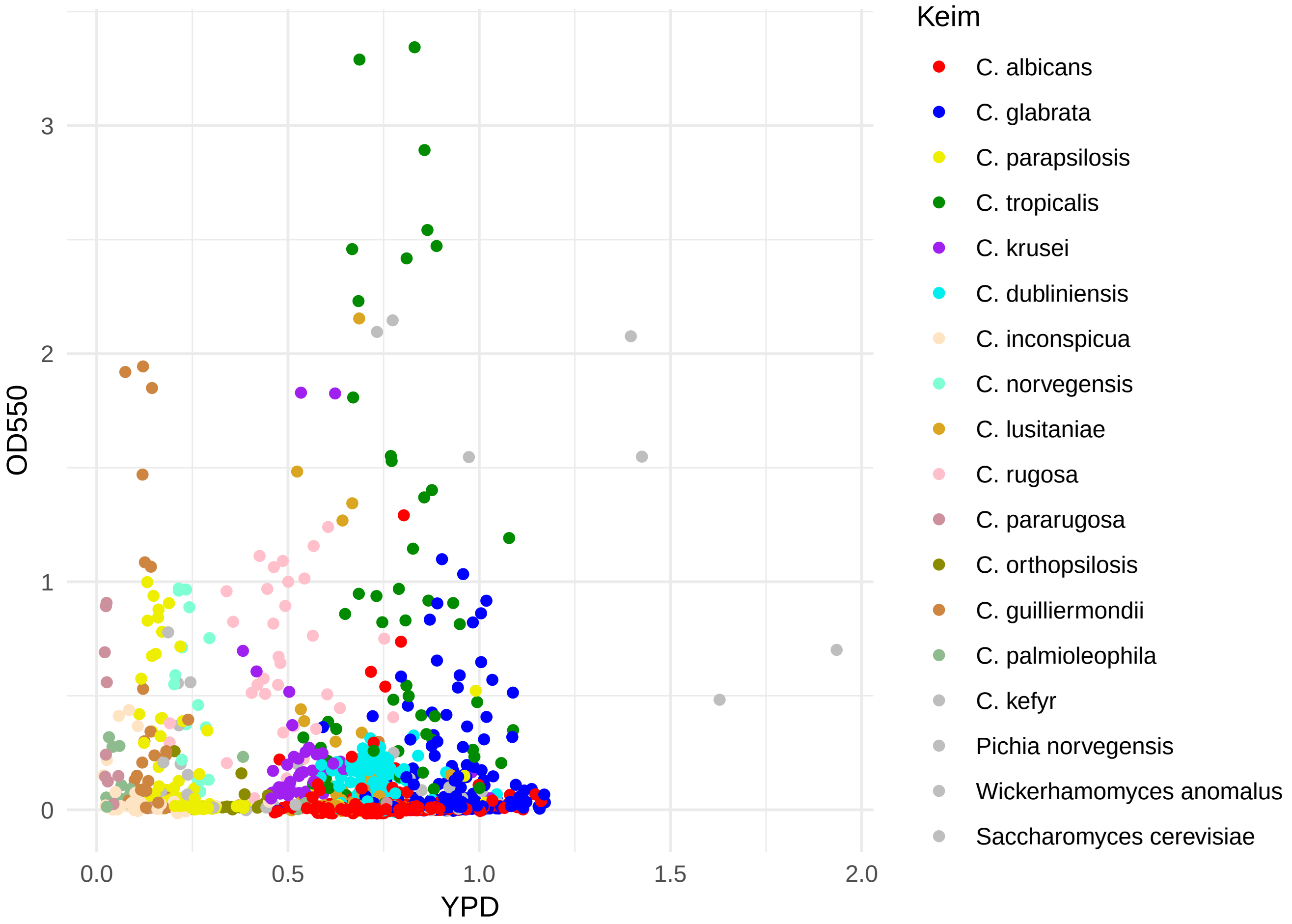
Keim
C. albicans
C. glabrata
C. parapsilosis
C. tropicalis
C. krusei
C. dubliniensis
C. inconspicua
C. norvegensis
C. lusitaniae
C. rugosa
C. pararugosa
C. orthopsilosis
C. guilliermondii
C. palmioleophila
C. kefyr
Pichia norvegensis
Wickerhamomyces anomalus
Saccharomyces cerevisiae
OD550
YPD
0
1
2
3
0.0
0.5
1.0
1.5
2.0

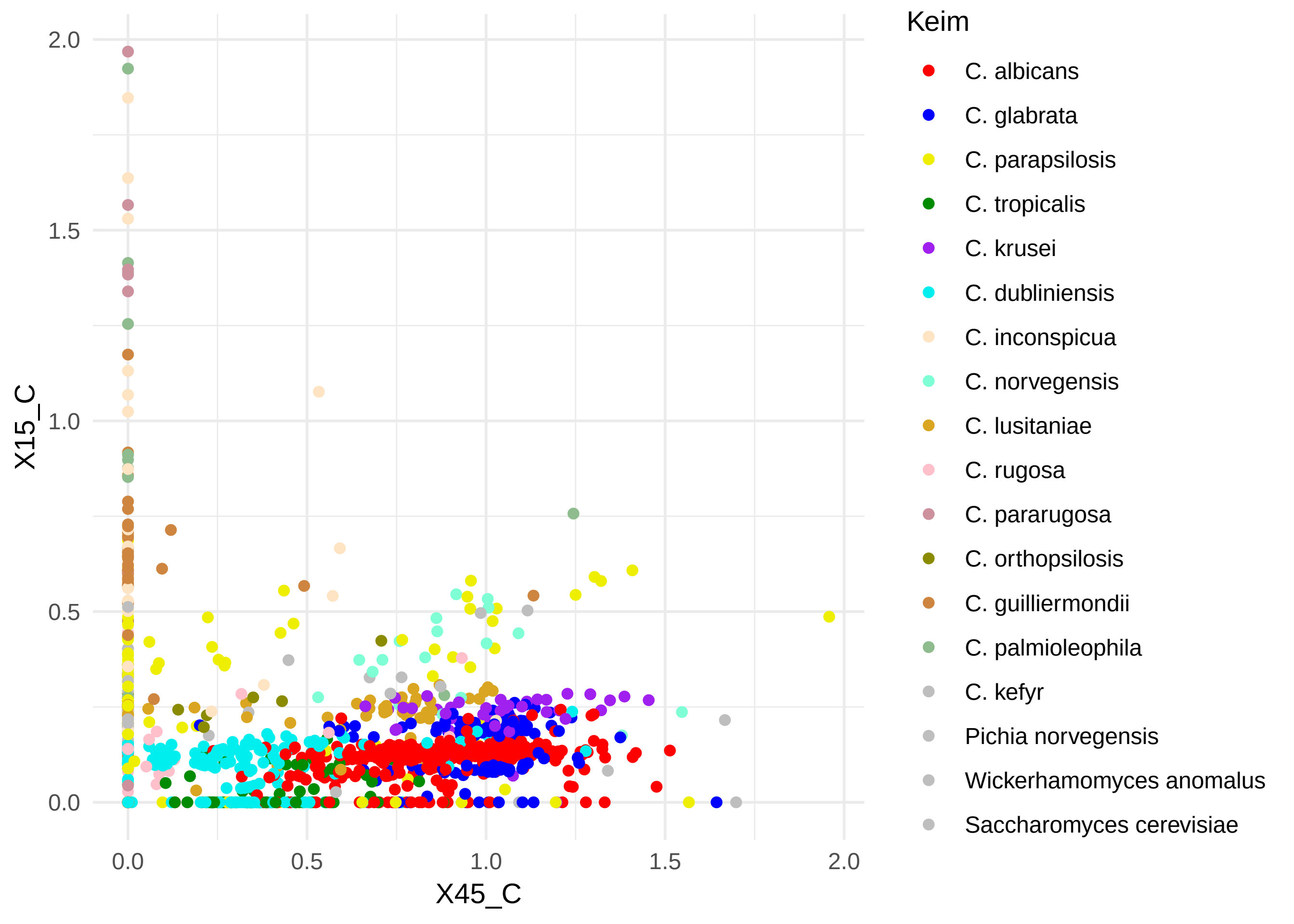
Keim
C. albicans
C. glabrata
C. parapsilosis
C. tropicalis
C. krusei
C. dubliniensis
C. inconspicua
C. norvegensis
C. lusitaniae
C. rugosa
C. pararugosa
C. orthopsilosis
C. guilliermondii
C. palmioleophila
C. kefyr
Pichia norvegensis
Wickerhamomyces anomalus
Saccharomyces cerevisiae
X15_C
X45_C
0.0
0.5
1.0
1.5
2.0

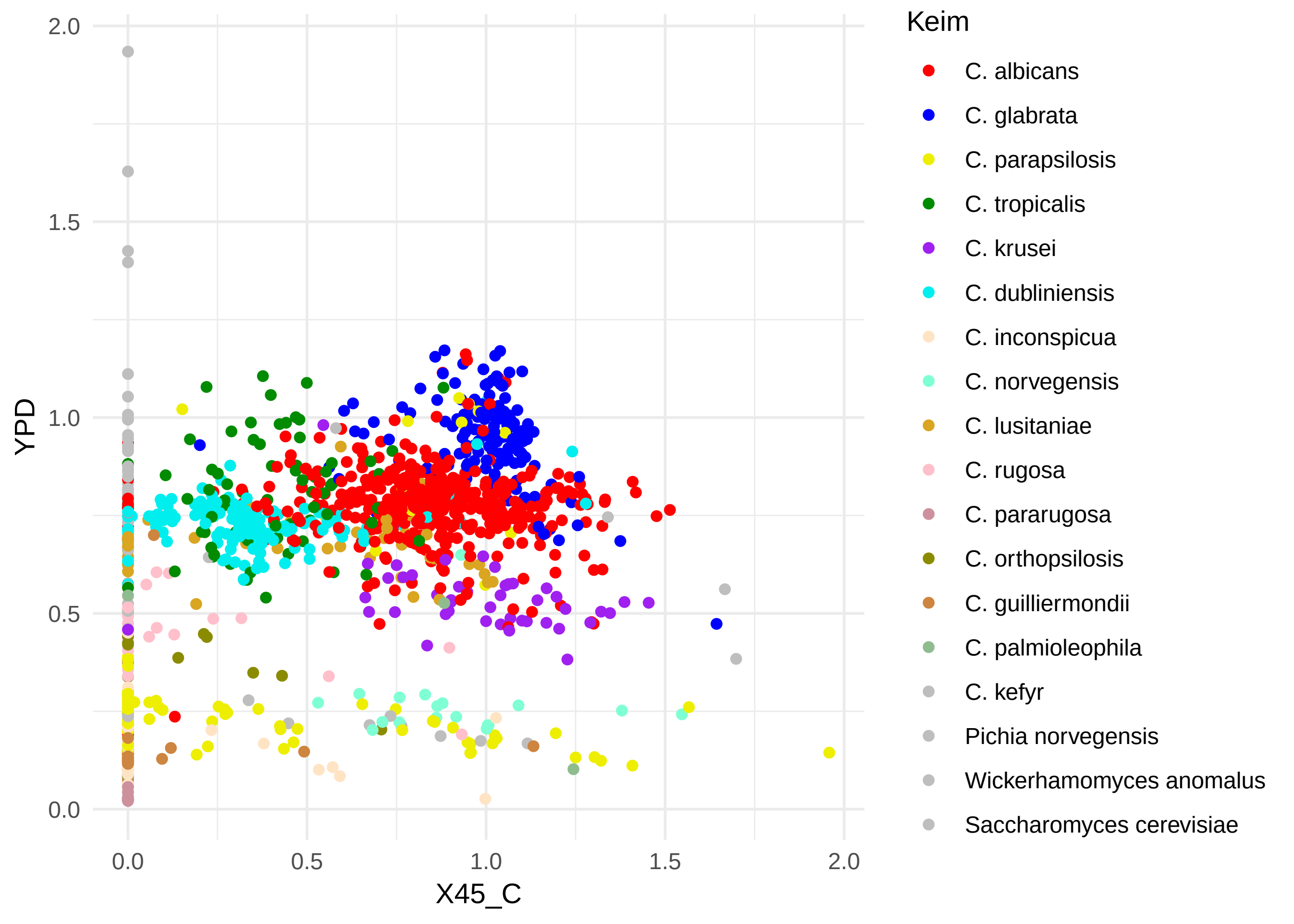
Keim
C. albicans
C. glabrata
C. parapsilosis
C. tropicalis
C. krusei
C. dubliniensis
C. inconspicua
C. norvegensis
C. lusitaniae
C. rugosa
C. pararugosa
C. orthopsilosis
C. guilliermondii
C. palmioleophila
C. kefyr
Pichia norvegensis
Wickerhamomyces anomalus
Saccharomyces cerevisiae
YPD
X45_C
0.0
0.5
1.0
1.5
2.0

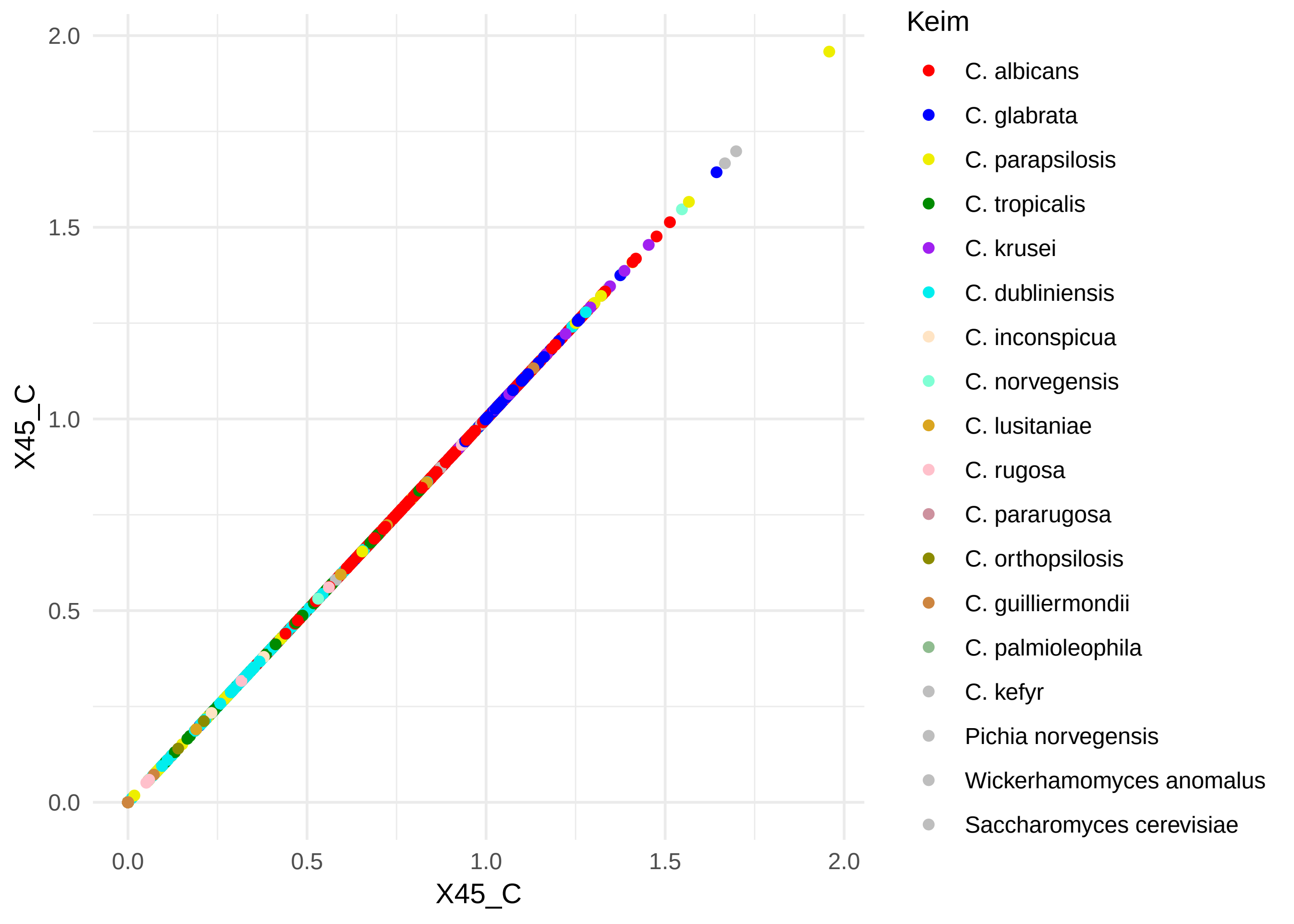
Keim
C. albicans
C. glabrata
C. parapsilosis
C. tropicalis
C. krusei
C. dubliniensis
C. inconspicua
C. norvegensis
C. lusitaniae
C. rugosa
C. pararugosa
C. orthopsilosis
C. guilliermondii
C. palmioleophila
C. kefyr
Pichia norvegensis
Wickerhamomyces anomalus
Saccharomyces cerevisiae
X45_C
X45_C
0.0
0.5
1.0
1.5
2.0

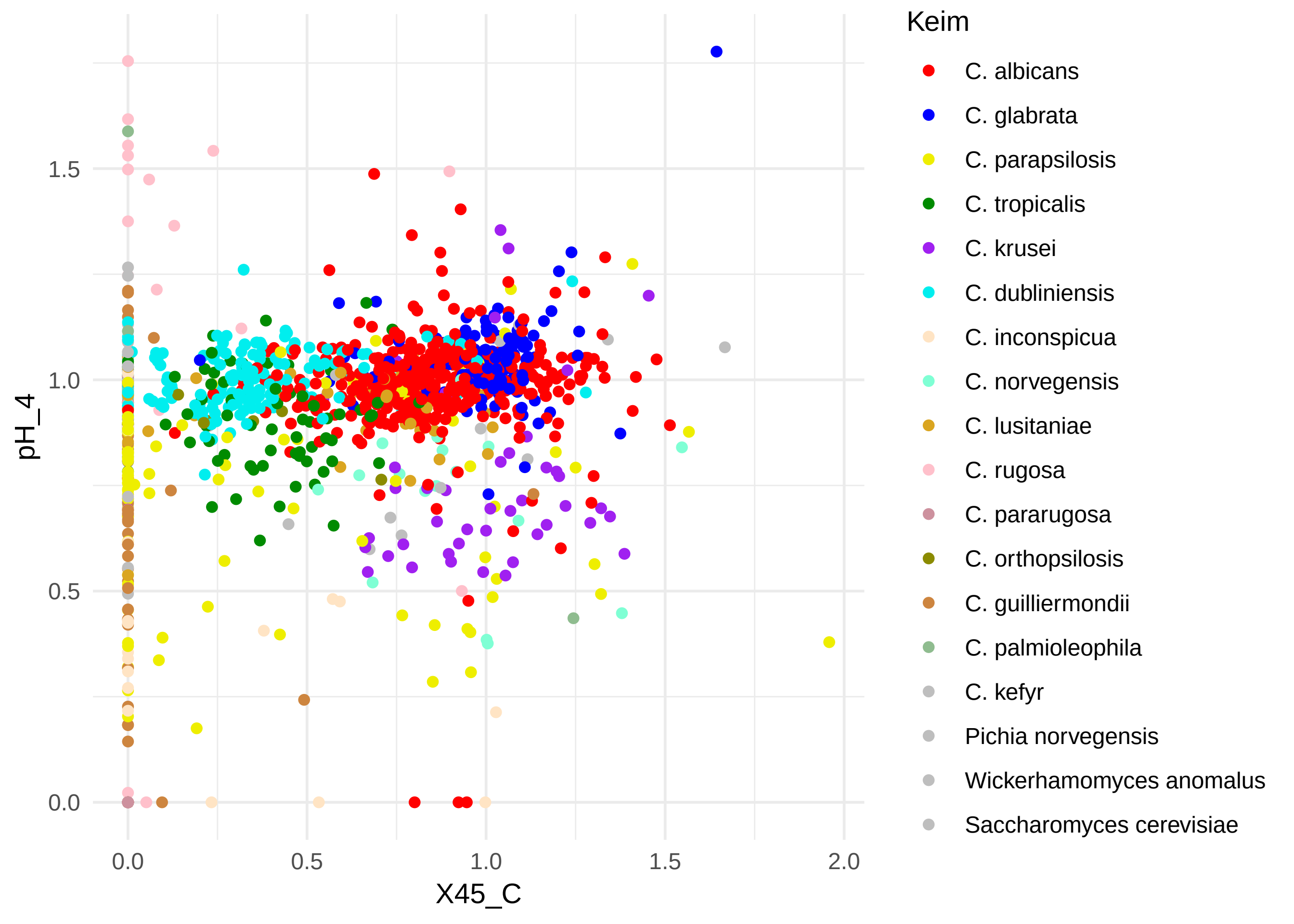

Keim
C. albicans
C. glabrata
C. parapsilosis
C. tropicalis
C. krusei
C. dubliniensis
C. inconspicua
C. norvegensis
C. lusitaniae
C. rugosa
C. pararugosa
C. orthopsilosis
C. guilliermondii
C. palmioleophila
C. kefyr
Pichia norvegensis
Wickerhamomyces anomalus
Saccharomyces cerevisiae
pH_4
X45_C
0.0
0.5
1.0
1.5
2.0

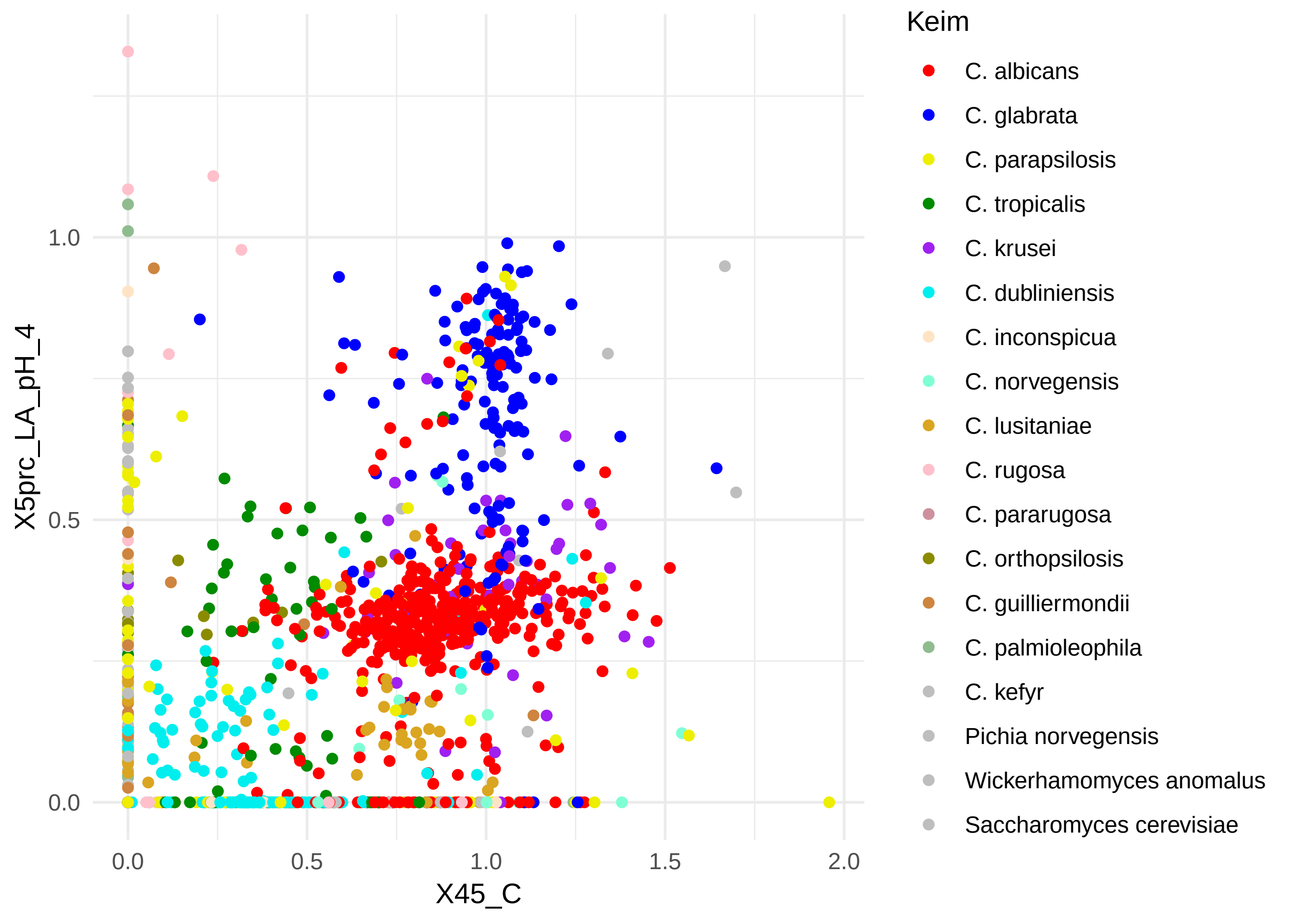

Keim
C. albicans
C. glabrata
C. parapsilosis
C. tropicalis
C. krusei
C. dubliniensis
C. inconspicua
C. norvegensis
C. lusitaniae
C. rugosa
C. pararugosa
C. orthopsilosis
C. guilliermondii
C. palmioleophila
C. kefyr
Pichia norvegensis
Wickerhamomyces anomalus
Saccharomyces cerevisiae
X5prc_LA_pH_4
1.0
0.5
0.0
0.0
0.5
1.0
1.5
2.0
X45_C

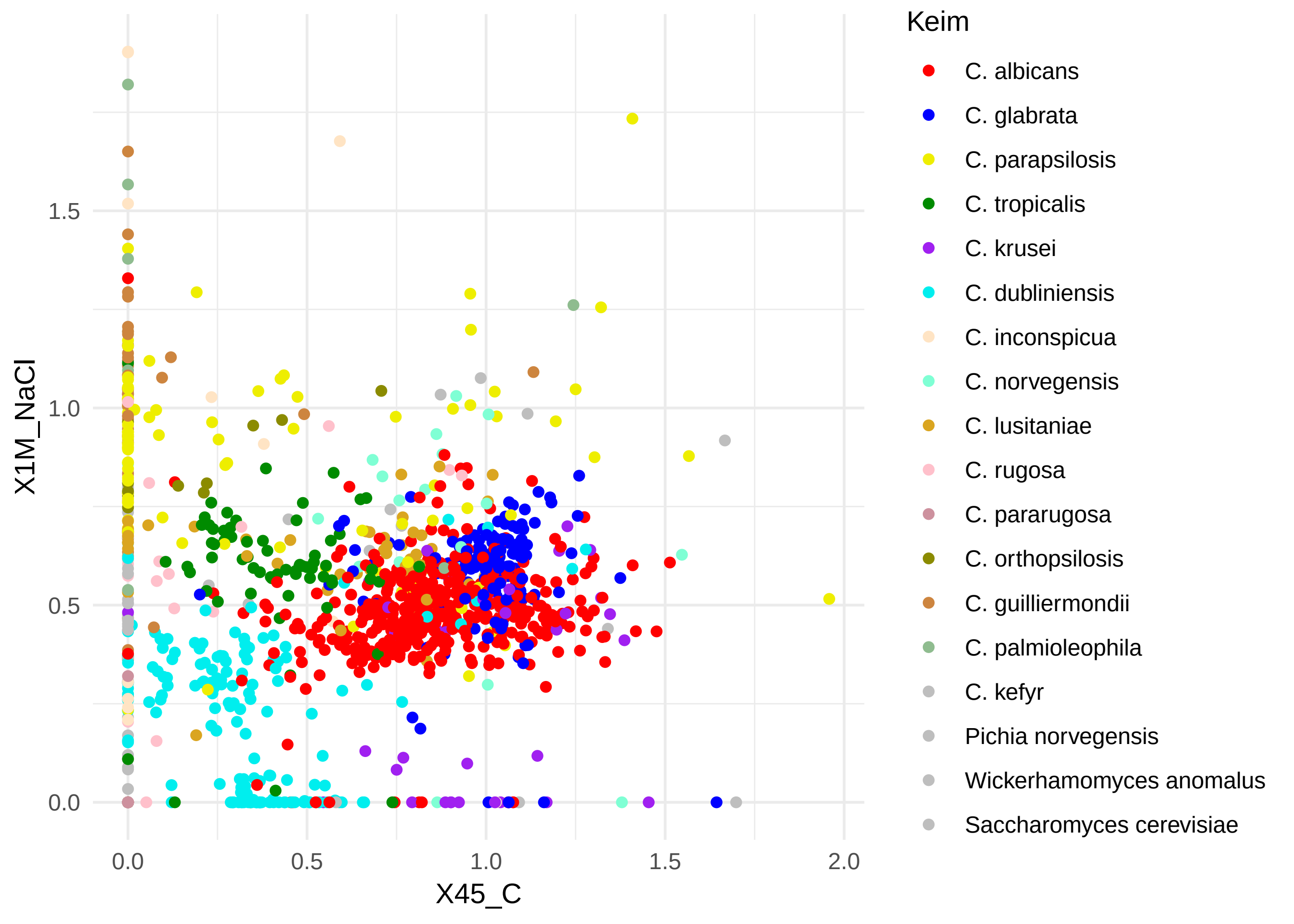

Keim
C. albicans
C. glabrata
C. parapsilosis
C. tropicalis
C. krusei
C. dubliniensis
C. inconspicua
C. norvegensis
C. lusitaniae
C. rugosa
C. pararugosa
C. orthopsilosis
C. guilliermondii
C. palmioleophila
C. kefyr
Pichia norvegensis
Wickerhamomyces anomalus
Saccharomyces cerevisiae
X1M_NaCl
X45_C
0.0
0.5
1.0
1.5
2.0

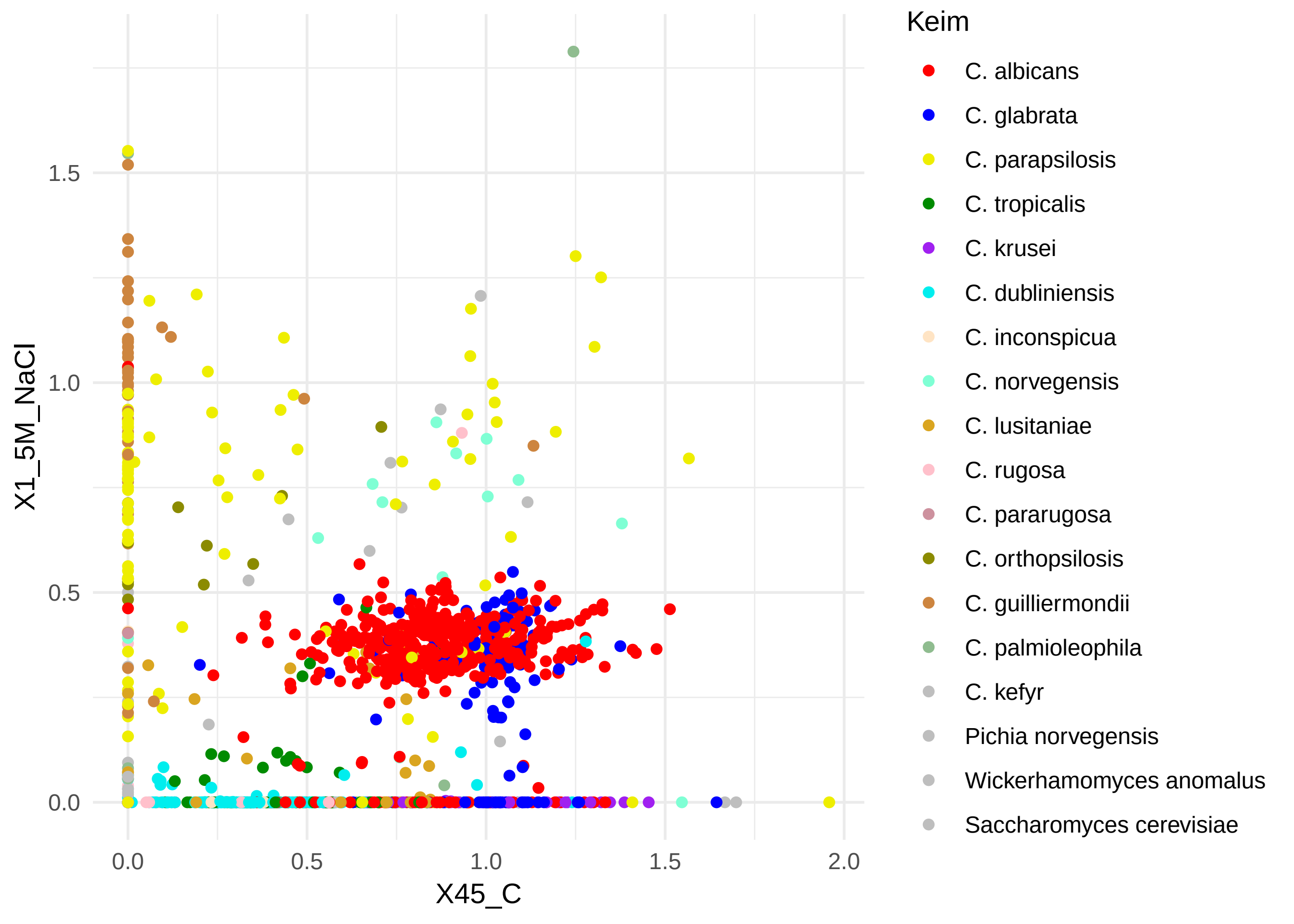

Keim
C. albicans
C. glabrata
C. parapsilosis
C. tropicalis
C. krusei
C. dubliniensis
C. inconspicua
C. norvegensis
C. lusitaniae
C. rugosa
C. pararugosa
C. orthopsilosis
C. guilliermondii
C. palmioleophila
C. kefyr
Pichia norvegensis
Wickerhamomyces anomalus
Saccharomyces cerevisiae
X1_5M_NaCl
1.5
1.0
0.5
0.0
X45_C
0.0
0.5
1.0
1.5
2.0

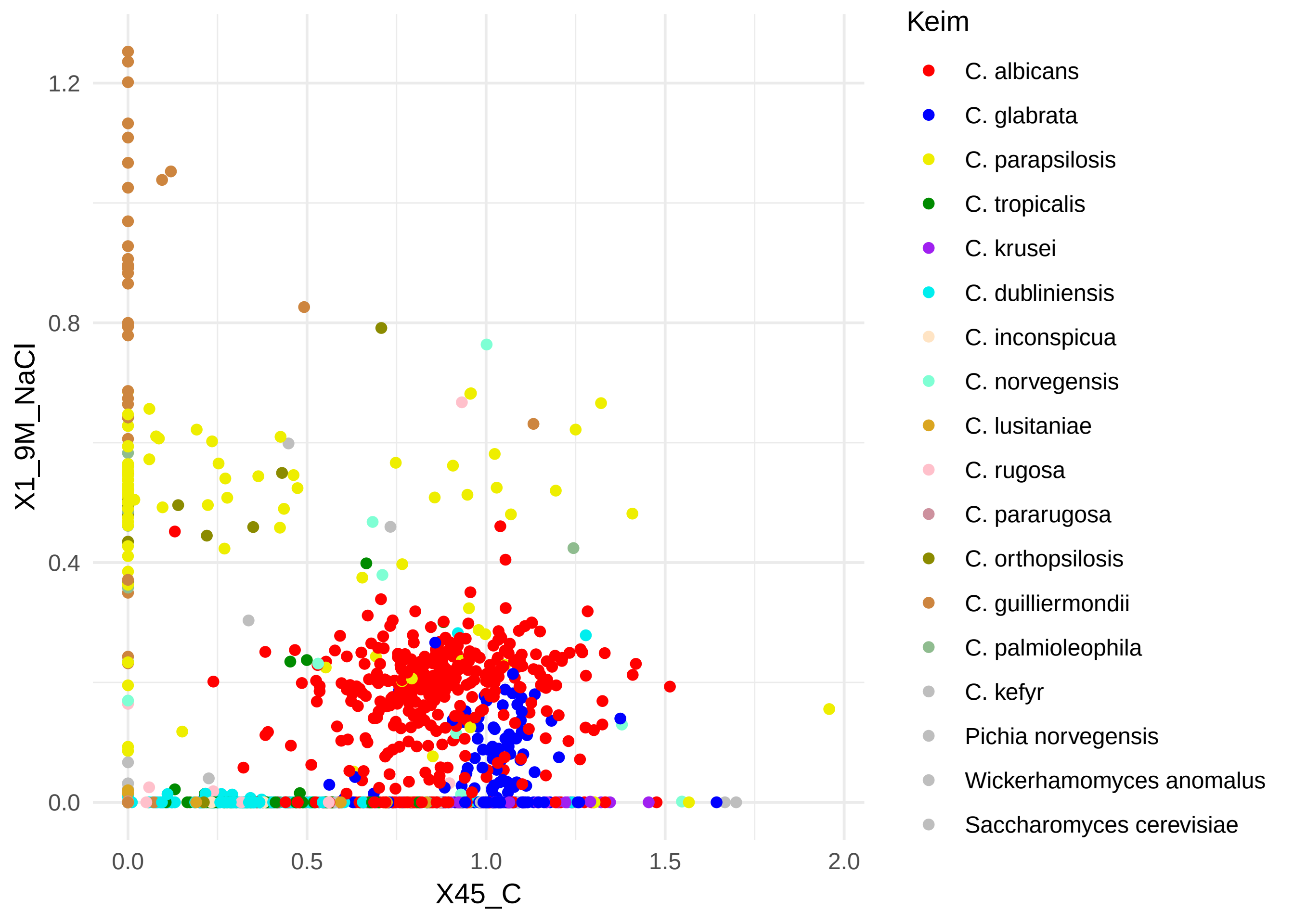

Keim
C. albicans
C. glabrata
C. parapsilosis
C. tropicalis
C. krusei
C. dubliniensis
C. inconspicua
C. norvegensis
C. lusitaniae
C. rugosa
C. pararugosa
C. orthopsilosis
C. guilliermondii
C. palmioleophila
C. kefyr
Pichia norvegensis
Wickerhamomyces anomalus
Saccharomyces cerevisiae
X1_9M_NaCl
1.2
0.8
0.4
0.0
0.0
0.5
1.0
1.5
2.0
X45_C

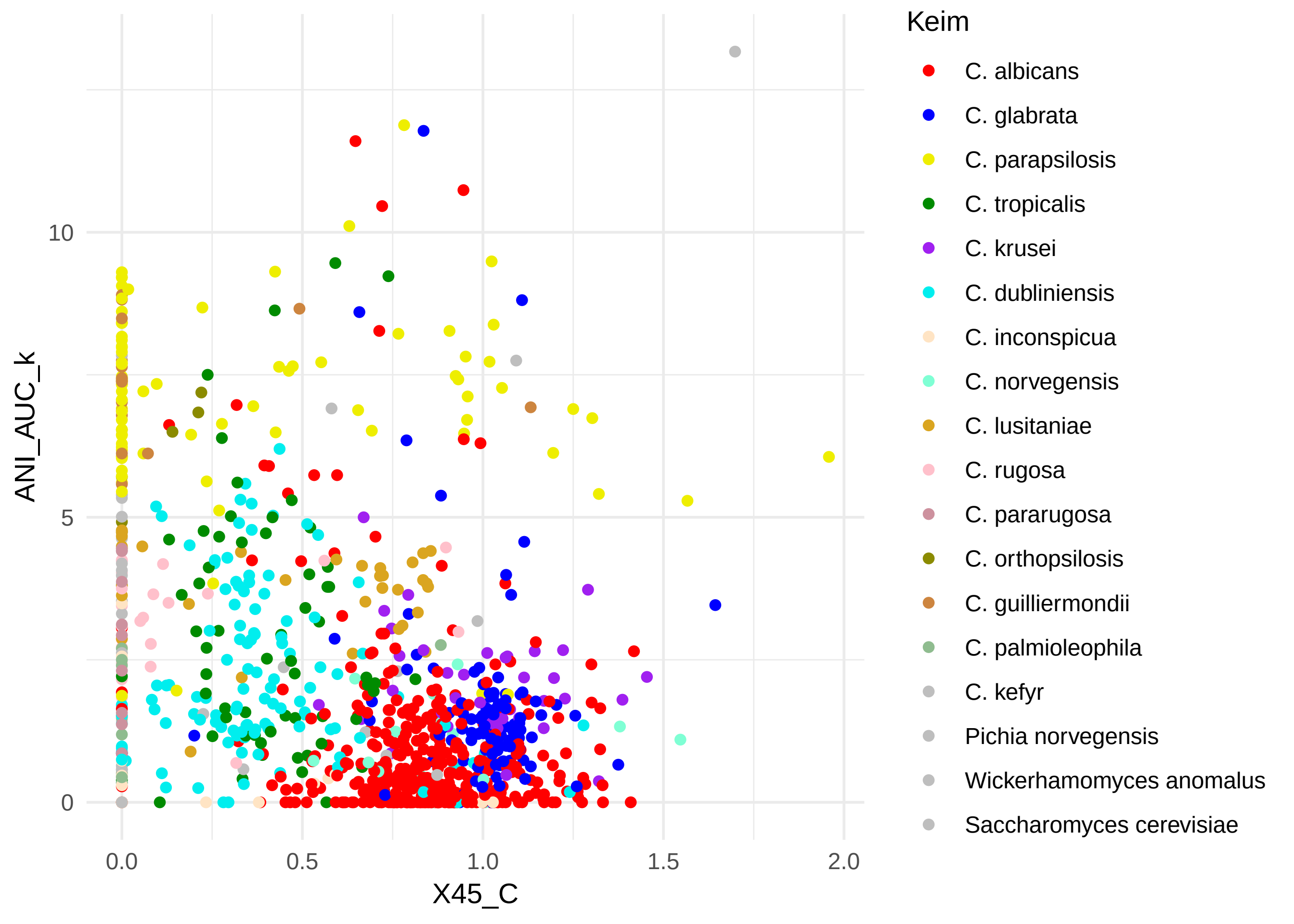
ANI_AUC_k
X45_C
0
5
10
0.0
0.5
1.0
1.5
2.0
Keim
C. albicans
C. glabrata
C. parapsilosis
C. tropicalis
C. krusei
C. dubliniensis
C. inconspicua
C. norvegensis
C. lusitaniae
C. rugosa
C. pararugosa
C. orthopsilosis
C. guilliermondii
C. palmioleophila
C. kefyr
Pichia norvegensis
Wickerhamomyces anomalus
Saccharomyces cerevisiae

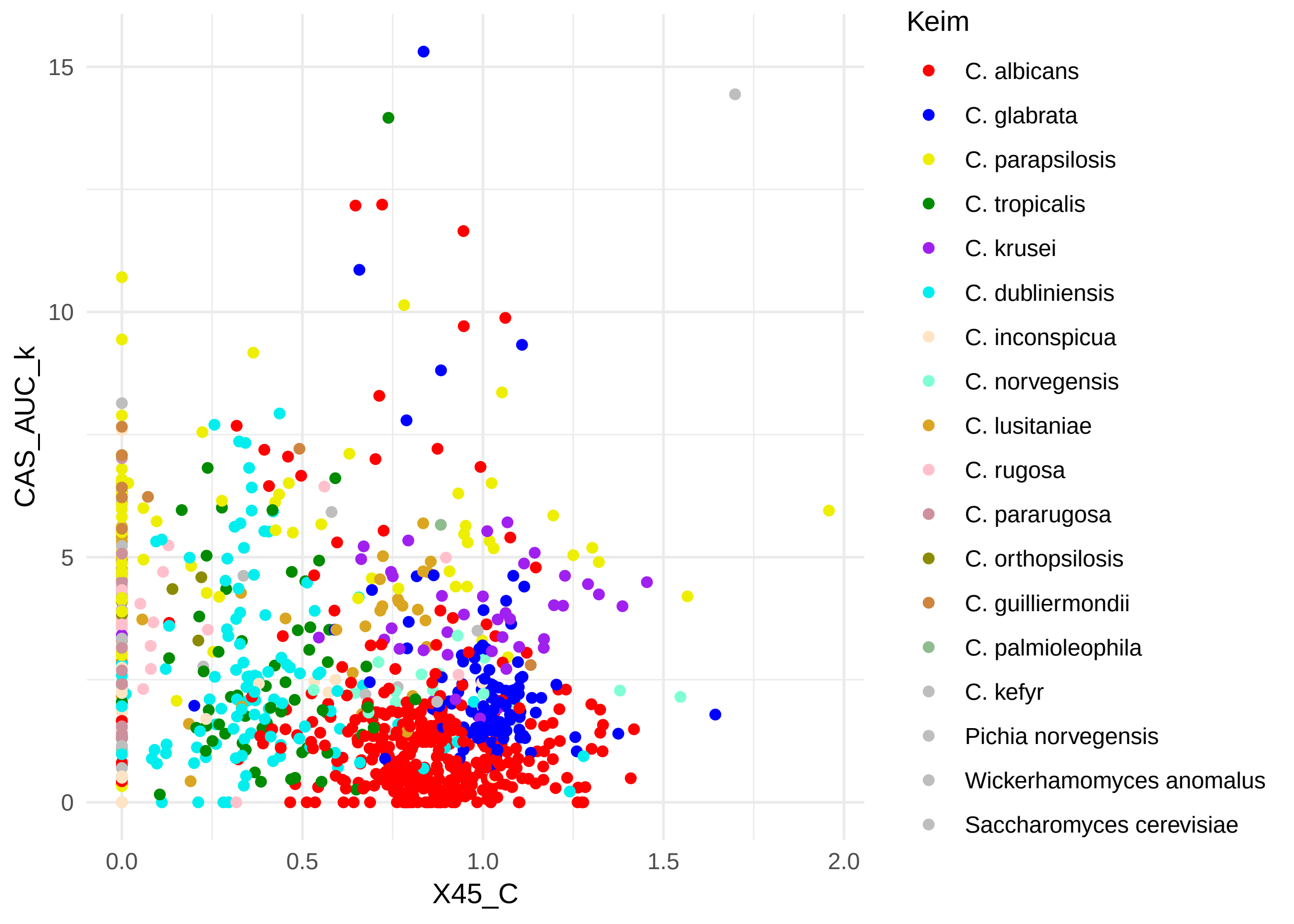
Keim
C. albicans
C. glabrata
C. parapsilosis
C. tropicalis
C. krusei
C. dubliniensis
C. inconspicua
C. norvegensis
C. lusitaniae
C. rugosa
C. pararugosa
C. orthopsilosis
C. guilliermondii
C. palmioleophila
C. kefyr
Pichia norvegensis
Wickerhamomyces anomalus
Saccharomyces cerevisiae
CAS_AUC_k
X45_C
0
5
10
15
0.0
0.5
1.0
1.5
2.0

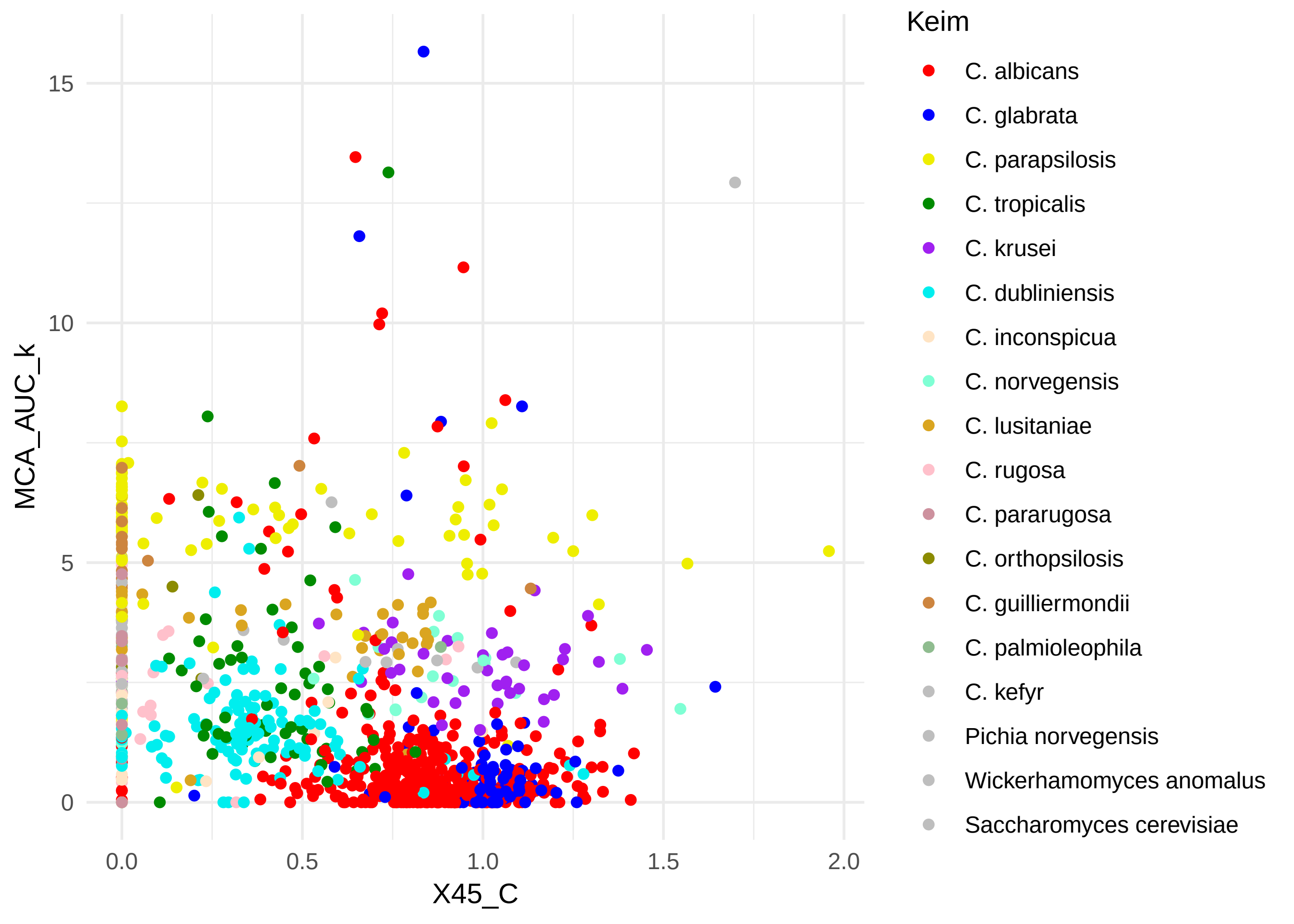

Keim
C. albicans
C. glabrata
C. parapsilosis
C. tropicalis
C. krusei
C. dubliniensis
C. inconspicua
C. norvegensis
C. lusitaniae
C. rugosa
C. pararugosa
C. orthopsilosis
C. guilliermondii
C. palmioleophila
C. kefyr
Pichia norvegensis
Wickerhamomyces anomalus
Saccharomyces cerevisiae
MCA_AUC_k
X45_C
0
5
10
15
0.0
0.5
1.0
1.5
2.0

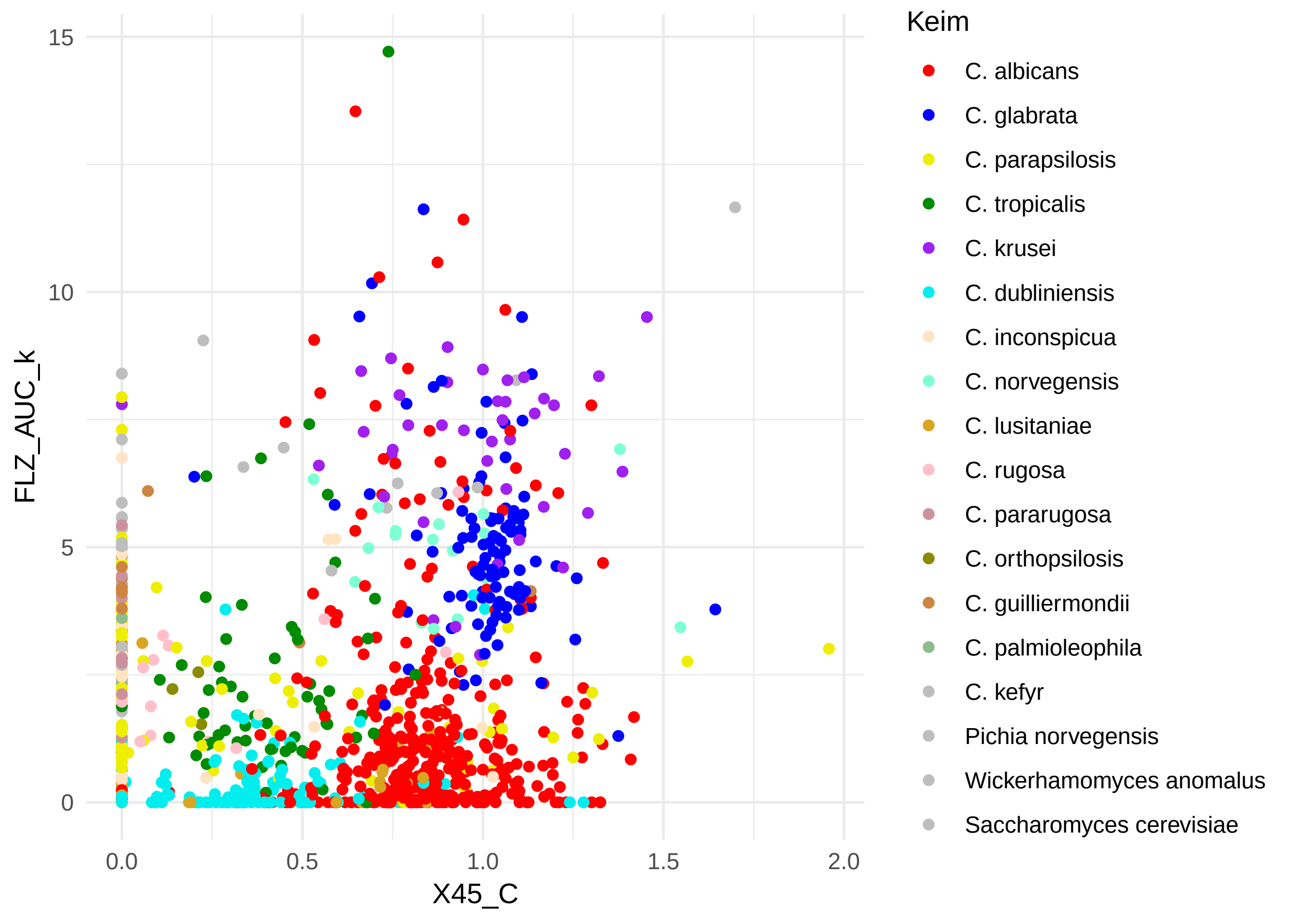
Keim
C. albicans
C. glabrata
C. parapsilosis
C. tropicalis
C. krusei
C. dubliniensis
C. inconspicua
C. norvegensis
C. lusitaniae
C. rugosa
C. pararugosa
C. orthopsilosis
C. guilliermondii
C. palmioleophila
C. kefyr
Pichia norvegensis
Wickerhamomyces anomalus
Saccharomyces cerevisiae
FLZ_AUC_k
X45_C
0
5
10
15
0.0
0.5
1.0
1.5
2.0

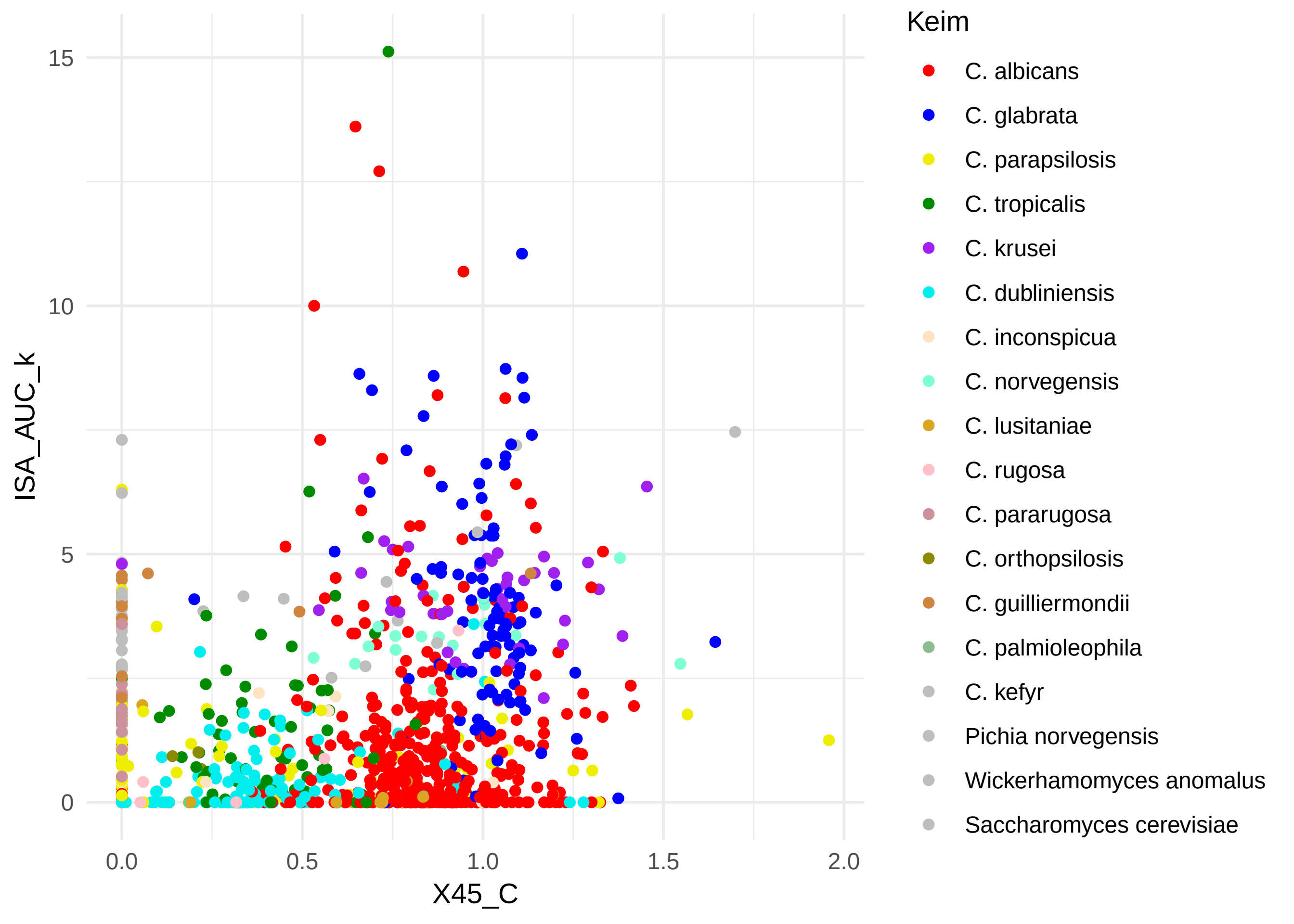

Keim
C. albicans
C. glabrata
C. parapsilosis
C. tropicalis
C. krusei
C. dubliniensis
C. inconspicua
C. norvegensis
C. lusitaniae
C. rugosa
C. pararugosa
C. orthopsilosis
C. guilliermondii
C. palmioleophila
C. kefyr
Pichia norvegensis
Wickerhamomyces anomalus
Saccharomyces cerevisiae
ISA_AUC_k
X45_C
15
10
5
0
0.0
0.5
1.0
1.5
2.0

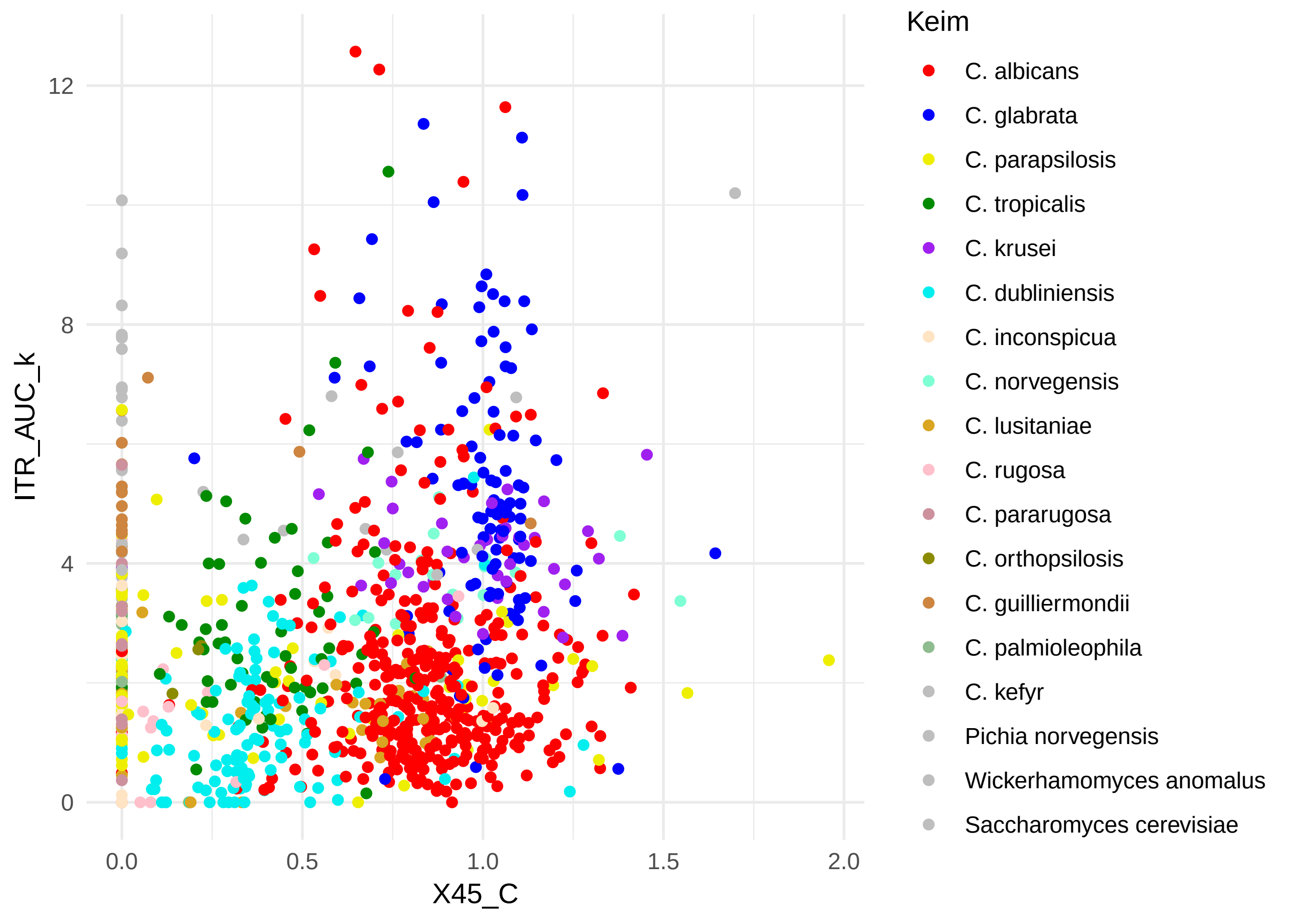
Keim
C. albicans
C. glabrata
C. parapsilosis
C. tropicalis
C. krusei
C. dubliniensis
C. inconspicua
C. norvegensis
C. lusitaniae
C. rugosa
C. pararugosa
C. orthopsilosis
C. guilliermondii
C. palmioleophila
C. kefyr
Pichia norvegensis
Wickerhamomyces anomalus
Saccharomyces cerevisiae
ITR_AUC_k
X45_C
0
4
8
12
0.0
0.5
1.0
1.5
2.0

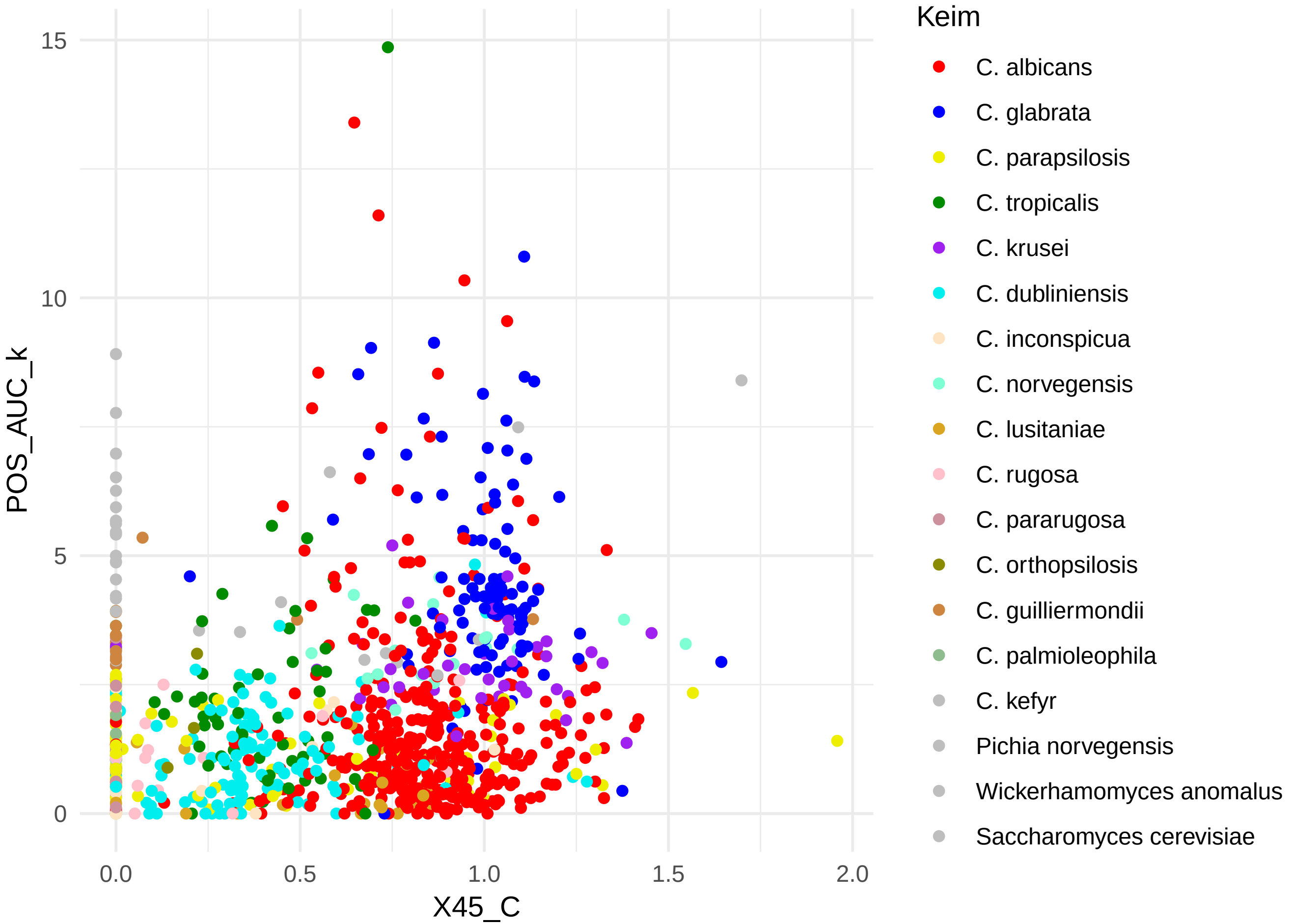

Keim
C. albicans
C. glabrata
C. parapsilosis
C. tropicalis
C. krusei
C. dubliniensis
C. inconspicua
C. norvegensis
C. lusitaniae
C. rugosa
C. pararugosa
C. orthopsilosis
C. guilliermondii
C. palmioleophila
C. kefyr
Pichia norvegensis
Wickerhamomyces anomalus
Saccharomyces cerevisiae
POS_AUC_k
15
10
5
0
0.0
0.5
1.0
1.5
2.0
X45_C

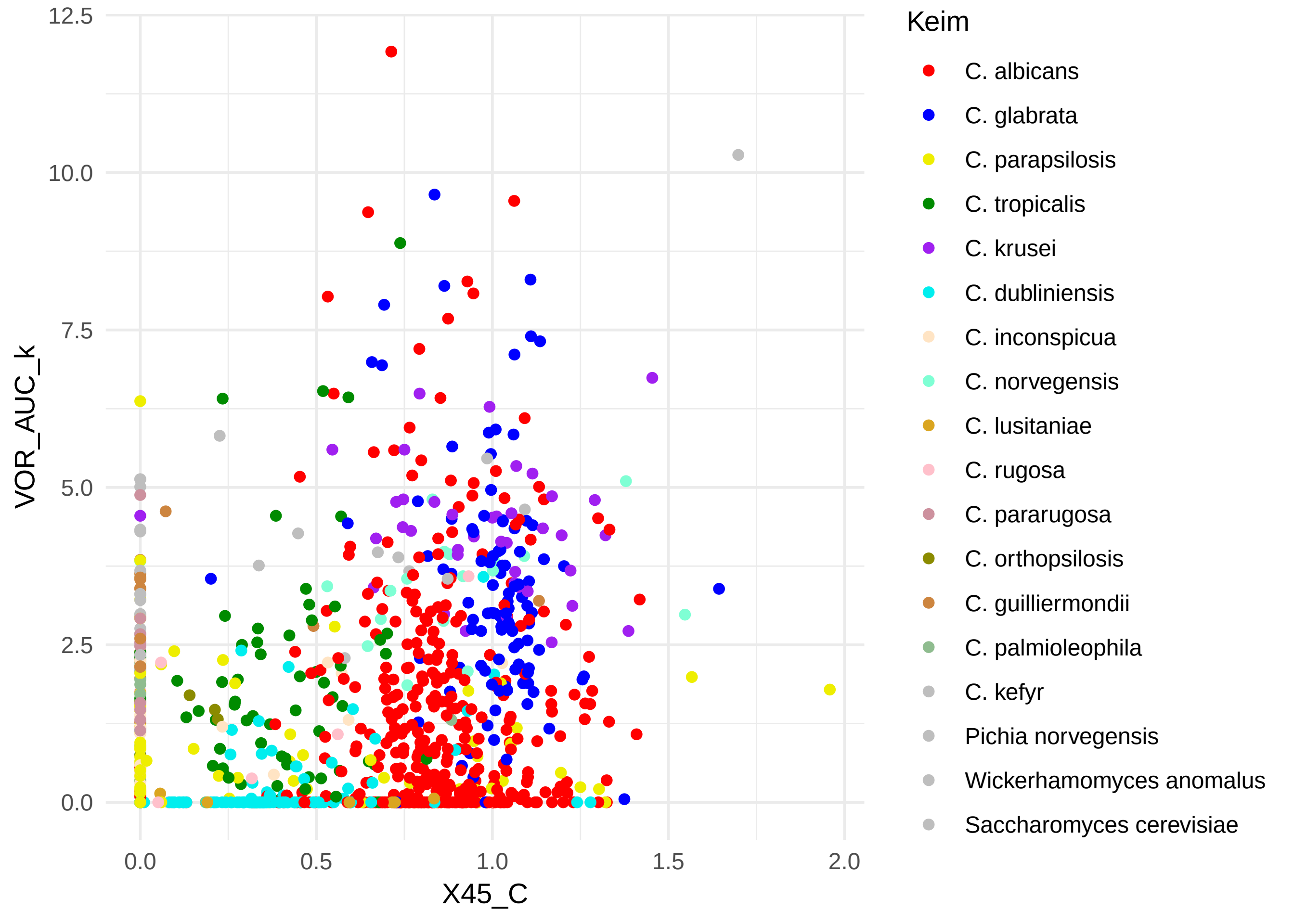

VOR_AUC_k
12.5
10.0
7.5
5.0
2.5
0.0
0.0
0.5
1.0
1.5
2.0
X45_C
Keim
C. albicans
C. glabrata
C. parapsilosis
C. tropicalis
C. krusei
C. dubliniensis
C. inconspicua
C. norvegensis
C. lusitaniae
C. rugosa
C. pararugosa
C. orthopsilosis
C. guilliermondii
C. palmioleophila
C. kefyr
Pichia norvegensis
Wickerhamomyces anomalus
Saccharomyces cerevisiae

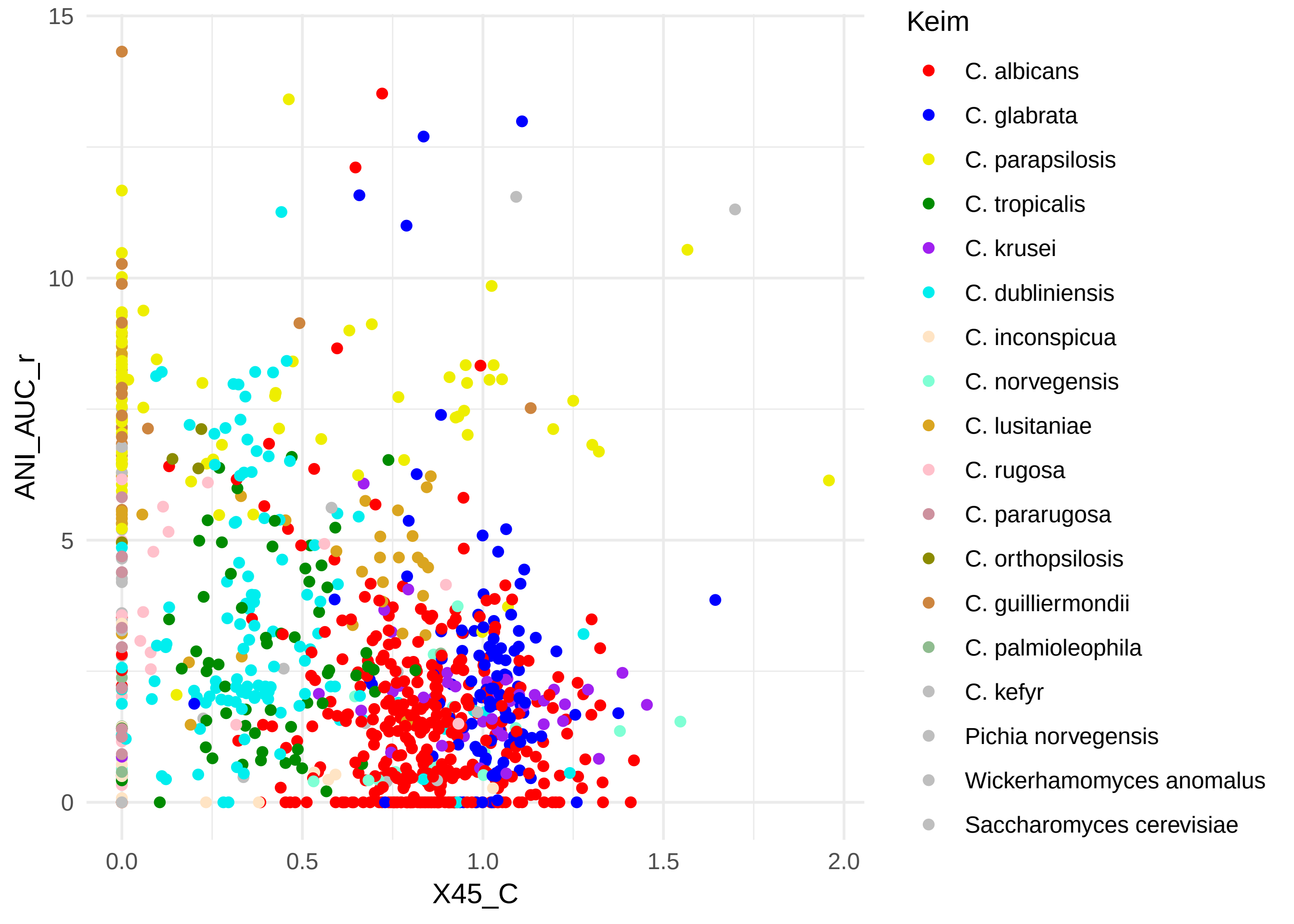

15
10
5
0
ANI_AUC_r
0.0
0.5
1.0
1.5
2.0
X45_C
Keim
C. albicans
C. glabrata
C. parapsilosis
C. tropicalis
C. krusei
C. dubliniensis
C. inconspicua
C. norvegensis
C. lusitaniae
C. rugosa
C. pararugosa
C. orthopsilosis
C. guilliermondii
C. palmioleophila
C. kefyr
Pichia norvegensis
Wickerhamomyces anomalus
Saccharomyces cerevisiae

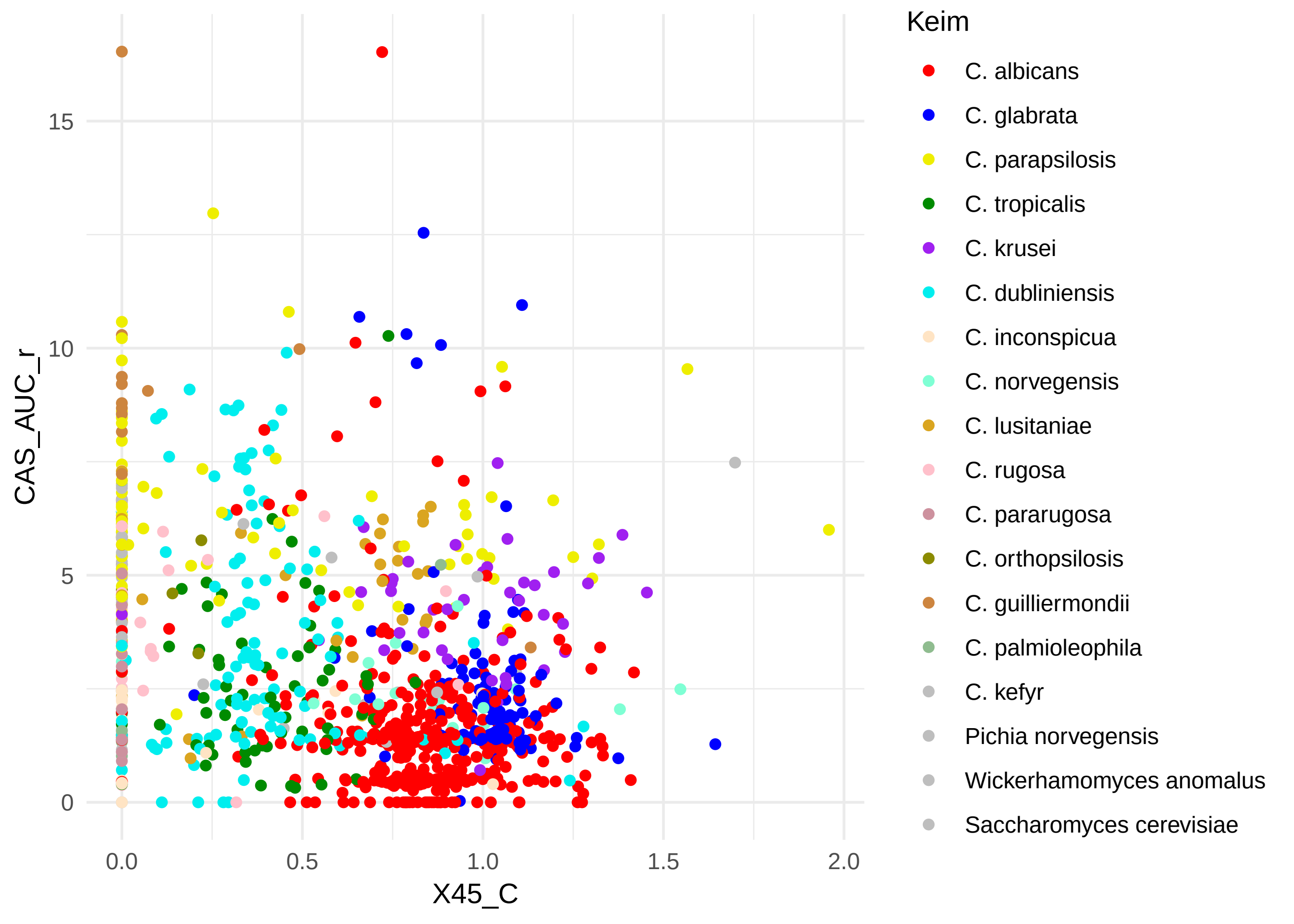

Keim
C. albicans
C. glabrata
C. parapsilosis
C. tropicalis
C. krusei
C. dubliniensis
C. inconspicua
C. norvegensis
C. lusitaniae
C. rugosa
C. pararugosa
C. orthopsilosis
C. guilliermondii
C. palmioleophila
C. kefyr
Pichia norvegensis
Wickerhamomyces anomalus
Saccharomyces cerevisiae
CAS_AUC_r
0
5
10
15
X45_C
0.0
0.5
1.0
1.5
2.0

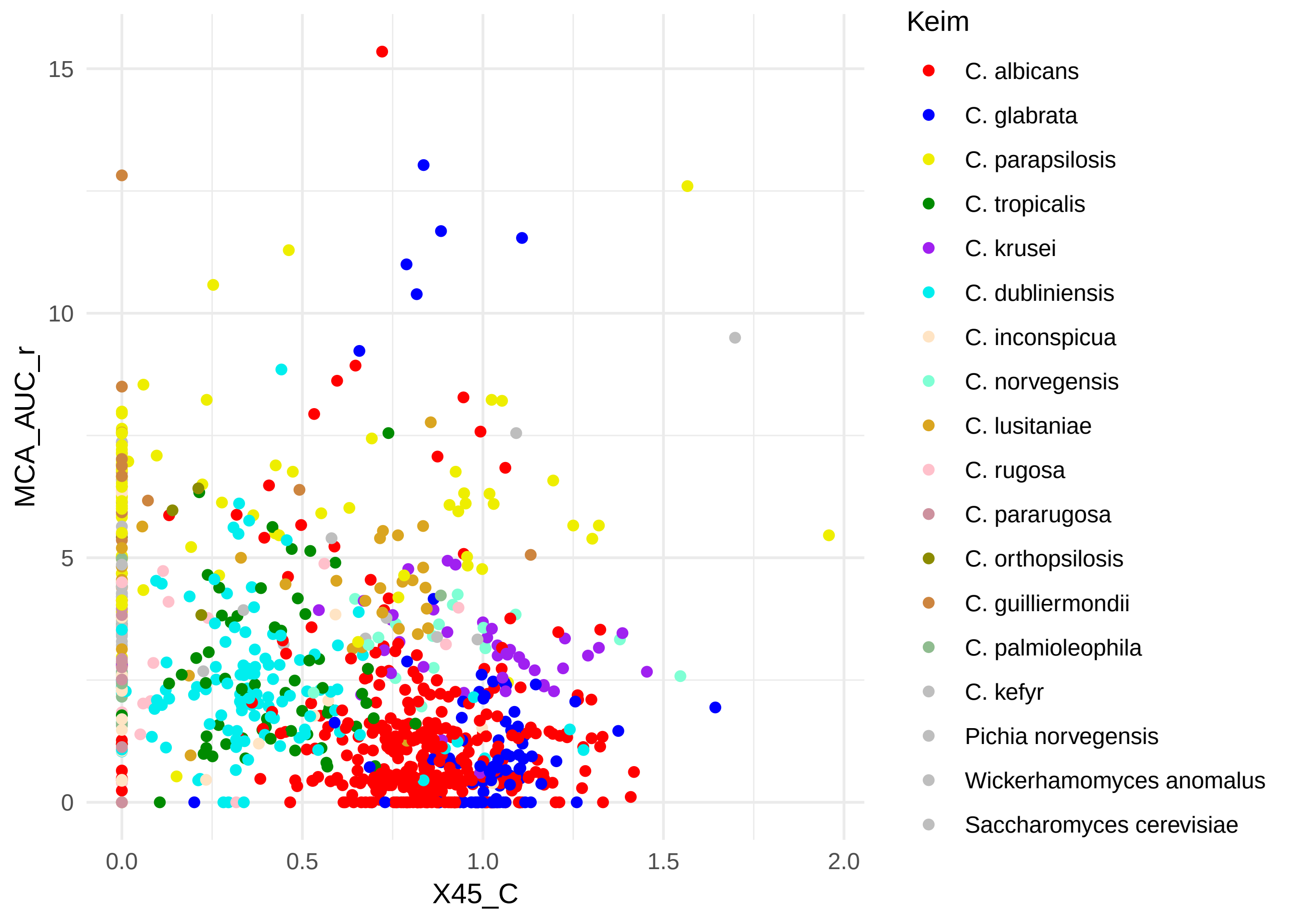

Keim
C. albicans
C. glabrata
C. parapsilosis
C. tropicalis
C. krusei
C. dubliniensis
C. inconspicua
C. norvegensis
C. lusitaniae
C. rugosa
C. pararugosa
C. orthopsilosis
C. guilliermondii
C. palmioleophila
C. kefyr
Pichia norvegensis
Wickerhamomyces anomalus
Saccharomyces cerevisiae
MCA_AUC_r
X45_C
0
5
10
15
0.0
0.5
1.0
1.5
2.0

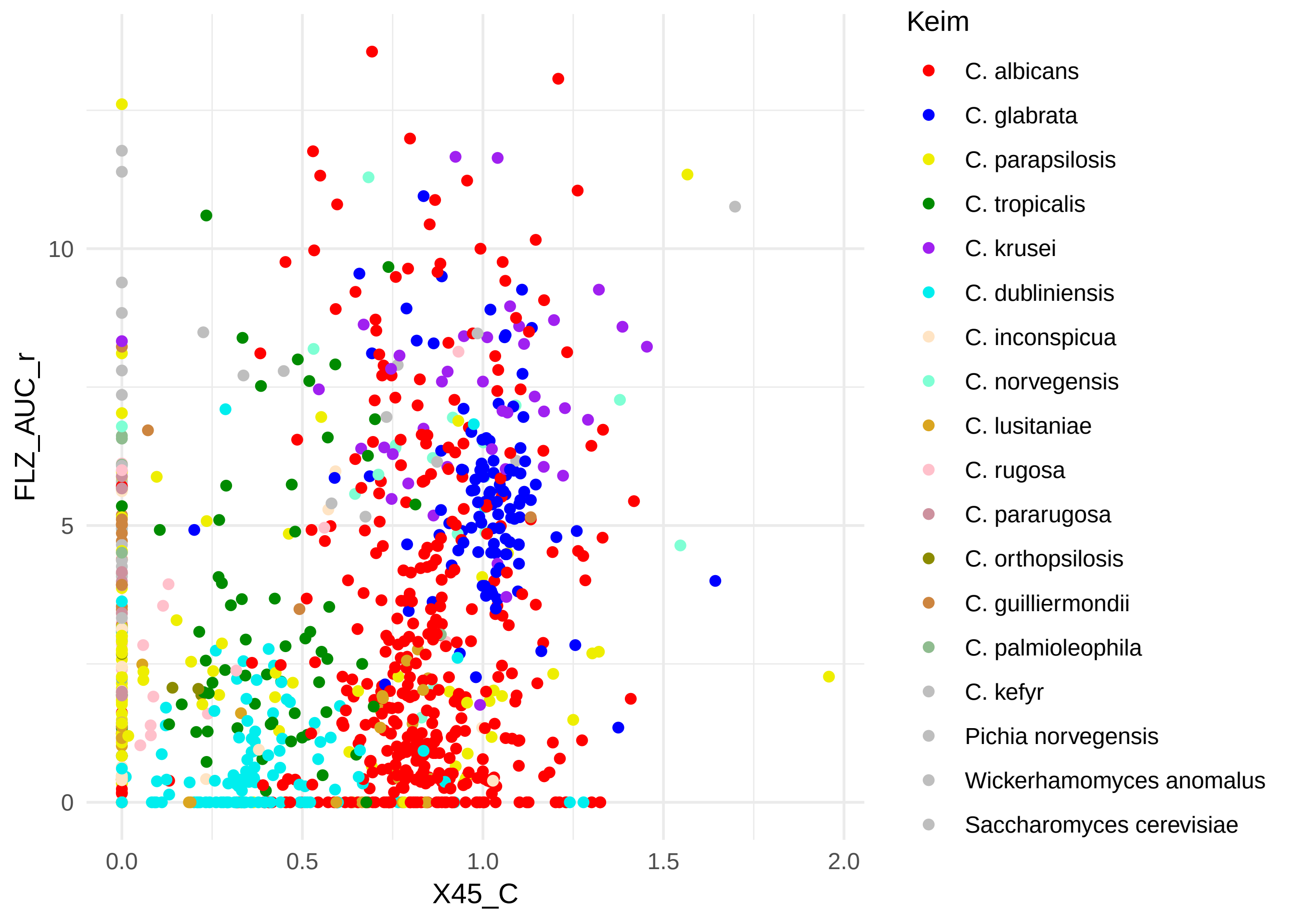

Keim
C. albicans
C. glabrata
C. parapsilosis
C. tropicalis
C. krusei
C. dubliniensis
C. inconspicua
C. norvegensis
C. lusitaniae
C. rugosa
C. pararugosa
C. orthopsilosis
C. guilliermondii
C. palmioleophila
C. kefyr
Pichia norvegensis
Wickerhamomyces anomalus
Saccharomyces cerevisiae
FLZ_AUC_r
10
5
0
0.0
0.5
1.0
1.5
2.0
X45_C

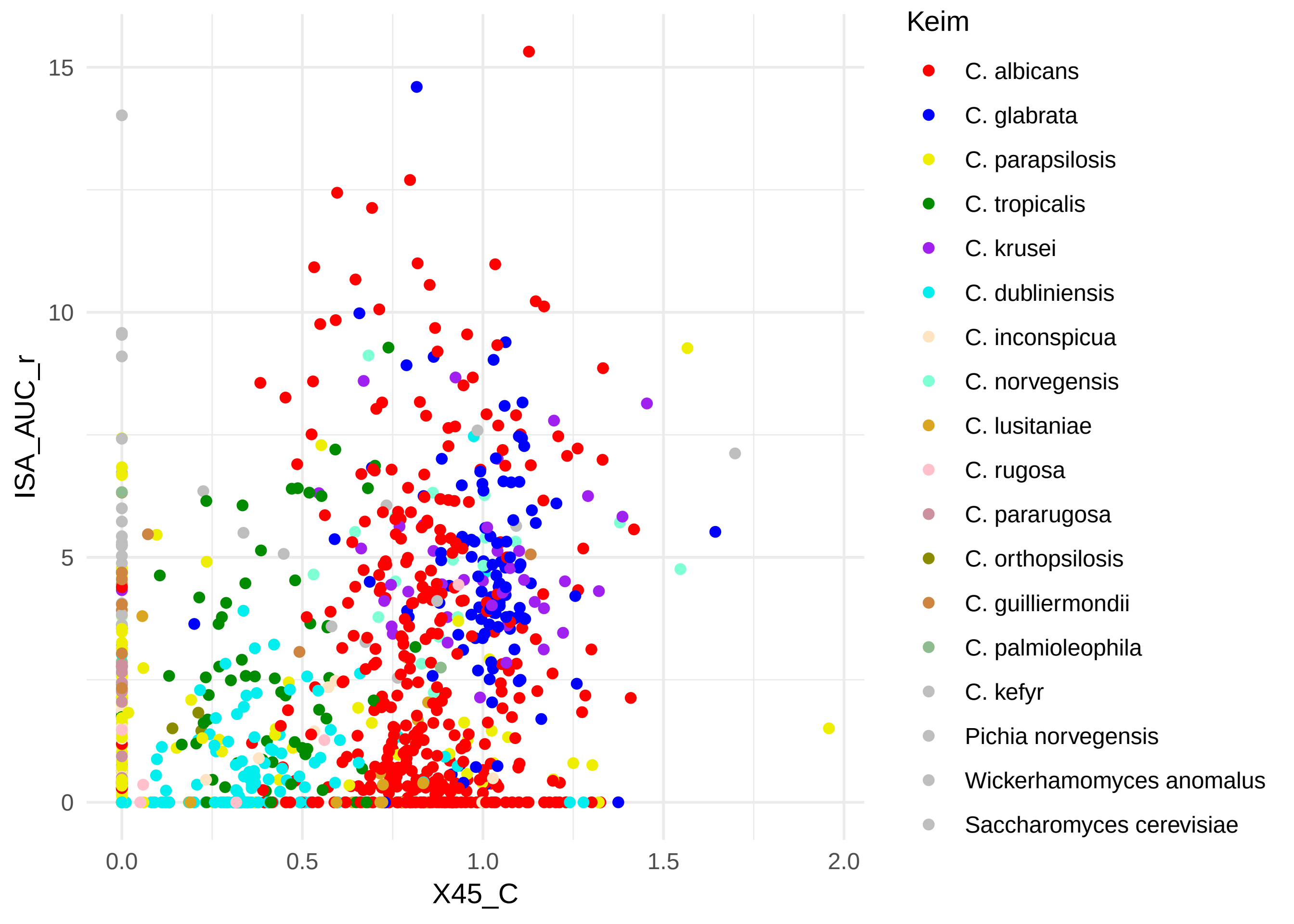

Keim
C. albicans
C. glabrata
C. parapsilosis
C. tropicalis
C. krusei
C. dubliniensis
C. inconspicua
C. norvegensis
C. lusitaniae
C. rugosa
C. pararugosa
C. orthopsilosis
C. guilliermondii
C. palmioleophila
C. kefyr
Pichia norvegensis
Wickerhamomyces anomalus
Saccharomyces cerevisiae
ISA_AUC_r
15
10
5
0
0.0
0.5
1.0
1.5
2.0
X45_C

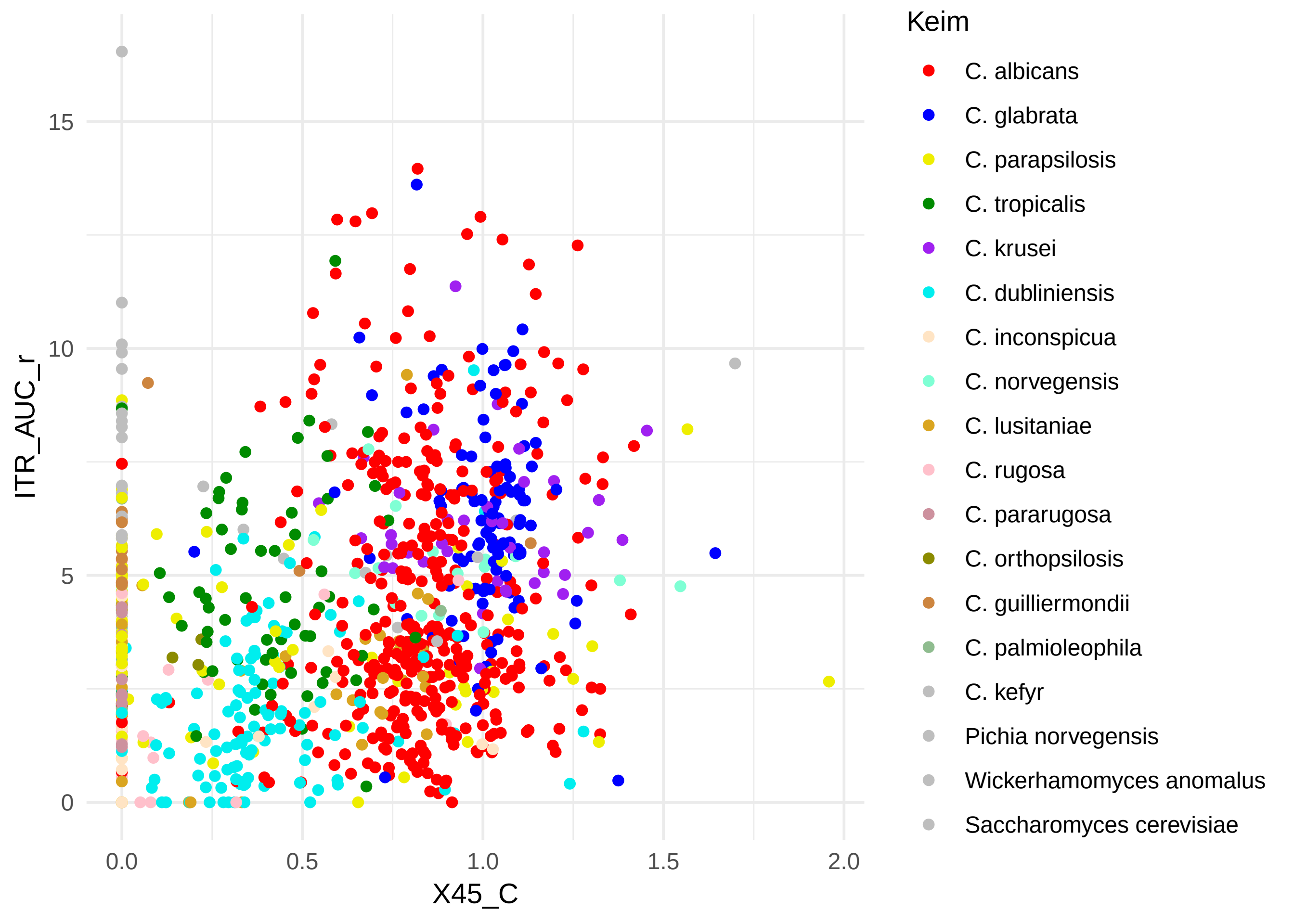

Keim
C. albicans
C. glabrata
C. parapsilosis
C. tropicalis
C. krusei
C. dubliniensis
C. inconspicua
C. norvegensis
C. lusitaniae
C. rugosa
C. pararugosa
C. orthopsilosis
C. guilliermondii
C. palmioleophila
C. kefyr
Pichia norvegensis
Wickerhamomyces anomalus
Saccharomyces cerevisiae
ITR_AUC_r
X45_C
0
5
10
15
0.0
0.5
1.0
1.5
2.0

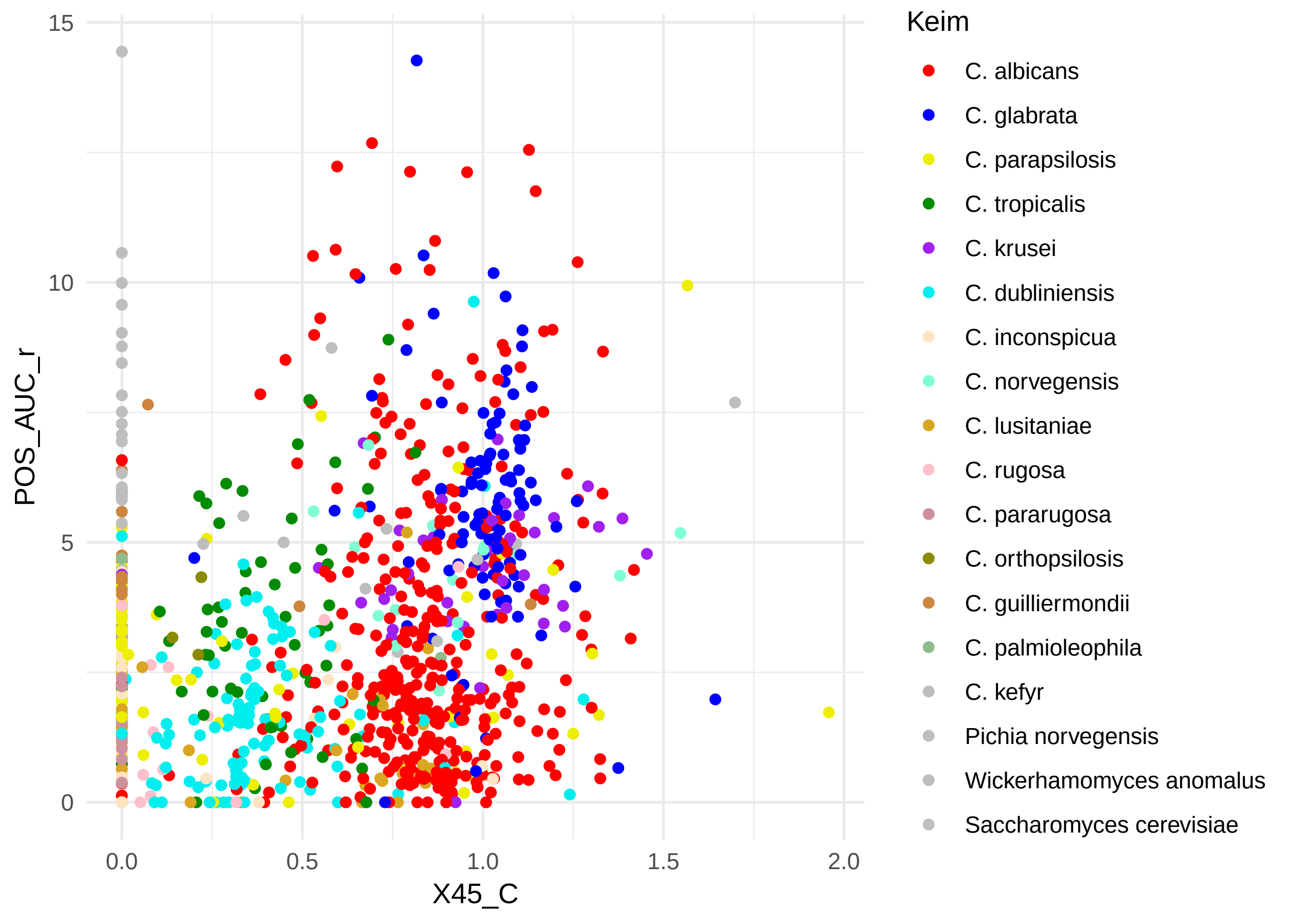
Keim
C. albicans
C. glabrata
C. parapsilosis
C. tropicalis
C. krusei
C. dubliniensis
C. inconspicua
C. norvegensis
C. lusitaniae
C. rugosa
C. pararugosa
C. orthopsilosis
C. guilliermondii
C. palmioleophila
C. kefyr
Pichia norvegensis
Wickerhamomyces anomalus
Saccharomyces cerevisiae
POS_AUC_r
X45_C
0
5
10
15
0.0
0.5
1.0
1.5
2.0

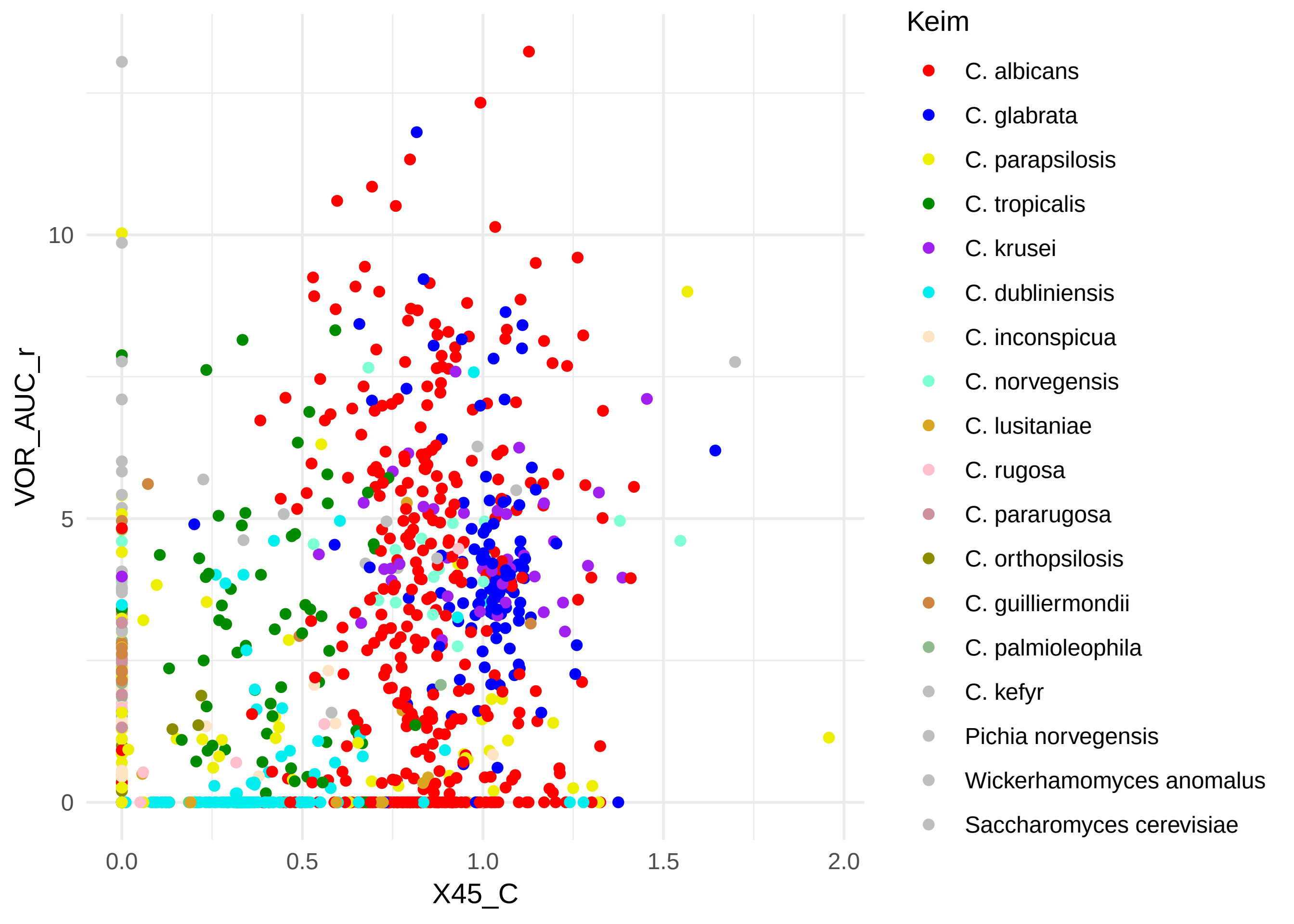

Keim
C. albicans
C. glabrata
C. parapsilosis
C. tropicalis
C. krusei
C. dubliniensis
C. inconspicua
C. norvegensis
C. lusitaniae
C. rugosa
C. pararugosa
C. orthopsilosis
C. guilliermondii
C. palmioleophila
C. kefyr
Pichia norvegensis
Wickerhamomyces anomalus
Saccharomyces cerevisiae
VOR_AUC_r
10
5
0
0.0
0.5
1.0
1.5
2.0
X45_C

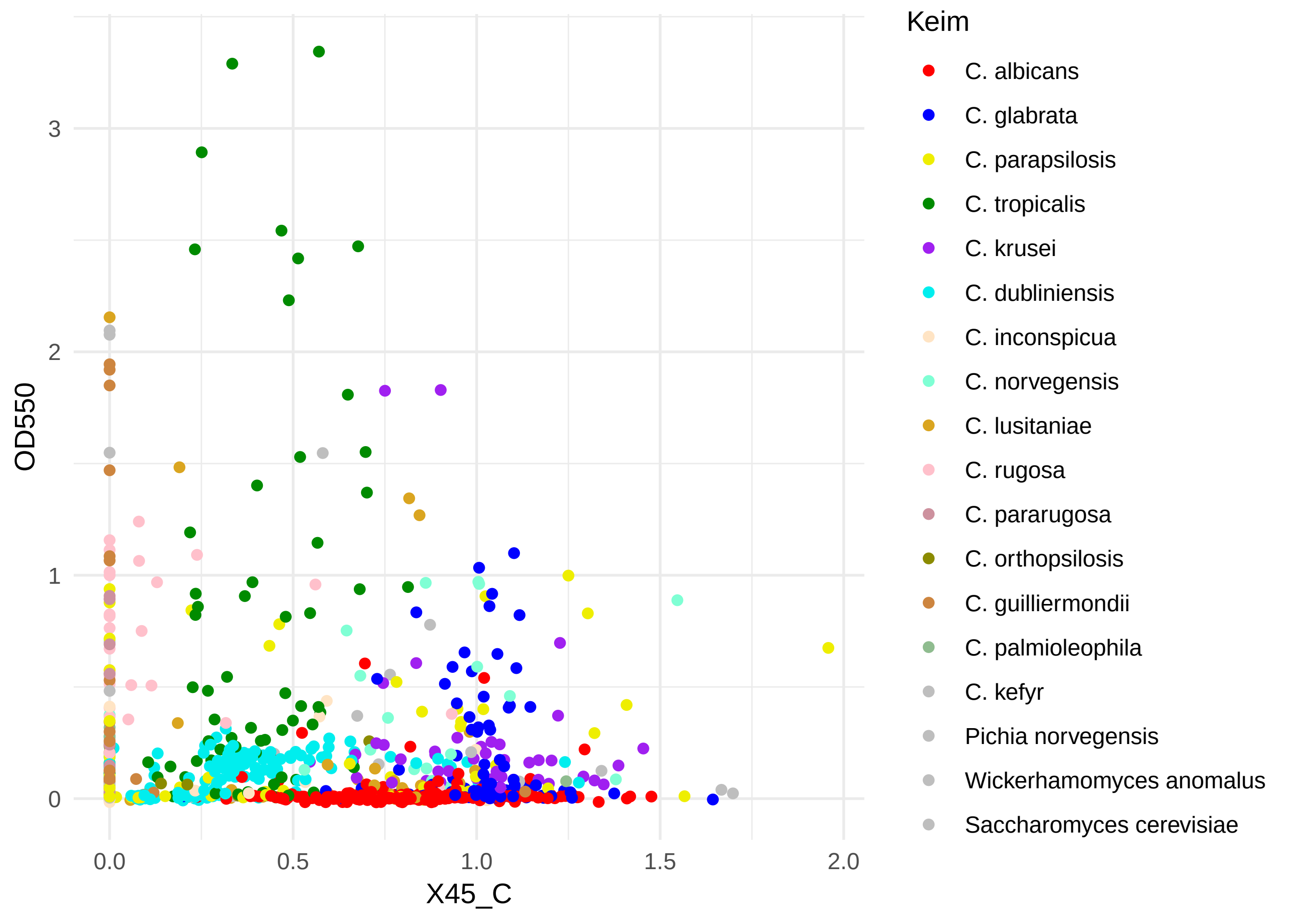

OD550
X45_C
0.0
0.5
1.0
1.5
2.0
0
1
2
3
Keim
C. albicans
C. glabrata
C. parapsilosis
C. tropicalis
C. krusei
C. dubliniensis
C. inconspicua
C. norvegensis
C. lusitaniae
C. rugosa
C. pararugosa
C. orthopsilosis
C. guilliermondii
C. palmioleophila
C. kefyr
Pichia norvegensis
Wickerhamomyces anomalus
Saccharomyces cerevisiae

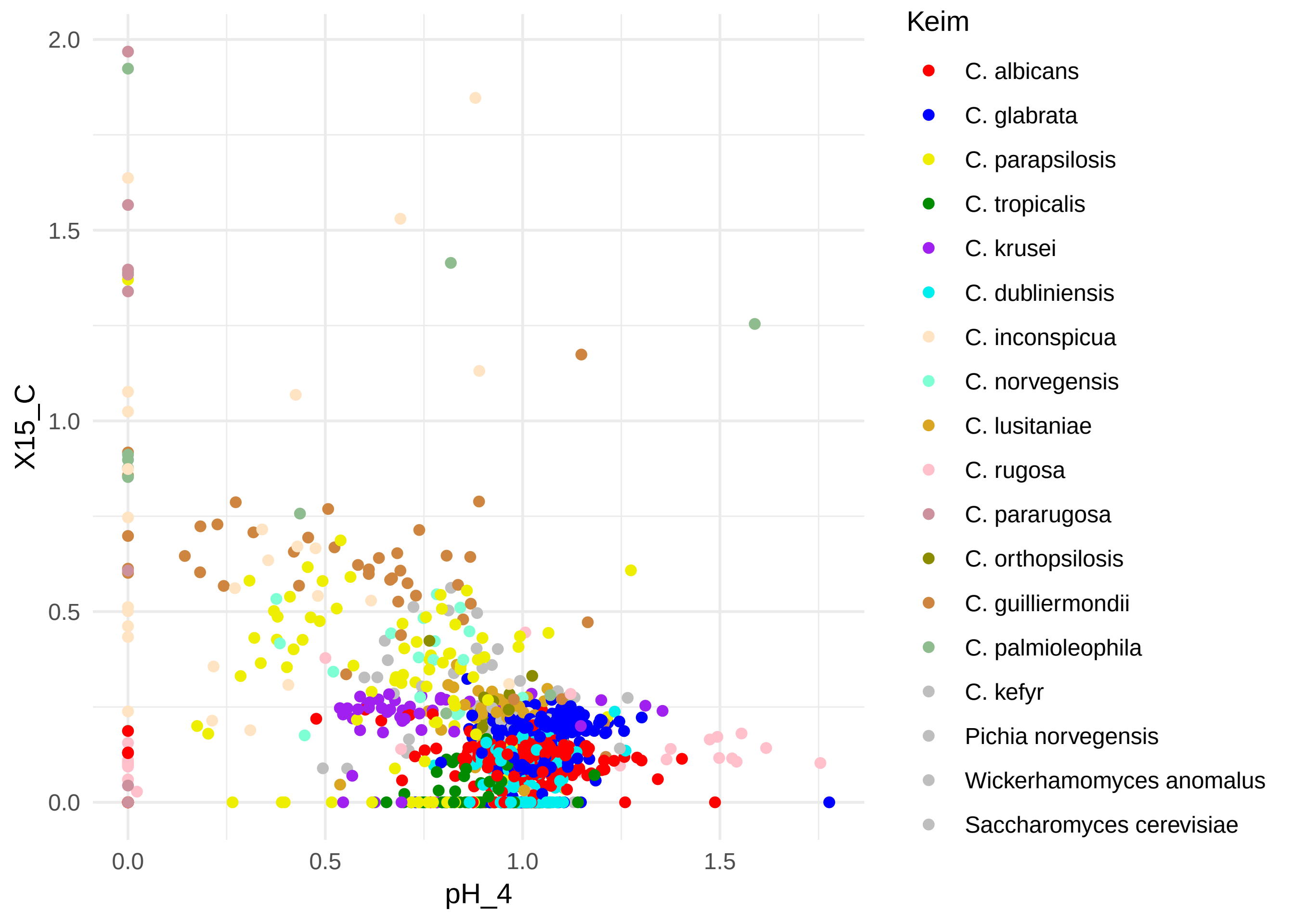

Keim
C. albicans
C. glabrata
C. parapsilosis
C. tropicalis
C. krusei
C. dubliniensis
C. inconspicua
C. norvegensis
C. lusitaniae
C. rugosa
C. pararugosa
C. orthopsilosis
C. guilliermondii
C. palmioleophila
C. kefyr
Pichia norvegensis
Wickerhamomyces anomalus
Saccharomyces cerevisiae
X15_C
2.0
1.5
1.0
0.5
0.0
0.0
0.5
1.0
1.5
pH_4

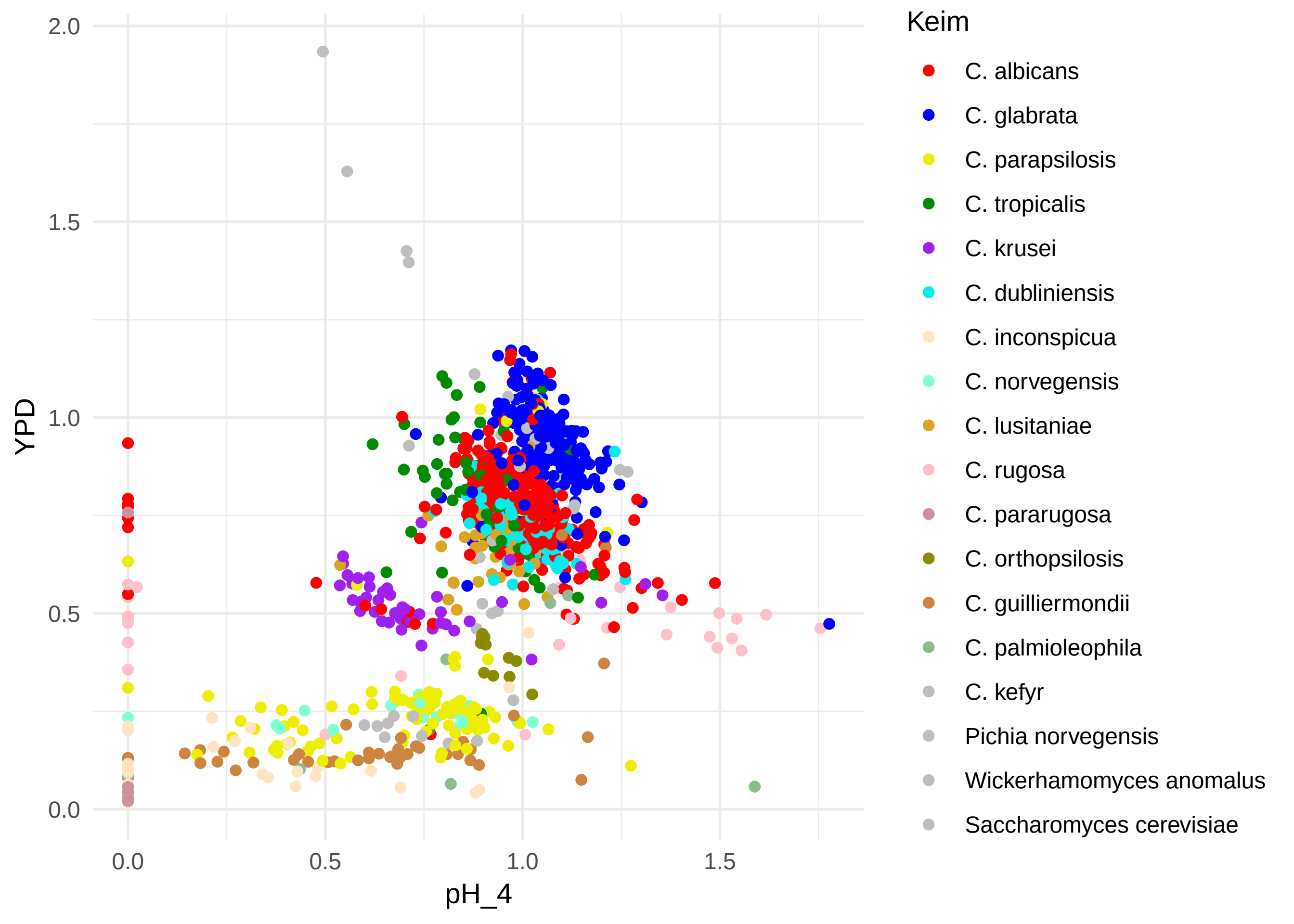

YPD
pH_4
2.0
1.5
1.0
0.5
0.0
0.0
0.5
1.0
1.5
Keim
C. albicans
C. glabrata
C. parapsilosis
C. tropicalis
C. krusei
C. dubliniensis
C. inconspicua
C. norvegensis
C. lusitaniae
C. rugosa
C. pararugosa
C. orthopsilosis
C. guilliermondii
C. palmioleophila
C. kefyr
Pichia norvegensis
Wickerhamomyces anomalus
Saccharomyces cerevisiae

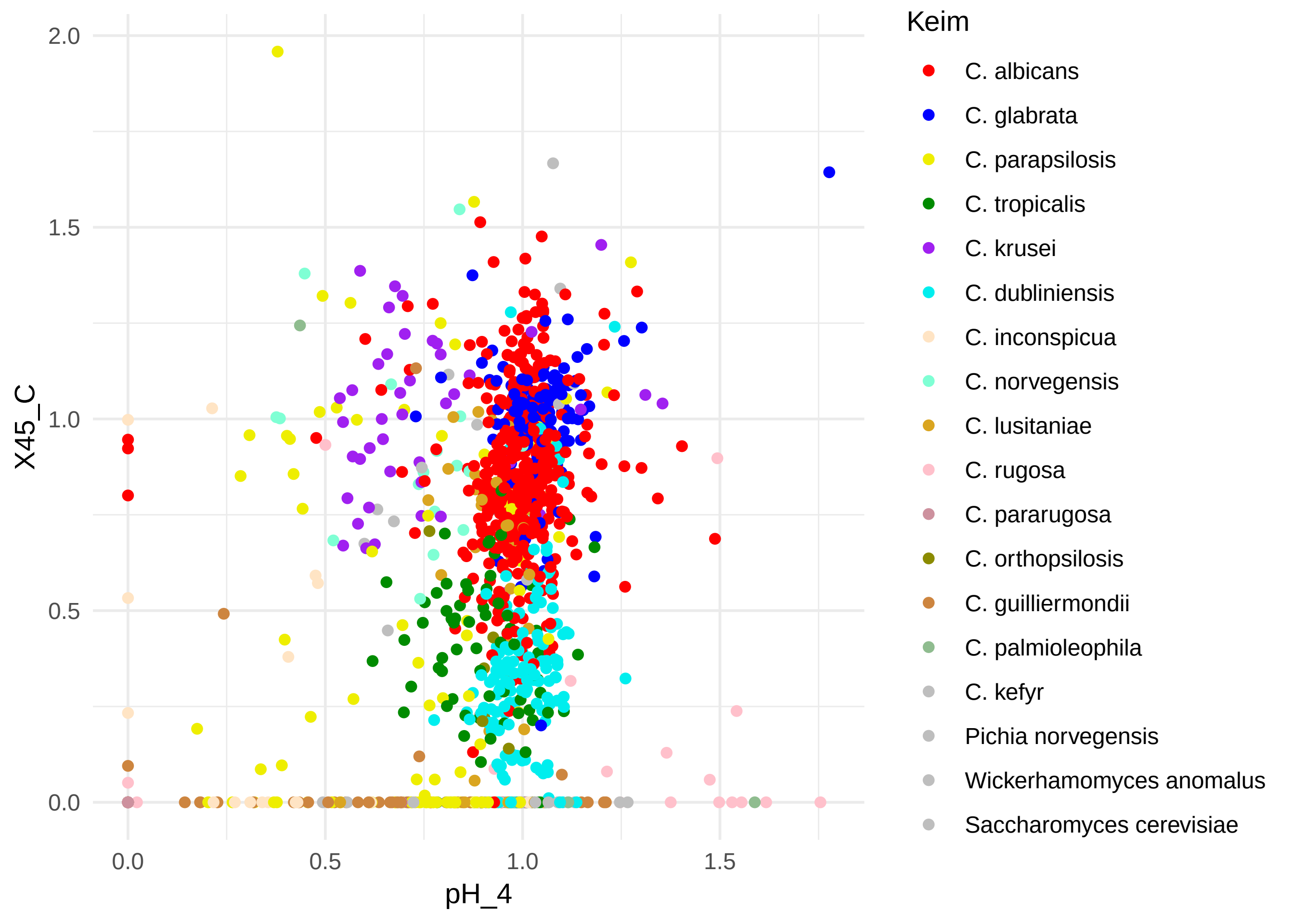

Keim
C. albicans
C. glabrata
C. parapsilosis
C. tropicalis
C. krusei
C. dubliniensis
C. inconspicua
C. norvegensis
C. lusitaniae
C. rugosa
C. pararugosa
C. orthopsilosis
C. guilliermondii
C. palmioleophila
C. kefyr
Pichia norvegensis
Wickerhamomyces anomalus
Saccharomyces cerevisiae
X45_C
pH_4
0.0
0.5
1.0
1.5
2.0

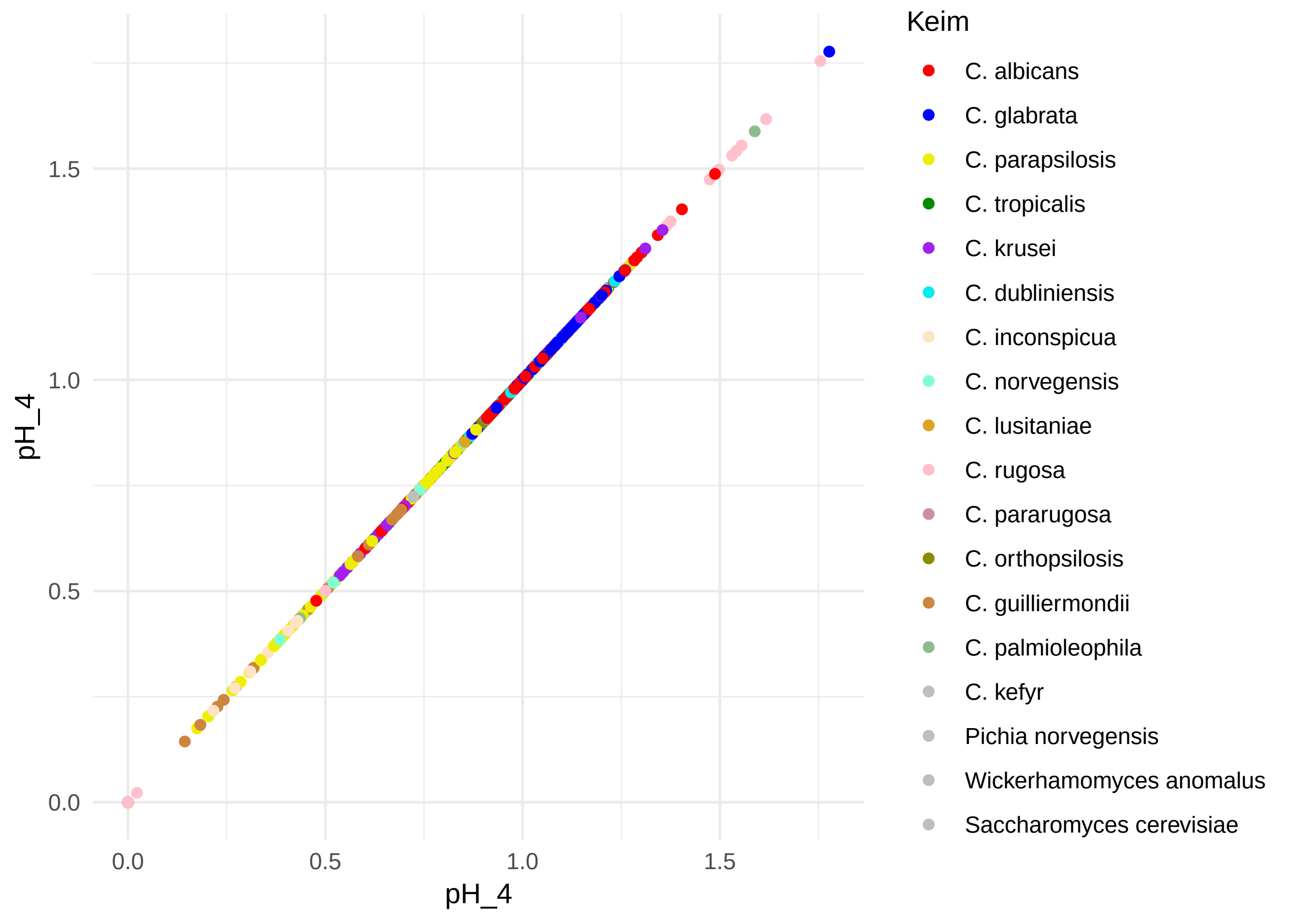
Keim
C. albicans
C. glabrata
C. parapsilosis
C. tropicalis
C. krusei
C. dubliniensis
C. inconspicua
C. norvegensis
C. lusitaniae
C. rugosa
C. pararugosa
C. orthopsilosis
C. guilliermondii
C. palmioleophila
C. kefyr
Pichia norvegensis
Wickerhamomyces anomalus
Saccharomyces cerevisiae
pH_4
pH_4
0.0
0.5
1.0
1.5

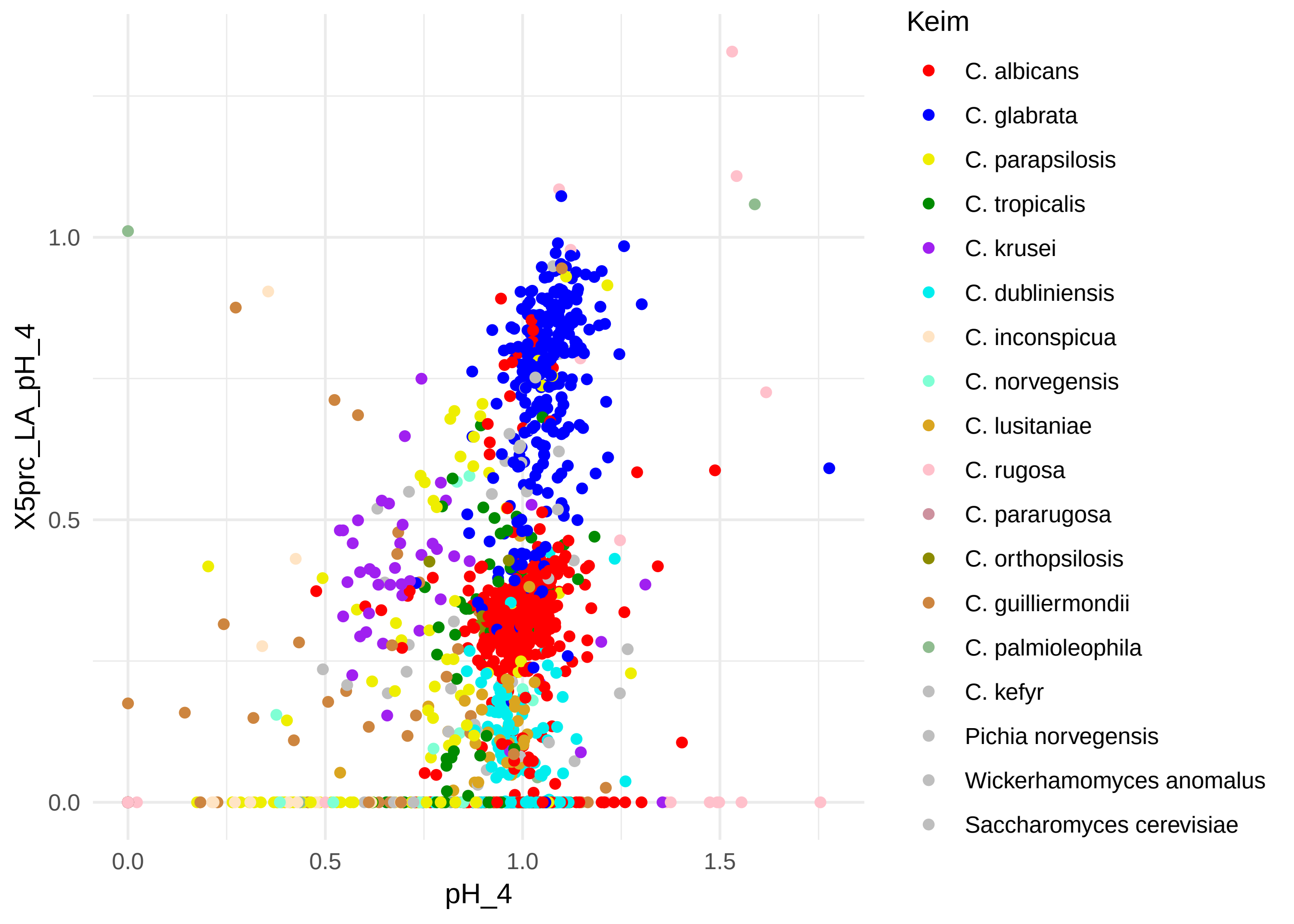
Keim
C. albicans
C. glabrata
C. parapsilosis
C. tropicalis
C. krusei
C. dubliniensis
C. inconspicua
C. norvegensis
C. lusitaniae
C. rugosa
C. pararugosa
C. orthopsilosis
C. guilliermondii
C. palmioleophila
C. kefyr
Pichia norvegensis
Wickerhamomyces anomalus
Saccharomyces cerevisiae
X5prc_LA_pH_4
1.0
0.5
0.0
0.0
0.5
1.0
1.5
pH_4

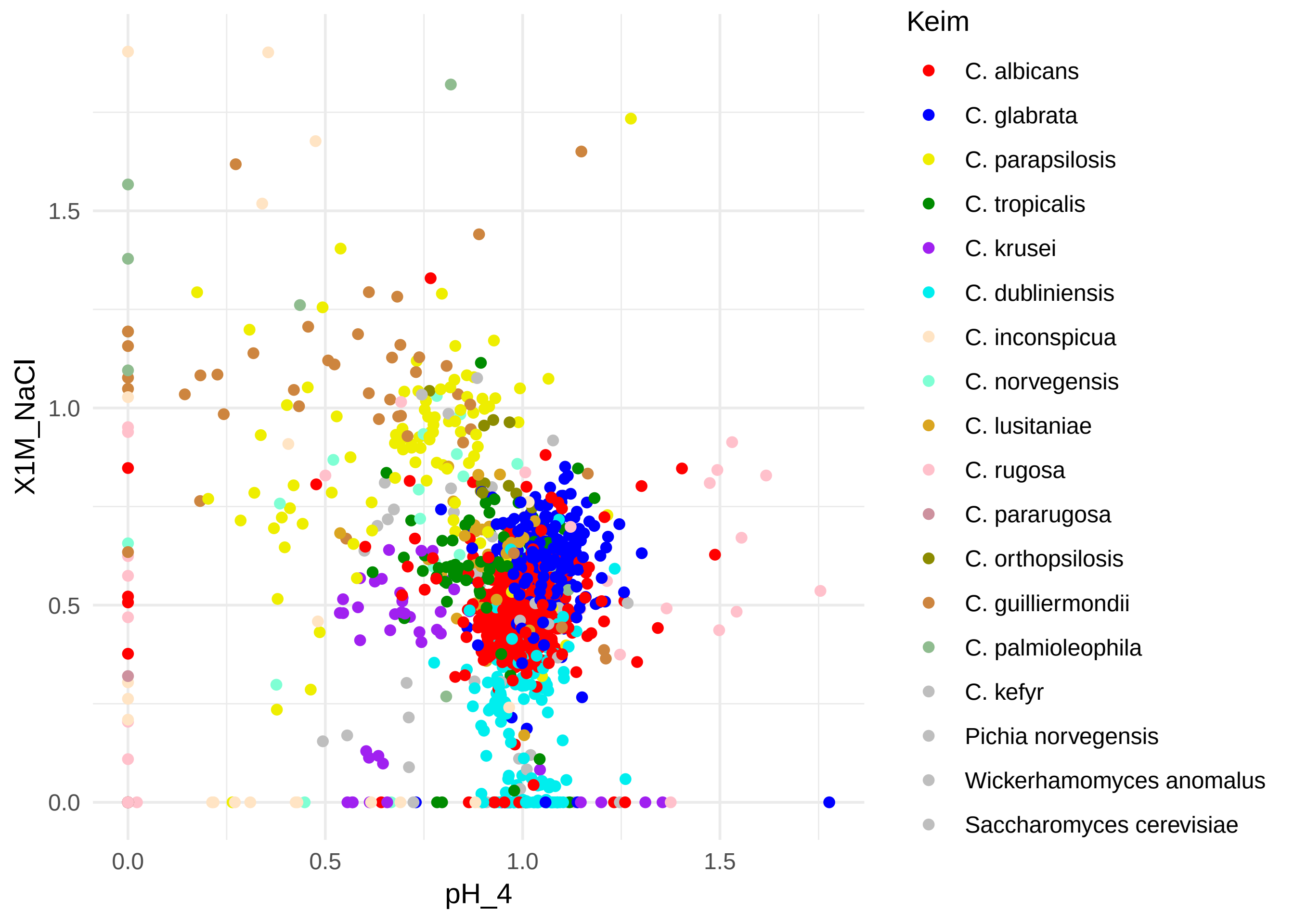

Keim
C. albicans
C. glabrata
C. parapsilosis
C. tropicalis
C. krusei
C. dubliniensis
C. inconspicua
C. norvegensis
C. lusitaniae
C. rugosa
C. pararugosa
C. orthopsilosis
C. guilliermondii
C. palmioleophila
C. kefyr
Pichia norvegensis
Wickerhamomyces anomalus
Saccharomyces cerevisiae
X1M_NaCl
1.5
1.0
0.5
0.0
0.0
0.5
1.0
1.5
pH_4

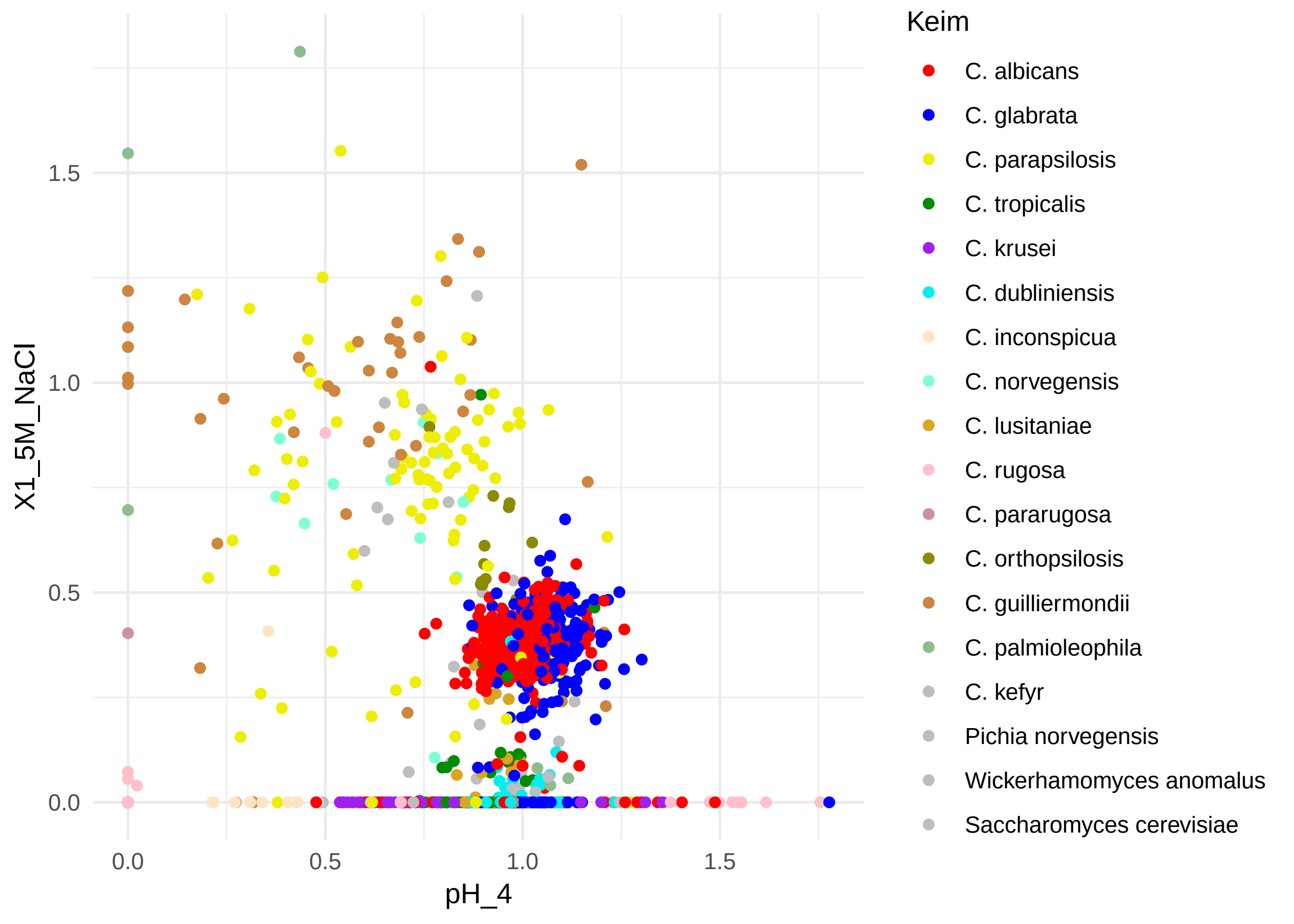

Keim
C. albicans
C. glabrata
C. parapsilosis
C. tropicalis
C. krusei
C. dubliniensis
C. inconspicua
C. norvegensis
C. lusitaniae
C. rugosa
C. pararugosa
C. orthopsilosis
C. guilliermondii
C. palmioleophila
C. kefyr
Pichia norvegensis
Wickerhamomyces anomalus
Saccharomyces cerevisiae
X1_5M_NaCl
pH_4
0.0
0.5
1.0
1.5

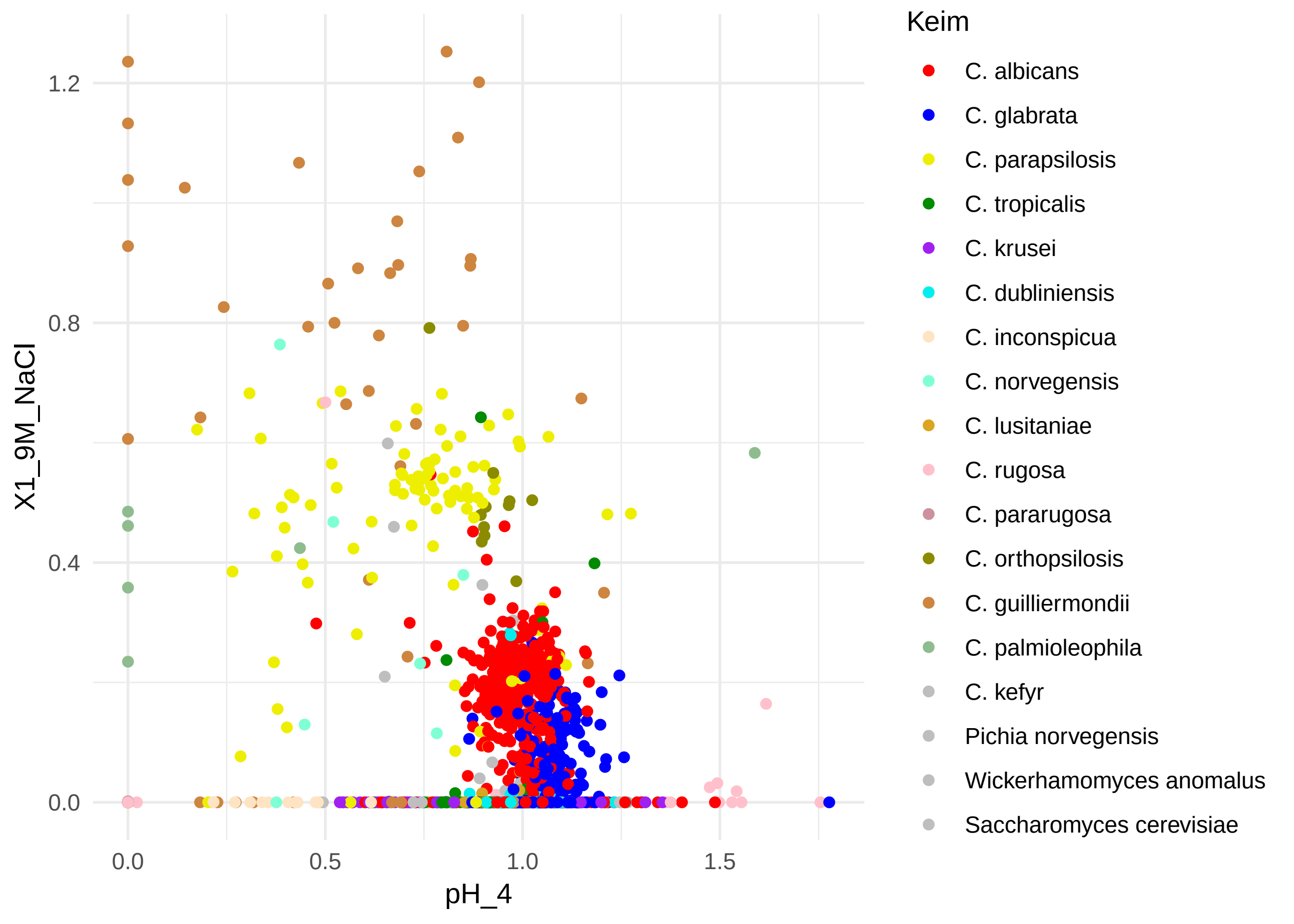

Keim
C. albicans
C. glabrata
C. parapsilosis
C. tropicalis
C. krusei
C. dubliniensis
C. inconspicua
C. norvegensis
C. lusitaniae
C. rugosa
C. pararugosa
C. orthopsilosis
C. guilliermondii
C. palmioleophila
C. kefyr
Pichia norvegensis
Wickerhamomyces anomalus
Saccharomyces cerevisiae
X1_9M_NaCl
1.2
0.8
0.4
0.0
0.0
0.5
1.0
1.5
pH_4

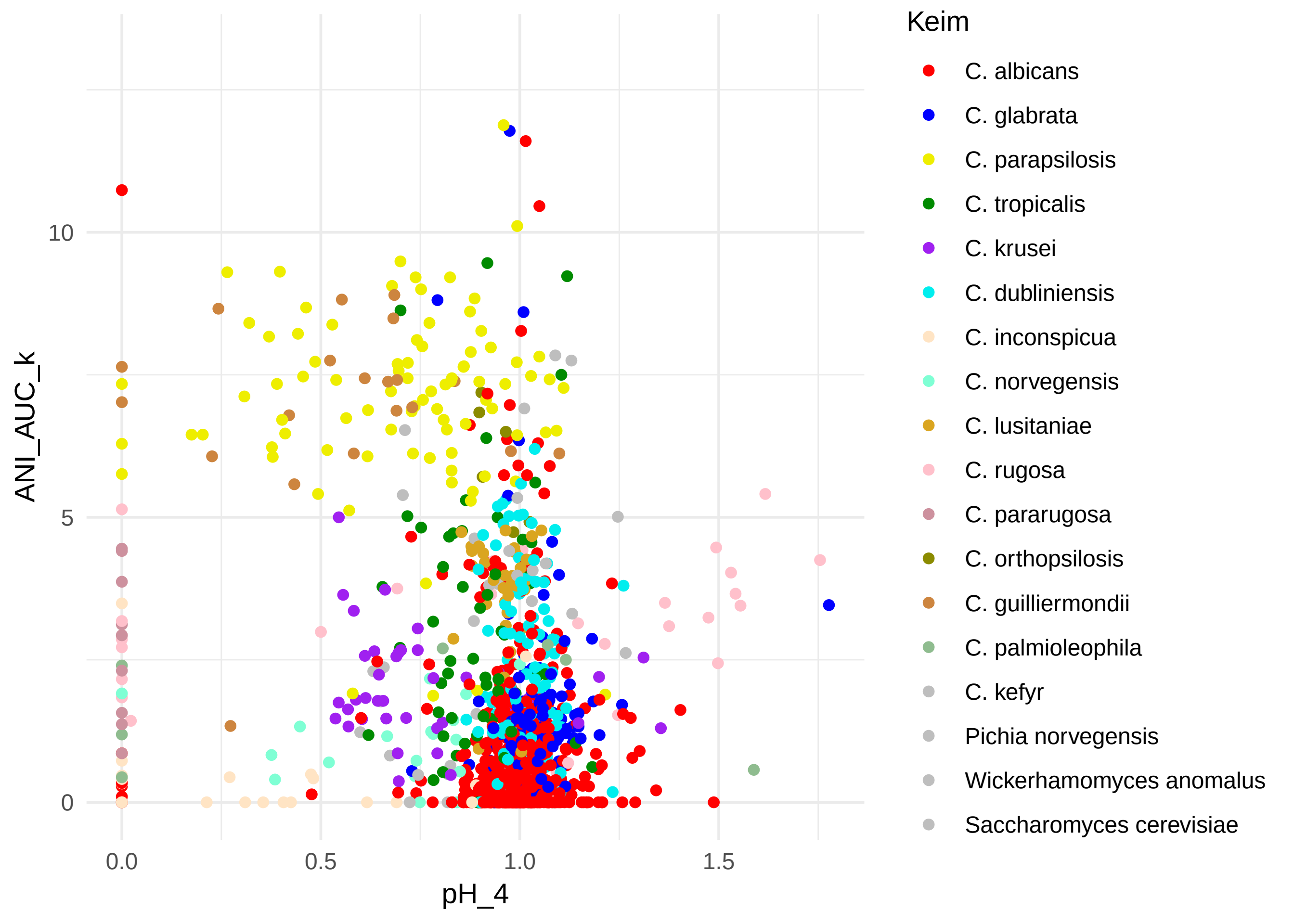
ANI_AUC_k
10
5
0
0.0
0.5
1.0
1.5
pH_4
Keim
C. albicans
C. glabrata
C. parapsilosis
C. tropicalis
C. krusei
C. dubliniensis
C. inconspicua
C. norvegensis
C. lusitaniae
C. rugosa
C. pararugosa
C. orthopsilosis
C. guilliermondii
C. palmioleophila
C. kefyr
Pichia norvegensis
Wickerhamomyces anomalus
Saccharomyces cerevisiae

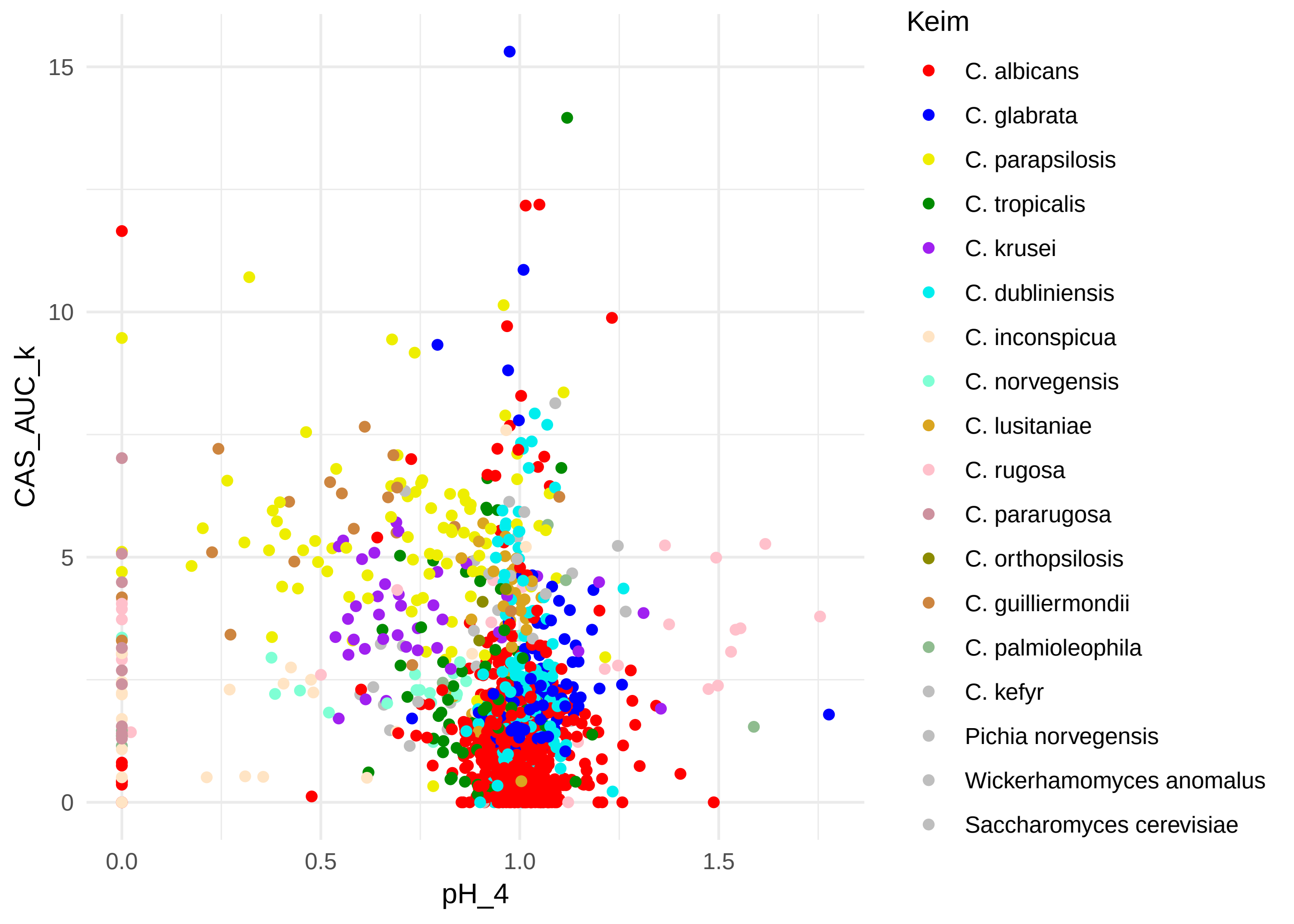
Keim
C. albicans
C. glabrata
C. parapsilosis
C. tropicalis
C. krusei
C. dubliniensis
C. inconspicua
C. norvegensis
C. lusitaniae
C. rugosa
C. pararugosa
C. orthopsilosis
C. guilliermondii
C. palmioleophila
C. kefyr
Pichia norvegensis
Wickerhamomyces anomalus
Saccharomyces cerevisiae
CAS_AUC_k
pH_4
0
5
10
15
0.0
0.5
1.0
1.5

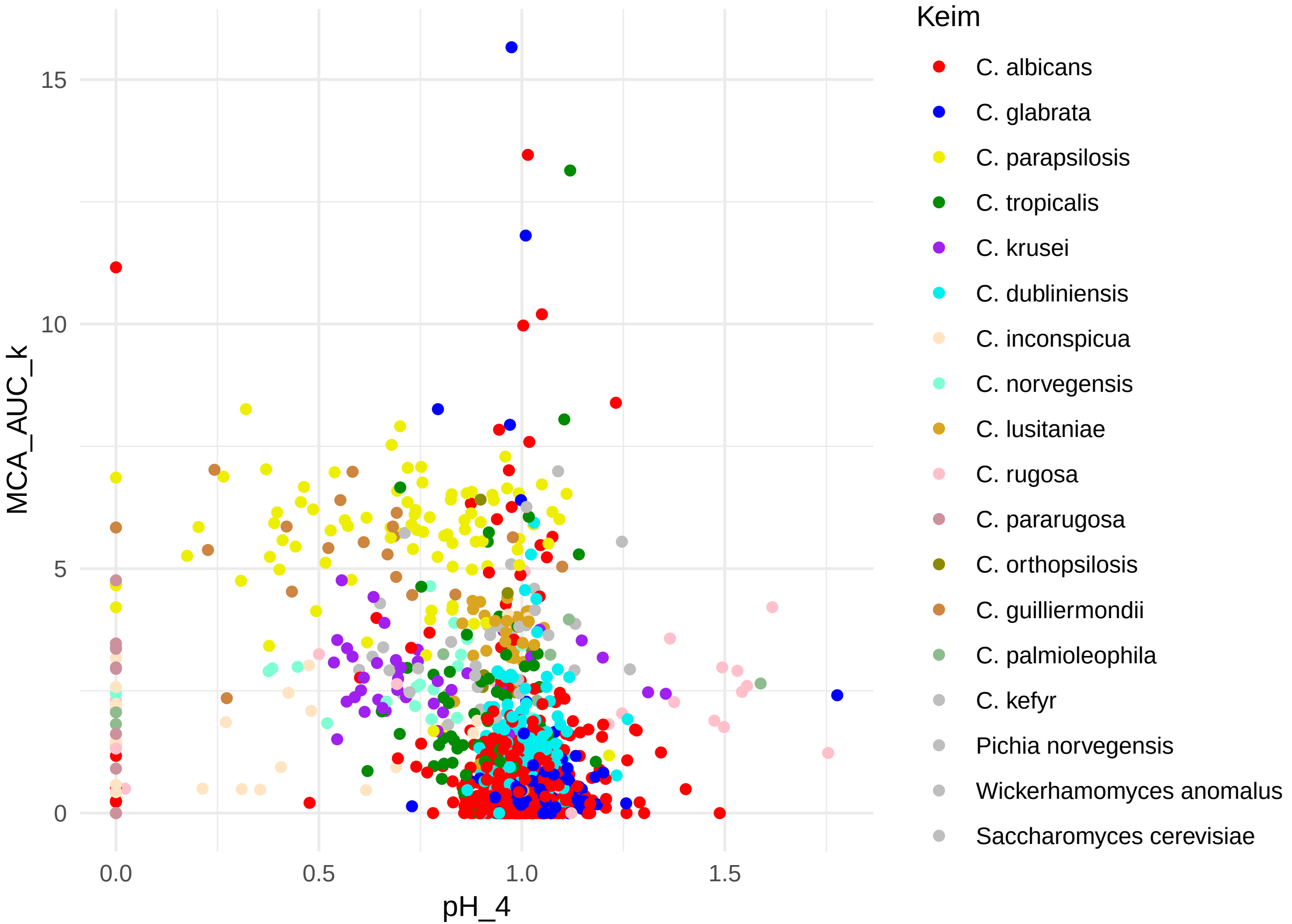

Keim
C. albicans
C. glabrata
C. parapsilosis
C. tropicalis
C. krusei
C. dubliniensis
C. inconspicua
C. norvegensis
C. lusitaniae
C. rugosa
C. pararugosa
C. orthopsilosis
C. guilliermondii
C. palmioleophila
C. kefyr
Pichia norvegensis
Wickerhamomyces anomalus
Saccharomyces cerevisiae
MCA_AUC_k
pH_4
0
5
10
15
0.0
0.5
1.0
1.5

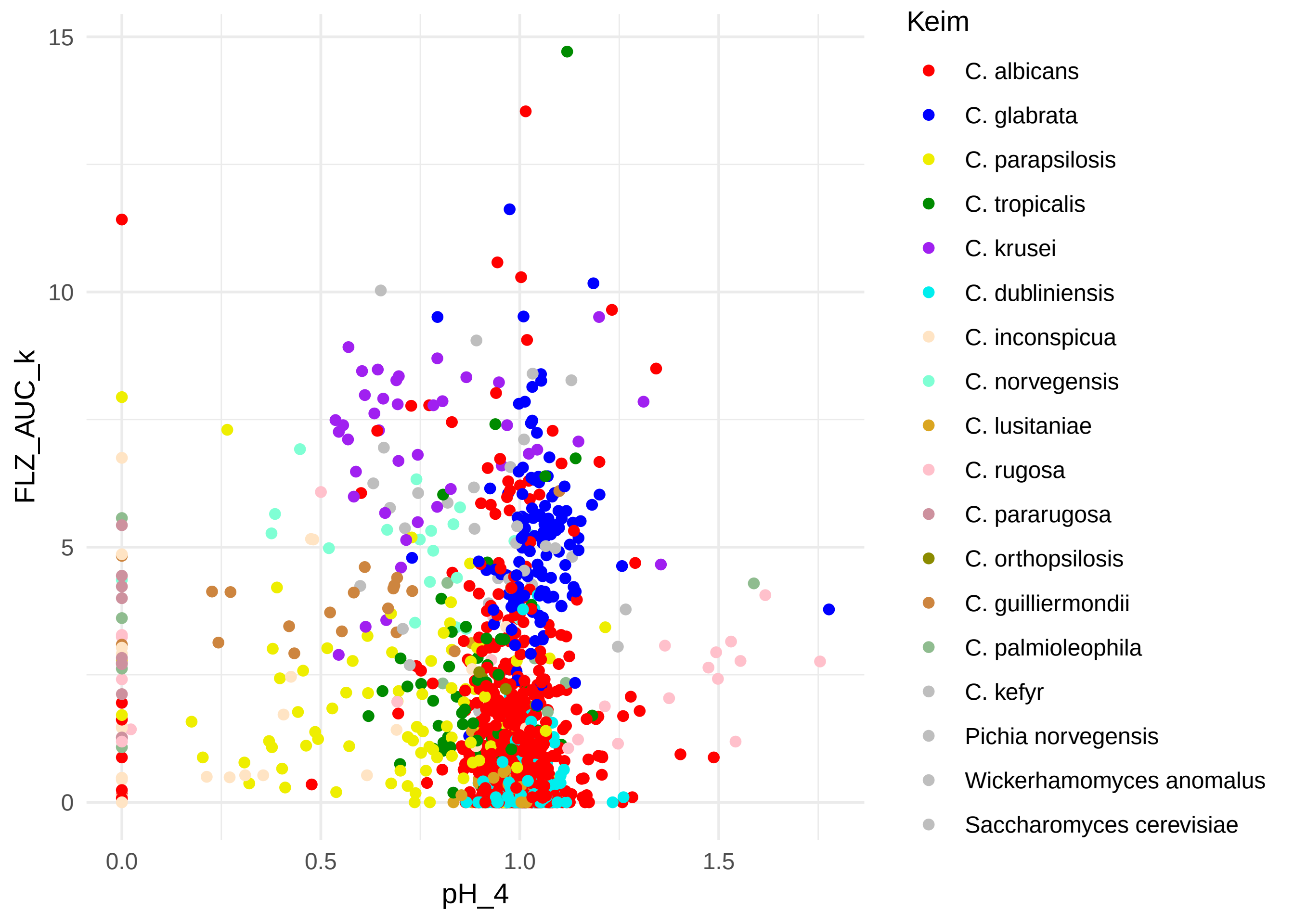
Keim
C. albicans
C. glabrata
C. parapsilosis
C. tropicalis
C. krusei
C. dubliniensis
C. inconspicua
C. norvegensis
C. lusitaniae
C. rugosa
C. pararugosa
C. orthopsilosis
C. guilliermondii
C. palmioleophila
C. kefyr
Pichia norvegensis
Wickerhamomyces anomalus
Saccharomyces cerevisiae
FLZ_AUC_k
pH_4
0
5
10
15
0.0
0.5
1.0
1.5

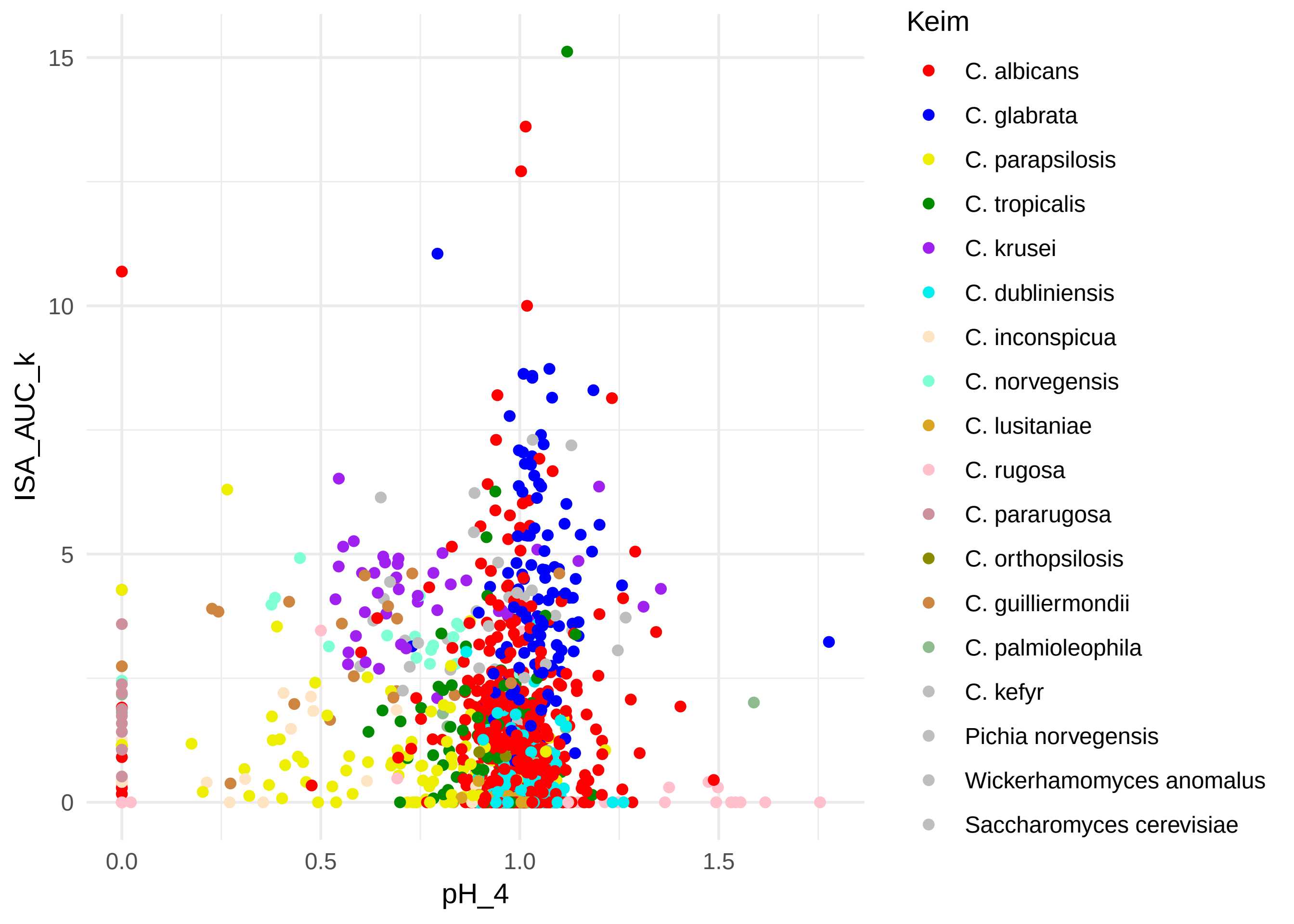
Keim
C. albicans
C. glabrata
C. parapsilosis
C. tropicalis
C. krusei
C. dubliniensis
C. inconspicua
C. norvegensis
C. lusitaniae
C. rugosa
C. pararugosa
C. orthopsilosis
C. guilliermondii
C. palmioleophila
C. kefyr
Pichia norvegensis
Wickerhamomyces anomalus
Saccharomyces cerevisiae
ISA_AUC_k
pH_4
0
5
10
15
0.0
0.5
1.0
1.5

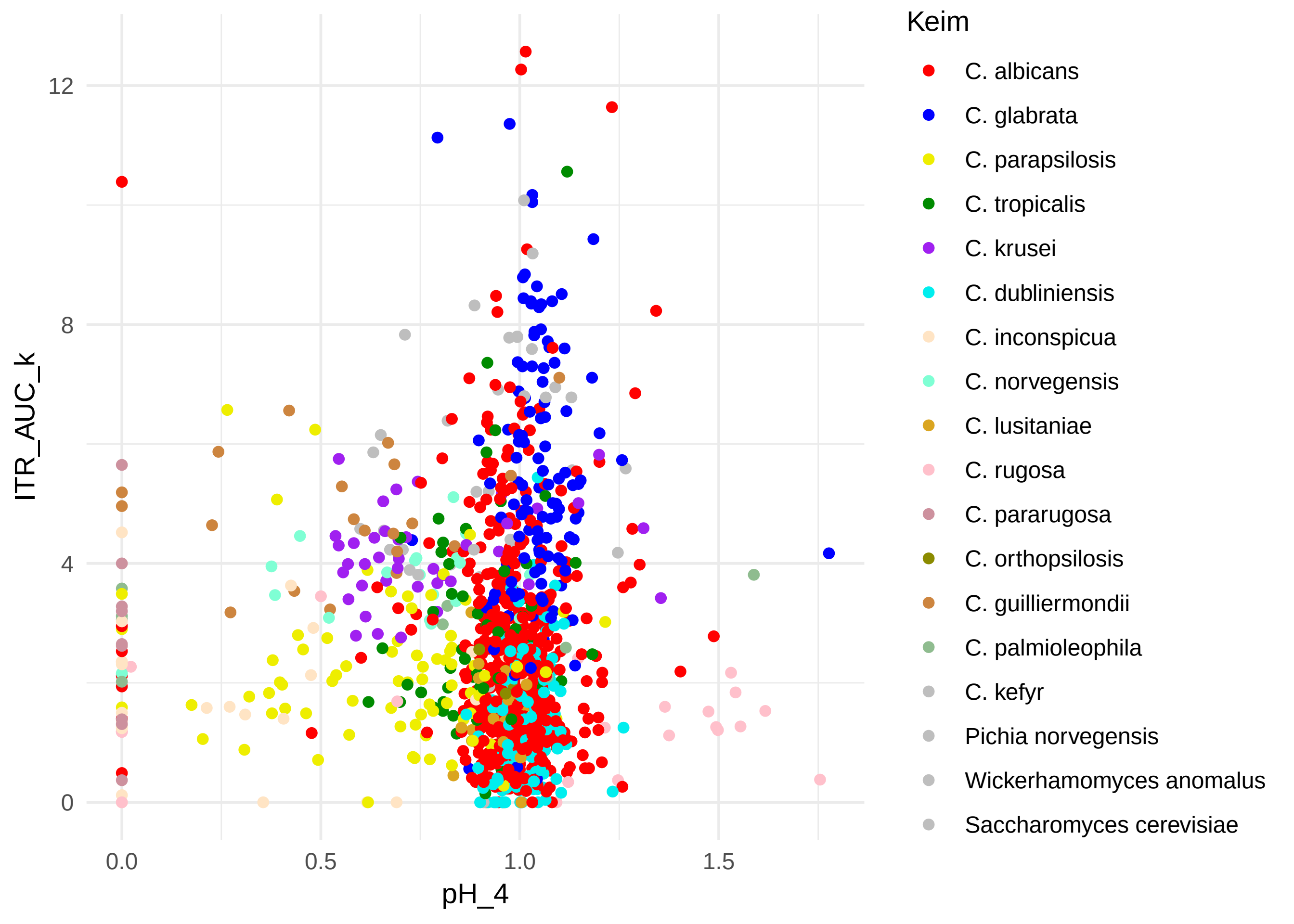

Keim
C. albicans
C. glabrata
C. parapsilosis
C. tropicalis
C. krusei
C. dubliniensis
C. inconspicua
C. norvegensis
C. lusitaniae
C. rugosa
C. pararugosa
C. orthopsilosis
C. guilliermondii
C. palmioleophila
C. kefyr
Pichia norvegensis
Wickerhamomyces anomalus
Saccharomyces cerevisiae
ITR_AUC_k
0
4
8
12
pH_4
0.0
0.5
1.0
1.5

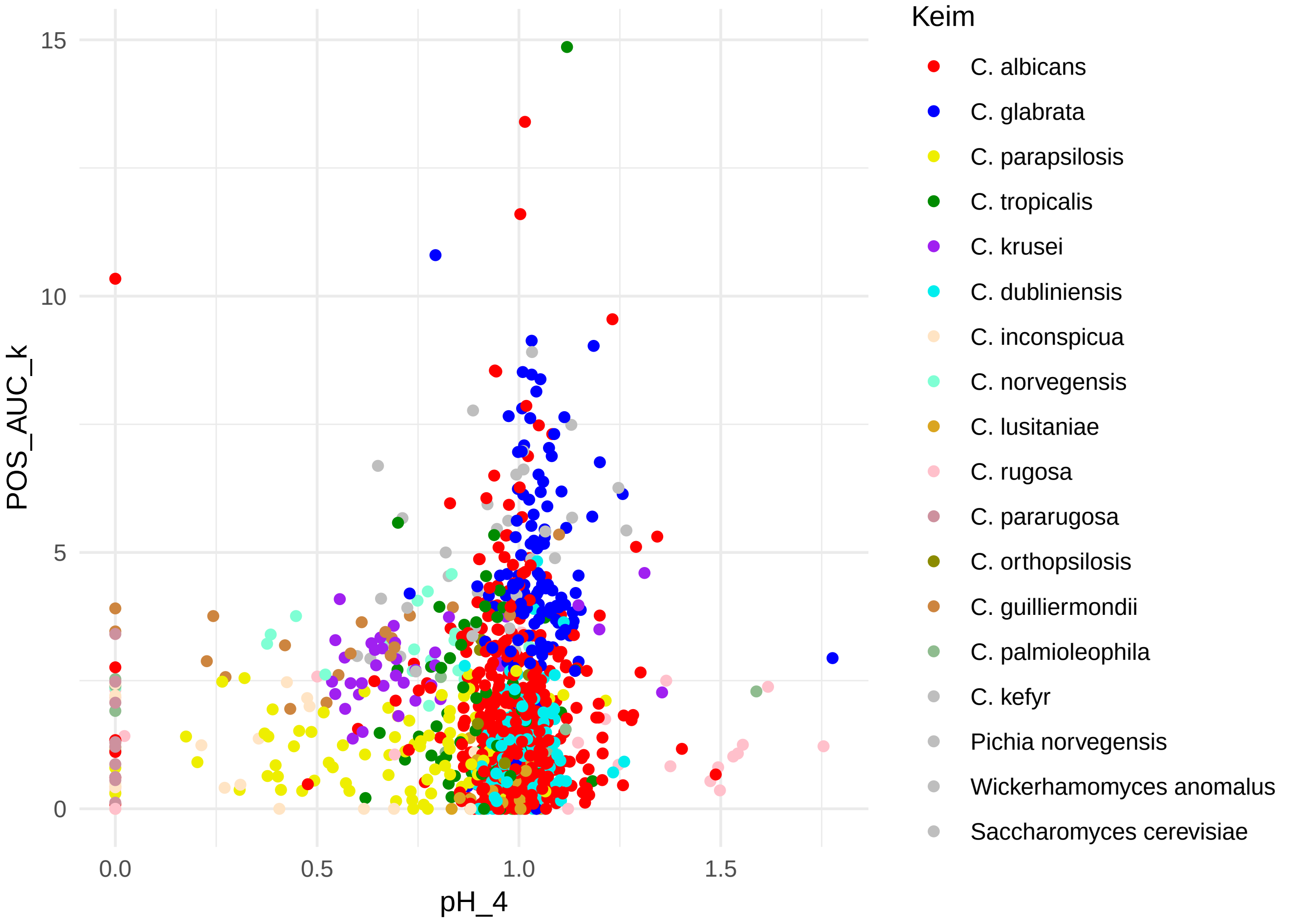

POS_AUC_k
15
10
5
0
0.0
0.5
1.0
1.5
pH_4
Keim
C. albicans
C. glabrata
C. parapsilosis
C. tropicalis
C. krusei
C. dubliniensis
C. inconspicua
C. norvegensis
C. lusitaniae
C. rugosa
C. pararugosa
C. orthopsilosis
C. guilliermondii
C. palmioleophila
C. kefyr
Pichia norvegensis
Wickerhamomyces anomalus
Saccharomyces cerevisiae

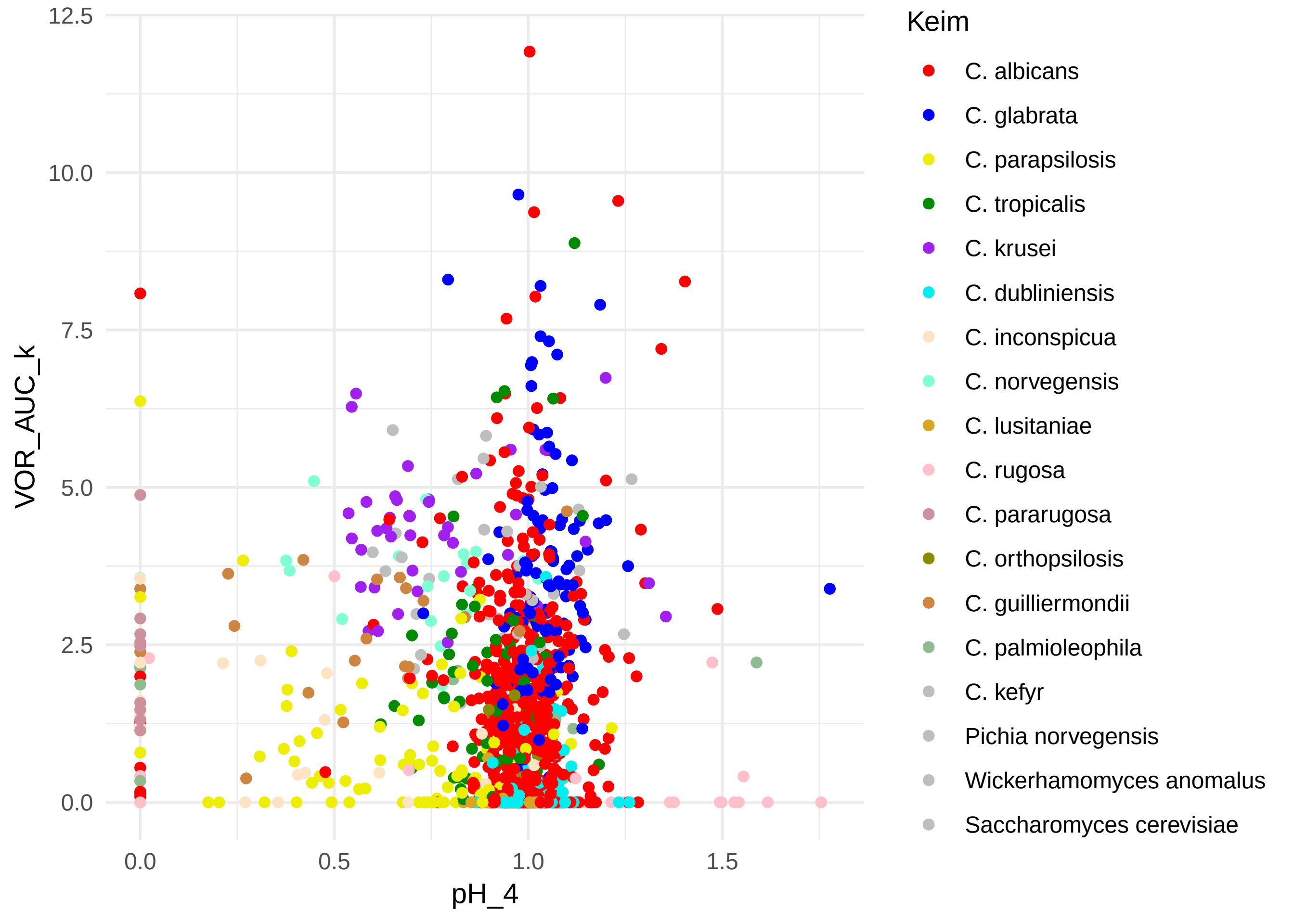

12.5
10.0
7.5
5.0
2.5
0.0
VOR_AUC_k
0.0
0.5
1.0
1.5
pH_4
Keim
C. albicans
C. glabrata
C. parapsilosis
C. tropicalis
C. krusei
C. dubliniensis
C. inconspicua
C. norvegensis
C. lusitaniae
C. rugosa
C. pararugosa
C. orthopsilosis
C. guilliermondii
C. palmioleophila
C. kefyr
Pichia norvegensis
Wickerhamomyces anomalus
Saccharomyces cerevisiae

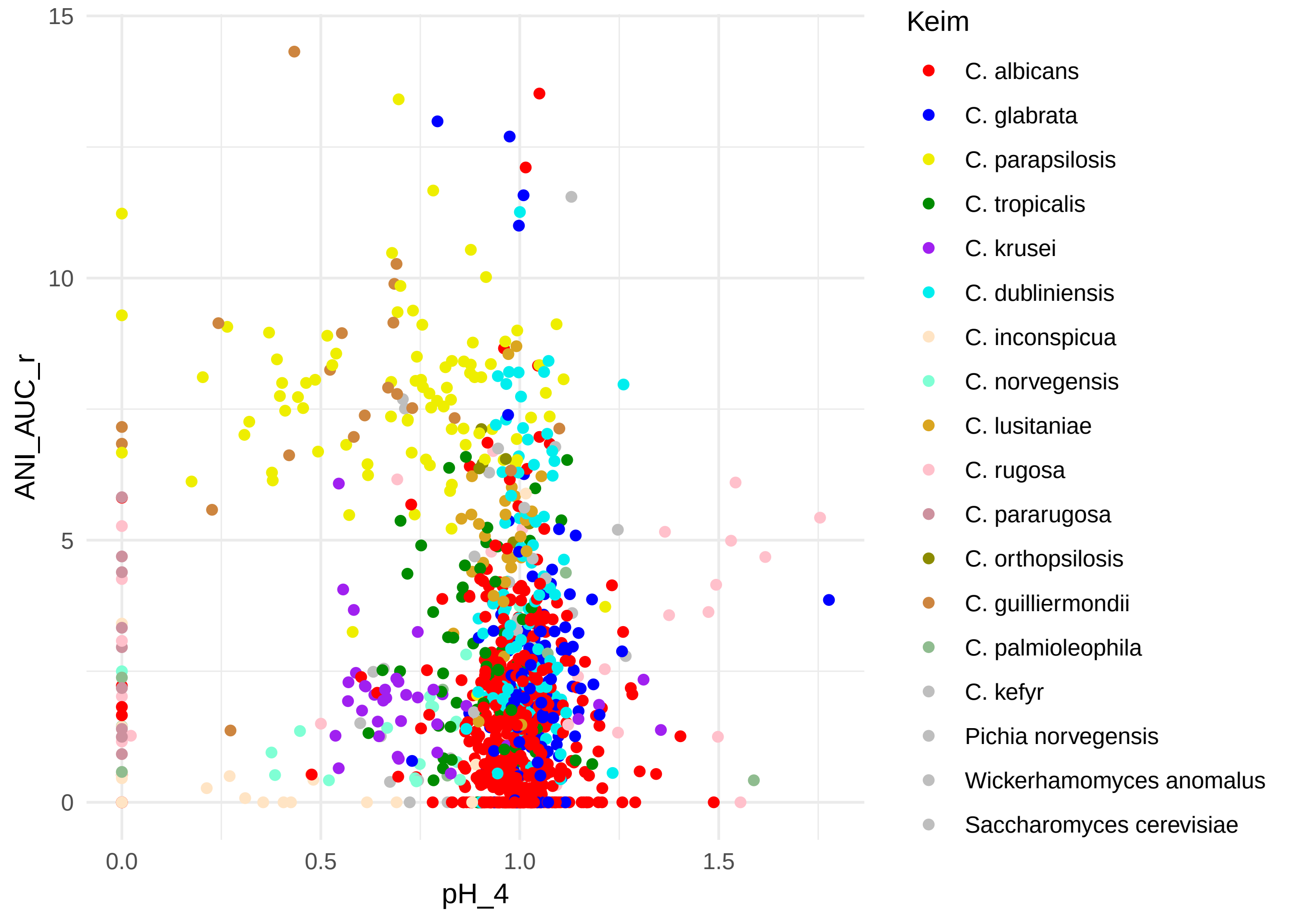

15
10
5
0
ANI_AUC_r
0.0
0.5
1.0
1.5
pH_4
Keim
C. albicans
C. glabrata
C. parapsilosis
C. tropicalis
C. krusei
C. dubliniensis
C. inconspicua
C. norvegensis
C. lusitaniae
C. rugosa
C. pararugosa
C. orthopsilosis
C. guilliermondii
C. palmioleophila
C. kefyr
Pichia norvegensis
Wickerhamomyces anomalus
Saccharomyces cerevisiae

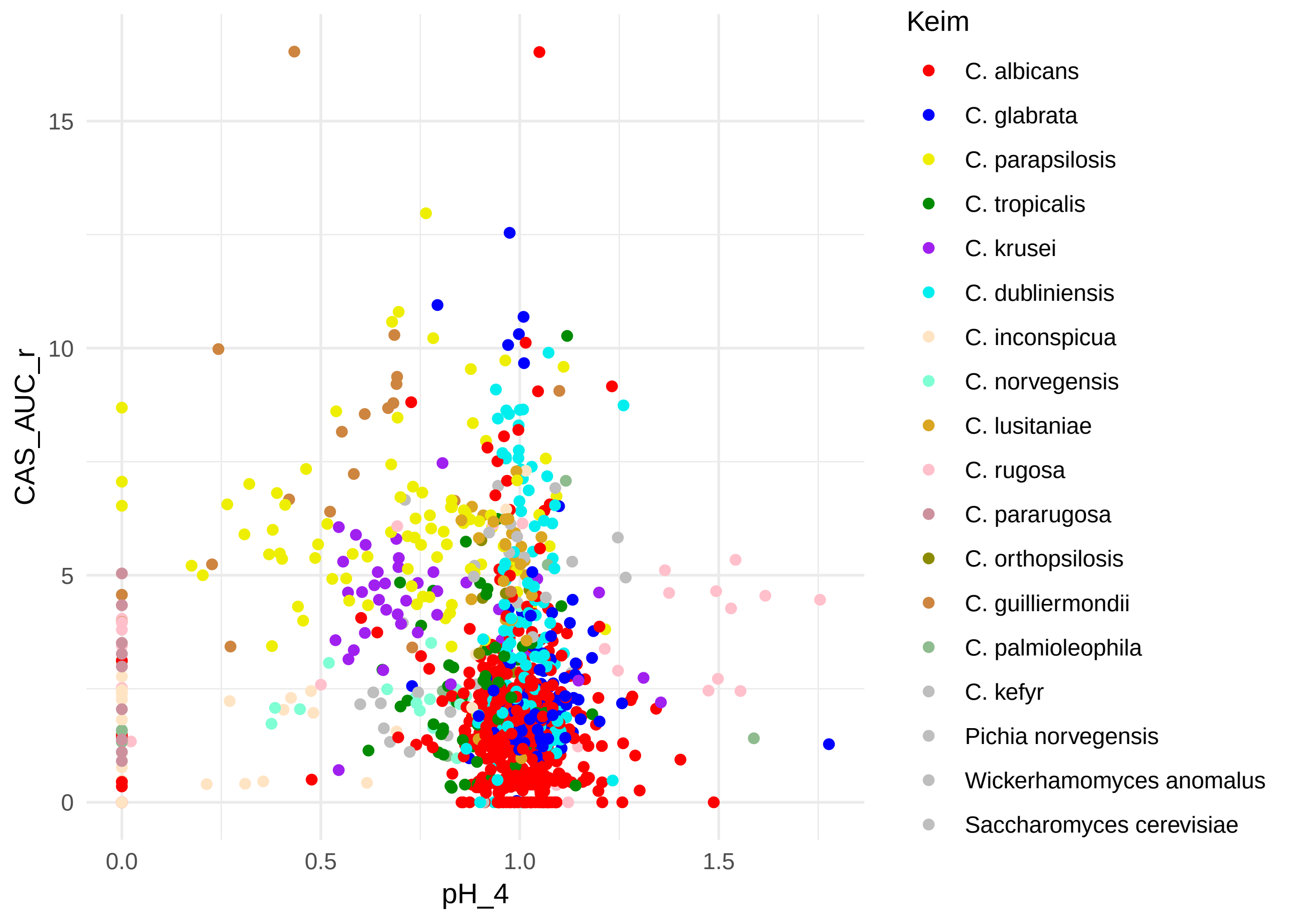

Keim
C. albicans
C. glabrata
C. parapsilosis
C. tropicalis
C. krusei
C. dubliniensis
C. inconspicua
C. norvegensis
C. lusitaniae
C. rugosa
C. pararugosa
C. orthopsilosis
C. guilliermondii
C. palmioleophila
C. kefyr
Pichia norvegensis
Wickerhamomyces anomalus
Saccharomyces cerevisiae
CAS_AUC_r
0
5
10
15
pH_4
0.0
0.5
1.0
1.5

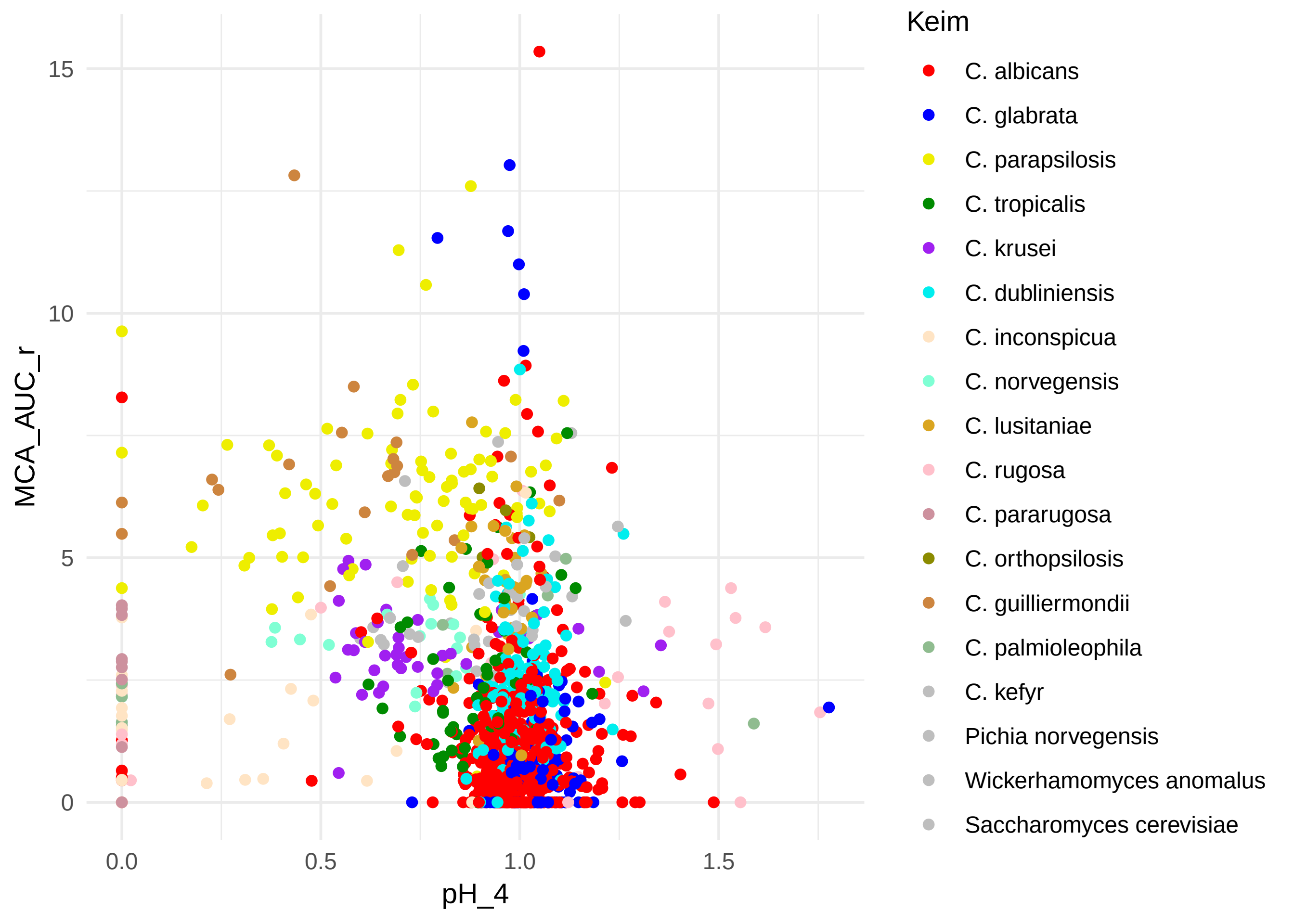

MCA_AUC_r
pH_4
0
5
10
15
0.0
0.5
1.0
1.5
Keim
C. albicans
C. glabrata
C. parapsilosis
C. tropicalis
C. krusei
C. dubliniensis
C. inconspicua
C. norvegensis
C. lusitaniae
C. rugosa
C. pararugosa
C. orthopsilosis
C. guilliermondii
C. palmioleophila
C. kefyr
Pichia norvegensis
Wickerhamomyces anomalus
Saccharomyces cerevisiae

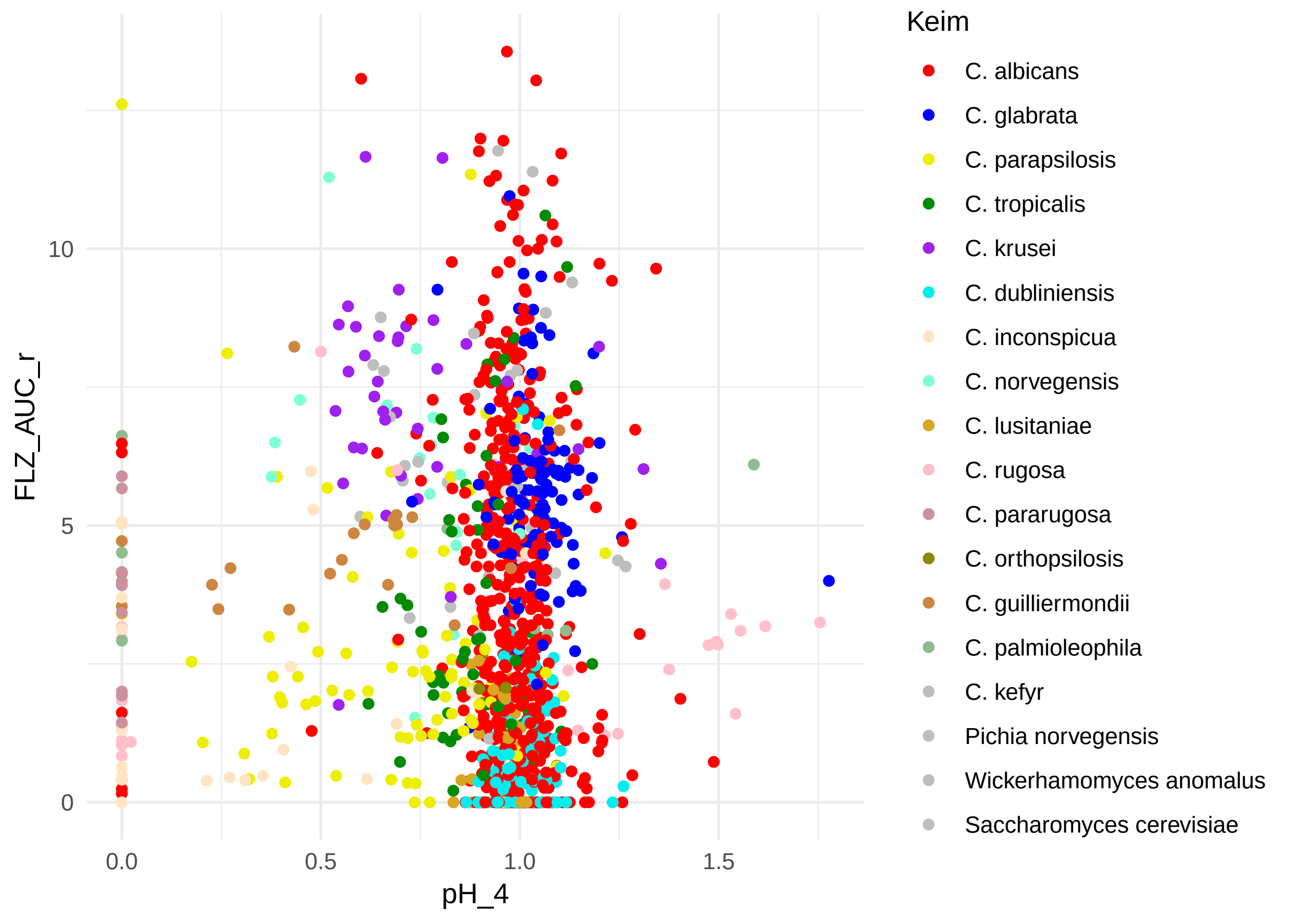
FLZ_AUC_r
10
5
0
0.0
0.5
1.0
1.5
pH_4
Keim
C. albicans
C. glabrata
C. parapsilosis
C. tropicalis
C. krusei
C. dubliniensis
C. inconspicua
C. norvegensis
C. lusitaniae
C. rugosa
C. pararugosa
C. orthopsilosis
C. guilliermondii
C. palmioleophila
C. kefyr
Pichia norvegensis
Wickerhamomyces anomalus
Saccharomyces cerevisiae

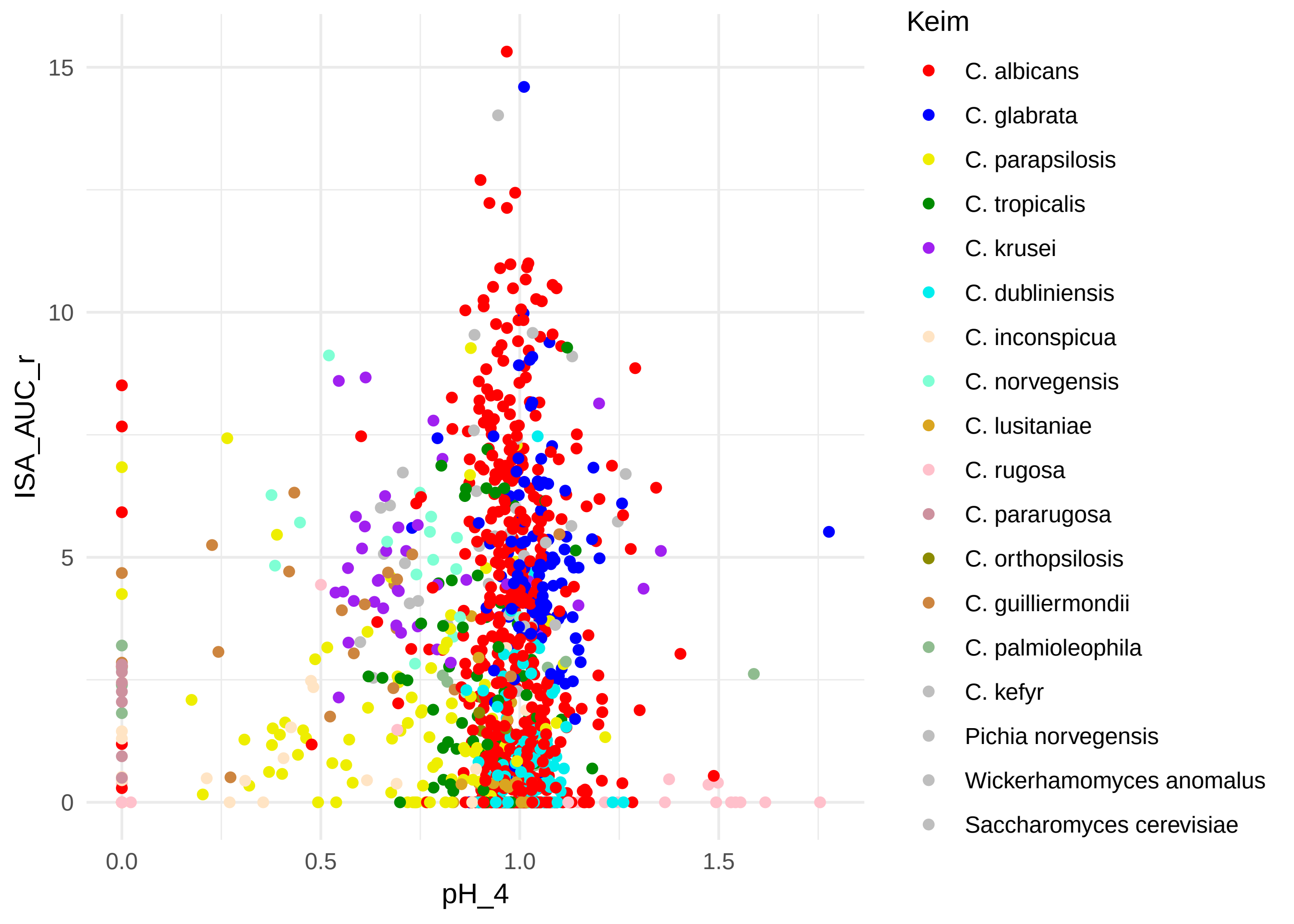

Keim
C. albicans
C. glabrata
C. parapsilosis
C. tropicalis
C. krusei
C. dubliniensis
C. inconspicua
C. norvegensis
C. lusitaniae
C. rugosa
C. pararugosa
C. orthopsilosis
C. guilliermondii
C. palmioleophila
C. kefyr
Pichia norvegensis
Wickerhamomyces anomalus
Saccharomyces cerevisiae
ISA_AUC_r
pH_4
0
5
10
15
0.0
0.5
1.0
1.5

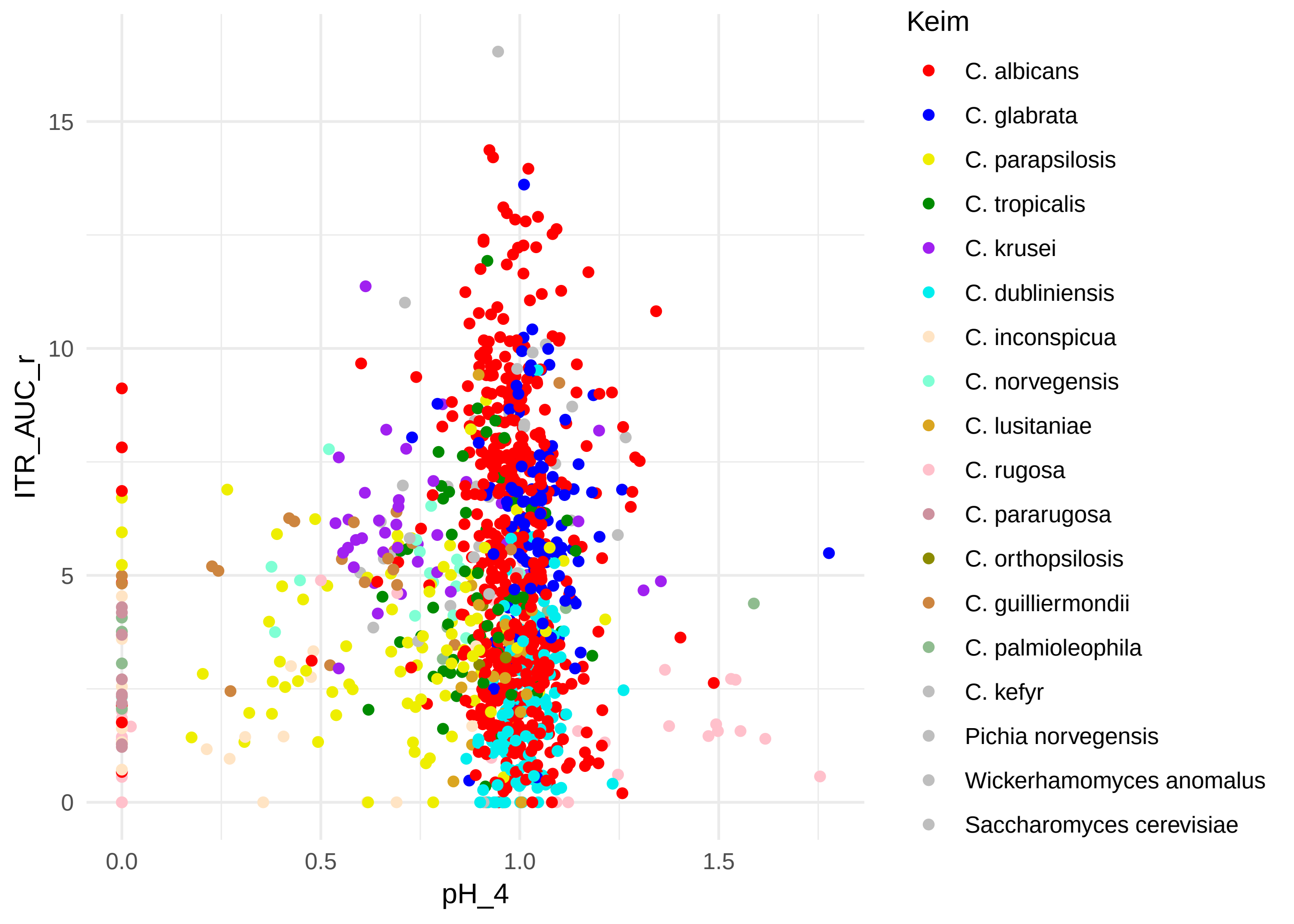

Keim
C. albicans
C. glabrata
C. parapsilosis
C. tropicalis
C. krusei
C. dubliniensis
C. inconspicua
C. norvegensis
C. lusitaniae
C. rugosa
C. pararugosa
C. orthopsilosis
C. guilliermondii
C. palmioleophila
C. kefyr
Pichia norvegensis
Wickerhamomyces anomalus
Saccharomyces cerevisiae
ITR_AUC_r
pH_4
0
5
10
15
0.0
0.5
1.0
1.5

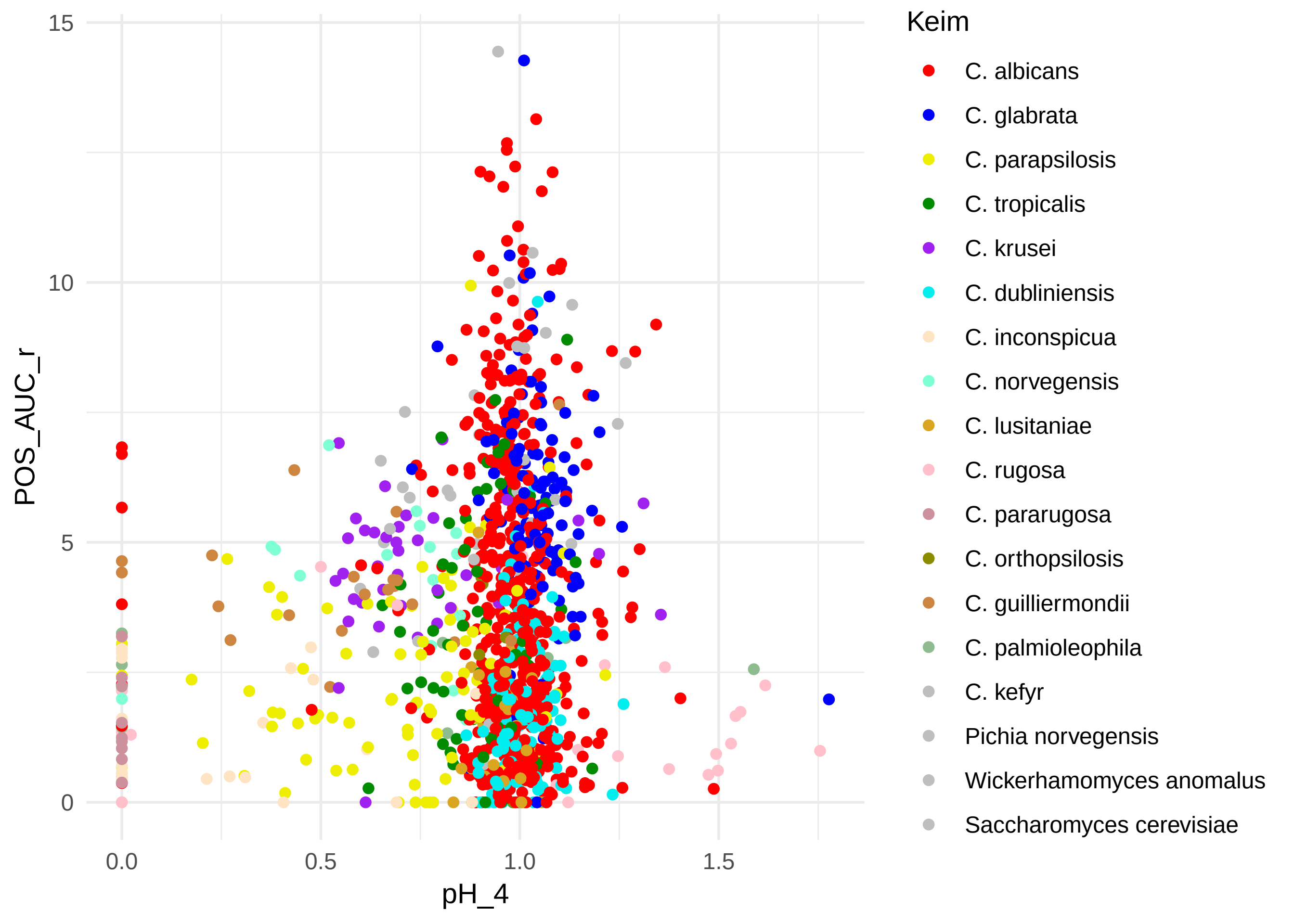

Keim
C. albicans
C. glabrata
C. parapsilosis
C. tropicalis
C. krusei
C. dubliniensis
C. inconspicua
C. norvegensis
C. lusitaniae
C. rugosa
C. pararugosa
C. orthopsilosis
C. guilliermondii
C. palmioleophila
C. kefyr
Pichia norvegensis
Wickerhamomyces anomalus
Saccharomyces cerevisiae
POS_AUC_r
pH_4
0
5
10
15
0.0
0.5
1.0
1.5

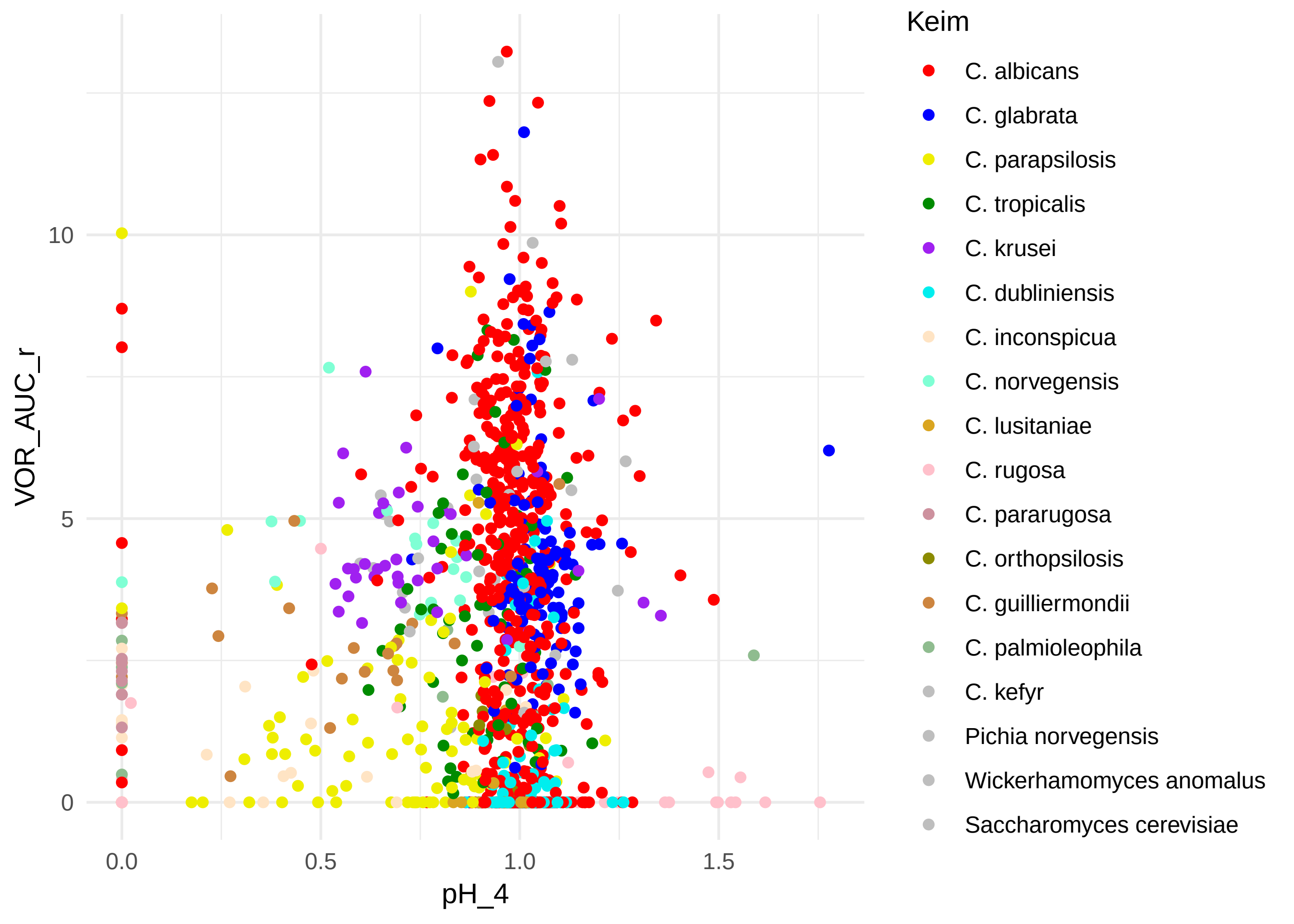
Keim
C. albicans
C. glabrata
C. parapsilosis
C. tropicalis
C. krusei
C. dubliniensis
C. inconspicua
C. norvegensis
C. lusitaniae
C. rugosa
C. pararugosa
C. orthopsilosis
C. guilliermondii
C. palmioleophila
C. kefyr
Pichia norvegensis
Wickerhamomyces anomalus
Saccharomyces cerevisiae
VOR_AUC_r
10
5
0
0.0
0.5
1.0
1.5
pH_4

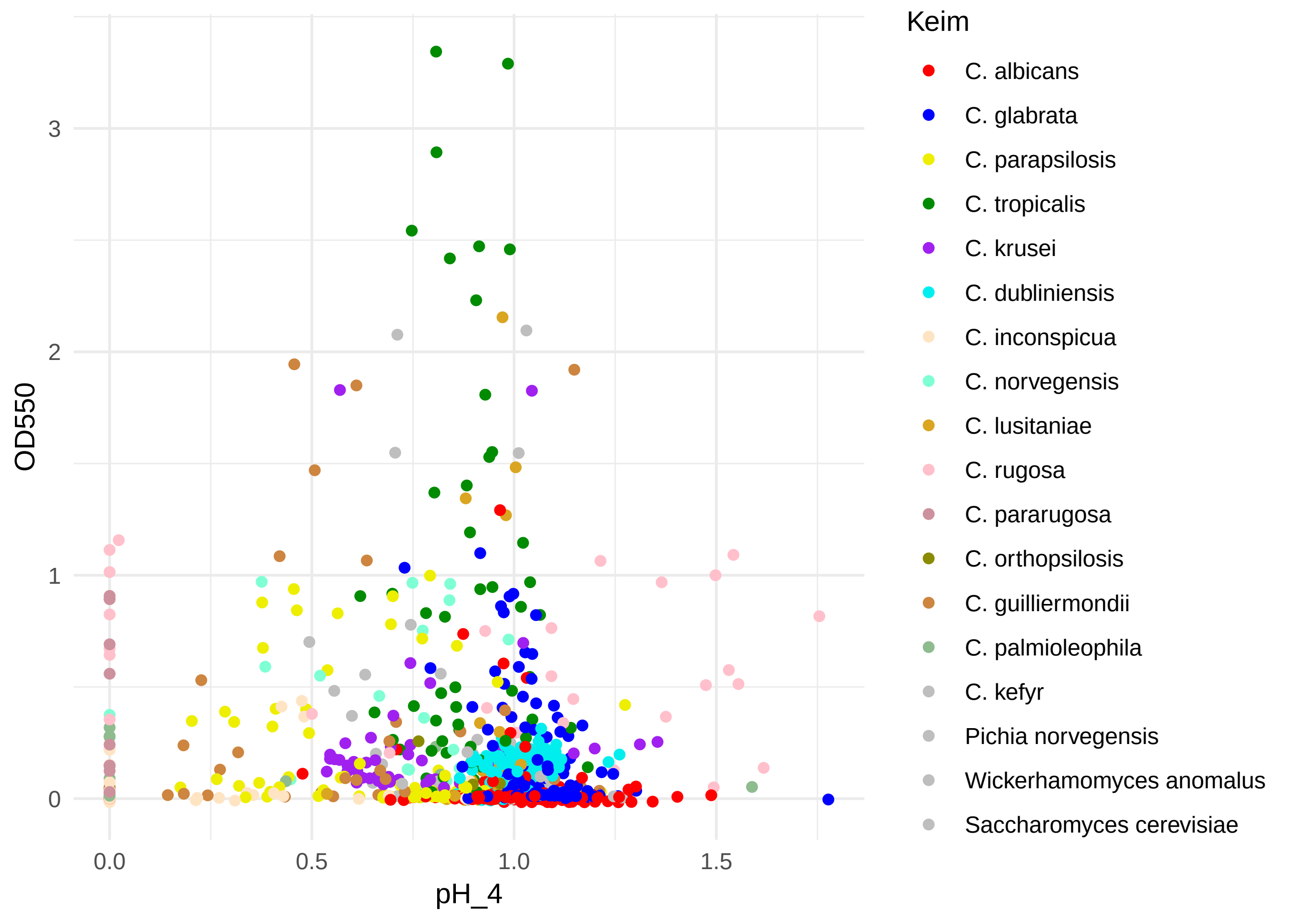

Keim
C. albicans
C. glabrata
C. parapsilosis
C. tropicalis
C. krusei
C. dubliniensis
C. inconspicua
C. norvegensis
C. lusitaniae
C. rugosa
C. pararugosa
C. orthopsilosis
C. guilliermondii
C. palmioleophila
C. kefyr
Pichia norvegensis
Wickerhamomyces anomalus
Saccharomyces cerevisiae
OD550
pH_4
0
1
2
3
0.0
0.5
1.0
1.5

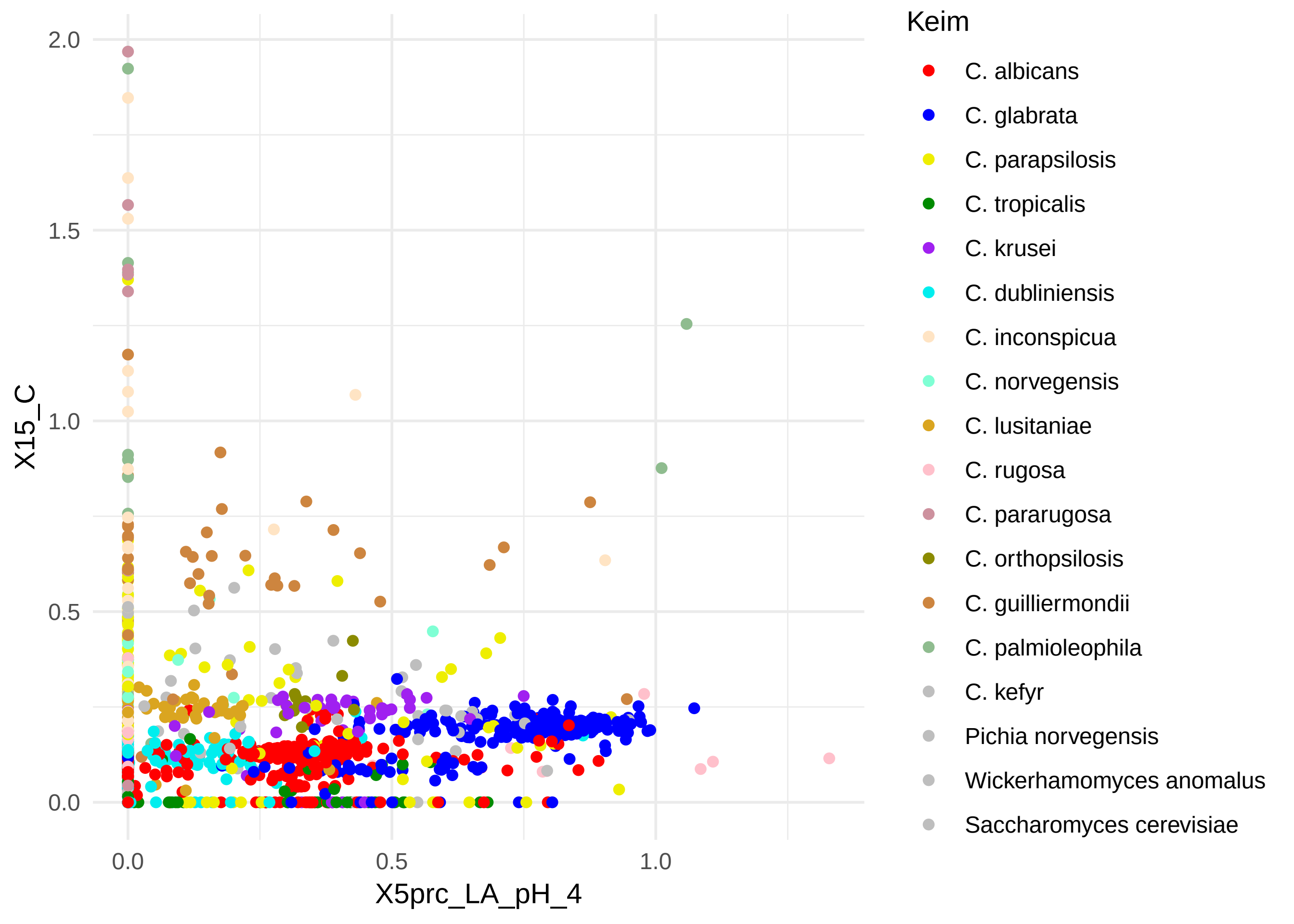
Keim
C. albicans
C. glabrata
C. parapsilosis
C. tropicalis
C. krusei
C. dubliniensis
C. inconspicua
C. norvegensis
C. lusitaniae
C. rugosa
C. pararugosa
C. orthopsilosis
C. guilliermondii
C. palmioleophila
C. kefyr
Pichia norvegensis
Wickerhamomyces anomalus
Saccharomyces cerevisiae
X15_C
X5prc_LA_pH_4
0.0
0.5
1.0
1.5
2.0
0.0
0.5
1.0

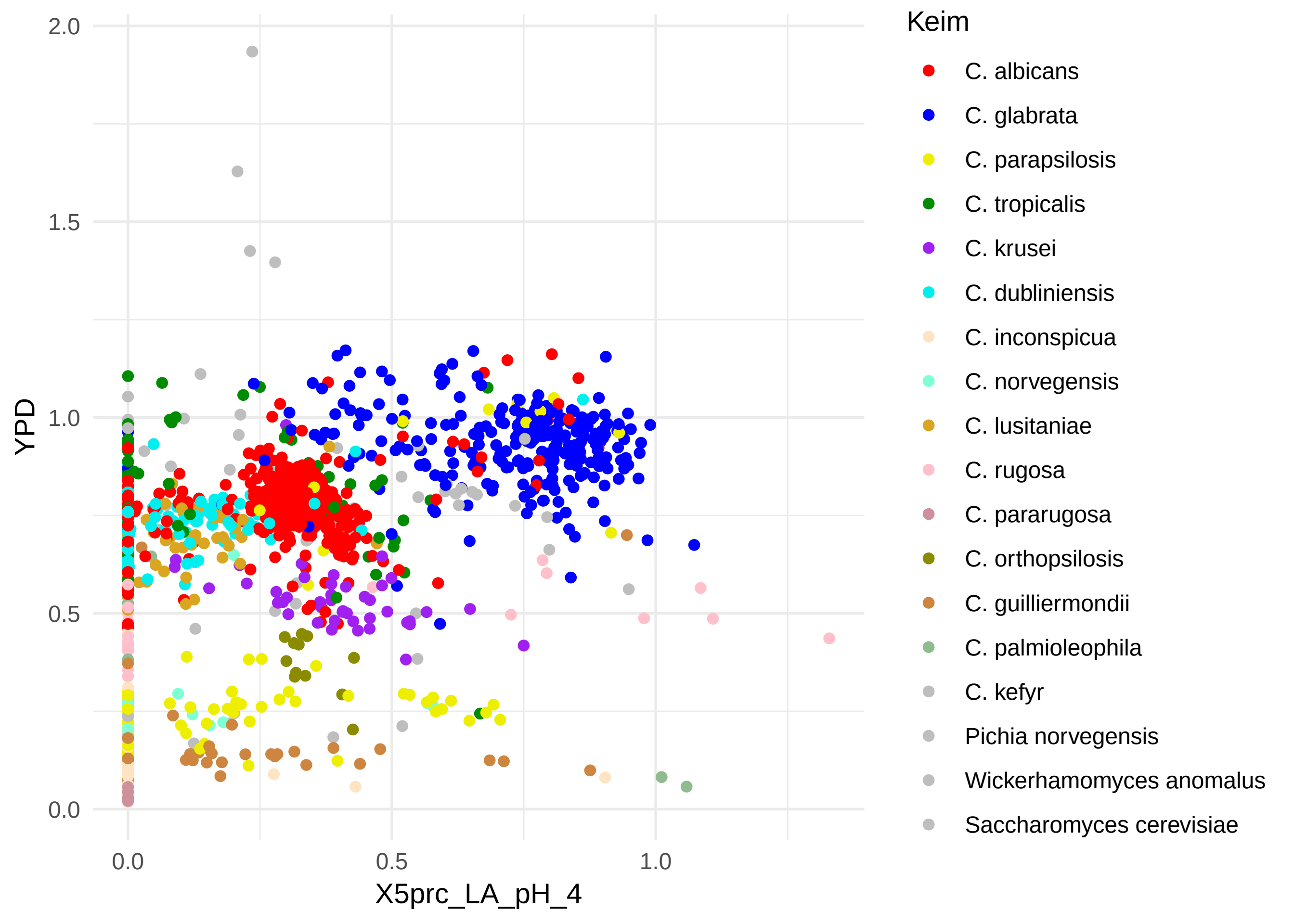
Keim
C. albicans
C. glabrata
C. parapsilosis
C. tropicalis
C. krusei
C. dubliniensis
C. inconspicua
C. norvegensis
C. lusitaniae
C. rugosa
C. pararugosa
C. orthopsilosis
C. guilliermondii
C. palmioleophila
C. kefyr
Pichia norvegensis
Wickerhamomyces anomalus
Saccharomyces cerevisiae
YPD
2.0
1.5
1.0
0.5
0.0
X5prc_LA_pH_4
0.0
0.5
1.0

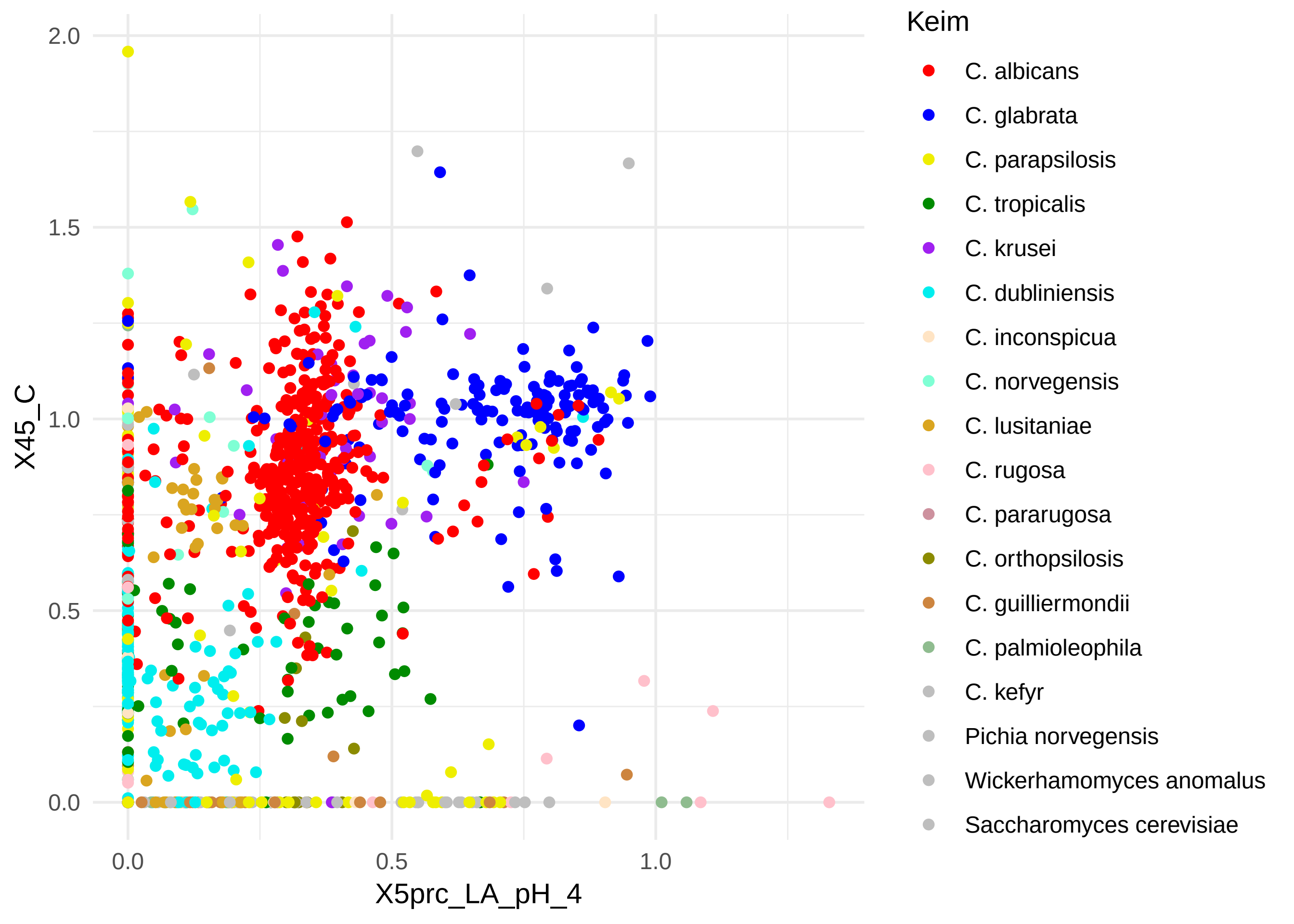
Keim
C. albicans
C. glabrata
C. parapsilosis
C. tropicalis
C. krusei
C. dubliniensis
C. inconspicua
C. norvegensis
C. lusitaniae
C. rugosa
C. pararugosa
C. orthopsilosis
C. guilliermondii
C. palmioleophila
C. kefyr
Pichia norvegensis
Wickerhamomyces anomalus
Saccharomyces cerevisiae
X45_C
2.0
1.5
1.0
0.5
0.0
0.0
0.5
1.0
X5prc_LA_pH_4

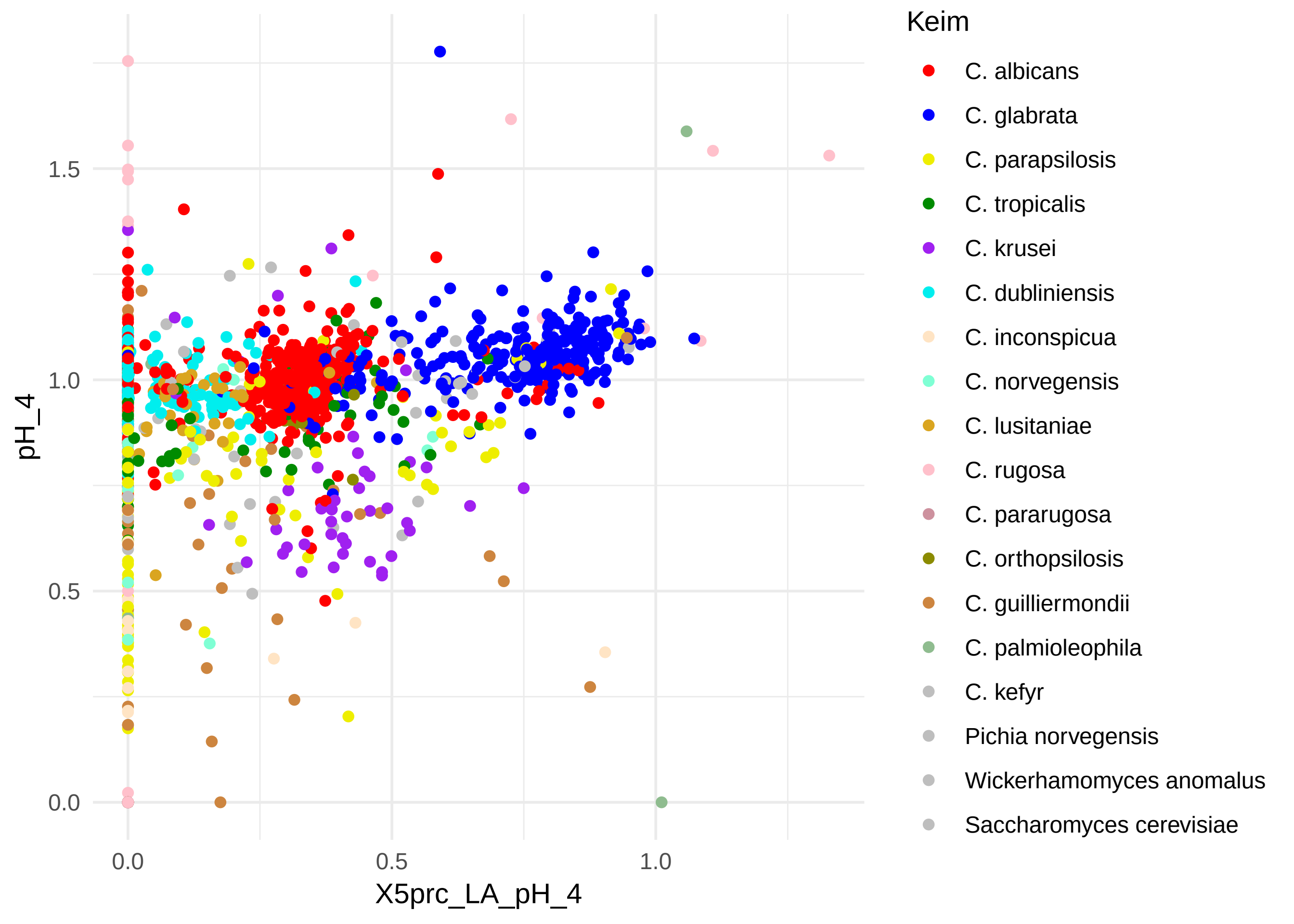

Keim
C. albicans
C. glabrata
C. parapsilosis
C. tropicalis
C. krusei
C. dubliniensis
C. inconspicua
C. norvegensis
C. lusitaniae
C. rugosa
C. pararugosa
C. orthopsilosis
C. guilliermondii
C. palmioleophila
C. kefyr
Pichia norvegensis
Wickerhamomyces anomalus
Saccharomyces cerevisiae
pH_4
X5prc_LA_pH_4
0.0
0.5
1.0
1.5

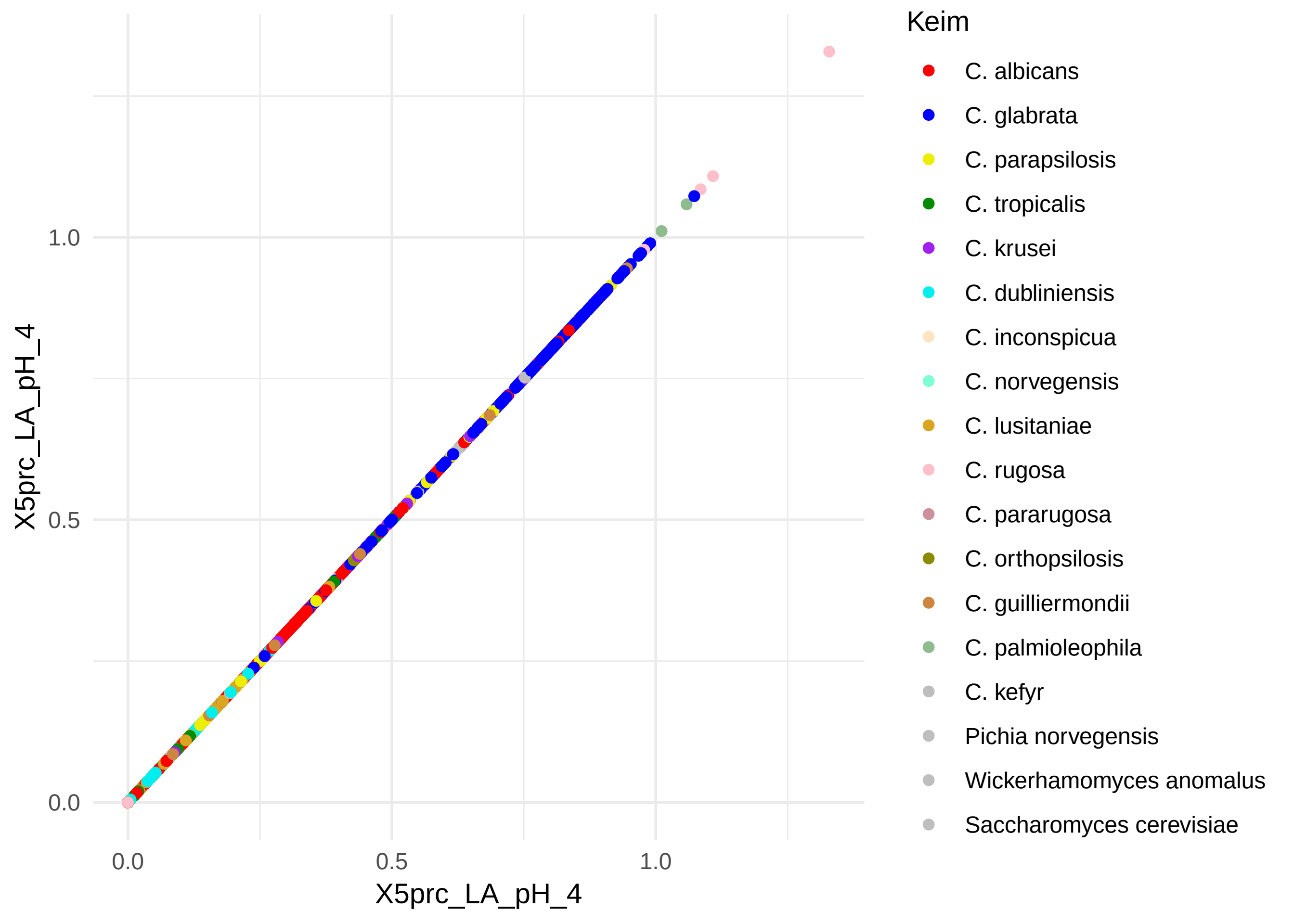

Keim
C. albicans
C. glabrata
C. parapsilosis
C. tropicalis
C. krusei
C. dubliniensis
C. inconspicua
C. norvegensis
C. lusitaniae
C. rugosa
C. pararugosa
C. orthopsilosis
C. guilliermondii
C. palmioleophila
C. kefyr
Pichia norvegensis
Wickerhamomyces anomalus
Saccharomyces cerevisiae
X5prc_LA_pH_4
X5prc_LA_pH_4
0.0
0.5
1.0
0.0
0.5
1.0

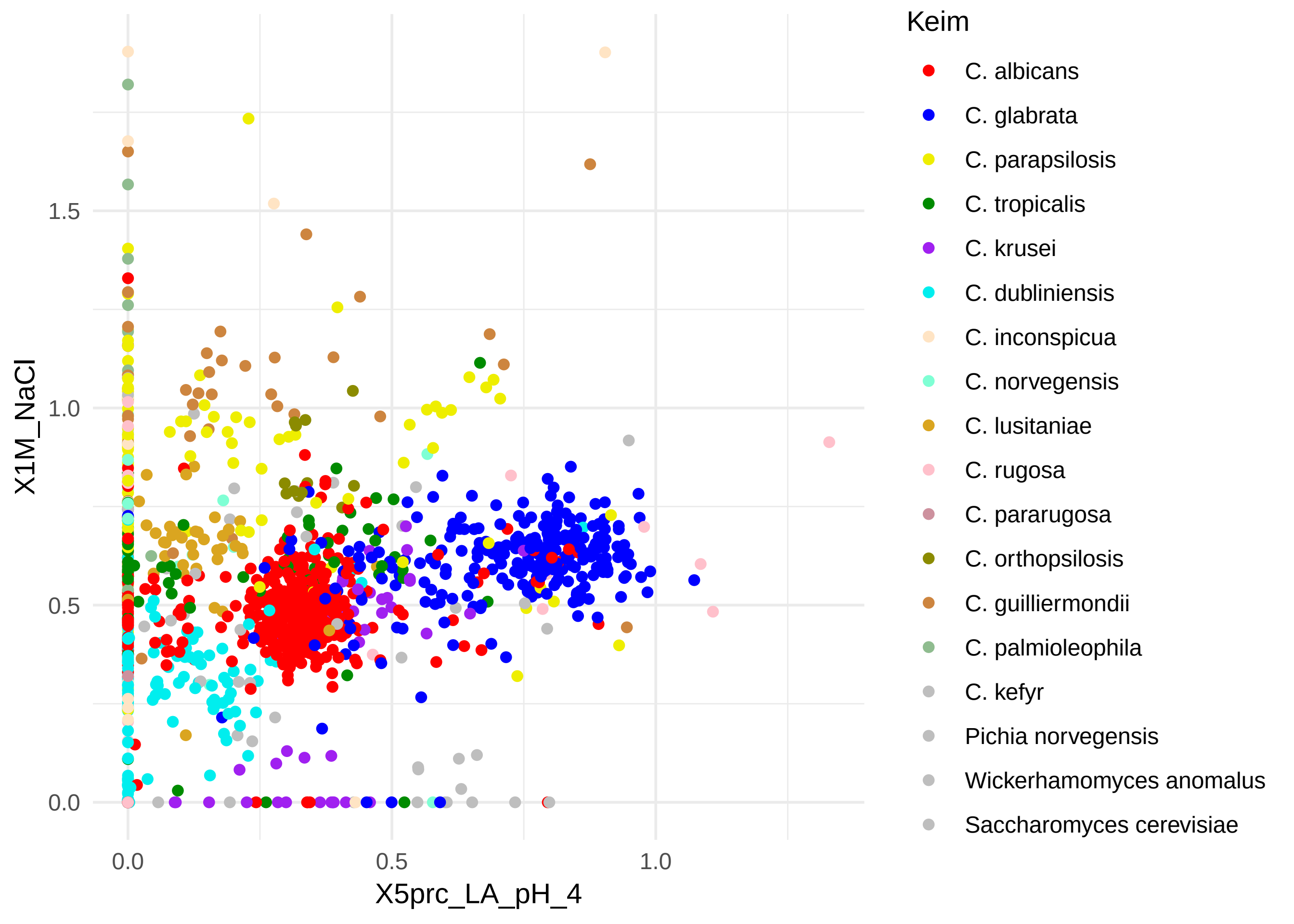
Keim
C. albicans
C. glabrata
C. parapsilosis
C. tropicalis
C. krusei
C. dubliniensis
C. inconspicua
C. norvegensis
C. lusitaniae
C. rugosa
C. pararugosa
C. orthopsilosis
C. guilliermondii
C. palmioleophila
C. kefyr
Pichia norvegensis
Wickerhamomyces anomalus
Saccharomyces cerevisiae
X1M_NaCl
1.5
1.0
0.5
0.0
0.0
0.5
1.0
X5prc_LA_pH_4

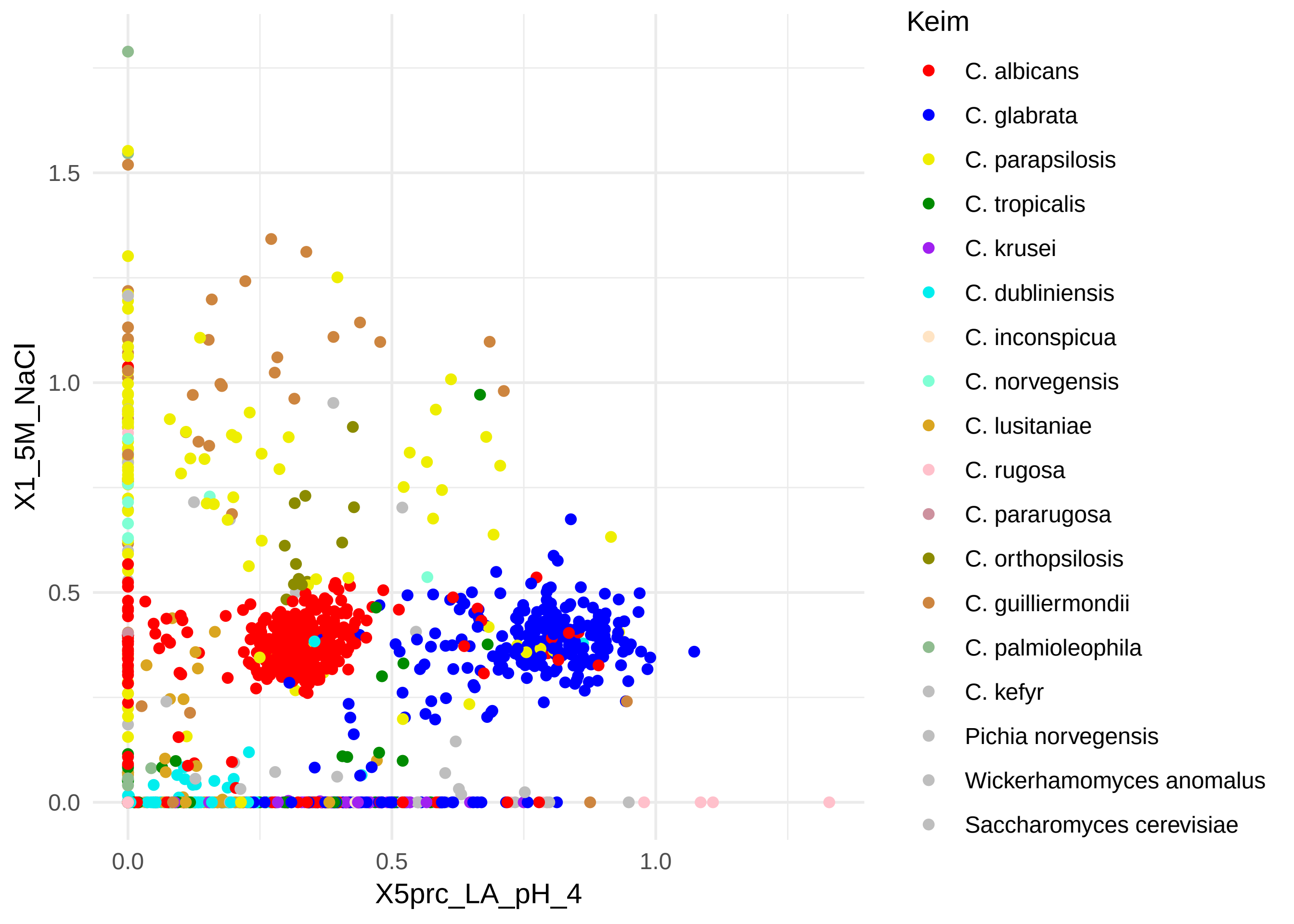

Keim
C. albicans
C. glabrata
C. parapsilosis
C. tropicalis
C. krusei
C. dubliniensis
C. inconspicua
C. norvegensis
C. lusitaniae
C. rugosa
C. pararugosa
C. orthopsilosis
C. guilliermondii
C. palmioleophila
C. kefyr
Pichia norvegensis
Wickerhamomyces anomalus
Saccharomyces cerevisiae
X1_5M_NaCl
X5prc_LA_pH_4
0.0
0.5
1.0
1.5
0.0
0.5
1.0

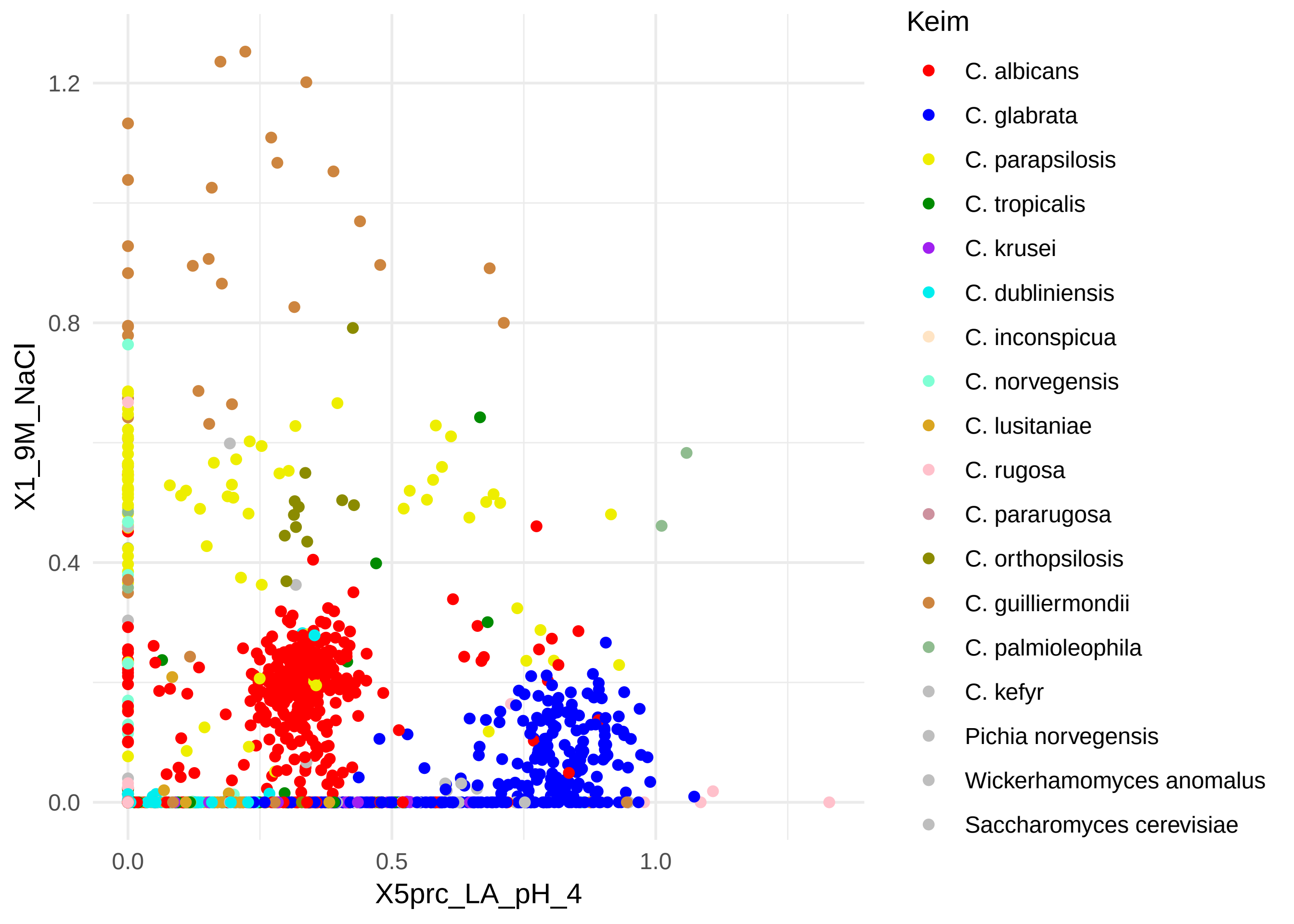

Keim
C. albicans
C. glabrata
C. parapsilosis
C. tropicalis
C. krusei
C. dubliniensis
C. inconspicua
C. norvegensis
C. lusitaniae
C. rugosa
C. pararugosa
C. orthopsilosis
C. guilliermondii
C. palmioleophila
C. kefyr
Pichia norvegensis
Wickerhamomyces anomalus
Saccharomyces cerevisiae
1.2
0.8
0.4
0.0
0.0
0.5
1.0
X1_9M_NaCl
X5prc_LA_pH_4

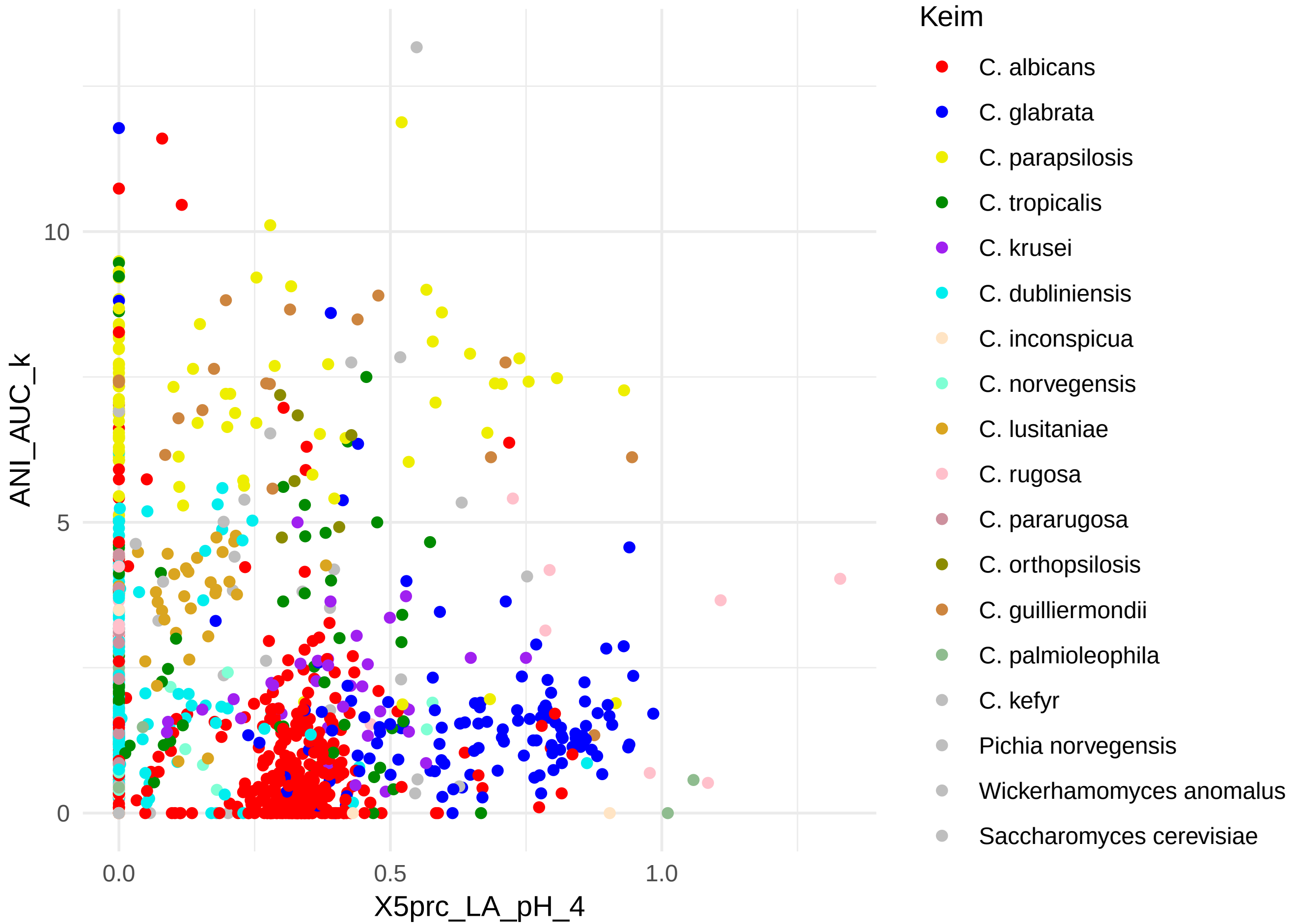
Keim
C. albicans
C. glabrata
C. parapsilosis
C. tropicalis
C. krusei
C. dubliniensis
C. inconspicua
C. norvegensis
C. lusitaniae
C. rugosa
C. pararugosa
C. orthopsilosis
C. guilliermondii
C. palmioleophila
C. kefyr
Pichia norvegensis
Wickerhamomyces anomalus
Saccharomyces cerevisiae
ANI_AUC_k
X5prc_LA_pH_4
10
5
0
0.0
0.5
1.0

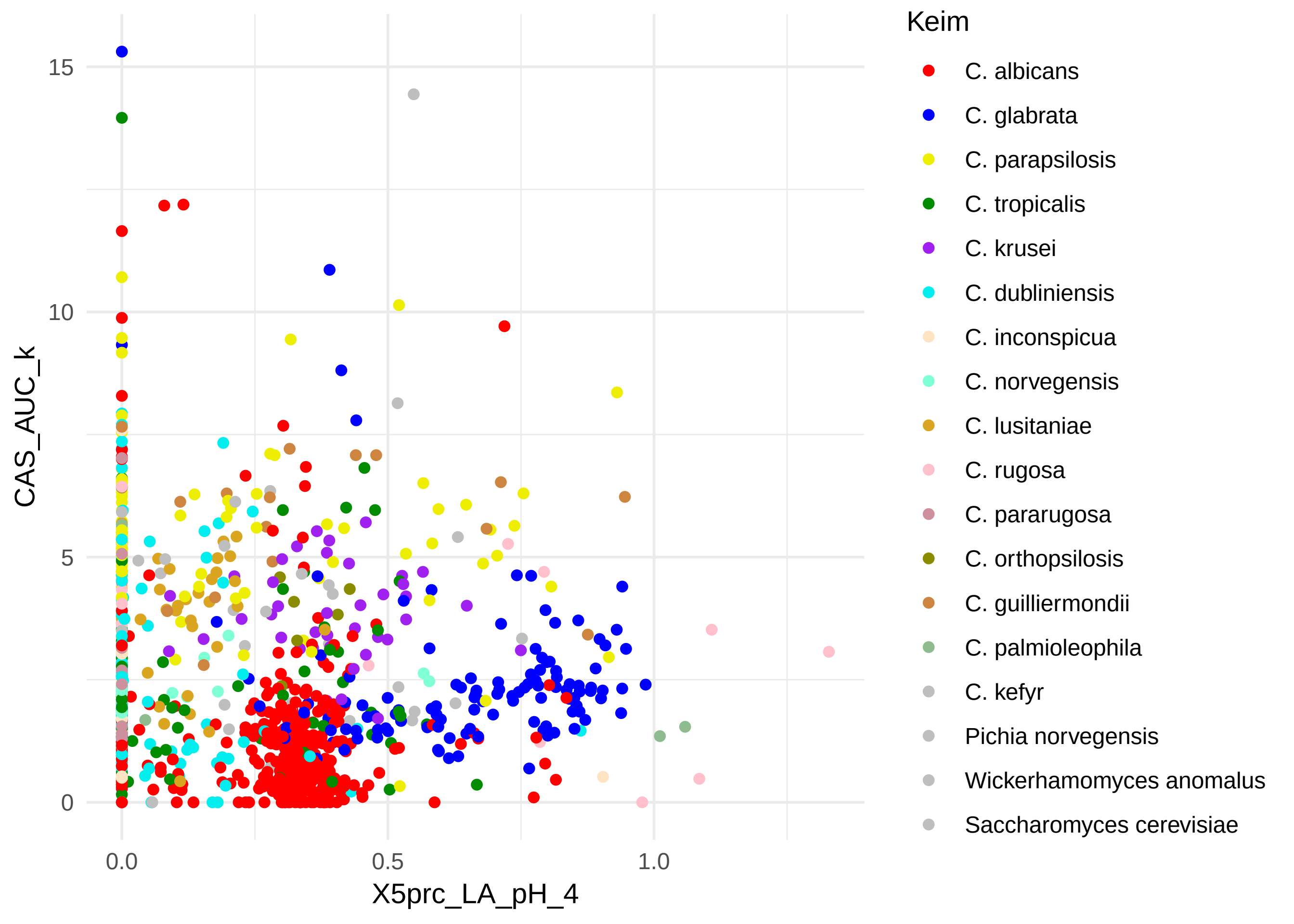

Keim
C. albicans
C. glabrata
C. parapsilosis
C. tropicalis
C. krusei
C. dubliniensis
C. inconspicua
C. norvegensis
C. lusitaniae
C. rugosa
C. pararugosa
C. orthopsilosis
C. guilliermondii
C. palmioleophila
C. kefyr
Pichia norvegensis
Wickerhamomyces anomalus
Saccharomyces cerevisiae
CAS_AUC_k
X5prc_LA_pH_4
0
5
10
15
0.0
0.5
1.0

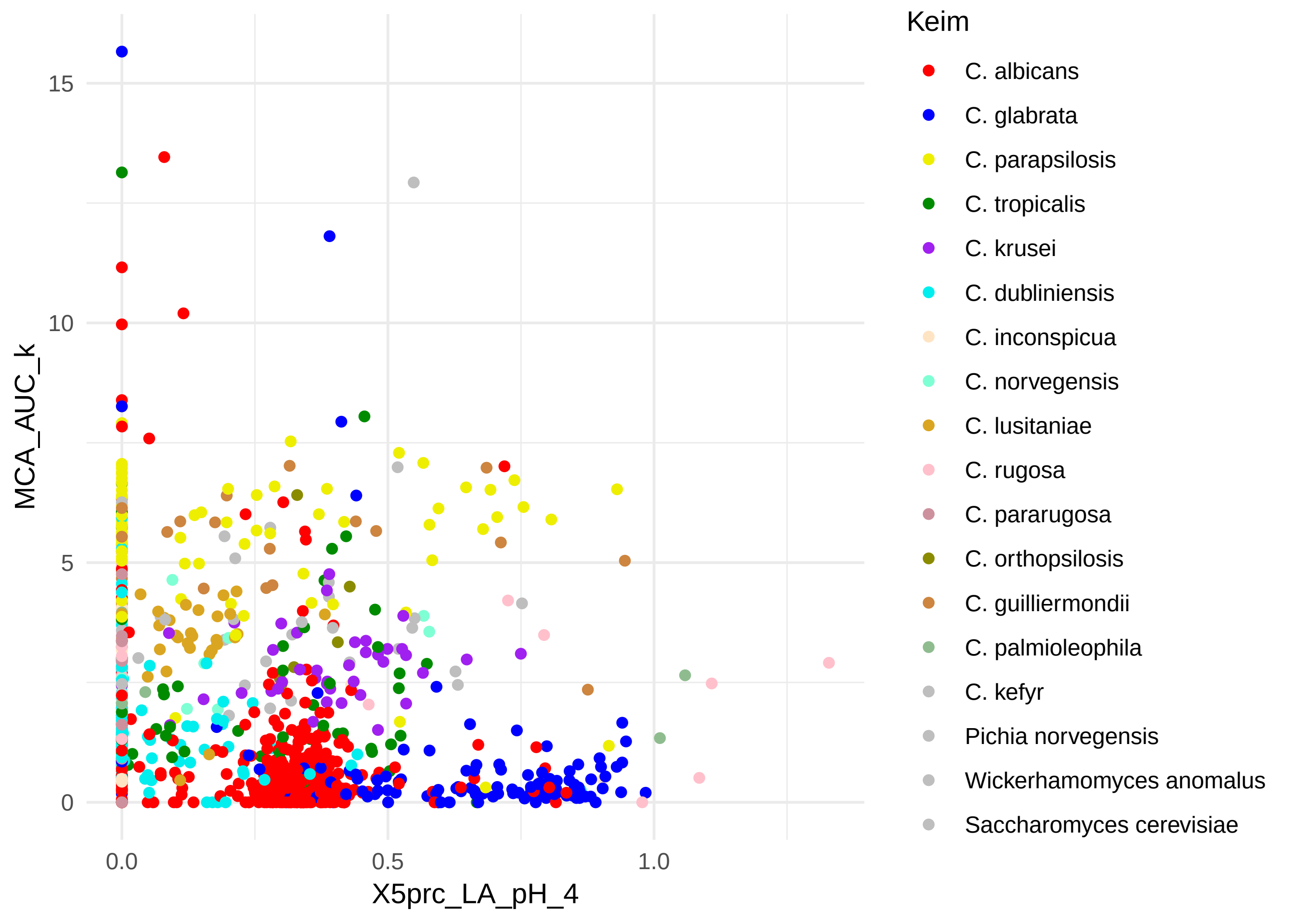
Keim
C. albicans
C. glabrata
C. parapsilosis
C. tropicalis
C. krusei
C. dubliniensis
C. inconspicua
C. norvegensis
C. lusitaniae
C. rugosa
C. pararugosa
C. orthopsilosis
C. guilliermondii
C. palmioleophila
C. kefyr
Pichia norvegensis
Wickerhamomyces anomalus
Saccharomyces cerevisiae
MCA_AUC_k
X5prc_LA_pH_4
0
5
10
15
0.0
0.5
1.0

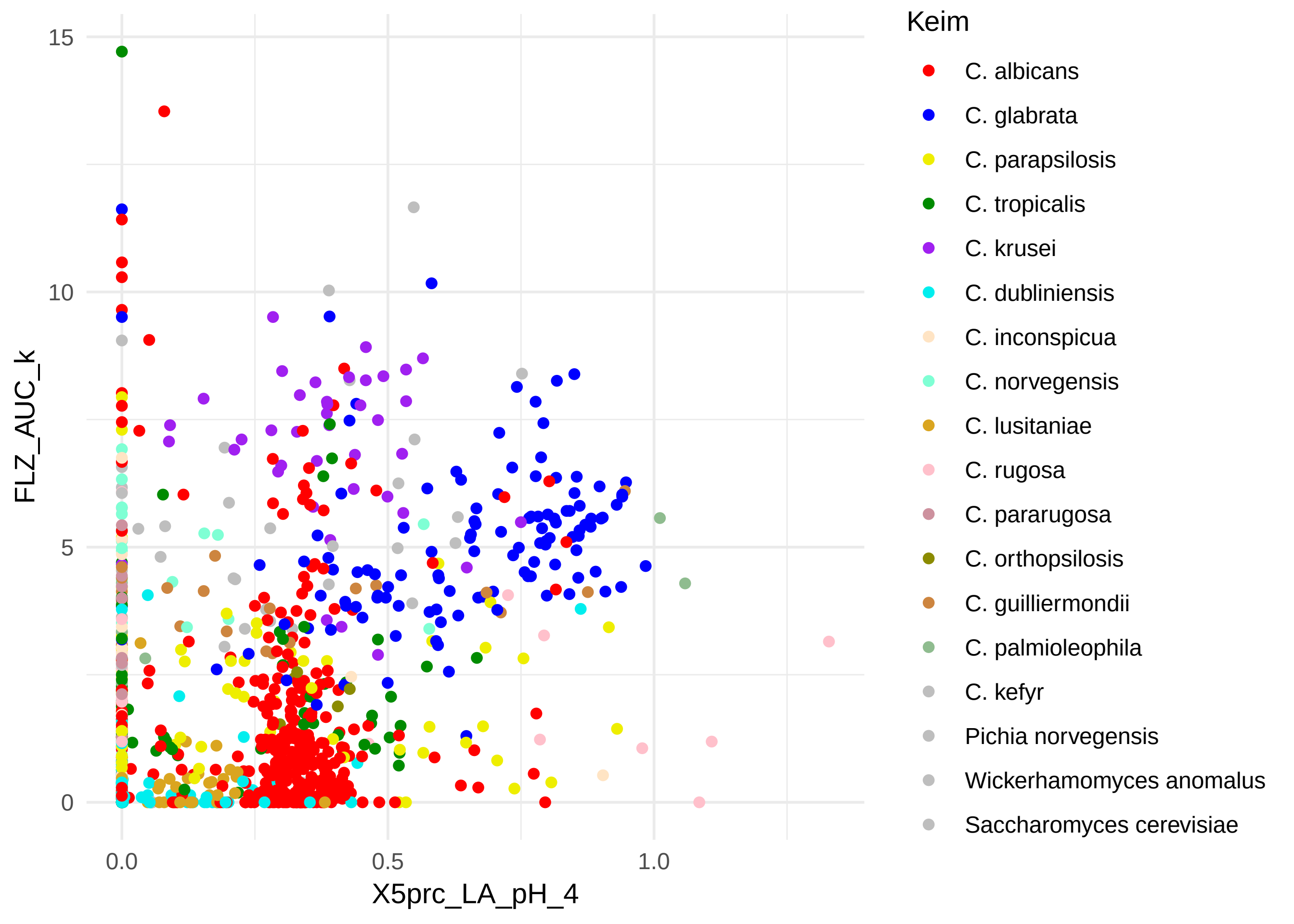

Keim
C. albicans
C. glabrata
C. parapsilosis
C. tropicalis
C. krusei
C. dubliniensis
C. inconspicua
C. norvegensis
C. lusitaniae
C. rugosa
C. pararugosa
C. orthopsilosis
C. guilliermondii
C. palmioleophila
C. kefyr
Pichia norvegensis
Wickerhamomyces anomalus
Saccharomyces cerevisiae
FLZ_AUC_k
X5prc_LA_pH_4
15
10
5
0
0.0
0.5
1.0

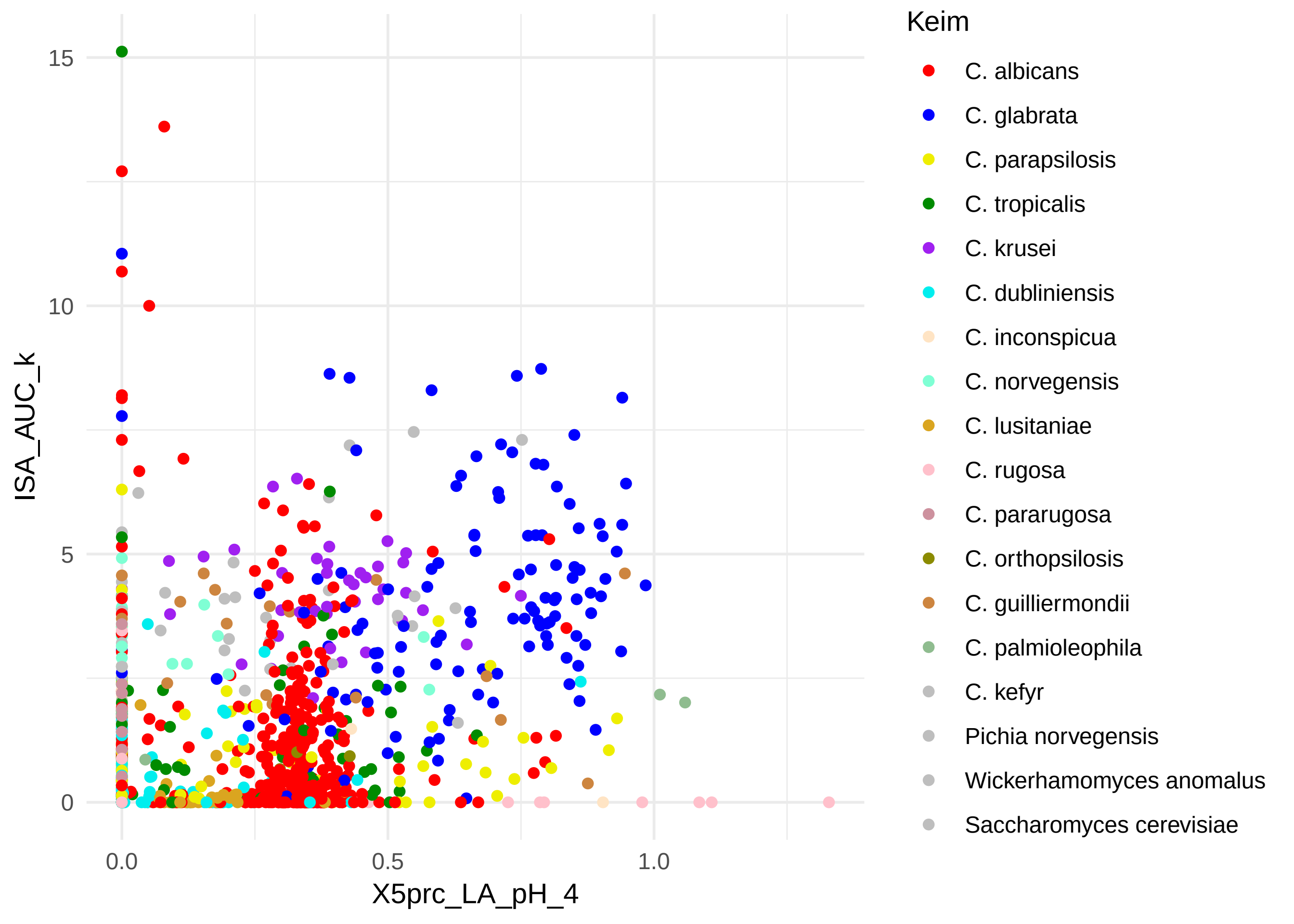

Keim
C. albicans
C. glabrata
C. parapsilosis
C. tropicalis
C. krusei
C. dubliniensis
C. inconspicua
C. norvegensis
C. lusitaniae
C. rugosa
C. pararugosa
C. orthopsilosis
C. guilliermondii
C. palmioleophila
C. kefyr
Pichia norvegensis
Wickerhamomyces anomalus
Saccharomyces cerevisiae
ISA_AUC_k
15
10
5
0
0.0
0.5
1.0
X5prc_LA_pH_4

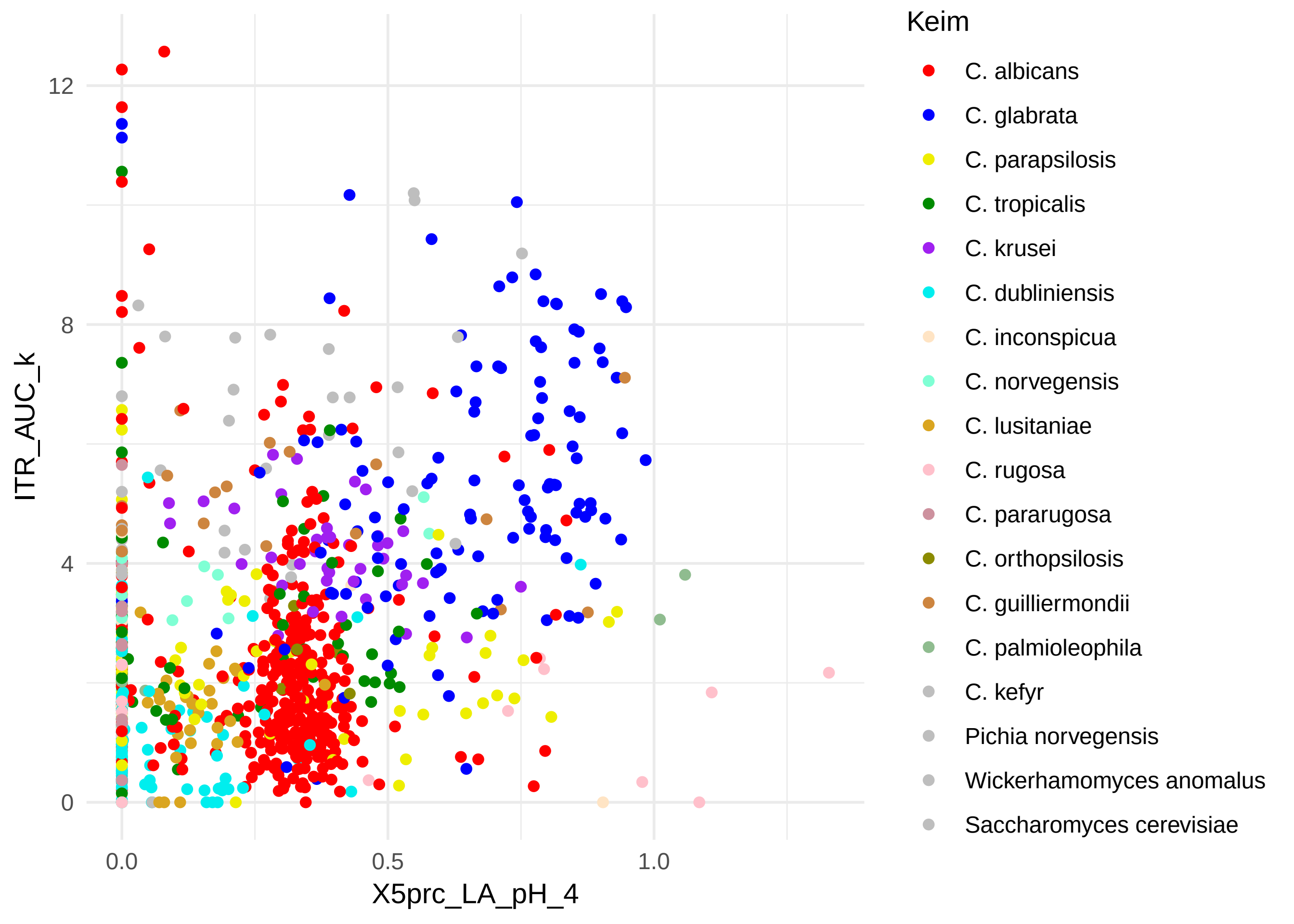

Keim
C. albicans
C. glabrata
C. parapsilosis
C. tropicalis
C. krusei
C. dubliniensis
C. inconspicua
C. norvegensis
C. lusitaniae
C. rugosa
C. pararugosa
C. orthopsilosis
C. guilliermondii
C. palmioleophila
C. kefyr
Pichia norvegensis
Wickerhamomyces anomalus
Saccharomyces cerevisiae
ITR_AUC_k
X5prc_LA_pH_4
0
4
8
12
0.0
0.5
1.0

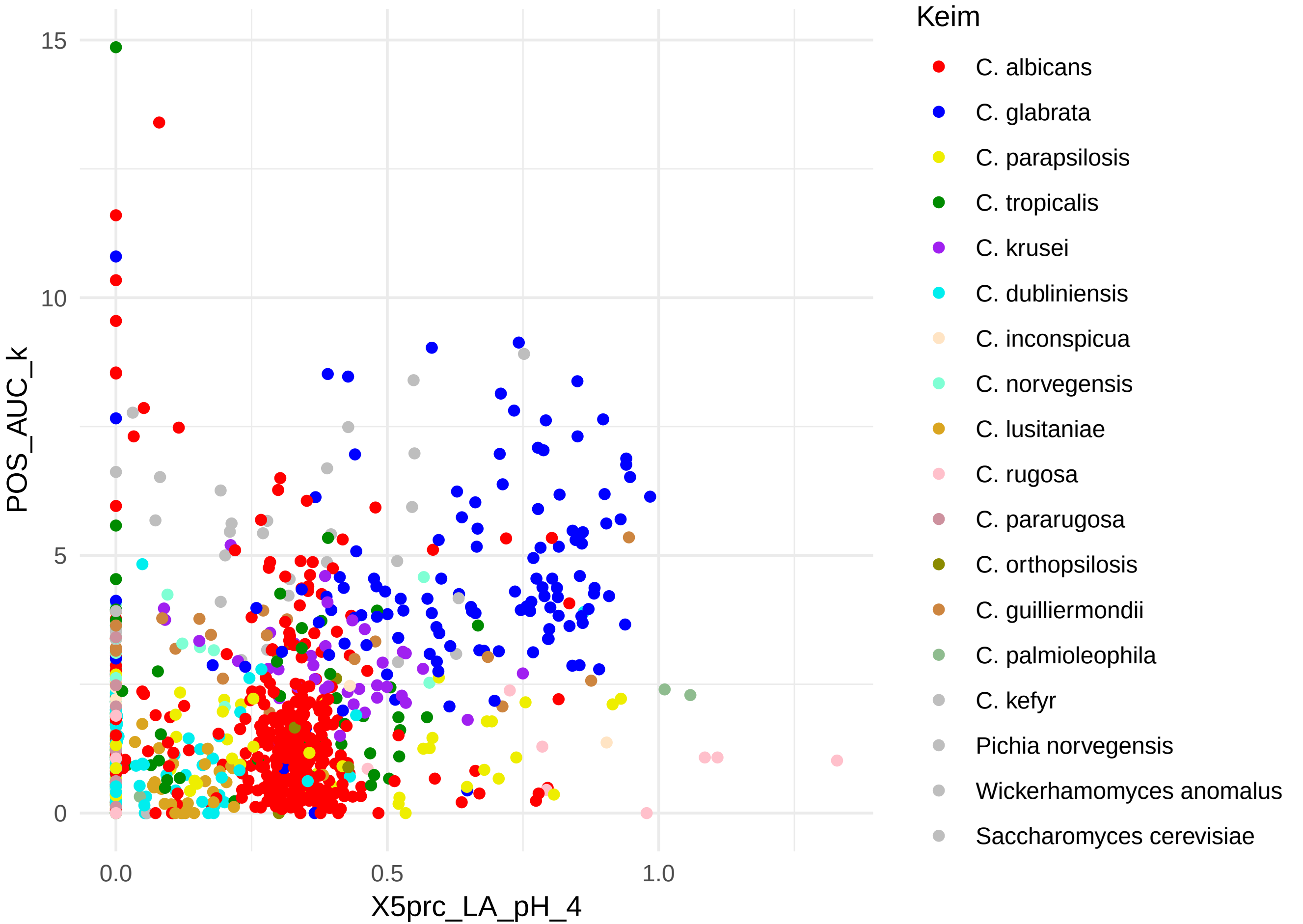

Keim
C. albicans
C. glabrata
C. parapsilosis
C. tropicalis
C. krusei
C. dubliniensis
C. inconspicua
C. norvegensis
C. lusitaniae
C. rugosa
C. pararugosa
C. orthopsilosis
C. guilliermondii
C. palmioleophila
C. kefyr
Pichia norvegensis
Wickerhamomyces anomalus
Saccharomyces cerevisiae
POS_AUC_k
X5prc_LA_pH_4
0
5
10
15
0.0
0.5
1.0

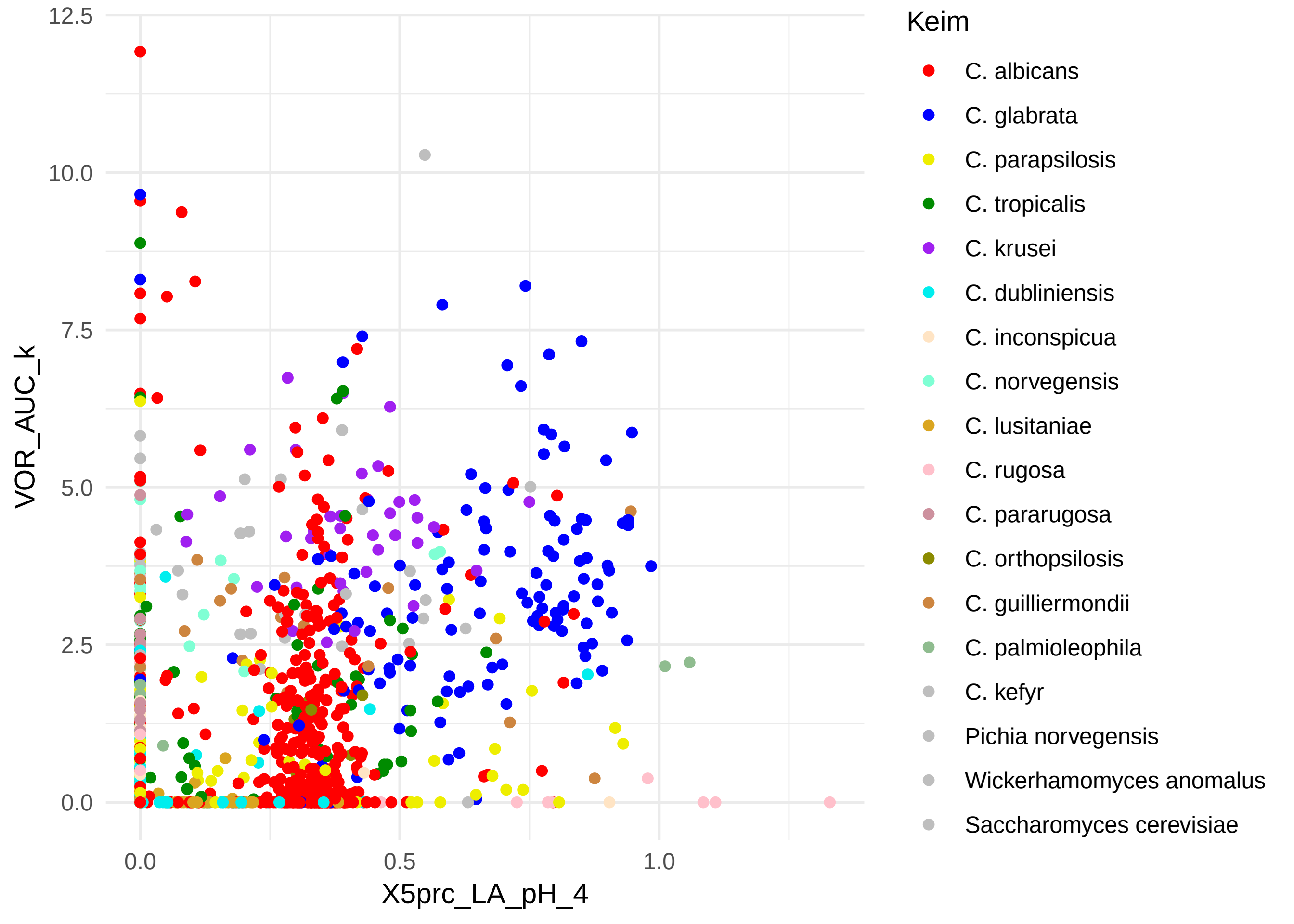

12.5
10.0
7.5
5.0
2.5
0.0
VOR_AUC_k
0.0
0.5
1.0
X5prc_LA_pH_4
Keim
C. albicans
C. glabrata
C. parapsilosis
C. tropicalis
C. krusei
C. dubliniensis
C. inconspicua
C. norvegensis
C. lusitaniae
C. rugosa
C. pararugosa
C. orthopsilosis
C. guilliermondii
C. palmioleophila
C. kefyr
Pichia norvegensis
Wickerhamomyces anomalus
Saccharomyces cerevisiae

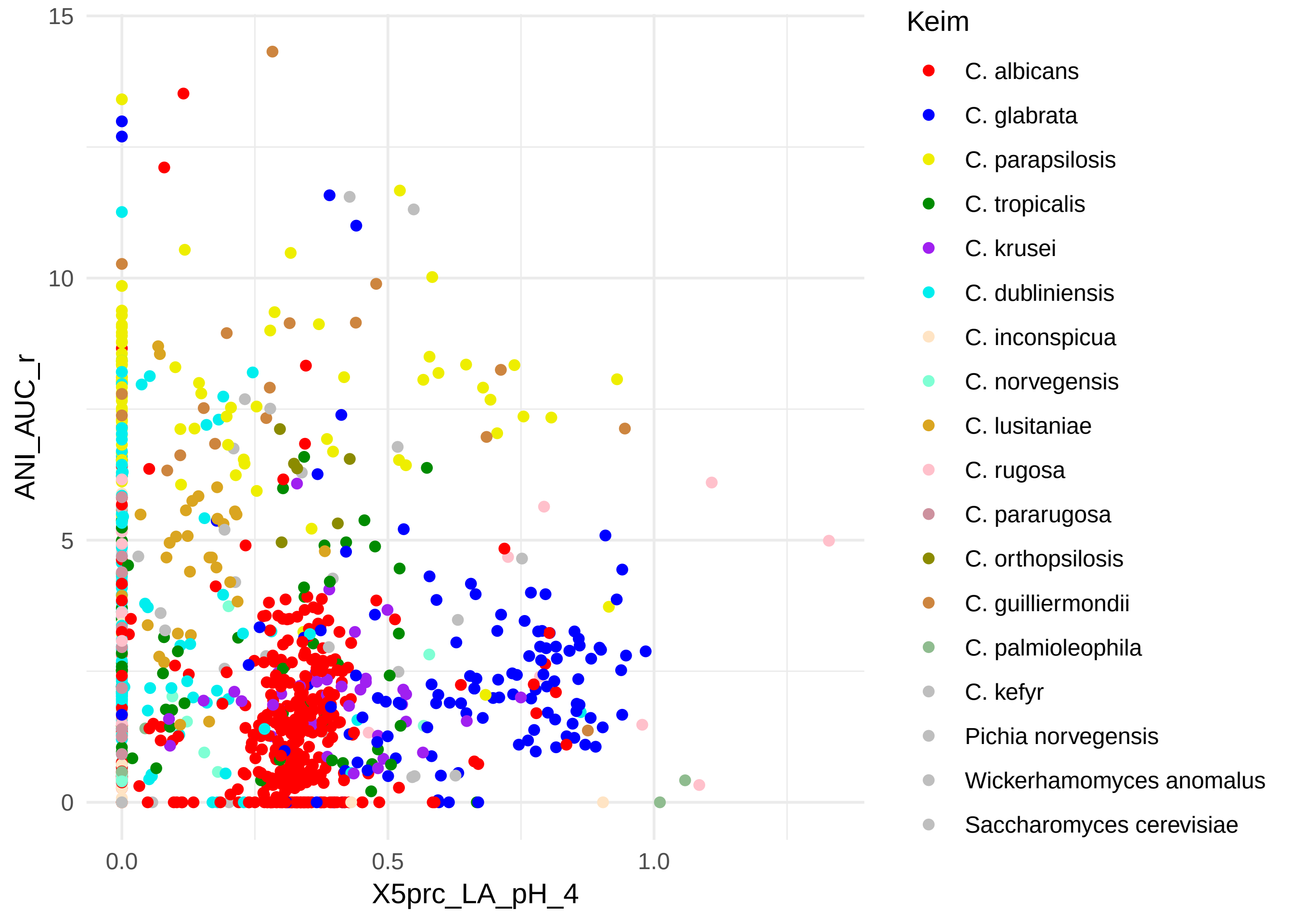

15
10
5
0
0.0
0.5
1.0
ANI_AUC_r
X5prc_LA_pH_4
Keim
C. albicans
C. glabrata
C. parapsilosis
C. tropicalis
C. krusei
C. dubliniensis
C. inconspicua
C. norvegensis
C. lusitaniae
C. rugosa
C. pararugosa
C. orthopsilosis
C. guilliermondii
C. palmioleophila
C. kefyr
Pichia norvegensis
Wickerhamomyces anomalus
Saccharomyces cerevisiae

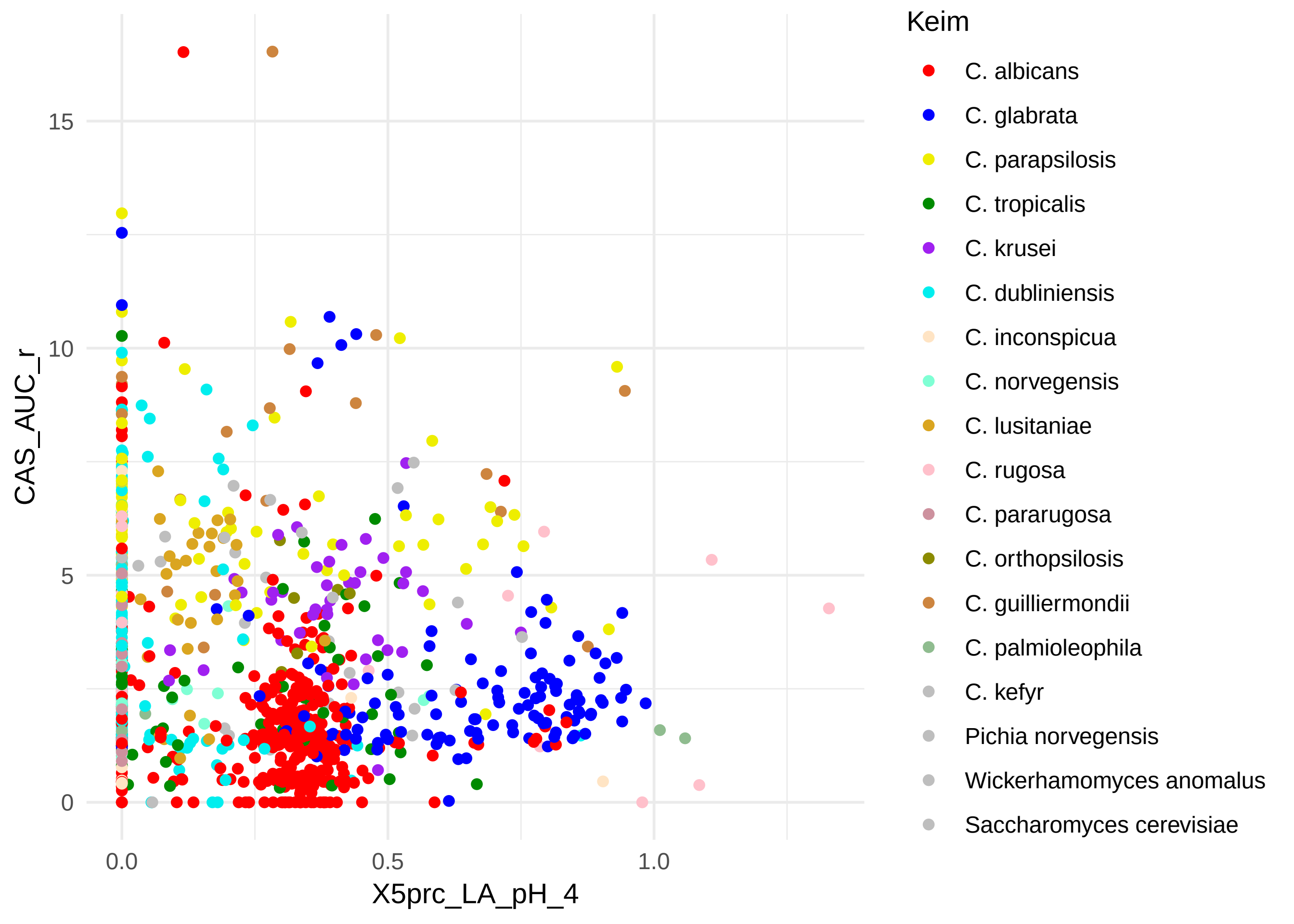
CAS_AUC_r
0
5
10
15
0.0
0.5
1.0
X5prc_LA_pH_4
Keim
C. albicans
C. glabrata
C. parapsilosis
C. tropicalis
C. krusei
C. dubliniensis
C. inconspicua
C. norvegensis
C. lusitaniae
C. rugosa
C. pararugosa
C. orthopsilosis
C. guilliermondii
C. palmioleophila
C. kefyr
Pichia norvegensis
Wickerhamomyces anomalus
Saccharomyces cerevisiae

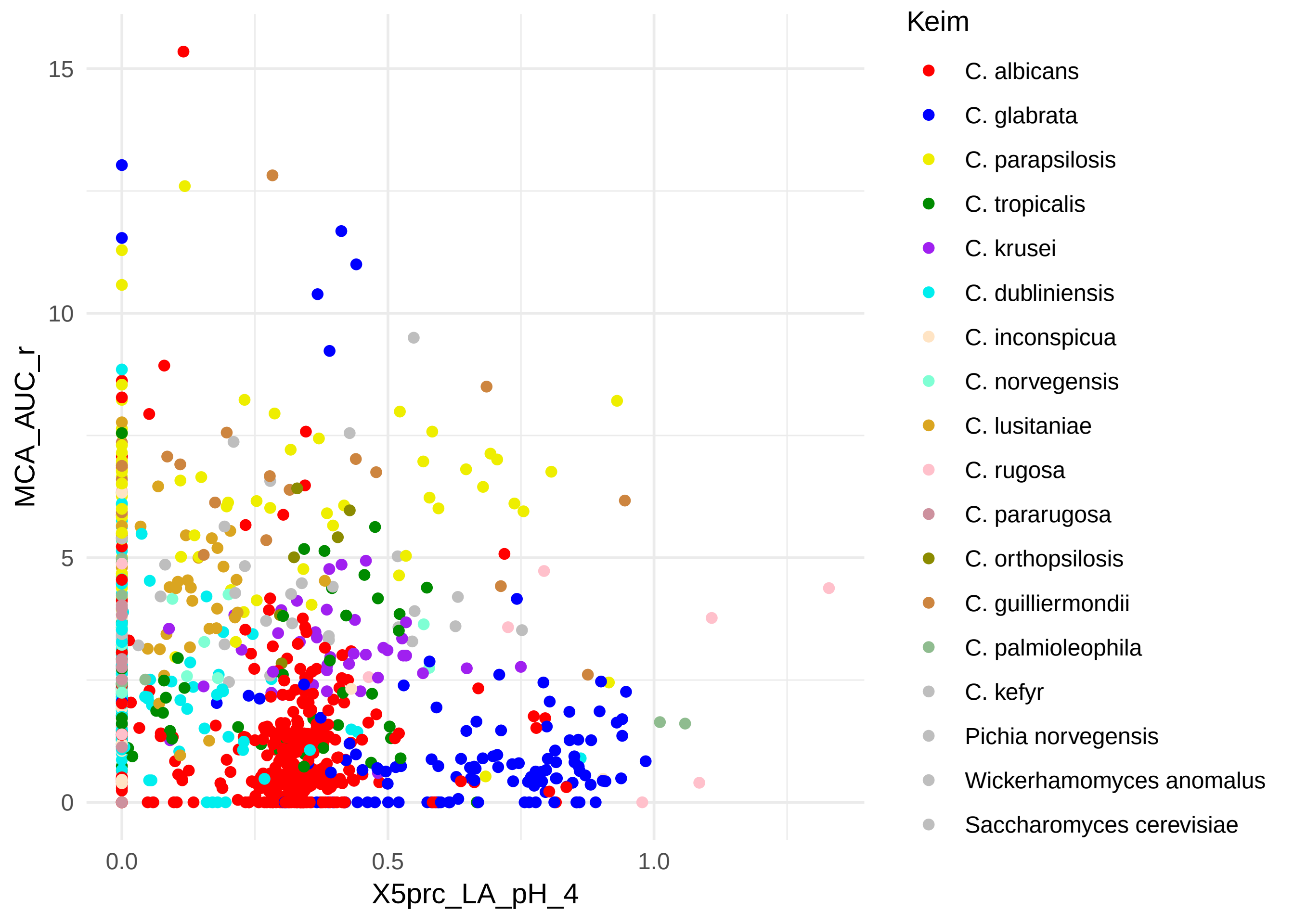

Keim
C. albicans
C. glabrata
C. parapsilosis
C. tropicalis
C. krusei
C. dubliniensis
C. inconspicua
C. norvegensis
C. lusitaniae
C. rugosa
C. pararugosa
C. orthopsilosis
C. guilliermondii
C. palmioleophila
C. kefyr
Pichia norvegensis
Wickerhamomyces anomalus
Saccharomyces cerevisiae
MCA_AUC_r
X5prc_LA_pH_4
15
10
5
0
0.0
0.5
1.0

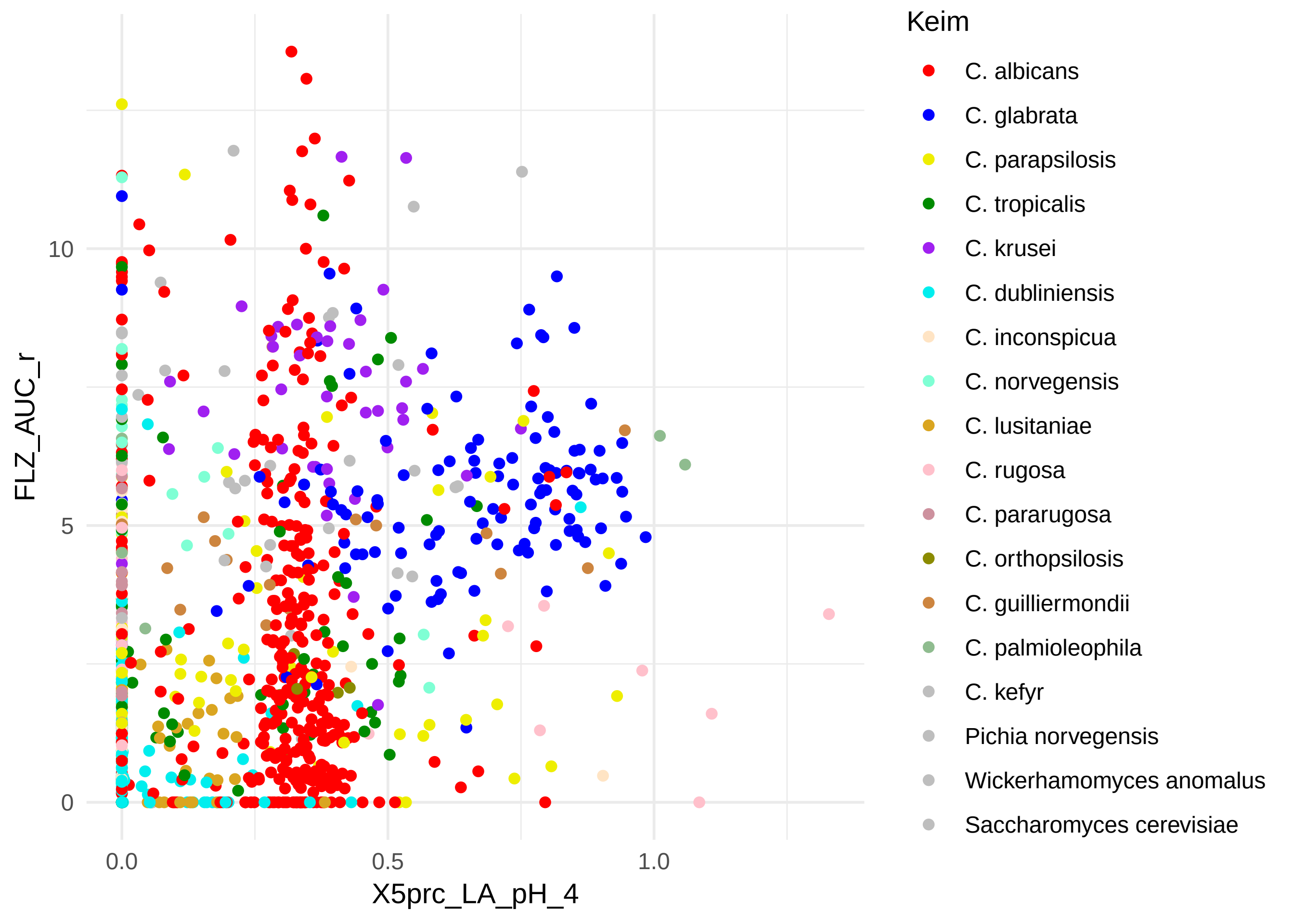
Keim
C. albicans
C. glabrata
C. parapsilosis
C. tropicalis
C. krusei
C. dubliniensis
C. inconspicua
C. norvegensis
C. lusitaniae
C. rugosa
C. pararugosa
C. orthopsilosis
C. guilliermondii
C. palmioleophila
C. kefyr
Pichia norvegensis
Wickerhamomyces anomalus
Saccharomyces cerevisiae
FLZ_AUC_r
10
5
0
0.0
0.5
1.0
X5prc_LA_pH_4

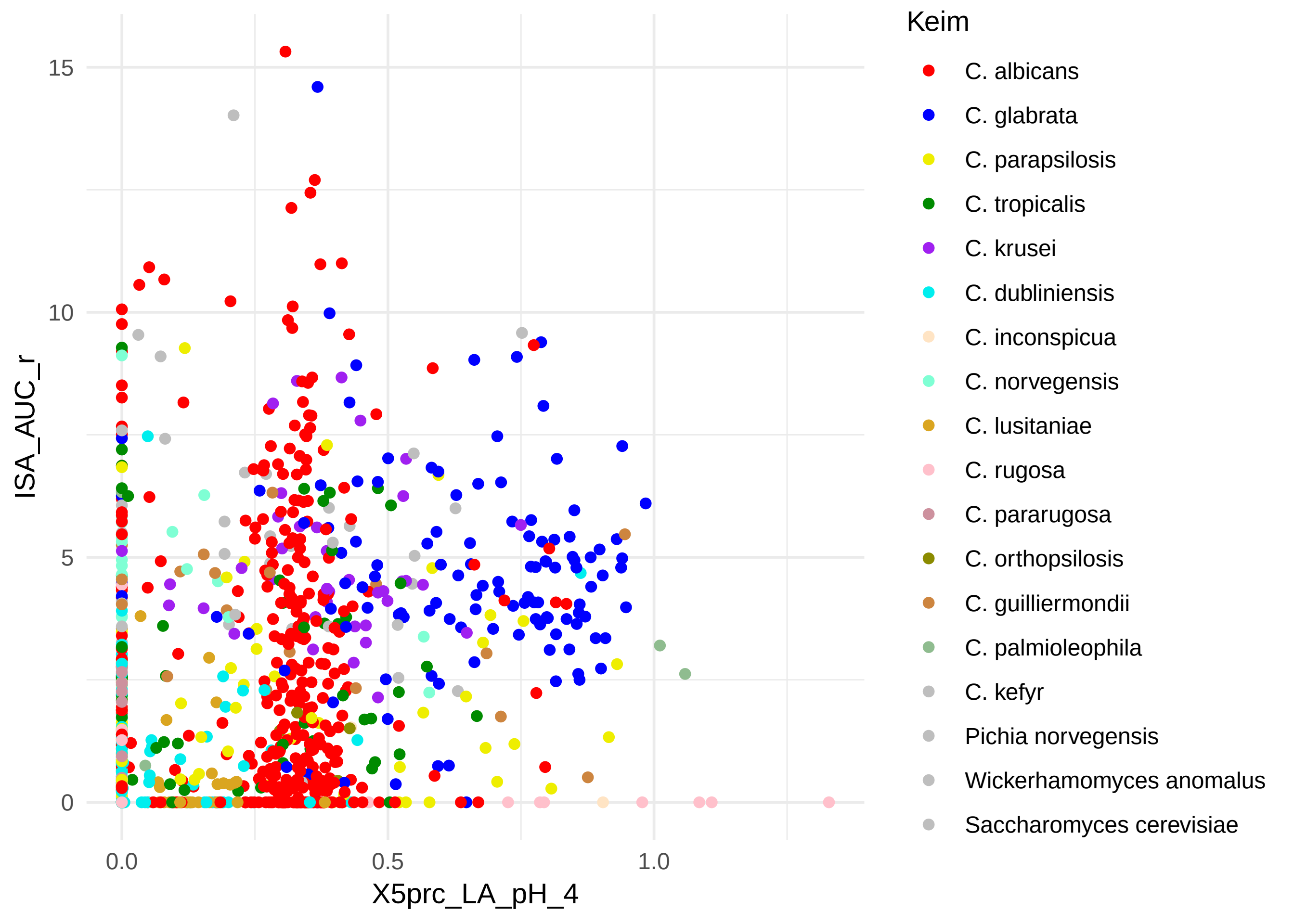
Keim
C. albicans
C. glabrata
C. parapsilosis
C. tropicalis
C. krusei
C. dubliniensis
C. inconspicua
C. norvegensis
C. lusitaniae
C. rugosa
C. pararugosa
C. orthopsilosis
C. guilliermondii
C. palmioleophila
C. kefyr
Pichia norvegensis
Wickerhamomyces anomalus
Saccharomyces cerevisiae
ISA_AUC_r
X5prc_LA_pH_4
0
5
10
15
0.0
0.5
1.0

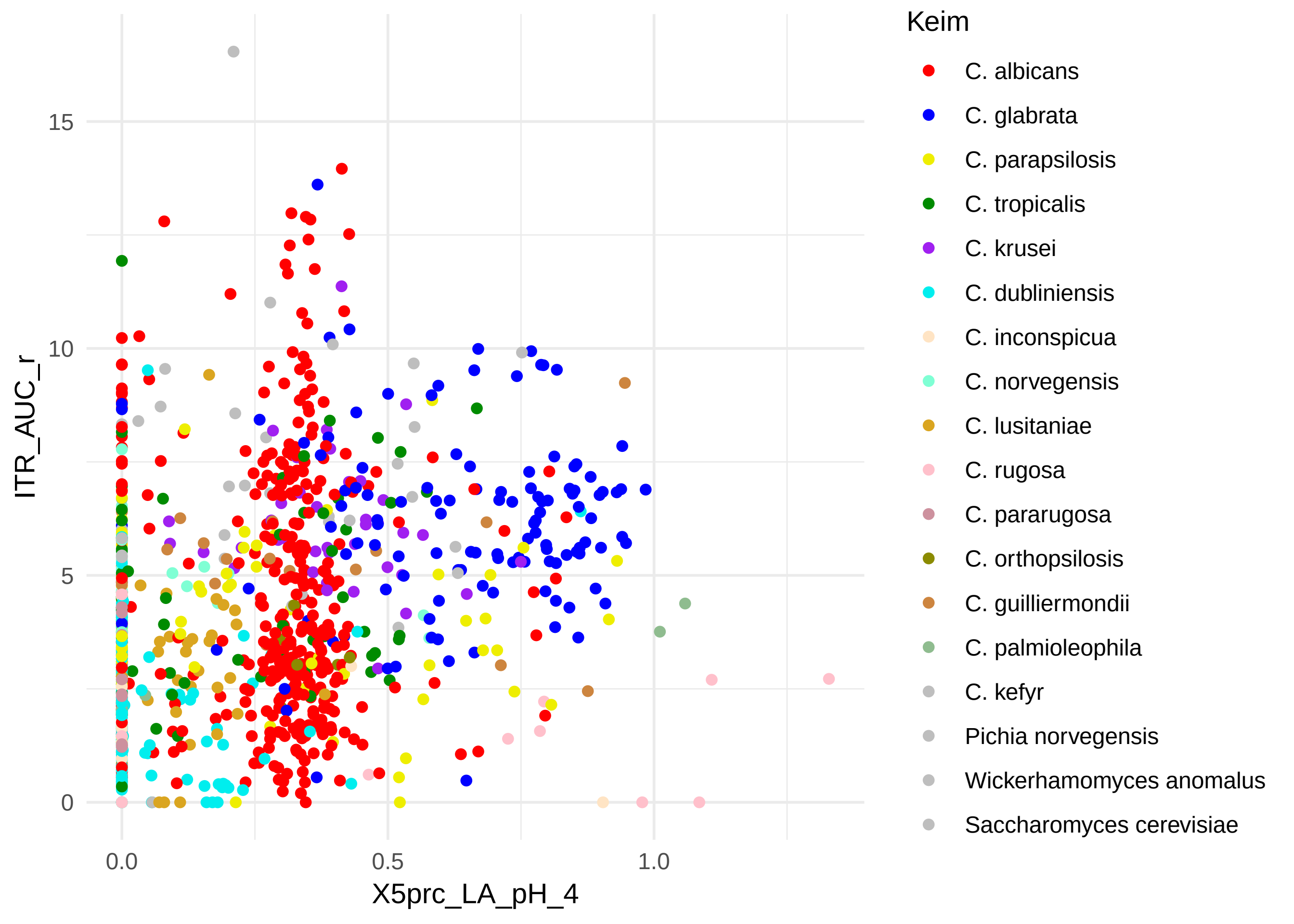

Keim
C. albicans
C. glabrata
C. parapsilosis
C. tropicalis
C. krusei
C. dubliniensis
C. inconspicua
C. norvegensis
C. lusitaniae
C. rugosa
C. pararugosa
C. orthopsilosis
C. guilliermondii
C. palmioleophila
C. kefyr
Pichia norvegensis
Wickerhamomyces anomalus
Saccharomyces cerevisiae
ITR_AUC_r
0
5
10
15
0.0
0.5
1.0
X5prc_LA_pH_4

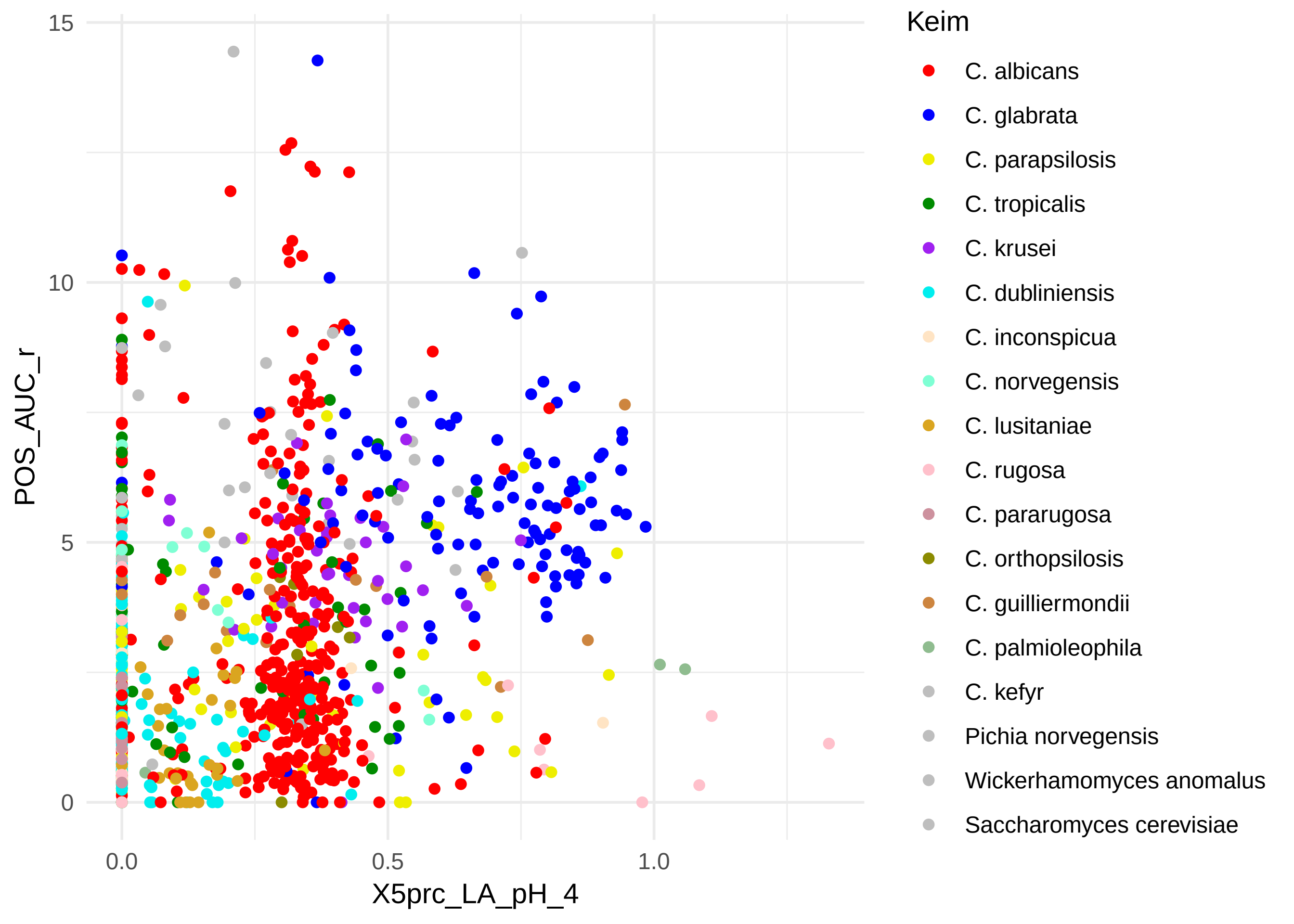
15
10
5
0
POS_AUC_r
0.0
0.5
1.0
X5prc_LA_pH_4
Keim
C. albicans
C. glabrata
C. parapsilosis
C. tropicalis
C. krusei
C. dubliniensis
C. inconspicua
C. norvegensis
C. lusitaniae
C. rugosa
C. pararugosa
C. orthopsilosis
C. guilliermondii
C. palmioleophila
C. kefyr
Pichia norvegensis
Wickerhamomyces anomalus
Saccharomyces cerevisiae

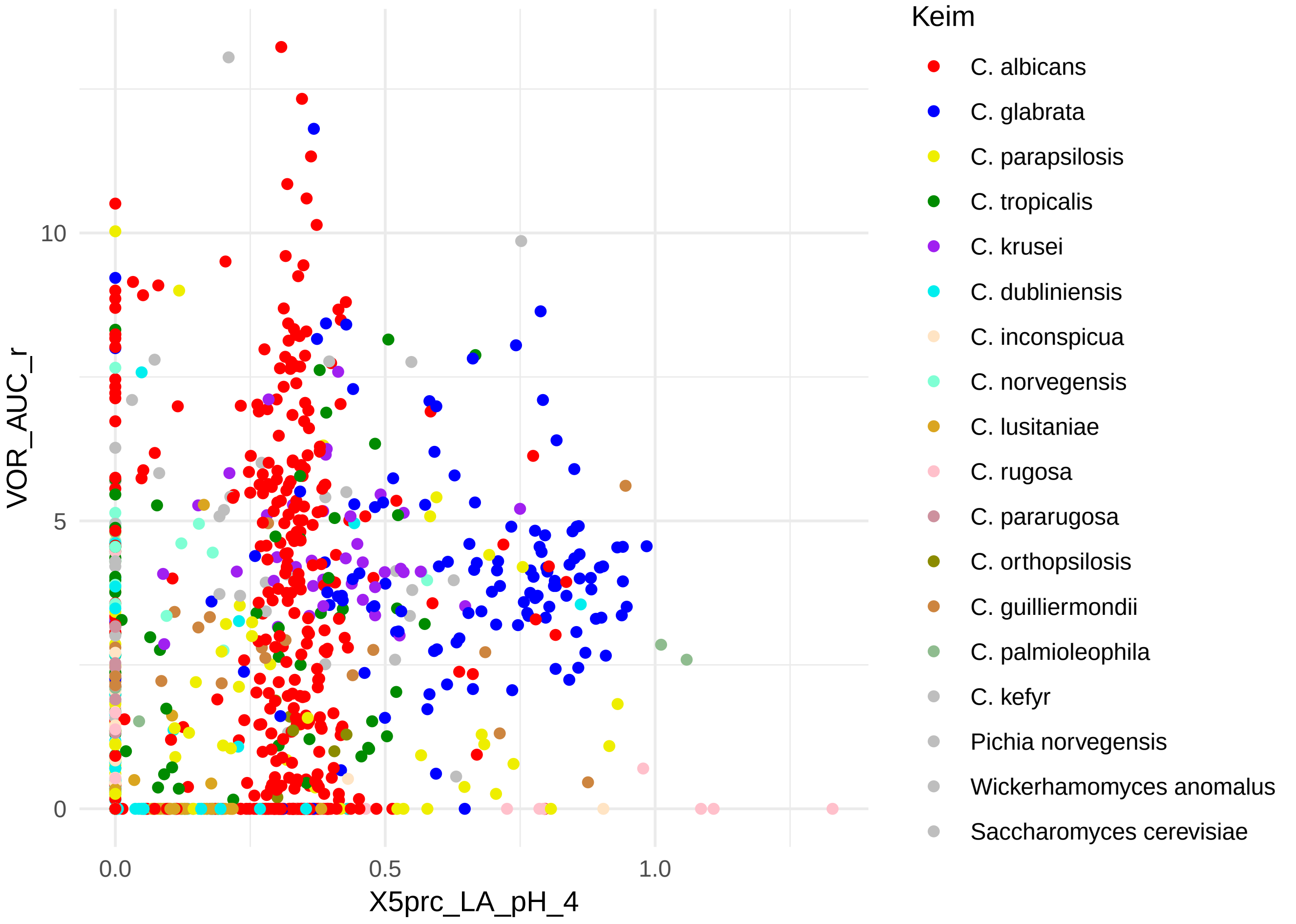
Keim
C. albicans
C. glabrata
C. parapsilosis
C. tropicalis
C. krusei
C. dubliniensis
C. inconspicua
C. norvegensis
C. lusitaniae
C. rugosa
C. pararugosa
C. orthopsilosis
C. guilliermondii
C. palmioleophila
C. kefyr
Pichia norvegensis
Wickerhamomyces anomalus
Saccharomyces cerevisiae
VOR_AUC_r
X5prc_LA_pH_4
0
5
10
0.0
0.5
1.0

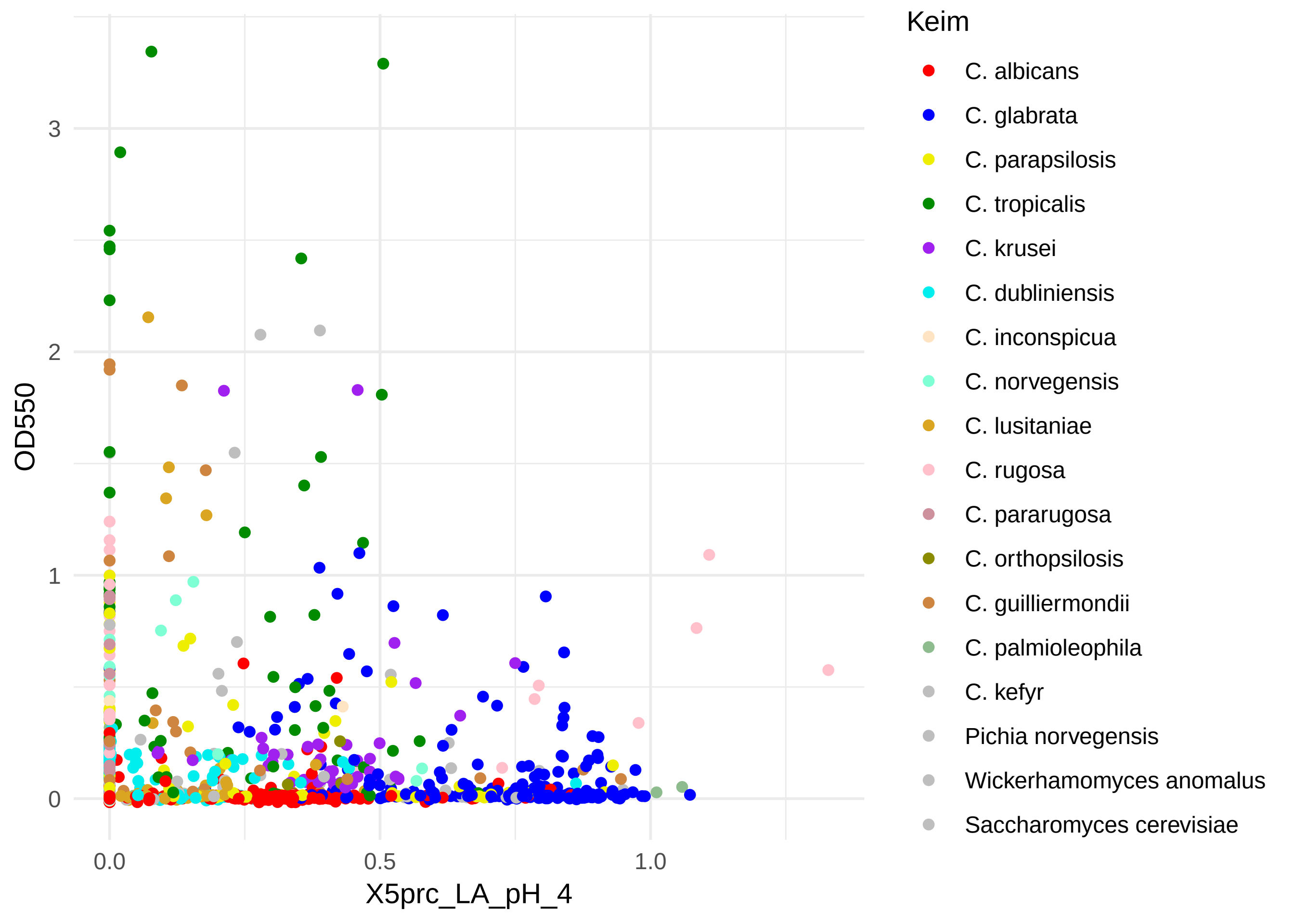

Keim
C. albicans
C. glabrata
C. parapsilosis
C. tropicalis
C. krusei
C. dubliniensis
C. inconspicua
C. norvegensis
C. lusitaniae
C. rugosa
C. pararugosa
C. orthopsilosis
C. guilliermondii
C. palmioleophila
C. kefyr
Pichia norvegensis
Wickerhamomyces anomalus
Saccharomyces cerevisiae
OD550
3
2
1
0
0.0
0.5
1.0
X5prc_LA_pH_4

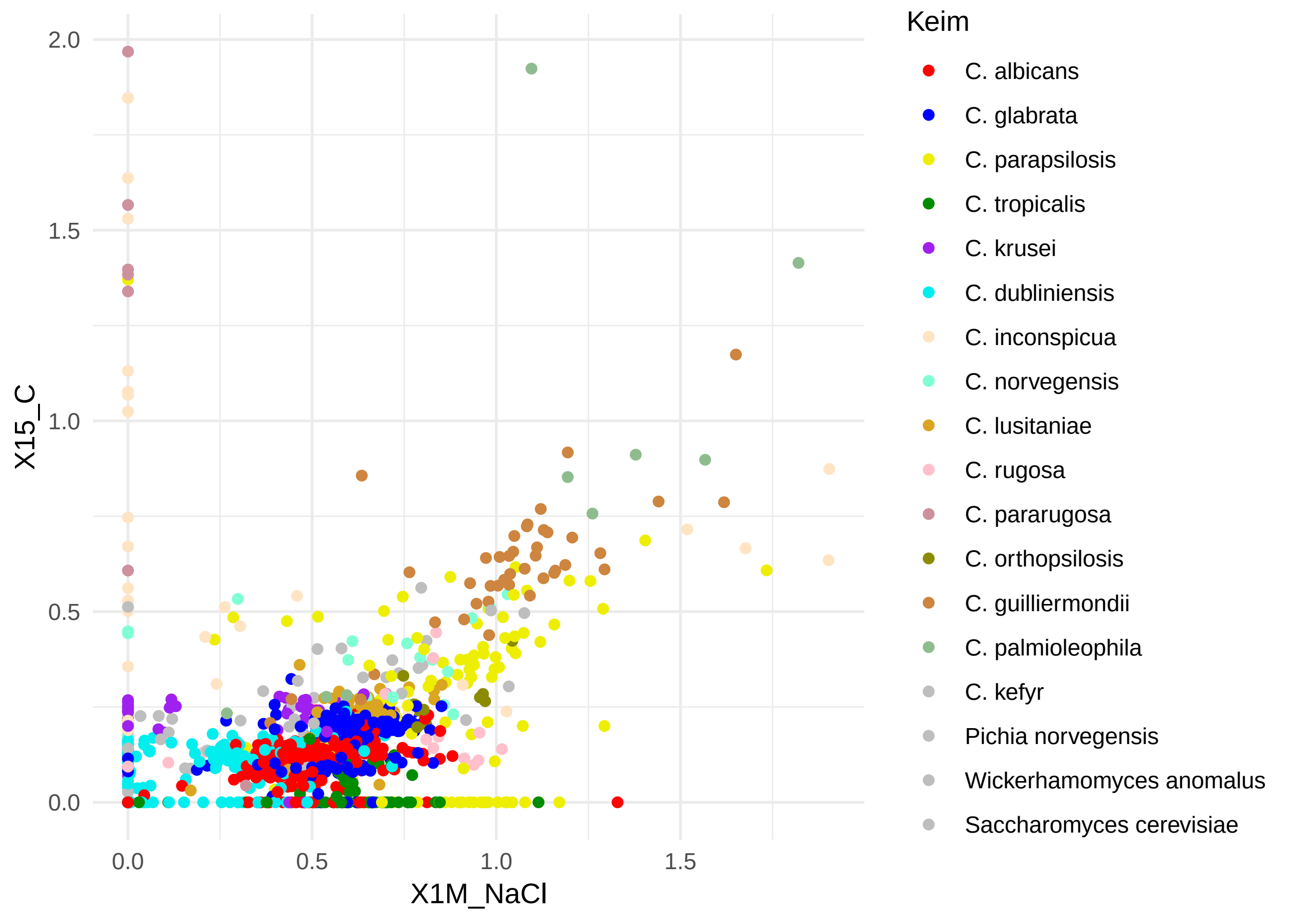
Keim
C. albicans
C. glabrata
C. parapsilosis
C. tropicalis
C. krusei
C. dubliniensis
C. inconspicua
C. norvegensis
C. lusitaniae
C. rugosa
C. pararugosa
C. orthopsilosis
C. guilliermondii
C. palmioleophila
C. kefyr
Pichia norvegensis
Wickerhamomyces anomalus
Saccharomyces cerevisiae
X15_C
X1M_NaCl
0.0
0.5
1.0
1.5
2.0

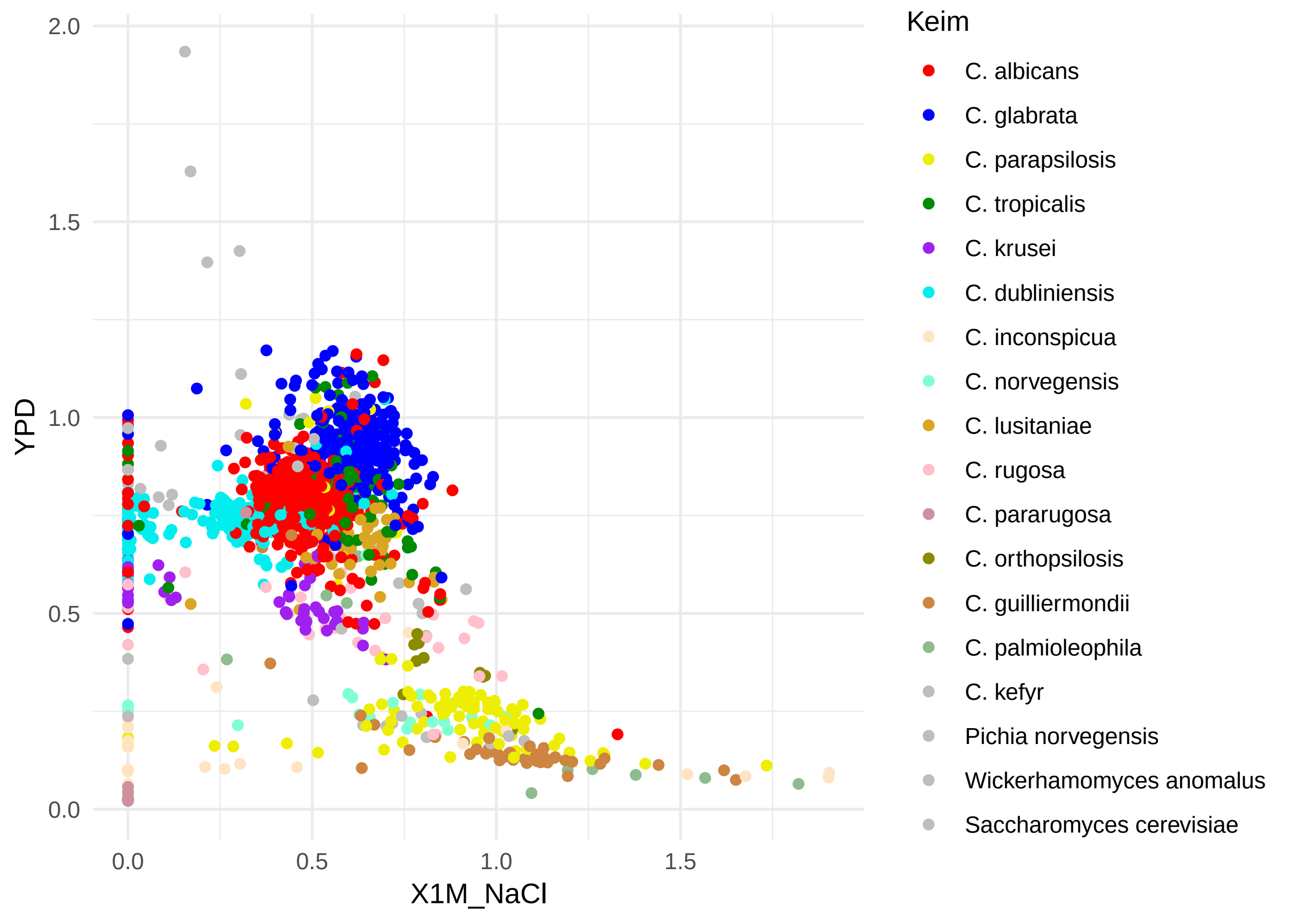

2.0
1.5
1.0
0.5
0.0
YPD
0.0
0.5
1.0
1.5
X1M_NaCl
Keim
C. albicans
C. glabrata
C. parapsilosis
C. tropicalis
C. krusei
C. dubliniensis
C. inconspicua
C. norvegensis
C. lusitaniae
C. rugosa
C. pararugosa
C. orthopsilosis
C. guilliermondii
C. palmioleophila
C. kefyr
Pichia norvegensis
Wickerhamomyces anomalus
Saccharomyces cerevisiae

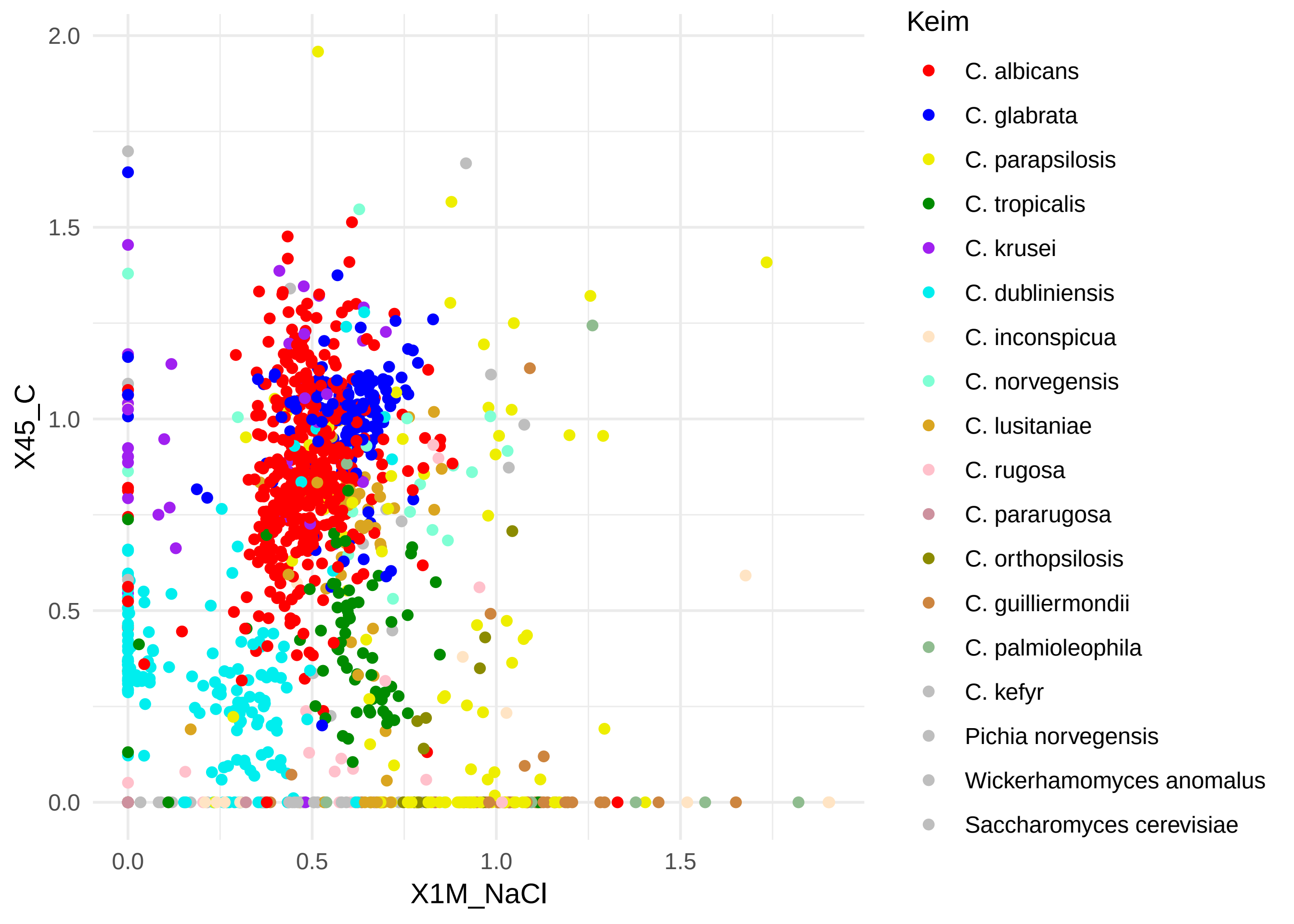

Keim
C. albicans
C. glabrata
C. parapsilosis
C. tropicalis
C. krusei
C. dubliniensis
C. inconspicua
C. norvegensis
C. lusitaniae
C. rugosa
C. pararugosa
C. orthopsilosis
C. guilliermondii
C. palmioleophila
C. kefyr
Pichia norvegensis
Wickerhamomyces anomalus
Saccharomyces cerevisiae
X45_C
2.0
1.5
1.0
0.5
0.0
0.0
0.5
1.0
1.5
X1M_NaCl

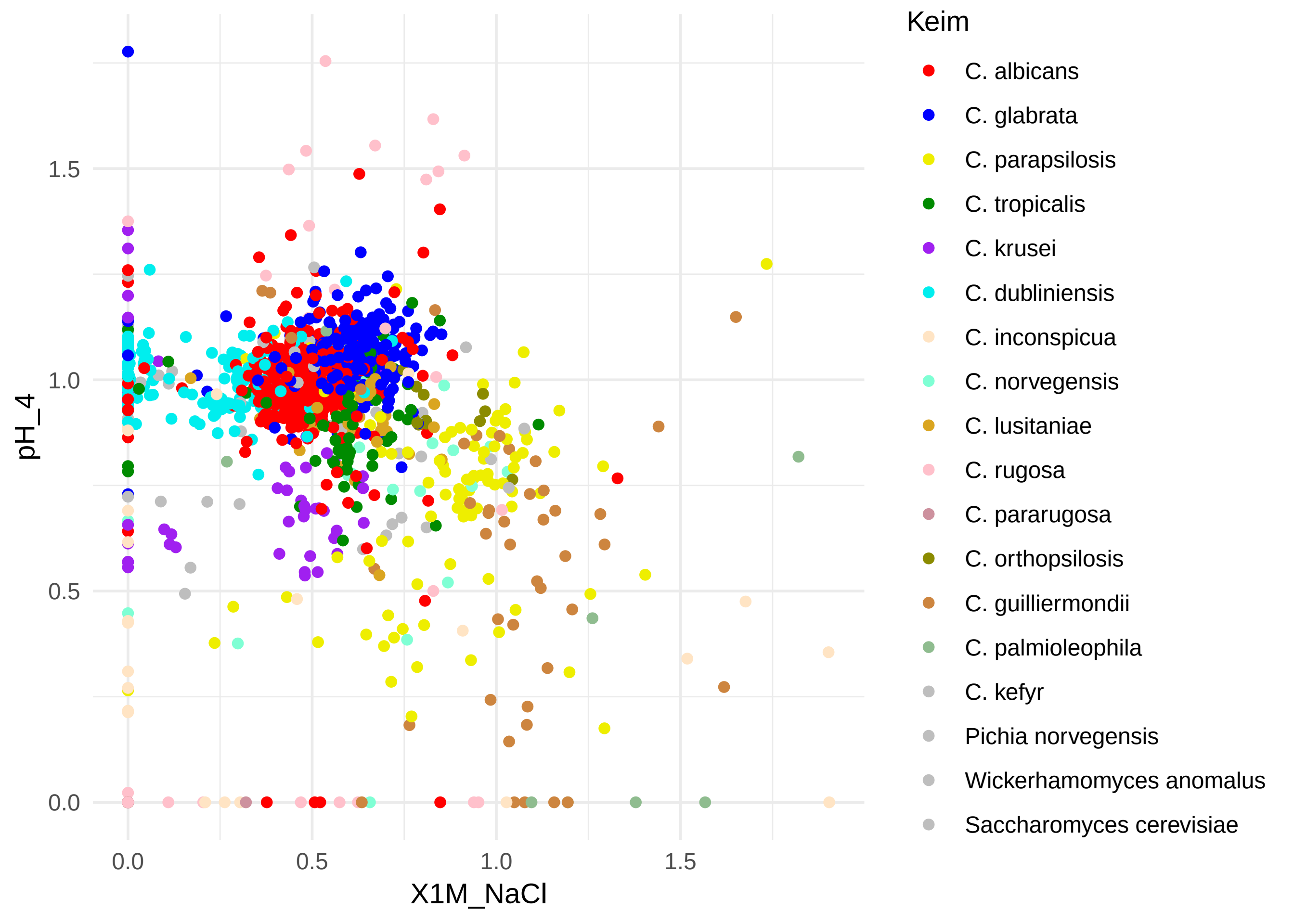
pH_4
X1M_NaCl
0.0
0.5
1.0
1.5
Keim
C. albicans
C. glabrata
C. parapsilosis
C. tropicalis
C. krusei
C. dubliniensis
C. inconspicua
C. norvegensis
C. lusitaniae
C. rugosa
C. pararugosa
C. orthopsilosis
C. guilliermondii
C. palmioleophila
C. kefyr
Pichia norvegensis
Wickerhamomyces anomalus
Saccharomyces cerevisiae

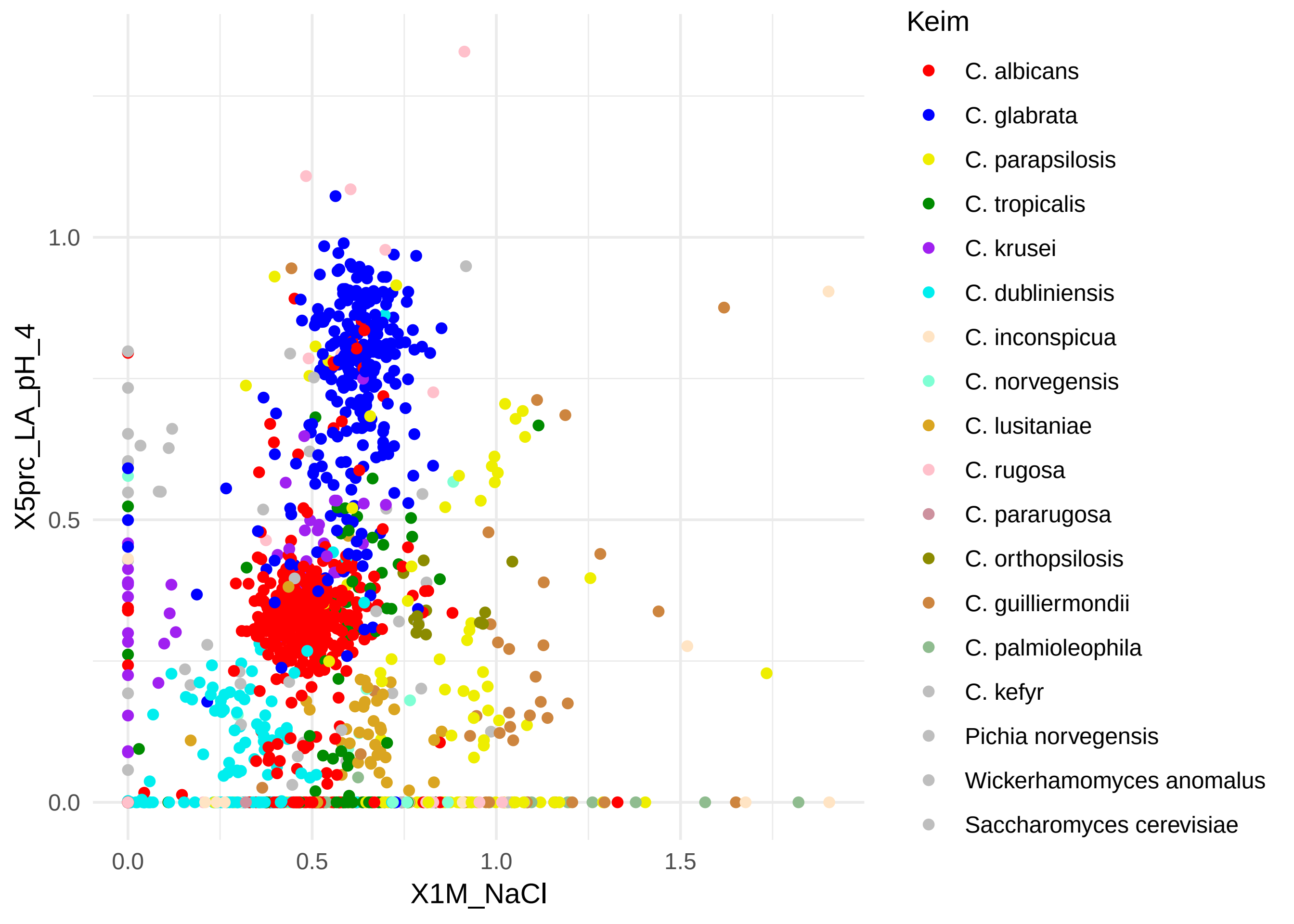
Keim
C. albicans
C. glabrata
C. parapsilosis
C. tropicalis
C. krusei
C. dubliniensis
C. inconspicua
C. norvegensis
C. lusitaniae
C. rugosa
C. pararugosa
C. orthopsilosis
C. guilliermondii
C. palmioleophila
C. kefyr
Pichia norvegensis
Wickerhamomyces anomalus
Saccharomyces cerevisiae
X5prc_LA_pH_4
1.0
0.5
0.0
0.0
0.5
1.0
1.5
X1M_NaCl

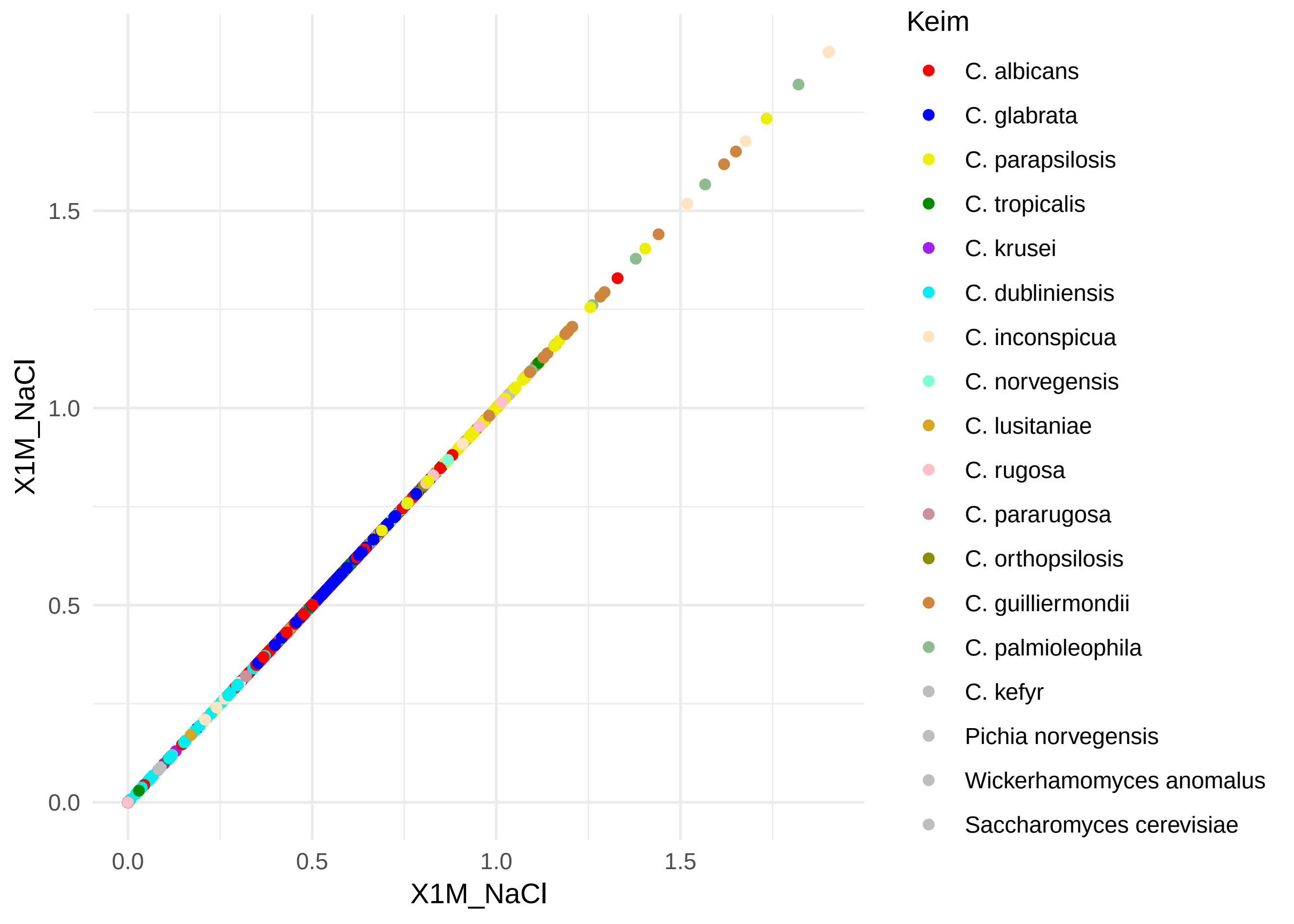
Keim
C. albicans
C. glabrata
C. parapsilosis
C. tropicalis
C. krusei
C. dubliniensis
C. inconspicua
C. norvegensis
C. lusitaniae
C. rugosa
C. pararugosa
C. orthopsilosis
C. guilliermondii
C. palmioleophila
C. kefyr
Pichia norvegensis
Wickerhamomyces anomalus
Saccharomyces cerevisiae
X1M_NaCl
X1M_NaCl
0.0
0.5
1.0
1.5

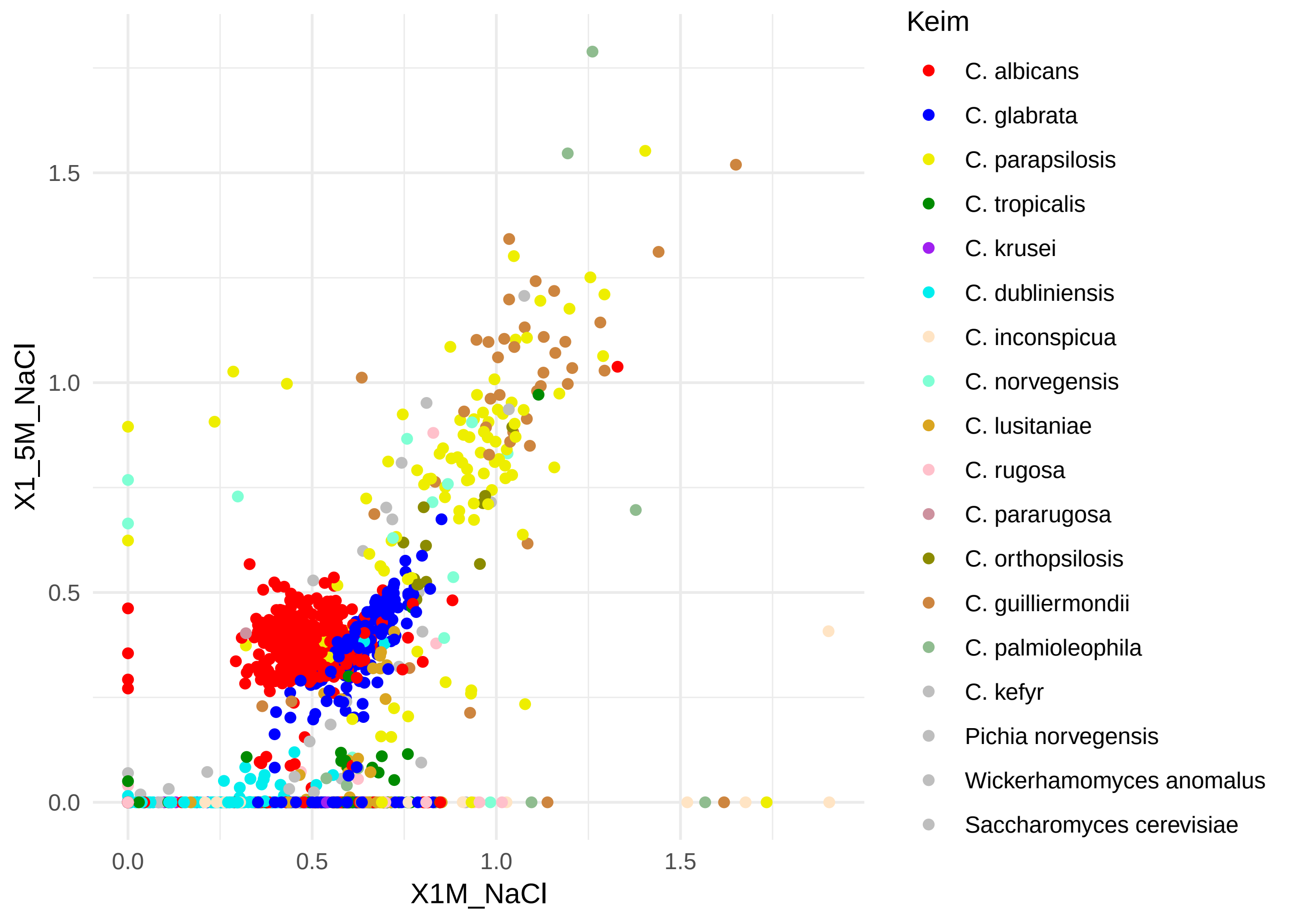
1.5
1.0
0.5
0.0
X1_5M_NaCl
0.0
0.5
1.0
1.5
X1M_NaCl
Keim
C. albicans
C. glabrata
C. parapsilosis
C. tropicalis
C. krusei
C. dubliniensis
C. inconspicua
C. norvegensis
C. lusitaniae
C. rugosa
C. pararugosa
C. orthopsilosis
C. guilliermondii
C. palmioleophila
C. kefyr
Pichia norvegensis
Wickerhamomyces anomalus
Saccharomyces cerevisiae

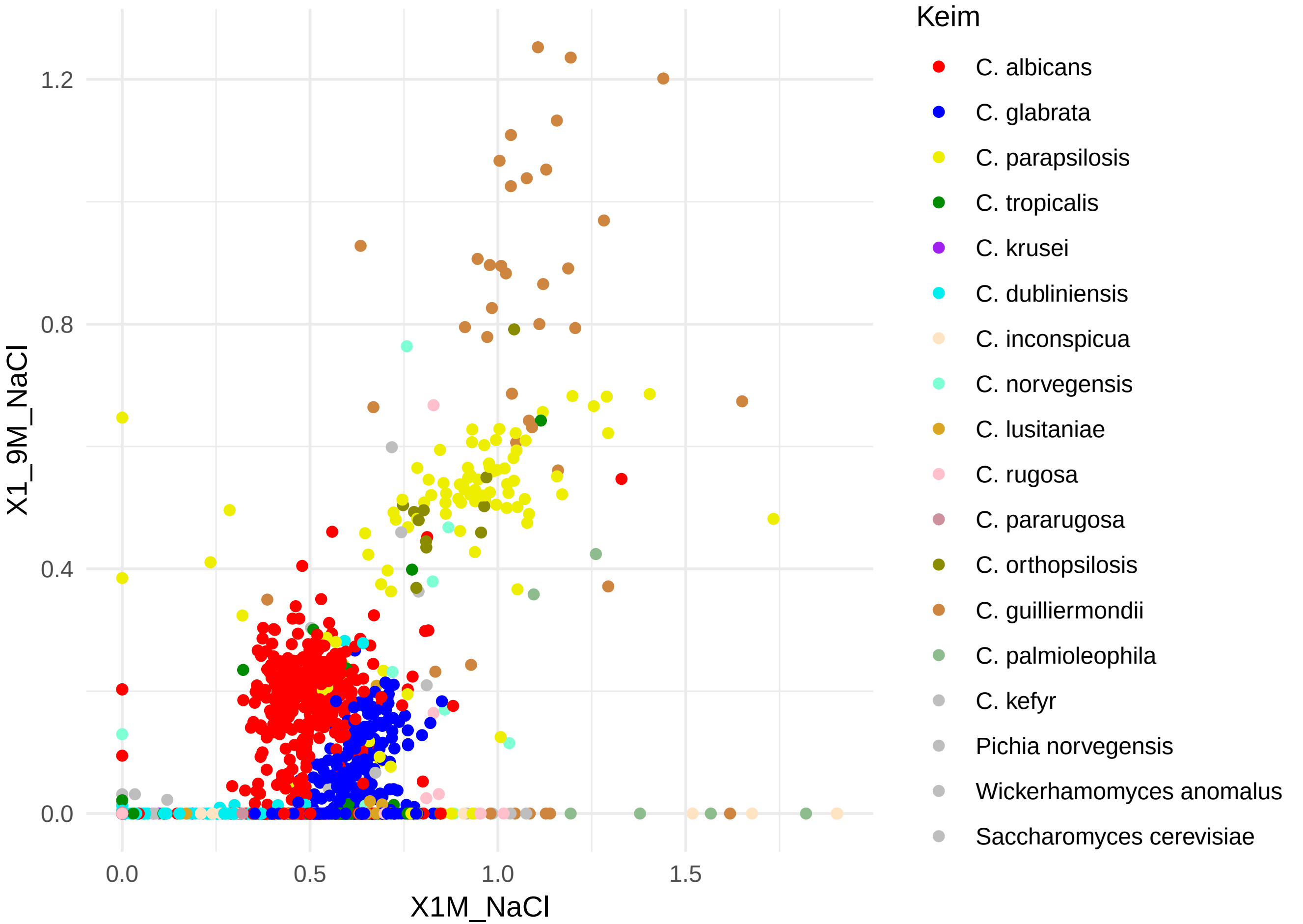
Keim
C. albicans
C. glabrata
C. parapsilosis
C. tropicalis
C. krusei
C. dubliniensis
C. inconspicua
C. norvegensis
C. lusitaniae
C. rugosa
C. pararugosa
C. orthopsilosis
C. guilliermondii
C. palmioleophila
C. kefyr
Pichia norvegensis
Wickerhamomyces anomalus
Saccharomyces cerevisiae
X1_9M_NaCl
X1M_NaCl
0.0
0.4
0.8
1.2
0.0
0.5
1.0
1.5

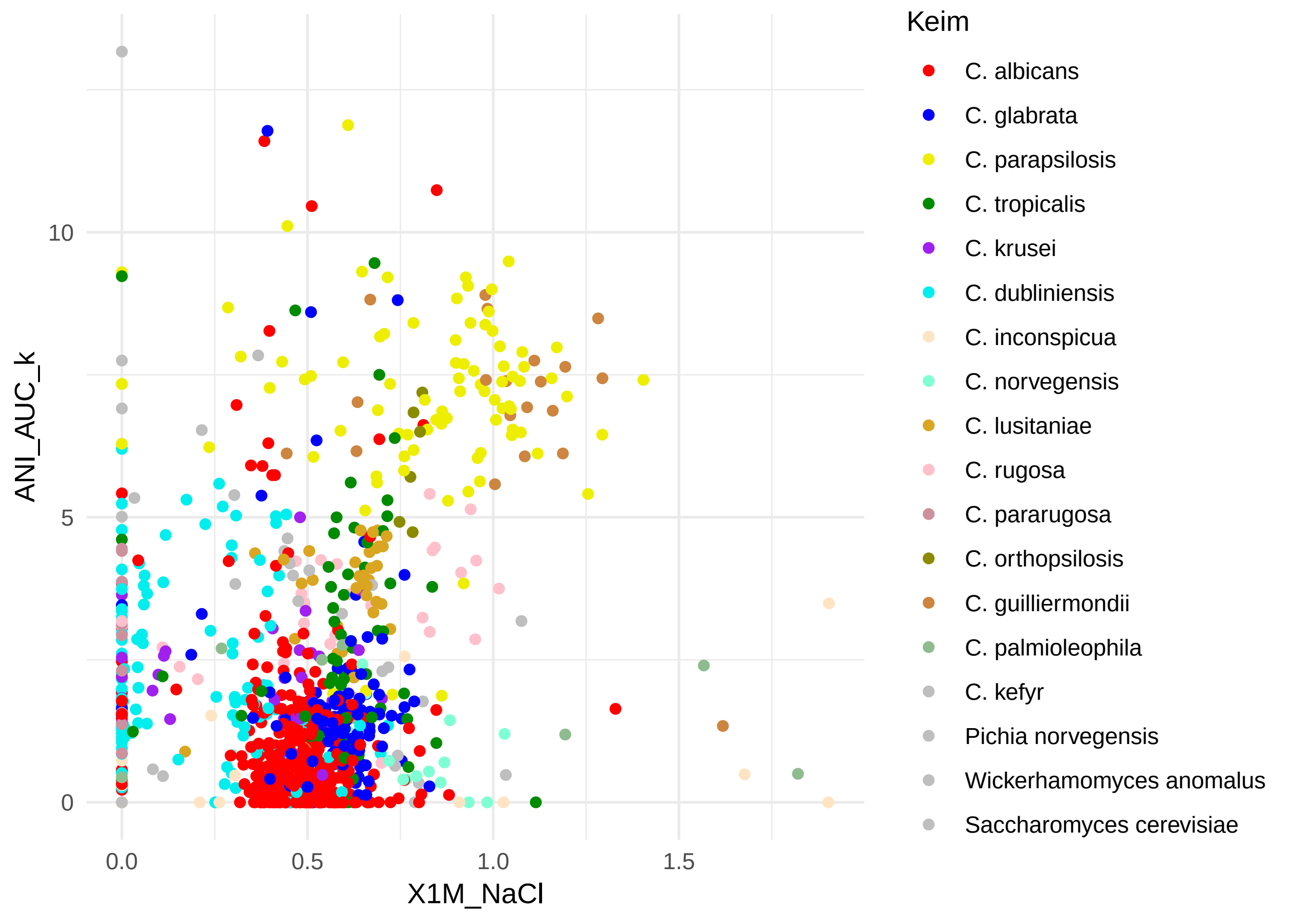
Keim
C. albicans
C. glabrata
C. parapsilosis
C. tropicalis
C. krusei
C. dubliniensis
C. inconspicua
C. norvegensis
C. lusitaniae
C. rugosa
C. pararugosa
C. orthopsilosis
C. guilliermondii
C. palmioleophila
C. kefyr
Pichia norvegensis
Wickerhamomyces anomalus
Saccharomyces cerevisiae
ANI_AUC_k
X1M_NaCl
0
5
10
0.0
0.5
1.0
1.5

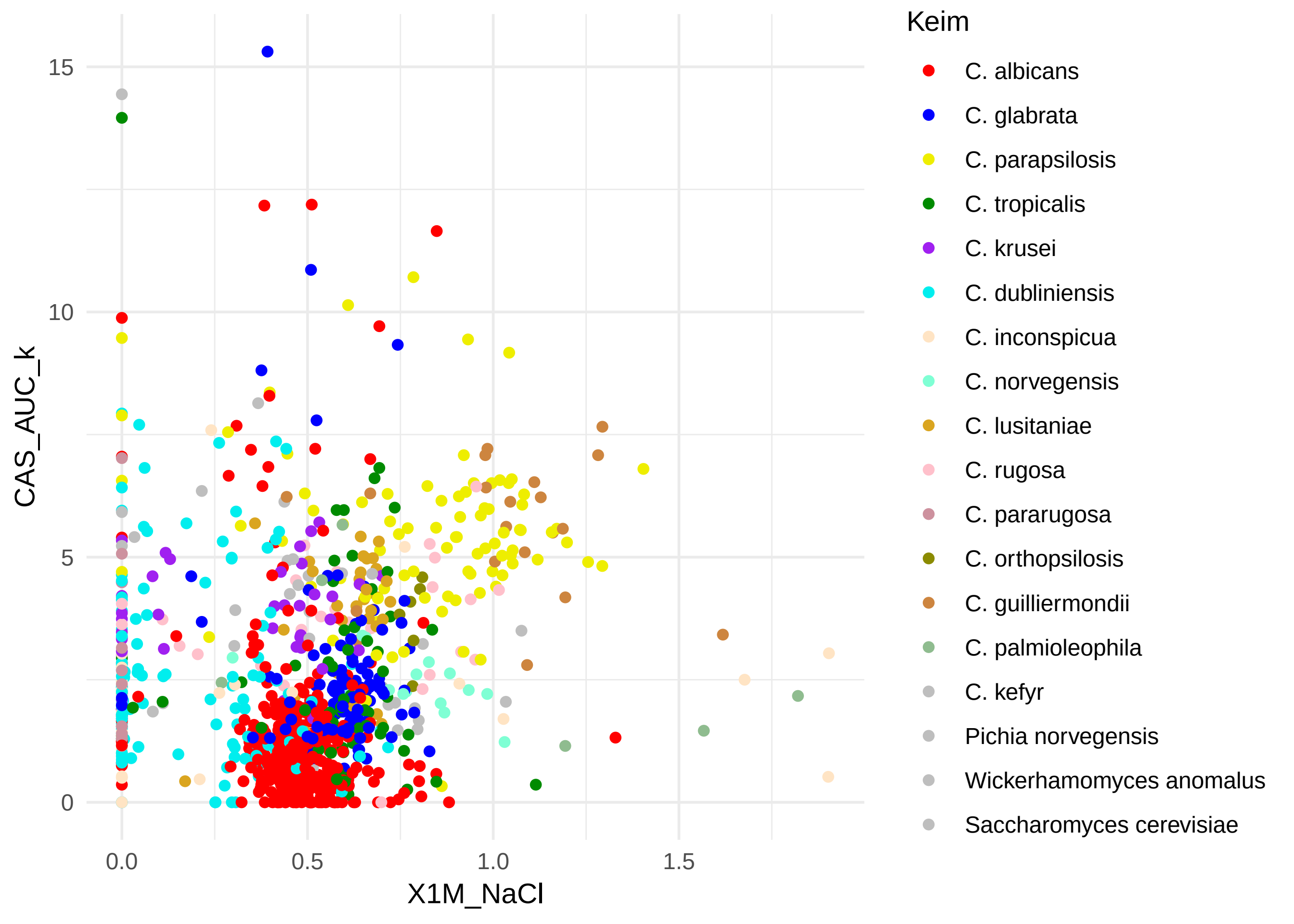
Keim
C. albicans
C. glabrata
C. parapsilosis
C. tropicalis
C. krusei
C. dubliniensis
C. inconspicua
C. norvegensis
C. lusitaniae
C. rugosa
C. pararugosa
C. orthopsilosis
C. guilliermondii
C. palmioleophila
C. kefyr
Pichia norvegensis
Wickerhamomyces anomalus
Saccharomyces cerevisiae
CAS_AUC_k
X1M_NaCl
0
5
10
15
0.0
0.5
1.0
1.5

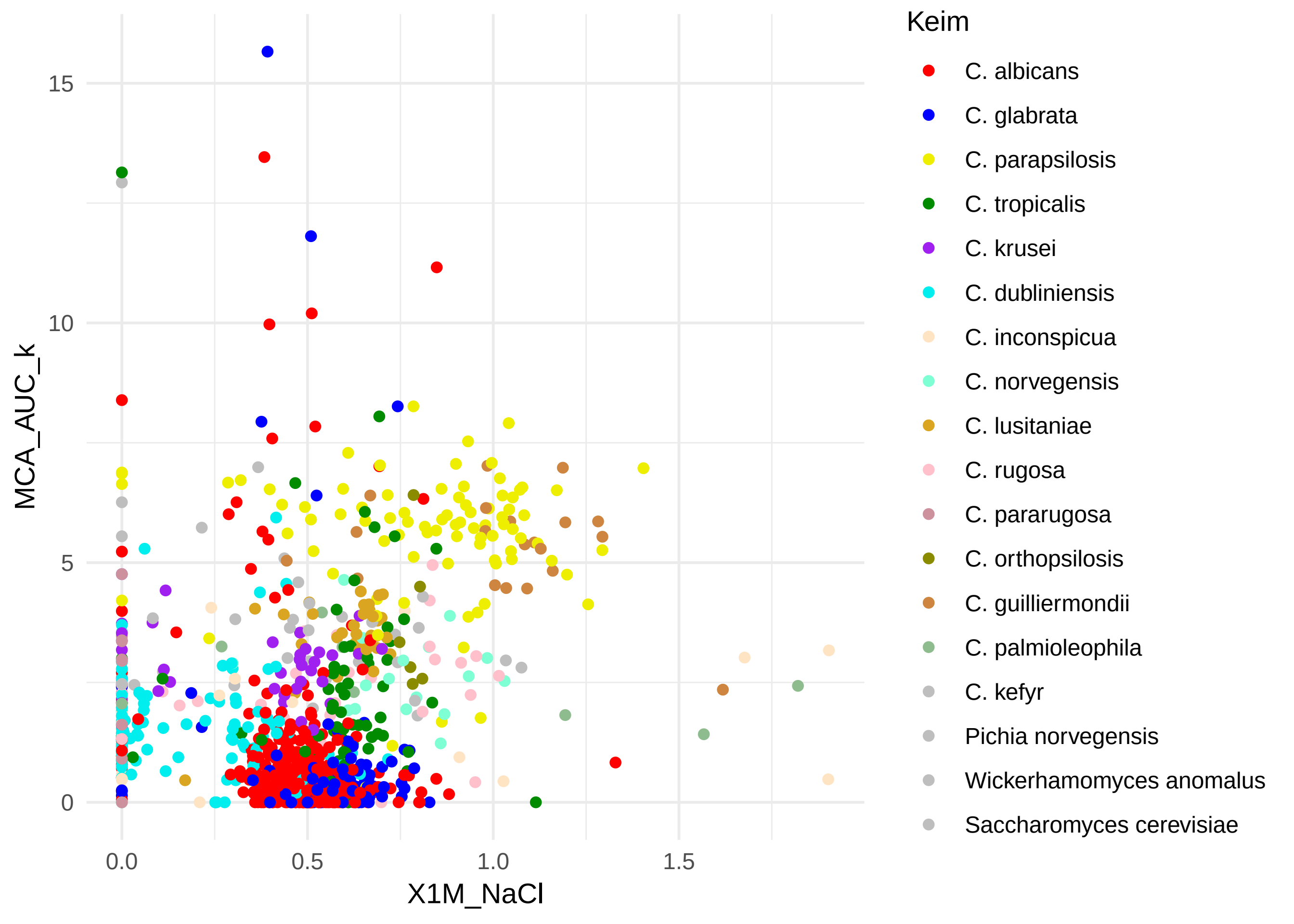
Keim
C. albicans
C. glabrata
C. parapsilosis
C. tropicalis
C. krusei
C. dubliniensis
C. inconspicua
C. norvegensis
C. lusitaniae
C. rugosa
C. pararugosa
C. orthopsilosis
C. guilliermondii
C. palmioleophila
C. kefyr
Pichia norvegensis
Wickerhamomyces anomalus
Saccharomyces cerevisiae
MCA_AUC_k
X1M_NaCl
0
5
10
15
0.0
0.5
1.0
1.5

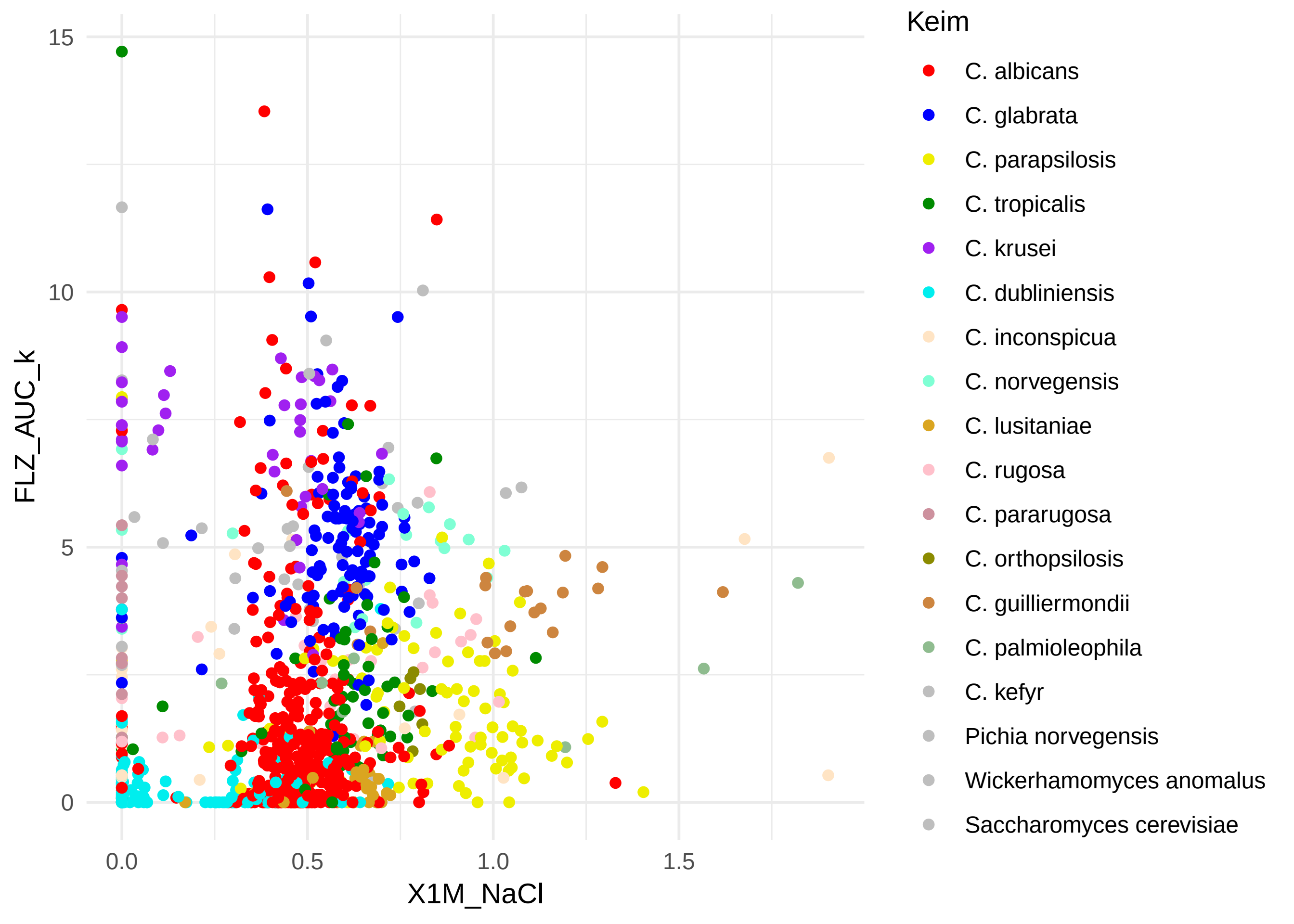

Keim
C. albicans
C. glabrata
C. parapsilosis
C. tropicalis
C. krusei
C. dubliniensis
C. inconspicua
C. norvegensis
C. lusitaniae
C. rugosa
C. pararugosa
C. orthopsilosis
C. guilliermondii
C. palmioleophila
C. kefyr
Pichia norvegensis
Wickerhamomyces anomalus
Saccharomyces cerevisiae
FLZ_AUC_k
X1M_NaCl
0
5
10
15
0.0
0.5
1.0
1.5

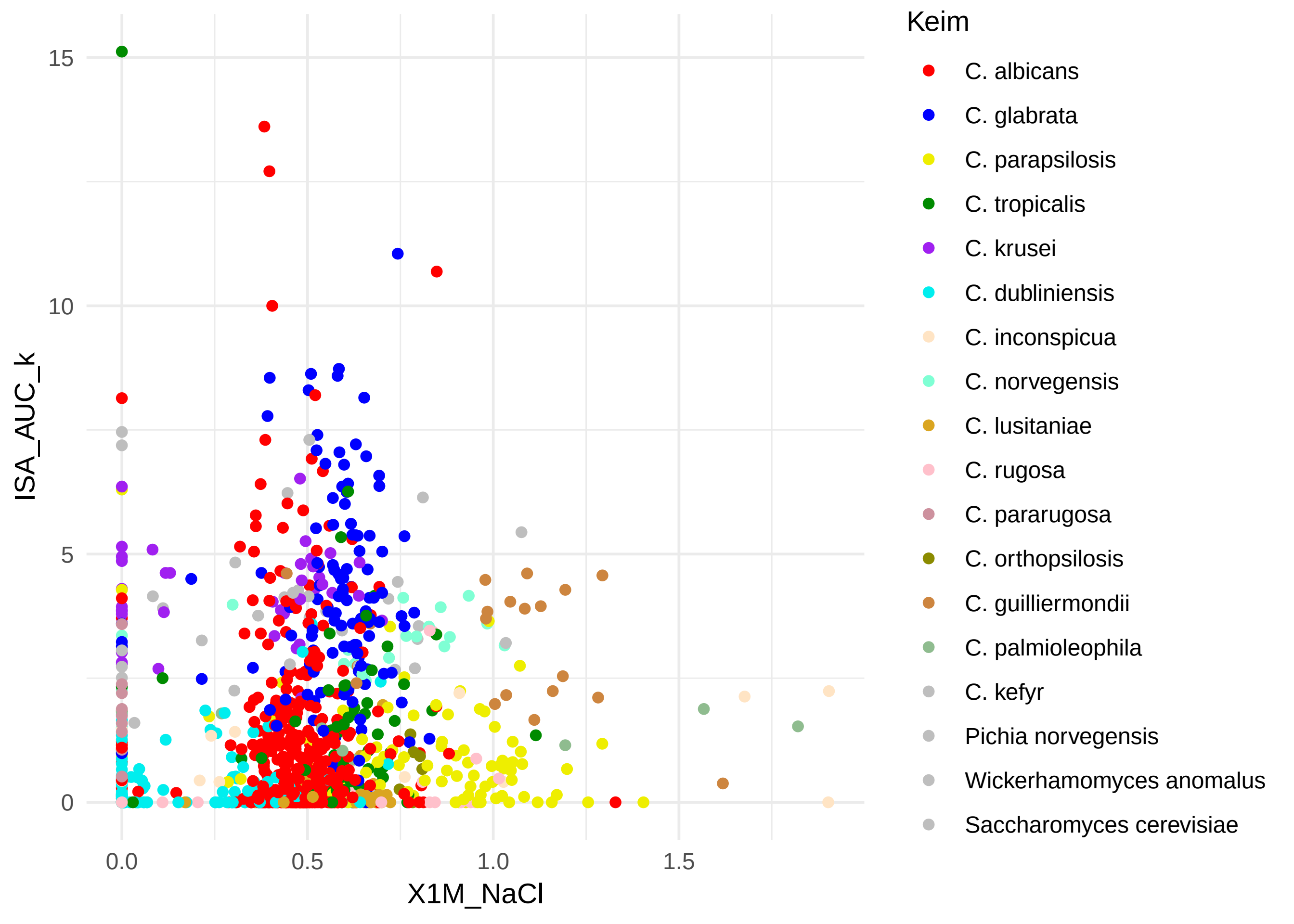

Keim
C. albicans
C. glabrata
C. parapsilosis
C. tropicalis
C. krusei
C. dubliniensis
C. inconspicua
C. norvegensis
C. lusitaniae
C. rugosa
C. pararugosa
C. orthopsilosis
C. guilliermondii
C. palmioleophila
C. kefyr
Pichia norvegensis
Wickerhamomyces anomalus
Saccharomyces cerevisiae
ISA_AUC_k
X1M_NaCl
15
10
5
0
0.0
0.5
1.0
1.5

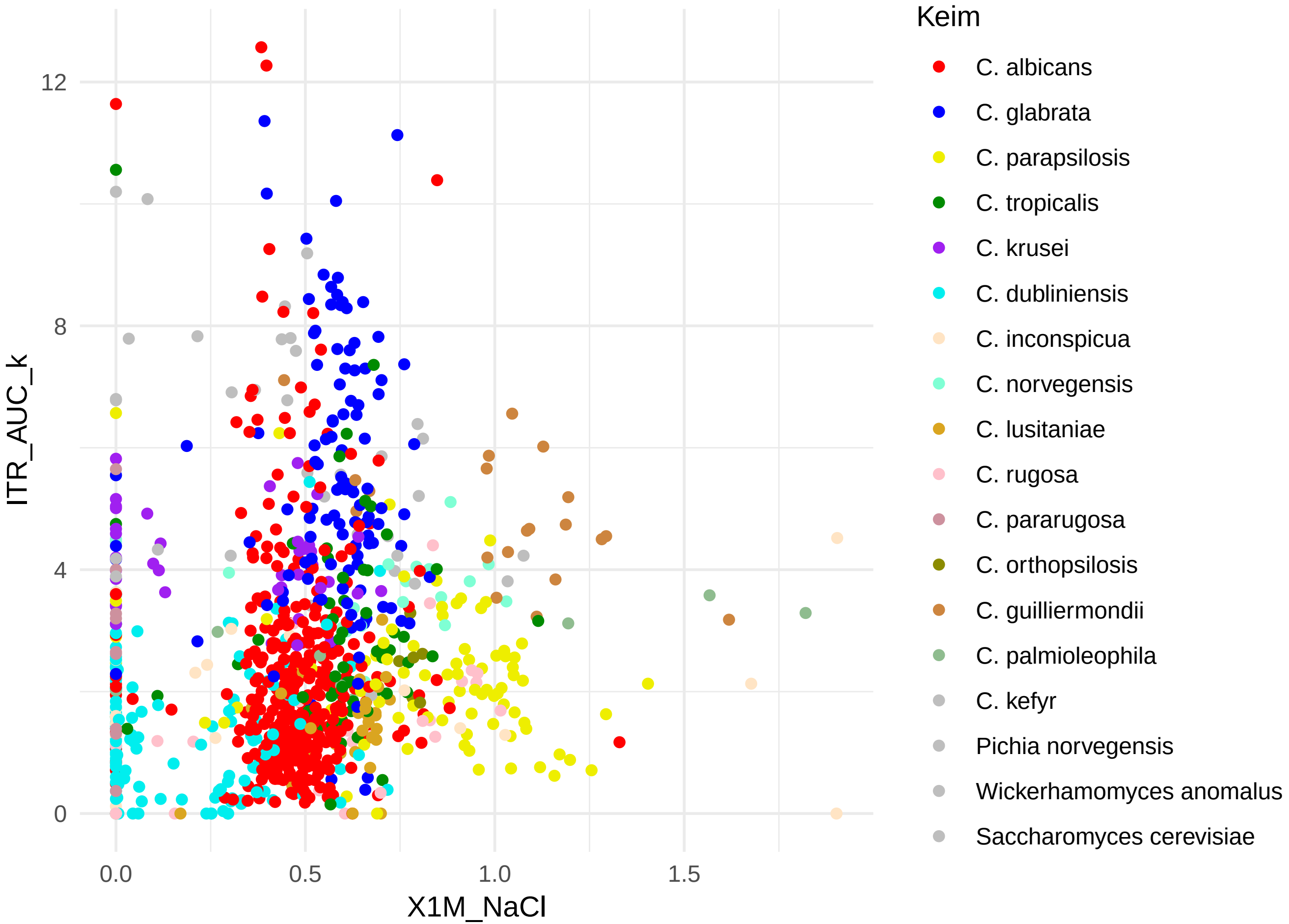
Keim
C. albicans
C. glabrata
C. parapsilosis
C. tropicalis
C. krusei
C. dubliniensis
C. inconspicua
C. norvegensis
C. lusitaniae
C. rugosa
C. pararugosa
C. orthopsilosis
C. guilliermondii
C. palmioleophila
C. kefyr
Pichia norvegensis
Wickerhamomyces anomalus
Saccharomyces cerevisiae
ITR_AUC_k
X1M_NaCl
0
4
8
12
0.0
0.5
1.0
1.5

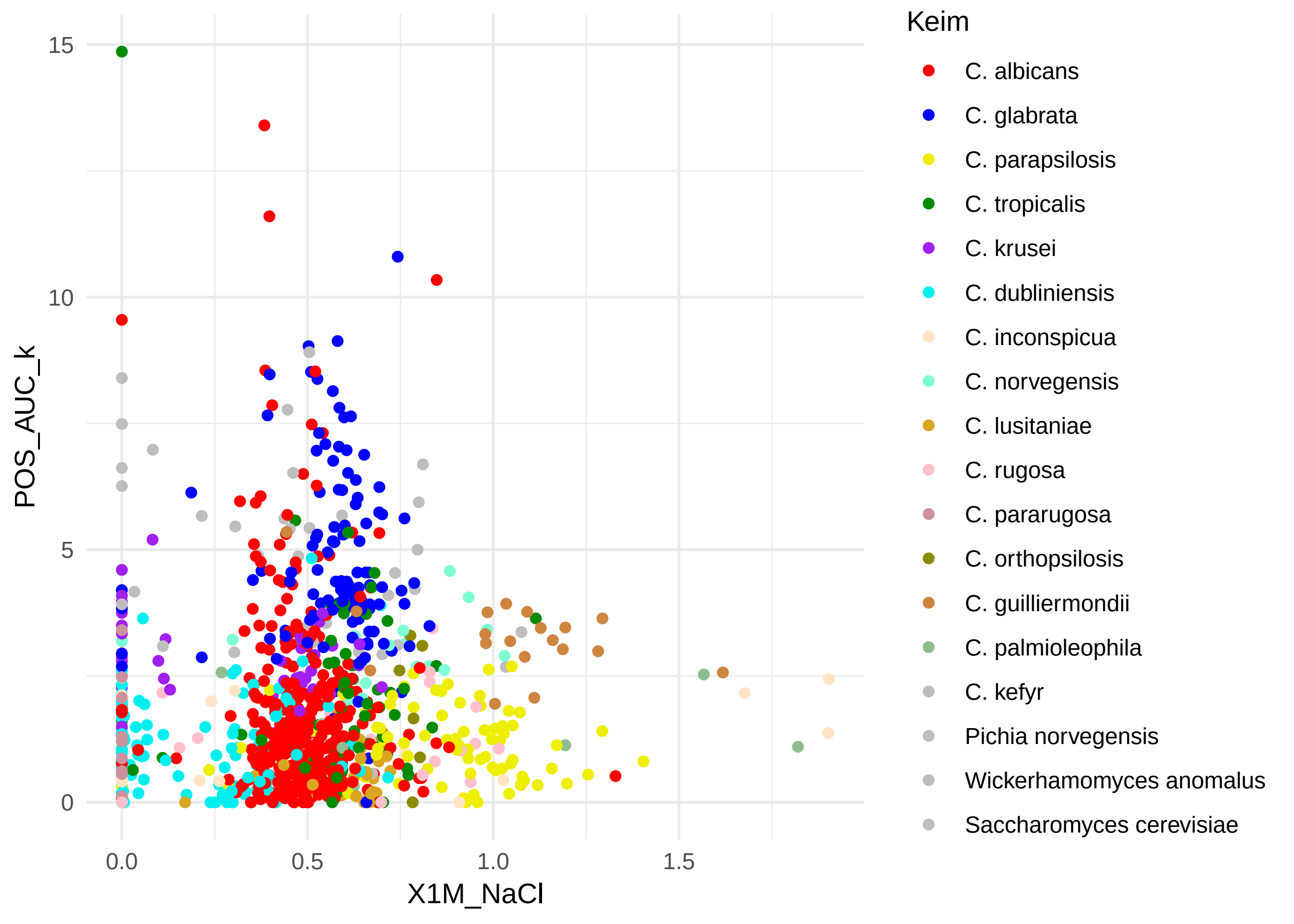

Keim
C. albicans
C. glabrata
C. parapsilosis
C. tropicalis
C. krusei
C. dubliniensis
C. inconspicua
C. norvegensis
C. lusitaniae
C. rugosa
C. pararugosa
C. orthopsilosis
C. guilliermondii
C. palmioleophila
C. kefyr
Pichia norvegensis
Wickerhamomyces anomalus
Saccharomyces cerevisiae
POS_AUC_k
X1M_NaCl
15
10
5
0
0.0
0.5
1.0
1.5

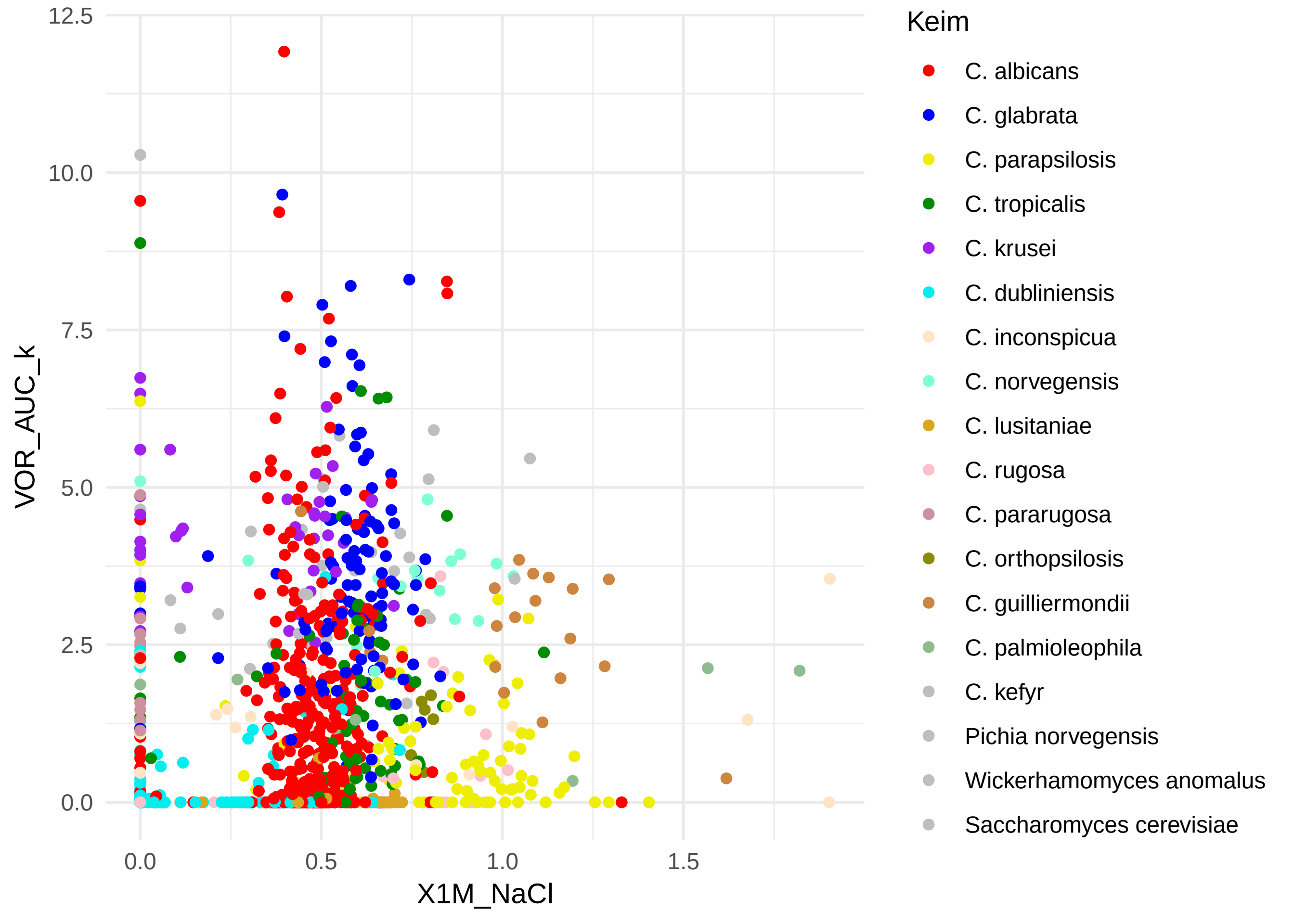

12.5
10.0
7.5
5.0
2.5
0.0
VOR_AUC_k
0.0
0.5
1.0
1.5
X1M_NaCl
Keim
C. albicans
C. glabrata
C. parapsilosis
C. tropicalis
C. krusei
C. dubliniensis
C. inconspicua
C. norvegensis
C. lusitaniae
C. rugosa
C. pararugosa
C. orthopsilosis
C. guilliermondii
C. palmioleophila
C. kefyr
Pichia norvegensis
Wickerhamomyces anomalus
Saccharomyces cerevisiae

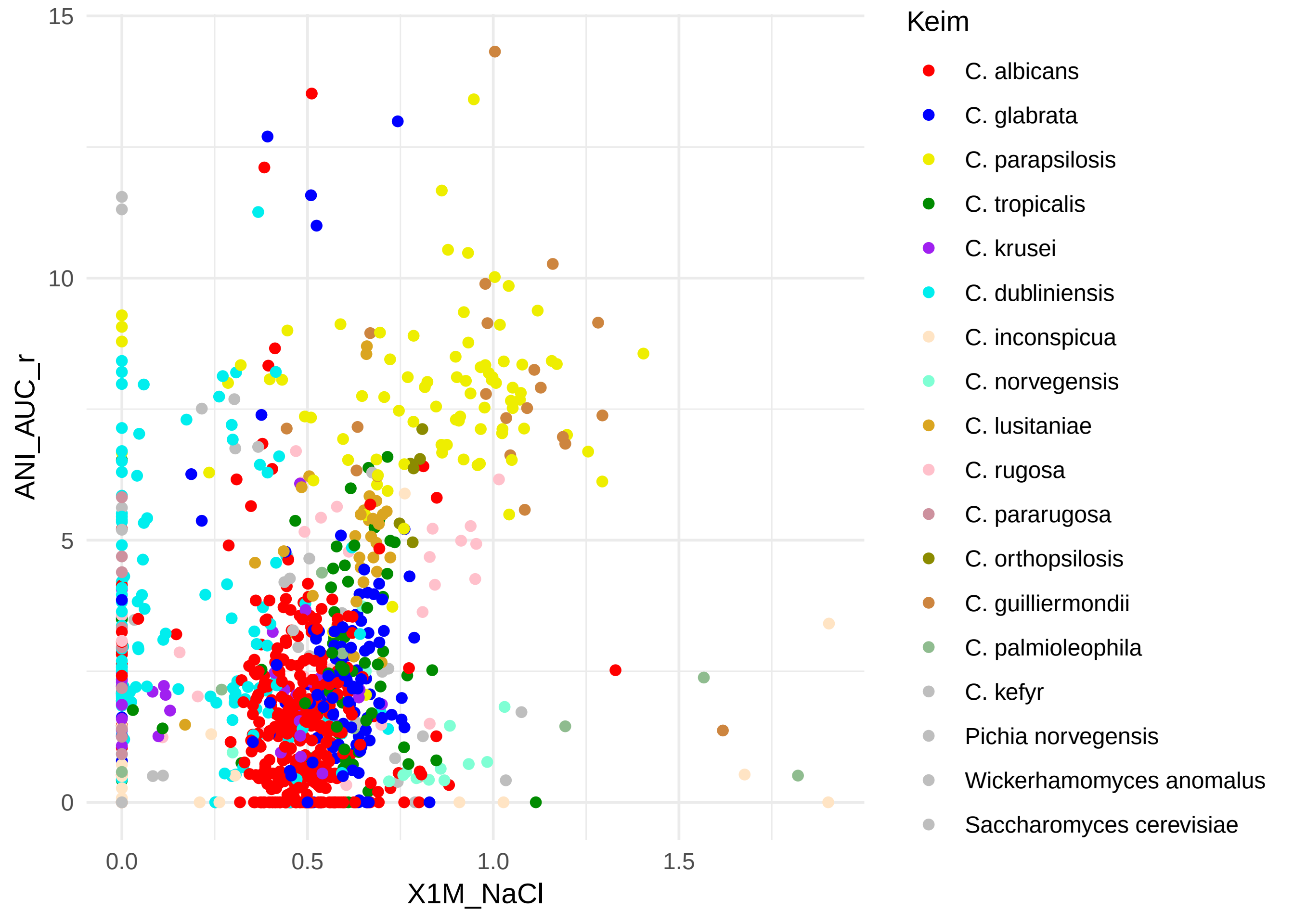
Keim
C. albicans
C. glabrata
C. parapsilosis
C. tropicalis
C. krusei
C. dubliniensis
C. inconspicua
C. norvegensis
C. lusitaniae
C. rugosa
C. pararugosa
C. orthopsilosis
C. guilliermondii
C. palmioleophila
C. kefyr
Pichia norvegensis
Wickerhamomyces anomalus
Saccharomyces cerevisiae
ANI_AUC_r
X1M_NaCl
0
5
10
15
0.0
0.5
1.0
1.5

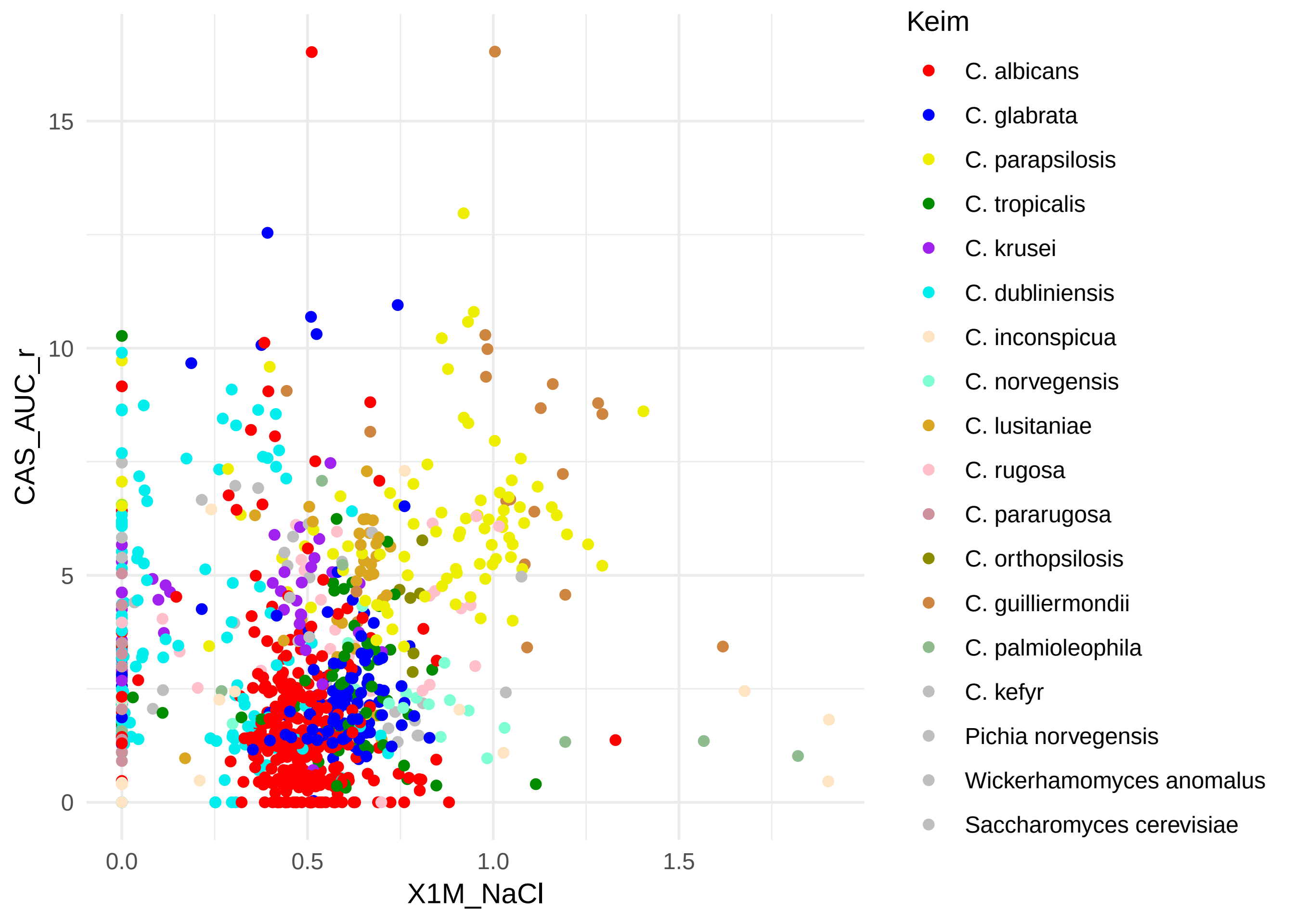
CAS_AUC_r
15
10
5
0
0.0
0.5
1.0
1.5
X1M_NaCl
Keim
C. albicans
C. glabrata
C. parapsilosis
C. tropicalis
C. krusei
C. dubliniensis
C. inconspicua
C. norvegensis
C. lusitaniae
C. rugosa
C. pararugosa
C. orthopsilosis
C. guilliermondii
C. palmioleophila
C. kefyr
Pichia norvegensis
Wickerhamomyces anomalus
Saccharomyces cerevisiae

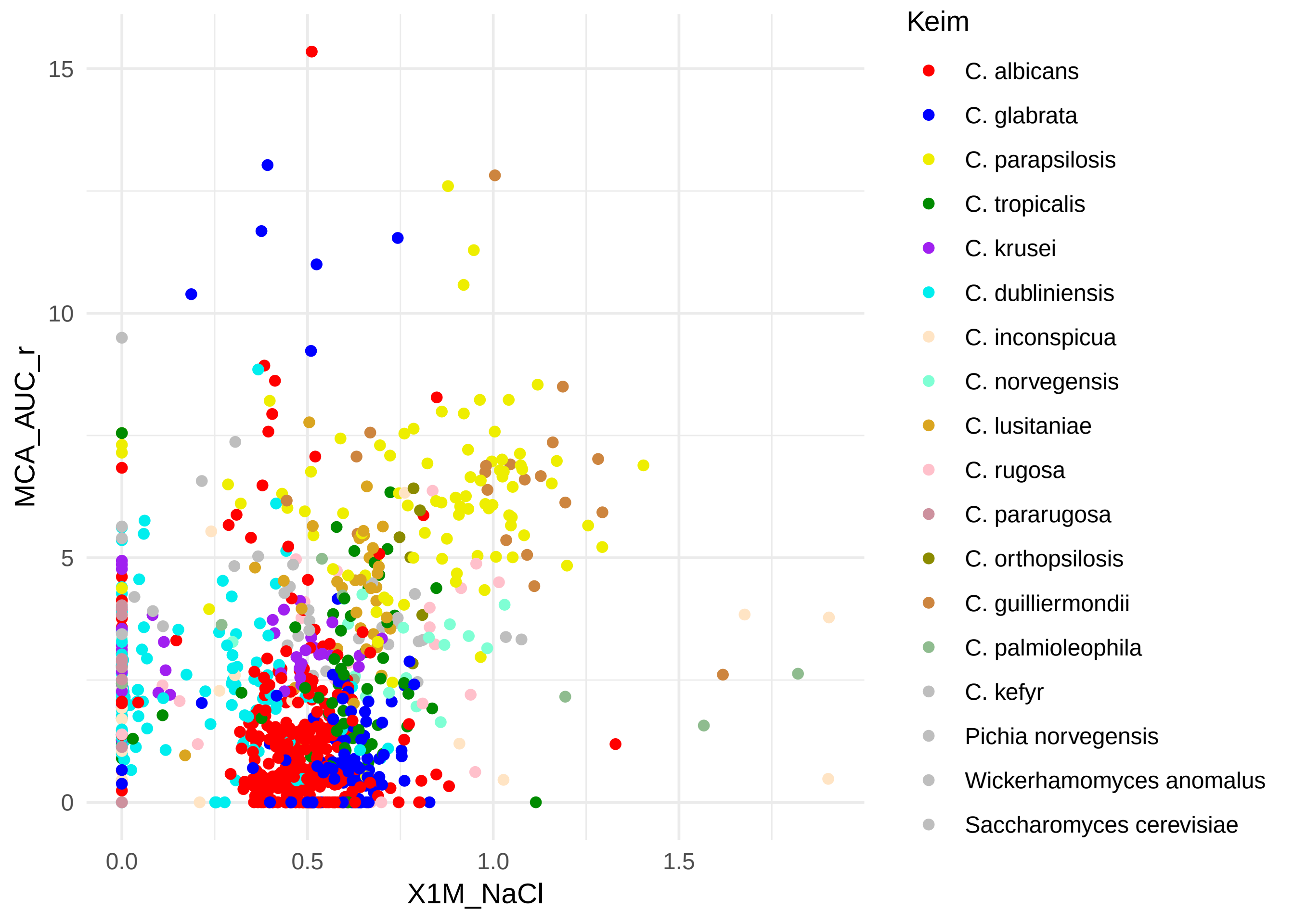

MCA_AUC_r
15
10
5
0
0.0
0.5
1.0
1.5
X1M_NaCl
Keim
C. albicans
C. glabrata
C. parapsilosis
C. tropicalis
C. krusei
C. dubliniensis
C. inconspicua
C. norvegensis
C. lusitaniae
C. rugosa
C. pararugosa
C. orthopsilosis
C. guilliermondii
C. palmioleophila
C. kefyr
Pichia norvegensis
Wickerhamomyces anomalus
Saccharomyces cerevisiae

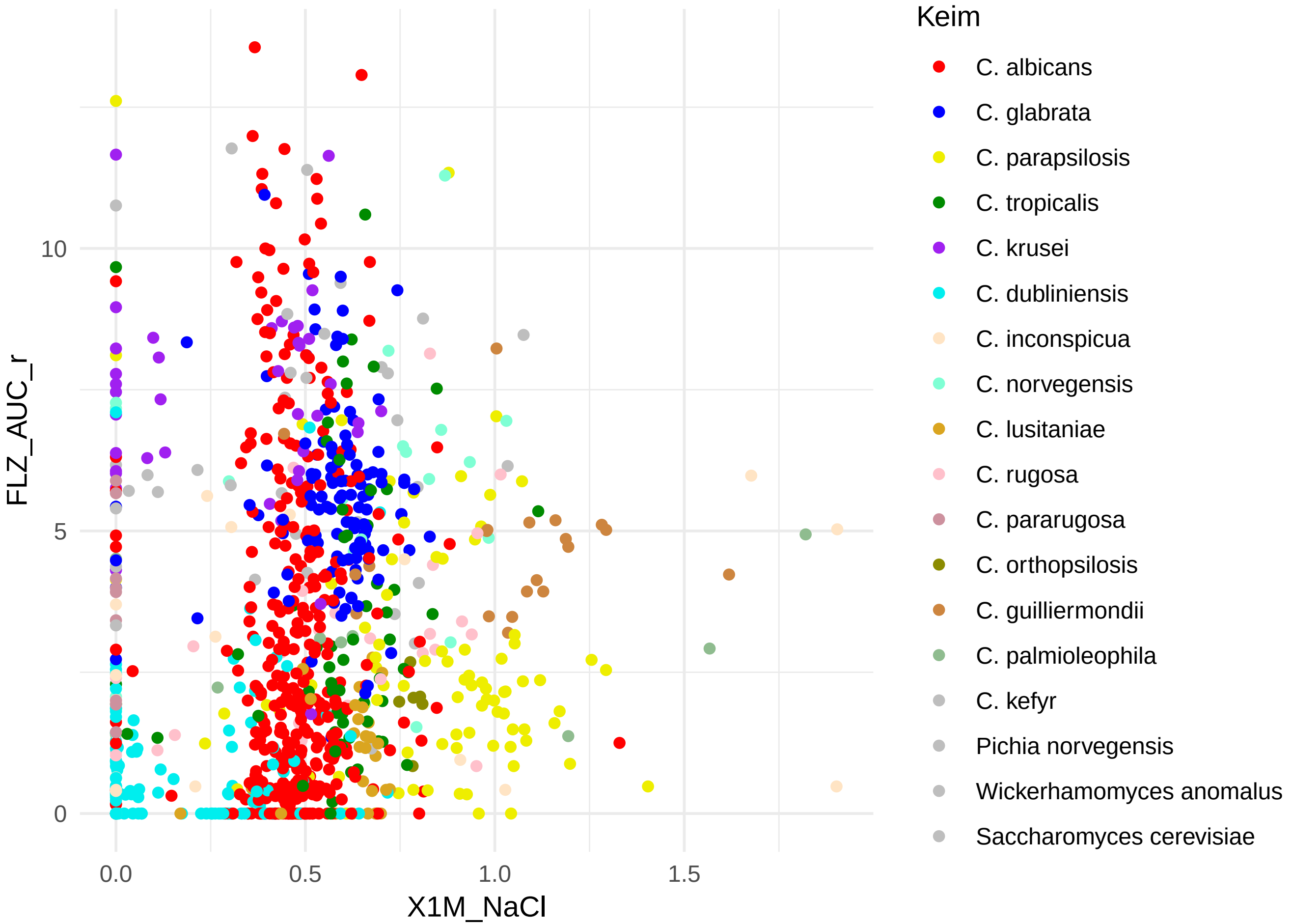

Keim
C. albicans
C. glabrata
C. parapsilosis
C. tropicalis
C. krusei
C. dubliniensis
C. inconspicua
C. norvegensis
C. lusitaniae
C. rugosa
C. pararugosa
C. orthopsilosis
C. guilliermondii
C. palmioleophila
C. kefyr
Pichia norvegensis
Wickerhamomyces anomalus
Saccharomyces cerevisiae
FLZ_AUC_r
10
5
0
0.0
0.5
1.0
1.5
X1M_NaCl

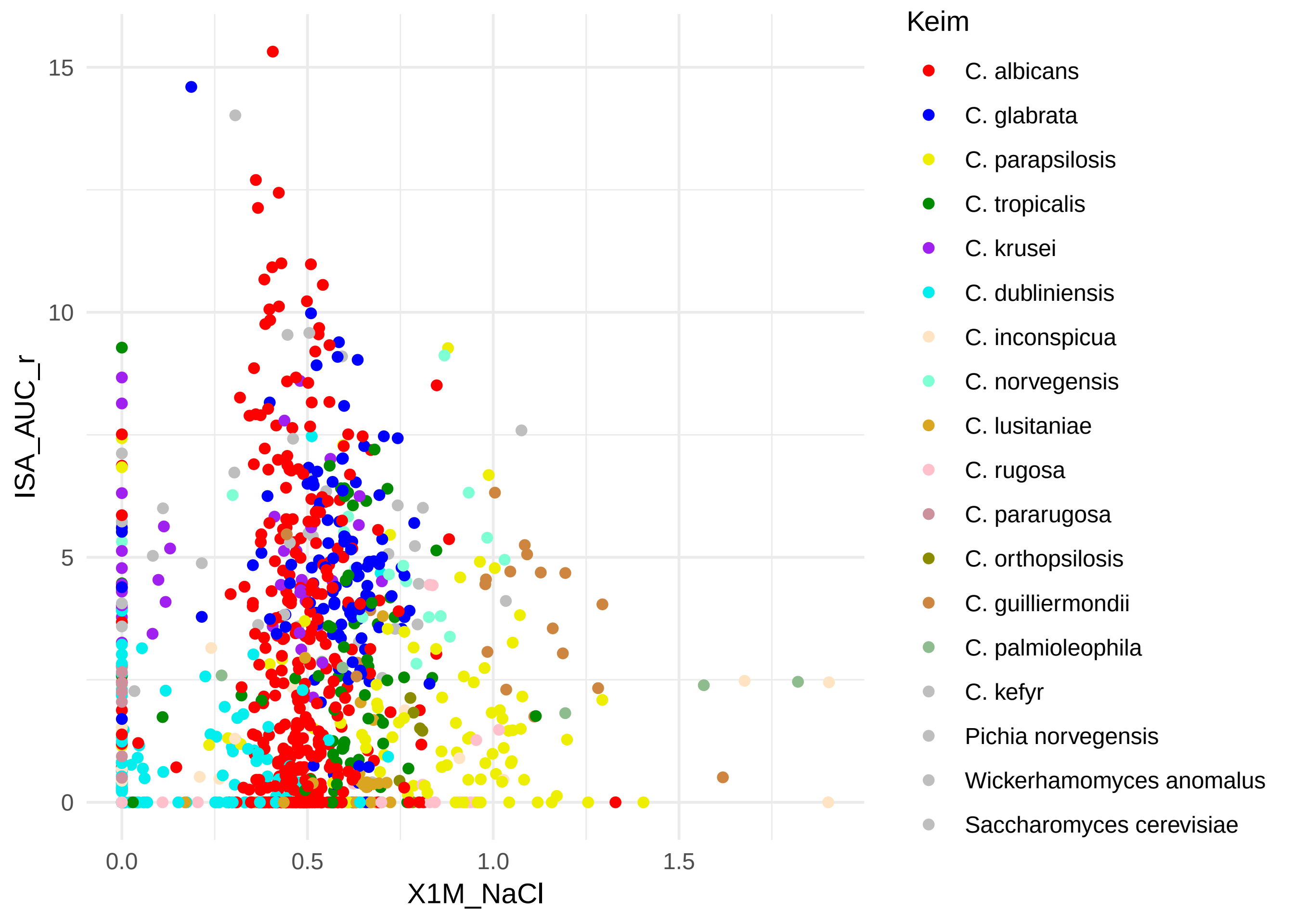

ISA_AUC_r
X1M_NaCl
0
5
10
15
0.0
0.5
1.0
1.5
Keim
C. albicans
C. glabrata
C. parapsilosis
C. tropicalis
C. krusei
C. dubliniensis
C. inconspicua
C. norvegensis
C. lusitaniae
C. rugosa
C. pararugosa
C. orthopsilosis
C. guilliermondii
C. palmioleophila
C. kefyr
Pichia norvegensis
Wickerhamomyces anomalus
Saccharomyces cerevisiae

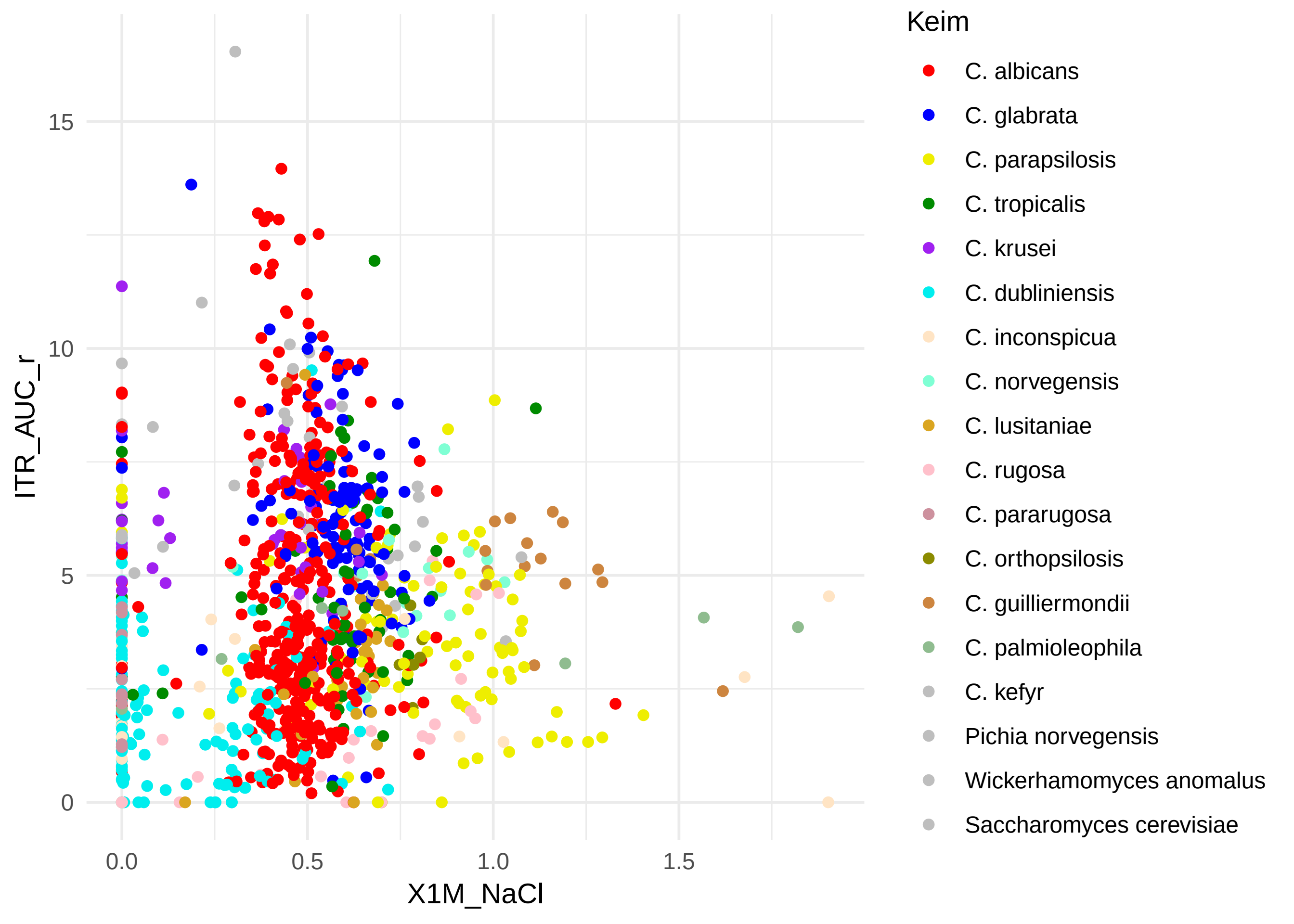

Keim
C. albicans
C. glabrata
C. parapsilosis
C. tropicalis
C. krusei
C. dubliniensis
C. inconspicua
C. norvegensis
C. lusitaniae
C. rugosa
C. pararugosa
C. orthopsilosis
C. guilliermondii
C. palmioleophila
C. kefyr
Pichia norvegensis
Wickerhamomyces anomalus
Saccharomyces cerevisiae
ITR_AUC_r
X1M_NaCl
0
5
10
15
0.0
0.5
1.0
1.5

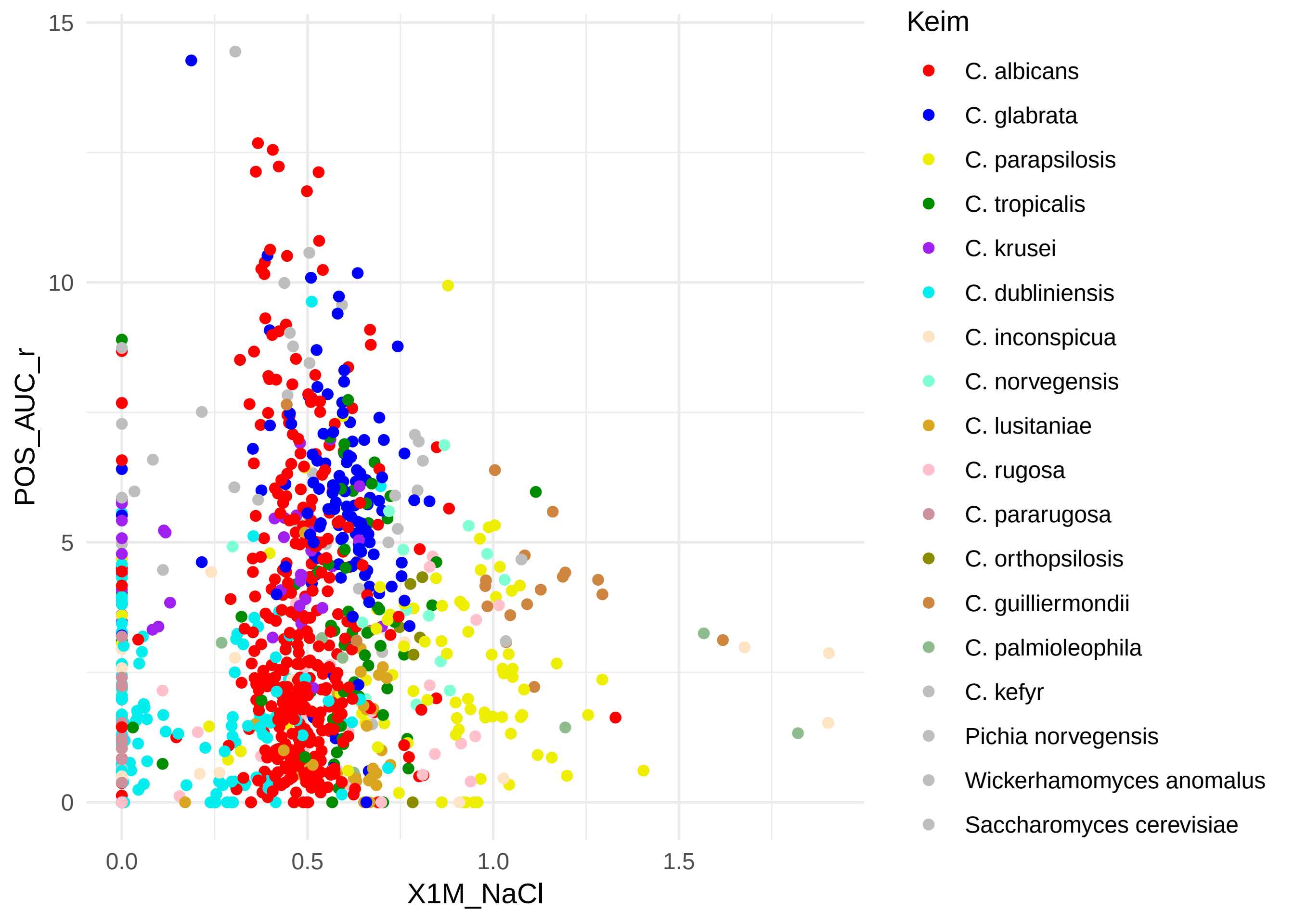
15
10
5
0
POS_AUC_r
0.0
0.5
1.0
1.5
X1M_NaCl
Keim
C. albicans
C. glabrata
C. parapsilosis
C. tropicalis
C. krusei
C. dubliniensis
C. inconspicua
C. norvegensis
C. lusitaniae
C. rugosa
C. pararugosa
C. orthopsilosis
C. guilliermondii
C. palmioleophila
C. kefyr
Pichia norvegensis
Wickerhamomyces anomalus
Saccharomyces cerevisiae

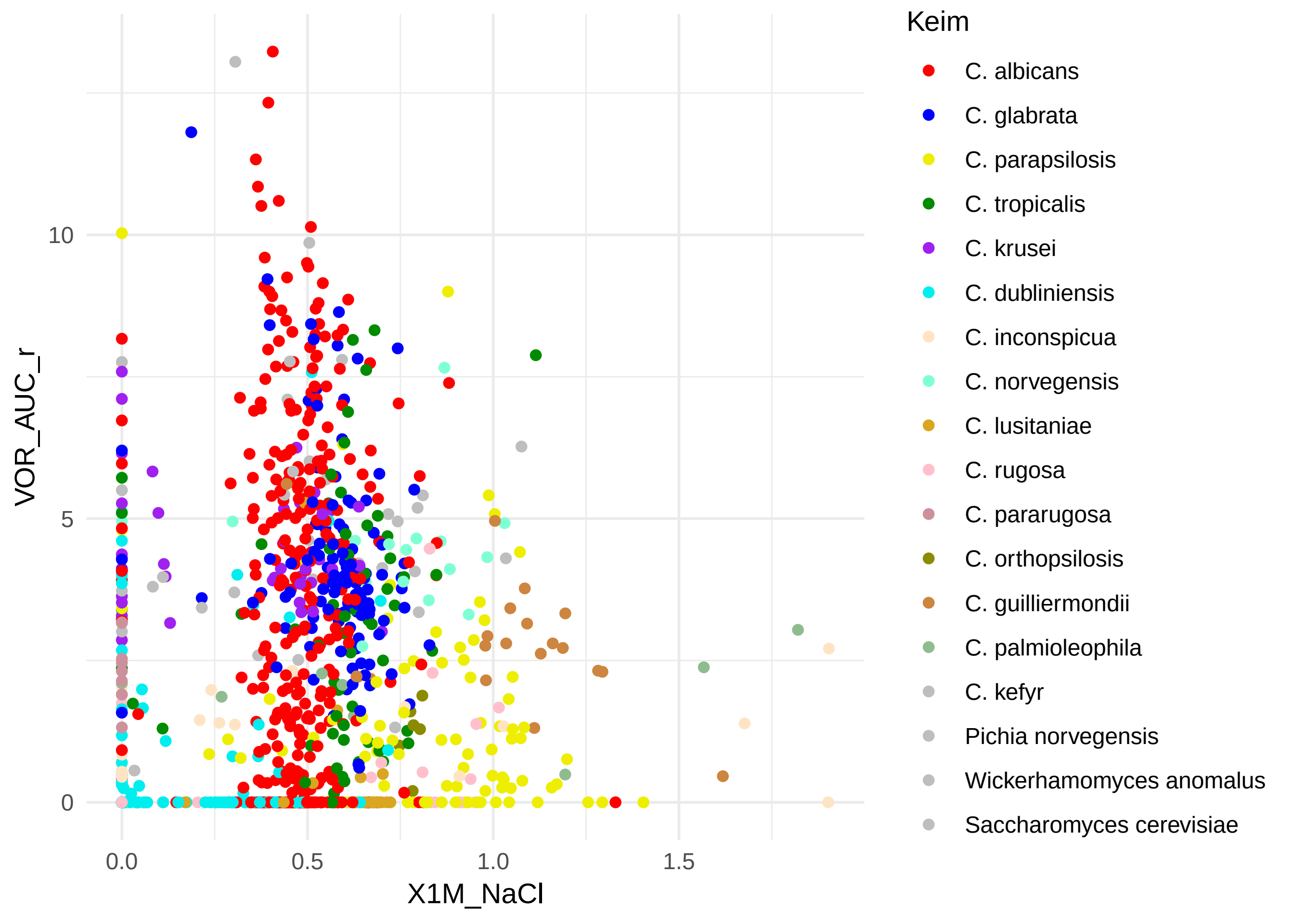

Keim
C. albicans
C. glabrata
C. parapsilosis
C. tropicalis
C. krusei
C. dubliniensis
C. inconspicua
C. norvegensis
C. lusitaniae
C. rugosa
C. pararugosa
C. orthopsilosis
C. guilliermondii
C. palmioleophila
C. kefyr
Pichia norvegensis
Wickerhamomyces anomalus
Saccharomyces cerevisiae
VOR_AUC_r
10
5
0
0.0
0.5
1.0
1.5
X1M_NaCl

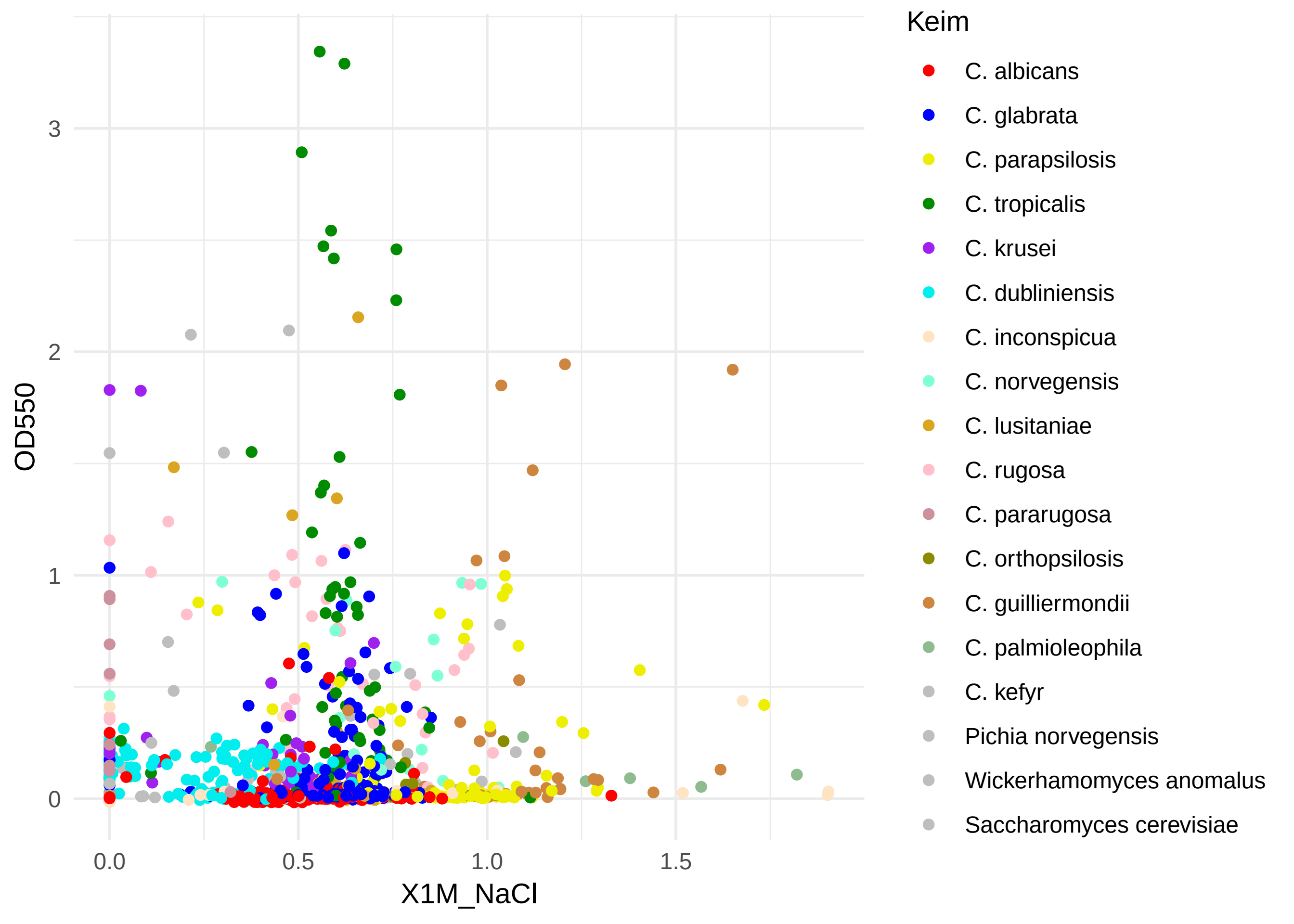

OD550
X1M_NaCl
0
1
2
3
0.0
0.5
1.0
1.5
Keim
C. albicans
C. glabrata
C. parapsilosis
C. tropicalis
C. krusei
C. dubliniensis
C. inconspicua
C. norvegensis
C. lusitaniae
C. rugosa
C. pararugosa
C. orthopsilosis
C. guilliermondii
C. palmioleophila
C. kefyr
Pichia norvegensis
Wickerhamomyces anomalus
Saccharomyces cerevisiae

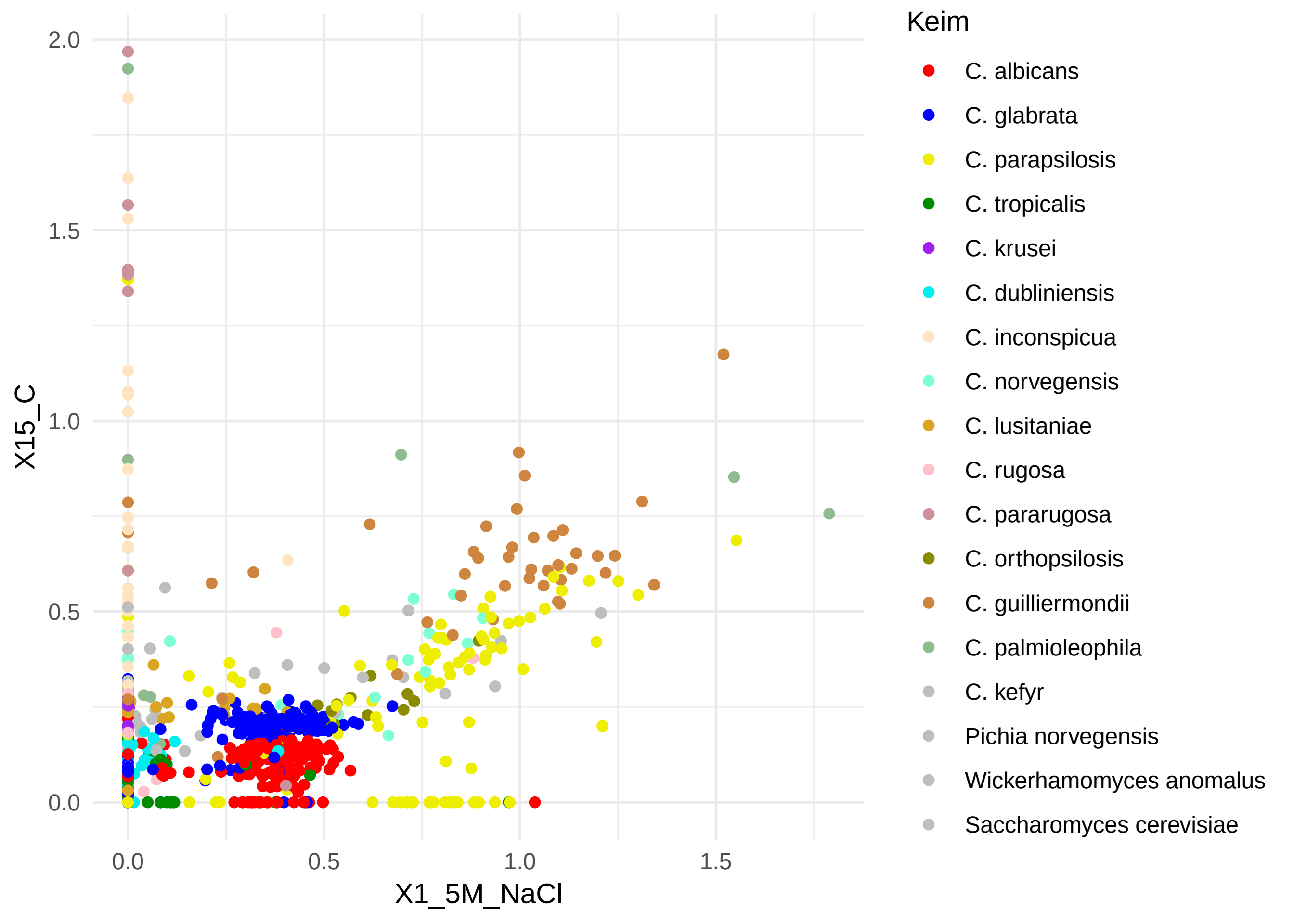

Keim
C. albicans
C. glabrata
C. parapsilosis
C. tropicalis
C. krusei
C. dubliniensis
C. inconspicua
C. norvegensis
C. lusitaniae
C. rugosa
C. pararugosa
C. orthopsilosis
C. guilliermondii
C. palmioleophila
C. kefyr
Pichia norvegensis
Wickerhamomyces anomalus
Saccharomyces cerevisiae
X15_C
2.0
1.5
1.0
0.5
0.0
0.0
0.5
1.0
1.5
X1_5M_NaCl

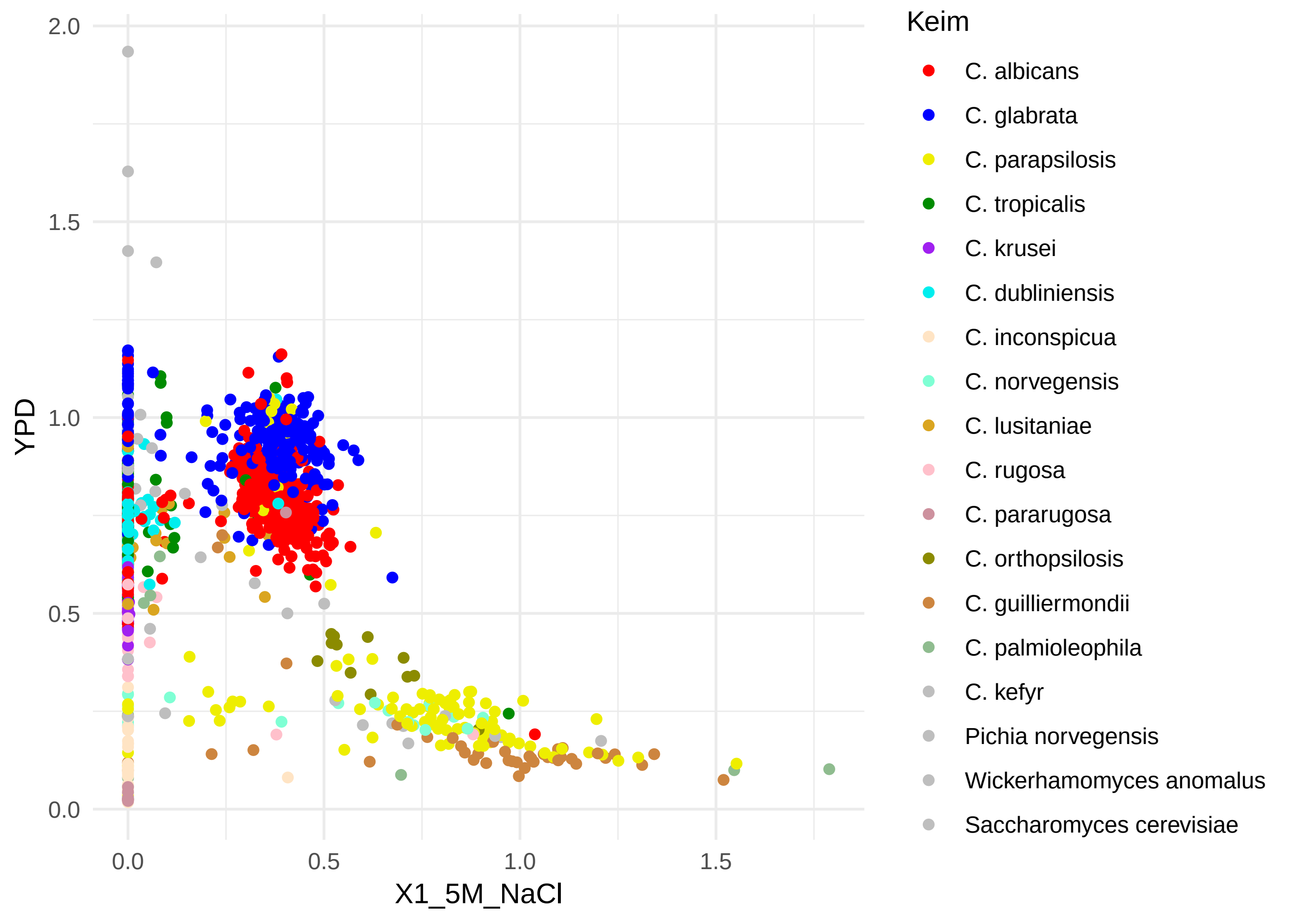
2.0
1.5
1.0
0.5
0.0
YPD
0.0
0.5
1.0
1.5
X1_5M_NaCl
Keim
C. albicans
C. glabrata
C. parapsilosis
C. tropicalis
C. krusei
C. dubliniensis
C. inconspicua
C. norvegensis
C. lusitaniae
C. rugosa
C. pararugosa
C. orthopsilosis
C. guilliermondii
C. palmioleophila
C. kefyr
Pichia norvegensis
Wickerhamomyces anomalus
Saccharomyces cerevisiae

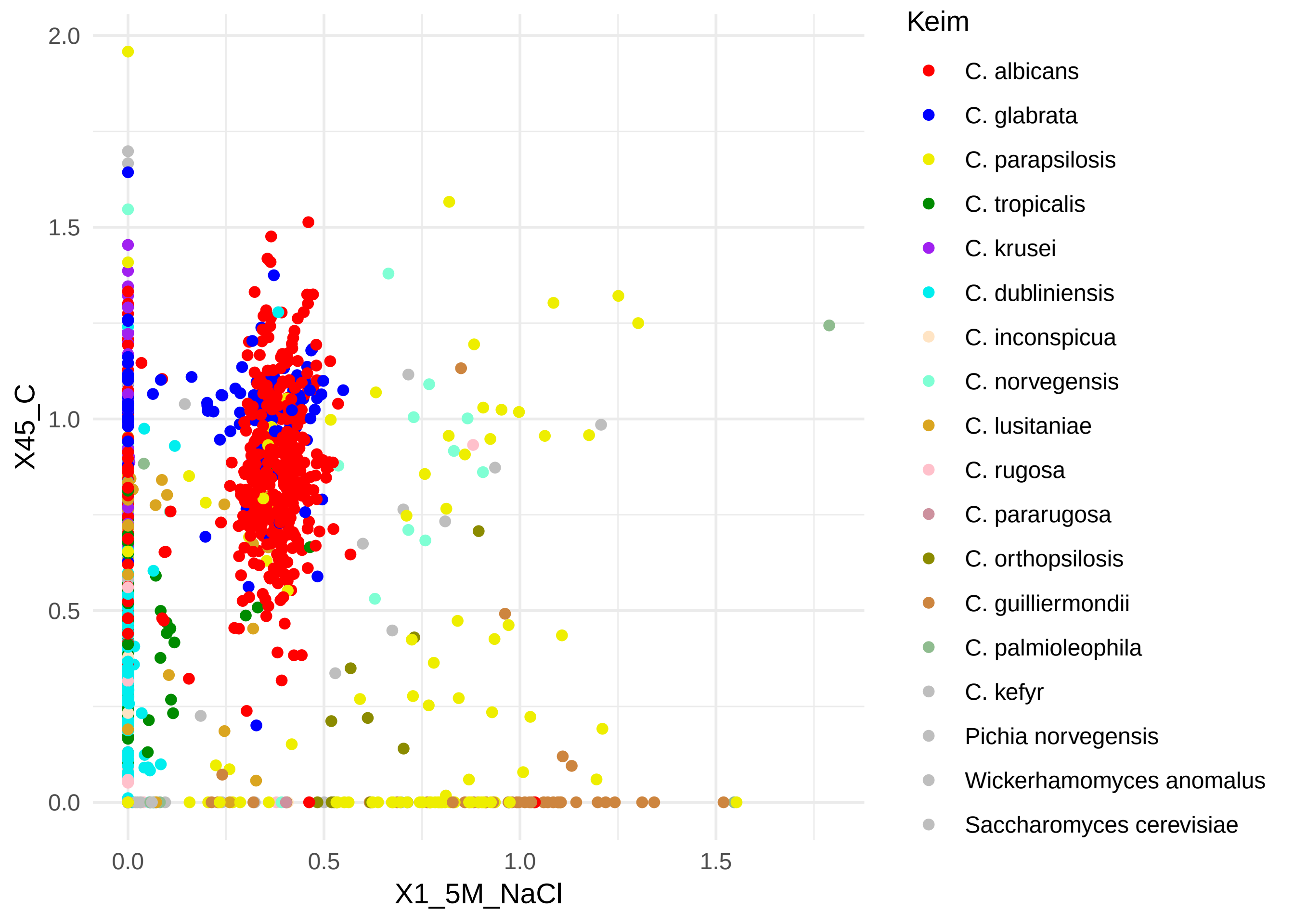
Keim
C. albicans
C. glabrata
C. parapsilosis
C. tropicalis
C. krusei
C. dubliniensis
C. inconspicua
C. norvegensis
C. lusitaniae
C. rugosa
C. pararugosa
C. orthopsilosis
C. guilliermondii
C. palmioleophila
C. kefyr
Pichia norvegensis
Wickerhamomyces anomalus
Saccharomyces cerevisiae
X45_C
X1_5M_NaCl
0.0
0.5
1.0
1.5
2.0

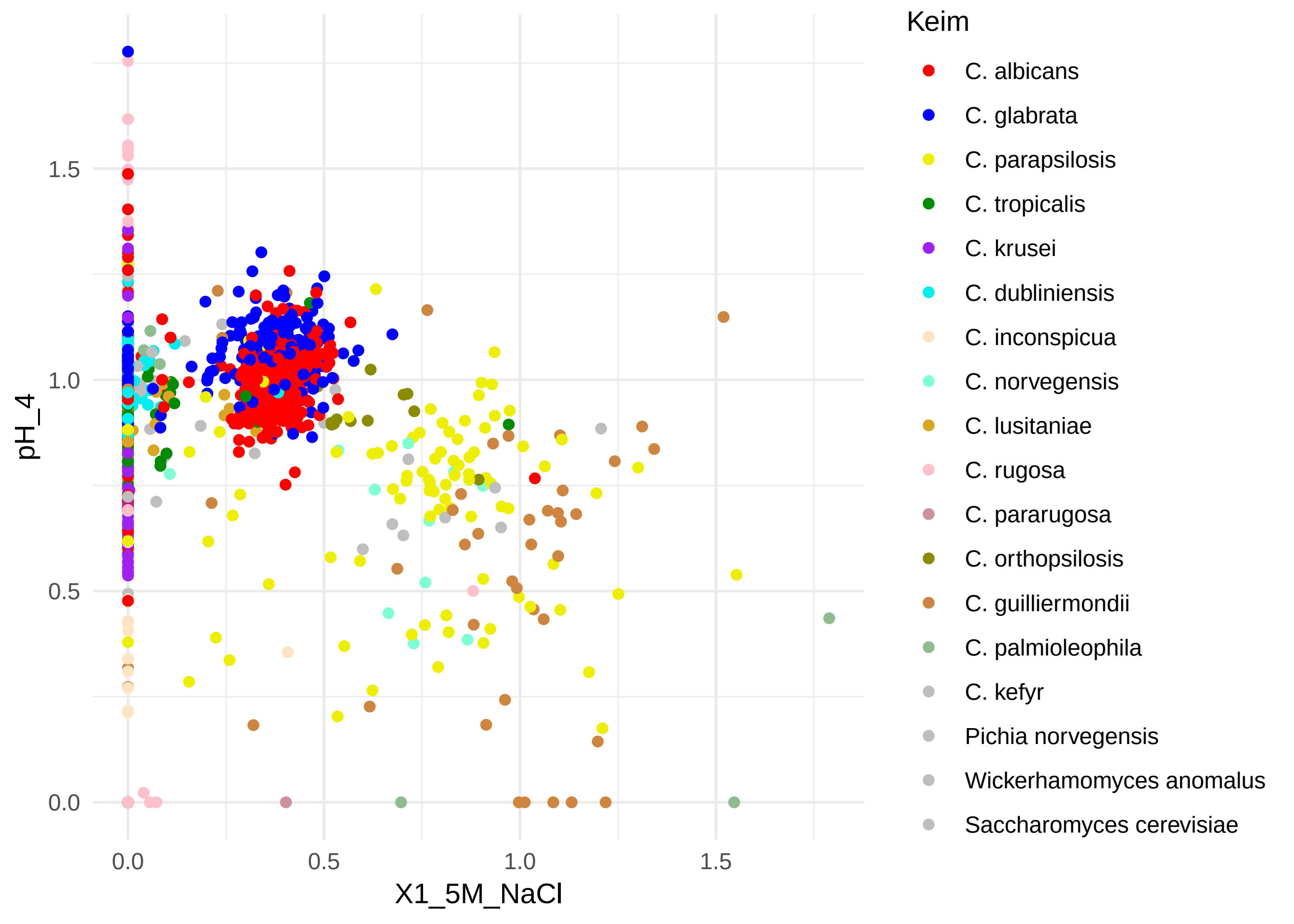

Keim
C. albicans
C. glabrata
C. parapsilosis
C. tropicalis
C. krusei
C. dubliniensis
C. inconspicua
C. norvegensis
C. lusitaniae
C. rugosa
C. pararugosa
C. orthopsilosis
C. guilliermondii
C. palmioleophila
C. kefyr
Pichia norvegensis
Wickerhamomyces anomalus
Saccharomyces cerevisiae
pH_4
X1_5M_NaCl
0.0
0.5
1.0
1.5

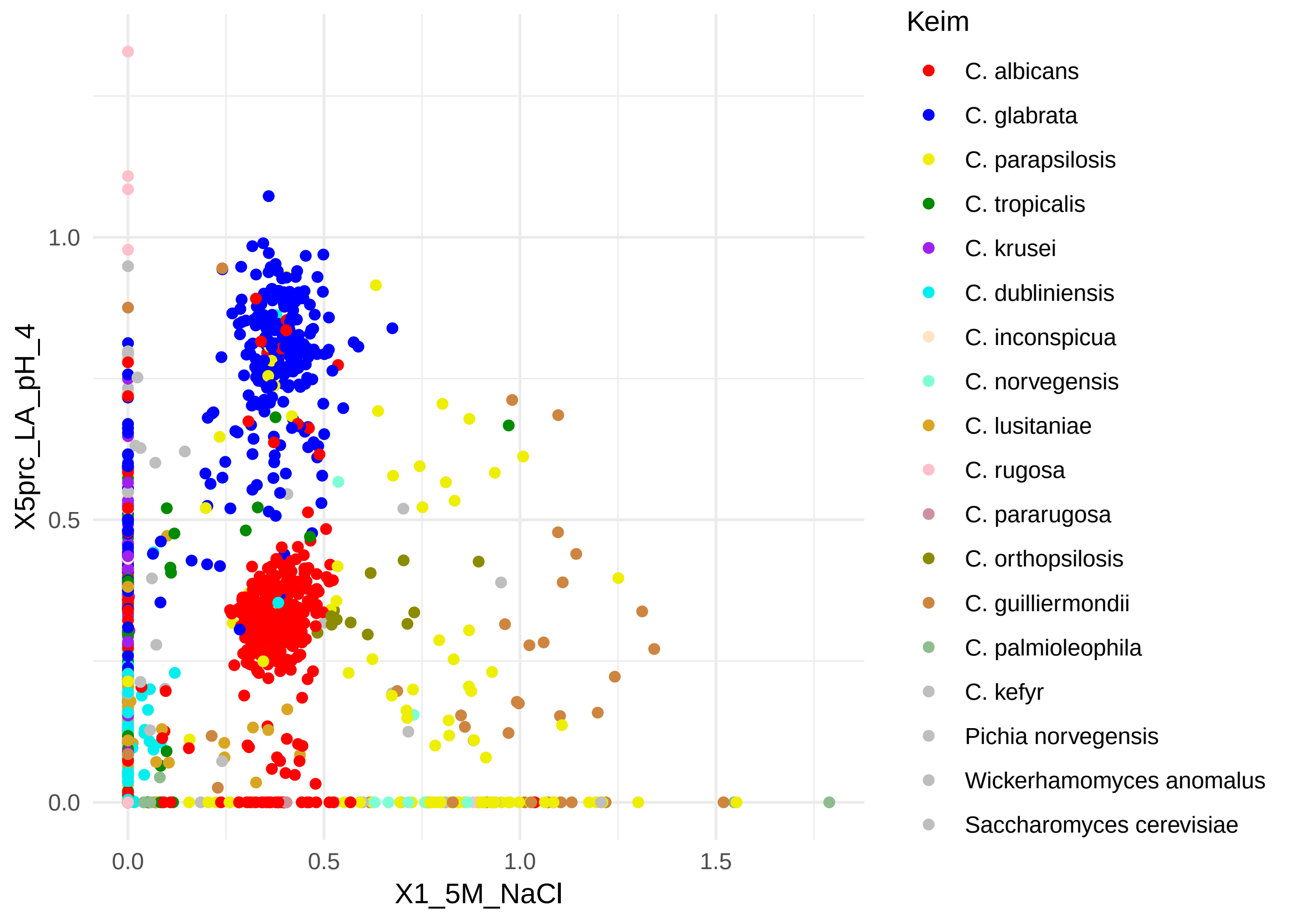

Keim
C. albicans
C. glabrata
C. parapsilosis
C. tropicalis
C. krusei
C. dubliniensis
C. inconspicua
C. norvegensis
C. lusitaniae
C. rugosa
C. pararugosa
C. orthopsilosis
C. guilliermondii
C. palmioleophila
C. kefyr
Pichia norvegensis
Wickerhamomyces anomalus
Saccharomyces cerevisiae
X5prc_LA_pH_4
1.0
0.5
0.0
0.0
0.5
1.0
1.5
X1_5M_NaCl

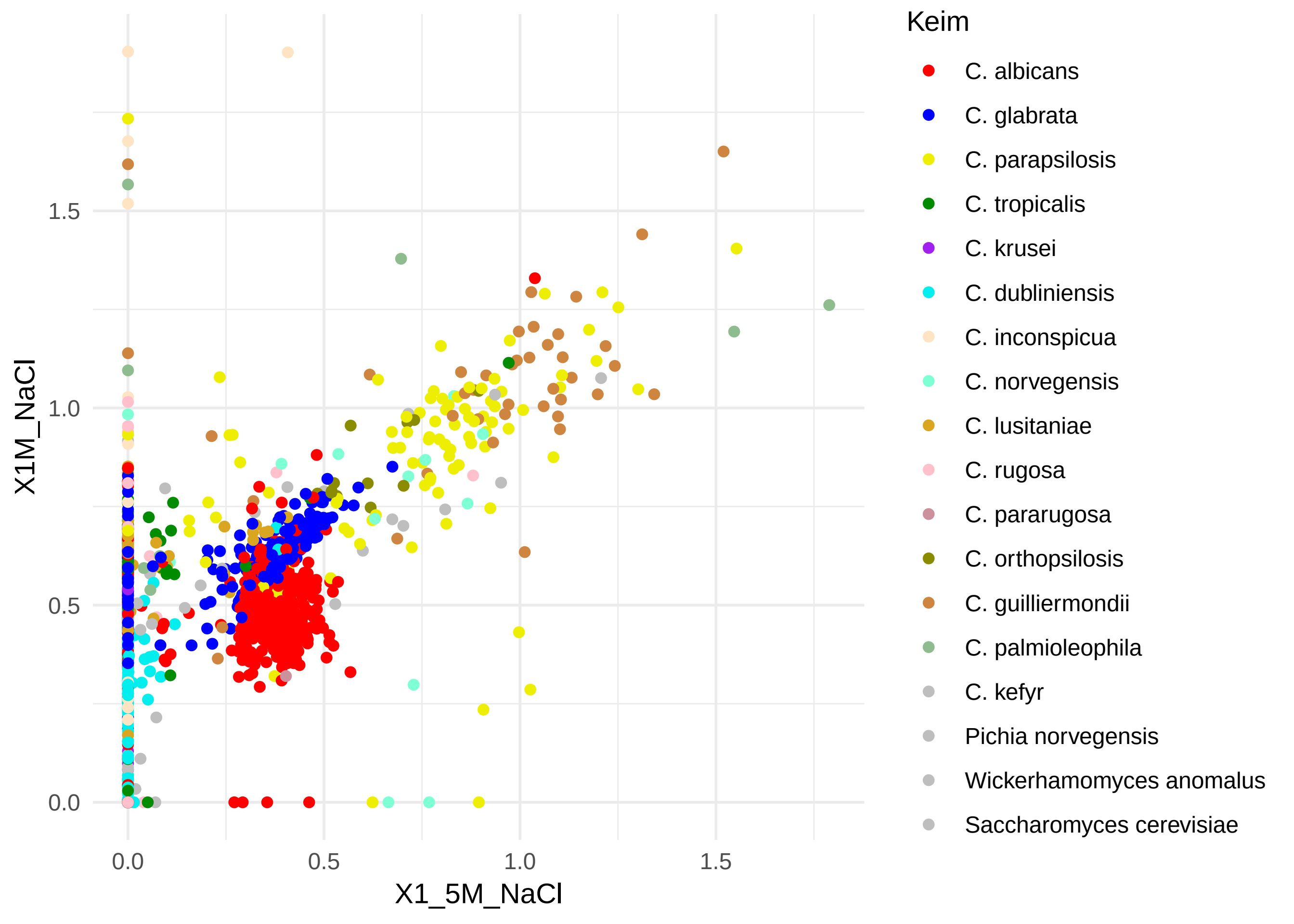
Keim
C. albicans
C. glabrata
C. parapsilosis
C. tropicalis
C. krusei
C. dubliniensis
C. inconspicua
C. norvegensis
C. lusitaniae
C. rugosa
C. pararugosa
C. orthopsilosis
C. guilliermondii
C. palmioleophila
C. kefyr
Pichia norvegensis
Wickerhamomyces anomalus
Saccharomyces cerevisiae
X1M_NaCl
X1_5M_NaCl
0.0
0.5
1.0
1.5

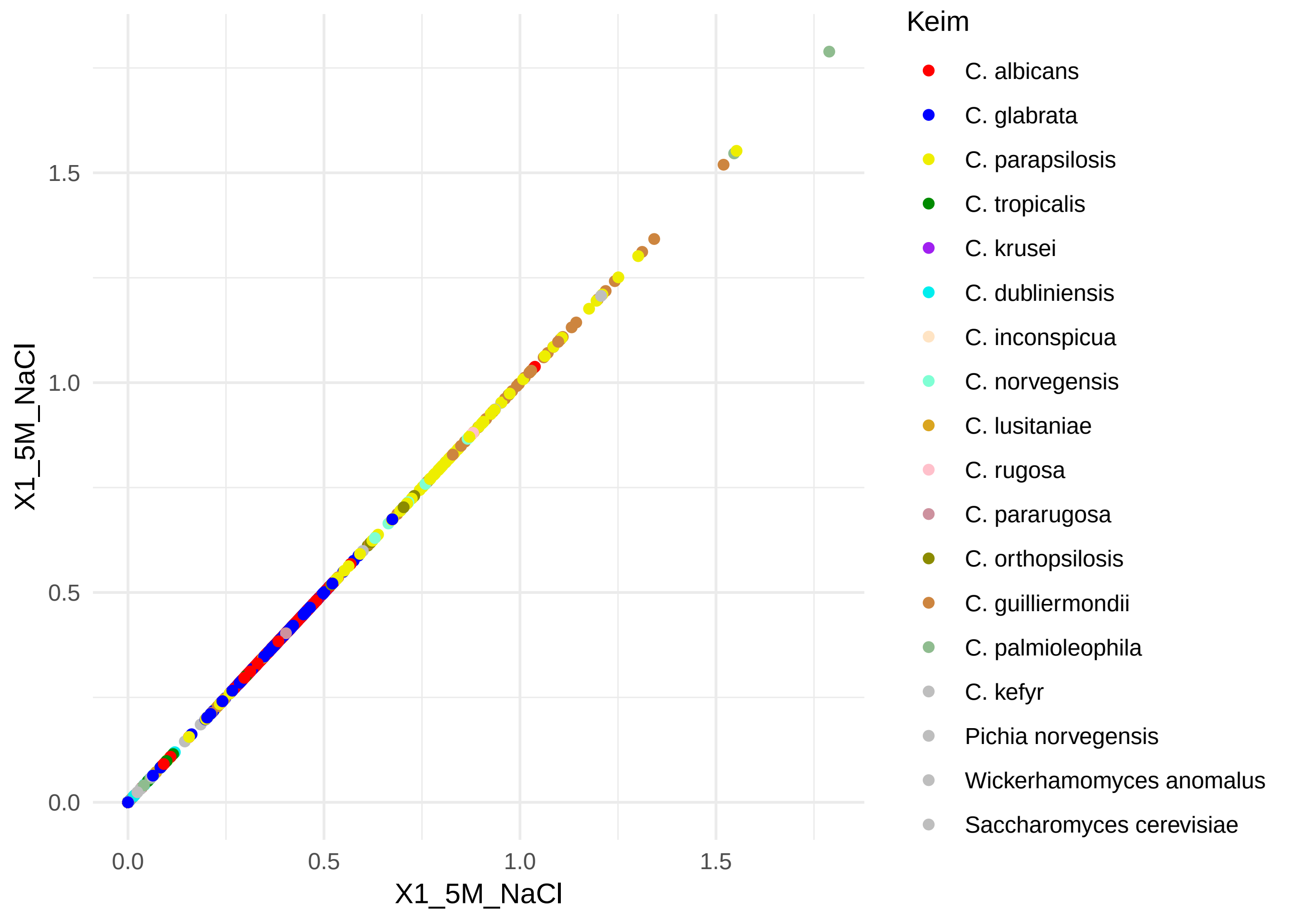
Keim
C. albicans
C. glabrata
C. parapsilosis
C. tropicalis
C. krusei
C. dubliniensis
C. inconspicua
C. norvegensis
C. lusitaniae
C. rugosa
C. pararugosa
C. orthopsilosis
C. guilliermondii
C. palmioleophila
C. kefyr
Pichia norvegensis
Wickerhamomyces anomalus
Saccharomyces cerevisiae
X1_5M_NaCl
X1_5M_NaCl
0.0
0.5
1.0
1.5
0.0
0.5
1.0
1.5

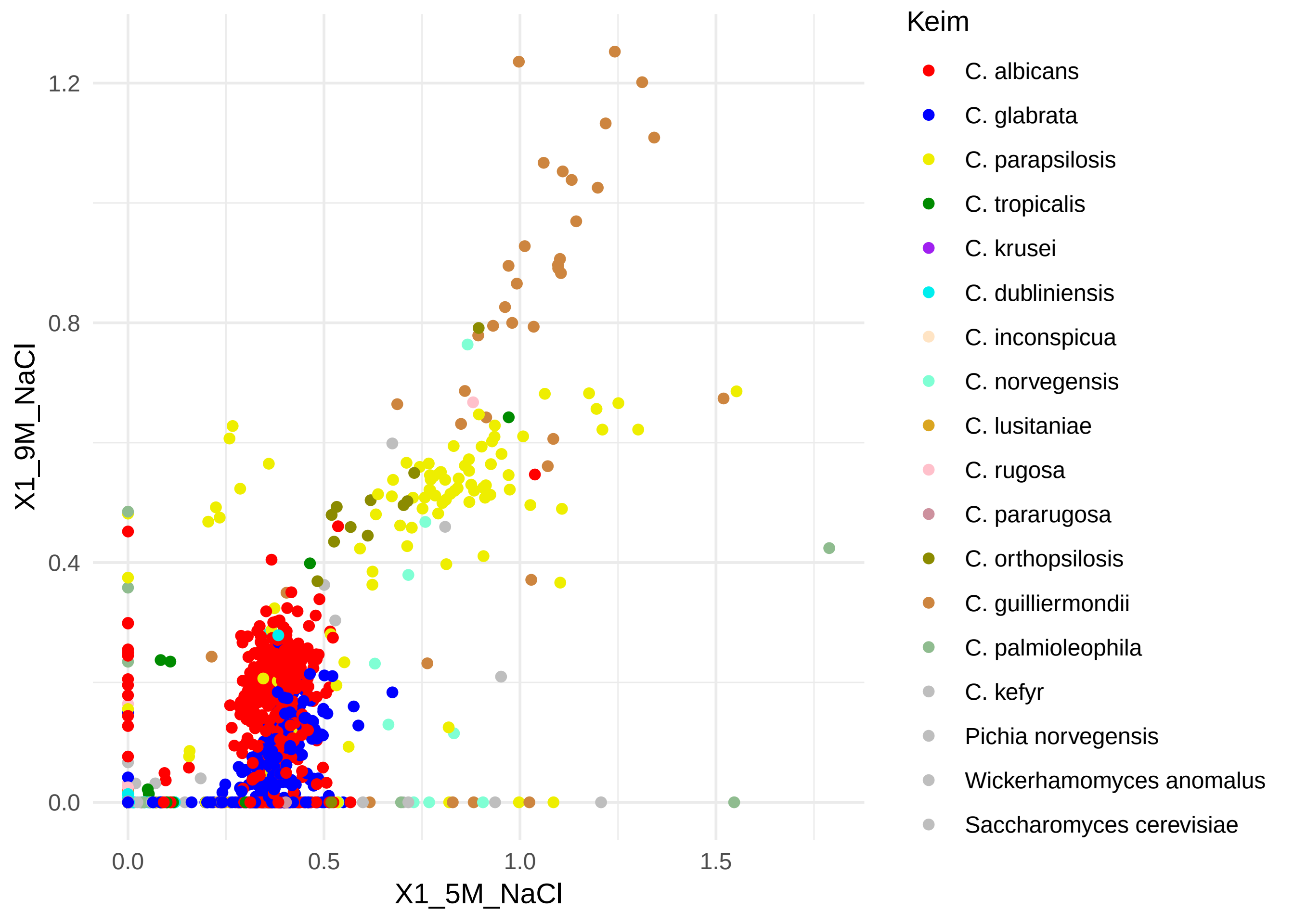

Keim
C. albicans
C. glabrata
C. parapsilosis
C. tropicalis
C. krusei
C. dubliniensis
C. inconspicua
C. norvegensis
C. lusitaniae
C. rugosa
C. pararugosa
C. orthopsilosis
C. guilliermondii
C. palmioleophila
C. kefyr
Pichia norvegensis
Wickerhamomyces anomalus
Saccharomyces cerevisiae
X1_9M_NaCl
X1_5M_NaCl
0.0
0.4
0.8
1.2
0.0
0.5
1.0
1.5

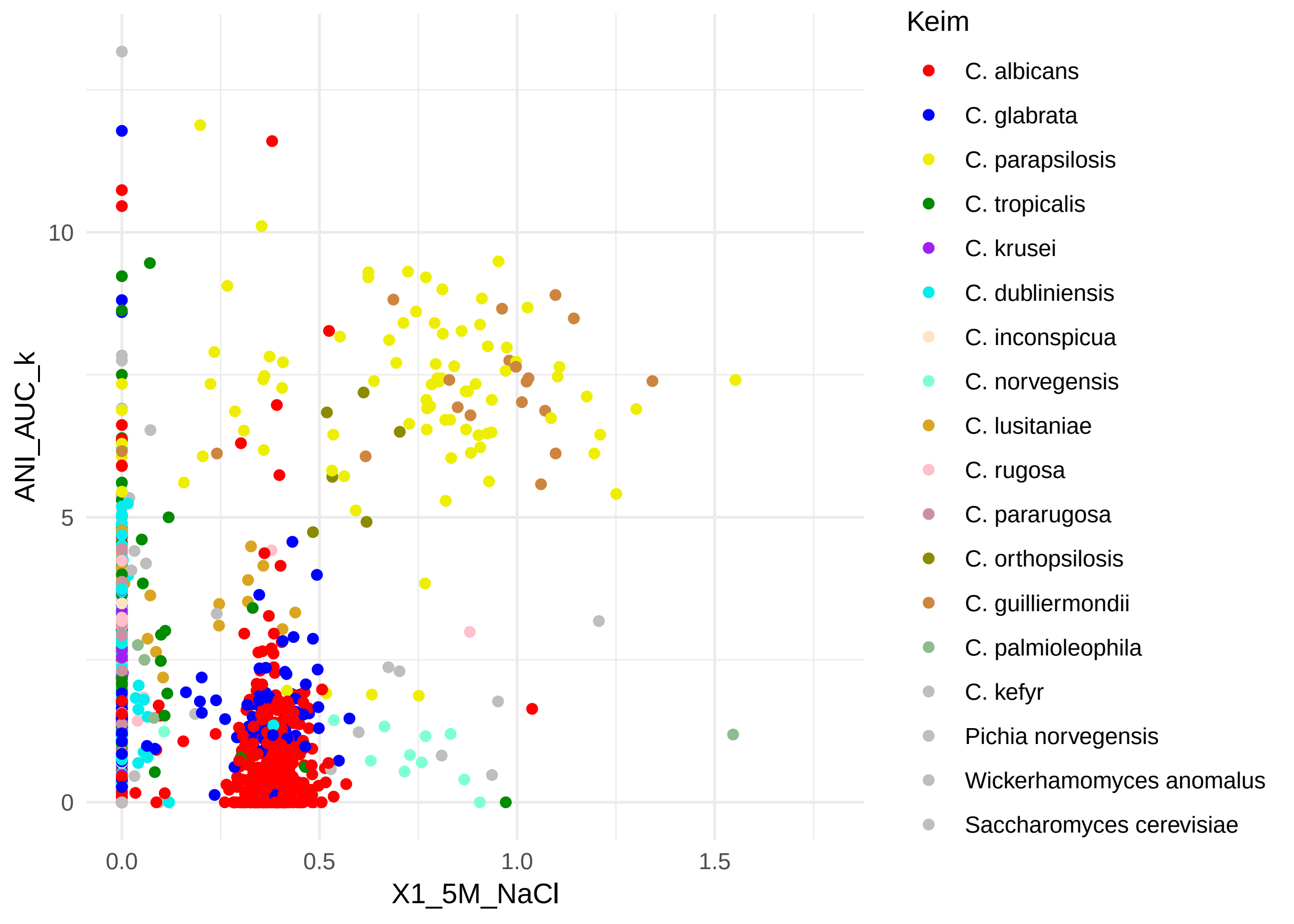

Keim
C. albicans
C. glabrata
C. parapsilosis
C. tropicalis
C. krusei
C. dubliniensis
C. inconspicua
C. norvegensis
C. lusitaniae
C. rugosa
C. pararugosa
C. orthopsilosis
C. guilliermondii
C. palmioleophila
C. kefyr
Pichia norvegensis
Wickerhamomyces anomalus
Saccharomyces cerevisiae
ANI_AUC_k
X1_5M_NaCl
0
5
10
0.0
0.5
1.0
1.5

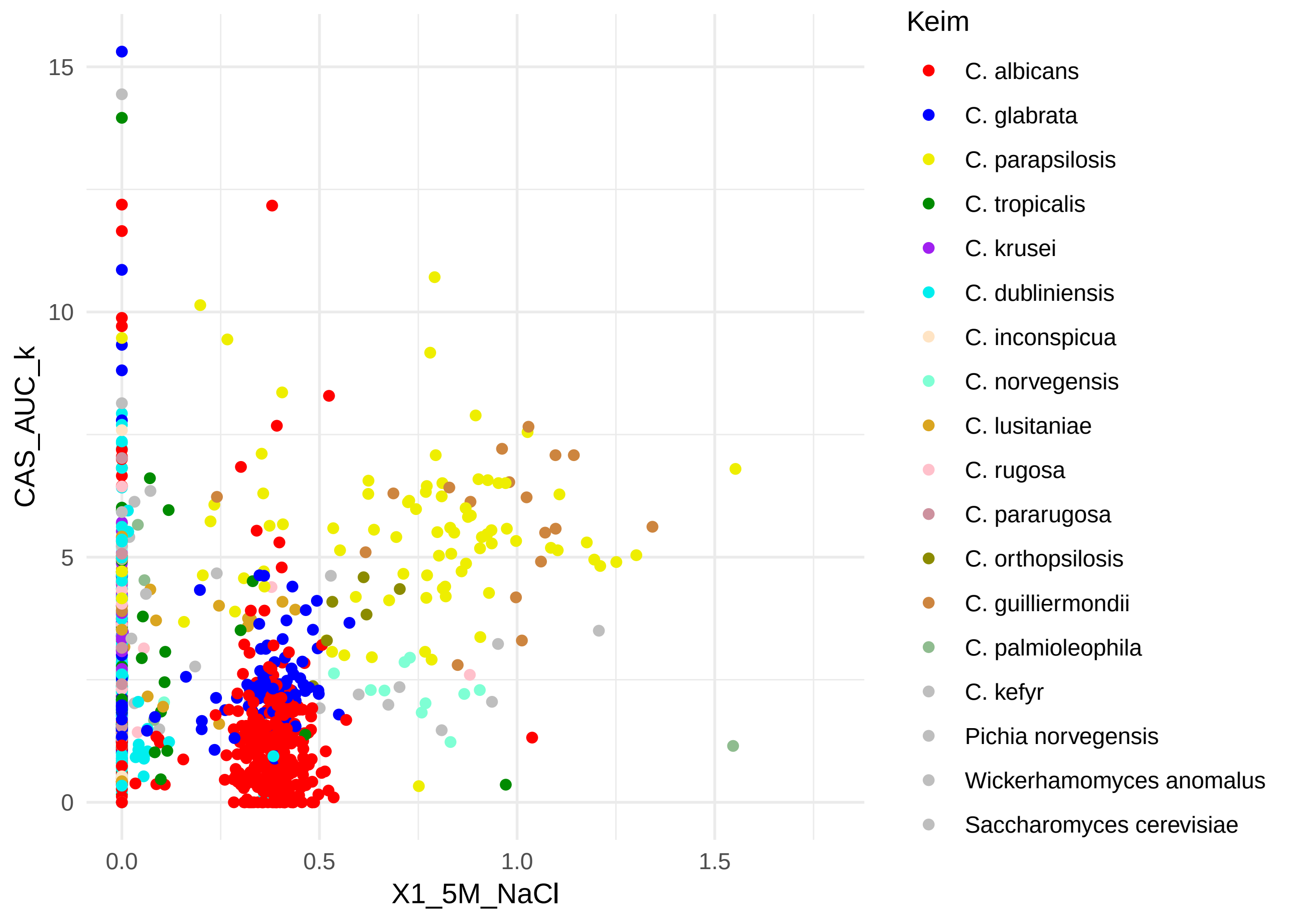

Keim
C. albicans
C. glabrata
C. parapsilosis
C. tropicalis
C. krusei
C. dubliniensis
C. inconspicua
C. norvegensis
C. lusitaniae
C. rugosa
C. pararugosa
C. orthopsilosis
C. guilliermondii
C. palmioleophila
C. kefyr
Pichia norvegensis
Wickerhamomyces anomalus
Saccharomyces cerevisiae
CAS_AUC_k
X1_5M_NaCl
0
5
10
15
0.0
0.5
1.0
1.5

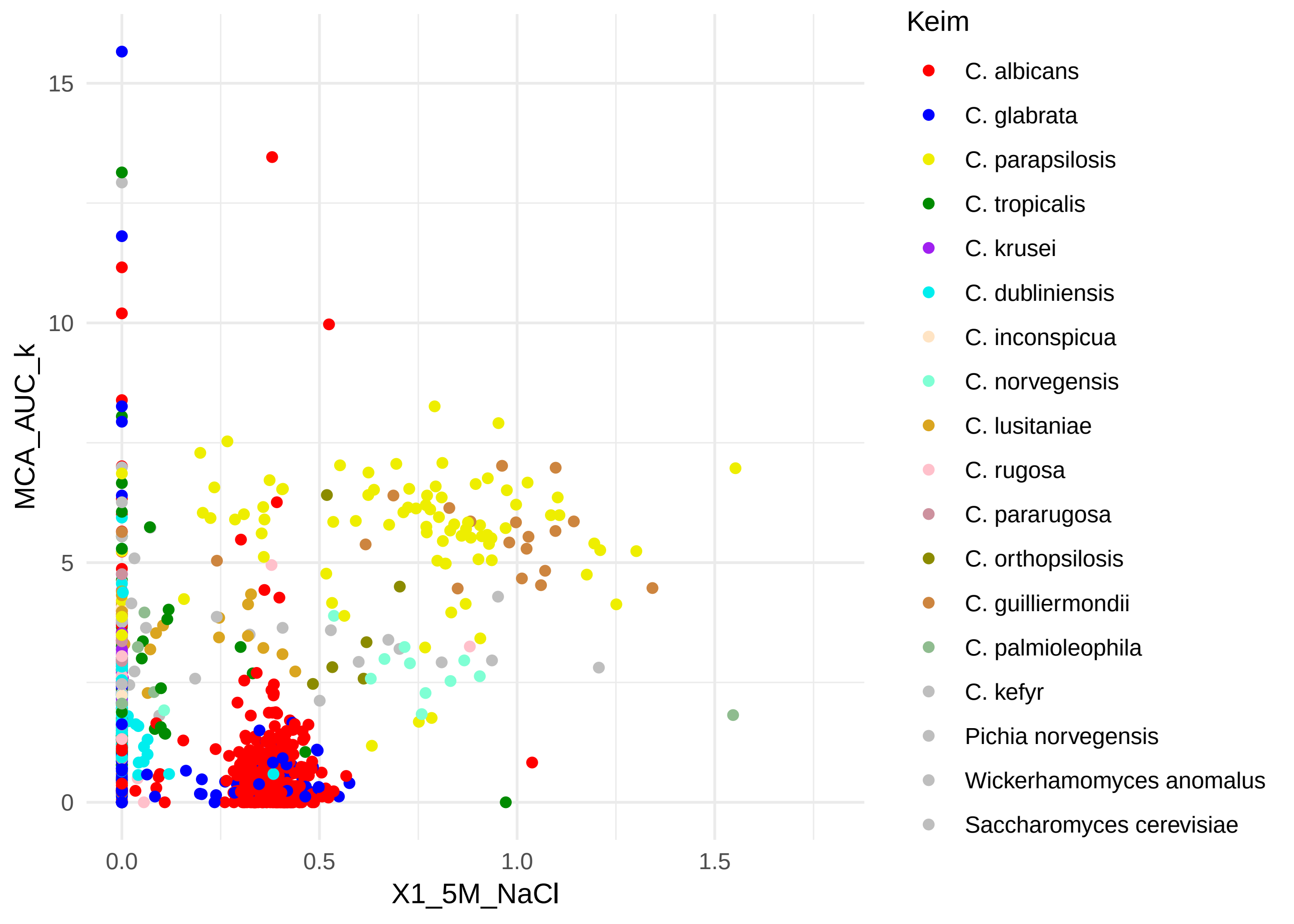

Keim
C. albicans
C. glabrata
C. parapsilosis
C. tropicalis
C. krusei
C. dubliniensis
C. inconspicua
C. norvegensis
C. lusitaniae
C. rugosa
C. pararugosa
C. orthopsilosis
C. guilliermondii
C. palmioleophila
C. kefyr
Pichia norvegensis
Wickerhamomyces anomalus
Saccharomyces cerevisiae
MCA_AUC_k
X1_5M_NaCl
0
5
10
15
0.0
0.5
1.0
1.5

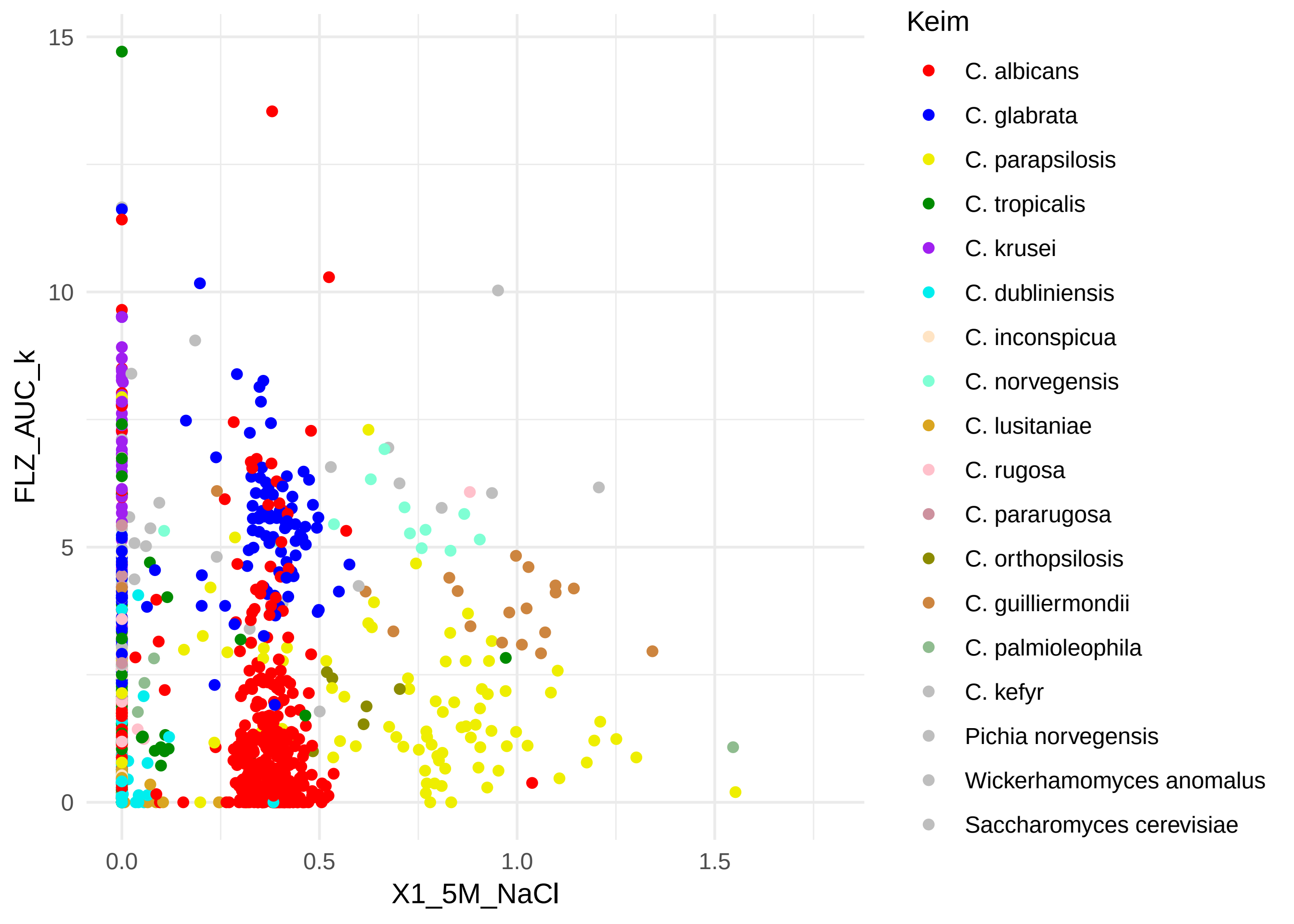

FLZ_AUC_k
15
10
5
0
0.0
0.5
1.0
1.5
X1_5M_NaCl
Keim
C. albicans
C. glabrata
C. parapsilosis
C. tropicalis
C. krusei
C. dubliniensis
C. inconspicua
C. norvegensis
C. lusitaniae
C. rugosa
C. pararugosa
C. orthopsilosis
C. guilliermondii
C. palmioleophila
C. kefyr
Pichia norvegensis
Wickerhamomyces anomalus
Saccharomyces cerevisiae

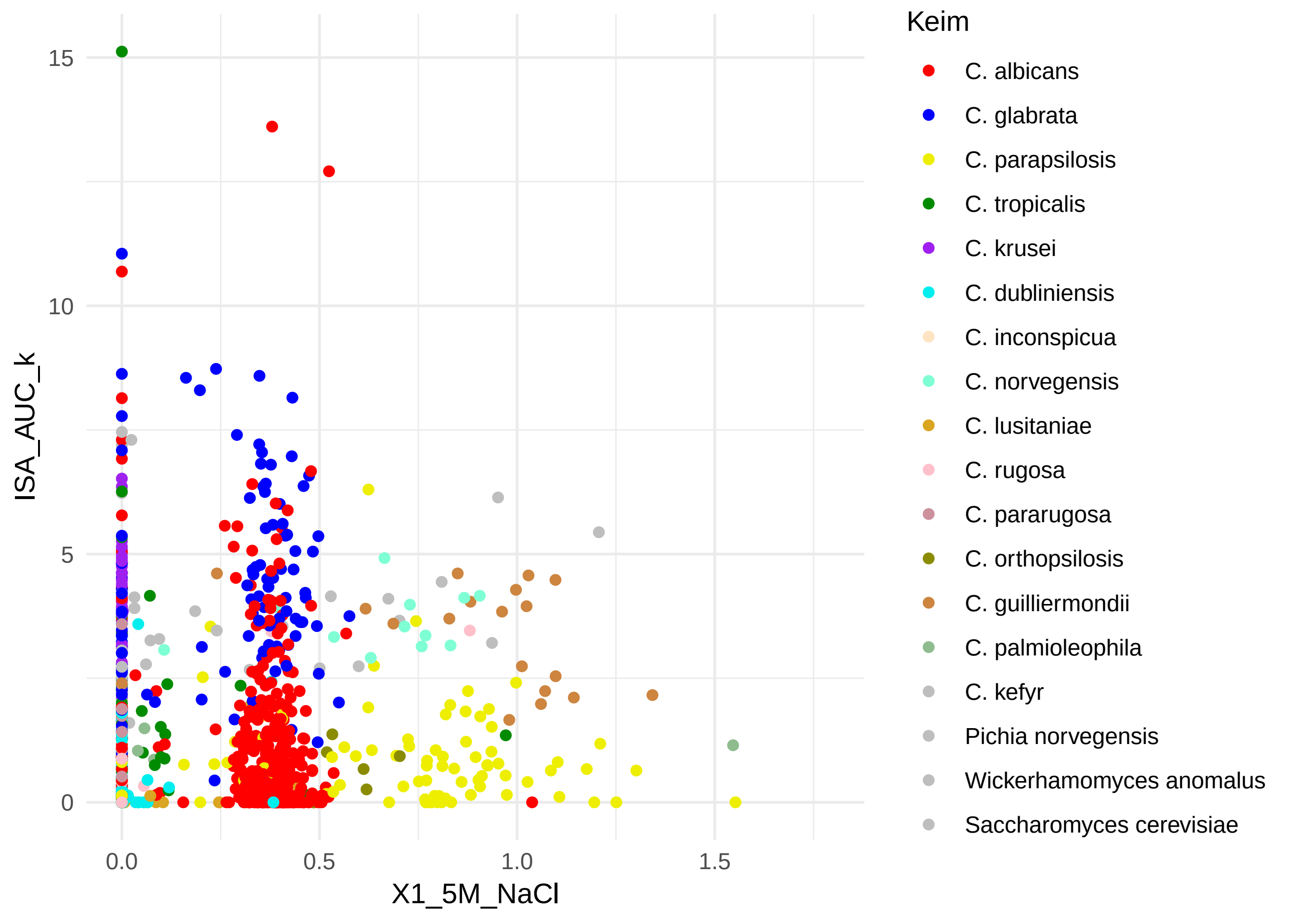

15
10
5
0
ISA_AUC_k
0.0
0.5
1.0
1.5
X1_5M_NaCl
Keim
C. albicans
C. glabrata
C. parapsilosis
C. tropicalis
C. krusei
C. dubliniensis
C. inconspicua
C. norvegensis
C. lusitaniae
C. rugosa
C. pararugosa
C. orthopsilosis
C. guilliermondii
C. palmioleophila
C. kefyr
Pichia norvegensis
Wickerhamomyces anomalus
Saccharomyces cerevisiae

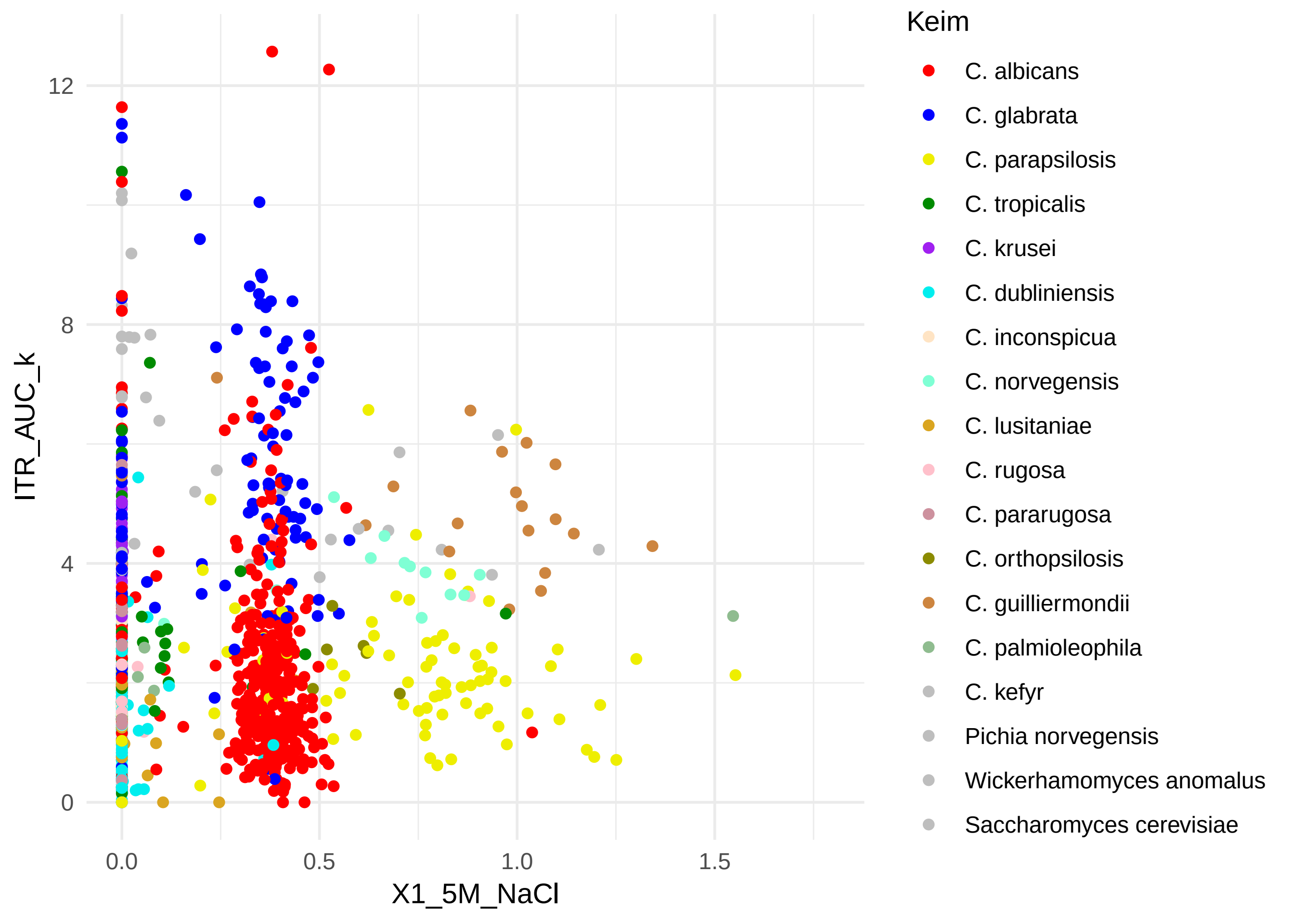

ITR_AUC_k
X1_5M_NaCl
0
4
8
12
0.0
0.5
1.0
1.5
Keim
C. albicans
C. glabrata
C. parapsilosis
C. tropicalis
C. krusei
C. dubliniensis
C. inconspicua
C. norvegensis
C. lusitaniae
C. rugosa
C. pararugosa
C. orthopsilosis
C. guilliermondii
C. palmioleophila
C. kefyr
Pichia norvegensis
Wickerhamomyces anomalus
Saccharomyces cerevisiae

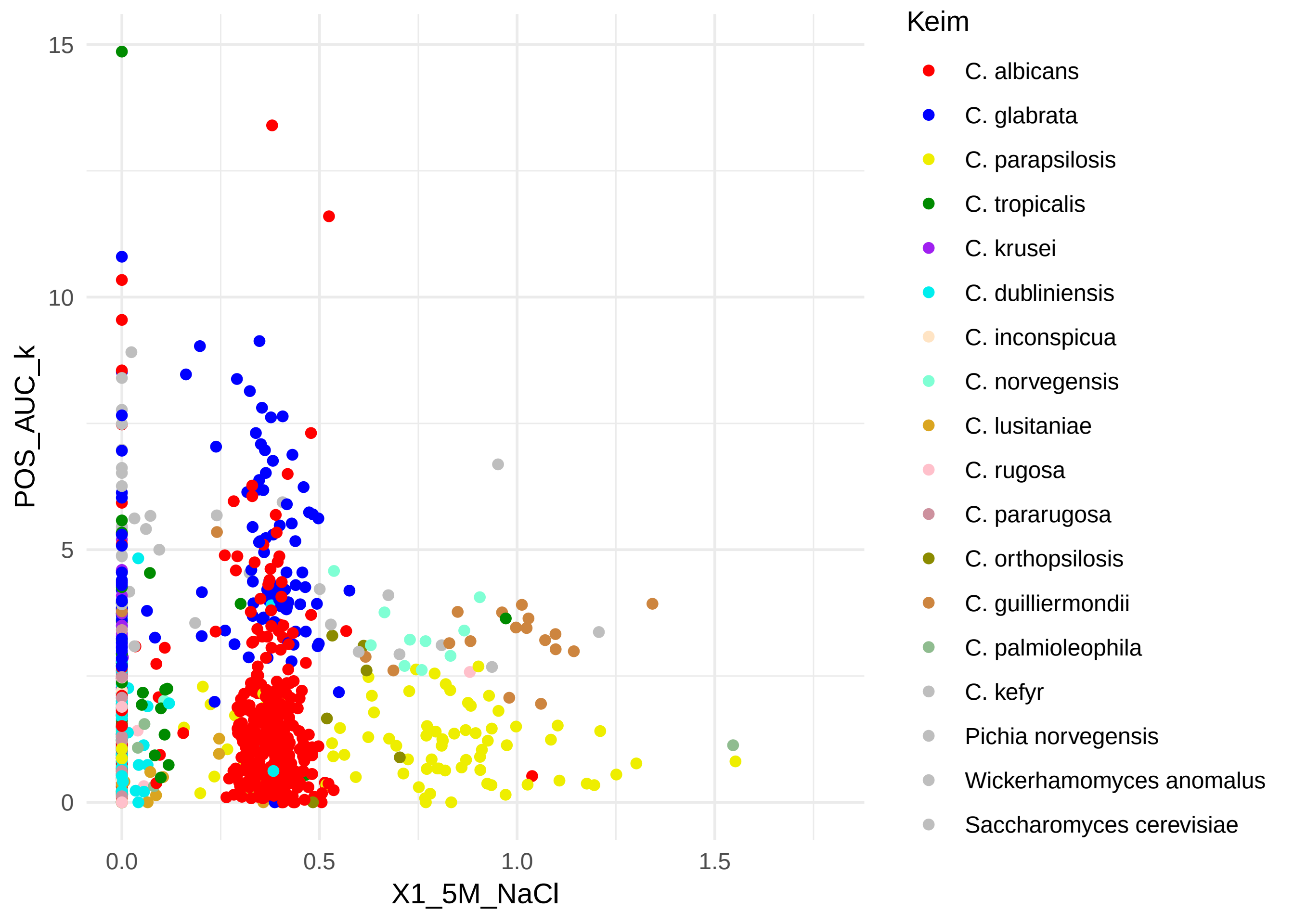
Keim
C. albicans
C. glabrata
C. parapsilosis
C. tropicalis
C. krusei
C. dubliniensis
C. inconspicua
C. norvegensis
C. lusitaniae
C. rugosa
C. pararugosa
C. orthopsilosis
C. guilliermondii
C. palmioleophila
C. kefyr
Pichia norvegensis
Wickerhamomyces anomalus
Saccharomyces cerevisiae
POS_AUC_k
X1_5M_NaCl
15
10
5
0
0.0
0.5
1.0
1.5

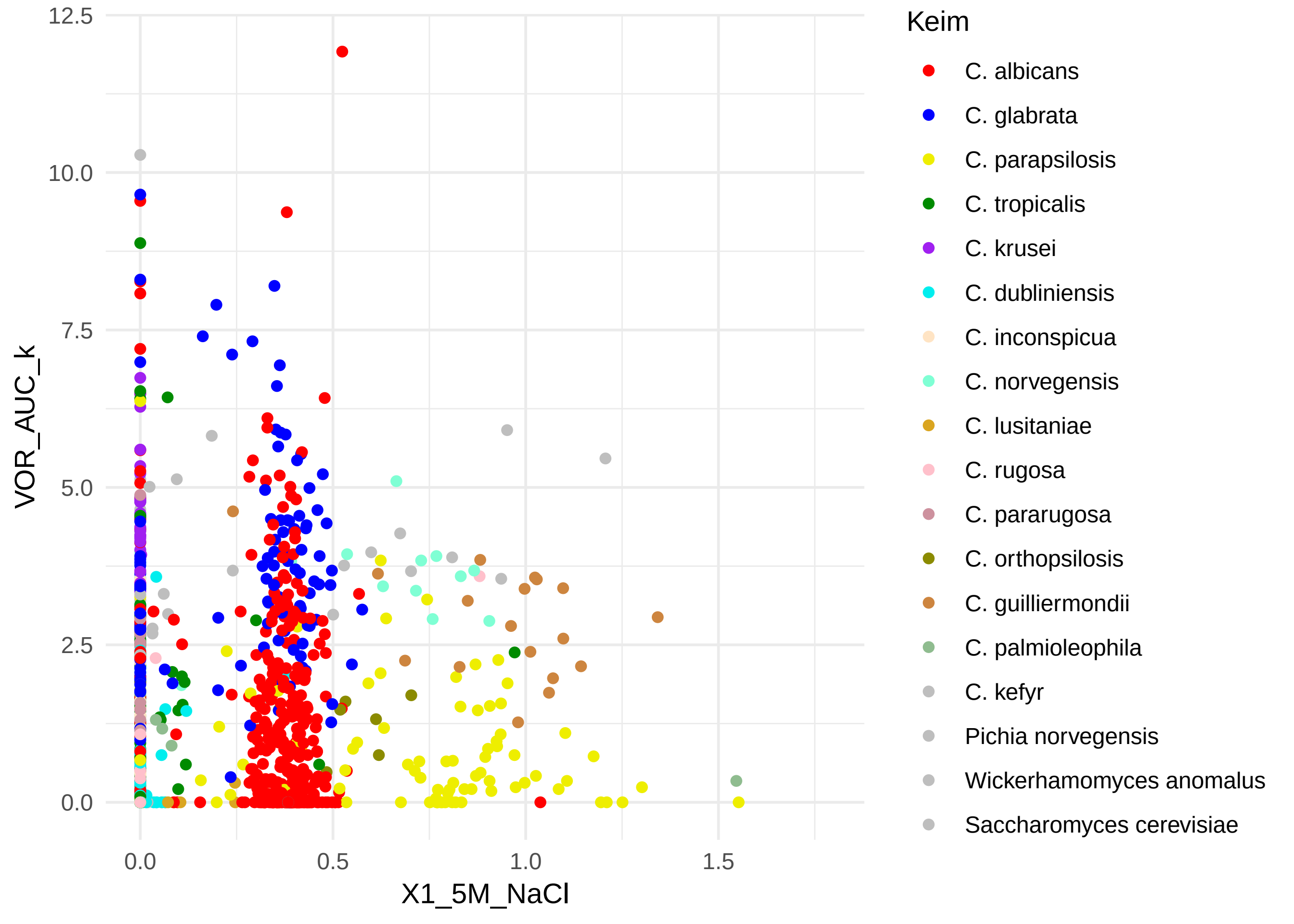

12.5
10.0
7.5
5.0
2.5
0.0
VOR_AUC_k
0.0
0.5
1.0
1.5
X1_5M_NaCl
Keim
C. albicans
C. glabrata
C. parapsilosis
C. tropicalis
C. krusei
C. dubliniensis
C. inconspicua
C. norvegensis
C. lusitaniae
C. rugosa
C. pararugosa
C. orthopsilosis
C. guilliermondii
C. palmioleophila
C. kefyr
Pichia norvegensis
Wickerhamomyces anomalus
Saccharomyces cerevisiae

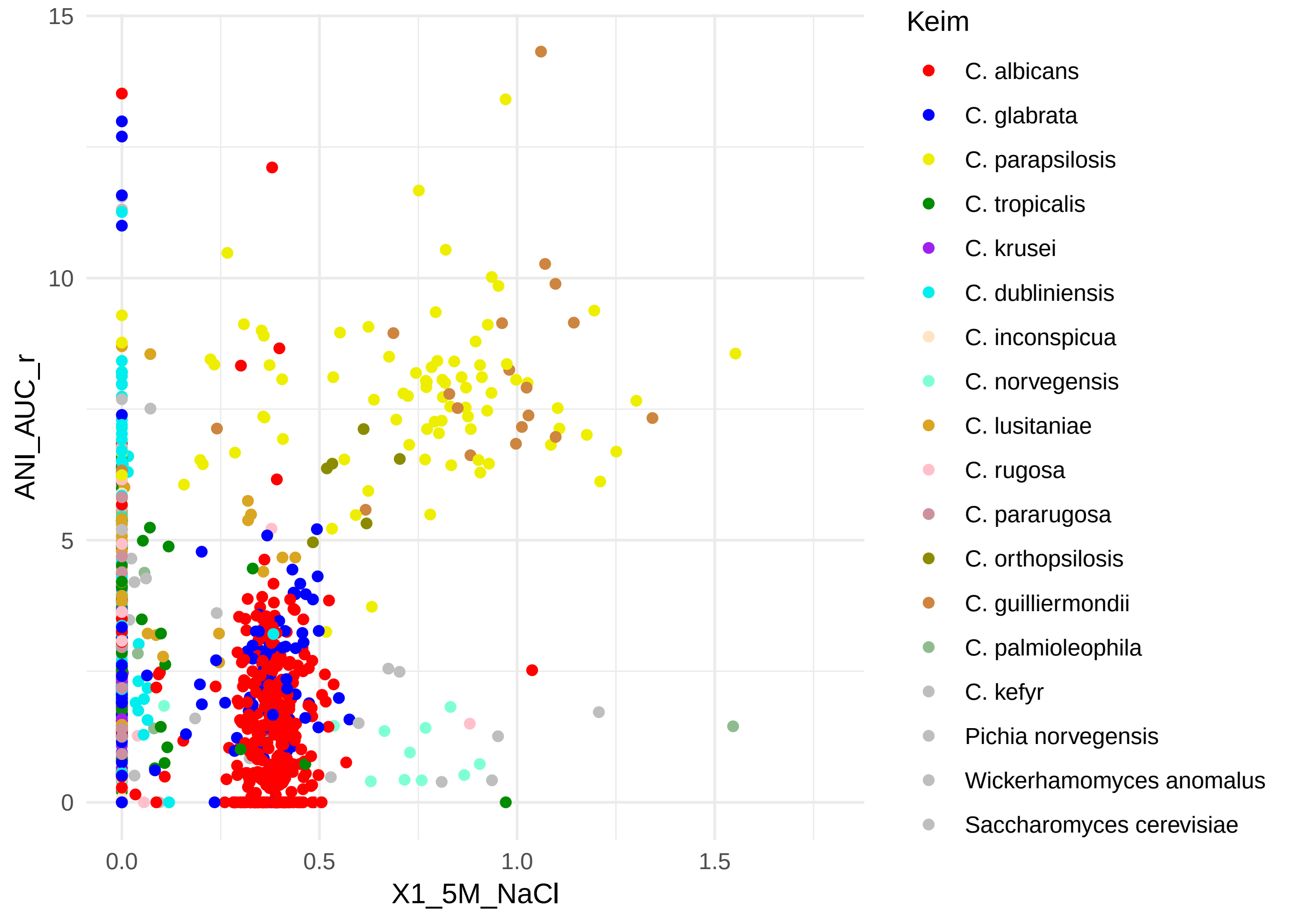

Keim
C. albicans
C. glabrata
C. parapsilosis
C. tropicalis
C. krusei
C. dubliniensis
C. inconspicua
C. norvegensis
C. lusitaniae
C. rugosa
C. pararugosa
C. orthopsilosis
C. guilliermondii
C. palmioleophila
C. kefyr
Pichia norvegensis
Wickerhamomyces anomalus
Saccharomyces cerevisiae
ANI_AUC_r
X1_5M_NaCl
0
5
10
15
0.0
0.5
1.0
1.5

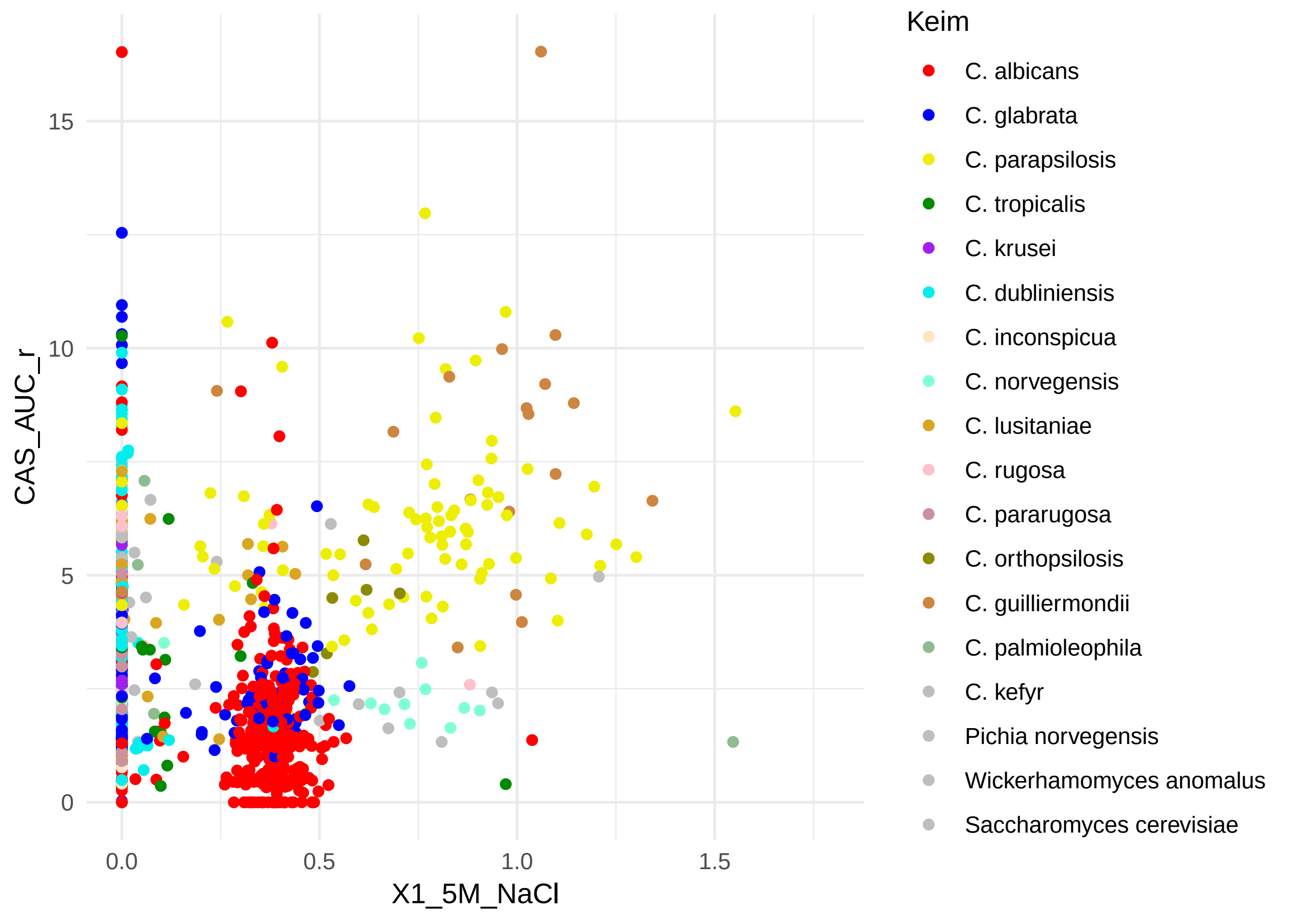
CAS_AUC_r
X1_5M_NaCl
0
5
10
15
0.0
0.5
1.0
1.5
Keim
C. albicans
C. glabrata
C. parapsilosis
C. tropicalis
C. krusei
C. dubliniensis
C. inconspicua
C. norvegensis
C. lusitaniae
C. rugosa
C. pararugosa
C. orthopsilosis
C. guilliermondii
C. palmioleophila
C. kefyr
Pichia norvegensis
Wickerhamomyces anomalus
Saccharomyces cerevisiae

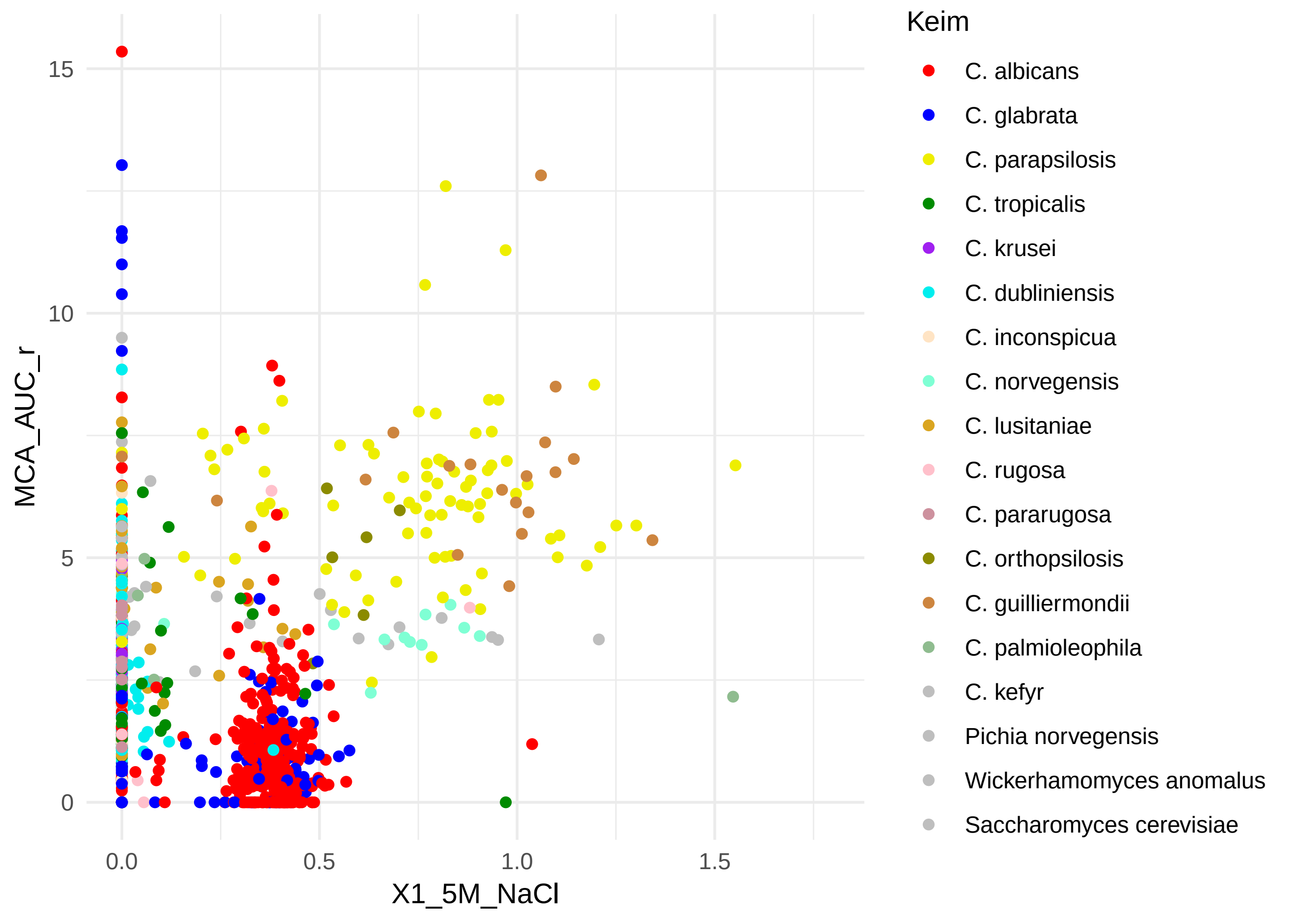

Keim
C. albicans
C. glabrata
C. parapsilosis
C. tropicalis
C. krusei
C. dubliniensis
C. inconspicua
C. norvegensis
C. lusitaniae
C. rugosa
C. pararugosa
C. orthopsilosis
C. guilliermondii
C. palmioleophila
C. kefyr
Pichia norvegensis
Wickerhamomyces anomalus
Saccharomyces cerevisiae
MCA_AUC_r
X1_5M_NaCl
0
5
10
15
0.0
0.5
1.0
1.5

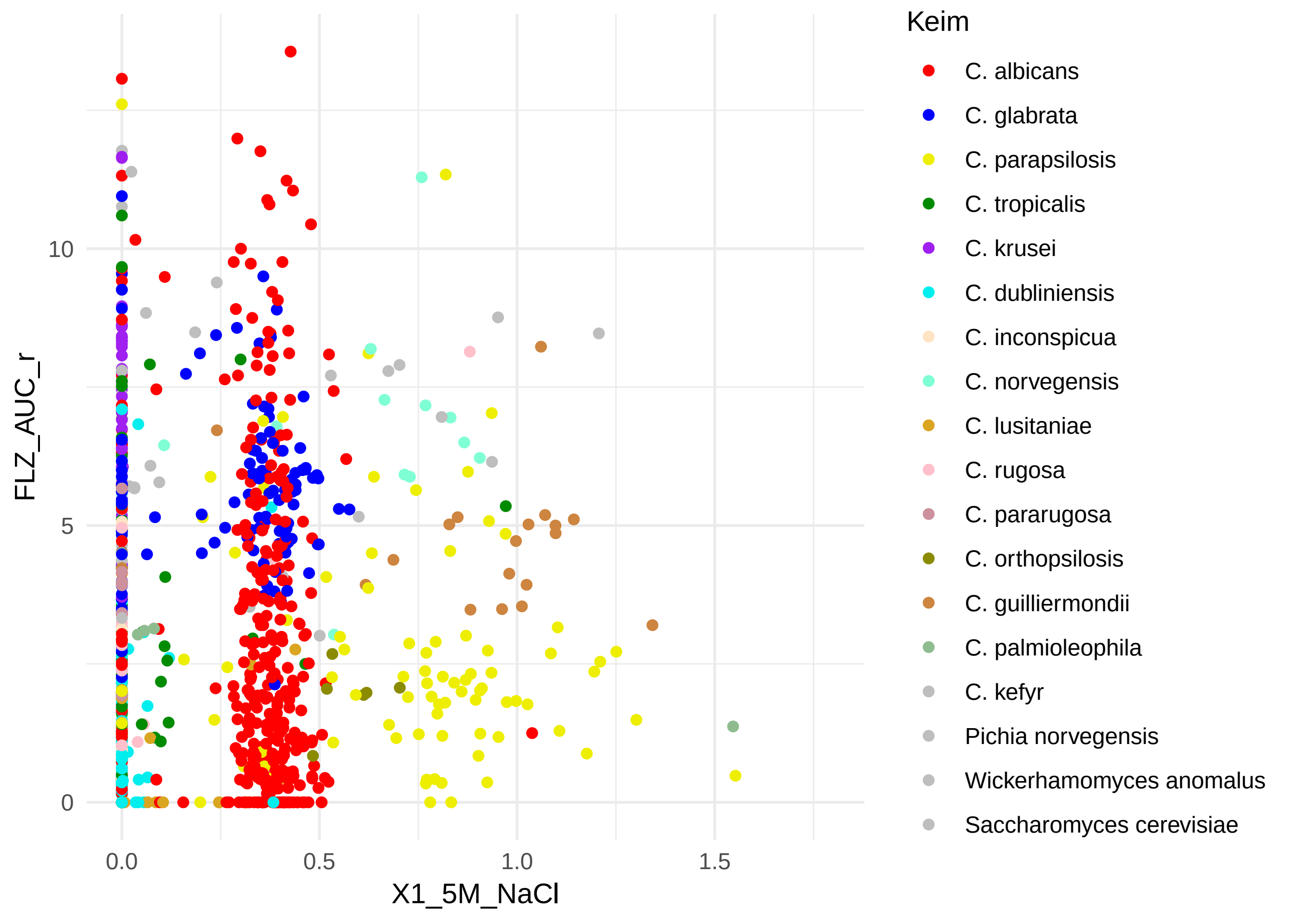
Keim
C. albicans
C. glabrata
C. parapsilosis
C. tropicalis
C. krusei
C. dubliniensis
C. inconspicua
C. norvegensis
C. lusitaniae
C. rugosa
C. pararugosa
C. orthopsilosis
C. guilliermondii
C. palmioleophila
C. kefyr
Pichia norvegensis
Wickerhamomyces anomalus
Saccharomyces cerevisiae
FLZ_AUC_r
10
5
0
0.0
0.5
1.0
1.5
X1_5M_NaCl

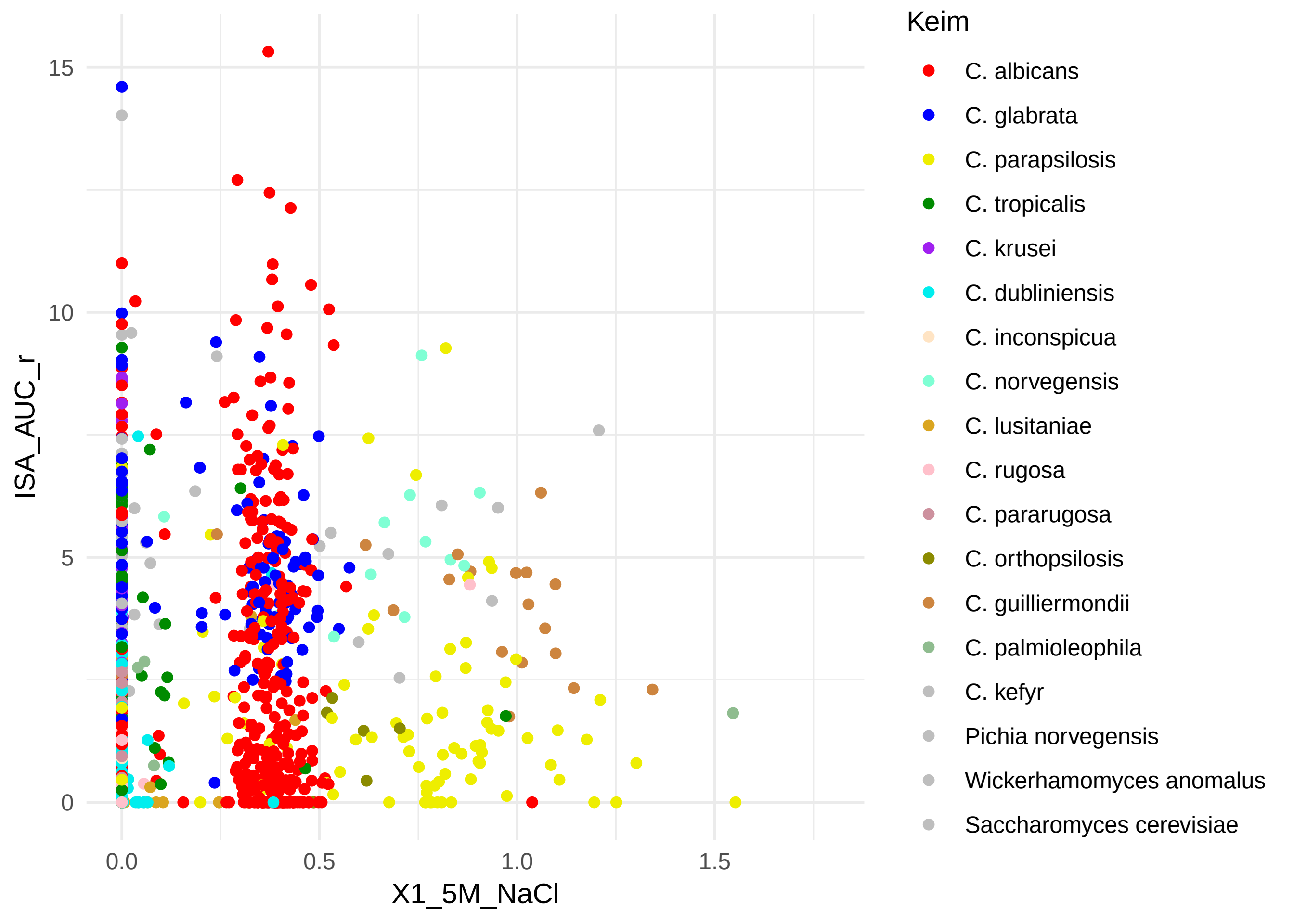
Keim
C. albicans
C. glabrata
C. parapsilosis
C. tropicalis
C. krusei
C. dubliniensis
C. inconspicua
C. norvegensis
C. lusitaniae
C. rugosa
C. pararugosa
C. orthopsilosis
C. guilliermondii
C. palmioleophila
C. kefyr
Pichia norvegensis
Wickerhamomyces anomalus
Saccharomyces cerevisiae
ISA_AUC_r
X1_5M_NaCl
0
5
10
15
0.0
0.5
1.0
1.5

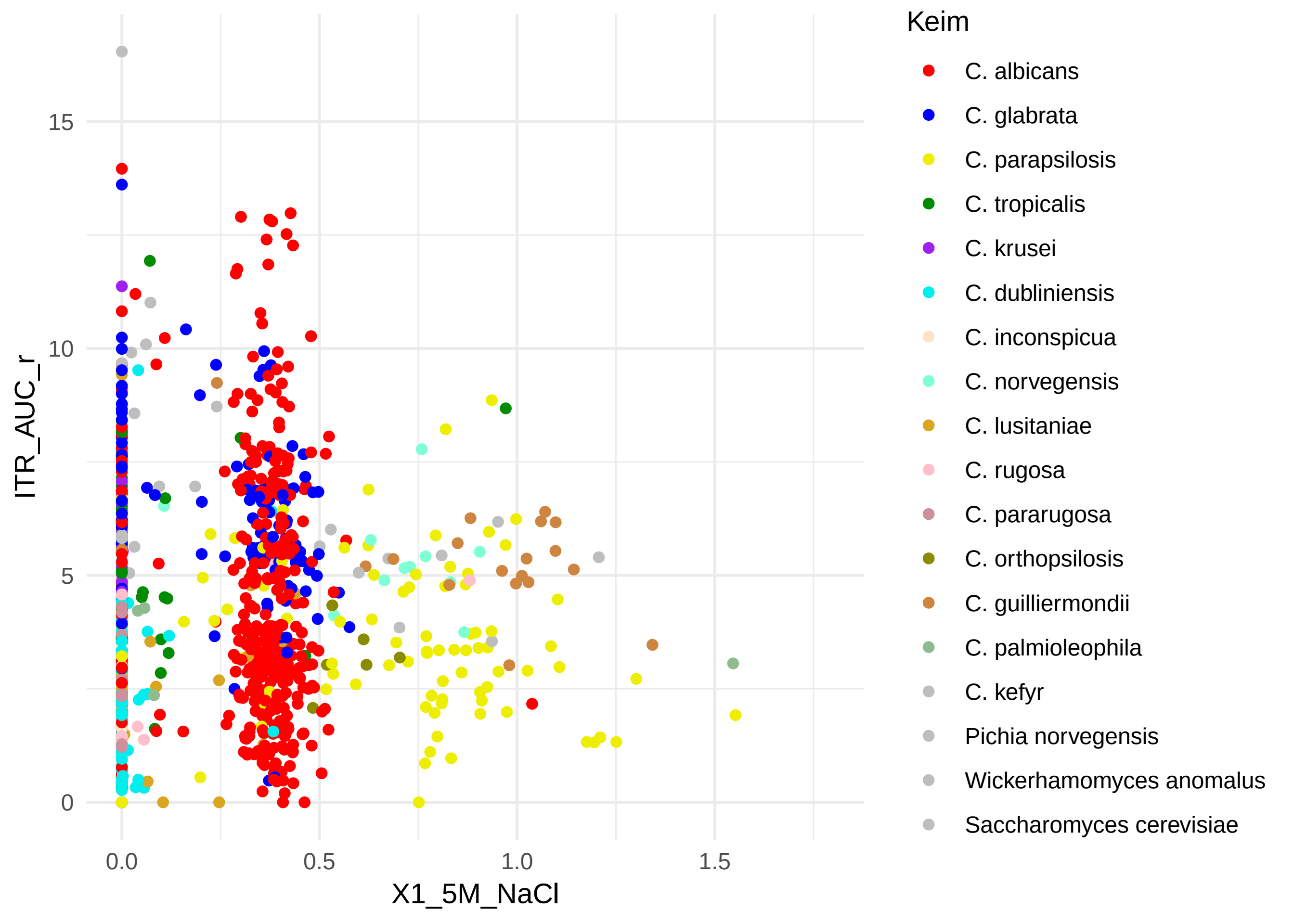
ITR_AUC_r
X1_5M_NaCl
0
5
10
15
0.0
0.5
1.0
1.5
Keim
C. albicans
C. glabrata
C. parapsilosis
C. tropicalis
C. krusei
C. dubliniensis
C. inconspicua
C. norvegensis
C. lusitaniae
C. rugosa
C. pararugosa
C. orthopsilosis
C. guilliermondii
C. palmioleophila
C. kefyr
Pichia norvegensis
Wickerhamomyces anomalus
Saccharomyces cerevisiae

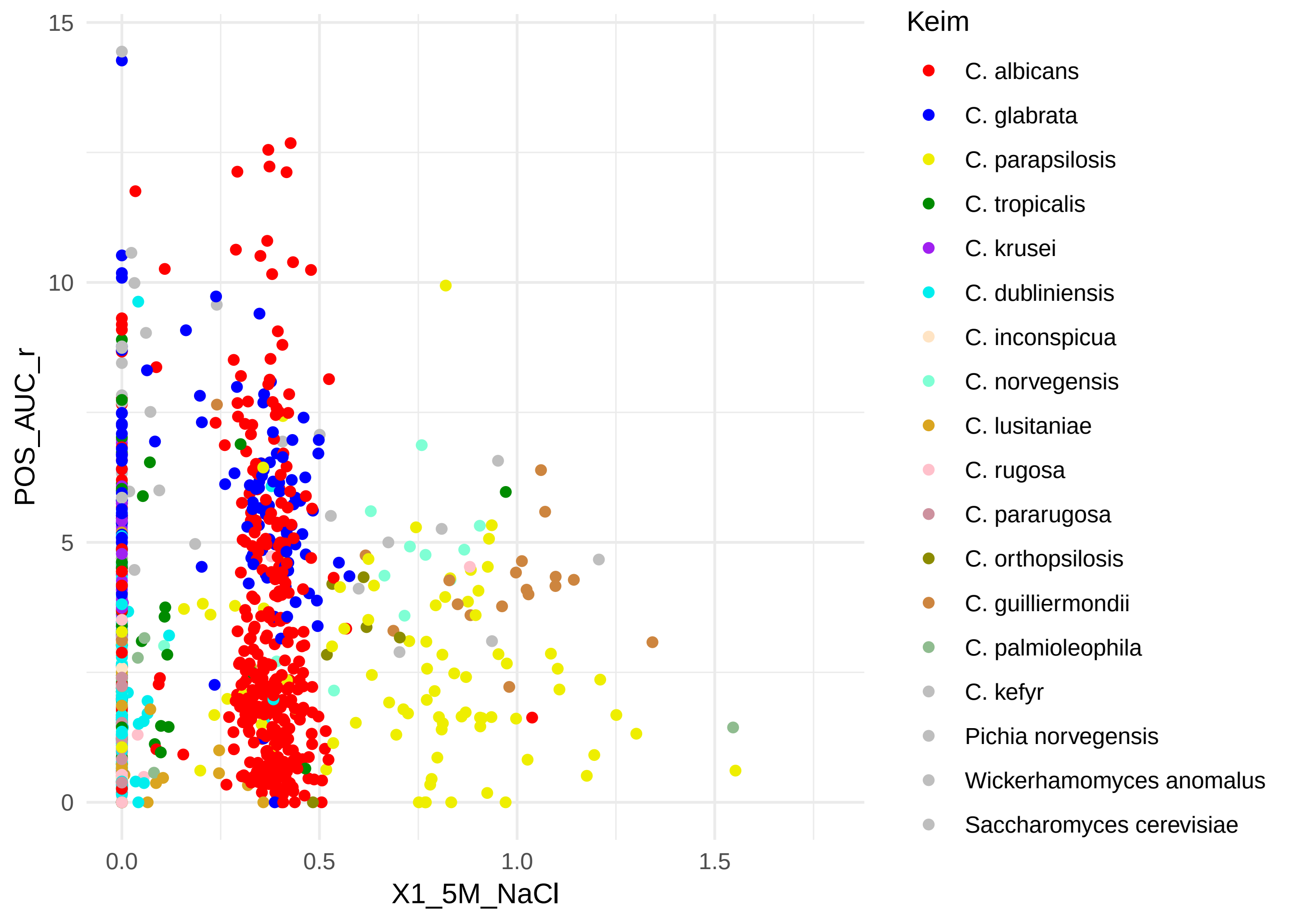
Keim
C. albicans
C. glabrata
C. parapsilosis
C. tropicalis
C. krusei
C. dubliniensis
C. inconspicua
C. norvegensis
C. lusitaniae
C. rugosa
C. pararugosa
C. orthopsilosis
C. guilliermondii
C. palmioleophila
C. kefyr
Pichia norvegensis
Wickerhamomyces anomalus
Saccharomyces cerevisiae
POS_AUC_r
X1_5M_NaCl
0
5
10
15
0.0
0.5
1.0
1.5

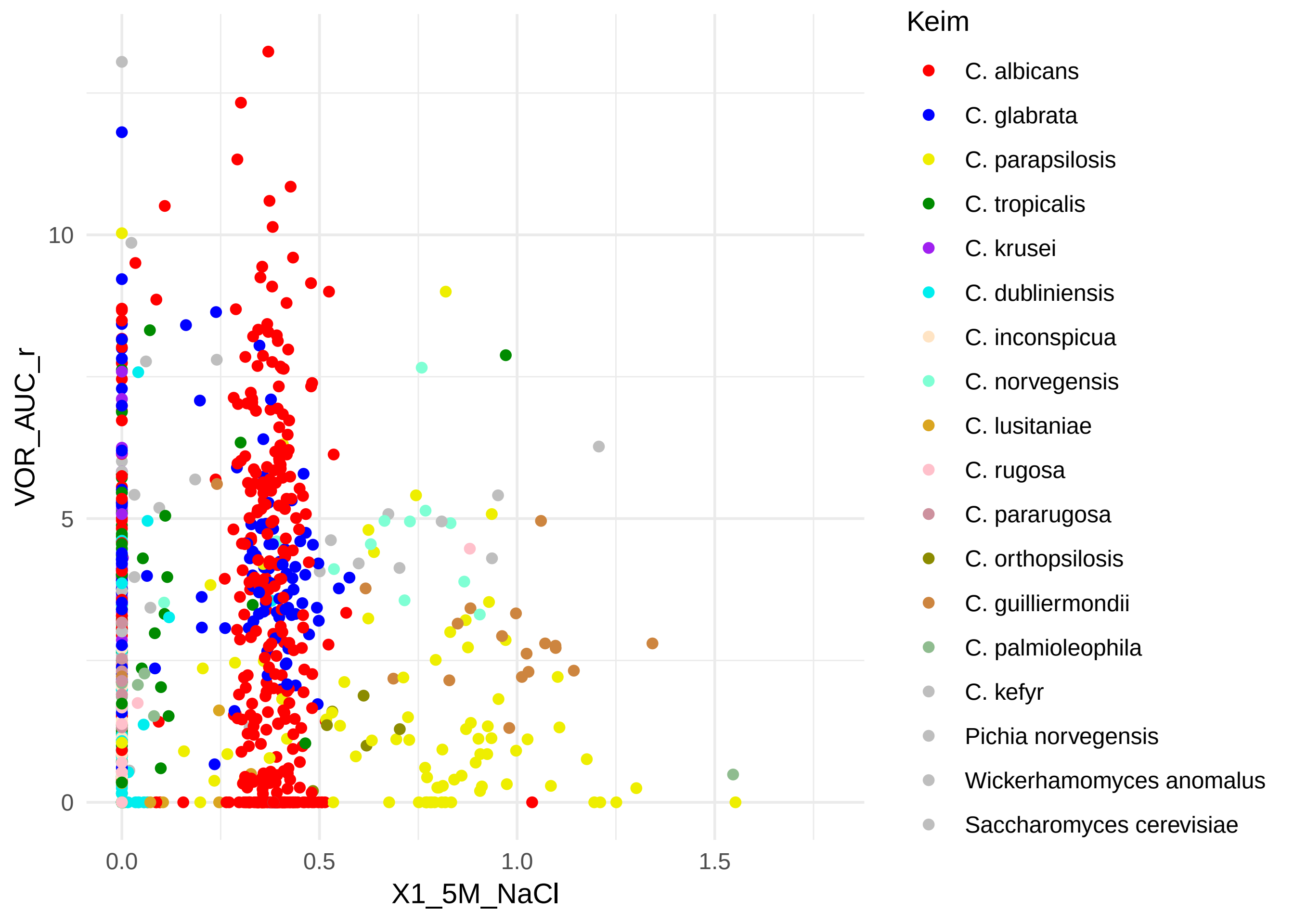
Keim
C. albicans
C. glabrata
C. parapsilosis
C. tropicalis
C. krusei
C. dubliniensis
C. inconspicua
C. norvegensis
C. lusitaniae
C. rugosa
C. pararugosa
C. orthopsilosis
C. guilliermondii
C. palmioleophila
C. kefyr
Pichia norvegensis
Wickerhamomyces anomalus
Saccharomyces cerevisiae
VOR_AUC_r
10
5
0
0.0
0.5
1.0
1.5
X1_5M_NaCl

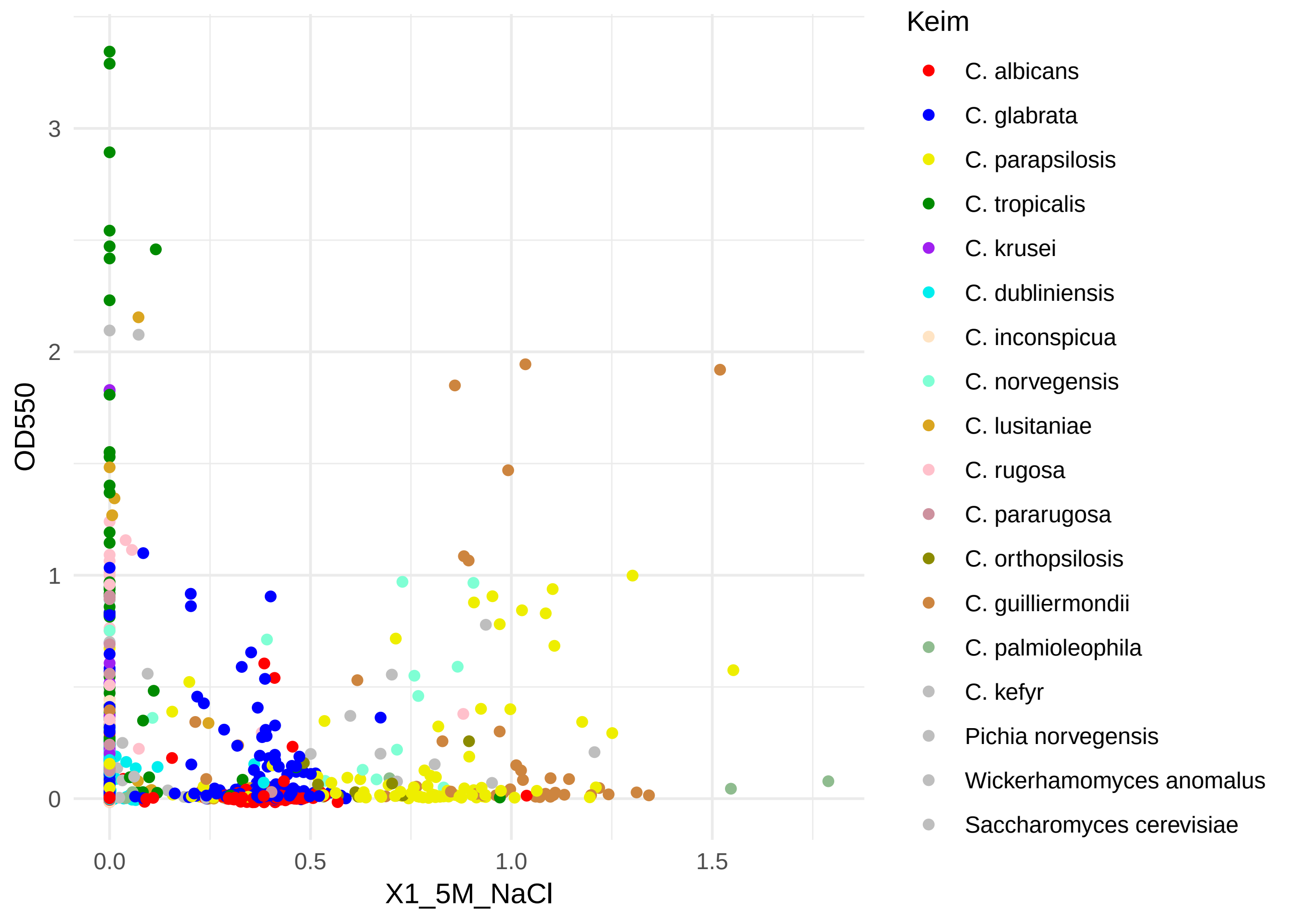
Keim
C. albicans
C. glabrata
C. parapsilosis
C. tropicalis
C. krusei
C. dubliniensis
C. inconspicua
C. norvegensis
C. lusitaniae
C. rugosa
C. pararugosa
C. orthopsilosis
C. guilliermondii
C. palmioleophila
C. kefyr
Pichia norvegensis
Wickerhamomyces anomalus
Saccharomyces cerevisiae
OD550
X1_5M_NaCl
0
1
2
3
0.0
0.5
1.0
1.5

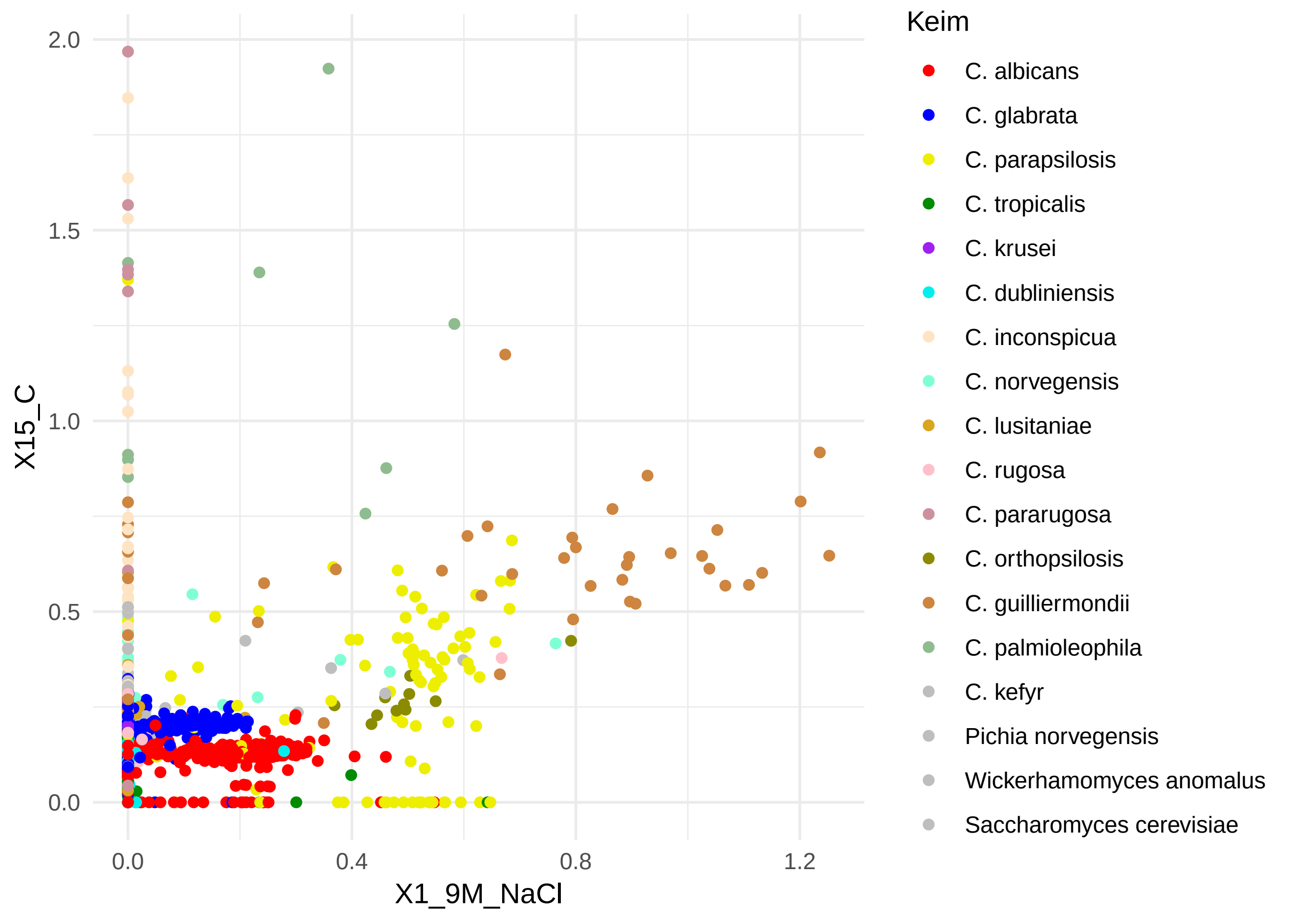

Keim
C. albicans
C. glabrata
C. parapsilosis
C. tropicalis
C. krusei
C. dubliniensis
C. inconspicua
C. norvegensis
C. lusitaniae
C. rugosa
C. pararugosa
C. orthopsilosis
C. guilliermondii
C. palmioleophila
C. kefyr
Pichia norvegensis
Wickerhamomyces anomalus
Saccharomyces cerevisiae
X15_C
X1_9M_NaCl
2.0
1.5
1.0
0.5
0.0
0.0
0.4
0.8
1.2

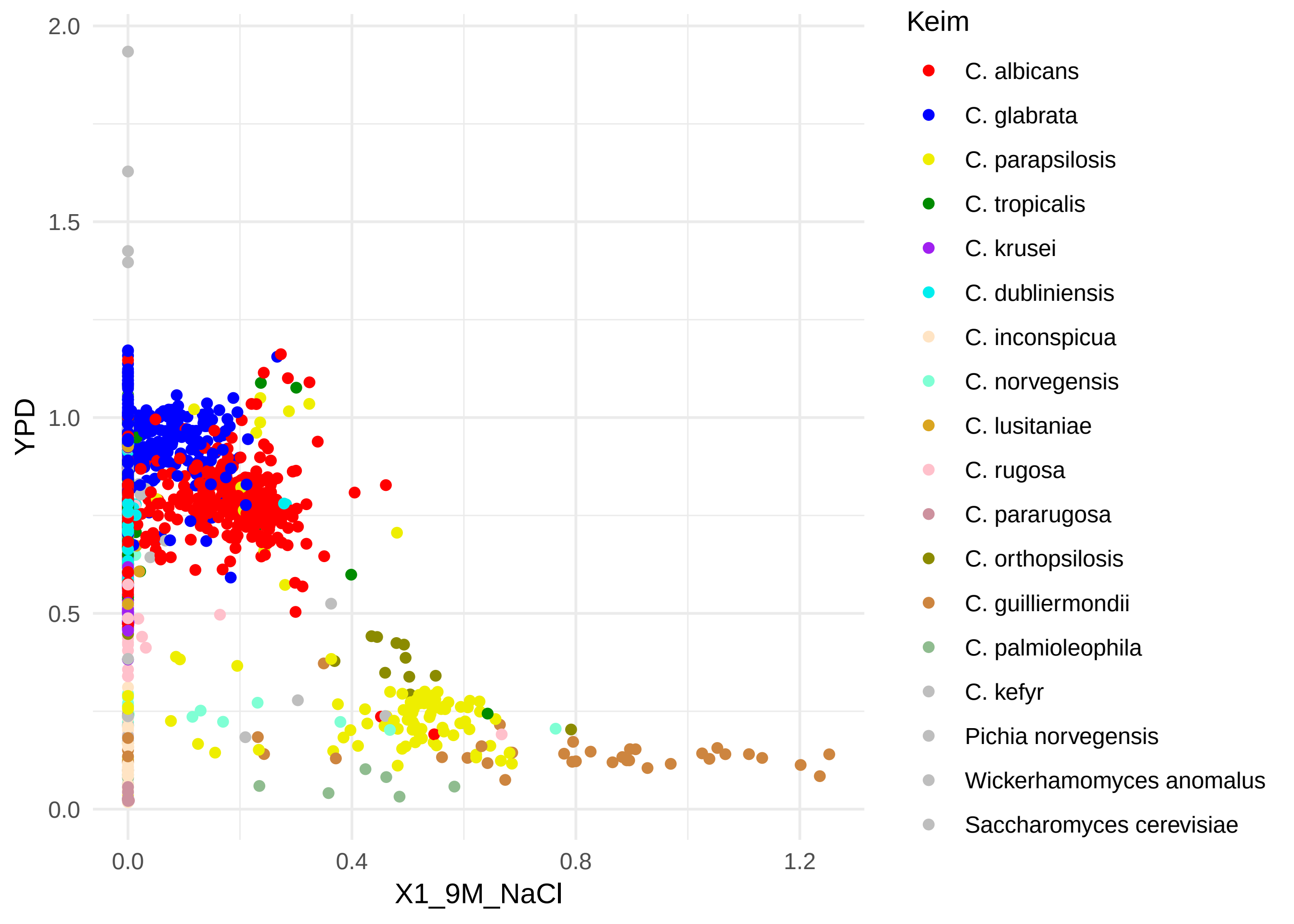

Keim
C. albicans
C. glabrata
C. parapsilosis
C. tropicalis
C. krusei
C. dubliniensis
C. inconspicua
C. norvegensis
C. lusitaniae
C. rugosa
C. pararugosa
C. orthopsilosis
C. guilliermondii
C. palmioleophila
C. kefyr
Pichia norvegensis
Wickerhamomyces anomalus
Saccharomyces cerevisiae
YPD
X1_9M_NaCl
0.0
0.5
1.0
1.5
2.0
0.0
0.4
0.8
1.2

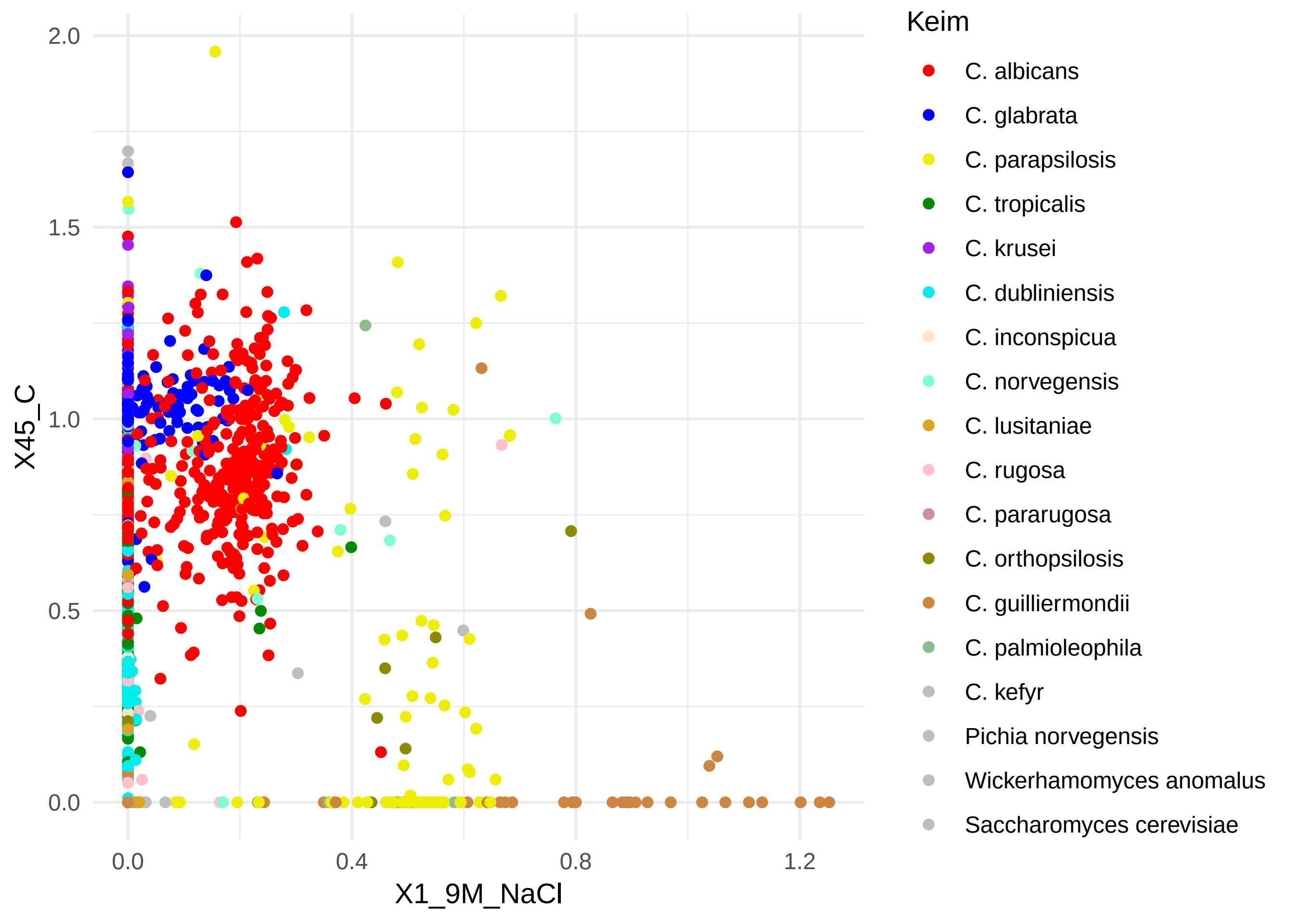

Keim
C. albicans
C. glabrata
C. parapsilosis
C. tropicalis
C. krusei
C. dubliniensis
C. inconspicua
C. norvegensis
C. lusitaniae
C. rugosa
C. pararugosa
C. orthopsilosis
C. guilliermondii
C. palmioleophila
C. kefyr
Pichia norvegensis
Wickerhamomyces anomalus
Saccharomyces cerevisiae
X45_C
2.0
1.5
1.0
0.5
0.0
0.0
0.4
0.8
1.2
X1_9M_NaCl

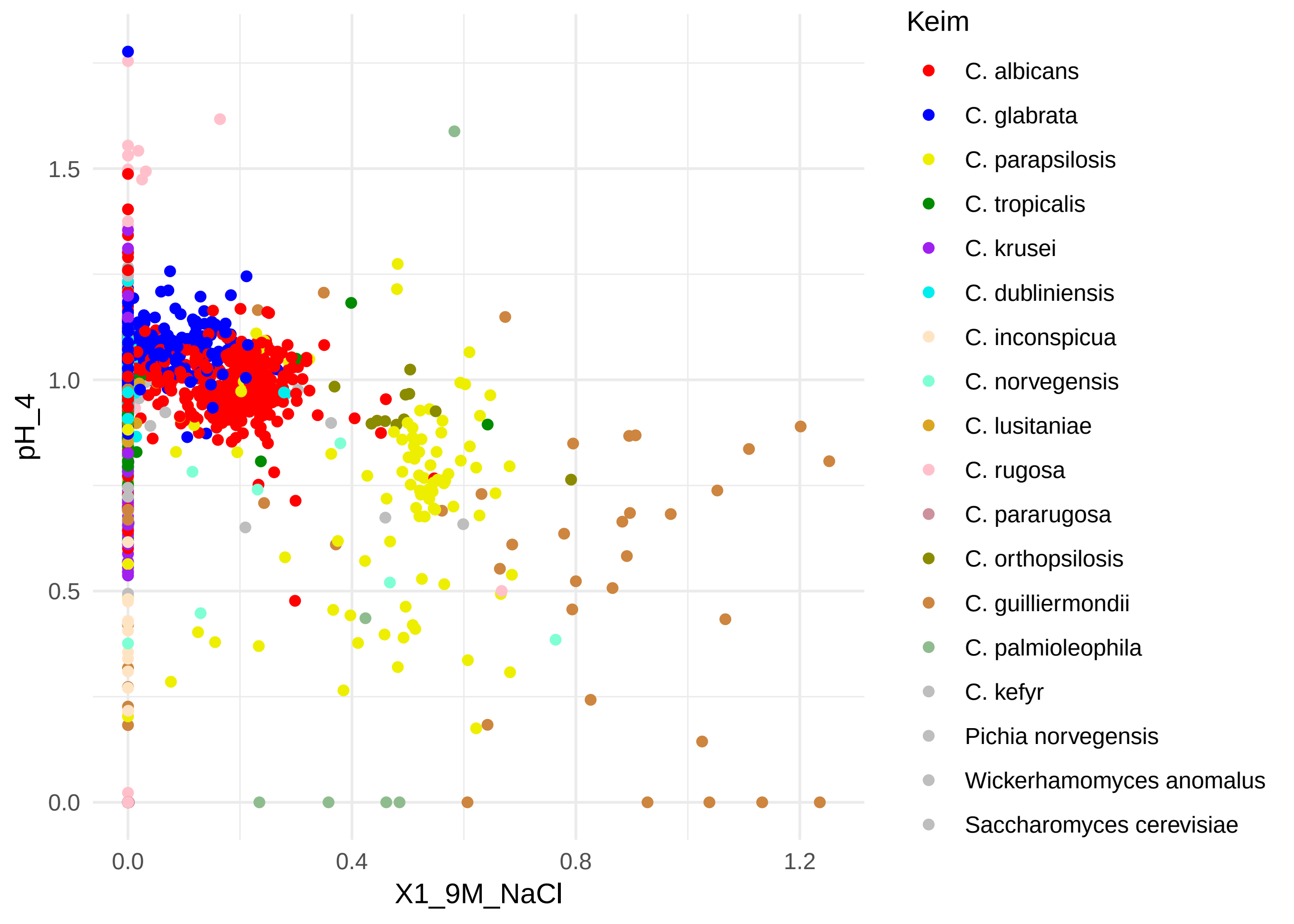

pH_4
1.5
1.0
0.5
0.0
0.0
0.4
0.8
1.2
X1_9M_NaCl
Keim
C. albicans
C. glabrata
C. parapsilosis
C. tropicalis
C. krusei
C. dubliniensis
C. inconspicua
C. norvegensis
C. lusitaniae
C. rugosa
C. pararugosa
C. orthopsilosis
C. guilliermondii
C. palmioleophila
C. kefyr
Pichia norvegensis
Wickerhamomyces anomalus
Saccharomyces cerevisiae

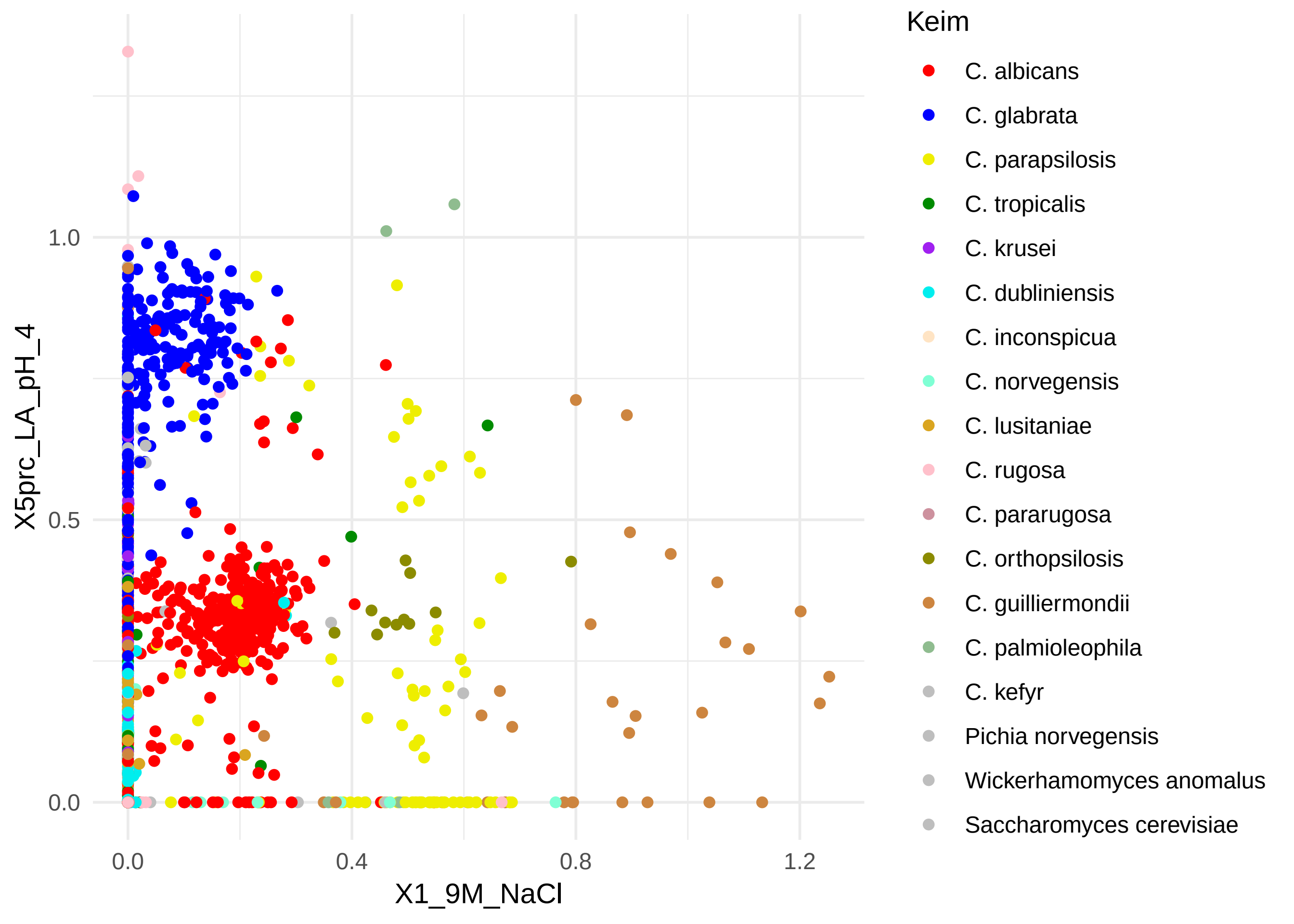

Keim
C. albicans
C. glabrata
C. parapsilosis
C. tropicalis
C. krusei
C. dubliniensis
C. inconspicua
C. norvegensis
C. lusitaniae
C. rugosa
C. pararugosa
C. orthopsilosis
C. guilliermondii
C. palmioleophila
C. kefyr
Pichia norvegensis
Wickerhamomyces anomalus
Saccharomyces cerevisiae
X5prc_LA_pH_4
1.0
0.5
0.0
0.0
0.4
0.8
1.2
X1_9M_NaCl

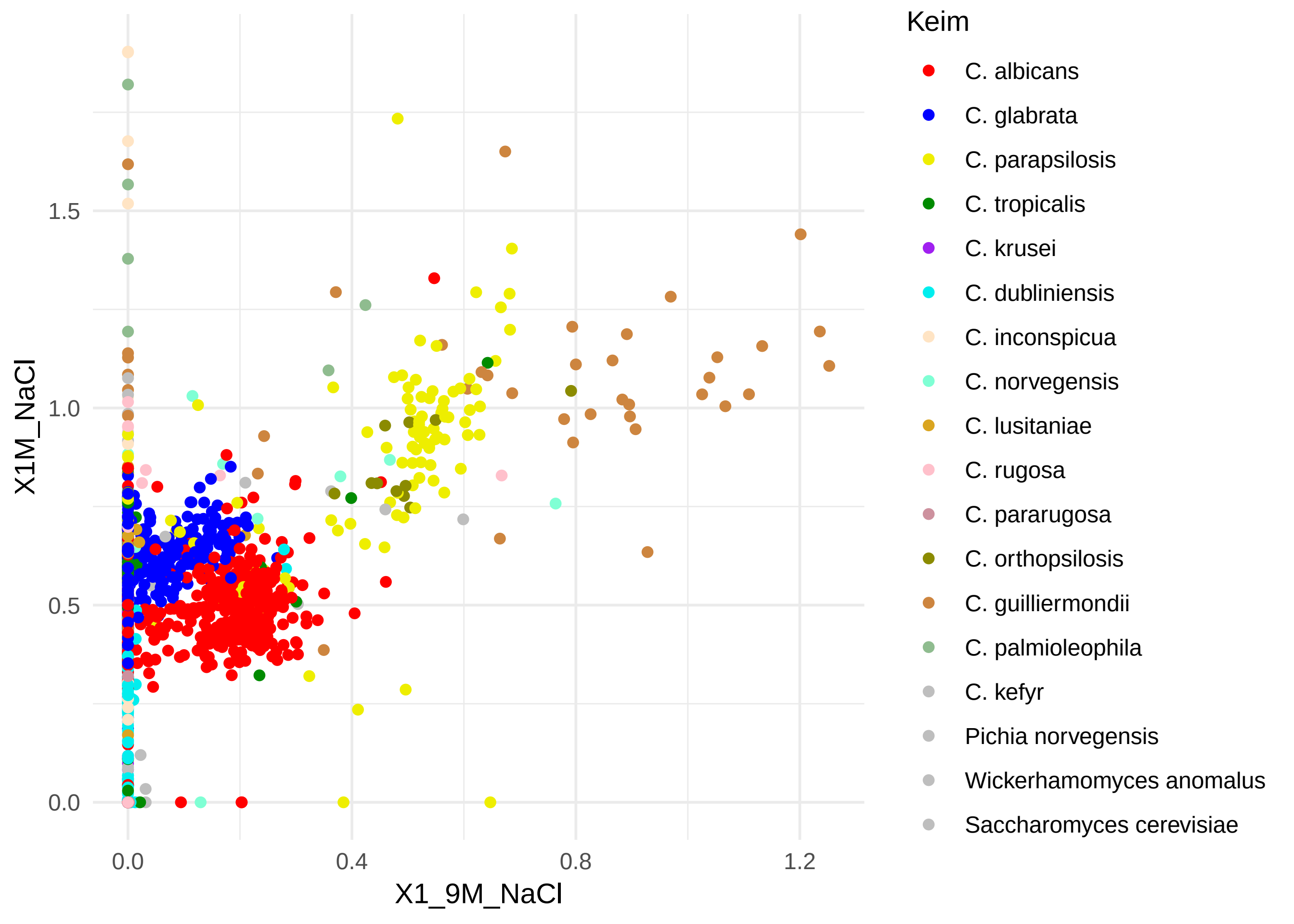
Keim
C. albicans
C. glabrata
C. parapsilosis
C. tropicalis
C. krusei
C. dubliniensis
C. inconspicua
C. norvegensis
C. lusitaniae
C. rugosa
C. pararugosa
C. orthopsilosis
C. guilliermondii
C. palmioleophila
C. kefyr
Pichia norvegensis
Wickerhamomyces anomalus
Saccharomyces cerevisiae
X1M_NaCl
1.5
1.0
0.5
0.0
0.0
0.4
0.8
1.2
X1_9M_NaCl

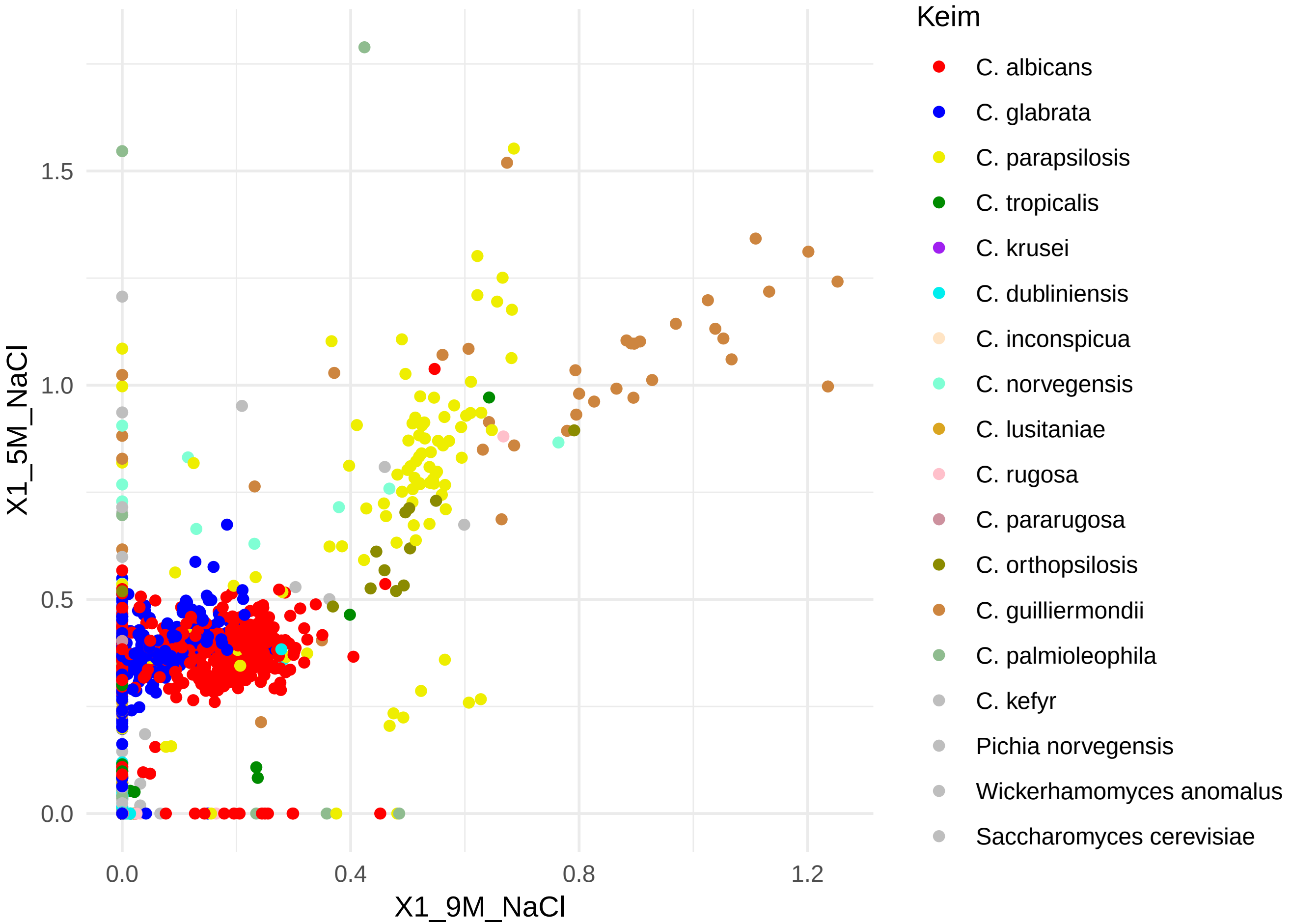
Keim
C. albicans
C. glabrata
C. parapsilosis
C. tropicalis
C. krusei
C. dubliniensis
C. inconspicua
C. norvegensis
C. lusitaniae
C. rugosa
C. pararugosa
C. orthopsilosis
C. guilliermondii
C. palmioleophila
C. kefyr
Pichia norvegensis
Wickerhamomyces anomalus
Saccharomyces cerevisiae
X1_5M_NaCl
X1_9M_NaCl
0.0
0.5
1.0
1.5
0.0
0.4
0.8
1.2

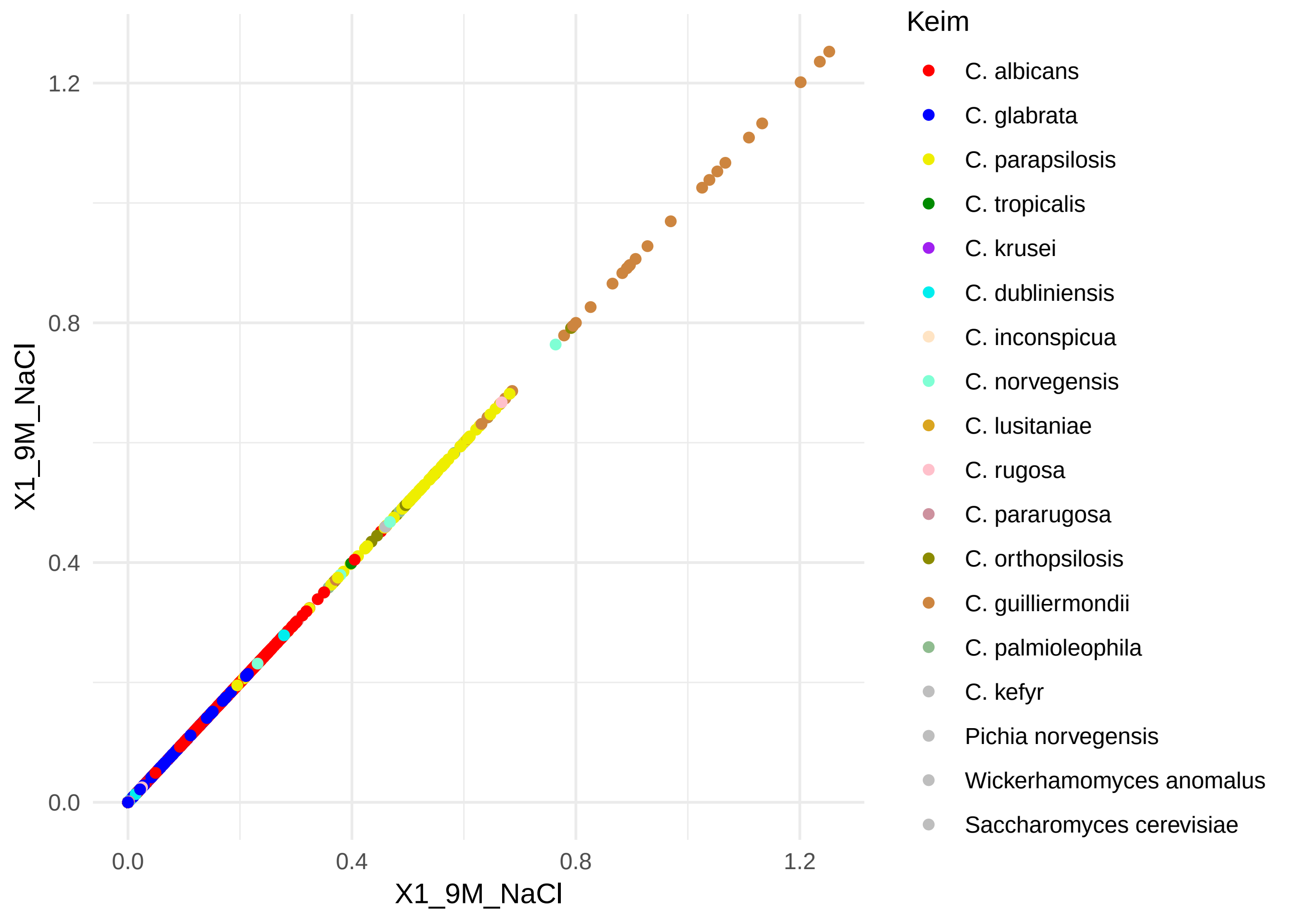

Keim
C. albicans
C. glabrata
C. parapsilosis
C. tropicalis
C. krusei
C. dubliniensis
C. inconspicua
C. norvegensis
C. lusitaniae
C. rugosa
C. pararugosa
C. orthopsilosis
C. guilliermondii
C. palmioleophila
C. kefyr
Pichia norvegensis
Wickerhamomyces anomalus
Saccharomyces cerevisiae
X1_9M_NaCl
X1_9M_NaCl
0.0
0.4
0.8
1.2
0.0
0.4
0.8
1.2

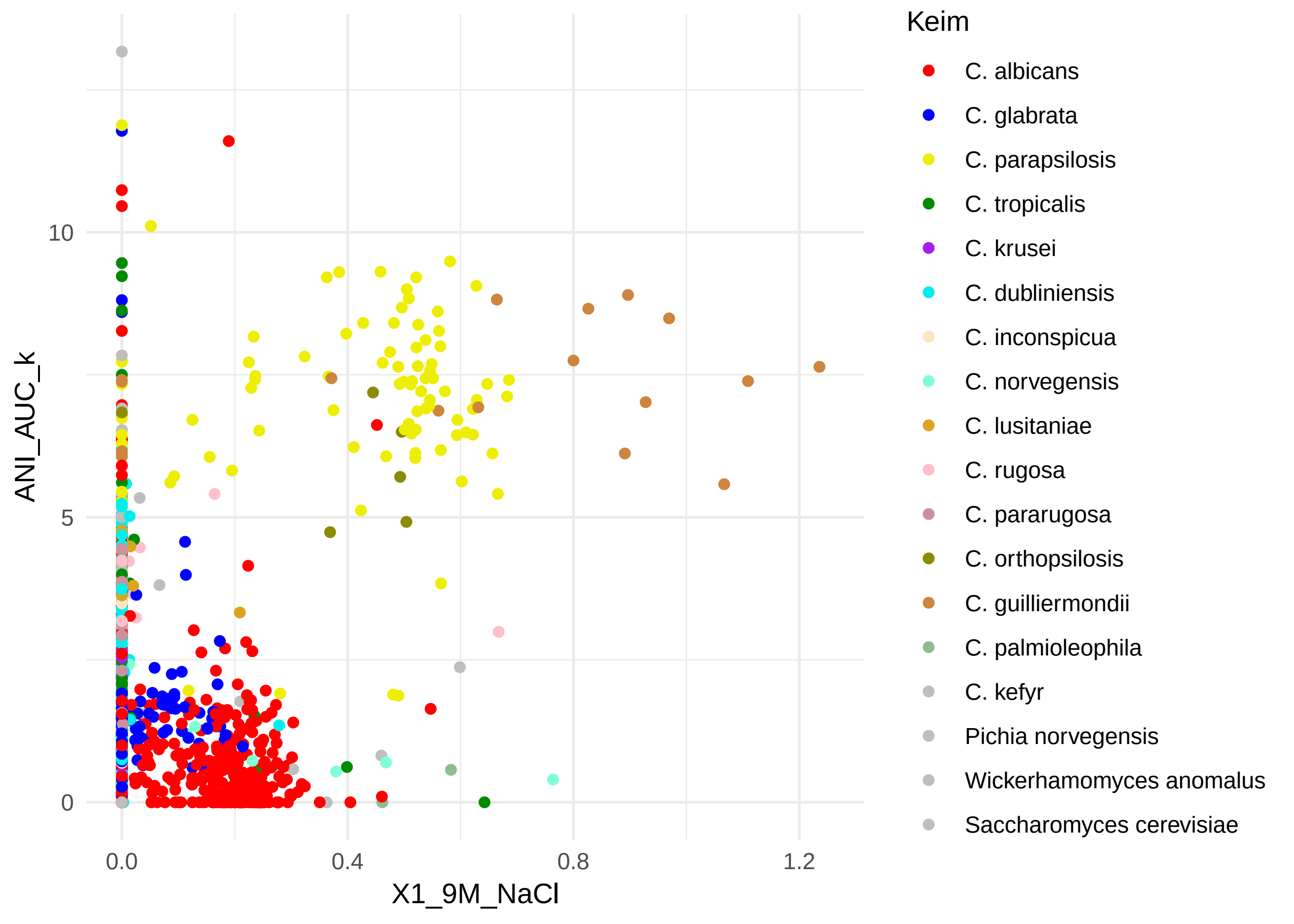

Keim
C. albicans
C. glabrata
C. parapsilosis
C. tropicalis
C. krusei
C. dubliniensis
C. inconspicua
C. norvegensis
C. lusitaniae
C. rugosa
C. pararugosa
C. orthopsilosis
C. guilliermondii
C. palmioleophila
C. kefyr
Pichia norvegensis
Wickerhamomyces anomalus
Saccharomyces cerevisiae
ANI_AUC_k
10
5
0
0.0
0.4
0.8
1.2
X1_9M_NaCl

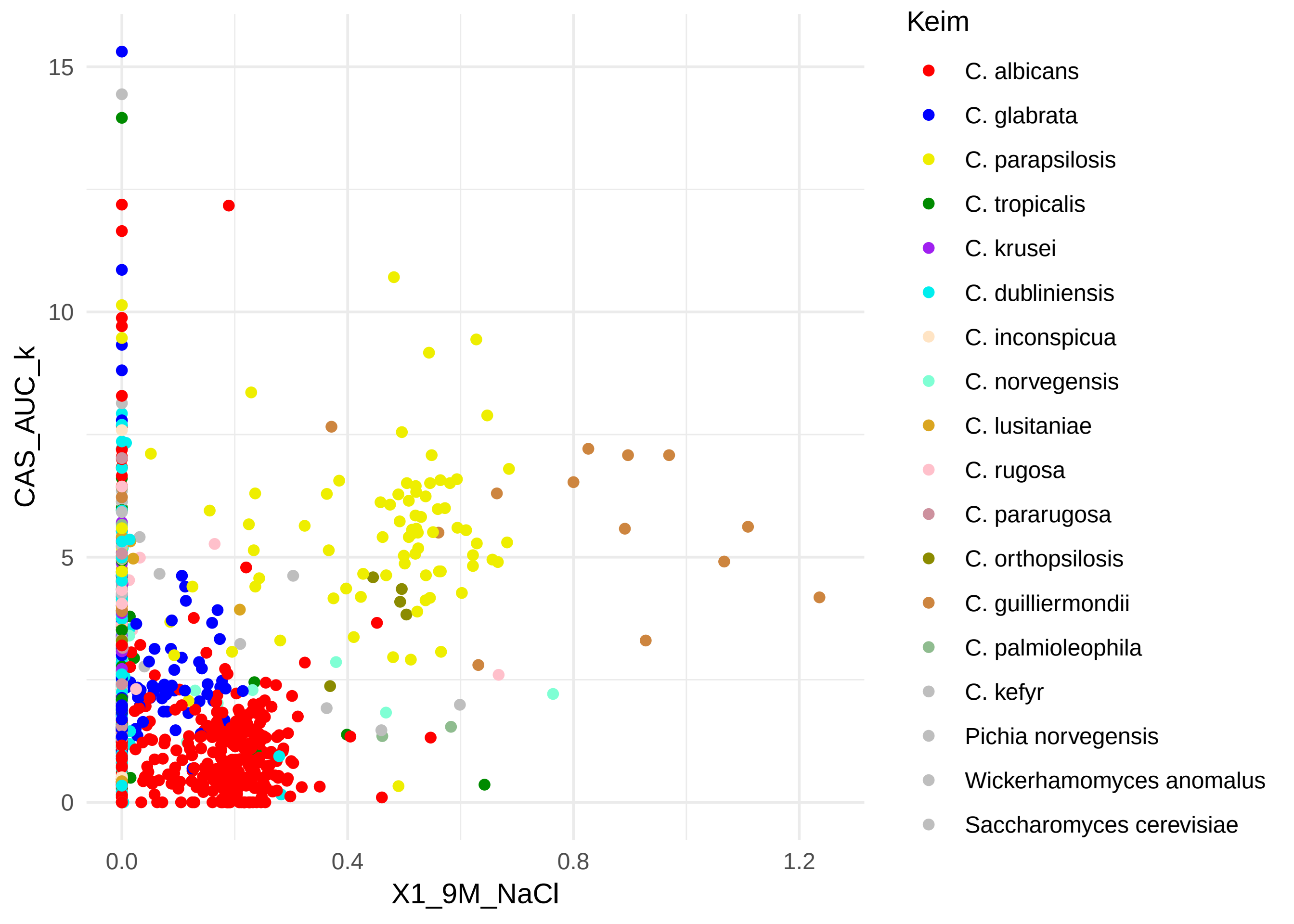

Keim
C. albicans
C. glabrata
C. parapsilosis
C. tropicalis
C. krusei
C. dubliniensis
C. inconspicua
C. norvegensis
C. lusitaniae
C. rugosa
C. pararugosa
C. orthopsilosis
C. guilliermondii
C. palmioleophila
C. kefyr
Pichia norvegensis
Wickerhamomyces anomalus
Saccharomyces cerevisiae
CAS_AUC_k
15
10
5
0
0.0
0.4
0.8
1.2
X1_9M_NaCl

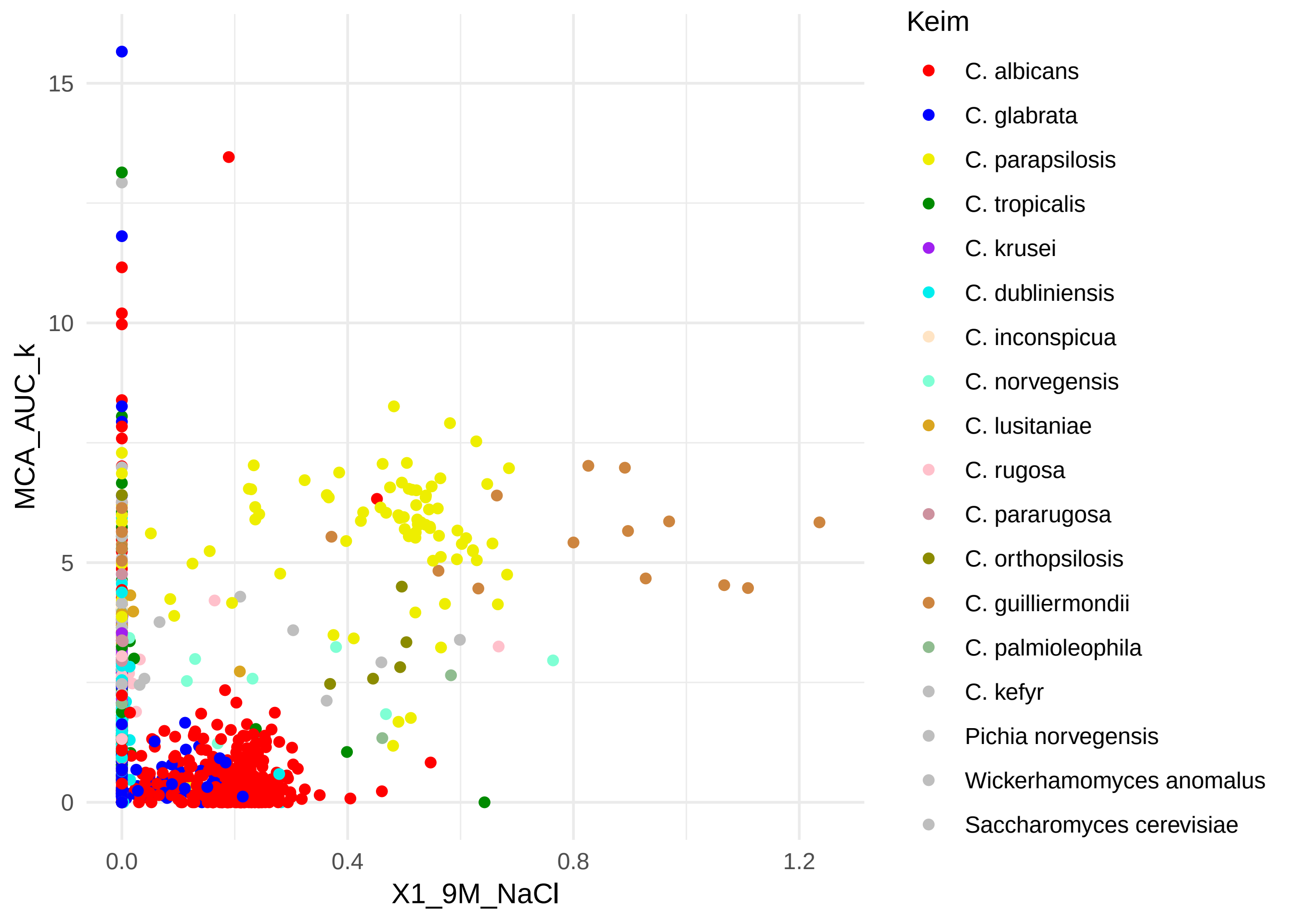

Keim
C. albicans
C. glabrata
C. parapsilosis
C. tropicalis
C. krusei
C. dubliniensis
C. inconspicua
C. norvegensis
C. lusitaniae
C. rugosa
C. pararugosa
C. orthopsilosis
C. guilliermondii
C. palmioleophila
C. kefyr
Pichia norvegensis
Wickerhamomyces anomalus
Saccharomyces cerevisiae
MCA_AUC_k
X1_9M_NaCl
0
5
10
15
0.0
0.4
0.8
1.2

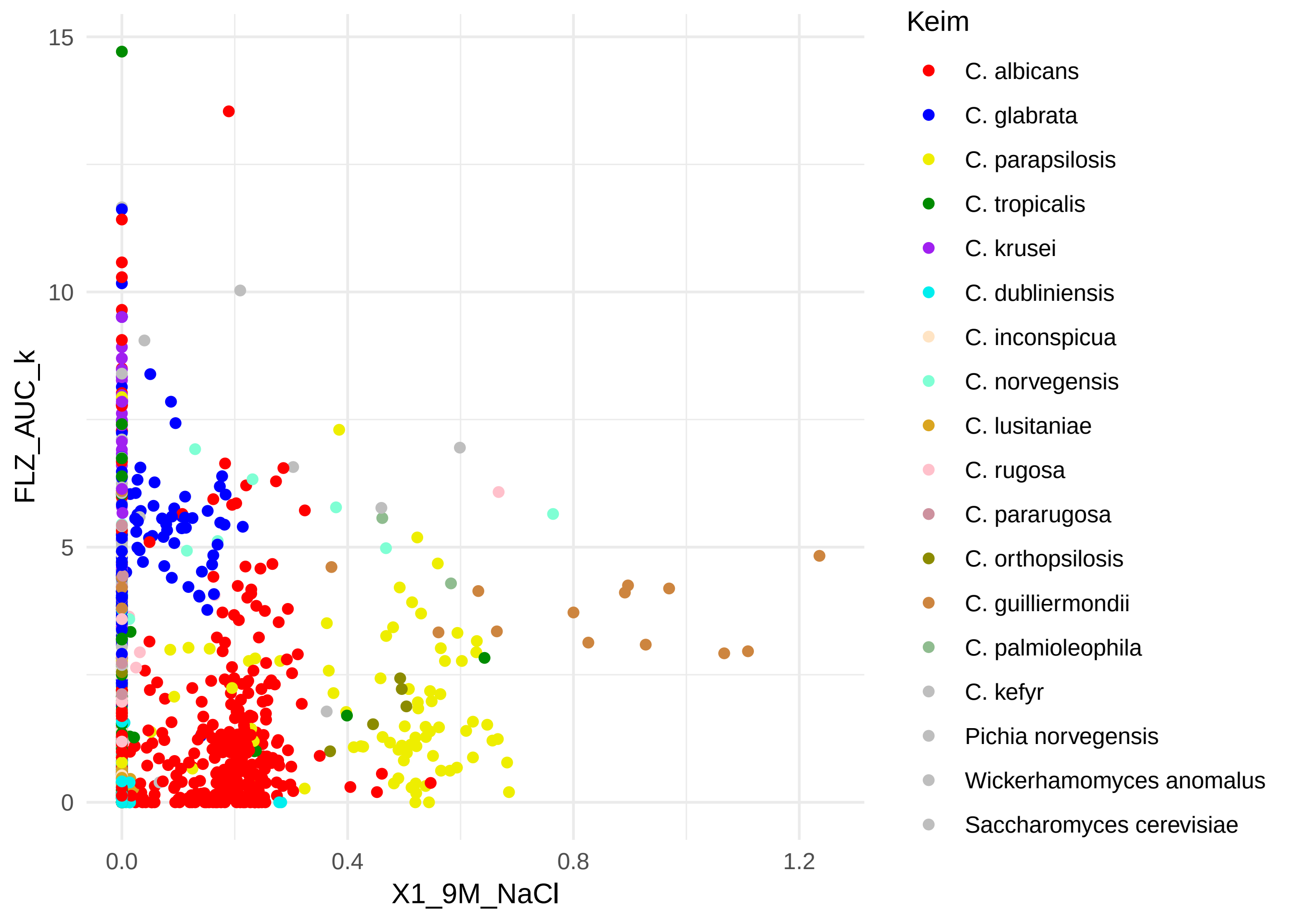
FLZ_AUC_k
X1_9M_NaCl
15
10
5
0
0.0
0.4
0.8
1.2
Keim
C. albicans
C. glabrata
C. parapsilosis
C. tropicalis
C. krusei
C. dubliniensis
C. inconspicua
C. norvegensis
C. lusitaniae
C. rugosa
C. pararugosa
C. orthopsilosis
C. guilliermondii
C. palmioleophila
C. kefyr
Pichia norvegensis
Wickerhamomyces anomalus
Saccharomyces cerevisiae

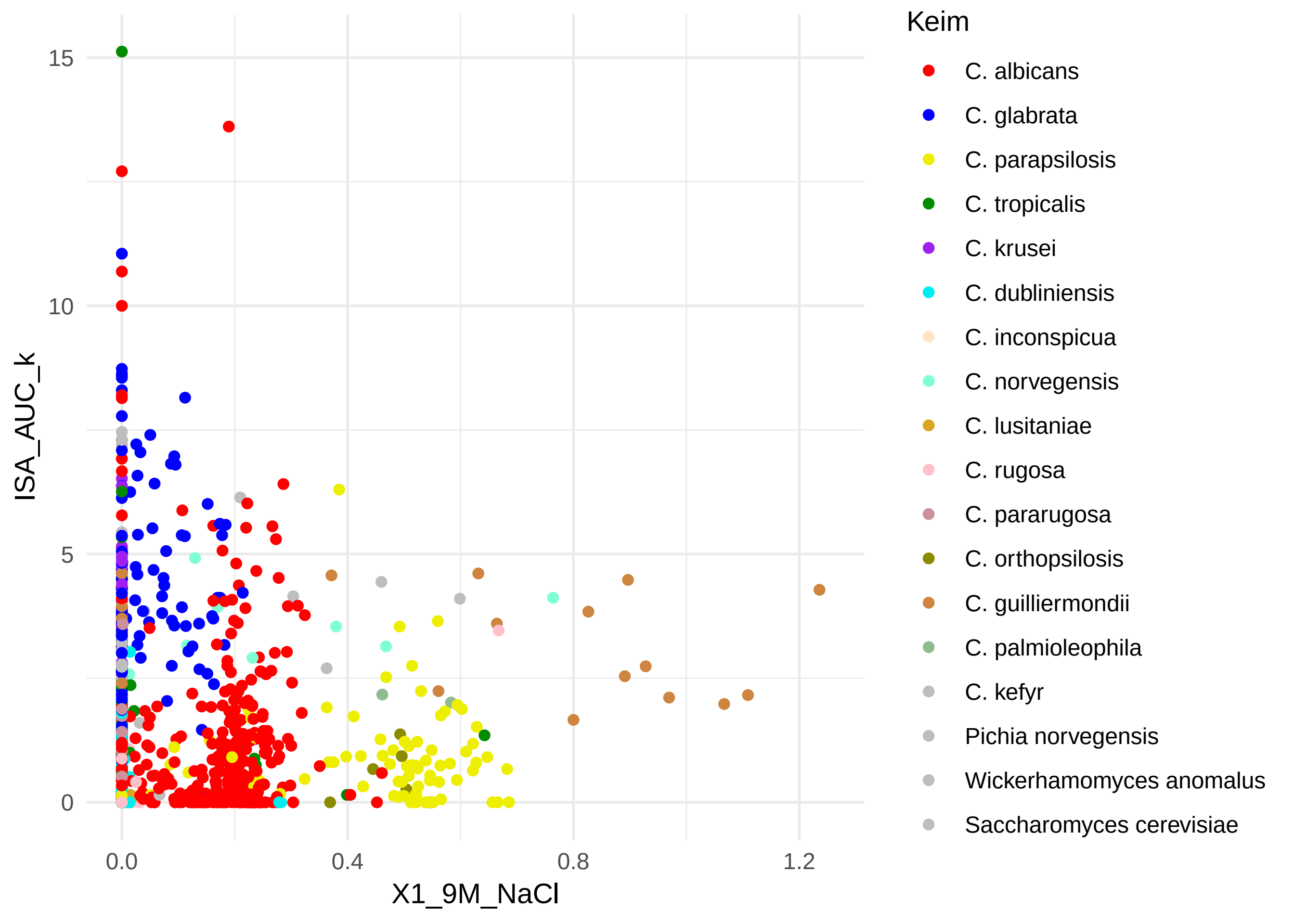
Keim
C. albicans
C. glabrata
C. parapsilosis
C. tropicalis
C. krusei
C. dubliniensis
C. inconspicua
C. norvegensis
C. lusitaniae
C. rugosa
C. pararugosa
C. orthopsilosis
C. guilliermondii
C. palmioleophila
C. kefyr
Pichia norvegensis
Wickerhamomyces anomalus
Saccharomyces cerevisiae
ISA_AUC_k
0
5
10
15
X1_9M_NaCl
0.0
0.4
0.8
1.2

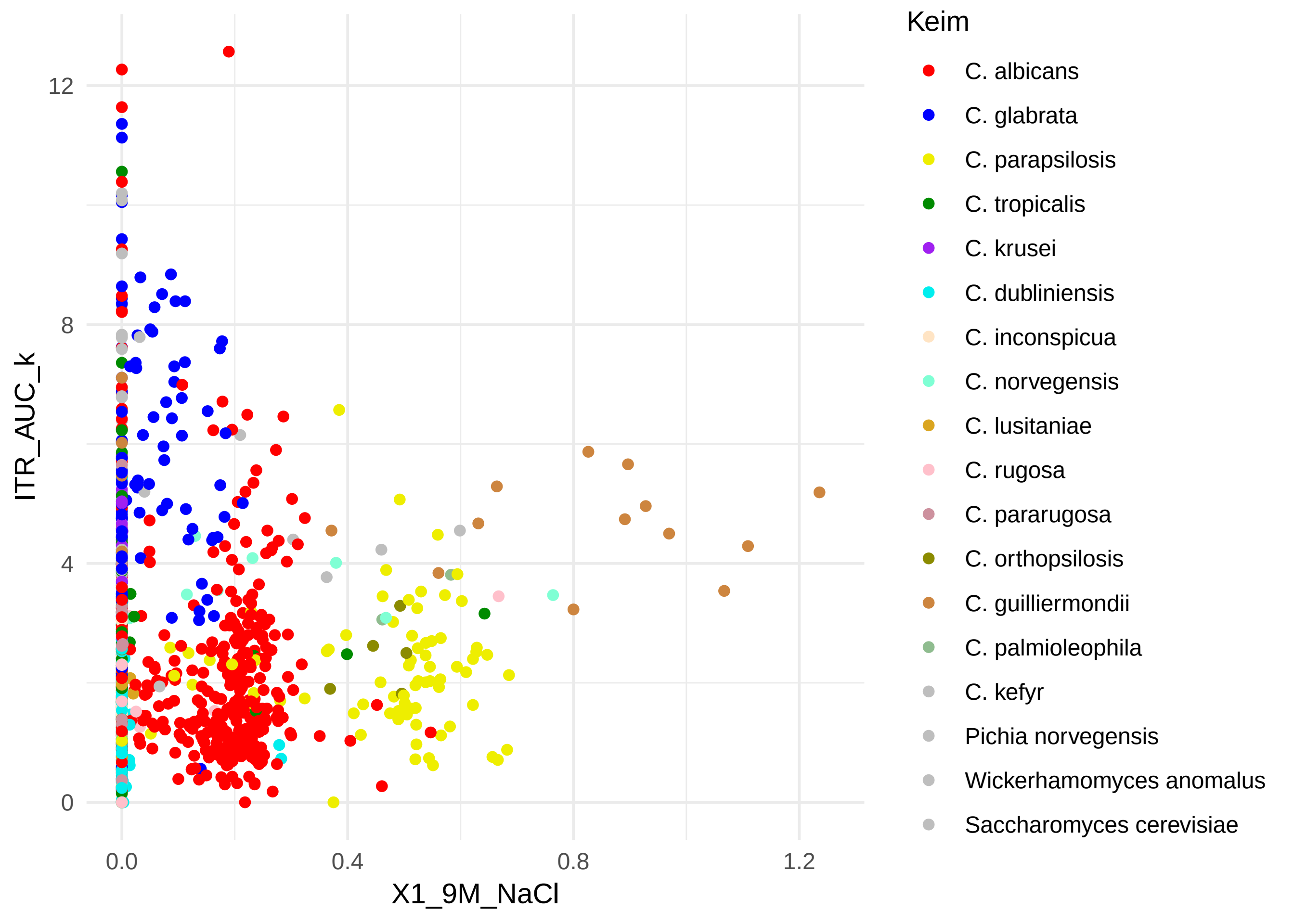

Keim
C. albicans
C. glabrata
C. parapsilosis
C. tropicalis
C. krusei
C. dubliniensis
C. inconspicua
C. norvegensis
C. lusitaniae
C. rugosa
C. pararugosa
C. orthopsilosis
C. guilliermondii
C. palmioleophila
C. kefyr
Pichia norvegensis
Wickerhamomyces anomalus
Saccharomyces cerevisiae
ITR_AUC_k
12
8
4
0
0.0
0.4
0.8
1.2
X1_9M_NaCl

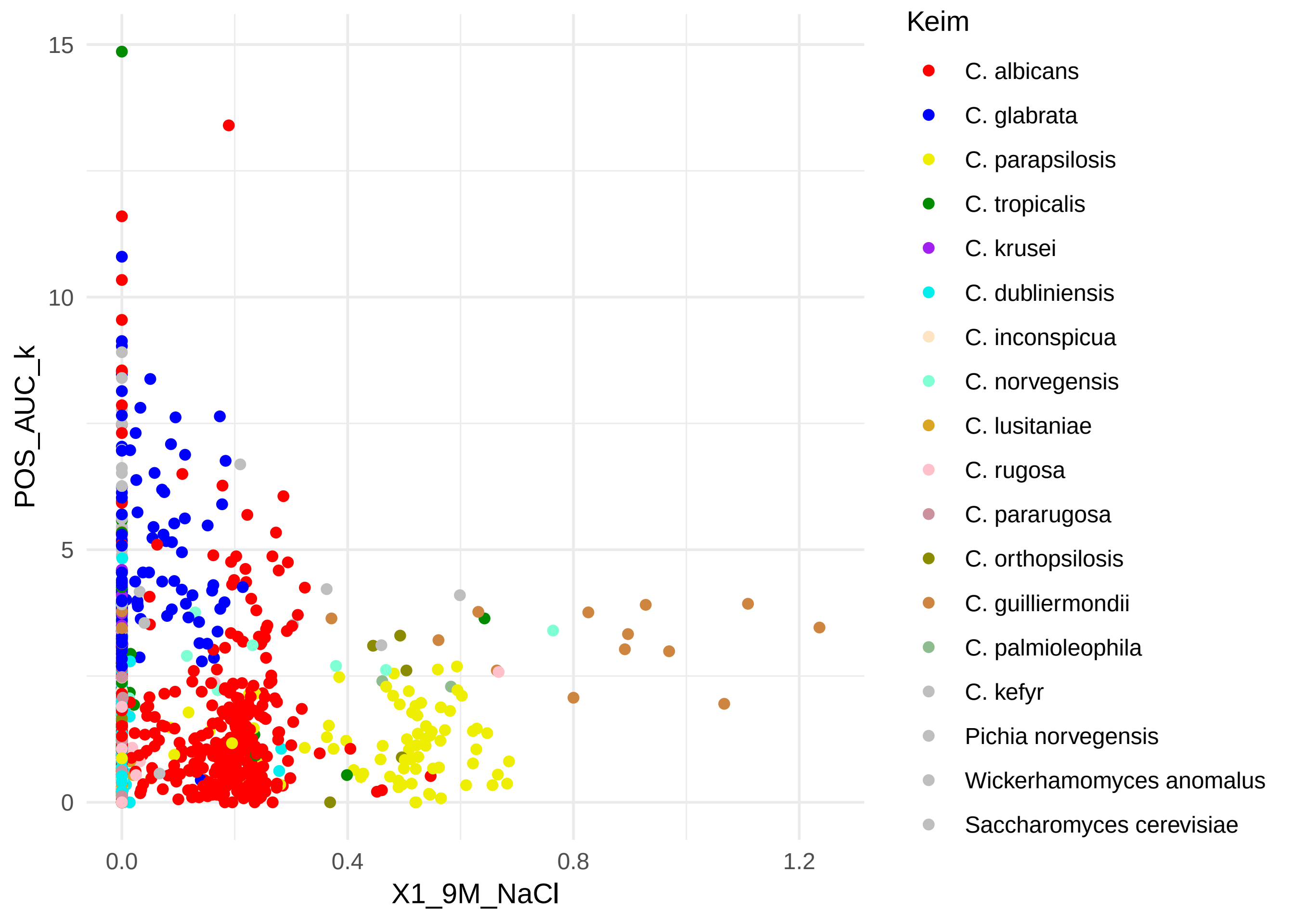

Keim
C. albicans
C. glabrata
C. parapsilosis
C. tropicalis
C. krusei
C. dubliniensis
C. inconspicua
C. norvegensis
C. lusitaniae
C. rugosa
C. pararugosa
C. orthopsilosis
C. guilliermondii
C. palmioleophila
C. kefyr
Pichia norvegensis
Wickerhamomyces anomalus
Saccharomyces cerevisiae
POS_AUC_k
X1_9M_NaCl
0
5
10
15
0.0
0.4
0.8
1.2

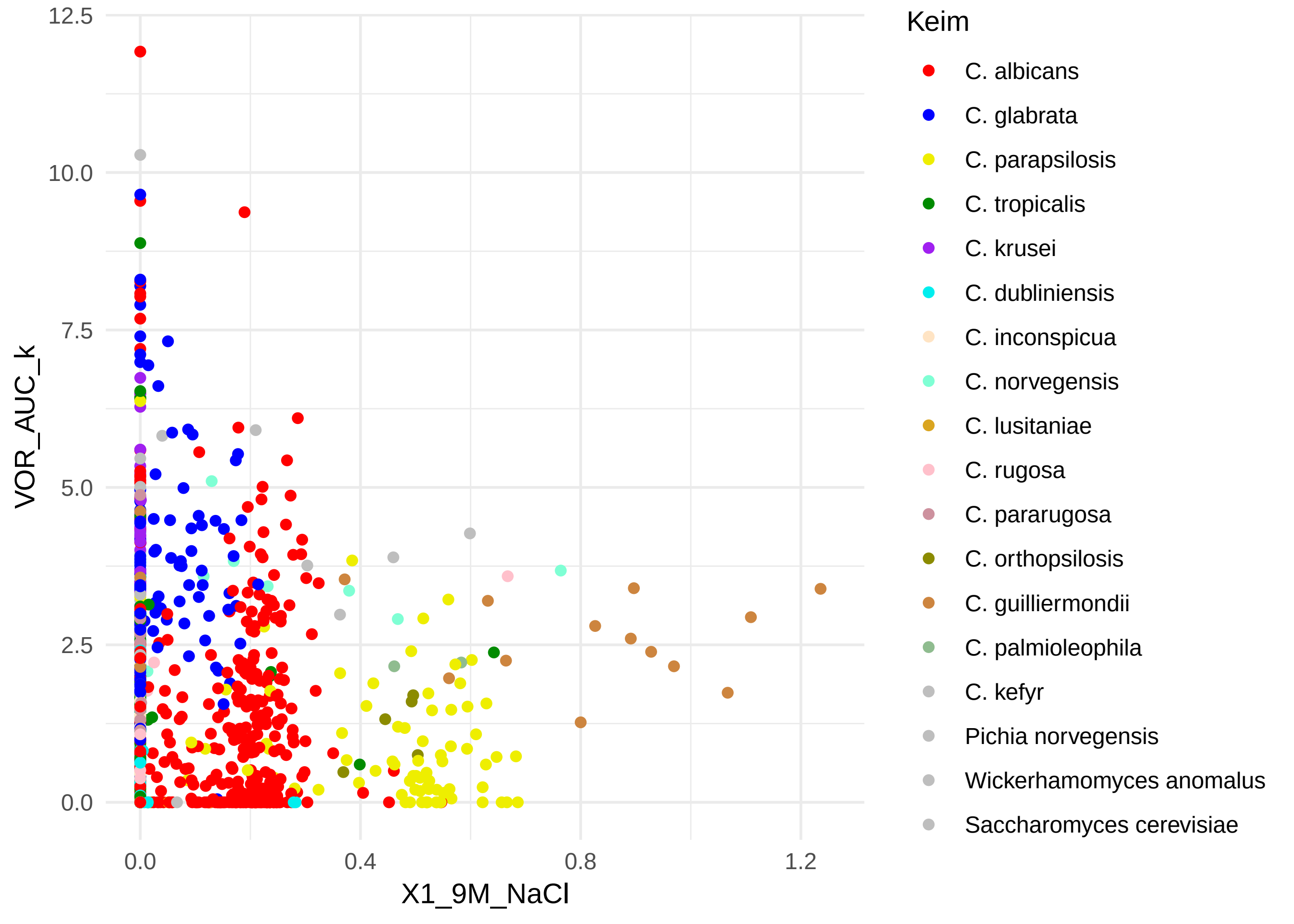

Keim
C. albicans
C. glabrata
C. parapsilosis
C. tropicalis
C. krusei
C. dubliniensis
C. inconspicua
C. norvegensis
C. lusitaniae
C. rugosa
C. pararugosa
C. orthopsilosis
C. guilliermondii
C. palmioleophila
C. kefyr
Pichia norvegensis
Wickerhamomyces anomalus
Saccharomyces cerevisiae
VOR_AUC_k
X1_9M_NaCl
12.5
10.0
7.5
5.0
2.5
0.0
0.0
0.4
0.8
1.2

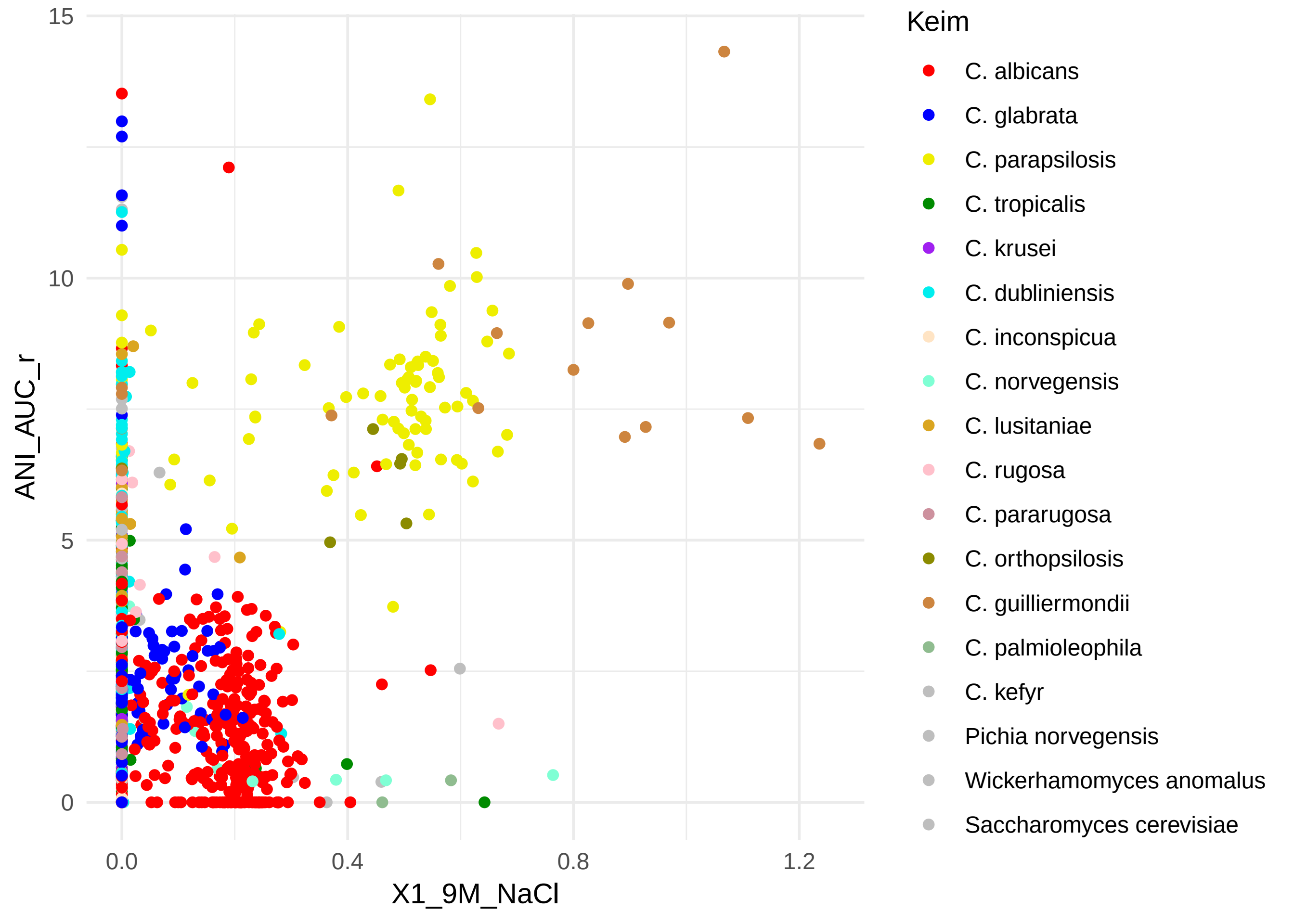

15
10
5
0
ANI_AUC_r
0.0
0.4
0.8
1.2
X1_9M_NaCl
Keim
C. albicans
C. glabrata
C. parapsilosis
C. tropicalis
C. krusei
C. dubliniensis
C. inconspicua
C. norvegensis
C. lusitaniae
C. rugosa
C. pararugosa
C. orthopsilosis
C. guilliermondii
C. palmioleophila
C. kefyr
Pichia norvegensis
Wickerhamomyces anomalus
Saccharomyces cerevisiae

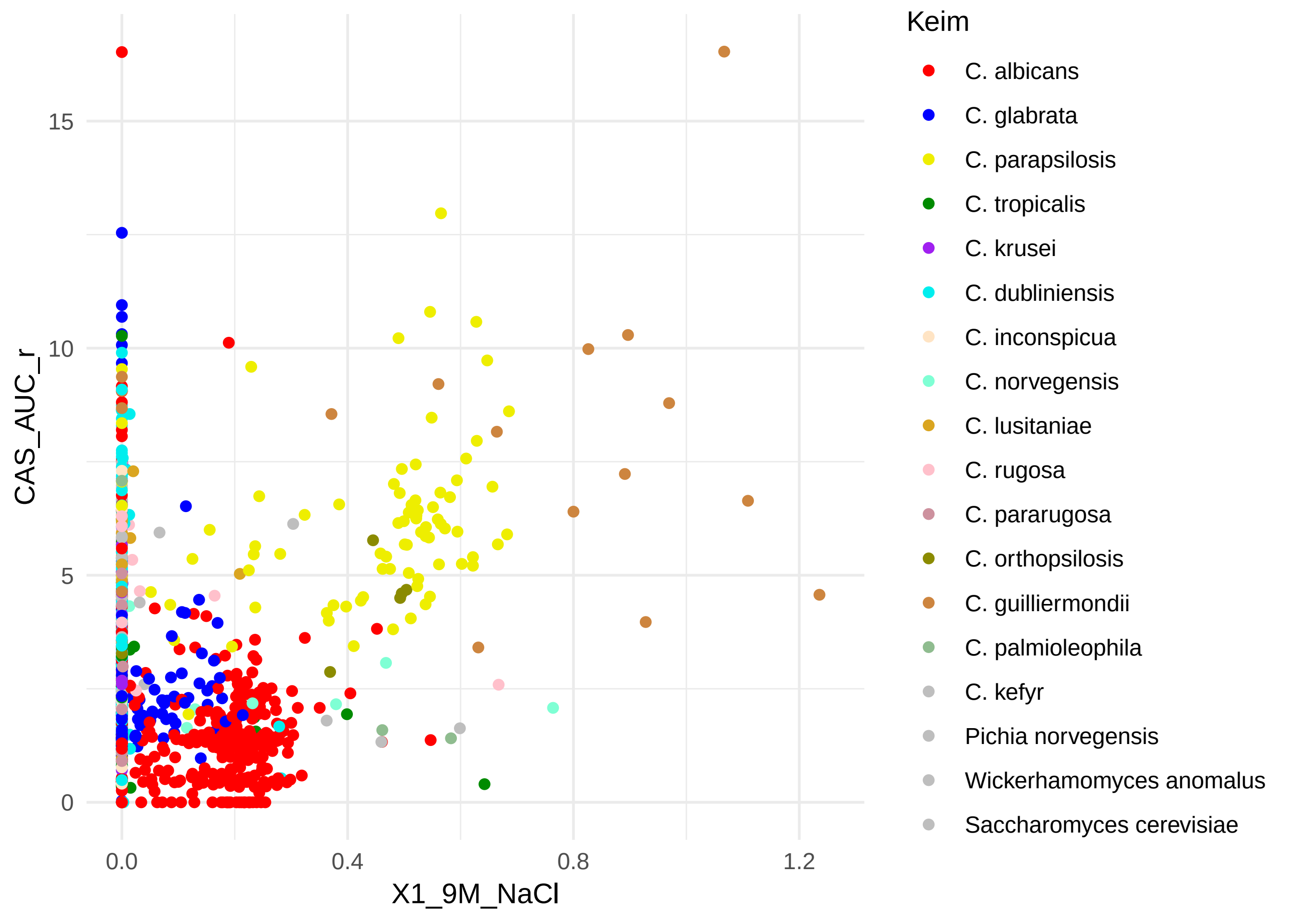

Keim
C. albicans
C. glabrata
C. parapsilosis
C. tropicalis
C. krusei
C. dubliniensis
C. inconspicua
C. norvegensis
C. lusitaniae
C. rugosa
C. pararugosa
C. orthopsilosis
C. guilliermondii
C. palmioleophila
C. kefyr
Pichia norvegensis
Wickerhamomyces anomalus
Saccharomyces cerevisiae
15
10
5
0
0.0
0.4
0.8
1.2
CAS_AUC_r
X1_9M_NaCl

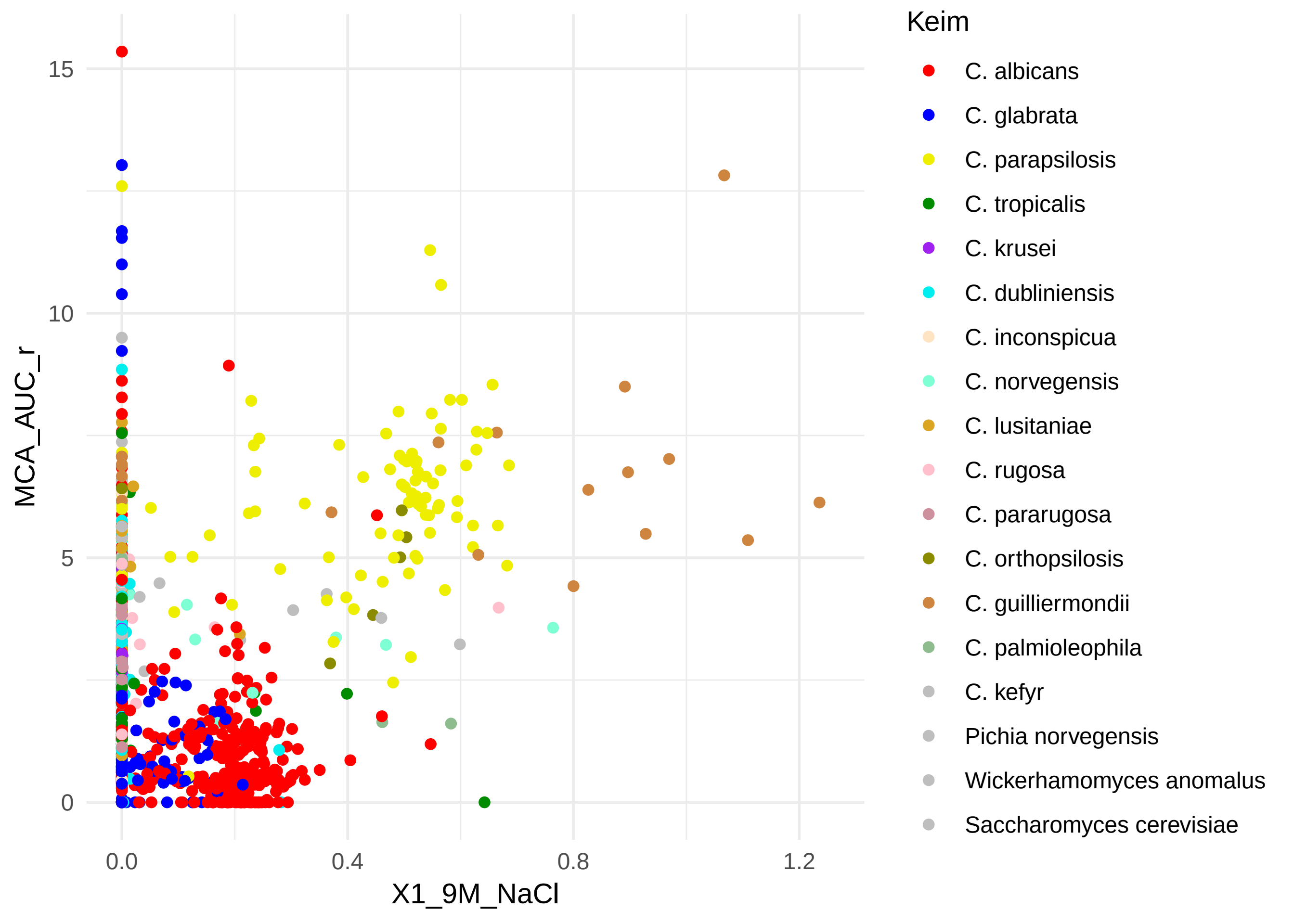

Keim
C. albicans
C. glabrata
C. parapsilosis
C. tropicalis
C. krusei
C. dubliniensis
C. inconspicua
C. norvegensis
C. lusitaniae
C. rugosa
C. pararugosa
C. orthopsilosis
C. guilliermondii
C. palmioleophila
C. kefyr
Pichia norvegensis
Wickerhamomyces anomalus
Saccharomyces cerevisiae
MCA_AUC_r
X1_9M_NaCl
15
10
5
0
0.0
0.4
0.8
1.2

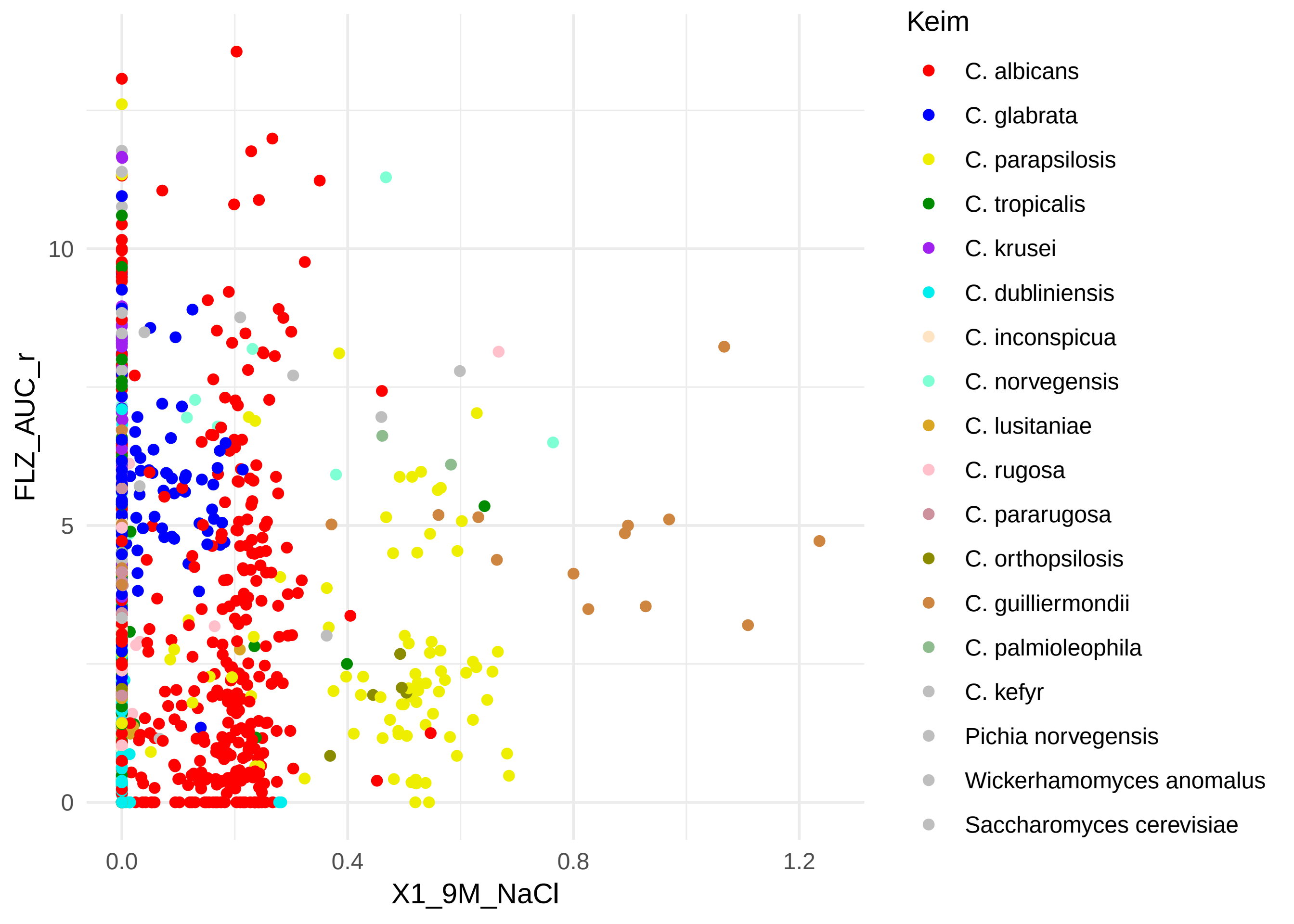
Keim
C. albicans
C. glabrata
C. parapsilosis
C. tropicalis
C. krusei
C. dubliniensis
C. inconspicua
C. norvegensis
C. lusitaniae
C. rugosa
C. pararugosa
C. orthopsilosis
C. guilliermondii
C. palmioleophila
C. kefyr
Pichia norvegensis
Wickerhamomyces anomalus
Saccharomyces cerevisiae
FLZ_AUC_r
10
5
0
0.0
0.4
0.8
1.2
X1_9M_NaCl

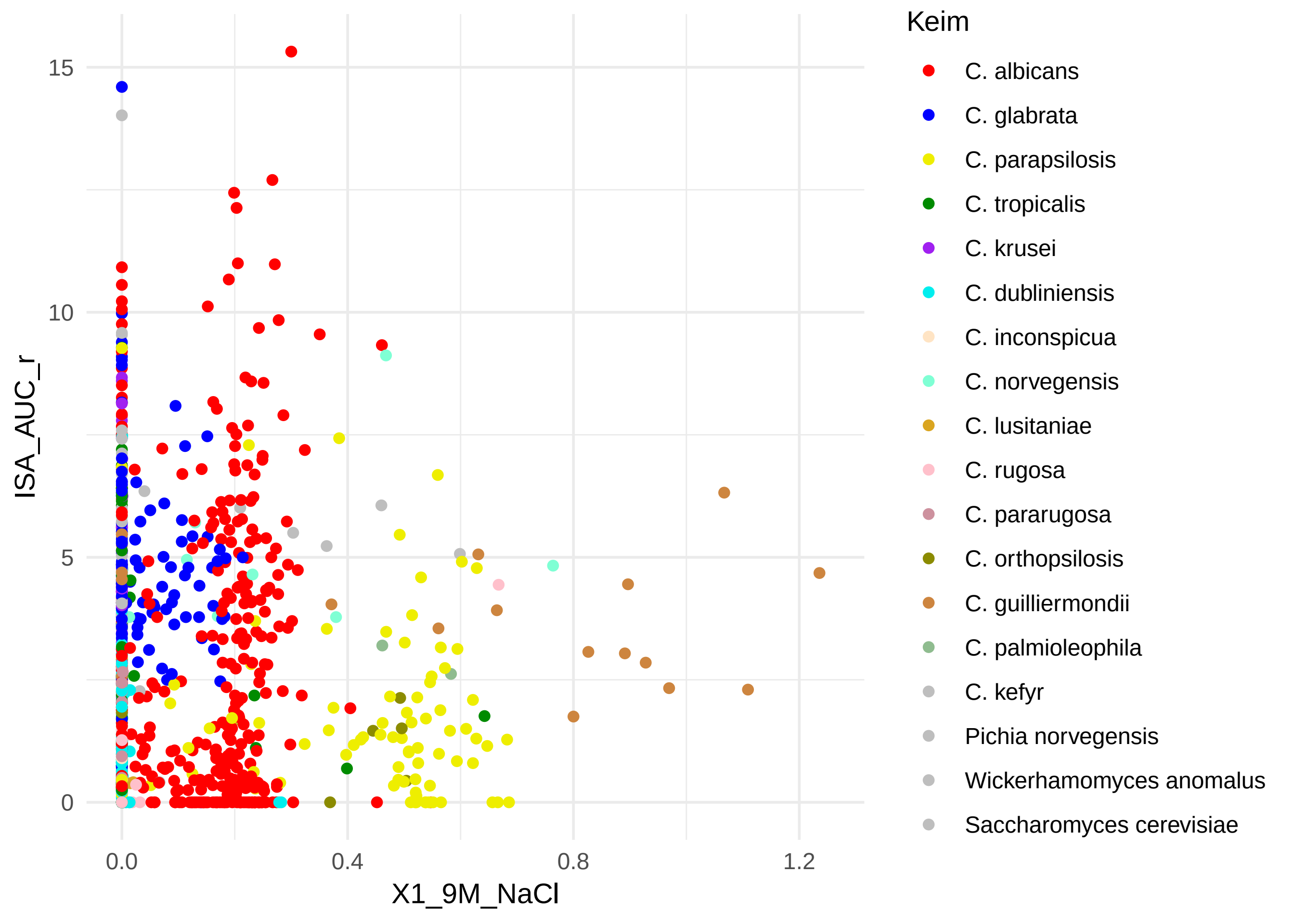
15
10
5
0
ISA_AUC_r
0.0
0.4
0.8
1.2
X1_9M_NaCl
Keim
C. albicans
C. glabrata
C. parapsilosis
C. tropicalis
C. krusei
C. dubliniensis
C. inconspicua
C. norvegensis
C. lusitaniae
C. rugosa
C. pararugosa
C. orthopsilosis
C. guilliermondii
C. palmioleophila
C. kefyr
Pichia norvegensis
Wickerhamomyces anomalus
Saccharomyces cerevisiae

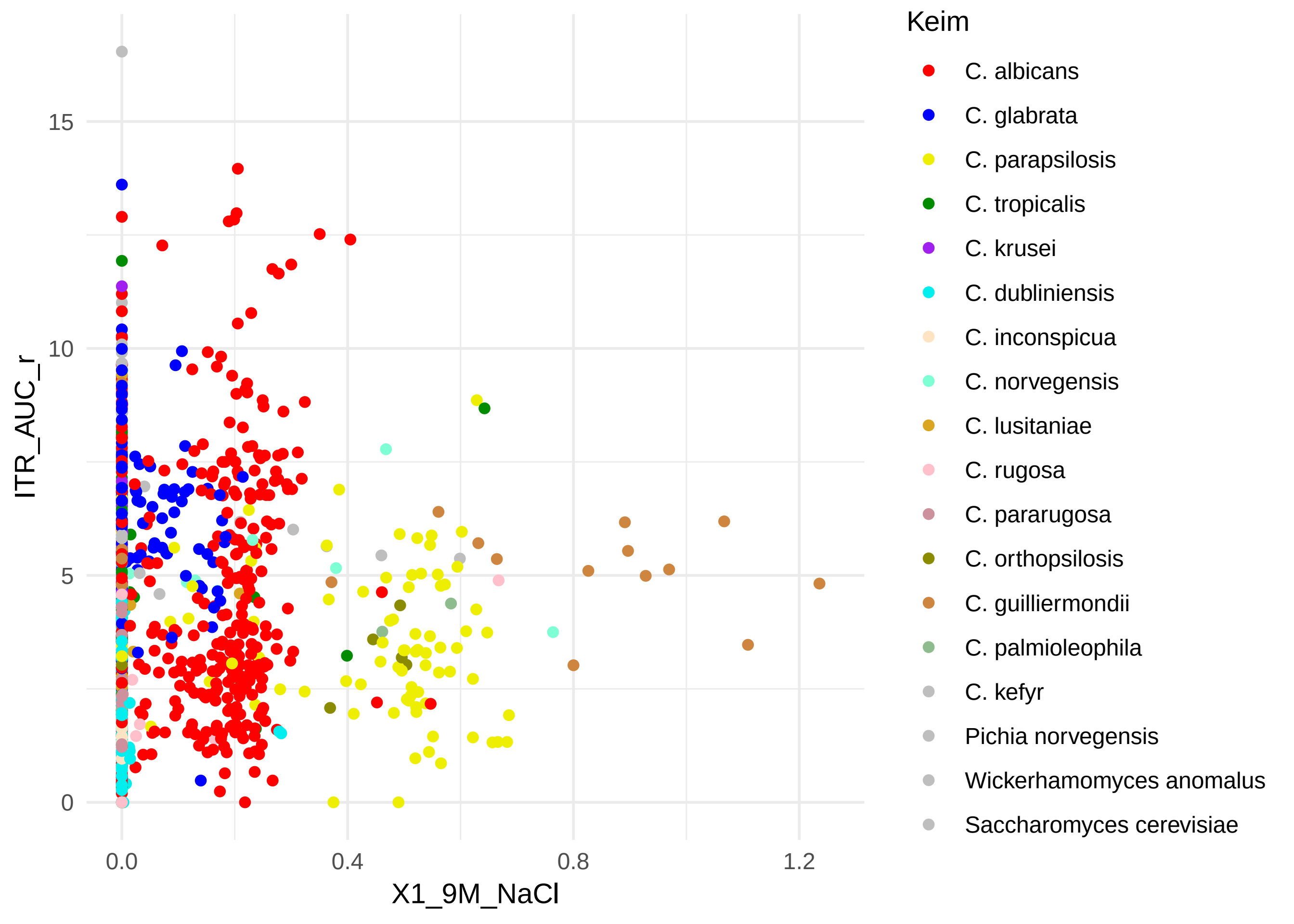
Keim
C. albicans
C. glabrata
C. parapsilosis
C. tropicalis
C. krusei
C. dubliniensis
C. inconspicua
C. norvegensis
C. lusitaniae
C. rugosa
C. pararugosa
C. orthopsilosis
C. guilliermondii
C. palmioleophila
C. kefyr
Pichia norvegensis
Wickerhamomyces anomalus
Saccharomyces cerevisiae
ITR_AUC_r
X1_9M_NaCl
0
5
10
15
0.0
0.4
0.8
1.2

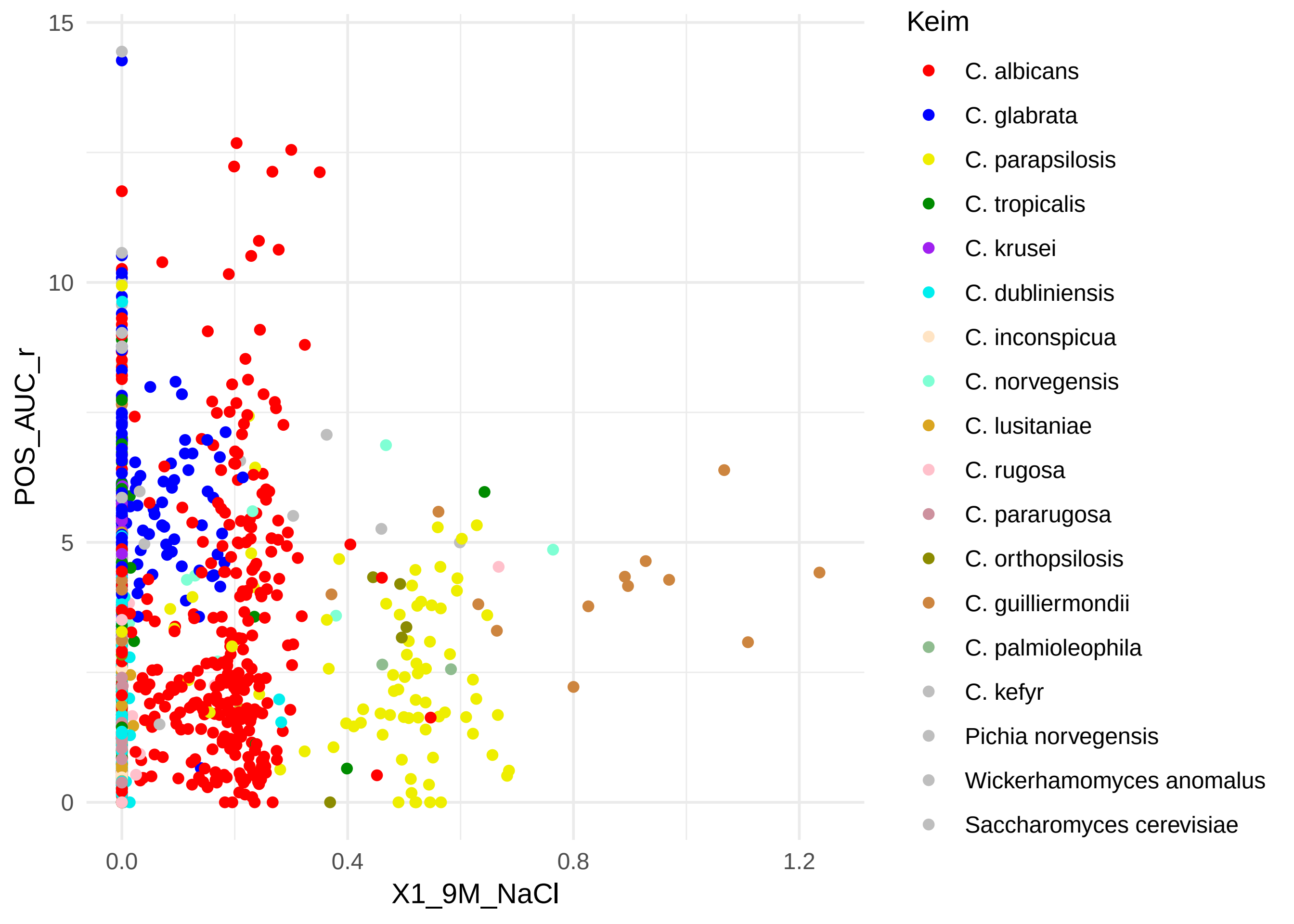

POS_AUC_r
X1_9M_NaCl
0
5
10
15
0.0
0.4
0.8
1.2
Keim
C. albicans
C. glabrata
C. parapsilosis
C. tropicalis
C. krusei
C. dubliniensis
C. inconspicua
C. norvegensis
C. lusitaniae
C. rugosa
C. pararugosa
C. orthopsilosis
C. guilliermondii
C. palmioleophila
C. kefyr
Pichia norvegensis
Wickerhamomyces anomalus
Saccharomyces cerevisiae

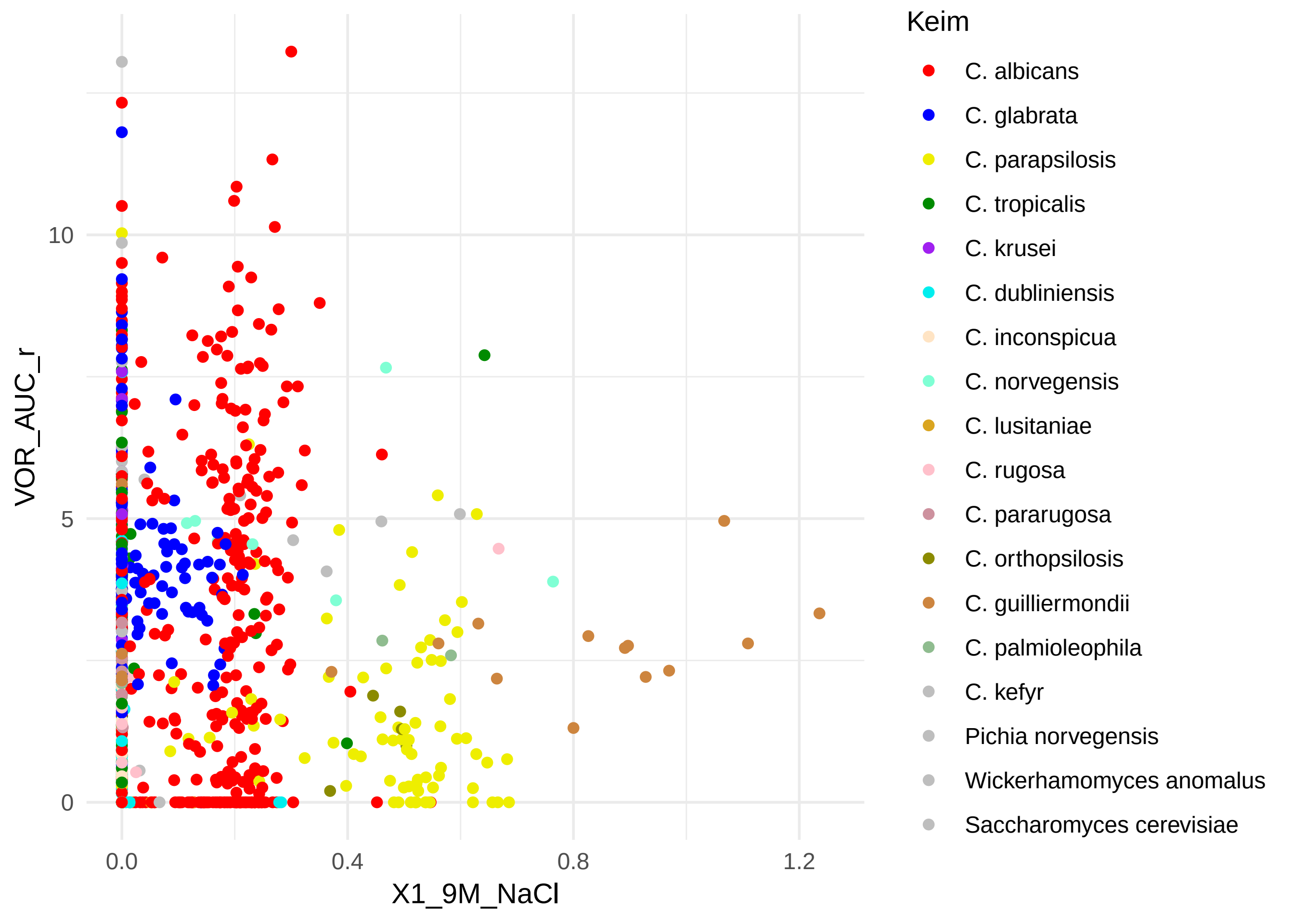

VOR_AUC_r
10
5
0
0.0
0.4
0.8
1.2
X1_9M_NaCl
Keim
C. albicans
C. glabrata
C. parapsilosis
C. tropicalis
C. krusei
C. dubliniensis
C. inconspicua
C. norvegensis
C. lusitaniae
C. rugosa
C. pararugosa
C. orthopsilosis
C. guilliermondii
C. palmioleophila
C. kefyr
Pichia norvegensis
Wickerhamomyces anomalus
Saccharomyces cerevisiae

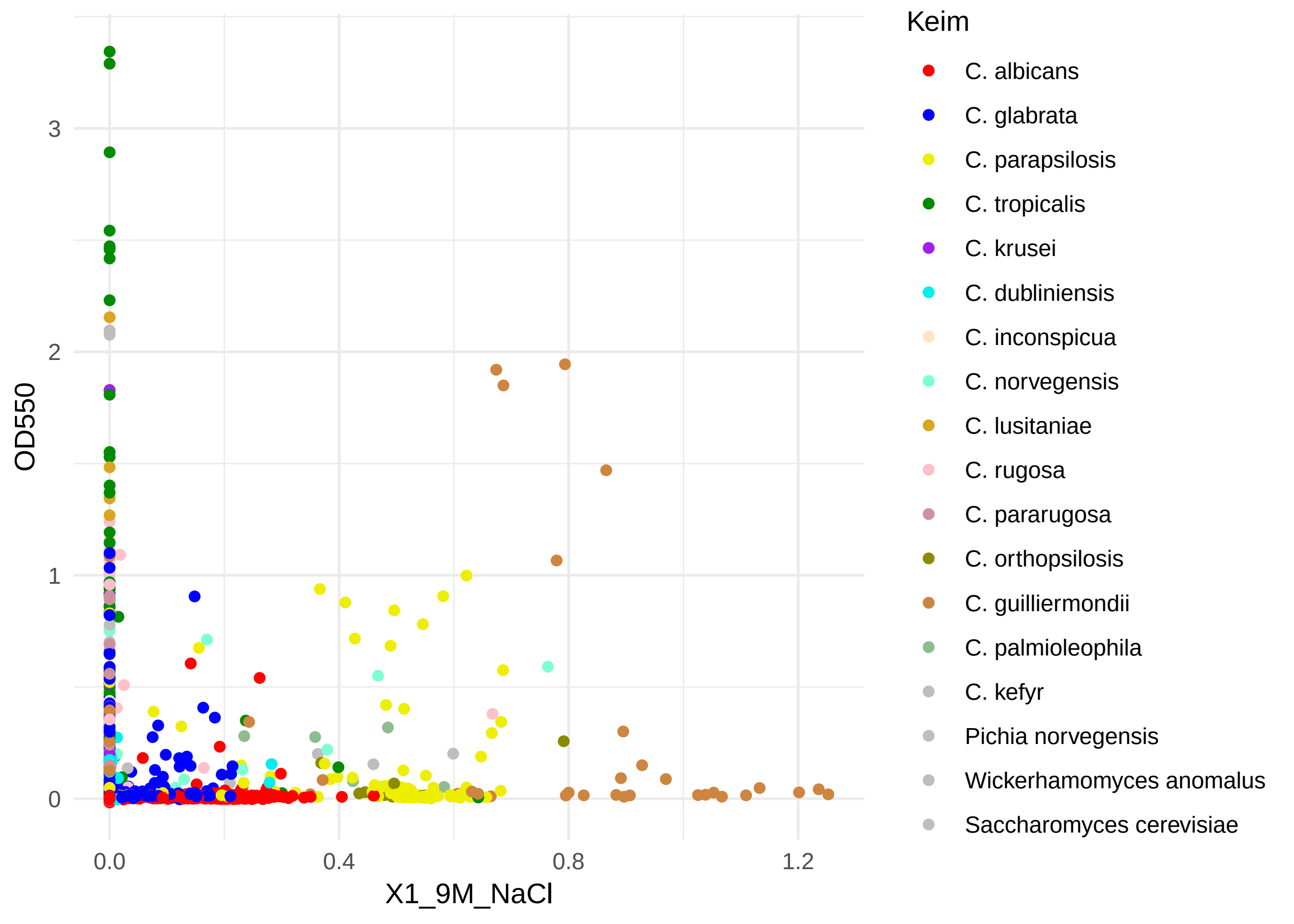

OD550
3
2
1
0
0.0
0.4
0.8
1.2
X1_9M_NaCl
Keim
C. albicans
C. glabrata
C. parapsilosis
C. tropicalis
C. krusei
C. dubliniensis
C. inconspicua
C. norvegensis
C. lusitaniae
C. rugosa
C. pararugosa
C. orthopsilosis
C. guilliermondii
C. palmioleophila
C. kefyr
Pichia norvegensis
Wickerhamomyces anomalus
Saccharomyces cerevisiae

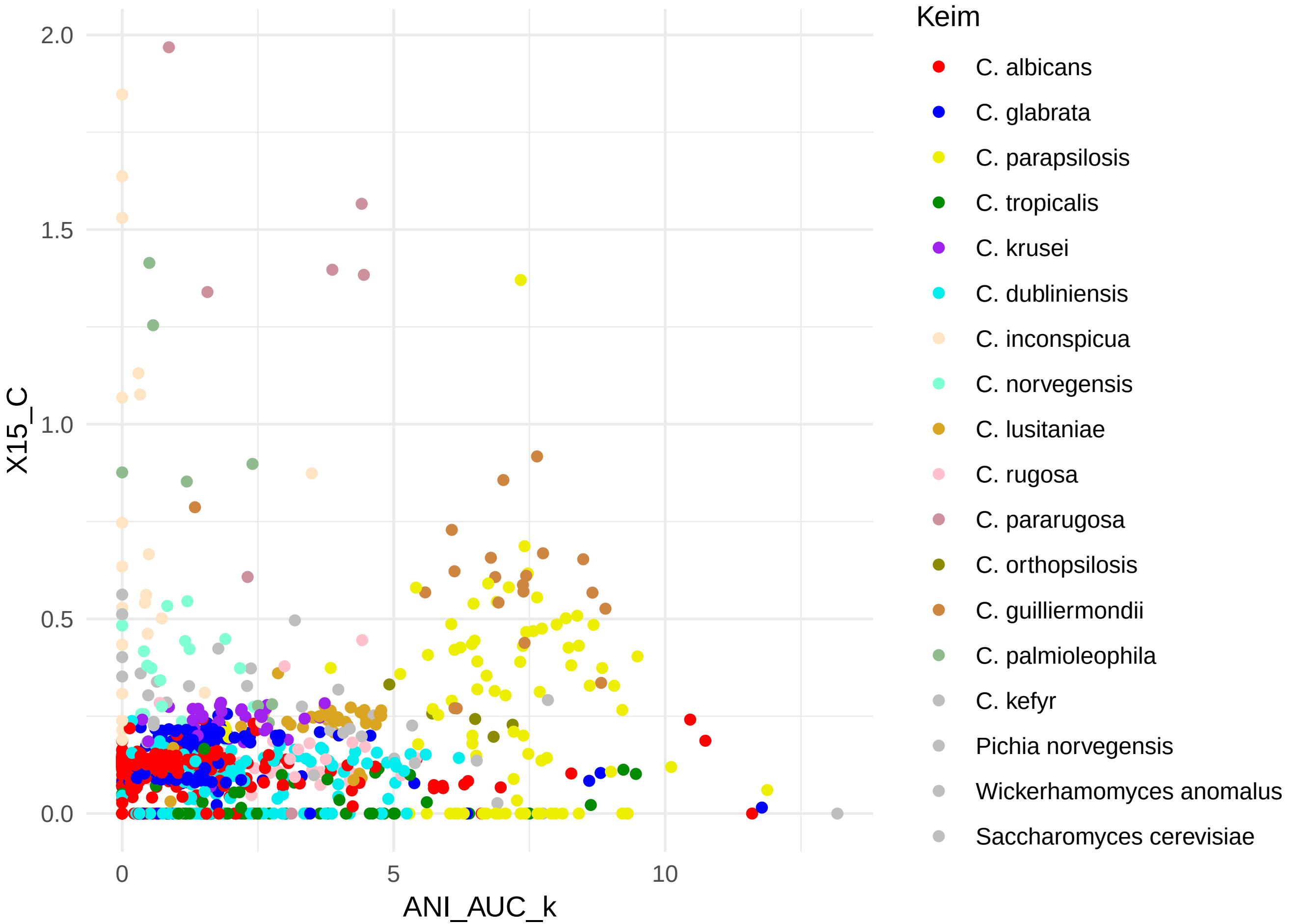

2.0
1.5
1.0
0.5
0.0
X15_C
0
5
10
ANI_AUC_k
Keim
C. albicans
C. glabrata
C. parapsilosis
C. tropicalis
C. krusei
C. dubliniensis
C. inconspicua
C. norvegensis
C. lusitaniae
C. rugosa
C. pararugosa
C. orthopsilosis
C. guilliermondii
C. palmioleophila
C. kefyr
Pichia norvegensis
Wickerhamomyces anomalus
Saccharomyces cerevisiae

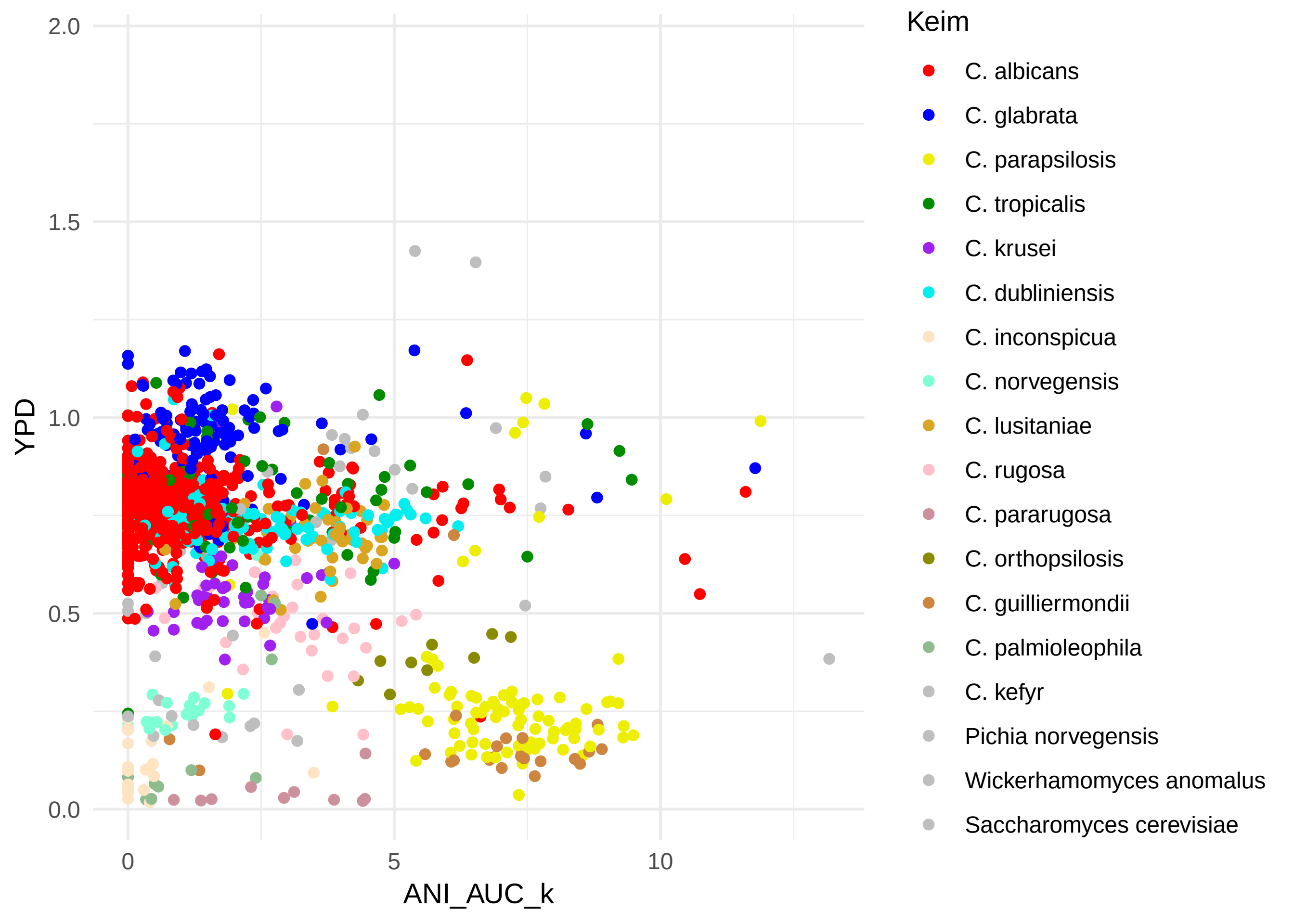
Keim
C. albicans
C. glabrata
C. parapsilosis
C. tropicalis
C. krusei
C. dubliniensis
C. inconspicua
C. norvegensis
C. lusitaniae
C. rugosa
C. pararugosa
C. orthopsilosis
C. guilliermondii
C. palmioleophila
C. kefyr
Pichia norvegensis
Wickerhamomyces anomalus
Saccharomyces cerevisiae
YPD
2.0
1.5
1.0
0.5
0.0
0
5
10
ANI_AUC_k

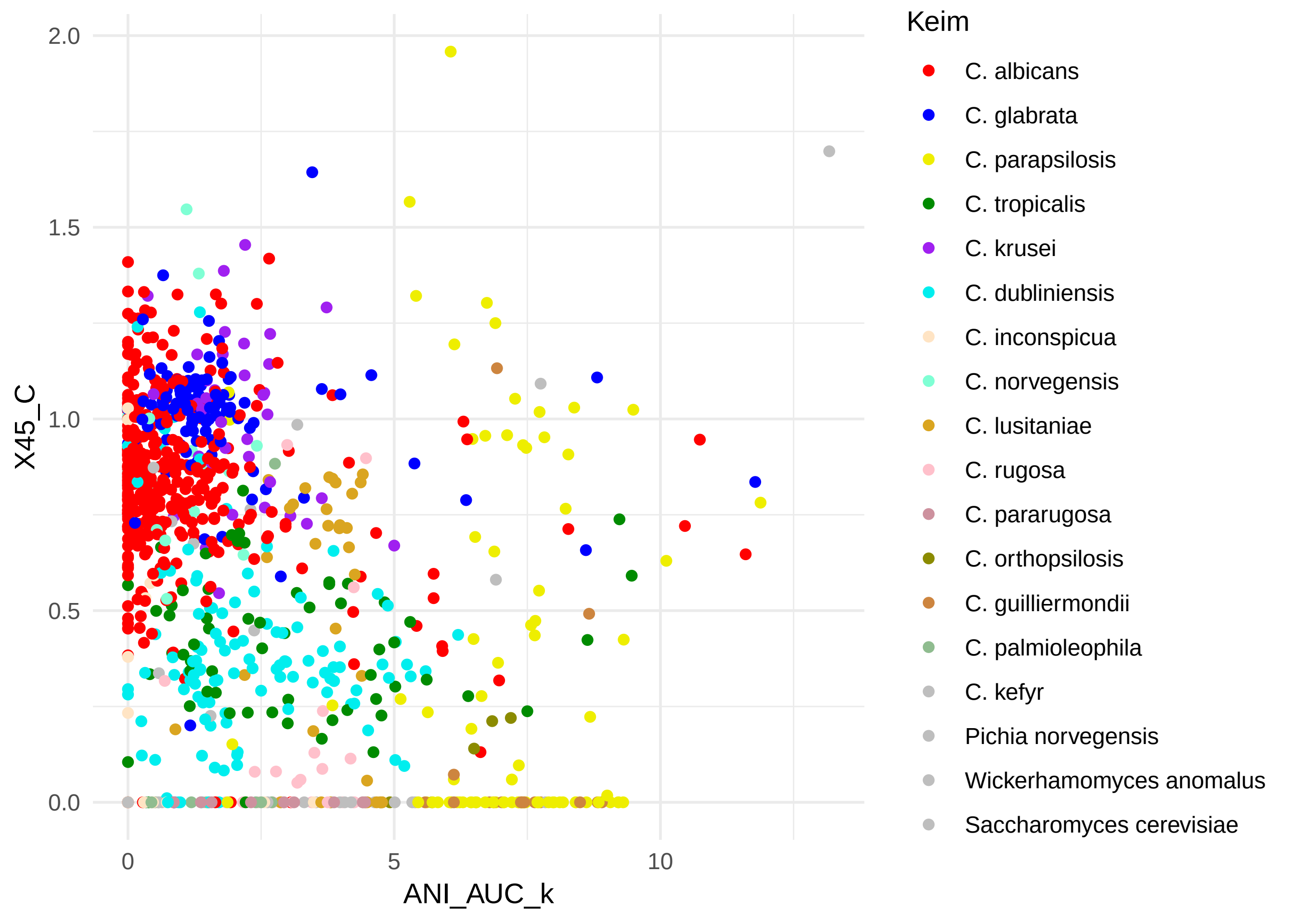
X45_C
ANI_AUC_k
2.0
1.5
1.0
0.5
0.0
0
5
10
Keim
C. albicans
C. glabrata
C. parapsilosis
C. tropicalis
C. krusei
C. dubliniensis
C. inconspicua
C. norvegensis
C. lusitaniae
C. rugosa
C. pararugosa
C. orthopsilosis
C. guilliermondii
C. palmioleophila
C. kefyr
Pichia norvegensis
Wickerhamomyces anomalus
Saccharomyces cerevisiae

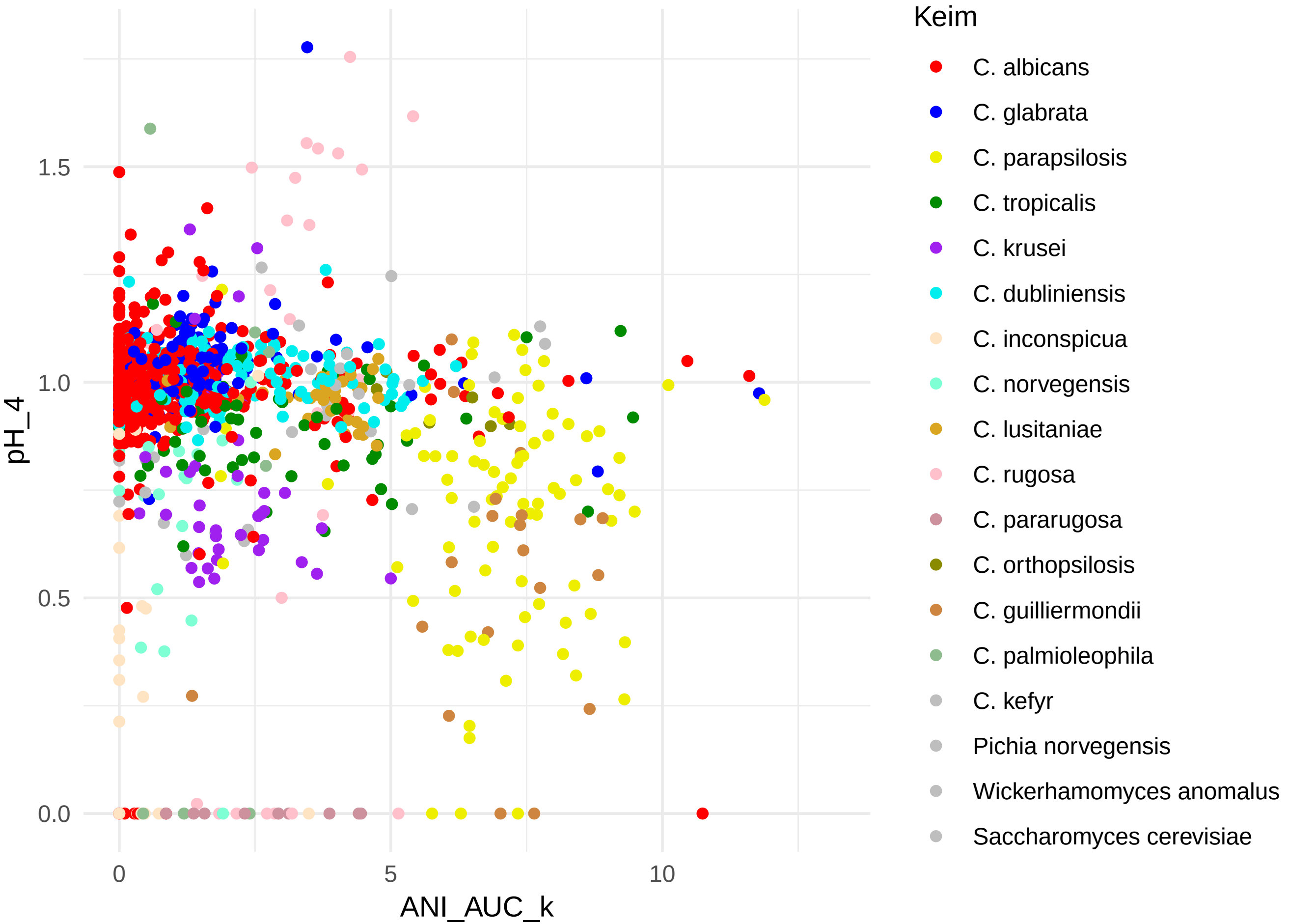

Keim
C. albicans
C. glabrata
C. parapsilosis
C. tropicalis
C. krusei
C. dubliniensis
C. inconspicua
C. norvegensis
C. lusitaniae
C. rugosa
C. pararugosa
C. orthopsilosis
C. guilliermondii
C. palmioleophila
C. kefyr
Pichia norvegensis
Wickerhamomyces anomalus
Saccharomyces cerevisiae
pH_4
1.5
1.0
0.5
0.0
0
5
10
ANI_AUC_k

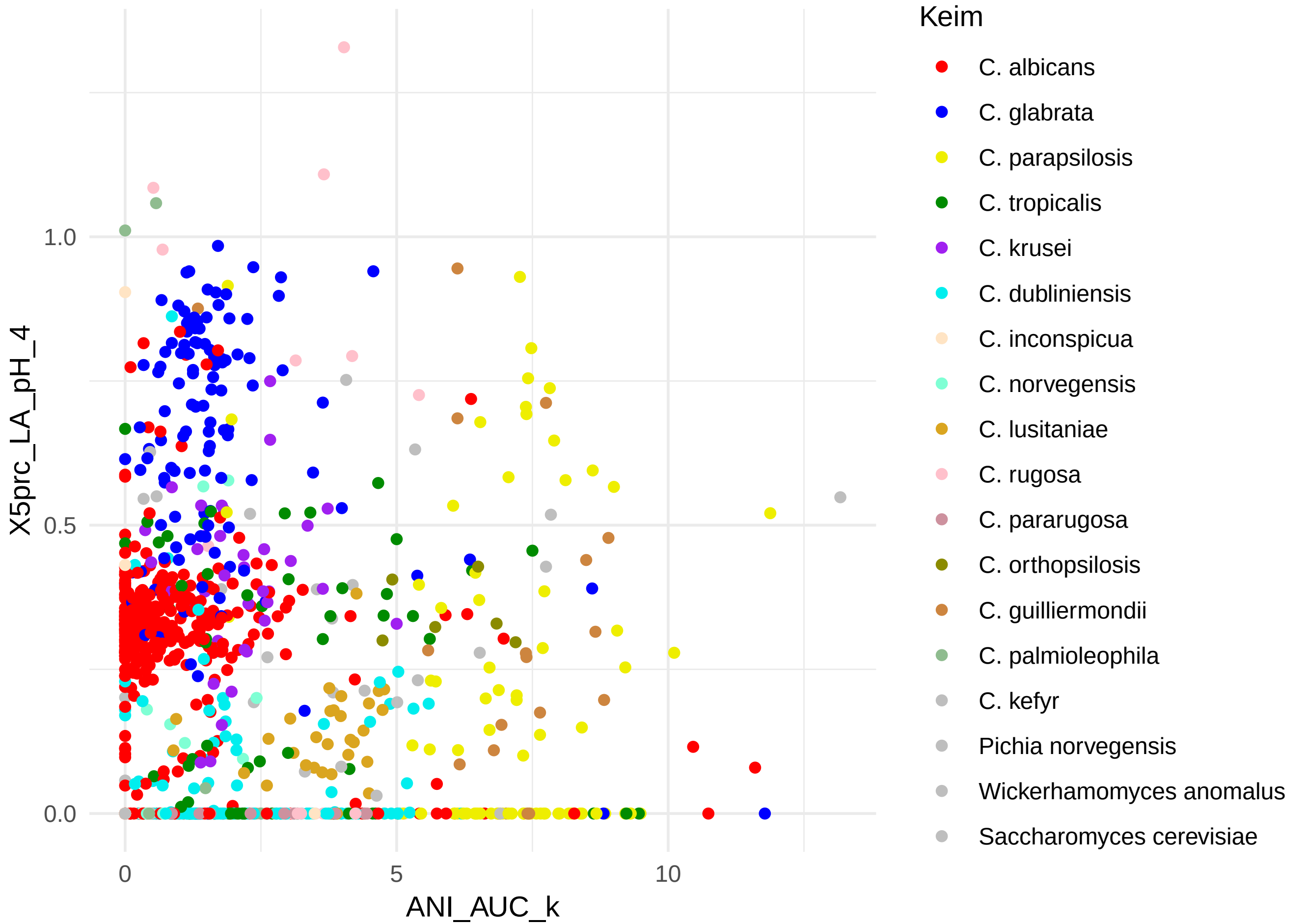
Keim
C. albicans
C. glabrata
C. parapsilosis
C. tropicalis
C. krusei
C. dubliniensis
C. inconspicua
C. norvegensis
C. lusitaniae
C. rugosa
C. pararugosa
C. orthopsilosis
C. guilliermondii
C. palmioleophila
C. kefyr
Pichia norvegensis
Wickerhamomyces anomalus
Saccharomyces cerevisiae
X5prc_LA_pH_4
1.0
0.5
0.0
0
5
10
ANI_AUC_k

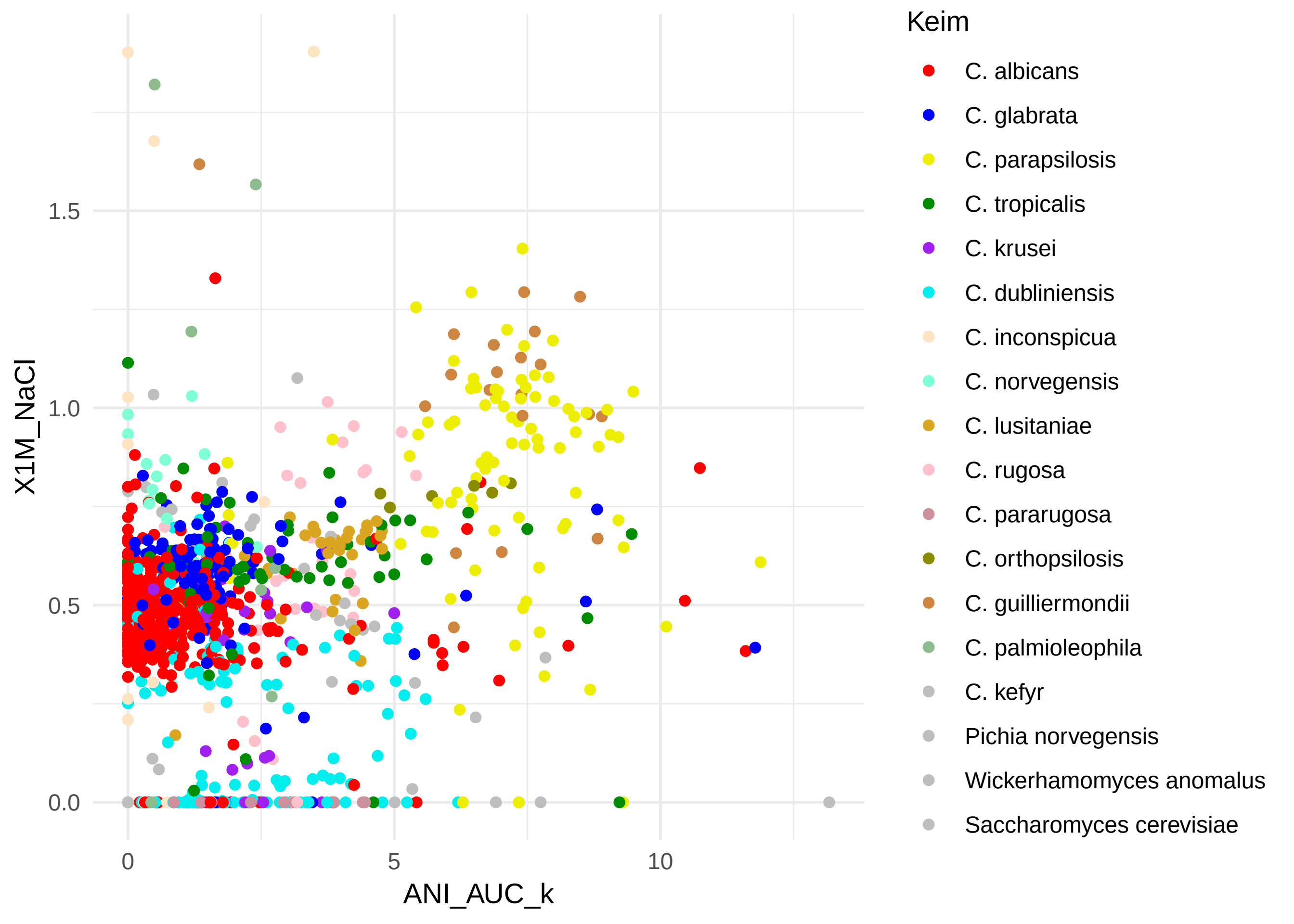

Keim
C. albicans
C. glabrata
C. parapsilosis
C. tropicalis
C. krusei
C. dubliniensis
C. inconspicua
C. norvegensis
C. lusitaniae
C. rugosa
C. pararugosa
C. orthopsilosis
C. guilliermondii
C. palmioleophila
C. kefyr
Pichia norvegensis
Wickerhamomyces anomalus
Saccharomyces cerevisiae
X1M_NaCl
ANI_AUC_k
0.0
0.5
1.0
1.5
0
5
10

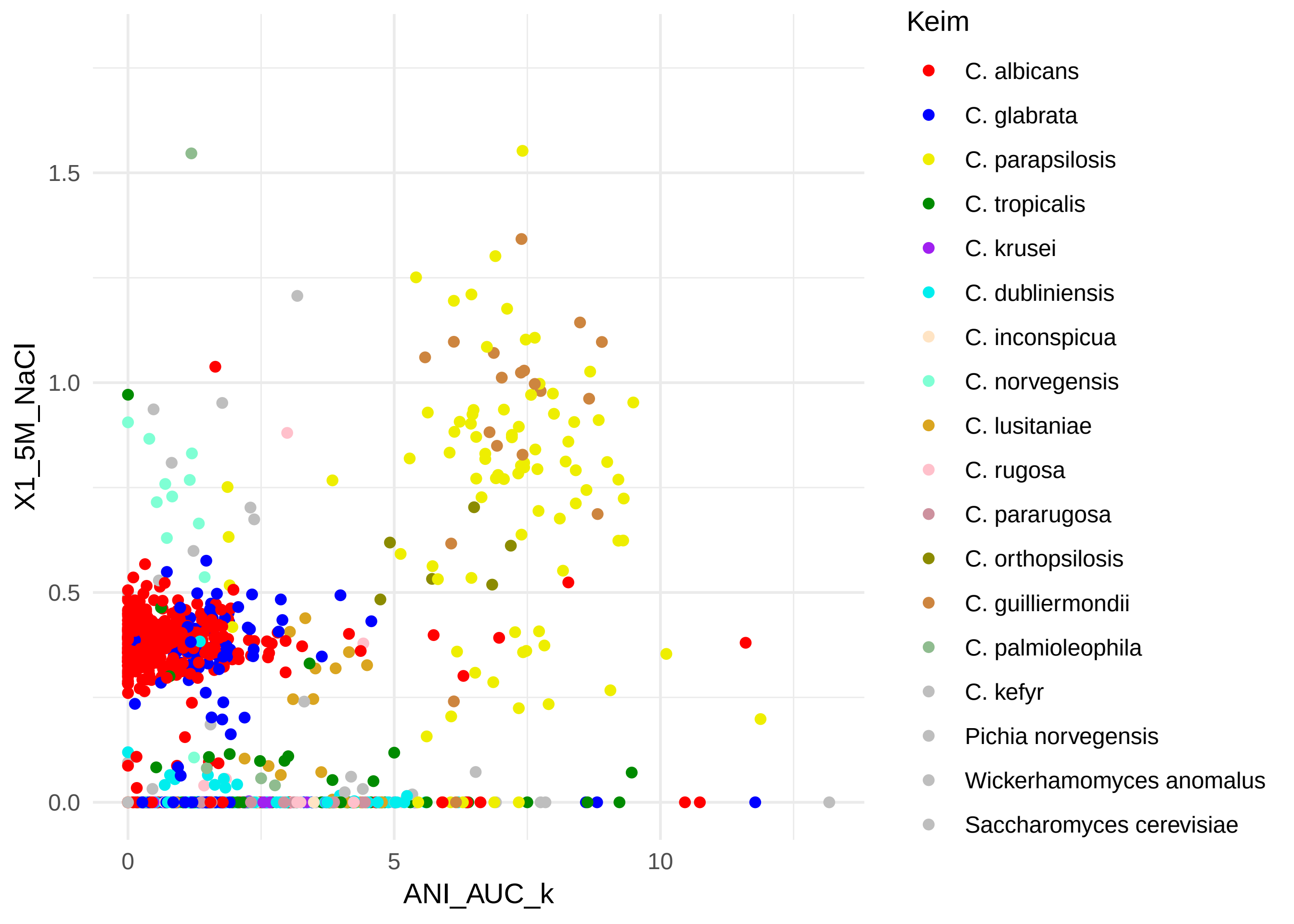

Keim
C. albicans
C. glabrata
C. parapsilosis
C. tropicalis
C. krusei
C. dubliniensis
C. inconspicua
C. norvegensis
C. lusitaniae
C. rugosa
C. pararugosa
C. orthopsilosis
C. guilliermondii
C. palmioleophila
C. kefyr
Pichia norvegensis
Wickerhamomyces anomalus
Saccharomyces cerevisiae
X1_5M_NaCl
1.5
1.0
0.5
0.0
0
5
10
ANI_AUC_k

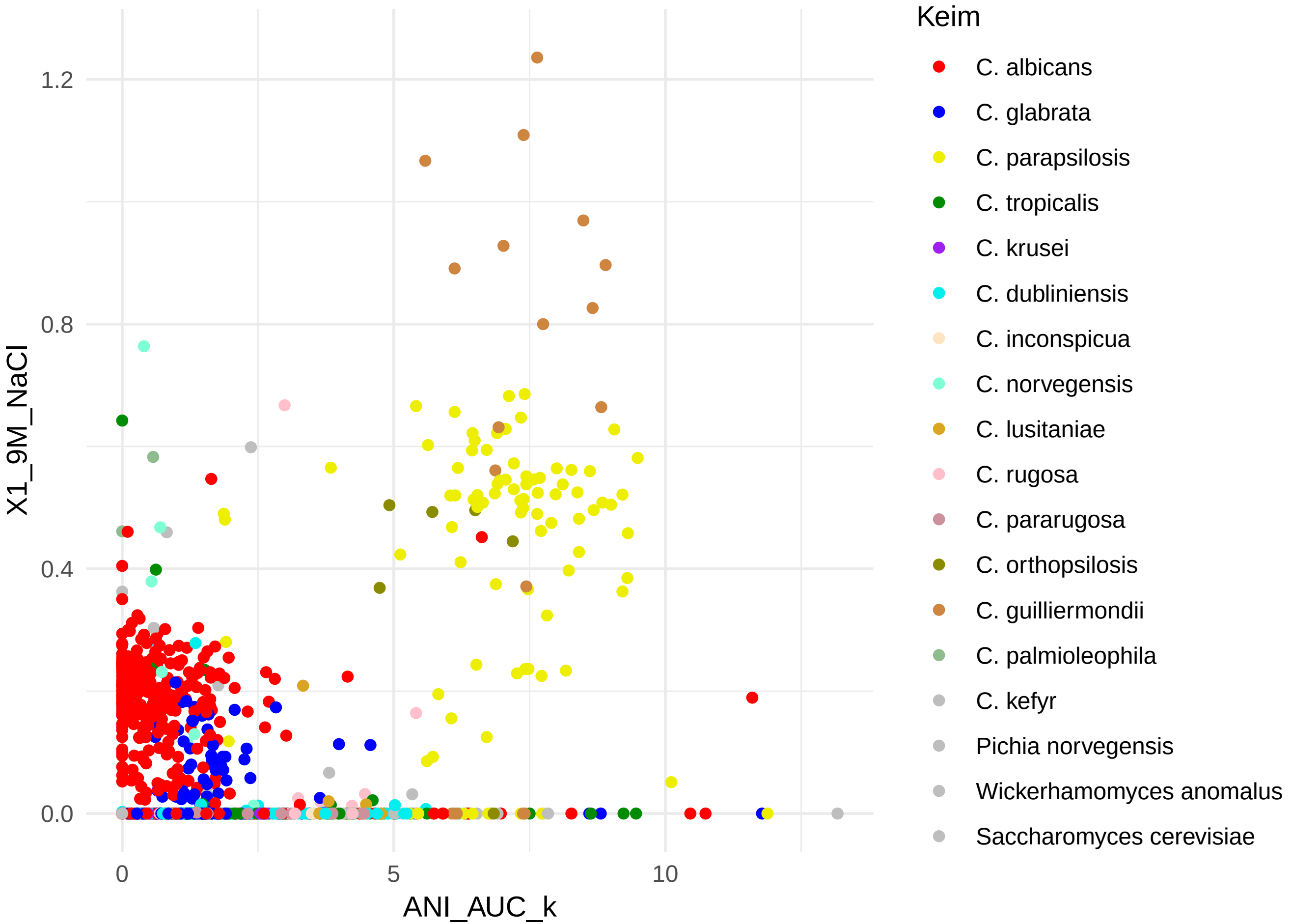
Keim
C. albicans
C. glabrata
C. parapsilosis
C. tropicalis
C. krusei
C. dubliniensis
C. inconspicua
C. norvegensis
C. lusitaniae
C. rugosa
C. pararugosa
C. orthopsilosis
C. guilliermondii
C. palmioleophila
C. kefyr
Pichia norvegensis
Wickerhamomyces anomalus
Saccharomyces cerevisiae
X1_9M_NaCl
1.2
0.8
0.4
0.0
0
5
10
ANI_AUC_k

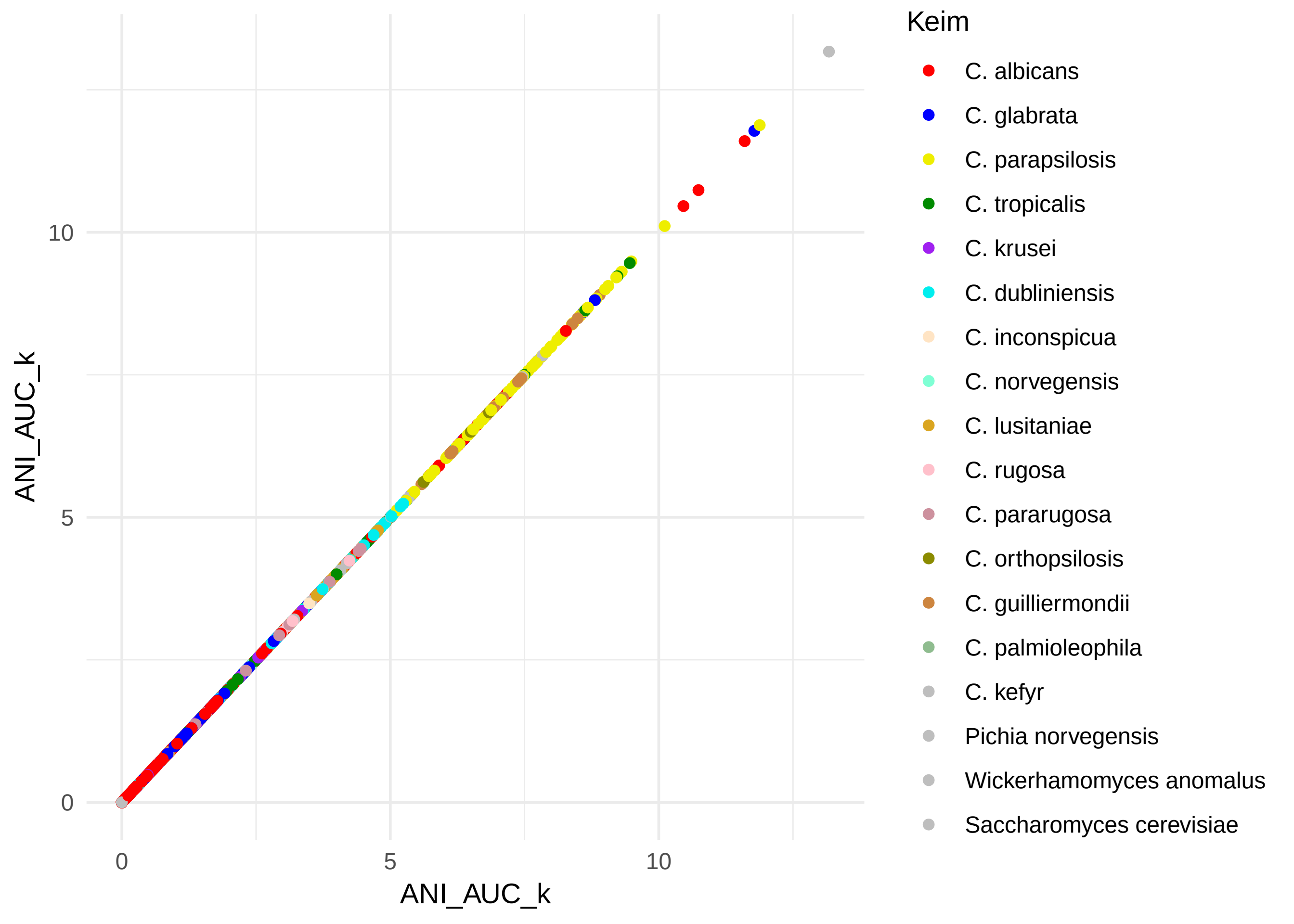
Keim
C. albicans
C. glabrata
C. parapsilosis
C. tropicalis
C. krusei
C. dubliniensis
C. inconspicua
C. norvegensis
C. lusitaniae
C. rugosa
C. pararugosa
C. orthopsilosis
C. guilliermondii
C. palmioleophila
C. kefyr
Pichia norvegensis
Wickerhamomyces anomalus
Saccharomyces cerevisiae
ANI_AUC_k
ANI_AUC_k
0
5
10
0
5
10

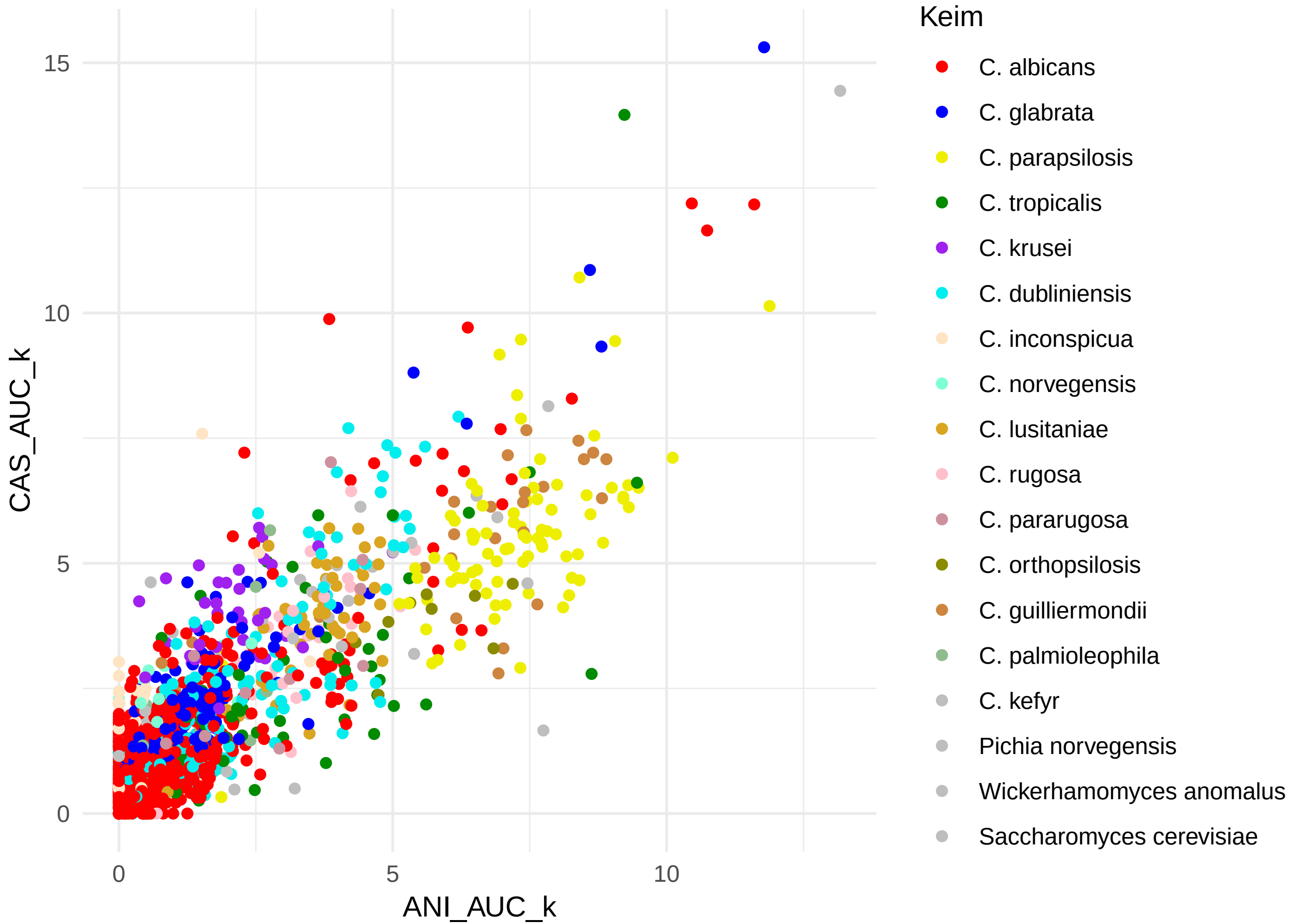

CAS_AUC_k
ANI_AUC_k
0
5
10
15
Keim
C. albicans
C. glabrata
C. parapsilosis
C. tropicalis
C. krusei
C. dubliniensis
C. inconspicua
C. norvegensis
C. lusitaniae
C. rugosa
C. pararugosa
C. orthopsilosis
C. guilliermondii
C. palmioleophila
C. kefyr
Pichia norvegensis
Wickerhamomyces anomalus
Saccharomyces cerevisiae

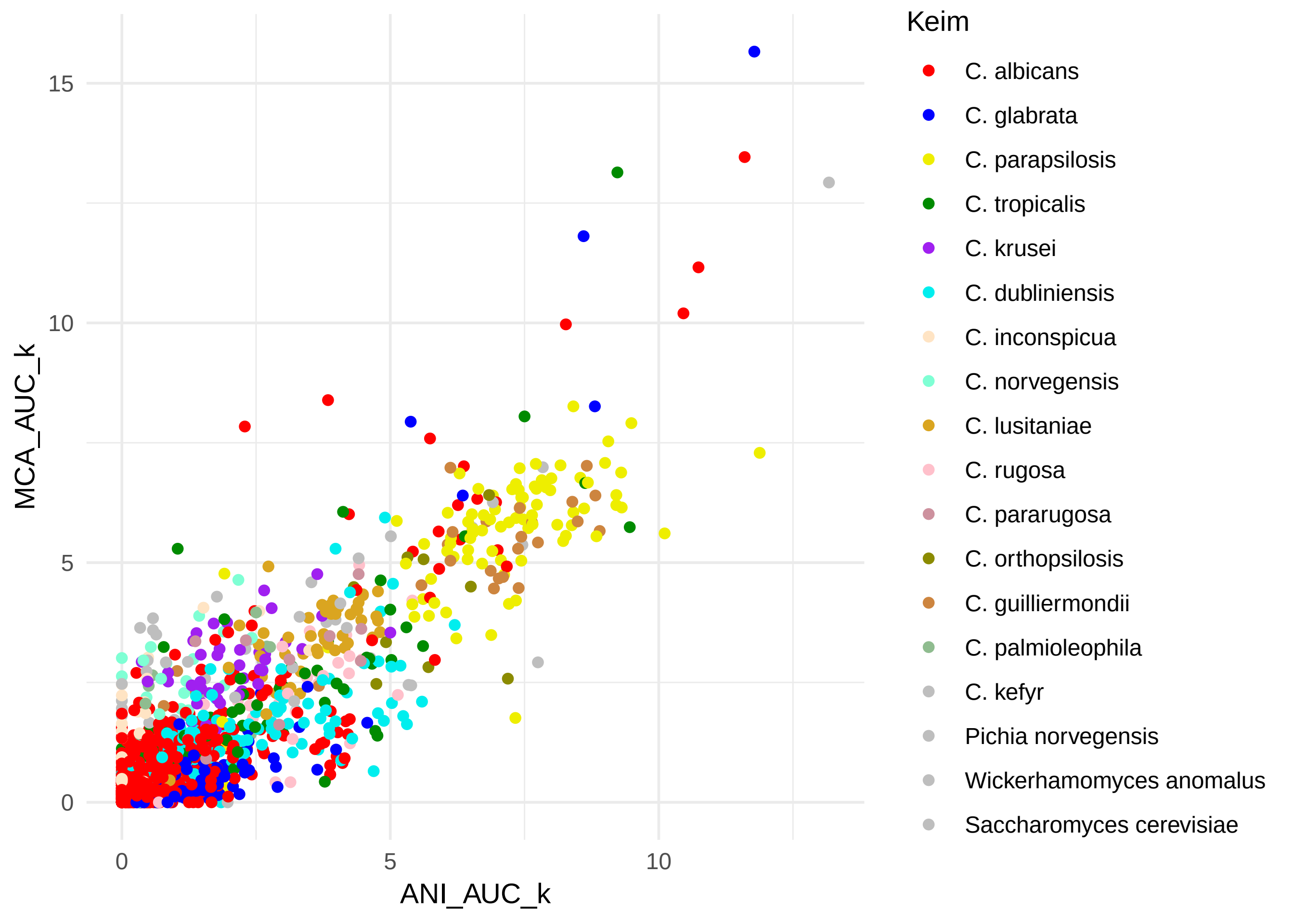
Keim
C. albicans
C. glabrata
C. parapsilosis
C. tropicalis
C. krusei
C. dubliniensis
C. inconspicua
C. norvegensis
C. lusitaniae
C. rugosa
C. pararugosa
C. orthopsilosis
C. guilliermondii
C. palmioleophila
C. kefyr
Pichia norvegensis
Wickerhamomyces anomalus
Saccharomyces cerevisiae
MCA_AUC_k
ANI_AUC_k
0
5
10
15
0
5
10

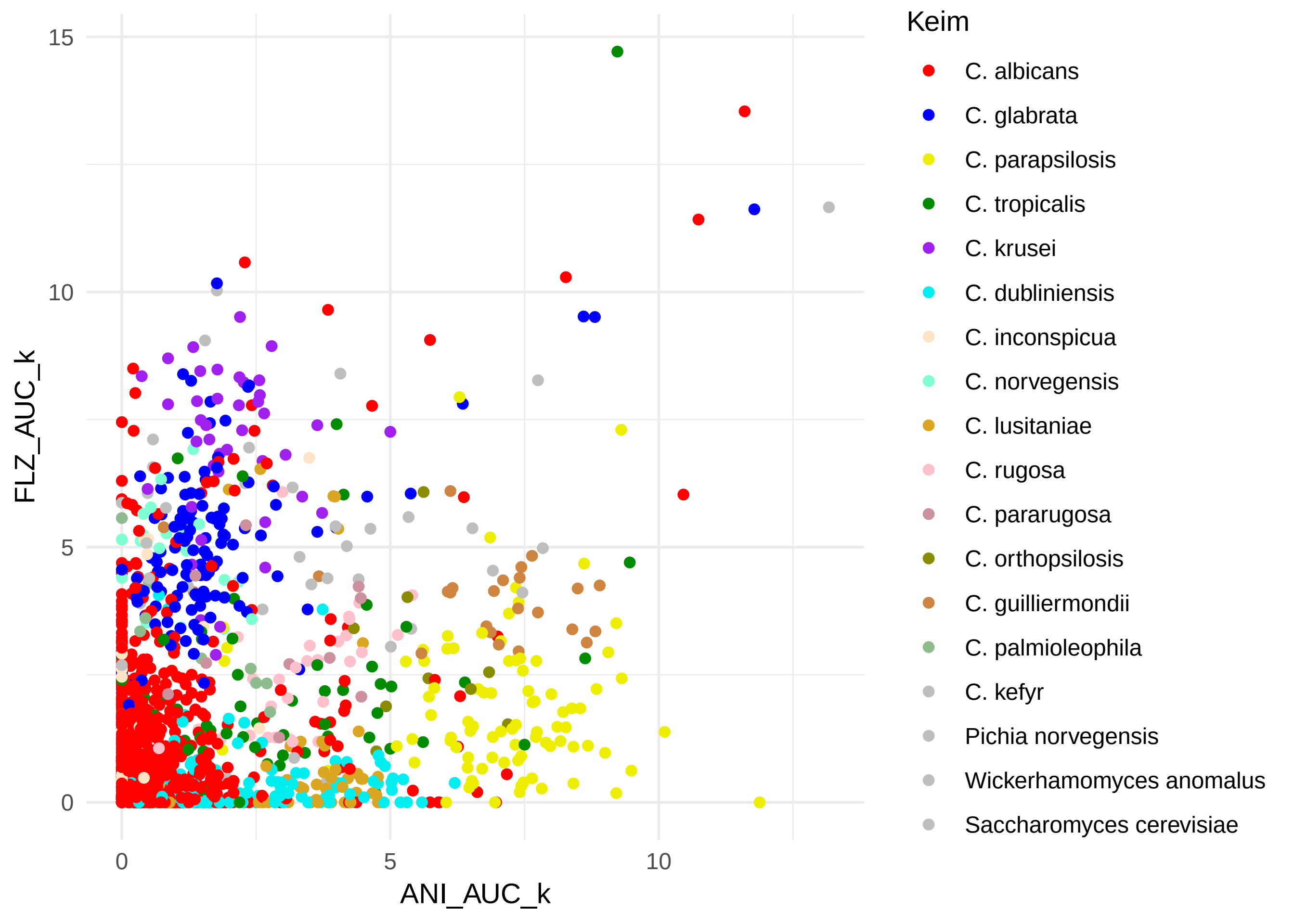
FLZ_AUC_k
ANI_AUC_k
0
5
10
15
Keim
C. albicans
C. glabrata
C. parapsilosis
C. tropicalis
C. krusei
C. dubliniensis
C. inconspicua
C. norvegensis
C. lusitaniae
C. rugosa
C. pararugosa
C. orthopsilosis
C. guilliermondii
C. palmioleophila
C. kefyr
Pichia norvegensis
Wickerhamomyces anomalus
Saccharomyces cerevisiae

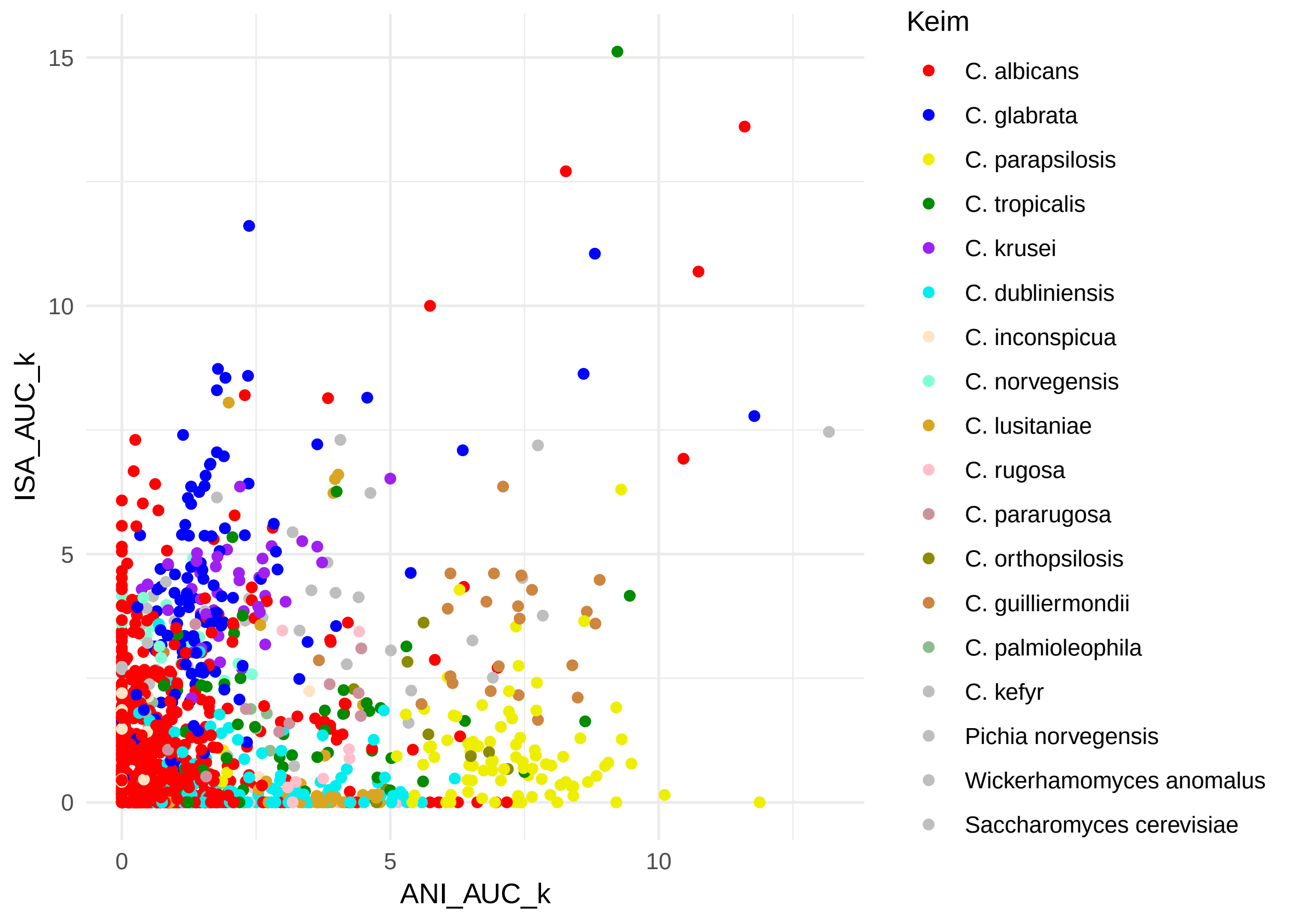
Keim
C. albicans
C. glabrata
C. parapsilosis
C. tropicalis
C. krusei
C. dubliniensis
C. inconspicua
C. norvegensis
C. lusitaniae
C. rugosa
C. pararugosa
C. orthopsilosis
C. guilliermondii
C. palmioleophila
C. kefyr
Pichia norvegensis
Wickerhamomyces anomalus
Saccharomyces cerevisiae
ISA_AUC_k
ANI_AUC_k
0
5
10
15
0
5
10

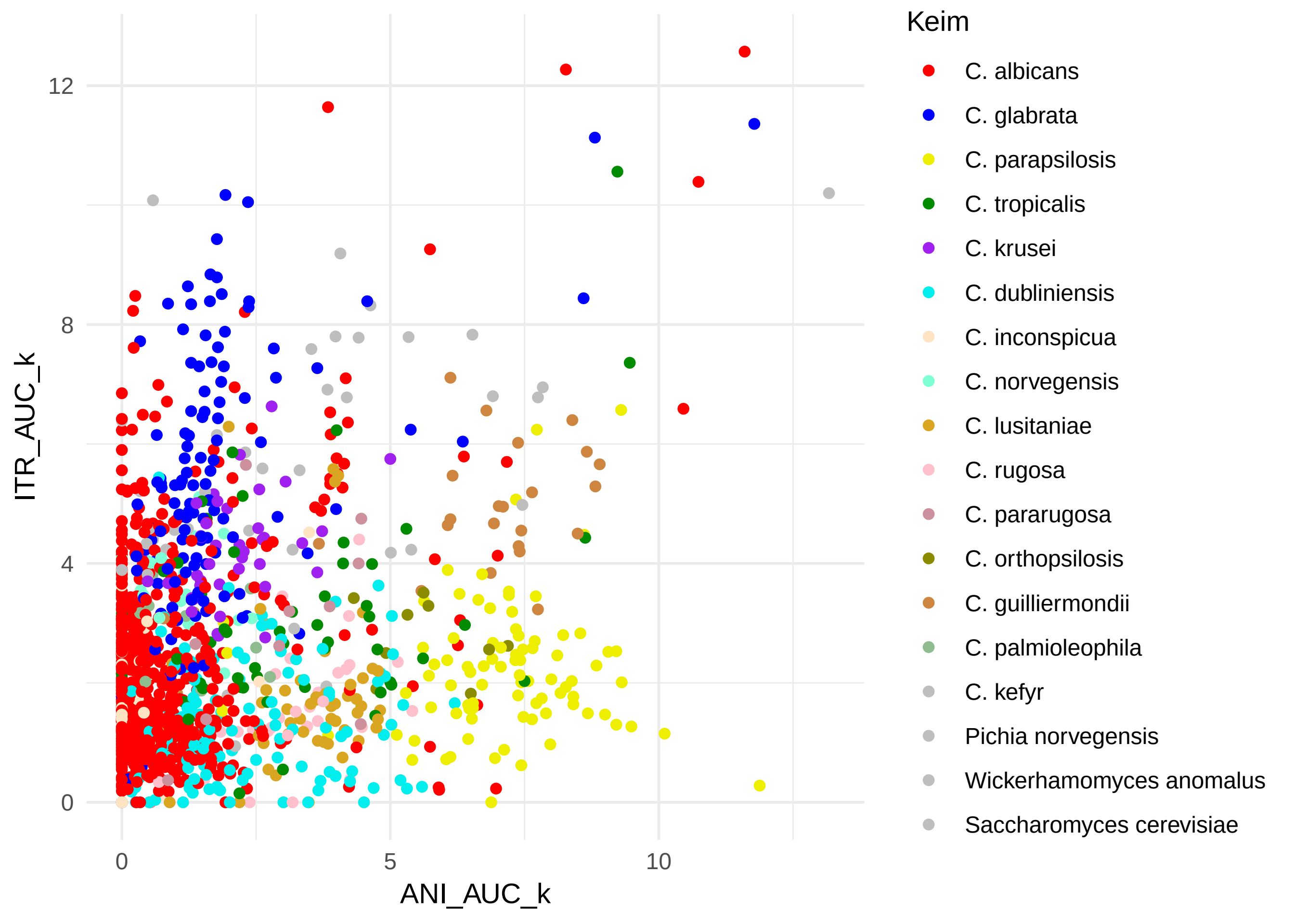

Keim
C. albicans
C. glabrata
C. parapsilosis
C. tropicalis
C. krusei
C. dubliniensis
C. inconspicua
C. norvegensis
C. lusitaniae
C. rugosa
C. pararugosa
C. orthopsilosis
C. guilliermondii
C. palmioleophila
C. kefyr
Pichia norvegensis
Wickerhamomyces anomalus
Saccharomyces cerevisiae
ITR_AUC_k
ANI_AUC_k
0
4
8
12
0
5
10

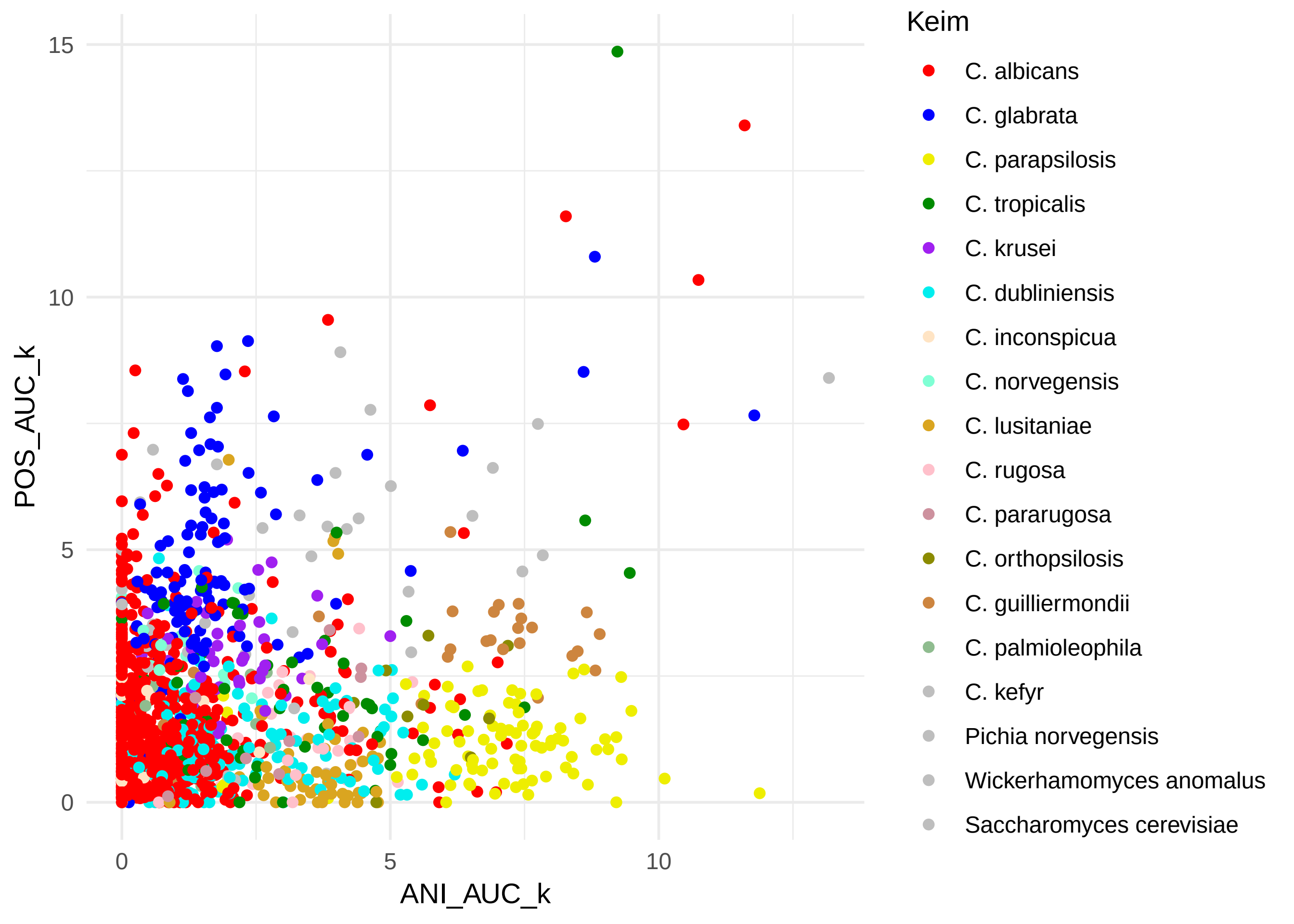

Keim
C. albicans
C. glabrata
C. parapsilosis
C. tropicalis
C. krusei
C. dubliniensis
C. inconspicua
C. norvegensis
C. lusitaniae
C. rugosa
C. pararugosa
C. orthopsilosis
C. guilliermondii
C. palmioleophila
C. kefyr
Pichia norvegensis
Wickerhamomyces anomalus
Saccharomyces cerevisiae
POS_AUC_k
ANI_AUC_k
15
10
5
0
0
5
10

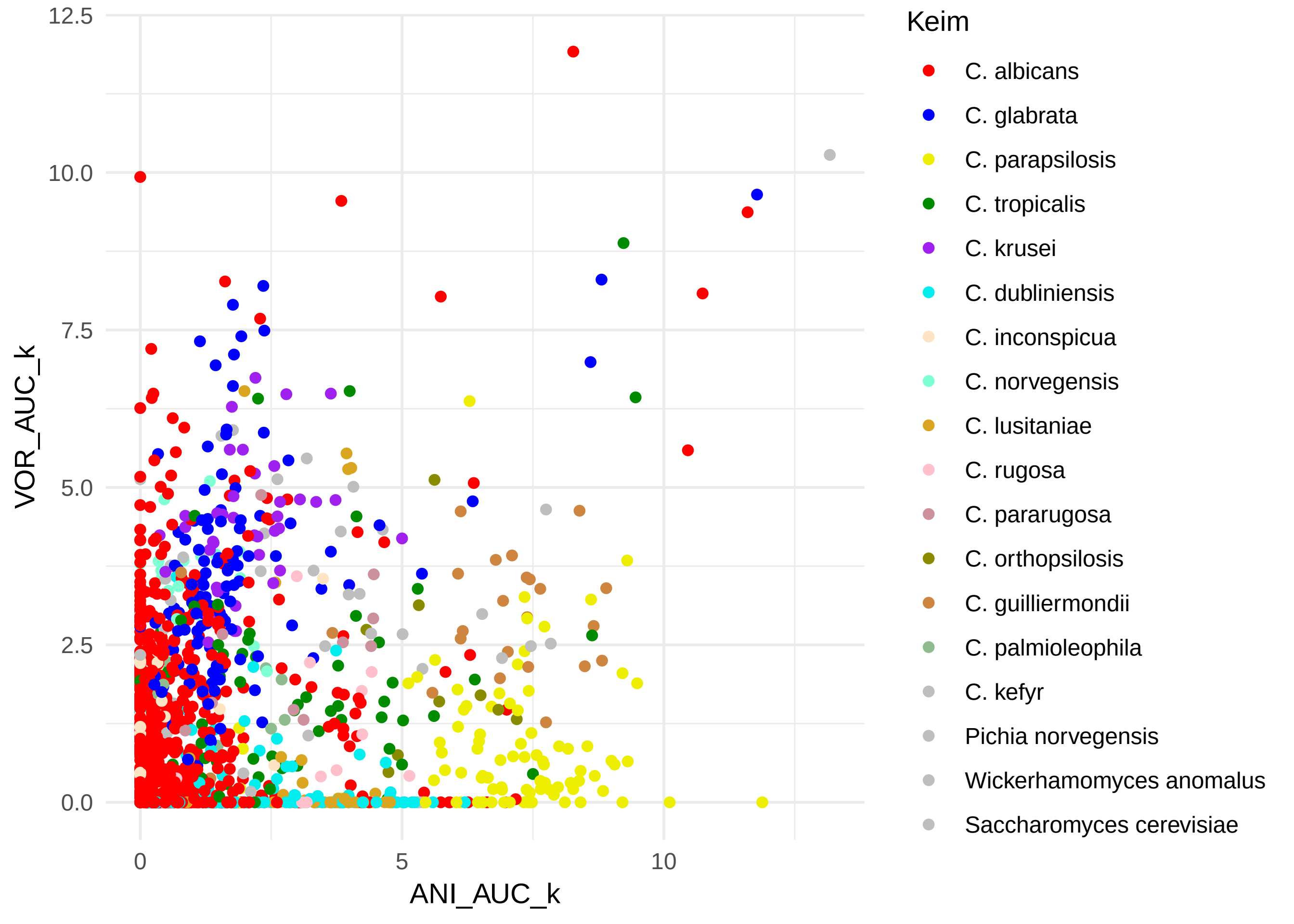

12.5
10.0
7.5
5.0
2.5
0.0
VOR_AUC_k
0
5
10
ANI_AUC_k
Keim
C. albicans
C. glabrata
C. parapsilosis
C. tropicalis
C. krusei
C. dubliniensis
C. inconspicua
C. norvegensis
C. lusitaniae
C. rugosa
C. pararugosa
C. orthopsilosis
C. guilliermondii
C. palmioleophila
C. kefyr
Pichia norvegensis
Wickerhamomyces anomalus
Saccharomyces cerevisiae

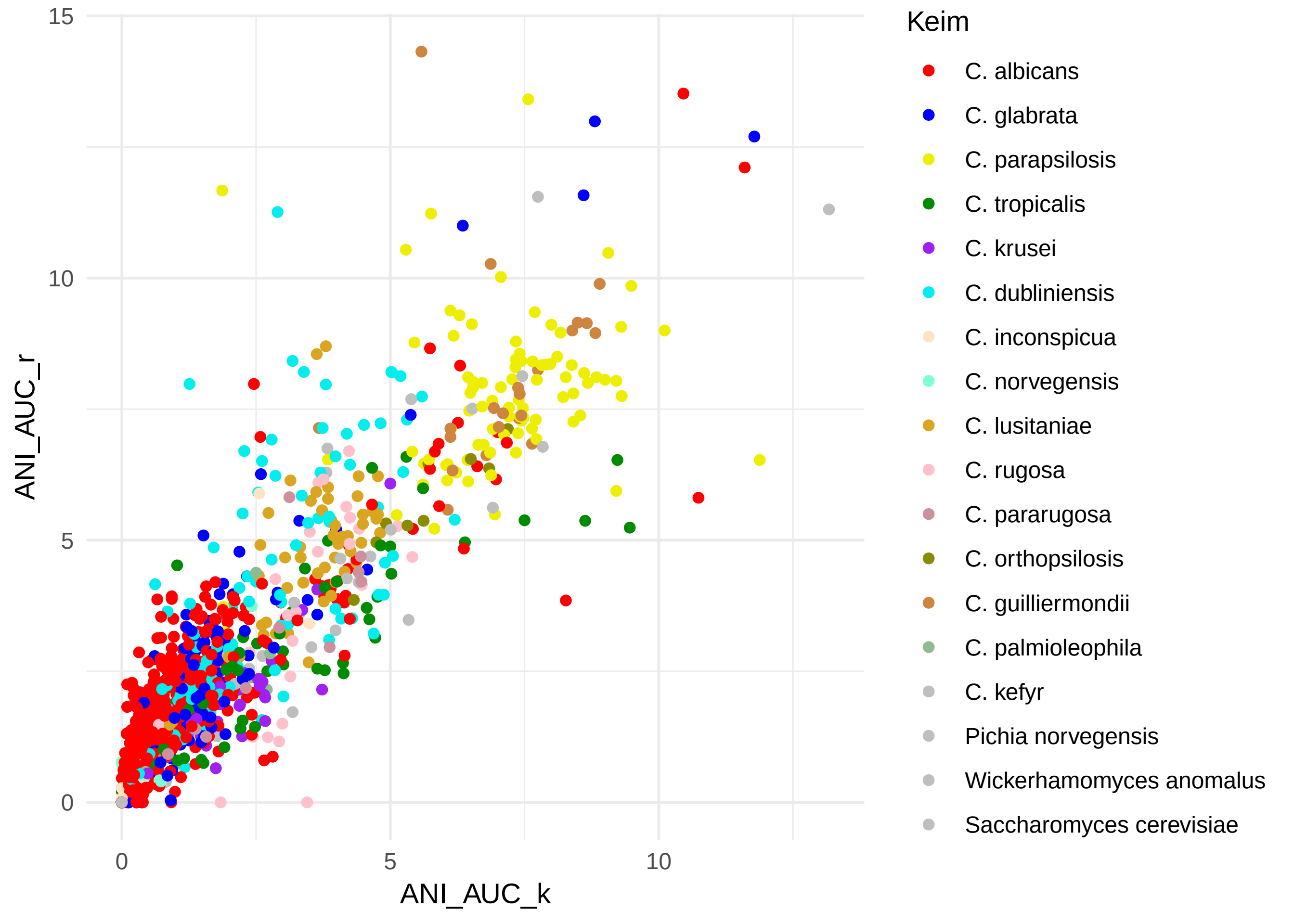
Keim
C. albicans
C. glabrata
C. parapsilosis
C. tropicalis
C. krusei
C. dubliniensis
C. inconspicua
C. norvegensis
C. lusitaniae
C. rugosa
C. pararugosa
C. orthopsilosis
C. guilliermondii
C. palmioleophila
C. kefyr
Pichia norvegensis
Wickerhamomyces anomalus
Saccharomyces cerevisiae
ANI_AUC_r
ANI_AUC_k
0
5
10
15

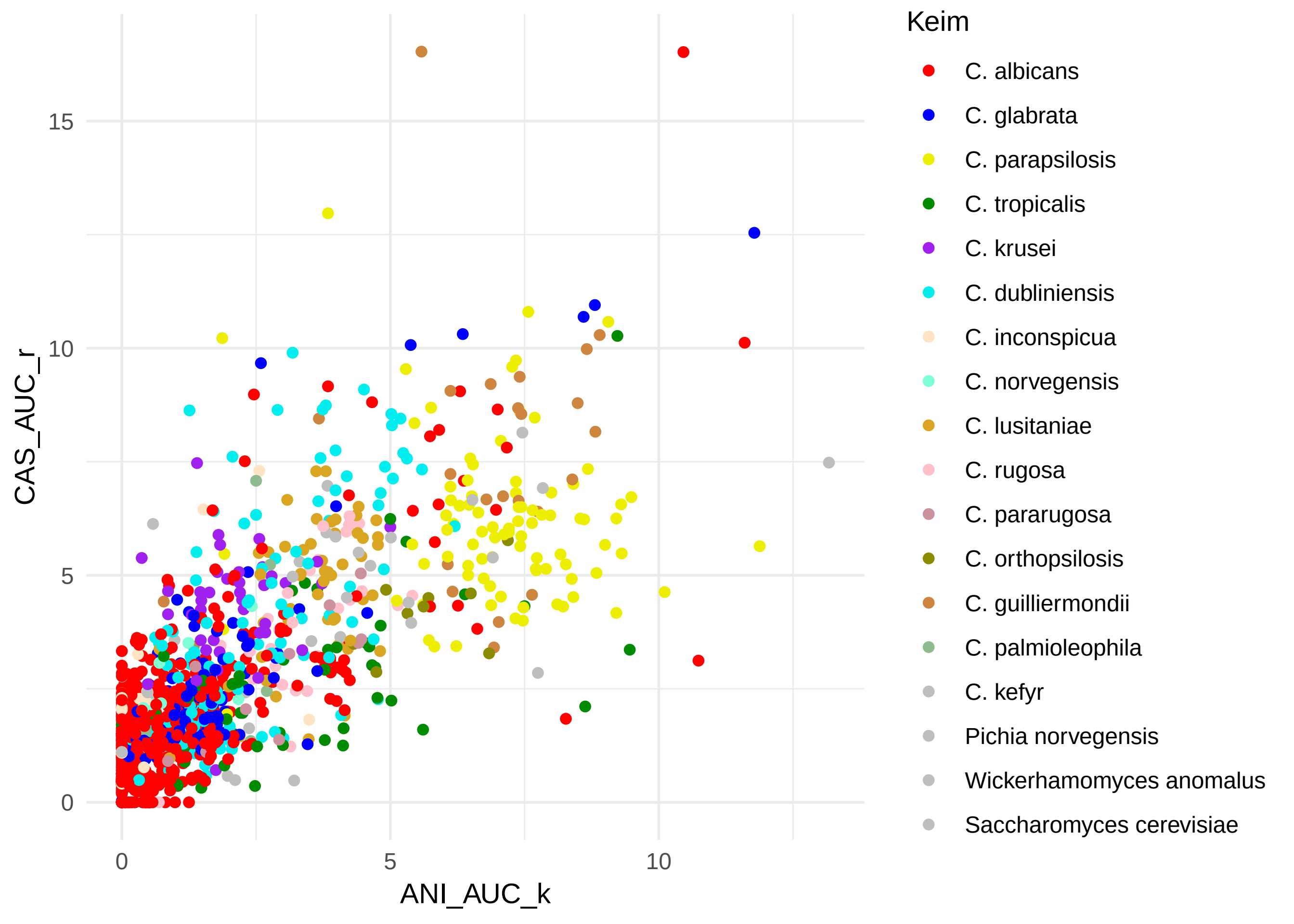

CAS_AUC_r
0
5
10
15
ANI_AUC_k
0
5
10
Keim
C. albicans
C. glabrata
C. parapsilosis
C. tropicalis
C. krusei
C. dubliniensis
C. inconspicua
C. norvegensis
C. lusitaniae
C. rugosa
C. pararugosa
C. orthopsilosis
C. guilliermondii
C. palmioleophila
C. kefyr
Pichia norvegensis
Wickerhamomyces anomalus
Saccharomyces cerevisiae

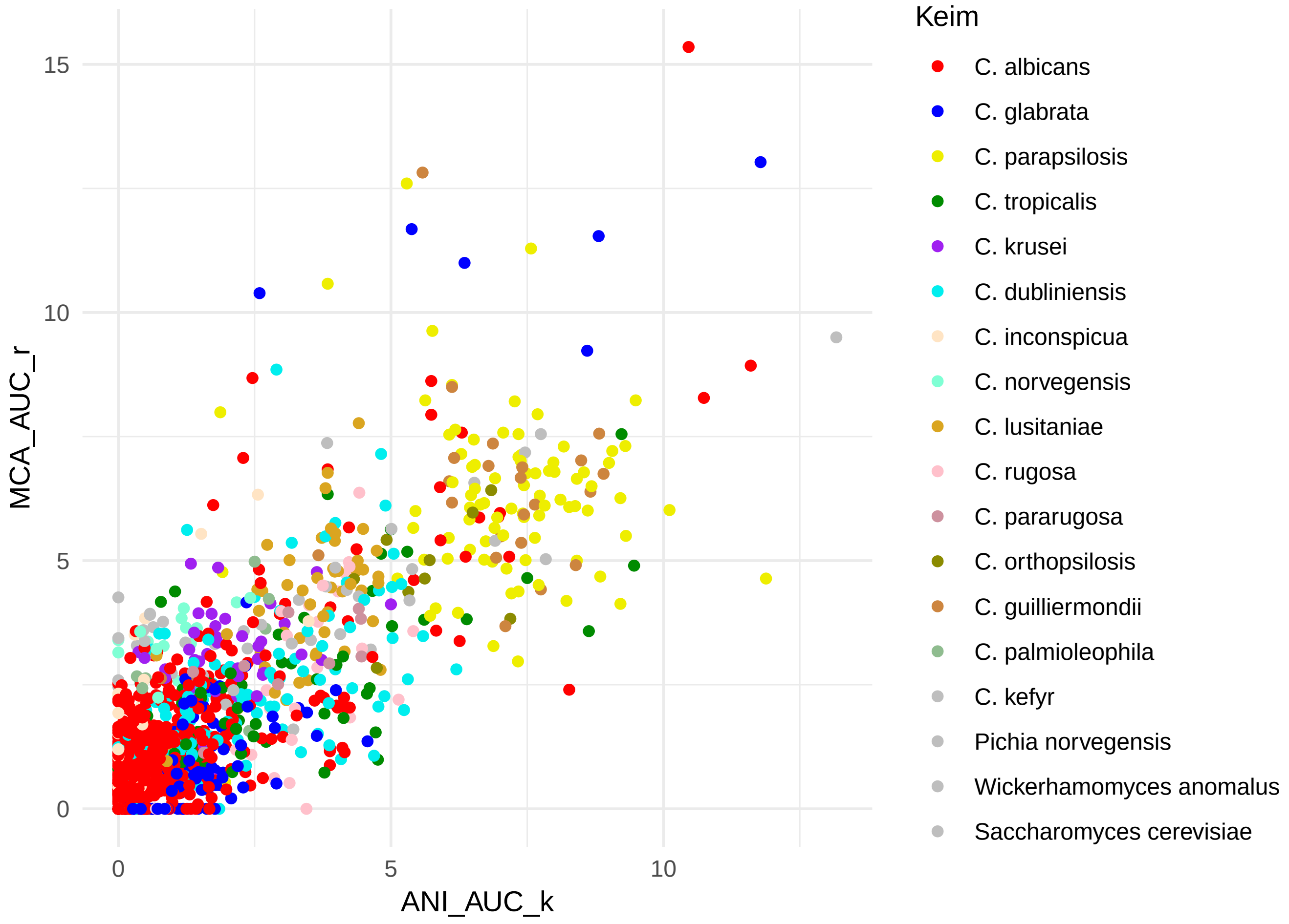

MCA_AUC_r
ANI_AUC_k
0
5
10
15
0
5
10
Keim
C. albicans
C. glabrata
C. parapsilosis
C. tropicalis
C. krusei
C. dubliniensis
C. inconspicua
C. norvegensis
C. lusitaniae
C. rugosa
C. pararugosa
C. orthopsilosis
C. guilliermondii
C. palmioleophila
C. kefyr
Pichia norvegensis
Wickerhamomyces anomalus
Saccharomyces cerevisiae

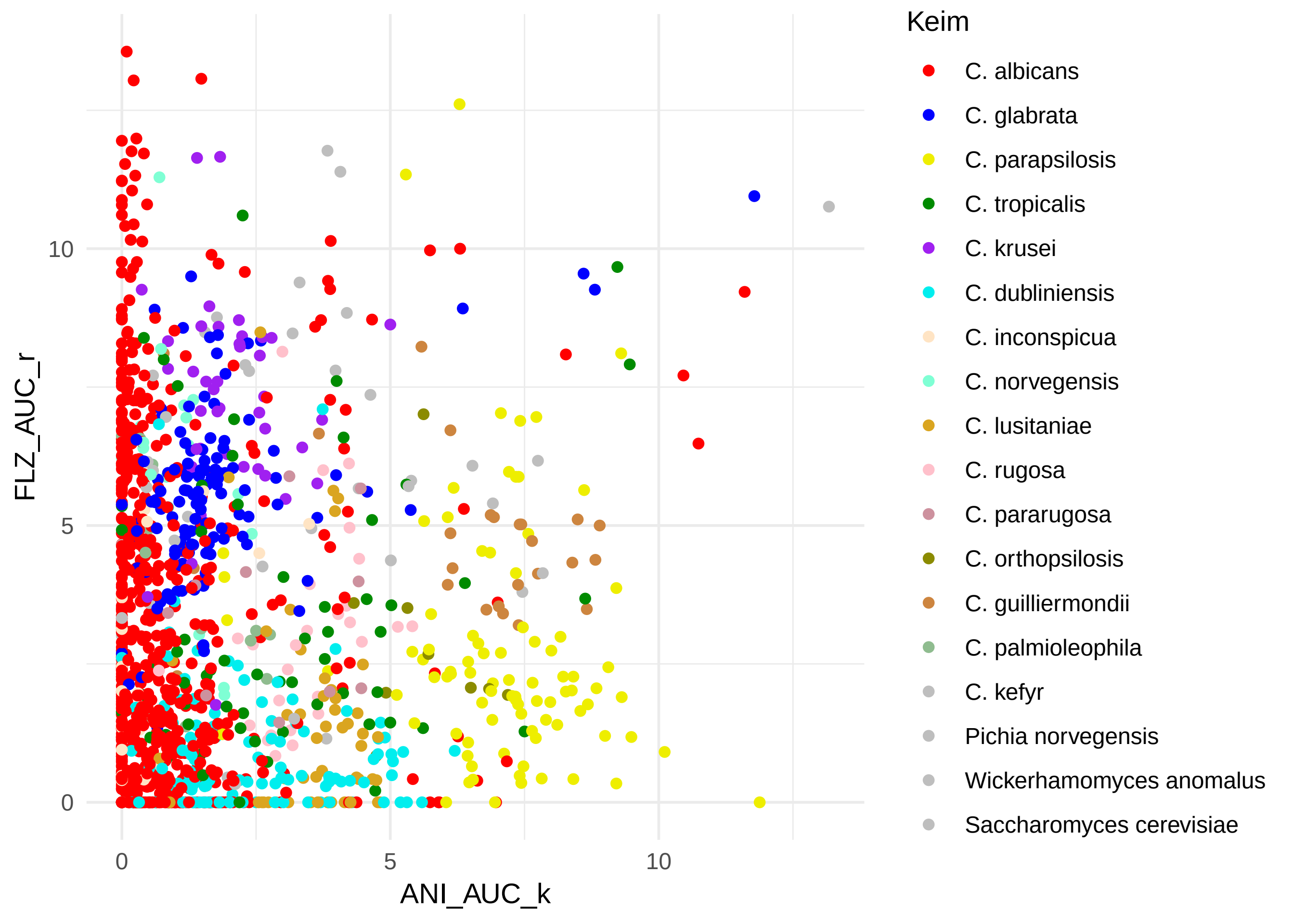
Keim
C. albicans
C. glabrata
C. parapsilosis
C. tropicalis
C. krusei
C. dubliniensis
C. inconspicua
C. norvegensis
C. lusitaniae
C. rugosa
C. pararugosa
C. orthopsilosis
C. guilliermondii
C. palmioleophila
C. kefyr
Pichia norvegensis
Wickerhamomyces anomalus
Saccharomyces cerevisiae
FLZ_AUC_r
10
5
0
0
5
10
ANI_AUC_k

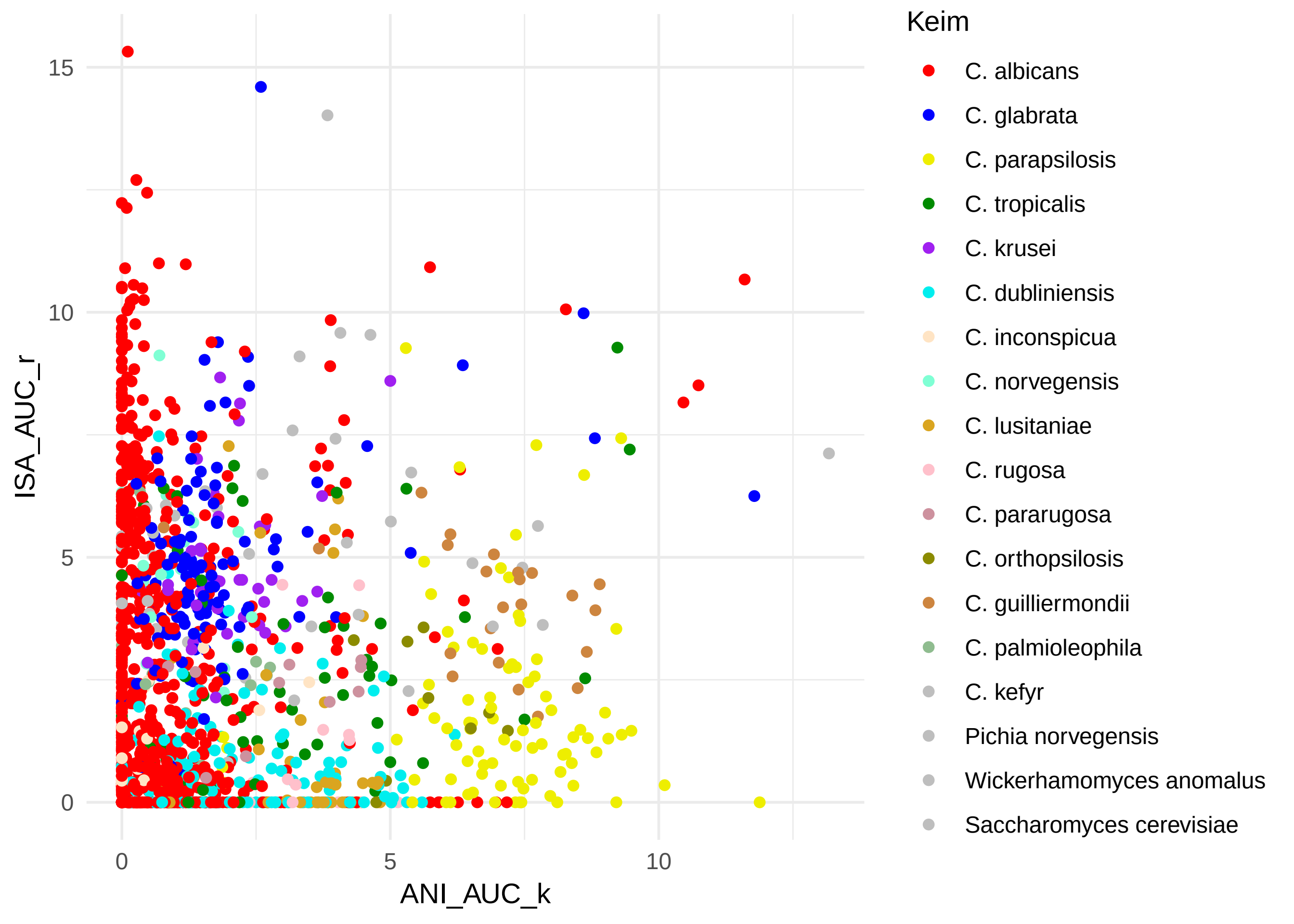
ISA_AUC_r
ANI_AUC_k
0
5
10
15
0
5
10
Keim
C. albicans
C. glabrata
C. parapsilosis
C. tropicalis
C. krusei
C. dubliniensis
C. inconspicua
C. norvegensis
C. lusitaniae
C. rugosa
C. pararugosa
C. orthopsilosis
C. guilliermondii
C. palmioleophila
C. kefyr
Pichia norvegensis
Wickerhamomyces anomalus
Saccharomyces cerevisiae

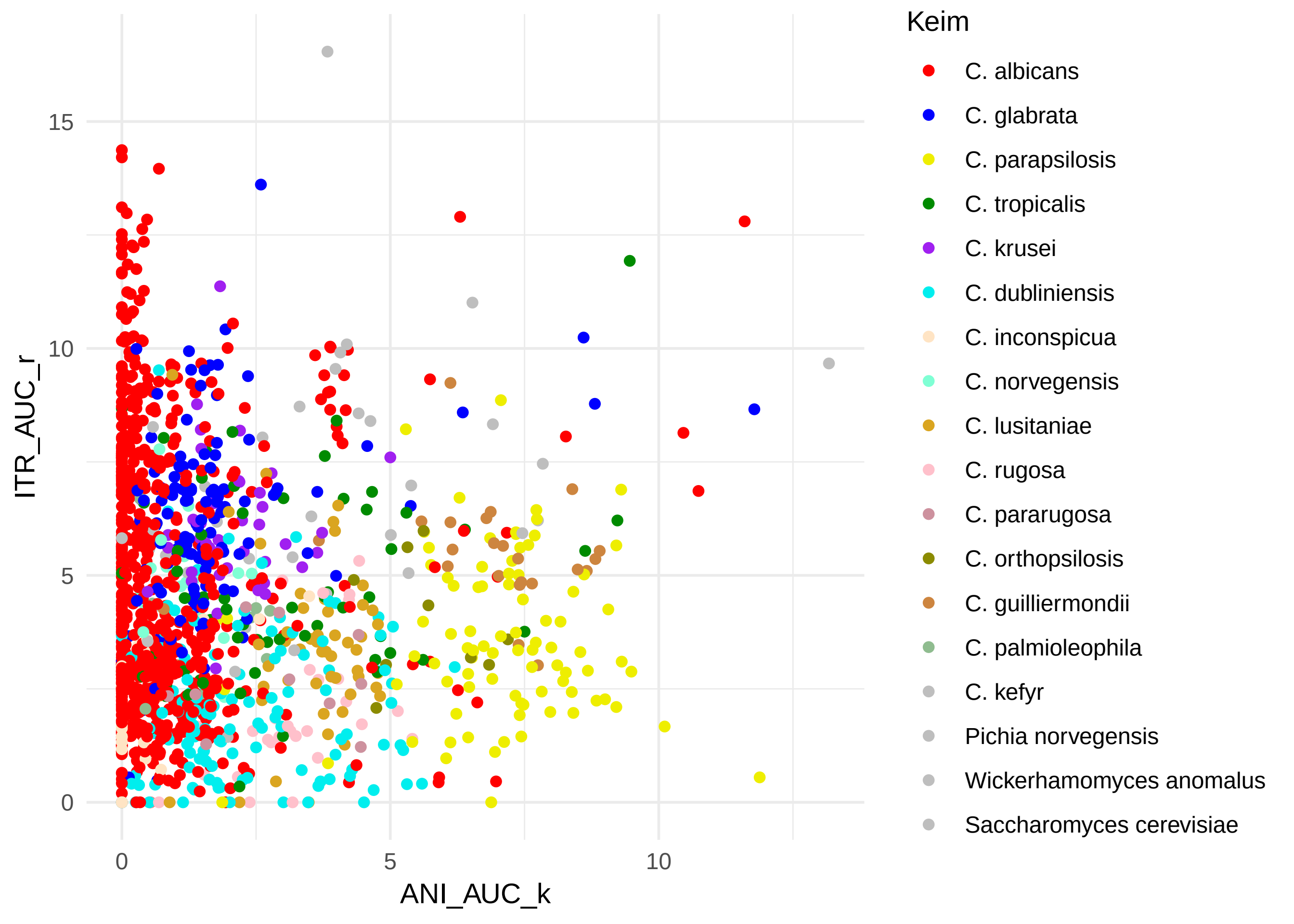
Keim
C. albicans
C. glabrata
C. parapsilosis
C. tropicalis
C. krusei
C. dubliniensis
C. inconspicua
C. norvegensis
C. lusitaniae
C. rugosa
C. pararugosa
C. orthopsilosis
C. guilliermondii
C. palmioleophila
C. kefyr
Pichia norvegensis
Wickerhamomyces anomalus
Saccharomyces cerevisiae
ITR_AUC_r
ANI_AUC_k
0
5
10
15

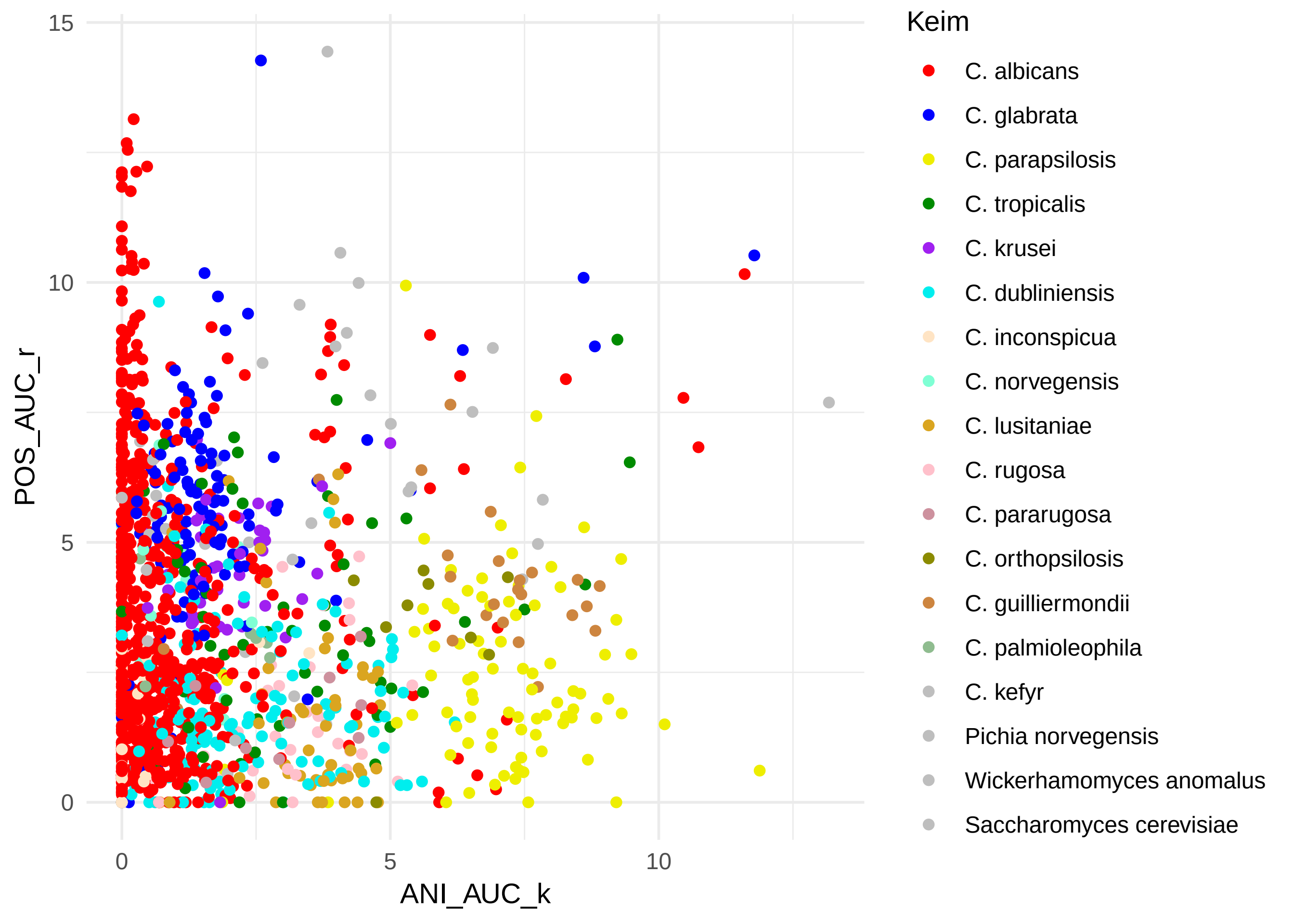

15
10
5
0
POS_AUC_r
0
5
10
ANI_AUC_k
Keim
C. albicans
C. glabrata
C. parapsilosis
C. tropicalis
C. krusei
C. dubliniensis
C. inconspicua
C. norvegensis
C. lusitaniae
C. rugosa
C. pararugosa
C. orthopsilosis
C. guilliermondii
C. palmioleophila
C. kefyr
Pichia norvegensis
Wickerhamomyces anomalus
Saccharomyces cerevisiae

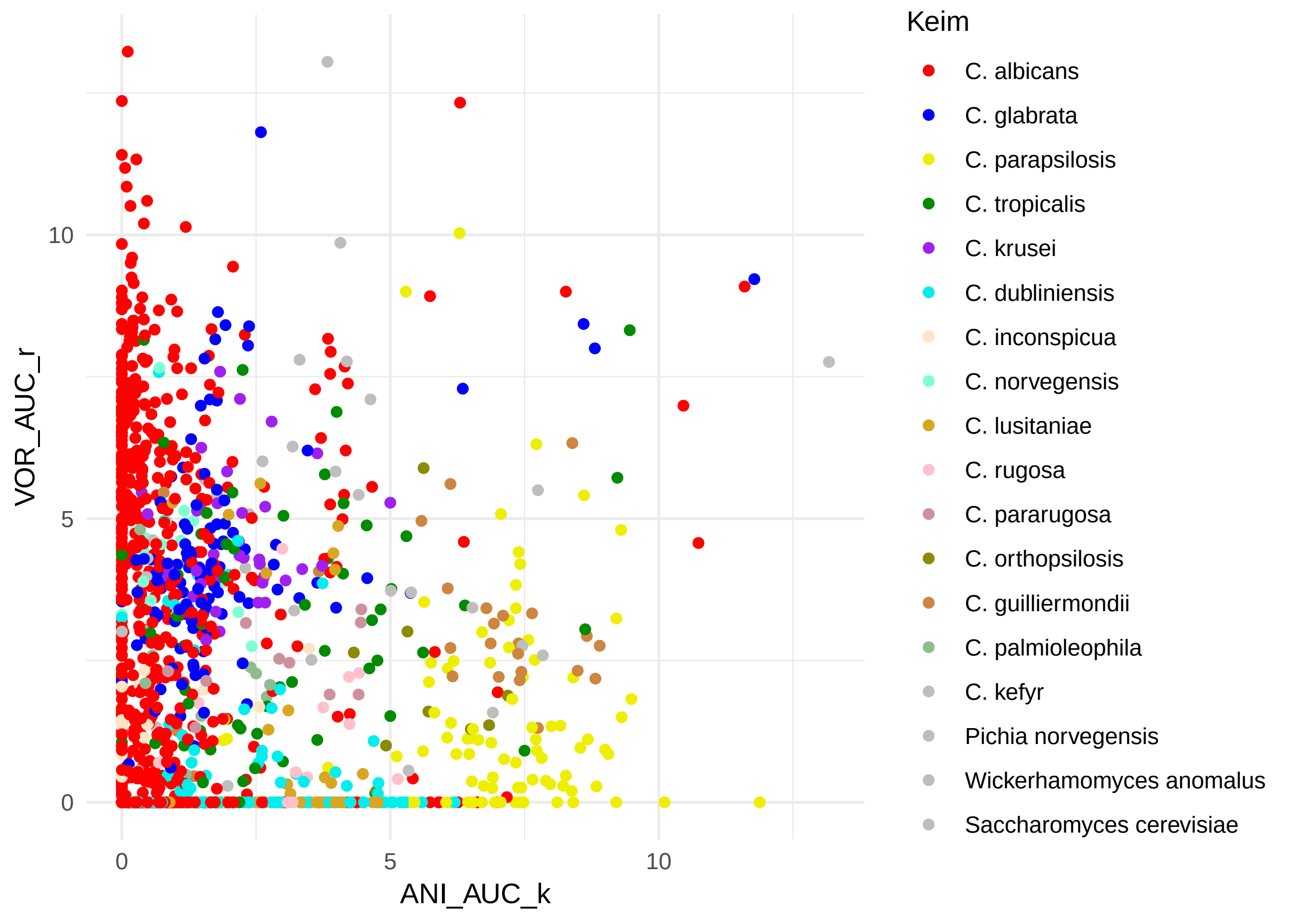

Keim
C. albicans
C. glabrata
C. parapsilosis
C. tropicalis
C. krusei
C. dubliniensis
C. inconspicua
C. norvegensis
C. lusitaniae
C. rugosa
C. pararugosa
C. orthopsilosis
C. guilliermondii
C. palmioleophila
C. kefyr
Pichia norvegensis
Wickerhamomyces anomalus
Saccharomyces cerevisiae
VOR_AUC_r
ANI_AUC_k
0
5
10
0
5
10

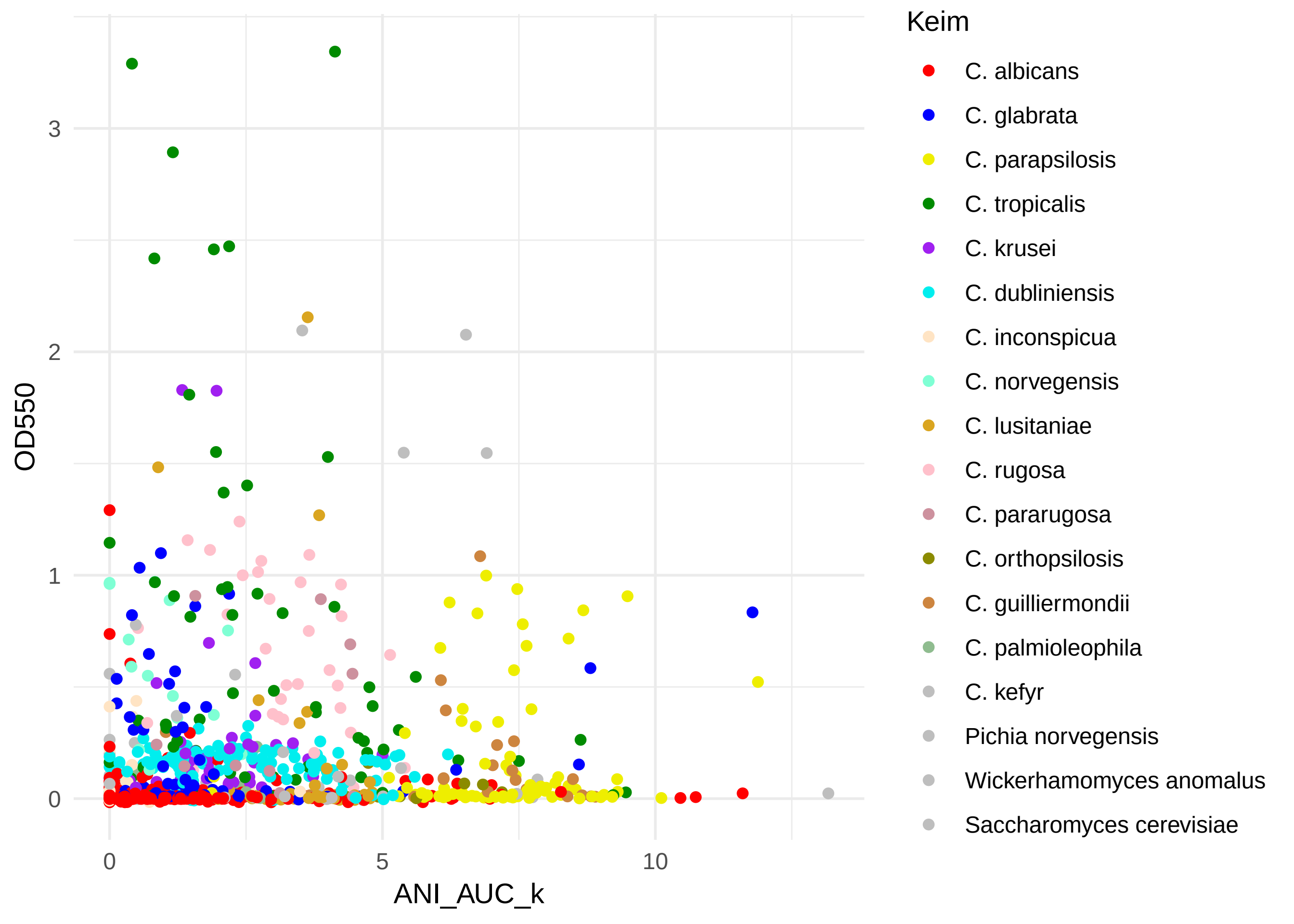

OD550
ANI_AUC_k
0
1
2
3
0
5
10
Keim
C. albicans
C. glabrata
C. parapsilosis
C. tropicalis
C. krusei
C. dubliniensis
C. inconspicua
C. norvegensis
C. lusitaniae
C. rugosa
C. pararugosa
C. orthopsilosis
C. guilliermondii
C. palmioleophila
C. kefyr
Pichia norvegensis
Wickerhamomyces anomalus
Saccharomyces cerevisiae

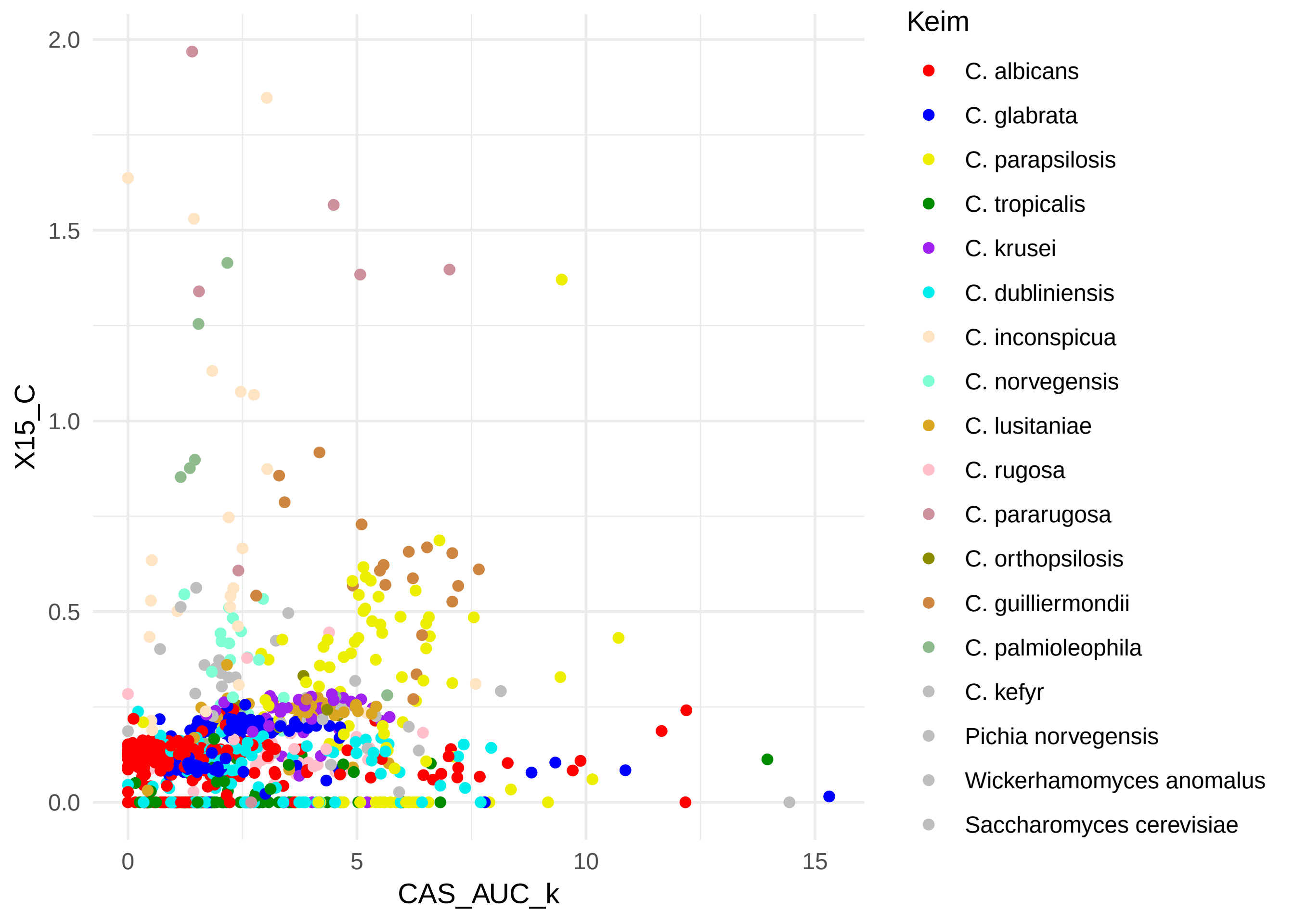
Keim
C. albicans
C. glabrata
C. parapsilosis
C. tropicalis
C. krusei
C. dubliniensis
C. inconspicua
C. norvegensis
C. lusitaniae
C. rugosa
C. pararugosa
C. orthopsilosis
C. guilliermondii
C. palmioleophila
C. kefyr
Pichia norvegensis
Wickerhamomyces anomalus
Saccharomyces cerevisiae
X15_C
2.0
1.5
1.0
0.5
0.0
0
5
10
15
CAS_AUC_k

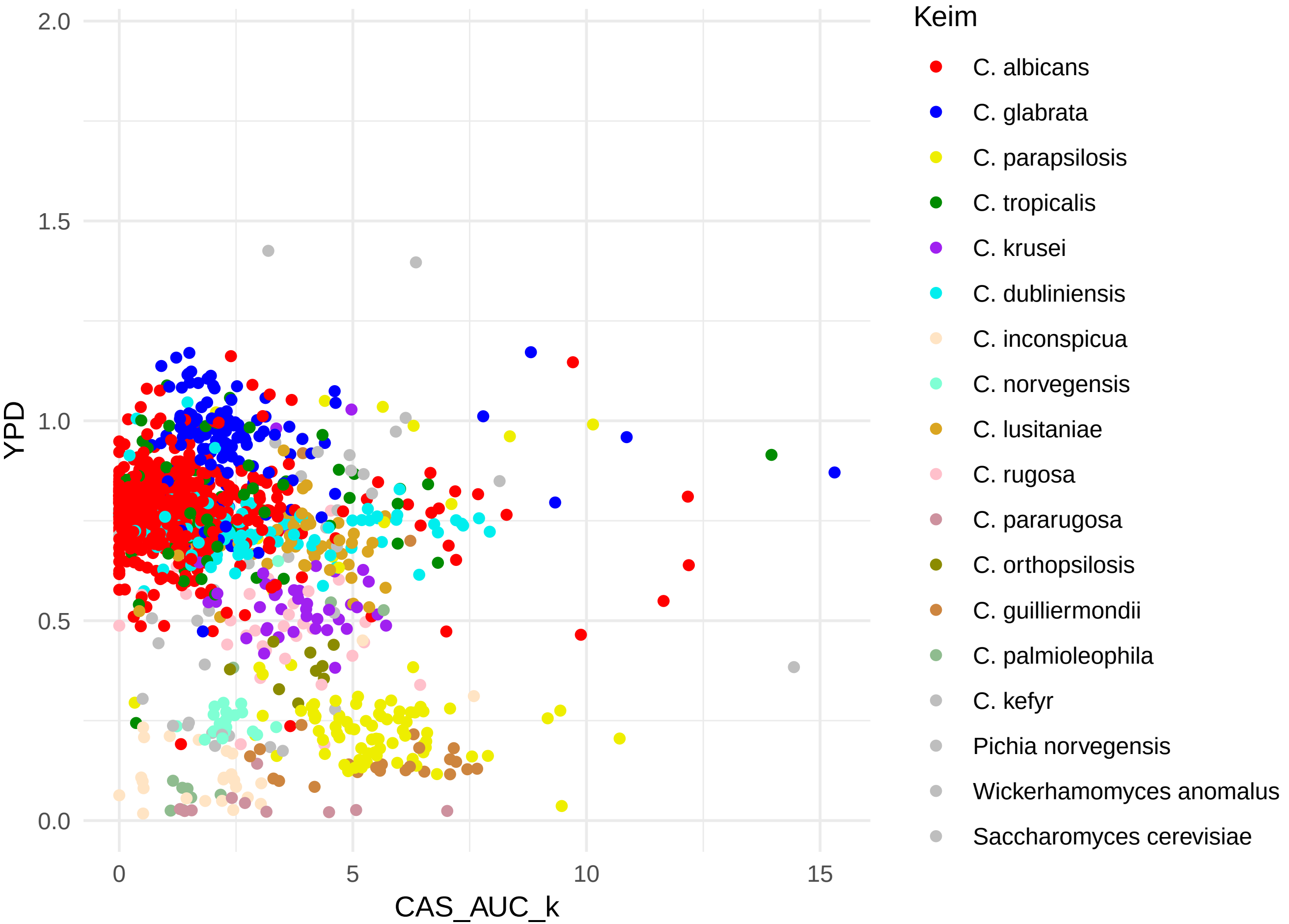
YPD
CAS_AUC_k
2.0
1.5
1.0
0.5
0.0
0
5
10
15
Keim
C. albicans
C. glabrata
C. parapsilosis
C. tropicalis
C. krusei
C. dubliniensis
C. inconspicua
C. norvegensis
C. lusitaniae
C. rugosa
C. pararugosa
C. orthopsilosis
C. guilliermondii
C. palmioleophila
C. kefyr
Pichia norvegensis
Wickerhamomyces anomalus
Saccharomyces cerevisiae

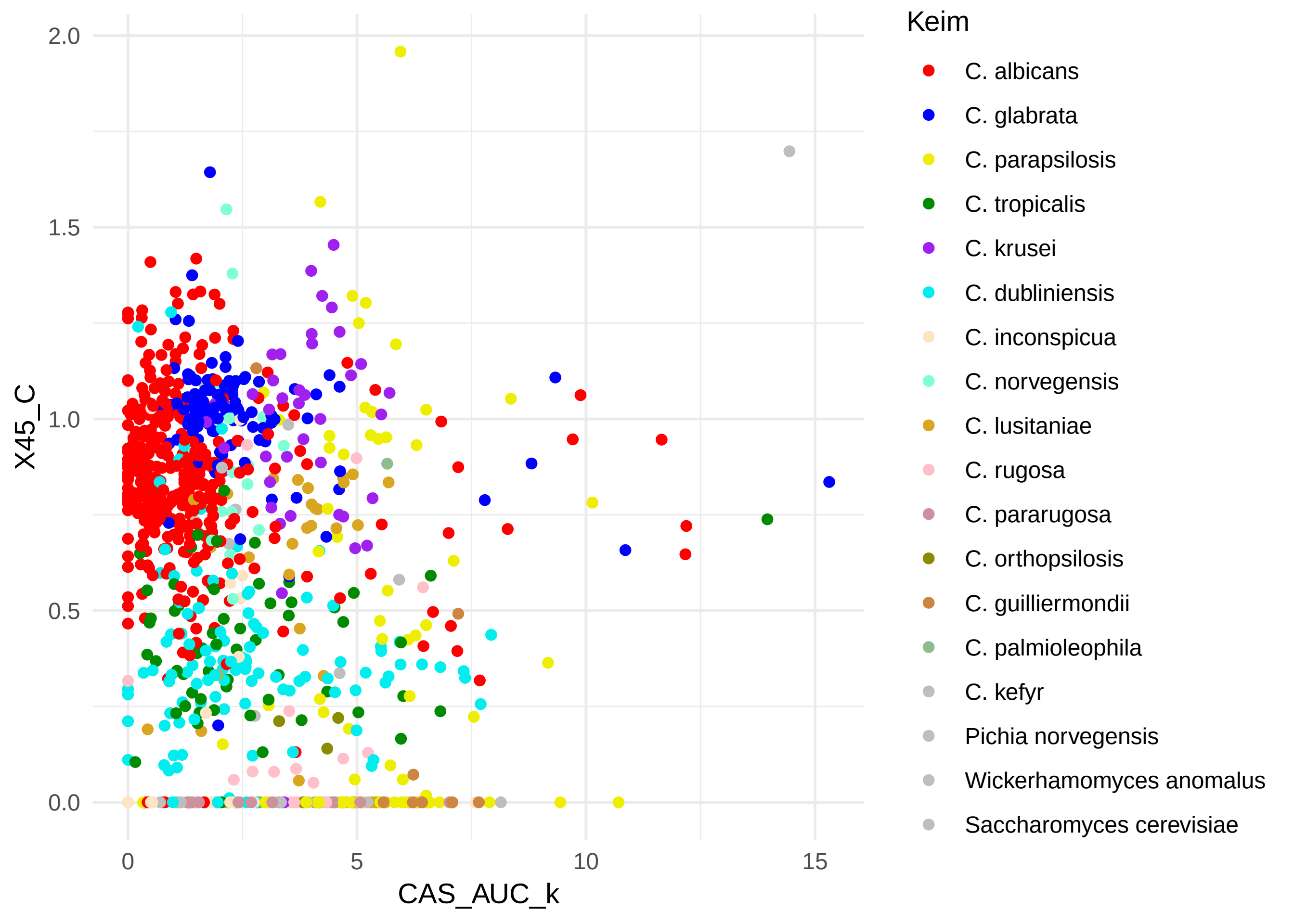
Keim
C. albicans
C. glabrata
C. parapsilosis
C. tropicalis
C. krusei
C. dubliniensis
C. inconspicua
C. norvegensis
C. lusitaniae
C. rugosa
C. pararugosa
C. orthopsilosis
C. guilliermondii
C. palmioleophila
C. kefyr
Pichia norvegensis
Wickerhamomyces anomalus
Saccharomyces cerevisiae
X45_C
2.0
1.5
1.0
0.5
0.0
0
5
10
15
CAS_AUC_k

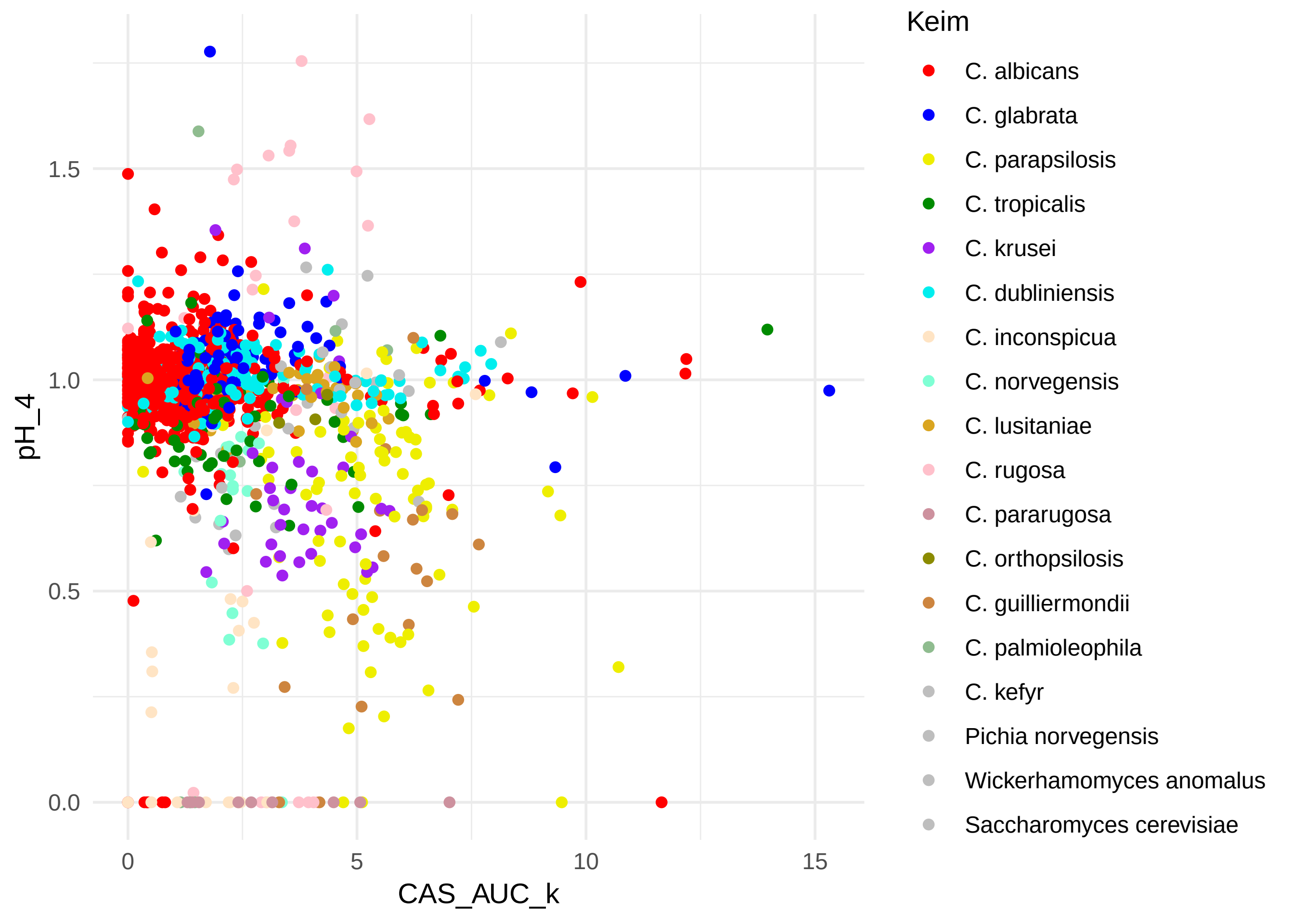

Keim
C. albicans
C. glabrata
C. parapsilosis
C. tropicalis
C. krusei
C. dubliniensis
C. inconspicua
C. norvegensis
C. lusitaniae
C. rugosa
C. pararugosa
C. orthopsilosis
C. guilliermondii
C. palmioleophila
C. kefyr
Pichia norvegensis
Wickerhamomyces anomalus
Saccharomyces cerevisiae
pH_4
1.5
1.0
0.5
0.0
0
5
10
15
CAS_AUC_k

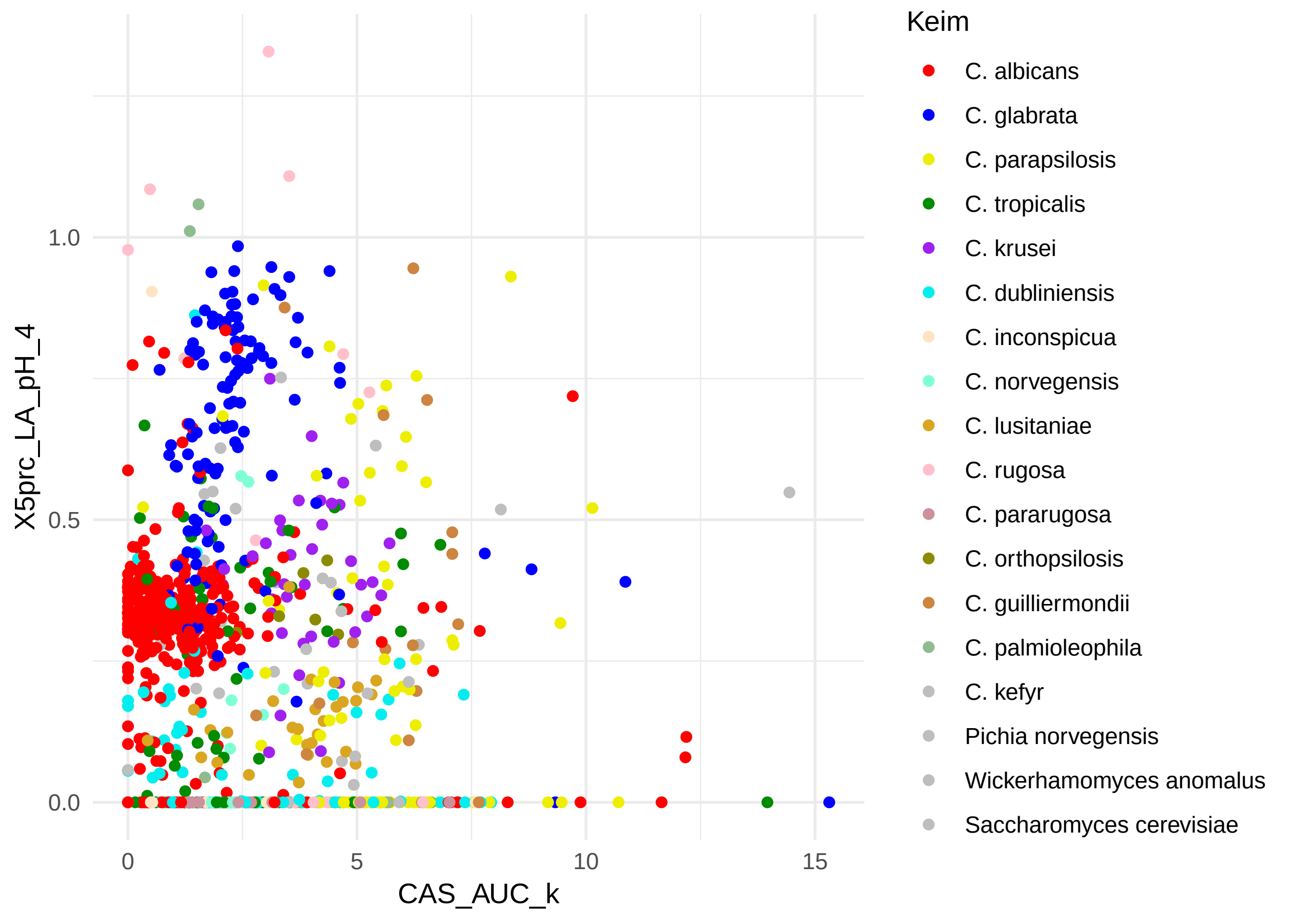

X5prc_LA_pH_4
1.0
0.5
0.0
0
5
10
15
CAS_AUC_k
Keim
C. albicans
C. glabrata
C. parapsilosis
C. tropicalis
C. krusei
C. dubliniensis
C. inconspicua
C. norvegensis
C. lusitaniae
C. rugosa
C. pararugosa
C. orthopsilosis
C. guilliermondii
C. palmioleophila
C. kefyr
Pichia norvegensis
Wickerhamomyces anomalus
Saccharomyces cerevisiae

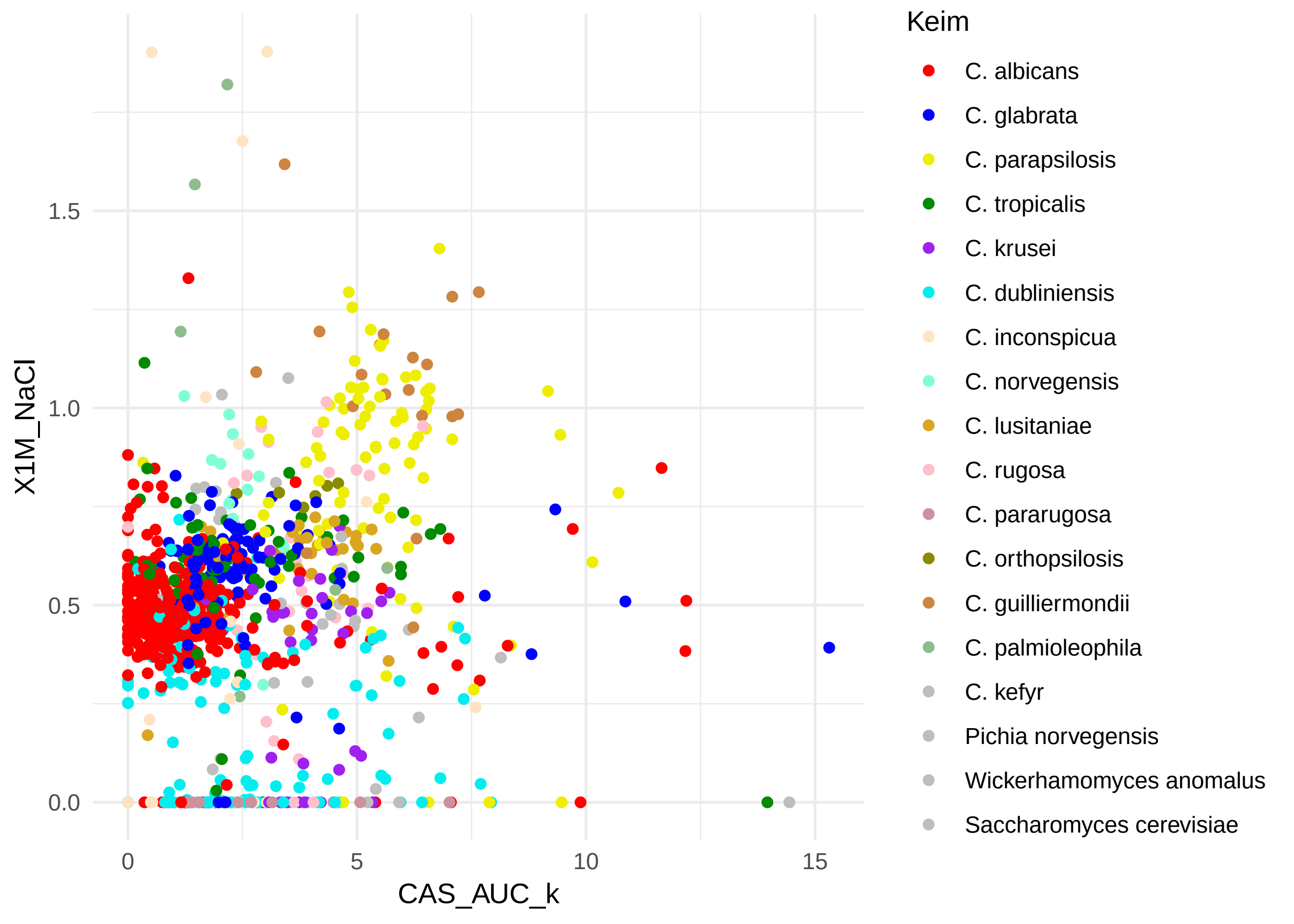
Keim
C. albicans
C. glabrata
C. parapsilosis
C. tropicalis
C. krusei
C. dubliniensis
C. inconspicua
C. norvegensis
C. lusitaniae
C. rugosa
C. pararugosa
C. orthopsilosis
C. guilliermondii
C. palmioleophila
C. kefyr
Pichia norvegensis
Wickerhamomyces anomalus
Saccharomyces cerevisiae
X1M_NaCl
0.0
0.5
1.0
1.5
CAS_AUC_k
0
5
10
15

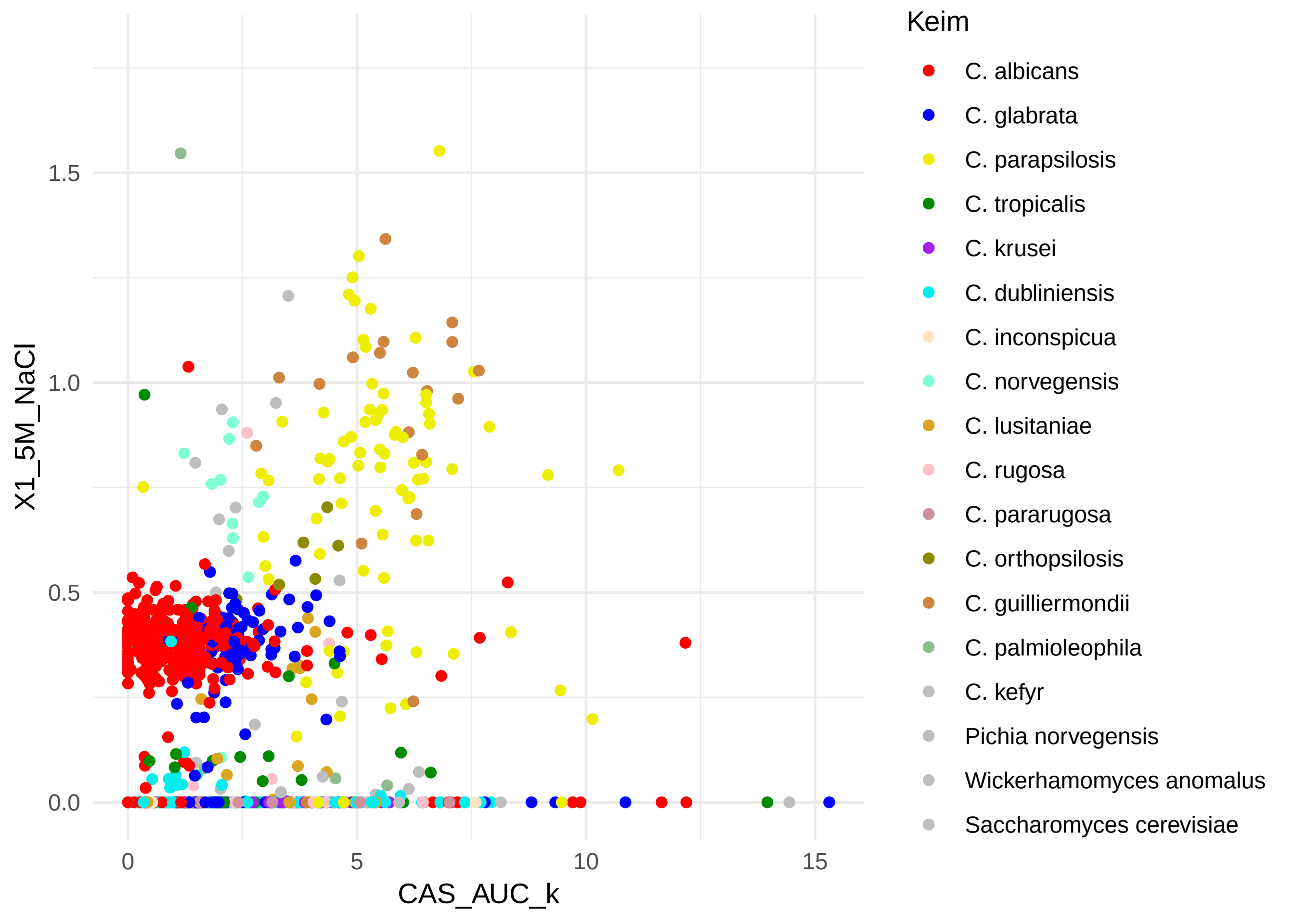
Keim
C. albicans
C. glabrata
C. parapsilosis
C. tropicalis
C. krusei
C. dubliniensis
C. inconspicua
C. norvegensis
C. lusitaniae
C. rugosa
C. pararugosa
C. orthopsilosis
C. guilliermondii
C. palmioleophila
C. kefyr
Pichia norvegensis
Wickerhamomyces anomalus
Saccharomyces cerevisiae
X1_5M_NaCl
CAS_AUC_k
0.0
0.5
1.0
1.5
0
5
10
15

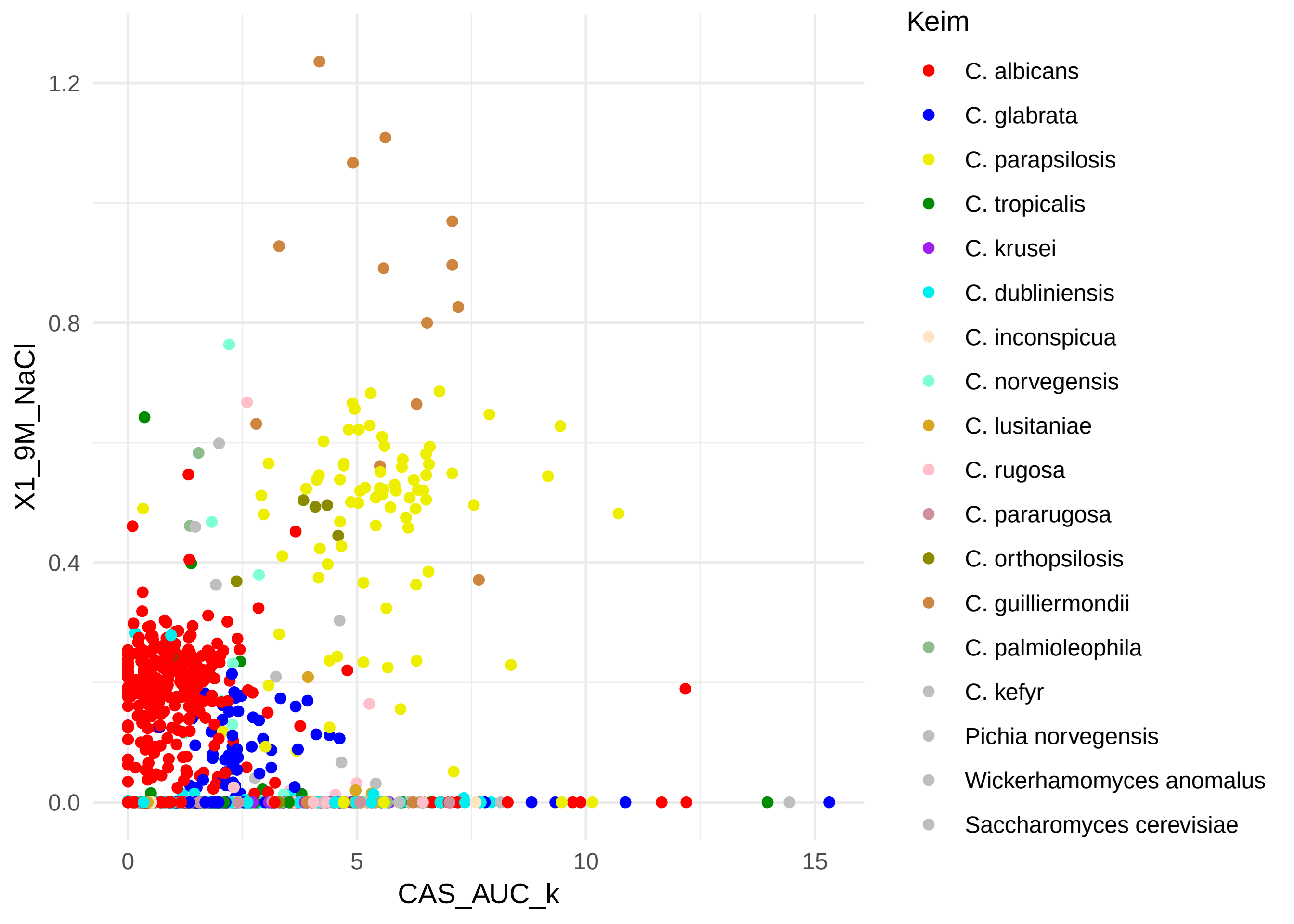
Keim
C. albicans
C. glabrata
C. parapsilosis
C. tropicalis
C. krusei
C. dubliniensis
C. inconspicua
C. norvegensis
C. lusitaniae
C. rugosa
C. pararugosa
C. orthopsilosis
C. guilliermondii
C. palmioleophila
C. kefyr
Pichia norvegensis
Wickerhamomyces anomalus
Saccharomyces cerevisiae
X1_9M_NaCl
1.2
0.8
0.4
0.0
0
5
10
15
CAS_AUC_k

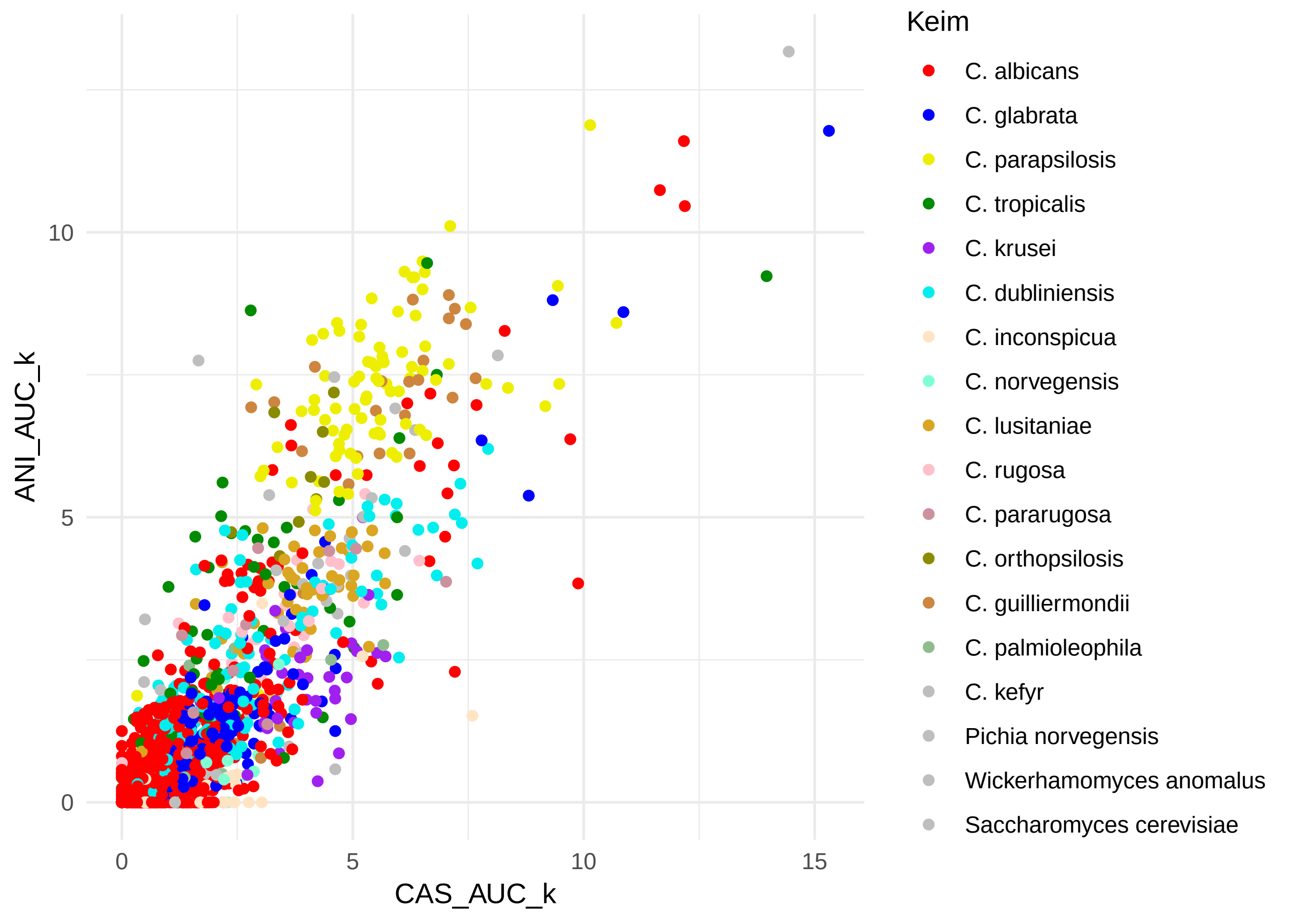

Keim
C. albicans
C. glabrata
C. parapsilosis
C. tropicalis
C. krusei
C. dubliniensis
C. inconspicua
C. norvegensis
C. lusitaniae
C. rugosa
C. pararugosa
C. orthopsilosis
C. guilliermondii
C. palmioleophila
C. kefyr
Pichia norvegensis
Wickerhamomyces anomalus
Saccharomyces cerevisiae
ANI_AUC_k
CAS_AUC_k
0
5
10
15

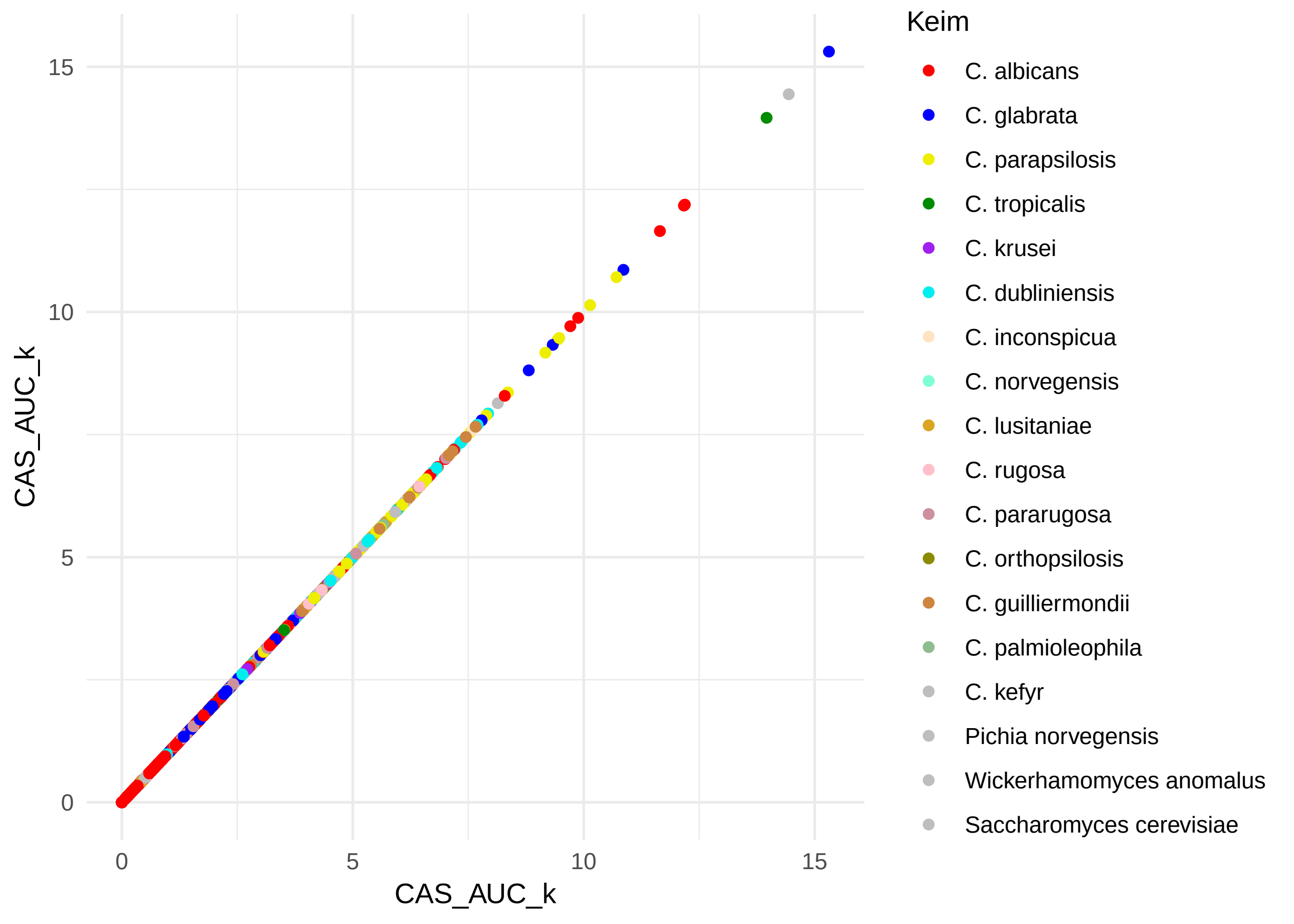
Keim
C. albicans
C. glabrata
C. parapsilosis
C. tropicalis
C. krusei
C. dubliniensis
C. inconspicua
C. norvegensis
C. lusitaniae
C. rugosa
C. pararugosa
C. orthopsilosis
C. guilliermondii
C. palmioleophila
C. kefyr
Pichia norvegensis
Wickerhamomyces anomalus
Saccharomyces cerevisiae
CAS_AUC_k
CAS_AUC_k
0
5
10
15

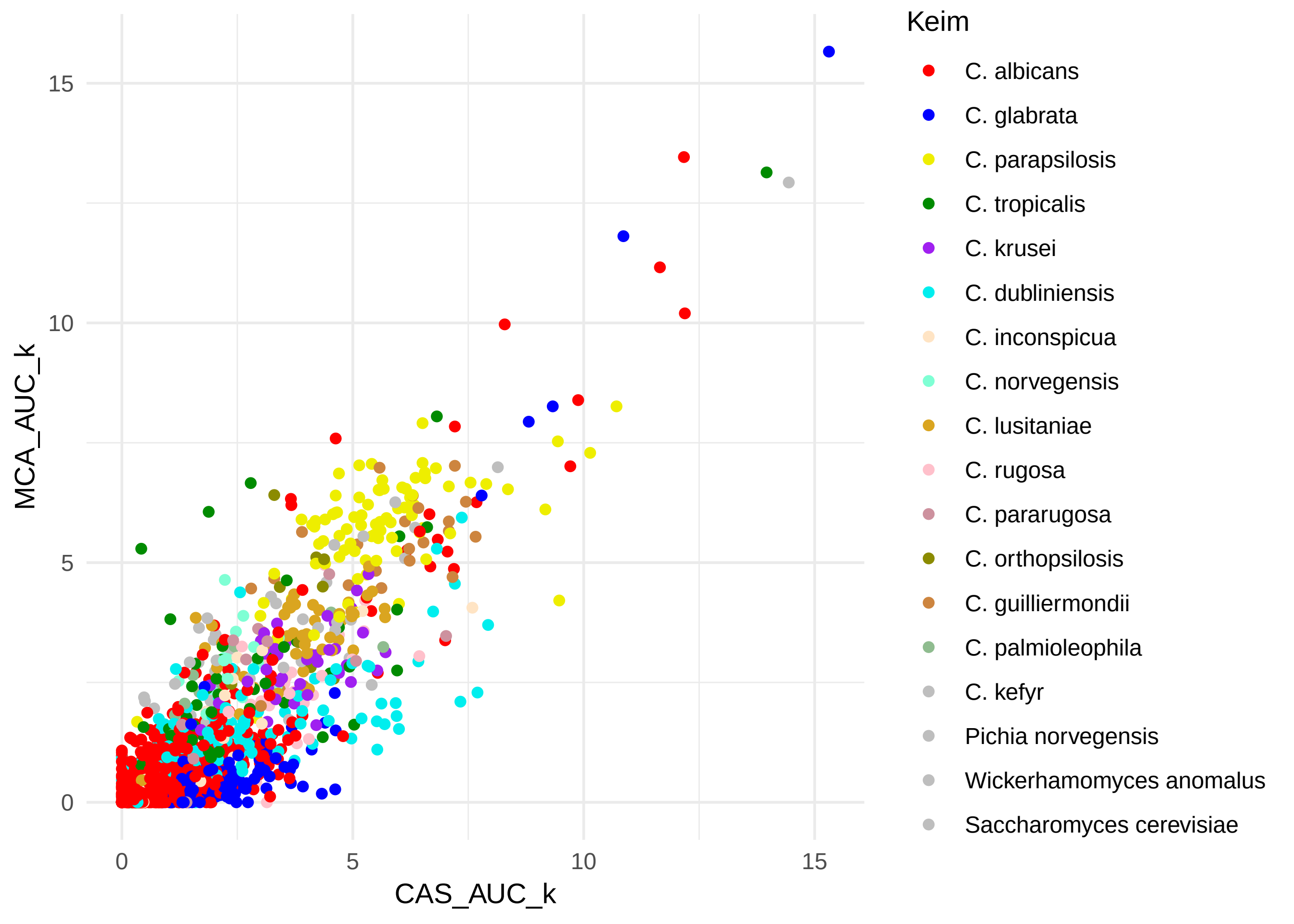

Keim
C. albicans
C. glabrata
C. parapsilosis
C. tropicalis
C. krusei
C. dubliniensis
C. inconspicua
C. norvegensis
C. lusitaniae
C. rugosa
C. pararugosa
C. orthopsilosis
C. guilliermondii
C. palmioleophila
C. kefyr
Pichia norvegensis
Wickerhamomyces anomalus
Saccharomyces cerevisiae
MCA_AUC_k
CAS_AUC_k
0
5
10
15
0
5
10
15

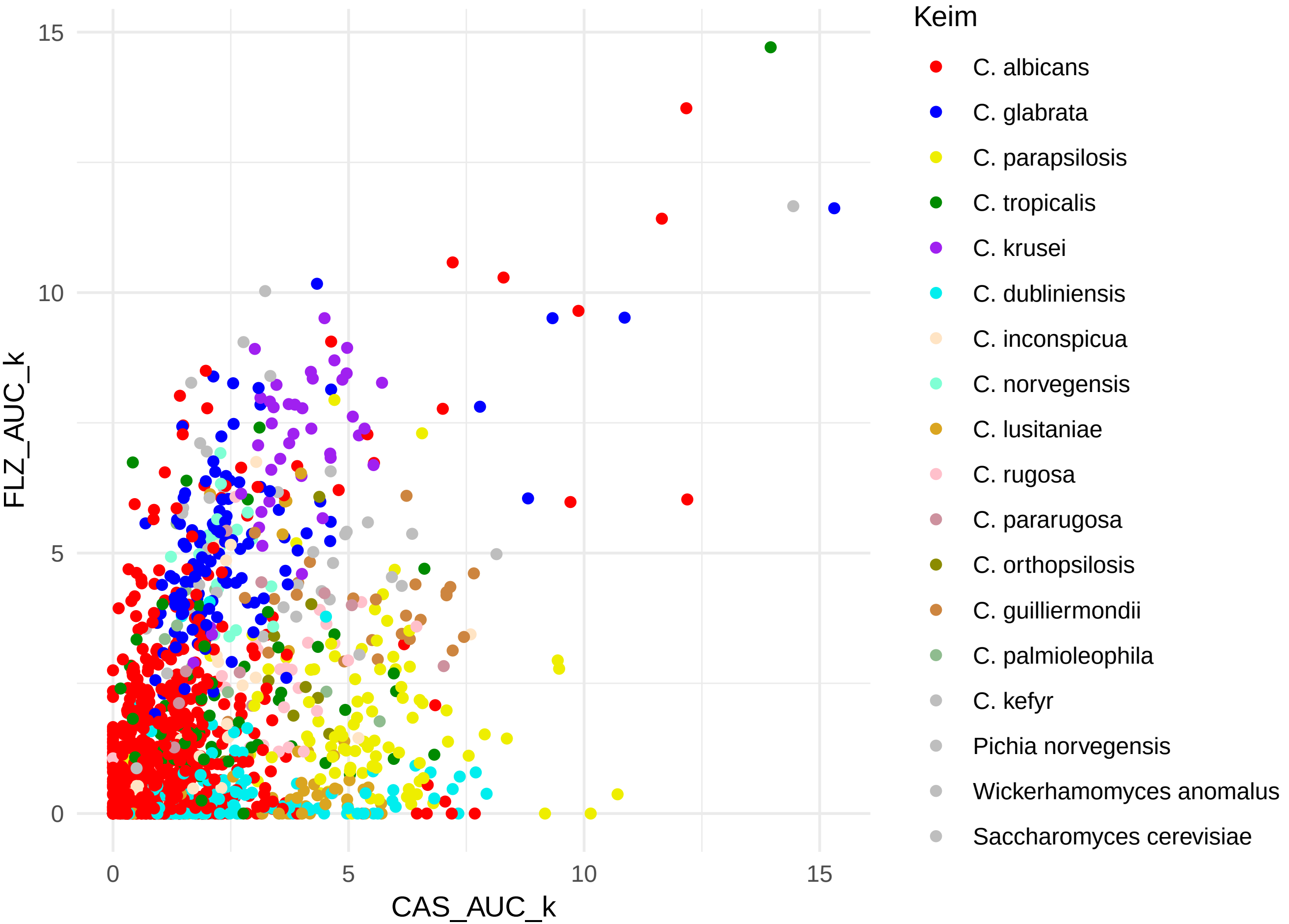

Keim
C. albicans
C. glabrata
C. parapsilosis
C. tropicalis
C. krusei
C. dubliniensis
C. inconspicua
C. norvegensis
C. lusitaniae
C. rugosa
C. pararugosa
C. orthopsilosis
C. guilliermondii
C. palmioleophila
C. kefyr
Pichia norvegensis
Wickerhamomyces anomalus
Saccharomyces cerevisiae
FLZ_AUC_k
CAS_AUC_k
0
5
10
15

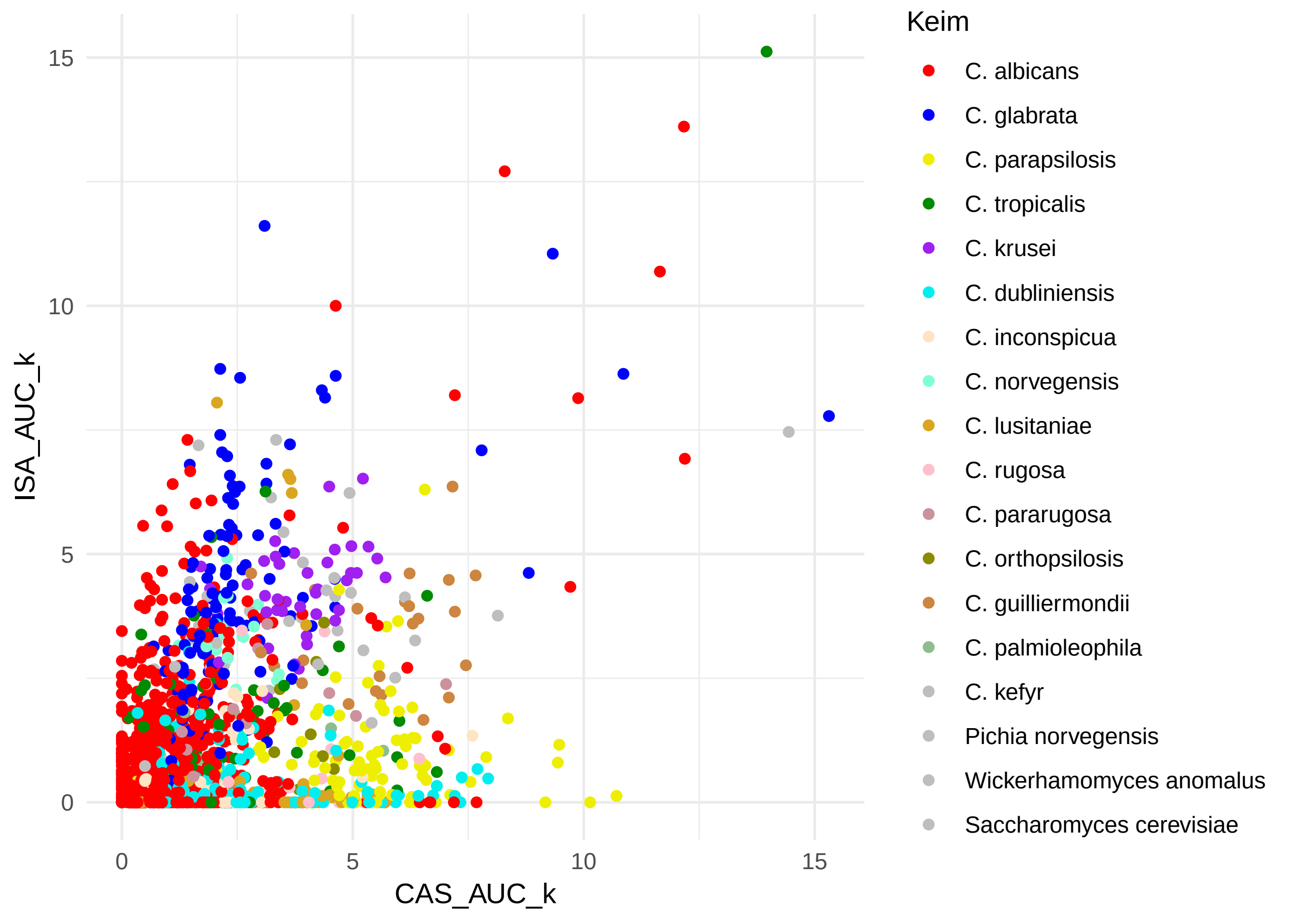

Keim
C. albicans
C. glabrata
C. parapsilosis
C. tropicalis
C. krusei
C. dubliniensis
C. inconspicua
C. norvegensis
C. lusitaniae
C. rugosa
C. pararugosa
C. orthopsilosis
C. guilliermondii
C. palmioleophila
C. kefyr
Pichia norvegensis
Wickerhamomyces anomalus
Saccharomyces cerevisiae
ISA_AUC_k
CAS_AUC_k
0
5
10
15
0
5
10
15

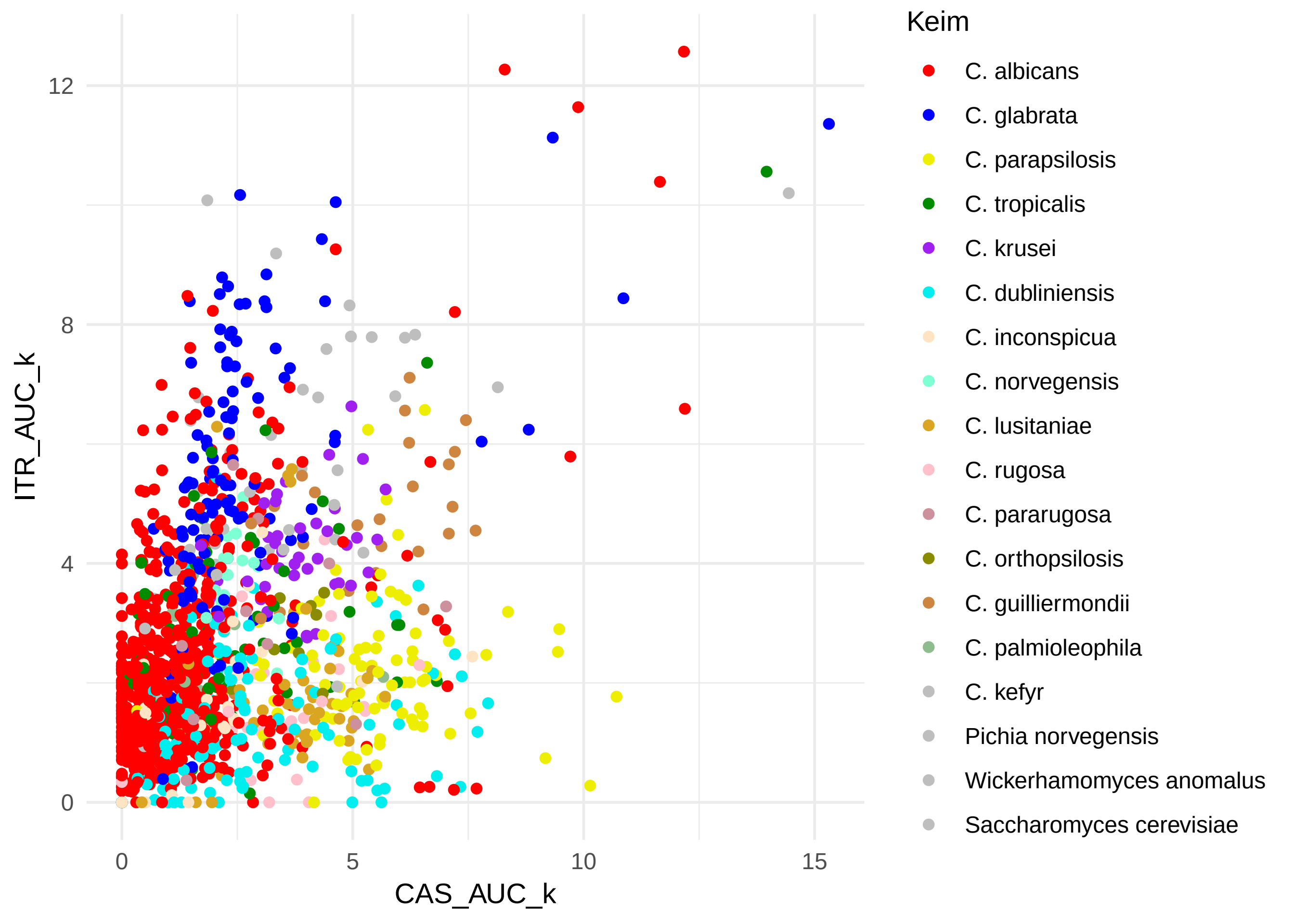
Keim
C. albicans
C. glabrata
C. parapsilosis
C. tropicalis
C. krusei
C. dubliniensis
C. inconspicua
C. norvegensis
C. lusitaniae
C. rugosa
C. pararugosa
C. orthopsilosis
C. guilliermondii
C. palmioleophila
C. kefyr
Pichia norvegensis
Wickerhamomyces anomalus
Saccharomyces cerevisiae
ITR_AUC_k
CAS_AUC_k
0
4
8
12
0
5
10
15

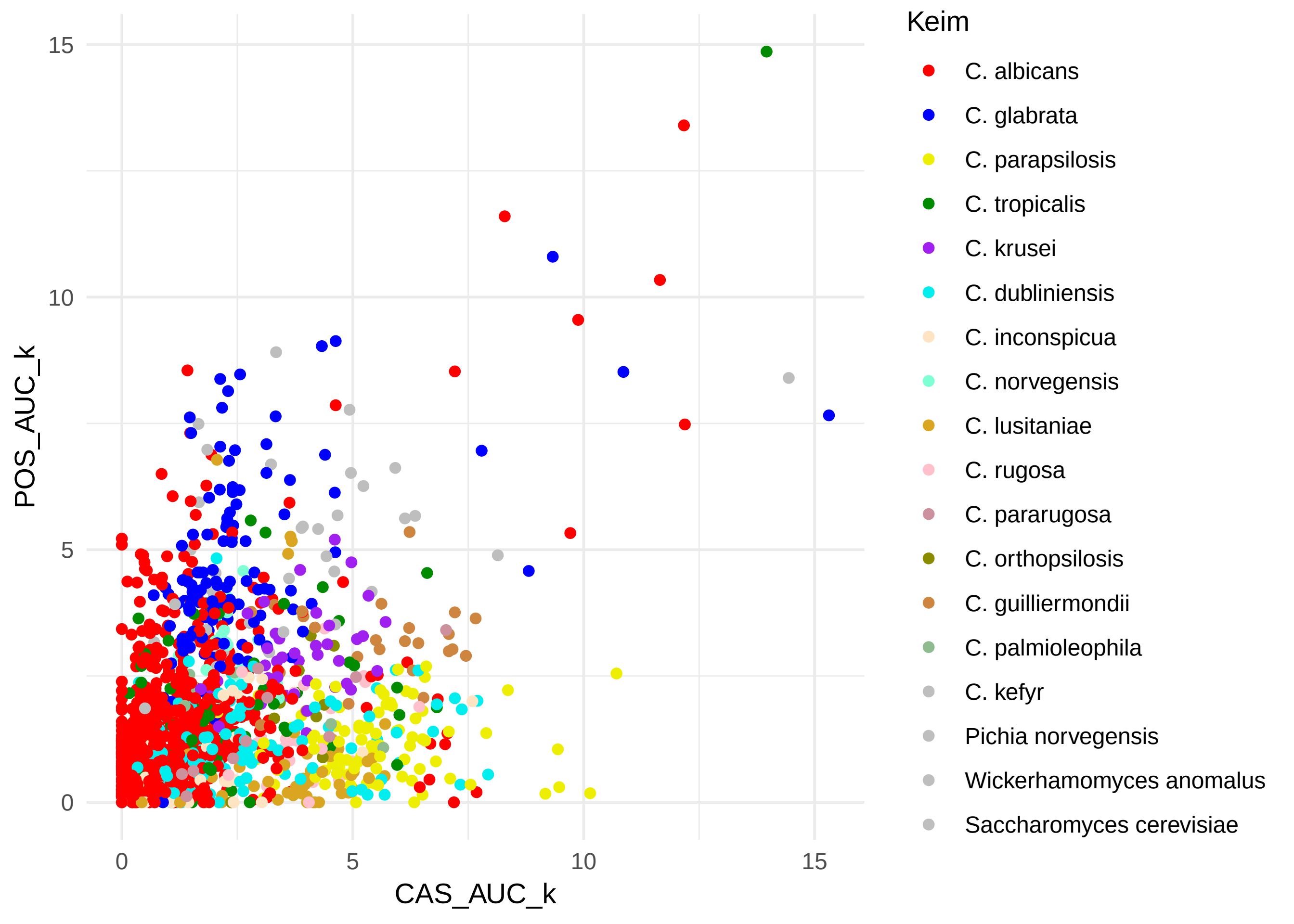
Keim
C. albicans
C. glabrata
C. parapsilosis
C. tropicalis
C. krusei
C. dubliniensis
C. inconspicua
C. norvegensis
C. lusitaniae
C. rugosa
C. pararugosa
C. orthopsilosis
C. guilliermondii
C. palmioleophila
C. kefyr
Pichia norvegensis
Wickerhamomyces anomalus
Saccharomyces cerevisiae
POS_AUC_k
CAS_AUC_k
0
5
10
15
0
5
10
15

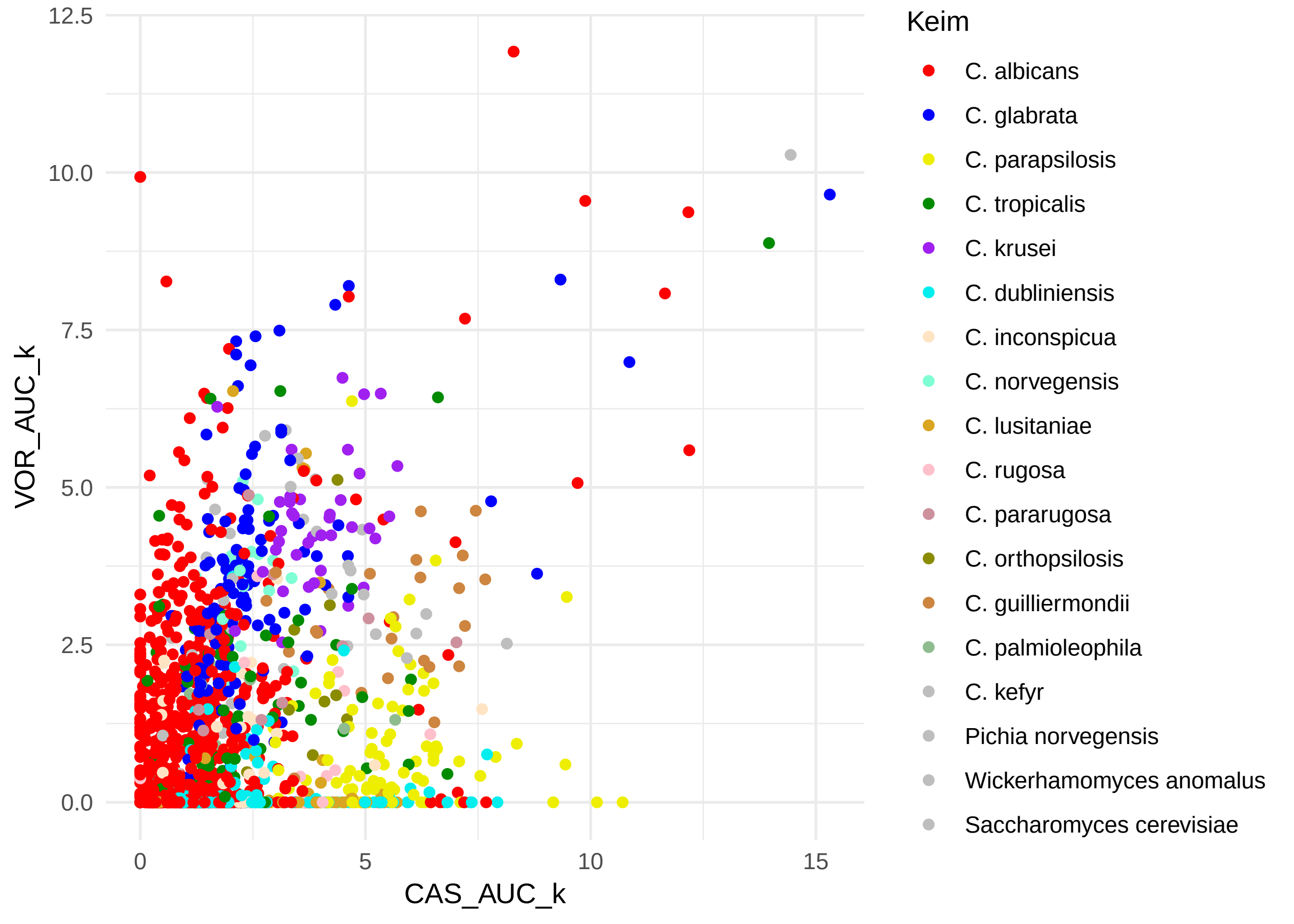

VOR_AUC_k
CAS_AUC_k
0.0
2.5
5.0
7.5
10.0
12.5
0
5
10
15
Keim
C. albicans
C. glabrata
C. parapsilosis
C. tropicalis
C. krusei
C. dubliniensis
C. inconspicua
C. norvegensis
C. lusitaniae
C. rugosa
C. pararugosa
C. orthopsilosis
C. guilliermondii
C. palmioleophila
C. kefyr
Pichia norvegensis
Wickerhamomyces anomalus
Saccharomyces cerevisiae

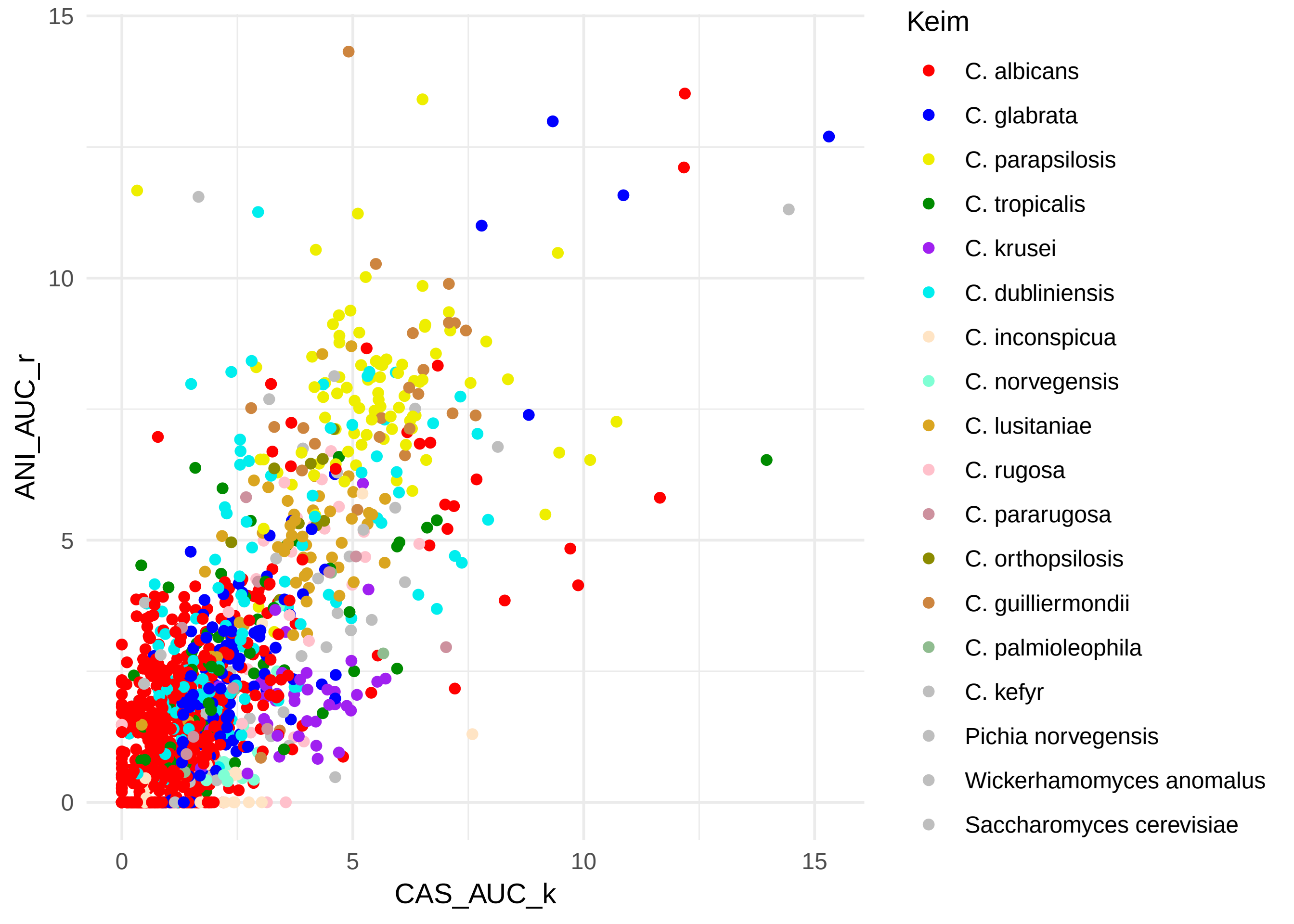

ANI_AUC_r
CAS_AUC_k
0
5
10
15
0
5
10
15
Keim
C. albicans
C. glabrata
C. parapsilosis
C. tropicalis
C. krusei
C. dubliniensis
C. inconspicua
C. norvegensis
C. lusitaniae
C. rugosa
C. pararugosa
C. orthopsilosis
C. guilliermondii
C. palmioleophila
C. kefyr
Pichia norvegensis
Wickerhamomyces anomalus
Saccharomyces cerevisiae

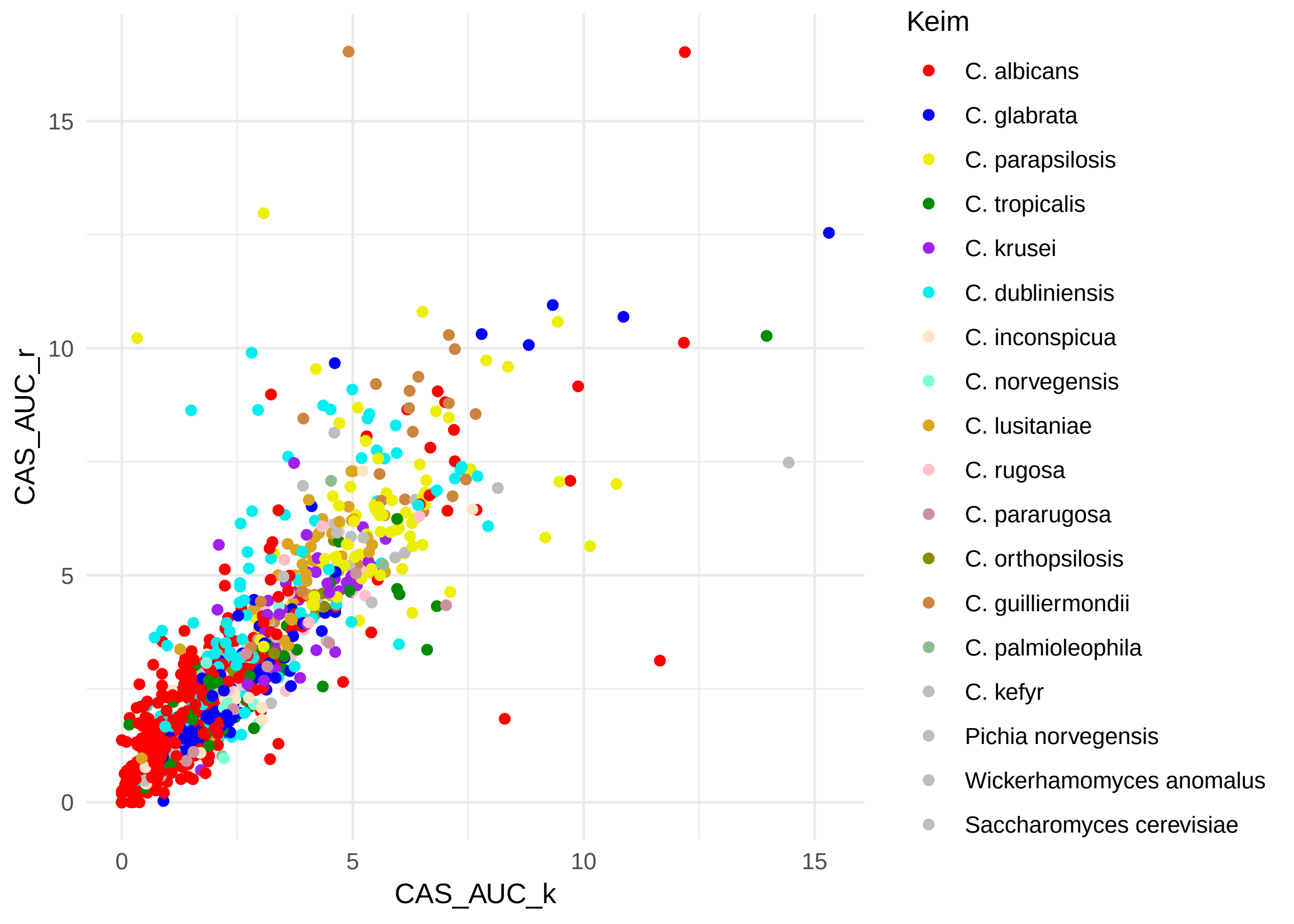

Keim
C. albicans
C. glabrata
C. parapsilosis
C. tropicalis
C. krusei
C. dubliniensis
C. inconspicua
C. norvegensis
C. lusitaniae
C. rugosa
C. pararugosa
C. orthopsilosis
C. guilliermondii
C. palmioleophila
C. kefyr
Pichia norvegensis
Wickerhamomyces anomalus
Saccharomyces cerevisiae
CAS_AUC_r
CAS_AUC_k
0
5
10
15

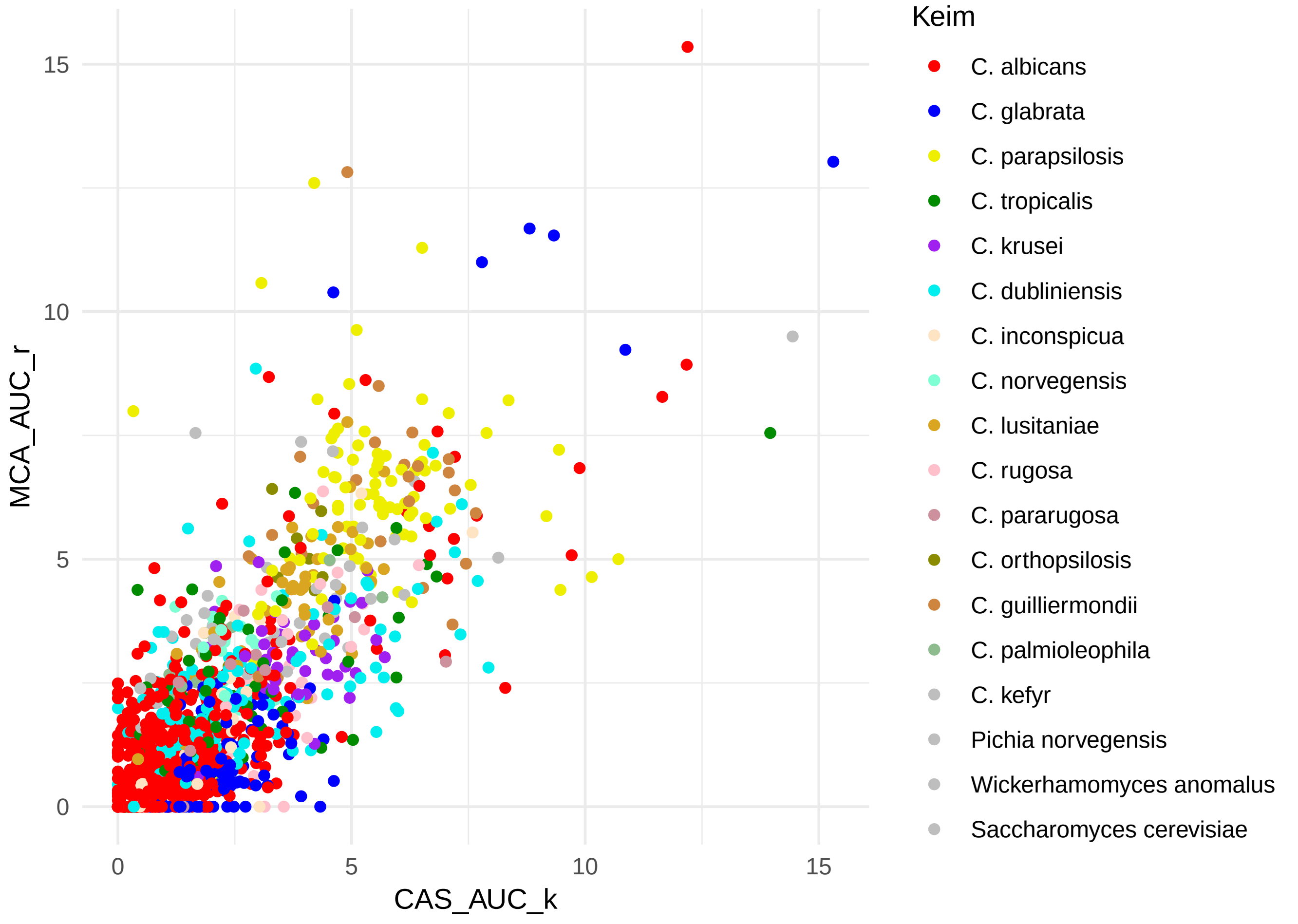
Keim
C. albicans
C. glabrata
C. parapsilosis
C. tropicalis
C. krusei
C. dubliniensis
C. inconspicua
C. norvegensis
C. lusitaniae
C. rugosa
C. pararugosa
C. orthopsilosis
C. guilliermondii
C. palmioleophila
C. kefyr
Pichia norvegensis
Wickerhamomyces anomalus
Saccharomyces cerevisiae
MCA_AUC_r
CAS_AUC_k
0
5
10
15

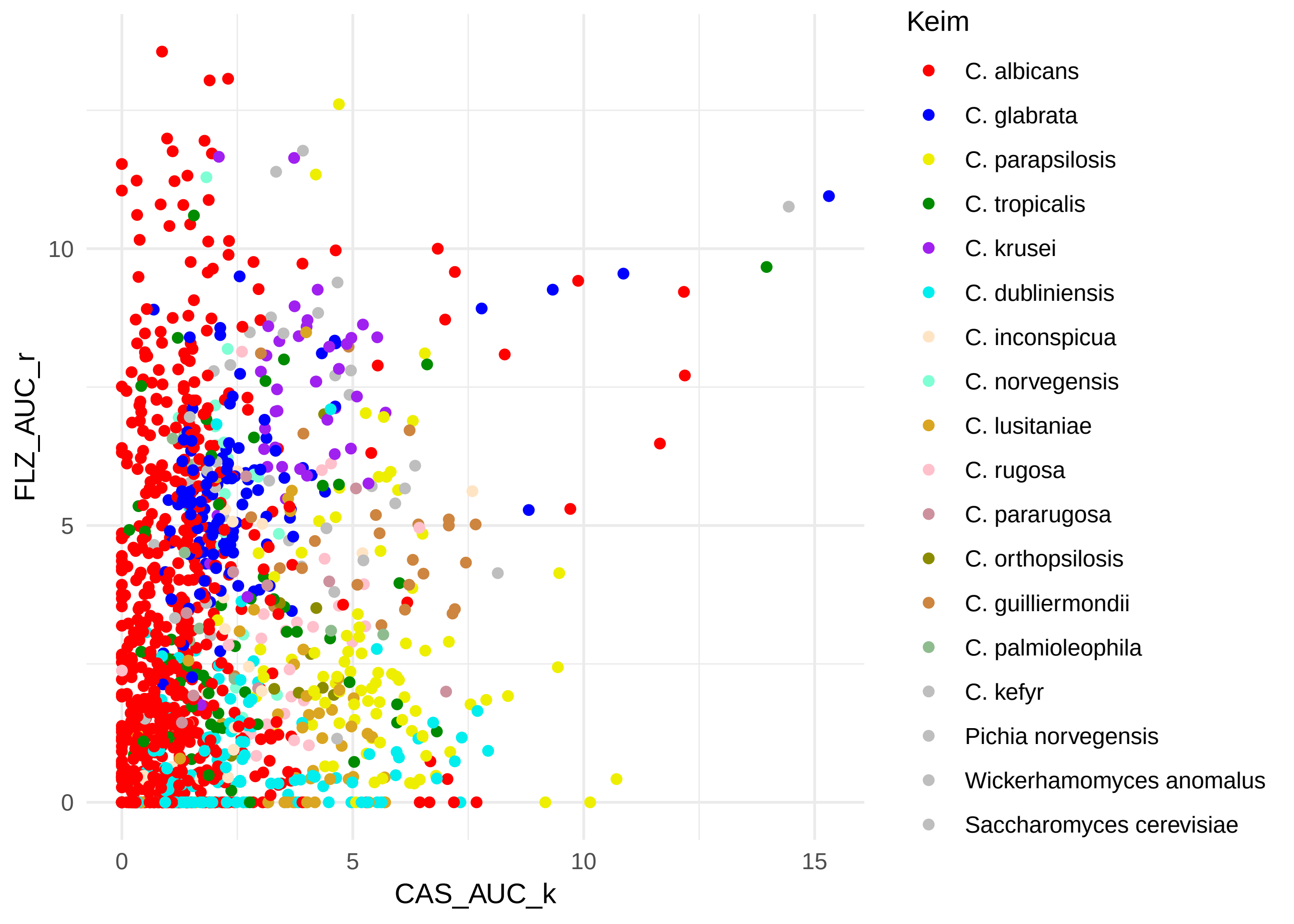

Keim
C. albicans
C. glabrata
C. parapsilosis
C. tropicalis
C. krusei
C. dubliniensis
C. inconspicua
C. norvegensis
C. lusitaniae
C. rugosa
C. pararugosa
C. orthopsilosis
C. guilliermondii
C. palmioleophila
C. kefyr
Pichia norvegensis
Wickerhamomyces anomalus
Saccharomyces cerevisiae
FLZ_AUC_r
CAS_AUC_k
0
5
10
15

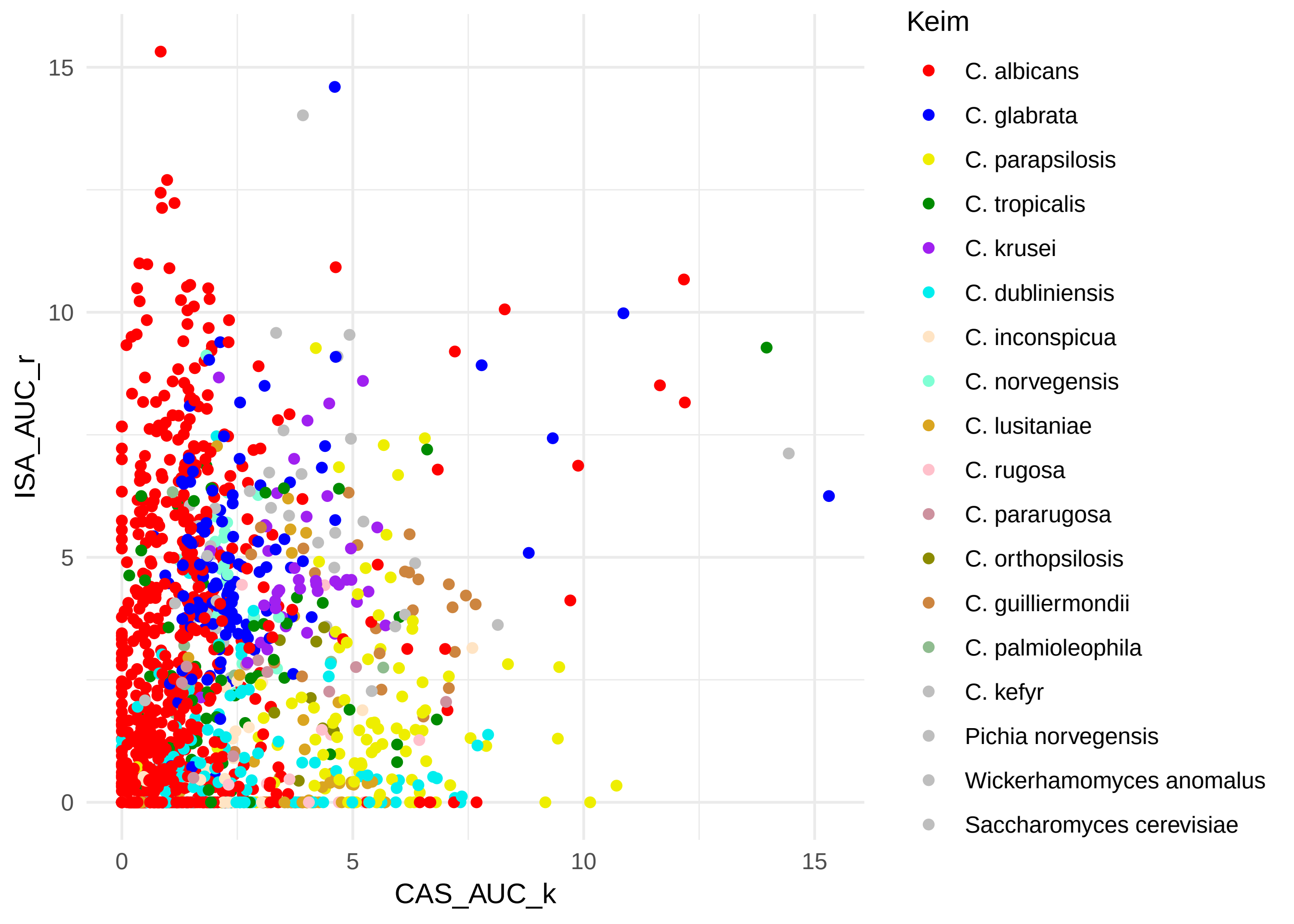

Keim
C. albicans
C. glabrata
C. parapsilosis
C. tropicalis
C. krusei
C. dubliniensis
C. inconspicua
C. norvegensis
C. lusitaniae
C. rugosa
C. pararugosa
C. orthopsilosis
C. guilliermondii
C. palmioleophila
C. kefyr
Pichia norvegensis
Wickerhamomyces anomalus
Saccharomyces cerevisiae
ISA_AUC_r
CAS_AUC_k
0
5
10
15

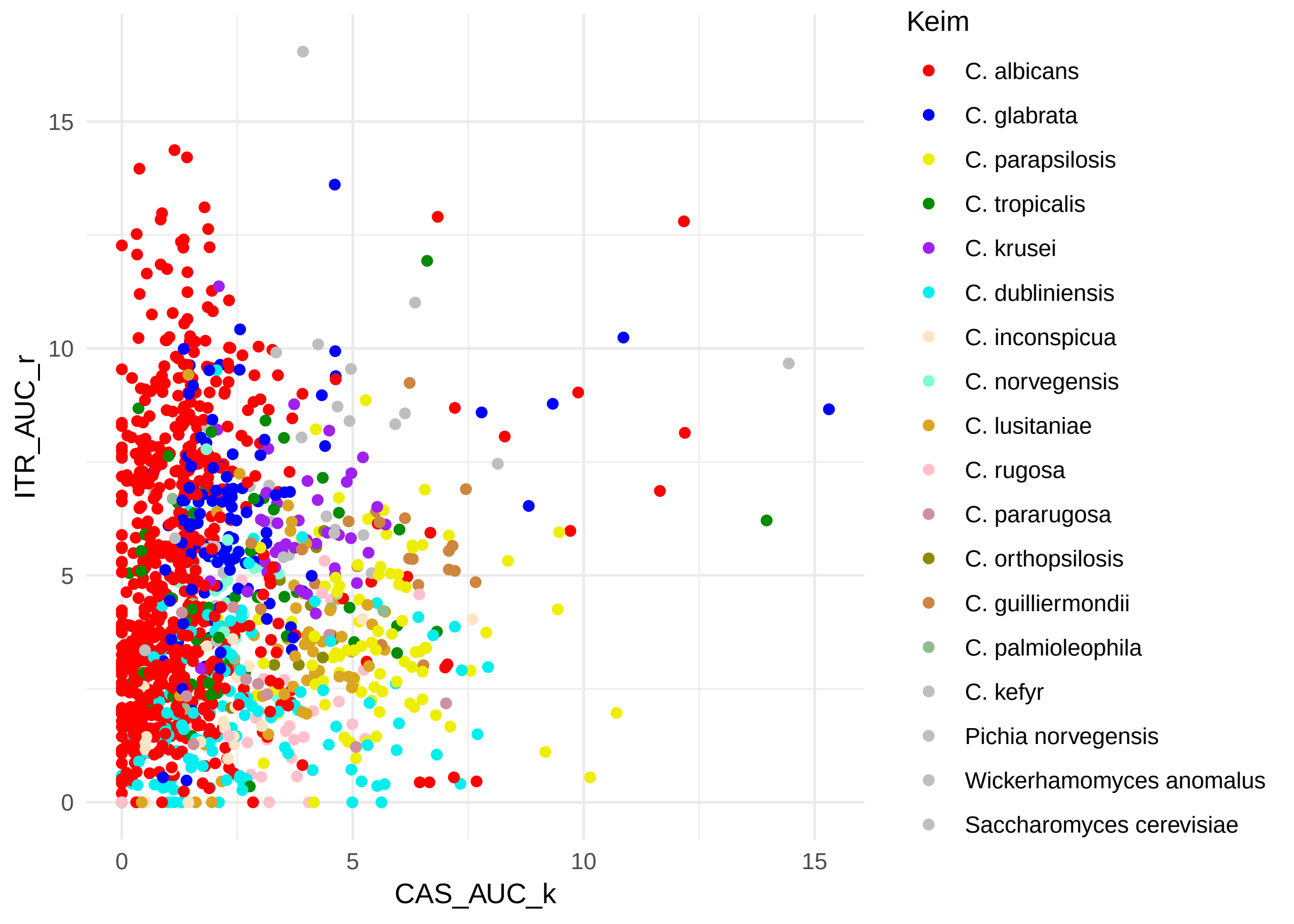

Keim
C. albicans
C. glabrata
C. parapsilosis
C. tropicalis
C. krusei
C. dubliniensis
C. inconspicua
C. norvegensis
C. lusitaniae
C. rugosa
C. pararugosa
C. orthopsilosis
C. guilliermondii
C. palmioleophila
C. kefyr
Pichia norvegensis
Wickerhamomyces anomalus
Saccharomyces cerevisiae
ITR_AUC_r
CAS_AUC_k
0
5
10
15

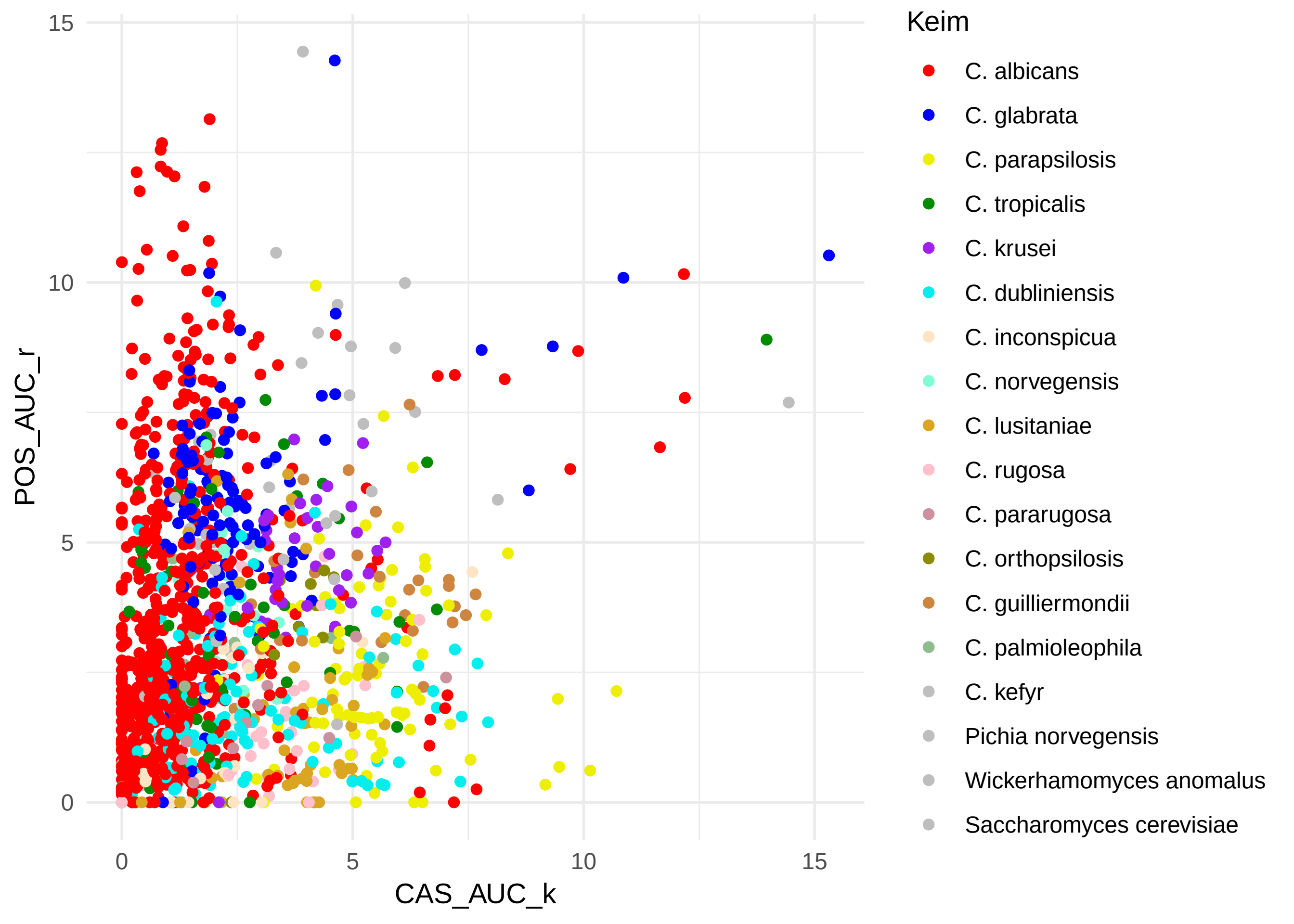
POS_AUC_r
CAS_AUC_k
0
5
10
15
0
5
10
15
Keim
C. albicans
C. glabrata
C. parapsilosis
C. tropicalis
C. krusei
C. dubliniensis
C. inconspicua
C. norvegensis
C. lusitaniae
C. rugosa
C. pararugosa
C. orthopsilosis
C. guilliermondii
C. palmioleophila
C. kefyr
Pichia norvegensis
Wickerhamomyces anomalus
Saccharomyces cerevisiae

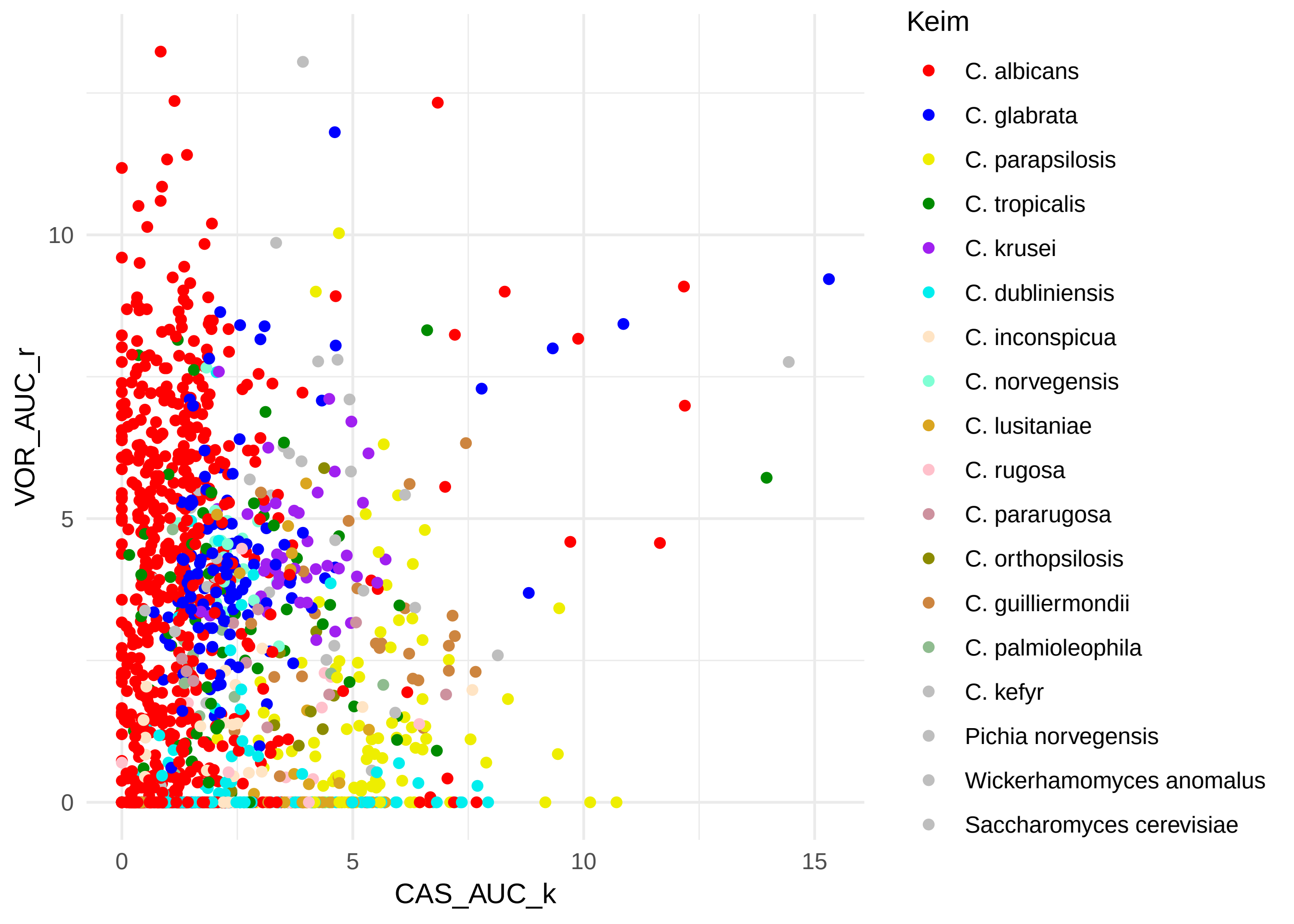
Keim
C. albicans
C. glabrata
C. parapsilosis
C. tropicalis
C. krusei
C. dubliniensis
C. inconspicua
C. norvegensis
C. lusitaniae
C. rugosa
C. pararugosa
C. orthopsilosis
C. guilliermondii
C. palmioleophila
C. kefyr
Pichia norvegensis
Wickerhamomyces anomalus
Saccharomyces cerevisiae
VOR_AUC_r
10
5
0
CAS_AUC_k
0
5
10
15

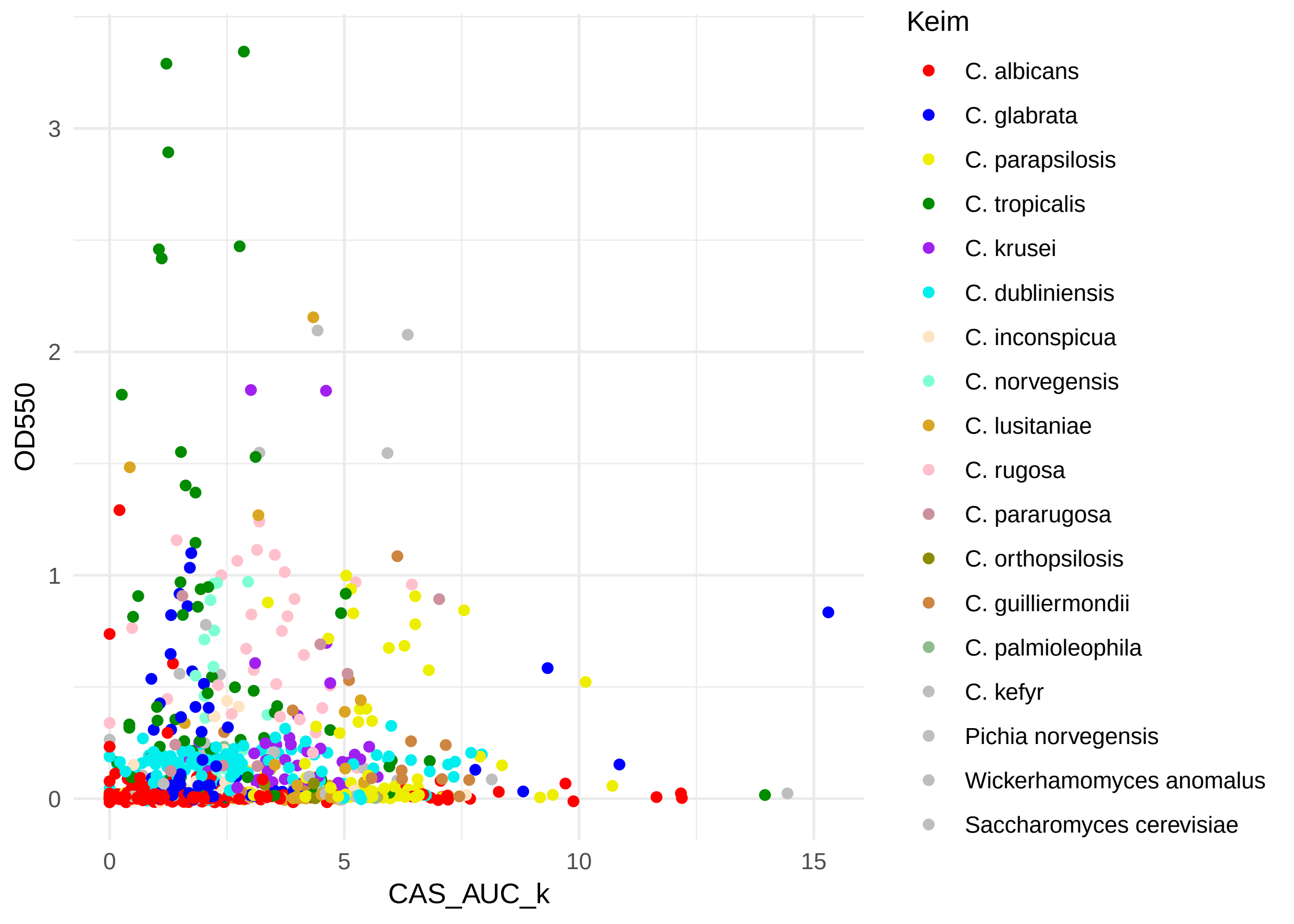

OD550
3
2
1
0
0
5
10
15
CAS_AUC_k
Keim
C. albicans
C. glabrata
C. parapsilosis
C. tropicalis
C. krusei
C. dubliniensis
C. inconspicua
C. norvegensis
C. lusitaniae
C. rugosa
C. pararugosa
C. orthopsilosis
C. guilliermondii
C. palmioleophila
C. kefyr
Pichia norvegensis
Wickerhamomyces anomalus
Saccharomyces cerevisiae

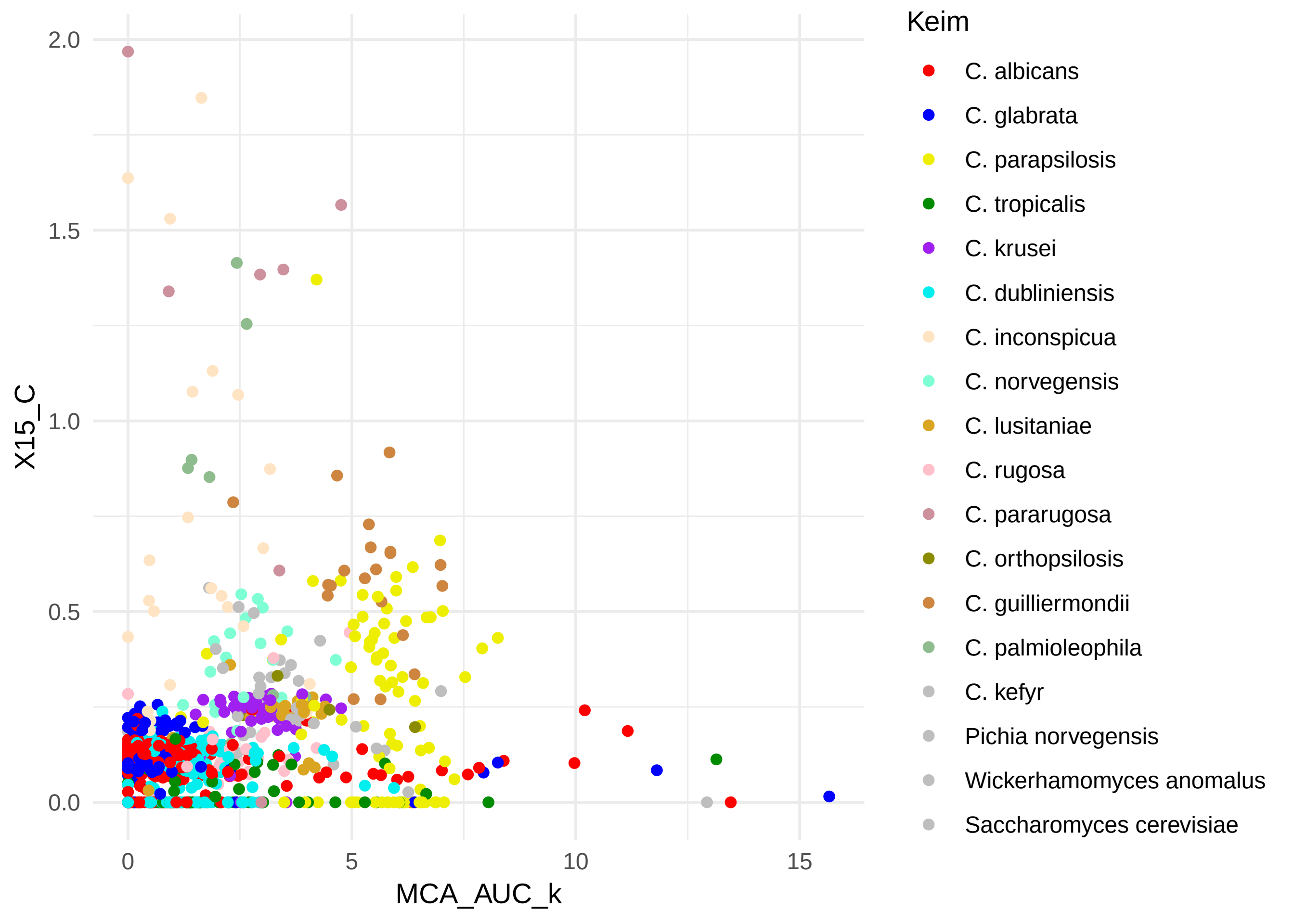
Keim
C. albicans
C. glabrata
C. parapsilosis
C. tropicalis
C. krusei
C. dubliniensis
C. inconspicua
C. norvegensis
C. lusitaniae
C. rugosa
C. pararugosa
C. orthopsilosis
C. guilliermondii
C. palmioleophila
C. kefyr
Pichia norvegensis
Wickerhamomyces anomalus
Saccharomyces cerevisiae
X15_C
MCA_AUC_k
2.0
1.5
1.0
0.5
0.0
0
5
10
15

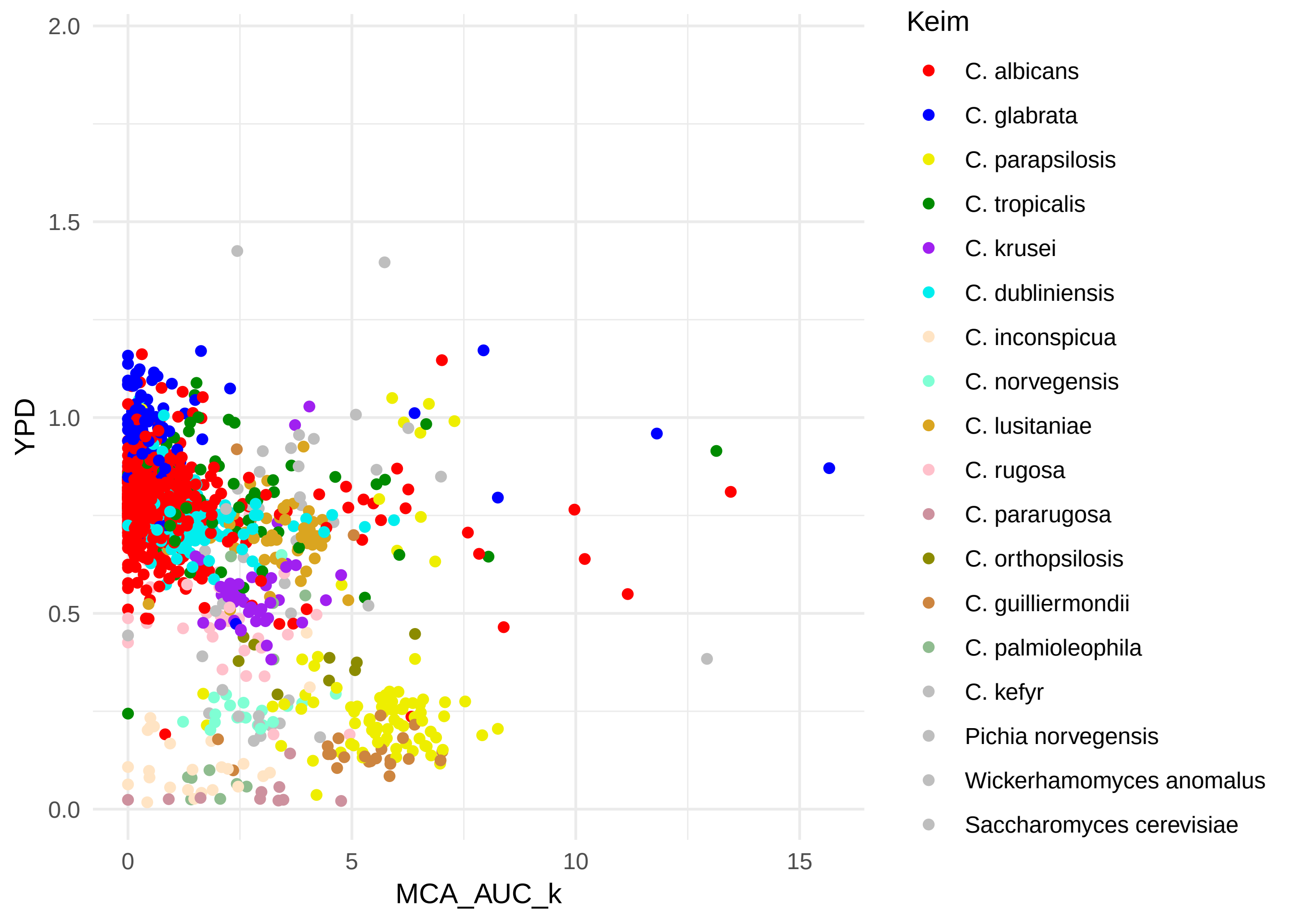

2.0
1.5
1.0
0.5
0.0
YPD
0
5
10
15
MCA_AUC_k
Keim
C. albicans
C. glabrata
C. parapsilosis
C. tropicalis
C. krusei
C. dubliniensis
C. inconspicua
C. norvegensis
C. lusitaniae
C. rugosa
C. pararugosa
C. orthopsilosis
C. guilliermondii
C. palmioleophila
C. kefyr
Pichia norvegensis
Wickerhamomyces anomalus
Saccharomyces cerevisiae

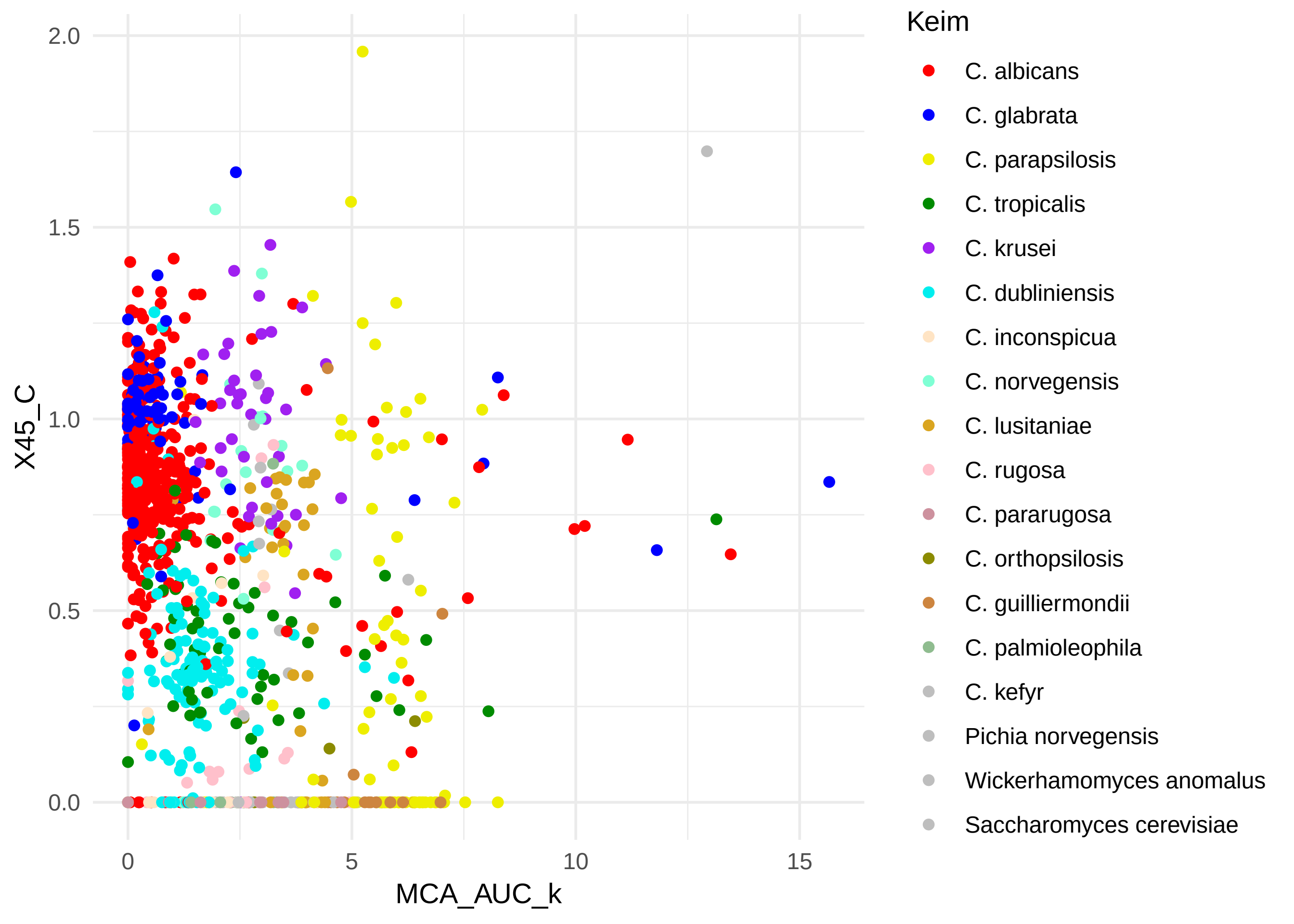

Keim
C. albicans
C. glabrata
C. parapsilosis
C. tropicalis
C. krusei
C. dubliniensis
C. inconspicua
C. norvegensis
C. lusitaniae
C. rugosa
C. pararugosa
C. orthopsilosis
C. guilliermondii
C. palmioleophila
C. kefyr
Pichia norvegensis
Wickerhamomyces anomalus
Saccharomyces cerevisiae
X45_C
2.0
1.5
1.0
0.5
0.0
0
5
10
15
MCA_AUC_k

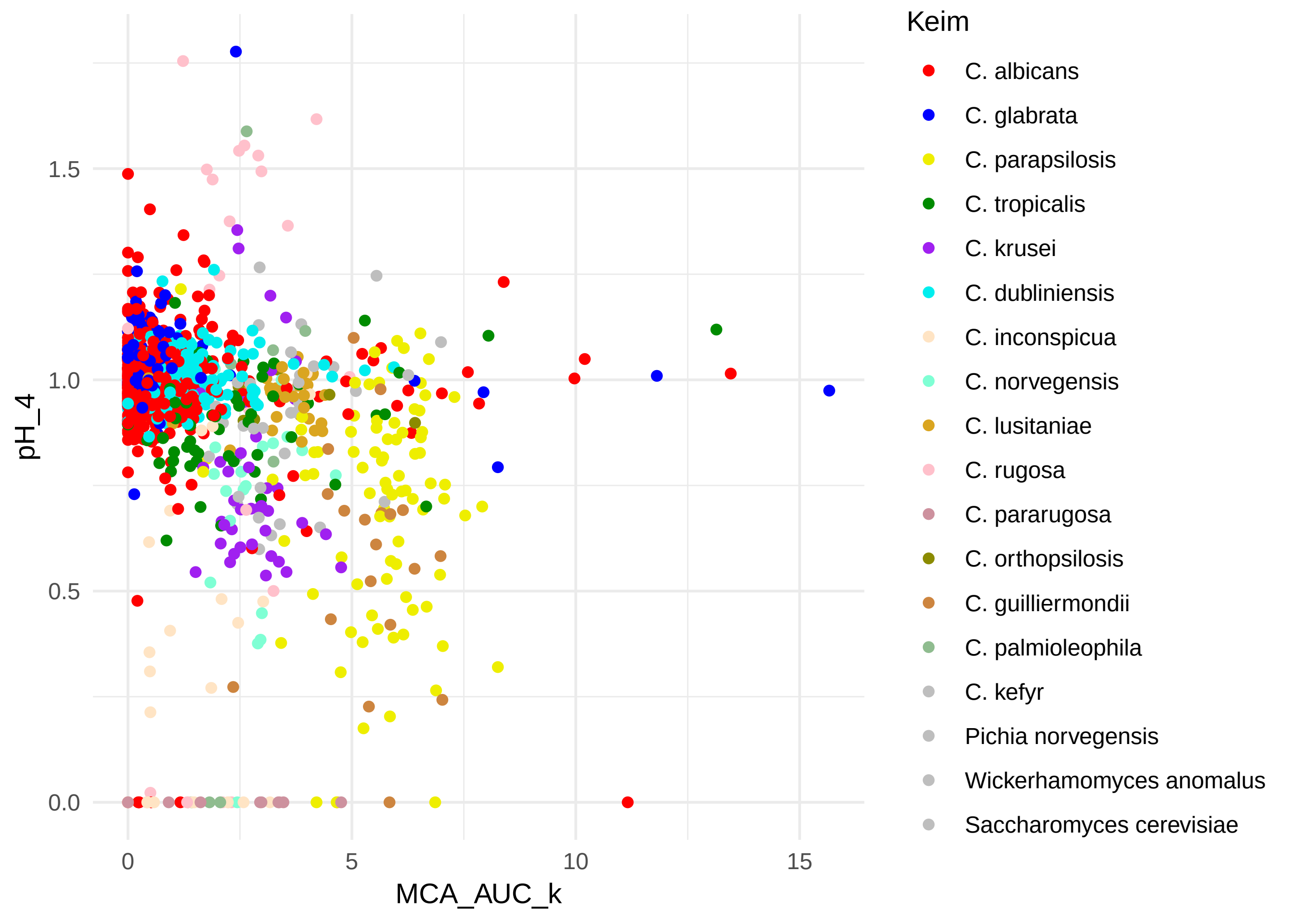

Keim
C. albicans
C. glabrata
C. parapsilosis
C. tropicalis
C. krusei
C. dubliniensis
C. inconspicua
C. norvegensis
C. lusitaniae
C. rugosa
C. pararugosa
C. orthopsilosis
C. guilliermondii
C. palmioleophila
C. kefyr
Pichia norvegensis
Wickerhamomyces anomalus
Saccharomyces cerevisiae
pH_4
MCA_AUC_k
0.0
0.5
1.0
1.5
0
5
10
15

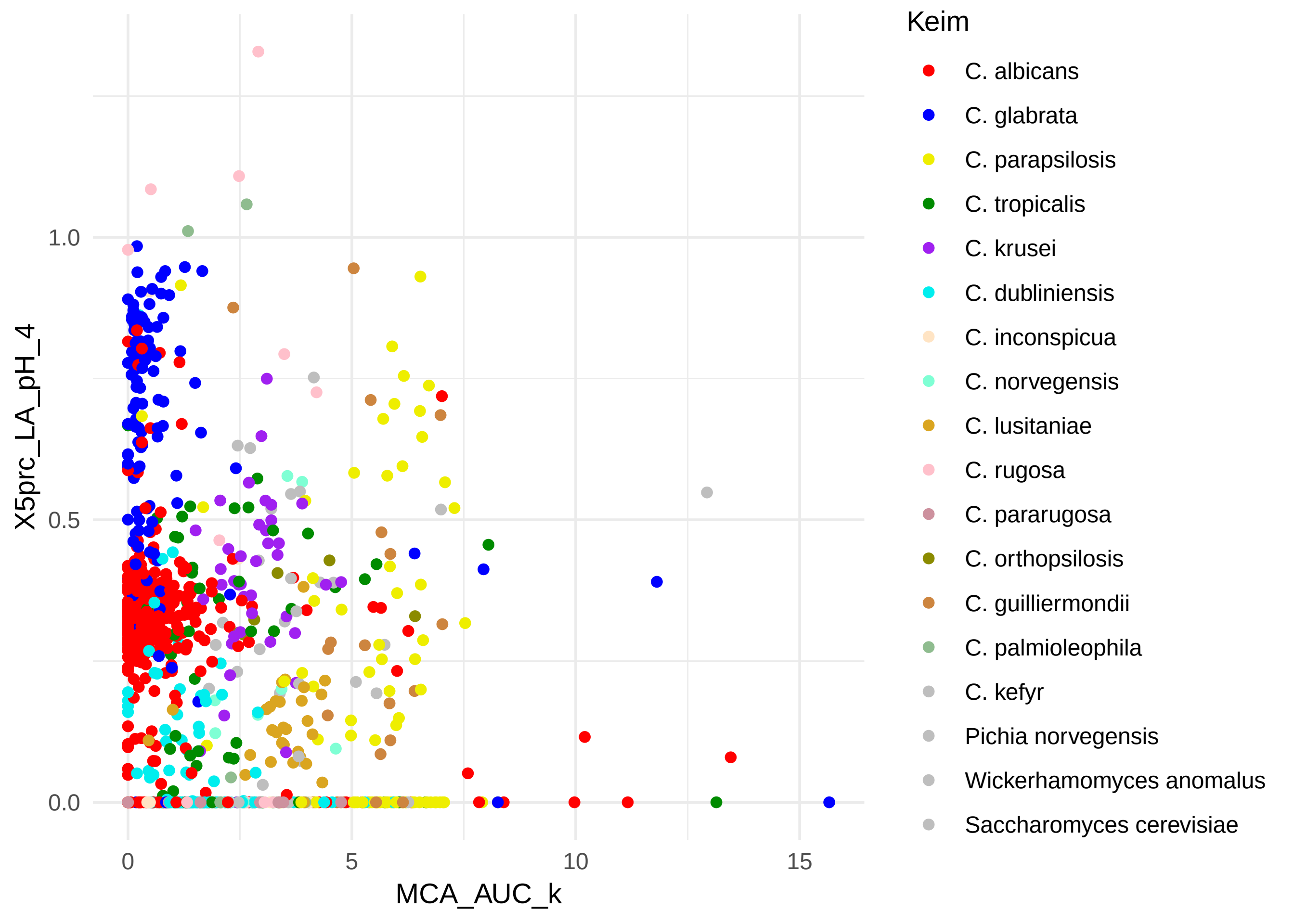
Keim
C. albicans
C. glabrata
C. parapsilosis
C. tropicalis
C. krusei
C. dubliniensis
C. inconspicua
C. norvegensis
C. lusitaniae
C. rugosa
C. pararugosa
C. orthopsilosis
C. guilliermondii
C. palmioleophila
C. kefyr
Pichia norvegensis
Wickerhamomyces anomalus
Saccharomyces cerevisiae
X5prc_LA_pH_4
1.0
0.5
0.0
0
5
10
15
MCA_AUC_k

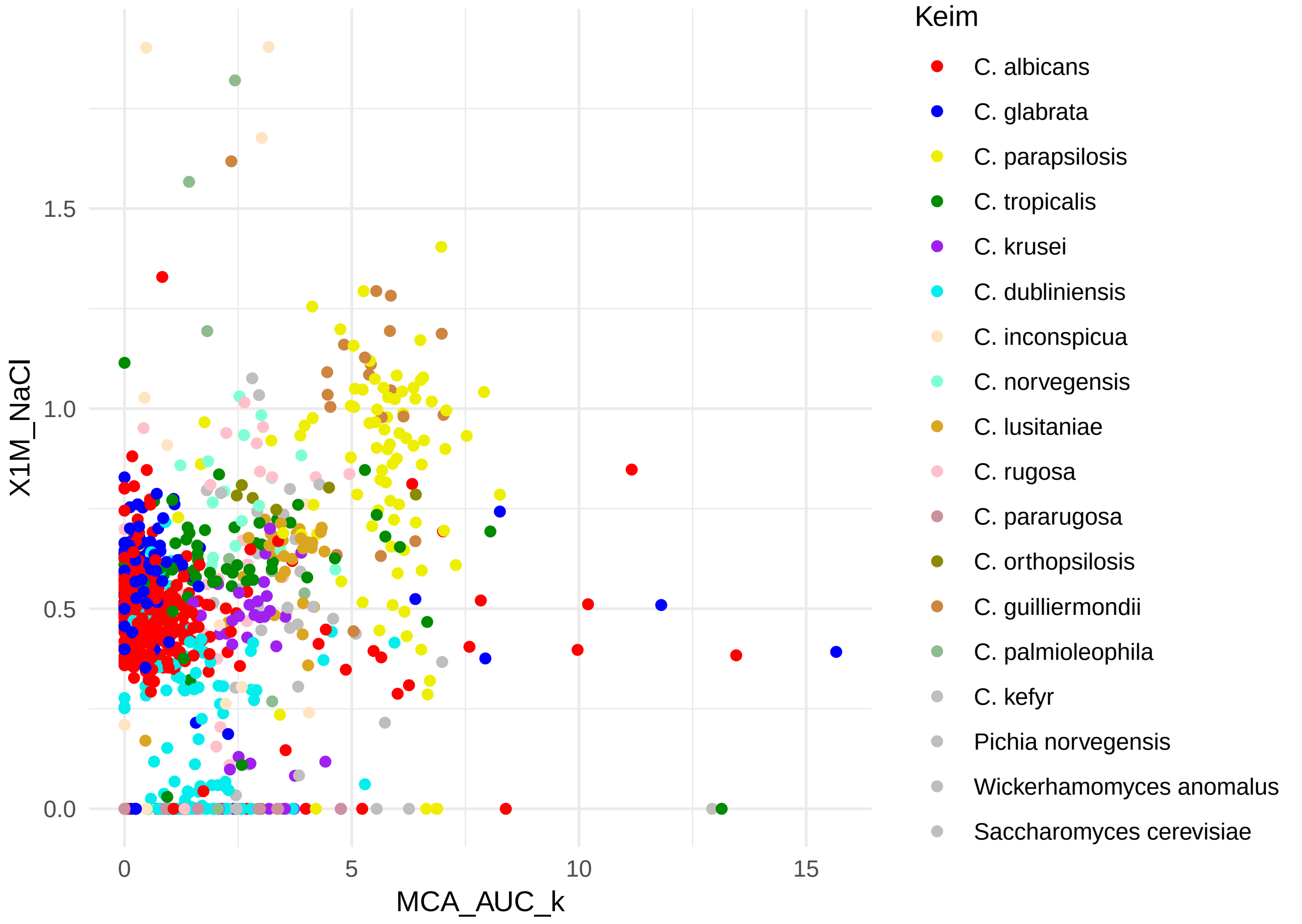

Keim
C. albicans
C. glabrata
C. parapsilosis
C. tropicalis
C. krusei
C. dubliniensis
C. inconspicua
C. norvegensis
C. lusitaniae
C. rugosa
C. pararugosa
C. orthopsilosis
C. guilliermondii
C. palmioleophila
C. kefyr
Pichia norvegensis
Wickerhamomyces anomalus
Saccharomyces cerevisiae
X1M_NaCl
MCA_AUC_k
1.5
1.0
0.5
0.0
0
5
10
15

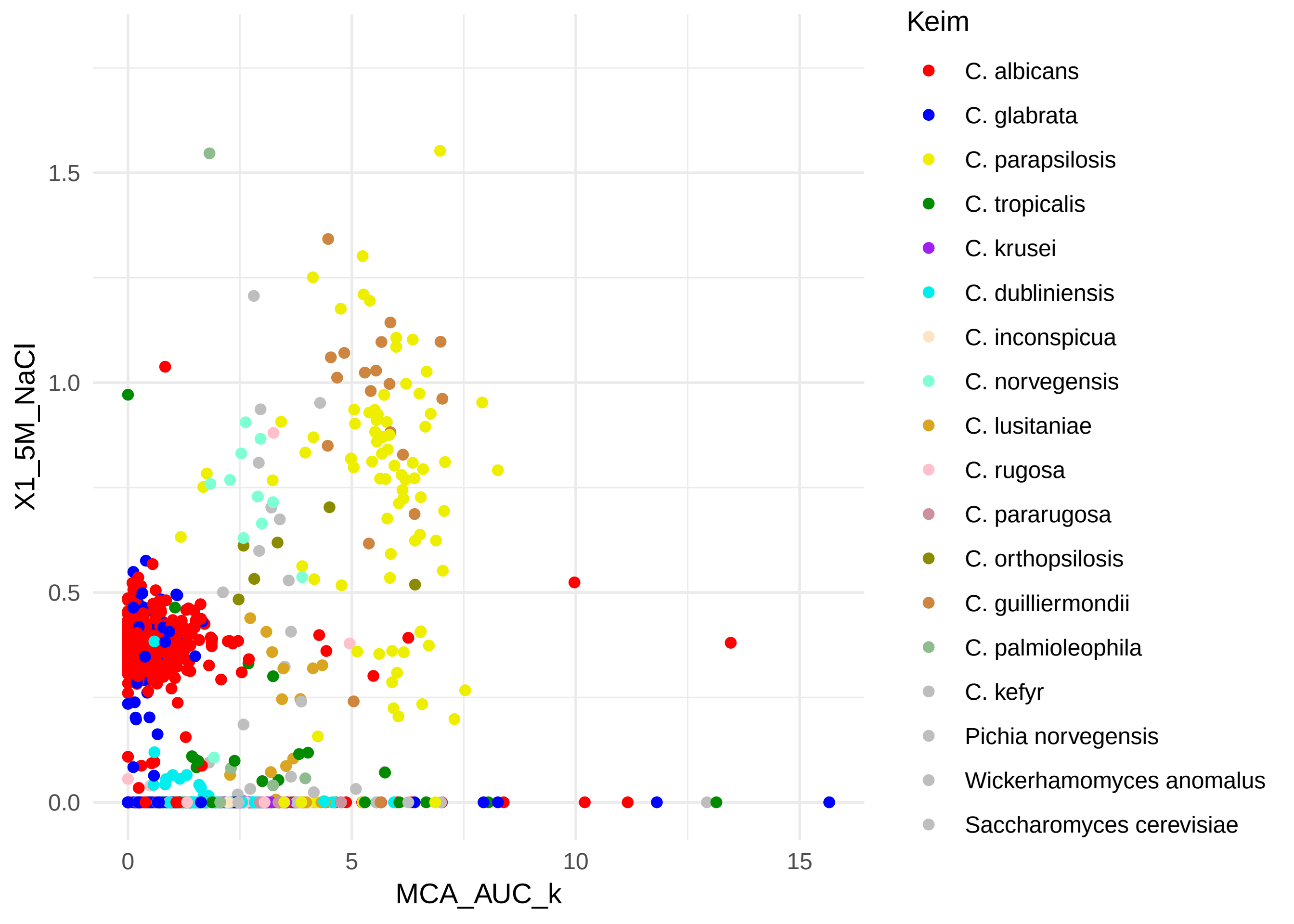
Keim
C. albicans
C. glabrata
C. parapsilosis
C. tropicalis
C. krusei
C. dubliniensis
C. inconspicua
C. norvegensis
C. lusitaniae
C. rugosa
C. pararugosa
C. orthopsilosis
C. guilliermondii
C. palmioleophila
C. kefyr
Pichia norvegensis
Wickerhamomyces anomalus
Saccharomyces cerevisiae
X1_5M_NaCl
1.5
1.0
0.5
0.0
0
5
10
15
MCA_AUC_k

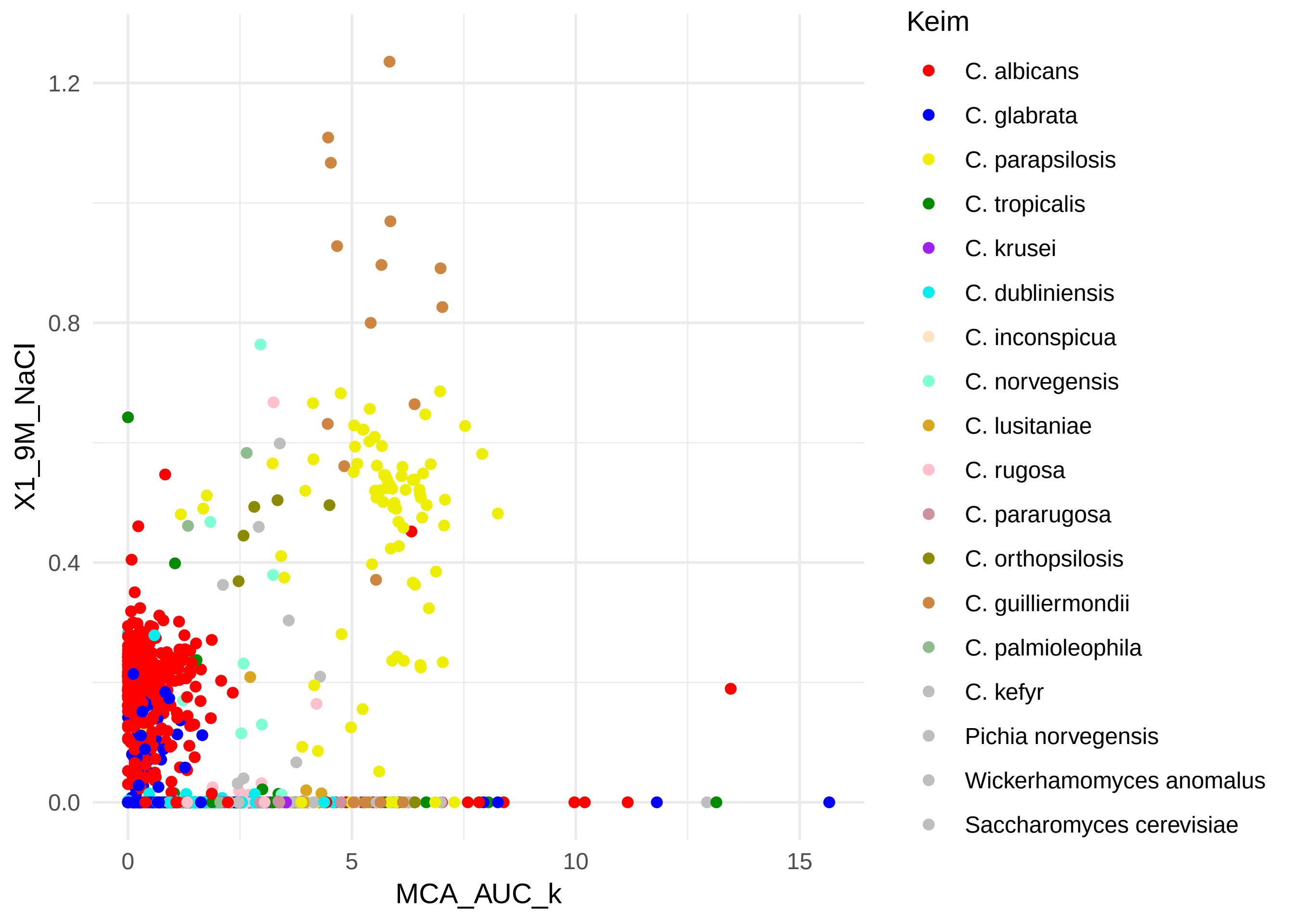
X1_9M_NaCl
1.2
0.8
0.4
0.0
0
5
10
15
MCA_AUC_k
Keim
C. albicans
C. glabrata
C. parapsilosis
C. tropicalis
C. krusei
C. dubliniensis
C. inconspicua
C. norvegensis
C. lusitaniae
C. rugosa
C. pararugosa
C. orthopsilosis
C. guilliermondii
C. palmioleophila
C. kefyr
Pichia norvegensis
Wickerhamomyces anomalus
Saccharomyces cerevisiae

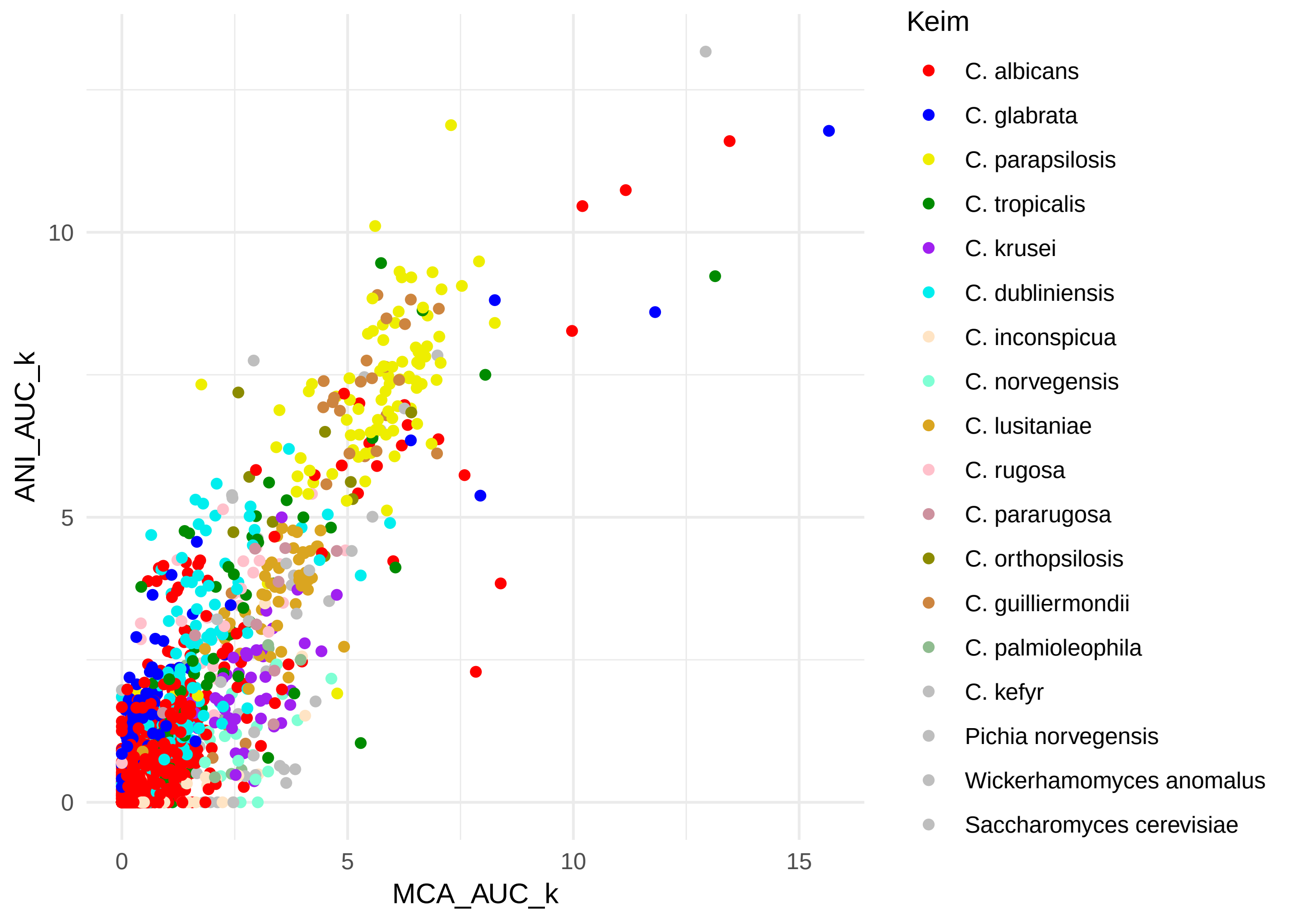
Keim
C. albicans
C. glabrata
C. parapsilosis
C. tropicalis
C. krusei
C. dubliniensis
C. inconspicua
C. norvegensis
C. lusitaniae
C. rugosa
C. pararugosa
C. orthopsilosis
C. guilliermondii
C. palmioleophila
C. kefyr
Pichia norvegensis
Wickerhamomyces anomalus
Saccharomyces cerevisiae
ANI_AUC_k
MCA_AUC_k
0
5
10
15

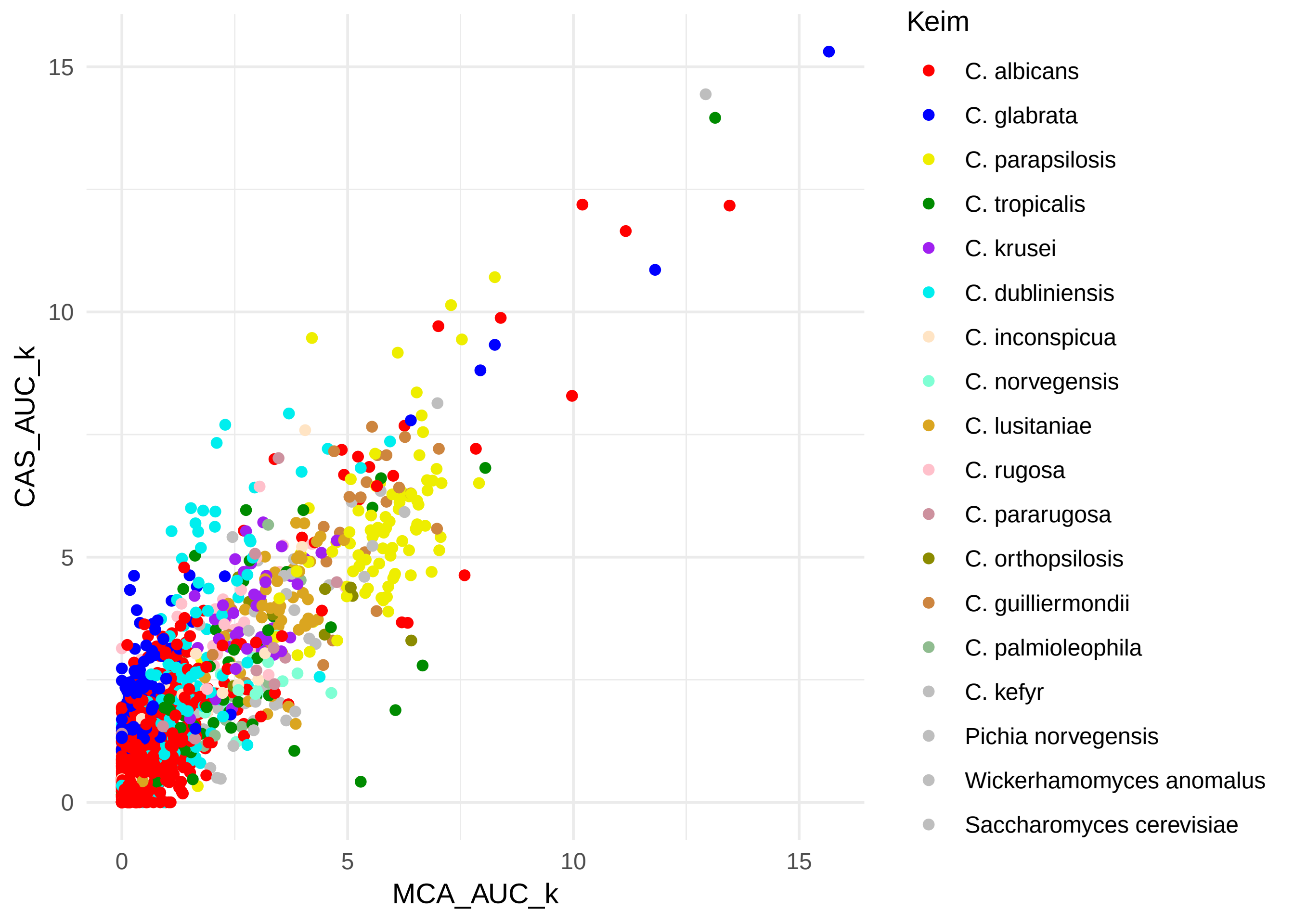

Keim
C. albicans
C. glabrata
C. parapsilosis
C. tropicalis
C. krusei
C. dubliniensis
C. inconspicua
C. norvegensis
C. lusitaniae
C. rugosa
C. pararugosa
C. orthopsilosis
C. guilliermondii
C. palmioleophila
C. kefyr
Pichia norvegensis
Wickerhamomyces anomalus
Saccharomyces cerevisiae
CAS_AUC_k
MCA_AUC_k
0
5
10
15

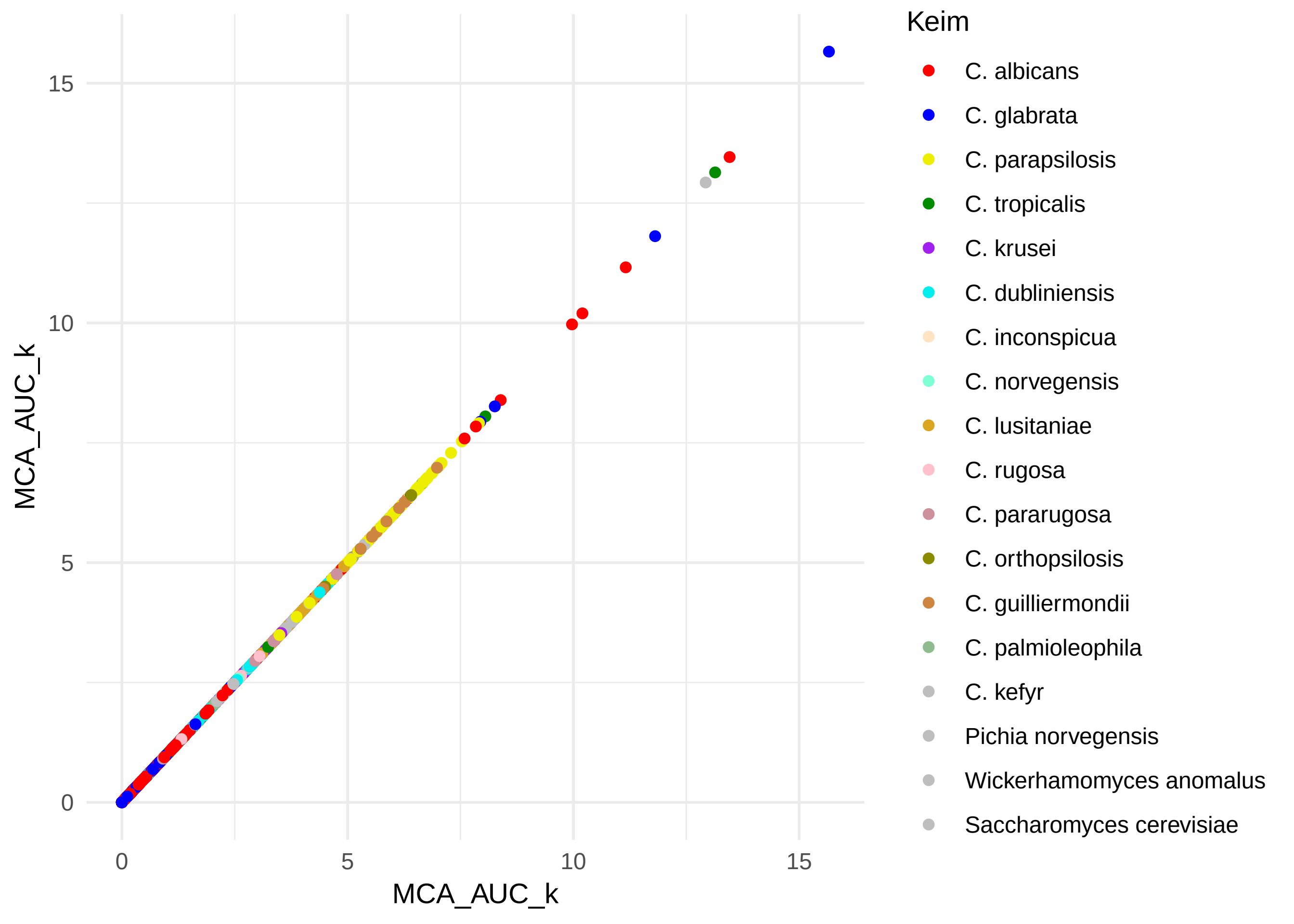

Keim
C. albicans
C. glabrata
C. parapsilosis
C. tropicalis
C. krusei
C. dubliniensis
C. inconspicua
C. norvegensis
C. lusitaniae
C. rugosa
C. pararugosa
C. orthopsilosis
C. guilliermondii
C. palmioleophila
C. kefyr
Pichia norvegensis
Wickerhamomyces anomalus
Saccharomyces cerevisiae
MCA_AUC_k
MCA_AUC_k
0
5
10
15
0
5
10
15

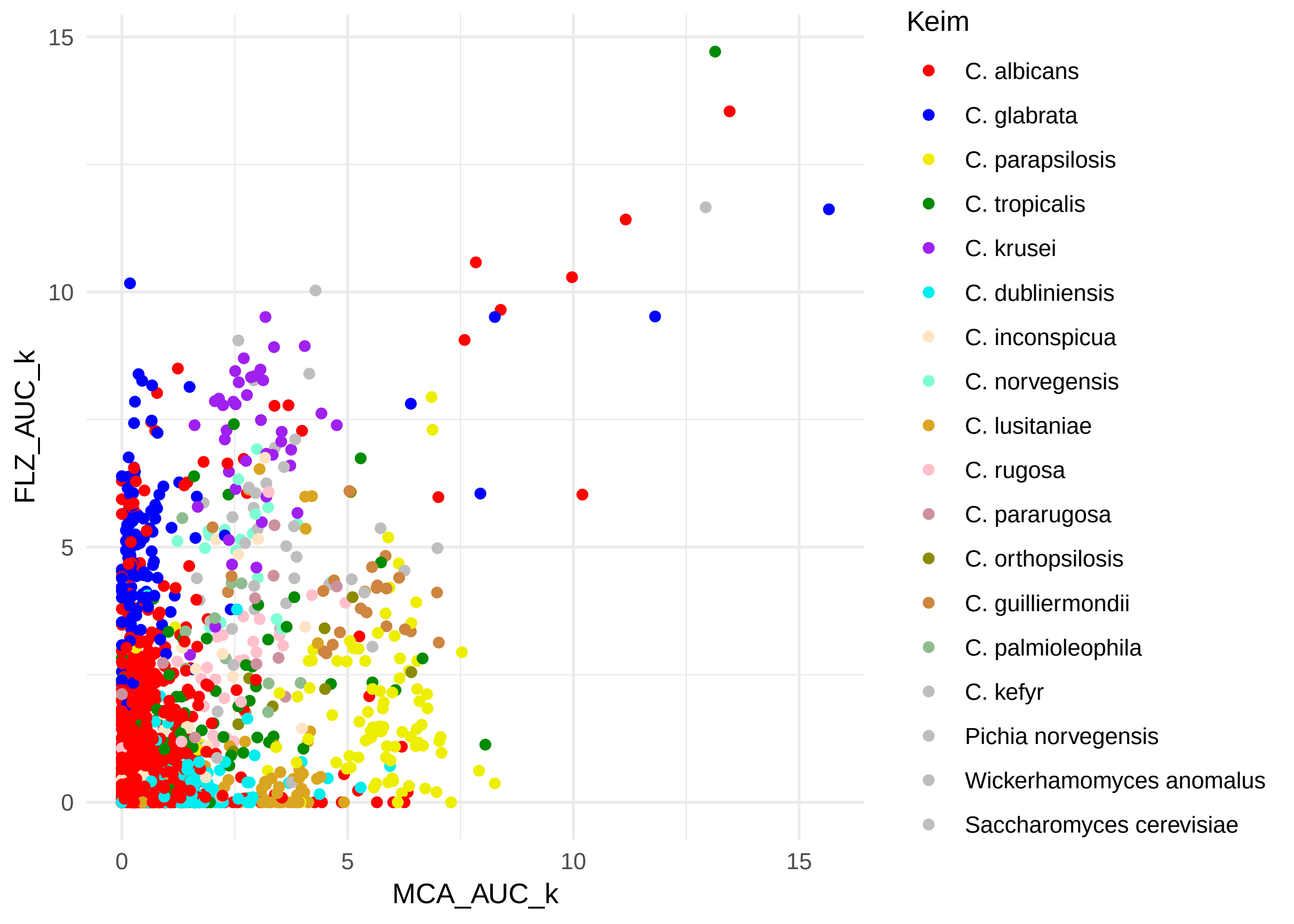

Keim
C. albicans
C. glabrata
C. parapsilosis
C. tropicalis
C. krusei
C. dubliniensis
C. inconspicua
C. norvegensis
C. lusitaniae
C. rugosa
C. pararugosa
C. orthopsilosis
C. guilliermondii
C. palmioleophila
C. kefyr
Pichia norvegensis
Wickerhamomyces anomalus
Saccharomyces cerevisiae
FLZ_AUC_k
MCA_AUC_k
0
5
10
15

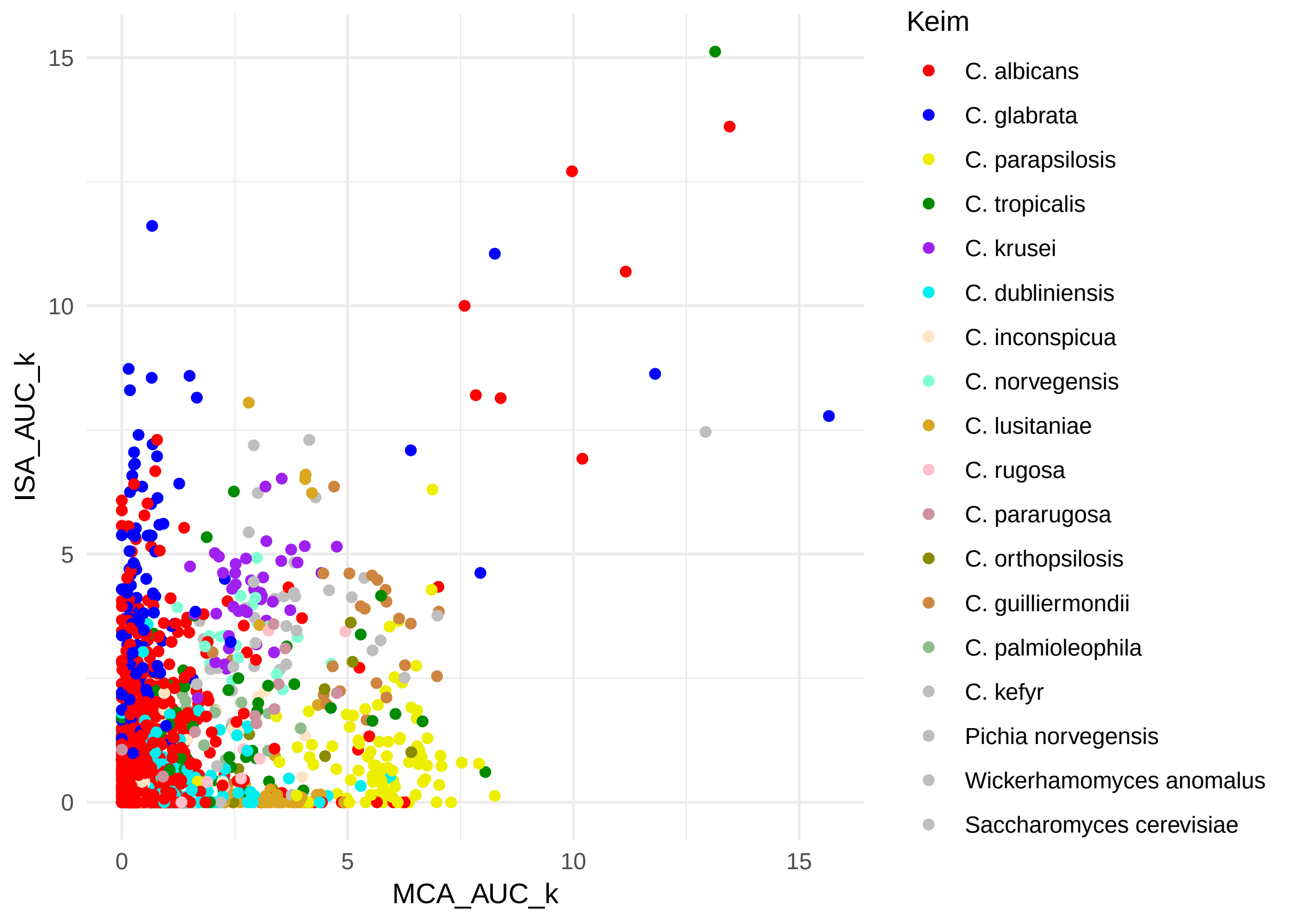

Keim
C. albicans
C. glabrata
C. parapsilosis
C. tropicalis
C. krusei
C. dubliniensis
C. inconspicua
C. norvegensis
C. lusitaniae
C. rugosa
C. pararugosa
C. orthopsilosis
C. guilliermondii
C. palmioleophila
C. kefyr
Pichia norvegensis
Wickerhamomyces anomalus
Saccharomyces cerevisiae
ISA_AUC_k
MCA_AUC_k
0
5
10
15

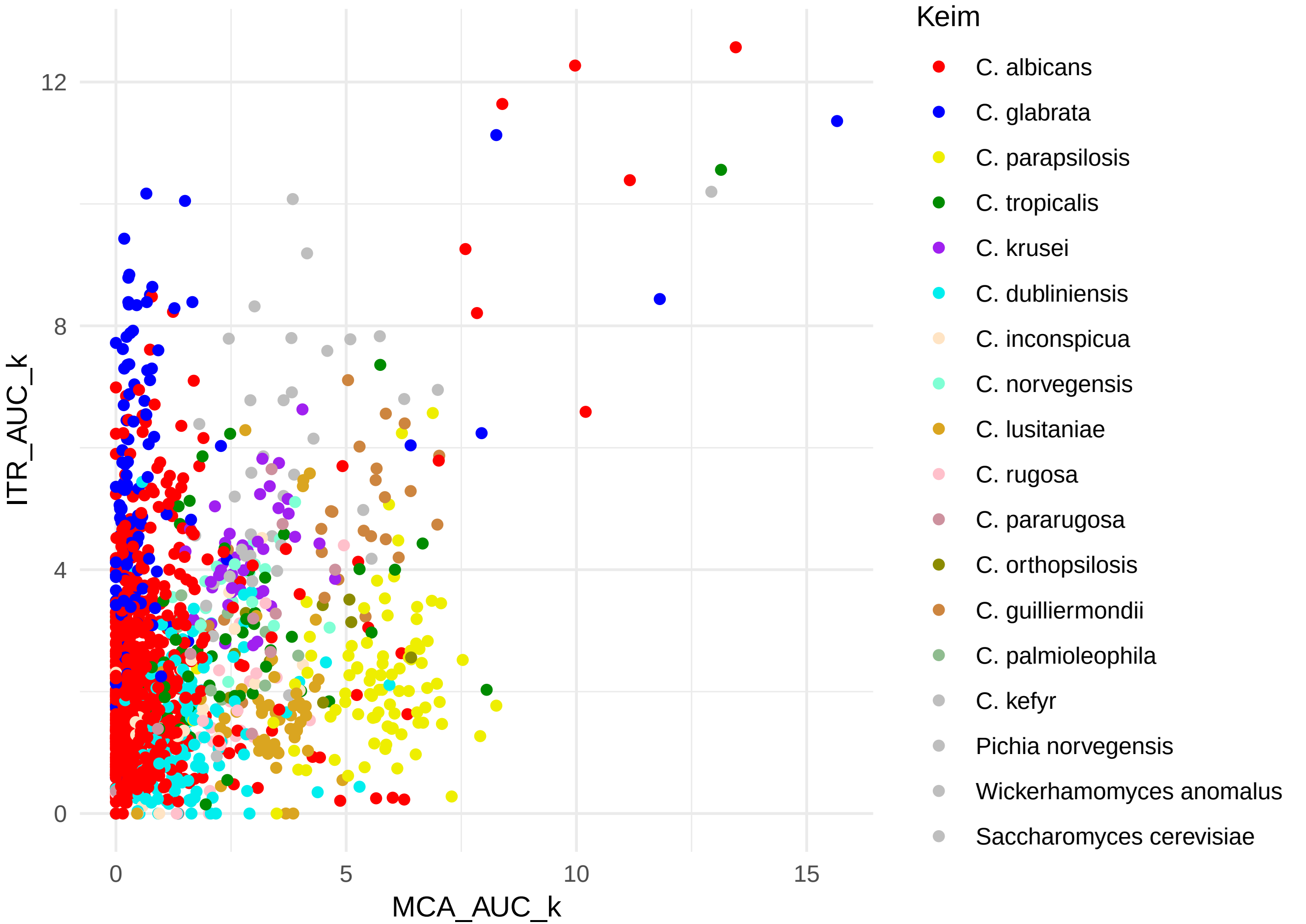

Keim
C. albicans
C. glabrata
C. parapsilosis
C. tropicalis
C. krusei
C. dubliniensis
C. inconspicua
C. norvegensis
C. lusitaniae
C. rugosa
C. pararugosa
C. orthopsilosis
C. guilliermondii
C. palmioleophila
C. kefyr
Pichia norvegensis
Wickerhamomyces anomalus
Saccharomyces cerevisiae
ITR_AUC_k
MCA_AUC_k
0
4
8
12
0
5
10
15

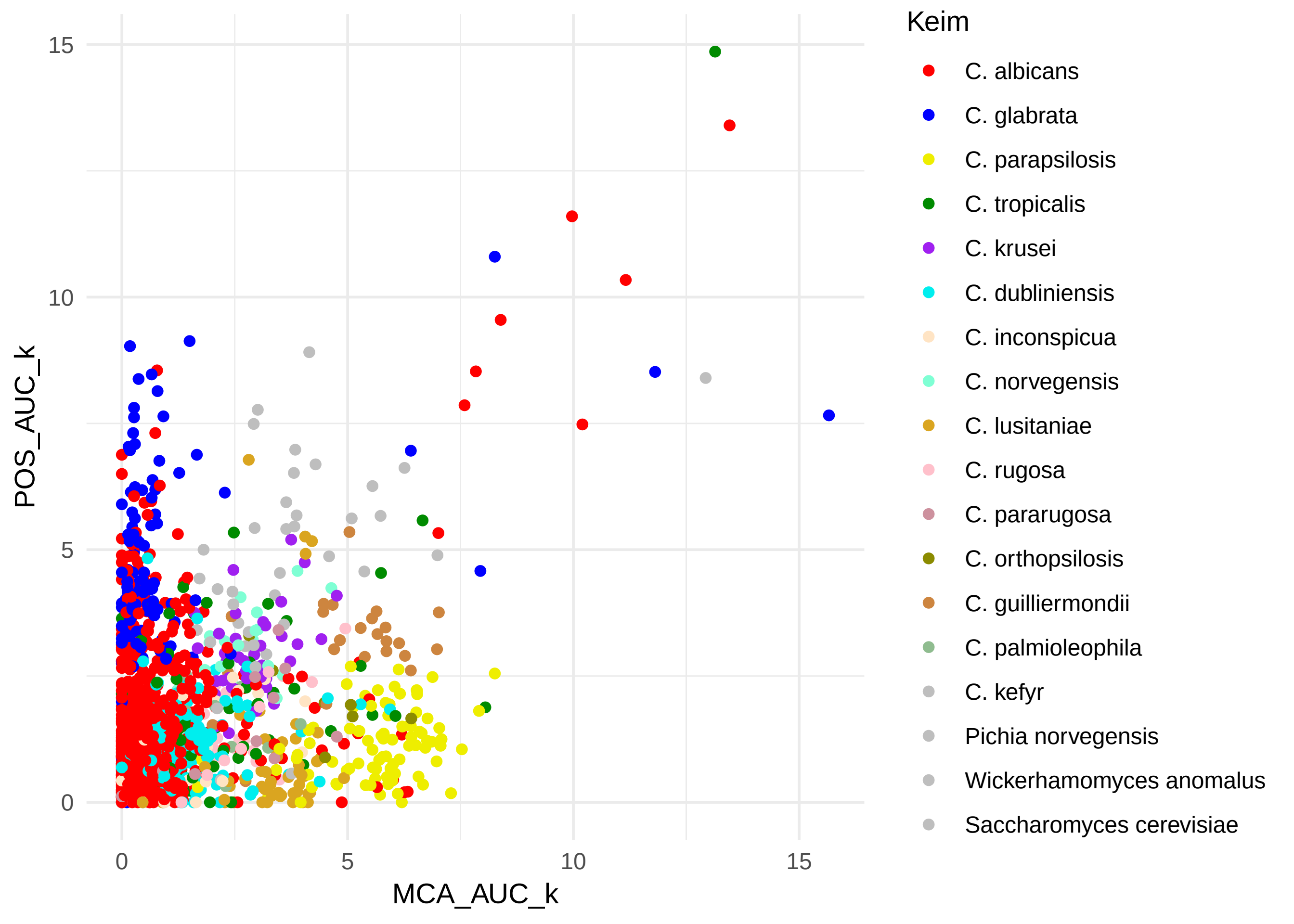
Keim
C. albicans
C. glabrata
C. parapsilosis
C. tropicalis
C. krusei
C. dubliniensis
C. inconspicua
C. norvegensis
C. lusitaniae
C. rugosa
C. pararugosa
C. orthopsilosis
C. guilliermondii
C. palmioleophila
C. kefyr
Pichia norvegensis
Wickerhamomyces anomalus
Saccharomyces cerevisiae
POS_AUC_k
MCA_AUC_k
0
5
10
15
0
5
10
15

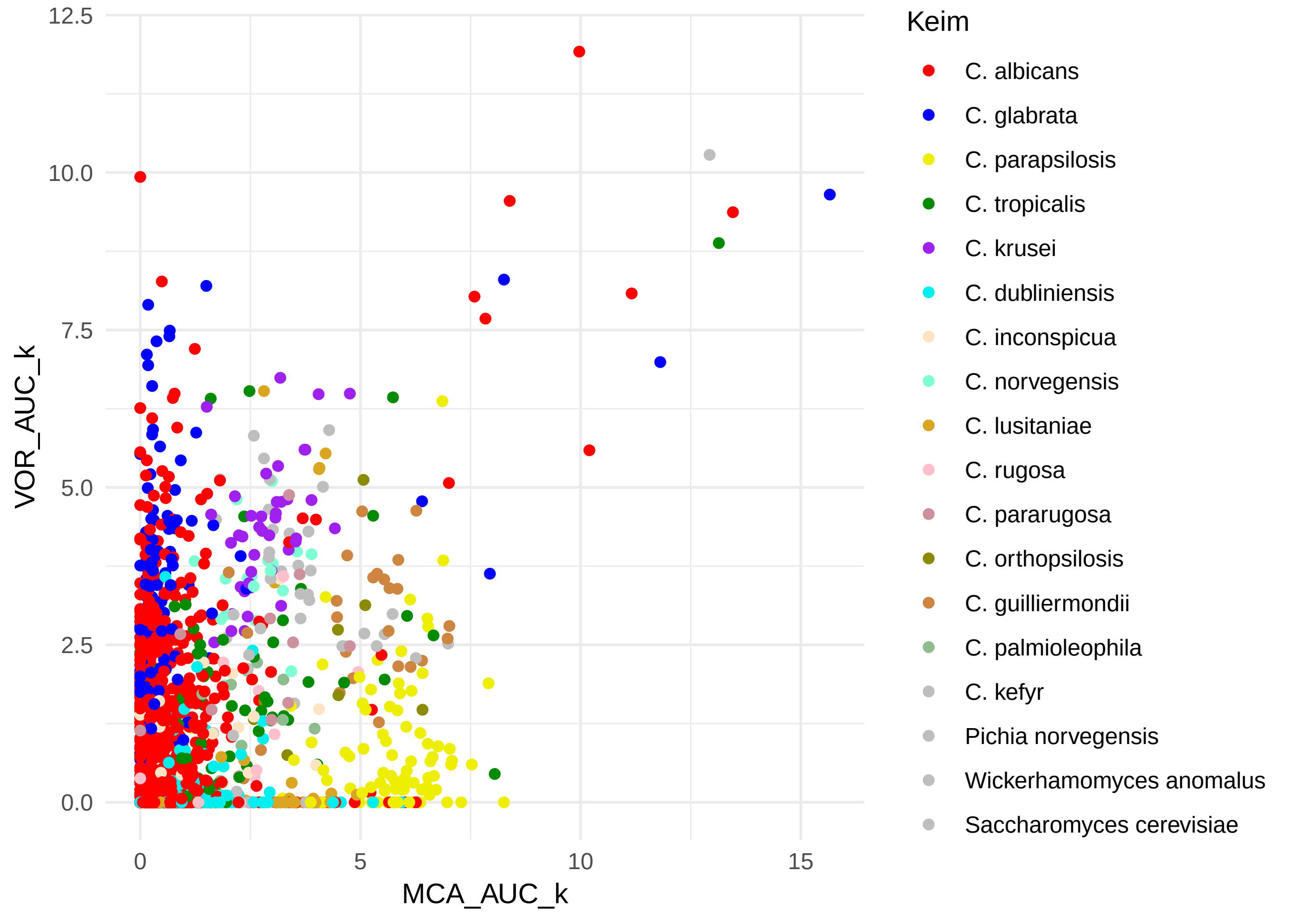

12.5
10.0
7.5
5.0
2.5
0.0
VOR_AUC_k
0
5
10
15
MCA_AUC_k
Keim
C. albicans
C. glabrata
C. parapsilosis
C. tropicalis
C. krusei
C. dubliniensis
C. inconspicua
C. norvegensis
C. lusitaniae
C. rugosa
C. pararugosa
C. orthopsilosis
C. guilliermondii
C. palmioleophila
C. kefyr
Pichia norvegensis
Wickerhamomyces anomalus
Saccharomyces cerevisiae

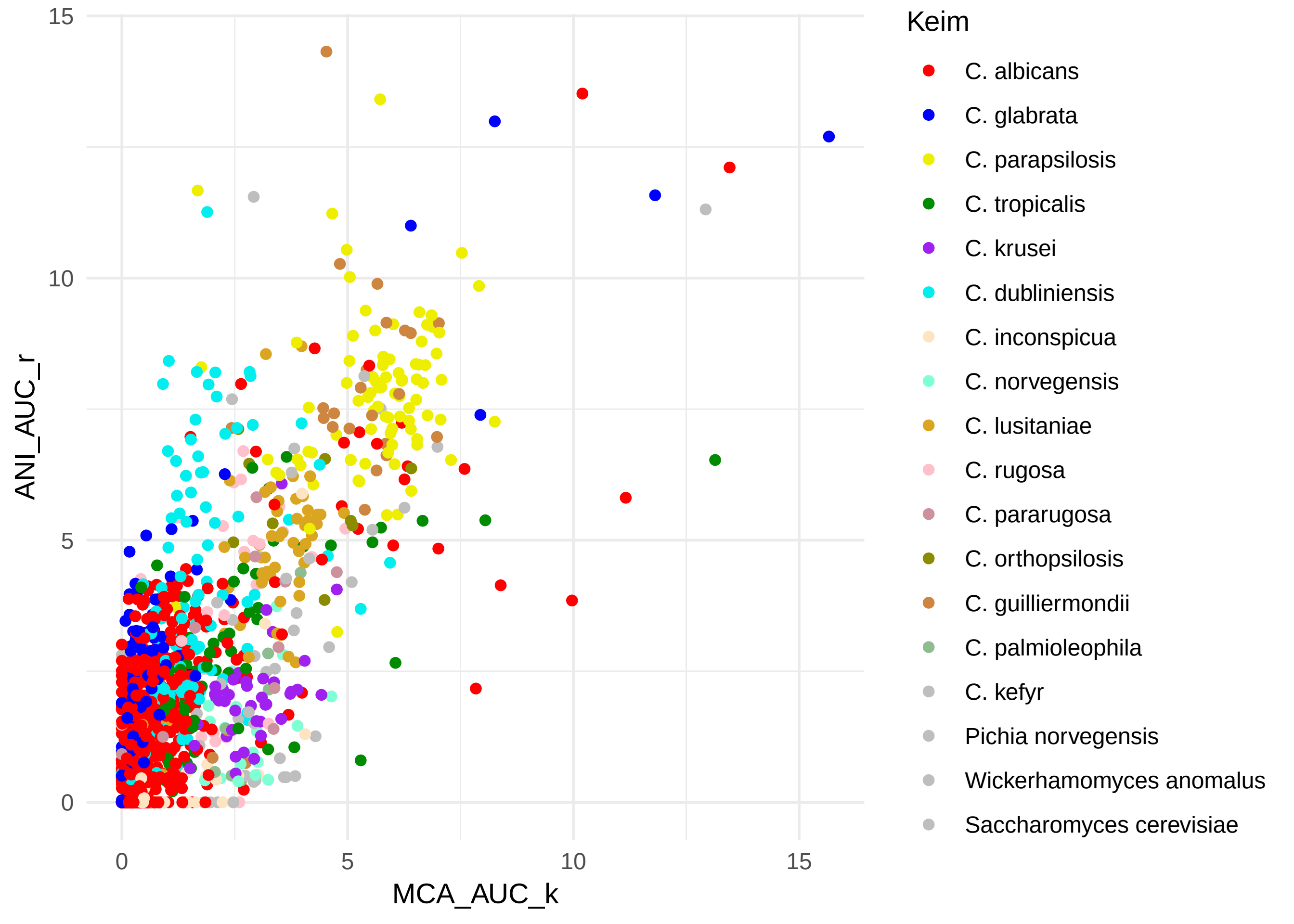
Keim
C. albicans
C. glabrata
C. parapsilosis
C. tropicalis
C. krusei
C. dubliniensis
C. inconspicua
C. norvegensis
C. lusitaniae
C. rugosa
C. pararugosa
C. orthopsilosis
C. guilliermondii
C. palmioleophila
C. kefyr
Pichia norvegensis
Wickerhamomyces anomalus
Saccharomyces cerevisiae
ANI_AUC_r
MCA_AUC_k
0
5
10
15
0
5
10
15

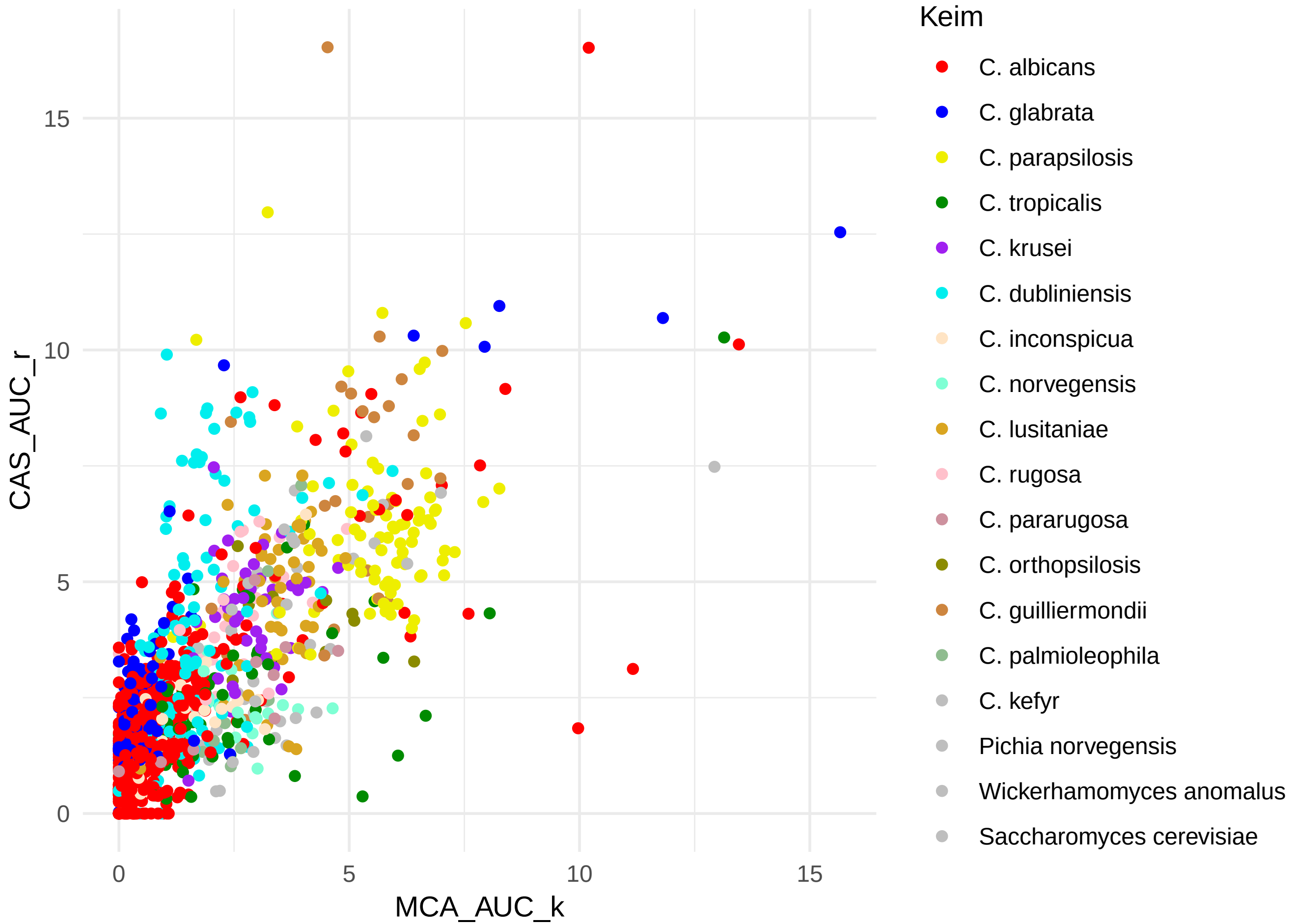
Keim
C. albicans
C. glabrata
C. parapsilosis
C. tropicalis
C. krusei
C. dubliniensis
C. inconspicua
C. norvegensis
C. lusitaniae
C. rugosa
C. pararugosa
C. orthopsilosis
C. guilliermondii
C. palmioleophila
C. kefyr
Pichia norvegensis
Wickerhamomyces anomalus
Saccharomyces cerevisiae
CAS_AUC_r
MCA_AUC_k
0
5
10
15
0
5
10
15

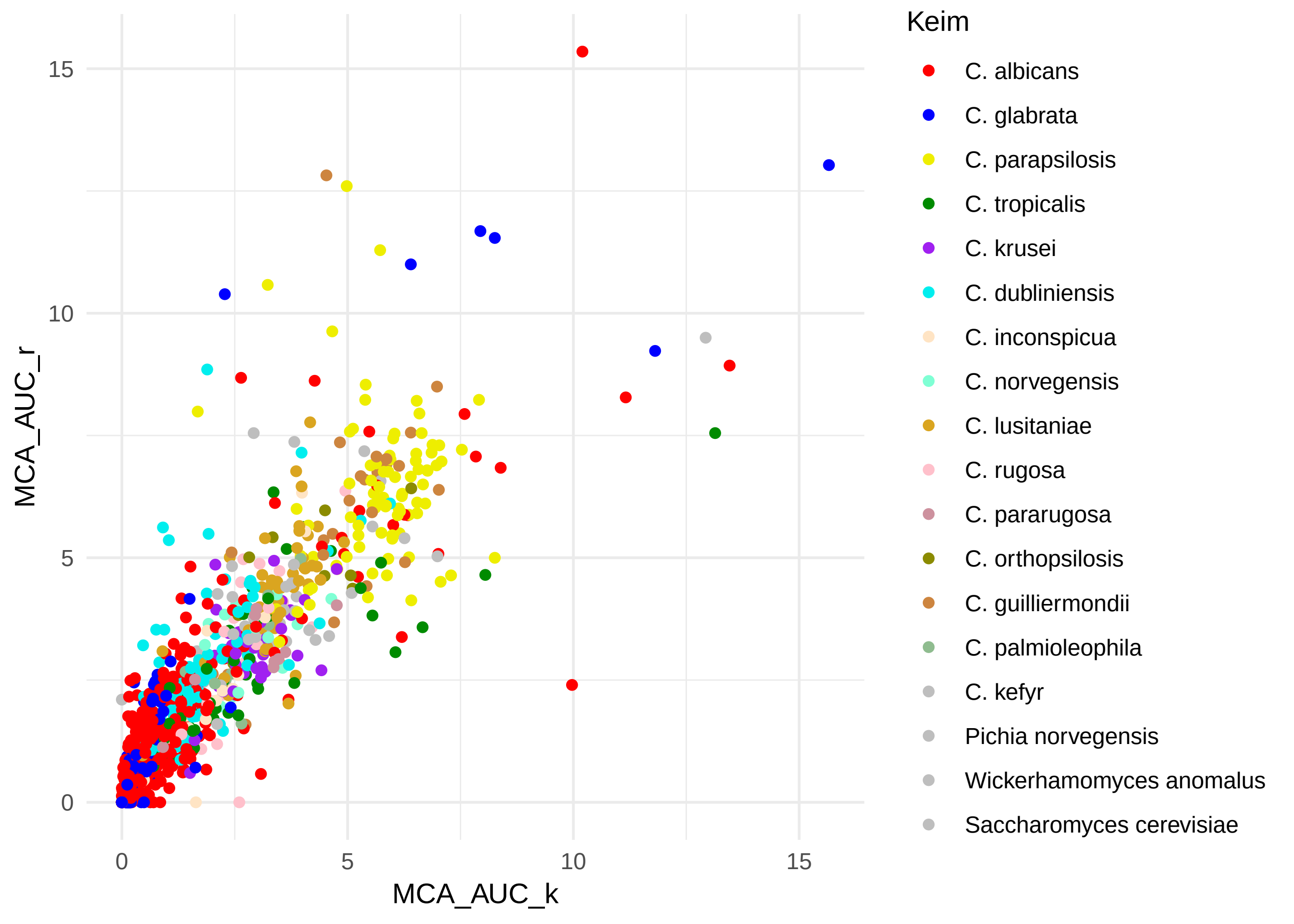
Keim
C. albicans
C. glabrata
C. parapsilosis
C. tropicalis
C. krusei
C. dubliniensis
C. inconspicua
C. norvegensis
C. lusitaniae
C. rugosa
C. pararugosa
C. orthopsilosis
C. guilliermondii
C. palmioleophila
C. kefyr
Pichia norvegensis
Wickerhamomyces anomalus
Saccharomyces cerevisiae
MCA_AUC_r
MCA_AUC_k
0
5
10
15

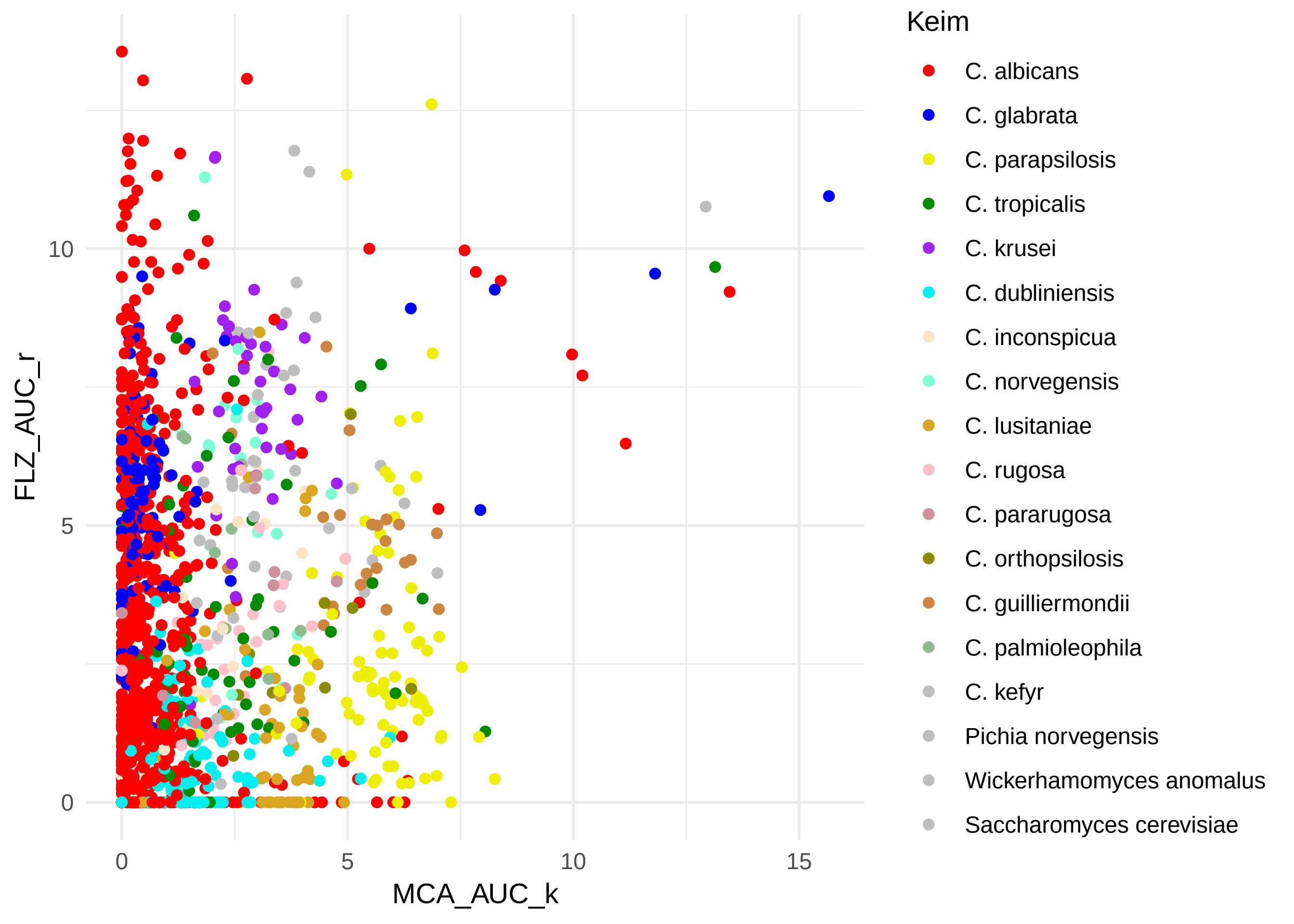

FLZ_AUC_r
MCA_AUC_k
0
5
10
15
Keim
C. albicans
C. glabrata
C. parapsilosis
C. tropicalis
C. krusei
C. dubliniensis
C. inconspicua
C. norvegensis
C. lusitaniae
C. rugosa
C. pararugosa
C. orthopsilosis
C. guilliermondii
C. palmioleophila
C. kefyr
Pichia norvegensis
Wickerhamomyces anomalus
Saccharomyces cerevisiae

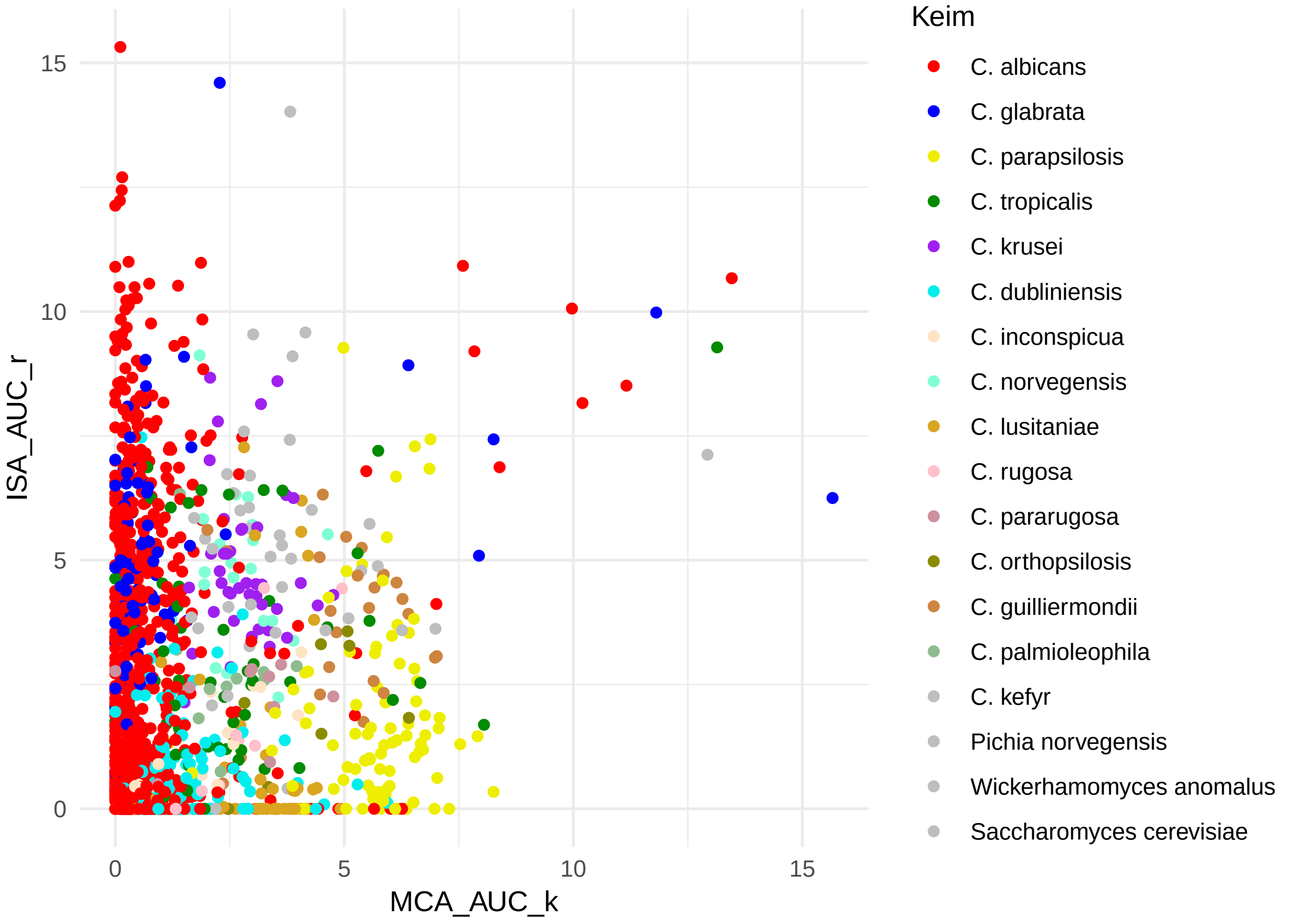

ISA_AUC_r
15
10
5
0
MCA_AUC_k
0
5
10
15
Keim
C. albicans
C. glabrata
C. parapsilosis
C. tropicalis
C. krusei
C. dubliniensis
C. inconspicua
C. norvegensis
C. lusitaniae
C. rugosa
C. pararugosa
C. orthopsilosis
C. guilliermondii
C. palmioleophila
C. kefyr
Pichia norvegensis
Wickerhamomyces anomalus
Saccharomyces cerevisiae

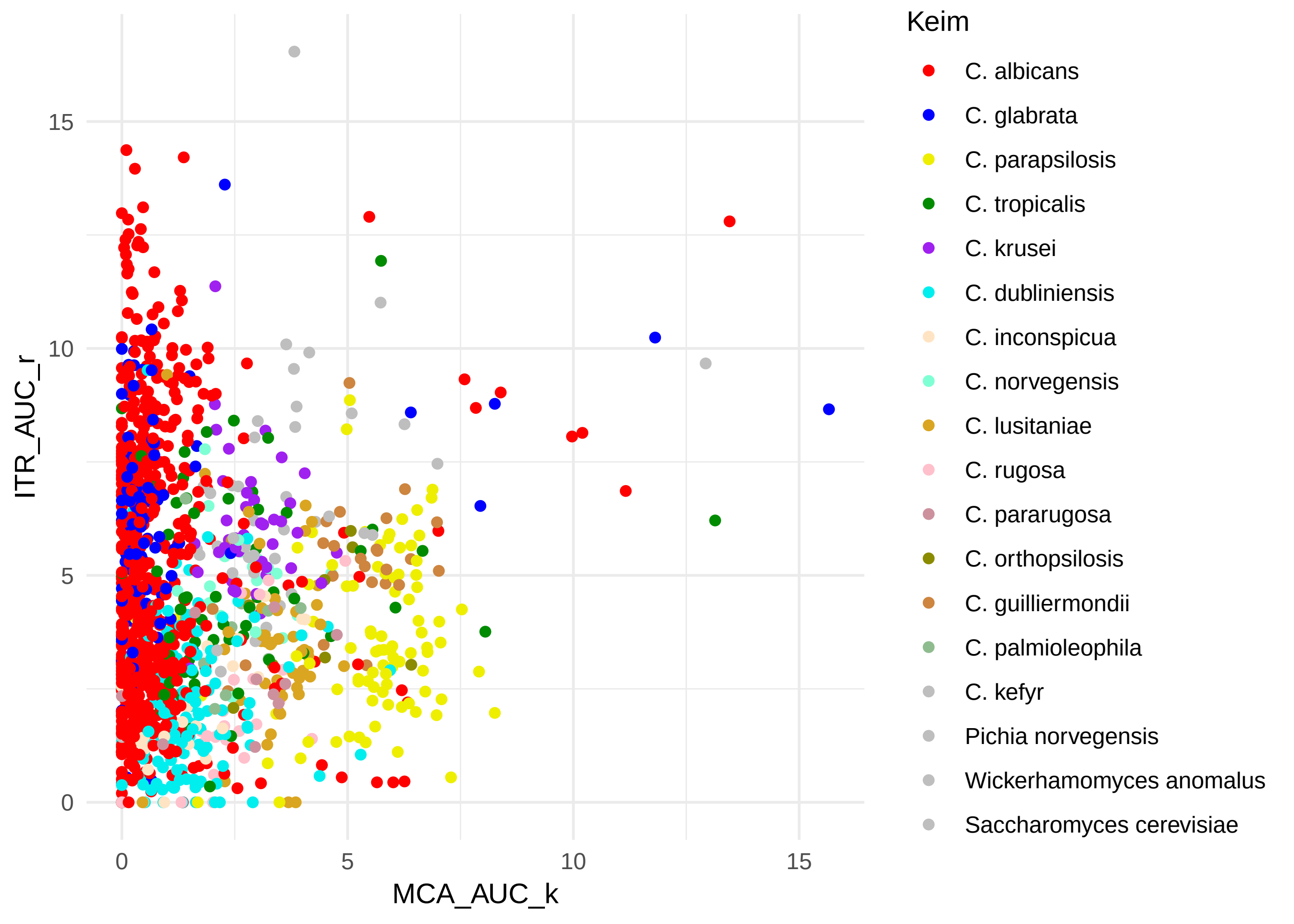

Keim
C. albicans
C. glabrata
C. parapsilosis
C. tropicalis
C. krusei
C. dubliniensis
C. inconspicua
C. norvegensis
C. lusitaniae
C. rugosa
C. pararugosa
C. orthopsilosis
C. guilliermondii
C. palmioleophila
C. kefyr
Pichia norvegensis
Wickerhamomyces anomalus
Saccharomyces cerevisiae
ITR_AUC_r
MCA_AUC_k
0
5
10
15

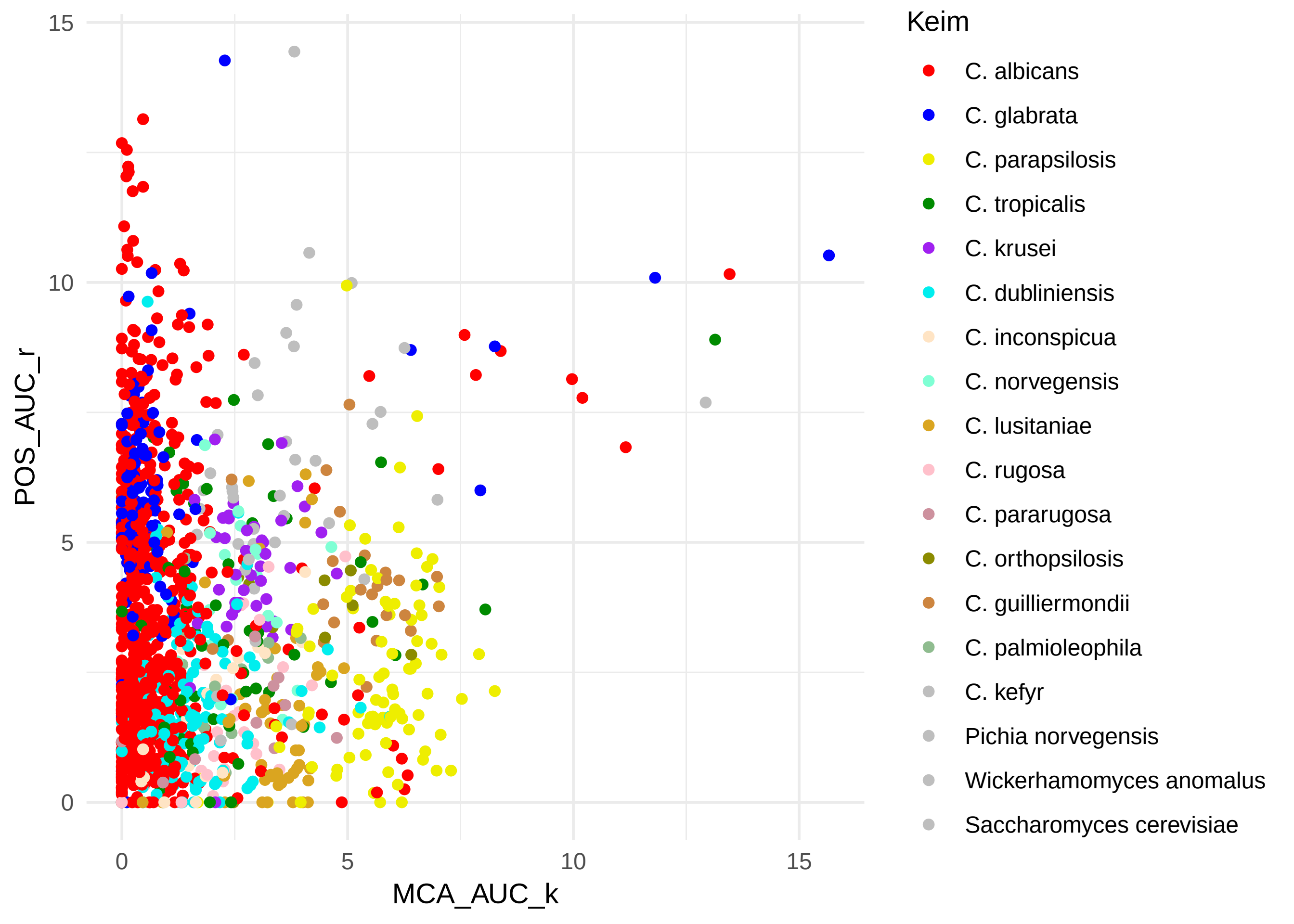

15
10
5
0
POS_AUC_r
0
5
10
15
MCA_AUC_k
Keim
C. albicans
C. glabrata
C. parapsilosis
C. tropicalis
C. krusei
C. dubliniensis
C. inconspicua
C. norvegensis
C. lusitaniae
C. rugosa
C. pararugosa
C. orthopsilosis
C. guilliermondii
C. palmioleophila
C. kefyr
Pichia norvegensis
Wickerhamomyces anomalus
Saccharomyces cerevisiae

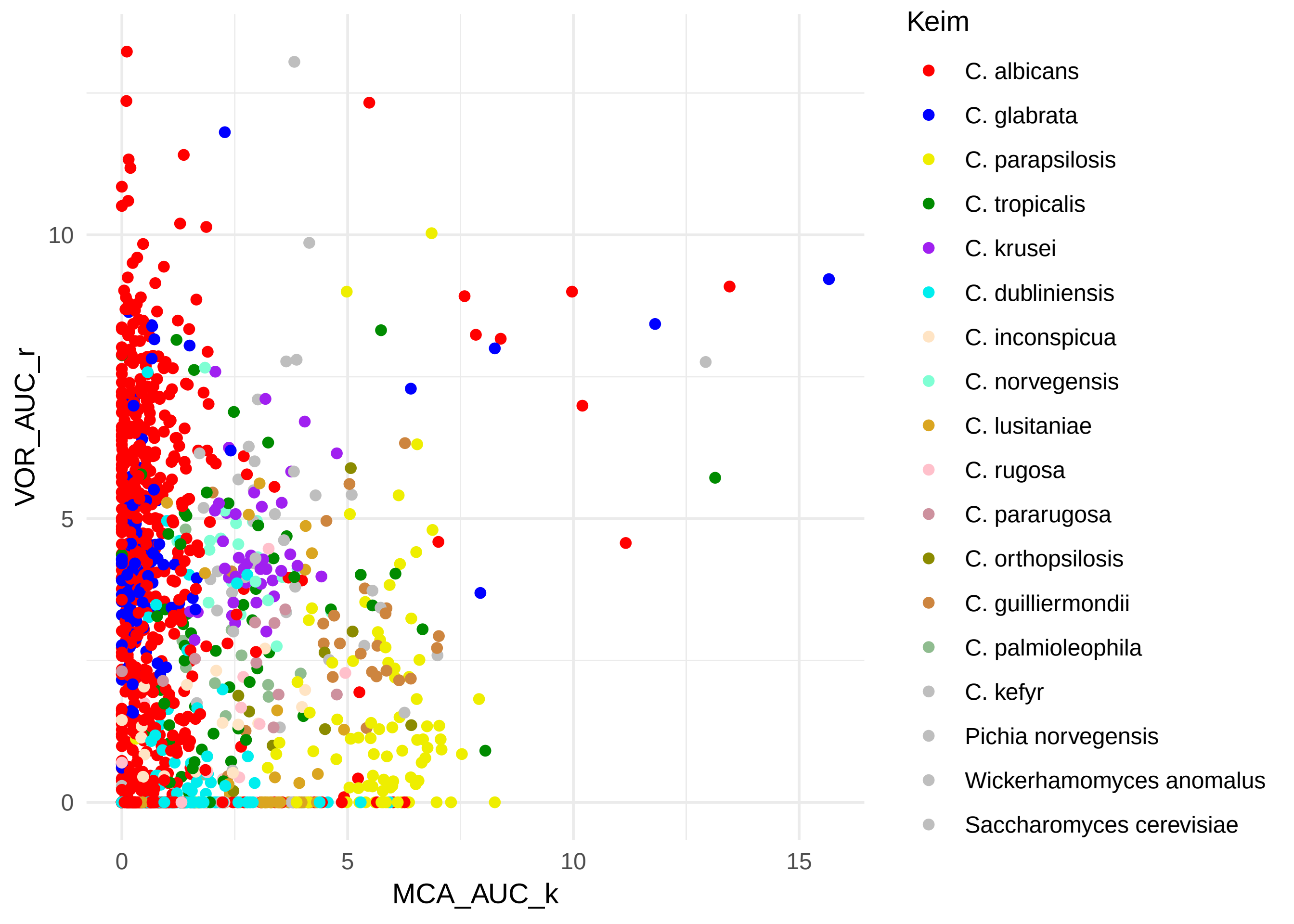
VOR_AUC_r
MCA_AUC_k
0
5
10
15
Keim
C. albicans
C. glabrata
C. parapsilosis
C. tropicalis
C. krusei
C. dubliniensis
C. inconspicua
C. norvegensis
C. lusitaniae
C. rugosa
C. pararugosa
C. orthopsilosis
C. guilliermondii
C. palmioleophila
C. kefyr
Pichia norvegensis
Wickerhamomyces anomalus
Saccharomyces cerevisiae

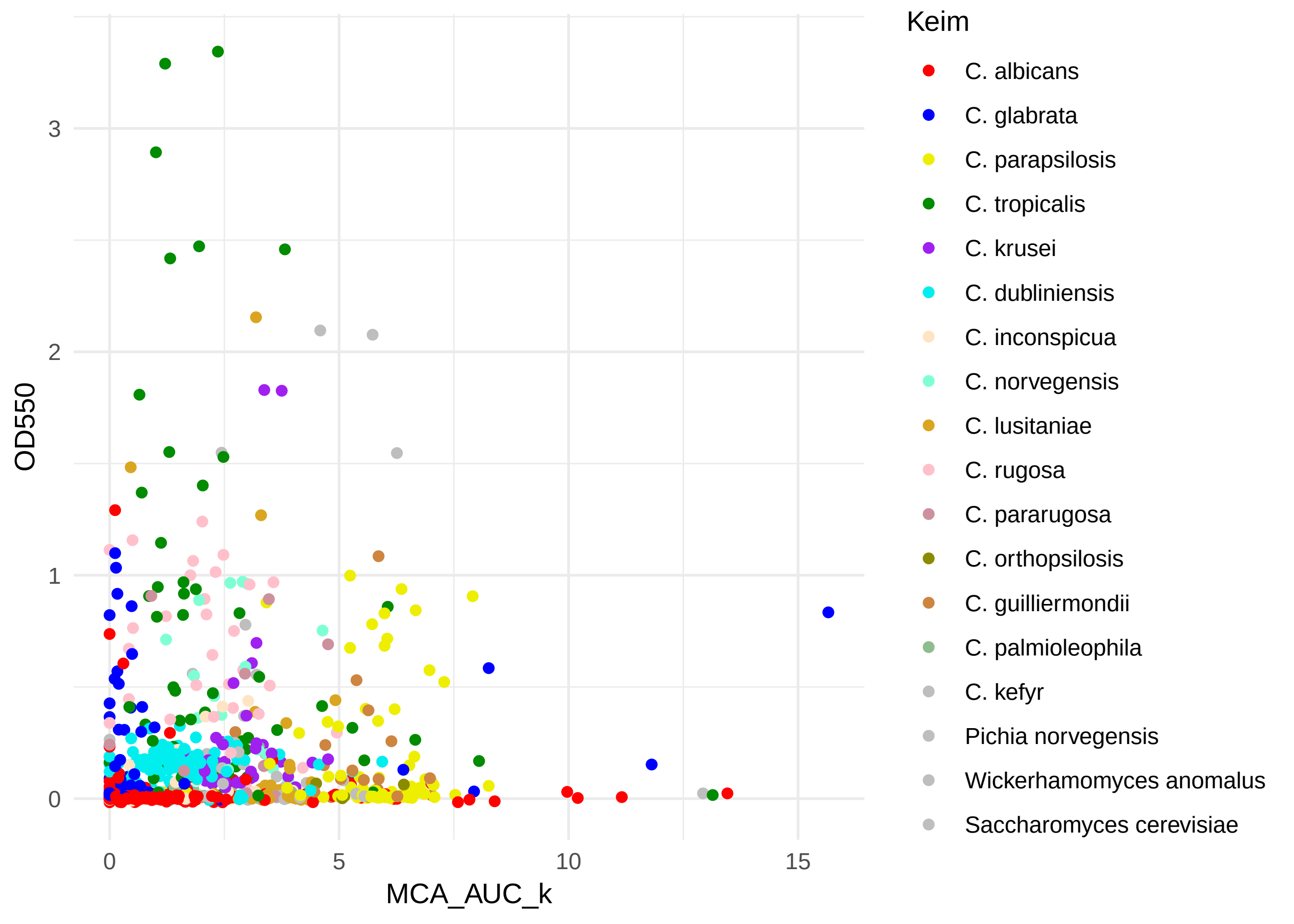
Keim
C. albicans
C. glabrata
C. parapsilosis
C. tropicalis
C. krusei
C. dubliniensis
C. inconspicua
C. norvegensis
C. lusitaniae
C. rugosa
C. pararugosa
C. orthopsilosis
C. guilliermondii
C. palmioleophila
C. kefyr
Pichia norvegensis
Wickerhamomyces anomalus
Saccharomyces cerevisiae
OD550
3
2
1
0
0
5
10
15
MCA_AUC_k

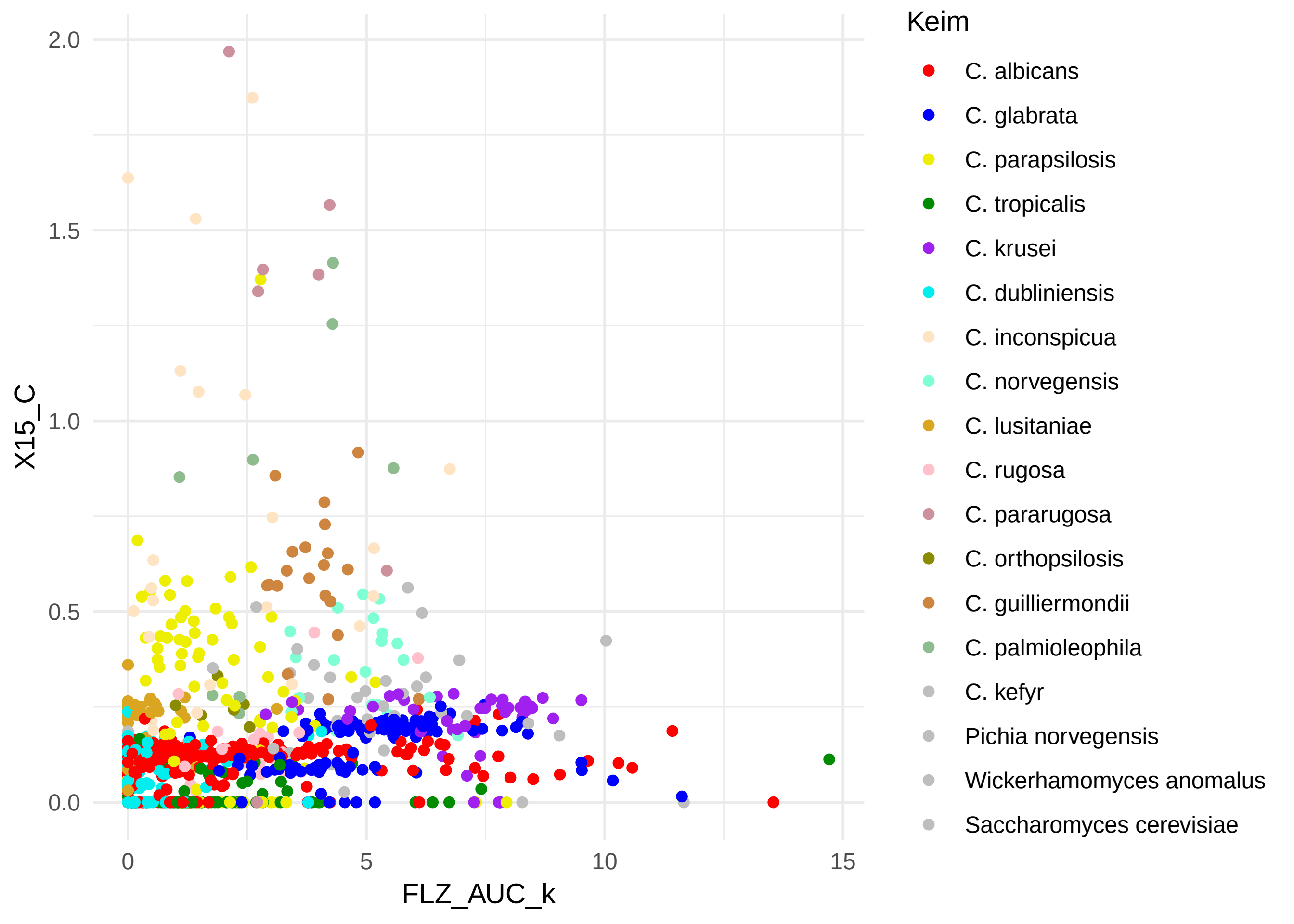

Keim
C. albicans
C. glabrata
C. parapsilosis
C. tropicalis
C. krusei
C. dubliniensis
C. inconspicua
C. norvegensis
C. lusitaniae
C. rugosa
C. pararugosa
C. orthopsilosis
C. guilliermondii
C. palmioleophila
C. kefyr
Pichia norvegensis
Wickerhamomyces anomalus
Saccharomyces cerevisiae
X15_C
2.0
1.5
1.0
0.5
0.0
0
5
10
15
FLZ_AUC_k

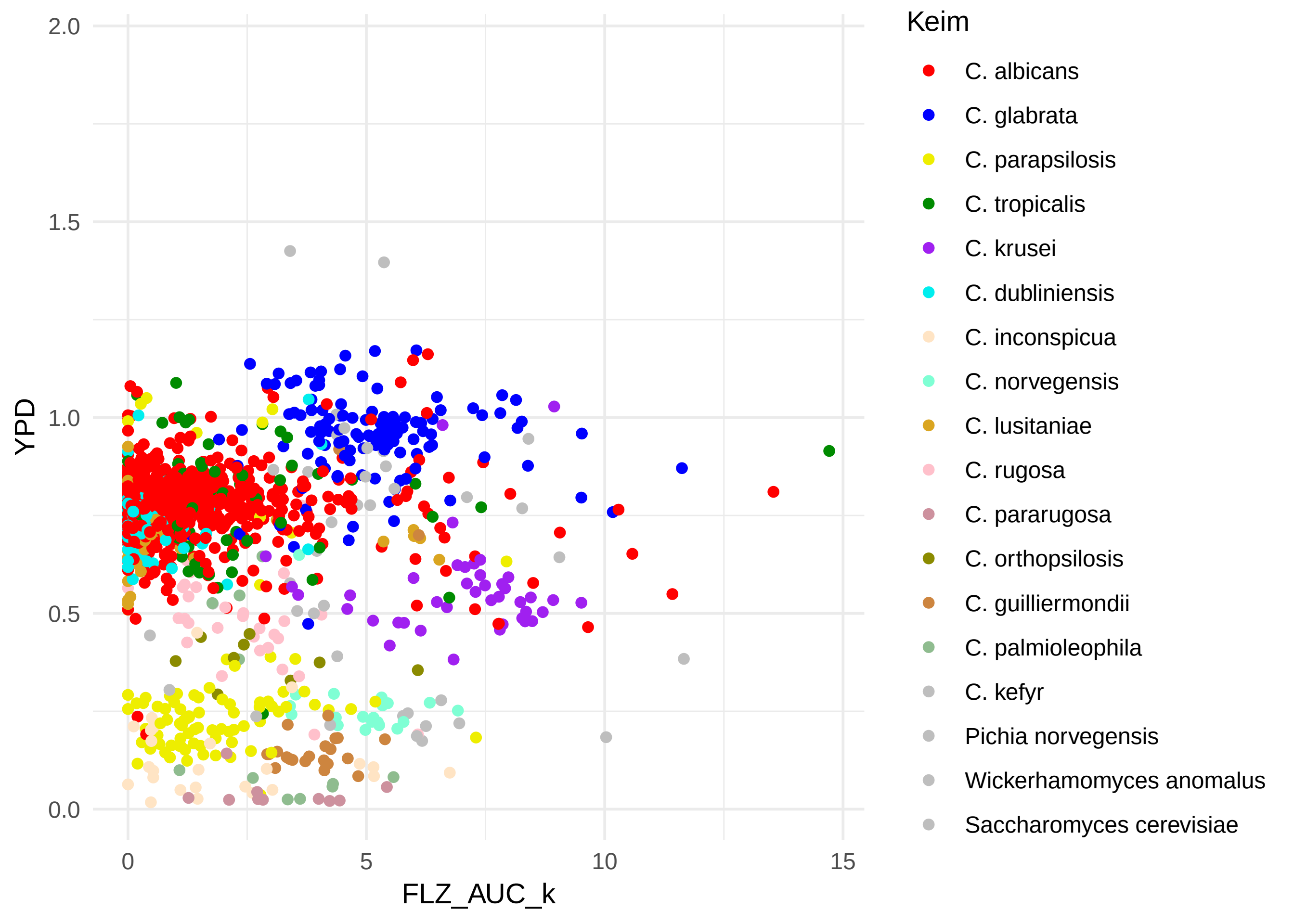
Keim
C. albicans
C. glabrata
C. parapsilosis
C. tropicalis
C. krusei
C. dubliniensis
C. inconspicua
C. norvegensis
C. lusitaniae
C. rugosa
C. pararugosa
C. orthopsilosis
C. guilliermondii
C. palmioleophila
C. kefyr
Pichia norvegensis
Wickerhamomyces anomalus
Saccharomyces cerevisiae
YPD
2.0
1.5
1.0
0.5
0.0
FLZ_AUC_k
0
5
10
15

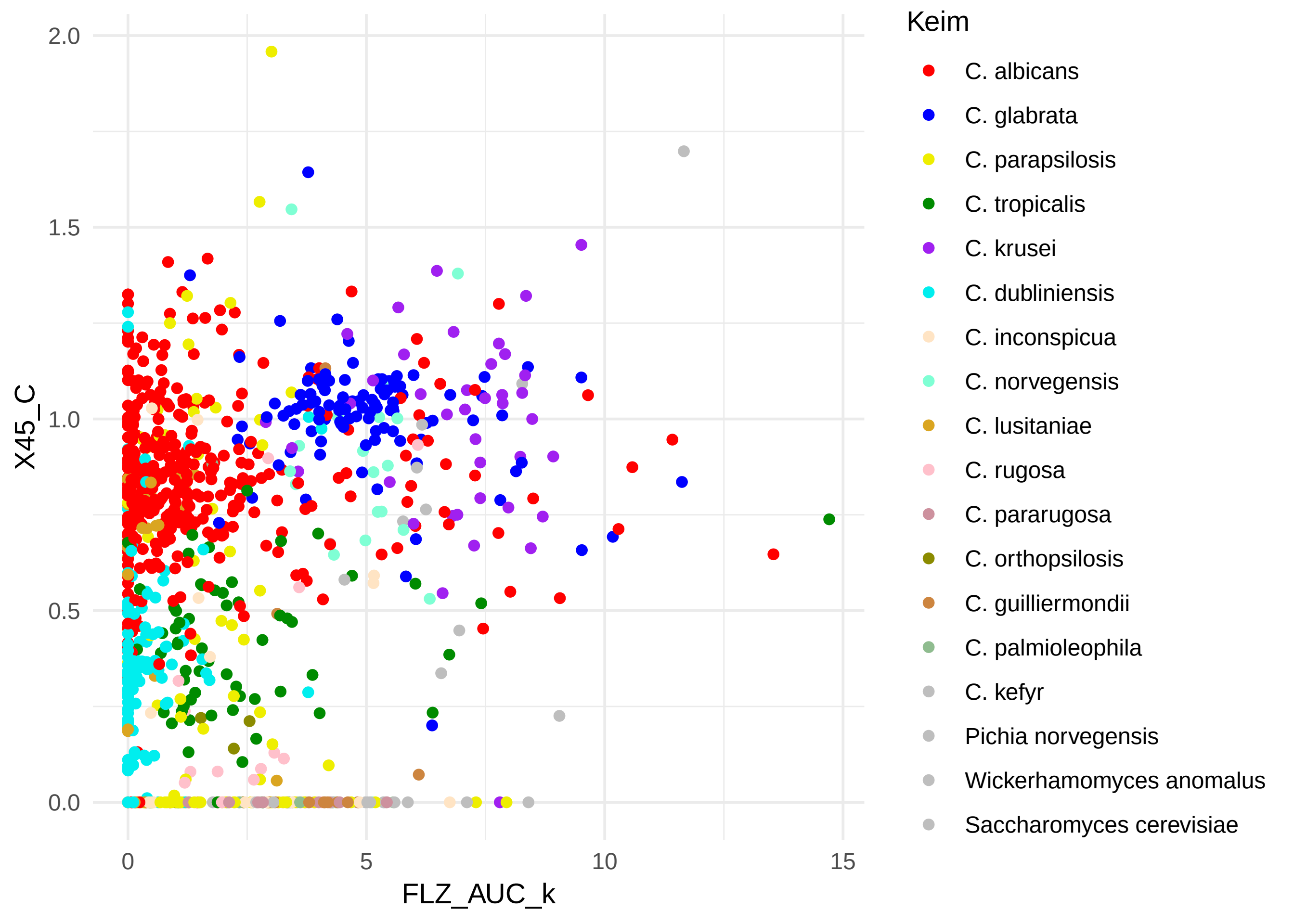

Keim
C. albicans
C. glabrata
C. parapsilosis
C. tropicalis
C. krusei
C. dubliniensis
C. inconspicua
C. norvegensis
C. lusitaniae
C. rugosa
C. pararugosa
C. orthopsilosis
C. guilliermondii
C. palmioleophila
C. kefyr
Pichia norvegensis
Wickerhamomyces anomalus
Saccharomyces cerevisiae
X45_C
FLZ_AUC_k
2.0
1.5
1.0
0.5
0.0
0
5
10
15

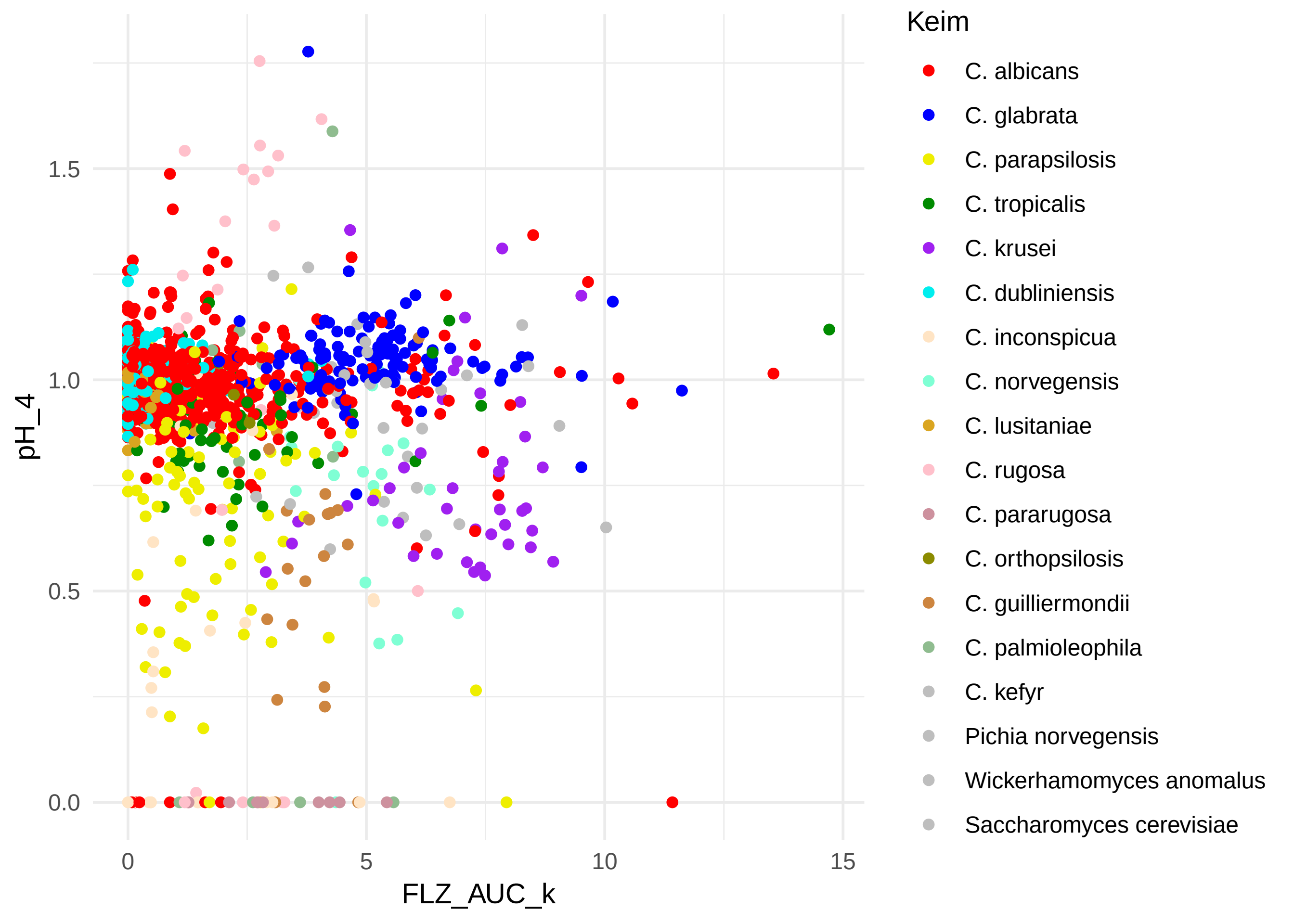
pH_4
FLZ_AUC_k
0.0
0.5
1.0
1.5
0
5
10
15
Keim
C. albicans
C. glabrata
C. parapsilosis
C. tropicalis
C. krusei
C. dubliniensis
C. inconspicua
C. norvegensis
C. lusitaniae
C. rugosa
C. pararugosa
C. orthopsilosis
C. guilliermondii
C. palmioleophila
C. kefyr
Pichia norvegensis
Wickerhamomyces anomalus
Saccharomyces cerevisiae

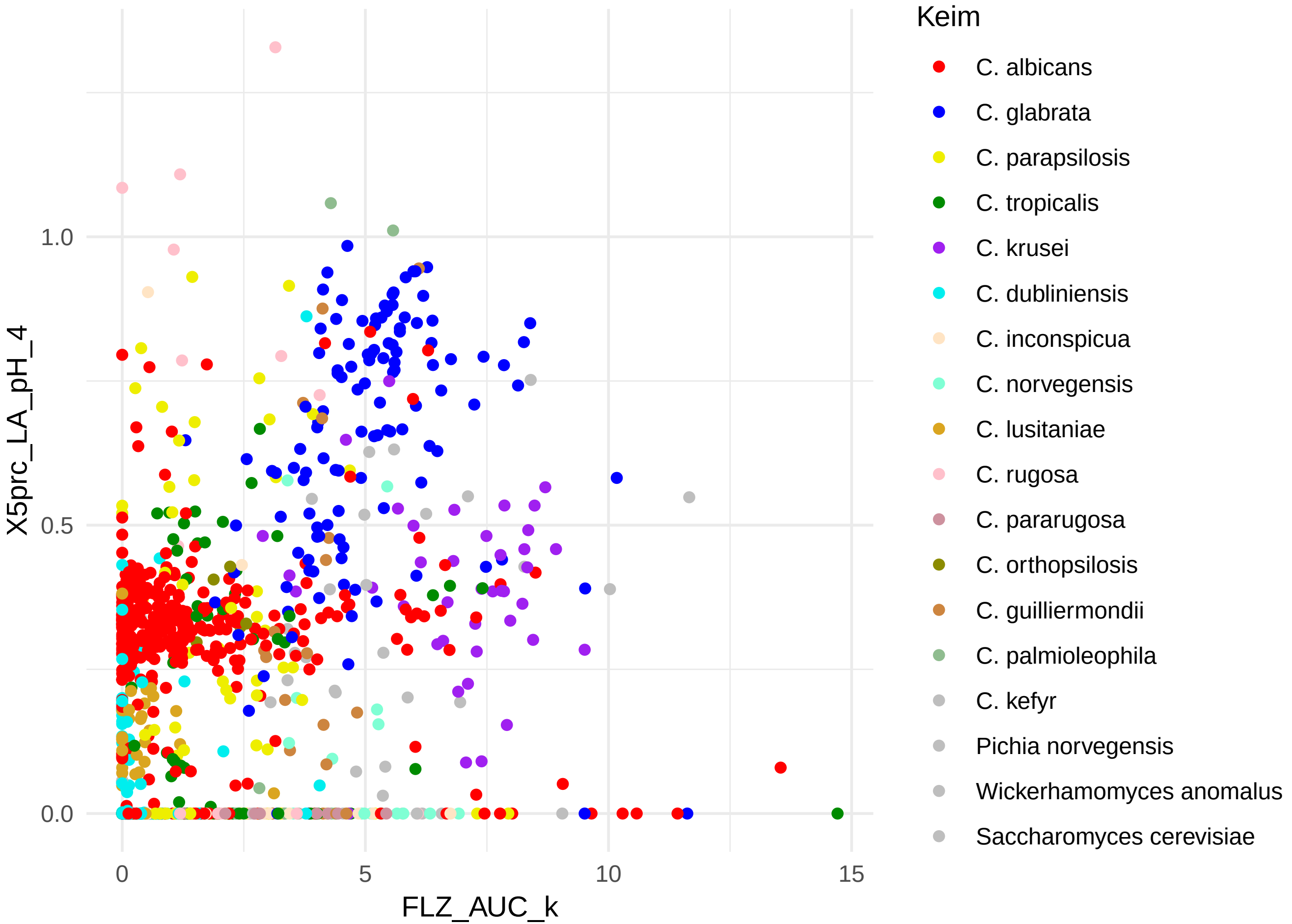
Keim
C. albicans
C. glabrata
C. parapsilosis
C. tropicalis
C. krusei
C. dubliniensis
C. inconspicua
C. norvegensis
C. lusitaniae
C. rugosa
C. pararugosa
C. orthopsilosis
C. guilliermondii
C. palmioleophila
C. kefyr
Pichia norvegensis
Wickerhamomyces anomalus
Saccharomyces cerevisiae
X5prc_LA_pH_4
1.0
0.5
0.0
0
5
10
15
FLZ_AUC_k

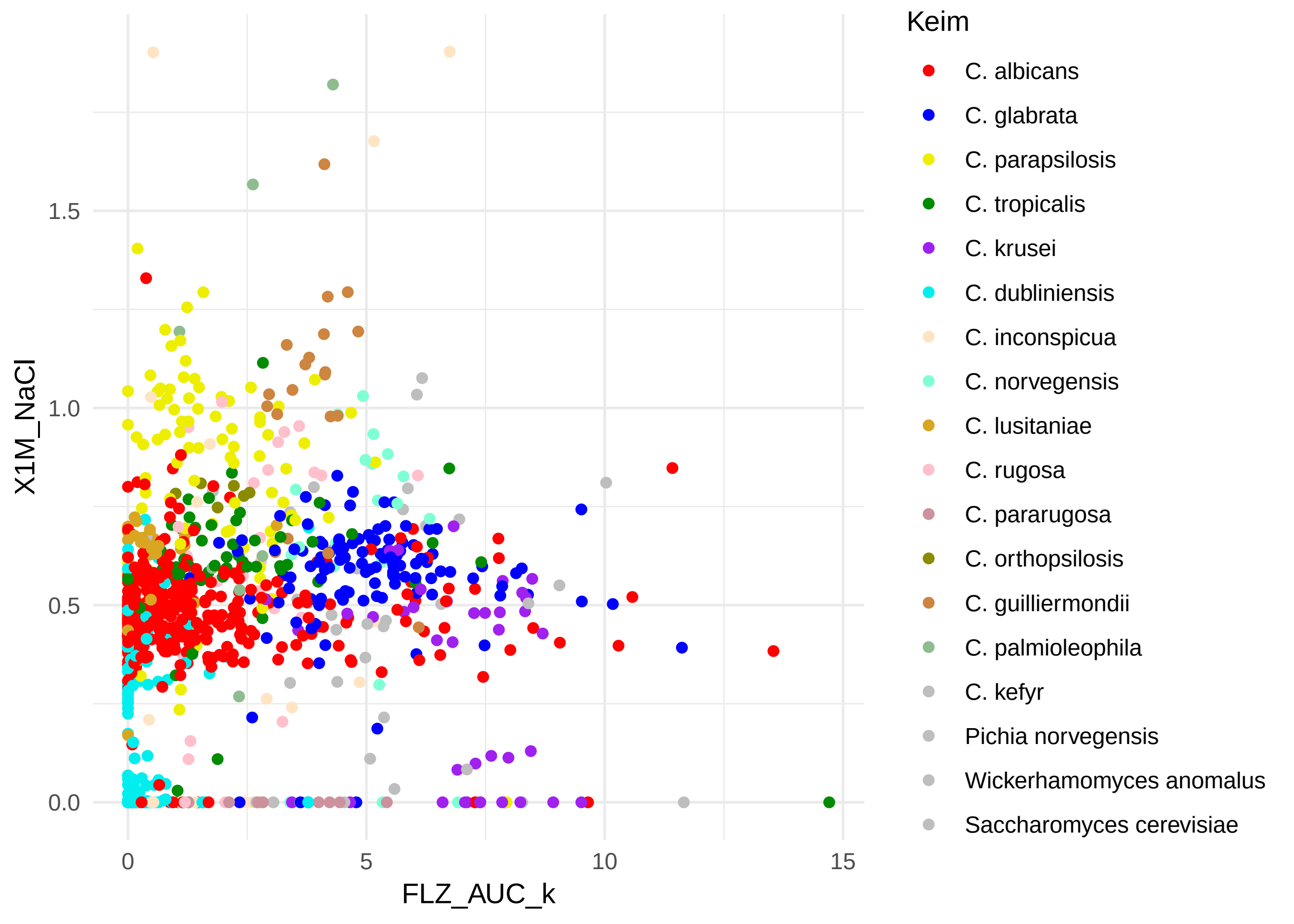

Keim
C. albicans
C. glabrata
C. parapsilosis
C. tropicalis
C. krusei
C. dubliniensis
C. inconspicua
C. norvegensis
C. lusitaniae
C. rugosa
C. pararugosa
C. orthopsilosis
C. guilliermondii
C. palmioleophila
C. kefyr
Pichia norvegensis
Wickerhamomyces anomalus
Saccharomyces cerevisiae
X1M_NaCl
1.5
1.0
0.5
0.0
0
5
10
15
FLZ_AUC_k

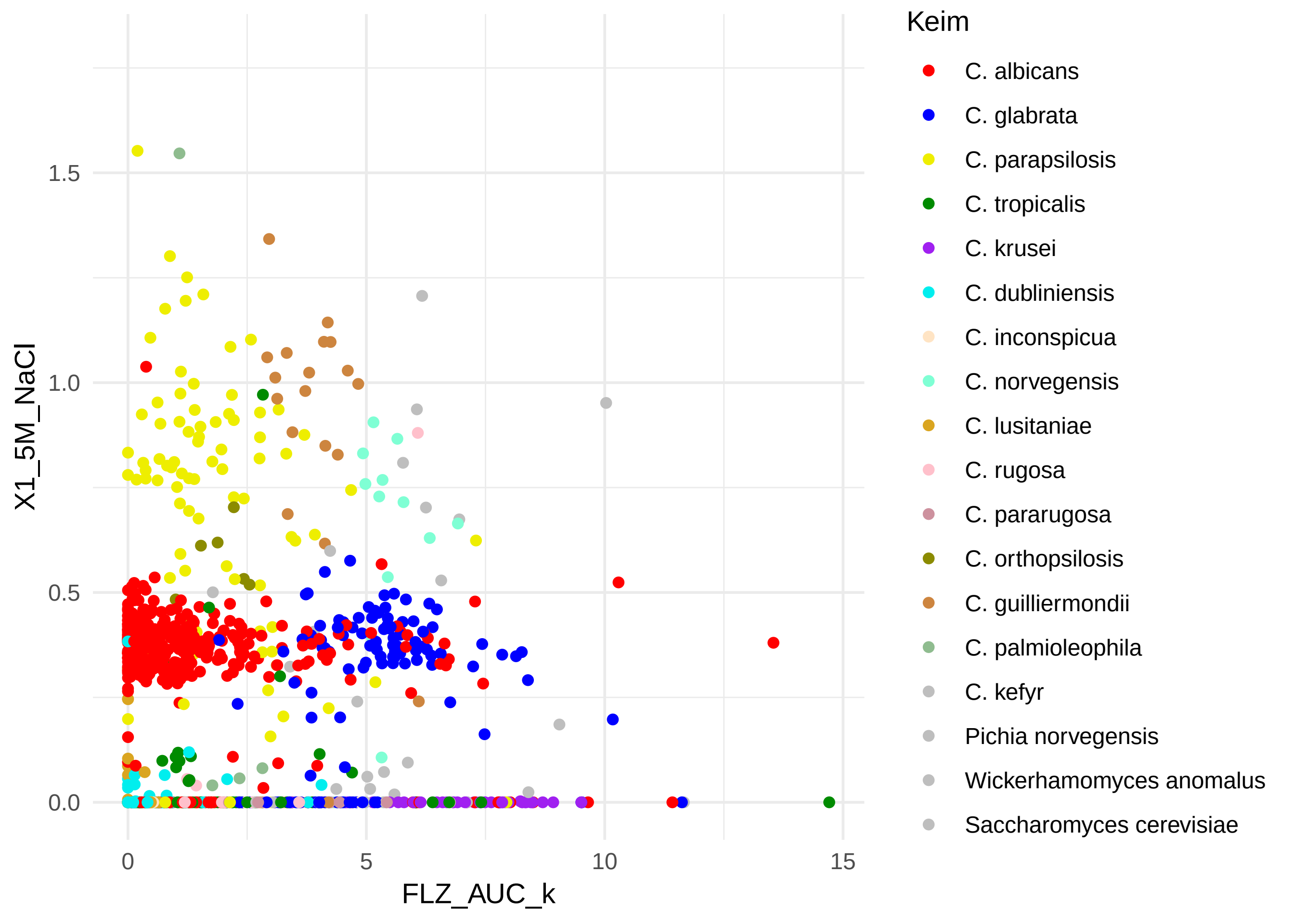
X1_5M_NaCl
1.5
1.0
0.5
0.0
0
5
10
15
FLZ_AUC_k
Keim
C. albicans
C. glabrata
C. parapsilosis
C. tropicalis
C. krusei
C. dubliniensis
C. inconspicua
C. norvegensis
C. lusitaniae
C. rugosa
C. pararugosa
C. orthopsilosis
C. guilliermondii
C. palmioleophila
C. kefyr
Pichia norvegensis
Wickerhamomyces anomalus
Saccharomyces cerevisiae

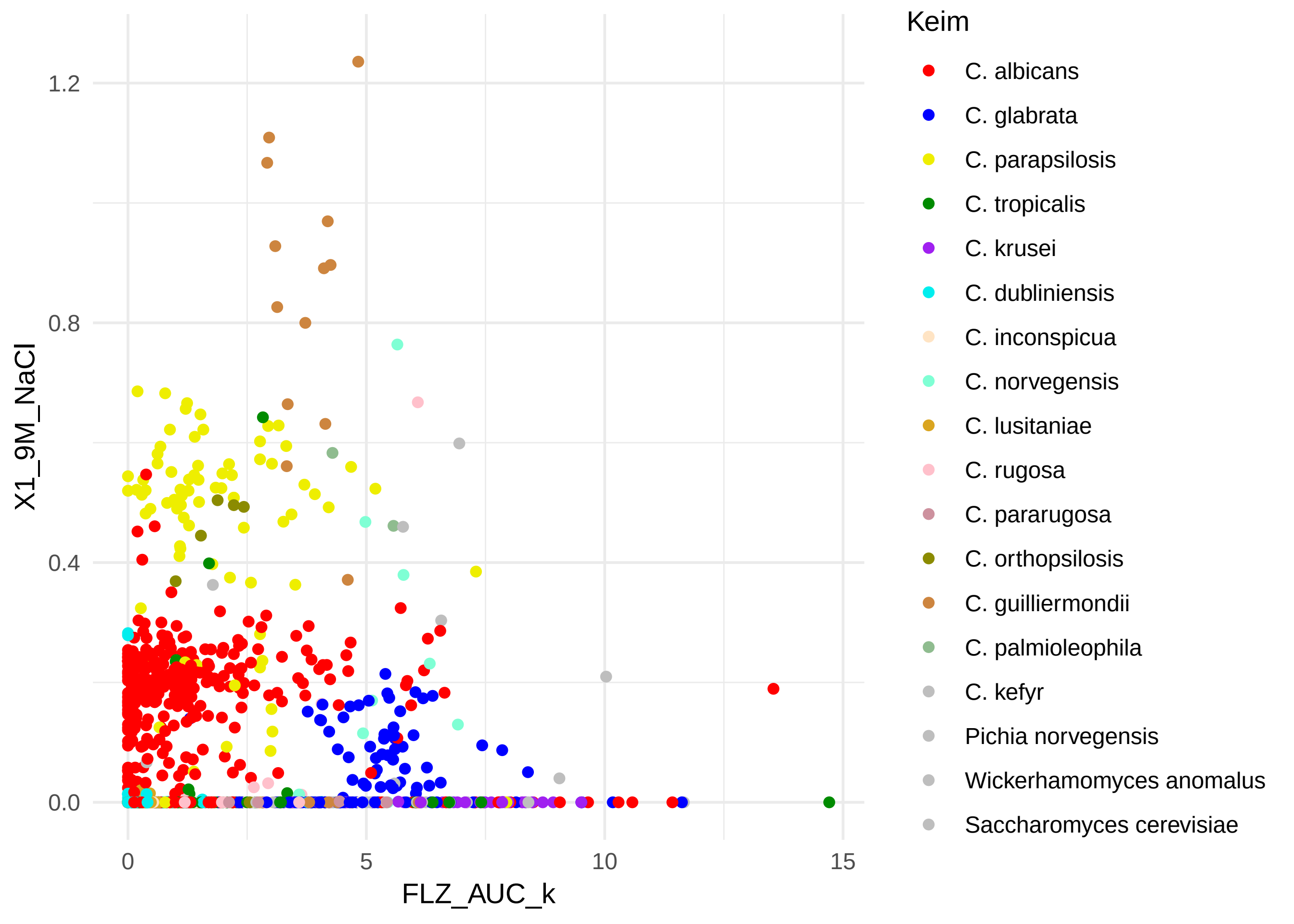

Keim
C. albicans
C. glabrata
C. parapsilosis
C. tropicalis
C. krusei
C. dubliniensis
C. inconspicua
C. norvegensis
C. lusitaniae
C. rugosa
C. pararugosa
C. orthopsilosis
C. guilliermondii
C. palmioleophila
C. kefyr
Pichia norvegensis
Wickerhamomyces anomalus
Saccharomyces cerevisiae
X1_9M_NaCl
FLZ_AUC_k
1.2
0.8
0.4
0.0
0
5
10
15

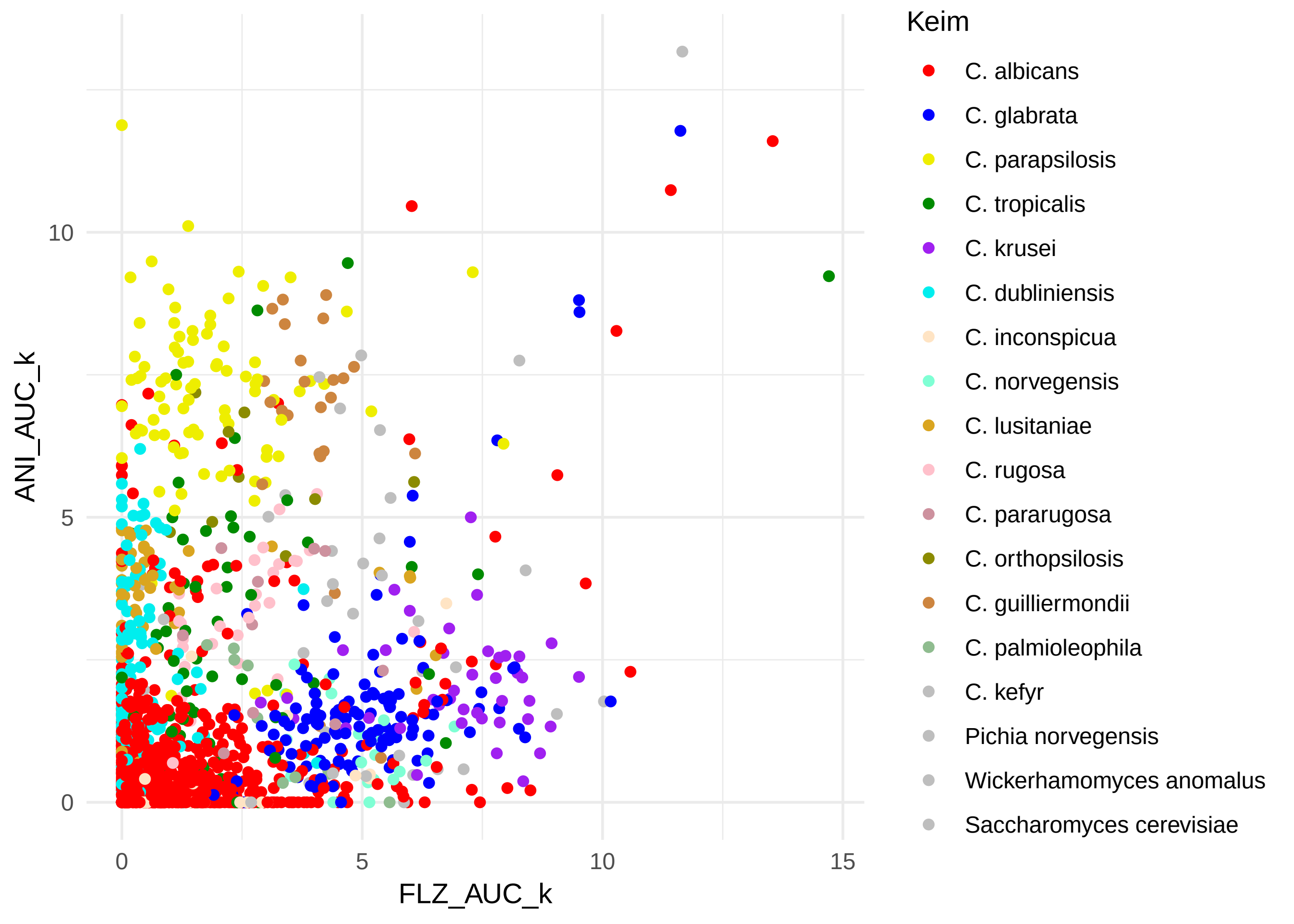
Keim
C. albicans
C. glabrata
C. parapsilosis
C. tropicalis
C. krusei
C. dubliniensis
C. inconspicua
C. norvegensis
C. lusitaniae
C. rugosa
C. pararugosa
C. orthopsilosis
C. guilliermondii
C. palmioleophila
C. kefyr
Pichia norvegensis
Wickerhamomyces anomalus
Saccharomyces cerevisiae
ANI_AUC_k
FLZ_AUC_k
0
5
10
15
0
5
10

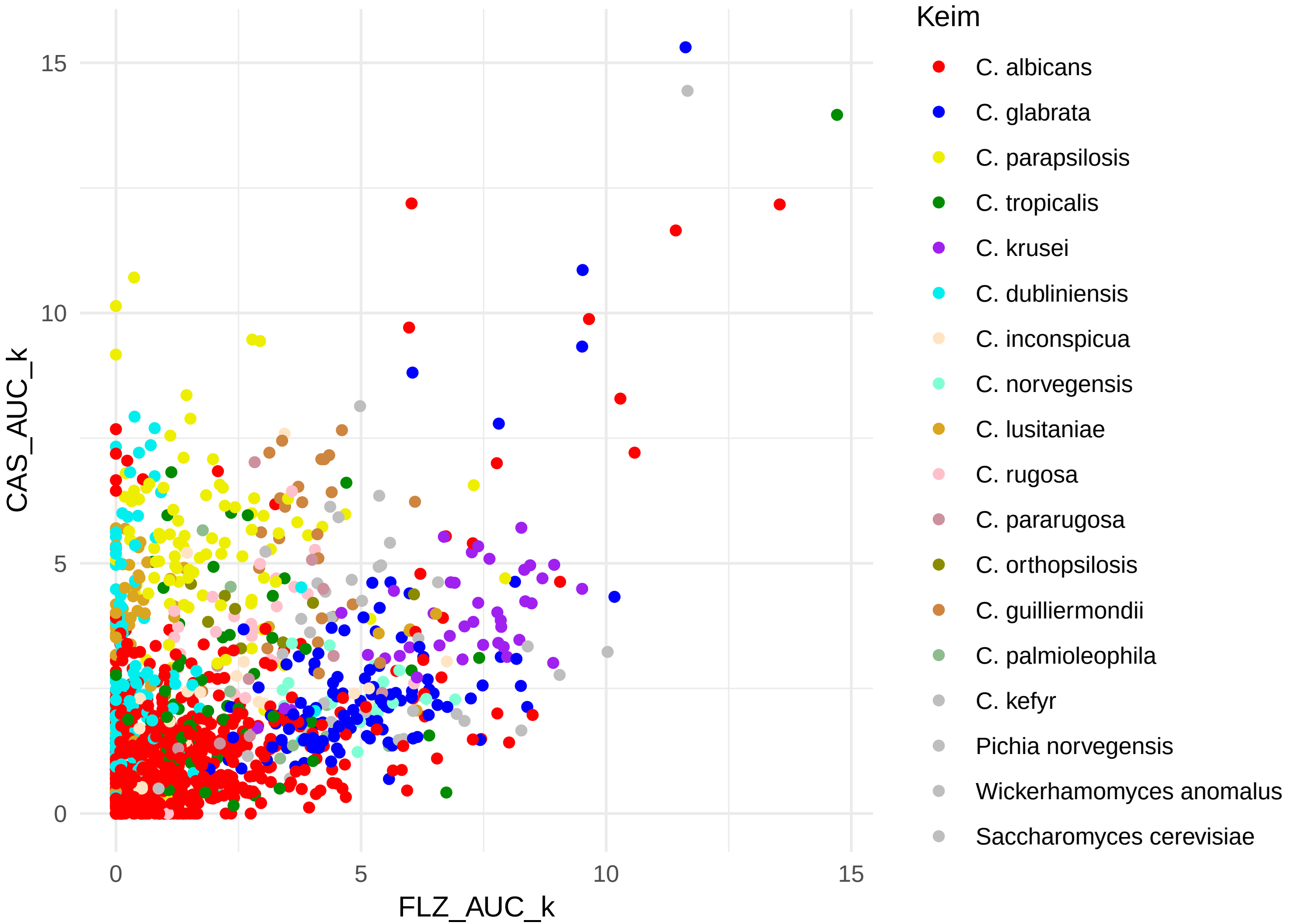

Keim
C. albicans
C. glabrata
C. parapsilosis
C. tropicalis
C. krusei
C. dubliniensis
C. inconspicua
C. norvegensis
C. lusitaniae
C. rugosa
C. pararugosa
C. orthopsilosis
C. guilliermondii
C. palmioleophila
C. kefyr
Pichia norvegensis
Wickerhamomyces anomalus
Saccharomyces cerevisiae
CAS_AUC_k
FLZ_AUC_k
0
5
10
15

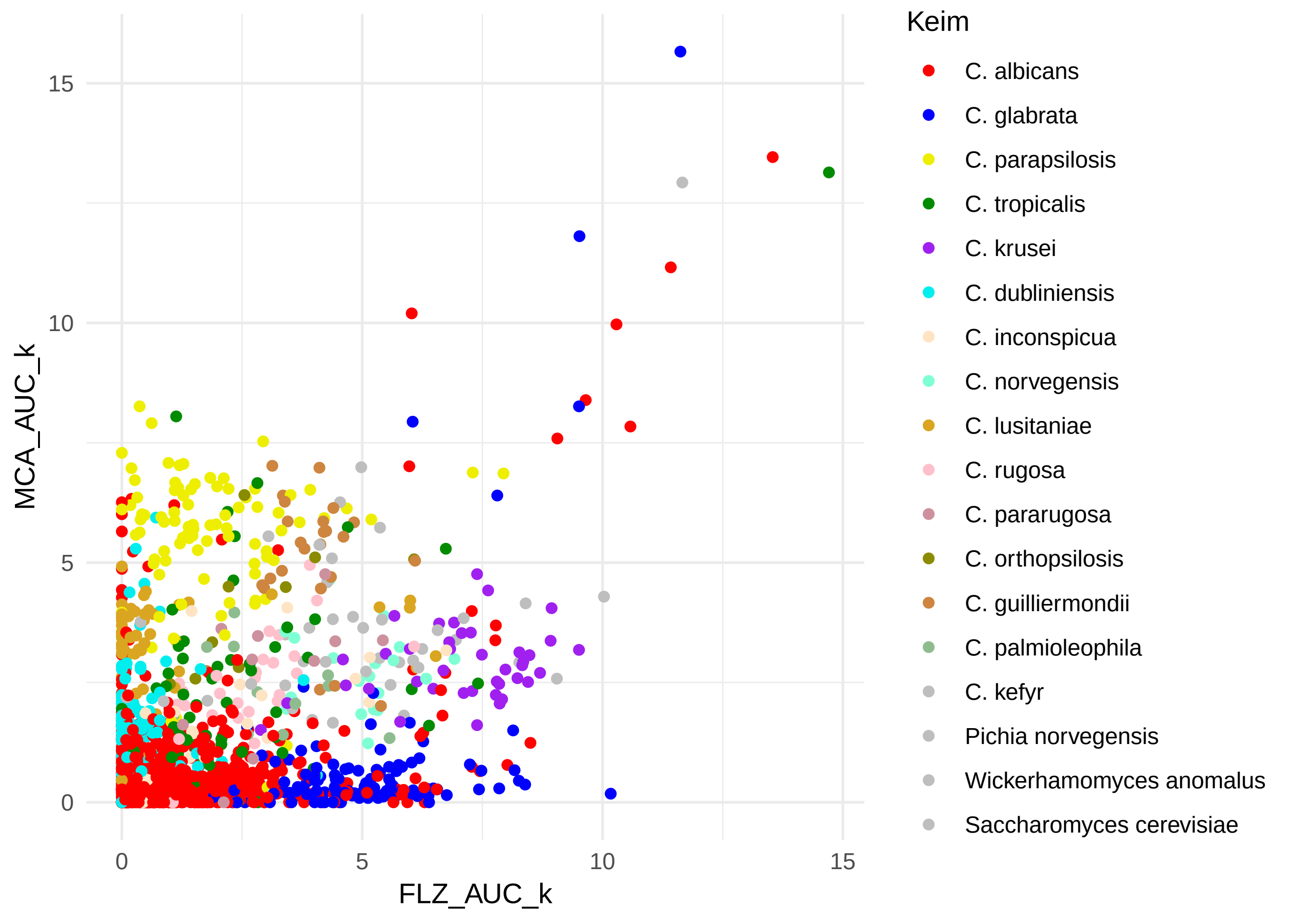

MCA_AUC_k
FLZ_AUC_k
0
5
10
15
Keim
C. albicans
C. glabrata
C. parapsilosis
C. tropicalis
C. krusei
C. dubliniensis
C. inconspicua
C. norvegensis
C. lusitaniae
C. rugosa
C. pararugosa
C. orthopsilosis
C. guilliermondii
C. palmioleophila
C. kefyr
Pichia norvegensis
Wickerhamomyces anomalus
Saccharomyces cerevisiae

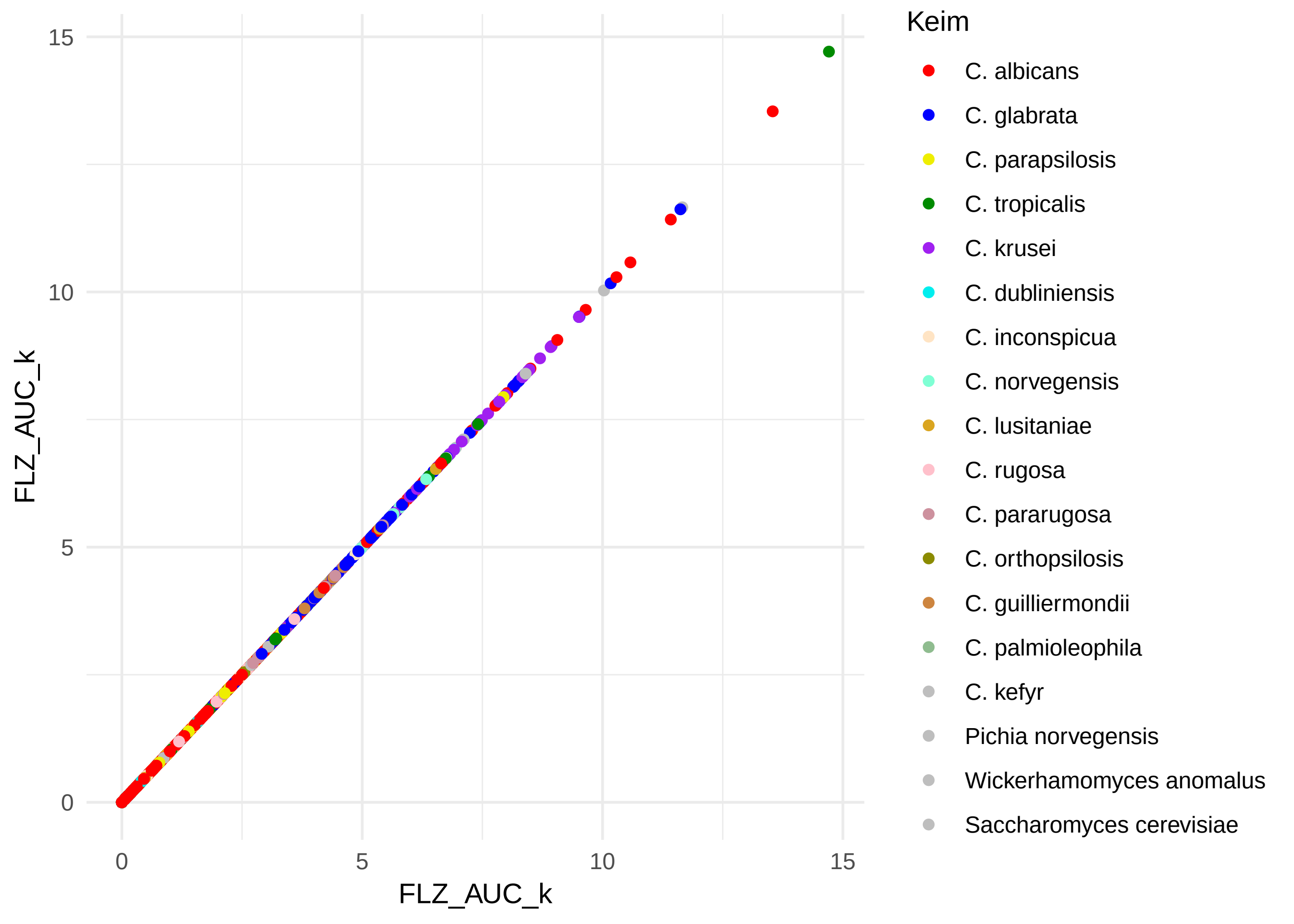
15
10
5
0
FLZ_AUC_k
0
5
10
15
FLZ_AUC_k
Keim
C. albicans
C. glabrata
C. parapsilosis
C. tropicalis
C. krusei
C. dubliniensis
C. inconspicua
C. norvegensis
C. lusitaniae
C. rugosa
C. pararugosa
C. orthopsilosis
C. guilliermondii
C. palmioleophila
C. kefyr
Pichia norvegensis
Wickerhamomyces anomalus
Saccharomyces cerevisiae

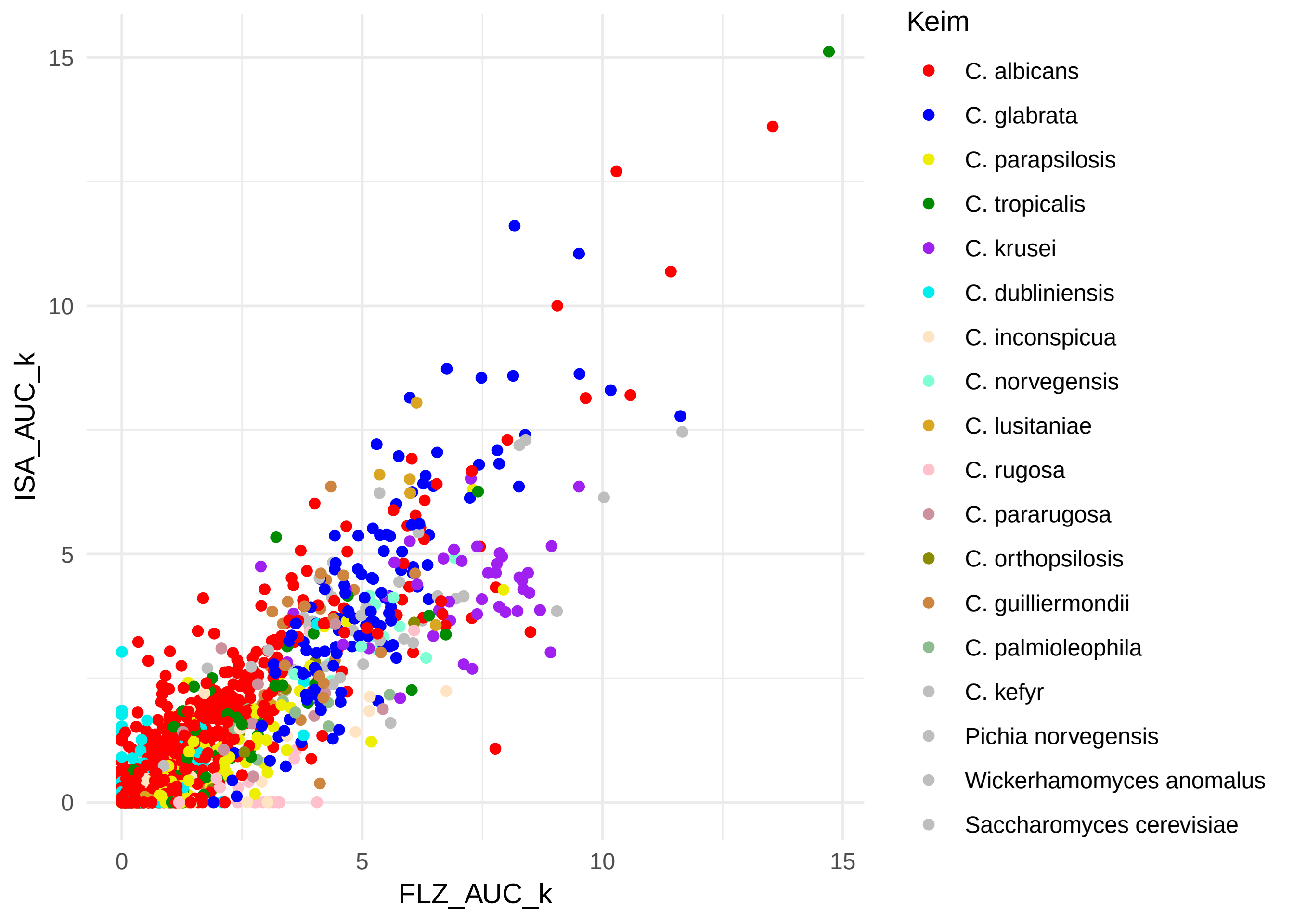
Keim
C. albicans
C. glabrata
C. parapsilosis
C. tropicalis
C. krusei
C. dubliniensis
C. inconspicua
C. norvegensis
C. lusitaniae
C. rugosa
C. pararugosa
C. orthopsilosis
C. guilliermondii
C. palmioleophila
C. kefyr
Pichia norvegensis
Wickerhamomyces anomalus
Saccharomyces cerevisiae
ISA_AUC_k
FLZ_AUC_k
0
5
10
15

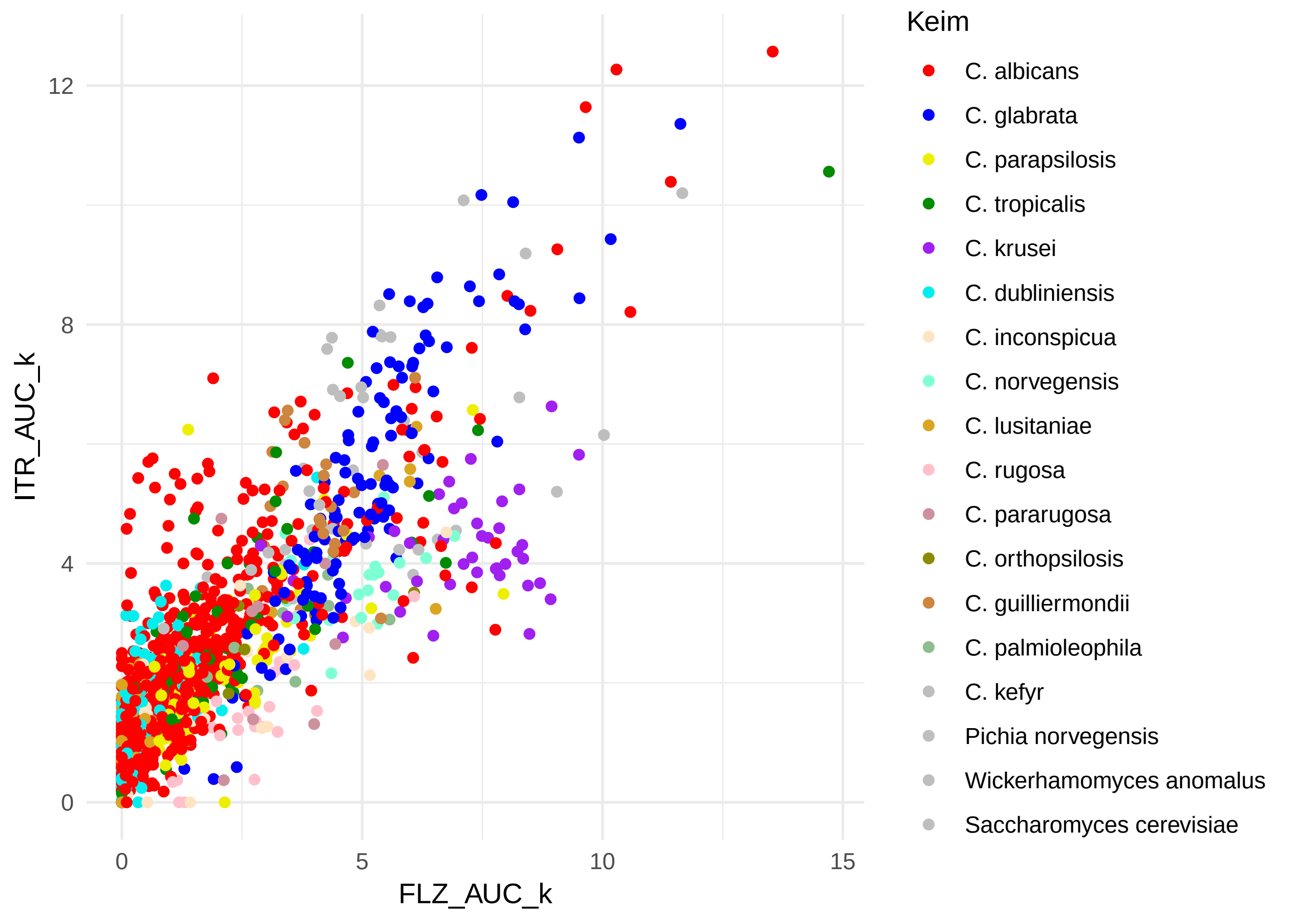
Keim
C. albicans
C. glabrata
C. parapsilosis
C. tropicalis
C. krusei
C. dubliniensis
C. inconspicua
C. norvegensis
C. lusitaniae
C. rugosa
C. pararugosa
C. orthopsilosis
C. guilliermondii
C. palmioleophila
C. kefyr
Pichia norvegensis
Wickerhamomyces anomalus
Saccharomyces cerevisiae
ITR_AUC_k
12
8
4
0
FLZ_AUC_k
0
5
10
15

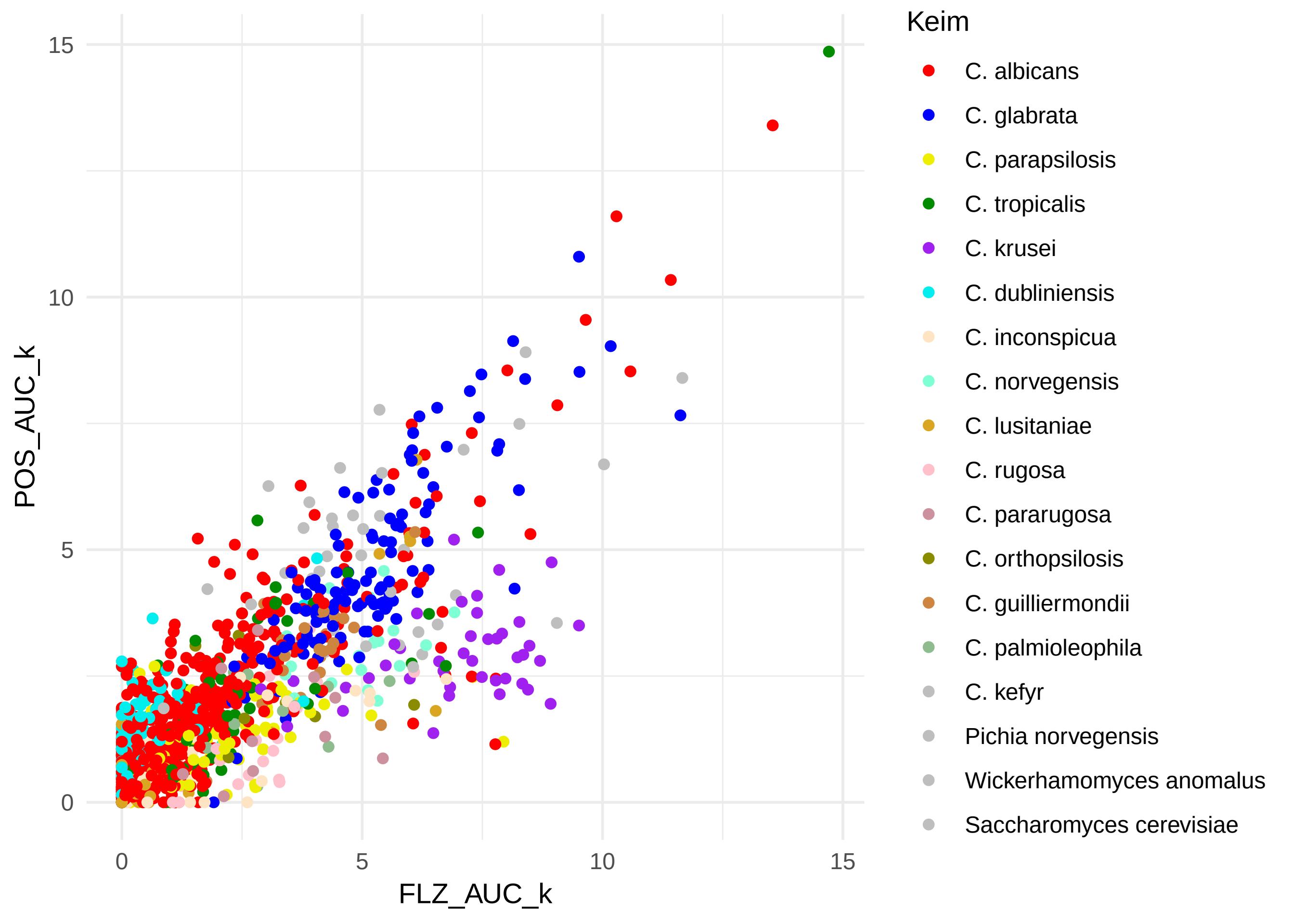
15
10
5
0
POS_AUC_k
0
5
10
15
FLZ_AUC_k
Keim
C. albicans
C. glabrata
C. parapsilosis
C. tropicalis
C. krusei
C. dubliniensis
C. inconspicua
C. norvegensis
C. lusitaniae
C. rugosa
C. pararugosa
C. orthopsilosis
C. guilliermondii
C. palmioleophila
C. kefyr
Pichia norvegensis
Wickerhamomyces anomalus
Saccharomyces cerevisiae

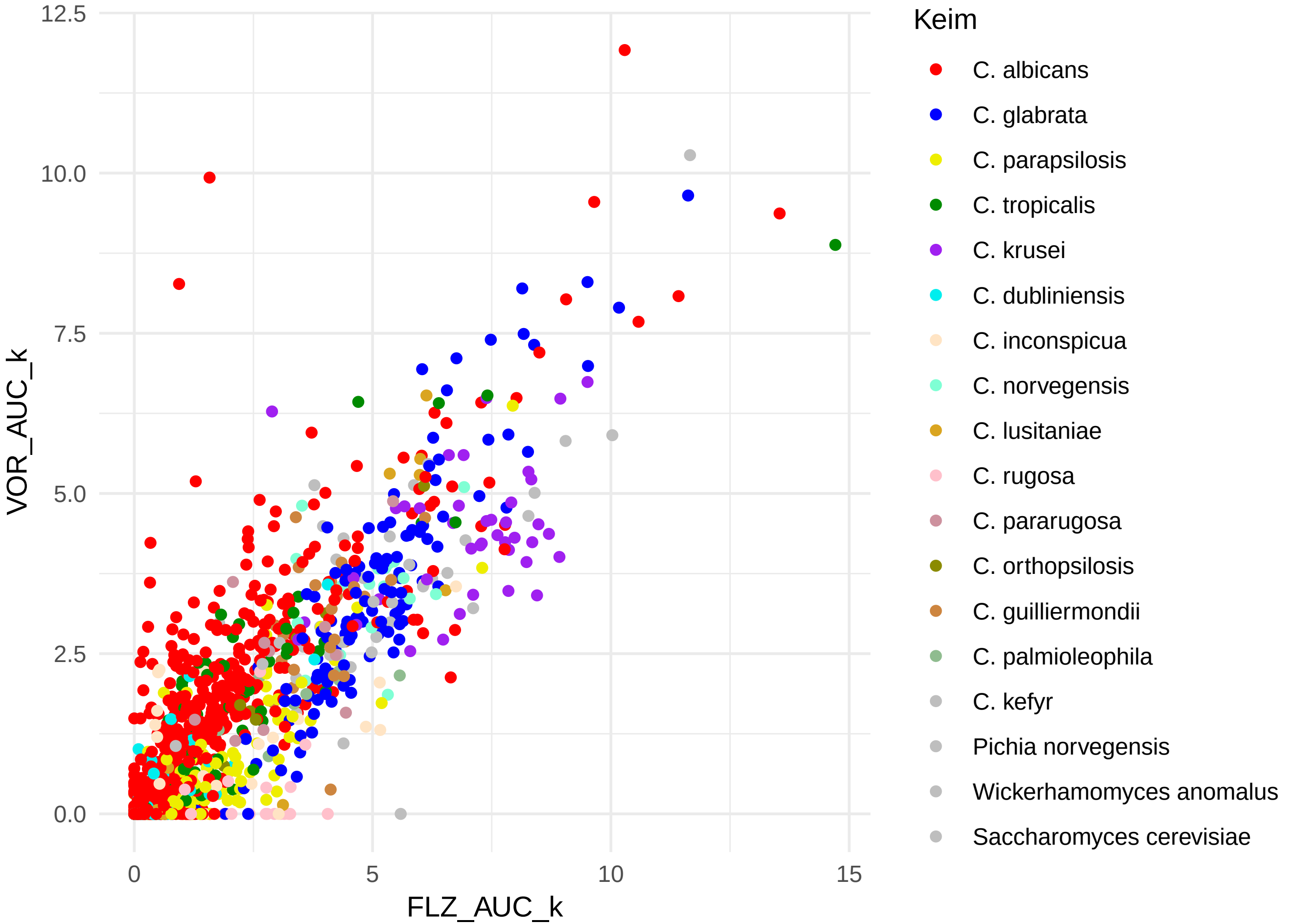

12.5
10.0
7.5
5.0
2.5
0.0
VOR_AUC_k
0
5
10
15
FLZ_AUC_k
Keim
C. albicans
C. glabrata
C. parapsilosis
C. tropicalis
C. krusei
C. dubliniensis
C. inconspicua
C. norvegensis
C. lusitaniae
C. rugosa
C. pararugosa
C. orthopsilosis
C. guilliermondii
C. palmioleophila
C. kefyr
Pichia norvegensis
Wickerhamomyces anomalus
Saccharomyces cerevisiae

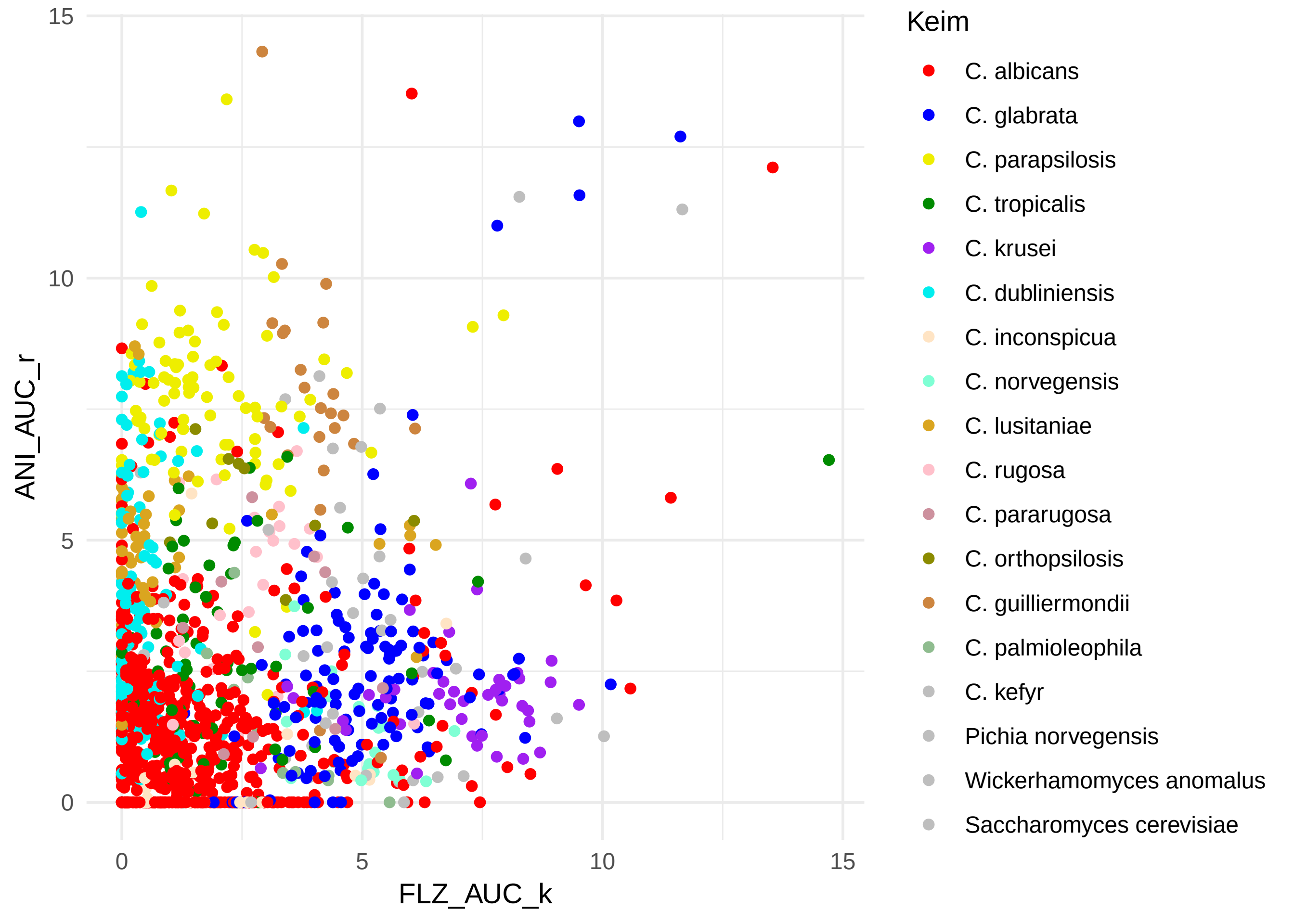
15
10
5
0
ANI_AUC_r
0
5
10
15
FLZ_AUC_k
Keim
C. albicans
C. glabrata
C. parapsilosis
C. tropicalis
C. krusei
C. dubliniensis
C. inconspicua
C. norvegensis
C. lusitaniae
C. rugosa
C. pararugosa
C. orthopsilosis
C. guilliermondii
C. palmioleophila
C. kefyr
Pichia norvegensis
Wickerhamomyces anomalus
Saccharomyces cerevisiae

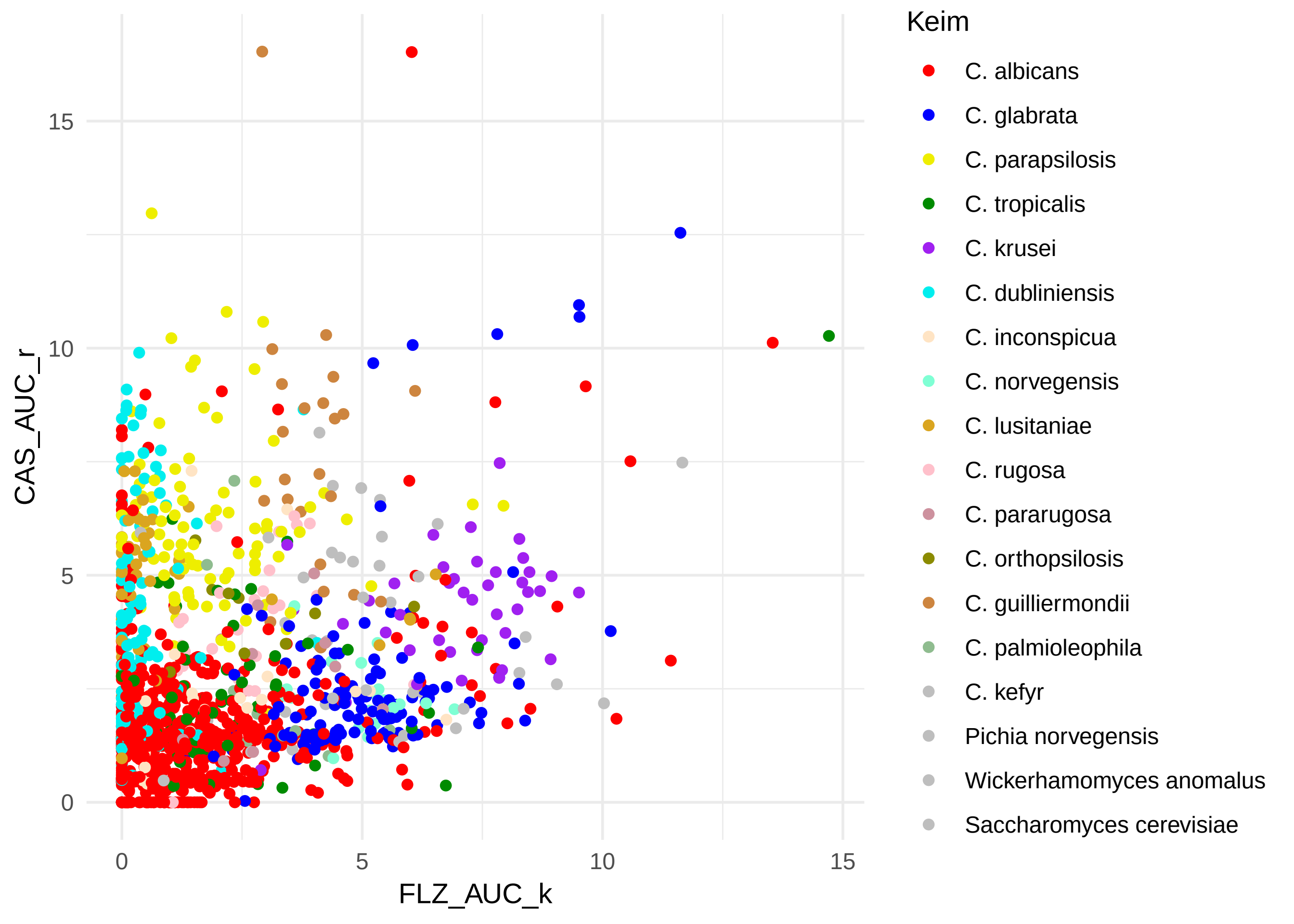

CAS_AUC_r
FLZ_AUC_k
0
5
10
15
0
5
10
15
Keim
C. albicans
C. glabrata
C. parapsilosis
C. tropicalis
C. krusei
C. dubliniensis
C. inconspicua
C. norvegensis
C. lusitaniae
C. rugosa
C. pararugosa
C. orthopsilosis
C. guilliermondii
C. palmioleophila
C. kefyr
Pichia norvegensis
Wickerhamomyces anomalus
Saccharomyces cerevisiae

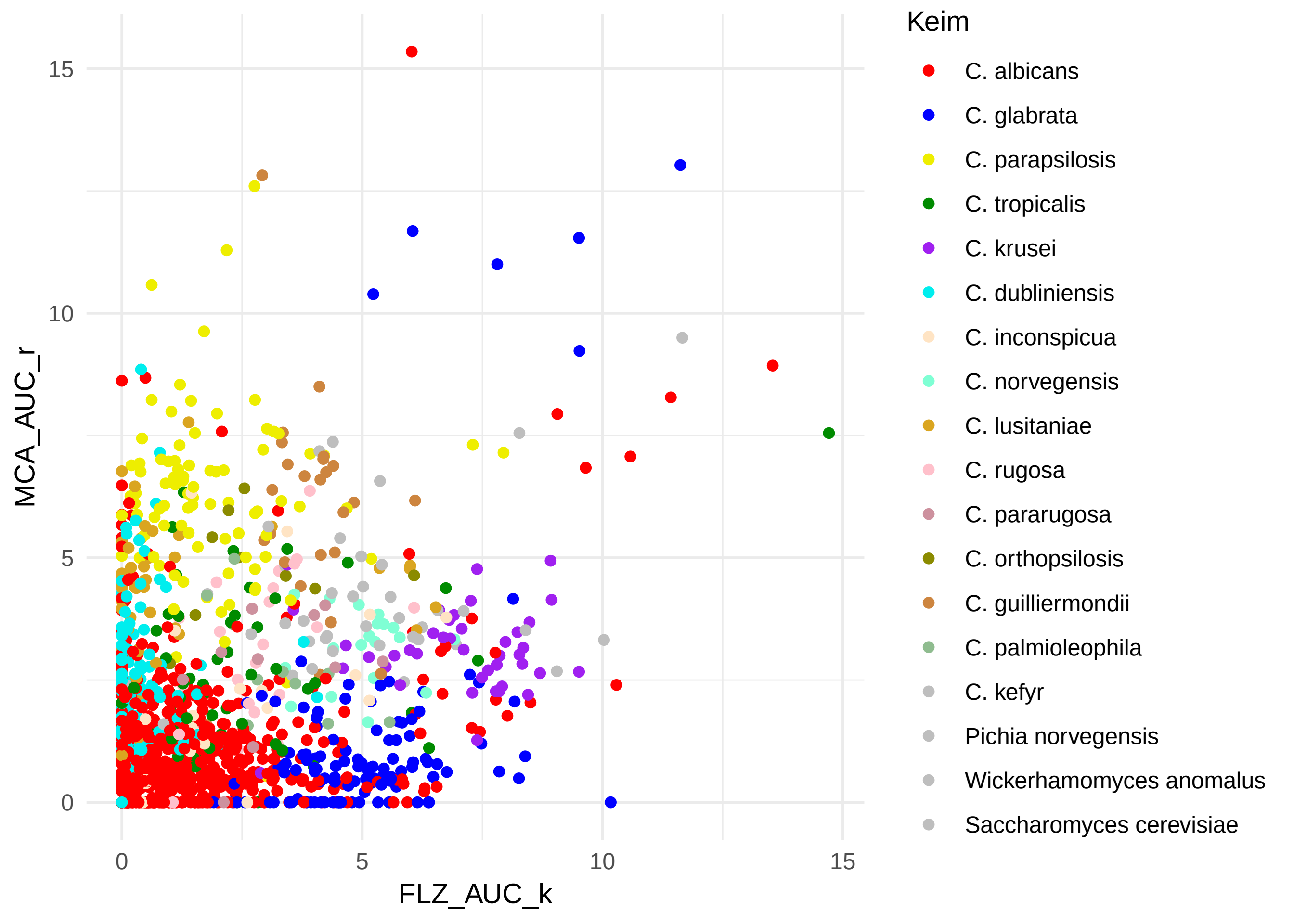

Keim
C. albicans
C. glabrata
C. parapsilosis
C. tropicalis
C. krusei
C. dubliniensis
C. inconspicua
C. norvegensis
C. lusitaniae
C. rugosa
C. pararugosa
C. orthopsilosis
C. guilliermondii
C. palmioleophila
C. kefyr
Pichia norvegensis
Wickerhamomyces anomalus
Saccharomyces cerevisiae
MCA_AUC_r
FLZ_AUC_k
0
5
10
15

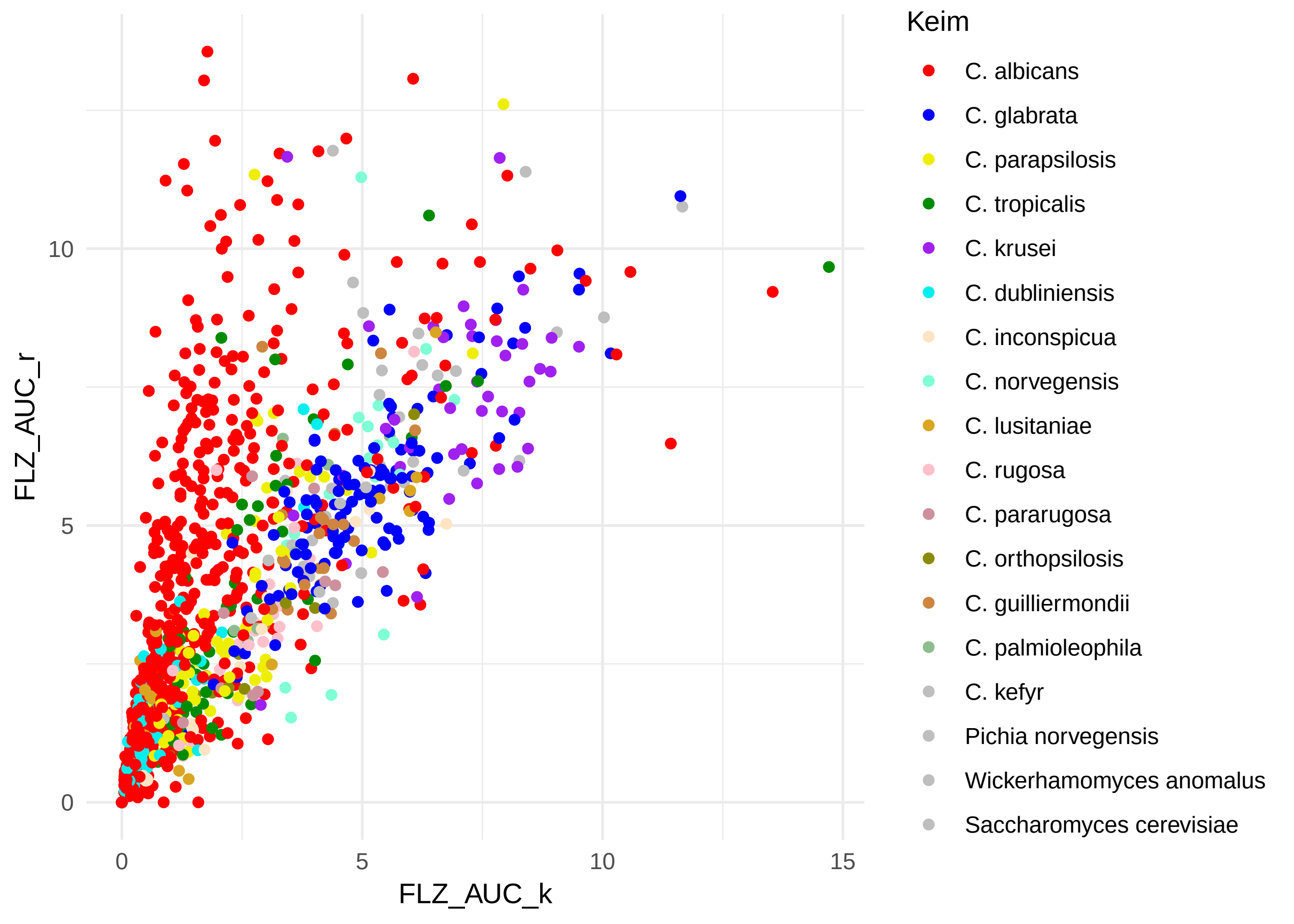
Keim
C. albicans
C. glabrata
C. parapsilosis
C. tropicalis
C. krusei
C. dubliniensis
C. inconspicua
C. norvegensis
C. lusitaniae
C. rugosa
C. pararugosa
C. orthopsilosis
C. guilliermondii
C. palmioleophila
C. kefyr
Pichia norvegensis
Wickerhamomyces anomalus
Saccharomyces cerevisiae
FLZ_AUC_r
FLZ_AUC_k
0
5
10
15
0
5
10

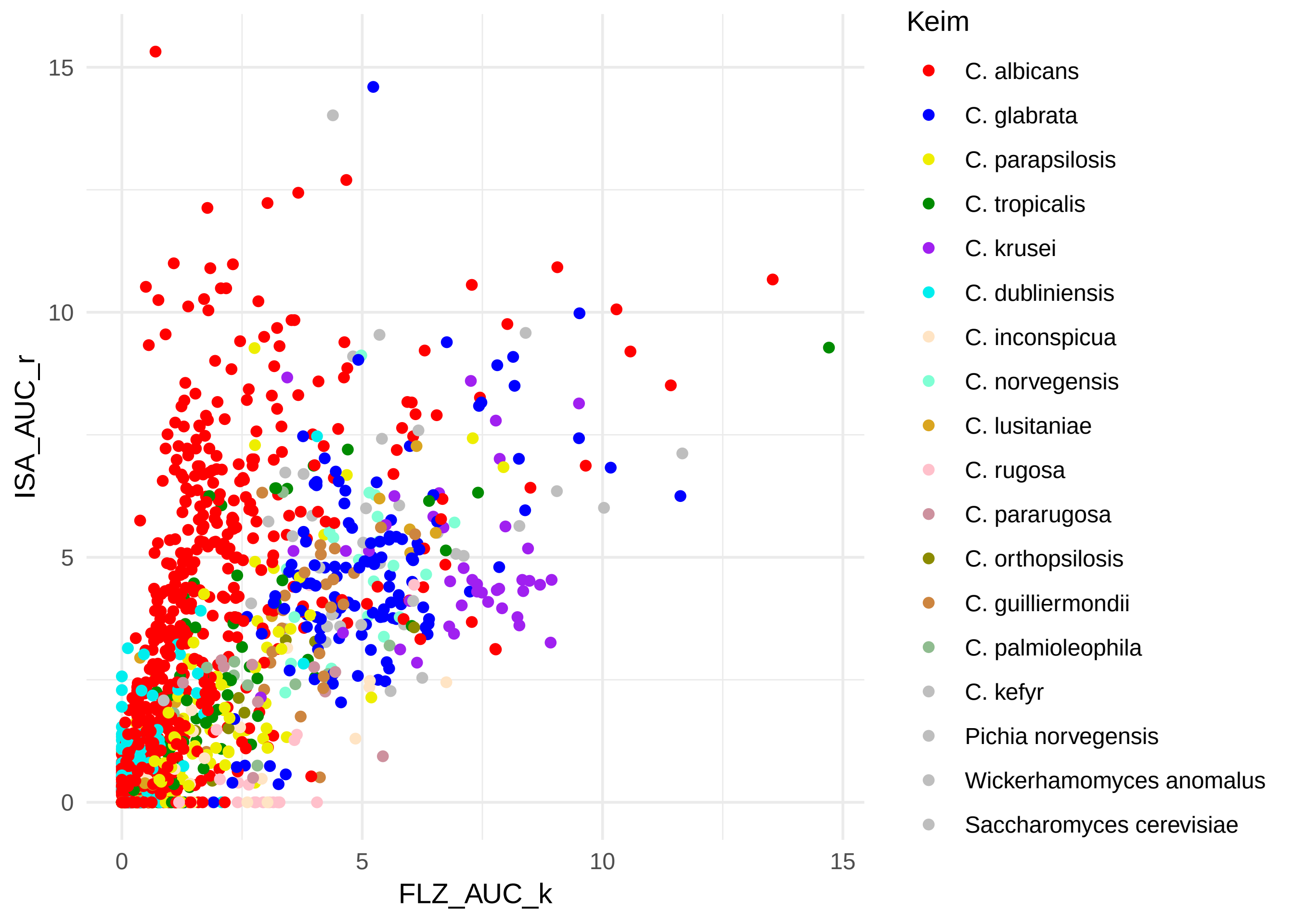

Keim
C. albicans
C. glabrata
C. parapsilosis
C. tropicalis
C. krusei
C. dubliniensis
C. inconspicua
C. norvegensis
C. lusitaniae
C. rugosa
C. pararugosa
C. orthopsilosis
C. guilliermondii
C. palmioleophila
C. kefyr
Pichia norvegensis
Wickerhamomyces anomalus
Saccharomyces cerevisiae
ISA_AUC_r
FLZ_AUC_k
0
5
10
15
0
5
10
15

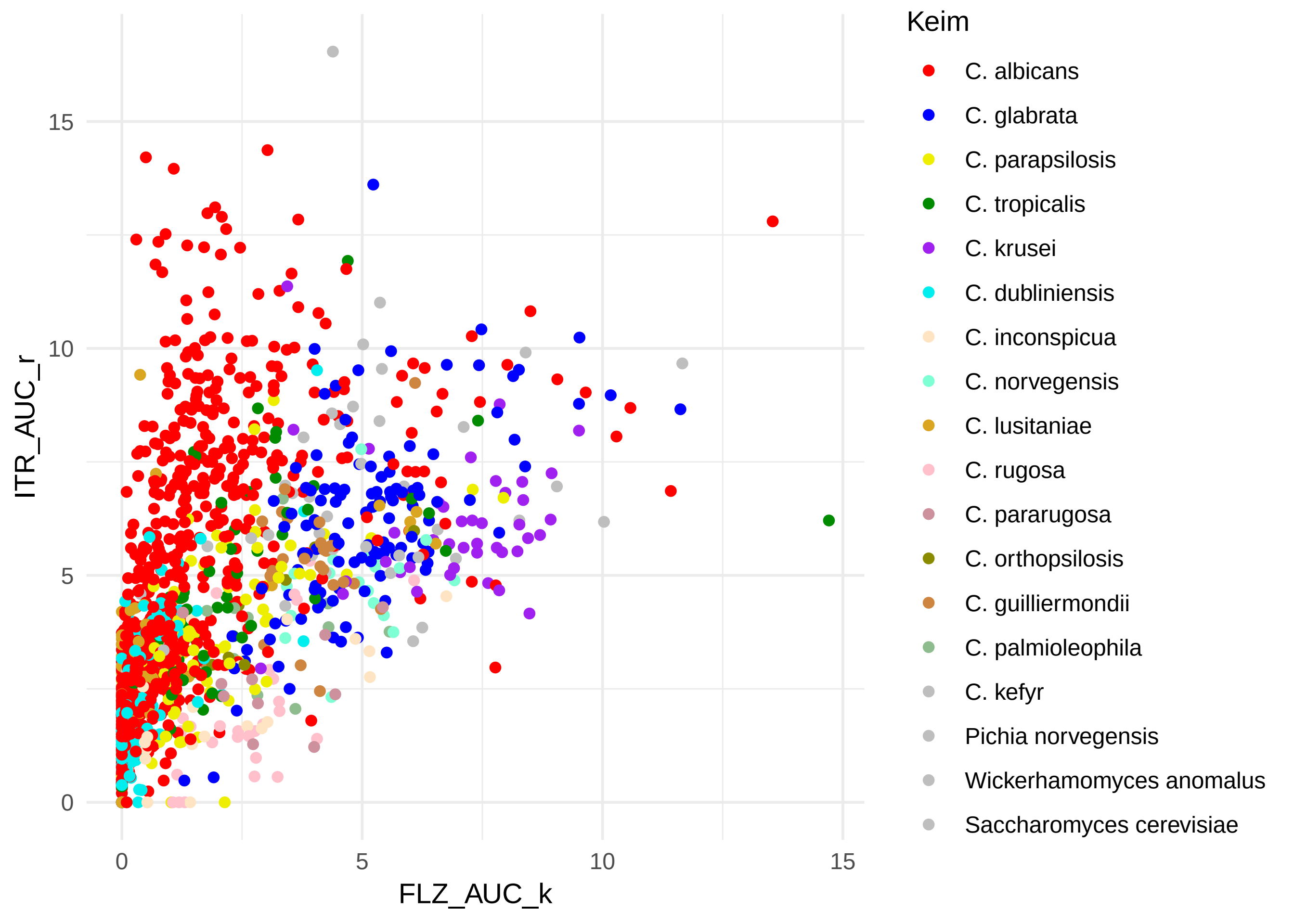

Keim
C. albicans
C. glabrata
C. parapsilosis
C. tropicalis
C. krusei
C. dubliniensis
C. inconspicua
C. norvegensis
C. lusitaniae
C. rugosa
C. pararugosa
C. orthopsilosis
C. guilliermondii
C. palmioleophila
C. kefyr
Pichia norvegensis
Wickerhamomyces anomalus
Saccharomyces cerevisiae
ITR_AUC_r
FLZ_AUC_k
0
5
10
15

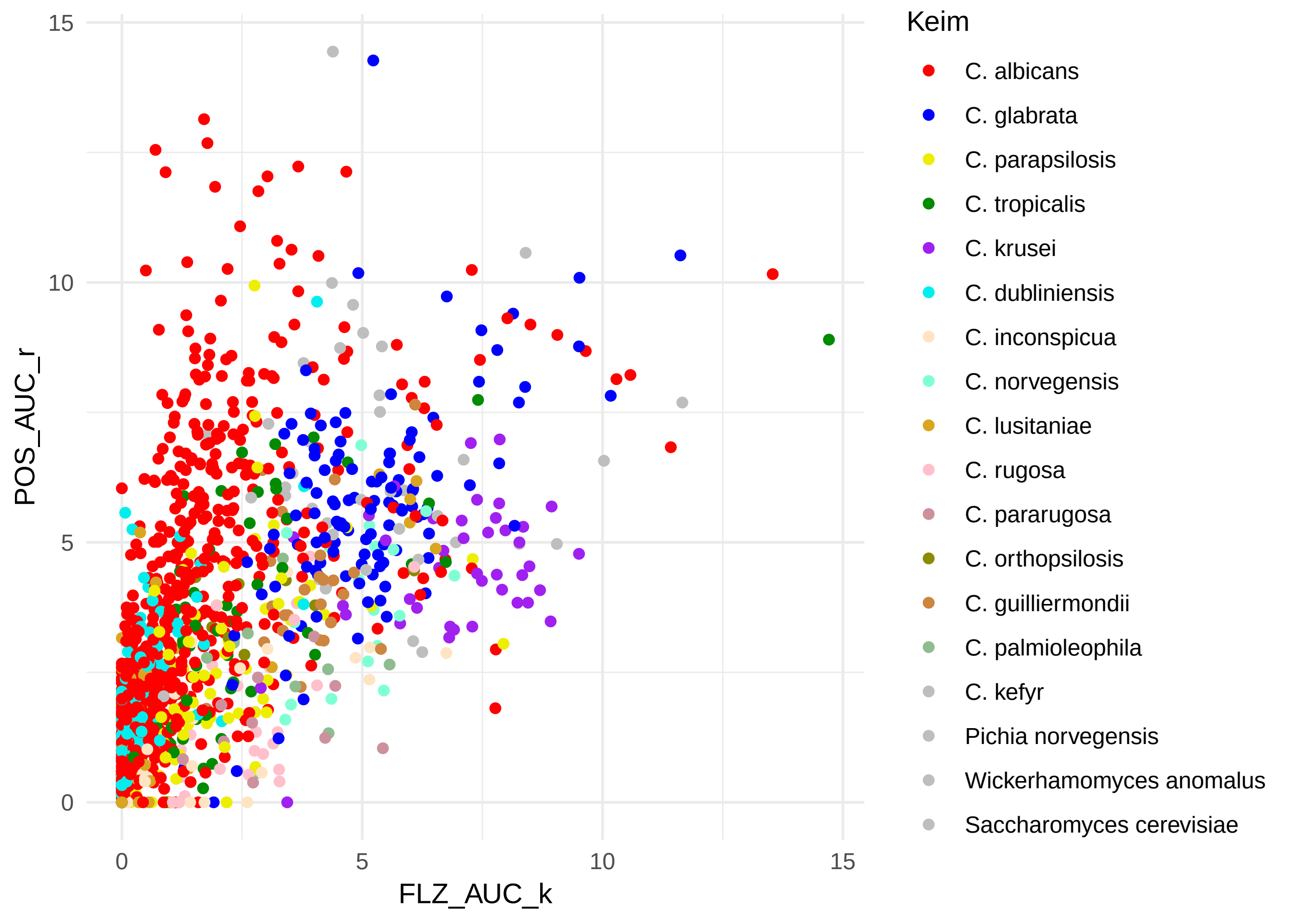
15
10
5
0
POS_AUC_r
0
5
10
15
FLZ_AUC_k
Keim
C. albicans
C. glabrata
C. parapsilosis
C. tropicalis
C. krusei
C. dubliniensis
C. inconspicua
C. norvegensis
C. lusitaniae
C. rugosa
C. pararugosa
C. orthopsilosis
C. guilliermondii
C. palmioleophila
C. kefyr
Pichia norvegensis
Wickerhamomyces anomalus
Saccharomyces cerevisiae

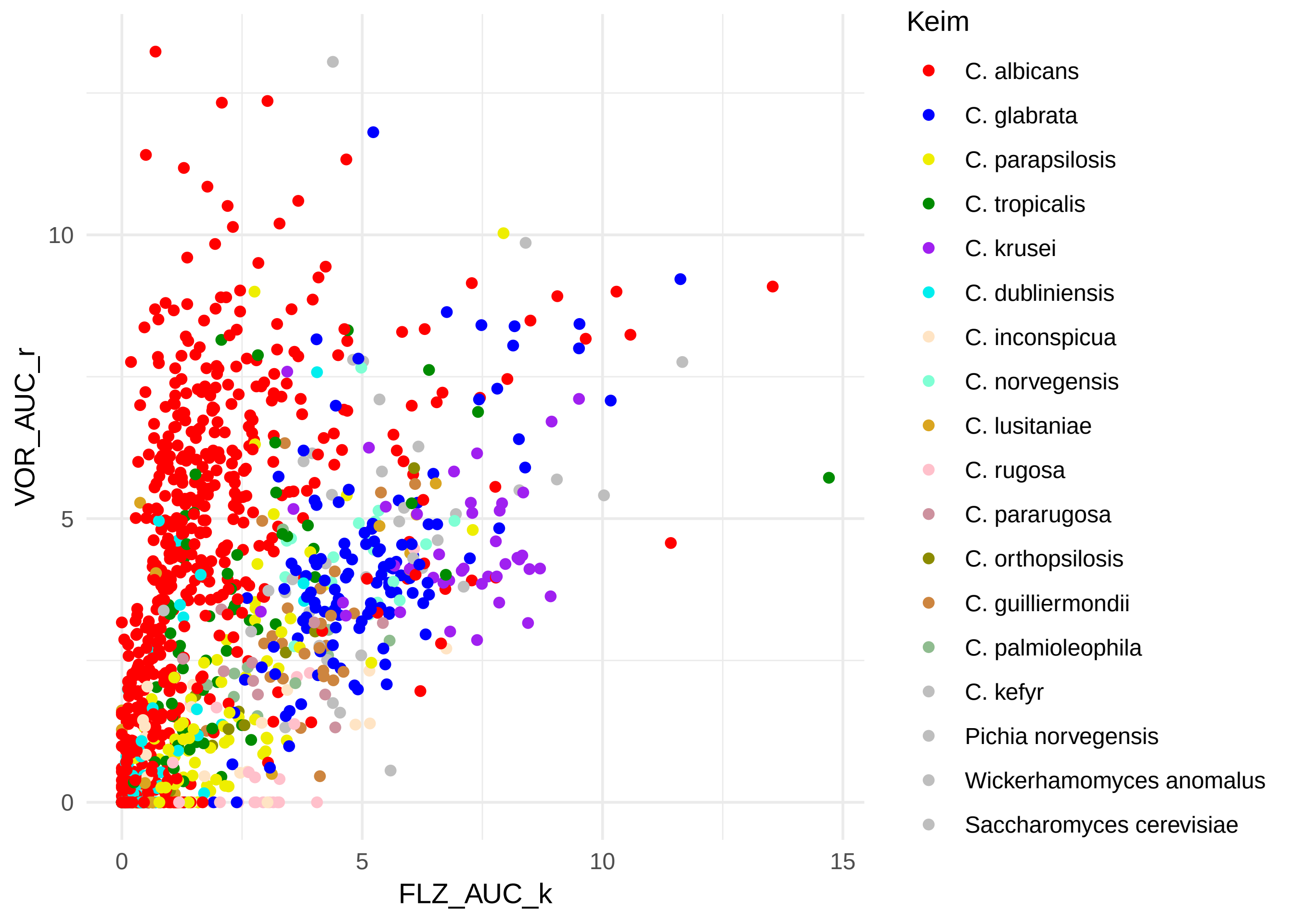
Keim
C. albicans
C. glabrata
C. parapsilosis
C. tropicalis
C. krusei
C. dubliniensis
C. inconspicua
C. norvegensis
C. lusitaniae
C. rugosa
C. pararugosa
C. orthopsilosis
C. guilliermondii
C. palmioleophila
C. kefyr
Pichia norvegensis
Wickerhamomyces anomalus
Saccharomyces cerevisiae
VOR_AUC_r
FLZ_AUC_k
0
5
10
15
0
5
10

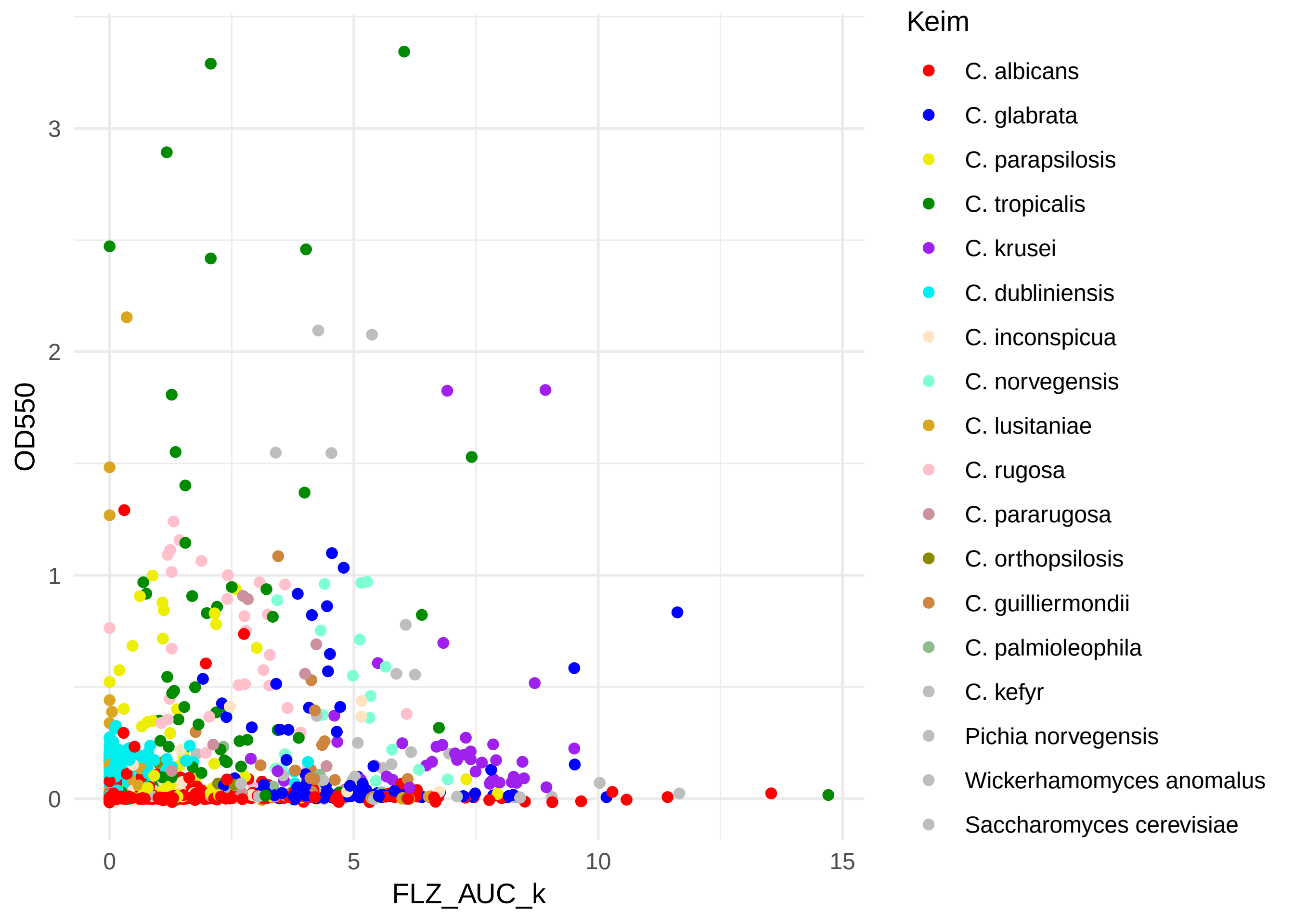

Keim
C. albicans
C. glabrata
C. parapsilosis
C. tropicalis
C. krusei
C. dubliniensis
C. inconspicua
C. norvegensis
C. lusitaniae
C. rugosa
C. pararugosa
C. orthopsilosis
C. guilliermondii
C. palmioleophila
C. kefyr
Pichia norvegensis
Wickerhamomyces anomalus
Saccharomyces cerevisiae
OD550
3
2
1
0
0
5
10
15
FLZ_AUC_k

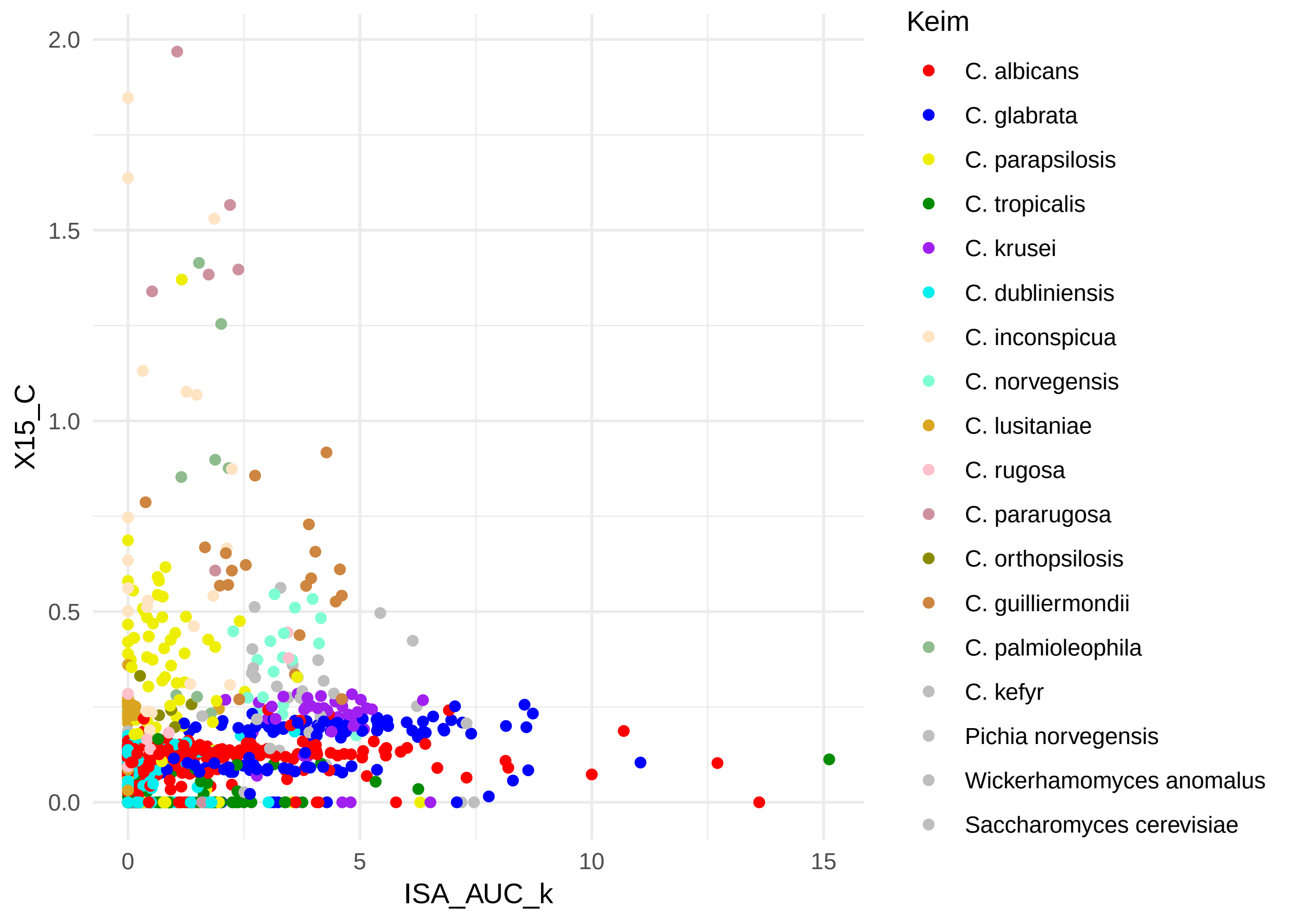

Keim
C. albicans
C. glabrata
C. parapsilosis
C. tropicalis
C. krusei
C. dubliniensis
C. inconspicua
C. norvegensis
C. lusitaniae
C. rugosa
C. pararugosa
C. orthopsilosis
C. guilliermondii
C. palmioleophila
C. kefyr
Pichia norvegensis
Wickerhamomyces anomalus
Saccharomyces cerevisiae
X15_C
2.0
1.5
1.0
0.5
0.0
0
5
10
15
ISA_AUC_k

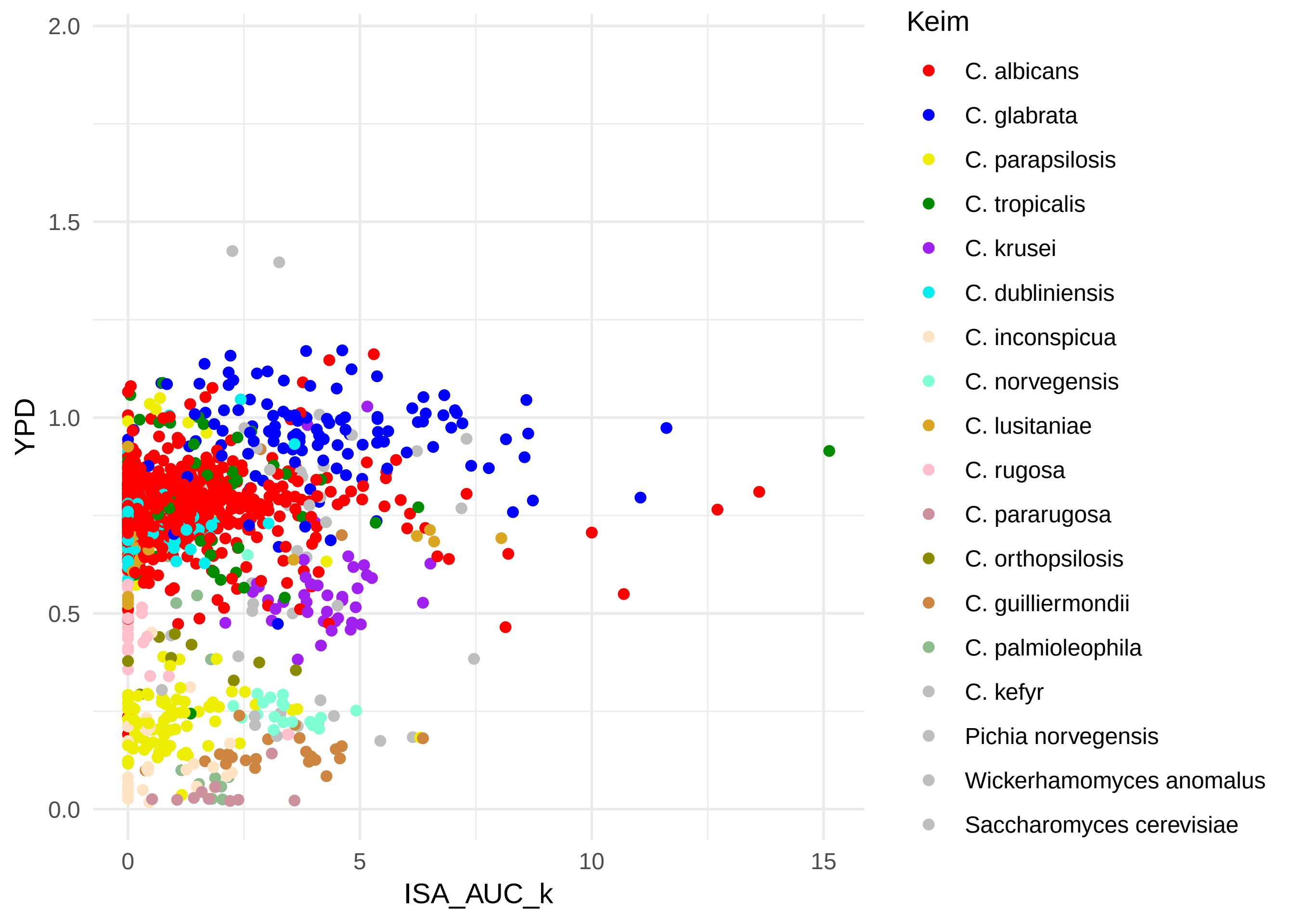

YPD
2.0
1.5
1.0
0.5
0.0
0
5
10
15
ISA_AUC_k
Keim
C. albicans
C. glabrata
C. parapsilosis
C. tropicalis
C. krusei
C. dubliniensis
C. inconspicua
C. norvegensis
C. lusitaniae
C. rugosa
C. pararugosa
C. orthopsilosis
C. guilliermondii
C. palmioleophila
C. kefyr
Pichia norvegensis
Wickerhamomyces anomalus
Saccharomyces cerevisiae

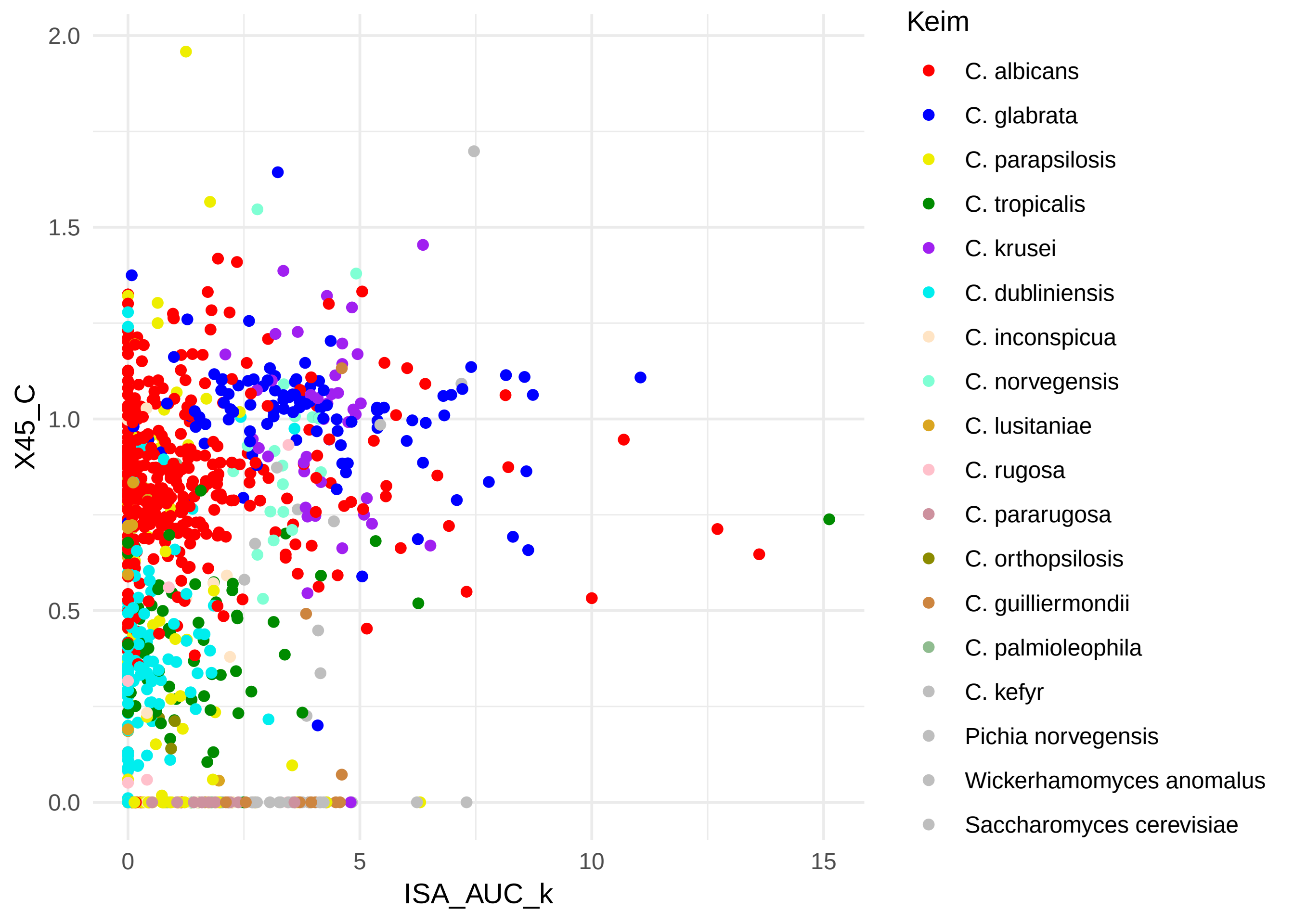

Keim
C. albicans
C. glabrata
C. parapsilosis
C. tropicalis
C. krusei
C. dubliniensis
C. inconspicua
C. norvegensis
C. lusitaniae
C. rugosa
C. pararugosa
C. orthopsilosis
C. guilliermondii
C. palmioleophila
C. kefyr
Pichia norvegensis
Wickerhamomyces anomalus
Saccharomyces cerevisiae
X45_C
ISA_AUC_k
2.0
1.5
1.0
0.5
0.0
0
5
10
15

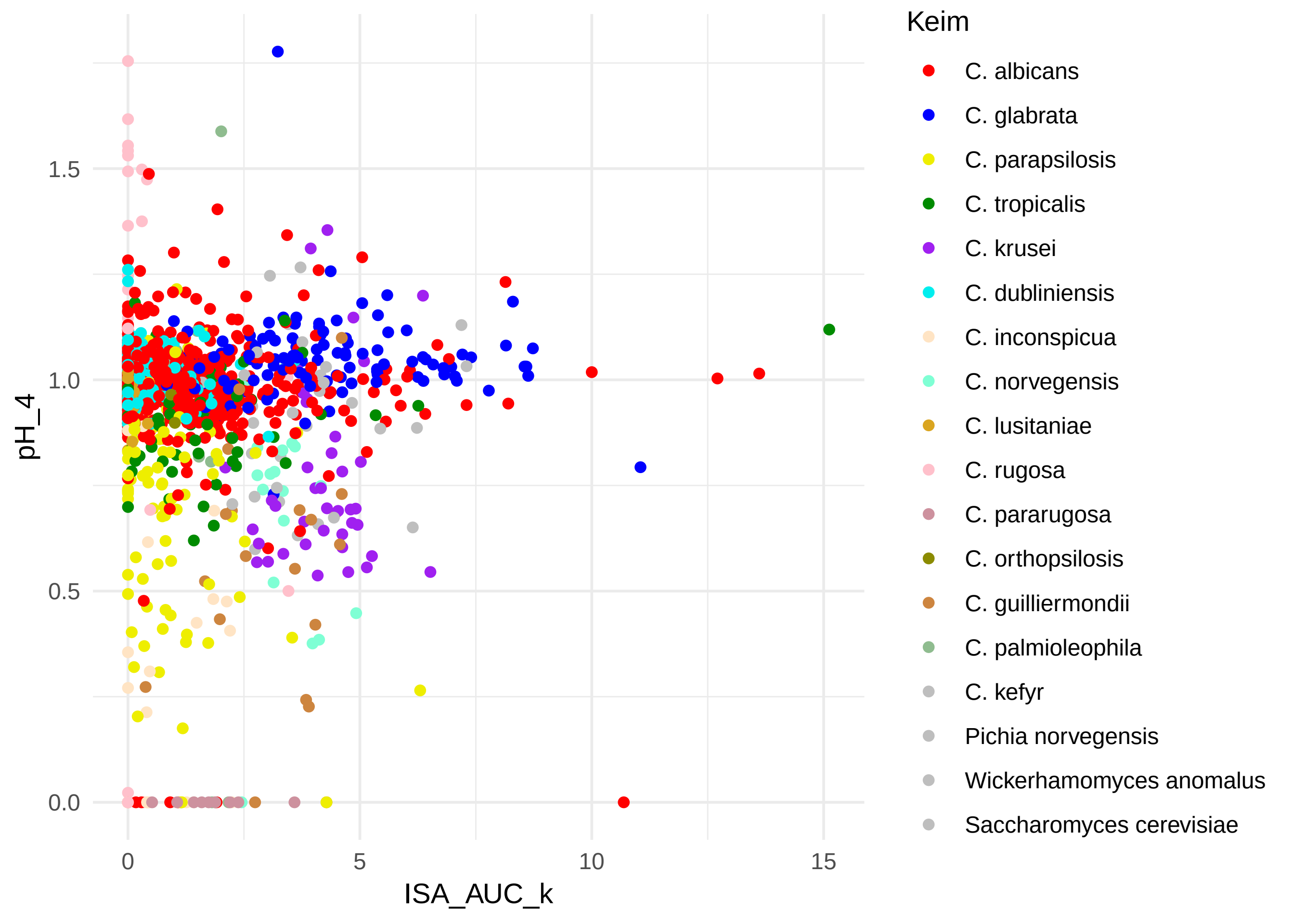

Keim
C. albicans
C. glabrata
C. parapsilosis
C. tropicalis
C. krusei
C. dubliniensis
C. inconspicua
C. norvegensis
C. lusitaniae
C. rugosa
C. pararugosa
C. orthopsilosis
C. guilliermondii
C. palmioleophila
C. kefyr
Pichia norvegensis
Wickerhamomyces anomalus
Saccharomyces cerevisiae
pH_4
ISA_AUC_k
0.0
0.5
1.0
1.5
0
5
10
15

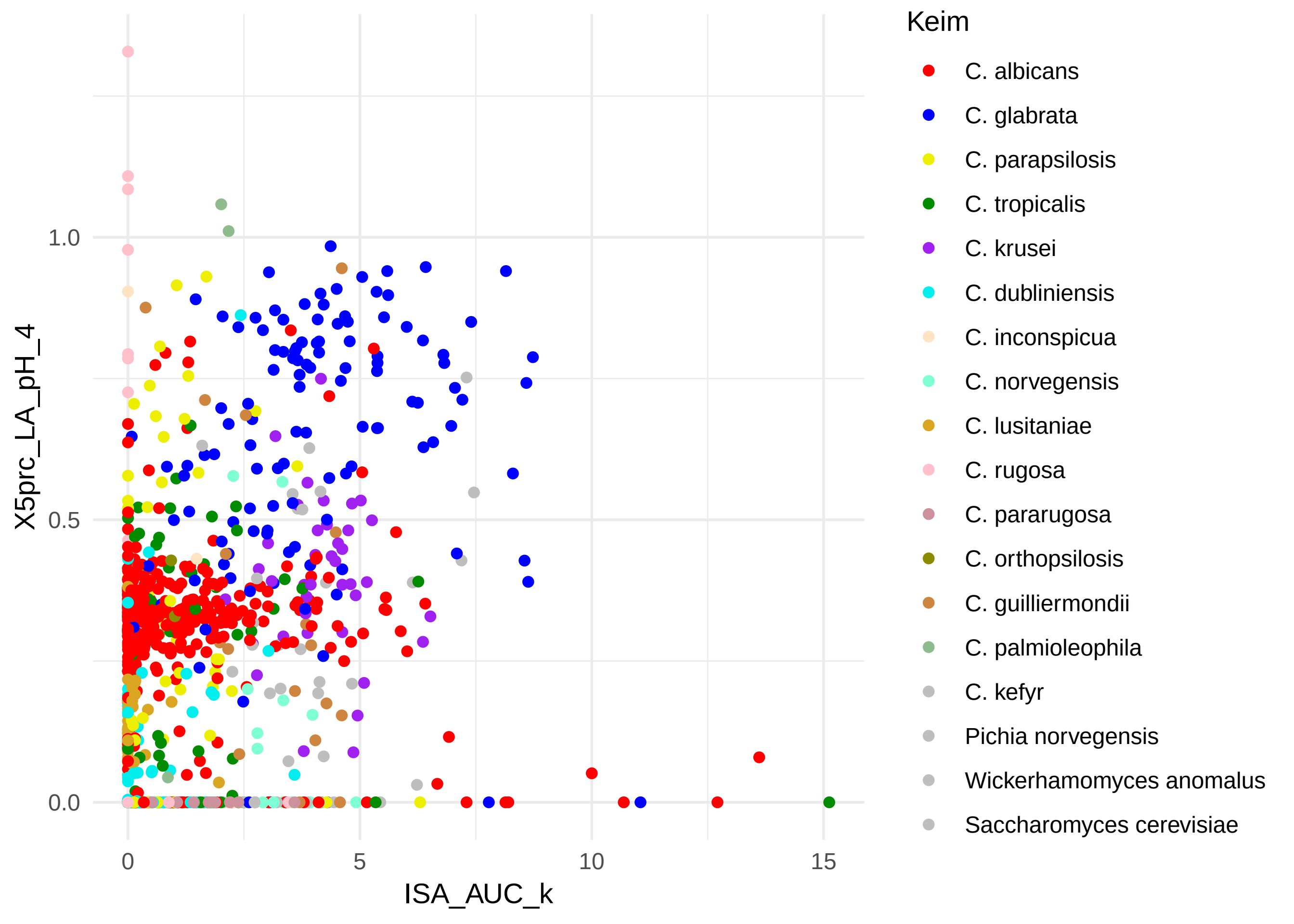
X5prc_LA_pH_4
1.0
0.5
0.0
0
5
10
15
ISA_AUC_k
Keim
C. albicans
C. glabrata
C. parapsilosis
C. tropicalis
C. krusei
C. dubliniensis
C. inconspicua
C. norvegensis
C. lusitaniae
C. rugosa
C. pararugosa
C. orthopsilosis
C. guilliermondii
C. palmioleophila
C. kefyr
Pichia norvegensis
Wickerhamomyces anomalus
Saccharomyces cerevisiae

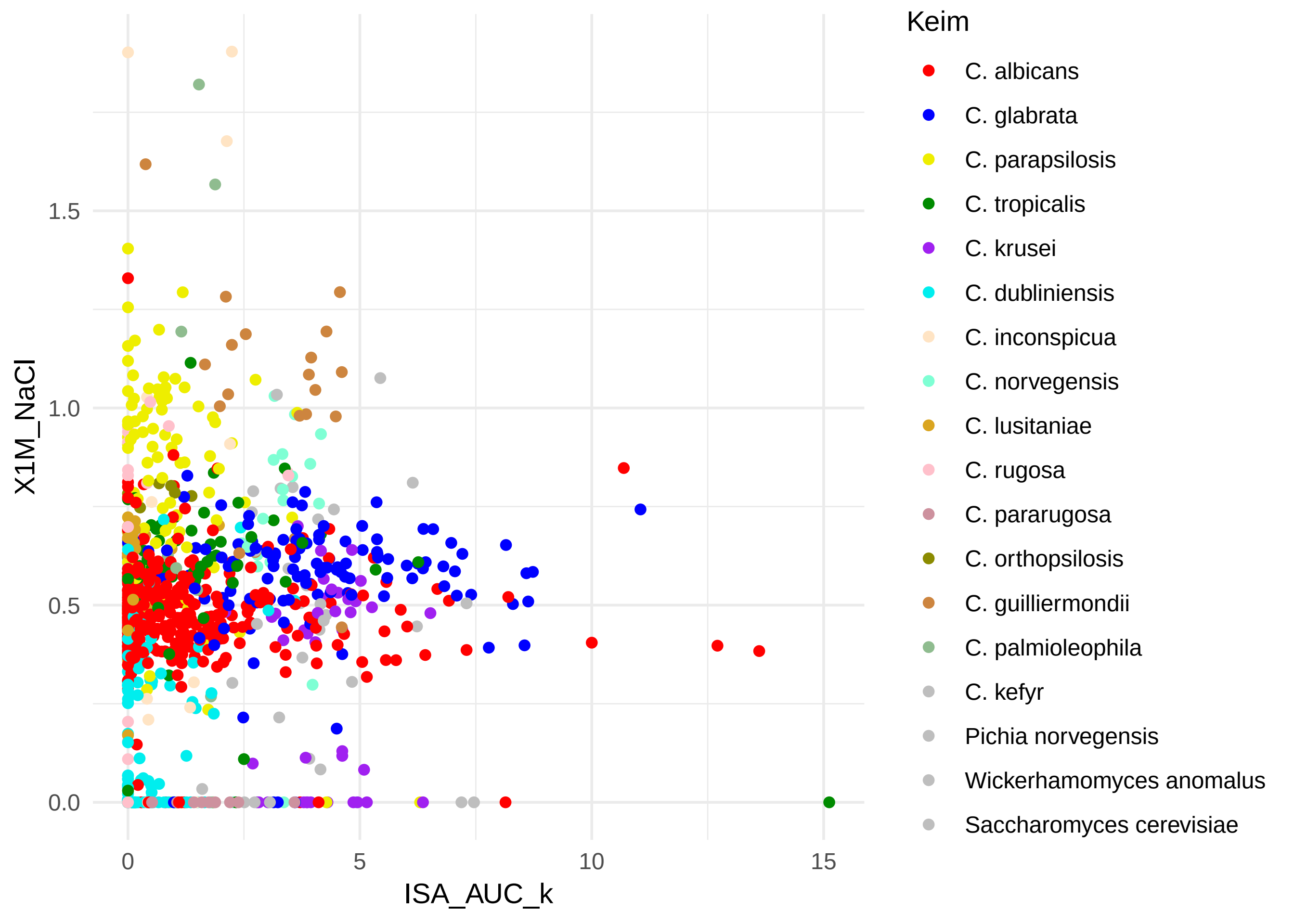
Keim
C. albicans
C. glabrata
C. parapsilosis
C. tropicalis
C. krusei
C. dubliniensis
C. inconspicua
C. norvegensis
C. lusitaniae
C. rugosa
C. pararugosa
C. orthopsilosis
C. guilliermondii
C. palmioleophila
C. kefyr
Pichia norvegensis
Wickerhamomyces anomalus
Saccharomyces cerevisiae
X1M_NaCl
1.5
1.0
0.5
0.0
0
5
10
15
ISA_AUC_k

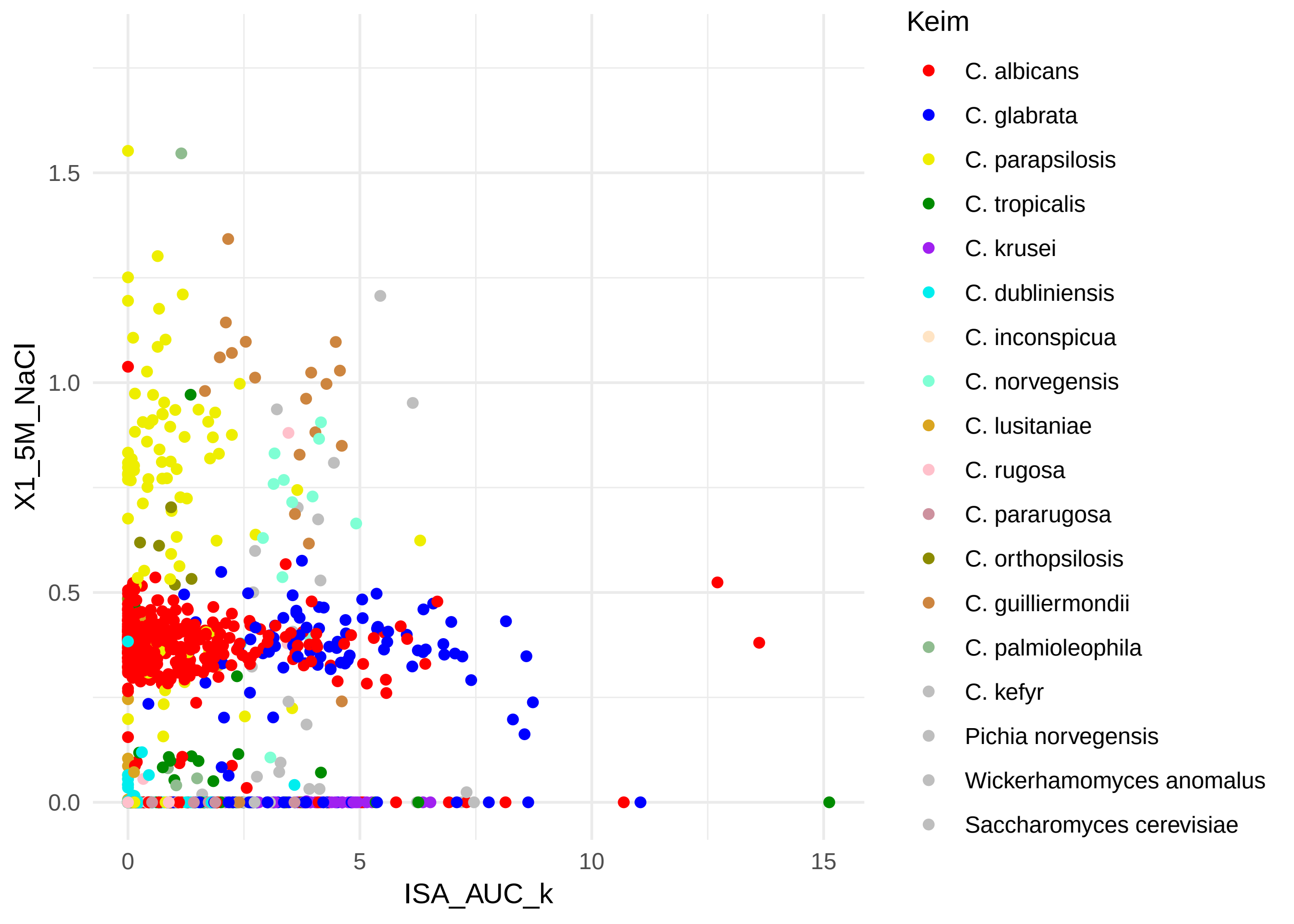
Keim
C. albicans
C. glabrata
C. parapsilosis
C. tropicalis
C. krusei
C. dubliniensis
C. inconspicua
C. norvegensis
C. lusitaniae
C. rugosa
C. pararugosa
C. orthopsilosis
C. guilliermondii
C. palmioleophila
C. kefyr
Pichia norvegensis
Wickerhamomyces anomalus
Saccharomyces cerevisiae
X1_5M_NaCl
1.5
1.0
0.5
0.0
0
5
10
15
ISA_AUC_k

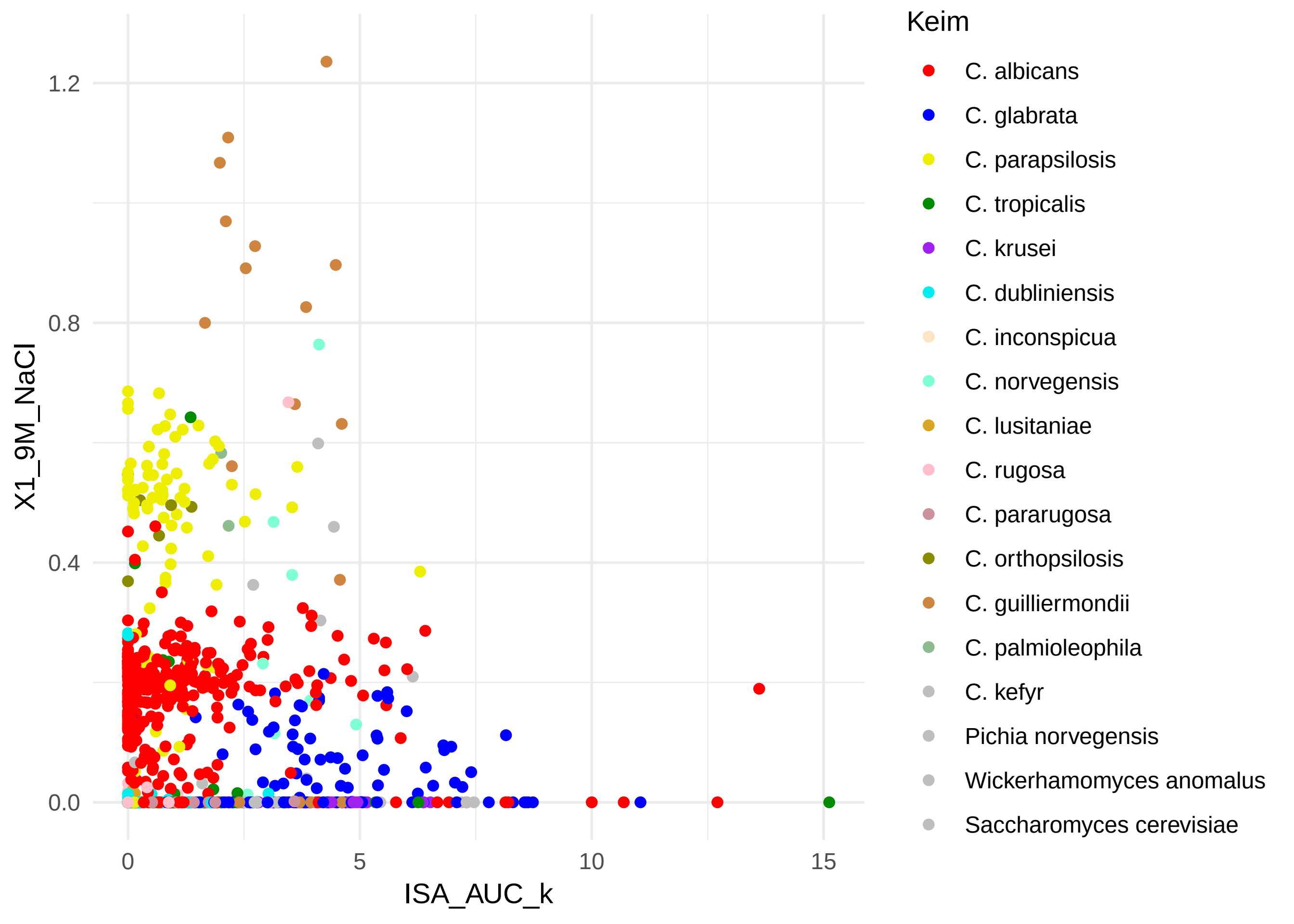

Keim
C. albicans
C. glabrata
C. parapsilosis
C. tropicalis
C. krusei
C. dubliniensis
C. inconspicua
C. norvegensis
C. lusitaniae
C. rugosa
C. pararugosa
C. orthopsilosis
C. guilliermondii
C. palmioleophila
C. kefyr
Pichia norvegensis
Wickerhamomyces anomalus
Saccharomyces cerevisiae
X1_9M_NaCl
1.2
0.8
0.4
0.0
0
5
10
15
ISA_AUC_k

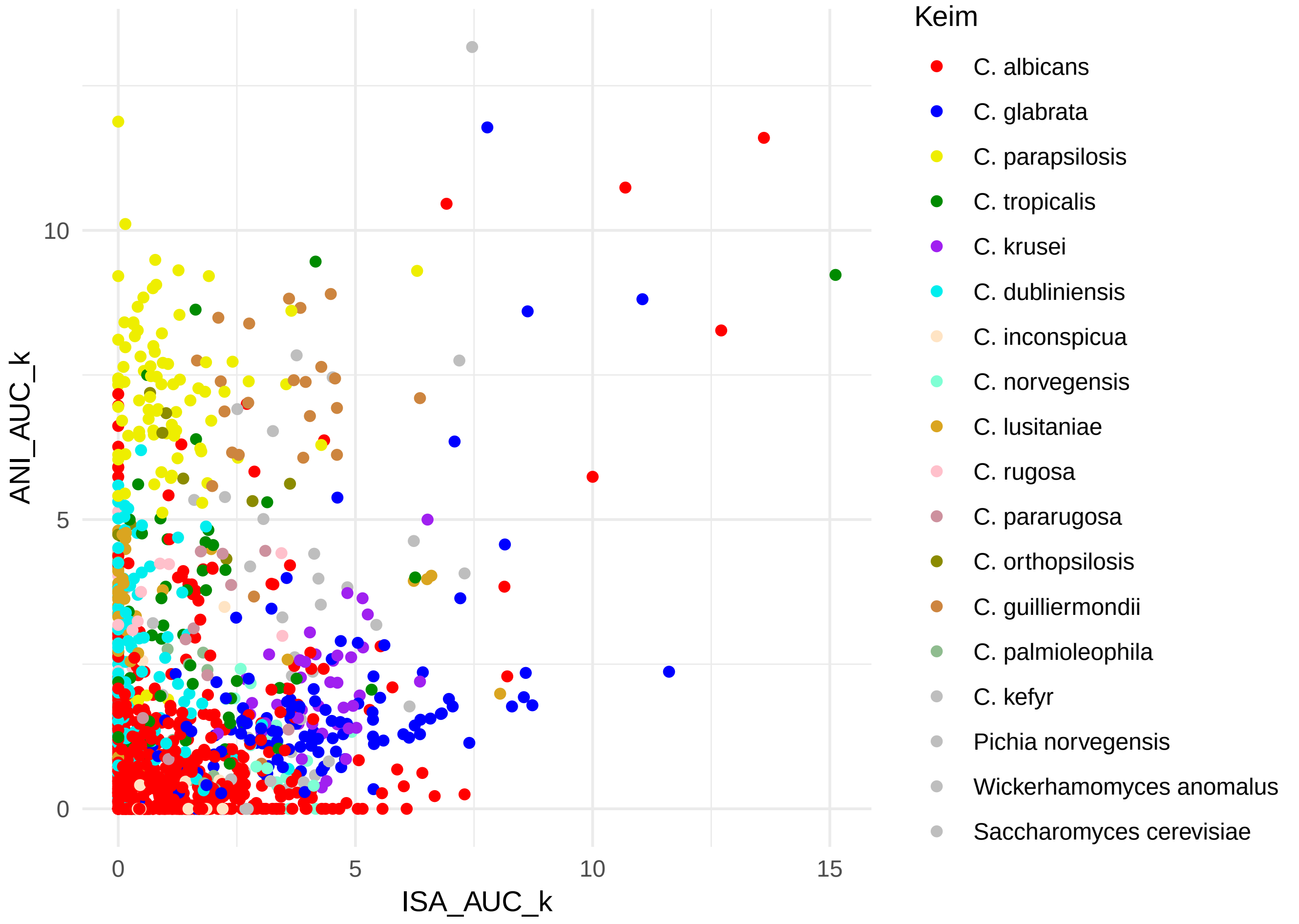

Keim
C. albicans
C. glabrata
C. parapsilosis
C. tropicalis
C. krusei
C. dubliniensis
C. inconspicua
C. norvegensis
C. lusitaniae
C. rugosa
C. pararugosa
C. orthopsilosis
C. guilliermondii
C. palmioleophila
C. kefyr
Pichia norvegensis
Wickerhamomyces anomalus
Saccharomyces cerevisiae
ANI_AUC_k
10
5
0
0
5
10
15
ISA_AUC_k

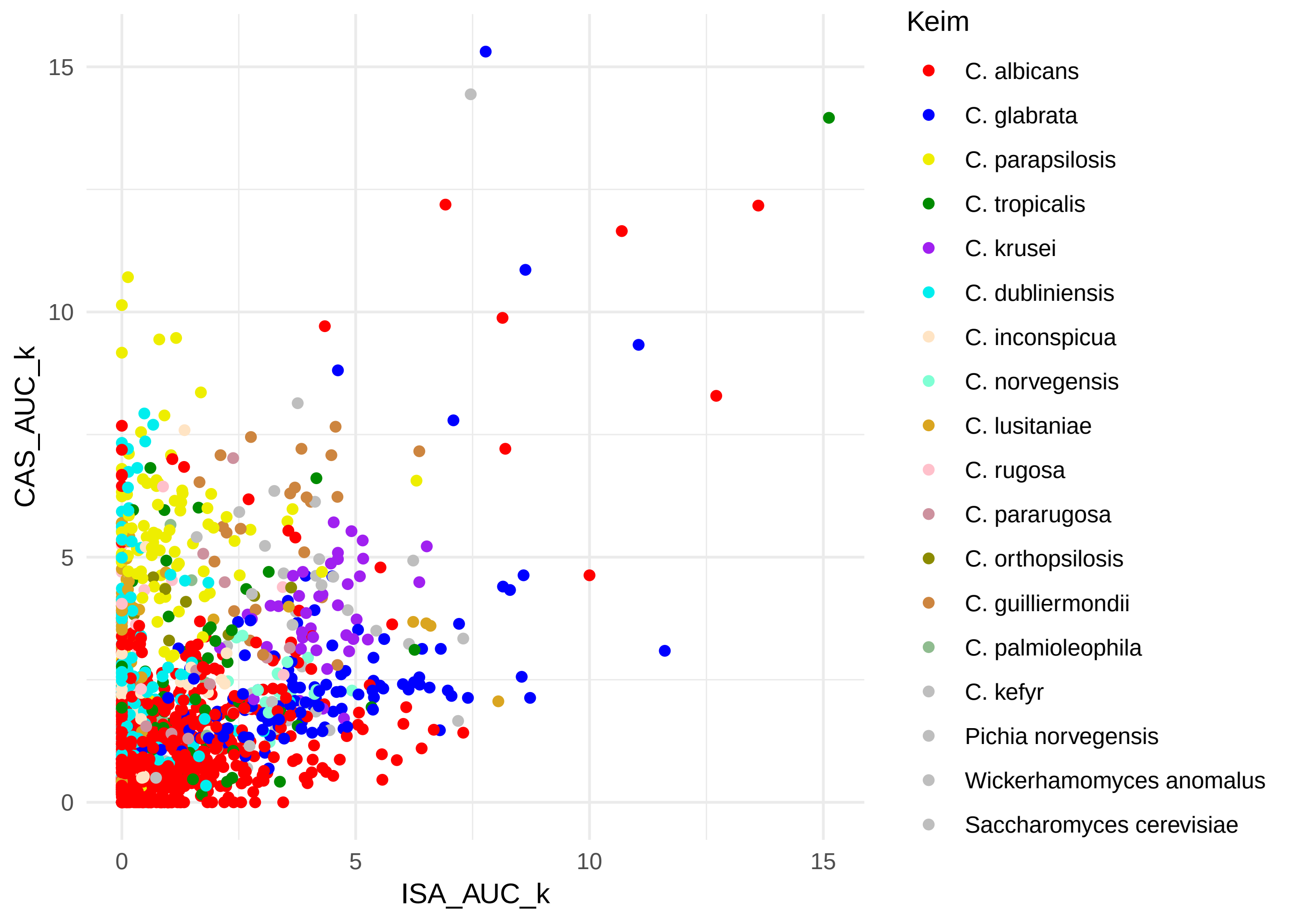
Keim
C. albicans
C. glabrata
C. parapsilosis
C. tropicalis
C. krusei
C. dubliniensis
C. inconspicua
C. norvegensis
C. lusitaniae
C. rugosa
C. pararugosa
C. orthopsilosis
C. guilliermondii
C. palmioleophila
C. kefyr
Pichia norvegensis
Wickerhamomyces anomalus
Saccharomyces cerevisiae
CAS_AUC_k
ISA_AUC_k
0
5
10
15

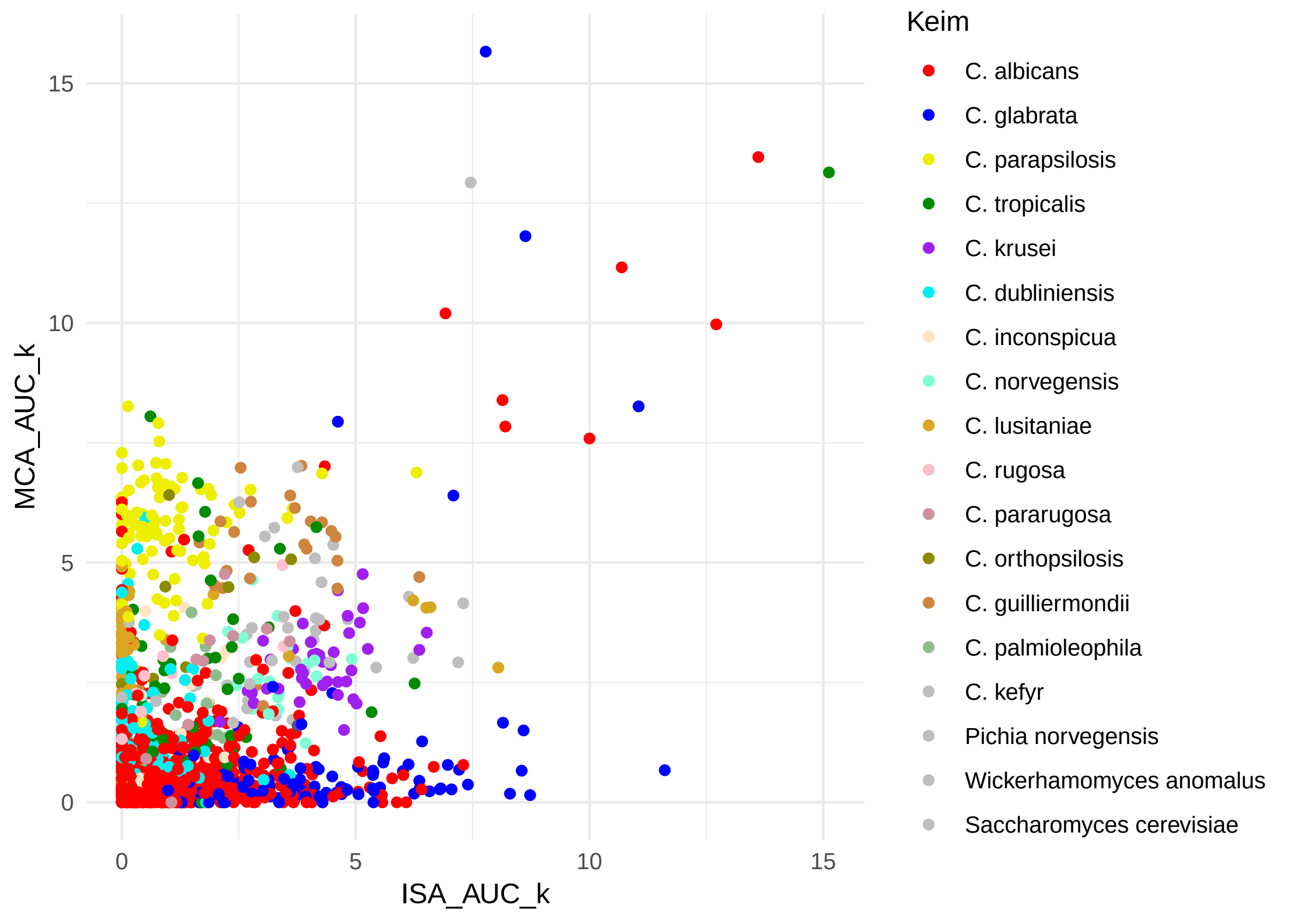

Keim
C. albicans
C. glabrata
C. parapsilosis
C. tropicalis
C. krusei
C. dubliniensis
C. inconspicua
C. norvegensis
C. lusitaniae
C. rugosa
C. pararugosa
C. orthopsilosis
C. guilliermondii
C. palmioleophila
C. kefyr
Pichia norvegensis
Wickerhamomyces anomalus
Saccharomyces cerevisiae
MCA_AUC_k
ISA_AUC_k
0
5
10
15

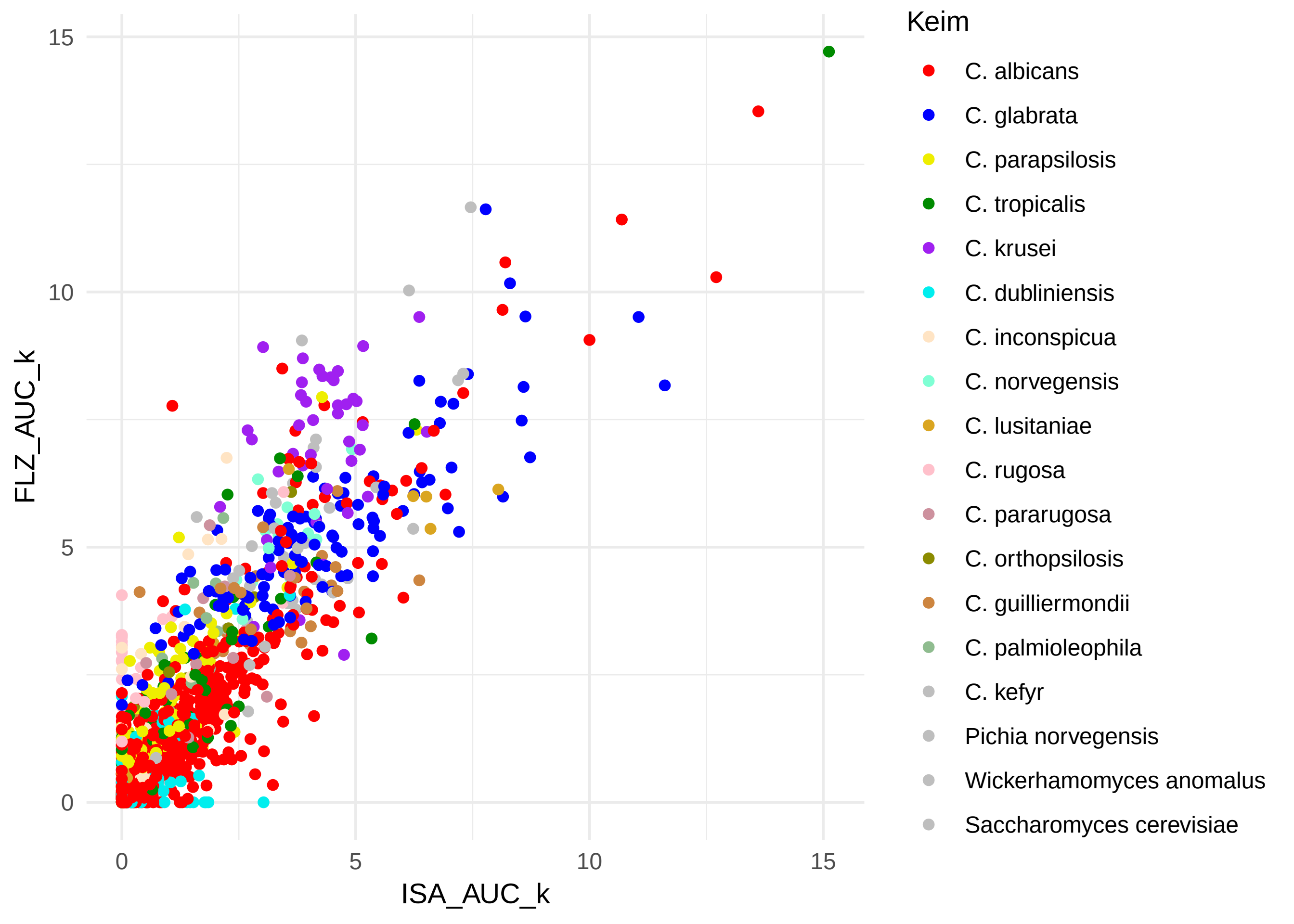

15
10
5
0
FLZ_AUC_k
0
5
10
15
ISA_AUC_k
Keim
C. albicans
C. glabrata
C. parapsilosis
C. tropicalis
C. krusei
C. dubliniensis
C. inconspicua
C. norvegensis
C. lusitaniae
C. rugosa
C. pararugosa
C. orthopsilosis
C. guilliermondii
C. palmioleophila
C. kefyr
Pichia norvegensis
Wickerhamomyces anomalus
Saccharomyces cerevisiae

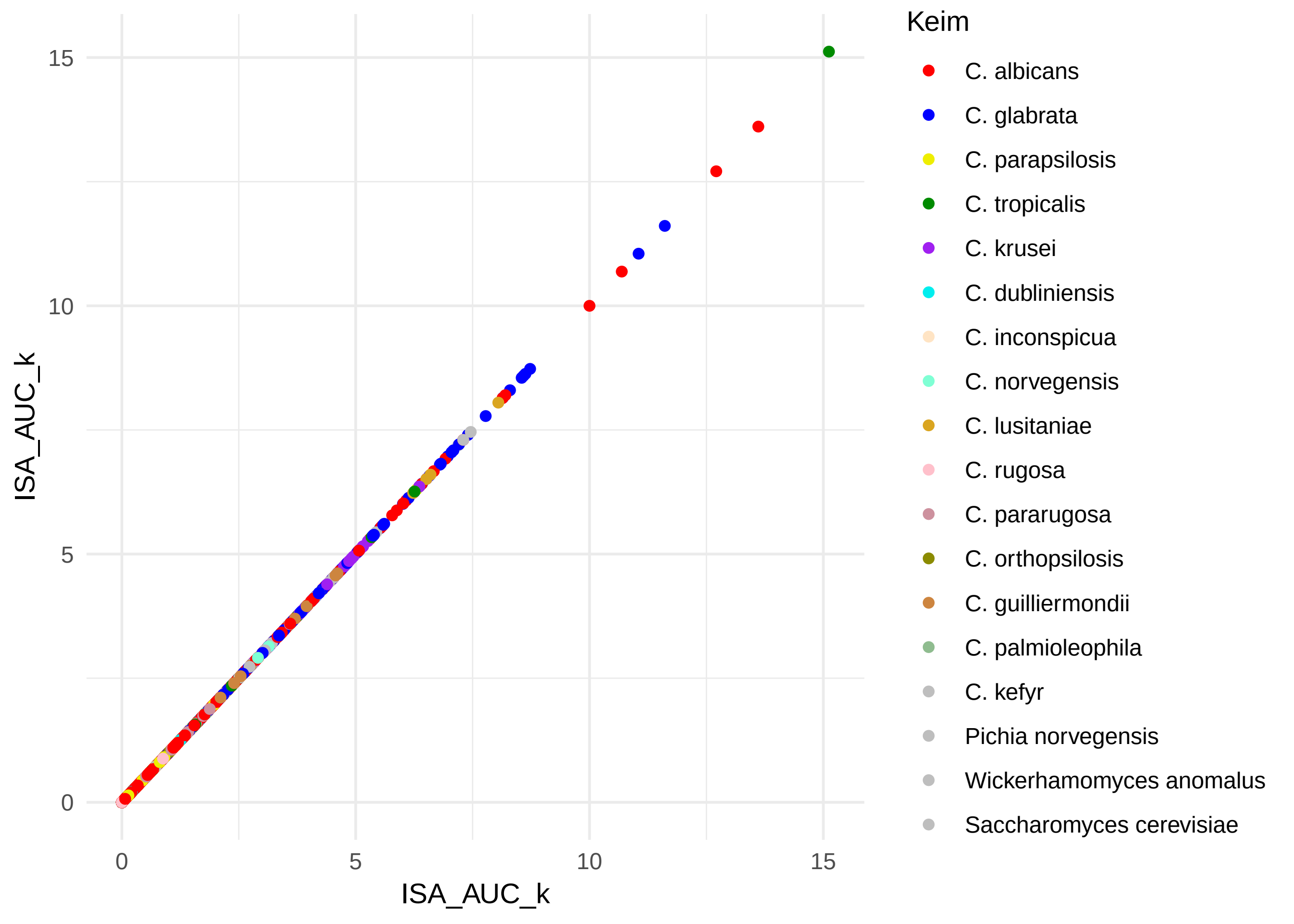
ISA_AUC_k
0
5
10
15
0
5
10
15
ISA_AUC_k
Keim
C. albicans
C. glabrata
C. parapsilosis
C. tropicalis
C. krusei
C. dubliniensis
C. inconspicua
C. norvegensis
C. lusitaniae
C. rugosa
C. pararugosa
C. orthopsilosis
C. guilliermondii
C. palmioleophila
C. kefyr
Pichia norvegensis
Wickerhamomyces anomalus
Saccharomyces cerevisiae

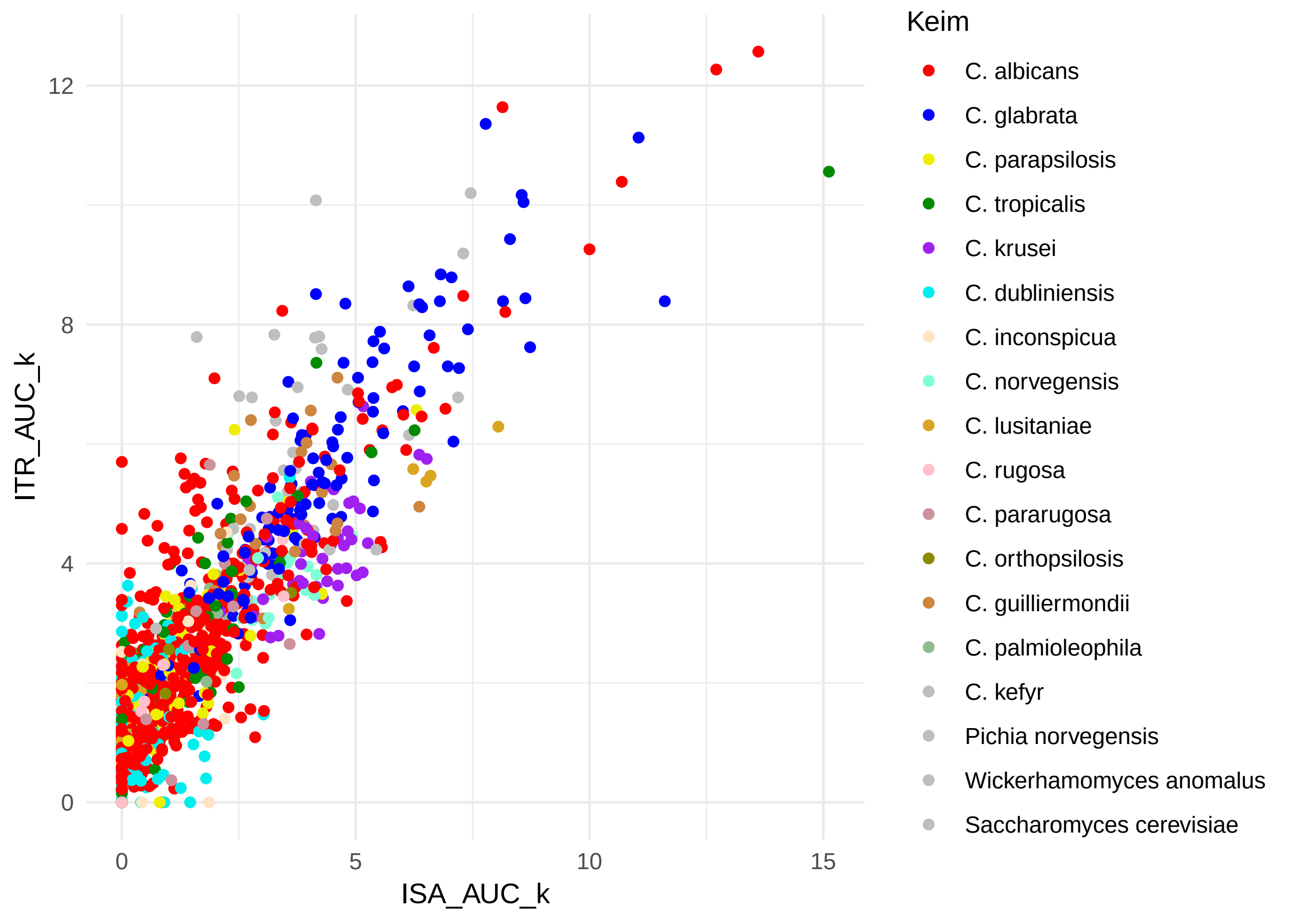
Keim
C. albicans
C. glabrata
C. parapsilosis
C. tropicalis
C. krusei
C. dubliniensis
C. inconspicua
C. norvegensis
C. lusitaniae
C. rugosa
C. pararugosa
C. orthopsilosis
C. guilliermondii
C. palmioleophila
C. kefyr
Pichia norvegensis
Wickerhamomyces anomalus
Saccharomyces cerevisiae
ITR_AUC_k
ISA_AUC_k
0
4
8
12
0
5
10
15

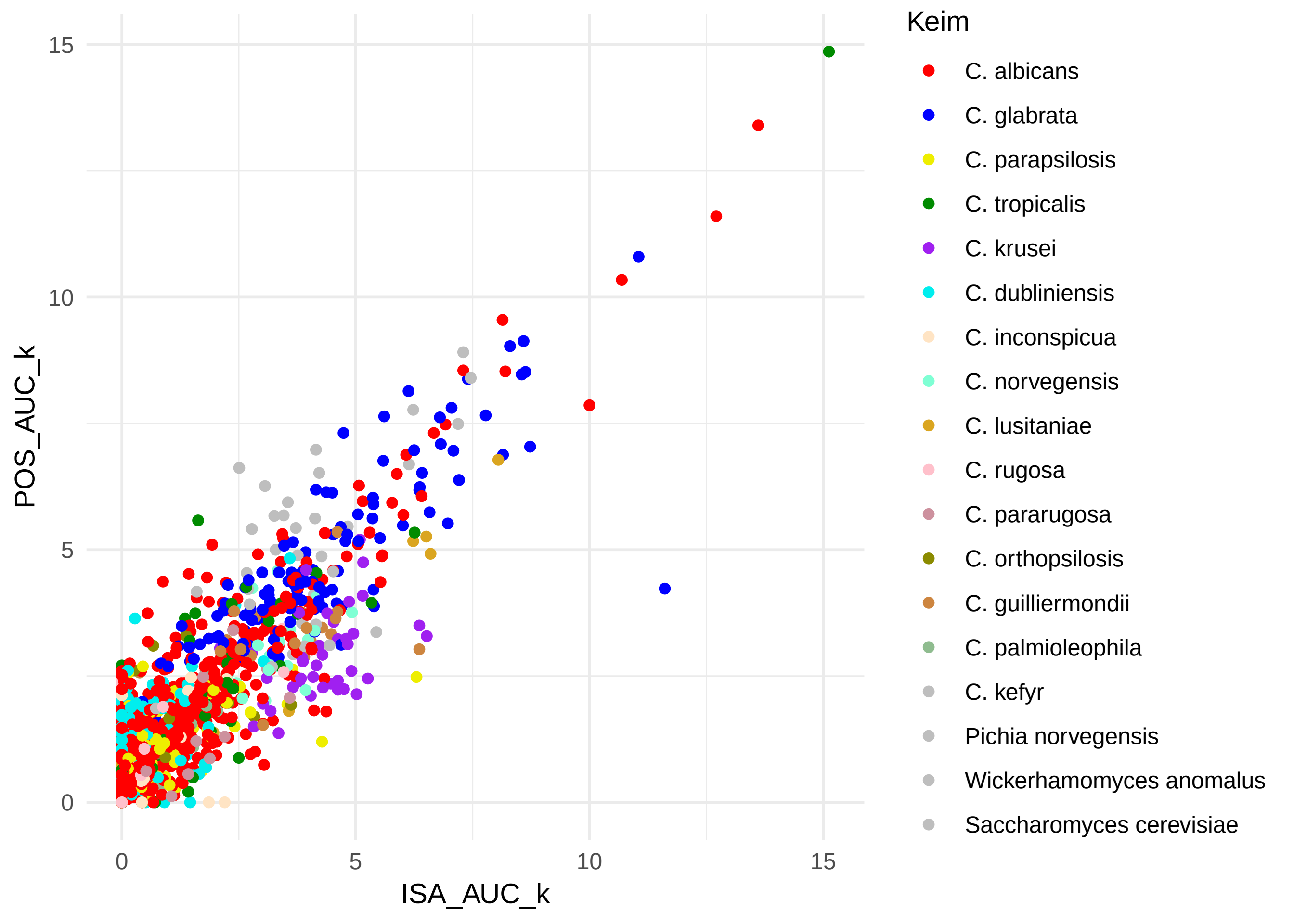

15
10
5
0
POS_AUC_k
0
5
10
15
ISA_AUC_k
Keim
C. albicans
C. glabrata
C. parapsilosis
C. tropicalis
C. krusei
C. dubliniensis
C. inconspicua
C. norvegensis
C. lusitaniae
C. rugosa
C. pararugosa
C. orthopsilosis
C. guilliermondii
C. palmioleophila
C. kefyr
Pichia norvegensis
Wickerhamomyces anomalus
Saccharomyces cerevisiae

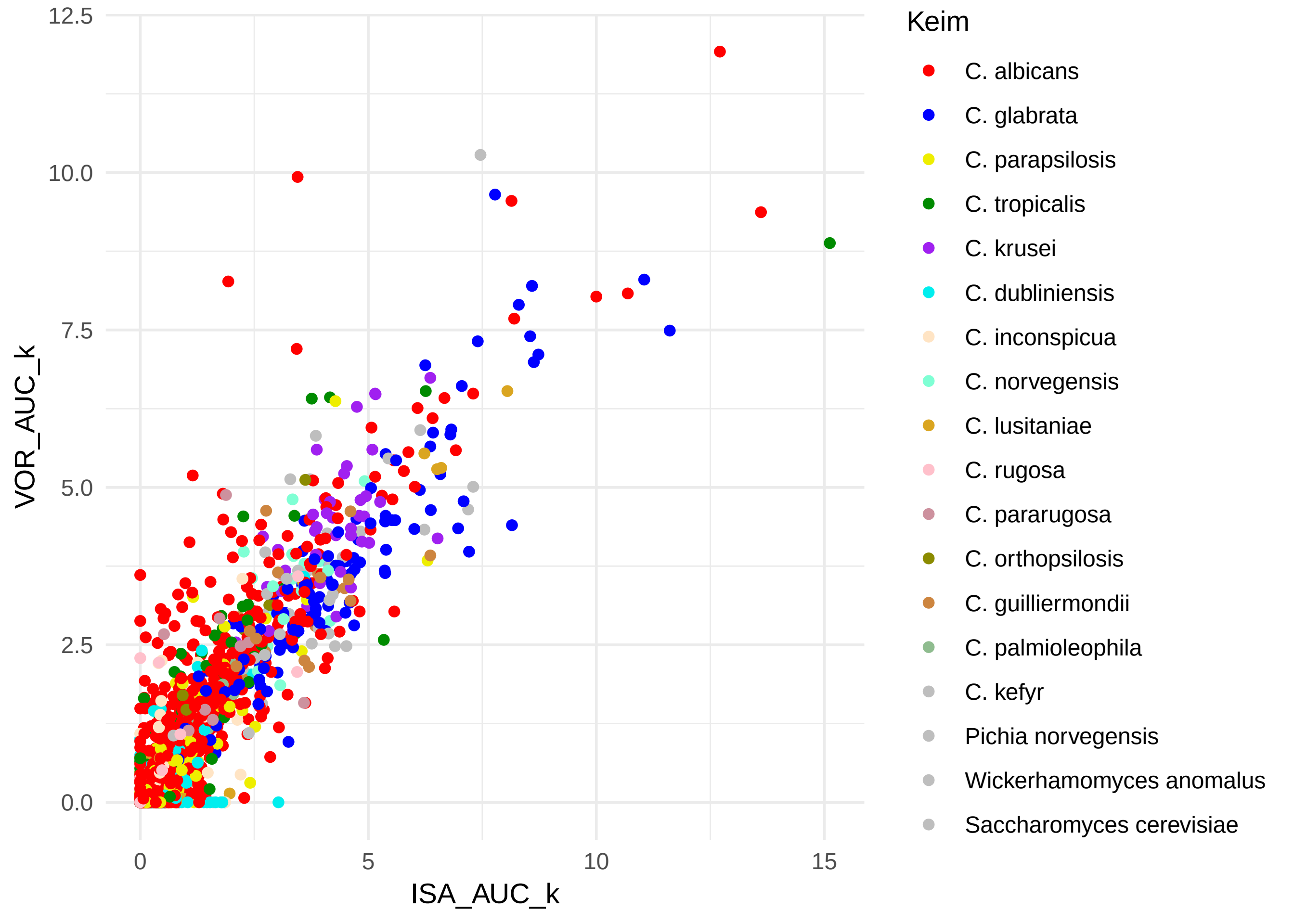

12.5
10.0
7.5
5.0
2.5
0.0
VOR_AUC_k
0
5
10
15
ISA_AUC_k
Keim
C. albicans
C. glabrata
C. parapsilosis
C. tropicalis
C. krusei
C. dubliniensis
C. inconspicua
C. norvegensis
C. lusitaniae
C. rugosa
C. pararugosa
C. orthopsilosis
C. guilliermondii
C. palmioleophila
C. kefyr
Pichia norvegensis
Wickerhamomyces anomalus
Saccharomyces cerevisiae

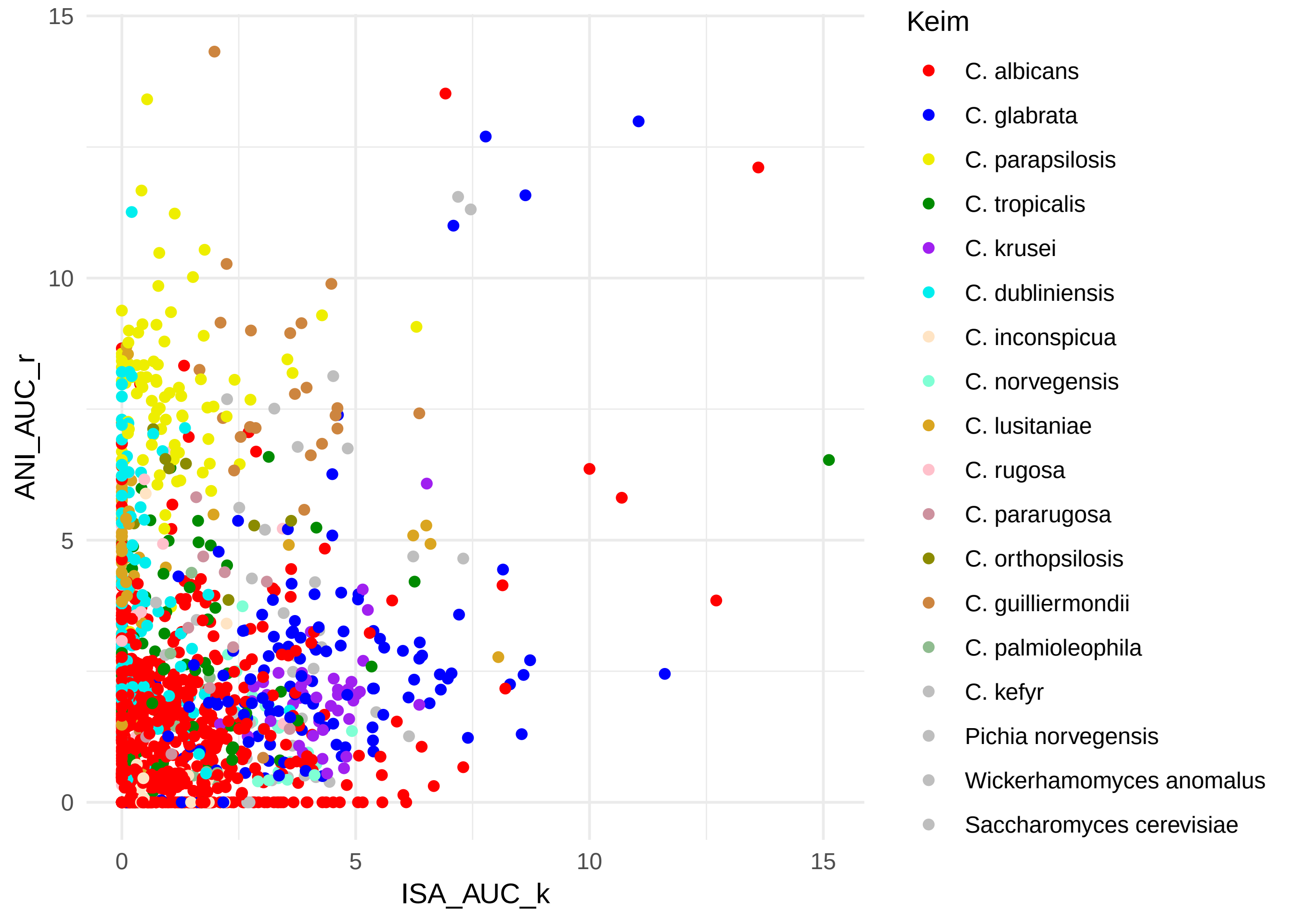
ANI_AUC_r
ISA_AUC_k
0
5
10
15
0
5
10
15
Keim
C. albicans
C. glabrata
C. parapsilosis
C. tropicalis
C. krusei
C. dubliniensis
C. inconspicua
C. norvegensis
C. lusitaniae
C. rugosa
C. pararugosa
C. orthopsilosis
C. guilliermondii
C. palmioleophila
C. kefyr
Pichia norvegensis
Wickerhamomyces anomalus
Saccharomyces cerevisiae

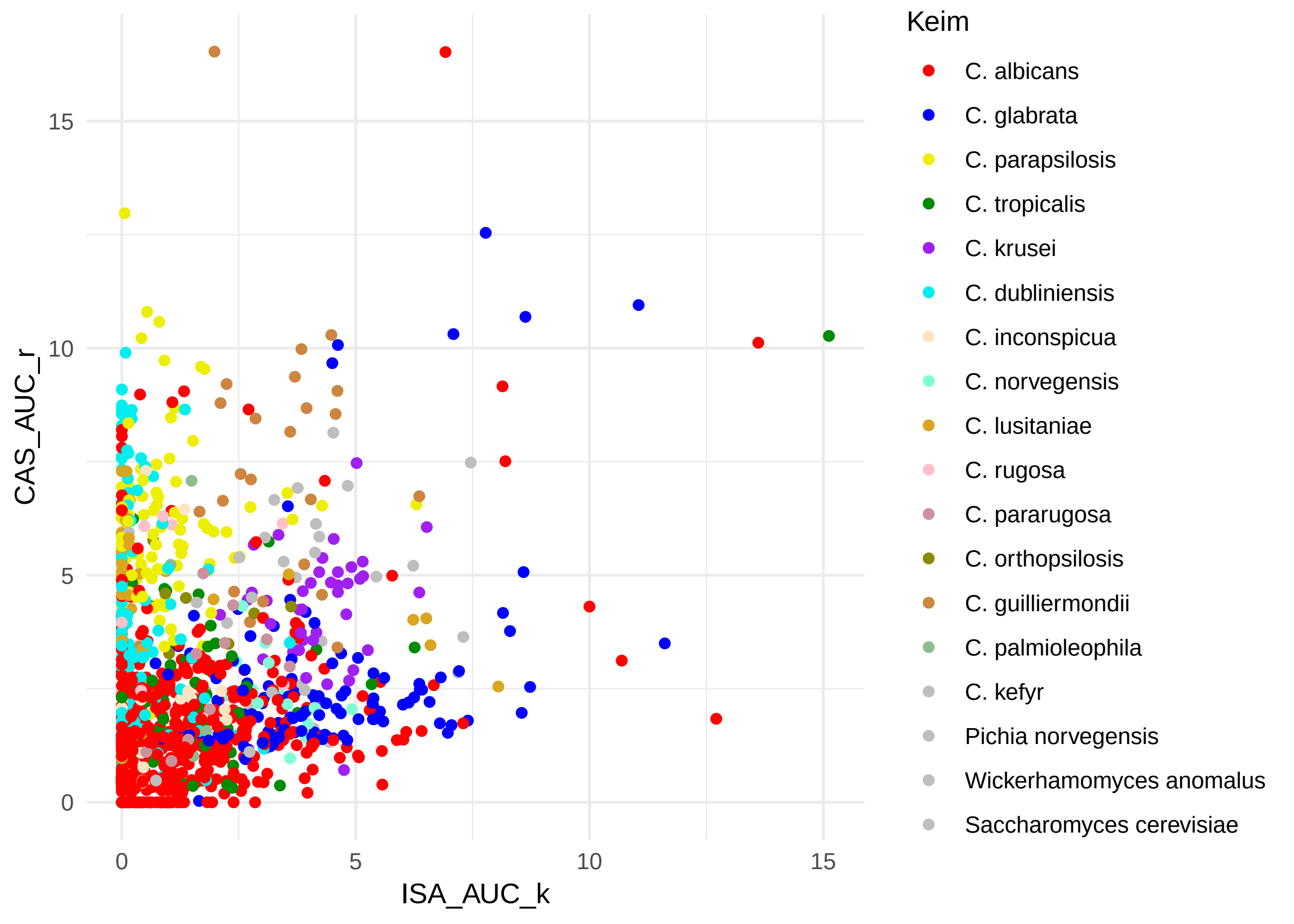
CAS_AUC_r
ISA_AUC_k
0
5
10
15
0
5
10
15
Keim
C. albicans
C. glabrata
C. parapsilosis
C. tropicalis
C. krusei
C. dubliniensis
C. inconspicua
C. norvegensis
C. lusitaniae
C. rugosa
C. pararugosa
C. orthopsilosis
C. guilliermondii
C. palmioleophila
C. kefyr
Pichia norvegensis
Wickerhamomyces anomalus
Saccharomyces cerevisiae

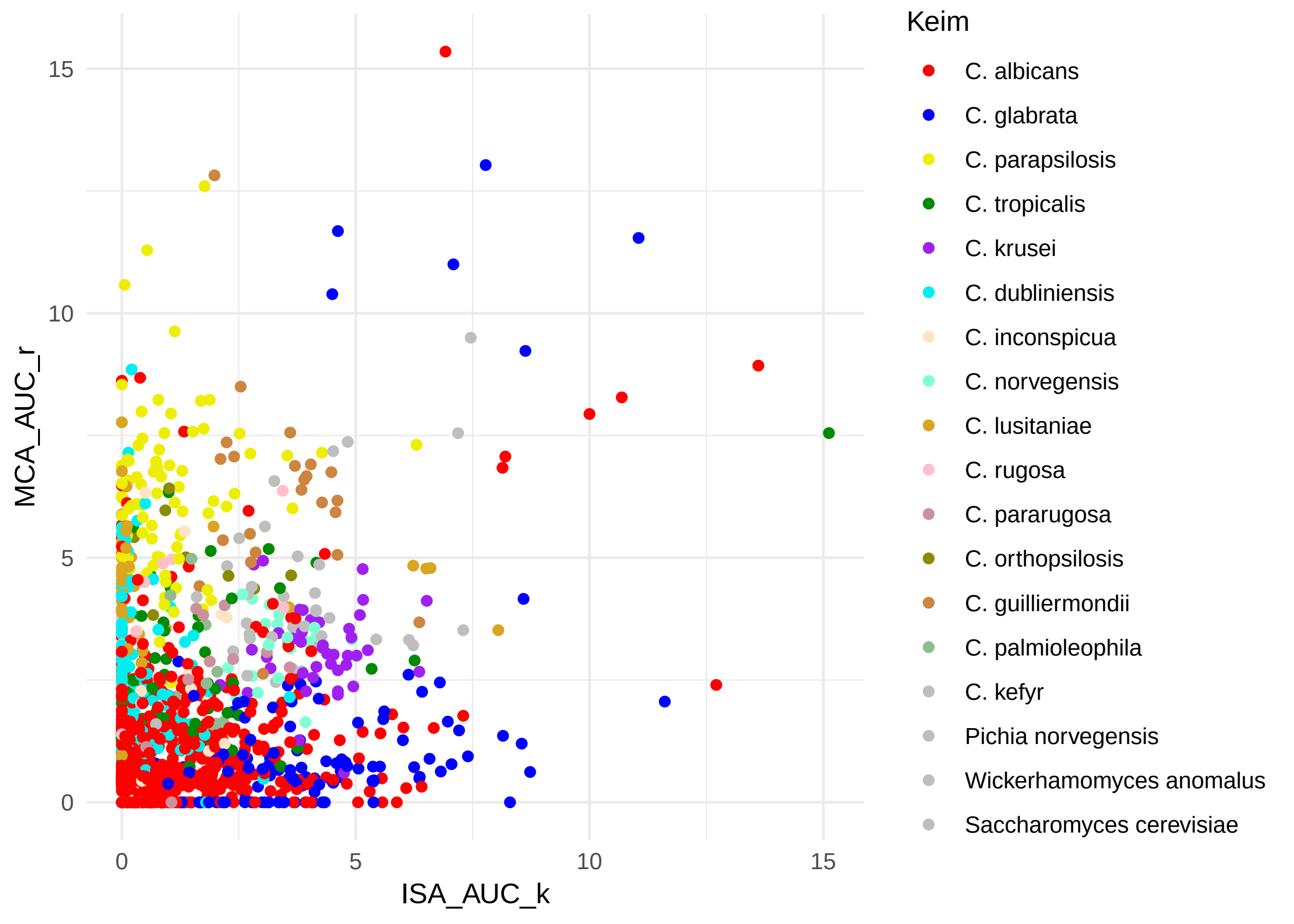

Keim
C. albicans
C. glabrata
C. parapsilosis
C. tropicalis
C. krusei
C. dubliniensis
C. inconspicua
C. norvegensis
C. lusitaniae
C. rugosa
C. pararugosa
C. orthopsilosis
C. guilliermondii
C. palmioleophila
C. kefyr
Pichia norvegensis
Wickerhamomyces anomalus
Saccharomyces cerevisiae
MCA_AUC_r
ISA_AUC_k
0
5
10
15

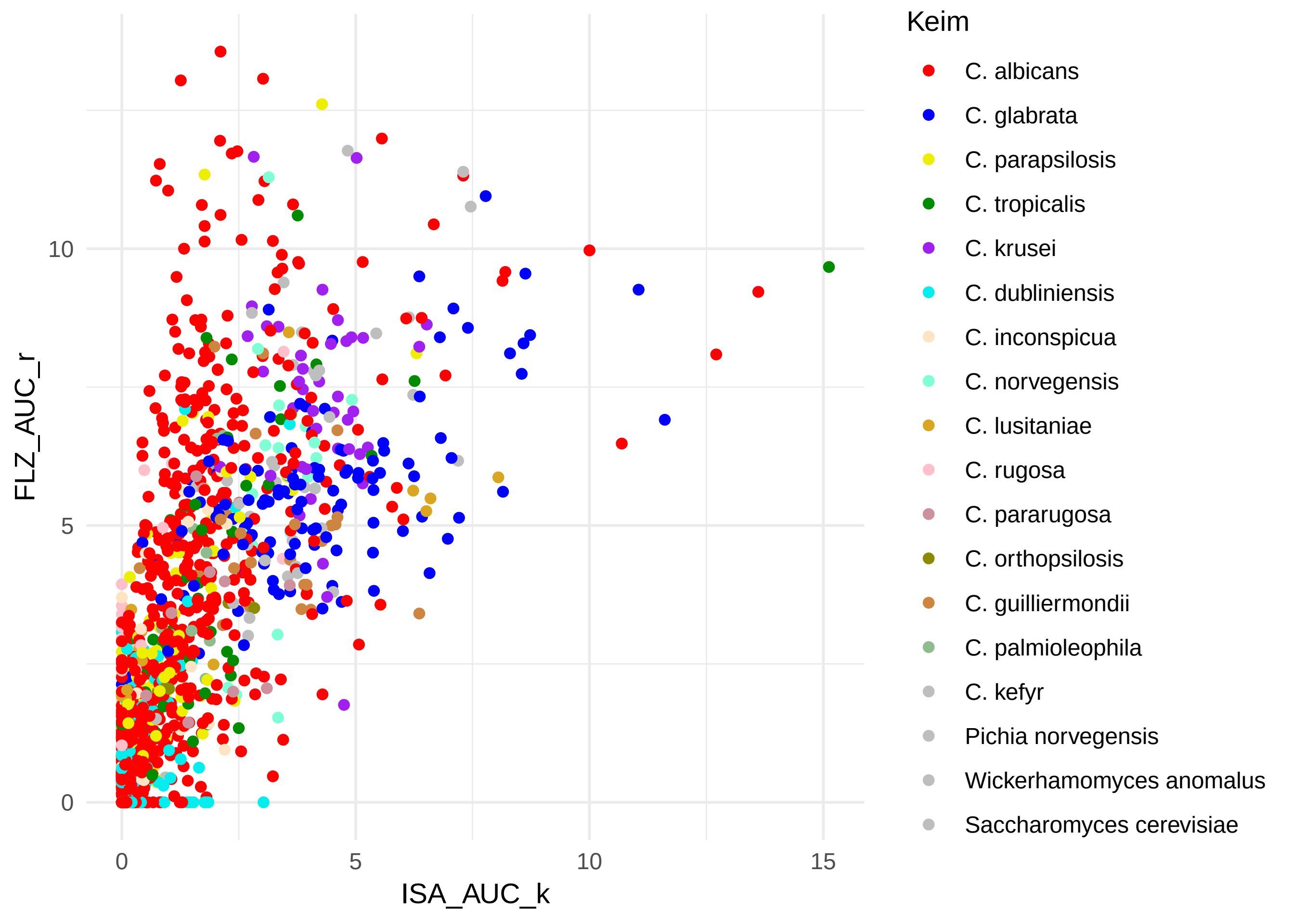

Keim
C. albicans
C. glabrata
C. parapsilosis
C. tropicalis
C. krusei
C. dubliniensis
C. inconspicua
C. norvegensis
C. lusitaniae
C. rugosa
C. pararugosa
C. orthopsilosis
C. guilliermondii
C. palmioleophila
C. kefyr
Pichia norvegensis
Wickerhamomyces anomalus
Saccharomyces cerevisiae
FLZ_AUC_r
ISA_AUC_k
0
5
10
15

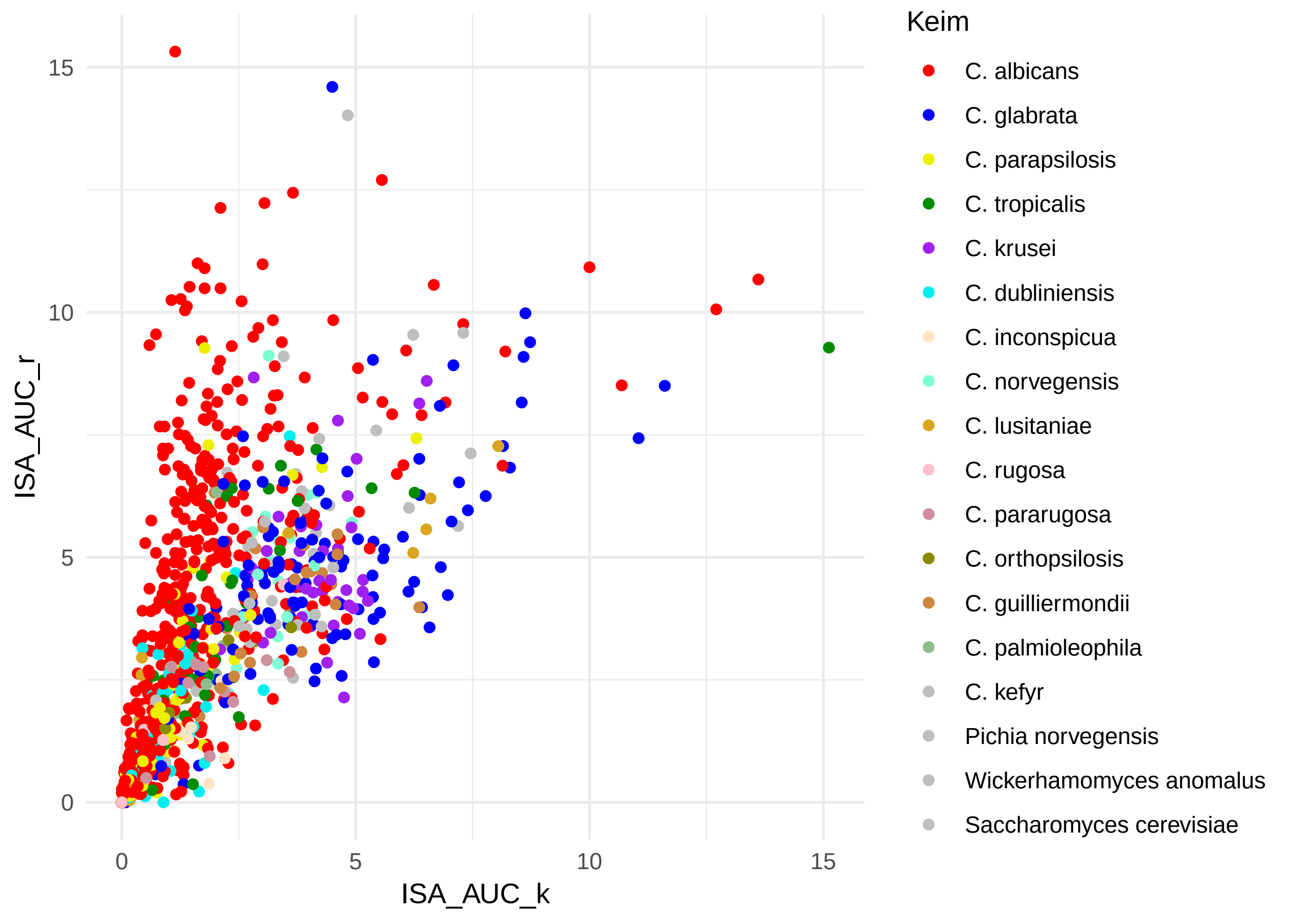
ISA_AUC_r
ISA_AUC_k
0
5
10
15
0
5
10
15
Keim
C. albicans
C. glabrata
C. parapsilosis
C. tropicalis
C. krusei
C. dubliniensis
C. inconspicua
C. norvegensis
C. lusitaniae
C. rugosa
C. pararugosa
C. orthopsilosis
C. guilliermondii
C. palmioleophila
C. kefyr
Pichia norvegensis
Wickerhamomyces anomalus
Saccharomyces cerevisiae

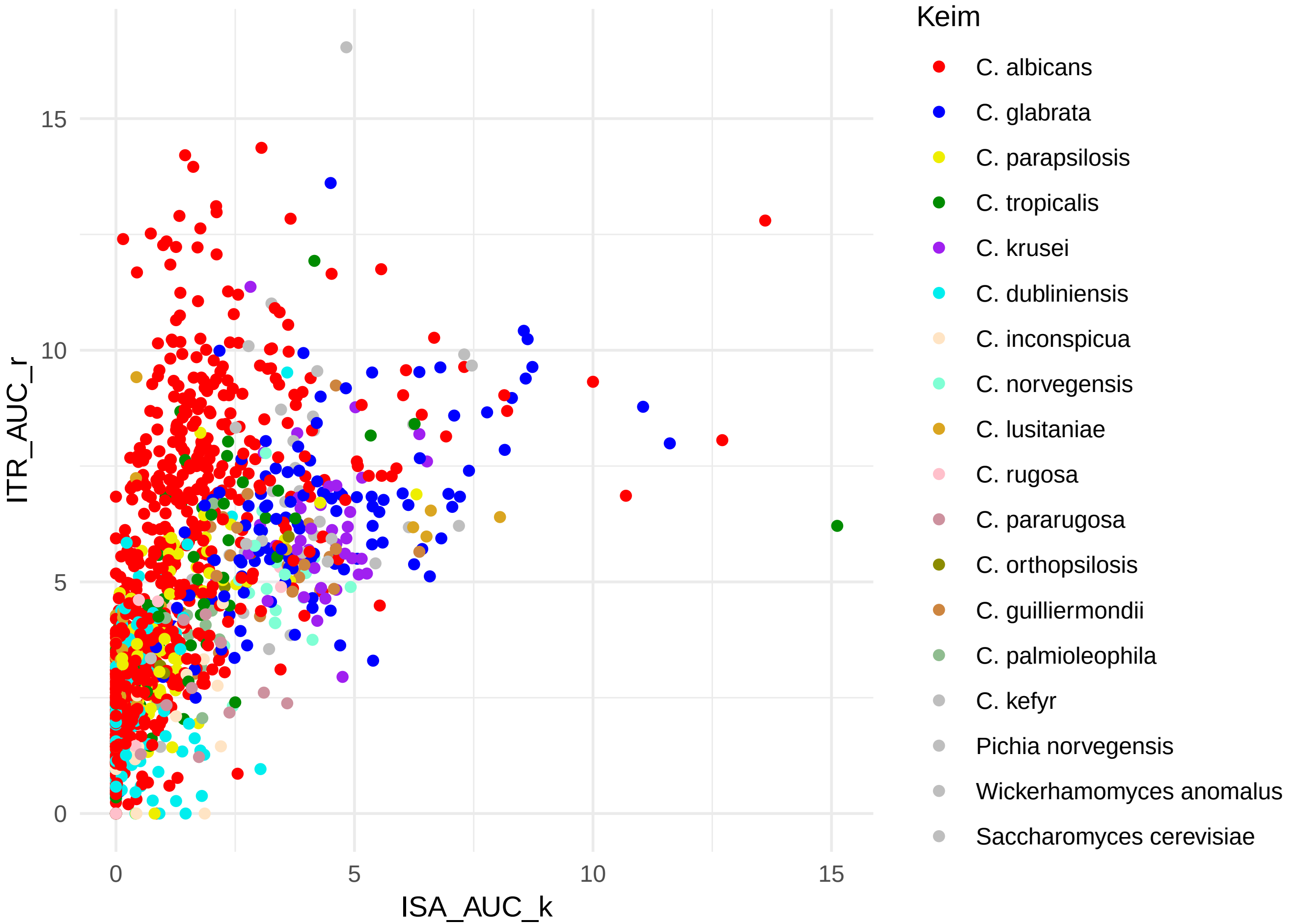

Keim
C. albicans
C. glabrata
C. parapsilosis
C. tropicalis
C. krusei
C. dubliniensis
C. inconspicua
C. norvegensis
C. lusitaniae
C. rugosa
C. pararugosa
C. orthopsilosis
C. guilliermondii
C. palmioleophila
C. kefyr
Pichia norvegensis
Wickerhamomyces anomalus
Saccharomyces cerevisiae
ITR_AUC_r
ISA_AUC_k
0
5
10
15
0
5
10
15

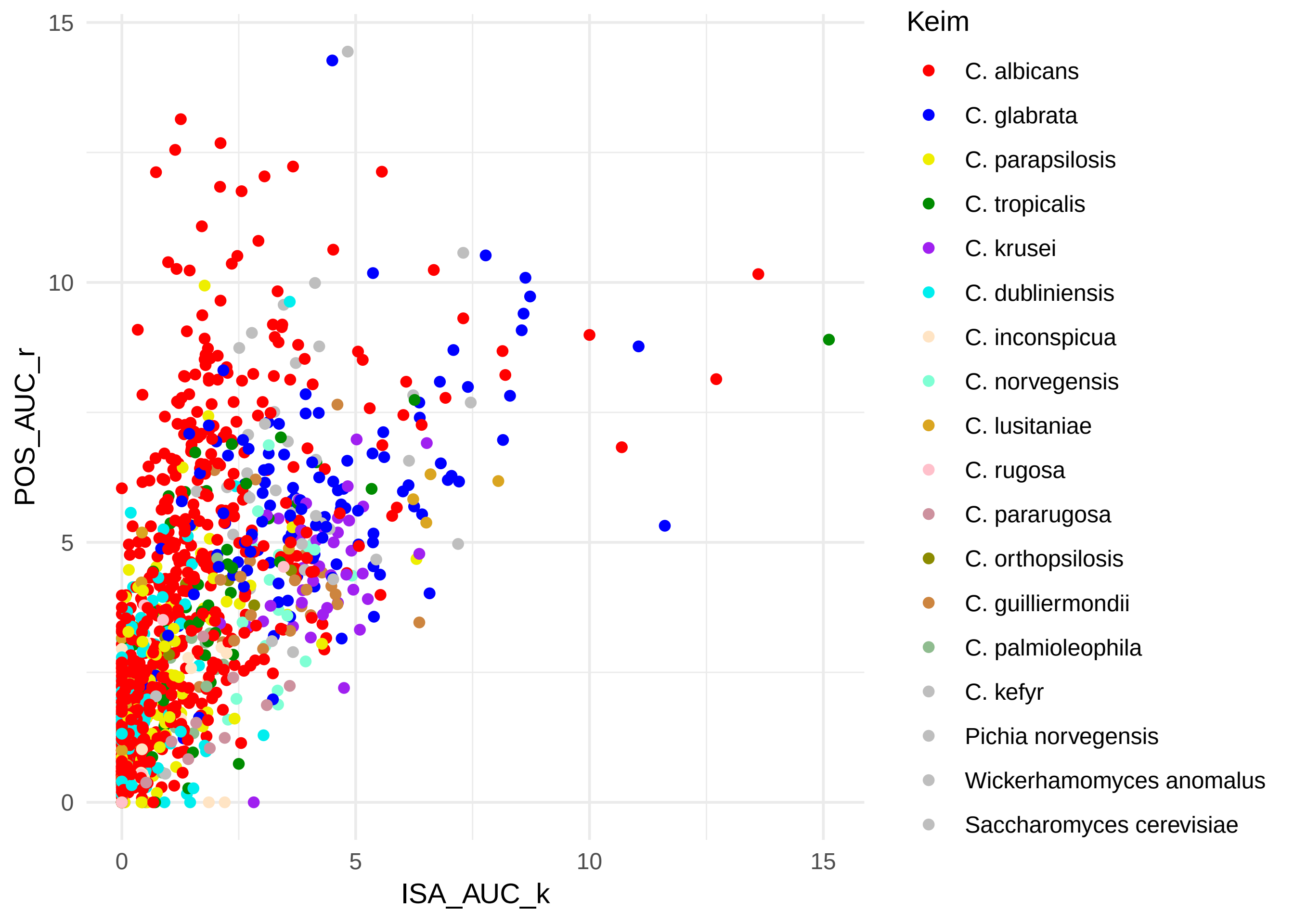

15
10
5
0
POS_AUC_r
0
5
10
15
ISA_AUC_k
Keim
C. albicans
C. glabrata
C. parapsilosis
C. tropicalis
C. krusei
C. dubliniensis
C. inconspicua
C. norvegensis
C. lusitaniae
C. rugosa
C. pararugosa
C. orthopsilosis
C. guilliermondii
C. palmioleophila
C. kefyr
Pichia norvegensis
Wickerhamomyces anomalus
Saccharomyces cerevisiae

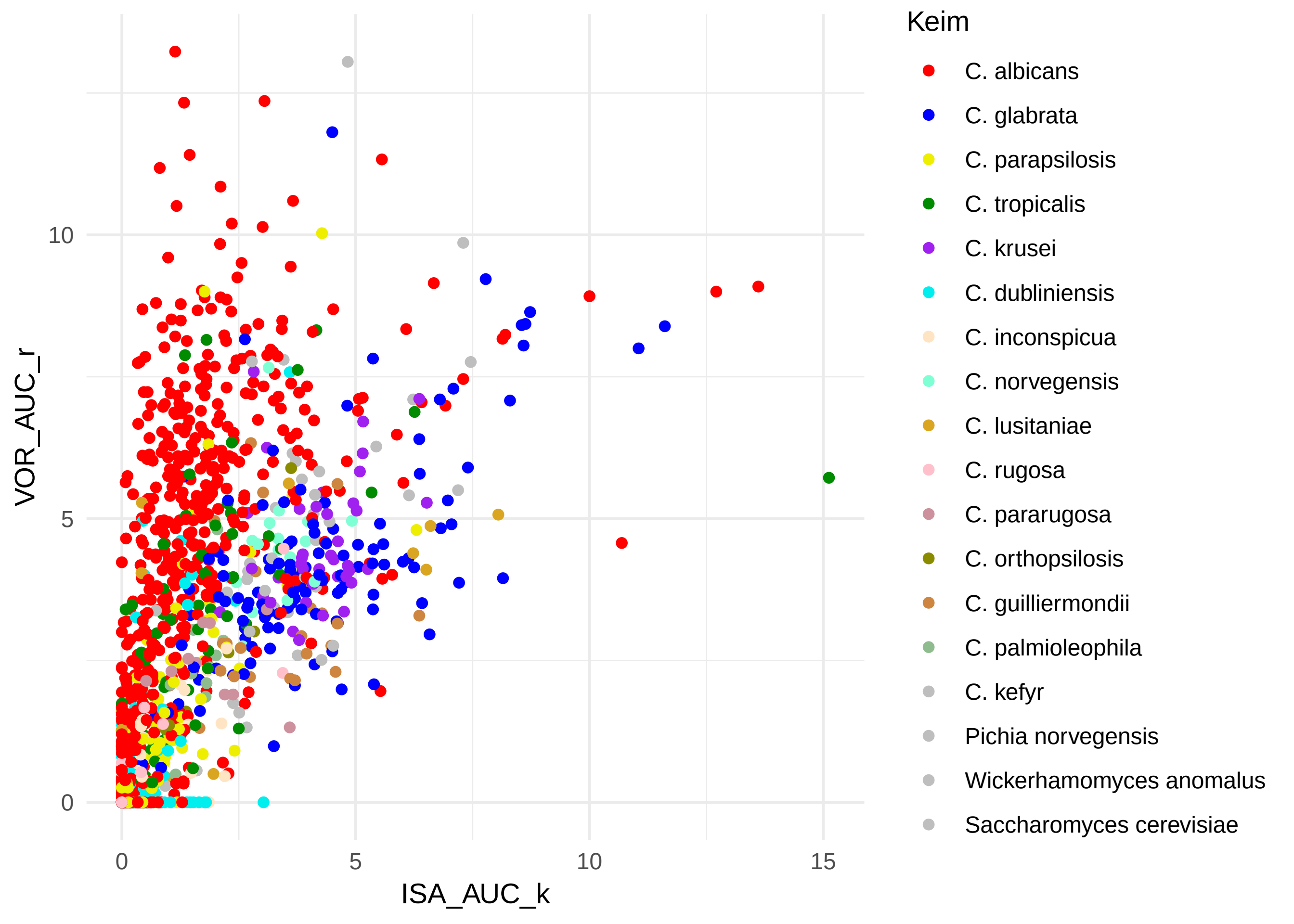

Keim
C. albicans
C. glabrata
C. parapsilosis
C. tropicalis
C. krusei
C. dubliniensis
C. inconspicua
C. norvegensis
C. lusitaniae
C. rugosa
C. pararugosa
C. orthopsilosis
C. guilliermondii
C. palmioleophila
C. kefyr
Pichia norvegensis
Wickerhamomyces anomalus
Saccharomyces cerevisiae
VOR_AUC_r
ISA_AUC_k
0
5
10
15
0
5
10

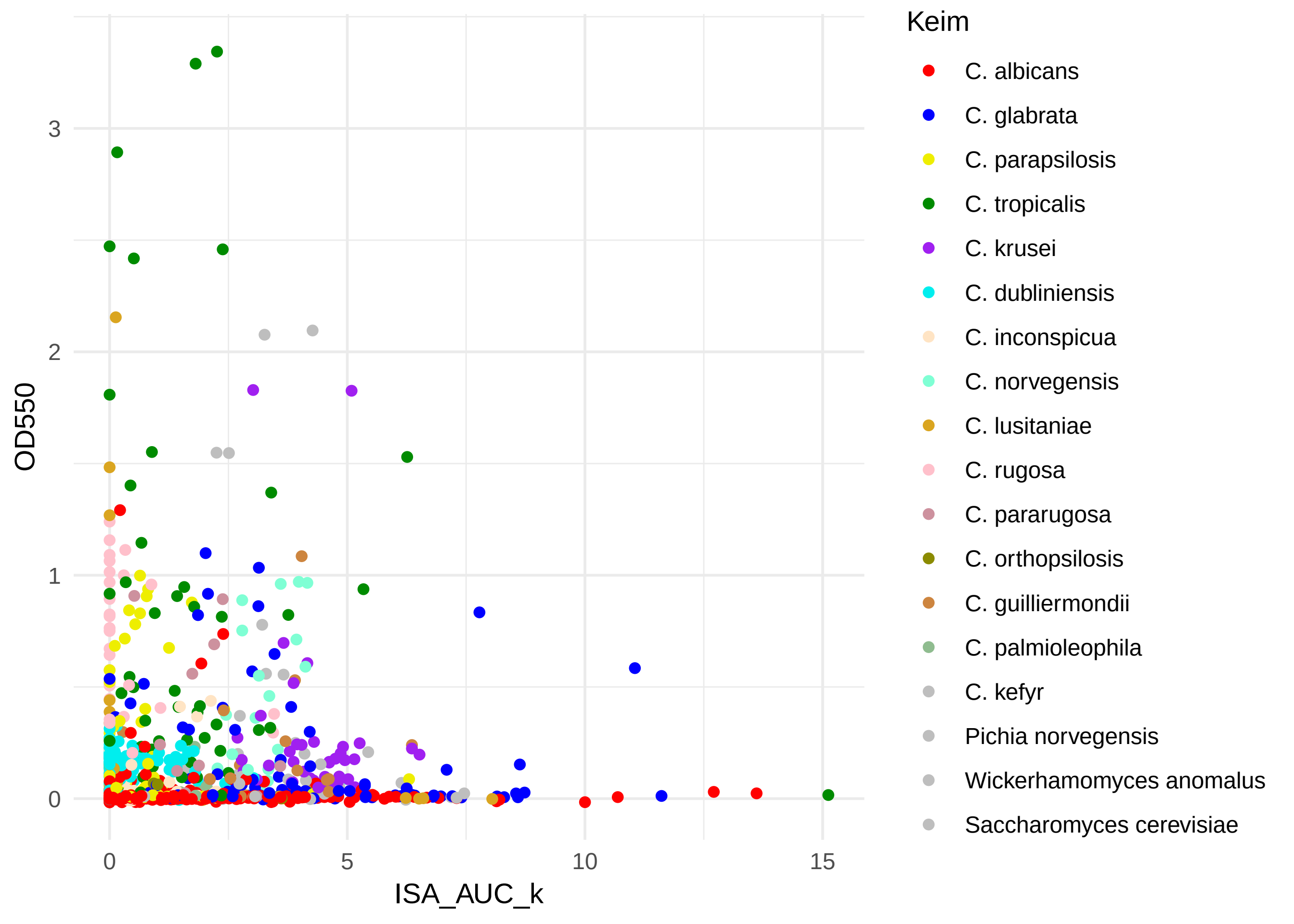

OD550
ISA_AUC_k
0
1
2
3
0
5
10
15
Keim
C. albicans
C. glabrata
C. parapsilosis
C. tropicalis
C. krusei
C. dubliniensis
C. inconspicua
C. norvegensis
C. lusitaniae
C. rugosa
C. pararugosa
C. orthopsilosis
C. guilliermondii
C. palmioleophila
C. kefyr
Pichia norvegensis
Wickerhamomyces anomalus
Saccharomyces cerevisiae

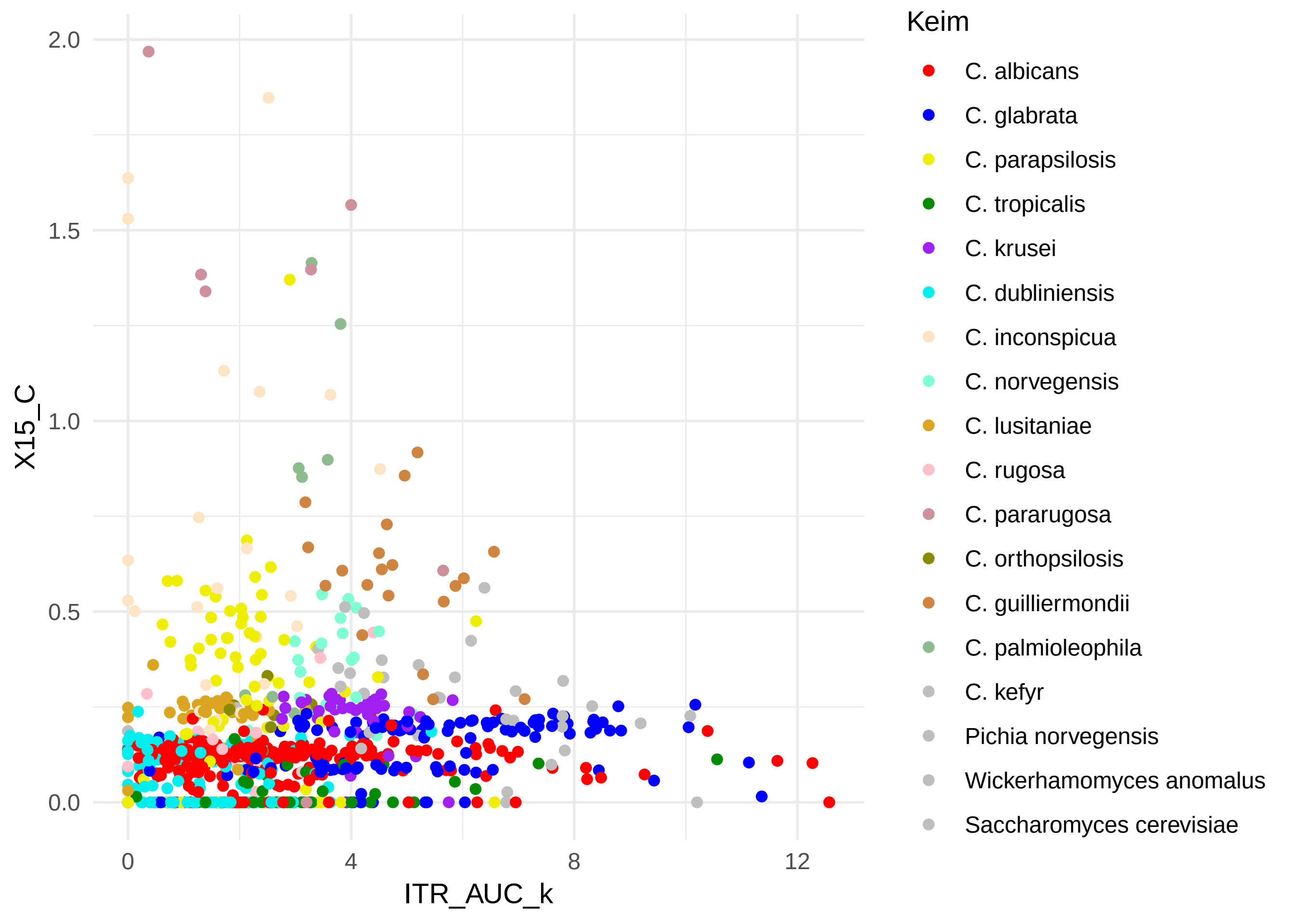
Keim
C. albicans
C. glabrata
C. parapsilosis
C. tropicalis
C. krusei
C. dubliniensis
C. inconspicua
C. norvegensis
C. lusitaniae
C. rugosa
C. pararugosa
C. orthopsilosis
C. guilliermondii
C. palmioleophila
C. kefyr
Pichia norvegensis
Wickerhamomyces anomalus
Saccharomyces cerevisiae
X15_C
2.0
1.5
1.0
0.5
0.0
0
4
8
12
ITR_AUC_k

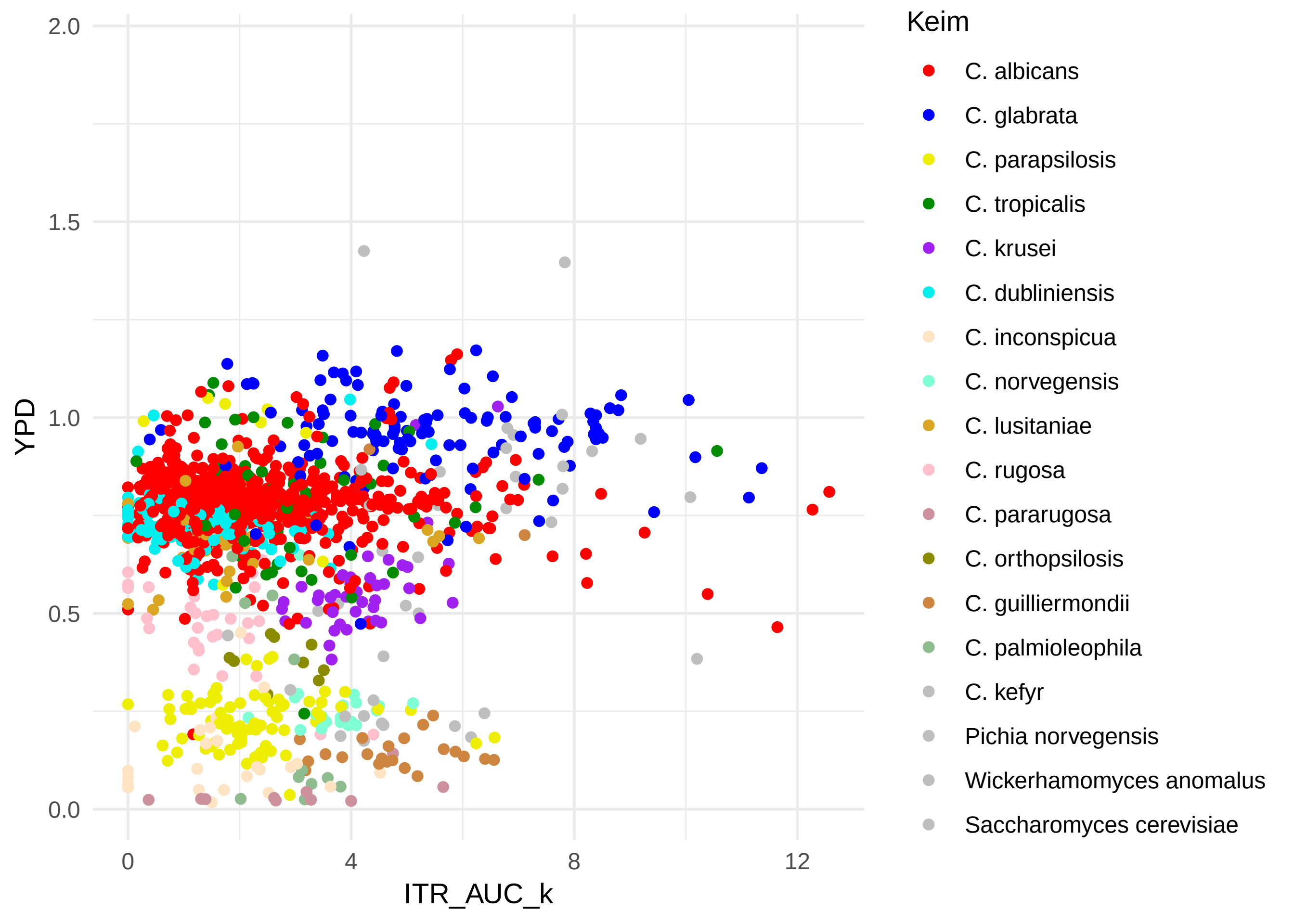

2.0
1.5
1.0
0.5
0.0
YPD
0
4
8
12
ITR_AUC_k
Keim
C. albicans
C. glabrata
C. parapsilosis
C. tropicalis
C. krusei
C. dubliniensis
C. inconspicua
C. norvegensis
C. lusitaniae
C. rugosa
C. pararugosa
C. orthopsilosis
C. guilliermondii
C. palmioleophila
C. kefyr
Pichia norvegensis
Wickerhamomyces anomalus
Saccharomyces cerevisiae

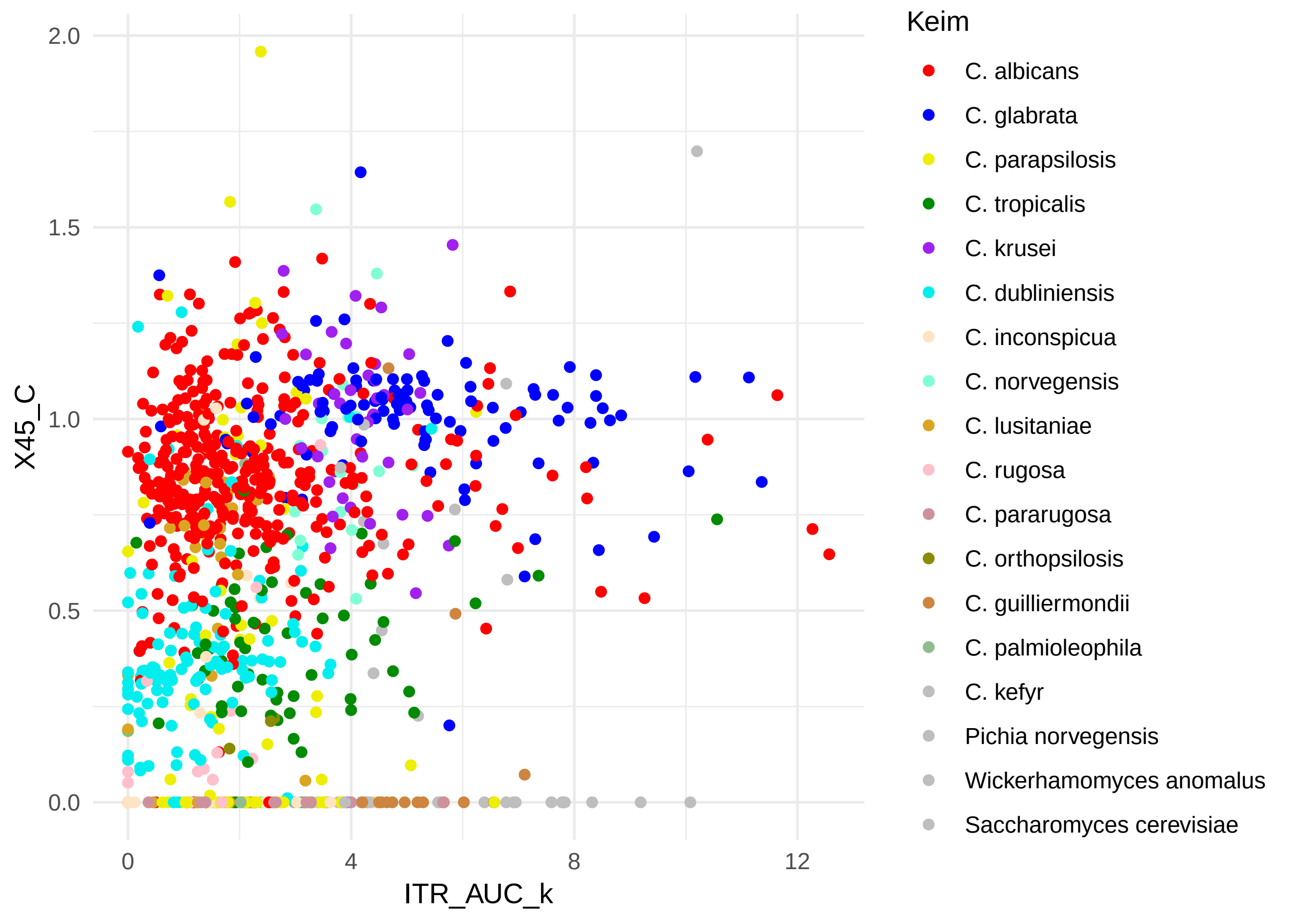
Keim
C. albicans
C. glabrata
C. parapsilosis
C. tropicalis
C. krusei
C. dubliniensis
C. inconspicua
C. norvegensis
C. lusitaniae
C. rugosa
C. pararugosa
C. orthopsilosis
C. guilliermondii
C. palmioleophila
C. kefyr
Pichia norvegensis
Wickerhamomyces anomalus
Saccharomyces cerevisiae
X45_C
2.0
1.5
1.0
0.5
0.0
ITR_AUC_k
0
4
8
12

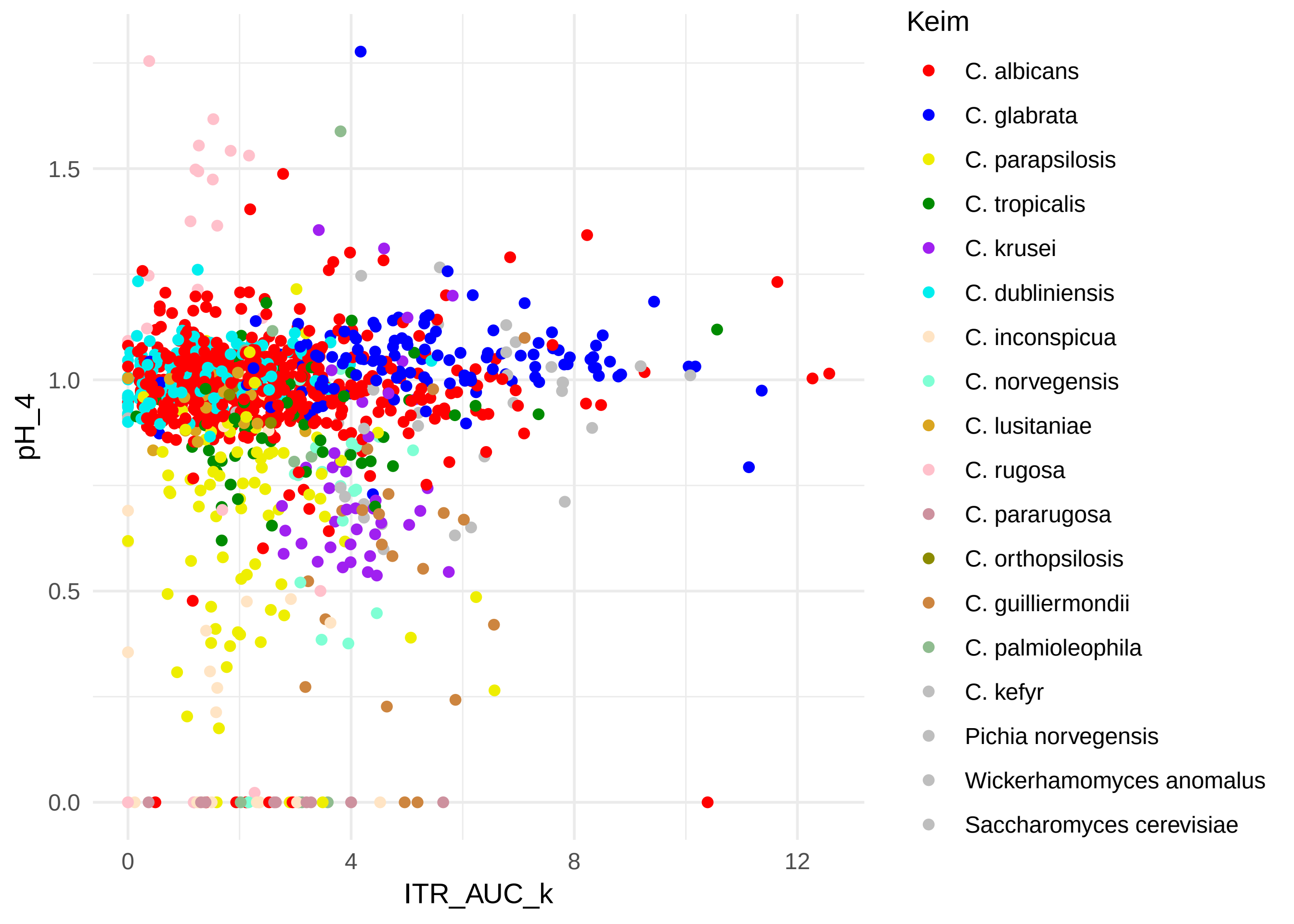

Keim
C. albicans
C. glabrata
C. parapsilosis
C. tropicalis
C. krusei
C. dubliniensis
C. inconspicua
C. norvegensis
C. lusitaniae
C. rugosa
C. pararugosa
C. orthopsilosis
C. guilliermondii
C. palmioleophila
C. kefyr
Pichia norvegensis
Wickerhamomyces anomalus
Saccharomyces cerevisiae
pH_4
1.5
1.0
0.5
0.0
0
4
8
12
ITR_AUC_k

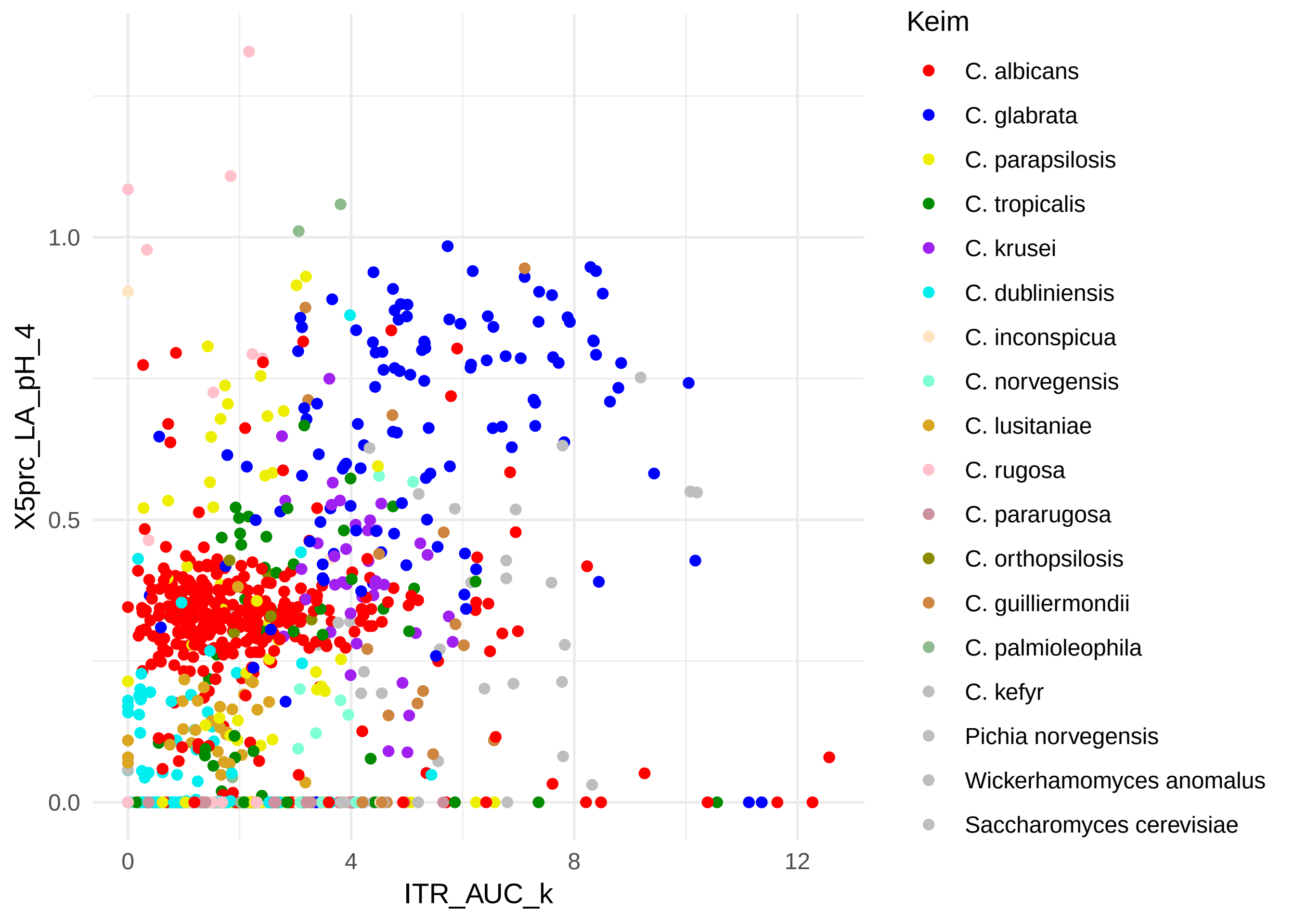

Keim
C. albicans
C. glabrata
C. parapsilosis
C. tropicalis
C. krusei
C. dubliniensis
C. inconspicua
C. norvegensis
C. lusitaniae
C. rugosa
C. pararugosa
C. orthopsilosis
C. guilliermondii
C. palmioleophila
C. kefyr
Pichia norvegensis
Wickerhamomyces anomalus
Saccharomyces cerevisiae
X5prc_LA_pH_4
1.0
0.5
0.0
0
4
8
12
ITR_AUC_k

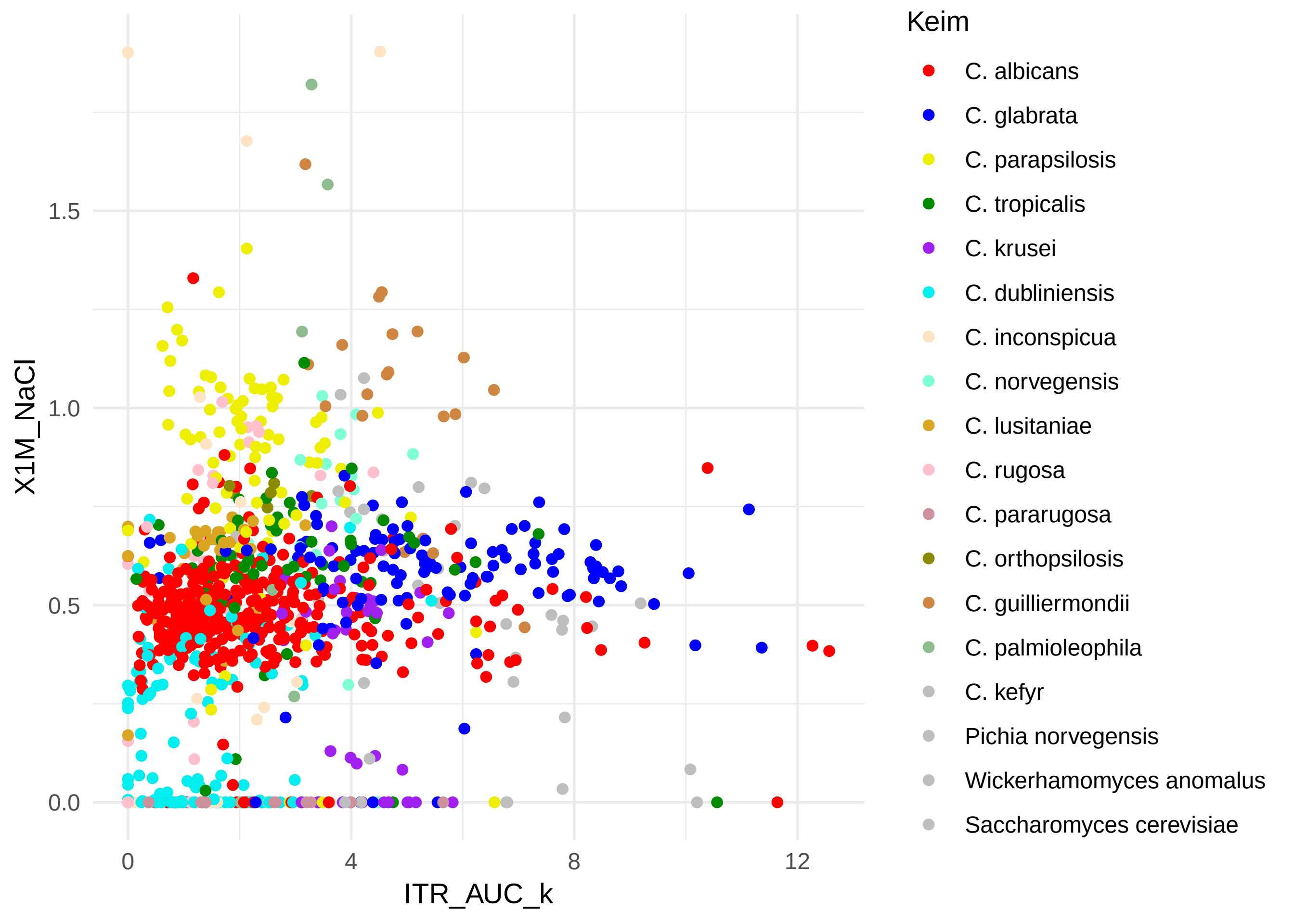
X1M_NaCl
0.0
0.5
1.0
1.5
0
4
8
12
ITR_AUC_k
Keim
C. albicans
C. glabrata
C. parapsilosis
C. tropicalis
C. krusei
C. dubliniensis
C. inconspicua
C. norvegensis
C. lusitaniae
C. rugosa
C. pararugosa
C. orthopsilosis
C. guilliermondii
C. palmioleophila
C. kefyr
Pichia norvegensis
Wickerhamomyces anomalus
Saccharomyces cerevisiae

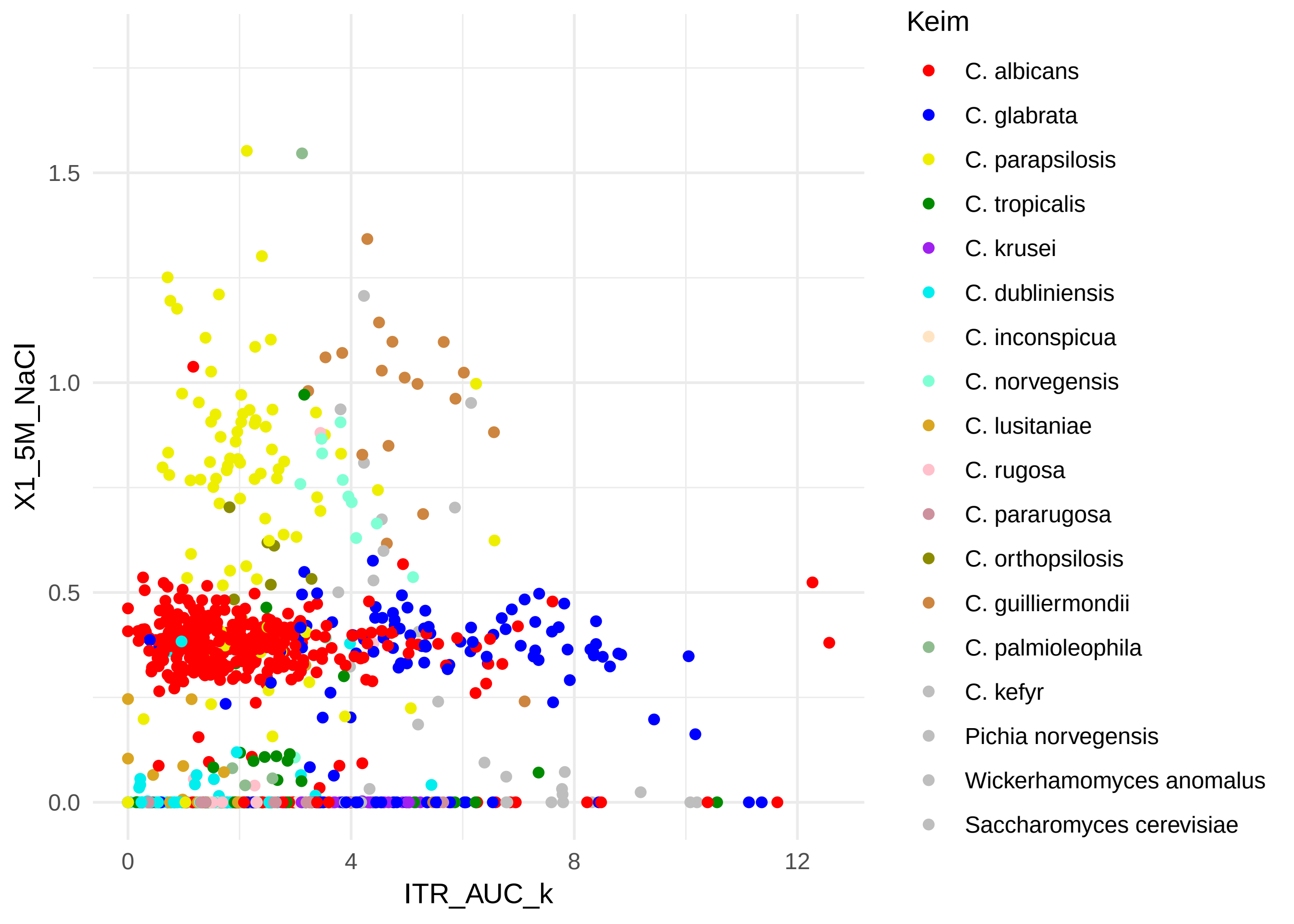

Keim
C. albicans
C. glabrata
C. parapsilosis
C. tropicalis
C. krusei
C. dubliniensis
C. inconspicua
C. norvegensis
C. lusitaniae
C. rugosa
C. pararugosa
C. orthopsilosis
C. guilliermondii
C. palmioleophila
C. kefyr
Pichia norvegensis
Wickerhamomyces anomalus
Saccharomyces cerevisiae
X1_5M_NaCl
1.5
1.0
0.5
0.0
0
4
8
12
ITR_AUC_k

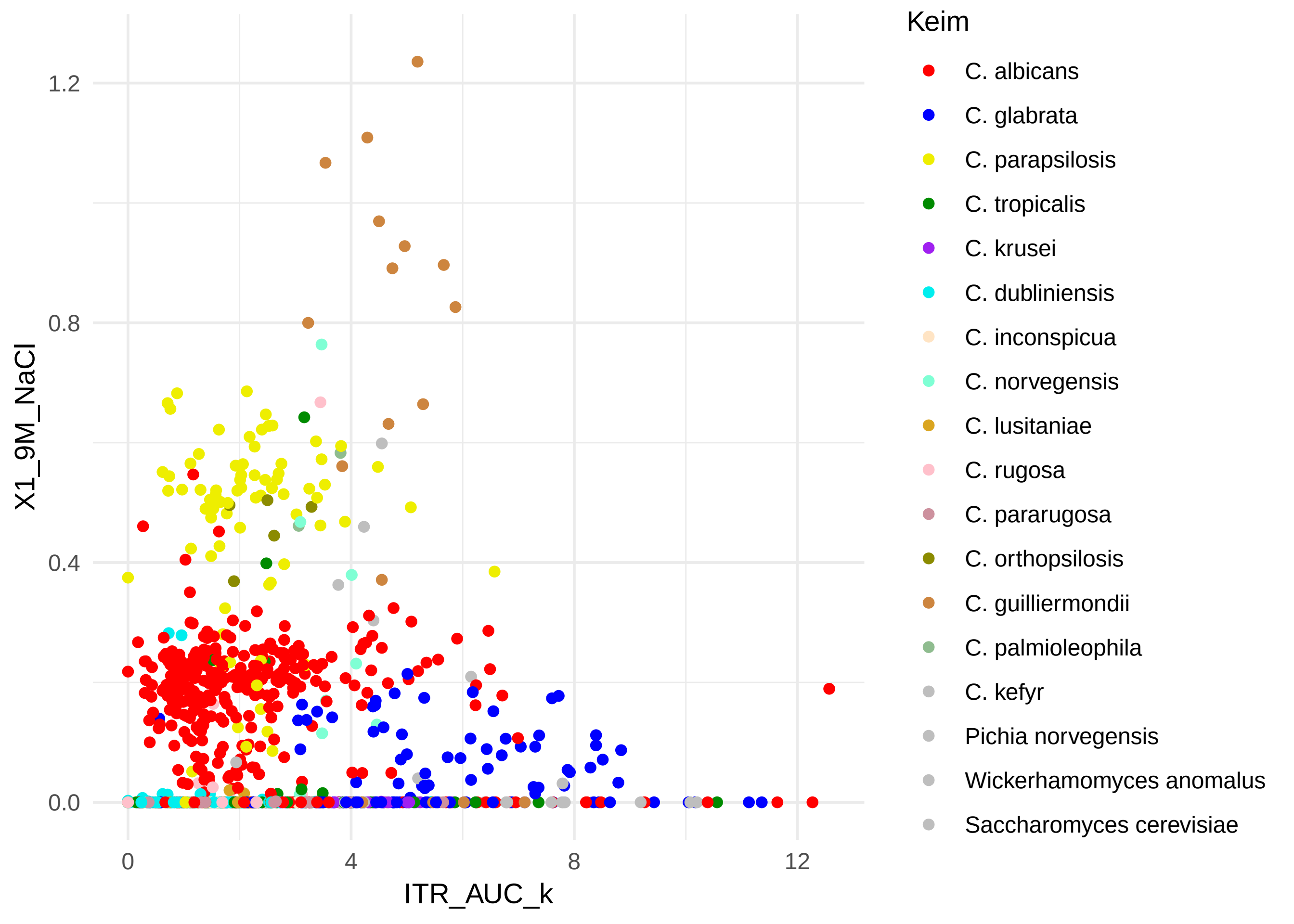

Keim
C. albicans
C. glabrata
C. parapsilosis
C. tropicalis
C. krusei
C. dubliniensis
C. inconspicua
C. norvegensis
C. lusitaniae
C. rugosa
C. pararugosa
C. orthopsilosis
C. guilliermondii
C. palmioleophila
C. kefyr
Pichia norvegensis
Wickerhamomyces anomalus
Saccharomyces cerevisiae
X1_9M_NaCl
0.0
0.4
0.8
1.2
ITR_AUC_k
0
4
8
12

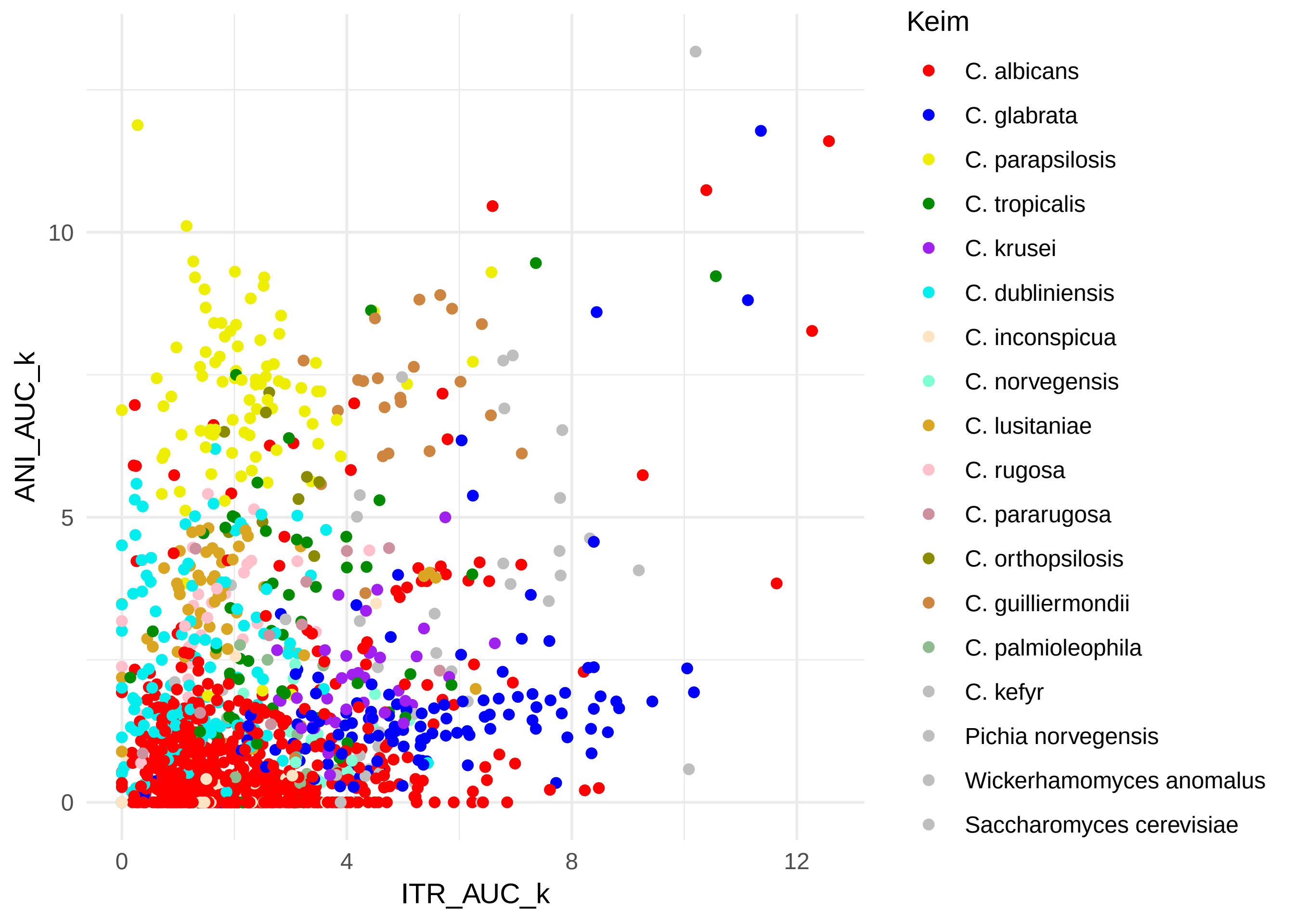

Keim
C. albicans
C. glabrata
C. parapsilosis
C. tropicalis
C. krusei
C. dubliniensis
C. inconspicua
C. norvegensis
C. lusitaniae
C. rugosa
C. pararugosa
C. orthopsilosis
C. guilliermondii
C. palmioleophila
C. kefyr
Pichia norvegensis
Wickerhamomyces anomalus
Saccharomyces cerevisiae
ANI_AUC_k
ITR_AUC_k
0
5
10
0
4
8
12

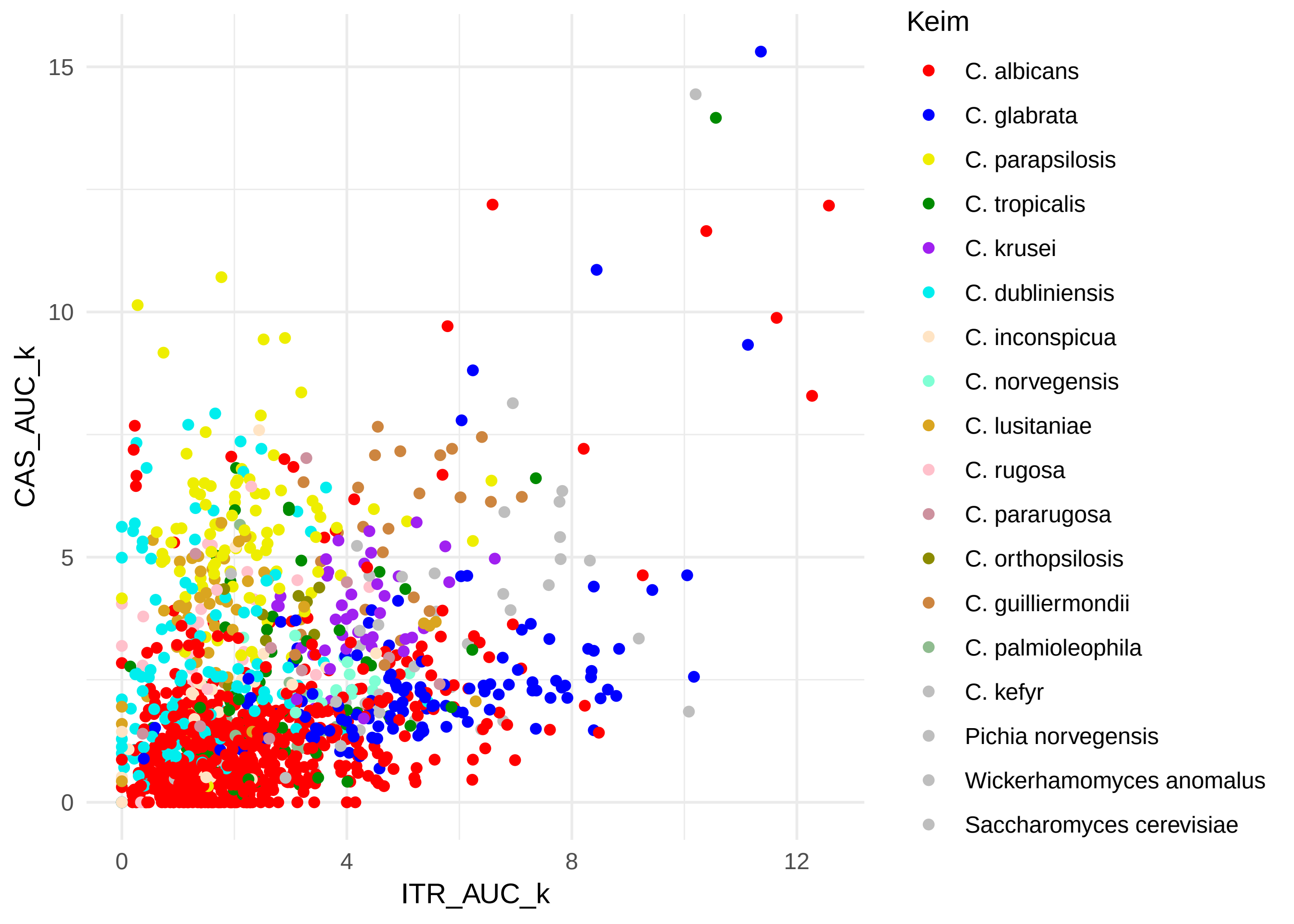

Keim
C. albicans
C. glabrata
C. parapsilosis
C. tropicalis
C. krusei
C. dubliniensis
C. inconspicua
C. norvegensis
C. lusitaniae
C. rugosa
C. pararugosa
C. orthopsilosis
C. guilliermondii
C. palmioleophila
C. kefyr
Pichia norvegensis
Wickerhamomyces anomalus
Saccharomyces cerevisiae
CAS_AUC_k
ITR_AUC_k
0
5
10
15
0
4
8
12

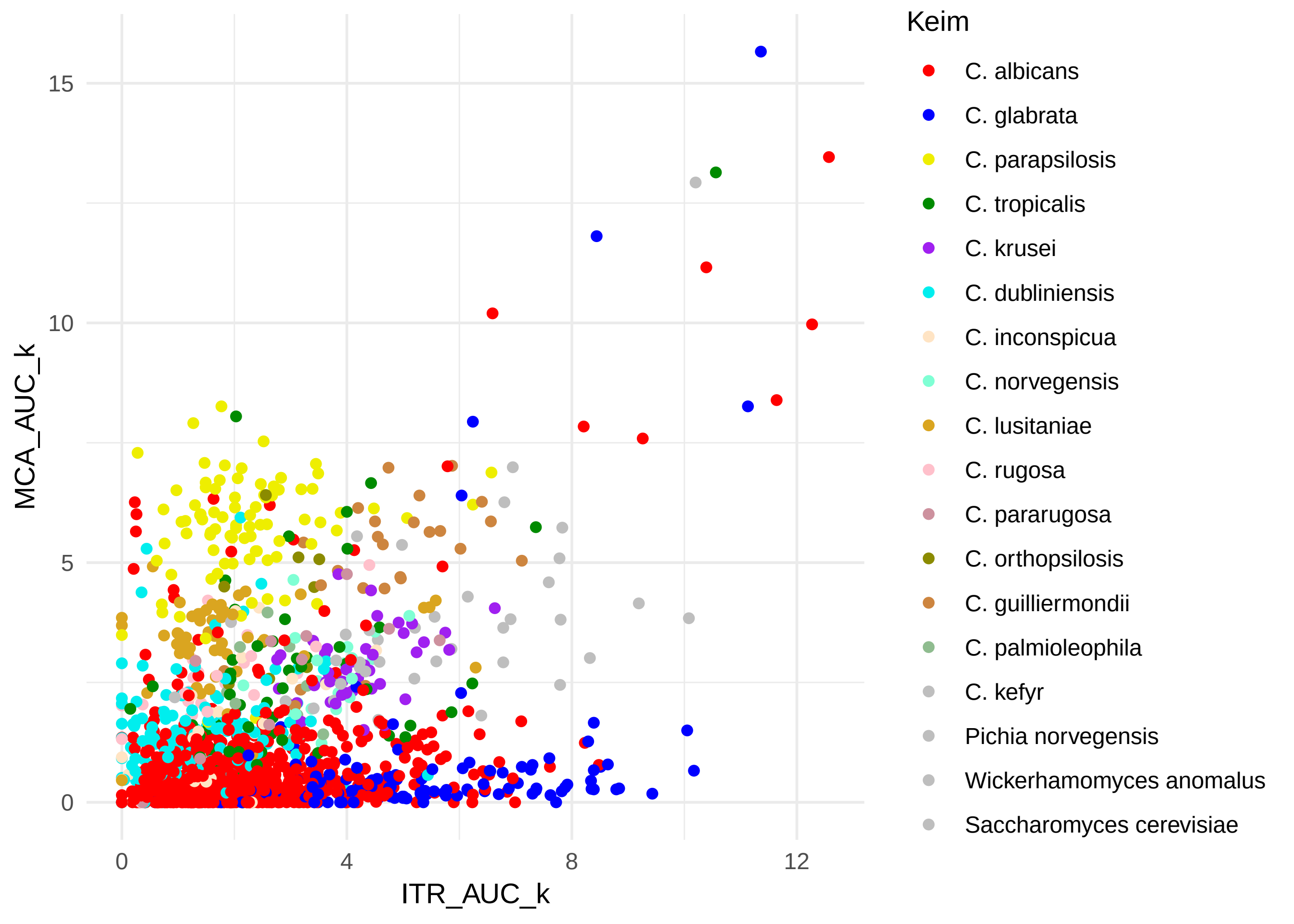

Keim
C. albicans
C. glabrata
C. parapsilosis
C. tropicalis
C. krusei
C. dubliniensis
C. inconspicua
C. norvegensis
C. lusitaniae
C. rugosa
C. pararugosa
C. orthopsilosis
C. guilliermondii
C. palmioleophila
C. kefyr
Pichia norvegensis
Wickerhamomyces anomalus
Saccharomyces cerevisiae
MCA_AUC_k
ITR_AUC_k
0
5
10
15
0
4
8
12

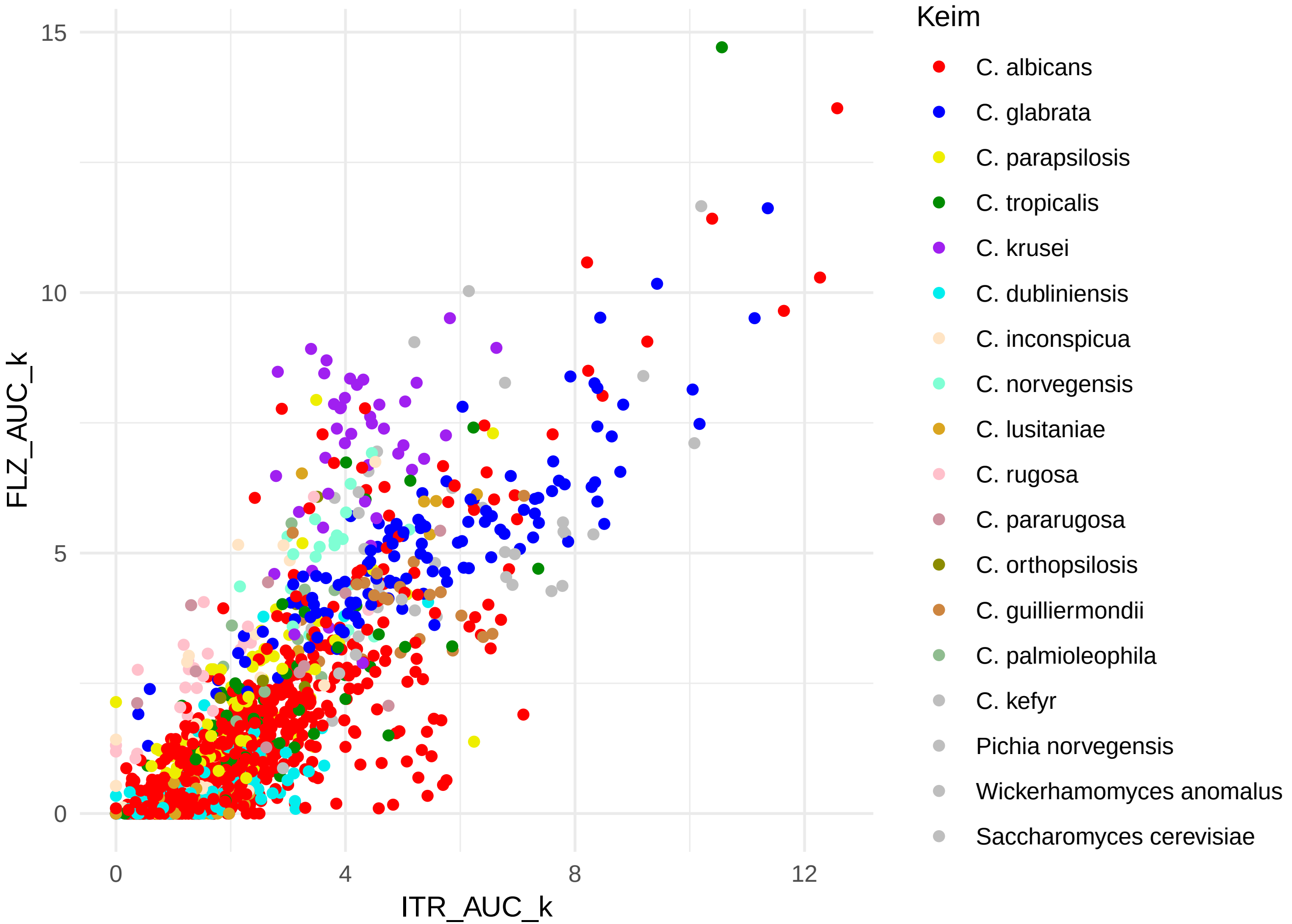

15
10
5
0
FLZ_AUC_k
0
4
8
12
ITR_AUC_k
Keim
C. albicans
C. glabrata
C. parapsilosis
C. tropicalis
C. krusei
C. dubliniensis
C. inconspicua
C. norvegensis
C. lusitaniae
C. rugosa
C. pararugosa
C. orthopsilosis
C. guilliermondii
C. palmioleophila
C. kefyr
Pichia norvegensis
Wickerhamomyces anomalus
Saccharomyces cerevisiae

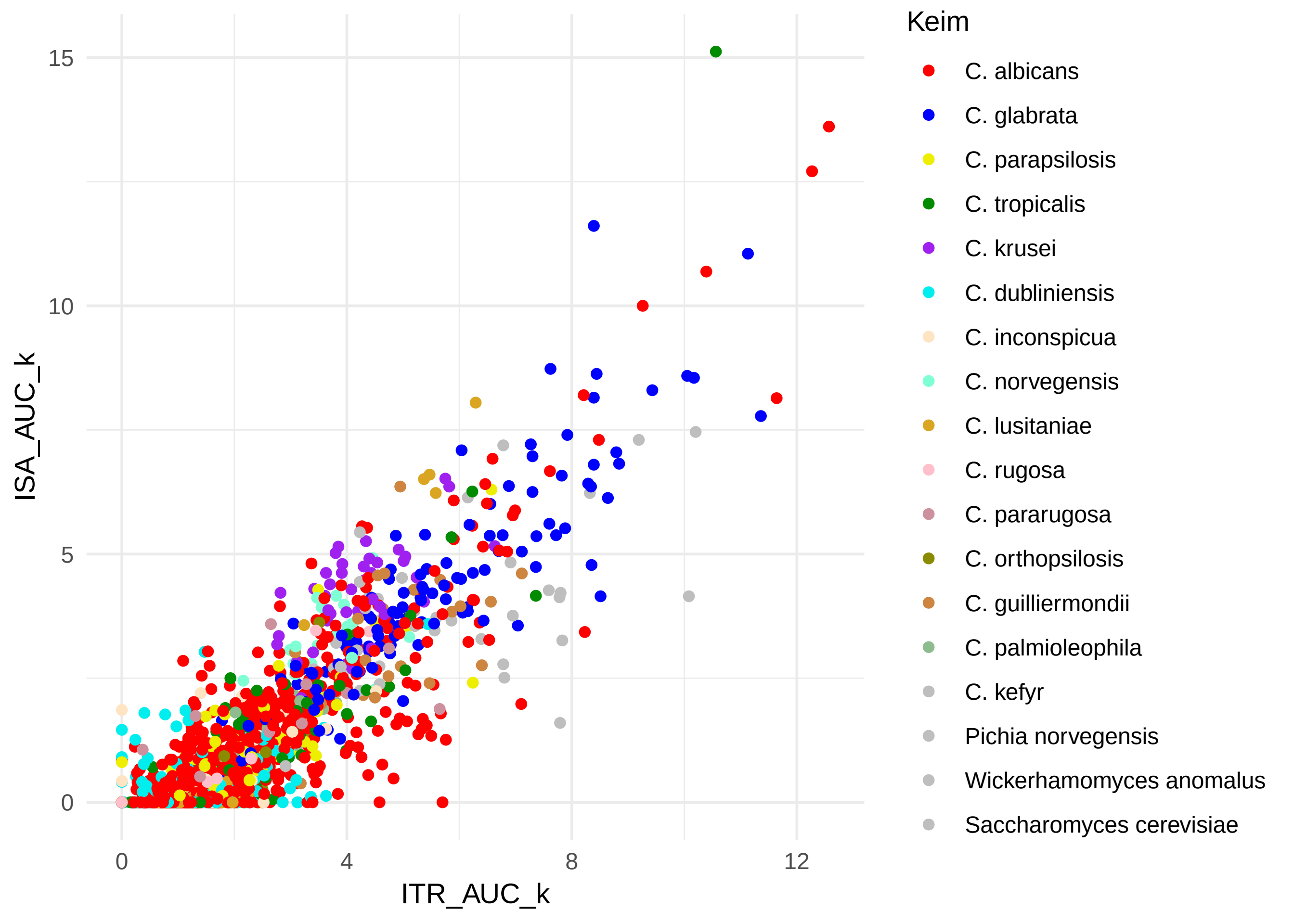

Keim
C. albicans
C. glabrata
C. parapsilosis
C. tropicalis
C. krusei
C. dubliniensis
C. inconspicua
C. norvegensis
C. lusitaniae
C. rugosa
C. pararugosa
C. orthopsilosis
C. guilliermondii
C. palmioleophila
C. kefyr
Pichia norvegensis
Wickerhamomyces anomalus
Saccharomyces cerevisiae
ISA_AUC_k
ITR_AUC_k
0
5
10
15
0
4
8
12

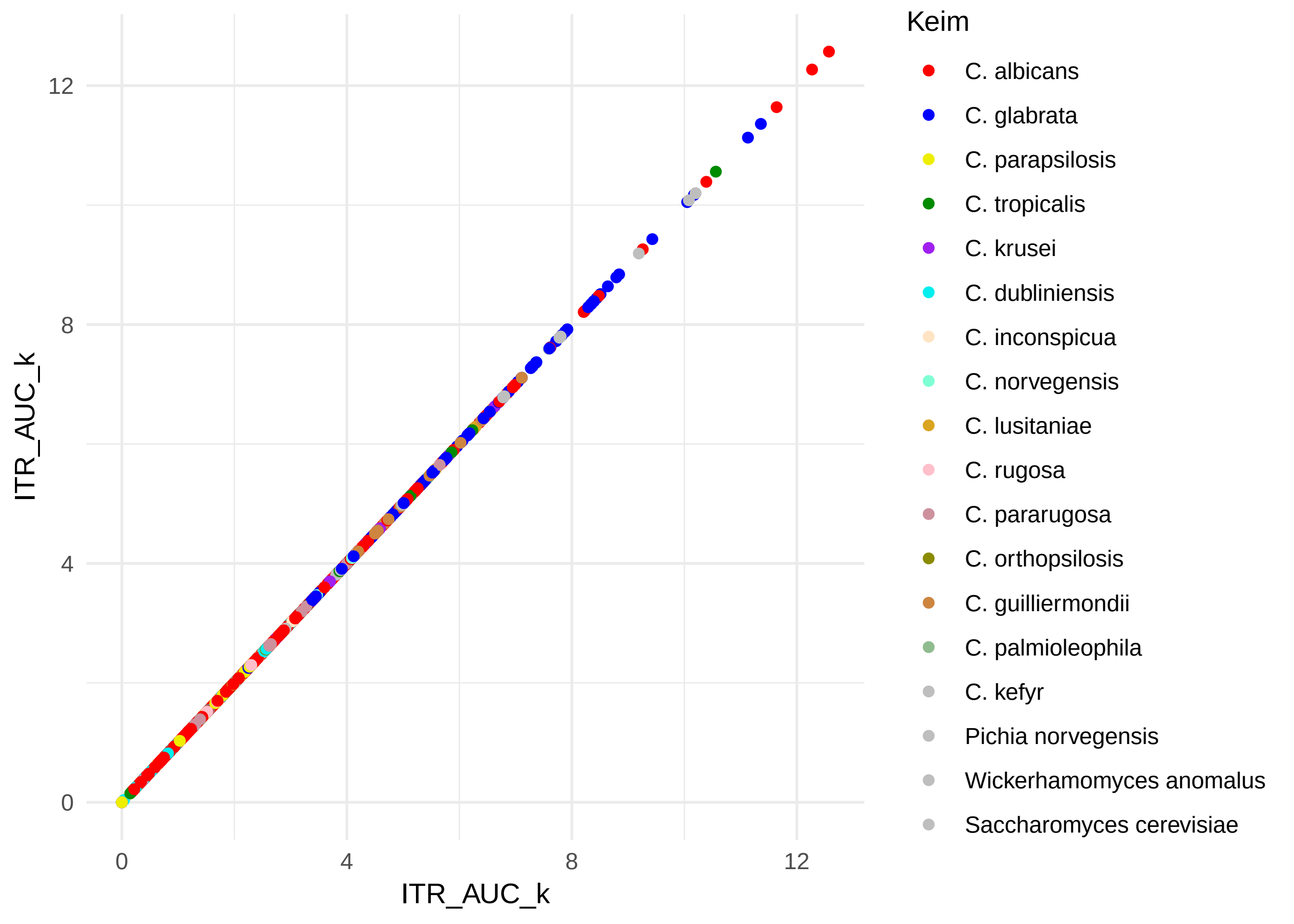

Keim
C. albicans
C. glabrata
C. parapsilosis
C. tropicalis
C. krusei
C. dubliniensis
C. inconspicua
C. norvegensis
C. lusitaniae
C. rugosa
C. pararugosa
C. orthopsilosis
C. guilliermondii
C. palmioleophila
C. kefyr
Pichia norvegensis
Wickerhamomyces anomalus
Saccharomyces cerevisiae
ITR_AUC_k
ITR_AUC_k
0
4
8
12
0
4
8
12

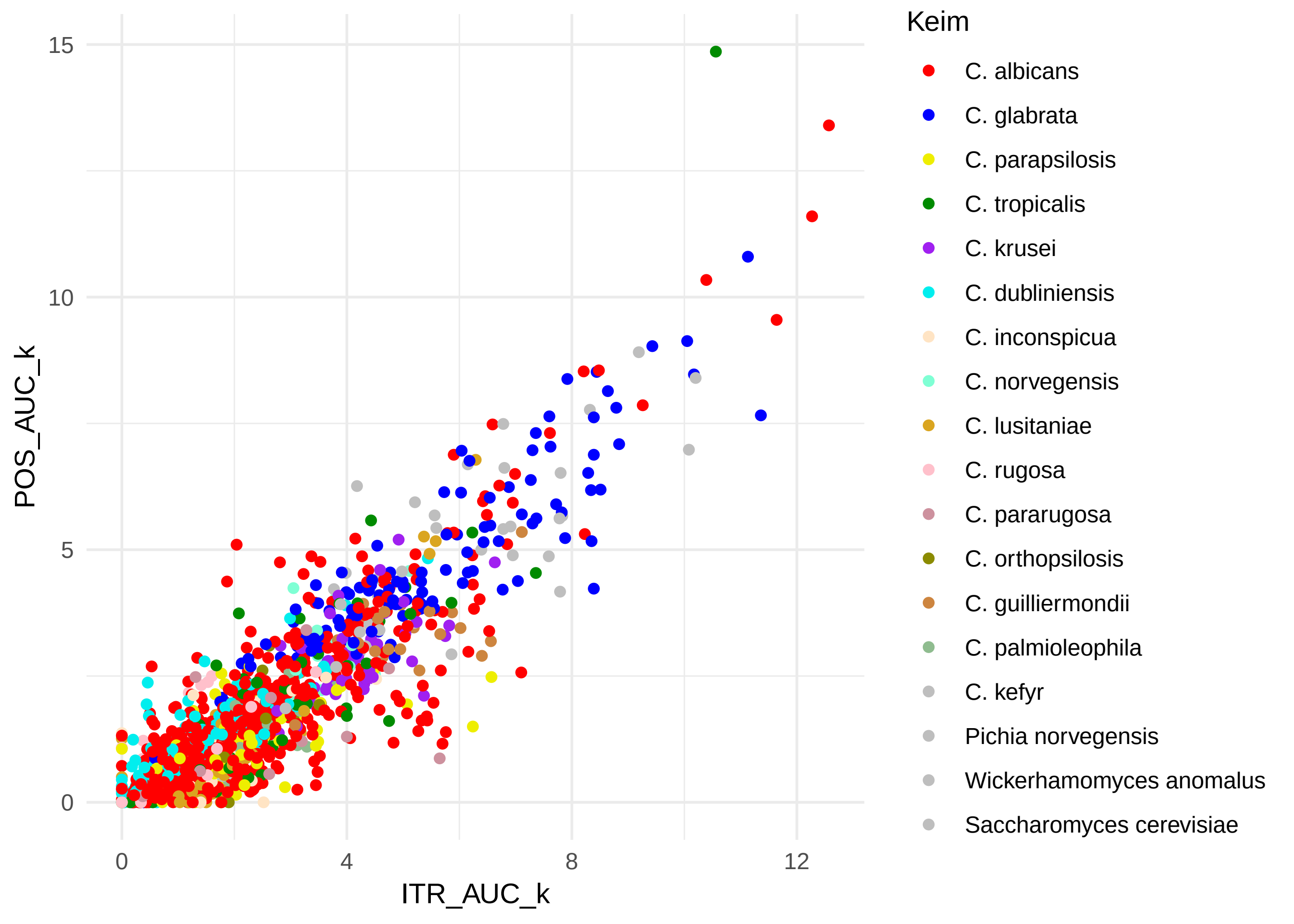

Keim
C. albicans
C. glabrata
C. parapsilosis
C. tropicalis
C. krusei
C. dubliniensis
C. inconspicua
C. norvegensis
C. lusitaniae
C. rugosa
C. pararugosa
C. orthopsilosis
C. guilliermondii
C. palmioleophila
C. kefyr
Pichia norvegensis
Wickerhamomyces anomalus
Saccharomyces cerevisiae
POS_AUC_k
ITR_AUC_k
0
5
10
15
0
4
8
12

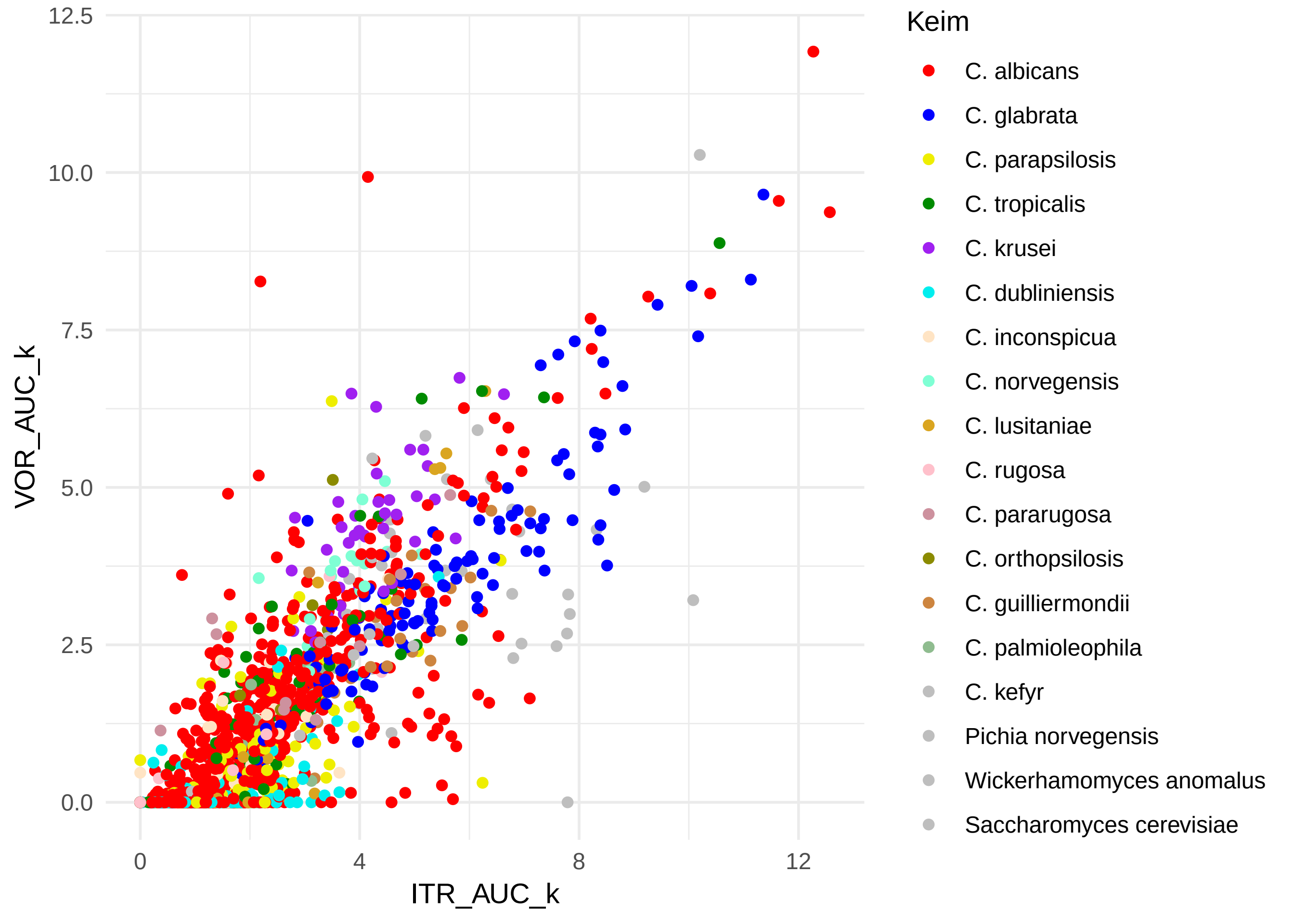

Keim
C. albicans
C. glabrata
C. parapsilosis
C. tropicalis
C. krusei
C. dubliniensis
C. inconspicua
C. norvegensis
C. lusitaniae
C. rugosa
C. pararugosa
C. orthopsilosis
C. guilliermondii
C. palmioleophila
C. kefyr
Pichia norvegensis
Wickerhamomyces anomalus
Saccharomyces cerevisiae
VOR_AUC_k
ITR_AUC_k
0.0
2.5
5.0
7.5
10.0
12.5
0
4
8
12

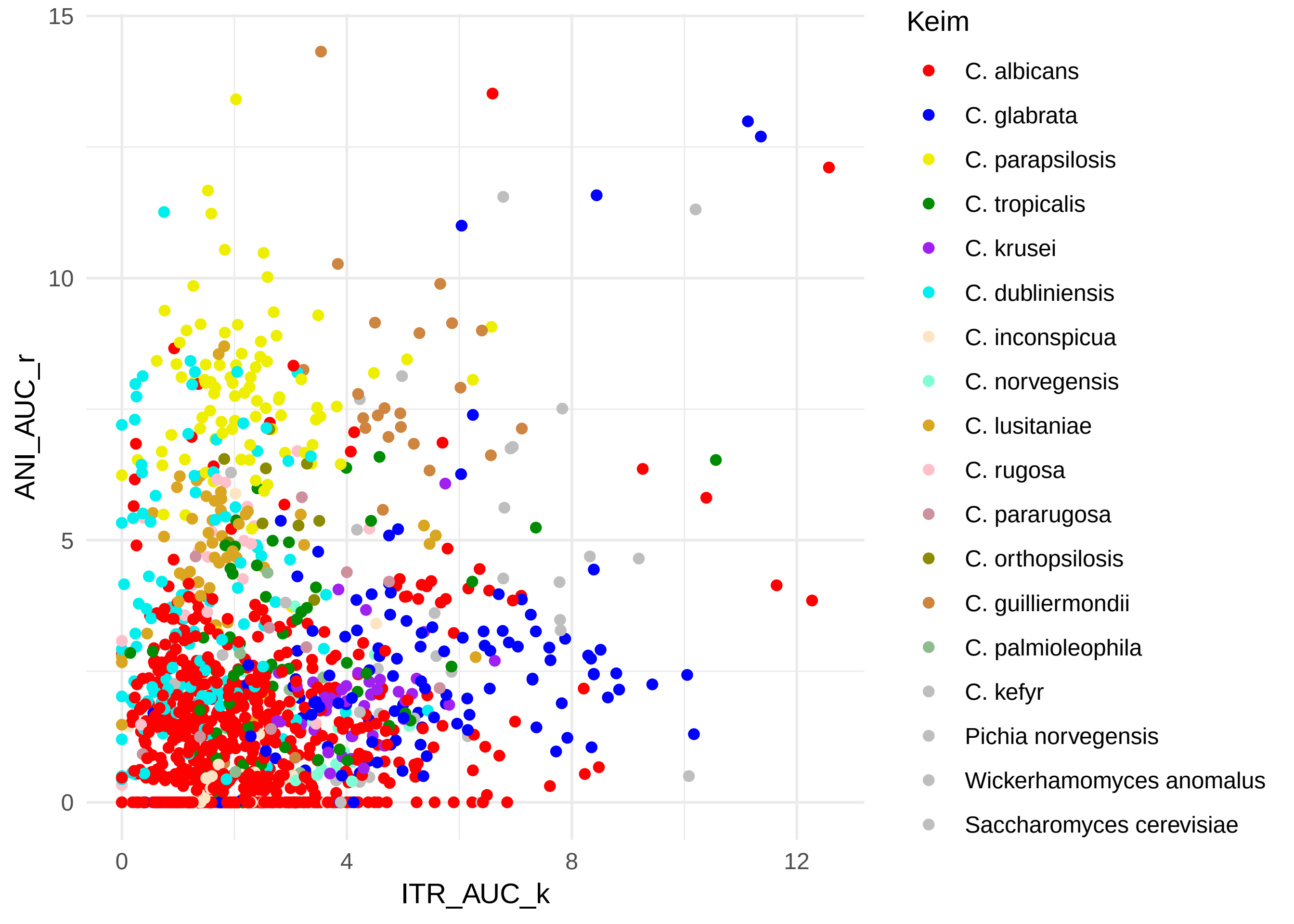
Keim
C. albicans
C. glabrata
C. parapsilosis
C. tropicalis
C. krusei
C. dubliniensis
C. inconspicua
C. norvegensis
C. lusitaniae
C. rugosa
C. pararugosa
C. orthopsilosis
C. guilliermondii
C. palmioleophila
C. kefyr
Pichia norvegensis
Wickerhamomyces anomalus
Saccharomyces cerevisiae
ANI_AUC_r
ITR_AUC_k
0
5
10
15
0
4
8
12

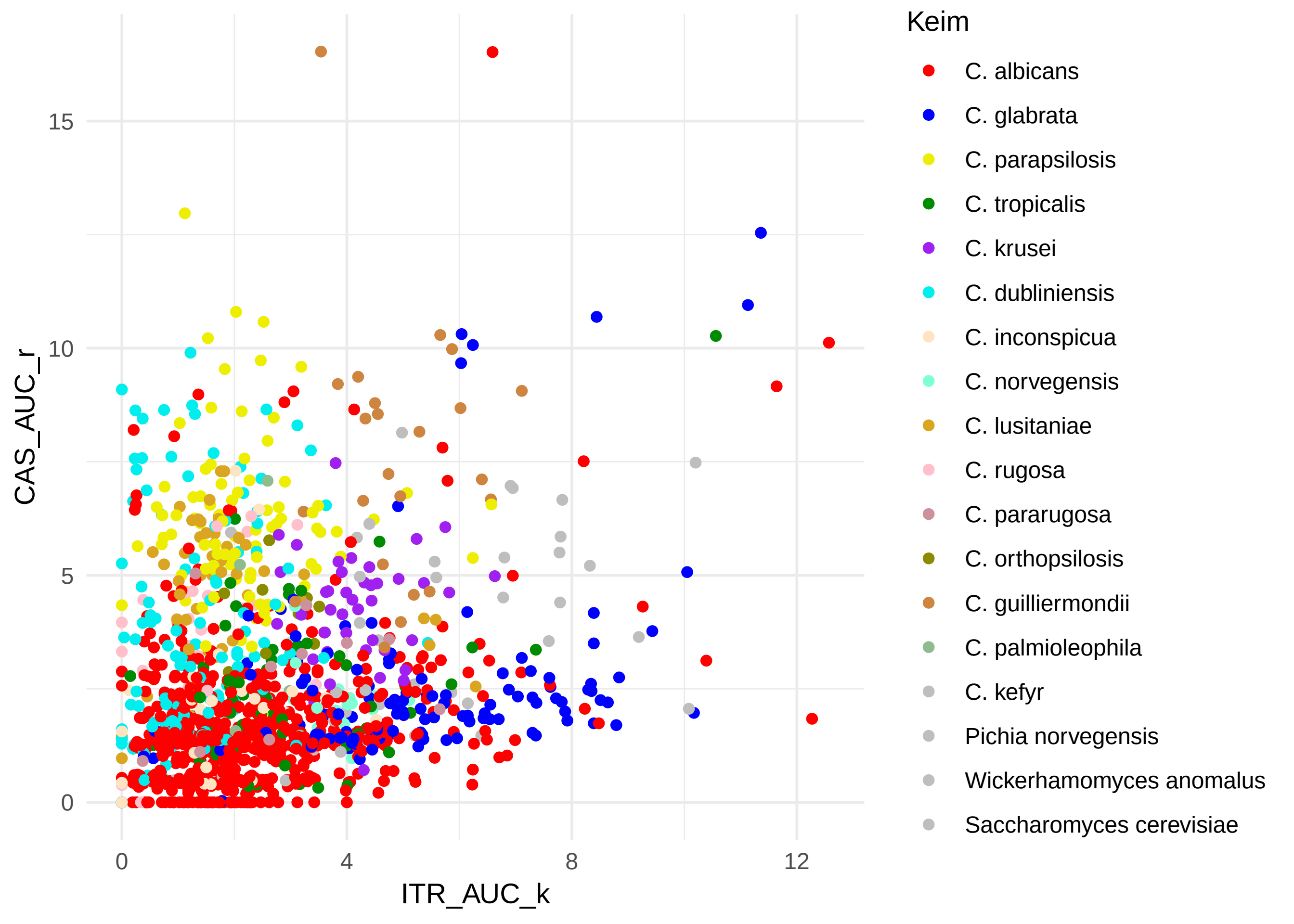

Keim
C. albicans
C. glabrata
C. parapsilosis
C. tropicalis
C. krusei
C. dubliniensis
C. inconspicua
C. norvegensis
C. lusitaniae
C. rugosa
C. pararugosa
C. orthopsilosis
C. guilliermondii
C. palmioleophila
C. kefyr
Pichia norvegensis
Wickerhamomyces anomalus
Saccharomyces cerevisiae
CAS_AUC_r
ITR_AUC_k
0
5
10
15
0
4
8
12

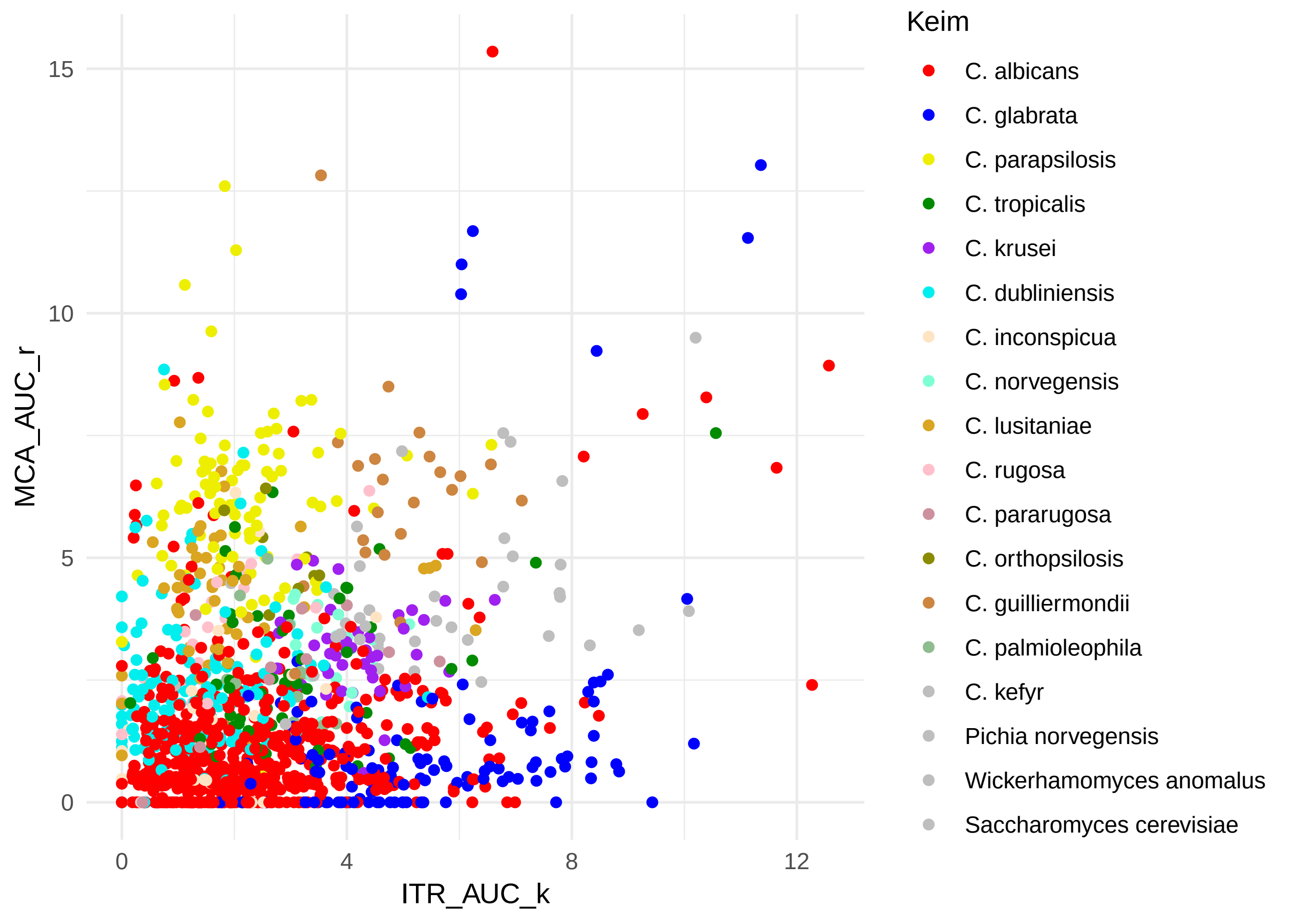

Keim
C. albicans
C. glabrata
C. parapsilosis
C. tropicalis
C. krusei
C. dubliniensis
C. inconspicua
C. norvegensis
C. lusitaniae
C. rugosa
C. pararugosa
C. orthopsilosis
C. guilliermondii
C. palmioleophila
C. kefyr
Pichia norvegensis
Wickerhamomyces anomalus
Saccharomyces cerevisiae
MCA_AUC_r
ITR_AUC_k
0
5
10
15
0
4
8
12

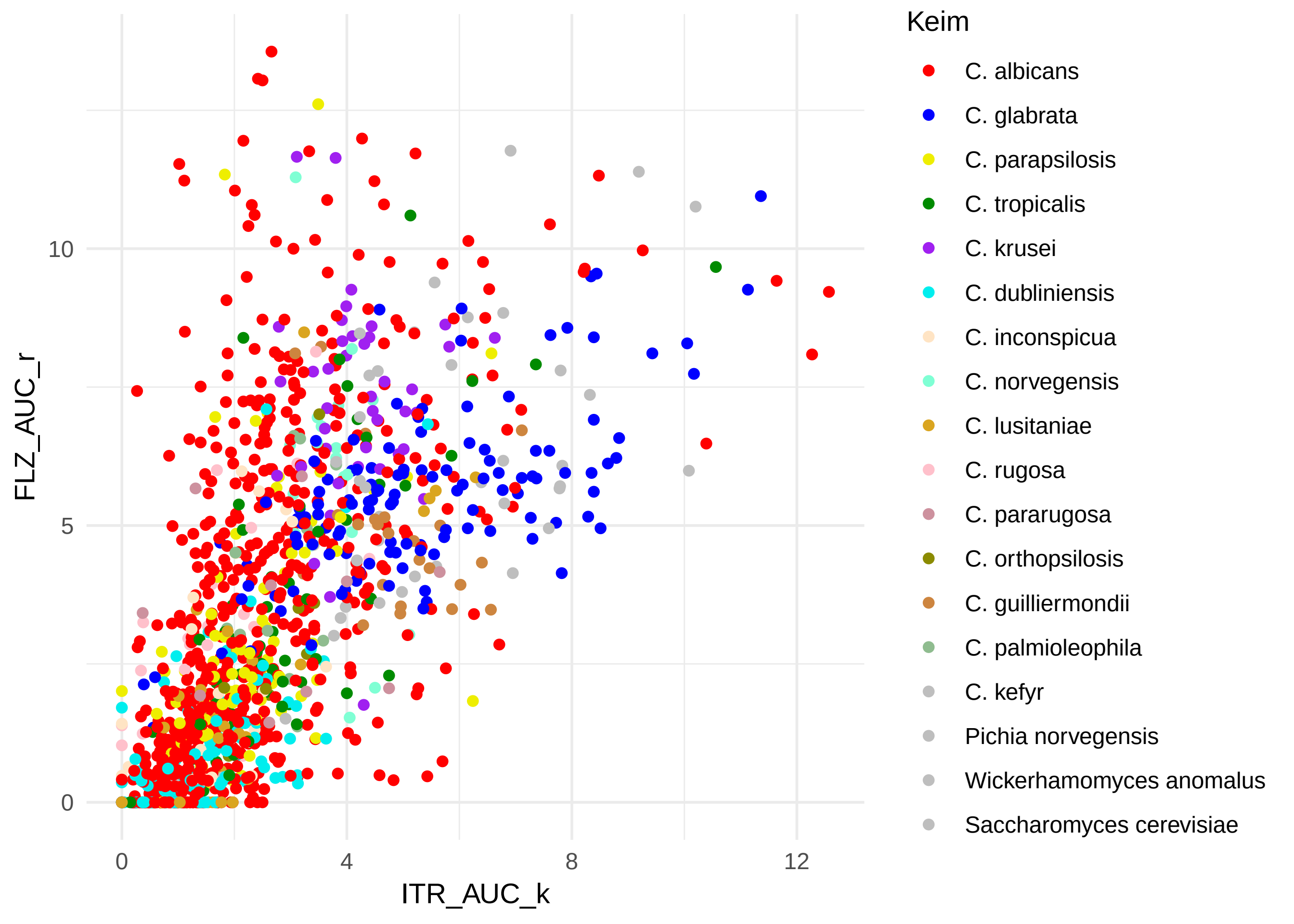

Keim
C. albicans
C. glabrata
C. parapsilosis
C. tropicalis
C. krusei
C. dubliniensis
C. inconspicua
C. norvegensis
C. lusitaniae
C. rugosa
C. pararugosa
C. orthopsilosis
C. guilliermondii
C. palmioleophila
C. kefyr
Pichia norvegensis
Wickerhamomyces anomalus
Saccharomyces cerevisiae
FLZ_AUC_r
ITR_AUC_k
0
5
10
0
4
8
12

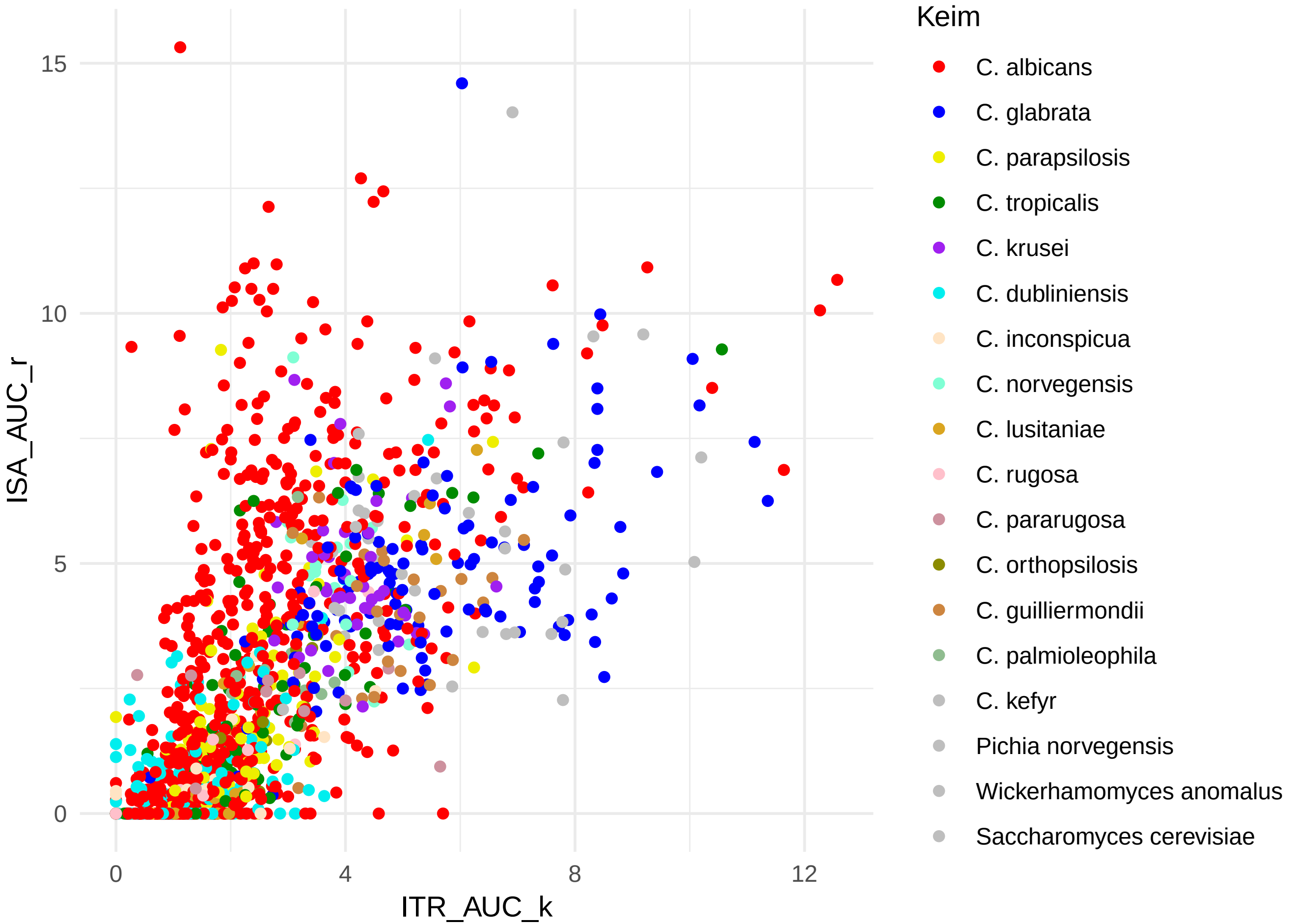

Keim
C. albicans
C. glabrata
C. parapsilosis
C. tropicalis
C. krusei
C. dubliniensis
C. inconspicua
C. norvegensis
C. lusitaniae
C. rugosa
C. pararugosa
C. orthopsilosis
C. guilliermondii
C. palmioleophila
C. kefyr
Pichia norvegensis
Wickerhamomyces anomalus
Saccharomyces cerevisiae
ISA_AUC_r
ITR_AUC_k
0
5
10
15
0
4
8
12

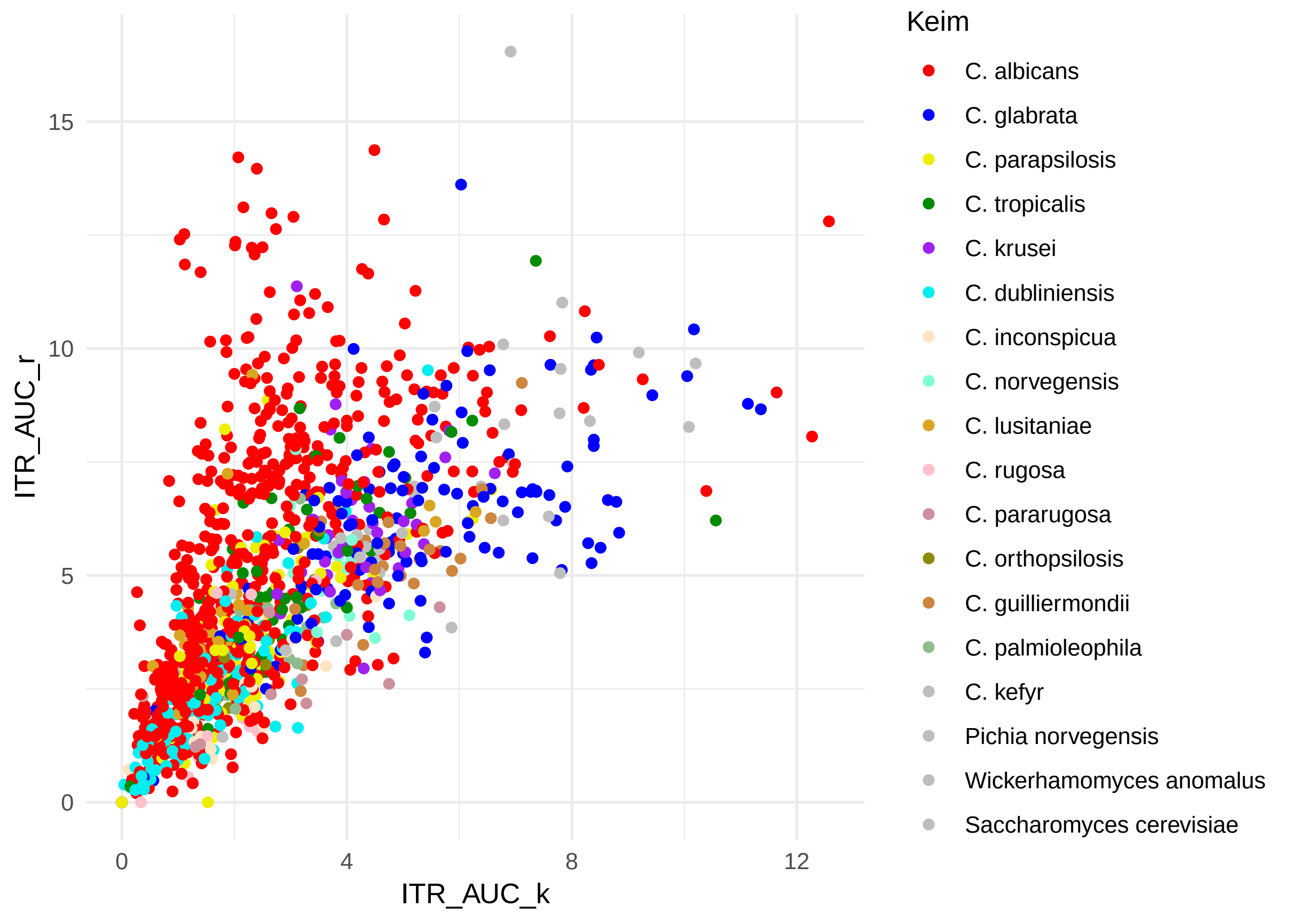

Keim
C. albicans
C. glabrata
C. parapsilosis
C. tropicalis
C. krusei
C. dubliniensis
C. inconspicua
C. norvegensis
C. lusitaniae
C. rugosa
C. pararugosa
C. orthopsilosis
C. guilliermondii
C. palmioleophila
C. kefyr
Pichia norvegensis
Wickerhamomyces anomalus
Saccharomyces cerevisiae
ITR_AUC_r
ITR_AUC_k
0
4
8
12
0
5
10
15

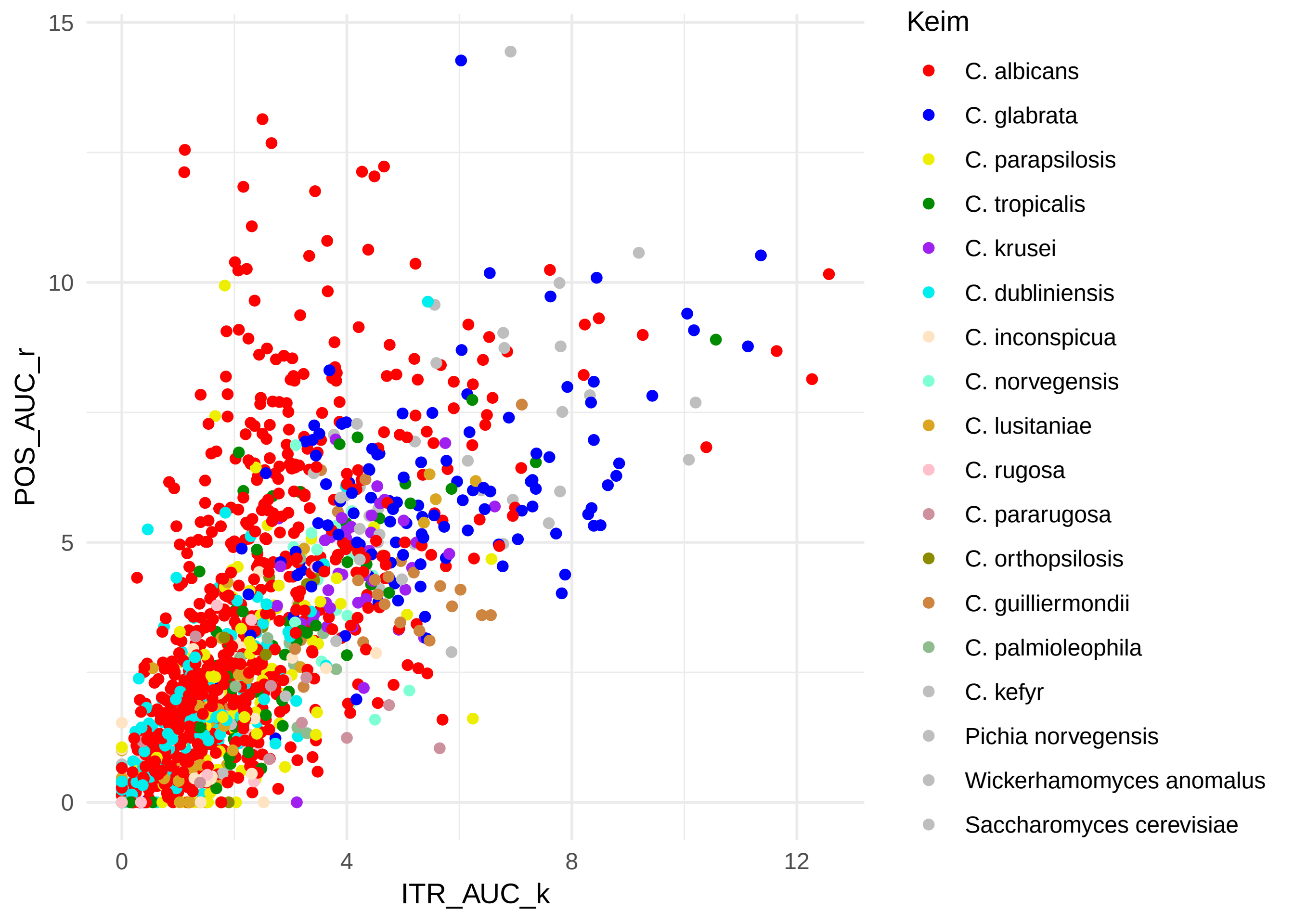
15
10
5
0
POS_AUC_r
0
4
8
12
ITR_AUC_k
Keim
C. albicans
C. glabrata
C. parapsilosis
C. tropicalis
C. krusei
C. dubliniensis
C. inconspicua
C. norvegensis
C. lusitaniae
C. rugosa
C. pararugosa
C. orthopsilosis
C. guilliermondii
C. palmioleophila
C. kefyr
Pichia norvegensis
Wickerhamomyces anomalus
Saccharomyces cerevisiae

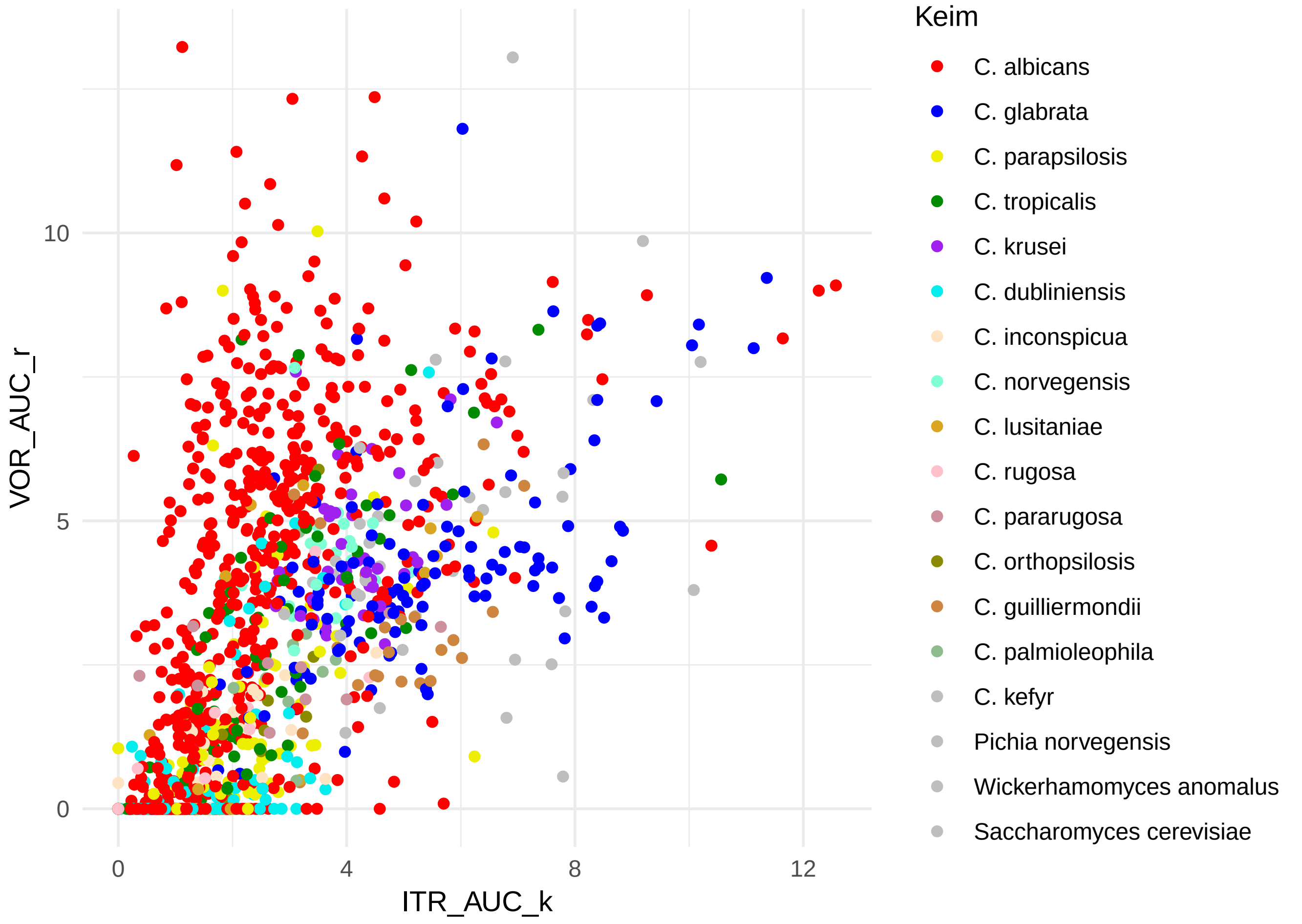

Keim
C. albicans
C. glabrata
C. parapsilosis
C. tropicalis
C. krusei
C. dubliniensis
C. inconspicua
C. norvegensis
C. lusitaniae
C. rugosa
C. pararugosa
C. orthopsilosis
C. guilliermondii
C. palmioleophila
C. kefyr
Pichia norvegensis
Wickerhamomyces anomalus
Saccharomyces cerevisiae
VOR_AUC_r
10
5
0
ITR_AUC_k
0
4
8
12

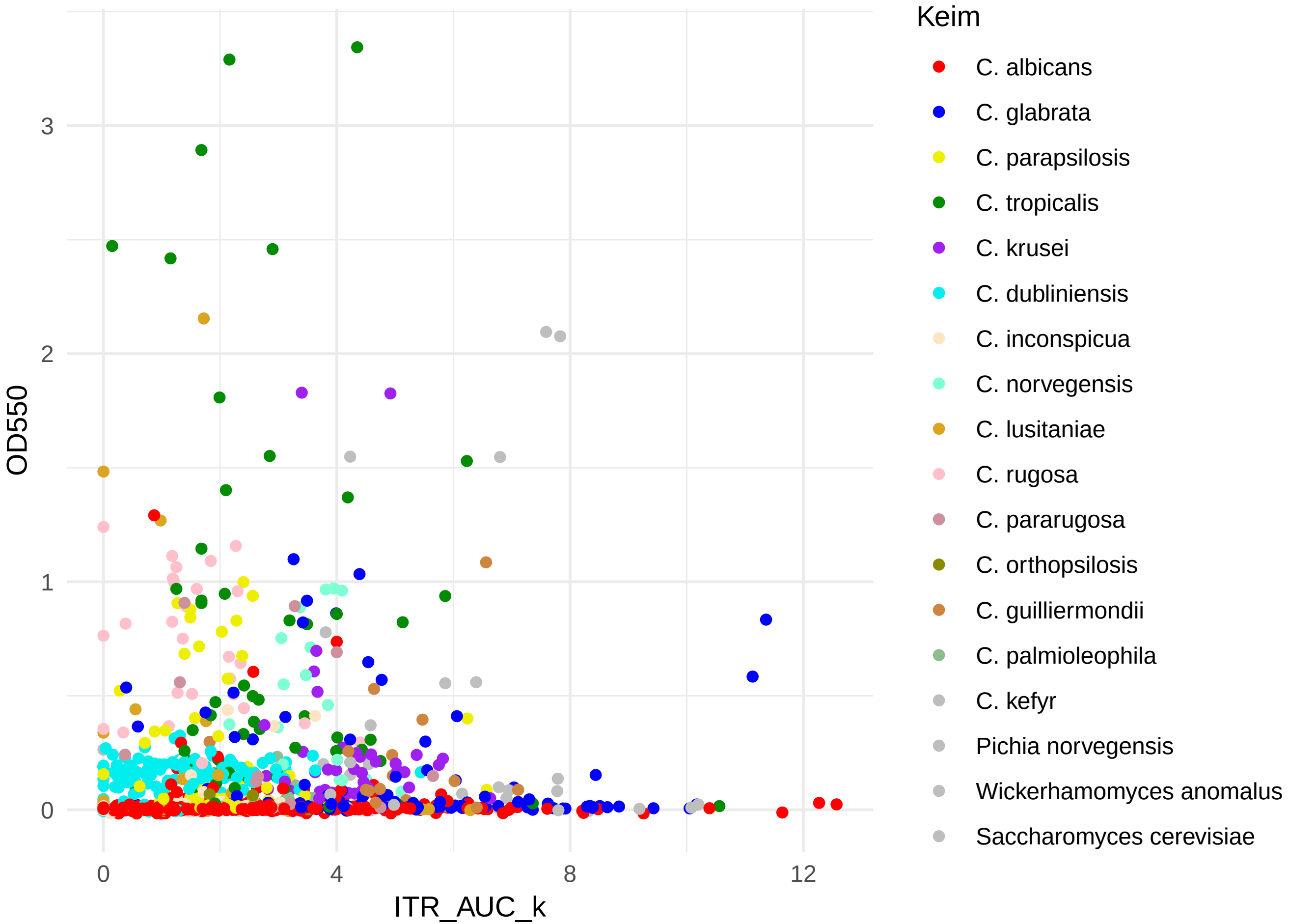
OD550
ITR_AUC_k
0
1
2
3
0
4
8
12
Keim
C. albicans
C. glabrata
C. parapsilosis
C. tropicalis
C. krusei
C. dubliniensis
C. inconspicua
C. norvegensis
C. lusitaniae
C. rugosa
C. pararugosa
C. orthopsilosis
C. guilliermondii
C. palmioleophila
C. kefyr
Pichia norvegensis
Wickerhamomyces anomalus
Saccharomyces cerevisiae

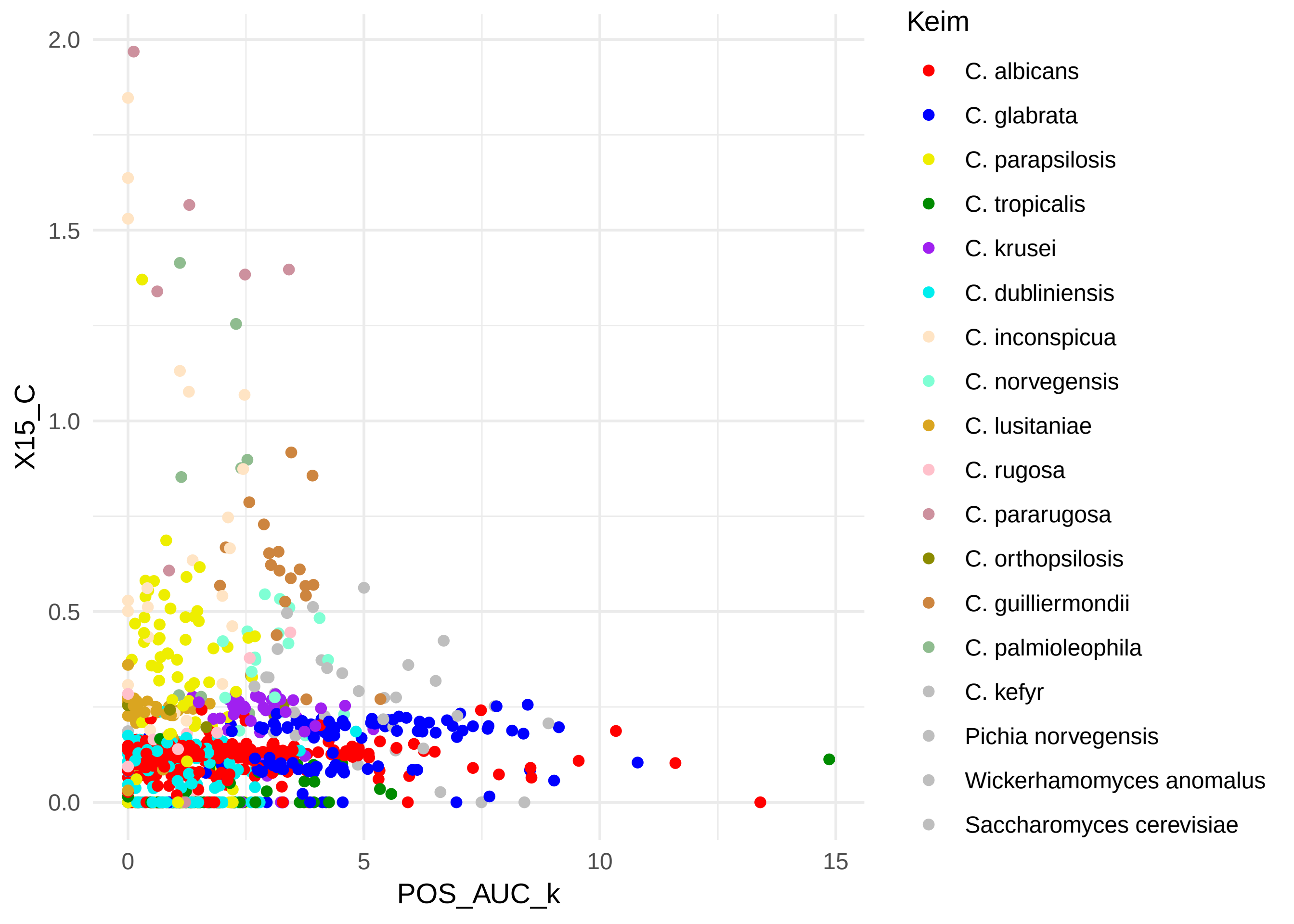

Keim
C. albicans
C. glabrata
C. parapsilosis
C. tropicalis
C. krusei
C. dubliniensis
C. inconspicua
C. norvegensis
C. lusitaniae
C. rugosa
C. pararugosa
C. orthopsilosis
C. guilliermondii
C. palmioleophila
C. kefyr
Pichia norvegensis
Wickerhamomyces anomalus
Saccharomyces cerevisiae
X15_C
2.0
1.5
1.0
0.5
0.0
0
5
10
15
POS_AUC_k

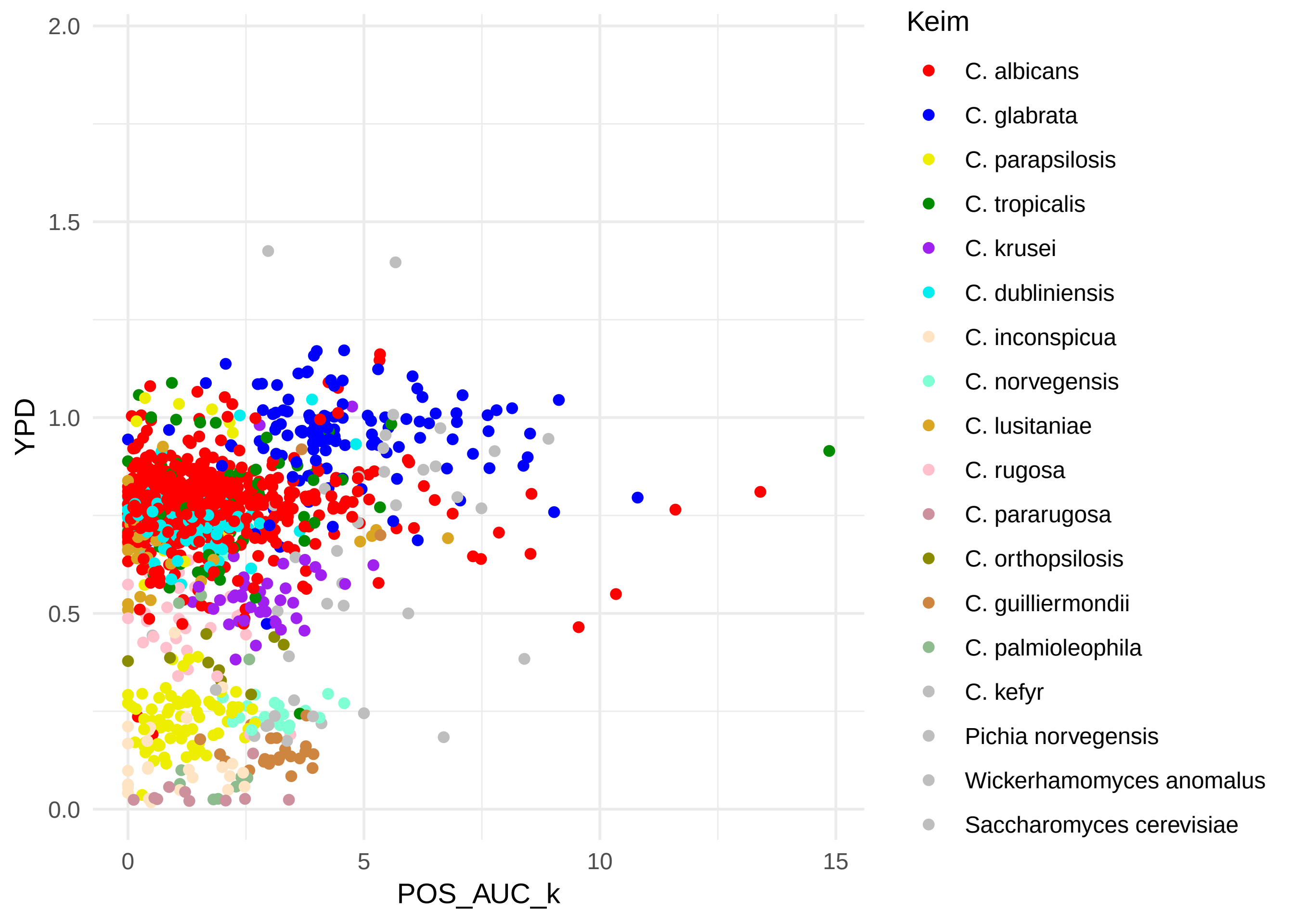

YPD
2.0
1.5
1.0
0.5
0.0
0
5
10
15
POS_AUC_k
Keim
C. albicans
C. glabrata
C. parapsilosis
C. tropicalis
C. krusei
C. dubliniensis
C. inconspicua
C. norvegensis
C. lusitaniae
C. rugosa
C. pararugosa
C. orthopsilosis
C. guilliermondii
C. palmioleophila
C. kefyr
Pichia norvegensis
Wickerhamomyces anomalus
Saccharomyces cerevisiae

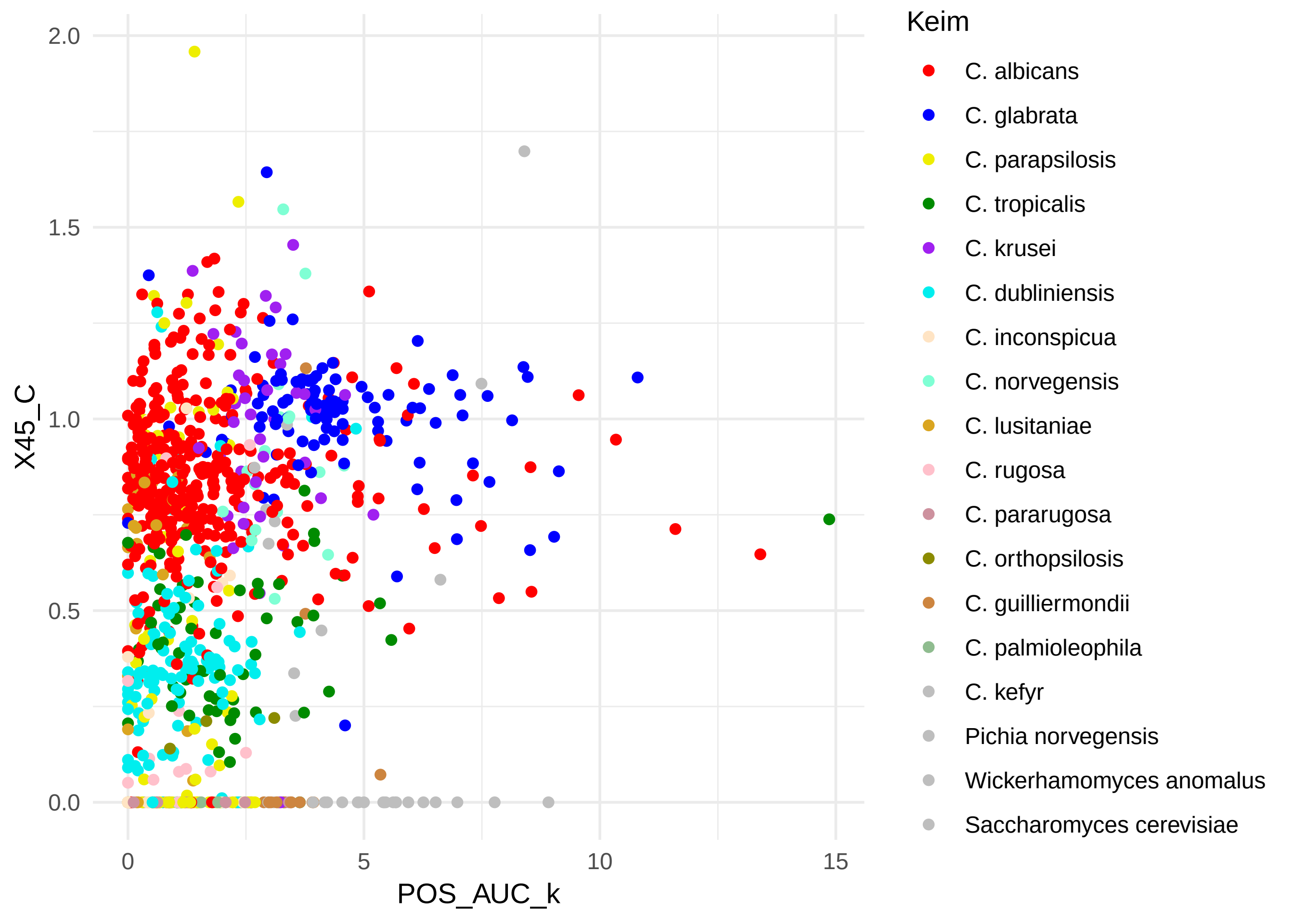
Keim
C. albicans
C. glabrata
C. parapsilosis
C. tropicalis
C. krusei
C. dubliniensis
C. inconspicua
C. norvegensis
C. lusitaniae
C. rugosa
C. pararugosa
C. orthopsilosis
C. guilliermondii
C. palmioleophila
C. kefyr
Pichia norvegensis
Wickerhamomyces anomalus
Saccharomyces cerevisiae
X45_C
2.0
1.5
1.0
0.5
0.0
0
5
10
15
POS_AUC_k

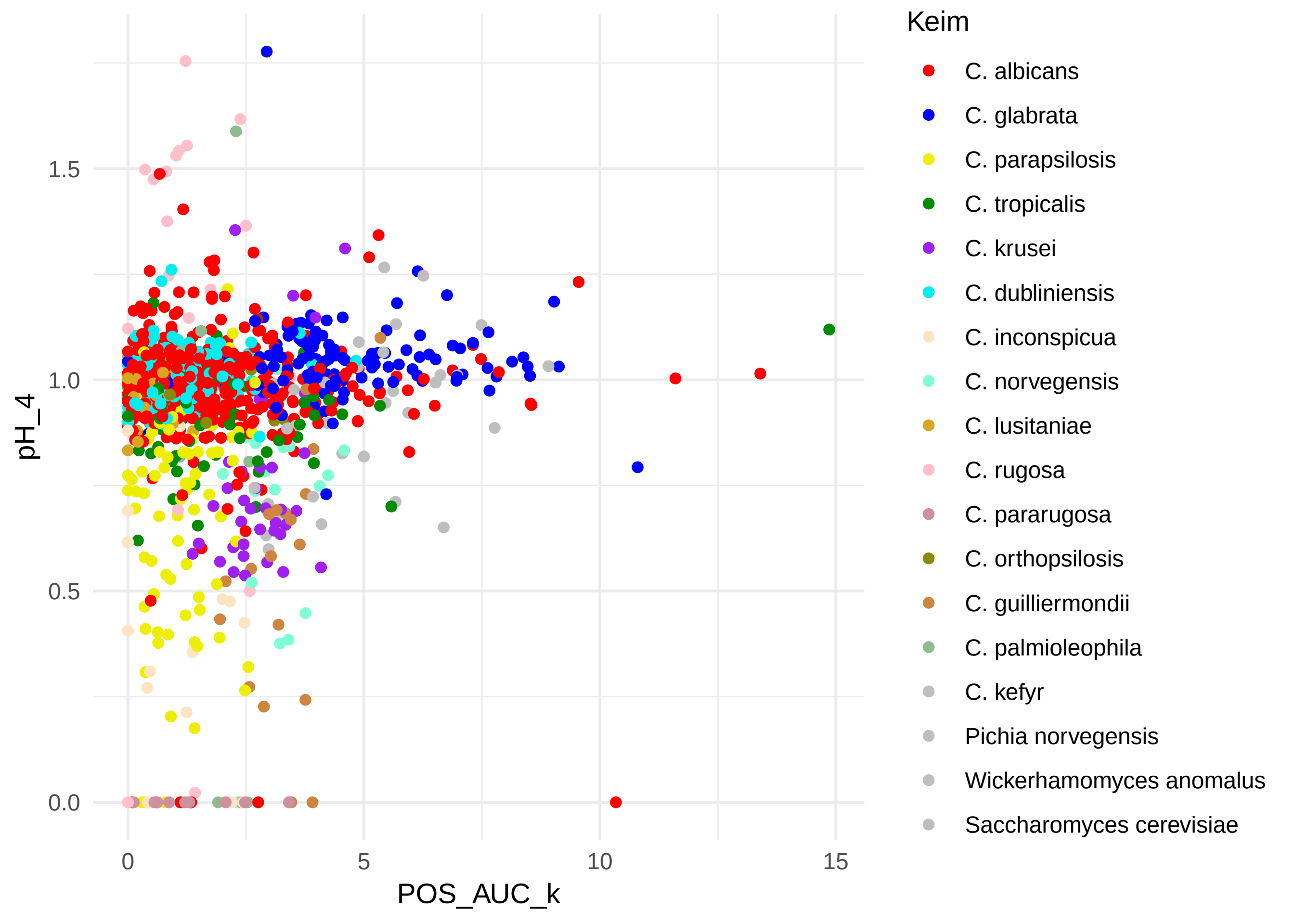
pH_4
1.5
1.0
0.5
0.0
0
5
10
15
POS_AUC_k
Keim
C. albicans
C. glabrata
C. parapsilosis
C. tropicalis
C. krusei
C. dubliniensis
C. inconspicua
C. norvegensis
C. lusitaniae
C. rugosa
C. pararugosa
C. orthopsilosis
C. guilliermondii
C. palmioleophila
C. kefyr
Pichia norvegensis
Wickerhamomyces anomalus
Saccharomyces cerevisiae

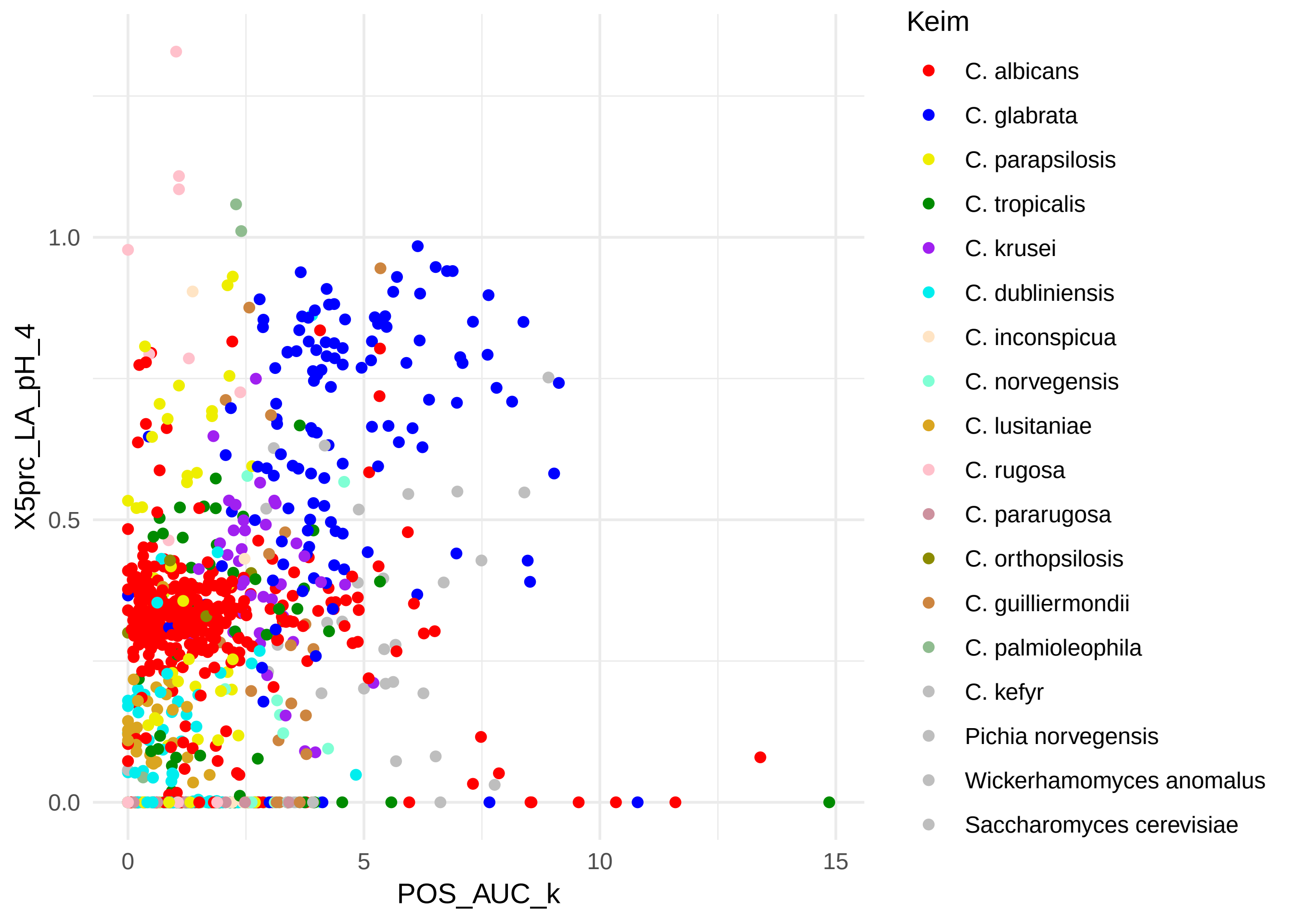
Keim
C. albicans
C. glabrata
C. parapsilosis
C. tropicalis
C. krusei
C. dubliniensis
C. inconspicua
C. norvegensis
C. lusitaniae
C. rugosa
C. pararugosa
C. orthopsilosis
C. guilliermondii
C. palmioleophila
C. kefyr
Pichia norvegensis
Wickerhamomyces anomalus
Saccharomyces cerevisiae
X5prc_LA_pH_4
1.0
0.5
0.0
0
5
10
15
POS_AUC_k

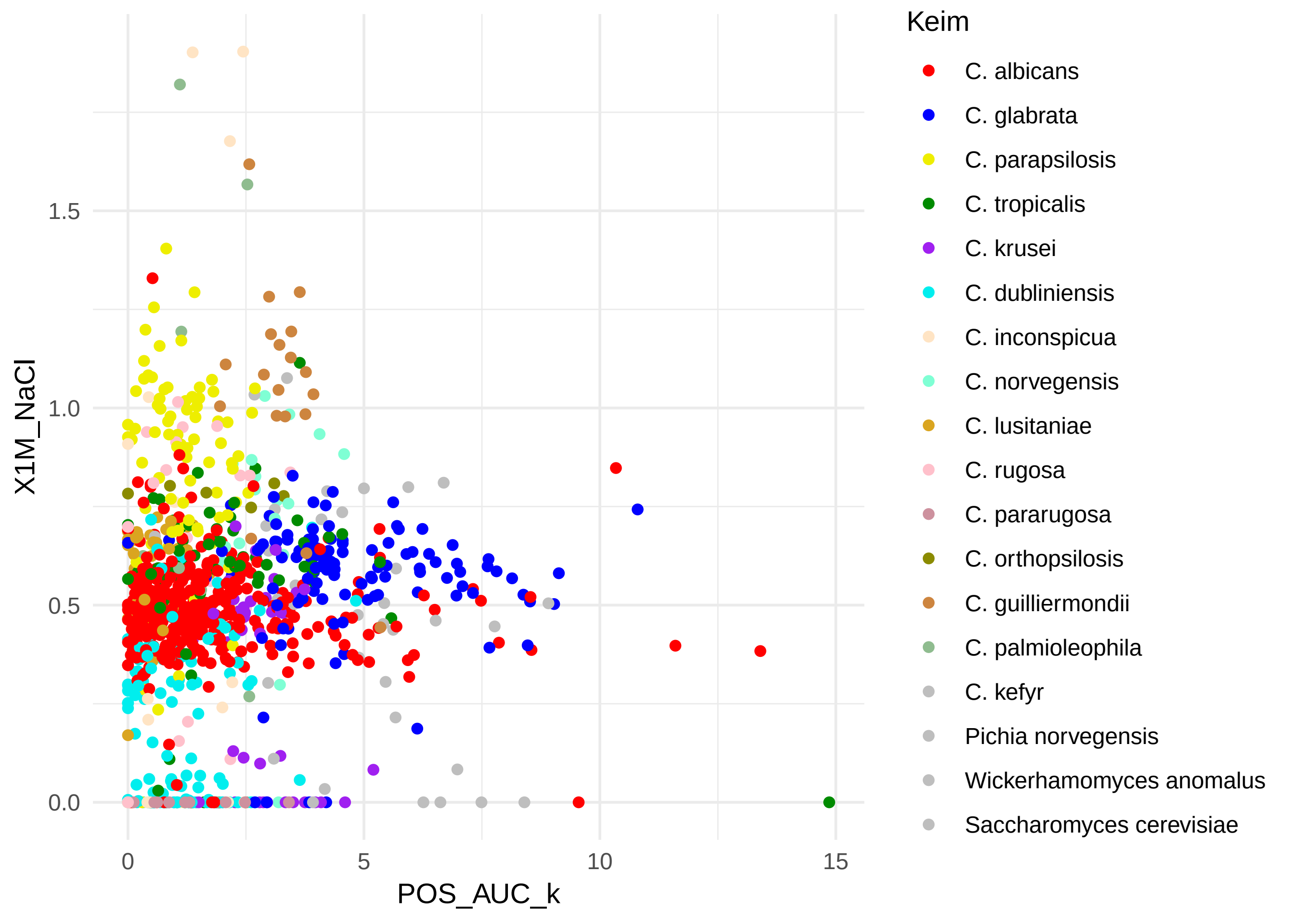

Keim
C. albicans
C. glabrata
C. parapsilosis
C. tropicalis
C. krusei
C. dubliniensis
C. inconspicua
C. norvegensis
C. lusitaniae
C. rugosa
C. pararugosa
C. orthopsilosis
C. guilliermondii
C. palmioleophila
C. kefyr
Pichia norvegensis
Wickerhamomyces anomalus
Saccharomyces cerevisiae
X1M_NaCl
1.5
1.0
0.5
0.0
0
5
10
15
POS_AUC_k

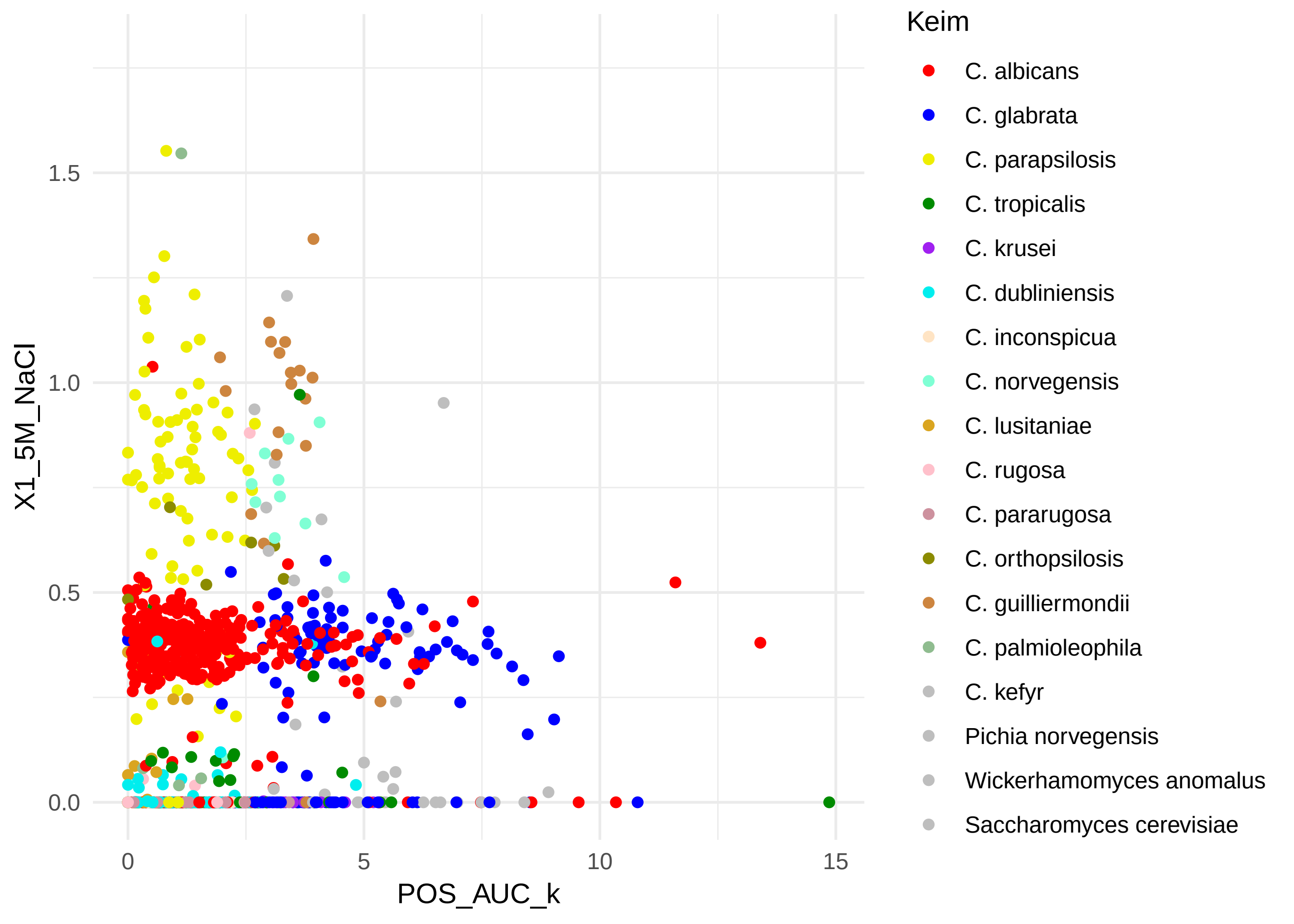

Keim
C. albicans
C. glabrata
C. parapsilosis
C. tropicalis
C. krusei
C. dubliniensis
C. inconspicua
C. norvegensis
C. lusitaniae
C. rugosa
C. pararugosa
C. orthopsilosis
C. guilliermondii
C. palmioleophila
C. kefyr
Pichia norvegensis
Wickerhamomyces anomalus
Saccharomyces cerevisiae
X1_5M_NaCl
POS_AUC_k
0.0
0.5
1.0
1.5
0
5
10
15

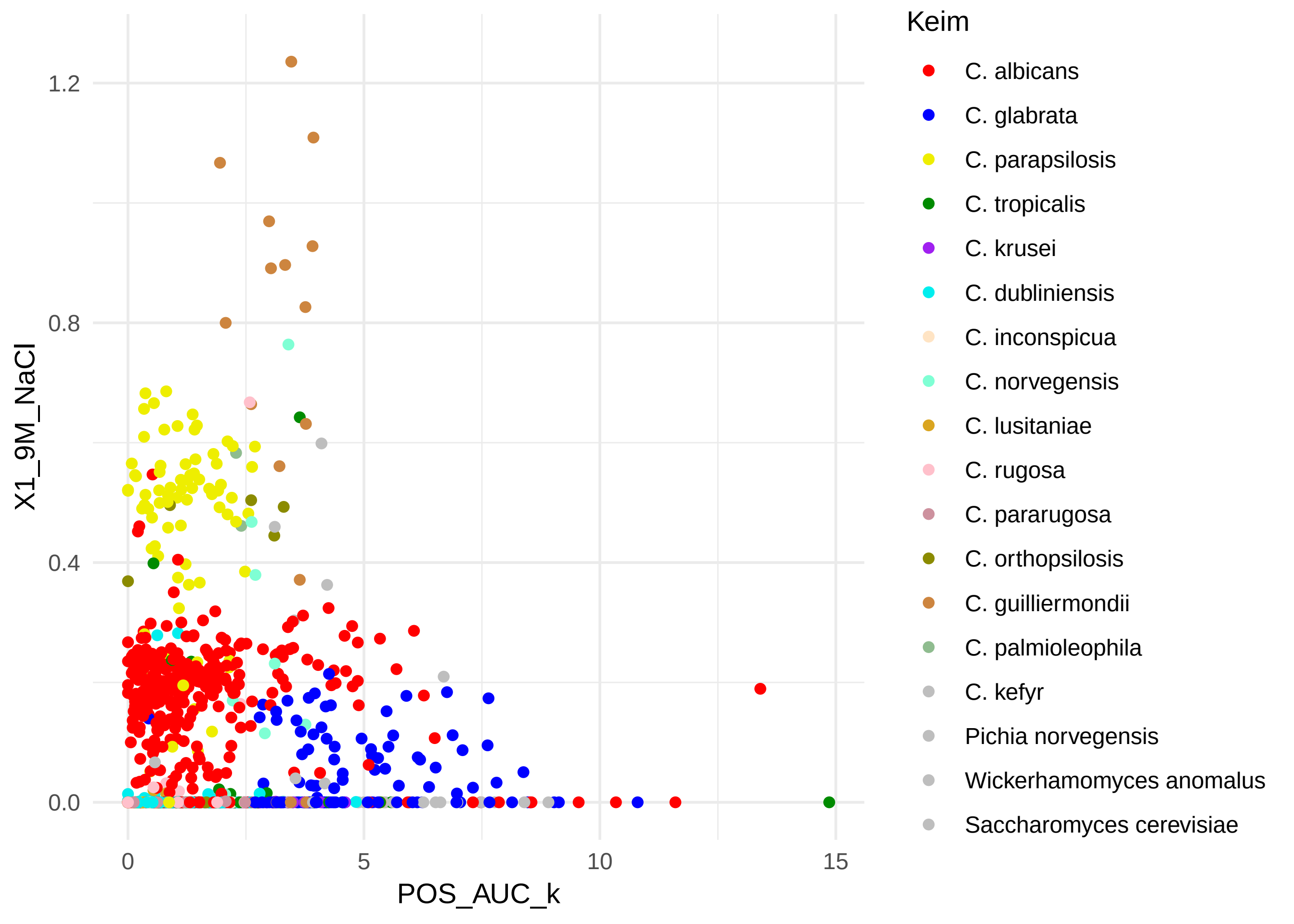

Keim
C. albicans
C. glabrata
C. parapsilosis
C. tropicalis
C. krusei
C. dubliniensis
C. inconspicua
C. norvegensis
C. lusitaniae
C. rugosa
C. pararugosa
C. orthopsilosis
C. guilliermondii
C. palmioleophila
C. kefyr
Pichia norvegensis
Wickerhamomyces anomalus
Saccharomyces cerevisiae
X1_9M_NaCl
1.2
0.8
0.4
0.0
0
5
10
15
POS_AUC_k

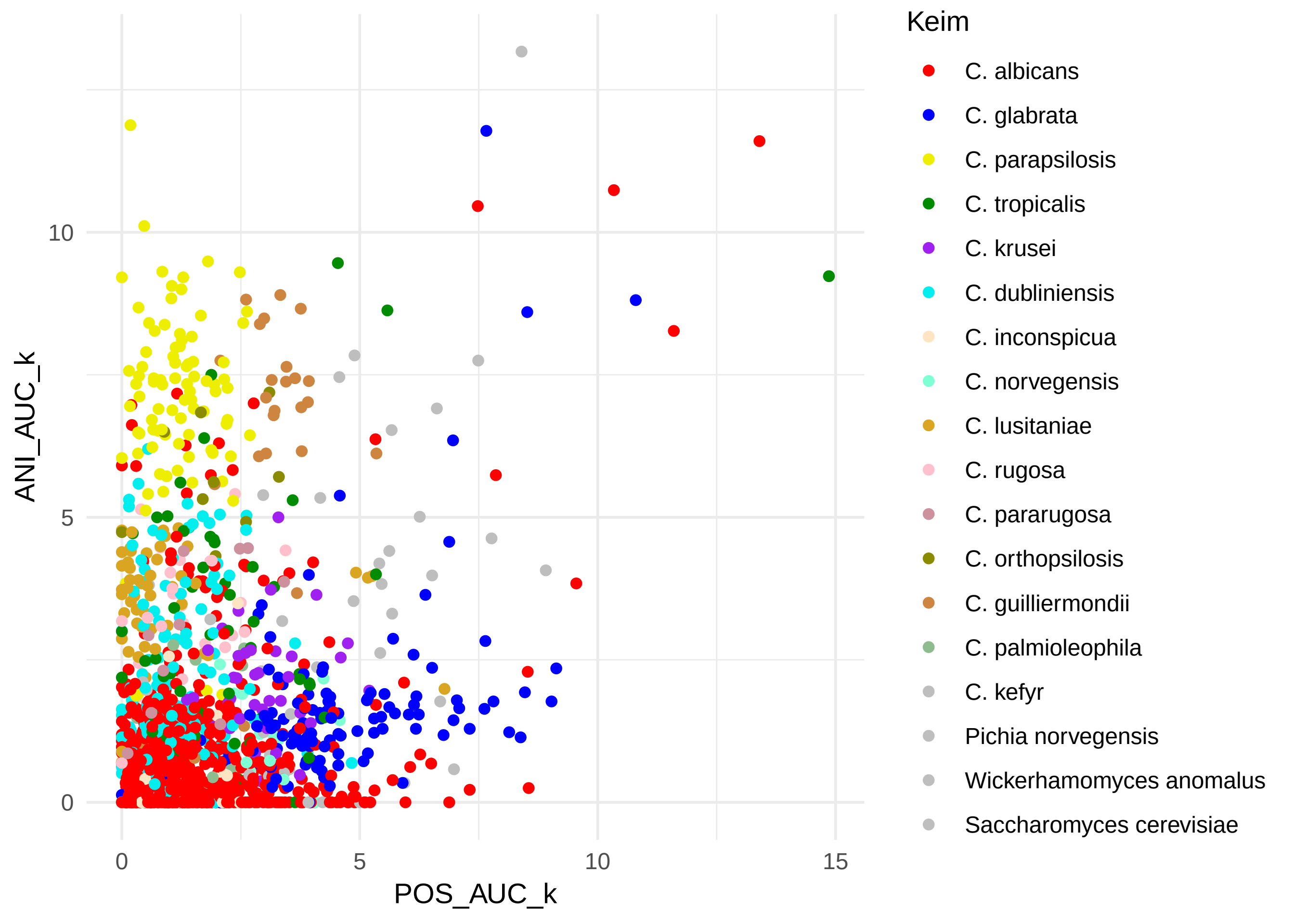
Keim
C. albicans
C. glabrata
C. parapsilosis
C. tropicalis
C. krusei
C. dubliniensis
C. inconspicua
C. norvegensis
C. lusitaniae
C. rugosa
C. pararugosa
C. orthopsilosis
C. guilliermondii
C. palmioleophila
C. kefyr
Pichia norvegensis
Wickerhamomyces anomalus
Saccharomyces cerevisiae
ANI_AUC_k
POS_AUC_k
0
5
10
15

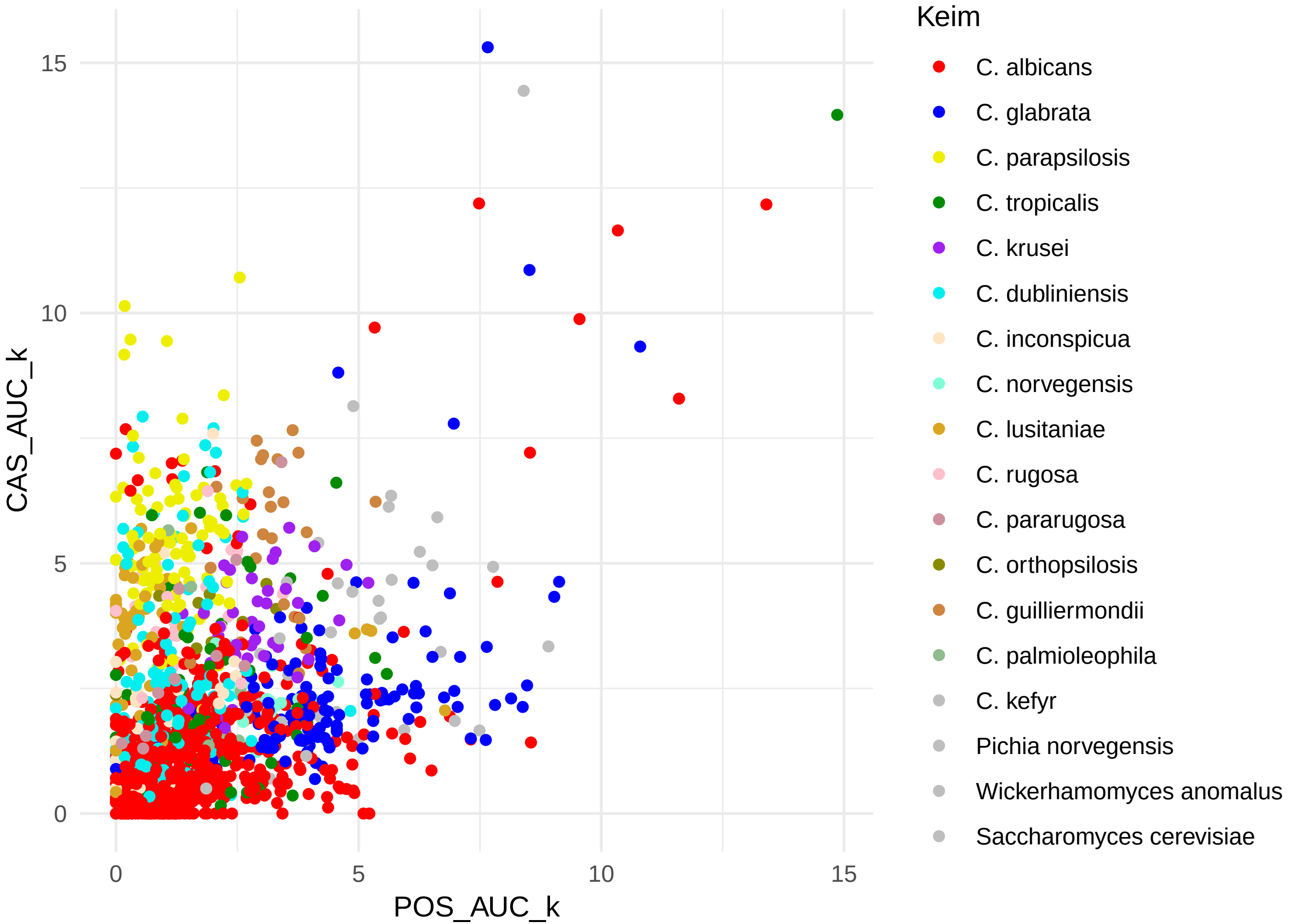

Keim
C. albicans
C. glabrata
C. parapsilosis
C. tropicalis
C. krusei
C. dubliniensis
C. inconspicua
C. norvegensis
C. lusitaniae
C. rugosa
C. pararugosa
C. orthopsilosis
C. guilliermondii
C. palmioleophila
C. kefyr
Pichia norvegensis
Wickerhamomyces anomalus
Saccharomyces cerevisiae
CAS_AUC_k
POS_AUC_k
0
5
10
15

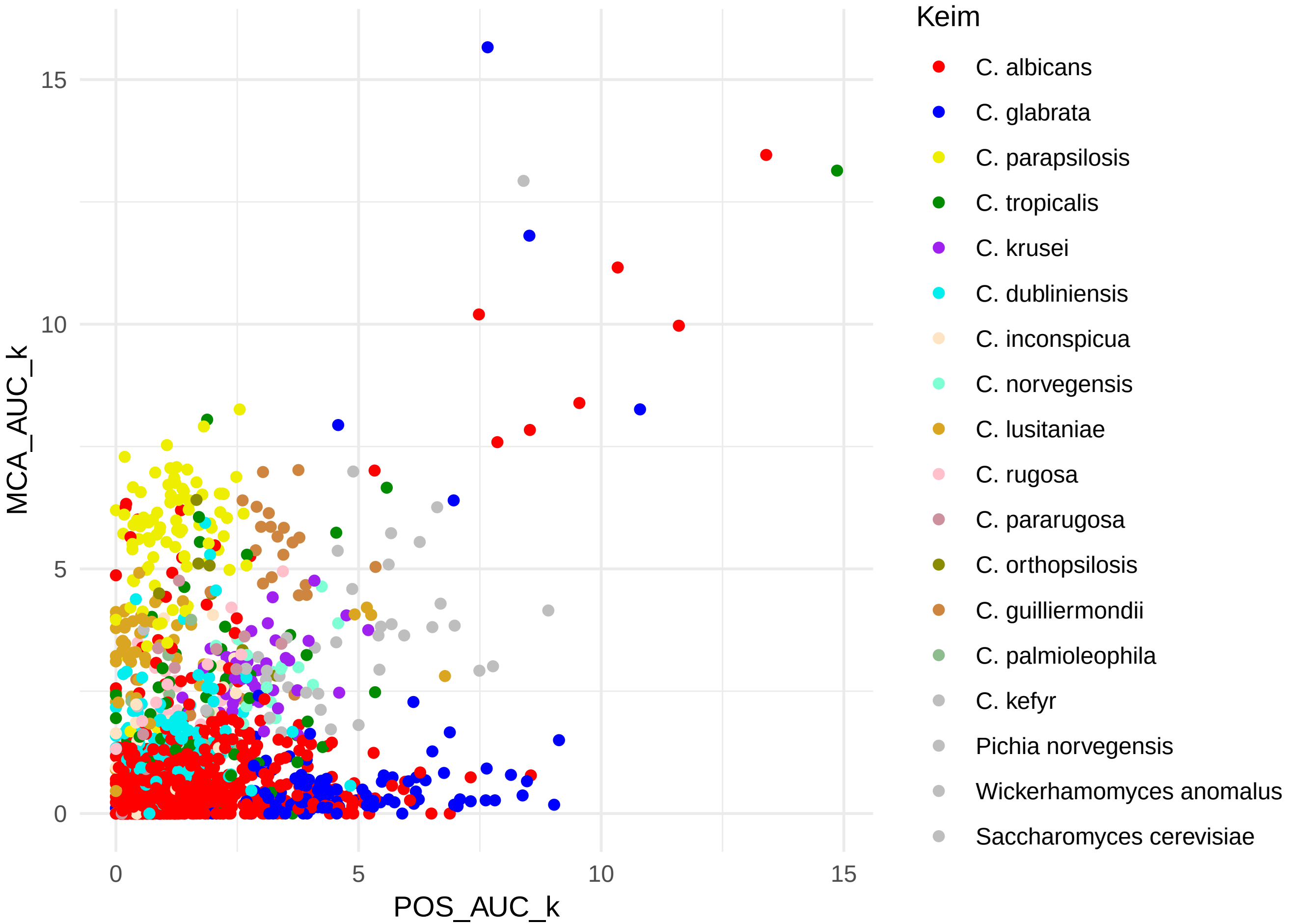

Keim
C. albicans
C. glabrata
C. parapsilosis
C. tropicalis
C. krusei
C. dubliniensis
C. inconspicua
C. norvegensis
C. lusitaniae
C. rugosa
C. pararugosa
C. orthopsilosis
C. guilliermondii
C. palmioleophila
C. kefyr
Pichia norvegensis
Wickerhamomyces anomalus
Saccharomyces cerevisiae
MCA_AUC_k
POS_AUC_k
0
5
10
15

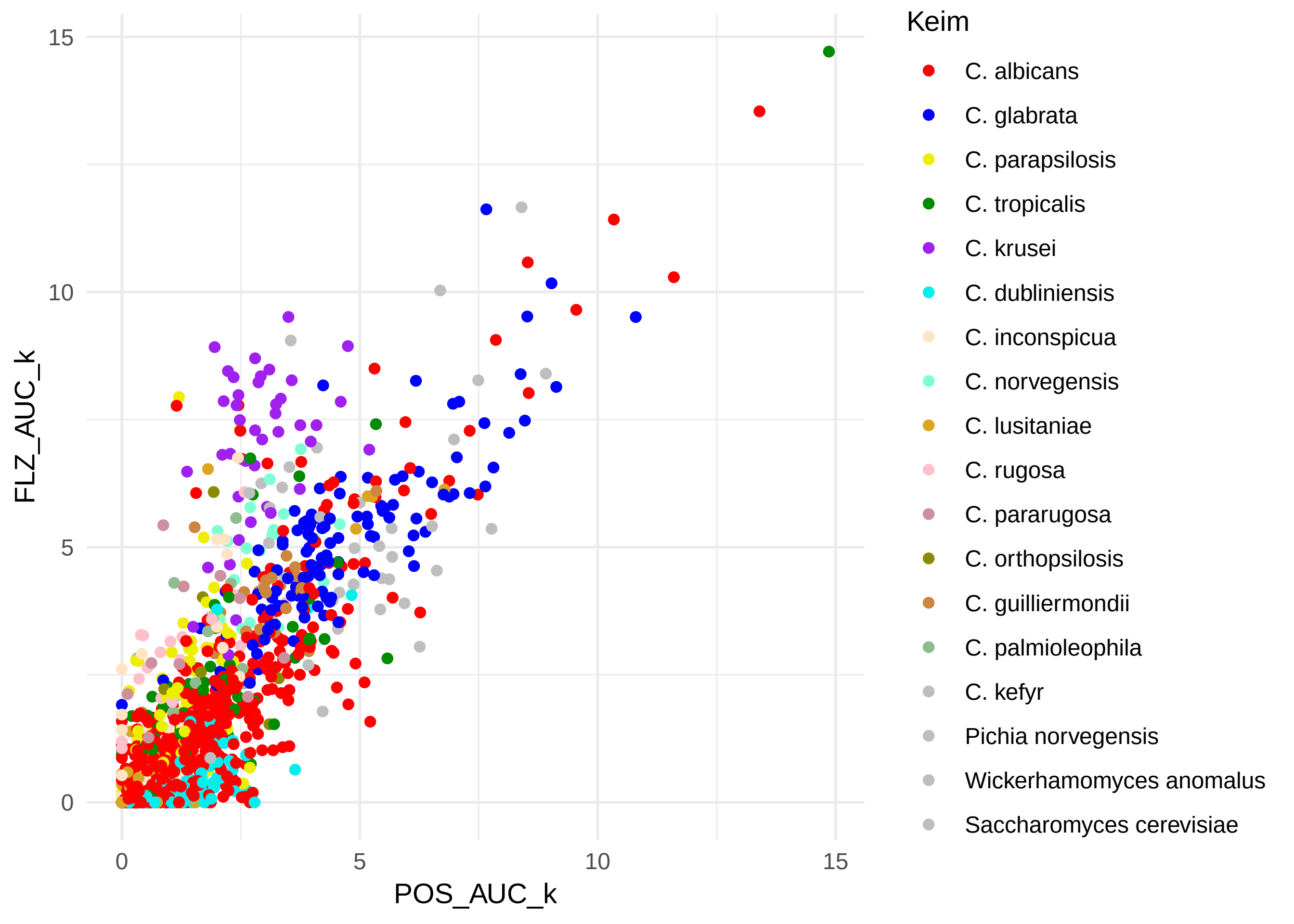
FLZ_AUC_k
POS_AUC_k
0
5
10
15
0
5
10
15
Keim
C. albicans
C. glabrata
C. parapsilosis
C. tropicalis
C. krusei
C. dubliniensis
C. inconspicua
C. norvegensis
C. lusitaniae
C. rugosa
C. pararugosa
C. orthopsilosis
C. guilliermondii
C. palmioleophila
C. kefyr
Pichia norvegensis
Wickerhamomyces anomalus
Saccharomyces cerevisiae

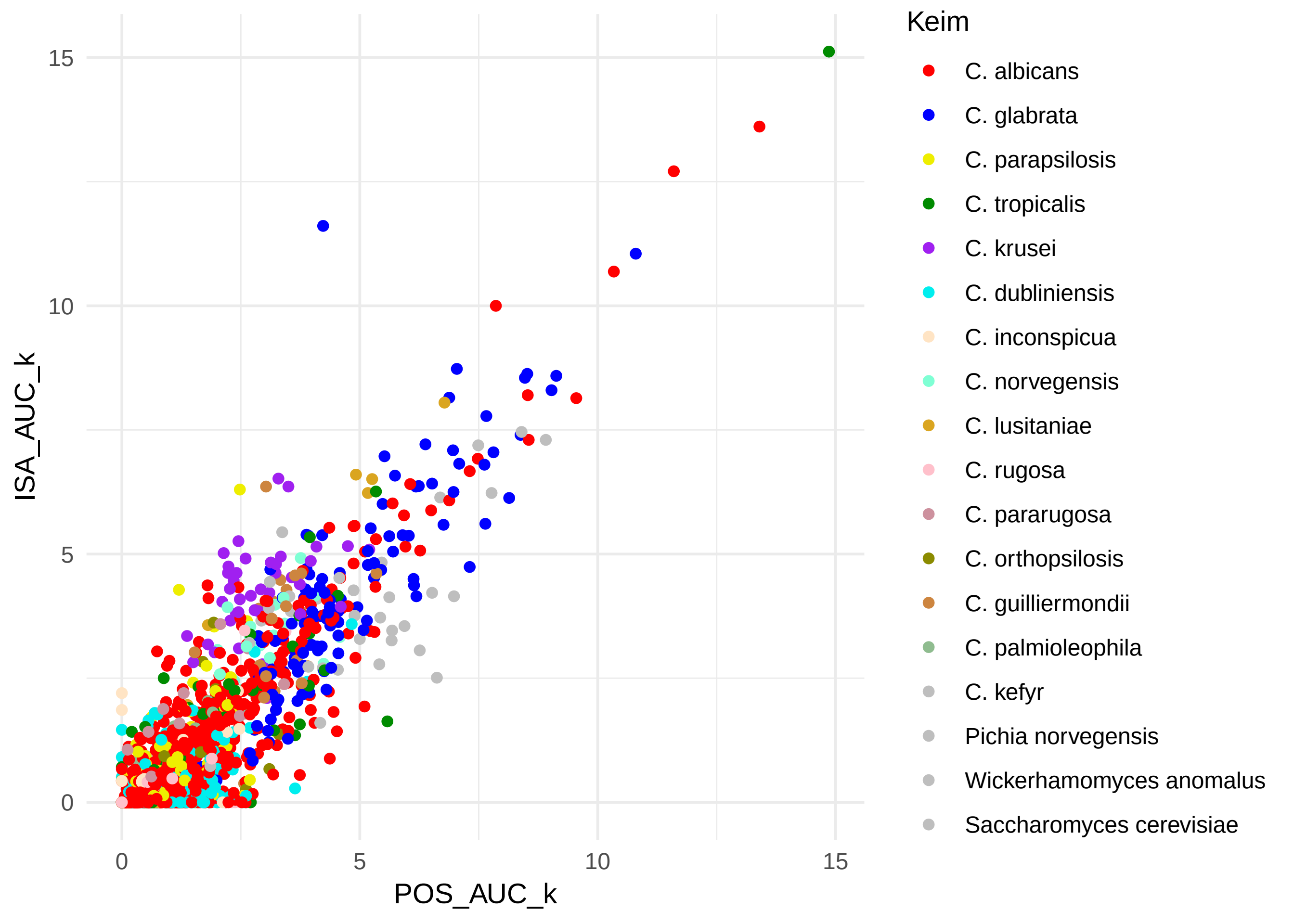
Keim
C. albicans
C. glabrata
C. parapsilosis
C. tropicalis
C. krusei
C. dubliniensis
C. inconspicua
C. norvegensis
C. lusitaniae
C. rugosa
C. pararugosa
C. orthopsilosis
C. guilliermondii
C. palmioleophila
C. kefyr
Pichia norvegensis
Wickerhamomyces anomalus
Saccharomyces cerevisiae
ISA_AUC_k
POS_AUC_k
0
5
10
15
0
5
10
15

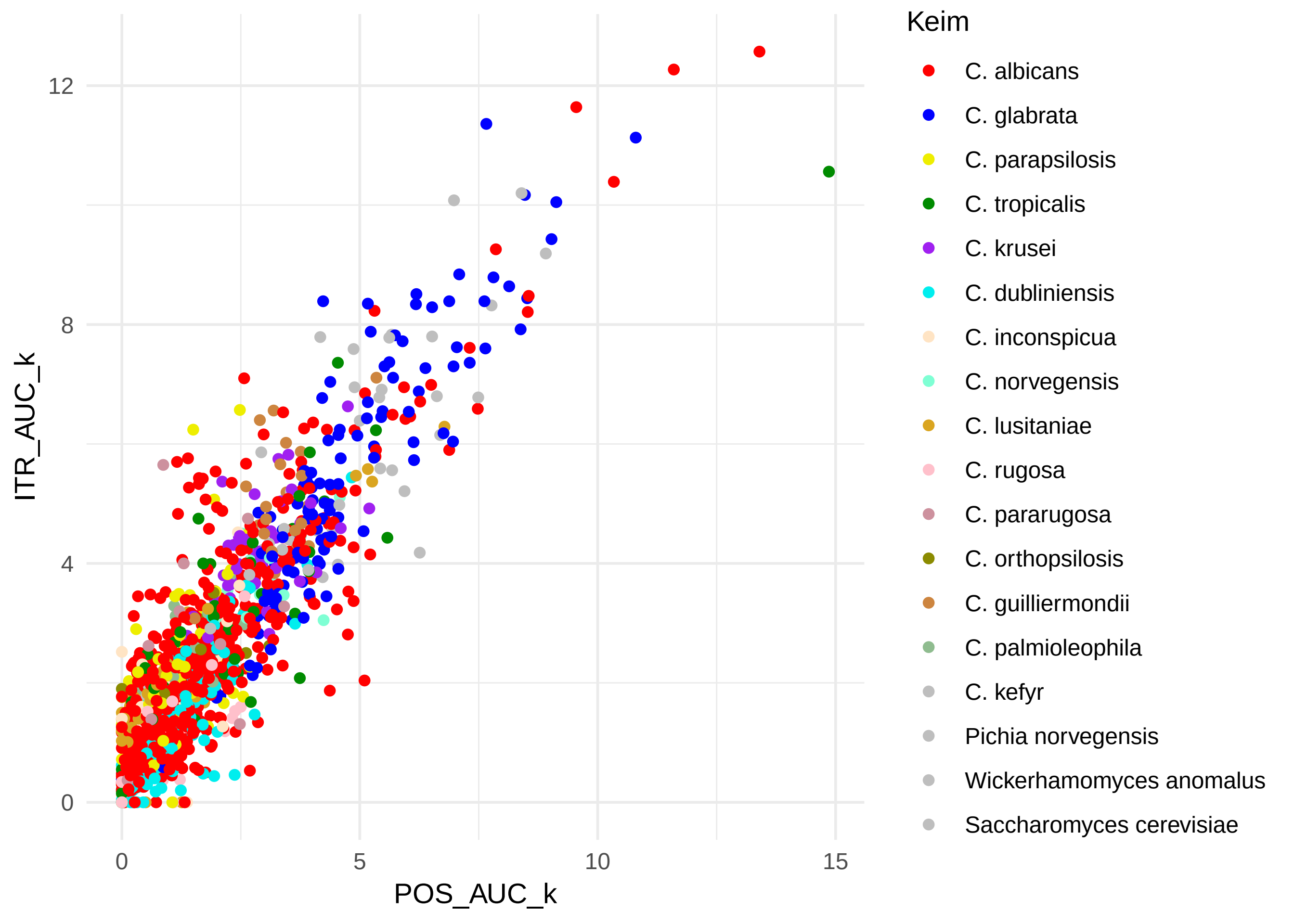
Keim
C. albicans
C. glabrata
C. parapsilosis
C. tropicalis
C. krusei
C. dubliniensis
C. inconspicua
C. norvegensis
C. lusitaniae
C. rugosa
C. pararugosa
C. orthopsilosis
C. guilliermondii
C. palmioleophila
C. kefyr
Pichia norvegensis
Wickerhamomyces anomalus
Saccharomyces cerevisiae
ITR_AUC_k
POS_AUC_k
0
4
8
12
0
5
10
15

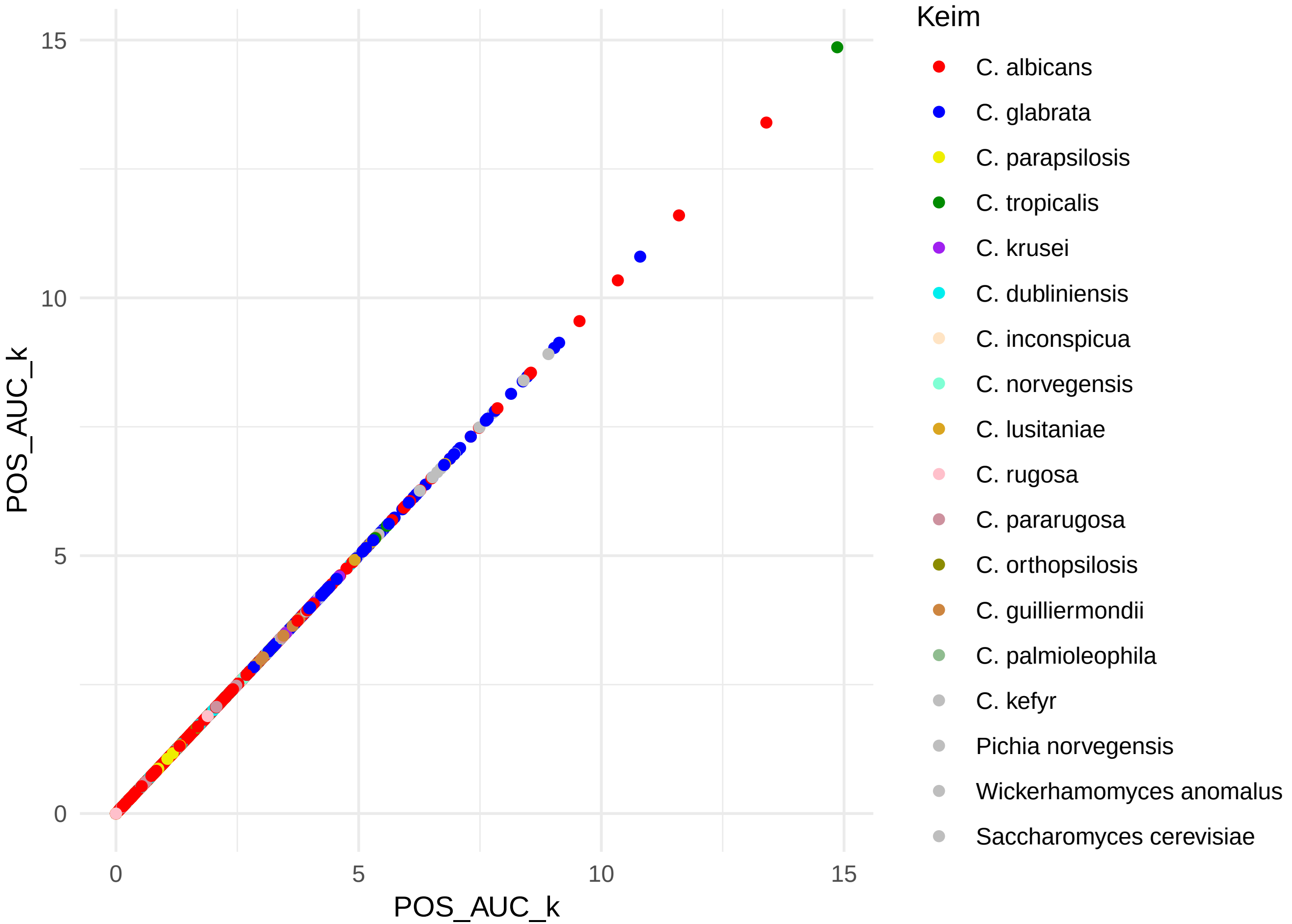

Keim
C. albicans
C. glabrata
C. parapsilosis
C. tropicalis
C. krusei
C. dubliniensis
C. inconspicua
C. norvegensis
C. lusitaniae
C. rugosa
C. pararugosa
C. orthopsilosis
C. guilliermondii
C. palmioleophila
C. kefyr
Pichia norvegensis
Wickerhamomyces anomalus
Saccharomyces cerevisiae
POS_AUC_k
POS_AUC_k
0
5
10
15
0
5
10
15

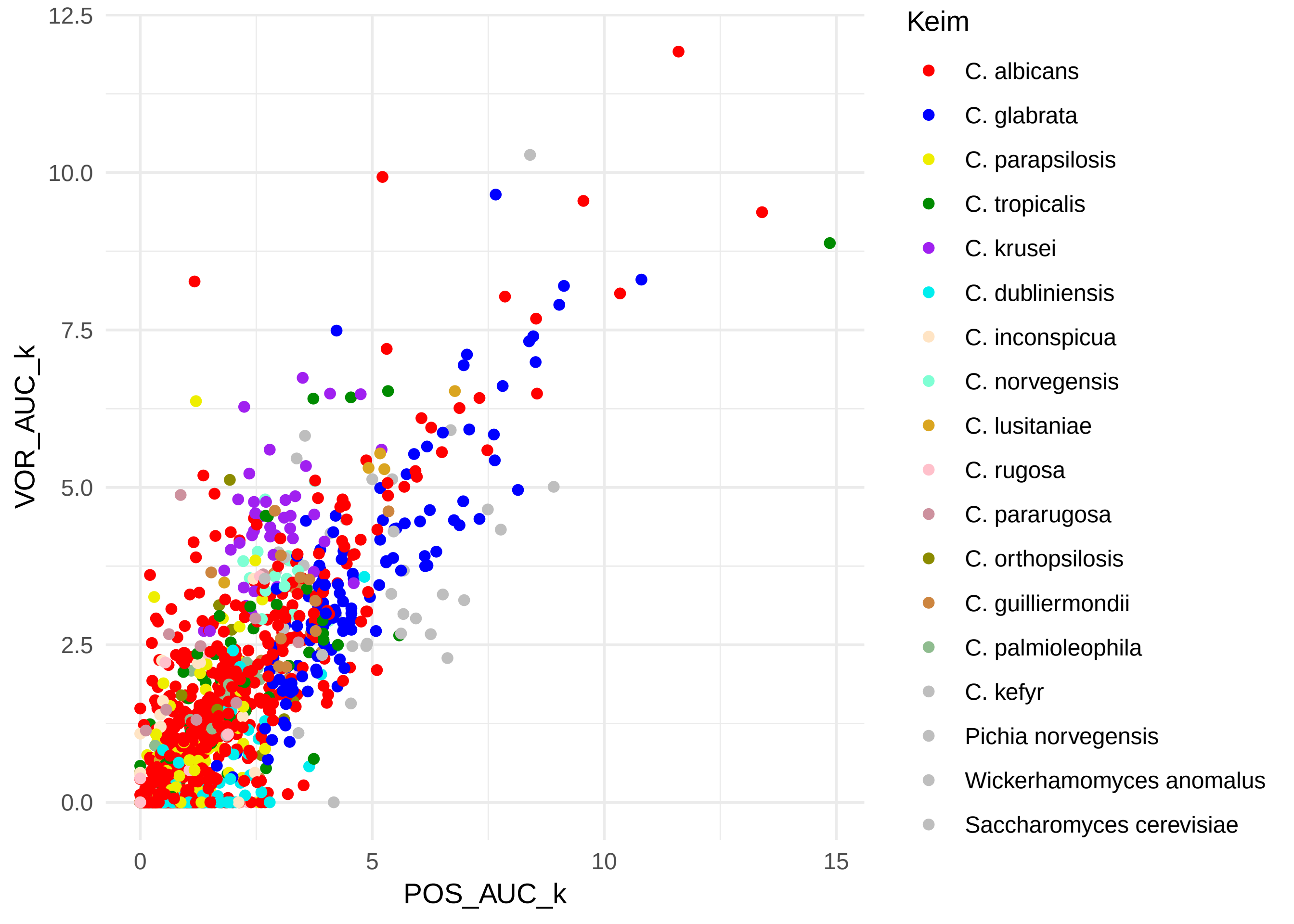
12.5
10.0
7.5
5.0
2.5
0.0
VOR_AUC_k
0
5
10
15
POS_AUC_k
Keim
C. albicans
C. glabrata
C. parapsilosis
C. tropicalis
C. krusei
C. dubliniensis
C. inconspicua
C. norvegensis
C. lusitaniae
C. rugosa
C. pararugosa
C. orthopsilosis
C. guilliermondii
C. palmioleophila
C. kefyr
Pichia norvegensis
Wickerhamomyces anomalus
Saccharomyces cerevisiae

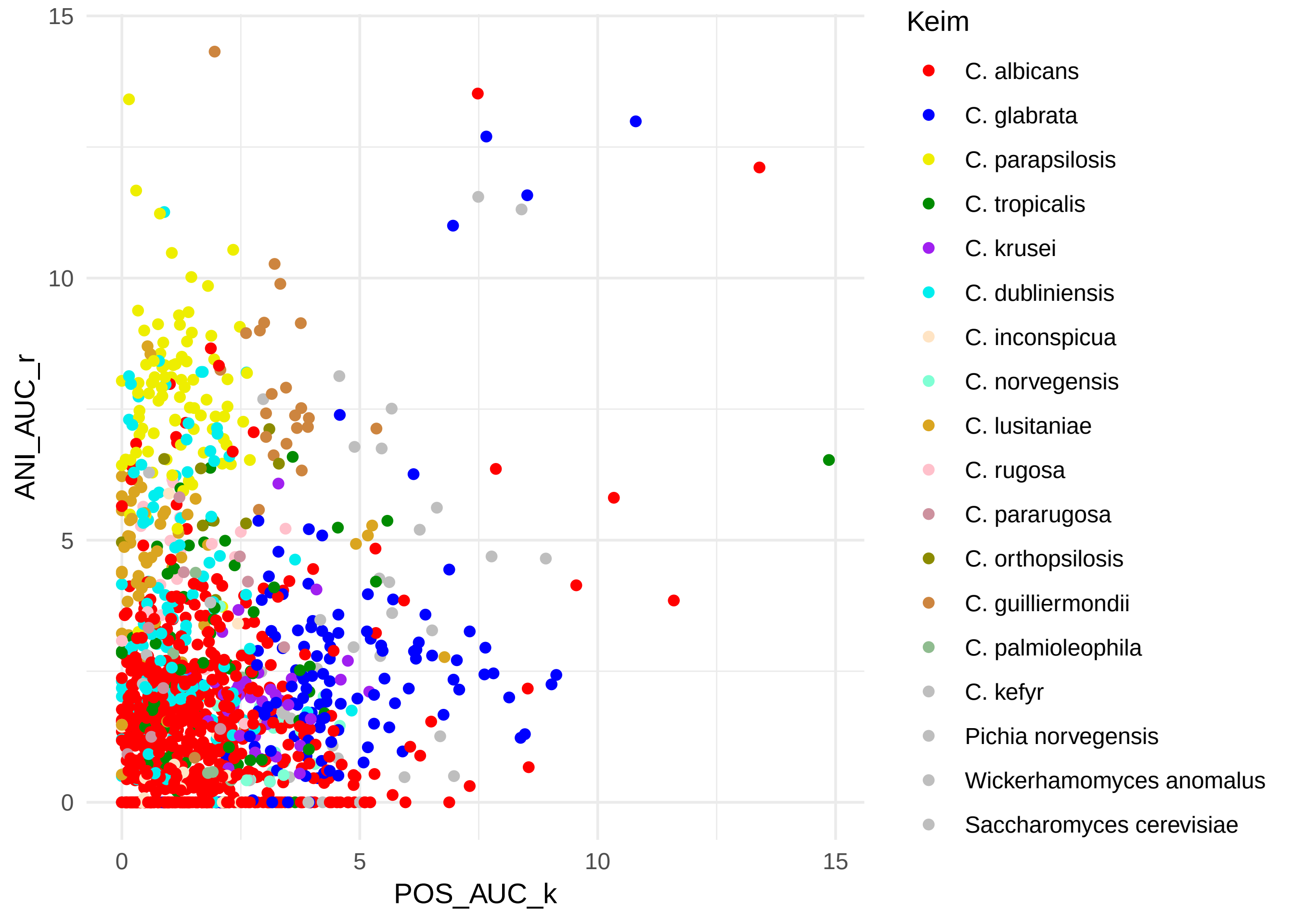

15
10
5
0
ANI_AUC_r
0
5
10
15
POS_AUC_k
Keim
C. albicans
C. glabrata
C. parapsilosis
C. tropicalis
C. krusei
C. dubliniensis
C. inconspicua
C. norvegensis
C. lusitaniae
C. rugosa
C. pararugosa
C. orthopsilosis
C. guilliermondii
C. palmioleophila
C. kefyr
Pichia norvegensis
Wickerhamomyces anomalus
Saccharomyces cerevisiae

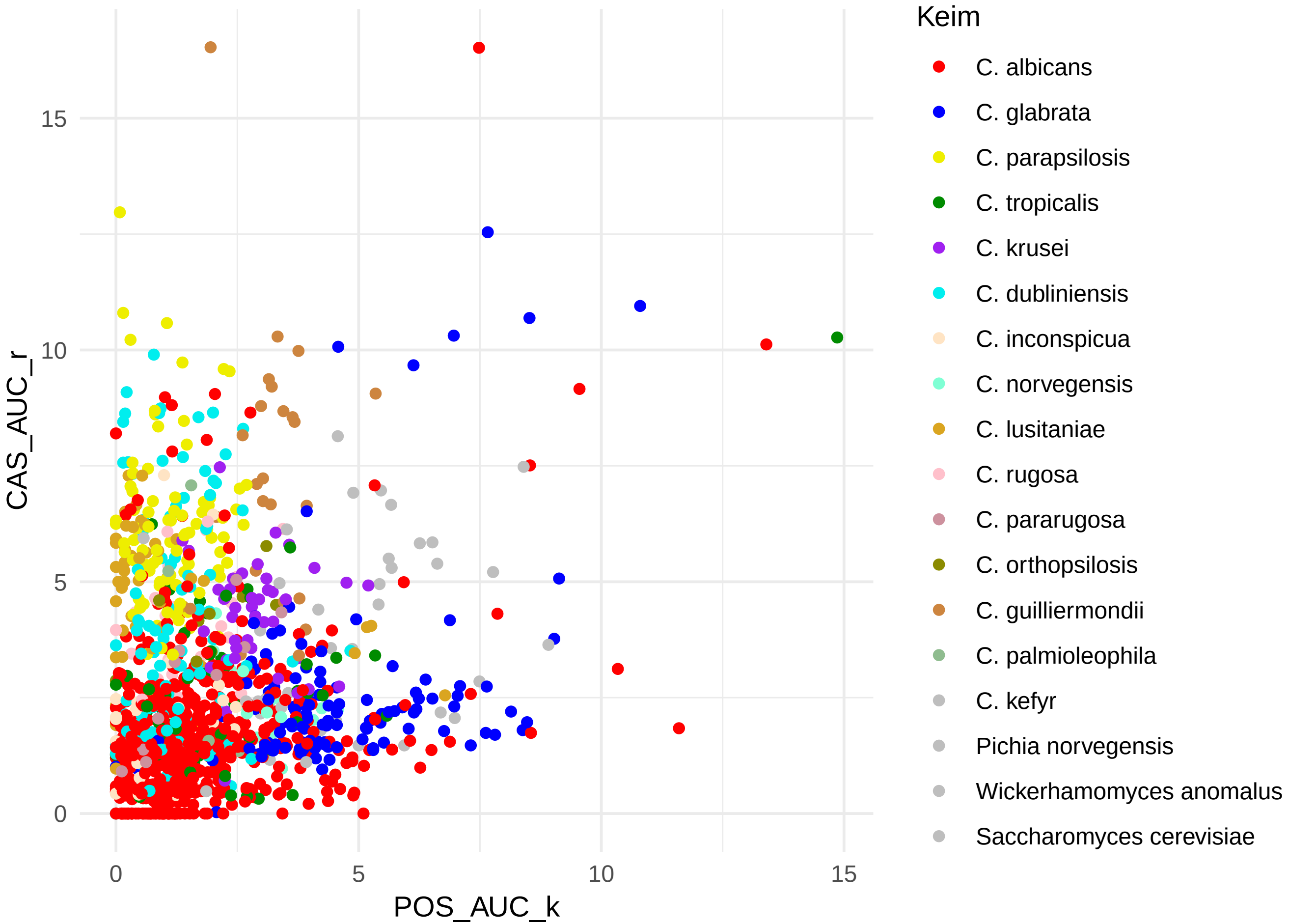

Keim
C. albicans
C. glabrata
C. parapsilosis
C. tropicalis
C. krusei
C. dubliniensis
C. inconspicua
C. norvegensis
C. lusitaniae
C. rugosa
C. pararugosa
C. orthopsilosis
C. guilliermondii
C. palmioleophila
C. kefyr
Pichia norvegensis
Wickerhamomyces anomalus
Saccharomyces cerevisiae
CAS_AUC_r
POS_AUC_k
0
5
10
15
0
5
10
15

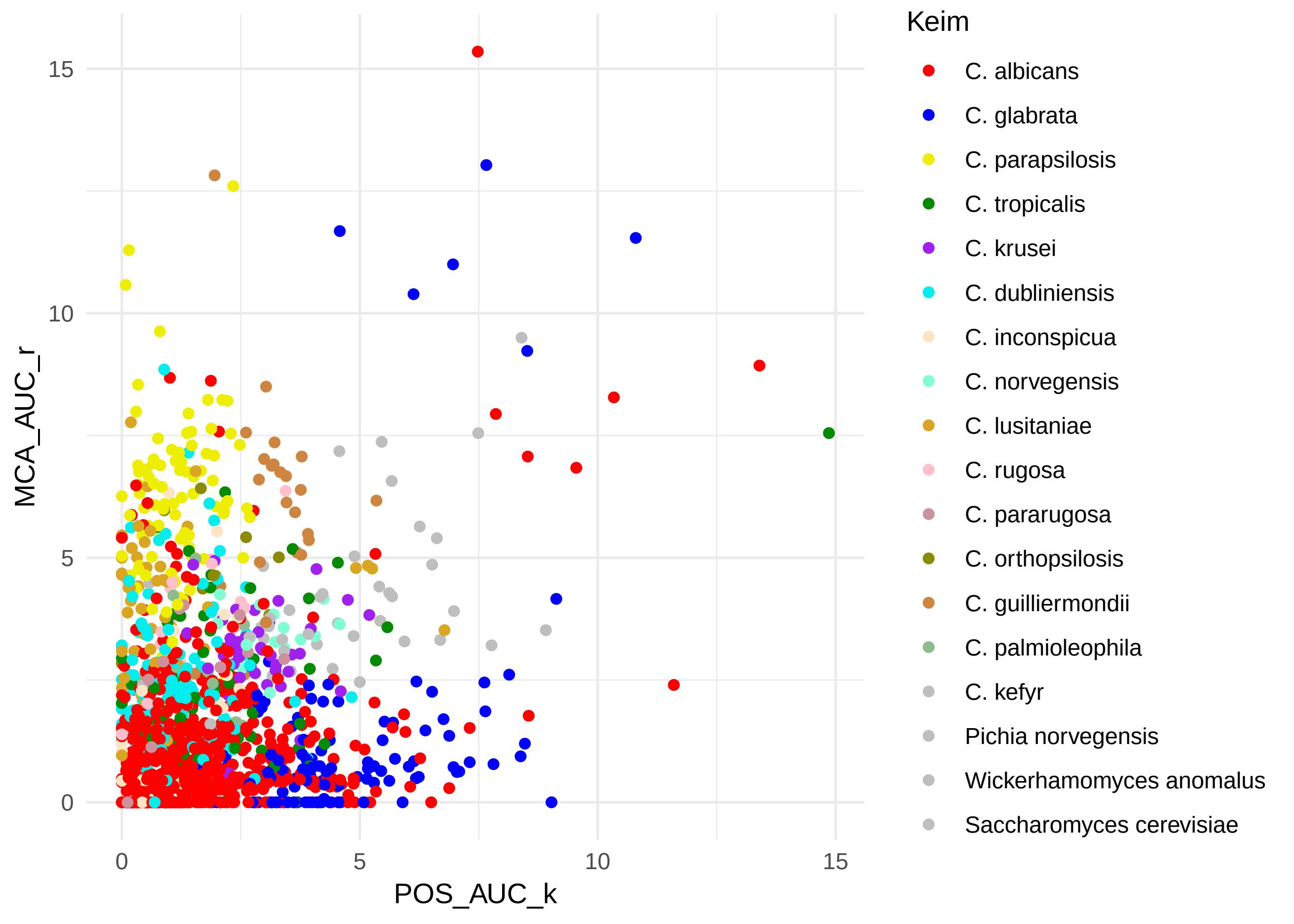

15
10
5
0
MCA_AUC_r
0
5
10
15
POS_AUC_k
Keim
C. albicans
C. glabrata
C. parapsilosis
C. tropicalis
C. krusei
C. dubliniensis
C. inconspicua
C. norvegensis
C. lusitaniae
C. rugosa
C. pararugosa
C. orthopsilosis
C. guilliermondii
C. palmioleophila
C. kefyr
Pichia norvegensis
Wickerhamomyces anomalus
Saccharomyces cerevisiae

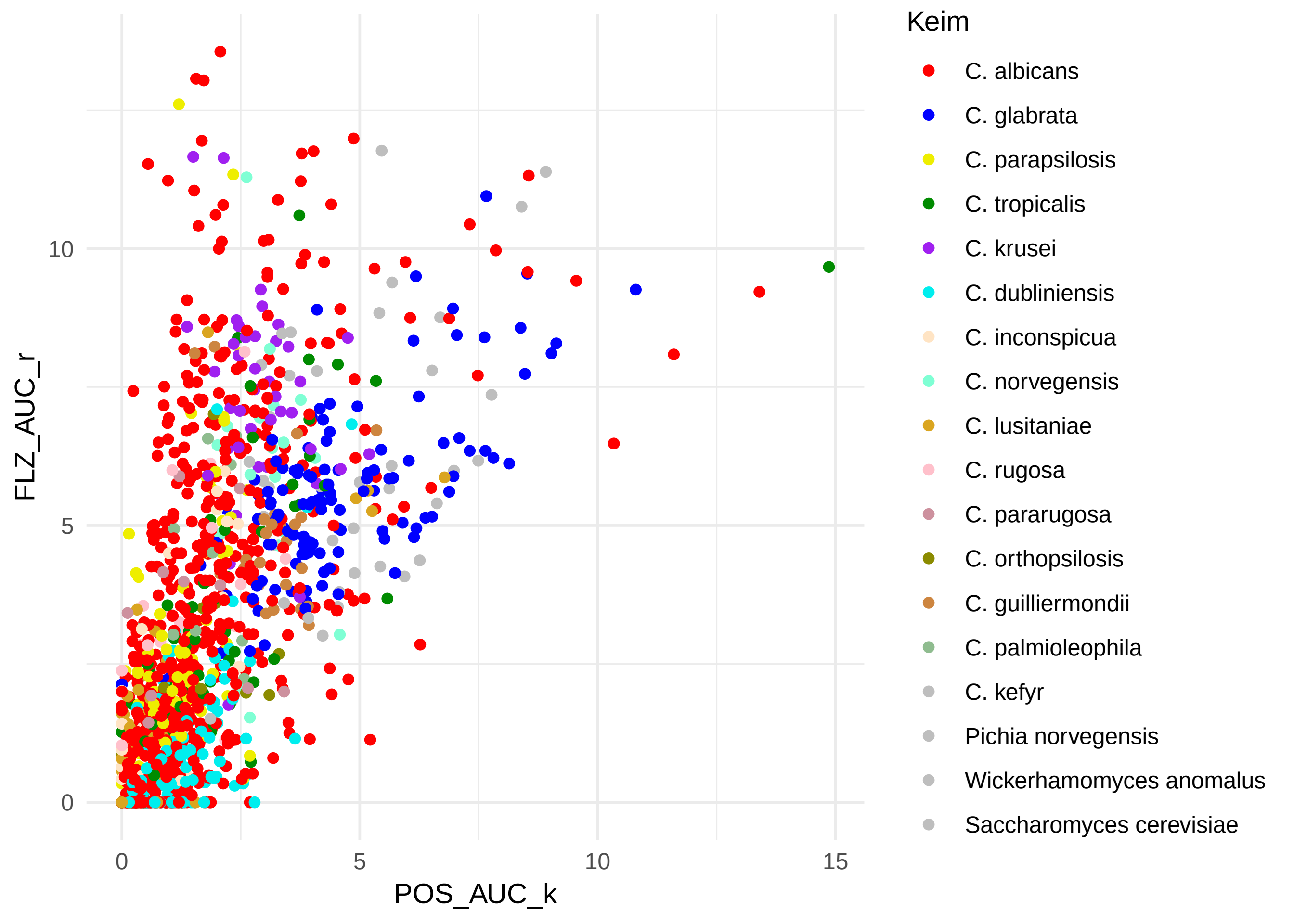

Keim
C. albicans
C. glabrata
C. parapsilosis
C. tropicalis
C. krusei
C. dubliniensis
C. inconspicua
C. norvegensis
C. lusitaniae
C. rugosa
C. pararugosa
C. orthopsilosis
C. guilliermondii
C. palmioleophila
C. kefyr
Pichia norvegensis
Wickerhamomyces anomalus
Saccharomyces cerevisiae
FLZ_AUC_r
POS_AUC_k
0
5
10
15
0
5
10

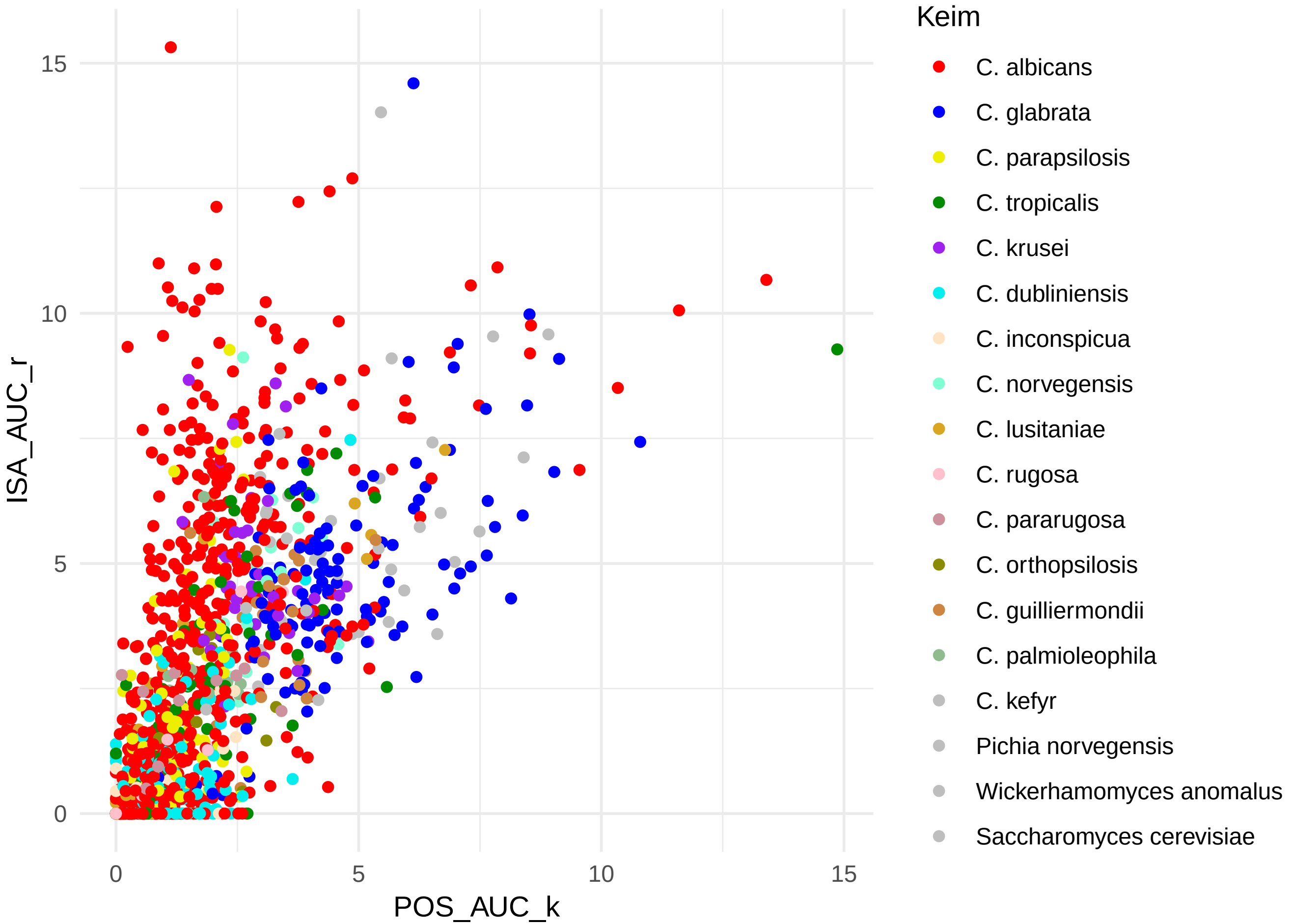
Keim
C. albicans
C. glabrata
C. parapsilosis
C. tropicalis
C. krusei
C. dubliniensis
C. inconspicua
C. norvegensis
C. lusitaniae
C. rugosa
C. pararugosa
C. orthopsilosis
C. guilliermondii
C. palmioleophila
C. kefyr
Pichia norvegensis
Wickerhamomyces anomalus
Saccharomyces cerevisiae
ISA_AUC_r
POS_AUC_k
0
5
10
15
0
5
10
15

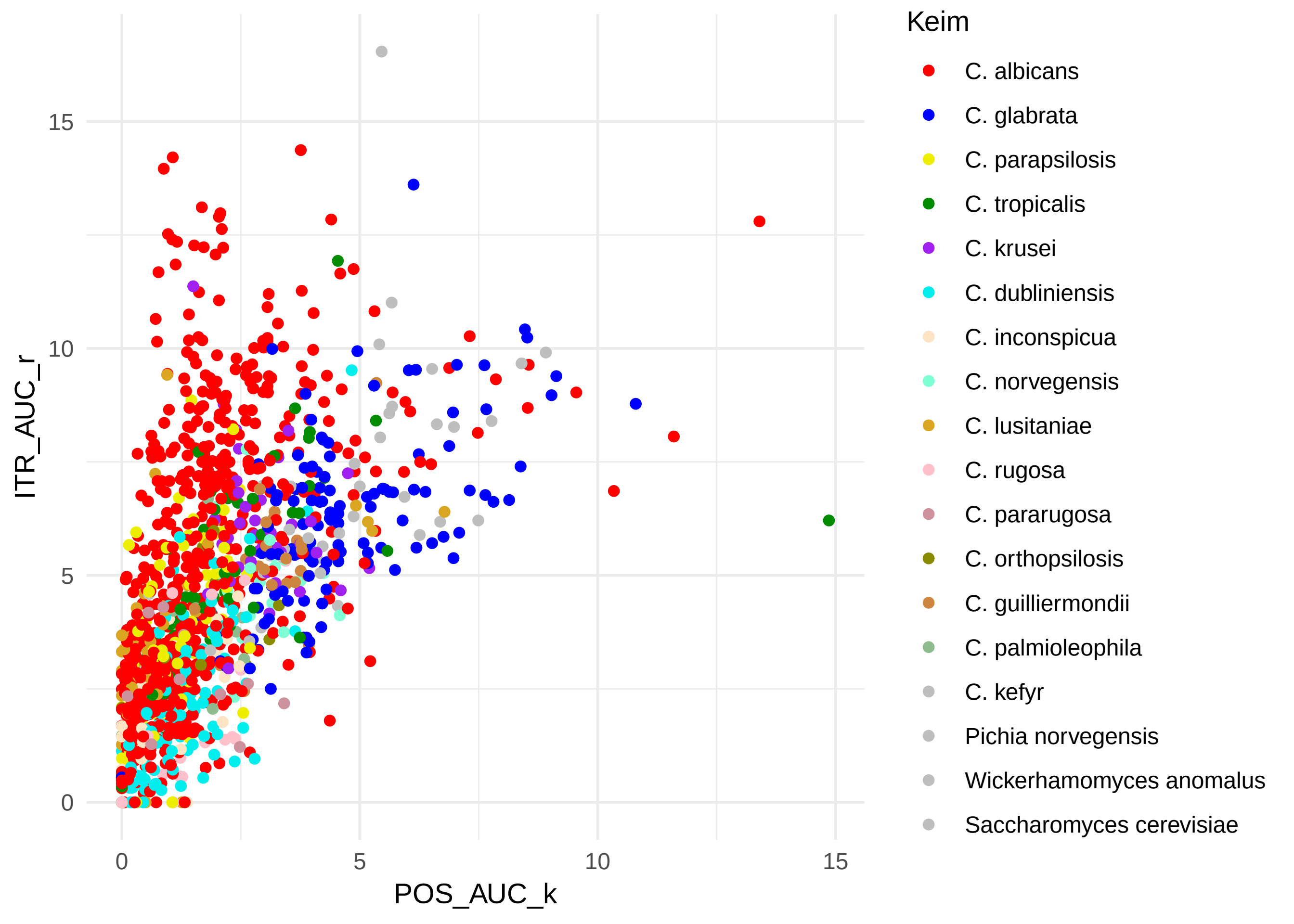
Keim
C. albicans
C. glabrata
C. parapsilosis
C. tropicalis
C. krusei
C. dubliniensis
C. inconspicua
C. norvegensis
C. lusitaniae
C. rugosa
C. pararugosa
C. orthopsilosis
C. guilliermondii
C. palmioleophila
C. kefyr
Pichia norvegensis
Wickerhamomyces anomalus
Saccharomyces cerevisiae
ITR_AUC_r
POS_AUC_k
0
5
10
15

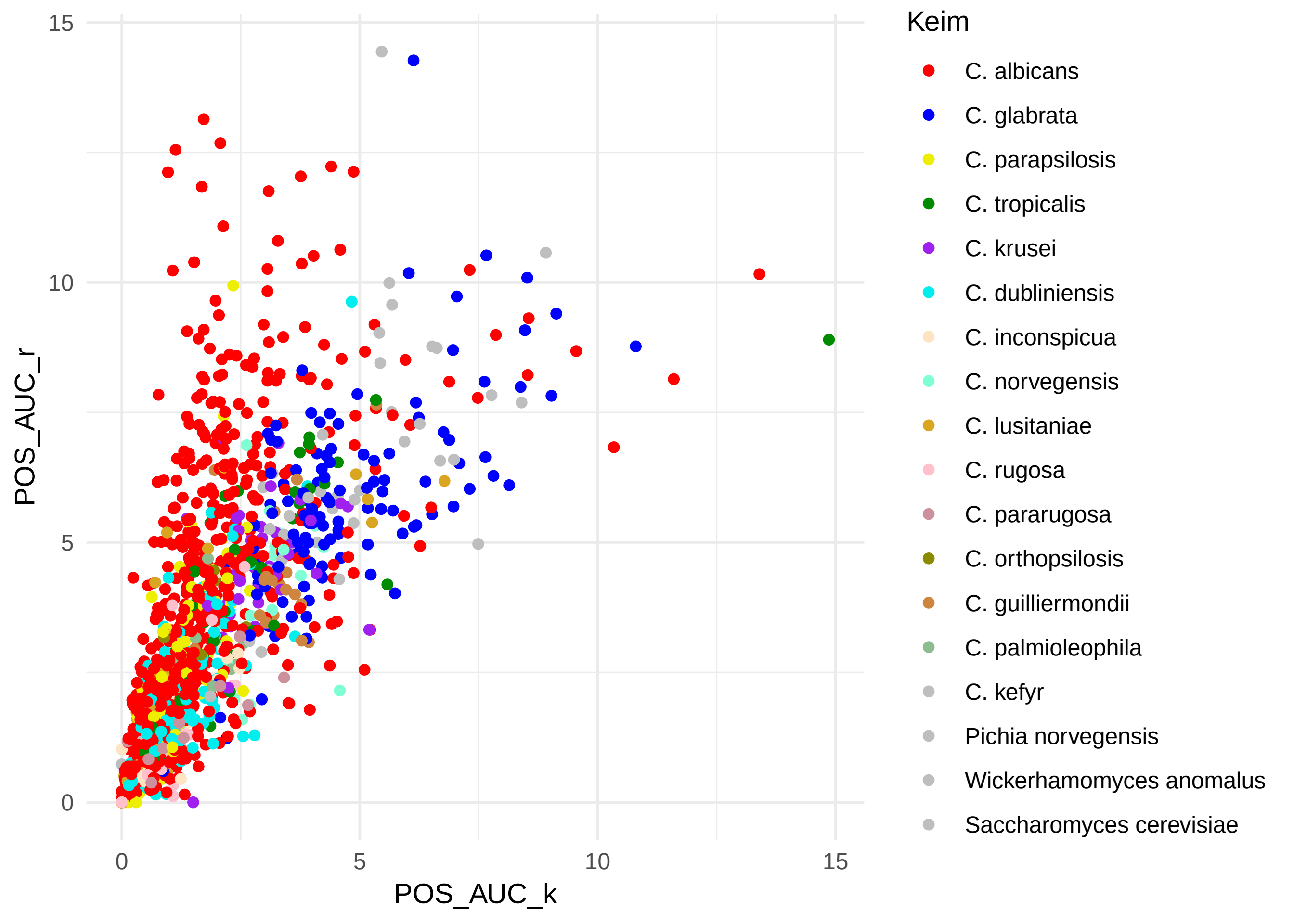

15
10
5
0
POS_AUC_r
0
5
10
15
POS_AUC_k
Keim
C. albicans
C. glabrata
C. parapsilosis
C. tropicalis
C. krusei
C. dubliniensis
C. inconspicua
C. norvegensis
C. lusitaniae
C. rugosa
C. pararugosa
C. orthopsilosis
C. guilliermondii
C. palmioleophila
C. kefyr
Pichia norvegensis
Wickerhamomyces anomalus
Saccharomyces cerevisiae

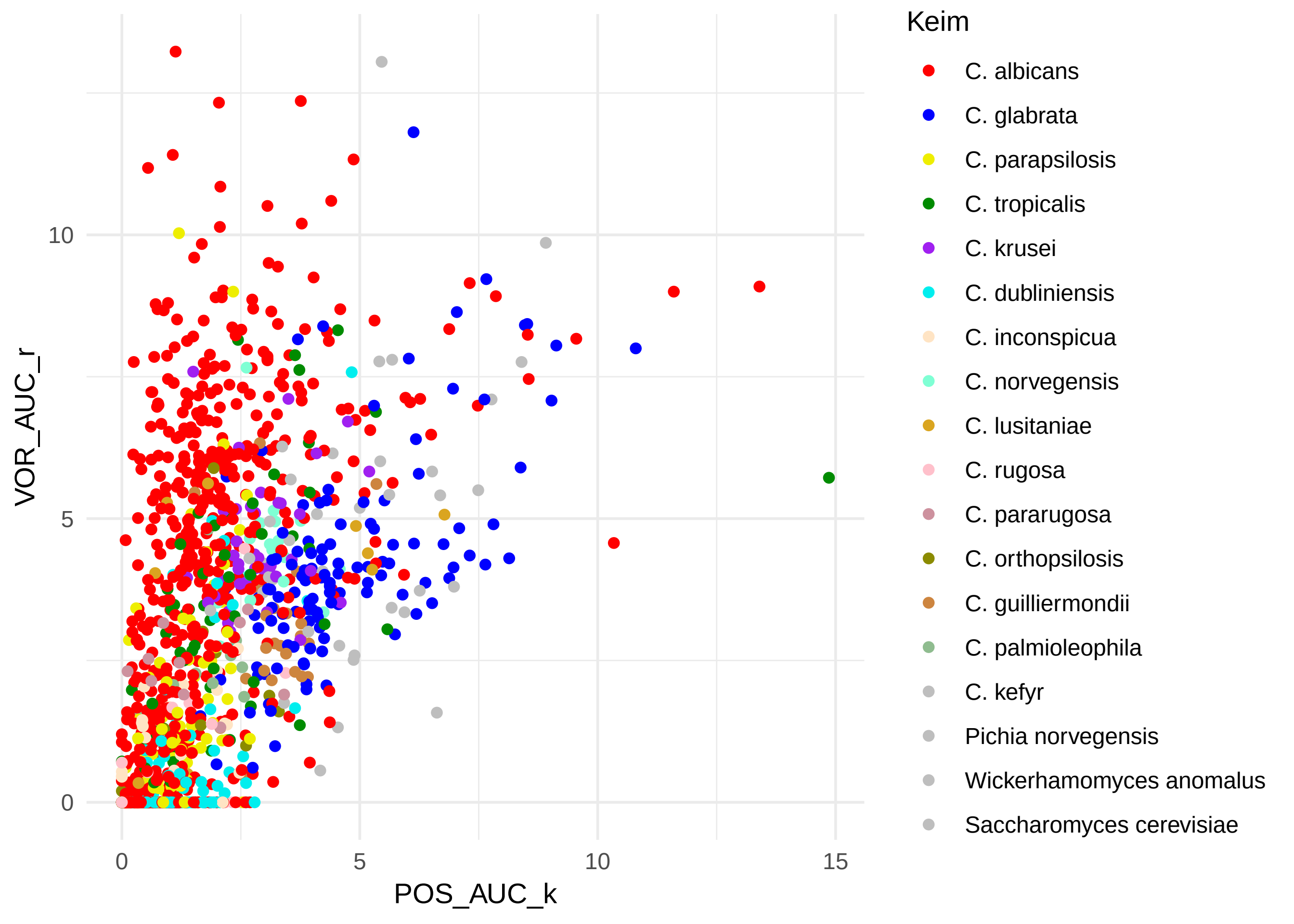
Keim
C. albicans
C. glabrata
C. parapsilosis
C. tropicalis
C. krusei
C. dubliniensis
C. inconspicua
C. norvegensis
C. lusitaniae
C. rugosa
C. pararugosa
C. orthopsilosis
C. guilliermondii
C. palmioleophila
C. kefyr
Pichia norvegensis
Wickerhamomyces anomalus
Saccharomyces cerevisiae
VOR_AUC_r
10
5
0
0
5
10
15
POS_AUC_k

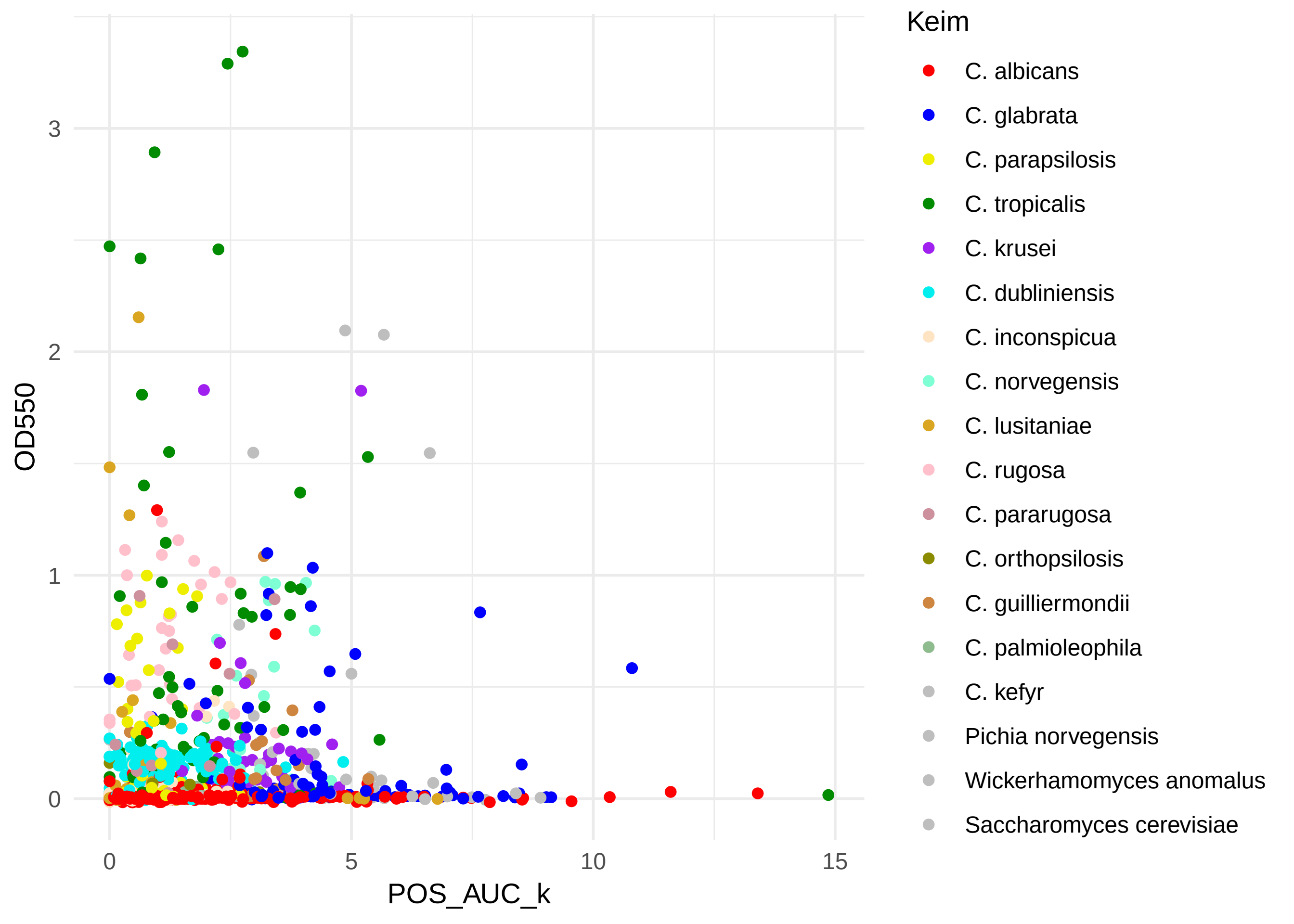
OD550
POS_AUC_k
0
1
2
3
0
5
10
15
Keim
C. albicans
C. glabrata
C. parapsilosis
C. tropicalis
C. krusei
C. dubliniensis
C. inconspicua
C. norvegensis
C. lusitaniae
C. rugosa
C. pararugosa
C. orthopsilosis
C. guilliermondii
C. palmioleophila
C. kefyr
Pichia norvegensis
Wickerhamomyces anomalus
Saccharomyces cerevisiae

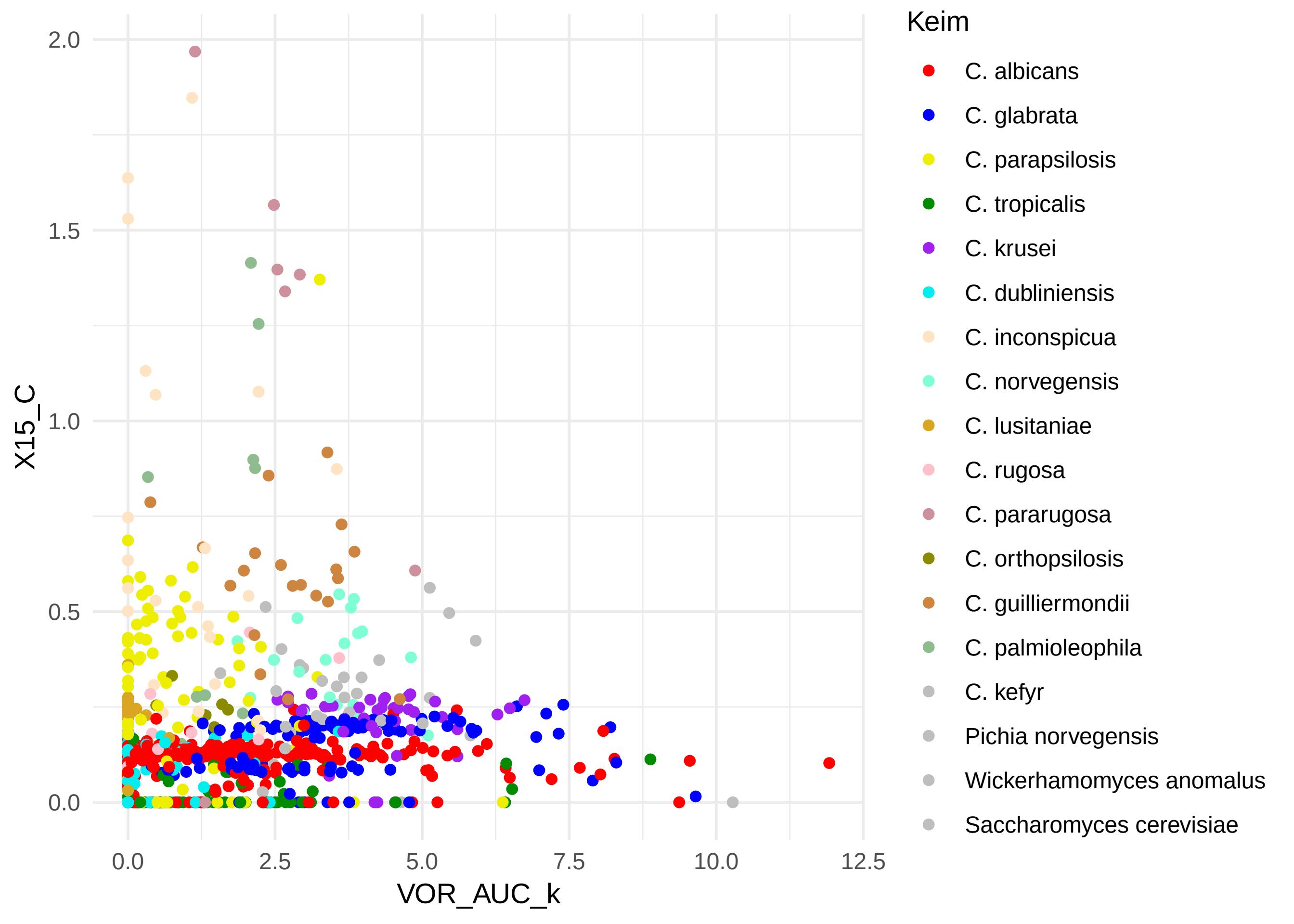

Keim
C. albicans
C. glabrata
C. parapsilosis
C. tropicalis
C. krusei
C. dubliniensis
C. inconspicua
C. norvegensis
C. lusitaniae
C. rugosa
C. pararugosa
C. orthopsilosis
C. guilliermondii
C. palmioleophila
C. kefyr
Pichia norvegensis
Wickerhamomyces anomalus
Saccharomyces cerevisiae
X15_C
2.0
1.5
1.0
0.5
0.0
0.0
2.5
5.0
7.5
10.0
12.5
VOR_AUC_k

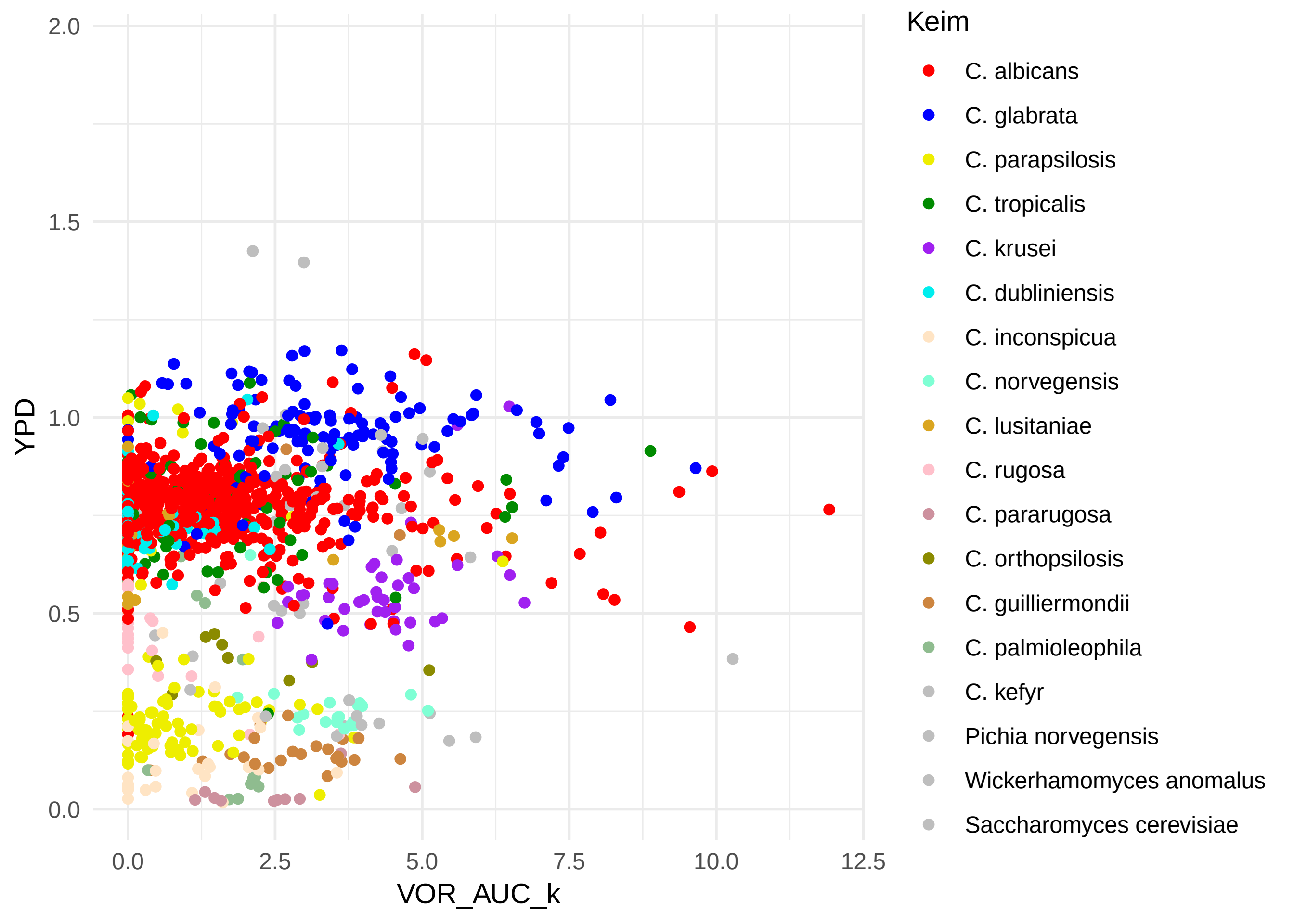

2.0
1.5
1.0
0.5
0.0
YPD
0.0
2.5
5.0
7.5
10.0
12.5
VOR_AUC_k
Keim
C. albicans
C. glabrata
C. parapsilosis
C. tropicalis
C. krusei
C. dubliniensis
C. inconspicua
C. norvegensis
C. lusitaniae
C. rugosa
C. pararugosa
C. orthopsilosis
C. guilliermondii
C. palmioleophila
C. kefyr
Pichia norvegensis
Wickerhamomyces anomalus
Saccharomyces cerevisiae

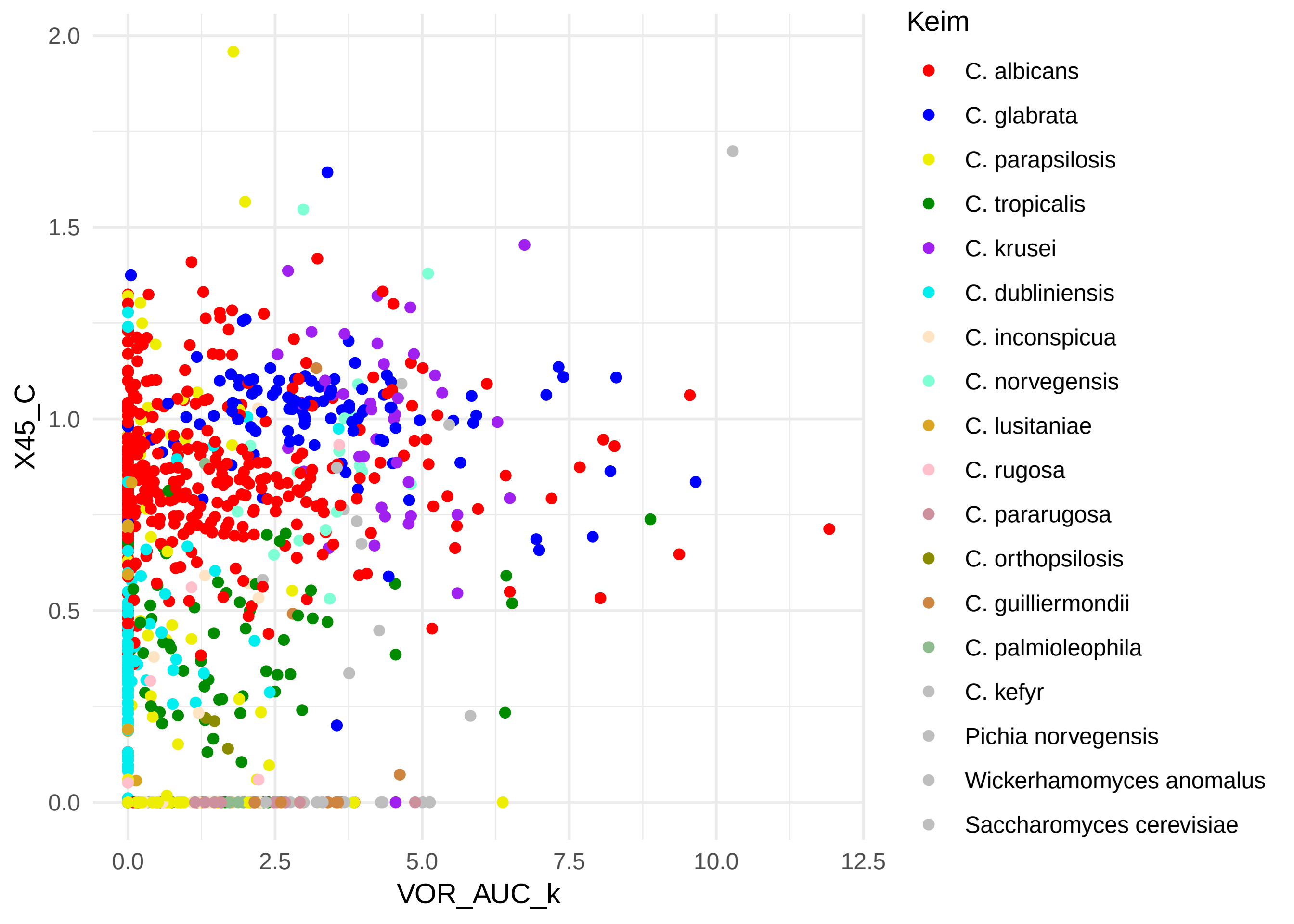

Keim
C. albicans
C. glabrata
C. parapsilosis
C. tropicalis
C. krusei
C. dubliniensis
C. inconspicua
C. norvegensis
C. lusitaniae
C. rugosa
C. pararugosa
C. orthopsilosis
C. guilliermondii
C. palmioleophila
C. kefyr
Pichia norvegensis
Wickerhamomyces anomalus
Saccharomyces cerevisiae
X45_C
2.0
1.5
1.0
0.5
0.0
0.0
2.5
5.0
7.5
10.0
12.5
VOR_AUC_k

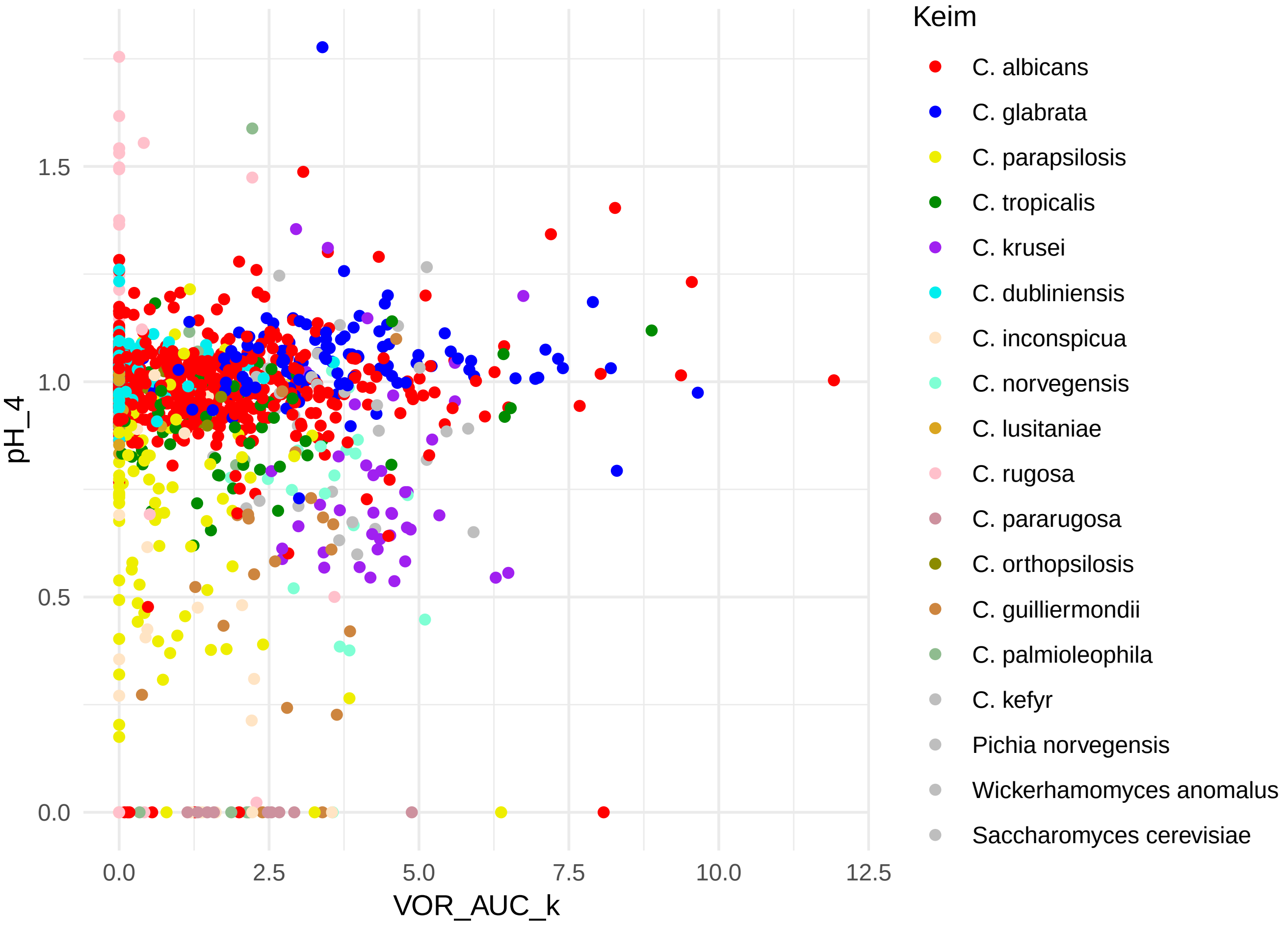

Keim
C. albicans
C. glabrata
C. parapsilosis
C. tropicalis
C. krusei
C. dubliniensis
C. inconspicua
C. norvegensis
C. lusitaniae
C. rugosa
C. pararugosa
C. orthopsilosis
C. guilliermondii
C. palmioleophila
C. kefyr
Pichia norvegensis
Wickerhamomyces anomalus
Saccharomyces cerevisiae
pH_4
1.5
1.0
0.5
0.0
0.0
2.5
5.0
7.5
10.0
12.5
VOR_AUC_k

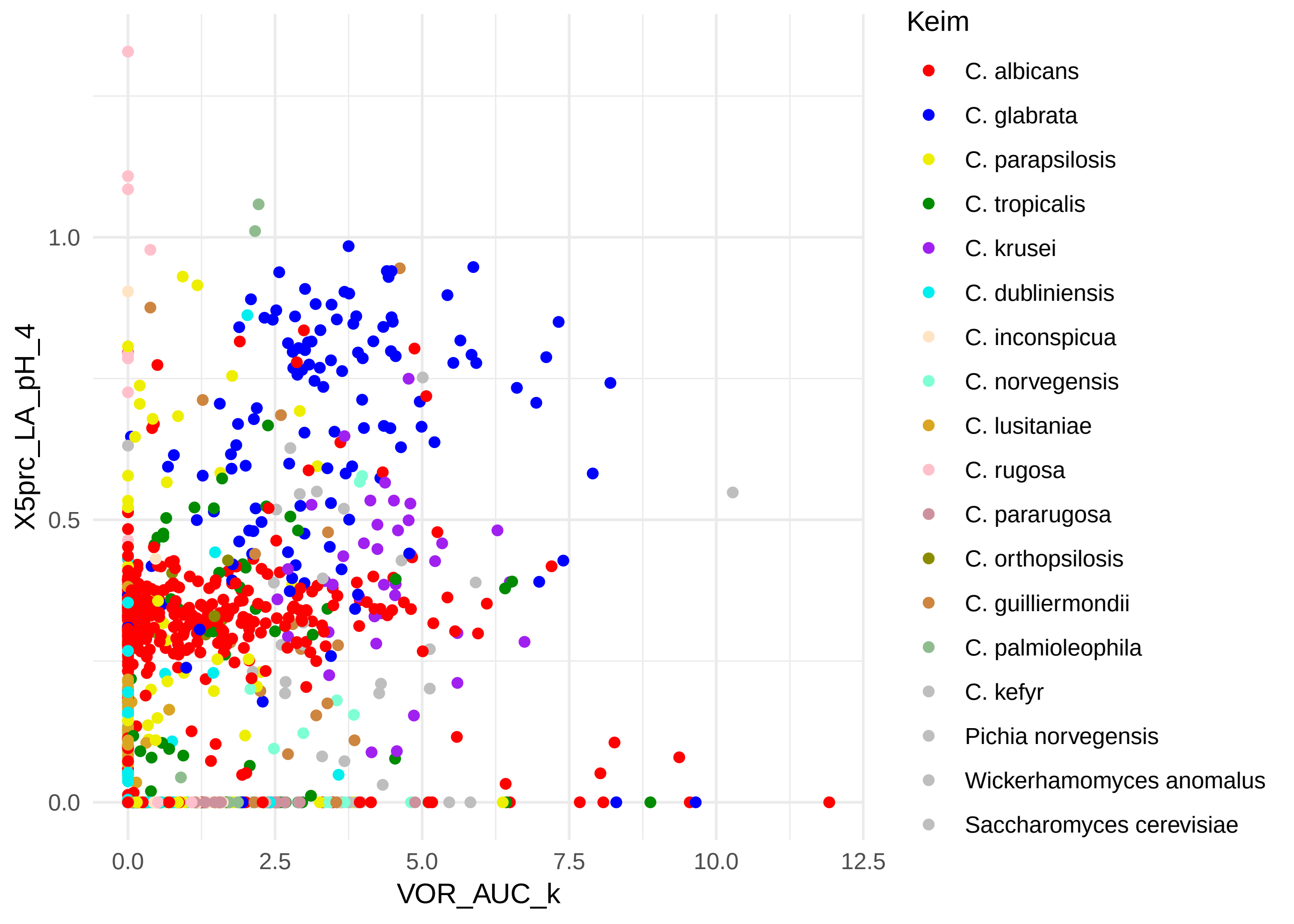
X5prc_LA_pH_4
0.0
0.5
1.0
VOR_AUC_k
0.0
2.5
5.0
7.5
10.0
12.5
Keim
C. albicans
C. glabrata
C. parapsilosis
C. tropicalis
C. krusei
C. dubliniensis
C. inconspicua
C. norvegensis
C. lusitaniae
C. rugosa
C. pararugosa
C. orthopsilosis
C. guilliermondii
C. palmioleophila
C. kefyr
Pichia norvegensis
Wickerhamomyces anomalus
Saccharomyces cerevisiae

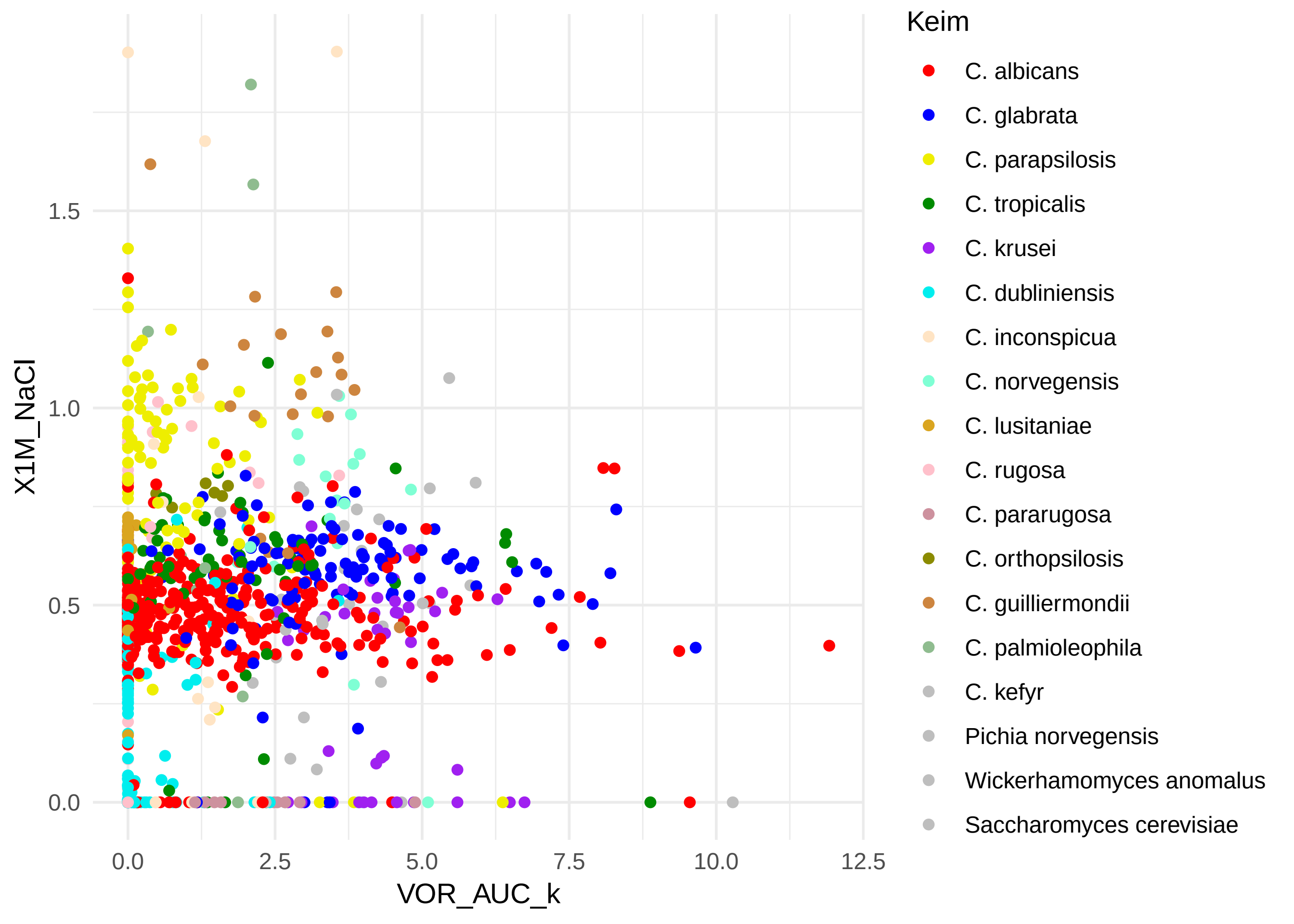
Keim
C. albicans
C. glabrata
C. parapsilosis
C. tropicalis
C. krusei
C. dubliniensis
C. inconspicua
C. norvegensis
C. lusitaniae
C. rugosa
C. pararugosa
C. orthopsilosis
C. guilliermondii
C. palmioleophila
C. kefyr
Pichia norvegensis
Wickerhamomyces anomalus
Saccharomyces cerevisiae
X1M_NaCl
1.5
1.0
0.5
0.0
0.0
2.5
5.0
7.5
10.0
12.5
VOR_AUC_k

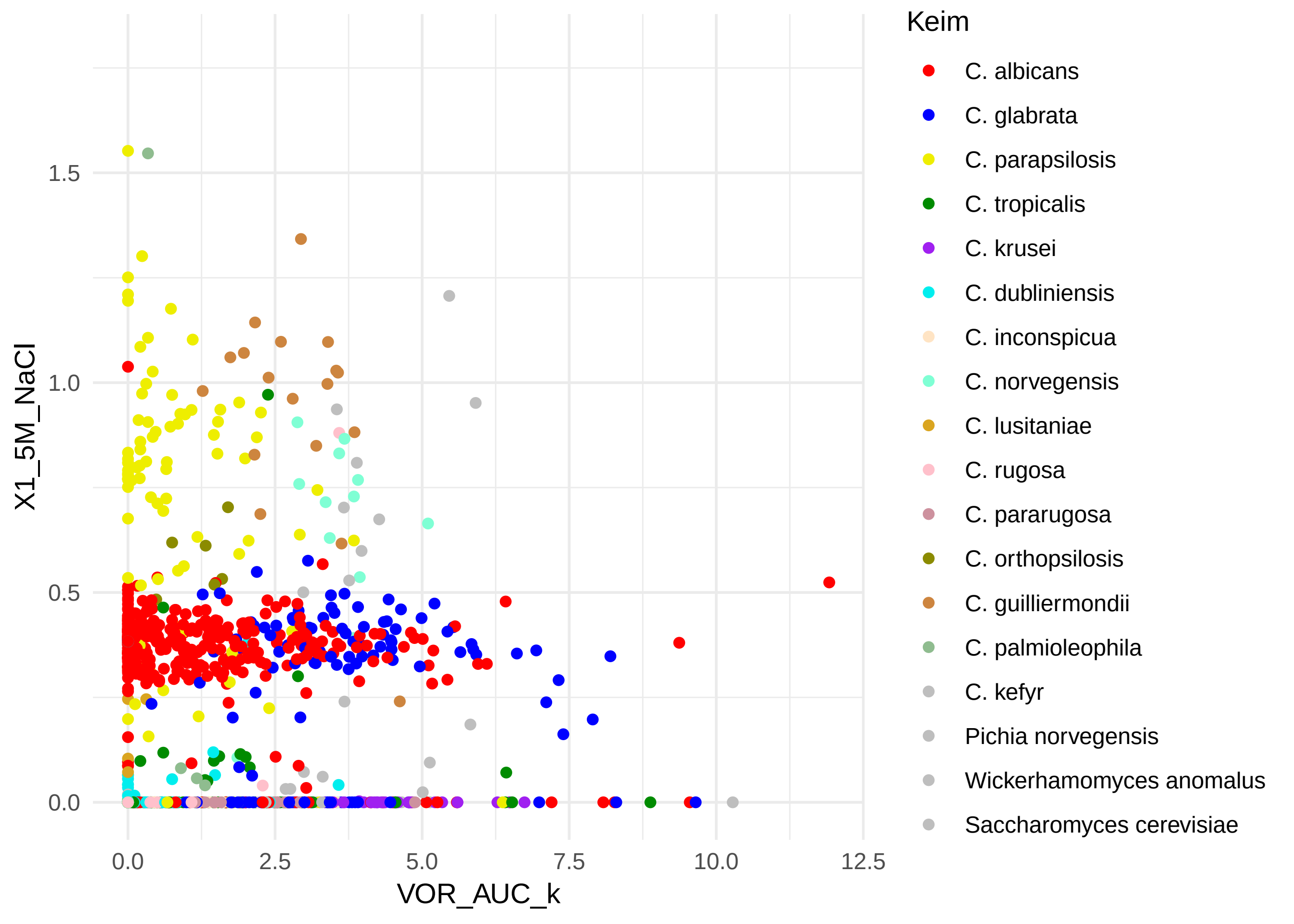

X1_5M_NaCl
VOR_AUC_k
0.0
0.5
1.0
1.5
0.0
2.5
5.0
7.5
10.0
12.5
Keim
C. albicans
C. glabrata
C. parapsilosis
C. tropicalis
C. krusei
C. dubliniensis
C. inconspicua
C. norvegensis
C. lusitaniae
C. rugosa
C. pararugosa
C. orthopsilosis
C. guilliermondii
C. palmioleophila
C. kefyr
Pichia norvegensis
Wickerhamomyces anomalus
Saccharomyces cerevisiae

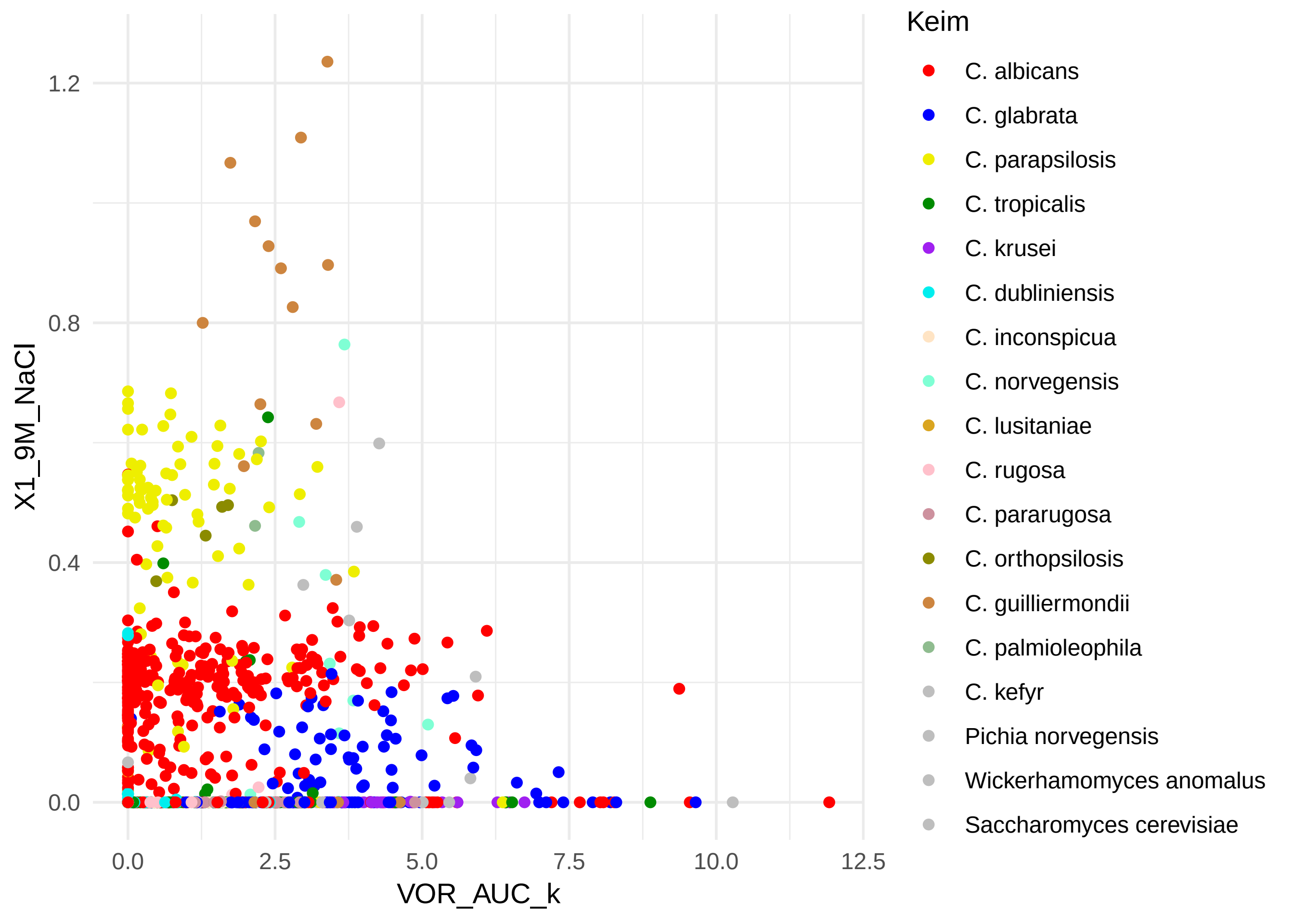

Keim
C. albicans
C. glabrata
C. parapsilosis
C. tropicalis
C. krusei
C. dubliniensis
C. inconspicua
C. norvegensis
C. lusitaniae
C. rugosa
C. pararugosa
C. orthopsilosis
C. guilliermondii
C. palmioleophila
C. kefyr
Pichia norvegensis
Wickerhamomyces anomalus
Saccharomyces cerevisiae
X1_9M_NaCl
1.2
0.8
0.4
0.0
0.0
2.5
5.0
7.5
10.0
12.5
VOR_AUC_k

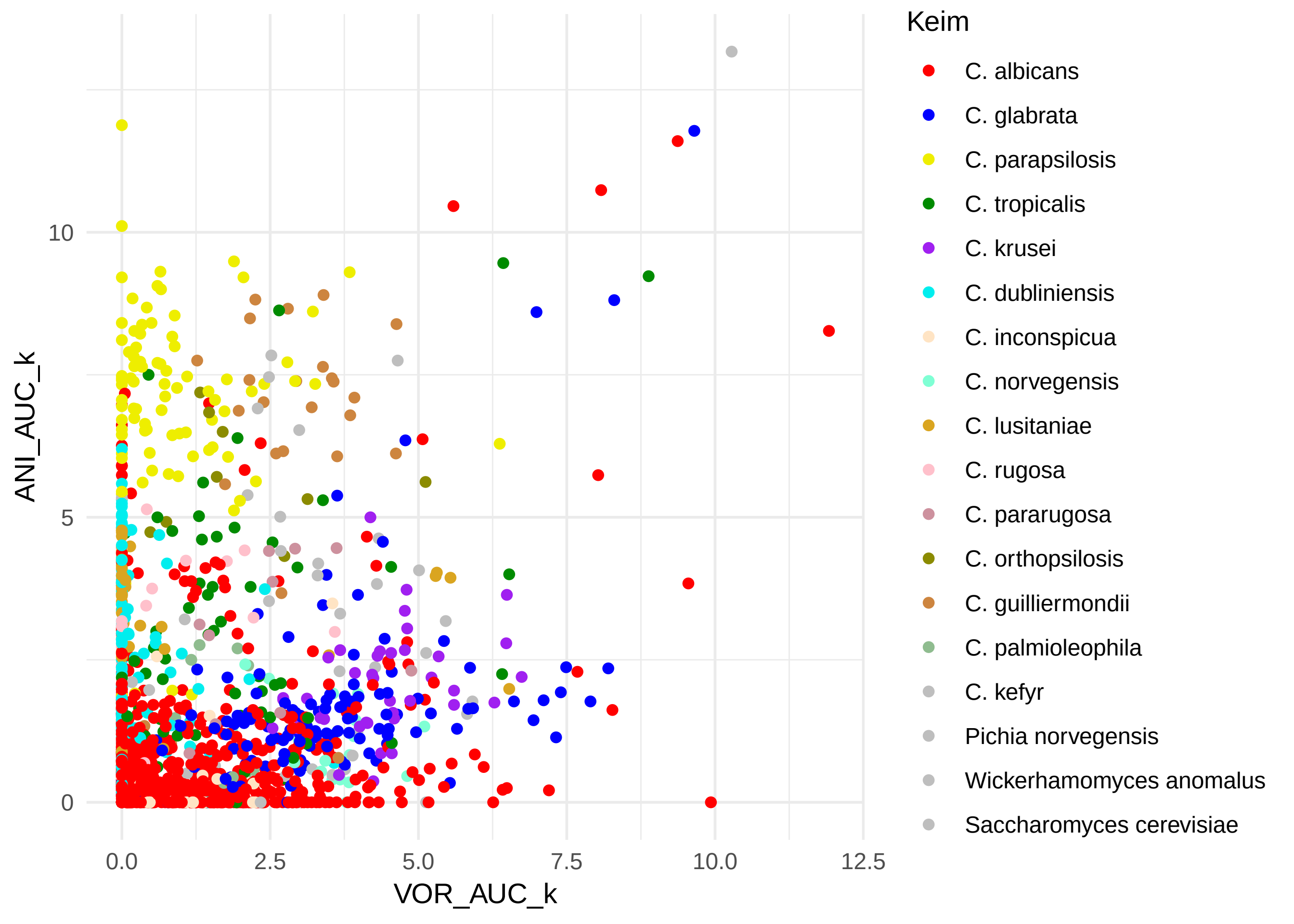

Keim
C. albicans
C. glabrata
C. parapsilosis
C. tropicalis
C. krusei
C. dubliniensis
C. inconspicua
C. norvegensis
C. lusitaniae
C. rugosa
C. pararugosa
C. orthopsilosis
C. guilliermondii
C. palmioleophila
C. kefyr
Pichia norvegensis
Wickerhamomyces anomalus
Saccharomyces cerevisiae
ANI_AUC_k
10
5
0
VOR_AUC_k
0.0
2.5
5.0
7.5
10.0
12.5

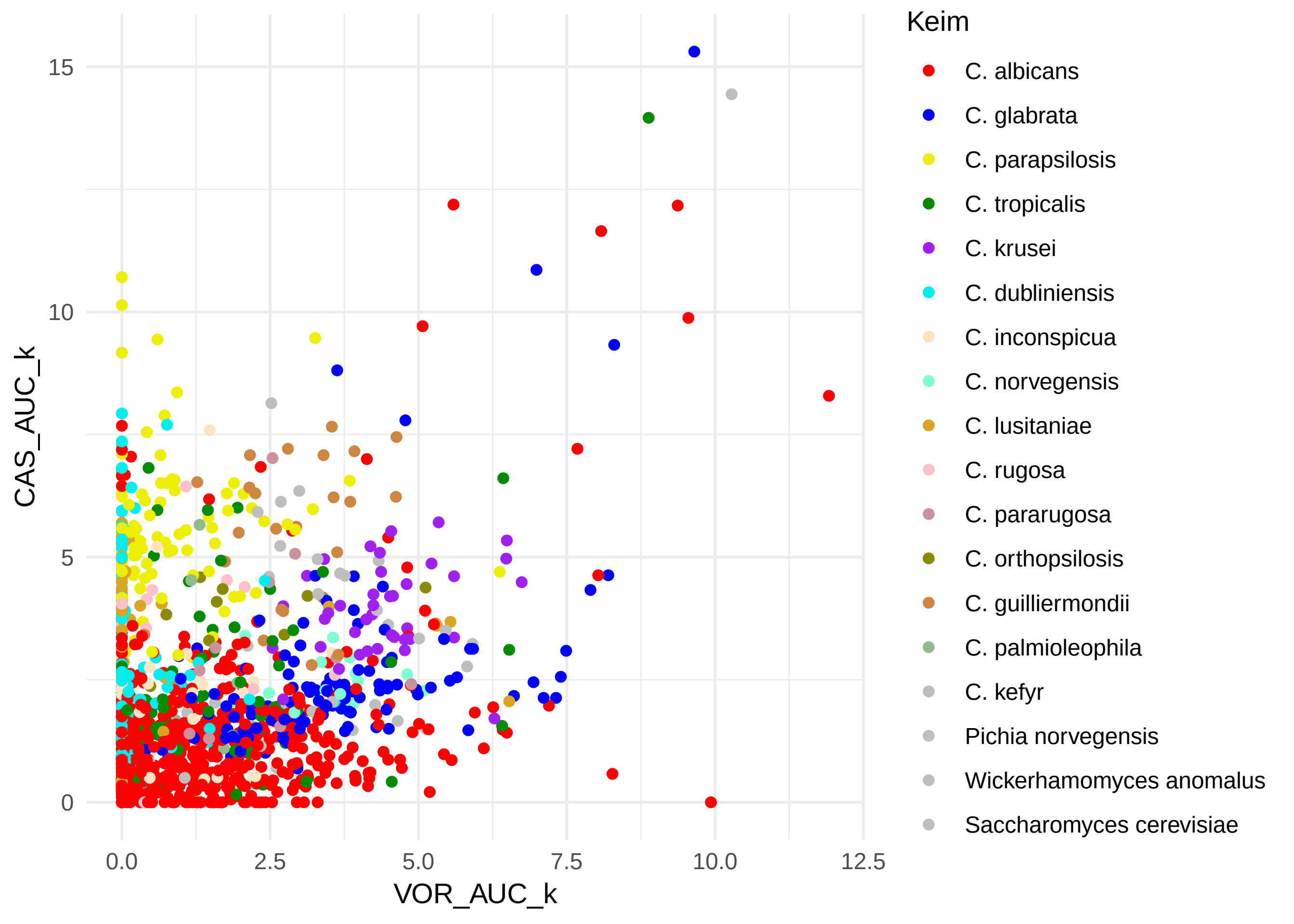

Keim
C. albicans
C. glabrata
C. parapsilosis
C. tropicalis
C. krusei
C. dubliniensis
C. inconspicua
C. norvegensis
C. lusitaniae
C. rugosa
C. pararugosa
C. orthopsilosis
C. guilliermondii
C. palmioleophila
C. kefyr
Pichia norvegensis
Wickerhamomyces anomalus
Saccharomyces cerevisiae
CAS_AUC_k
VOR_AUC_k
0
5
10
15
0.0
2.5
5.0
7.5
10.0
12.5

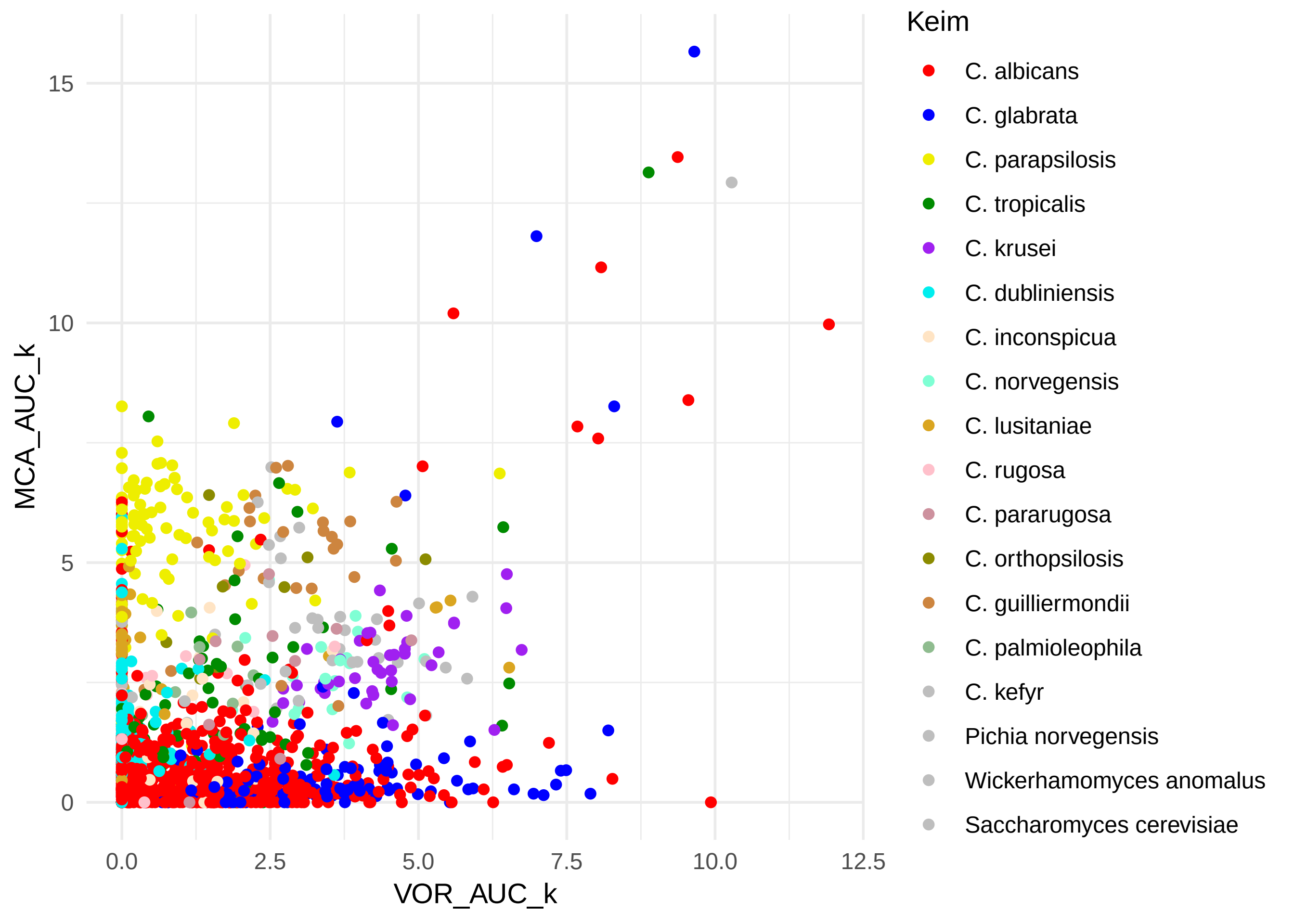

Keim
C. albicans
C. glabrata
C. parapsilosis
C. tropicalis
C. krusei
C. dubliniensis
C. inconspicua
C. norvegensis
C. lusitaniae
C. rugosa
C. pararugosa
C. orthopsilosis
C. guilliermondii
C. palmioleophila
C. kefyr
Pichia norvegensis
Wickerhamomyces anomalus
Saccharomyces cerevisiae
MCA_AUC_k
0
5
10
15
VOR_AUC_k
0.0
2.5
5.0
7.5
10.0
12.5

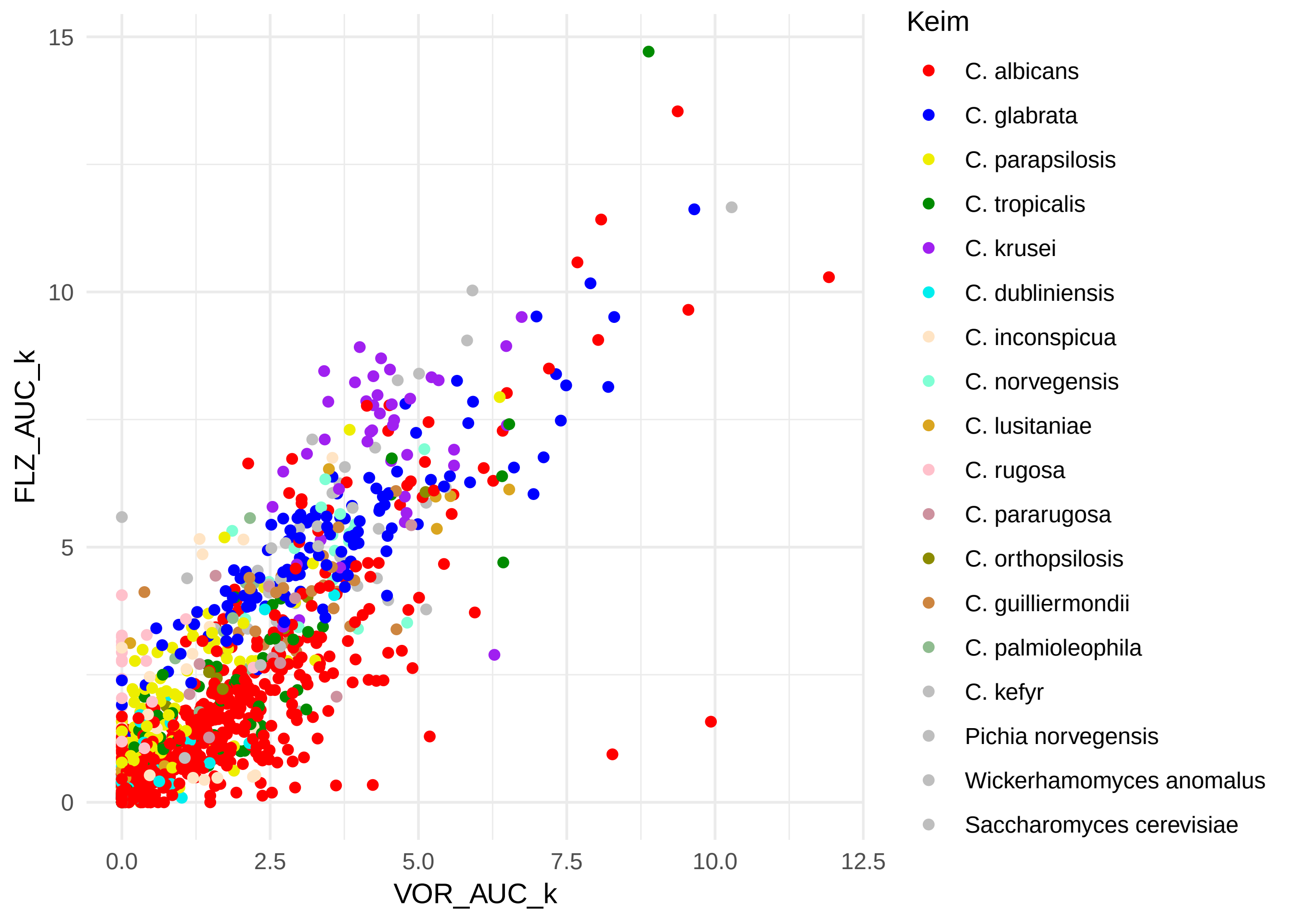

FLZ_AUC_k
VOR_AUC_k
0
5
10
15
0.0
2.5
5.0
7.5
10.0
12.5
Keim
C. albicans
C. glabrata
C. parapsilosis
C. tropicalis
C. krusei
C. dubliniensis
C. inconspicua
C. norvegensis
C. lusitaniae
C. rugosa
C. pararugosa
C. orthopsilosis
C. guilliermondii
C. palmioleophila
C. kefyr
Pichia norvegensis
Wickerhamomyces anomalus
Saccharomyces cerevisiae

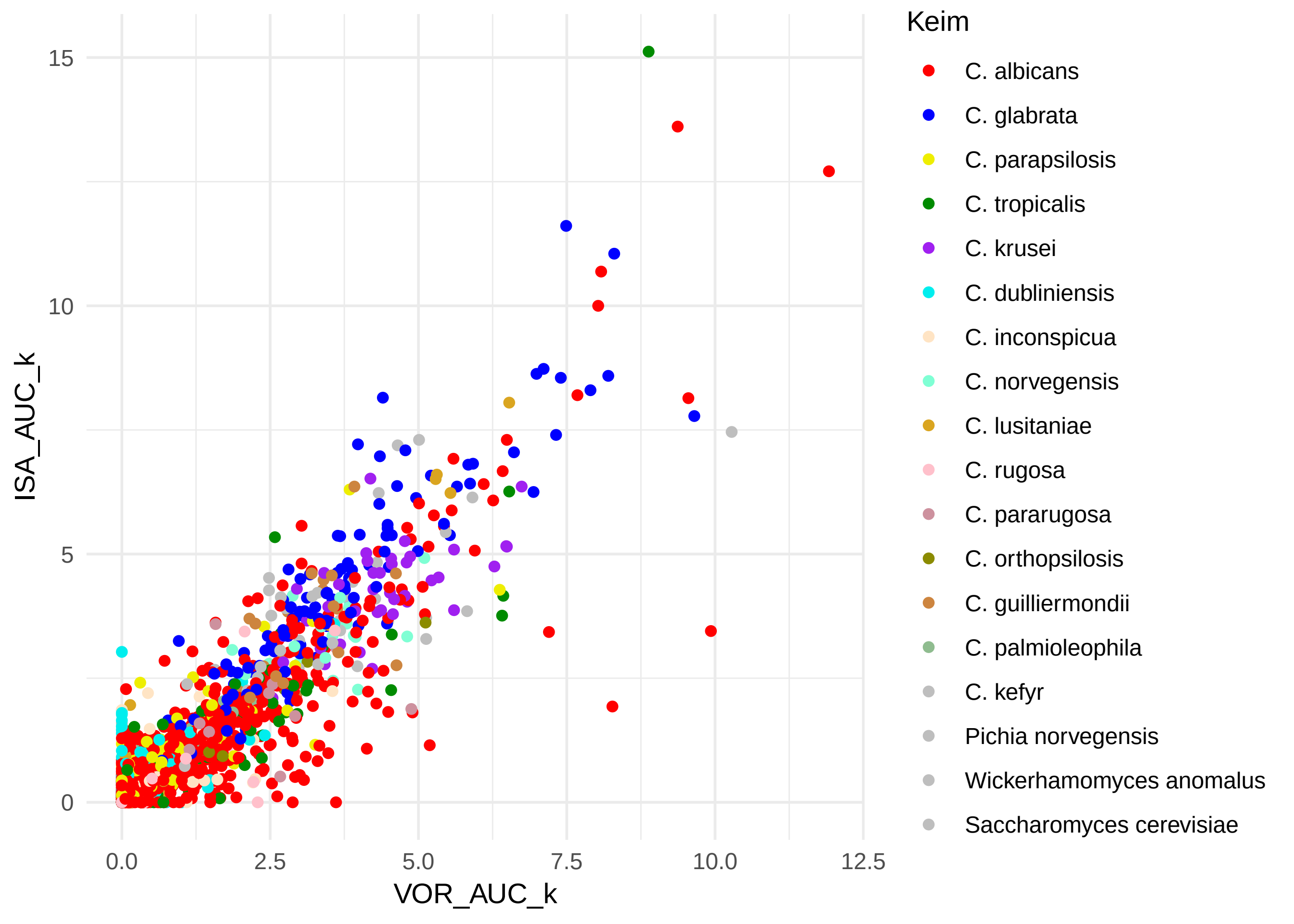
Keim
C. albicans
C. glabrata
C. parapsilosis
C. tropicalis
C. krusei
C. dubliniensis
C. inconspicua
C. norvegensis
C. lusitaniae
C. rugosa
C. pararugosa
C. orthopsilosis
C. guilliermondii
C. palmioleophila
C. kefyr
Pichia norvegensis
Wickerhamomyces anomalus
Saccharomyces cerevisiae
ISA_AUC_k
VOR_AUC_k
0
5
10
15
0.0
2.5
5.0
7.5
10.0
12.5

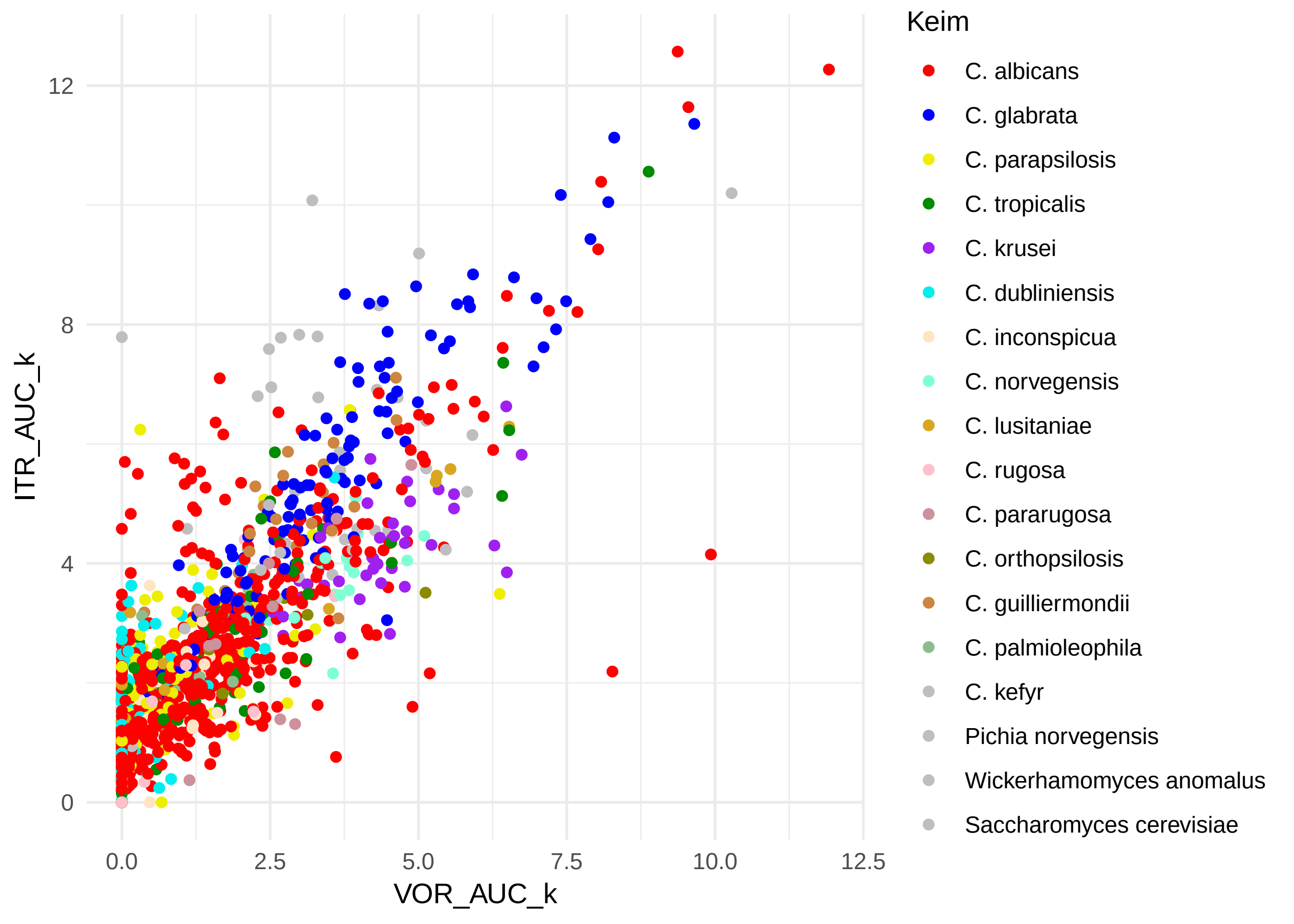

Keim
C. albicans
C. glabrata
C. parapsilosis
C. tropicalis
C. krusei
C. dubliniensis
C. inconspicua
C. norvegensis
C. lusitaniae
C. rugosa
C. pararugosa
C. orthopsilosis
C. guilliermondii
C. palmioleophila
C. kefyr
Pichia norvegensis
Wickerhamomyces anomalus
Saccharomyces cerevisiae
ITR_AUC_k
0
4
8
12
VOR_AUC_k
0.0
2.5
5.0
7.5
10.0
12.5

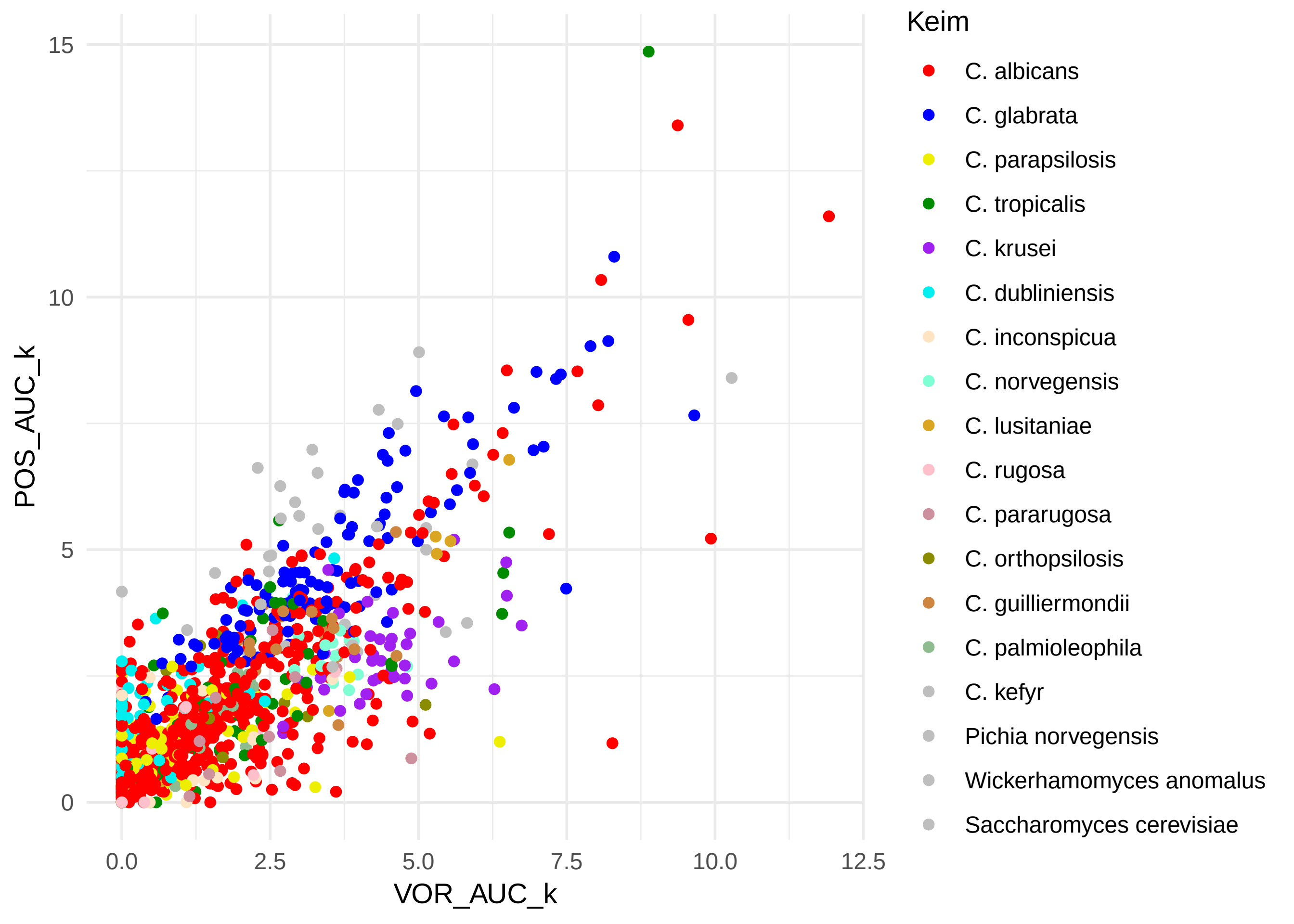
Keim
C. albicans
C. glabrata
C. parapsilosis
C. tropicalis
C. krusei
C. dubliniensis
C. inconspicua
C. norvegensis
C. lusitaniae
C. rugosa
C. pararugosa
C. orthopsilosis
C. guilliermondii
C. palmioleophila
C. kefyr
Pichia norvegensis
Wickerhamomyces anomalus
Saccharomyces cerevisiae
POS_AUC_k
VOR_AUC_k
0
5
10
15
0.0
2.5
5.0
7.5
10.0
12.5

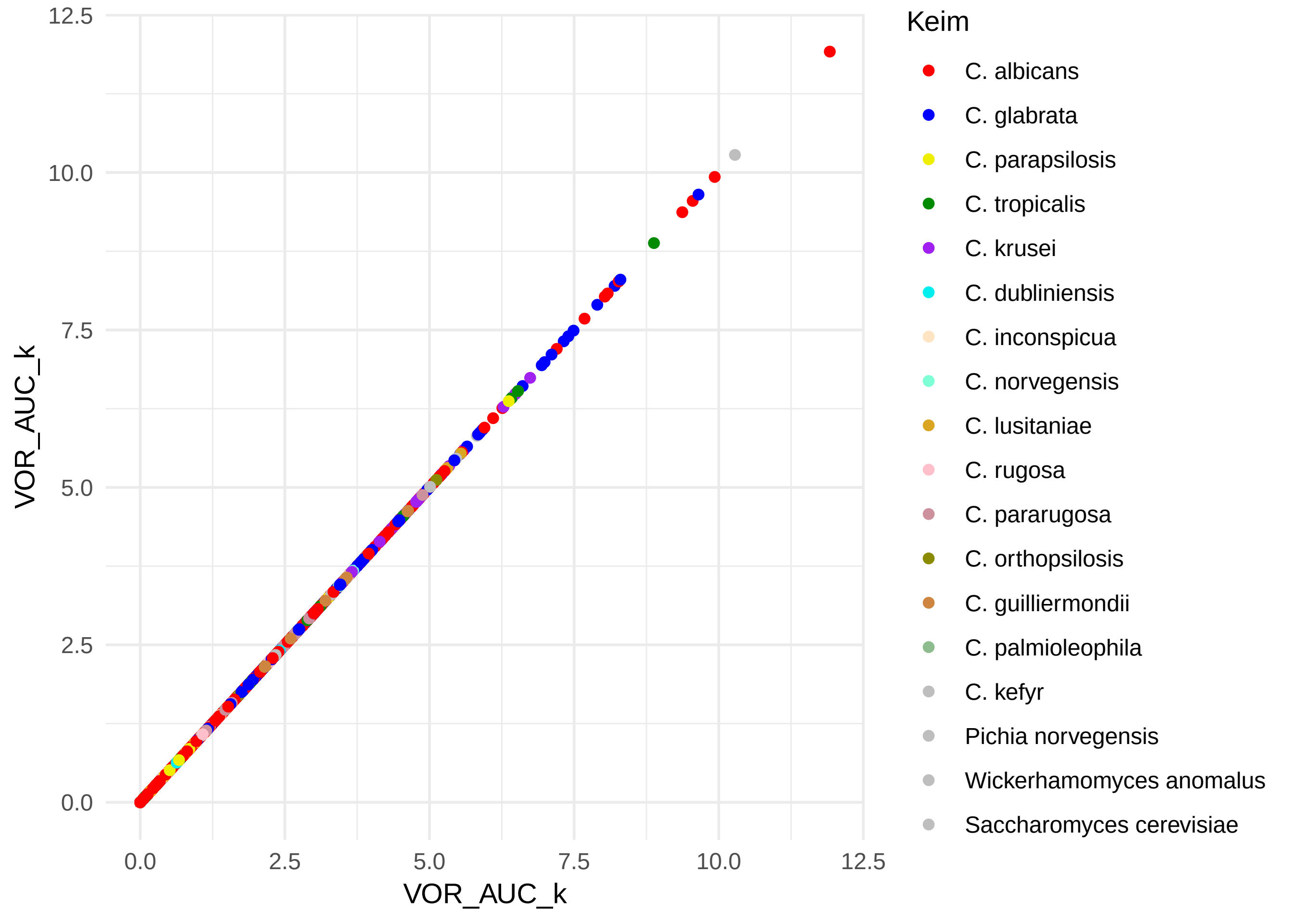
12.5
10.0
7.5
5.0
2.5
0.0
VOR_AUC_k
0.0
2.5
5.0
7.5
10.0
12.5
VOR_AUC_k
Keim
C. albicans
C. glabrata
C. parapsilosis
C. tropicalis
C. krusei
C. dubliniensis
C. inconspicua
C. norvegensis
C. lusitaniae
C. rugosa
C. pararugosa
C. orthopsilosis
C. guilliermondii
C. palmioleophila
C. kefyr
Pichia norvegensis
Wickerhamomyces anomalus
Saccharomyces cerevisiae

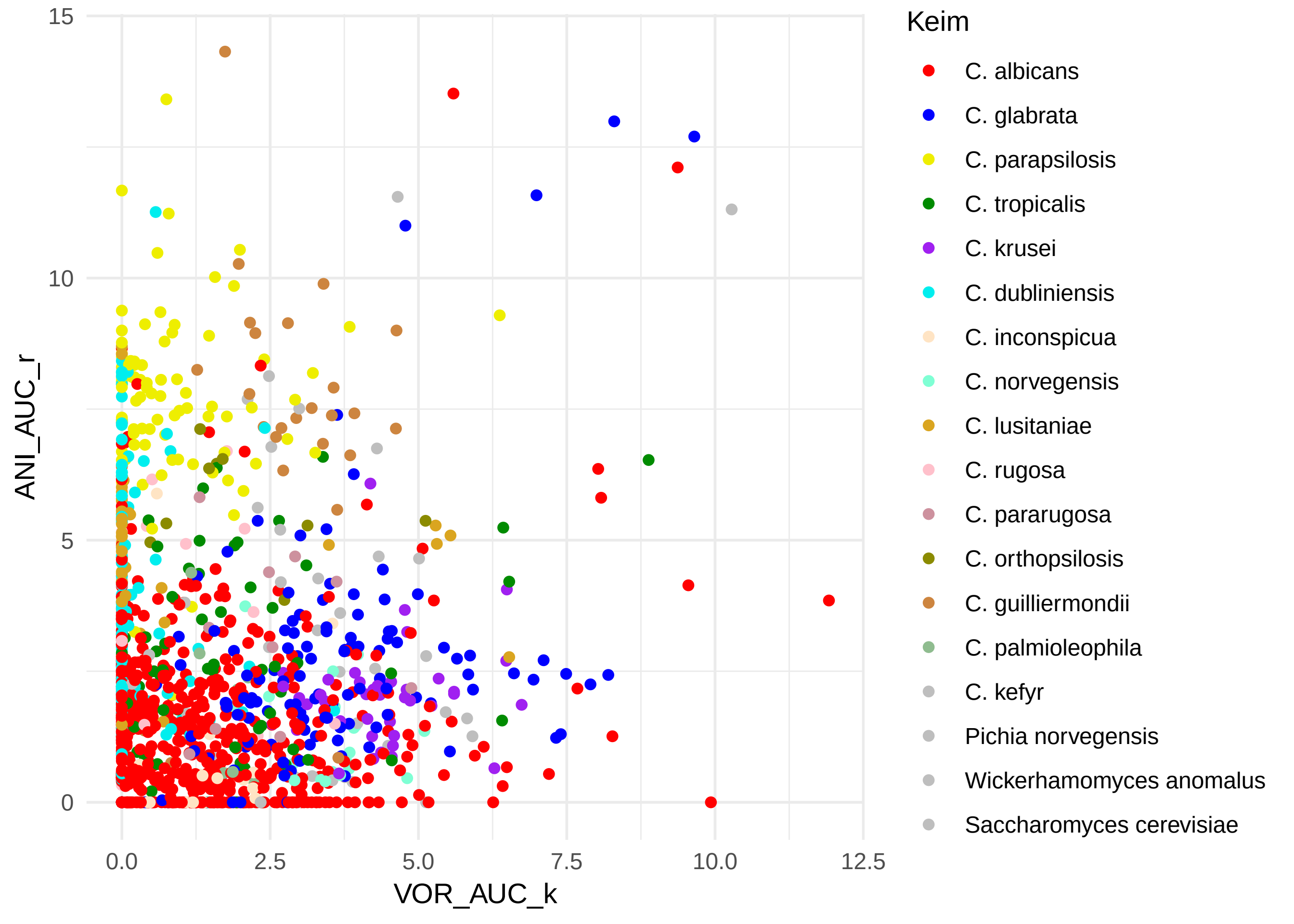
15
10
5
0
ANI_AUC_r
0.0
2.5
5.0
7.5
10.0
12.5
VOR_AUC_k
Keim
C. albicans
C. glabrata
C. parapsilosis
C. tropicalis
C. krusei
C. dubliniensis
C. inconspicua
C. norvegensis
C. lusitaniae
C. rugosa
C. pararugosa
C. orthopsilosis
C. guilliermondii
C. palmioleophila
C. kefyr
Pichia norvegensis
Wickerhamomyces anomalus
Saccharomyces cerevisiae

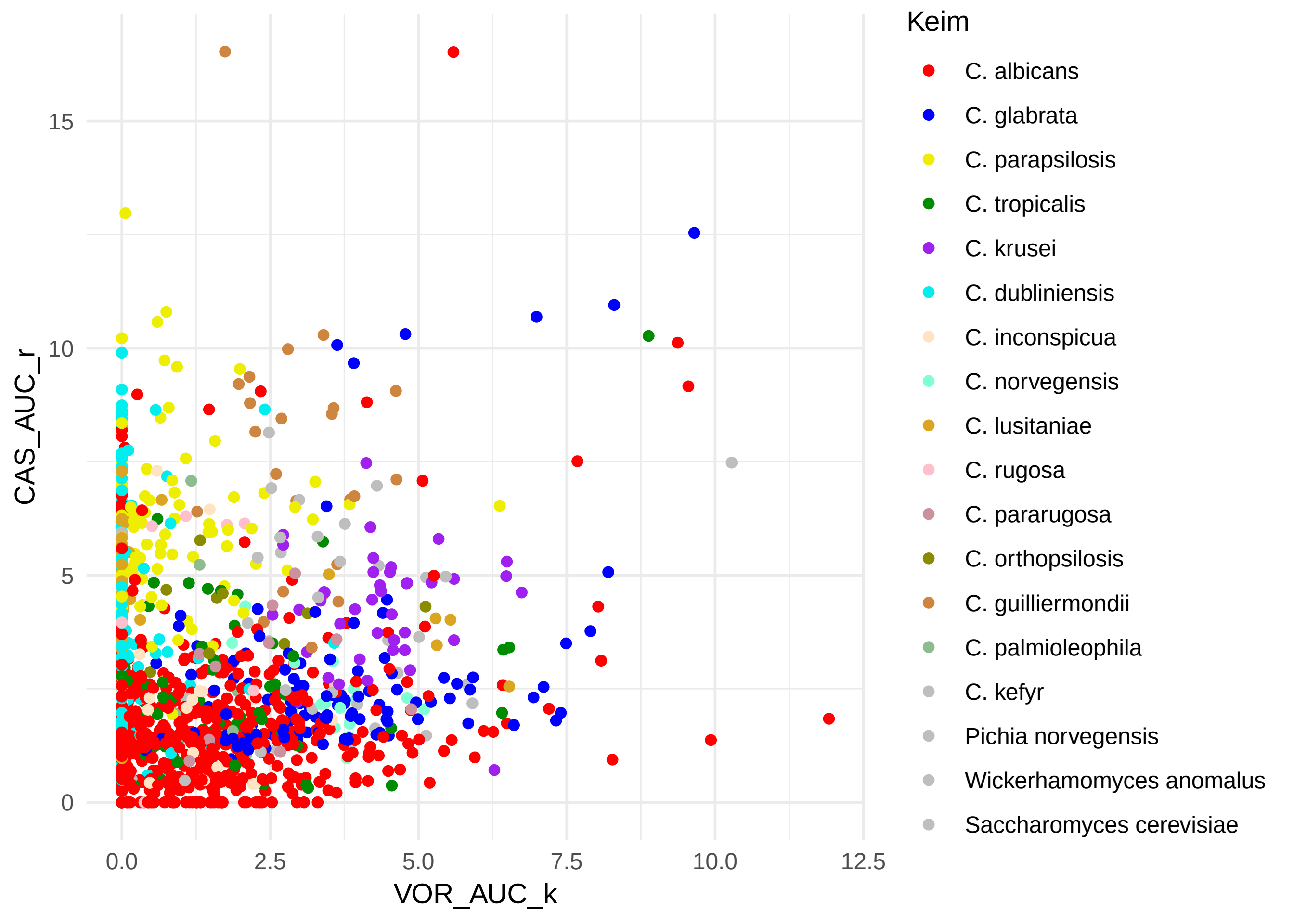
Keim
C. albicans
C. glabrata
C. parapsilosis
C. tropicalis
C. krusei
C. dubliniensis
C. inconspicua
C. norvegensis
C. lusitaniae
C. rugosa
C. pararugosa
C. orthopsilosis
C. guilliermondii
C. palmioleophila
C. kefyr
Pichia norvegensis
Wickerhamomyces anomalus
Saccharomyces cerevisiae
CAS_AUC_r
VOR_AUC_k
0
5
10
15
0.0
2.5
5.0
7.5
10.0
12.5

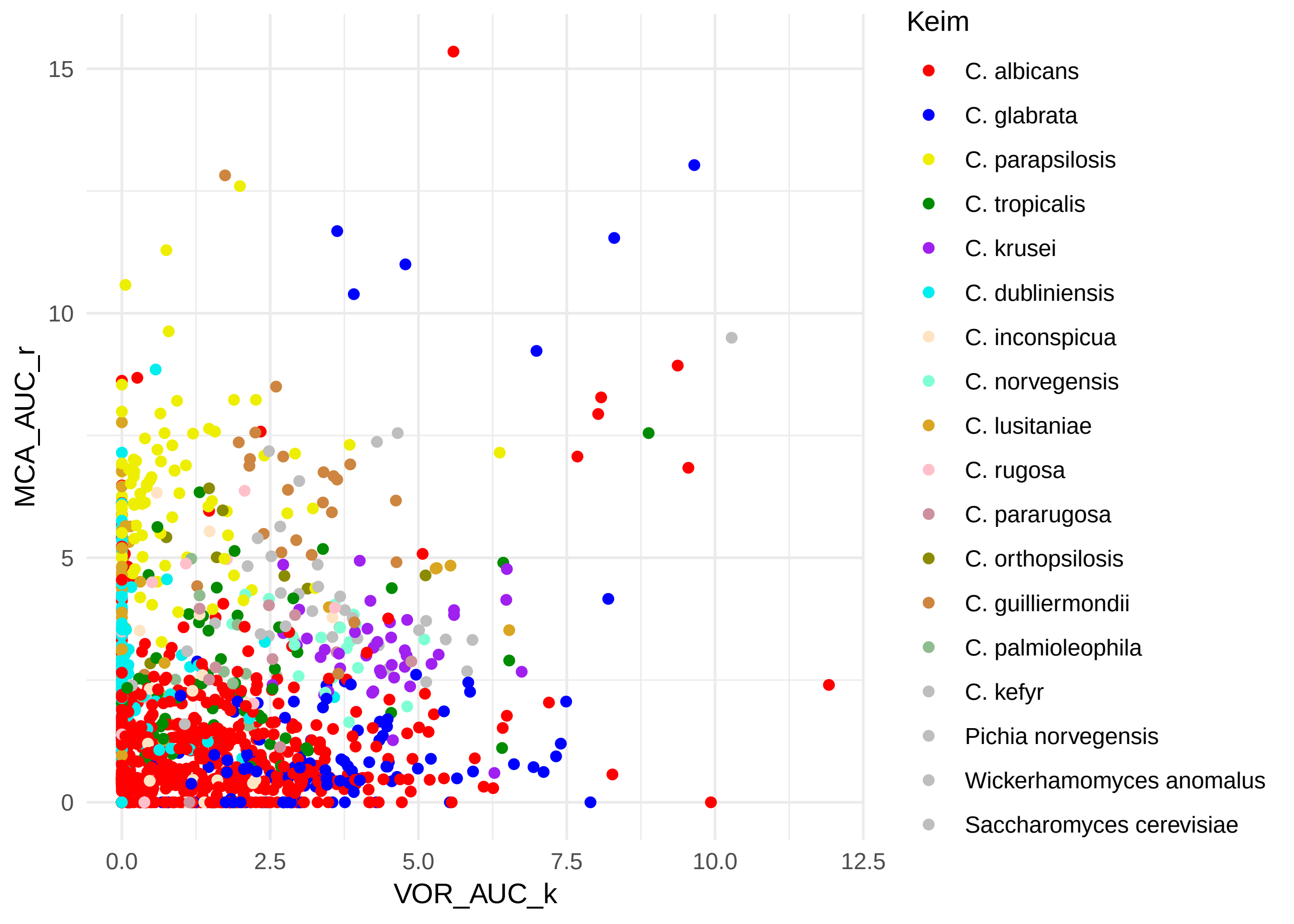
MCA_AUC_r
VOR_AUC_k
0
5
10
15
0.0
2.5
5.0
7.5
10.0
12.5
Keim
C. albicans
C. glabrata
C. parapsilosis
C. tropicalis
C. krusei
C. dubliniensis
C. inconspicua
C. norvegensis
C. lusitaniae
C. rugosa
C. pararugosa
C. orthopsilosis
C. guilliermondii
C. palmioleophila
C. kefyr
Pichia norvegensis
Wickerhamomyces anomalus
Saccharomyces cerevisiae

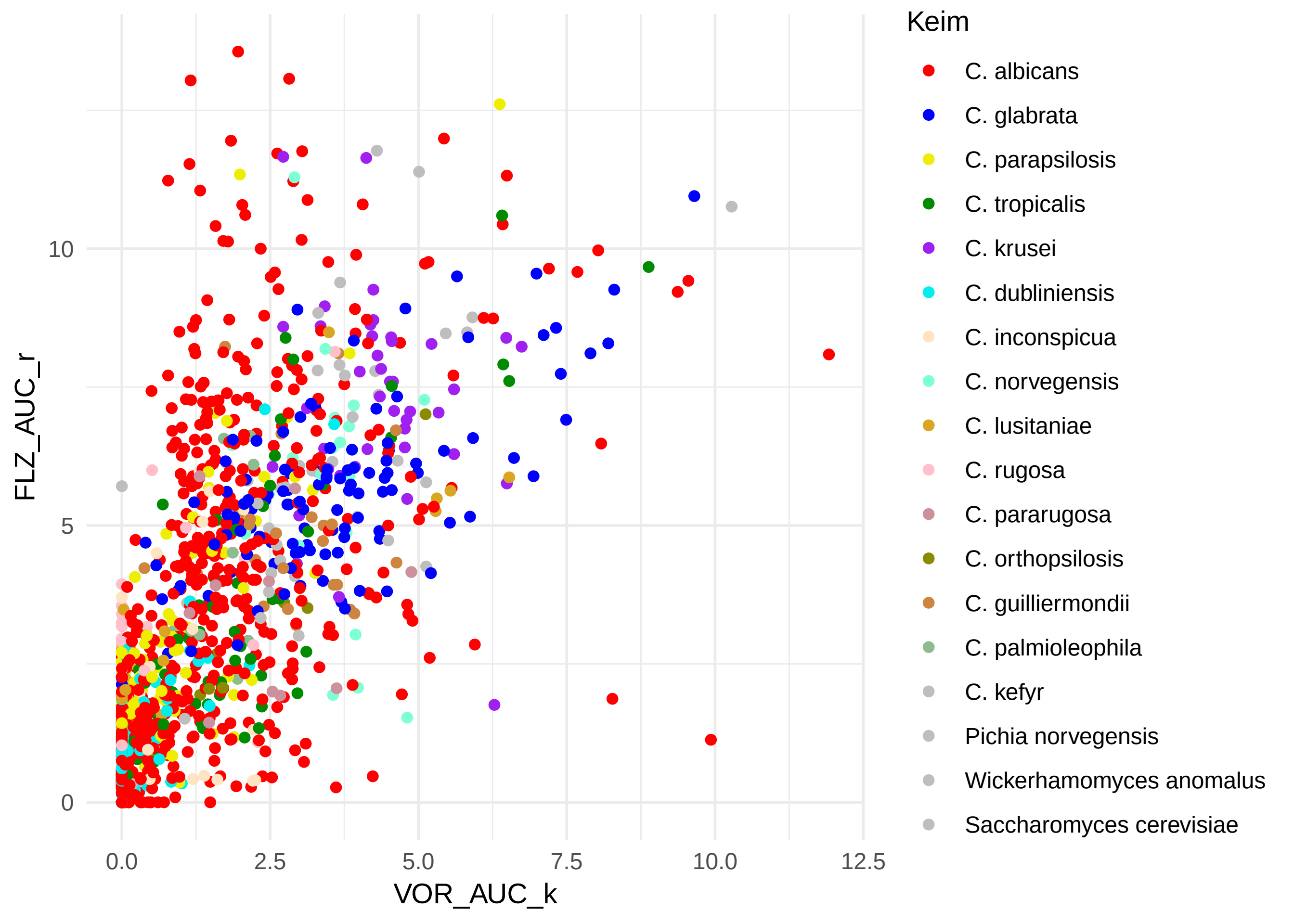

Keim
C. albicans
C. glabrata
C. parapsilosis
C. tropicalis
C. krusei
C. dubliniensis
C. inconspicua
C. norvegensis
C. lusitaniae
C. rugosa
C. pararugosa
C. orthopsilosis
C. guilliermondii
C. palmioleophila
C. kefyr
Pichia norvegensis
Wickerhamomyces anomalus
Saccharomyces cerevisiae
FLZ_AUC_r
10
5
0
0.0
2.5
5.0
7.5
10.0
12.5
VOR_AUC_k

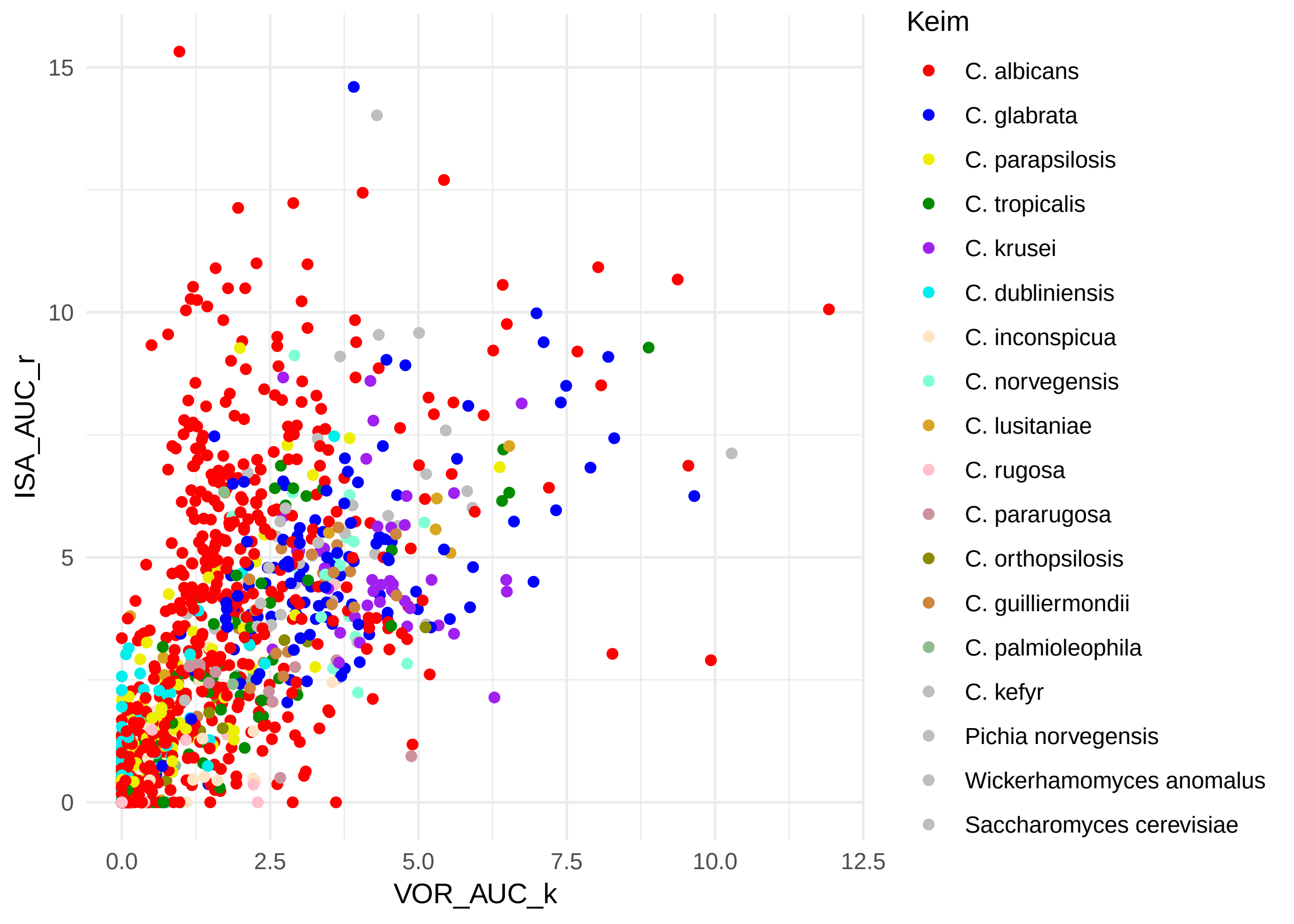
Keim
C. albicans
C. glabrata
C. parapsilosis
C. tropicalis
C. krusei
C. dubliniensis
C. inconspicua
C. norvegensis
C. lusitaniae
C. rugosa
C. pararugosa
C. orthopsilosis
C. guilliermondii
C. palmioleophila
C. kefyr
Pichia norvegensis
Wickerhamomyces anomalus
Saccharomyces cerevisiae
ISA_AUC_r
VOR_AUC_k
0
5
10
15
0.0
2.5
5.0
7.5
10.0
12.5

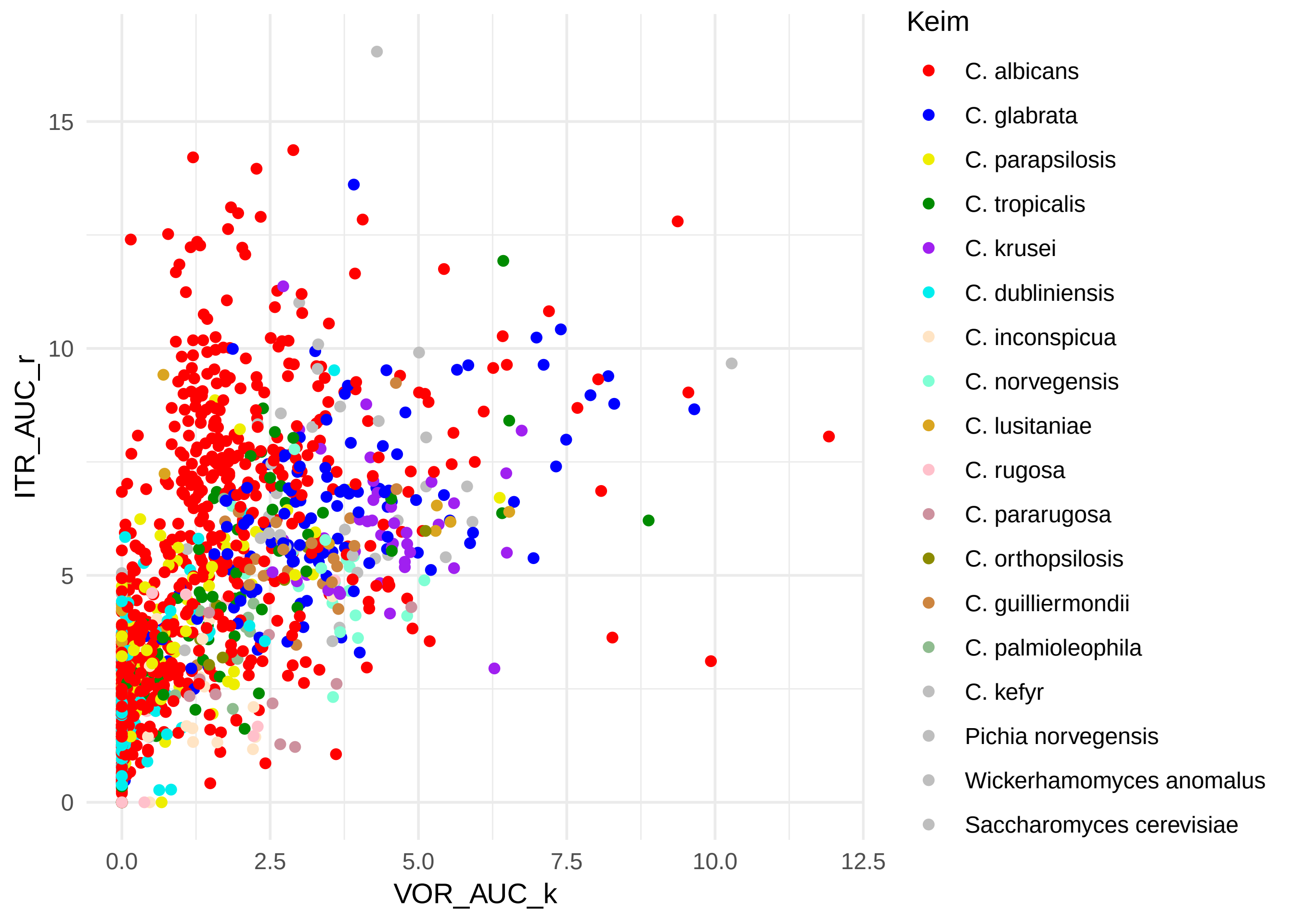

Keim
C. albicans
C. glabrata
C. parapsilosis
C. tropicalis
C. krusei
C. dubliniensis
C. inconspicua
C. norvegensis
C. lusitaniae
C. rugosa
C. pararugosa
C. orthopsilosis
C. guilliermondii
C. palmioleophila
C. kefyr
Pichia norvegensis
Wickerhamomyces anomalus
Saccharomyces cerevisiae
ITR_AUC_r
VOR_AUC_k
0
5
10
15
0.0
2.5
5.0
7.5
10.0
12.5

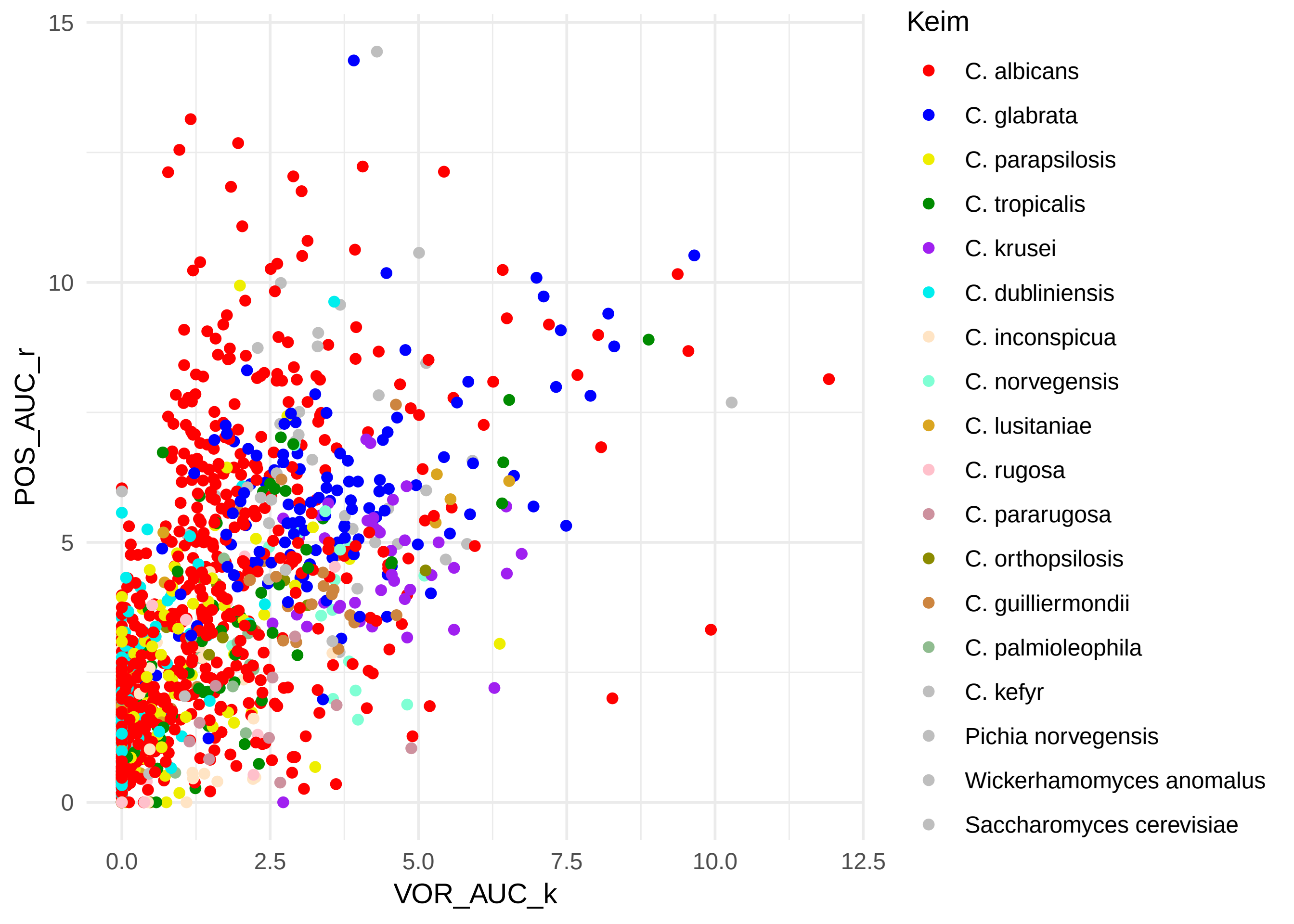

Keim
C. albicans
C. glabrata
C. parapsilosis
C. tropicalis
C. krusei
C. dubliniensis
C. inconspicua
C. norvegensis
C. lusitaniae
C. rugosa
C. pararugosa
C. orthopsilosis
C. guilliermondii
C. palmioleophila
C. kefyr
Pichia norvegensis
Wickerhamomyces anomalus
Saccharomyces cerevisiae
POS_AUC_r
VOR_AUC_k
0
5
10
15
0.0
2.5
5.0
7.5
10.0
12.5

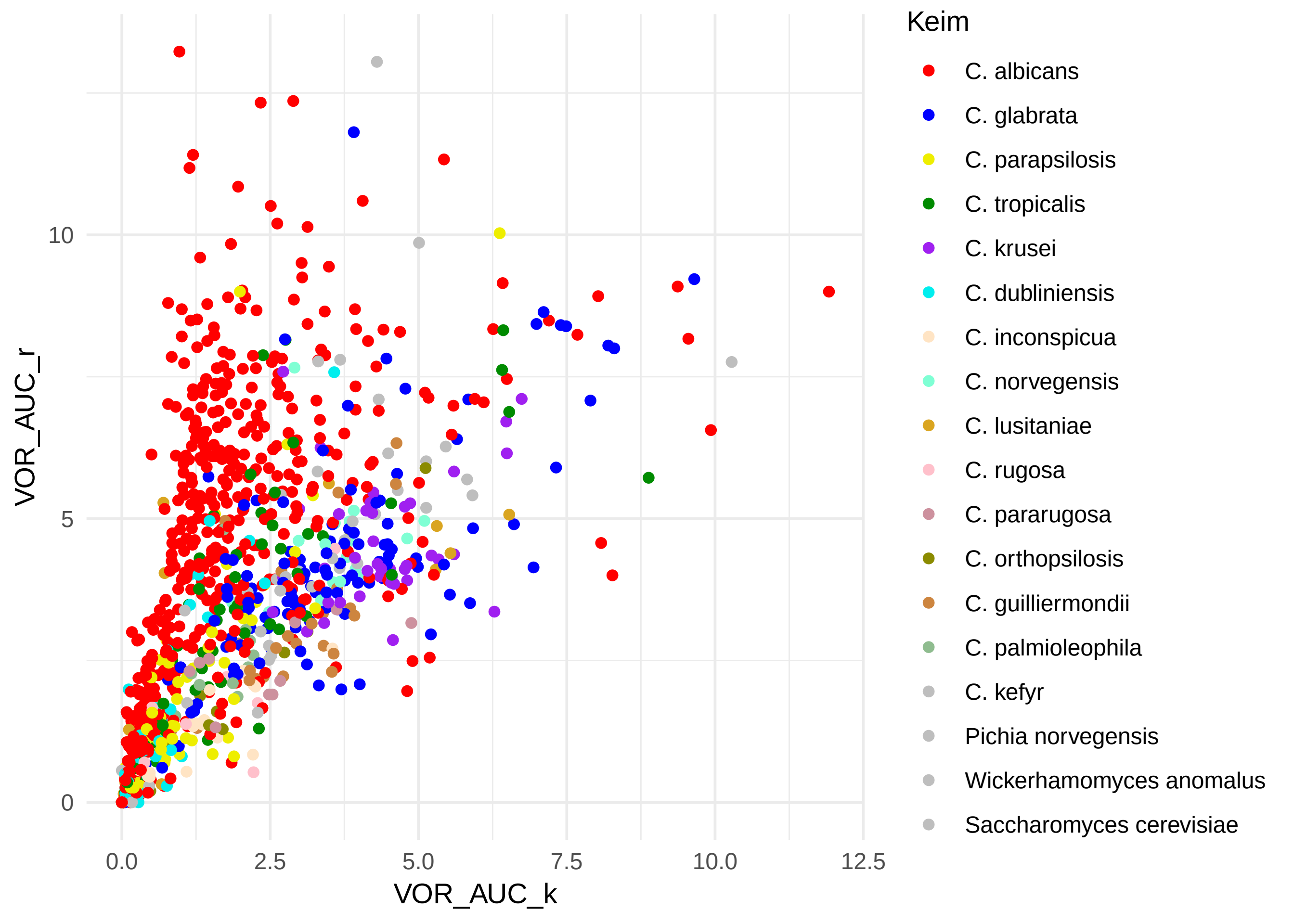
Keim
C. albicans
C. glabrata
C. parapsilosis
C. tropicalis
C. krusei
C. dubliniensis
C. inconspicua
C. norvegensis
C. lusitaniae
C. rugosa
C. pararugosa
C. orthopsilosis
C. guilliermondii
C. palmioleophila
C. kefyr
Pichia norvegensis
Wickerhamomyces anomalus
Saccharomyces cerevisiae
VOR_AUC_r
10
5
0
0.0
2.5
5.0
7.5
10.0
12.5
VOR_AUC_k

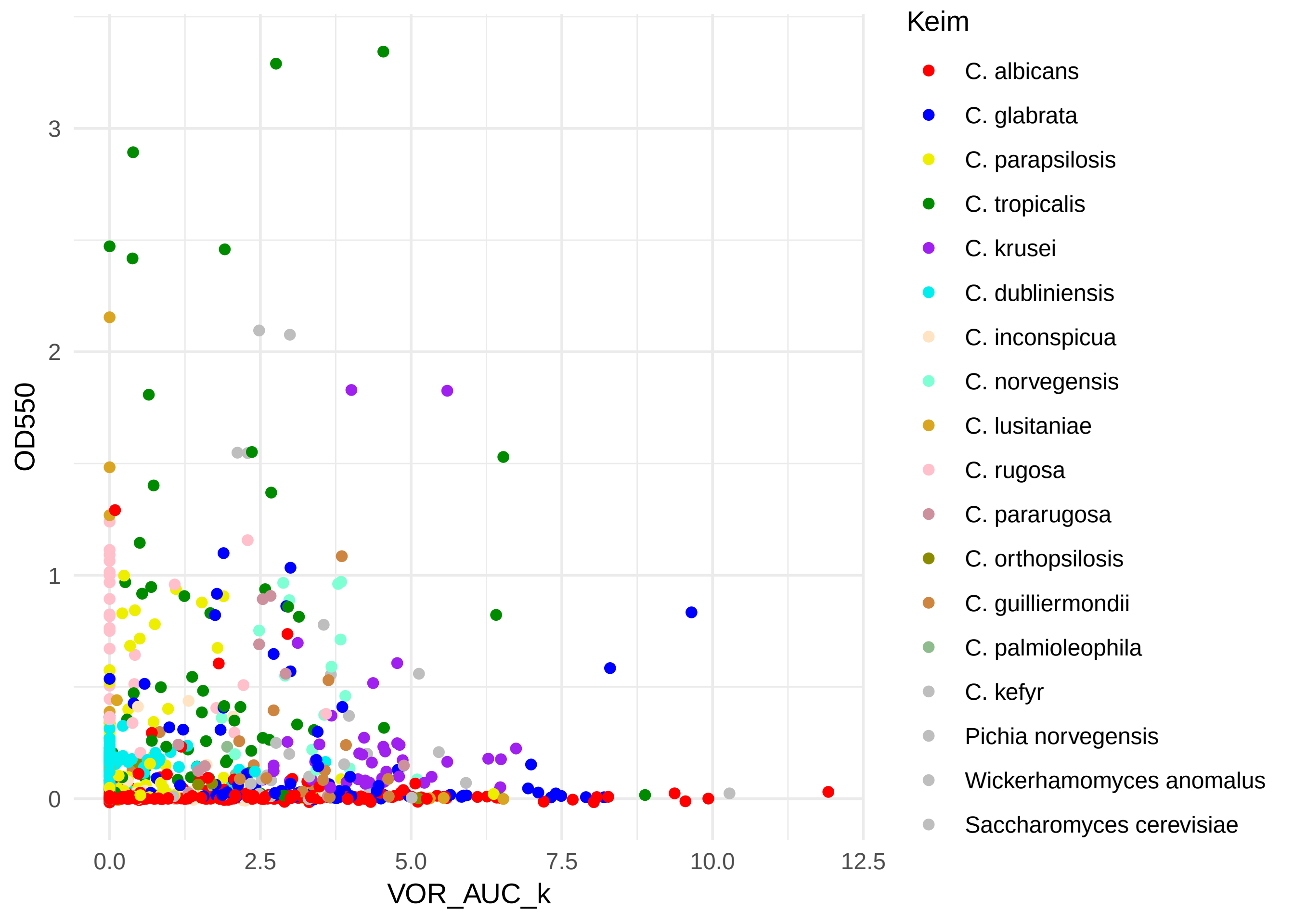
Keim
C. albicans
C. glabrata
C. parapsilosis
C. tropicalis
C. krusei
C. dubliniensis
C. inconspicua
C. norvegensis
C. lusitaniae
C. rugosa
C. pararugosa
C. orthopsilosis
C. guilliermondii
C. palmioleophila
C. kefyr
Pichia norvegensis
Wickerhamomyces anomalus
Saccharomyces cerevisiae
OD550
3
2
1
0
0.0
2.5
5.0
7.5
10.0
12.5
VOR_AUC_k

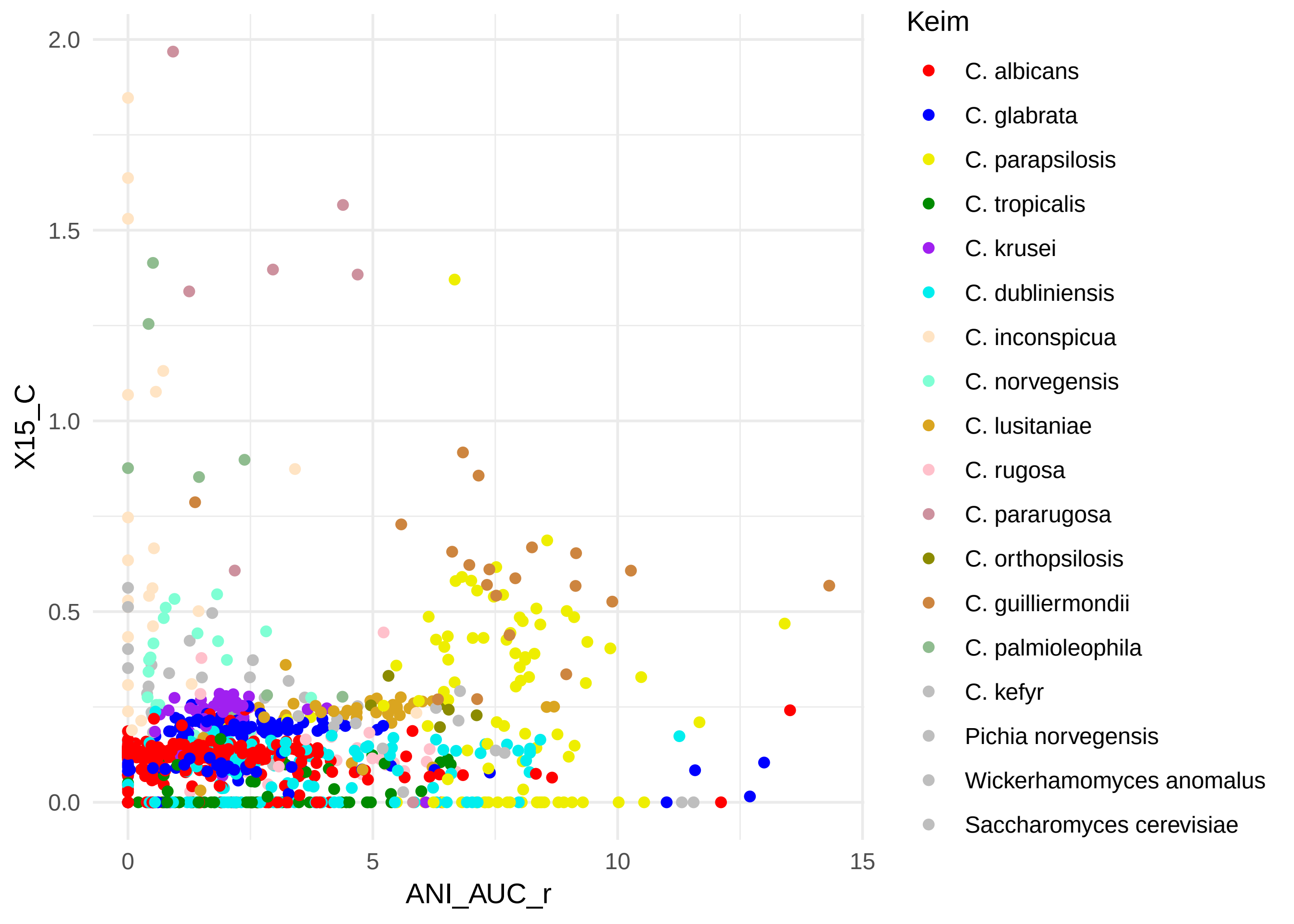

Keim
C. albicans
C. glabrata
C. parapsilosis
C. tropicalis
C. krusei
C. dubliniensis
C. inconspicua
C. norvegensis
C. lusitaniae
C. rugosa
C. pararugosa
C. orthopsilosis
C. guilliermondii
C. palmioleophila
C. kefyr
Pichia norvegensis
Wickerhamomyces anomalus
Saccharomyces cerevisiae
X15_C
2.0
1.5
1.0
0.5
0.0
0
5
10
15
ANI_AUC_r

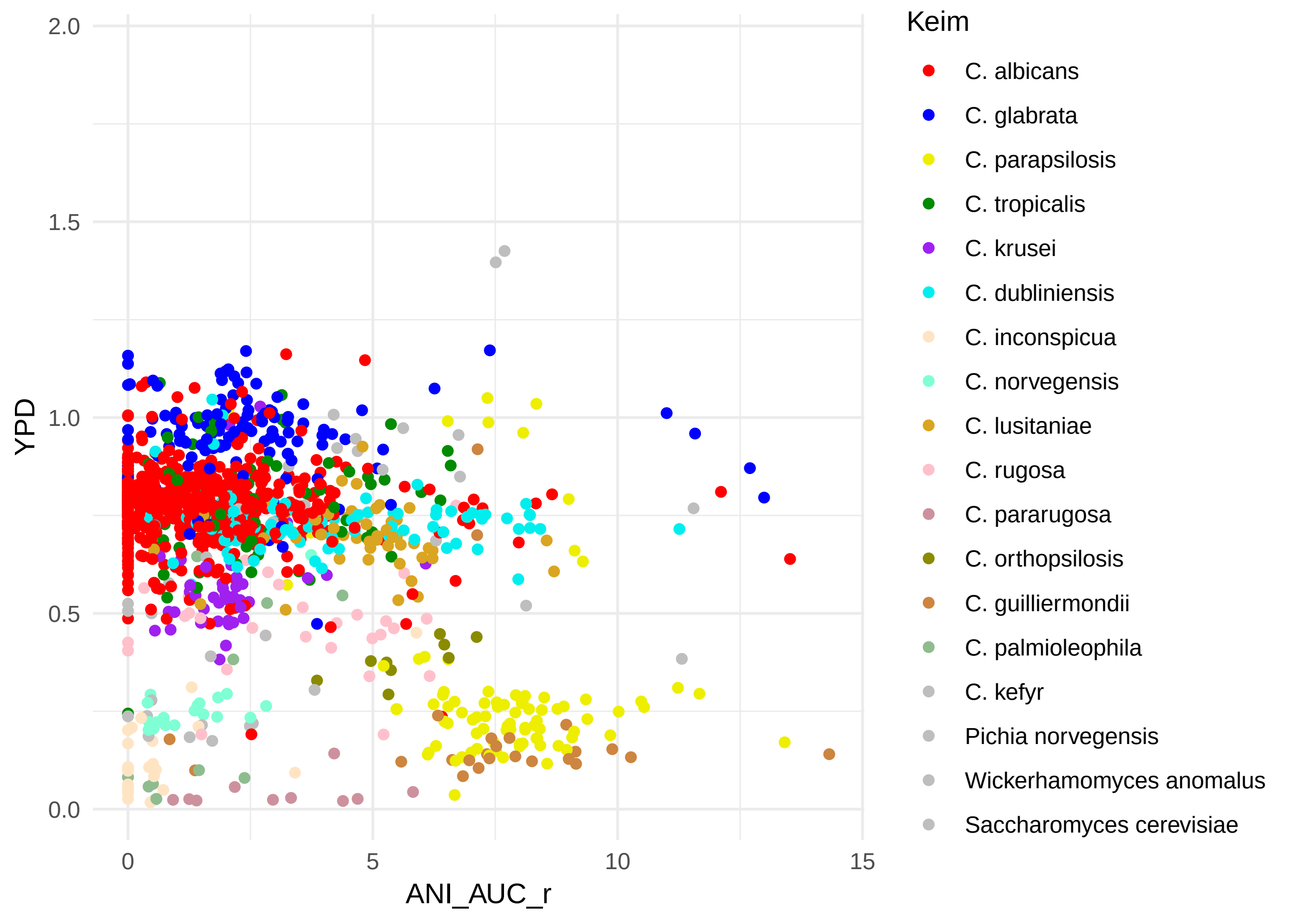
2.0
1.5
1.0
0.5
0.0
YPD
0
5
10
15
ANI_AUC_r
Keim
C. albicans
C. glabrata
C. parapsilosis
C. tropicalis
C. krusei
C. dubliniensis
C. inconspicua
C. norvegensis
C. lusitaniae
C. rugosa
C. pararugosa
C. orthopsilosis
C. guilliermondii
C. palmioleophila
C. kefyr
Pichia norvegensis
Wickerhamomyces anomalus
Saccharomyces cerevisiae

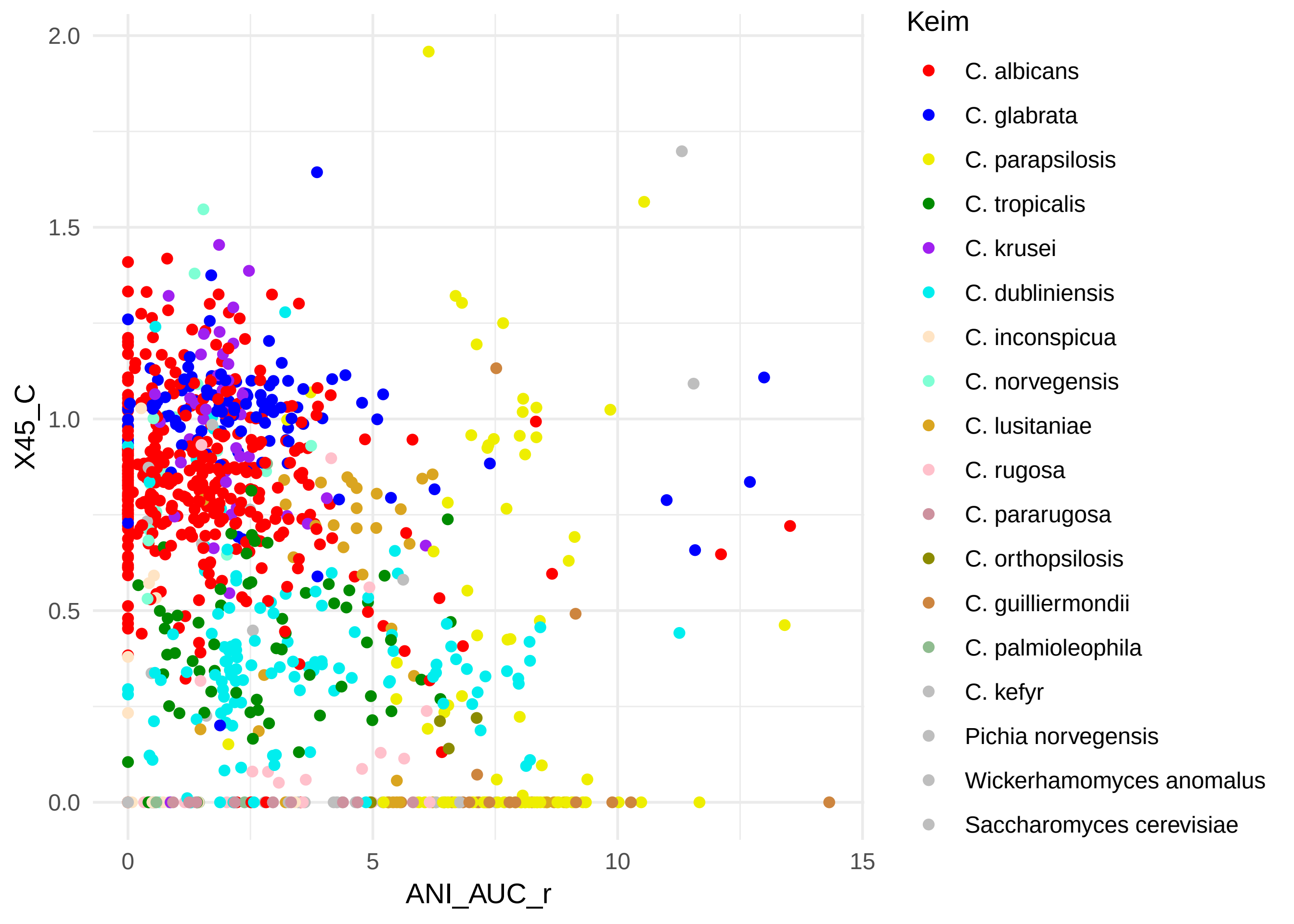

Keim
C. albicans
C. glabrata
C. parapsilosis
C. tropicalis
C. krusei
C. dubliniensis
C. inconspicua
C. norvegensis
C. lusitaniae
C. rugosa
C. pararugosa
C. orthopsilosis
C. guilliermondii
C. palmioleophila
C. kefyr
Pichia norvegensis
Wickerhamomyces anomalus
Saccharomyces cerevisiae
X45_C
ANI_AUC_r
2.0
1.5
1.0
0.5
0.0
0
5
10
15

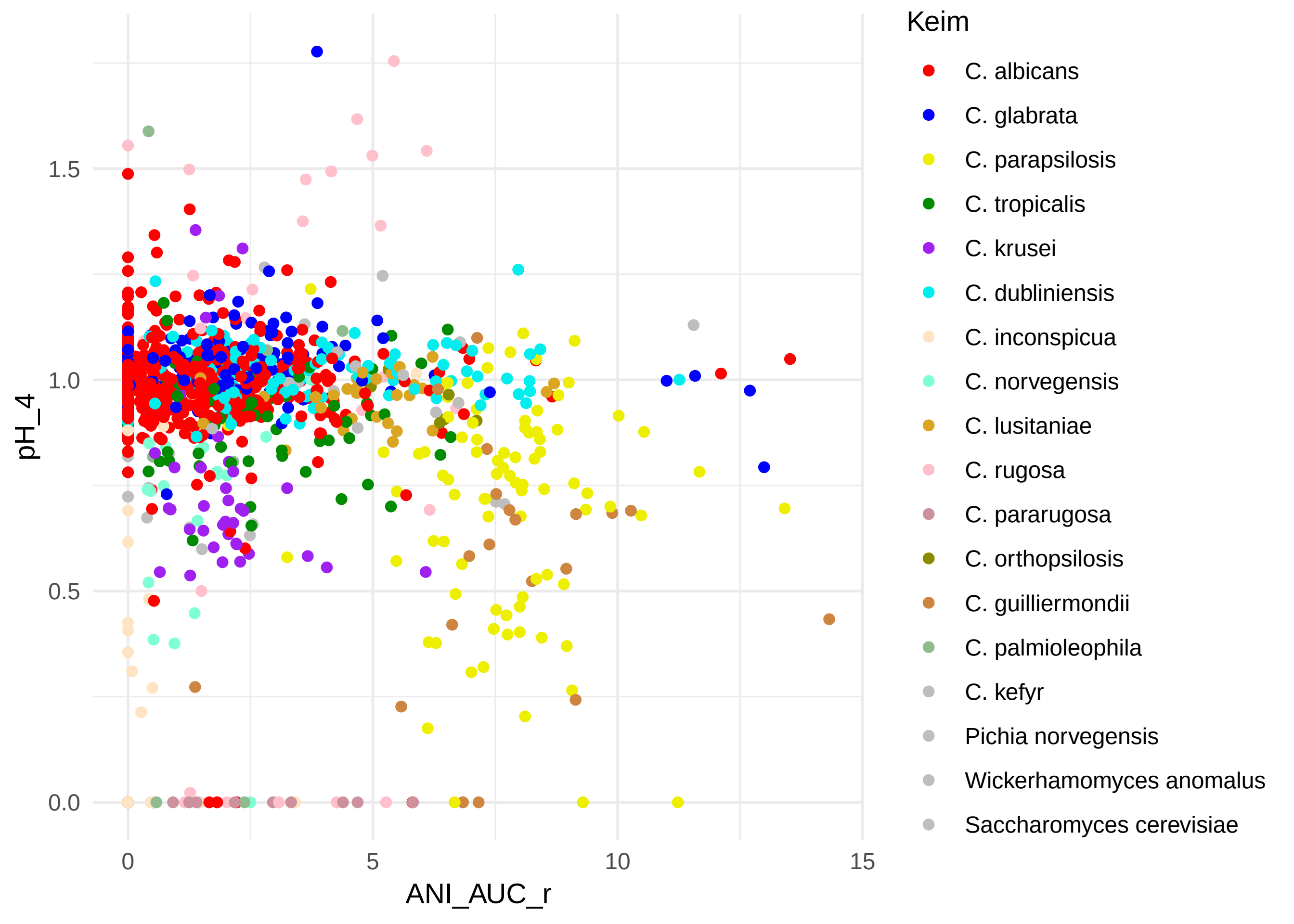

Keim
C. albicans
C. glabrata
C. parapsilosis
C. tropicalis
C. krusei
C. dubliniensis
C. inconspicua
C. norvegensis
C. lusitaniae
C. rugosa
C. pararugosa
C. orthopsilosis
C. guilliermondii
C. palmioleophila
C. kefyr
Pichia norvegensis
Wickerhamomyces anomalus
Saccharomyces cerevisiae
pH_4
1.5
1.0
0.5
0.0
0
5
10
15
ANI_AUC_r

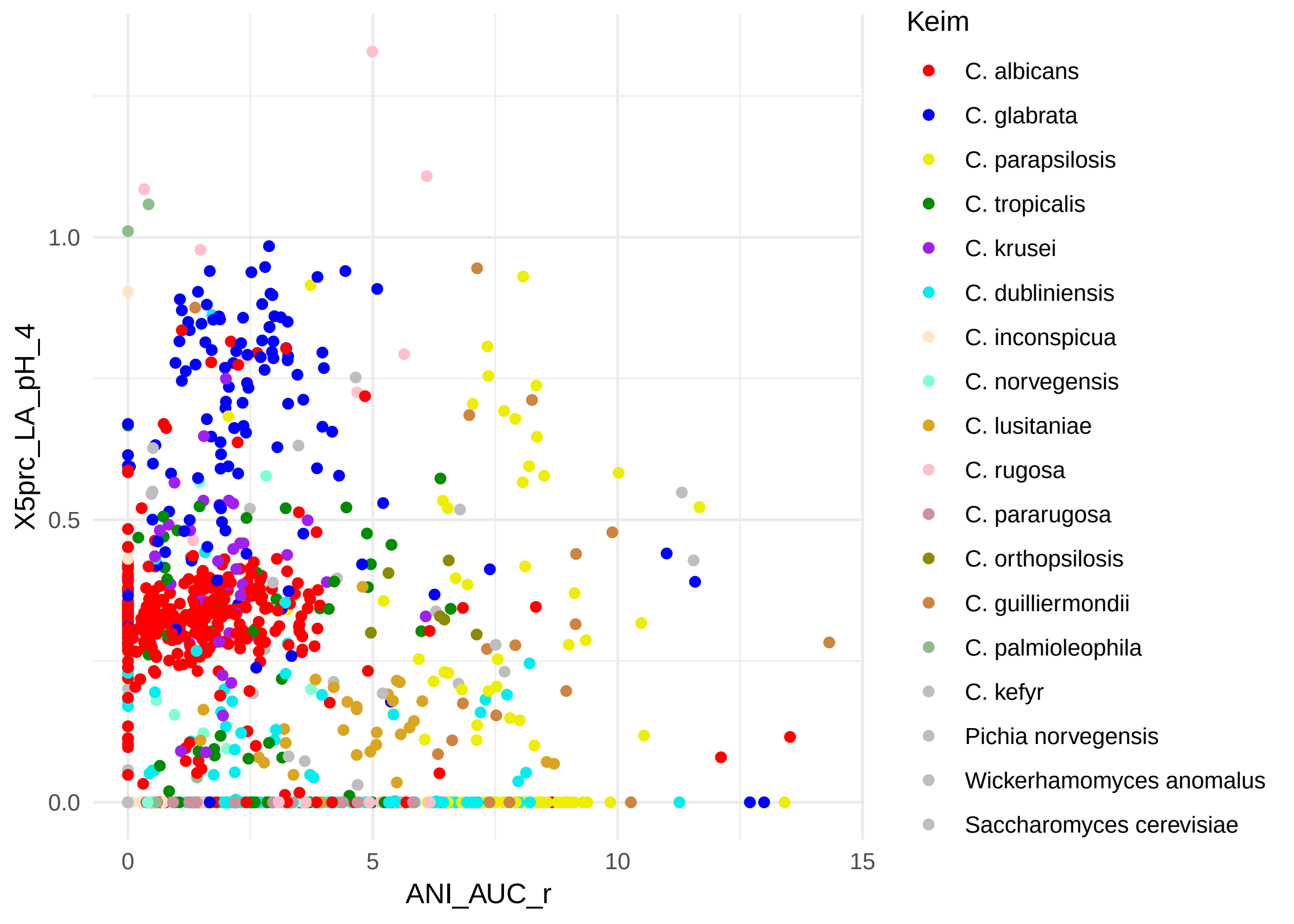
X5prc_LA_pH_4
ANI_AUC_r
0.0
0.5
1.0
0
5
10
15
Keim
C. albicans
C. glabrata
C. parapsilosis
C. tropicalis
C. krusei
C. dubliniensis
C. inconspicua
C. norvegensis
C. lusitaniae
C. rugosa
C. pararugosa
C. orthopsilosis
C. guilliermondii
C. palmioleophila
C. kefyr
Pichia norvegensis
Wickerhamomyces anomalus
Saccharomyces cerevisiae

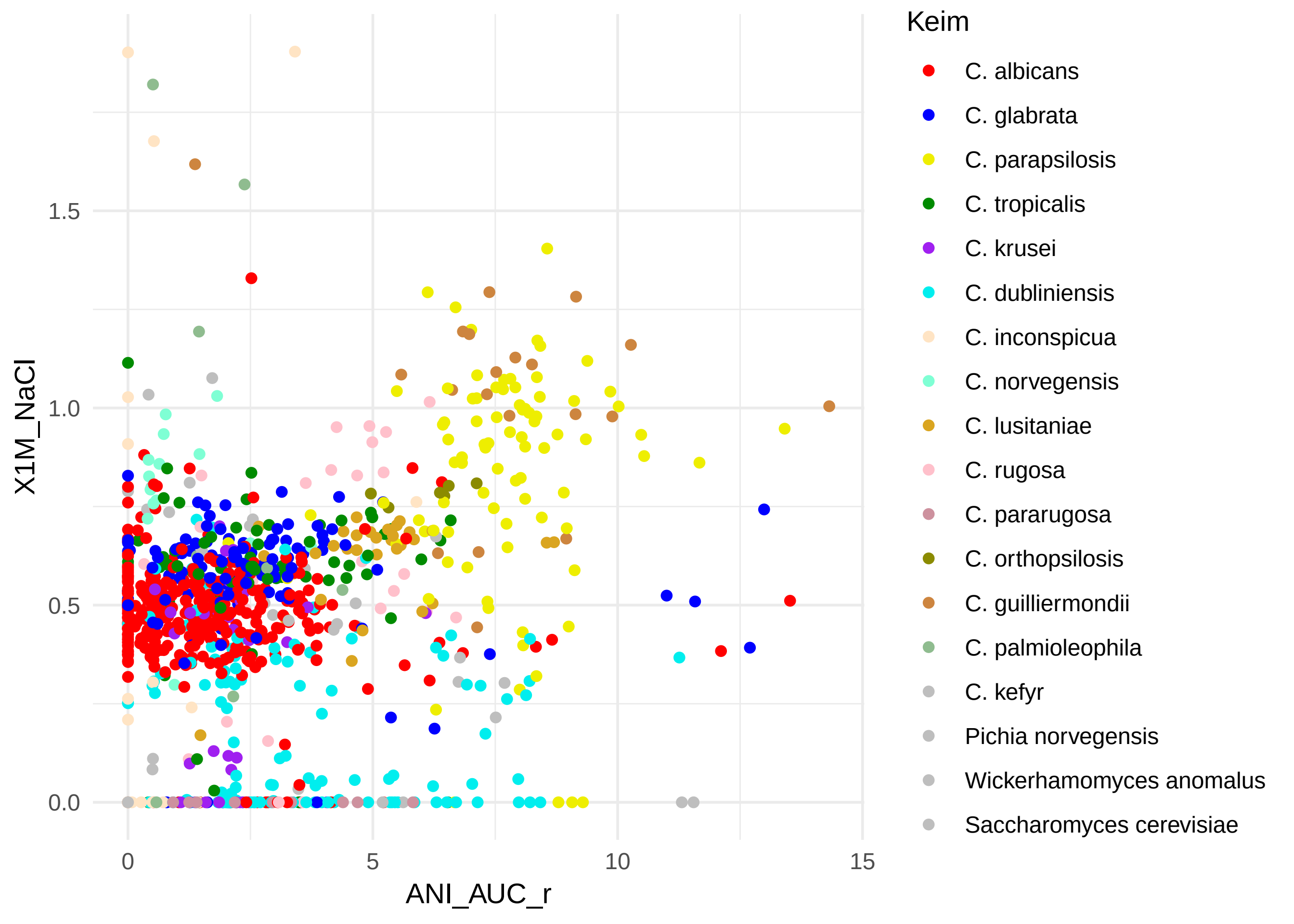

X1M_NaCl
0.0
0.5
1.0
1.5
0
5
10
15
ANI_AUC_r
Keim
C. albicans
C. glabrata
C. parapsilosis
C. tropicalis
C. krusei
C. dubliniensis
C. inconspicua
C. norvegensis
C. lusitaniae
C. rugosa
C. pararugosa
C. orthopsilosis
C. guilliermondii
C. palmioleophila
C. kefyr
Pichia norvegensis
Wickerhamomyces anomalus
Saccharomyces cerevisiae

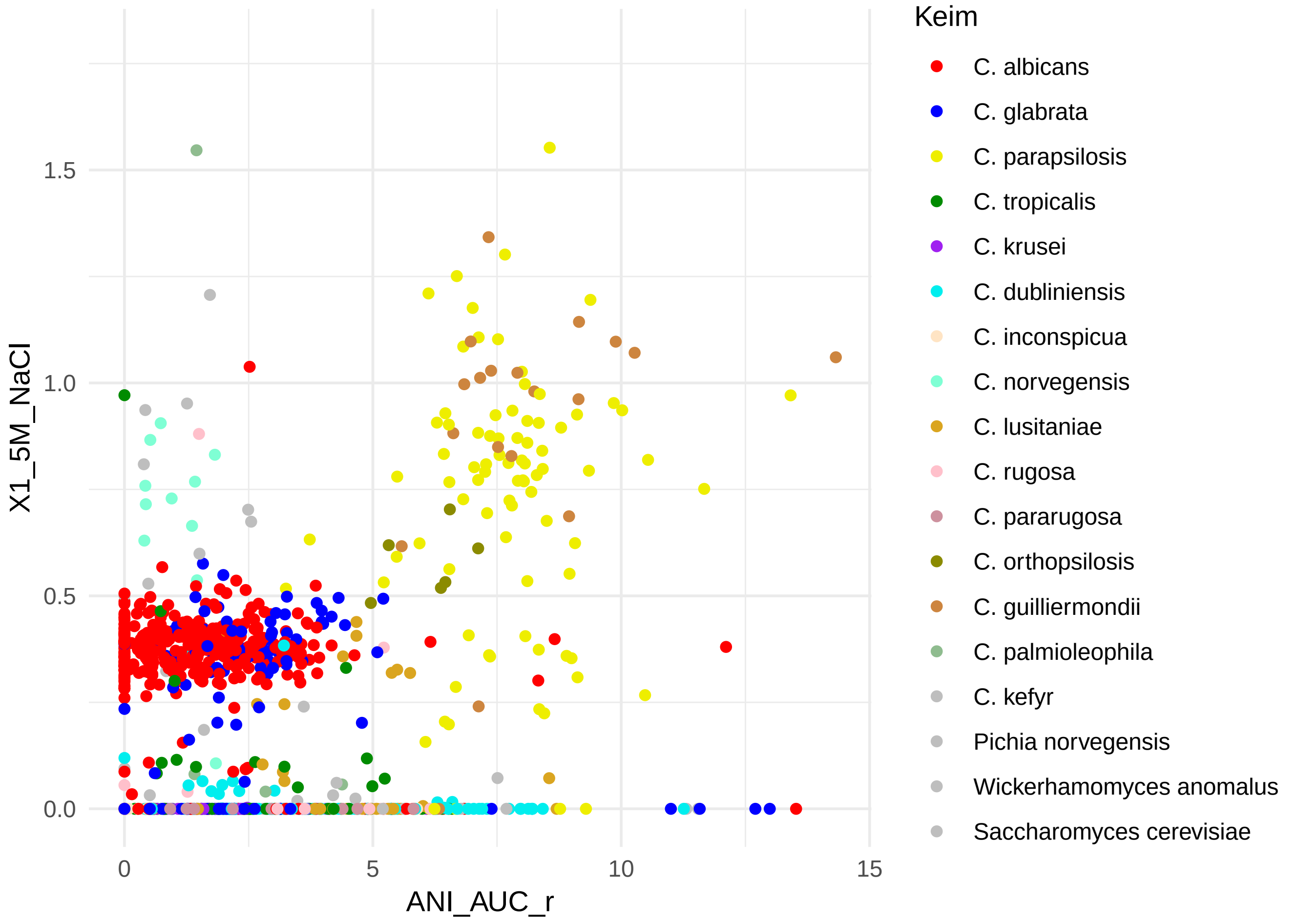

X1_5M_NaCl
ANI_AUC_r
0.0
0.5
1.0
1.5
0
5
10
15
Keim
C. albicans
C. glabrata
C. parapsilosis
C. tropicalis
C. krusei
C. dubliniensis
C. inconspicua
C. norvegensis
C. lusitaniae
C. rugosa
C. pararugosa
C. orthopsilosis
C. guilliermondii
C. palmioleophila
C. kefyr
Pichia norvegensis
Wickerhamomyces anomalus
Saccharomyces cerevisiae

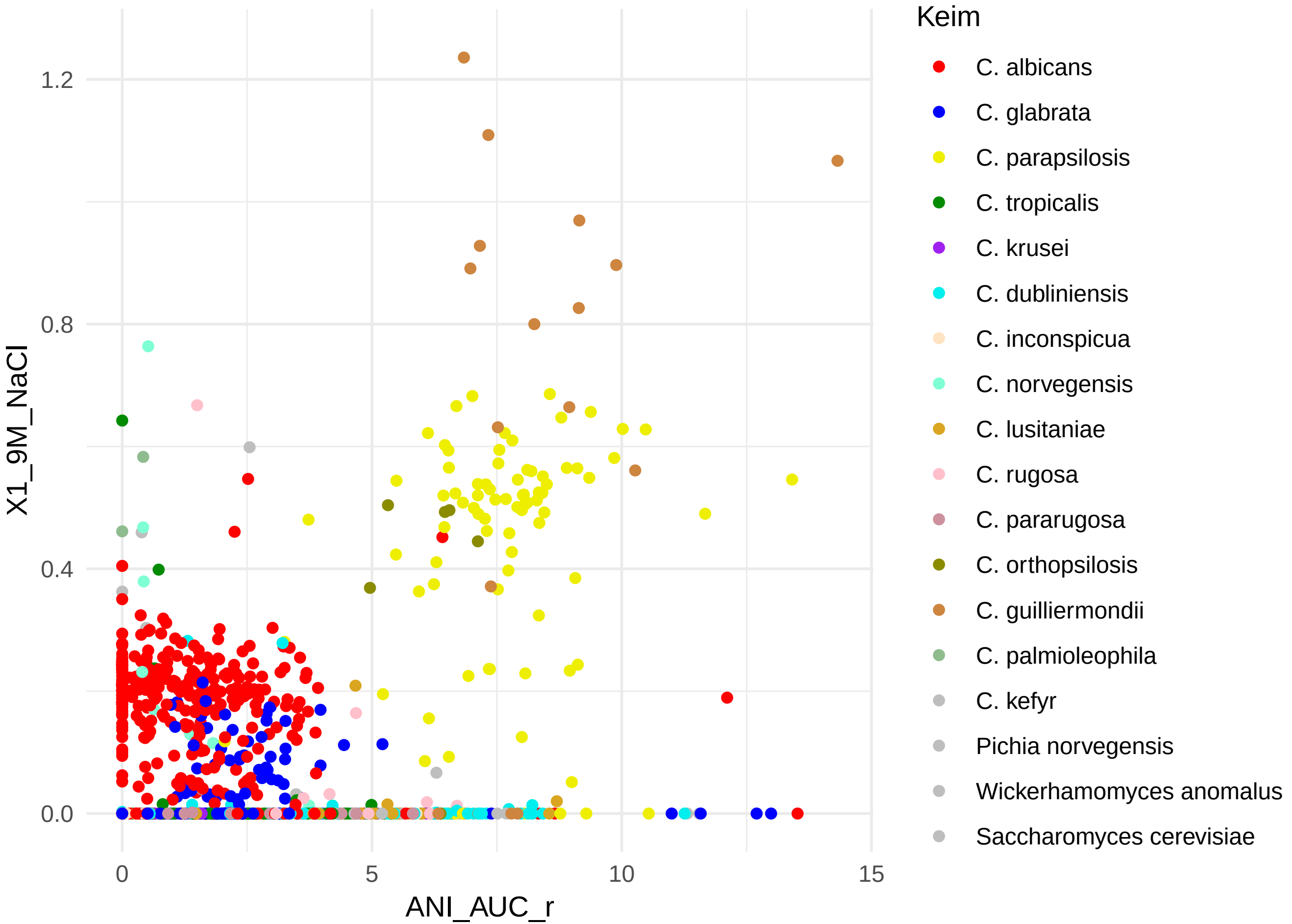

Keim
C. albicans
C. glabrata
C. parapsilosis
C. tropicalis
C. krusei
C. dubliniensis
C. inconspicua
C. norvegensis
C. lusitaniae
C. rugosa
C. pararugosa
C. orthopsilosis
C. guilliermondii
C. palmioleophila
C. kefyr
Pichia norvegensis
Wickerhamomyces anomalus
Saccharomyces cerevisiae
X1_9M_NaCl
1.2
0.8
0.4
0.0
0
5
10
15
ANI_AUC_r

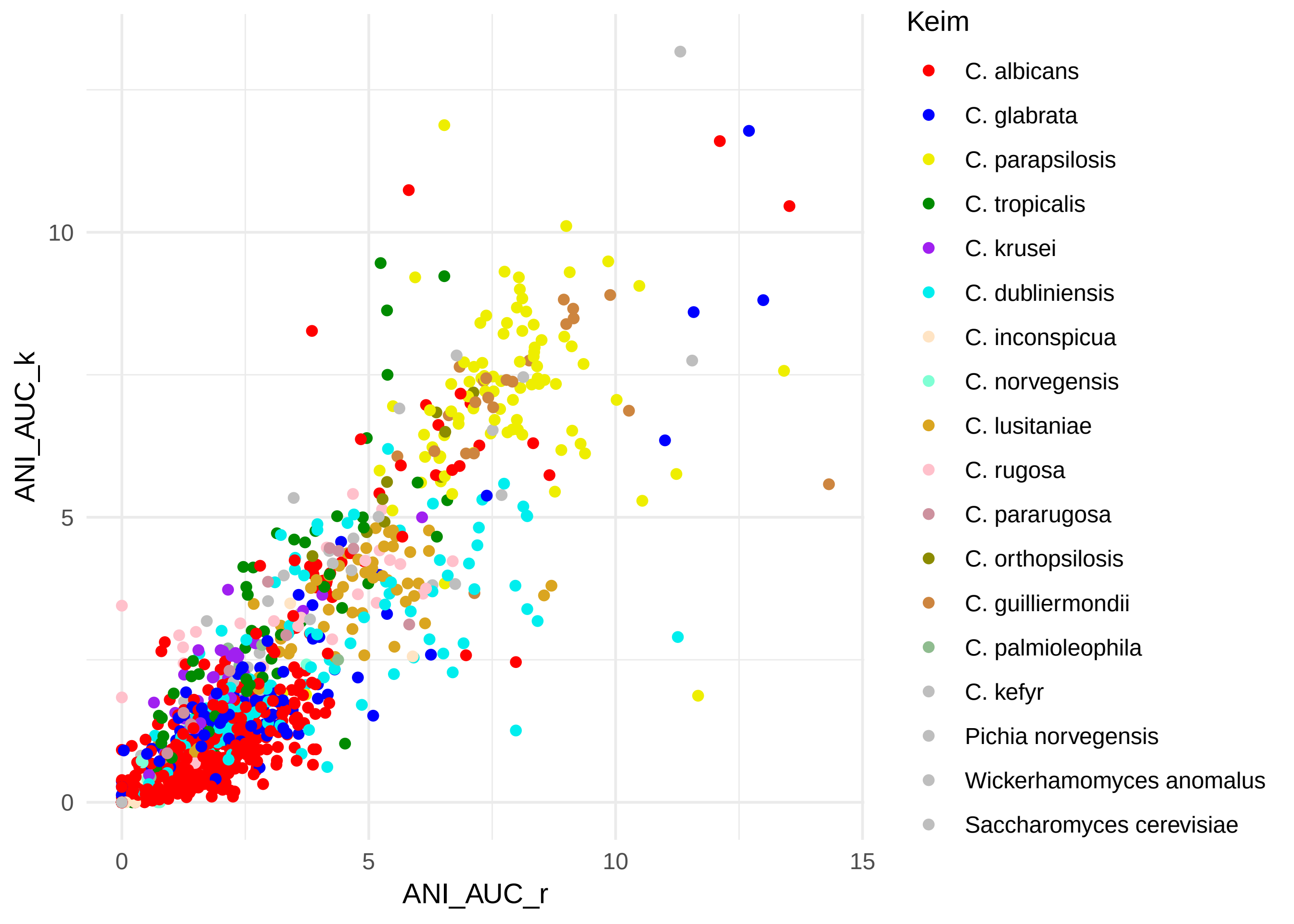

ANI_AUC_k
ANI_AUC_r
0
5
10
15
Keim
C. albicans
C. glabrata
C. parapsilosis
C. tropicalis
C. krusei
C. dubliniensis
C. inconspicua
C. norvegensis
C. lusitaniae
C. rugosa
C. pararugosa
C. orthopsilosis
C. guilliermondii
C. palmioleophila
C. kefyr
Pichia norvegensis
Wickerhamomyces anomalus
Saccharomyces cerevisiae

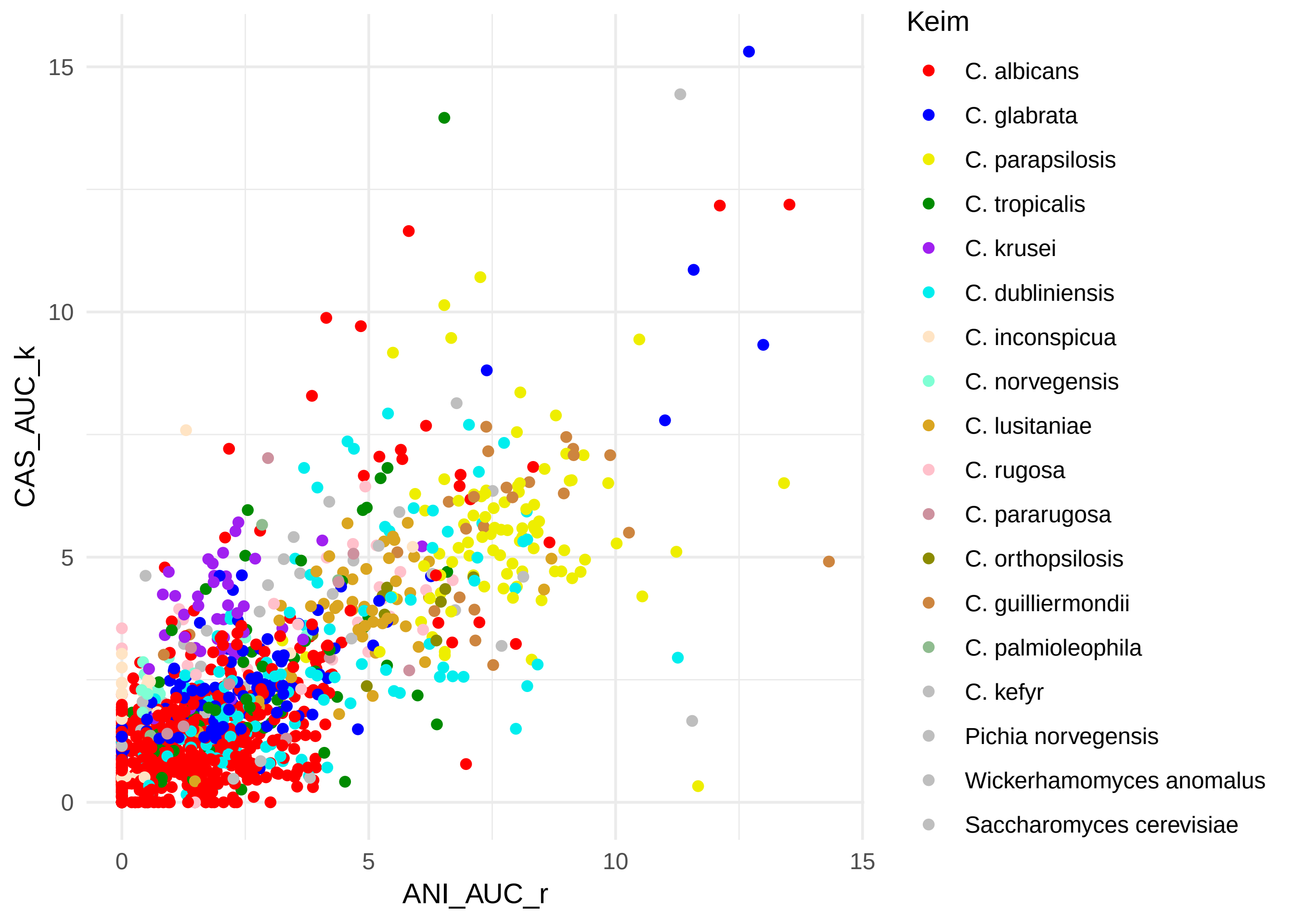
Keim
C. albicans
C. glabrata
C. parapsilosis
C. tropicalis
C. krusei
C. dubliniensis
C. inconspicua
C. norvegensis
C. lusitaniae
C. rugosa
C. pararugosa
C. orthopsilosis
C. guilliermondii
C. palmioleophila
C. kefyr
Pichia norvegensis
Wickerhamomyces anomalus
Saccharomyces cerevisiae
CAS_AUC_k
ANI_AUC_r
0
5
10
15
0
5
10
15

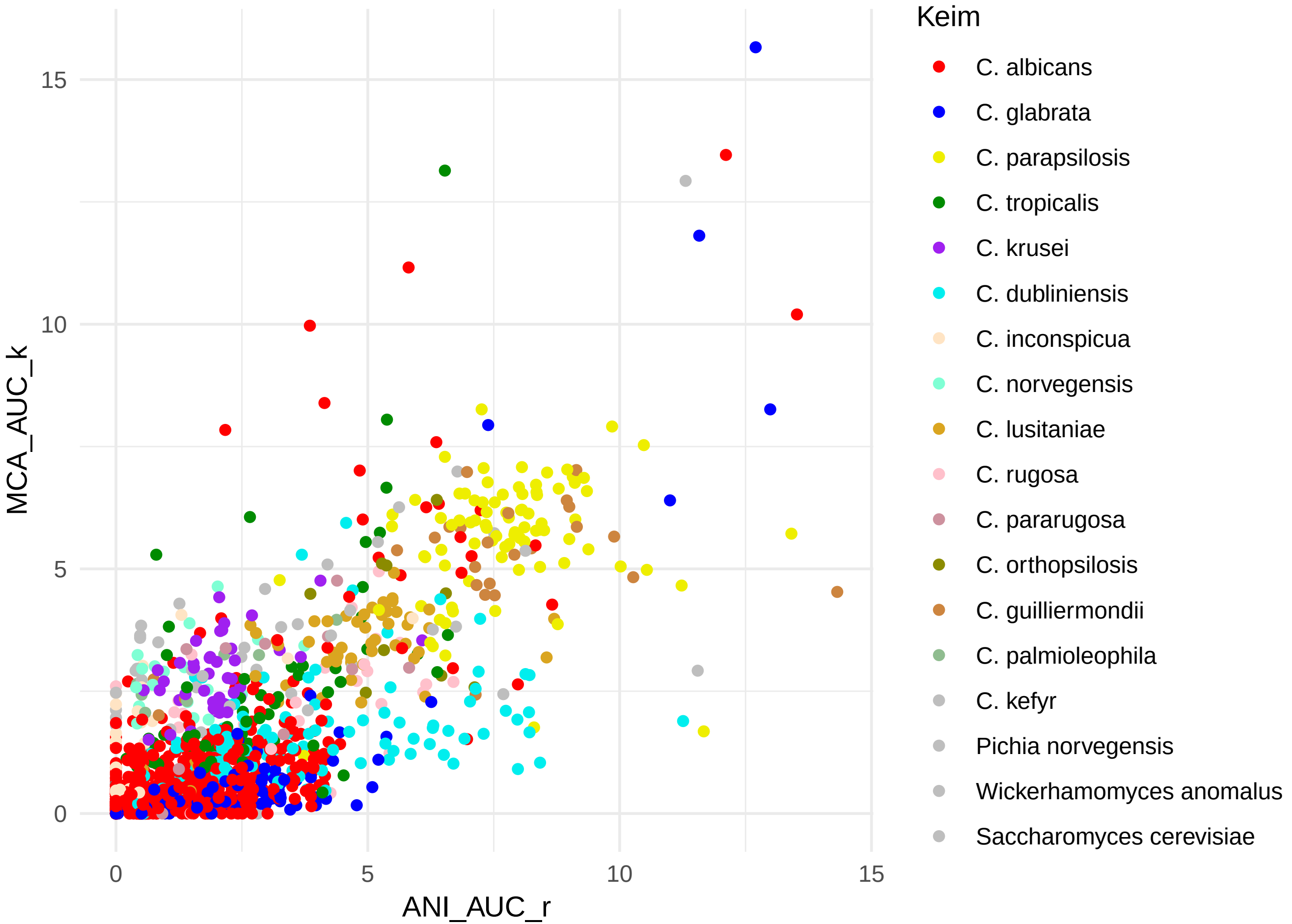

Keim
C. albicans
C. glabrata
C. parapsilosis
C. tropicalis
C. krusei
C. dubliniensis
C. inconspicua
C. norvegensis
C. lusitaniae
C. rugosa
C. pararugosa
C. orthopsilosis
C. guilliermondii
C. palmioleophila
C. kefyr
Pichia norvegensis
Wickerhamomyces anomalus
Saccharomyces cerevisiae
MCA_AUC_k
ANI_AUC_r
0
5
10
15

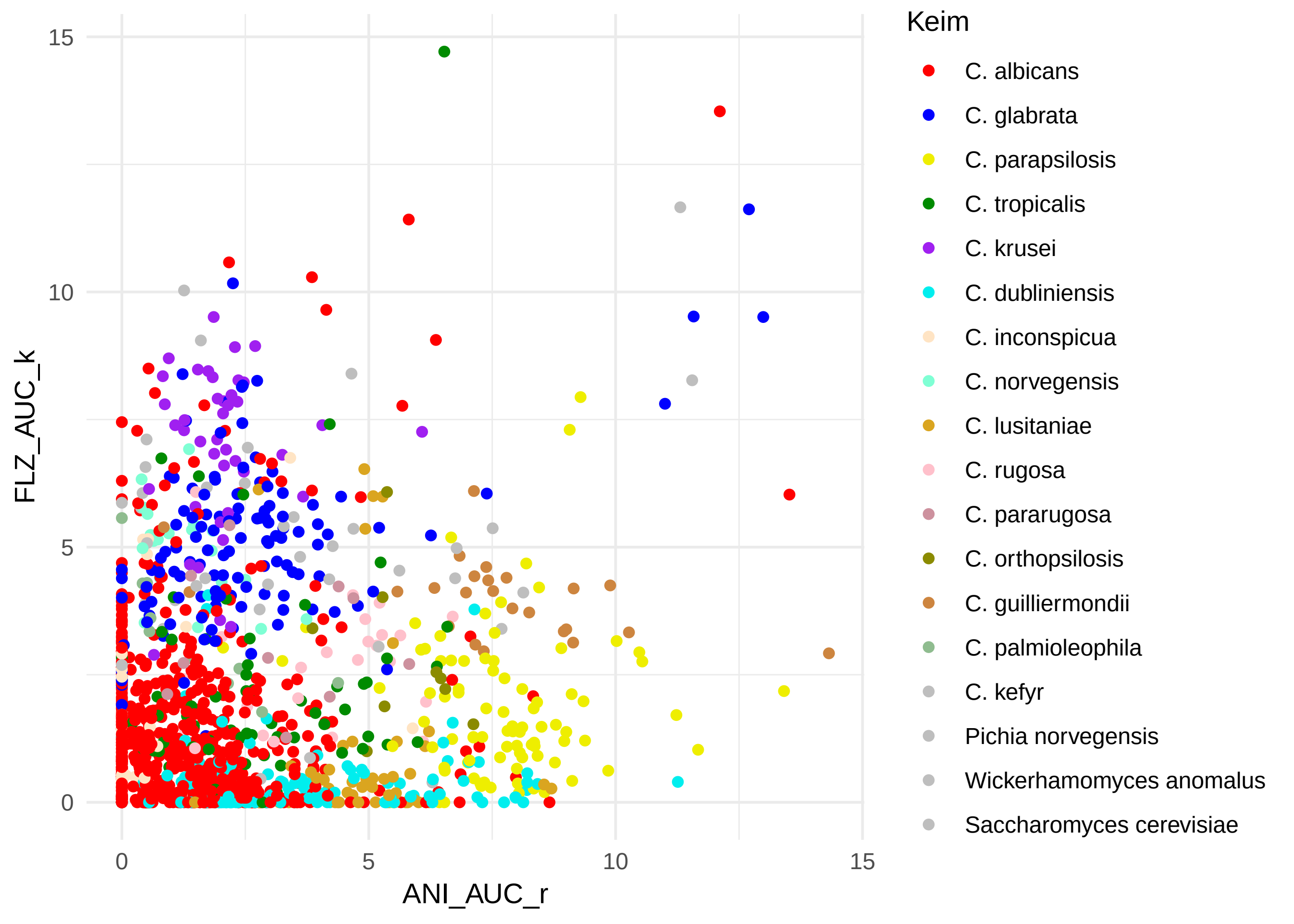
Keim
C. albicans
C. glabrata
C. parapsilosis
C. tropicalis
C. krusei
C. dubliniensis
C. inconspicua
C. norvegensis
C. lusitaniae
C. rugosa
C. pararugosa
C. orthopsilosis
C. guilliermondii
C. palmioleophila
C. kefyr
Pichia norvegensis
Wickerhamomyces anomalus
Saccharomyces cerevisiae
FLZ_AUC_k
ANI_AUC_r
0
5
10
15

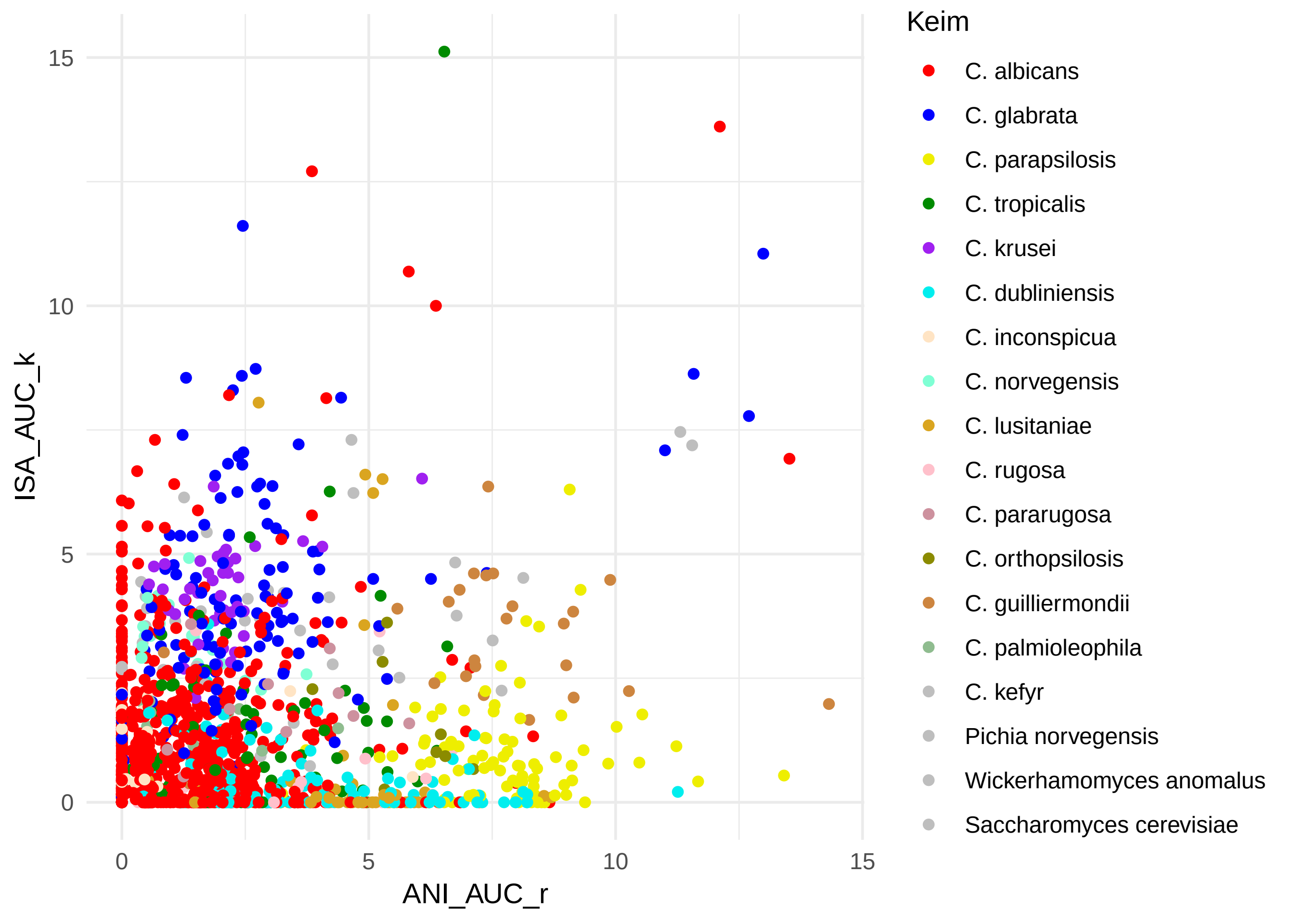

Keim
C. albicans
C. glabrata
C. parapsilosis
C. tropicalis
C. krusei
C. dubliniensis
C. inconspicua
C. norvegensis
C. lusitaniae
C. rugosa
C. pararugosa
C. orthopsilosis
C. guilliermondii
C. palmioleophila
C. kefyr
Pichia norvegensis
Wickerhamomyces anomalus
Saccharomyces cerevisiae
ISA_AUC_k
ANI_AUC_r
0
5
10
15

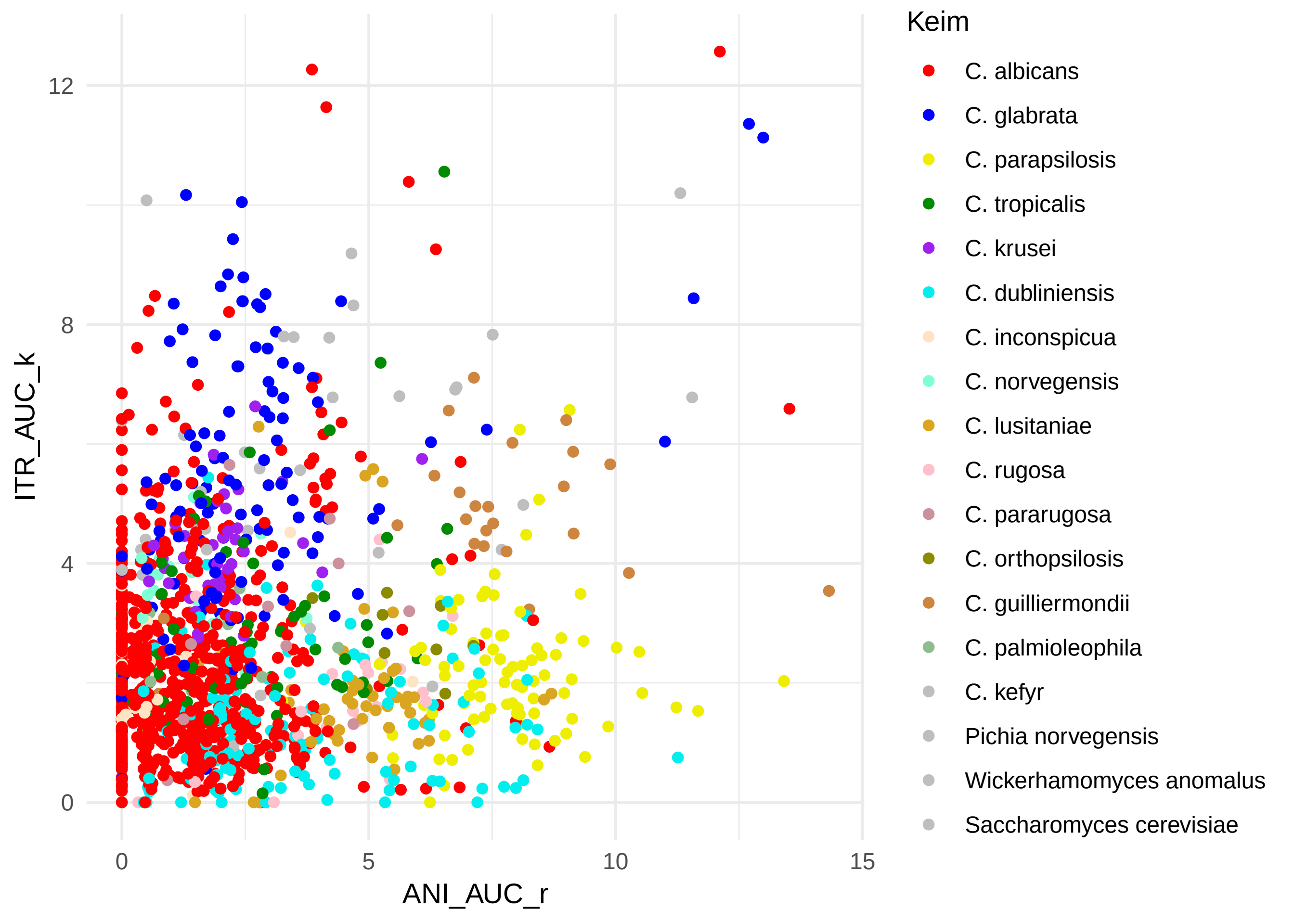

Keim
C. albicans
C. glabrata
C. parapsilosis
C. tropicalis
C. krusei
C. dubliniensis
C. inconspicua
C. norvegensis
C. lusitaniae
C. rugosa
C. pararugosa
C. orthopsilosis
C. guilliermondii
C. palmioleophila
C. kefyr
Pichia norvegensis
Wickerhamomyces anomalus
Saccharomyces cerevisiae
ITR_AUC_k
ANI_AUC_r
0
4
8
12
0
5
10
15

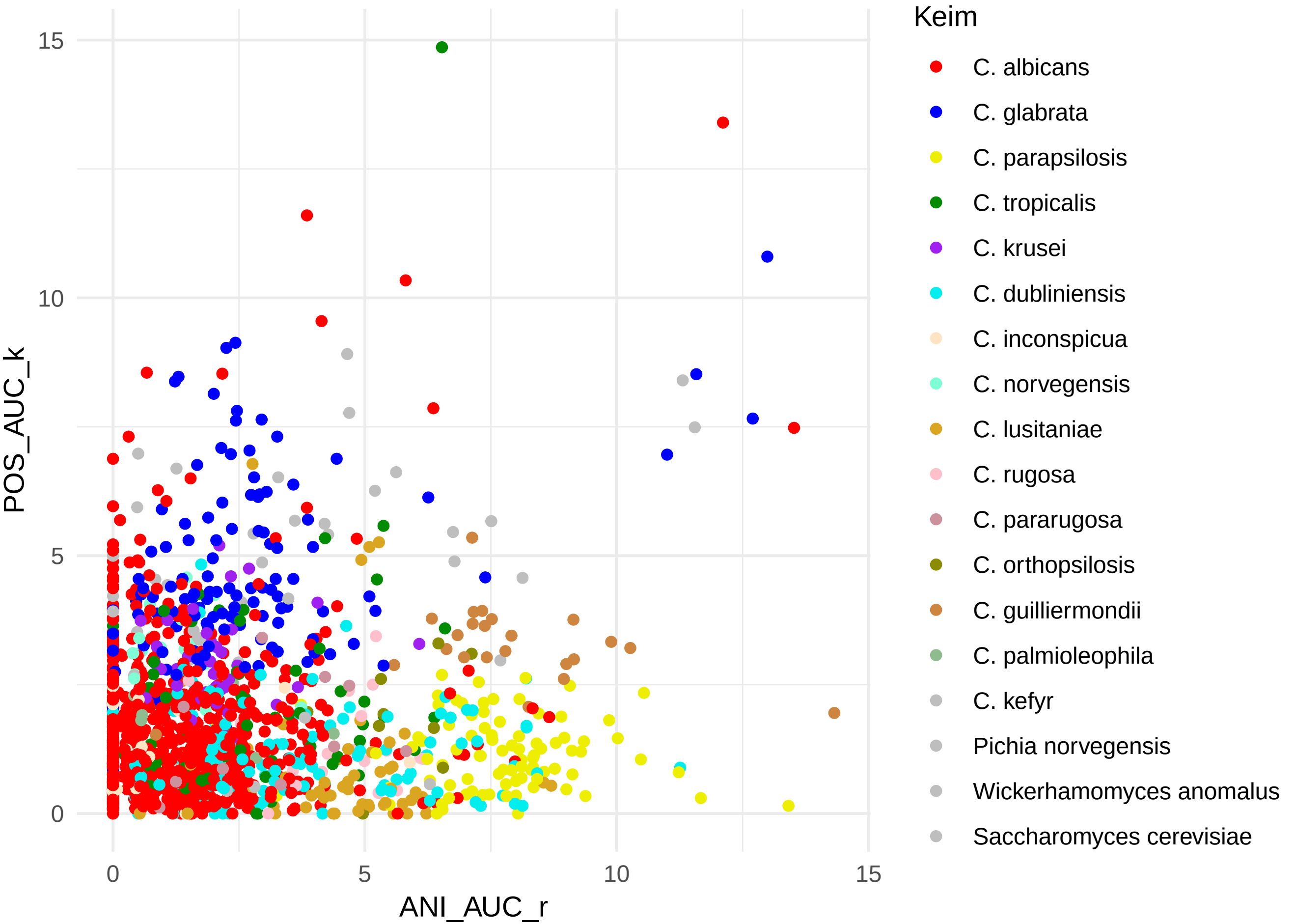
Keim
C. albicans
C. glabrata
C. parapsilosis
C. tropicalis
C. krusei
C. dubliniensis
C. inconspicua
C. norvegensis
C. lusitaniae
C. rugosa
C. pararugosa
C. orthopsilosis
C. guilliermondii
C. palmioleophila
C. kefyr
Pichia norvegensis
Wickerhamomyces anomalus
Saccharomyces cerevisiae
POS_AUC_k
ANI_AUC_r
0
5
10
15

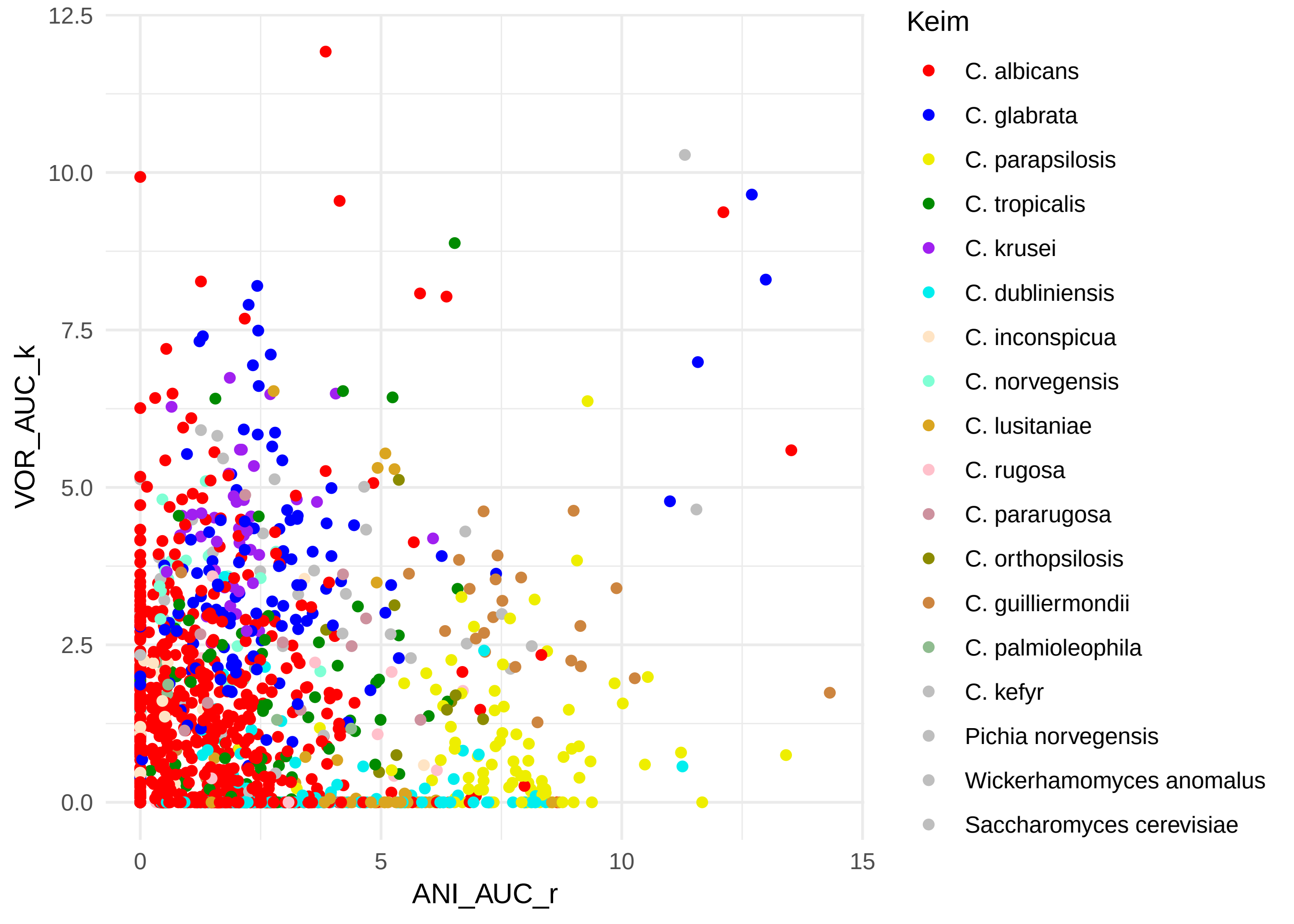

VOR_AUC_k
12.5
10.0
7.5
5.0
2.5
0.0
0
5
10
15
ANI_AUC_r
Keim
C. albicans
C. glabrata
C. parapsilosis
C. tropicalis
C. krusei
C. dubliniensis
C. inconspicua
C. norvegensis
C. lusitaniae
C. rugosa
C. pararugosa
C. orthopsilosis
C. guilliermondii
C. palmioleophila
C. kefyr
Pichia norvegensis
Wickerhamomyces anomalus
Saccharomyces cerevisiae

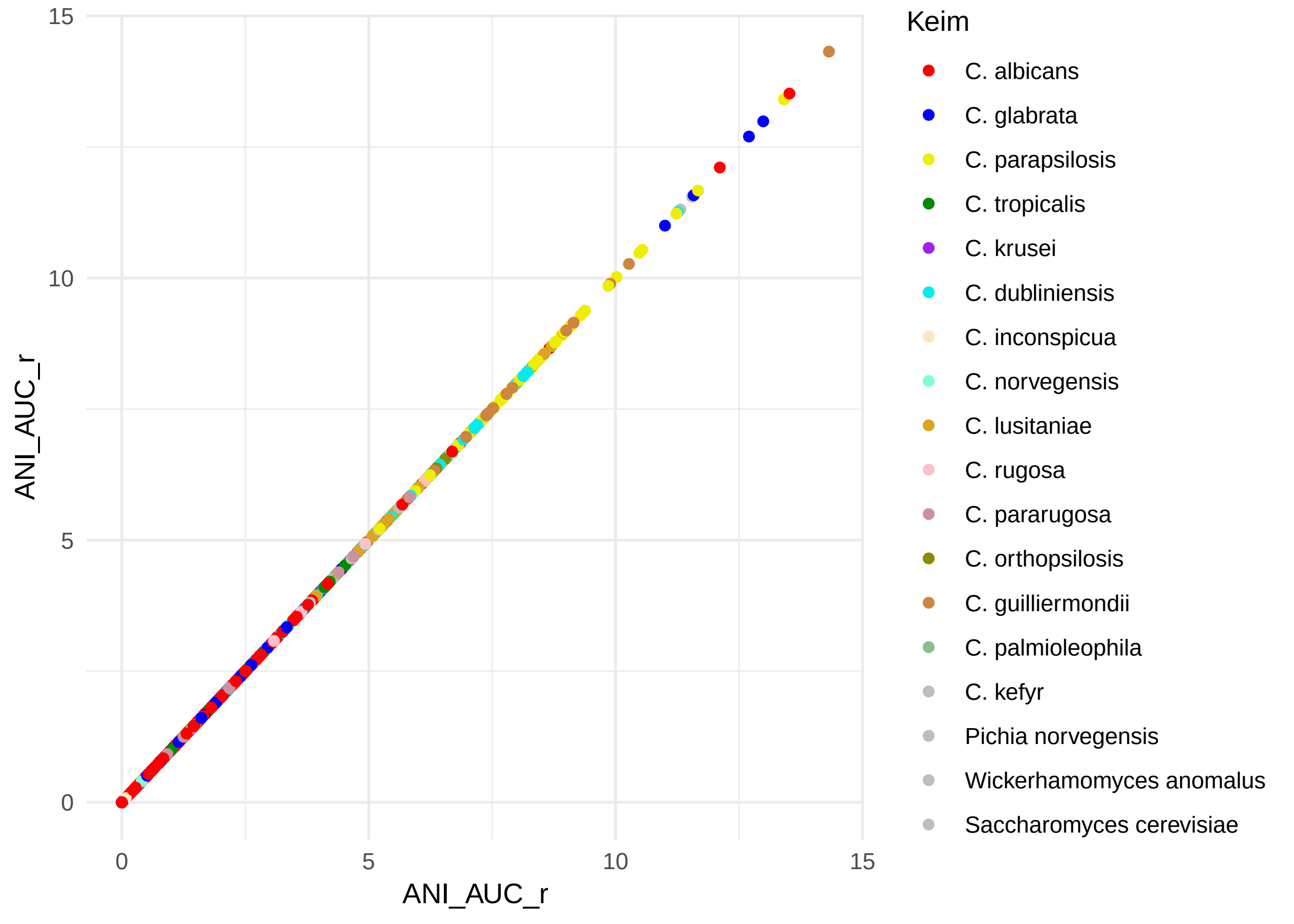
15
10
5
0
ANI_AUC_r
0
5
10
15
ANI_AUC_r
Keim
C. albicans
C. glabrata
C. parapsilosis
C. tropicalis
C. krusei
C. dubliniensis
C. inconspicua
C. norvegensis
C. lusitaniae
C. rugosa
C. pararugosa
C. orthopsilosis
C. guilliermondii
C. palmioleophila
C. kefyr
Pichia norvegensis
Wickerhamomyces anomalus
Saccharomyces cerevisiae

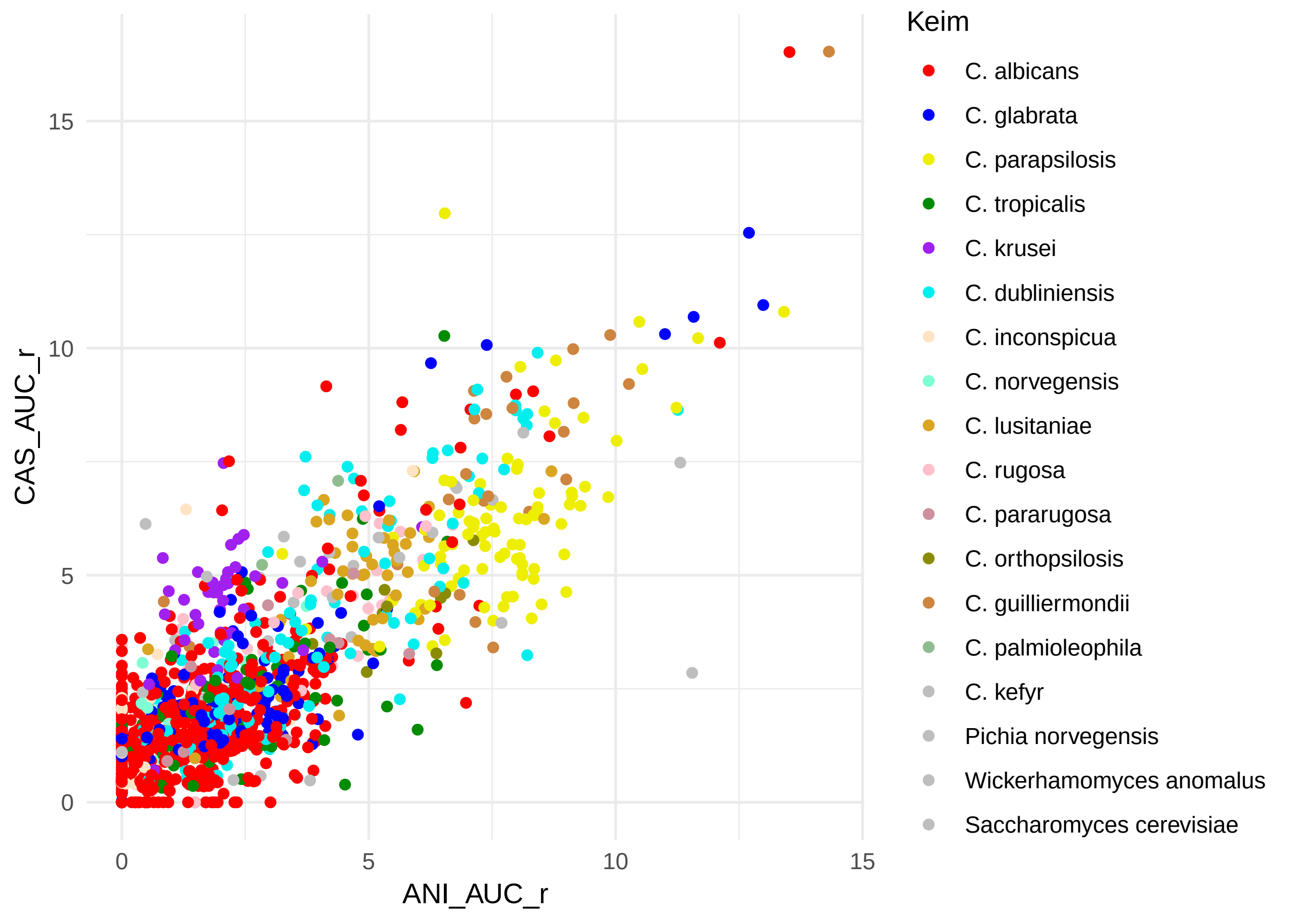
Keim
C. albicans
C. glabrata
C. parapsilosis
C. tropicalis
C. krusei
C. dubliniensis
C. inconspicua
C. norvegensis
C. lusitaniae
C. rugosa
C. pararugosa
C. orthopsilosis
C. guilliermondii
C. palmioleophila
C. kefyr
Pichia norvegensis
Wickerhamomyces anomalus
Saccharomyces cerevisiae
CAS_AUC_r
ANI_AUC_r
0
5
10
15
0
5
10
15

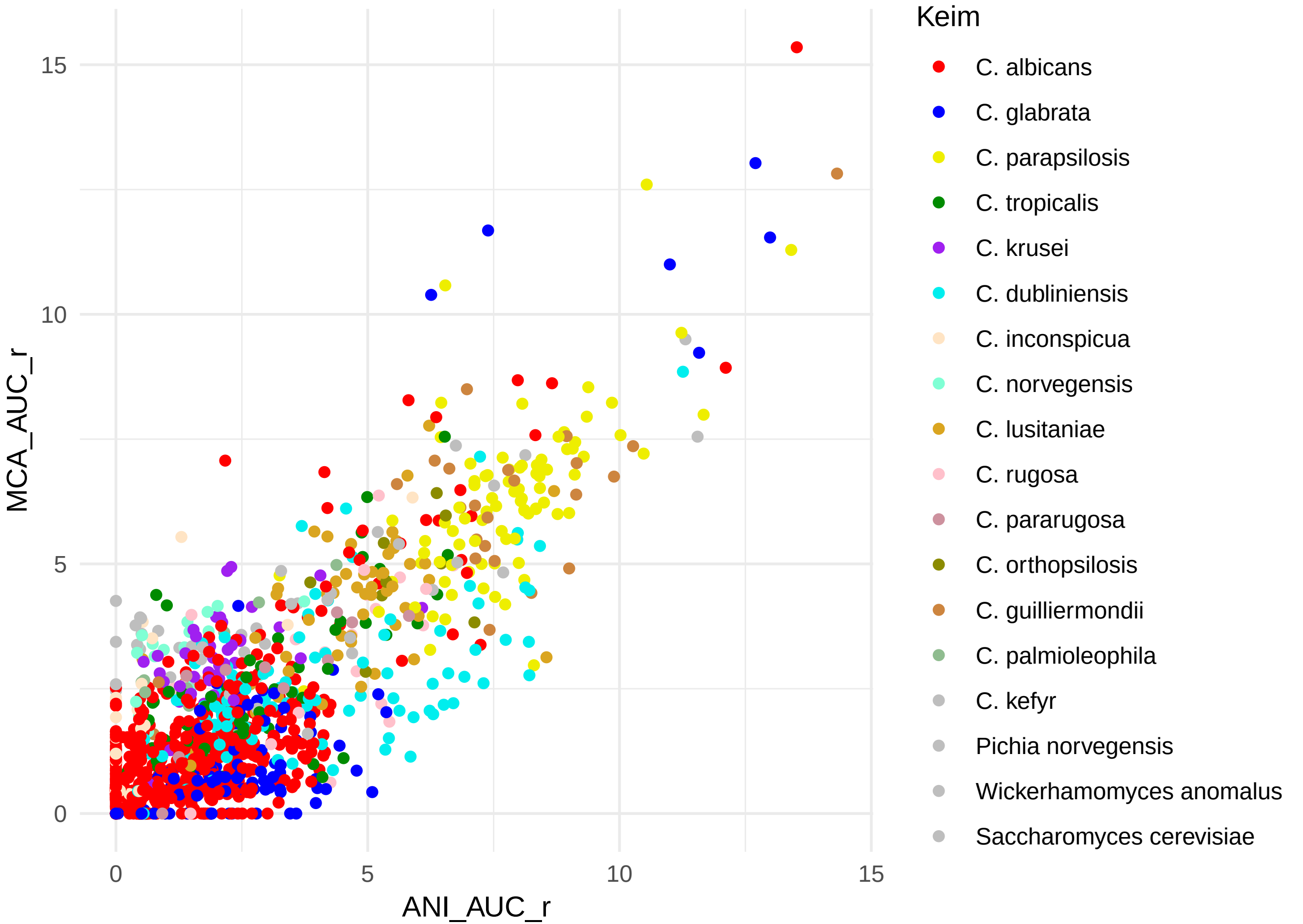
Keim
C. albicans
C. glabrata
C. parapsilosis
C. tropicalis
C. krusei
C. dubliniensis
C. inconspicua
C. norvegensis
C. lusitaniae
C. rugosa
C. pararugosa
C. orthopsilosis
C. guilliermondii
C. palmioleophila
C. kefyr
Pichia norvegensis
Wickerhamomyces anomalus
Saccharomyces cerevisiae
MCA_AUC_r
ANI_AUC_r
0
5
10
15

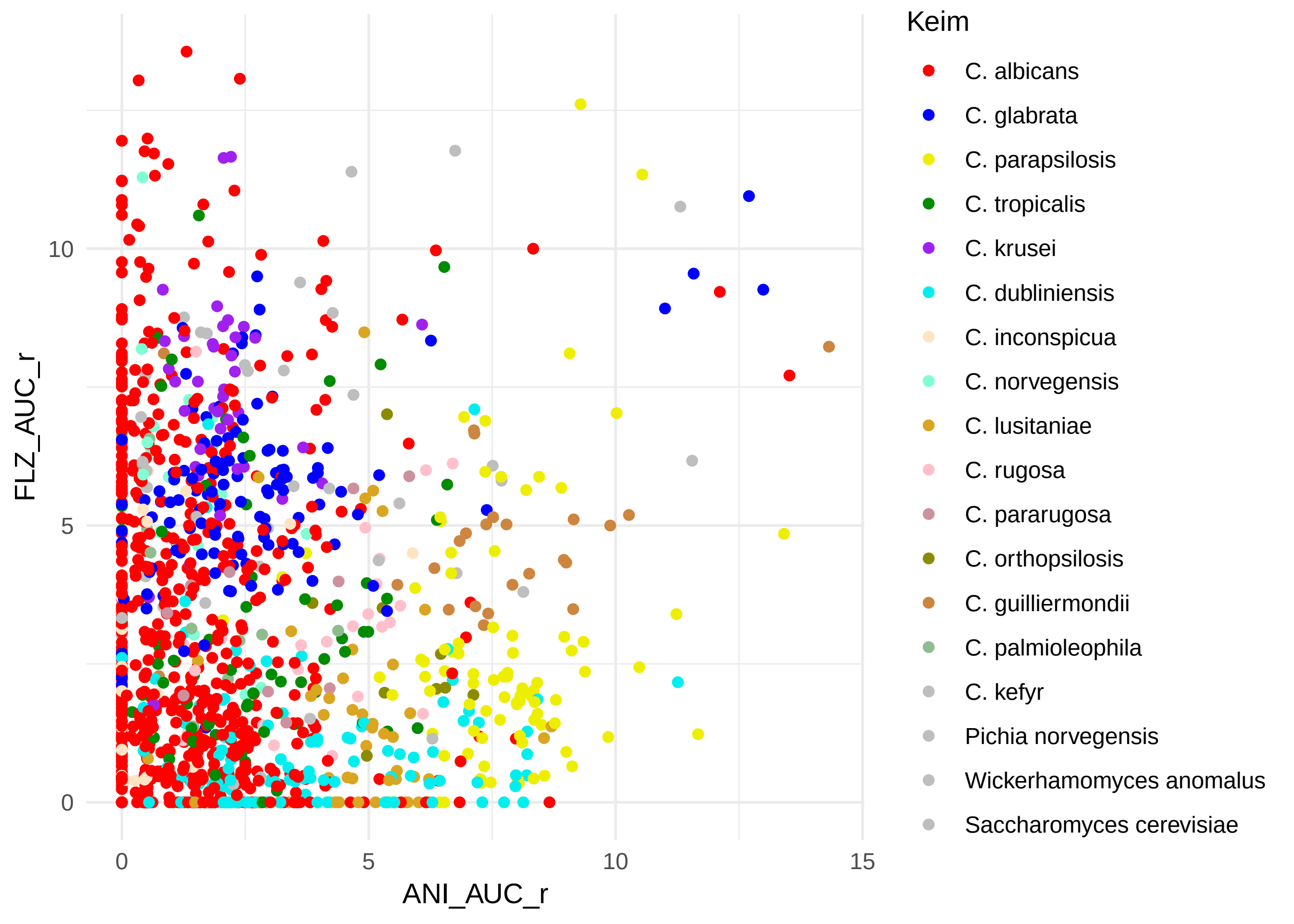
Keim
C. albicans
C. glabrata
C. parapsilosis
C. tropicalis
C. krusei
C. dubliniensis
C. inconspicua
C. norvegensis
C. lusitaniae
C. rugosa
C. pararugosa
C. orthopsilosis
C. guilliermondii
C. palmioleophila
C. kefyr
Pichia norvegensis
Wickerhamomyces anomalus
Saccharomyces cerevisiae
FLZ_AUC_r
ANI_AUC_r
0
5
10
15

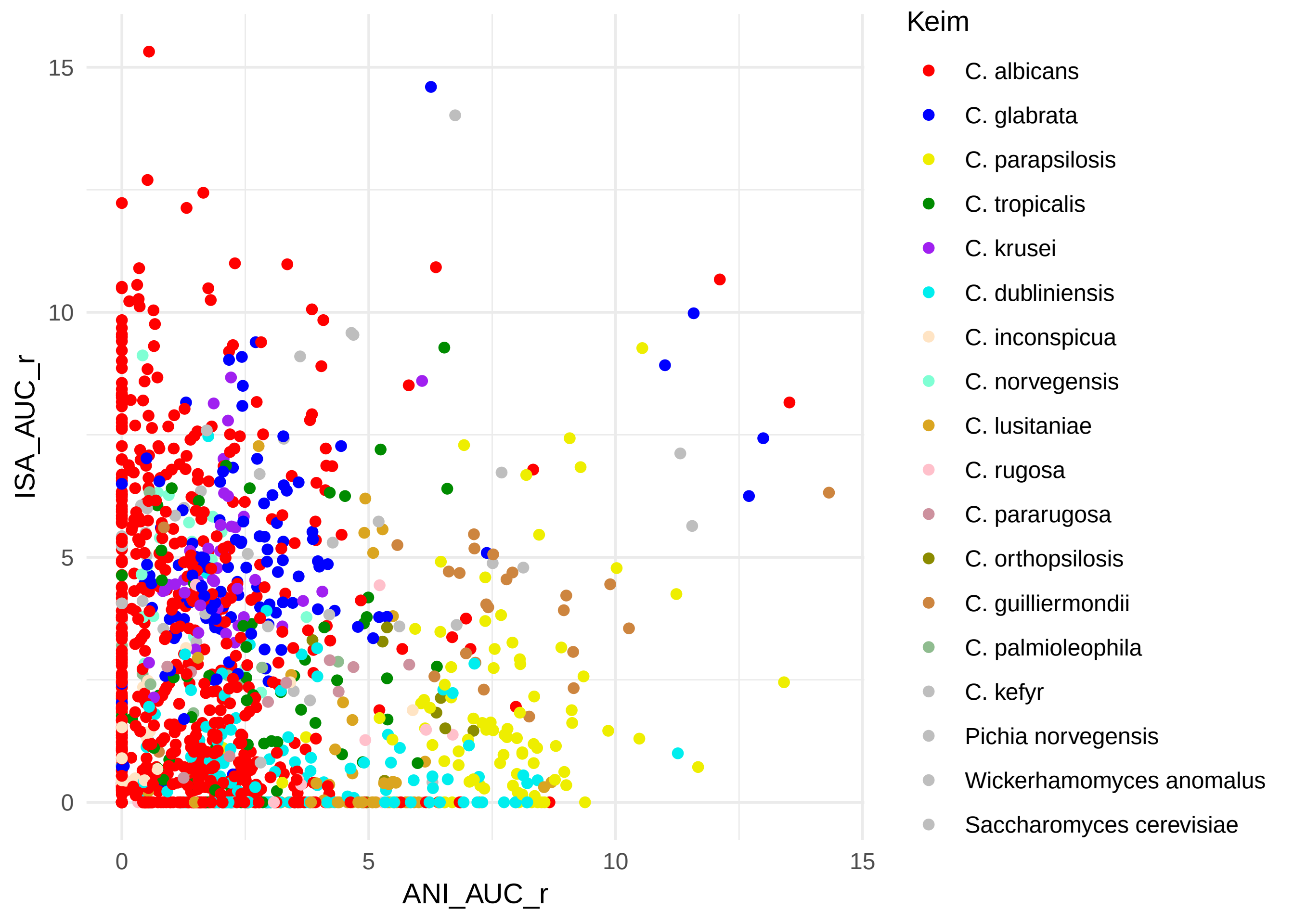

Keim
C. albicans
C. glabrata
C. parapsilosis
C. tropicalis
C. krusei
C. dubliniensis
C. inconspicua
C. norvegensis
C. lusitaniae
C. rugosa
C. pararugosa
C. orthopsilosis
C. guilliermondii
C. palmioleophila
C. kefyr
Pichia norvegensis
Wickerhamomyces anomalus
Saccharomyces cerevisiae
ISA_AUC_r
ANI_AUC_r
0
5
10
15

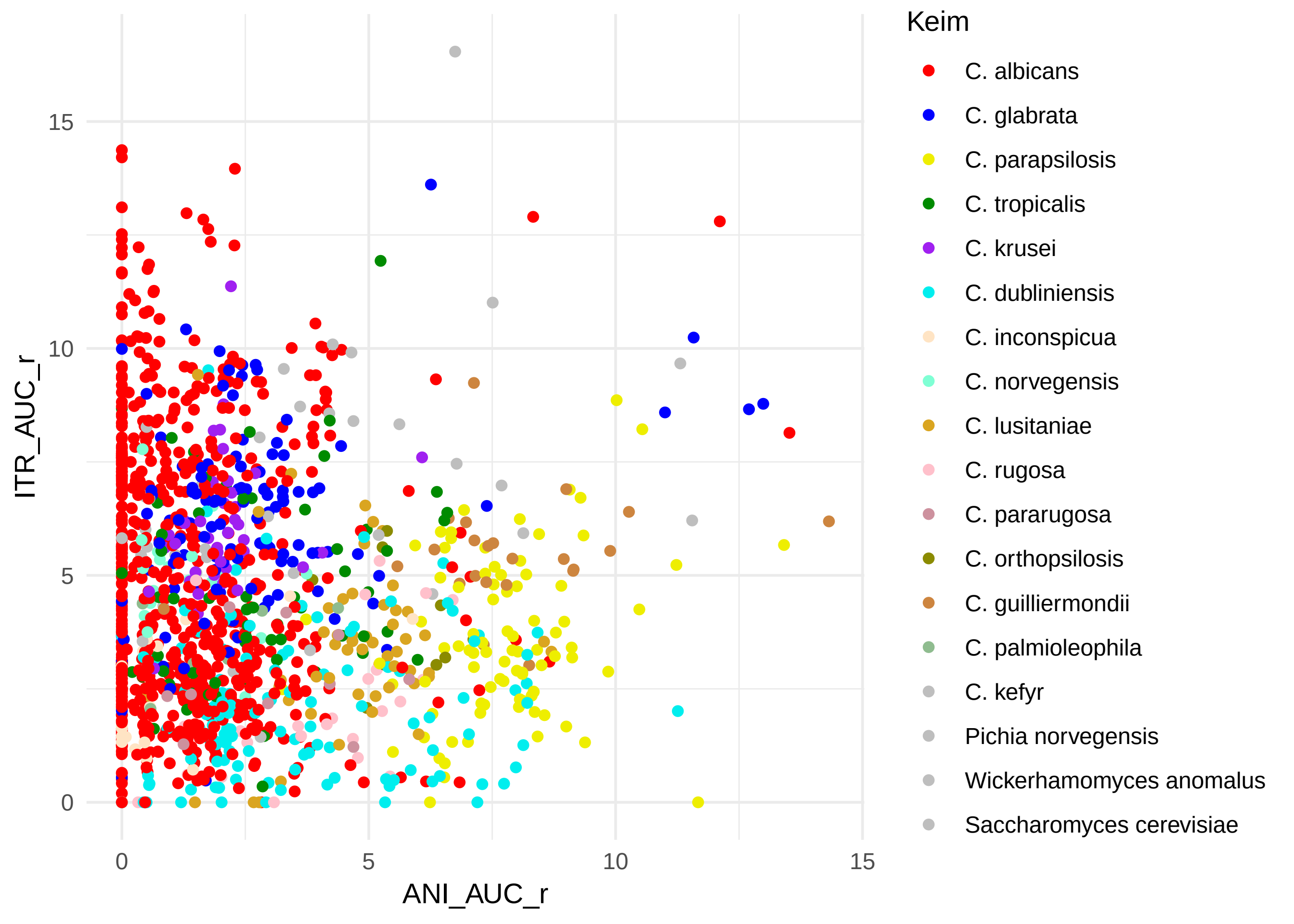
Keim
C. albicans
C. glabrata
C. parapsilosis
C. tropicalis
C. krusei
C. dubliniensis
C. inconspicua
C. norvegensis
C. lusitaniae
C. rugosa
C. pararugosa
C. orthopsilosis
C. guilliermondii
C. palmioleophila
C. kefyr
Pichia norvegensis
Wickerhamomyces anomalus
Saccharomyces cerevisiae
ITR_AUC_r
ANI_AUC_r
0
5
10
15

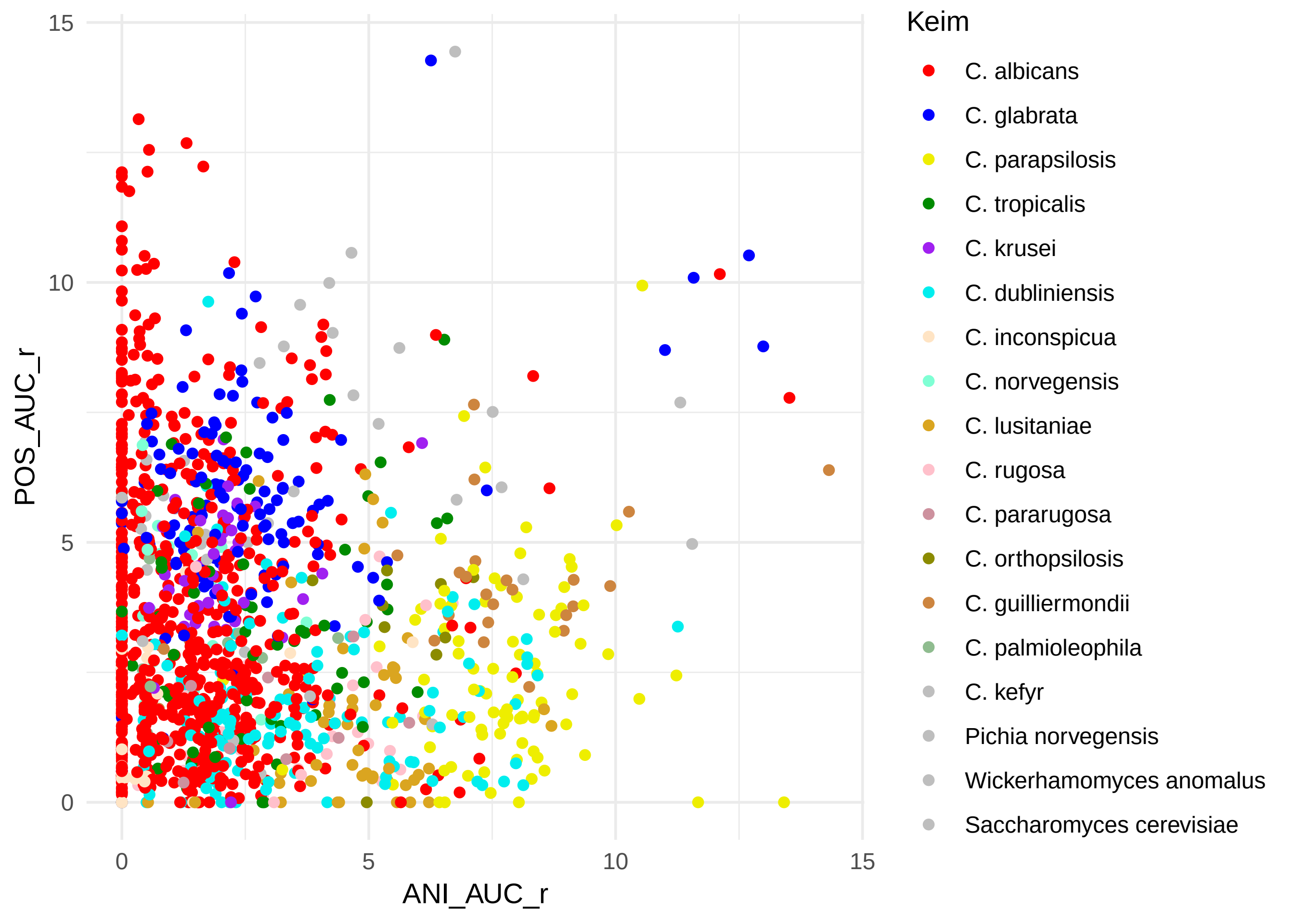

15
10
5
0
POS_AUC_r
0
5
10
15
ANI_AUC_r
Keim
C. albicans
C. glabrata
C. parapsilosis
C. tropicalis
C. krusei
C. dubliniensis
C. inconspicua
C. norvegensis
C. lusitaniae
C. rugosa
C. pararugosa
C. orthopsilosis
C. guilliermondii
C. palmioleophila
C. kefyr
Pichia norvegensis
Wickerhamomyces anomalus
Saccharomyces cerevisiae

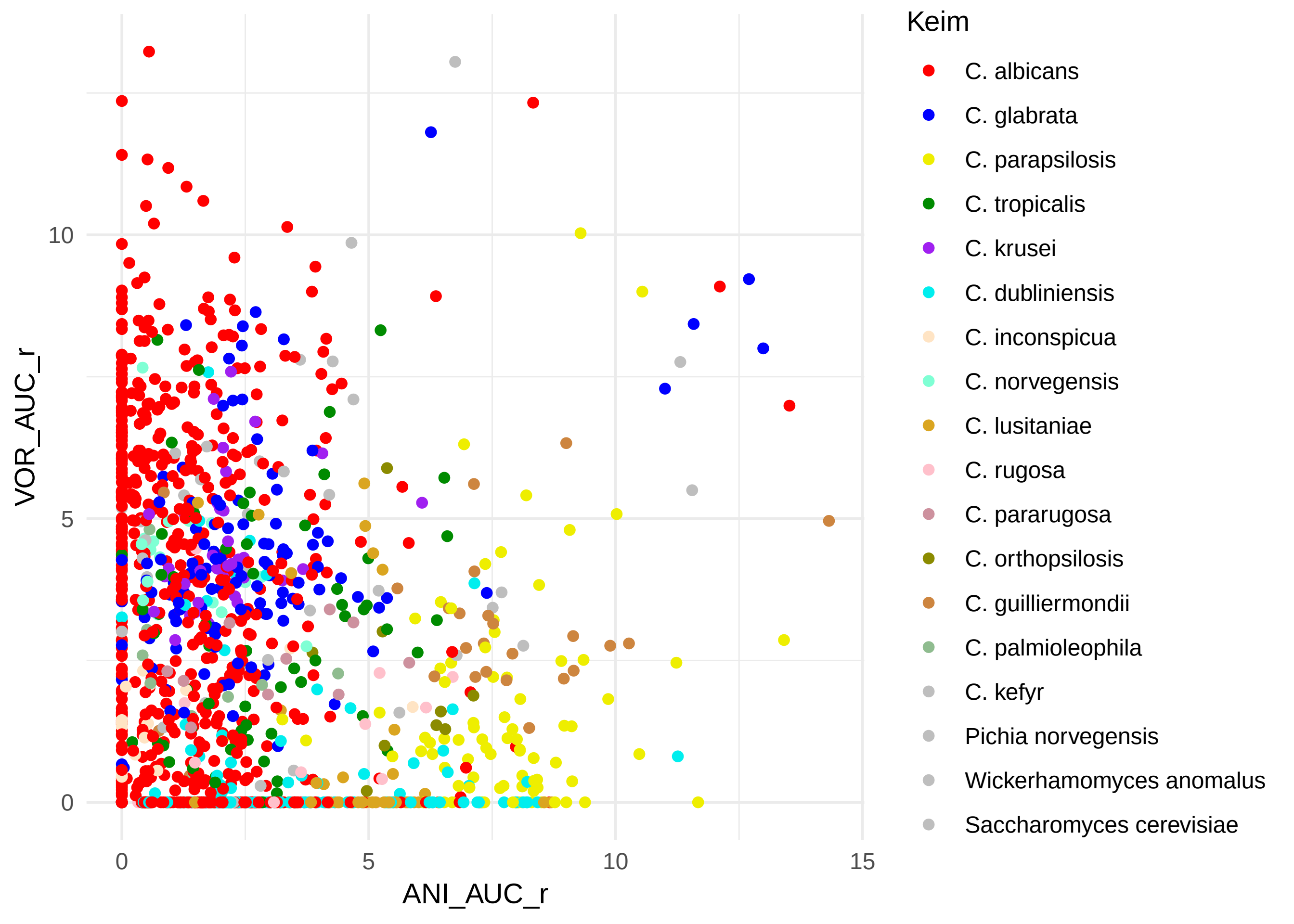

Keim
C. albicans
C. glabrata
C. parapsilosis
C. tropicalis
C. krusei
C. dubliniensis
C. inconspicua
C. norvegensis
C. lusitaniae
C. rugosa
C. pararugosa
C. orthopsilosis
C. guilliermondii
C. palmioleophila
C. kefyr
Pichia norvegensis
Wickerhamomyces anomalus
Saccharomyces cerevisiae
VOR_AUC_r
ANI_AUC_r
0
5
10
15

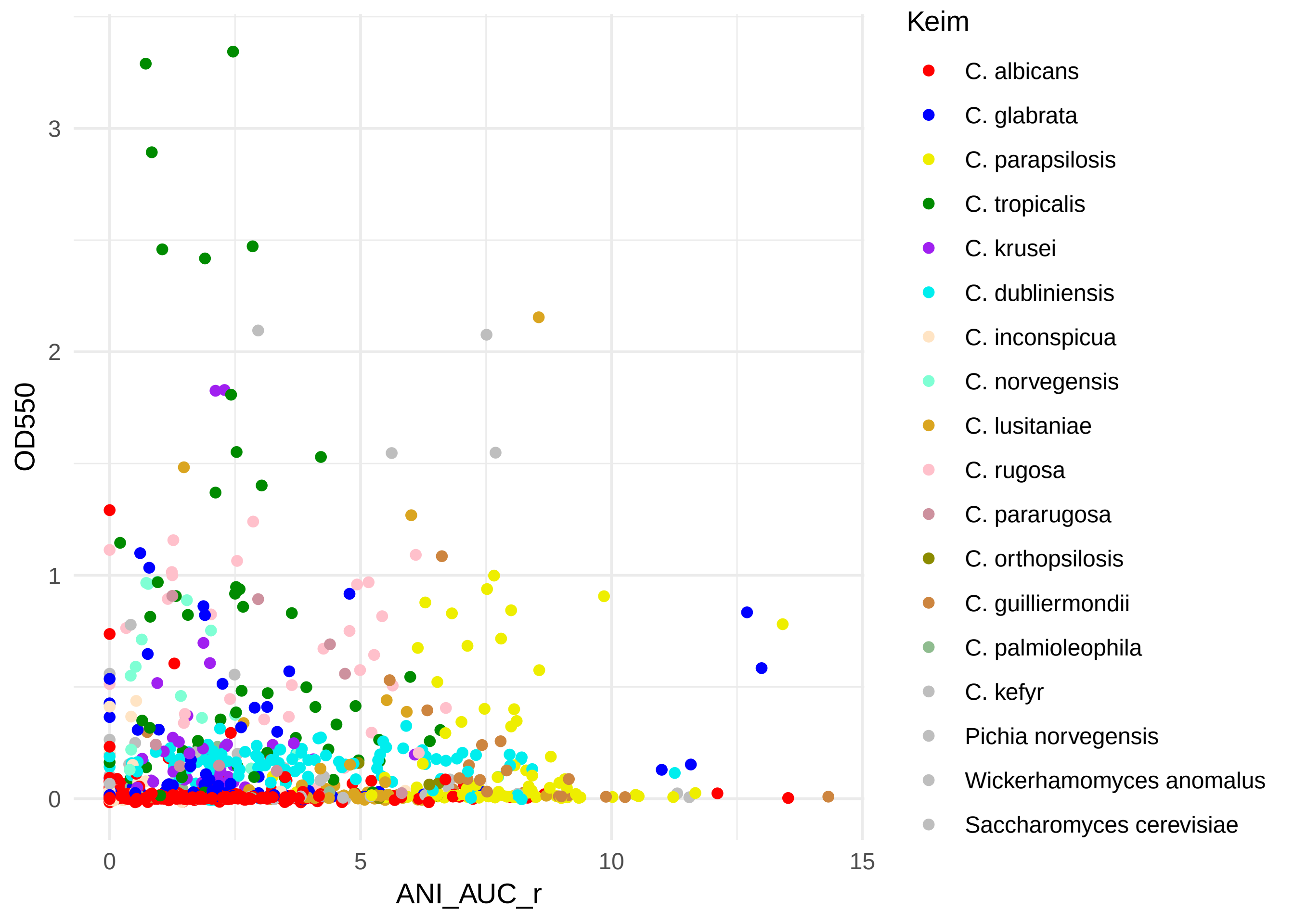
Keim
C. albicans
C. glabrata
C. parapsilosis
C. tropicalis
C. krusei
C. dubliniensis
C. inconspicua
C. norvegensis
C. lusitaniae
C. rugosa
C. pararugosa
C. orthopsilosis
C. guilliermondii
C. palmioleophila
C. kefyr
Pichia norvegensis
Wickerhamomyces anomalus
Saccharomyces cerevisiae
OD550
0
1
2
3
0
5
10
15
ANI_AUC_r

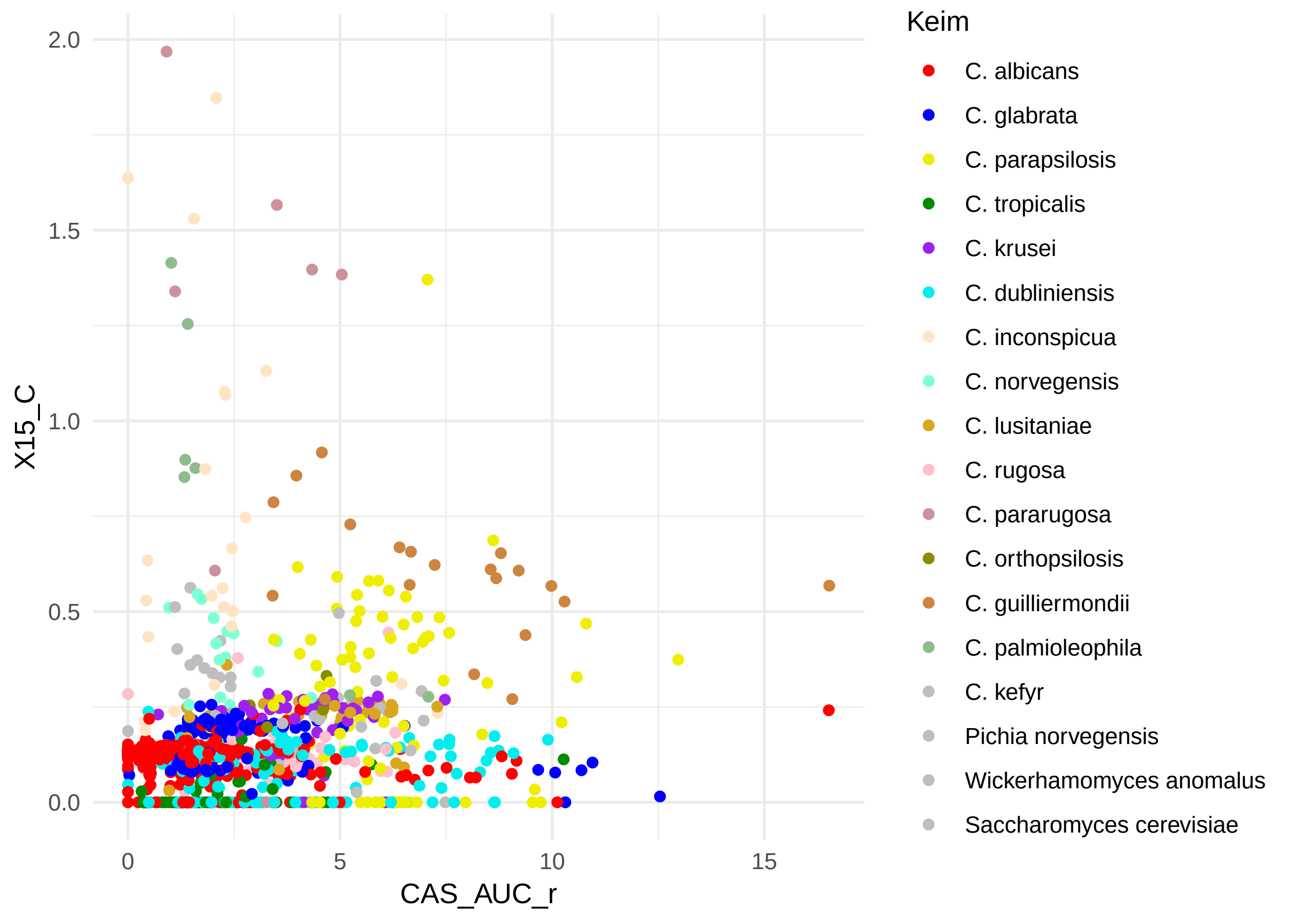

Keim
C. albicans
C. glabrata
C. parapsilosis
C. tropicalis
C. krusei
C. dubliniensis
C. inconspicua
C. norvegensis
C. lusitaniae
C. rugosa
C. pararugosa
C. orthopsilosis
C. guilliermondii
C. palmioleophila
C. kefyr
Pichia norvegensis
Wickerhamomyces anomalus
Saccharomyces cerevisiae
X15_C
2.0
1.5
1.0
0.5
0.0
0
5
10
15
CAS_AUC_r

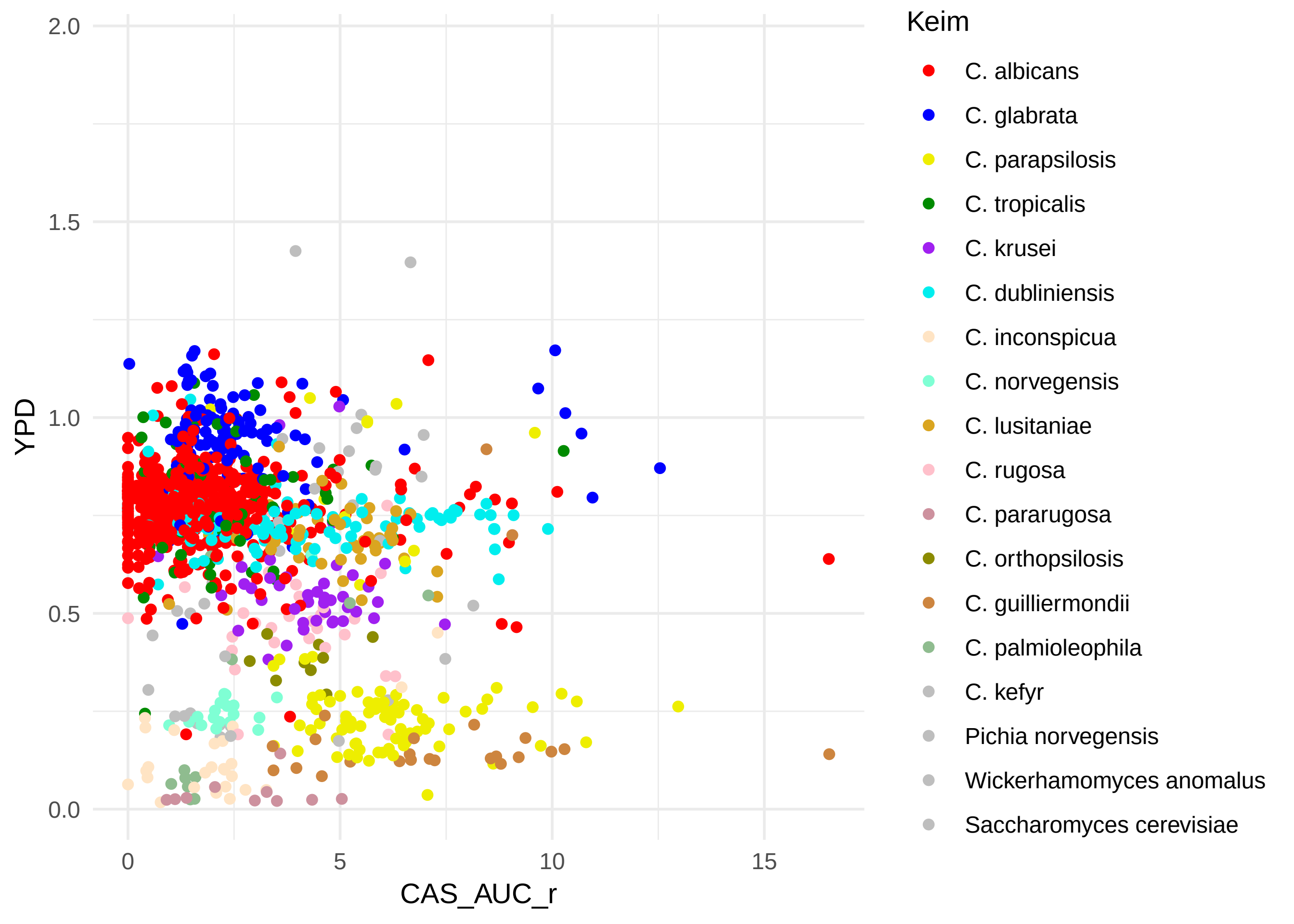

YPD
2.0
1.5
1.0
0.5
0.0
0
5
10
15
CAS_AUC_r
Keim
C. albicans
C. glabrata
C. parapsilosis
C. tropicalis
C. krusei
C. dubliniensis
C. inconspicua
C. norvegensis
C. lusitaniae
C. rugosa
C. pararugosa
C. orthopsilosis
C. guilliermondii
C. palmioleophila
C. kefyr
Pichia norvegensis
Wickerhamomyces anomalus
Saccharomyces cerevisiae

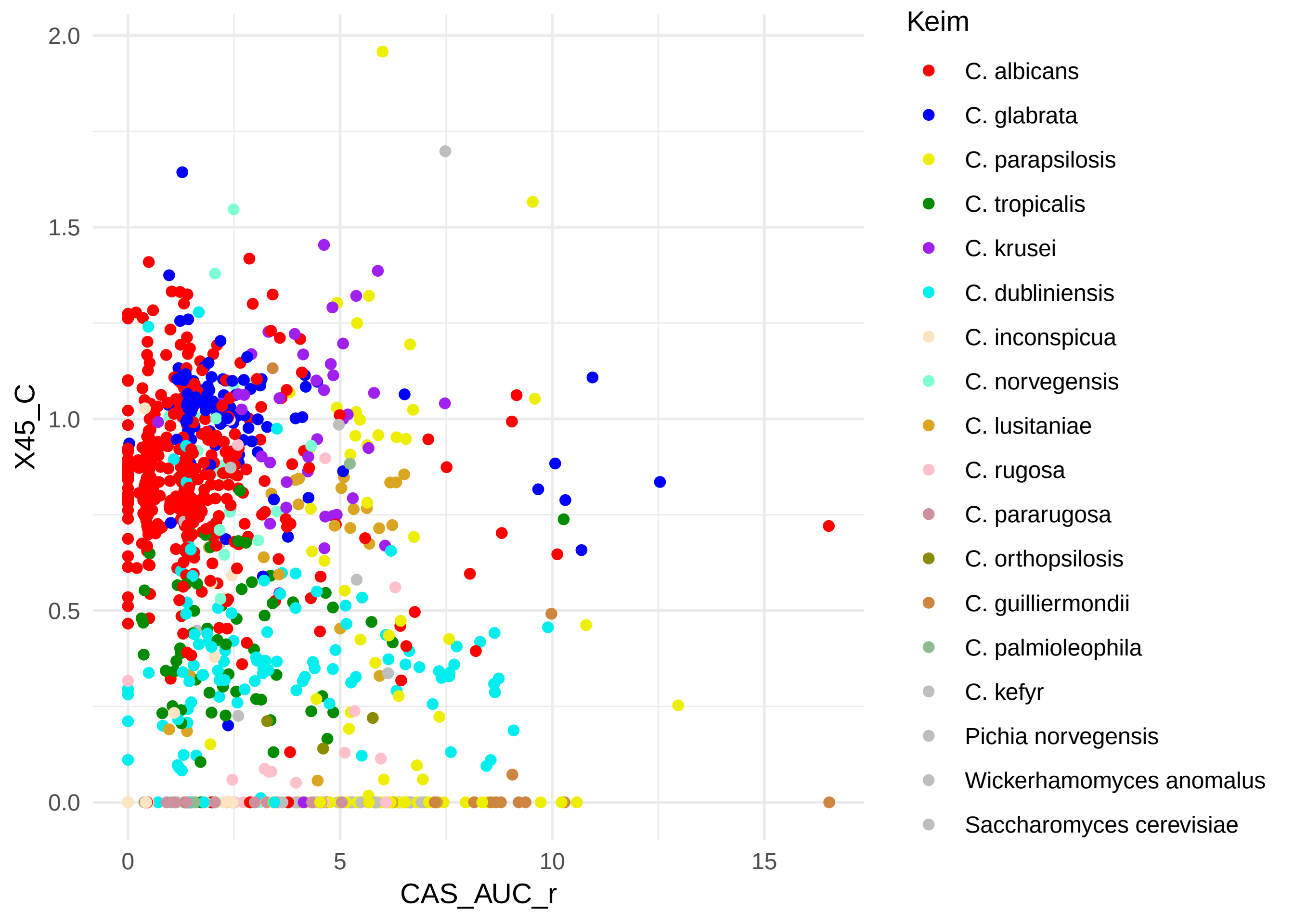

2.0
1.5
1.0
0.5
0.0
X45_C
0
5
10
15
CAS_AUC_r
Keim
C. albicans
C. glabrata
C. parapsilosis
C. tropicalis
C. krusei
C. dubliniensis
C. inconspicua
C. norvegensis
C. lusitaniae
C. rugosa
C. pararugosa
C. orthopsilosis
C. guilliermondii
C. palmioleophila
C. kefyr
Pichia norvegensis
Wickerhamomyces anomalus
Saccharomyces cerevisiae

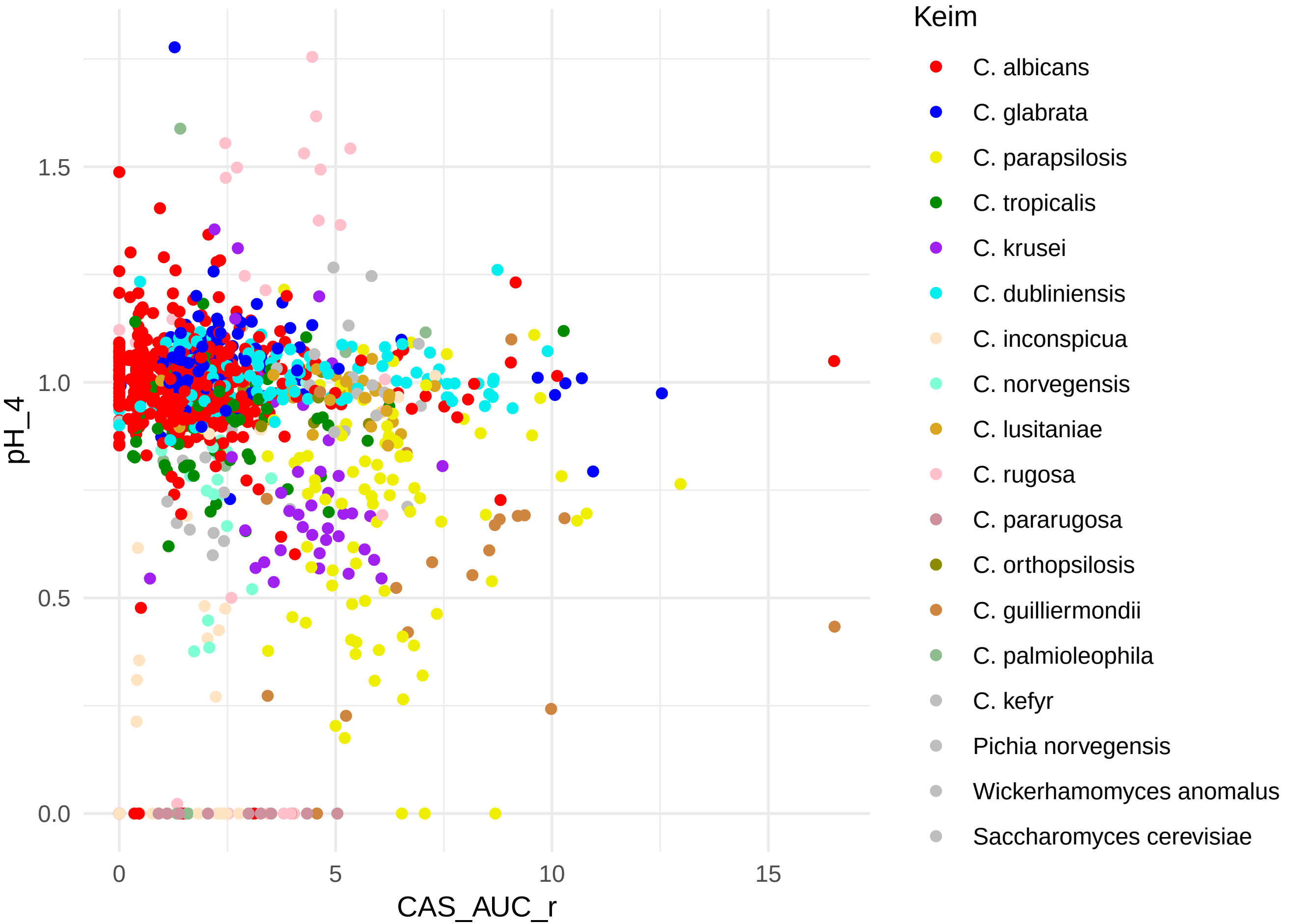

Keim
C. albicans
C. glabrata
C. parapsilosis
C. tropicalis
C. krusei
C. dubliniensis
C. inconspicua
C. norvegensis
C. lusitaniae
C. rugosa
C. pararugosa
C. orthopsilosis
C. guilliermondii
C. palmioleophila
C. kefyr
Pichia norvegensis
Wickerhamomyces anomalus
Saccharomyces cerevisiae
pH_4
1.5
1.0
0.5
0.0
0
5
10
15
CAS_AUC_r

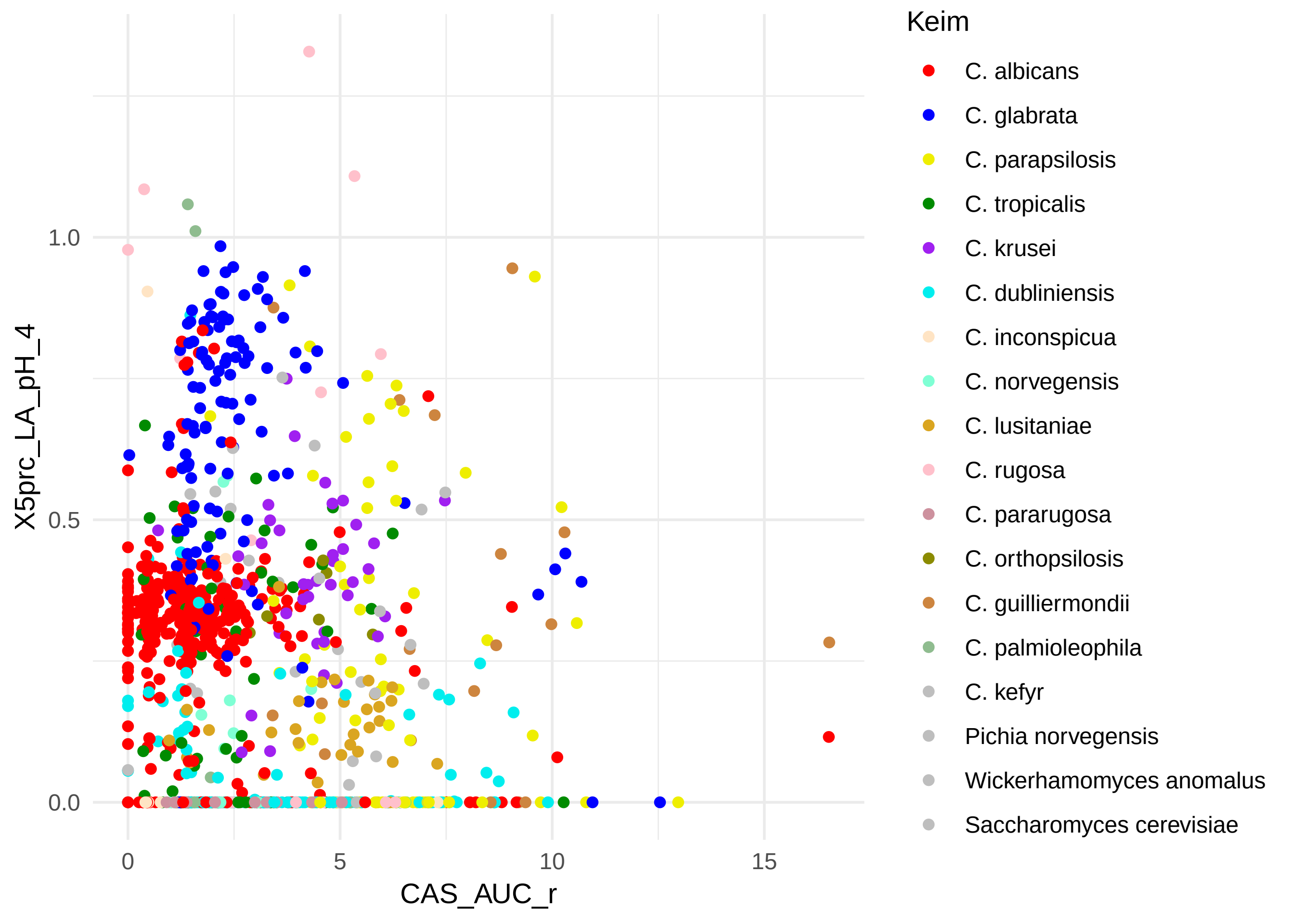
X5prc_LA_pH_4
1.0
0.5
0.0
0
5
10
15
CAS_AUC_r
Keim
C. albicans
C. glabrata
C. parapsilosis
C. tropicalis
C. krusei
C. dubliniensis
C. inconspicua
C. norvegensis
C. lusitaniae
C. rugosa
C. pararugosa
C. orthopsilosis
C. guilliermondii
C. palmioleophila
C. kefyr
Pichia norvegensis
Wickerhamomyces anomalus
Saccharomyces cerevisiae

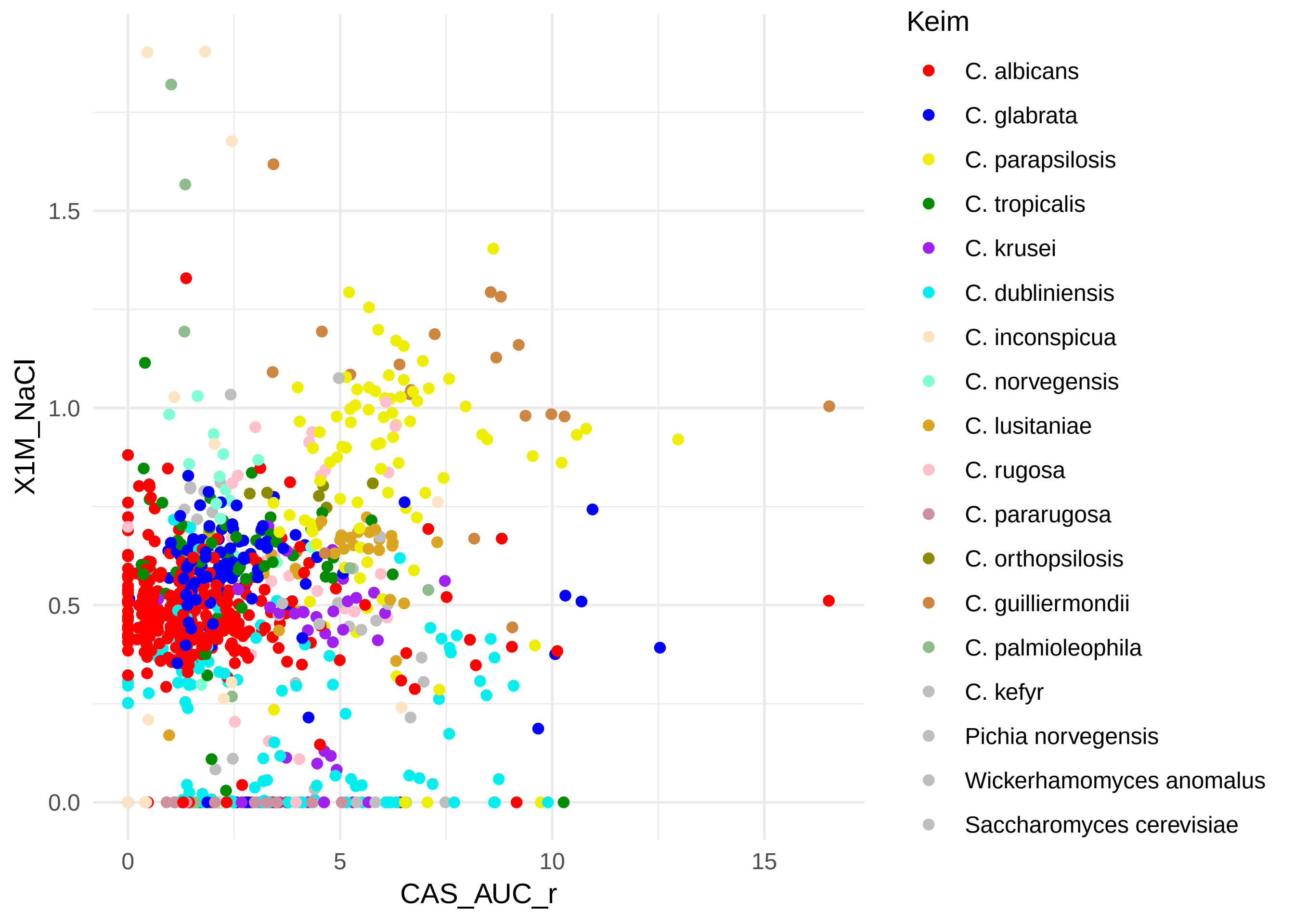
Keim
C. albicans
C. glabrata
C. parapsilosis
C. tropicalis
C. krusei
C. dubliniensis
C. inconspicua
C. norvegensis
C. lusitaniae
C. rugosa
C. pararugosa
C. orthopsilosis
C. guilliermondii
C. palmioleophila
C. kefyr
Pichia norvegensis
Wickerhamomyces anomalus
Saccharomyces cerevisiae
X1M_NaCl
0.0
0.5
1.0
1.5
0
5
10
15
CAS_AUC_r

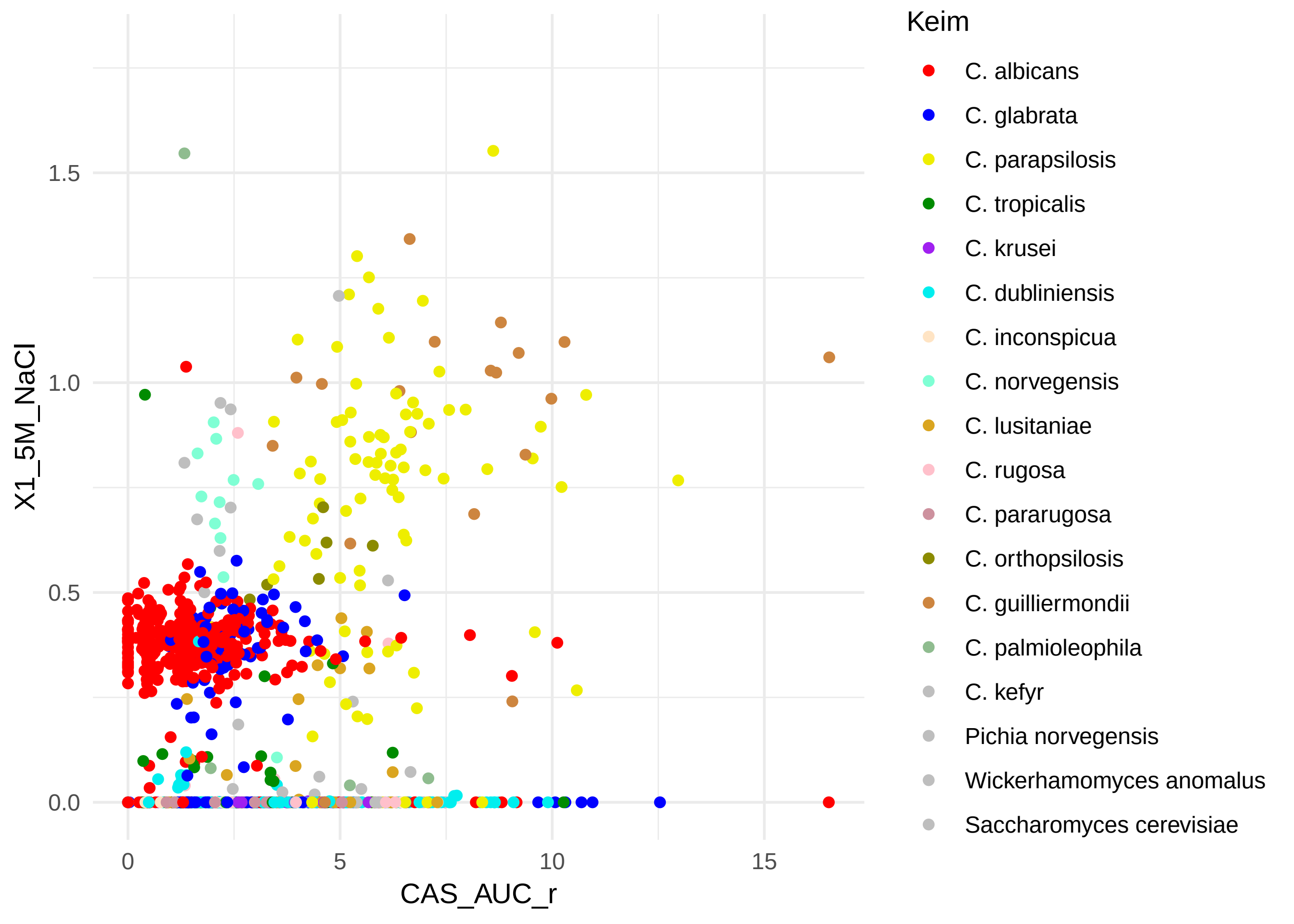
Keim
C. albicans
C. glabrata
C. parapsilosis
C. tropicalis
C. krusei
C. dubliniensis
C. inconspicua
C. norvegensis
C. lusitaniae
C. rugosa
C. pararugosa
C. orthopsilosis
C. guilliermondii
C. palmioleophila
C. kefyr
Pichia norvegensis
Wickerhamomyces anomalus
Saccharomyces cerevisiae
X1_5M_NaCl
1.5
1.0
0.5
0.0
0
5
10
15
CAS_AUC_r

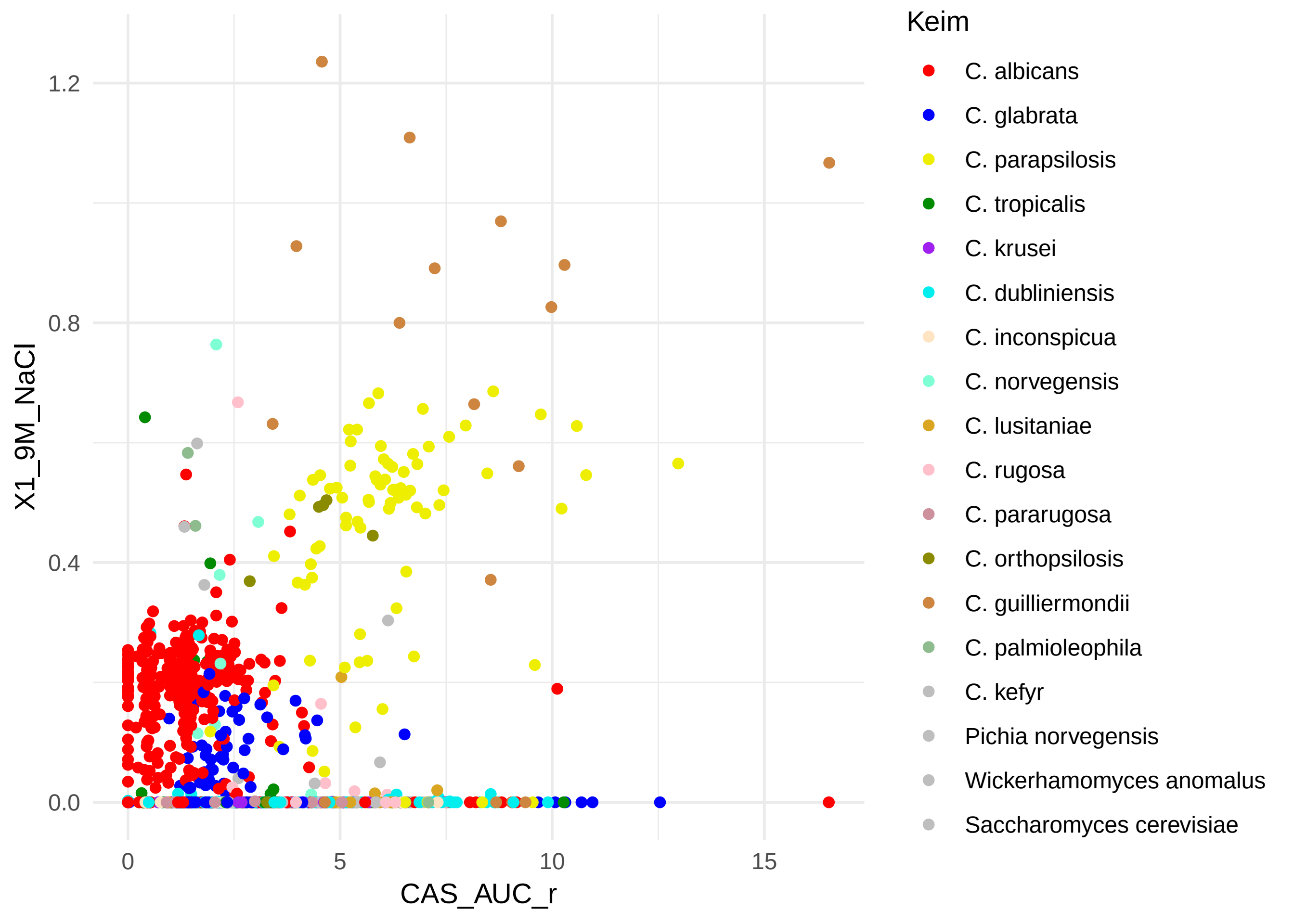

Keim
C. albicans
C. glabrata
C. parapsilosis
C. tropicalis
C. krusei
C. dubliniensis
C. inconspicua
C. norvegensis
C. lusitaniae
C. rugosa
C. pararugosa
C. orthopsilosis
C. guilliermondii
C. palmioleophila
C. kefyr
Pichia norvegensis
Wickerhamomyces anomalus
Saccharomyces cerevisiae
X1_9M_NaCl
1.2
0.8
0.4
0.0
0
5
10
15
CAS_AUC_r

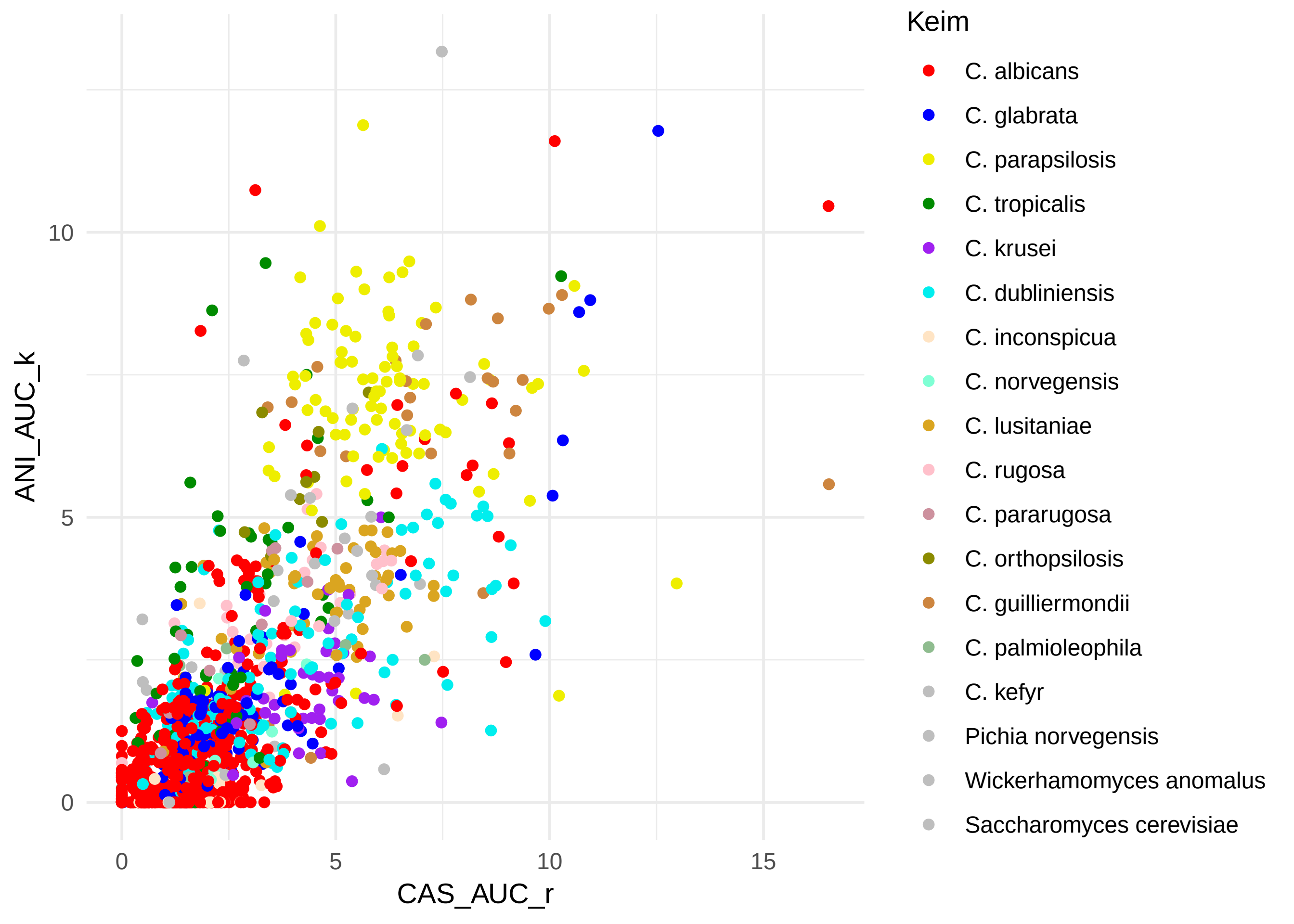
Keim
C. albicans
C. glabrata
C. parapsilosis
C. tropicalis
C. krusei
C. dubliniensis
C. inconspicua
C. norvegensis
C. lusitaniae
C. rugosa
C. pararugosa
C. orthopsilosis
C. guilliermondii
C. palmioleophila
C. kefyr
Pichia norvegensis
Wickerhamomyces anomalus
Saccharomyces cerevisiae
ANI_AUC_k
CAS_AUC_r
0
5
10
15

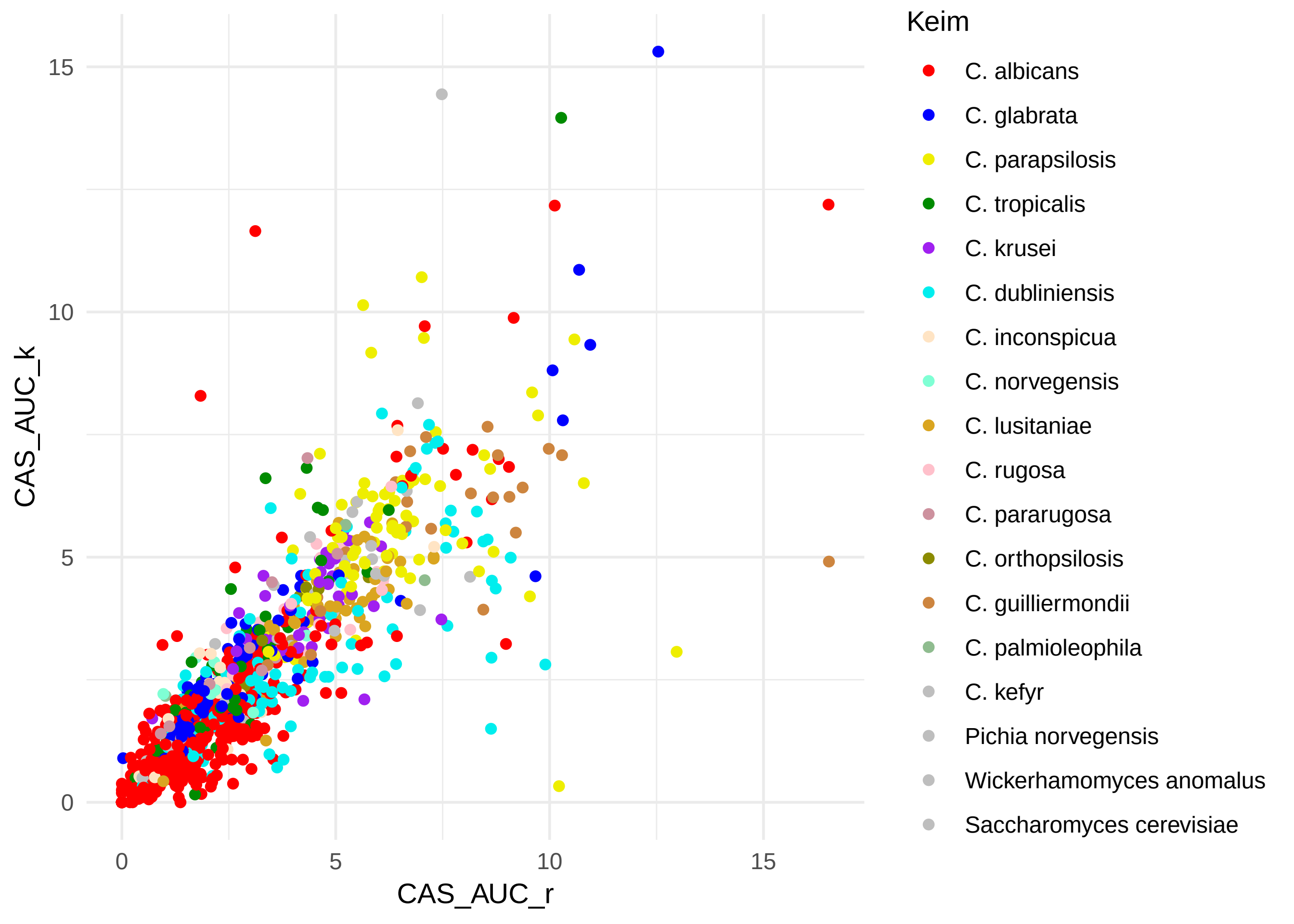

Keim
C. albicans
C. glabrata
C. parapsilosis
C. tropicalis
C. krusei
C. dubliniensis
C. inconspicua
C. norvegensis
C. lusitaniae
C. rugosa
C. pararugosa
C. orthopsilosis
C. guilliermondii
C. palmioleophila
C. kefyr
Pichia norvegensis
Wickerhamomyces anomalus
Saccharomyces cerevisiae
CAS_AUC_k
CAS_AUC_r
0
5
10
15

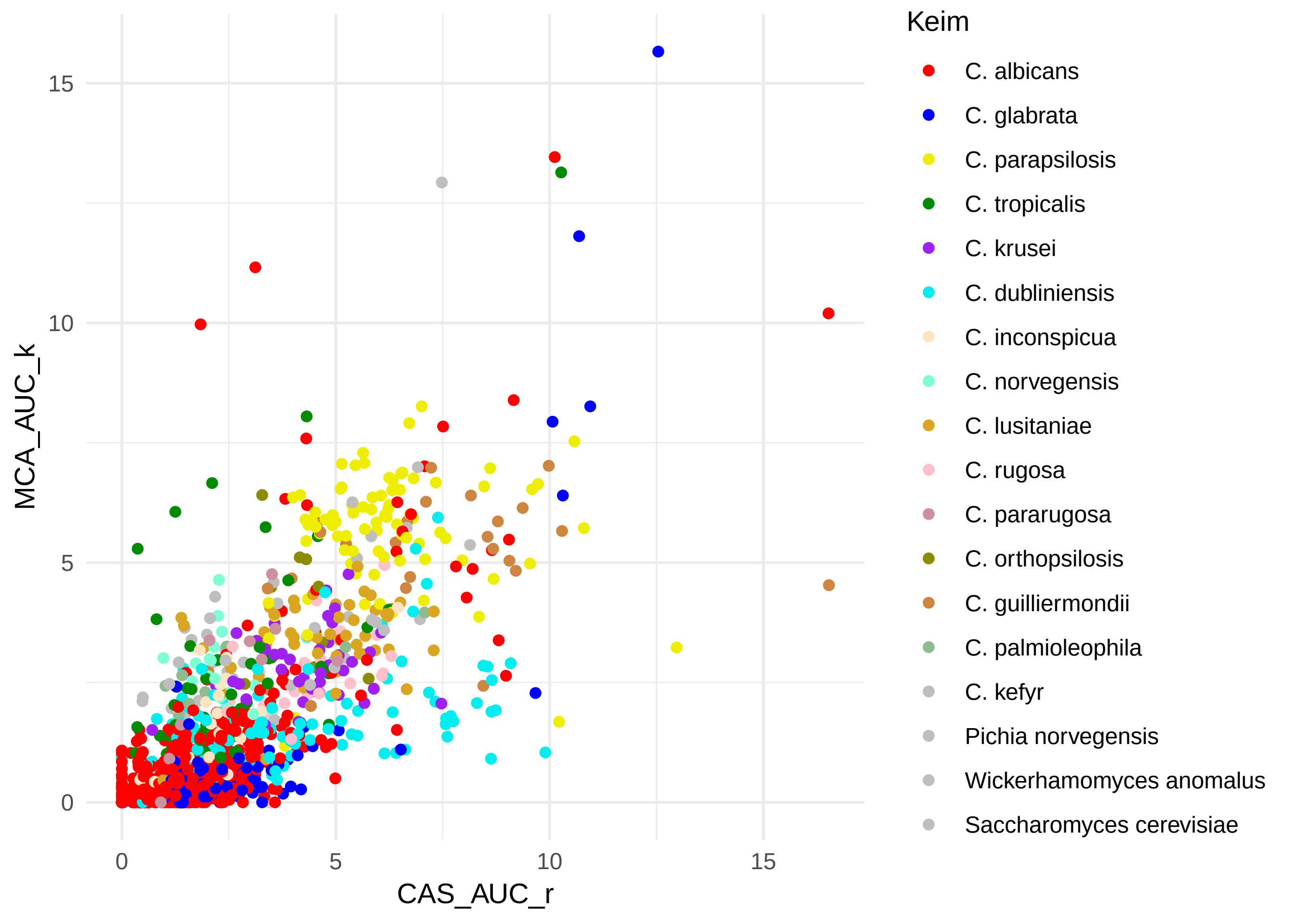
Keim
C. albicans
C. glabrata
C. parapsilosis
C. tropicalis
C. krusei
C. dubliniensis
C. inconspicua
C. norvegensis
C. lusitaniae
C. rugosa
C. pararugosa
C. orthopsilosis
C. guilliermondii
C. palmioleophila
C. kefyr
Pichia norvegensis
Wickerhamomyces anomalus
Saccharomyces cerevisiae
MCA_AUC_k
CAS_AUC_r
0
5
10
15

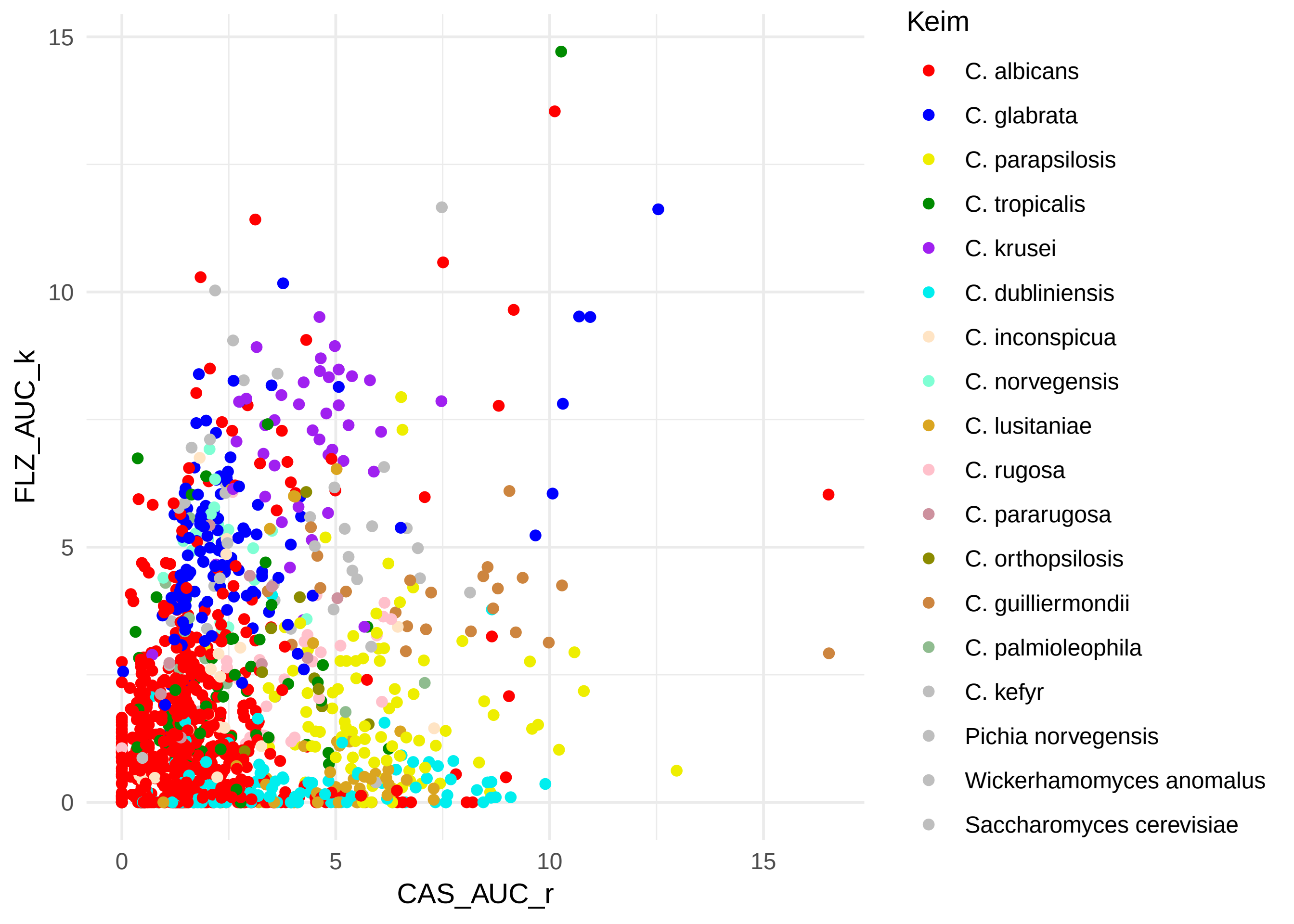
15
10
5
0
FLZ_AUC_k
0
5
10
15
CAS_AUC_r
Keim
C. albicans
C. glabrata
C. parapsilosis
C. tropicalis
C. krusei
C. dubliniensis
C. inconspicua
C. norvegensis
C. lusitaniae
C. rugosa
C. pararugosa
C. orthopsilosis
C. guilliermondii
C. palmioleophila
C. kefyr
Pichia norvegensis
Wickerhamomyces anomalus
Saccharomyces cerevisiae

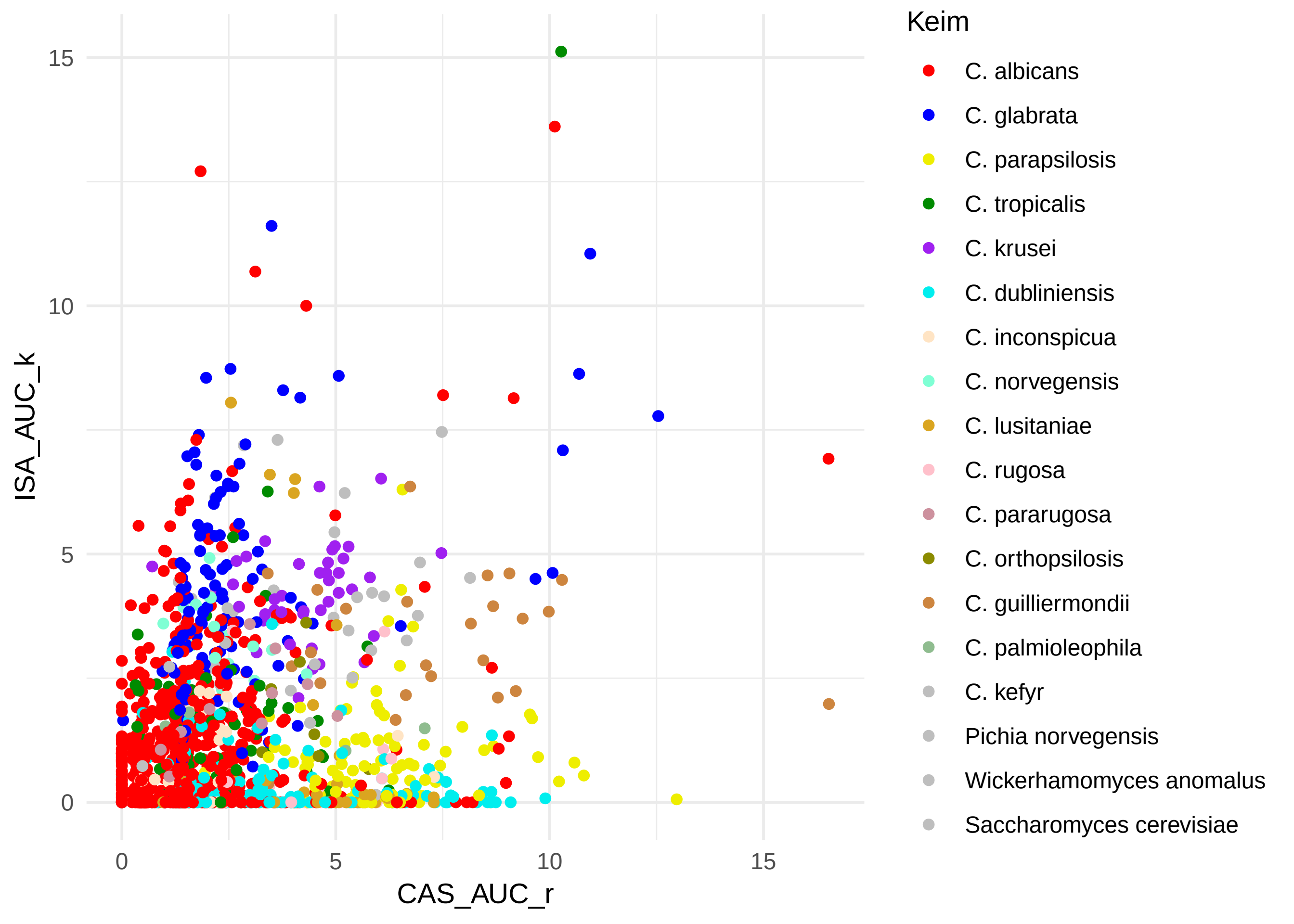

Keim
C. albicans
C. glabrata
C. parapsilosis
C. tropicalis
C. krusei
C. dubliniensis
C. inconspicua
C. norvegensis
C. lusitaniae
C. rugosa
C. pararugosa
C. orthopsilosis
C. guilliermondii
C. palmioleophila
C. kefyr
Pichia norvegensis
Wickerhamomyces anomalus
Saccharomyces cerevisiae
ISA_AUC_k
CAS_AUC_r
0
5
10
15

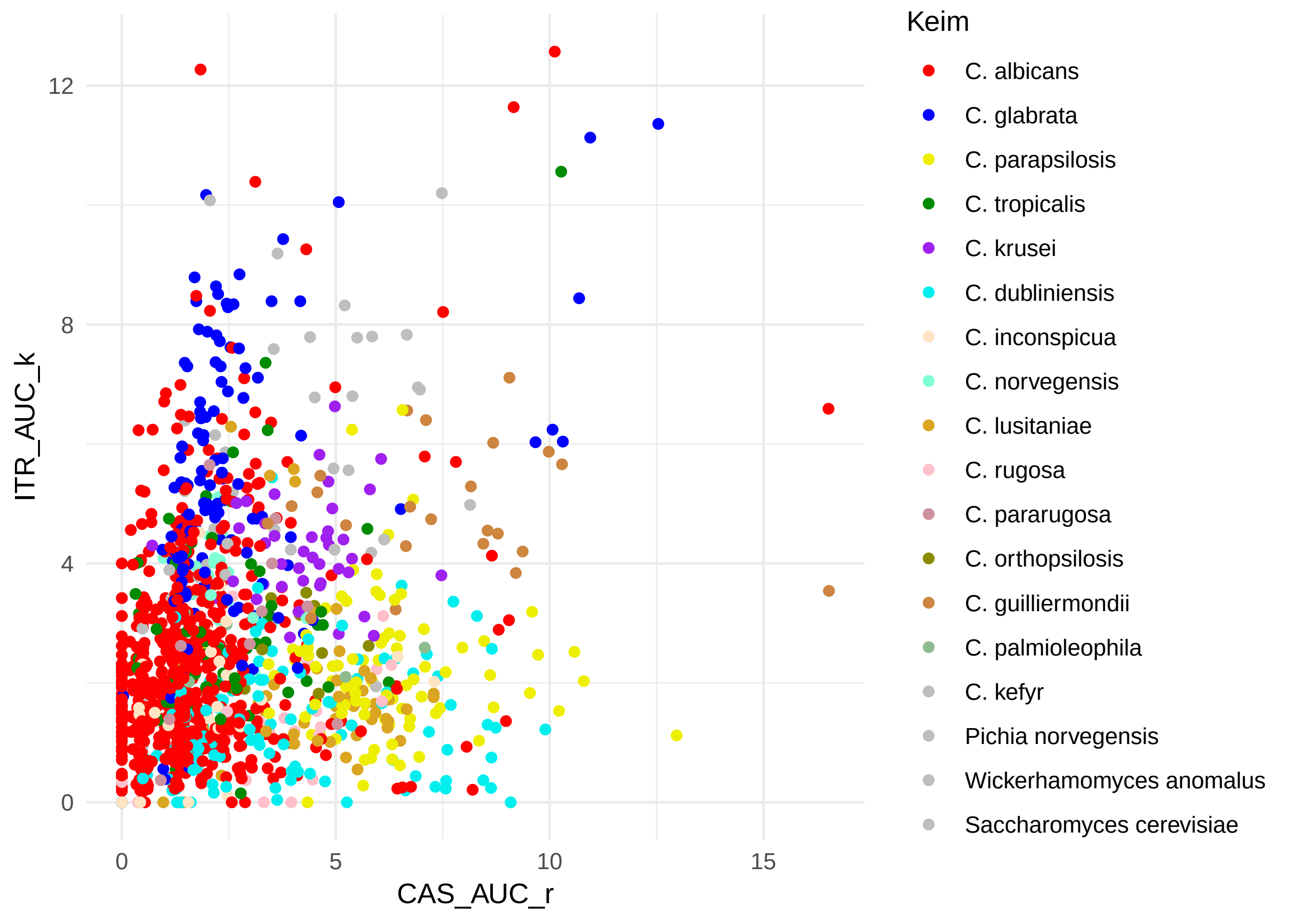

Keim
C. albicans
C. glabrata
C. parapsilosis
C. tropicalis
C. krusei
C. dubliniensis
C. inconspicua
C. norvegensis
C. lusitaniae
C. rugosa
C. pararugosa
C. orthopsilosis
C. guilliermondii
C. palmioleophila
C. kefyr
Pichia norvegensis
Wickerhamomyces anomalus
Saccharomyces cerevisiae
ITR_AUC_k
CAS_AUC_r
0
4
8
12
0
5
10
15

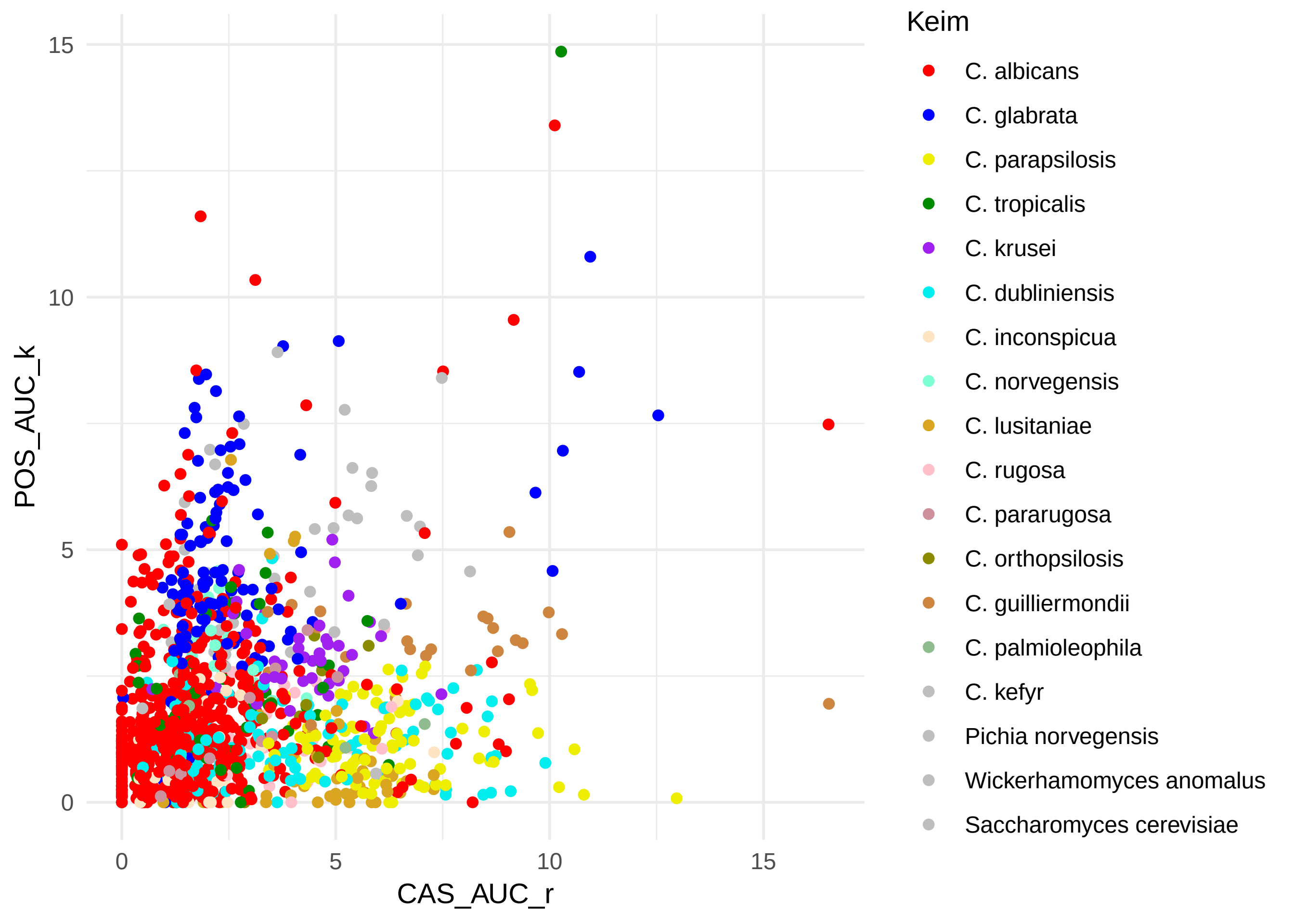

Keim
C. albicans
C. glabrata
C. parapsilosis
C. tropicalis
C. krusei
C. dubliniensis
C. inconspicua
C. norvegensis
C. lusitaniae
C. rugosa
C. pararugosa
C. orthopsilosis
C. guilliermondii
C. palmioleophila
C. kefyr
Pichia norvegensis
Wickerhamomyces anomalus
Saccharomyces cerevisiae
POS_AUC_k
CAS_AUC_r
0
5
10
15

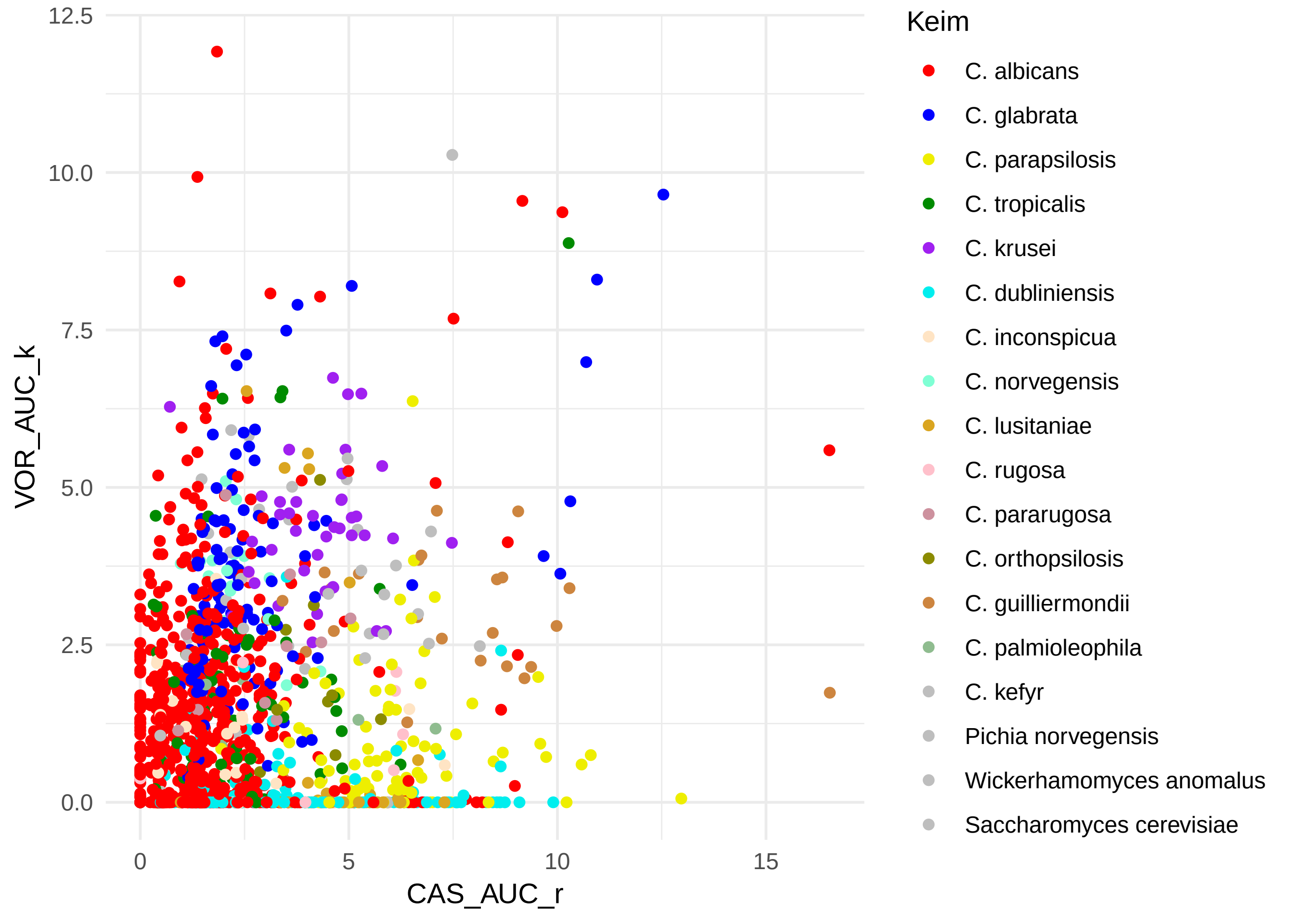

12.5
10.0
7.5
5.0
2.5
0.0
VOR_AUC_k
0
5
10
15
CAS_AUC_r
Keim
C. albicans
C. glabrata
C. parapsilosis
C. tropicalis
C. krusei
C. dubliniensis
C. inconspicua
C. norvegensis
C. lusitaniae
C. rugosa
C. pararugosa
C. orthopsilosis
C. guilliermondii
C. palmioleophila
C. kefyr
Pichia norvegensis
Wickerhamomyces anomalus
Saccharomyces cerevisiae

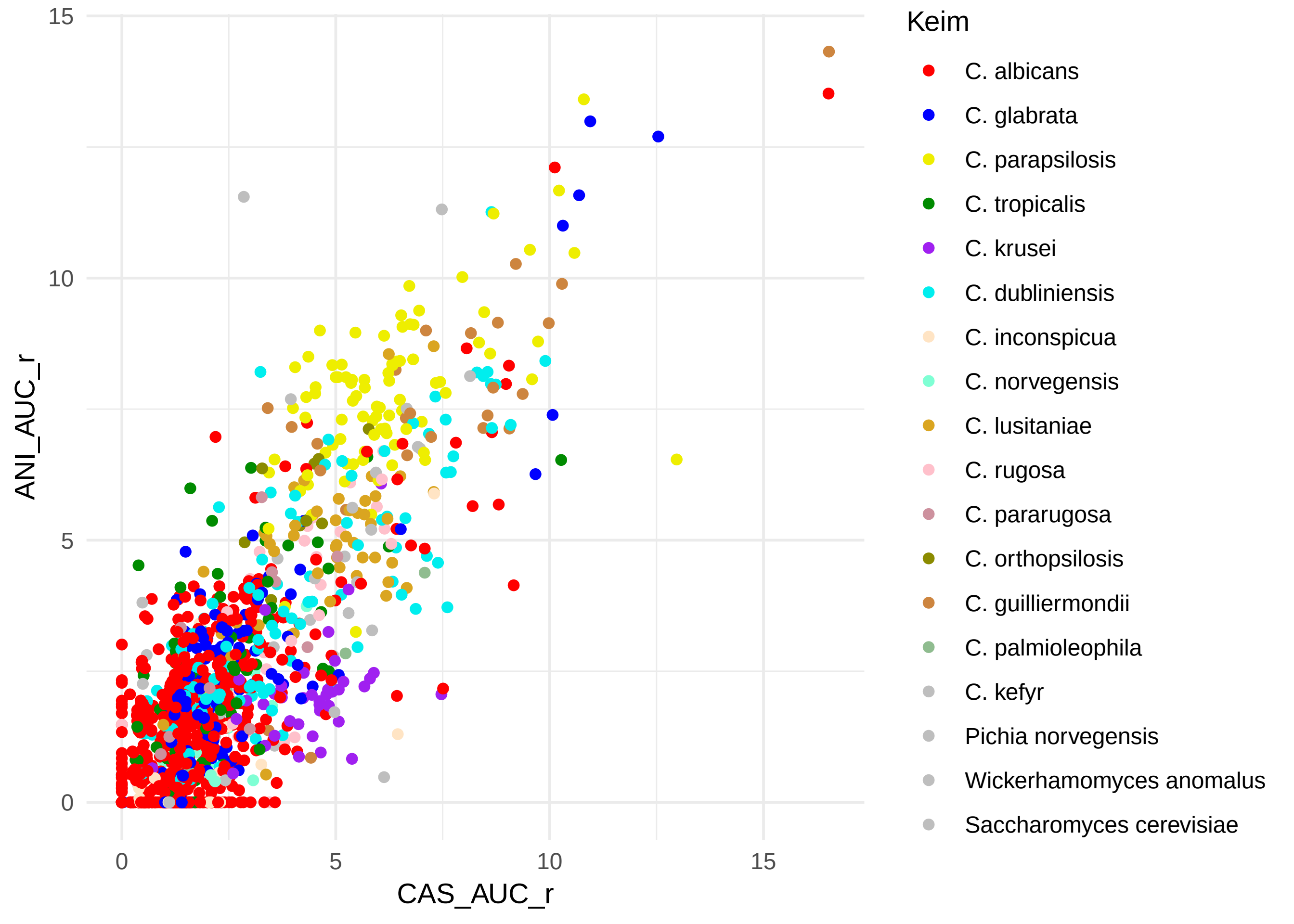

15
10
5
0
ANI_AUC_r
0
5
10
15
CAS_AUC_r
Keim
C. albicans
C. glabrata
C. parapsilosis
C. tropicalis
C. krusei
C. dubliniensis
C. inconspicua
C. norvegensis
C. lusitaniae
C. rugosa
C. pararugosa
C. orthopsilosis
C. guilliermondii
C. palmioleophila
C. kefyr
Pichia norvegensis
Wickerhamomyces anomalus
Saccharomyces cerevisiae

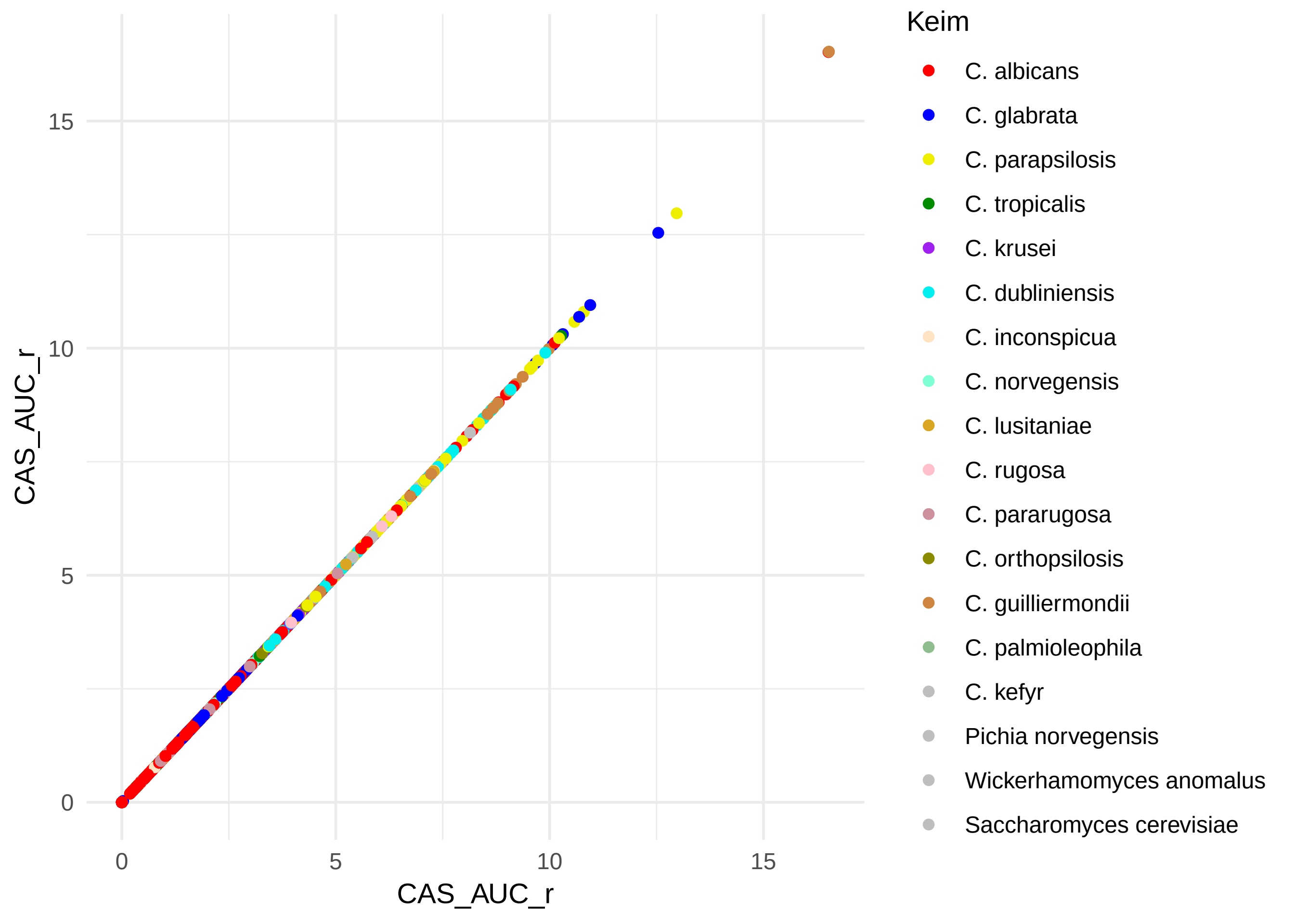

Keim
C. albicans
C. glabrata
C. parapsilosis
C. tropicalis
C. krusei
C. dubliniensis
C. inconspicua
C. norvegensis
C. lusitaniae
C. rugosa
C. pararugosa
C. orthopsilosis
C. guilliermondii
C. palmioleophila
C. kefyr
Pichia norvegensis
Wickerhamomyces anomalus
Saccharomyces cerevisiae
15
10
5
0
CAS_AUC_r
0
5
10
15
CAS_AUC_r

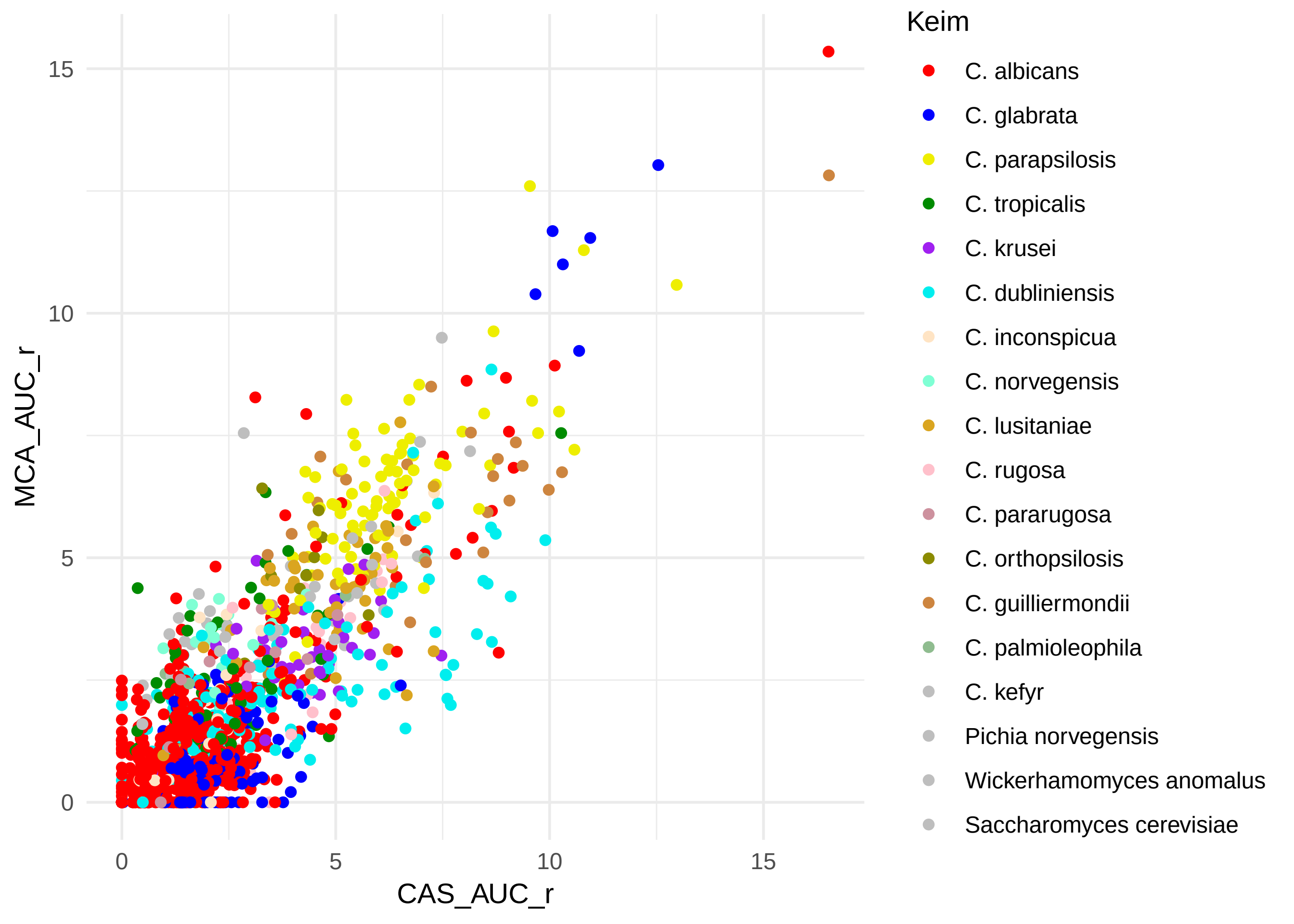

MCA_AUC_r
CAS_AUC_r
0
5
10
15
Keim
C. albicans
C. glabrata
C. parapsilosis
C. tropicalis
C. krusei
C. dubliniensis
C. inconspicua
C. norvegensis
C. lusitaniae
C. rugosa
C. pararugosa
C. orthopsilosis
C. guilliermondii
C. palmioleophila
C. kefyr
Pichia norvegensis
Wickerhamomyces anomalus
Saccharomyces cerevisiae

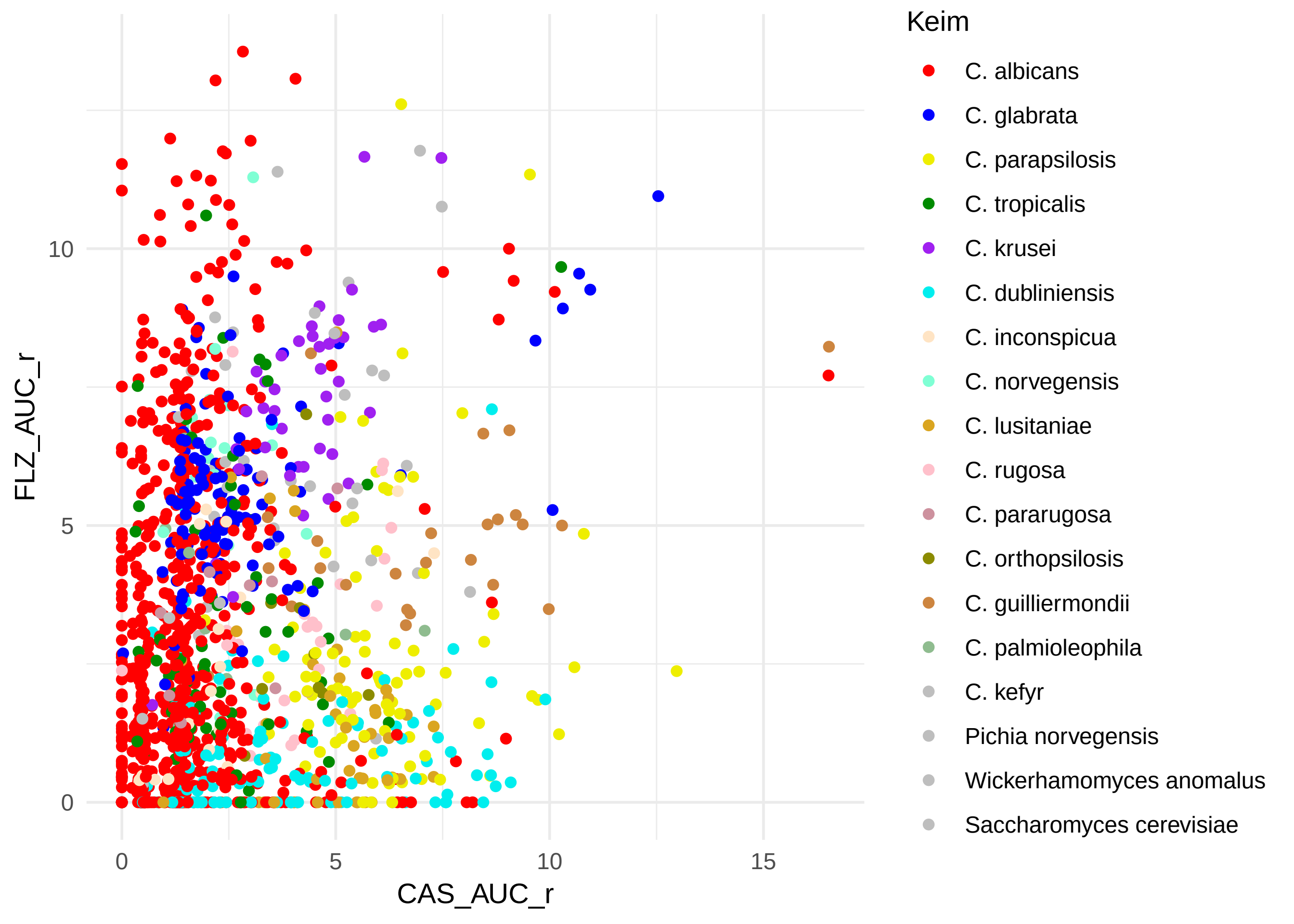
Keim
C. albicans
C. glabrata
C. parapsilosis
C. tropicalis
C. krusei
C. dubliniensis
C. inconspicua
C. norvegensis
C. lusitaniae
C. rugosa
C. pararugosa
C. orthopsilosis
C. guilliermondii
C. palmioleophila
C. kefyr
Pichia norvegensis
Wickerhamomyces anomalus
Saccharomyces cerevisiae
FLZ_AUC_r
10
5
0
CAS_AUC_r
0
5
10
15

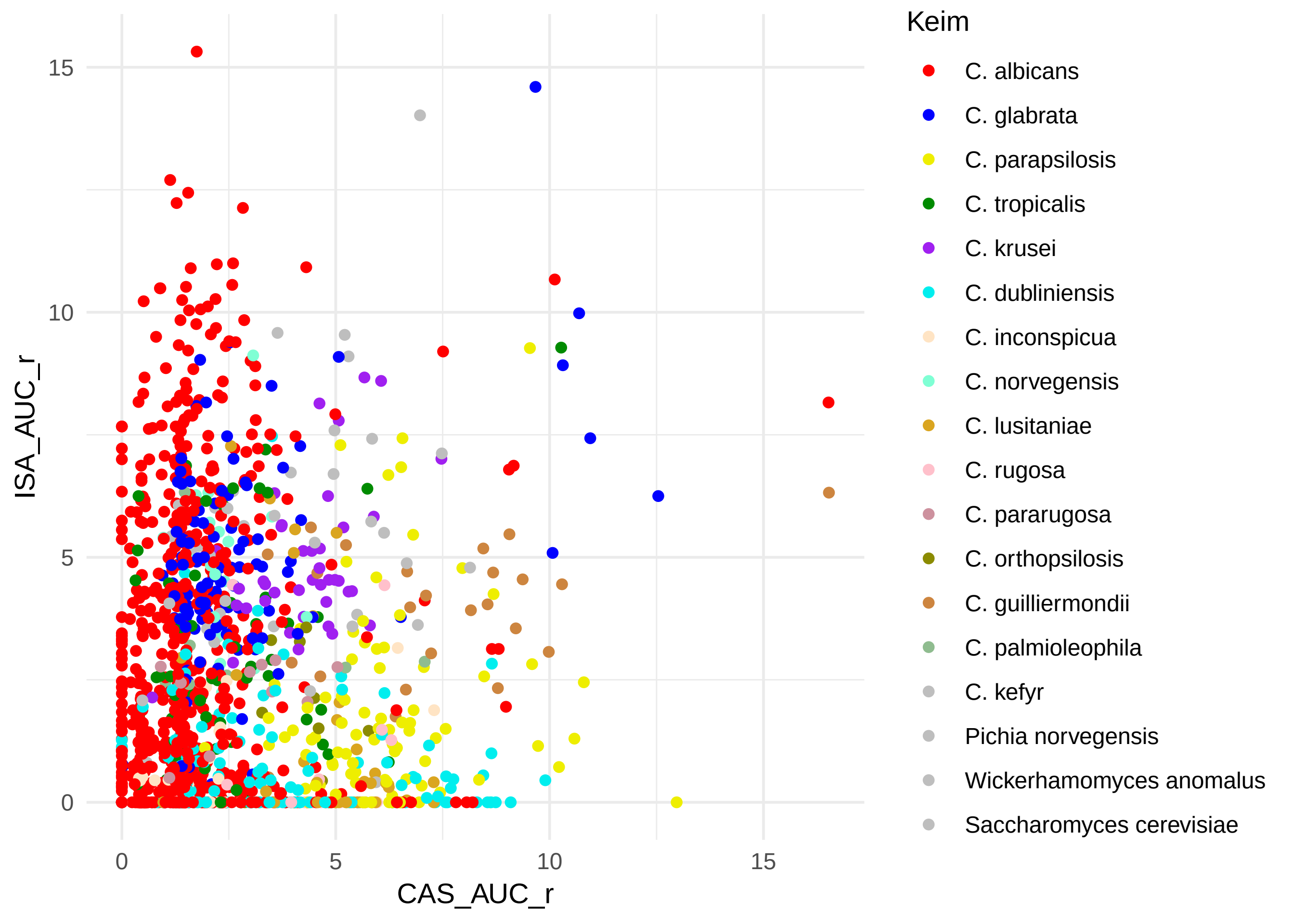

Keim
C. albicans
C. glabrata
C. parapsilosis
C. tropicalis
C. krusei
C. dubliniensis
C. inconspicua
C. norvegensis
C. lusitaniae
C. rugosa
C. pararugosa
C. orthopsilosis
C. guilliermondii
C. palmioleophila
C. kefyr
Pichia norvegensis
Wickerhamomyces anomalus
Saccharomyces cerevisiae
ISA_AUC_r
CAS_AUC_r
0
5
10
15

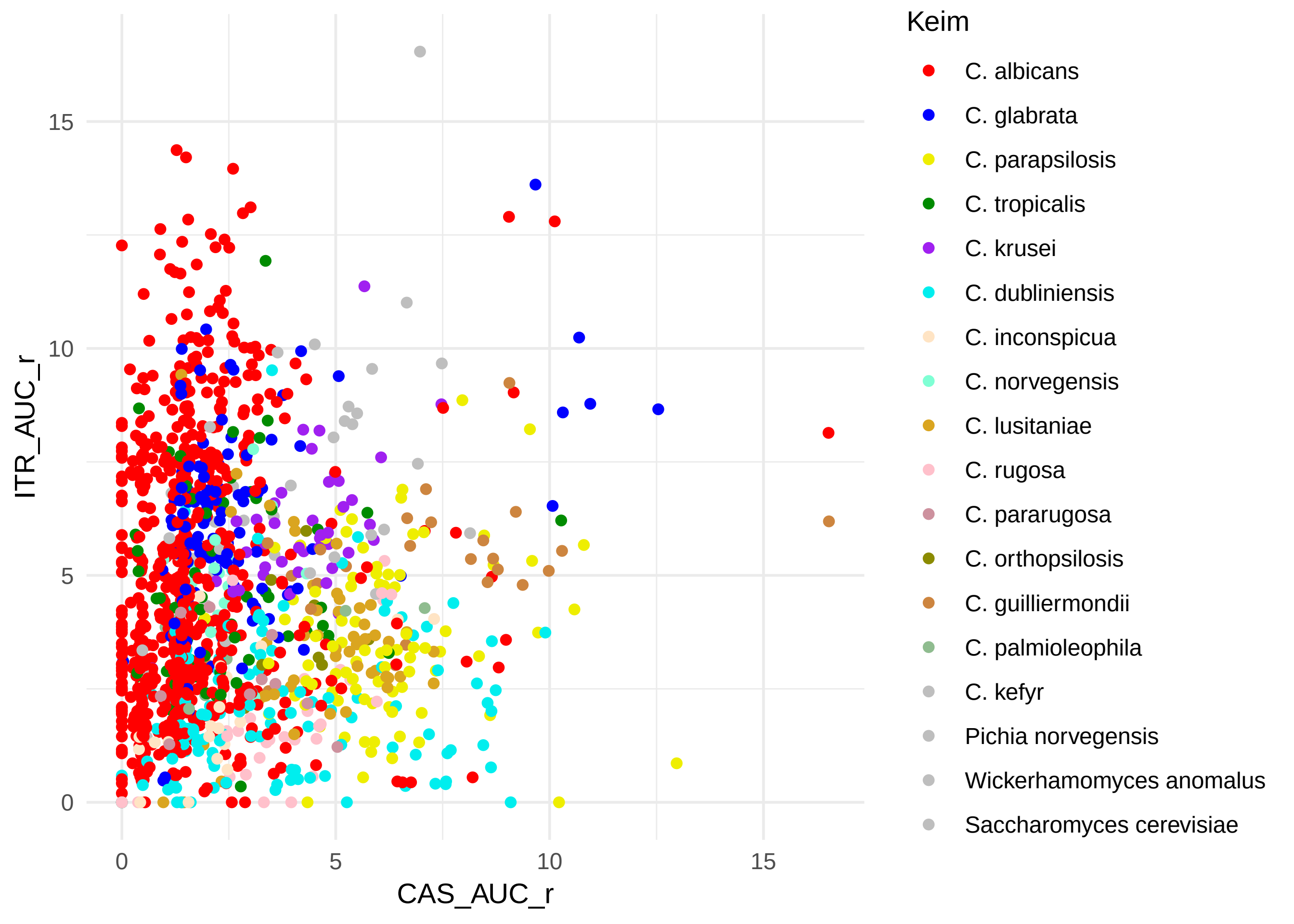

Keim
C. albicans
C. glabrata
C. parapsilosis
C. tropicalis
C. krusei
C. dubliniensis
C. inconspicua
C. norvegensis
C. lusitaniae
C. rugosa
C. pararugosa
C. orthopsilosis
C. guilliermondii
C. palmioleophila
C. kefyr
Pichia norvegensis
Wickerhamomyces anomalus
Saccharomyces cerevisiae
ITR_AUC_r
CAS_AUC_r
0
5
10
15

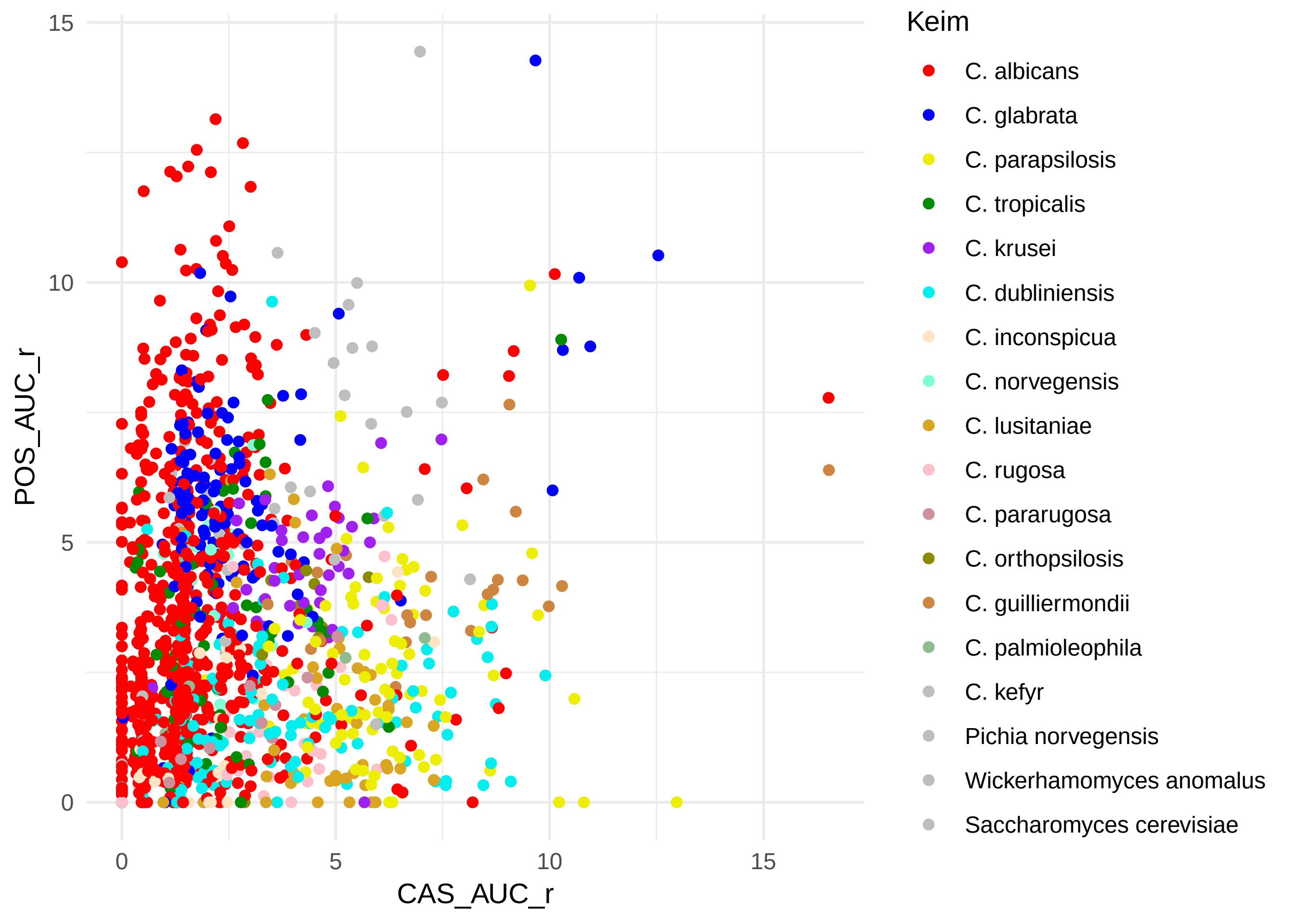
15
10
5
0
POS_AUC_r
0
5
10
15
CAS_AUC_r
Keim
C. albicans
C. glabrata
C. parapsilosis
C. tropicalis
C. krusei
C. dubliniensis
C. inconspicua
C. norvegensis
C. lusitaniae
C. rugosa
C. pararugosa
C. orthopsilosis
C. guilliermondii
C. palmioleophila
C. kefyr
Pichia norvegensis
Wickerhamomyces anomalus
Saccharomyces cerevisiae

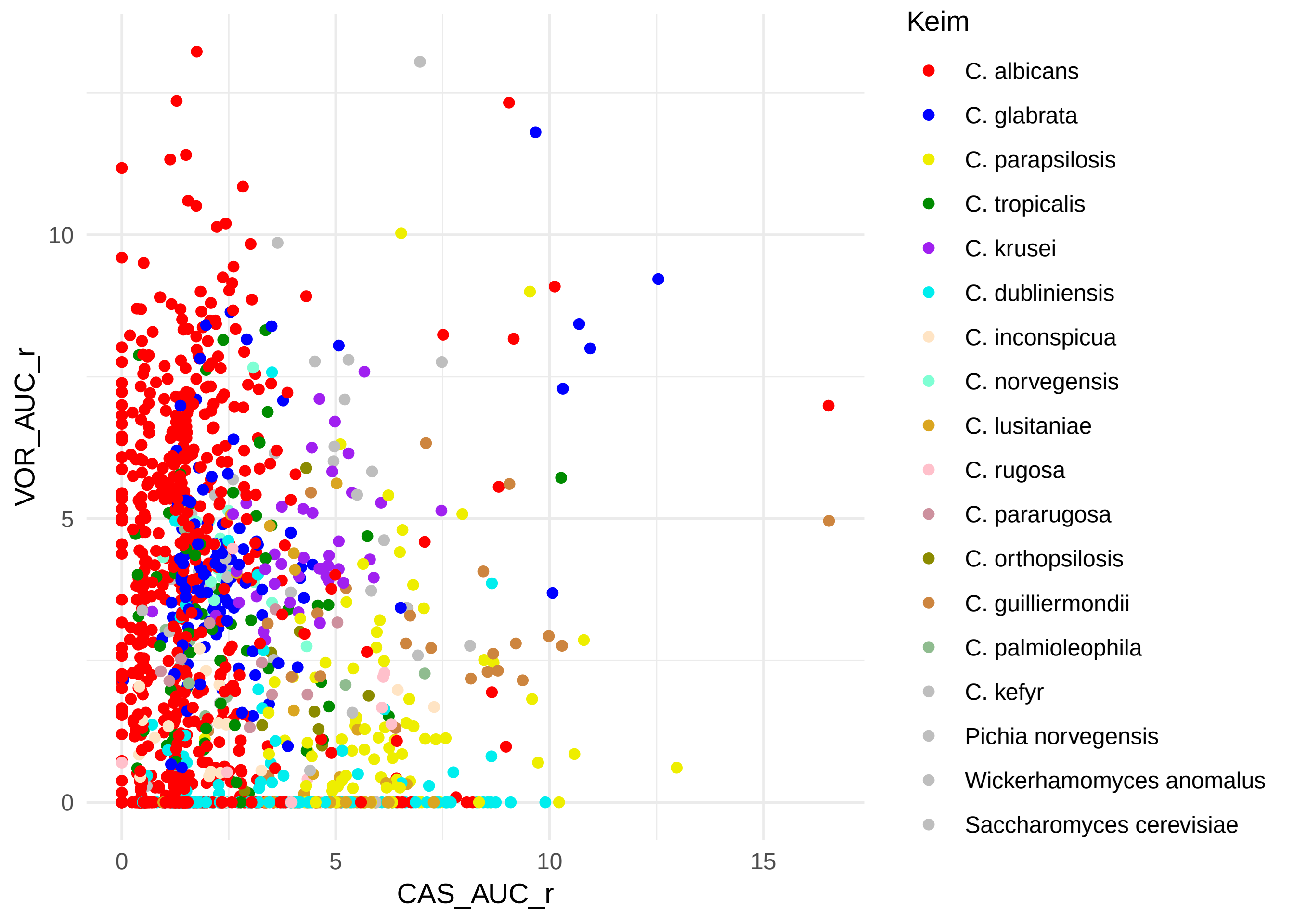

VOR_AUC_r
10
5
0
0
5
10
15
CAS_AUC_r
Keim
C. albicans
C. glabrata
C. parapsilosis
C. tropicalis
C. krusei
C. dubliniensis
C. inconspicua
C. norvegensis
C. lusitaniae
C. rugosa
C. pararugosa
C. orthopsilosis
C. guilliermondii
C. palmioleophila
C. kefyr
Pichia norvegensis
Wickerhamomyces anomalus
Saccharomyces cerevisiae

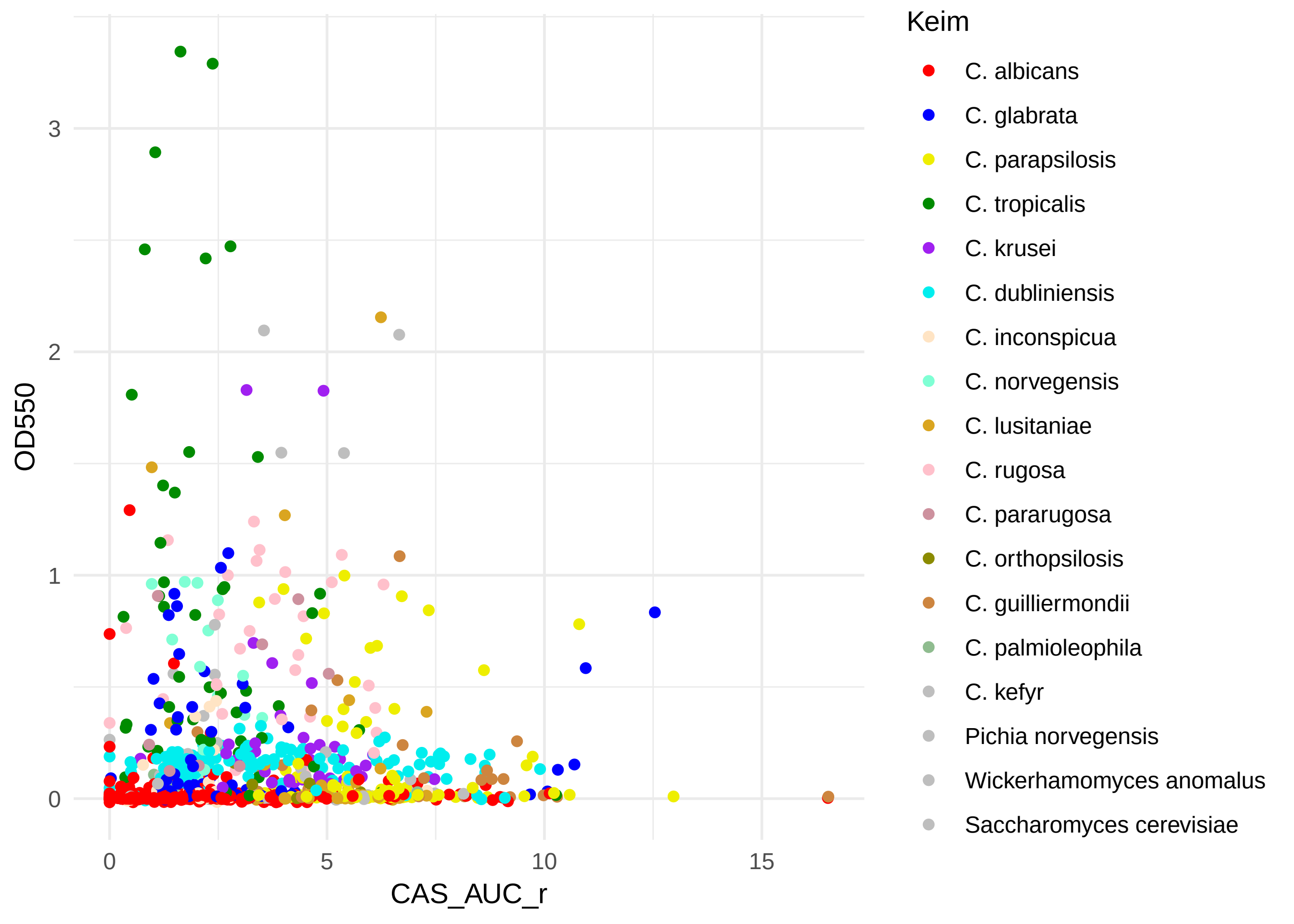

OD550
CAS_AUC_r
0
1
2
3
0
5
10
15
Keim
C. albicans
C. glabrata
C. parapsilosis
C. tropicalis
C. krusei
C. dubliniensis
C. inconspicua
C. norvegensis
C. lusitaniae
C. rugosa
C. pararugosa
C. orthopsilosis
C. guilliermondii
C. palmioleophila
C. kefyr
Pichia norvegensis
Wickerhamomyces anomalus
Saccharomyces cerevisiae

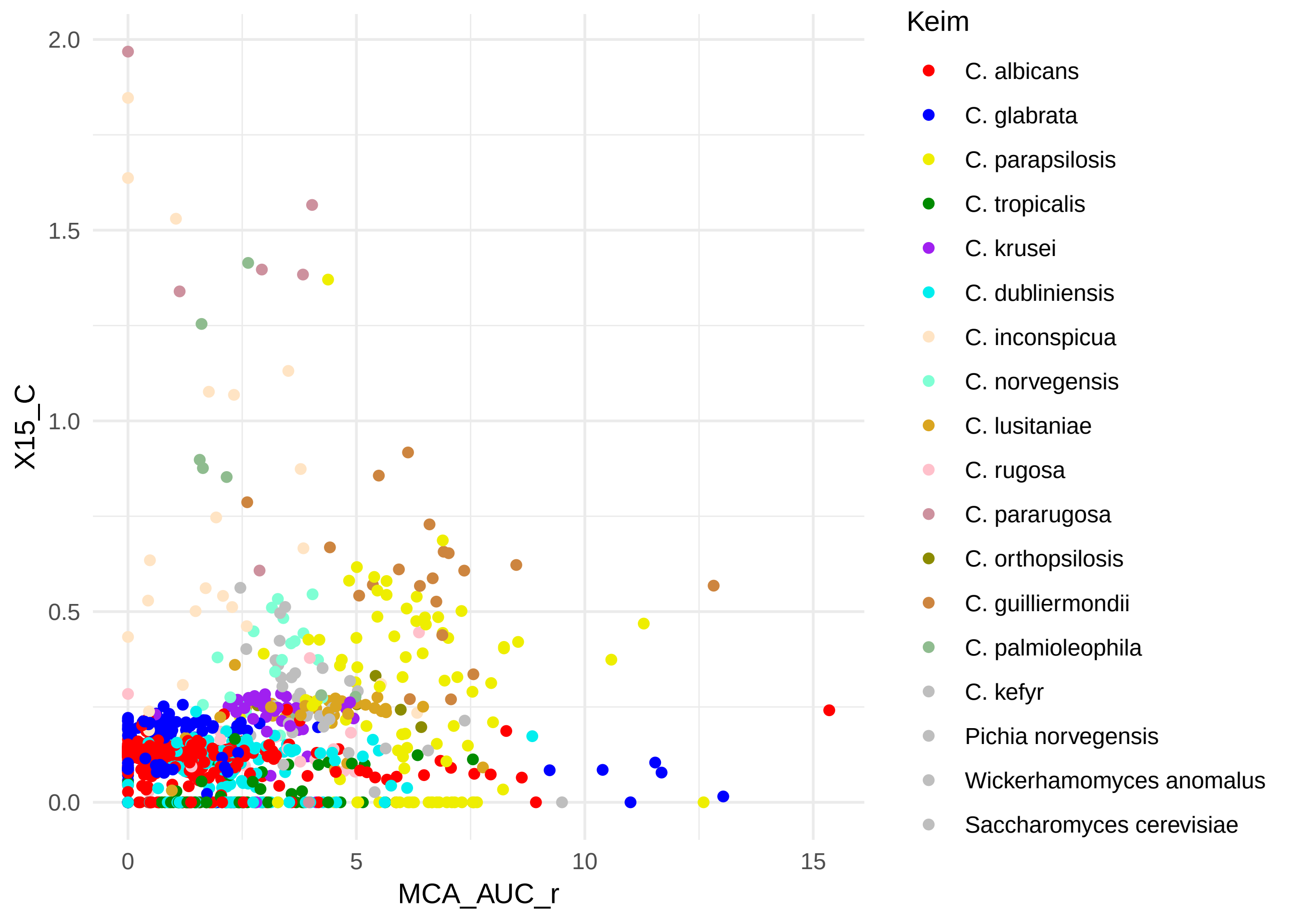

Keim
C. albicans
C. glabrata
C. parapsilosis
C. tropicalis
C. krusei
C. dubliniensis
C. inconspicua
C. norvegensis
C. lusitaniae
C. rugosa
C. pararugosa
C. orthopsilosis
C. guilliermondii
C. palmioleophila
C. kefyr
Pichia norvegensis
Wickerhamomyces anomalus
Saccharomyces cerevisiae
X15_C
2.0
1.5
1.0
0.5
0.0
0
5
10
15
MCA_AUC_r

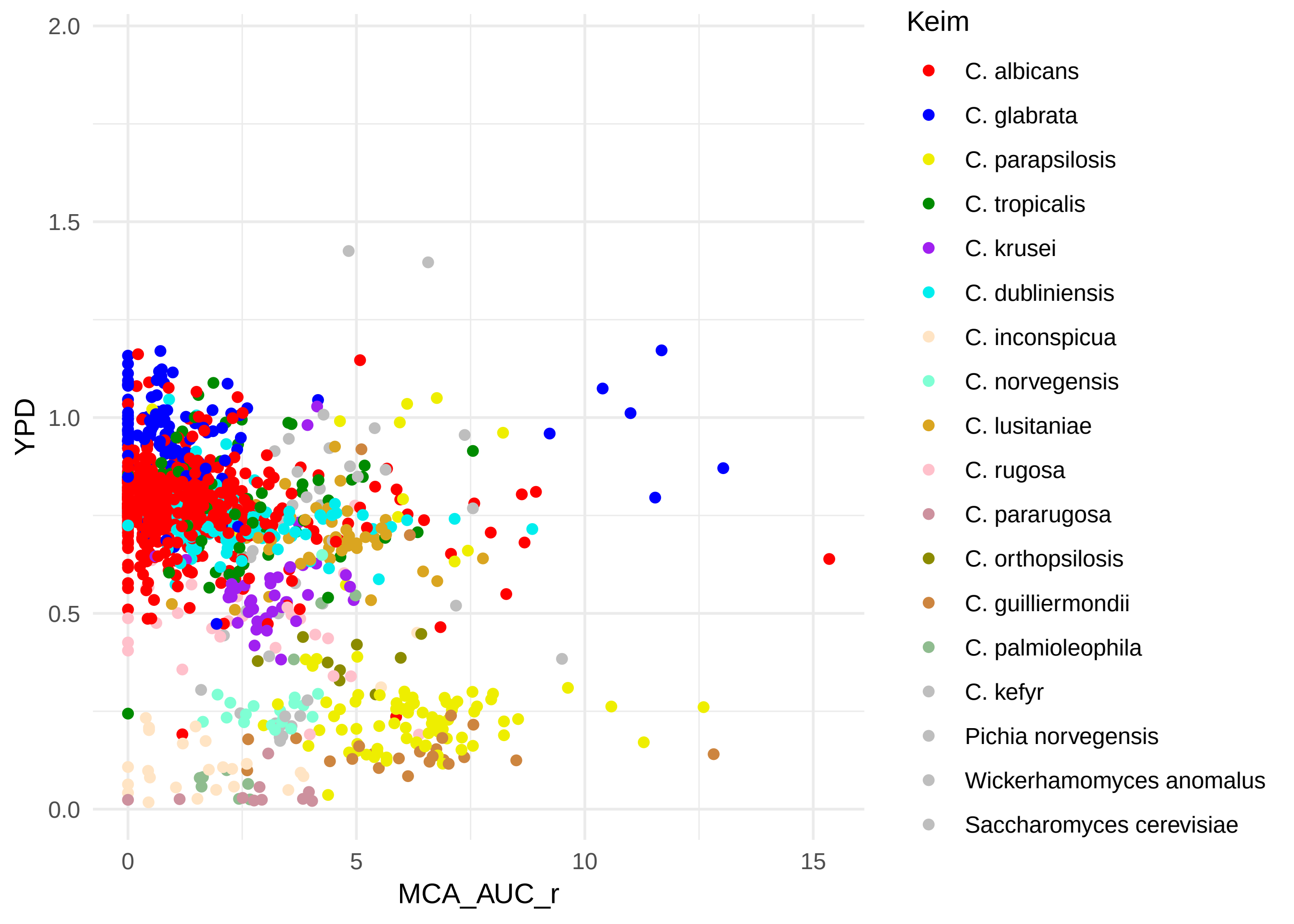

Keim
C. albicans
C. glabrata
C. parapsilosis
C. tropicalis
C. krusei
C. dubliniensis
C. inconspicua
C. norvegensis
C. lusitaniae
C. rugosa
C. pararugosa
C. orthopsilosis
C. guilliermondii
C. palmioleophila
C. kefyr
Pichia norvegensis
Wickerhamomyces anomalus
Saccharomyces cerevisiae
YPD
2.0
1.5
1.0
0.5
0.0
MCA_AUC_r
0
5
10
15

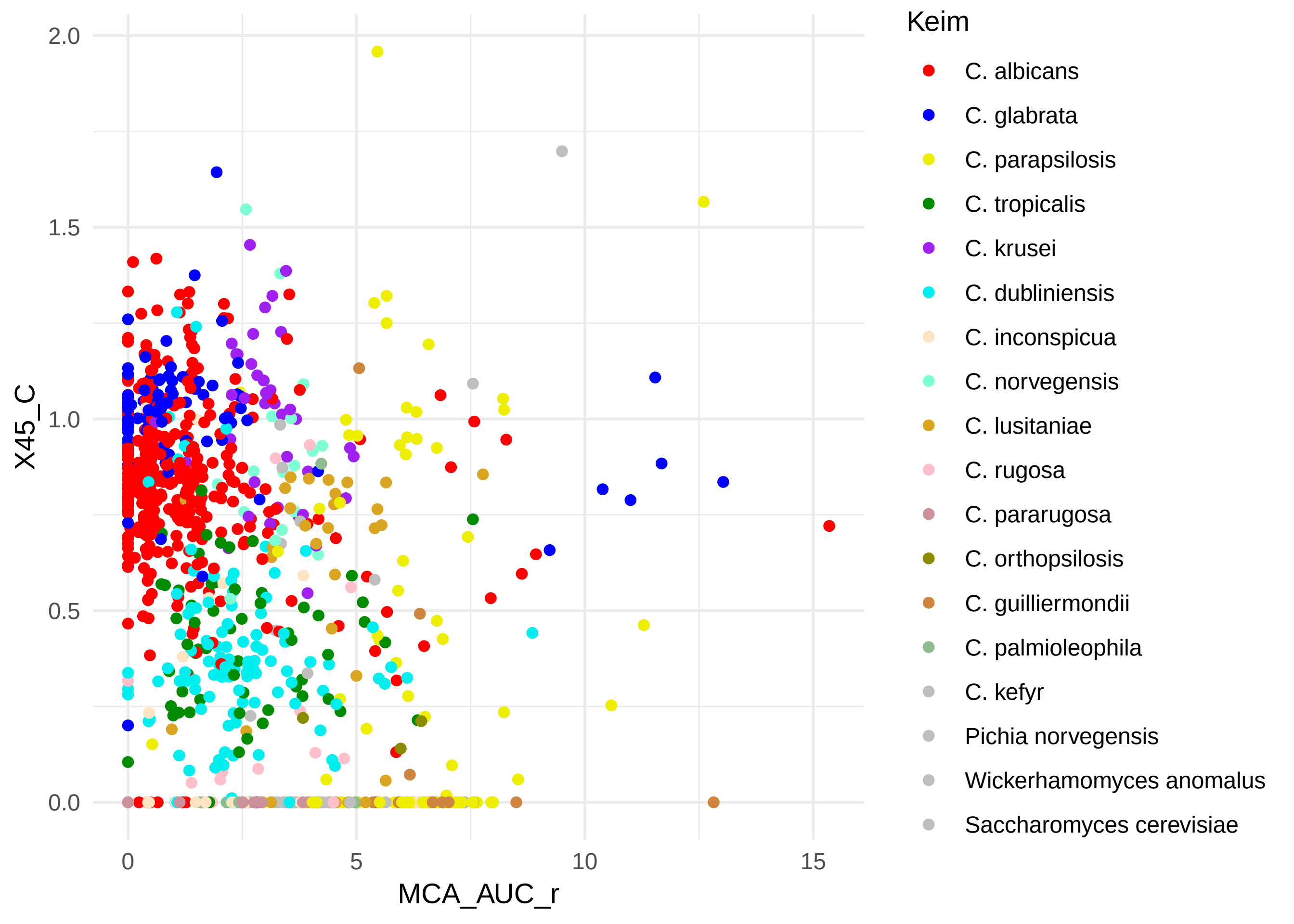

X45_C
MCA_AUC_r
2.0
1.5
1.0
0.5
0.0
0
5
10
15
Keim
C. albicans
C. glabrata
C. parapsilosis
C. tropicalis
C. krusei
C. dubliniensis
C. inconspicua
C. norvegensis
C. lusitaniae
C. rugosa
C. pararugosa
C. orthopsilosis
C. guilliermondii
C. palmioleophila
C. kefyr
Pichia norvegensis
Wickerhamomyces anomalus
Saccharomyces cerevisiae

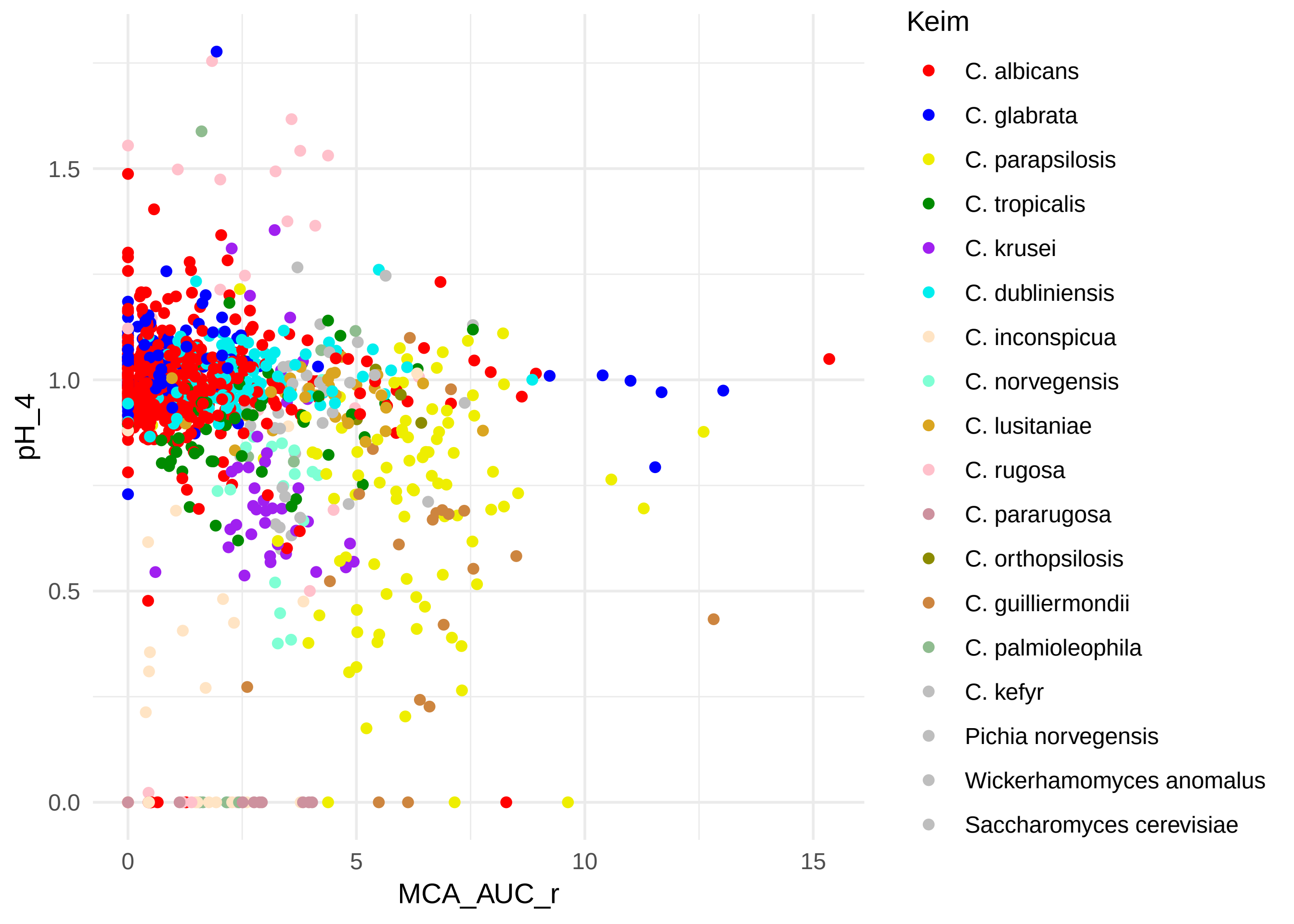

Keim
C. albicans
C. glabrata
C. parapsilosis
C. tropicalis
C. krusei
C. dubliniensis
C. inconspicua
C. norvegensis
C. lusitaniae
C. rugosa
C. pararugosa
C. orthopsilosis
C. guilliermondii
C. palmioleophila
C. kefyr
Pichia norvegensis
Wickerhamomyces anomalus
Saccharomyces cerevisiae
pH_4
1.5
1.0
0.5
0.0
0
5
10
15
MCA_AUC_r

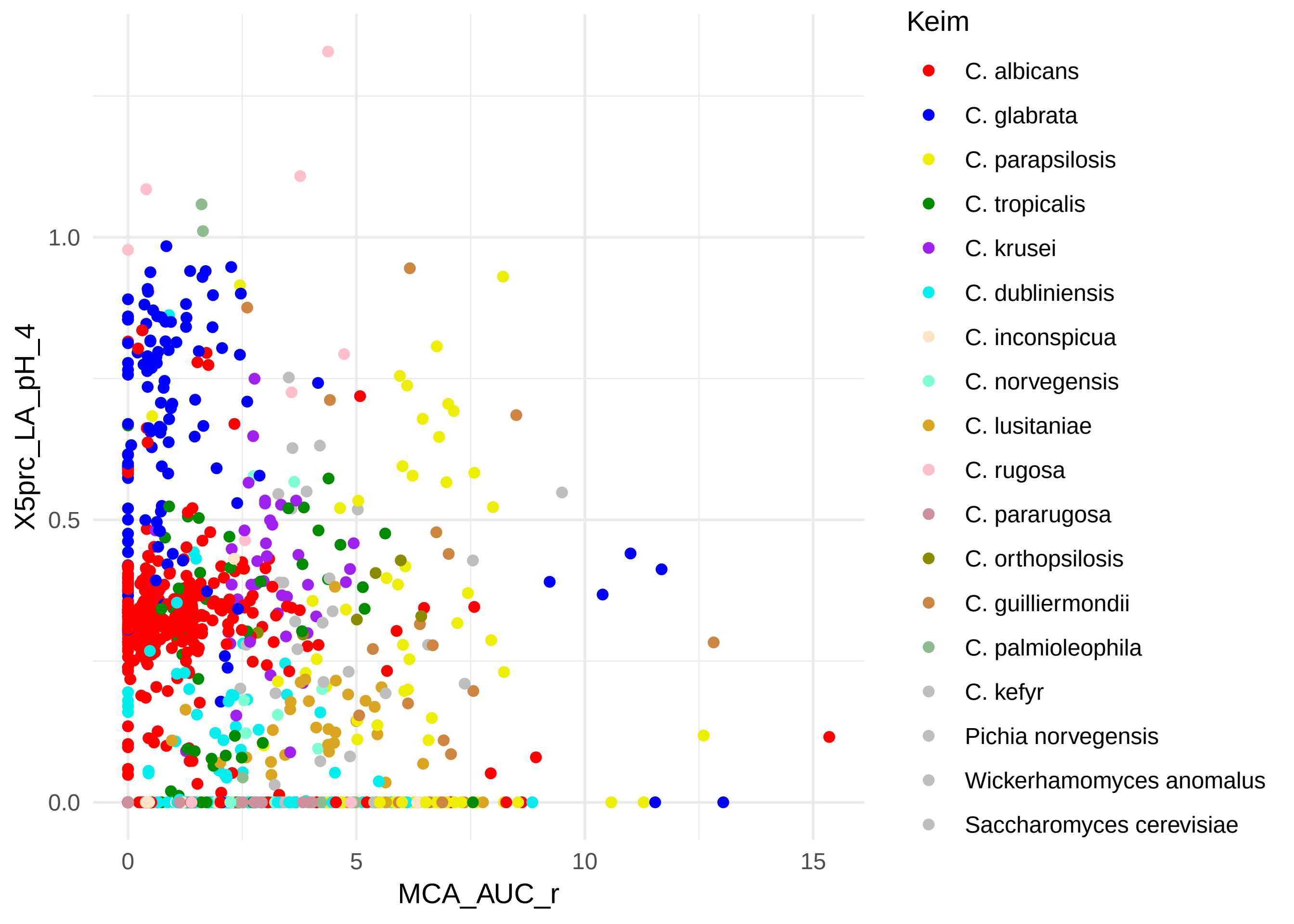

X5prc_LA_pH_4
1.0
0.5
0.0
0
5
10
15
MCA_AUC_r
Keim
C. albicans
C. glabrata
C. parapsilosis
C. tropicalis
C. krusei
C. dubliniensis
C. inconspicua
C. norvegensis
C. lusitaniae
C. rugosa
C. pararugosa
C. orthopsilosis
C. guilliermondii
C. palmioleophila
C. kefyr
Pichia norvegensis
Wickerhamomyces anomalus
Saccharomyces cerevisiae

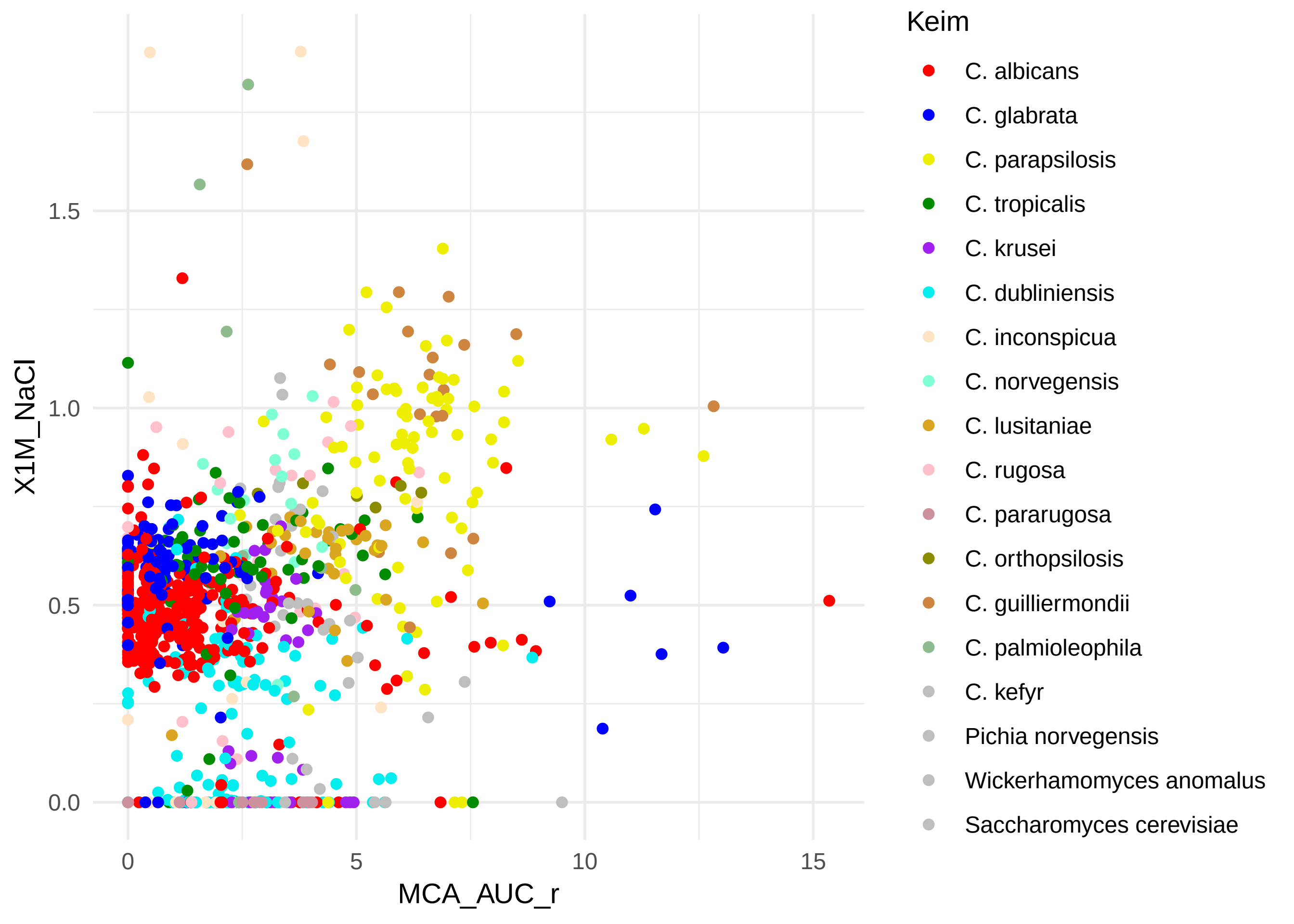
Keim
C. albicans
C. glabrata
C. parapsilosis
C. tropicalis
C. krusei
C. dubliniensis
C. inconspicua
C. norvegensis
C. lusitaniae
C. rugosa
C. pararugosa
C. orthopsilosis
C. guilliermondii
C. palmioleophila
C. kefyr
Pichia norvegensis
Wickerhamomyces anomalus
Saccharomyces cerevisiae
X1M_NaCl
MCA_AUC_r
0.0
0.5
1.0
1.5
0
5
10
15

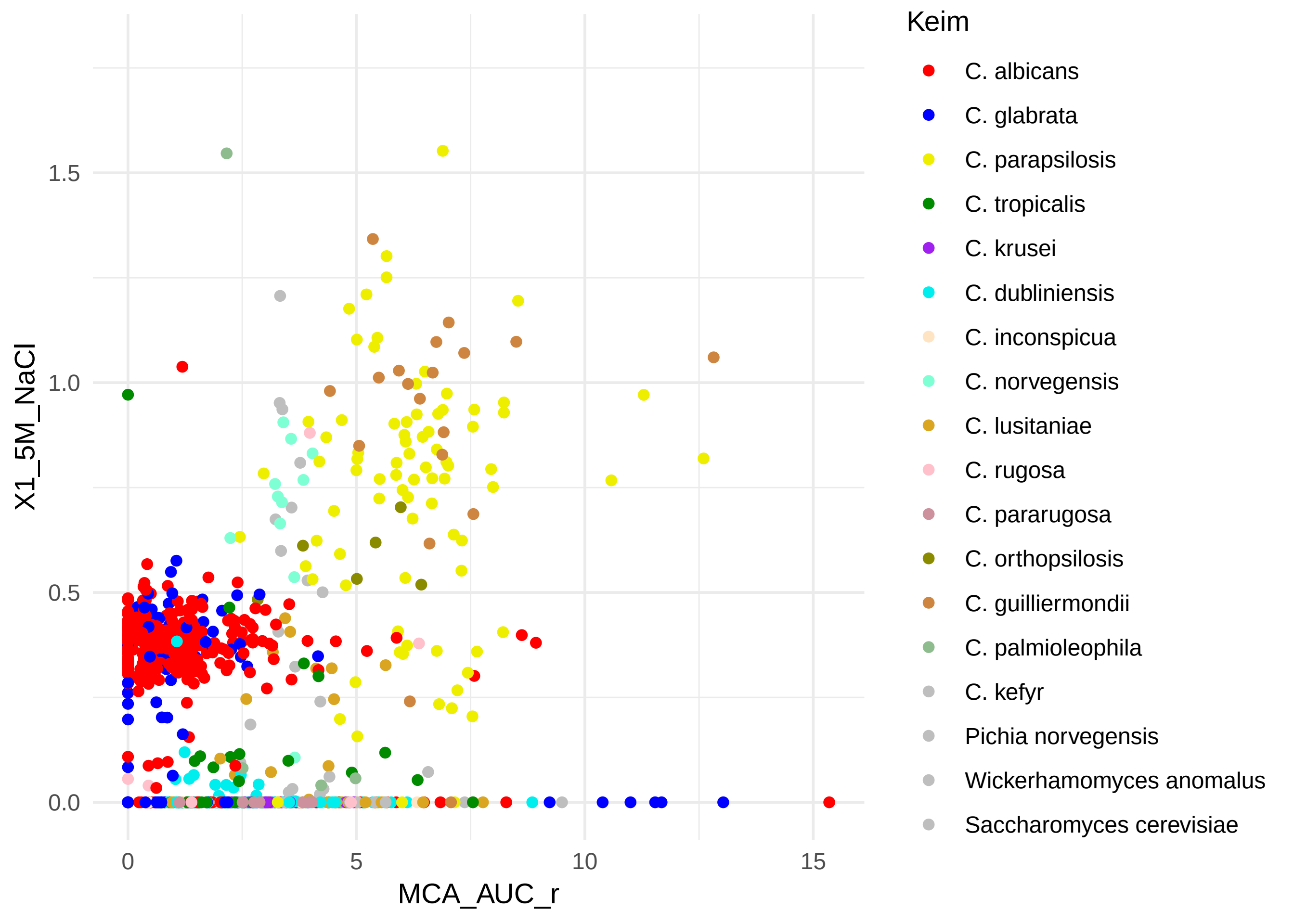

Keim
C. albicans
C. glabrata
C. parapsilosis
C. tropicalis
C. krusei
C. dubliniensis
C. inconspicua
C. norvegensis
C. lusitaniae
C. rugosa
C. pararugosa
C. orthopsilosis
C. guilliermondii
C. palmioleophila
C. kefyr
Pichia norvegensis
Wickerhamomyces anomalus
Saccharomyces cerevisiae
X1_5M_NaCl
MCA_AUC_r
0.0
0.5
1.0
1.5
0
5
10
15

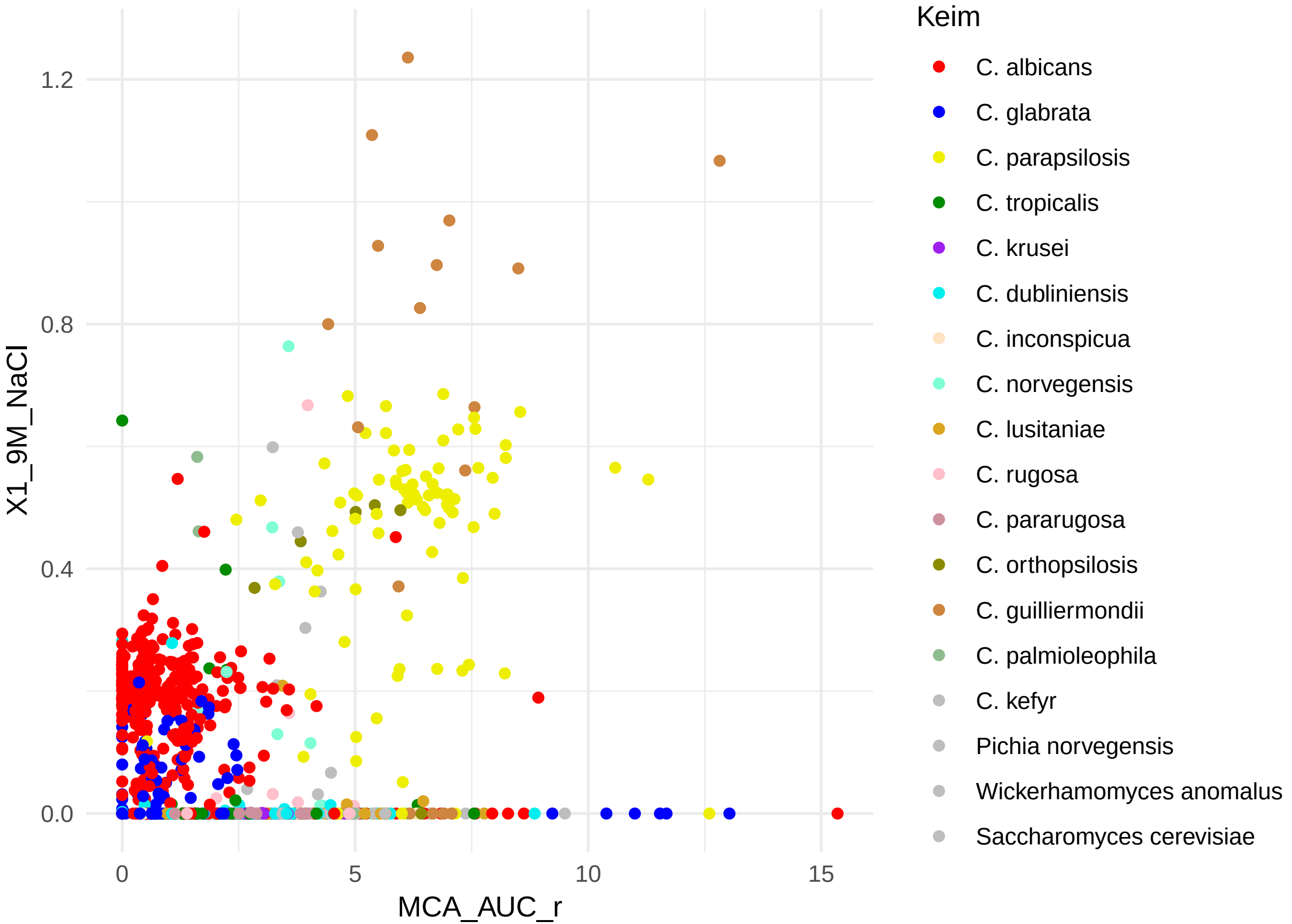
Keim
C. albicans
C. glabrata
C. parapsilosis
C. tropicalis
C. krusei
C. dubliniensis
C. inconspicua
C. norvegensis
C. lusitaniae
C. rugosa
C. pararugosa
C. orthopsilosis
C. guilliermondii
C. palmioleophila
C. kefyr
Pichia norvegensis
Wickerhamomyces anomalus
Saccharomyces cerevisiae
X1_9M_NaCl
1.2
0.8
0.4
0.0
0
5
10
15
MCA_AUC_r

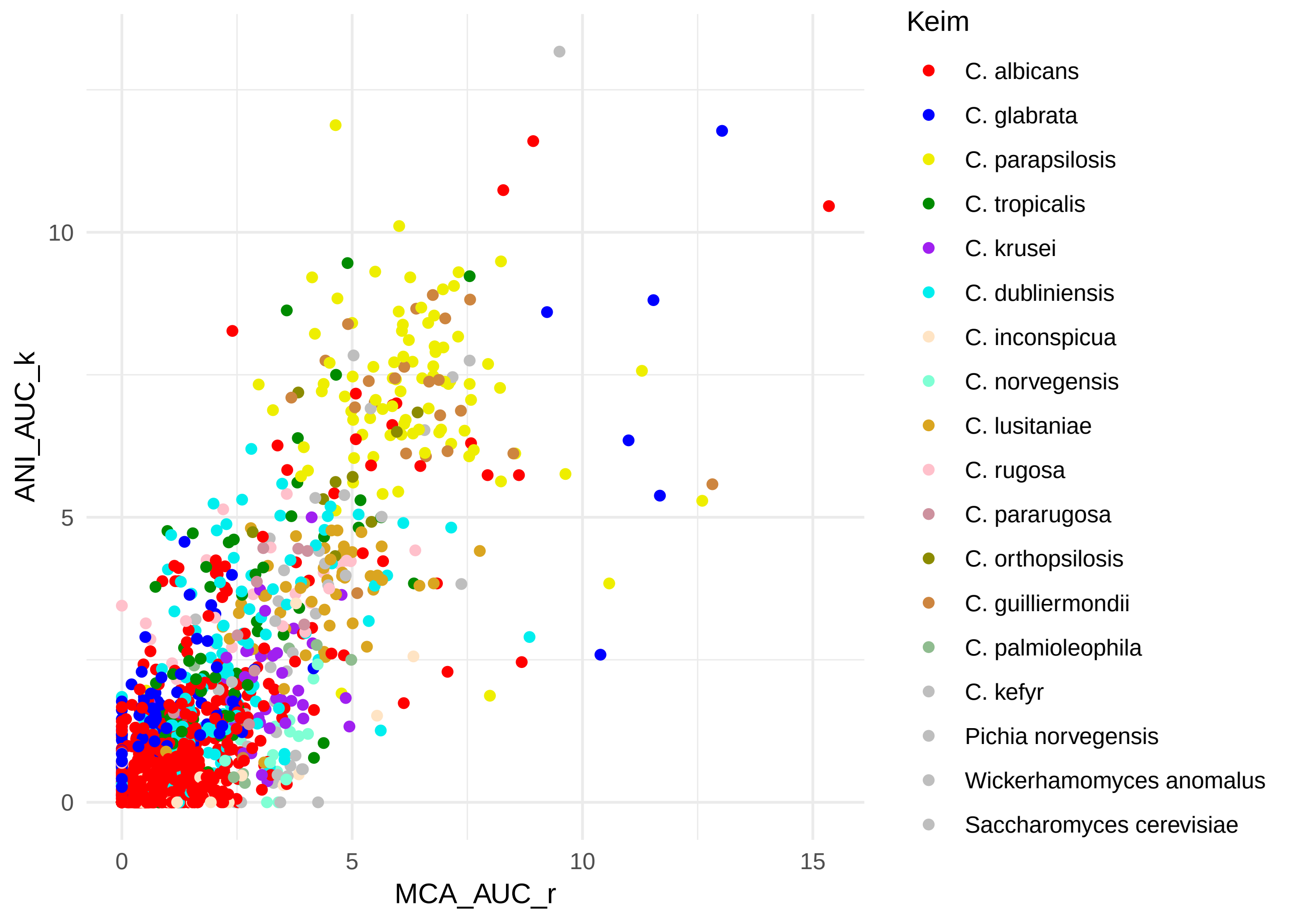

Keim
C. albicans
C. glabrata
C. parapsilosis
C. tropicalis
C. krusei
C. dubliniensis
C. inconspicua
C. norvegensis
C. lusitaniae
C. rugosa
C. pararugosa
C. orthopsilosis
C. guilliermondii
C. palmioleophila
C. kefyr
Pichia norvegensis
Wickerhamomyces anomalus
Saccharomyces cerevisiae
ANI_AUC_k
MCA_AUC_r
0
5
10
15

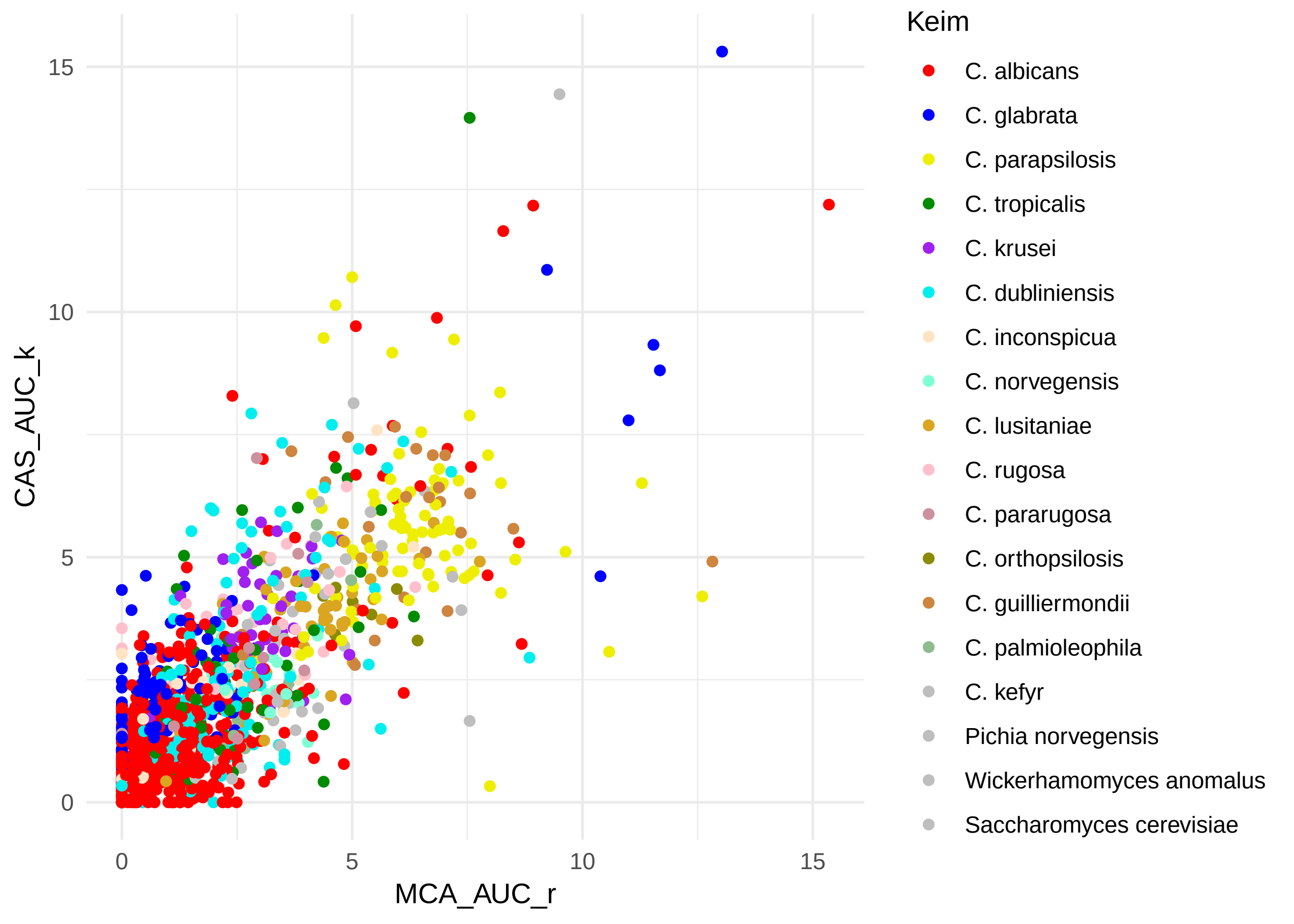
Keim
C. albicans
C. glabrata
C. parapsilosis
C. tropicalis
C. krusei
C. dubliniensis
C. inconspicua
C. norvegensis
C. lusitaniae
C. rugosa
C. pararugosa
C. orthopsilosis
C. guilliermondii
C. palmioleophila
C. kefyr
Pichia norvegensis
Wickerhamomyces anomalus
Saccharomyces cerevisiae
CAS_AUC_k
MCA_AUC_r
0
5
10
15

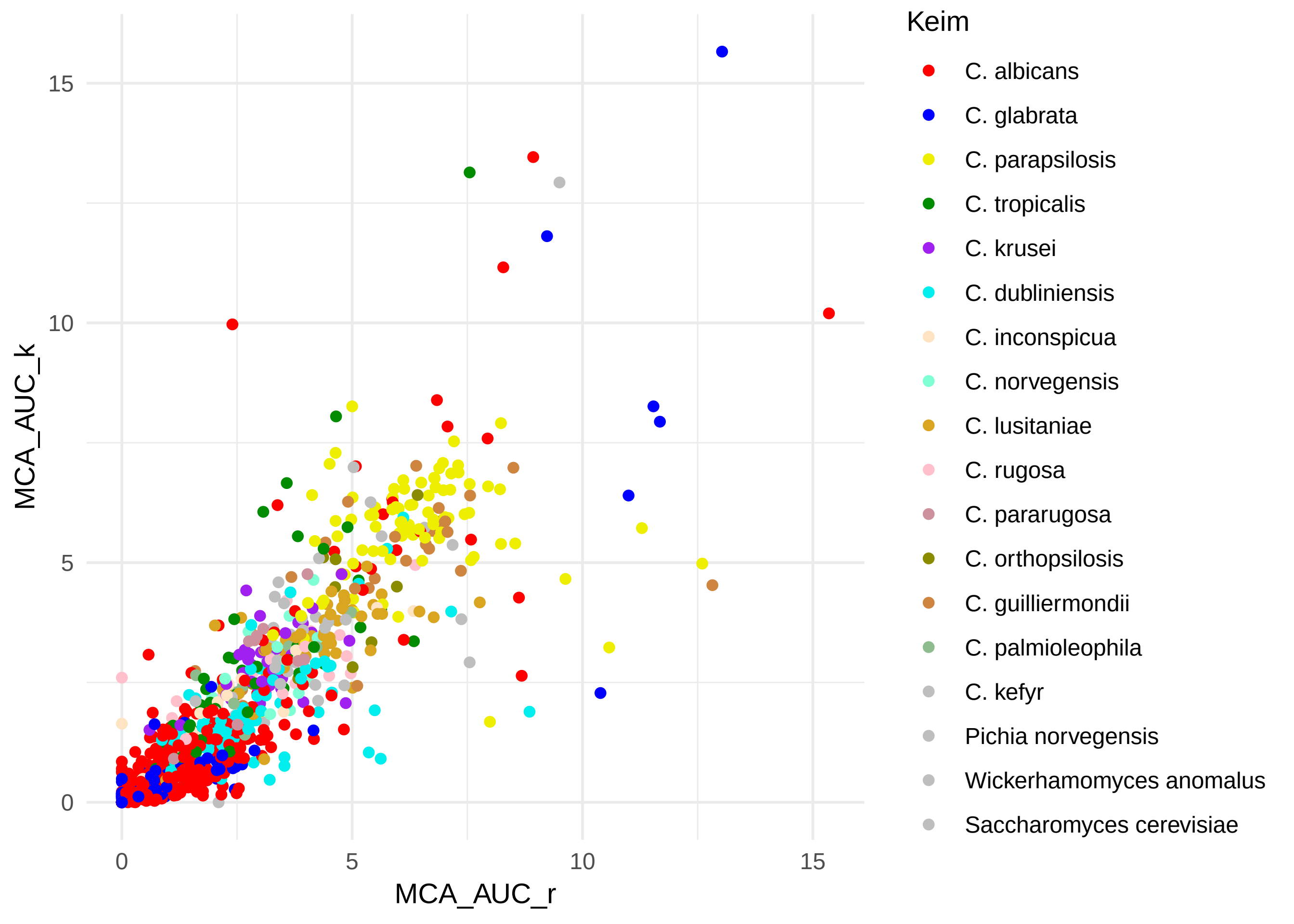

MCA_AUC_k
MCA_AUC_r
0
5
10
15
Keim
C. albicans
C. glabrata
C. parapsilosis
C. tropicalis
C. krusei
C. dubliniensis
C. inconspicua
C. norvegensis
C. lusitaniae
C. rugosa
C. pararugosa
C. orthopsilosis
C. guilliermondii
C. palmioleophila
C. kefyr
Pichia norvegensis
Wickerhamomyces anomalus
Saccharomyces cerevisiae

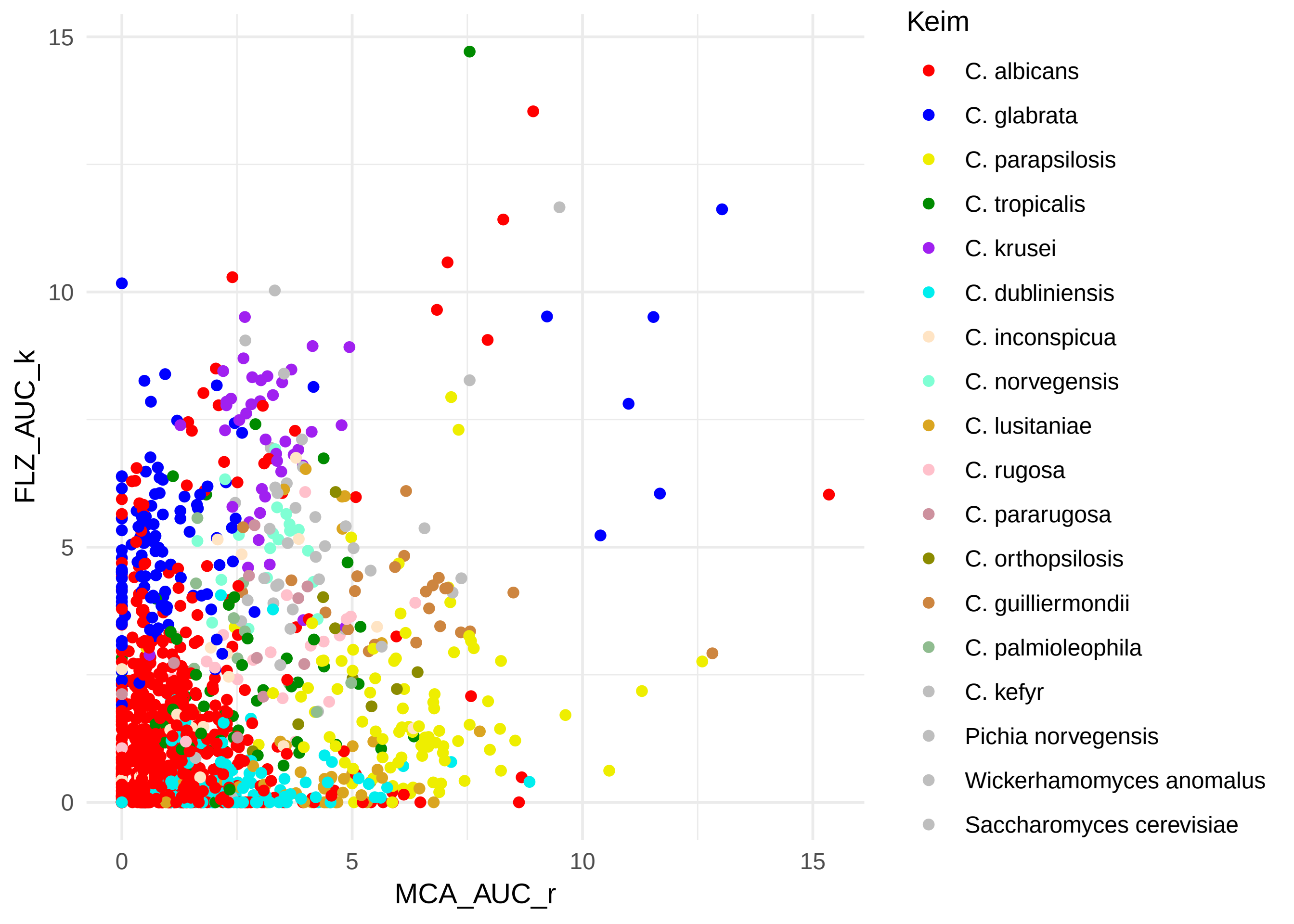

FLZ_AUC_k
MCA_AUC_r
0
5
10
15
0
5
10
15
Keim
C. albicans
C. glabrata
C. parapsilosis
C. tropicalis
C. krusei
C. dubliniensis
C. inconspicua
C. norvegensis
C. lusitaniae
C. rugosa
C. pararugosa
C. orthopsilosis
C. guilliermondii
C. palmioleophila
C. kefyr
Pichia norvegensis
Wickerhamomyces anomalus
Saccharomyces cerevisiae

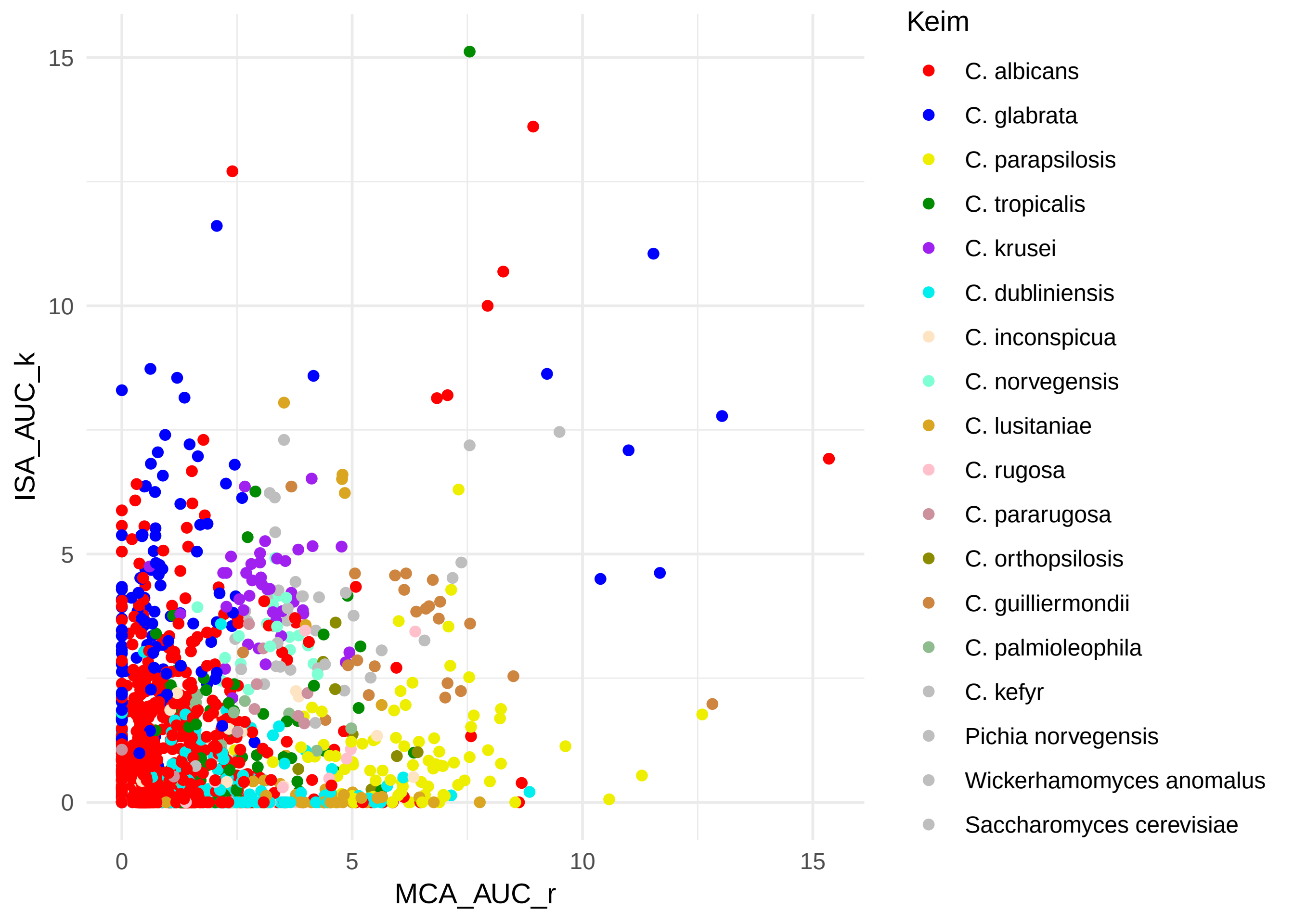
ISA_AUC_k
MCA_AUC_r
0
5
10
15
0
5
10
15
Keim
C. albicans
C. glabrata
C. parapsilosis
C. tropicalis
C. krusei
C. dubliniensis
C. inconspicua
C. norvegensis
C. lusitaniae
C. rugosa
C. pararugosa
C. orthopsilosis
C. guilliermondii
C. palmioleophila
C. kefyr
Pichia norvegensis
Wickerhamomyces anomalus
Saccharomyces cerevisiae

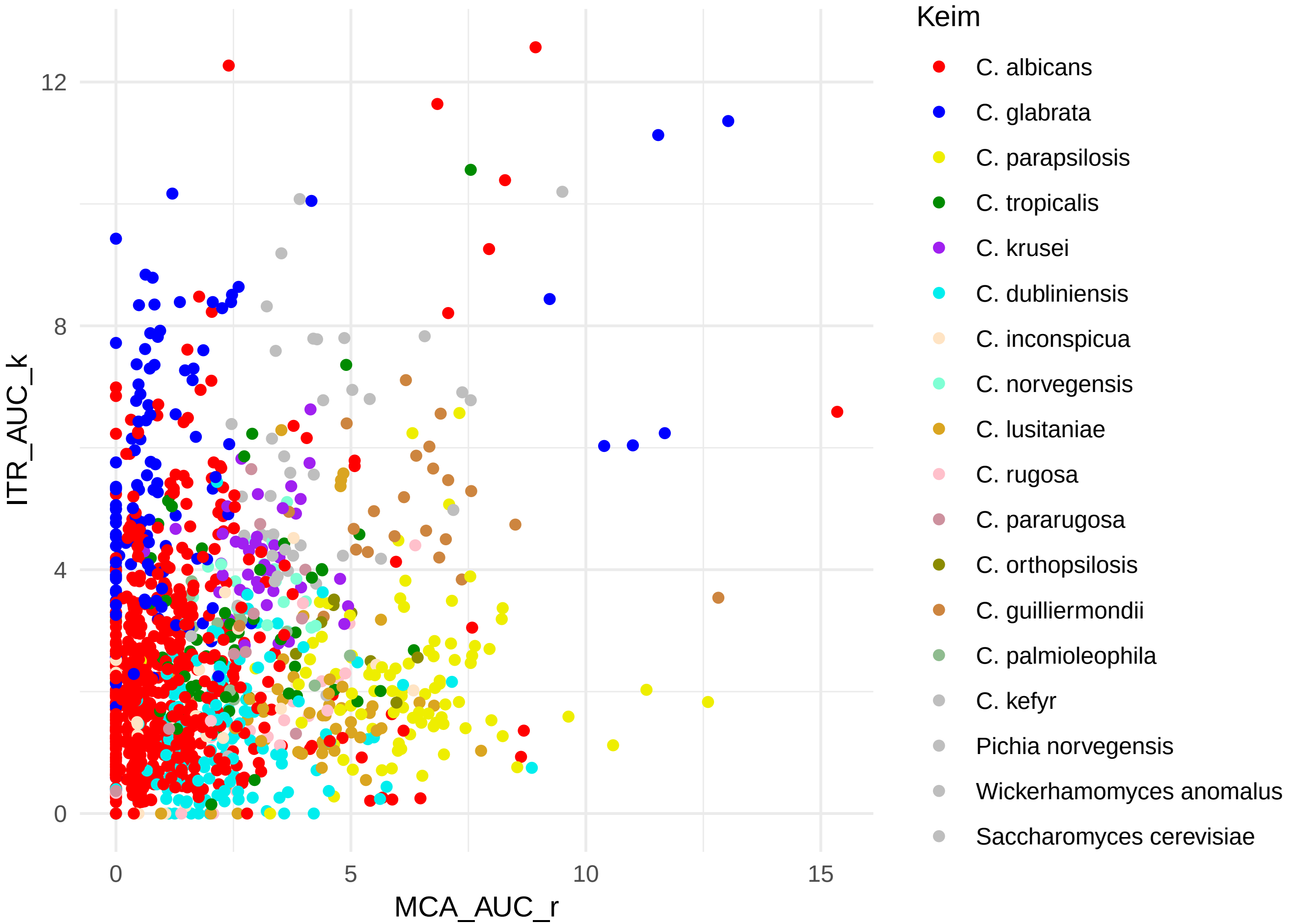

Keim
C. albicans
C. glabrata
C. parapsilosis
C. tropicalis
C. krusei
C. dubliniensis
C. inconspicua
C. norvegensis
C. lusitaniae
C. rugosa
C. pararugosa
C. orthopsilosis
C. guilliermondii
C. palmioleophila
C. kefyr
Pichia norvegensis
Wickerhamomyces anomalus
Saccharomyces cerevisiae
ITR_AUC_k
MCA_AUC_r
0
4
8
12
0
5
10
15

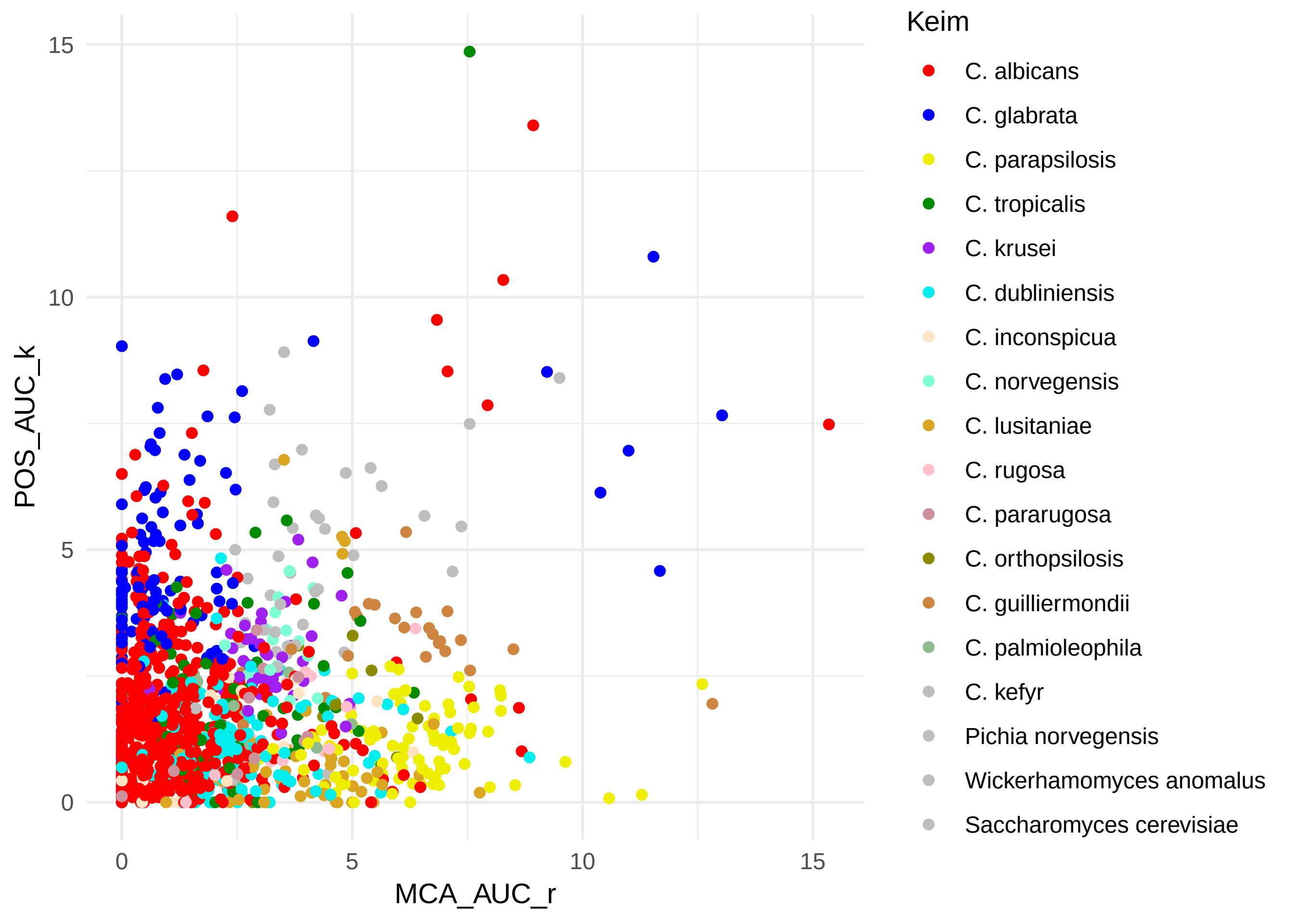
Keim
C. albicans
C. glabrata
C. parapsilosis
C. tropicalis
C. krusei
C. dubliniensis
C. inconspicua
C. norvegensis
C. lusitaniae
C. rugosa
C. pararugosa
C. orthopsilosis
C. guilliermondii
C. palmioleophila
C. kefyr
Pichia norvegensis
Wickerhamomyces anomalus
Saccharomyces cerevisiae
POS_AUC_k
MCA_AUC_r
0
5
10
15

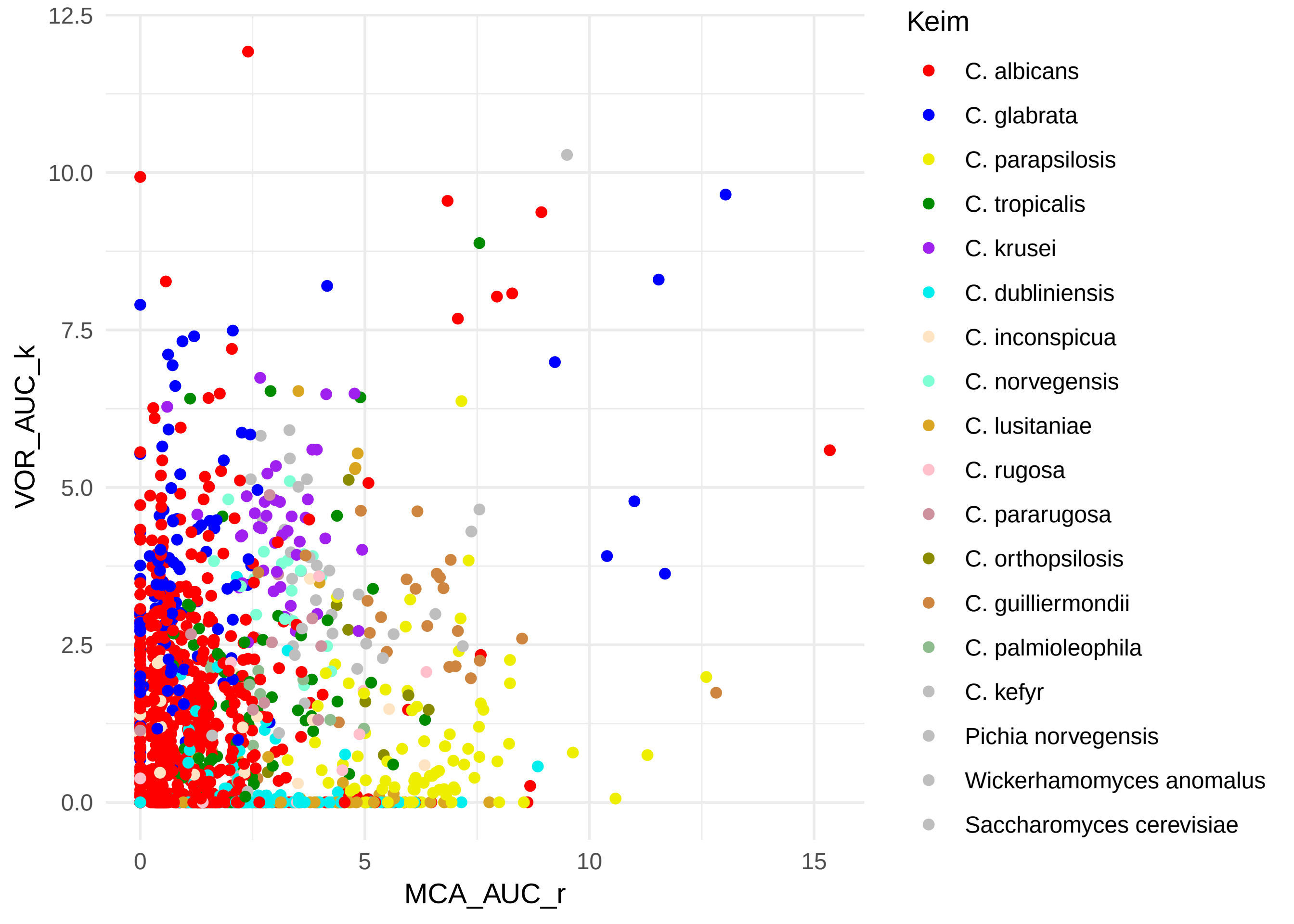

VOR_AUC_k
MCA_AUC_r
12.5
10.0
7.5
5.0
2.5
0.0
0
5
10
15
Keim
C. albicans
C. glabrata
C. parapsilosis
C. tropicalis
C. krusei
C. dubliniensis
C. inconspicua
C. norvegensis
C. lusitaniae
C. rugosa
C. pararugosa
C. orthopsilosis
C. guilliermondii
C. palmioleophila
C. kefyr
Pichia norvegensis
Wickerhamomyces anomalus
Saccharomyces cerevisiae

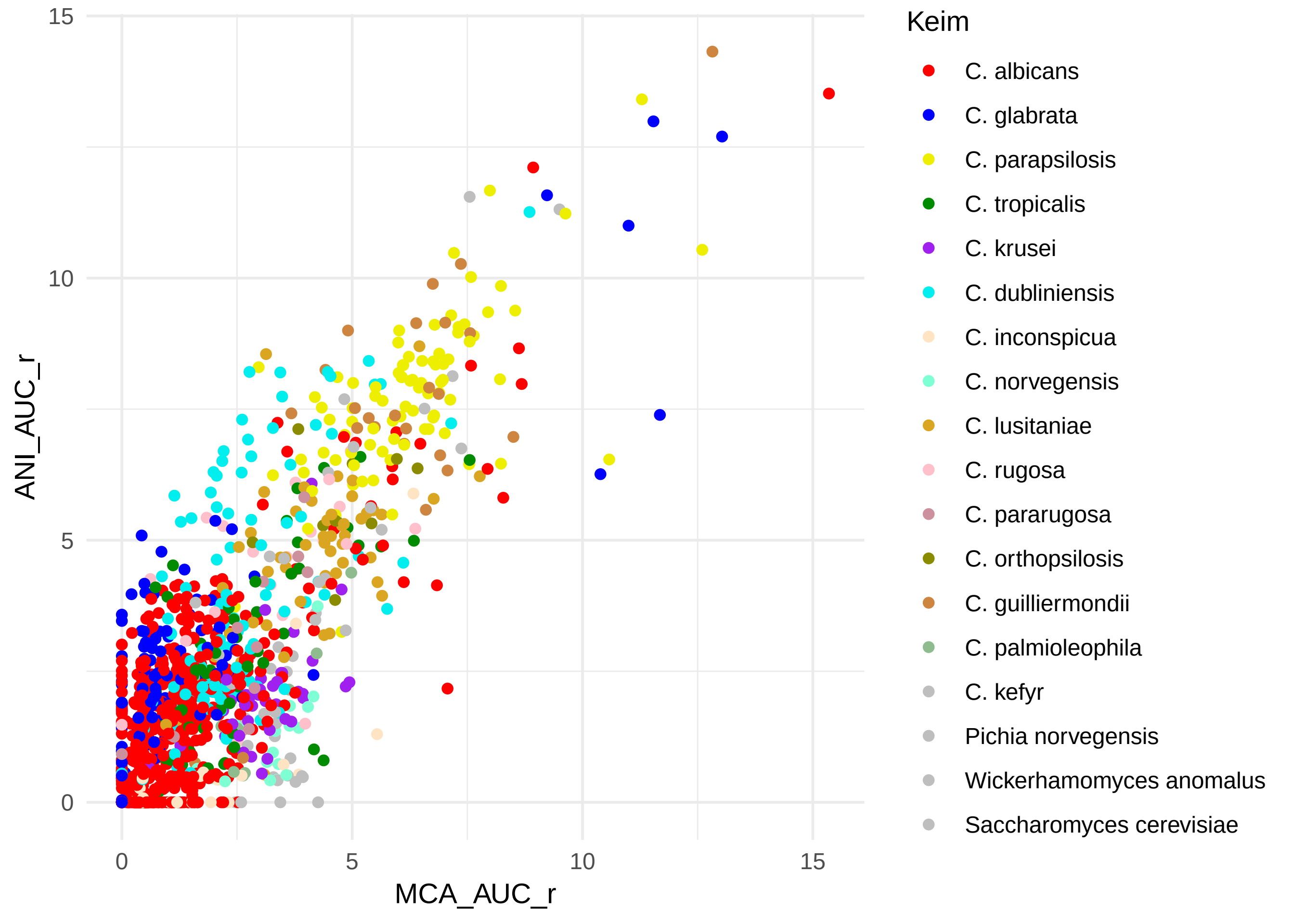
15
10
5
0
ANI_AUC_r
0
5
10
15
MCA_AUC_r
Keim
C. albicans
C. glabrata
C. parapsilosis
C. tropicalis
C. krusei
C. dubliniensis
C. inconspicua
C. norvegensis
C. lusitaniae
C. rugosa
C. pararugosa
C. orthopsilosis
C. guilliermondii
C. palmioleophila
C. kefyr
Pichia norvegensis
Wickerhamomyces anomalus
Saccharomyces cerevisiae

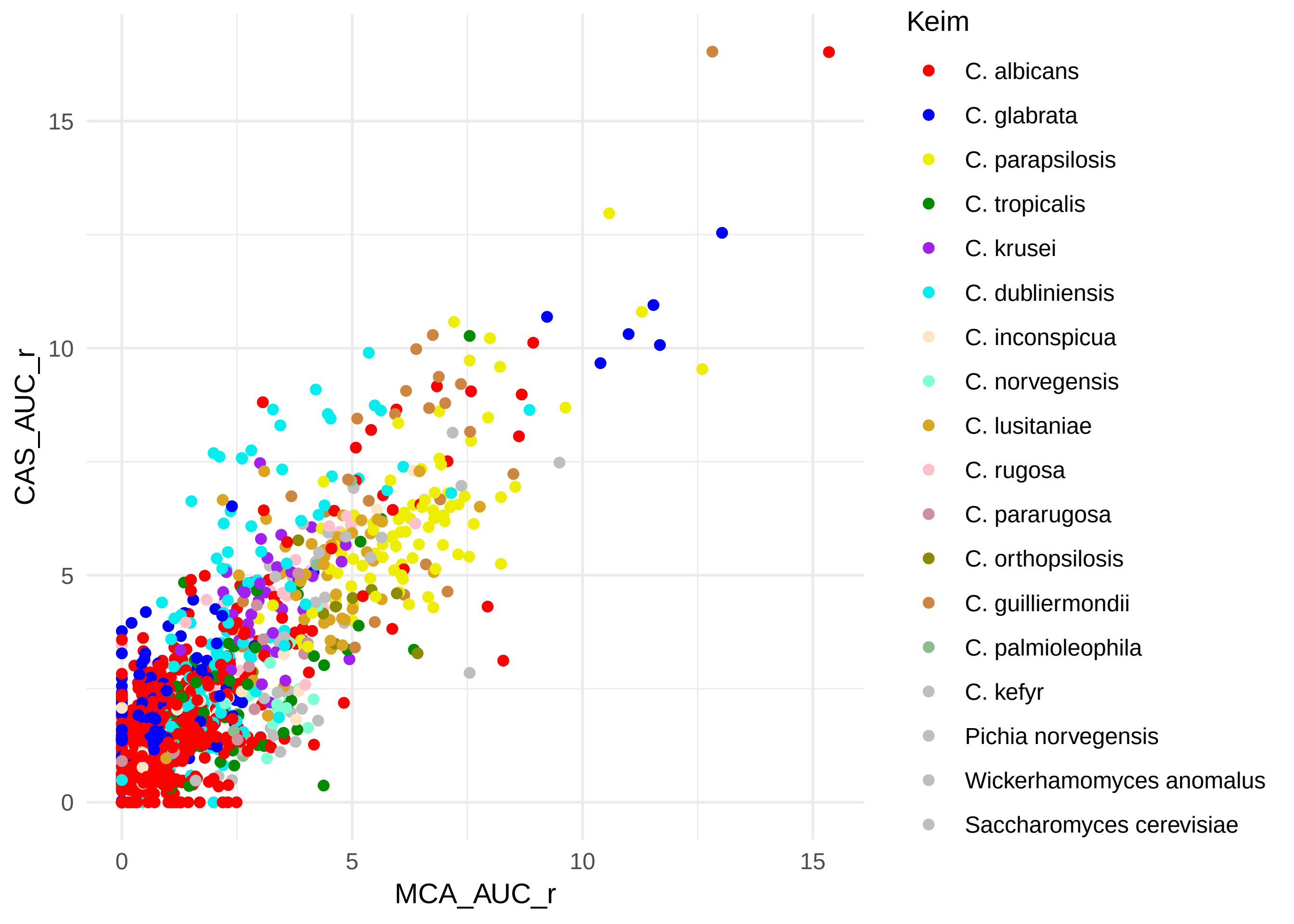
Keim
C. albicans
C. glabrata
C. parapsilosis
C. tropicalis
C. krusei
C. dubliniensis
C. inconspicua
C. norvegensis
C. lusitaniae
C. rugosa
C. pararugosa
C. orthopsilosis
C. guilliermondii
C. palmioleophila
C. kefyr
Pichia norvegensis
Wickerhamomyces anomalus
Saccharomyces cerevisiae
CAS_AUC_r
MCA_AUC_r
0
5
10
15
0
5
10
15

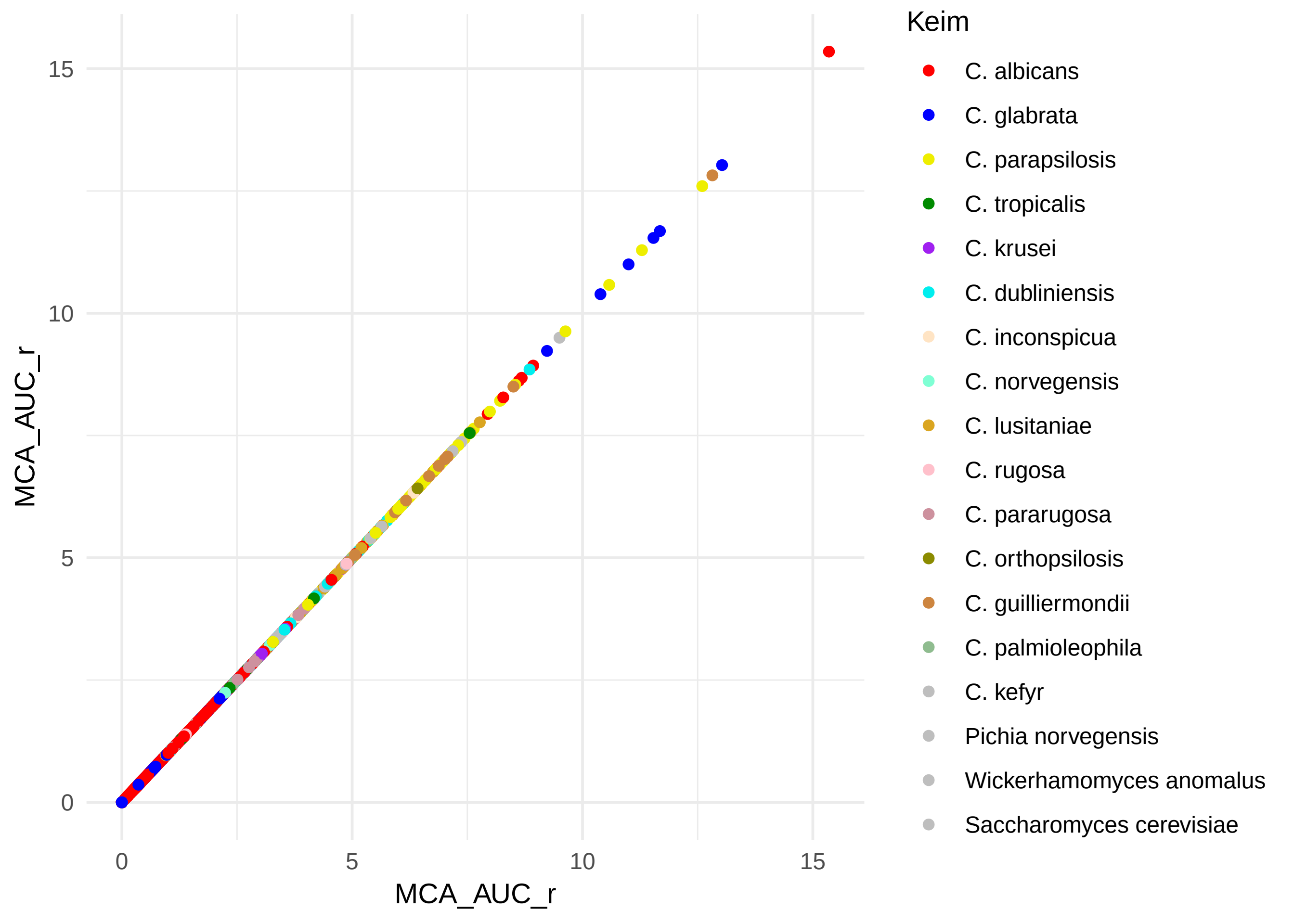

Keim
C. albicans
C. glabrata
C. parapsilosis
C. tropicalis
C. krusei
C. dubliniensis
C. inconspicua
C. norvegensis
C. lusitaniae
C. rugosa
C. pararugosa
C. orthopsilosis
C. guilliermondii
C. palmioleophila
C. kefyr
Pichia norvegensis
Wickerhamomyces anomalus
Saccharomyces cerevisiae
MCA_AUC_r
MCA_AUC_r
0
5
10
15
0
5
10
15

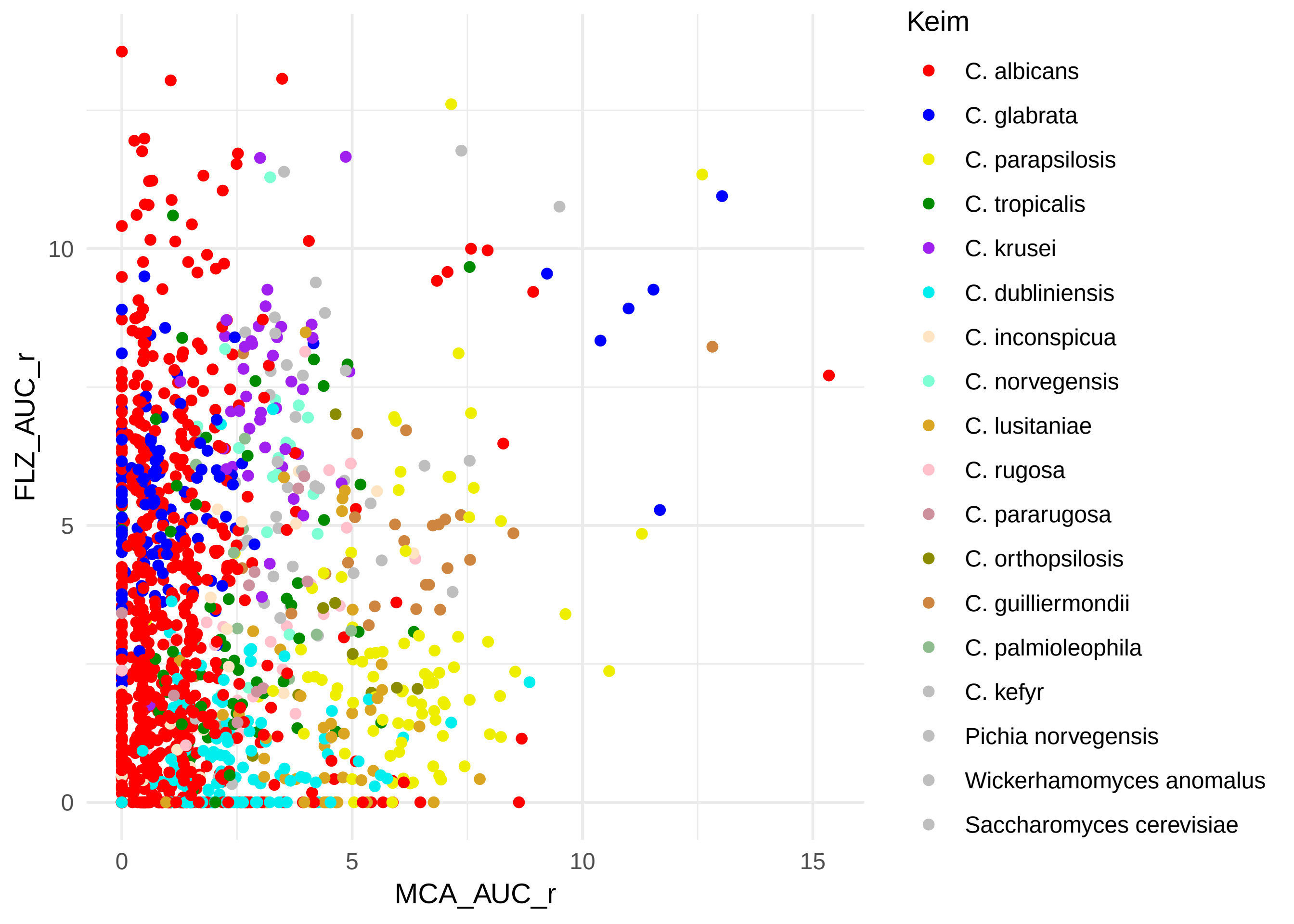
Keim
C. albicans
C. glabrata
C. parapsilosis
C. tropicalis
C. krusei
C. dubliniensis
C. inconspicua
C. norvegensis
C. lusitaniae
C. rugosa
C. pararugosa
C. orthopsilosis
C. guilliermondii
C. palmioleophila
C. kefyr
Pichia norvegensis
Wickerhamomyces anomalus
Saccharomyces cerevisiae
FLZ_AUC_r
MCA_AUC_r
0
5
10
15

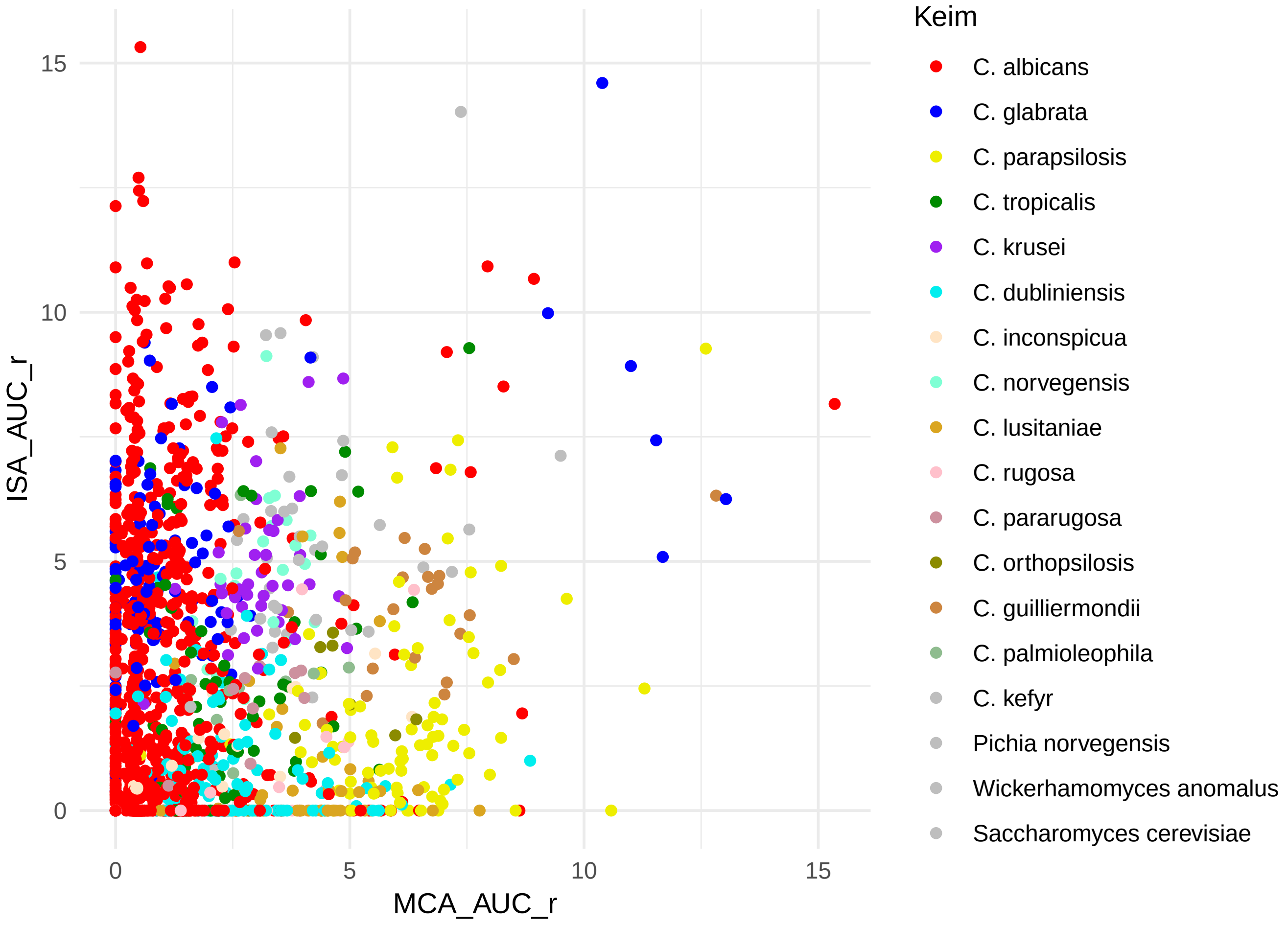

Keim
C. albicans
C. glabrata
C. parapsilosis
C. tropicalis
C. krusei
C. dubliniensis
C. inconspicua
C. norvegensis
C. lusitaniae
C. rugosa
C. pararugosa
C. orthopsilosis
C. guilliermondii
C. palmioleophila
C. kefyr
Pichia norvegensis
Wickerhamomyces anomalus
Saccharomyces cerevisiae
ISA_AUC_r
MCA_AUC_r
0
5
10
15

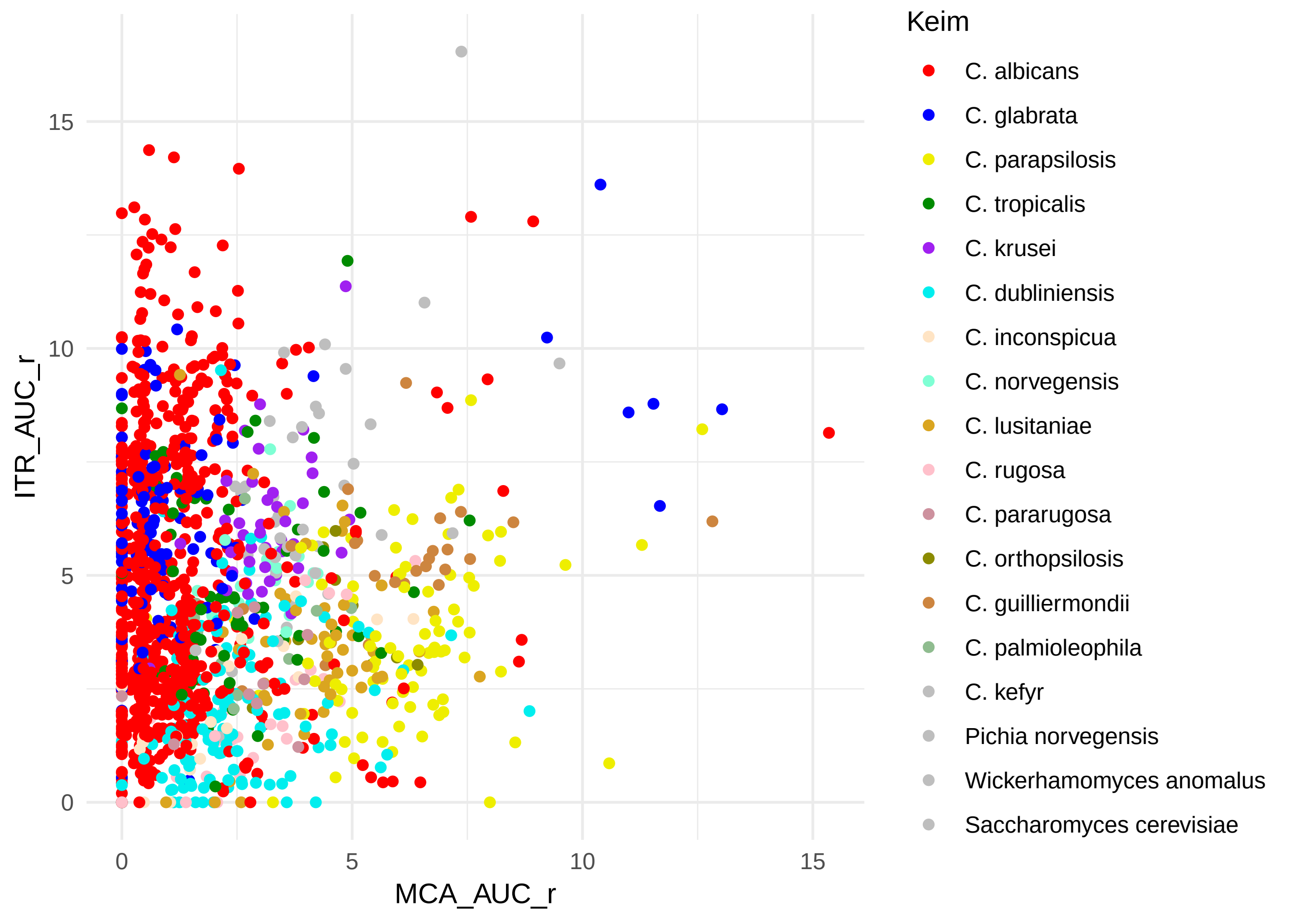
Keim
C. albicans
C. glabrata
C. parapsilosis
C. tropicalis
C. krusei
C. dubliniensis
C. inconspicua
C. norvegensis
C. lusitaniae
C. rugosa
C. pararugosa
C. orthopsilosis
C. guilliermondii
C. palmioleophila
C. kefyr
Pichia norvegensis
Wickerhamomyces anomalus
Saccharomyces cerevisiae
ITR_AUC_r
MCA_AUC_r
0
5
10
15
0
5
10
15

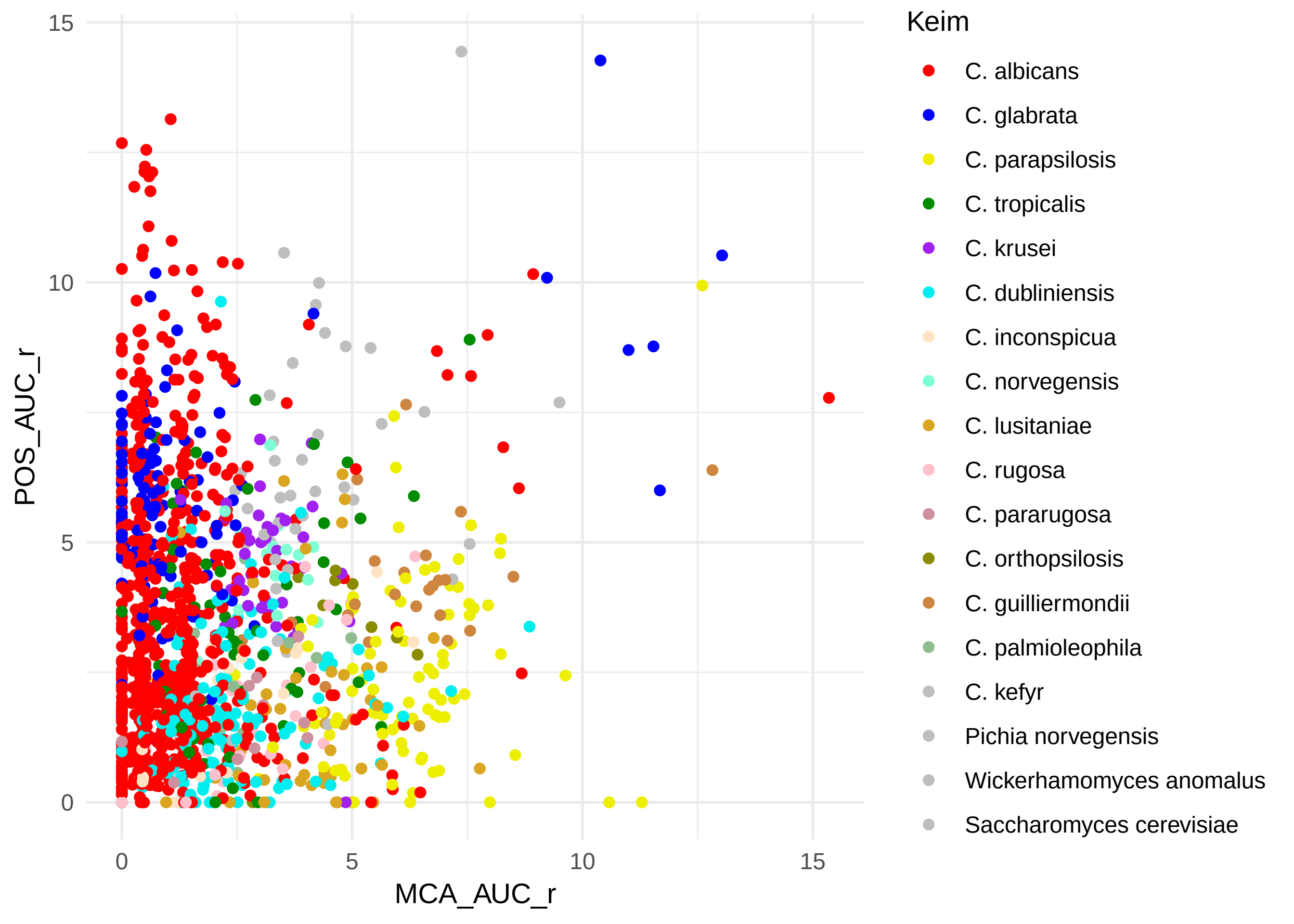
Keim
C. albicans
C. glabrata
C. parapsilosis
C. tropicalis
C. krusei
C. dubliniensis
C. inconspicua
C. norvegensis
C. lusitaniae
C. rugosa
C. pararugosa
C. orthopsilosis
C. guilliermondii
C. palmioleophila
C. kefyr
Pichia norvegensis
Wickerhamomyces anomalus
Saccharomyces cerevisiae
POS_AUC_r
MCA_AUC_r
0
5
10
15

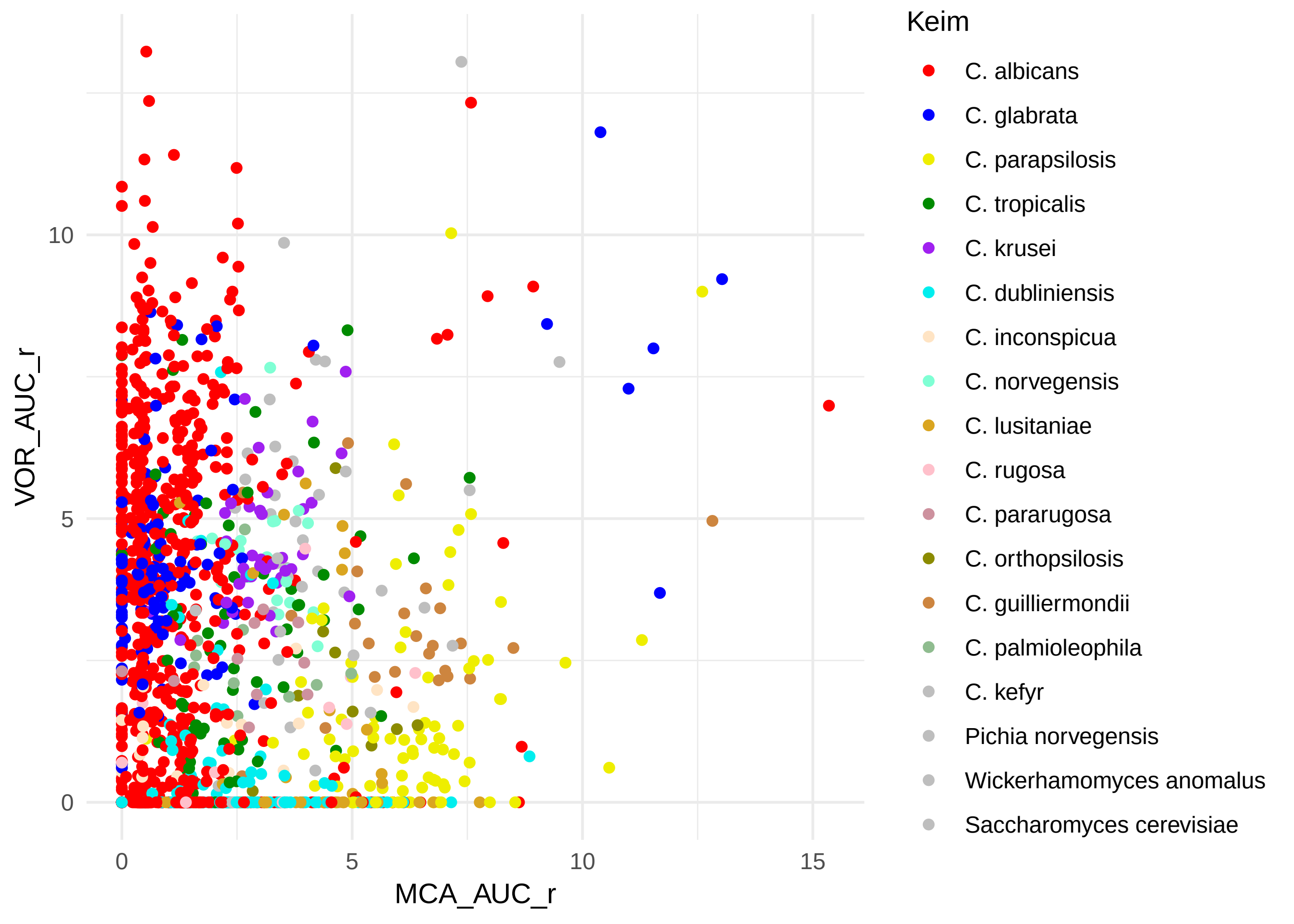

Keim
C. albicans
C. glabrata
C. parapsilosis
C. tropicalis
C. krusei
C. dubliniensis
C. inconspicua
C. norvegensis
C. lusitaniae
C. rugosa
C. pararugosa
C. orthopsilosis
C. guilliermondii
C. palmioleophila
C. kefyr
Pichia norvegensis
Wickerhamomyces anomalus
Saccharomyces cerevisiae
VOR_AUC_r
MCA_AUC_r
0
5
10
15

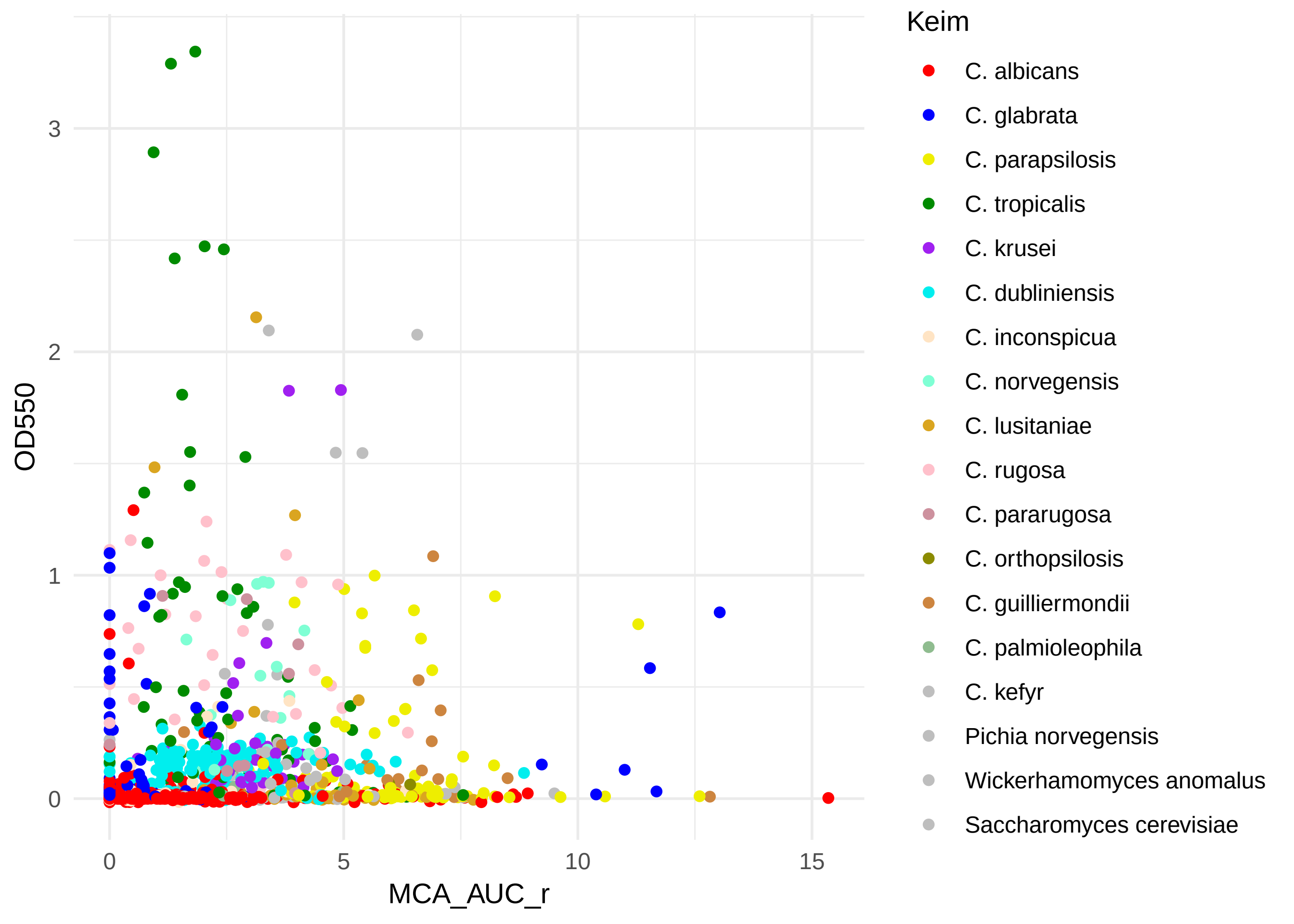

OD550
3
2
1
0
0
5
10
15
MCA_AUC_r
Keim
C. albicans
C. glabrata
C. parapsilosis
C. tropicalis
C. krusei
C. dubliniensis
C. inconspicua
C. norvegensis
C. lusitaniae
C. rugosa
C. pararugosa
C. orthopsilosis
C. guilliermondii
C. palmioleophila
C. kefyr
Pichia norvegensis
Wickerhamomyces anomalus
Saccharomyces cerevisiae

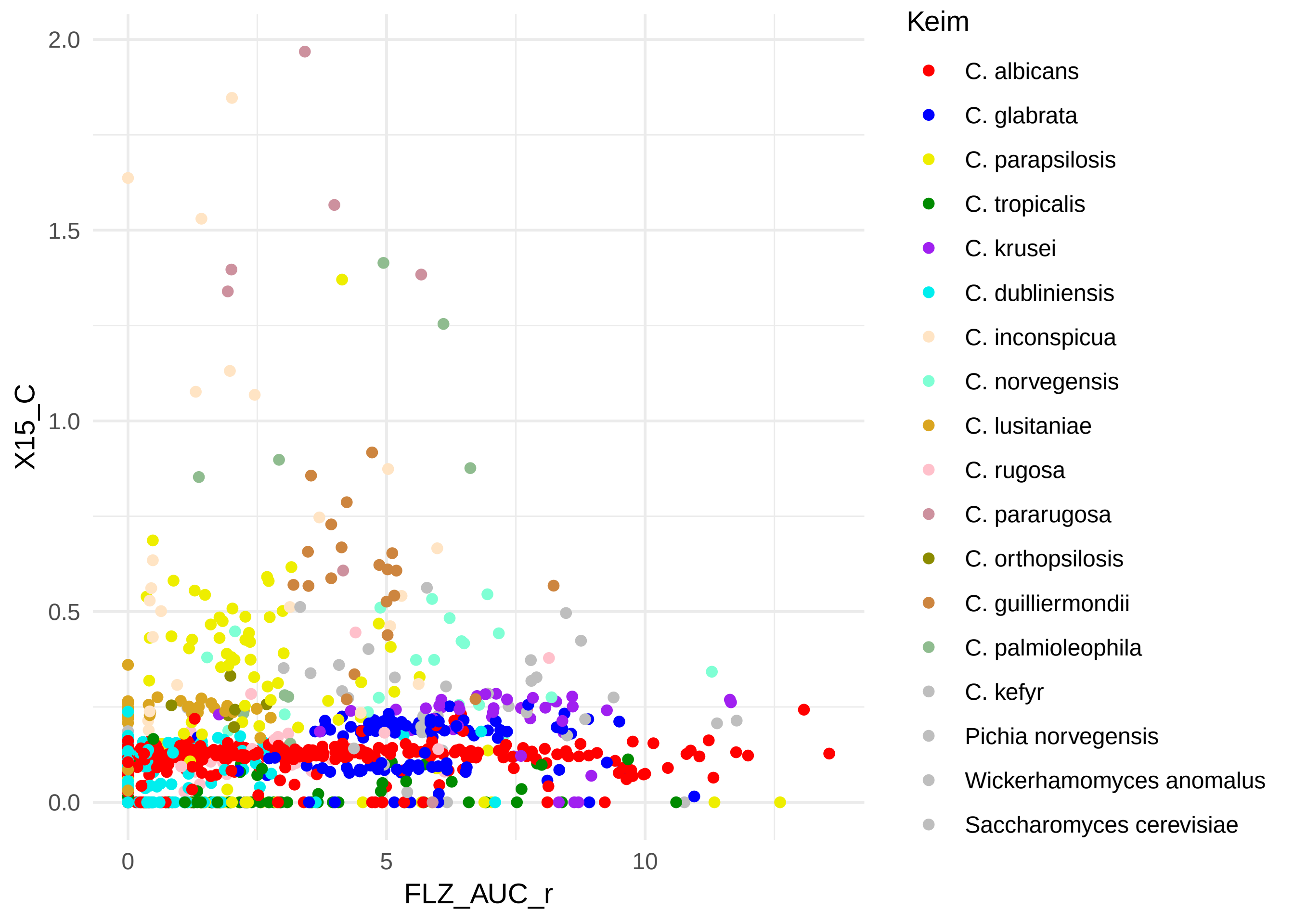

Keim
C. albicans
C. glabrata
C. parapsilosis
C. tropicalis
C. krusei
C. dubliniensis
C. inconspicua
C. norvegensis
C. lusitaniae
C. rugosa
C. pararugosa
C. orthopsilosis
C. guilliermondii
C. palmioleophila
C. kefyr
Pichia norvegensis
Wickerhamomyces anomalus
Saccharomyces cerevisiae
X15_C
2.0
1.5
1.0
0.5
0.0
0
5
10
FLZ_AUC_r

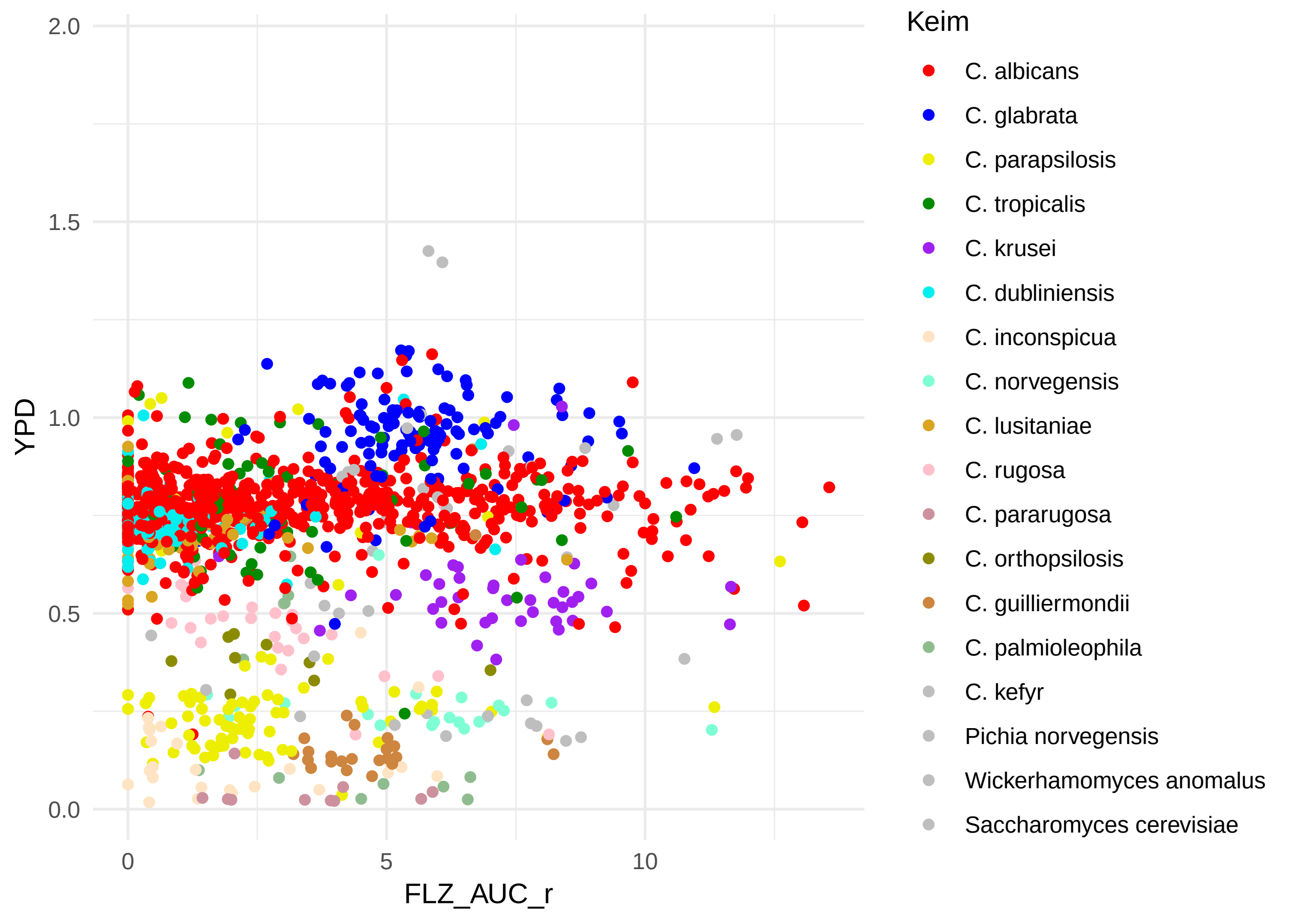

YPD
2.0
1.5
1.0
0.5
0.0
0
5
10
FLZ_AUC_r
Keim
C. albicans
C. glabrata
C. parapsilosis
C. tropicalis
C. krusei
C. dubliniensis
C. inconspicua
C. norvegensis
C. lusitaniae
C. rugosa
C. pararugosa
C. orthopsilosis
C. guilliermondii
C. palmioleophila
C. kefyr
Pichia norvegensis
Wickerhamomyces anomalus
Saccharomyces cerevisiae

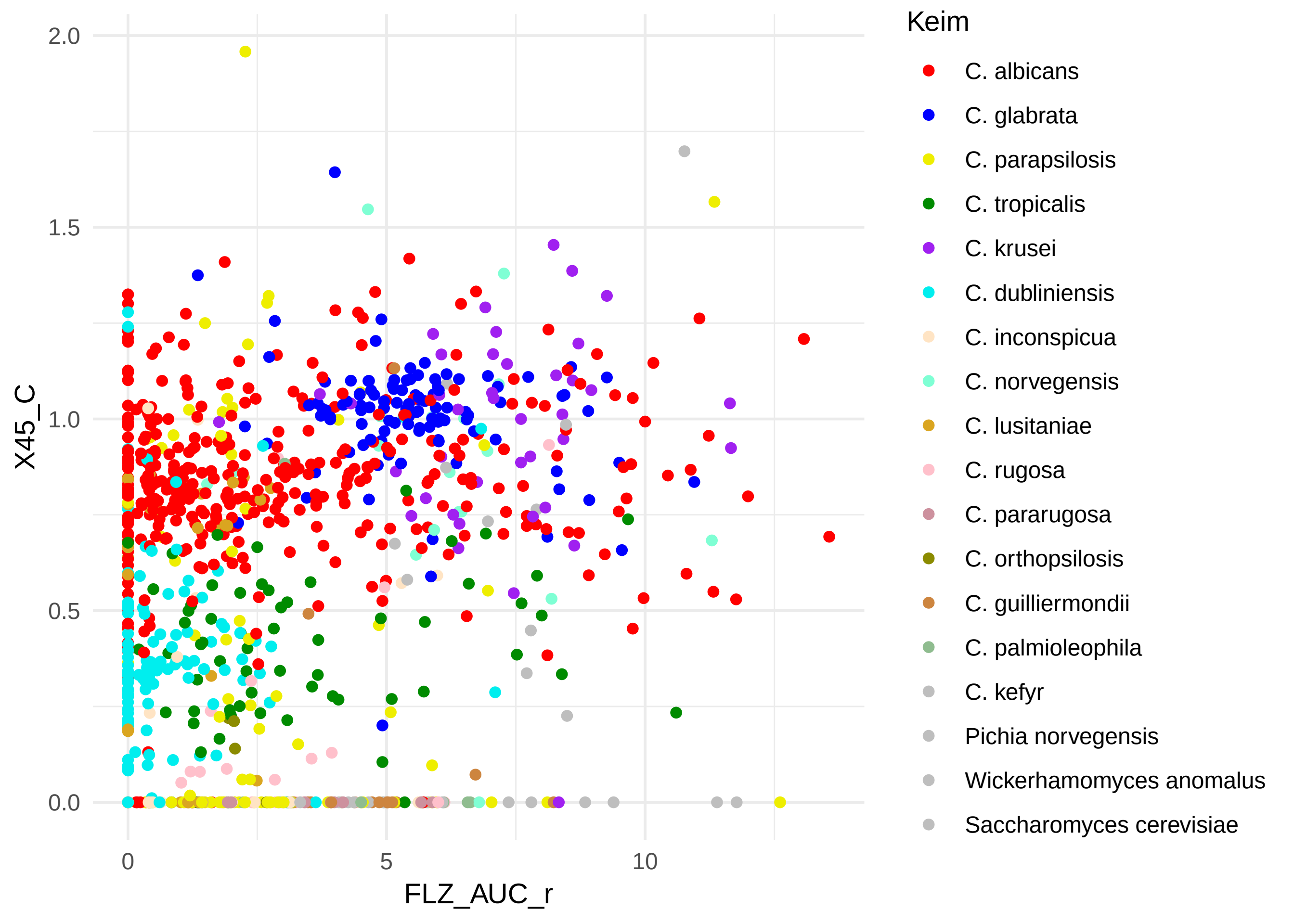
Keim
C. albicans
C. glabrata
C. parapsilosis
C. tropicalis
C. krusei
C. dubliniensis
C. inconspicua
C. norvegensis
C. lusitaniae
C. rugosa
C. pararugosa
C. orthopsilosis
C. guilliermondii
C. palmioleophila
C. kefyr
Pichia norvegensis
Wickerhamomyces anomalus
Saccharomyces cerevisiae
X45_C
2.0
1.5
1.0
0.5
0.0
0
5
10
FLZ_AUC_r

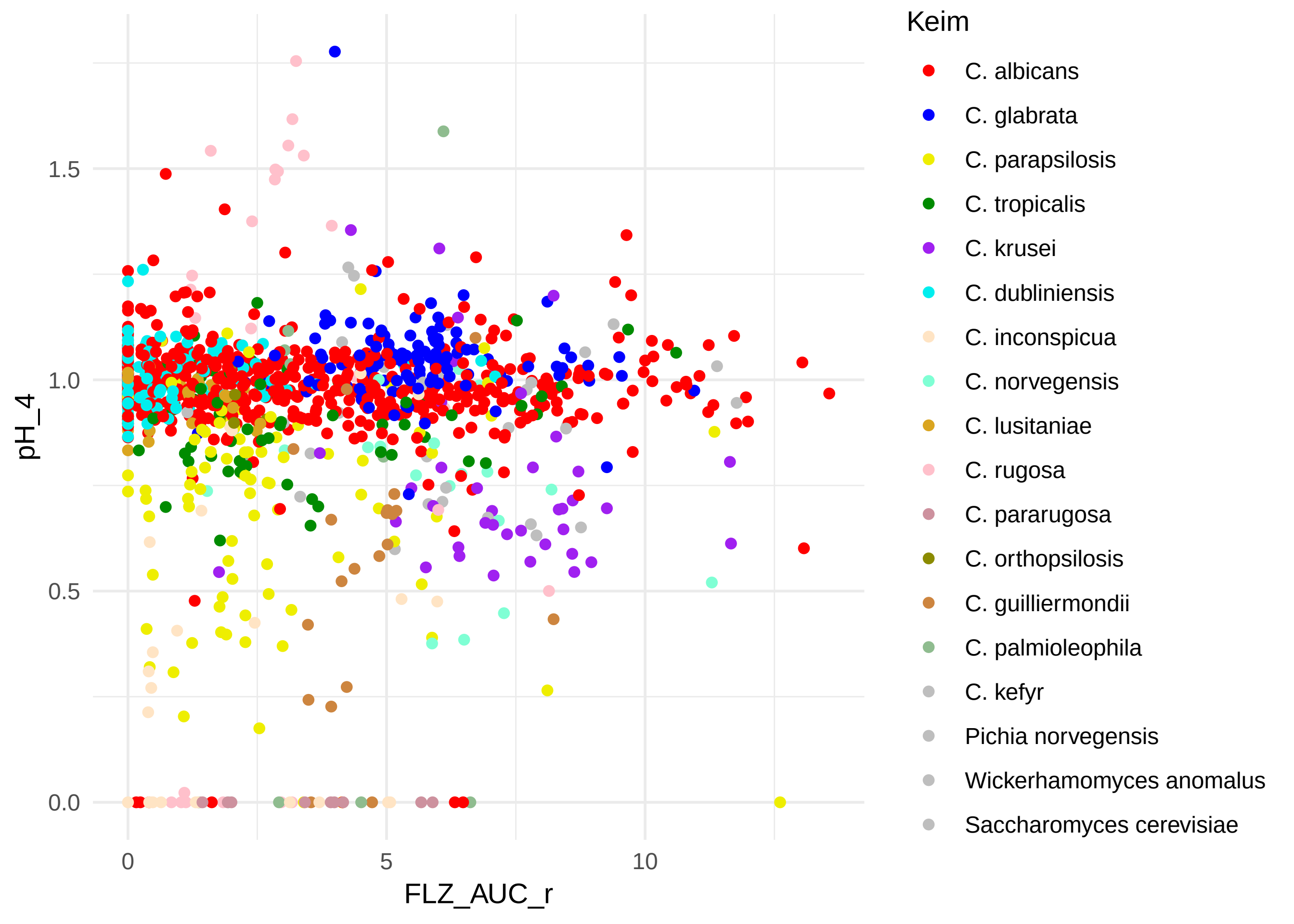
Keim
C. albicans
C. glabrata
C. parapsilosis
C. tropicalis
C. krusei
C. dubliniensis
C. inconspicua
C. norvegensis
C. lusitaniae
C. rugosa
C. pararugosa
C. orthopsilosis
C. guilliermondii
C. palmioleophila
C. kefyr
Pichia norvegensis
Wickerhamomyces anomalus
Saccharomyces cerevisiae
pH_4
FLZ_AUC_r
0.0
0.5
1.0
1.5
0
5
10

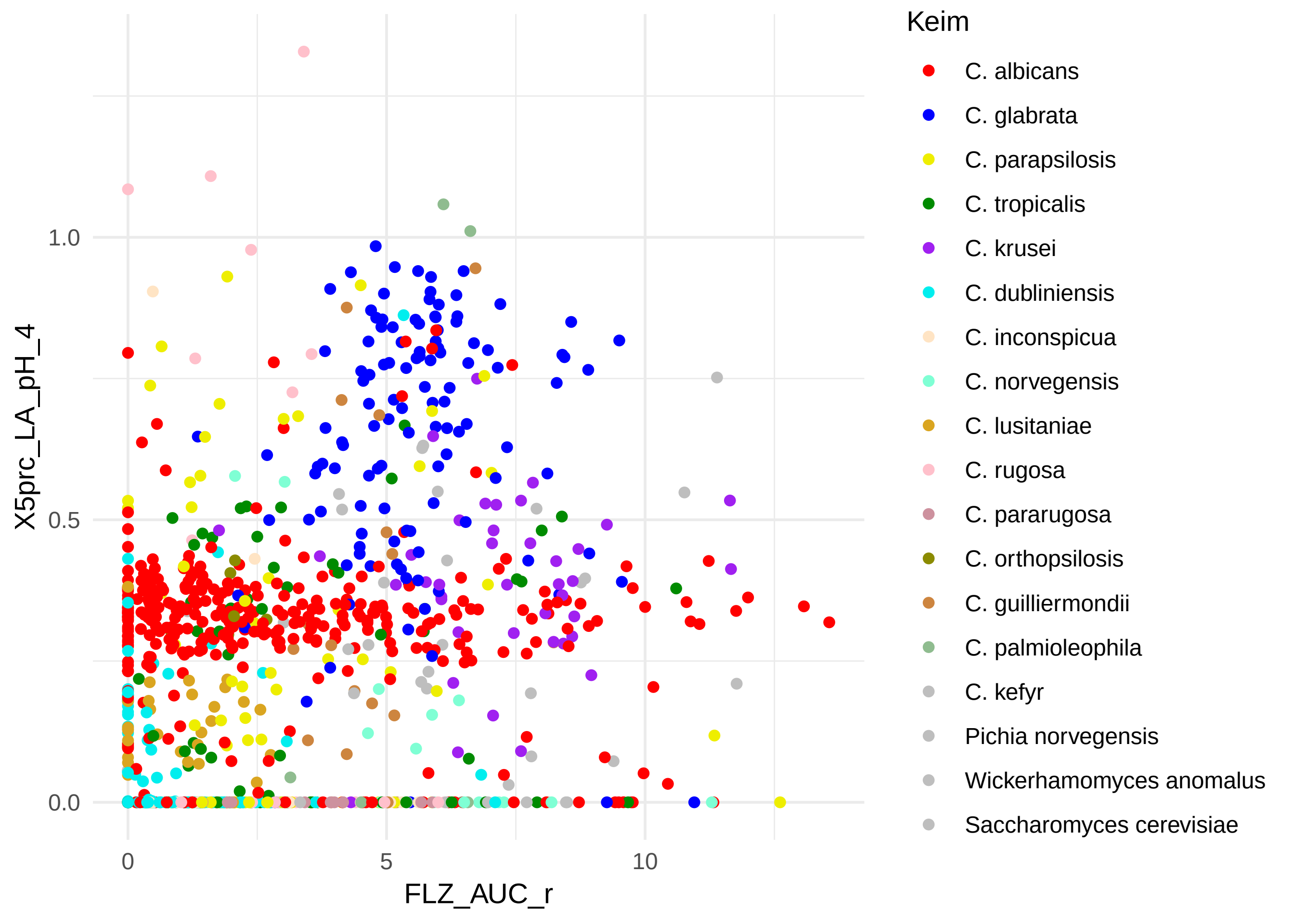

Keim
C. albicans
C. glabrata
C. parapsilosis
C. tropicalis
C. krusei
C. dubliniensis
C. inconspicua
C. norvegensis
C. lusitaniae
C. rugosa
C. pararugosa
C. orthopsilosis
C. guilliermondii
C. palmioleophila
C. kefyr
Pichia norvegensis
Wickerhamomyces anomalus
Saccharomyces cerevisiae
X5prc_LA_pH_4
1.0
0.5
0.0
0
5
10
FLZ_AUC_r

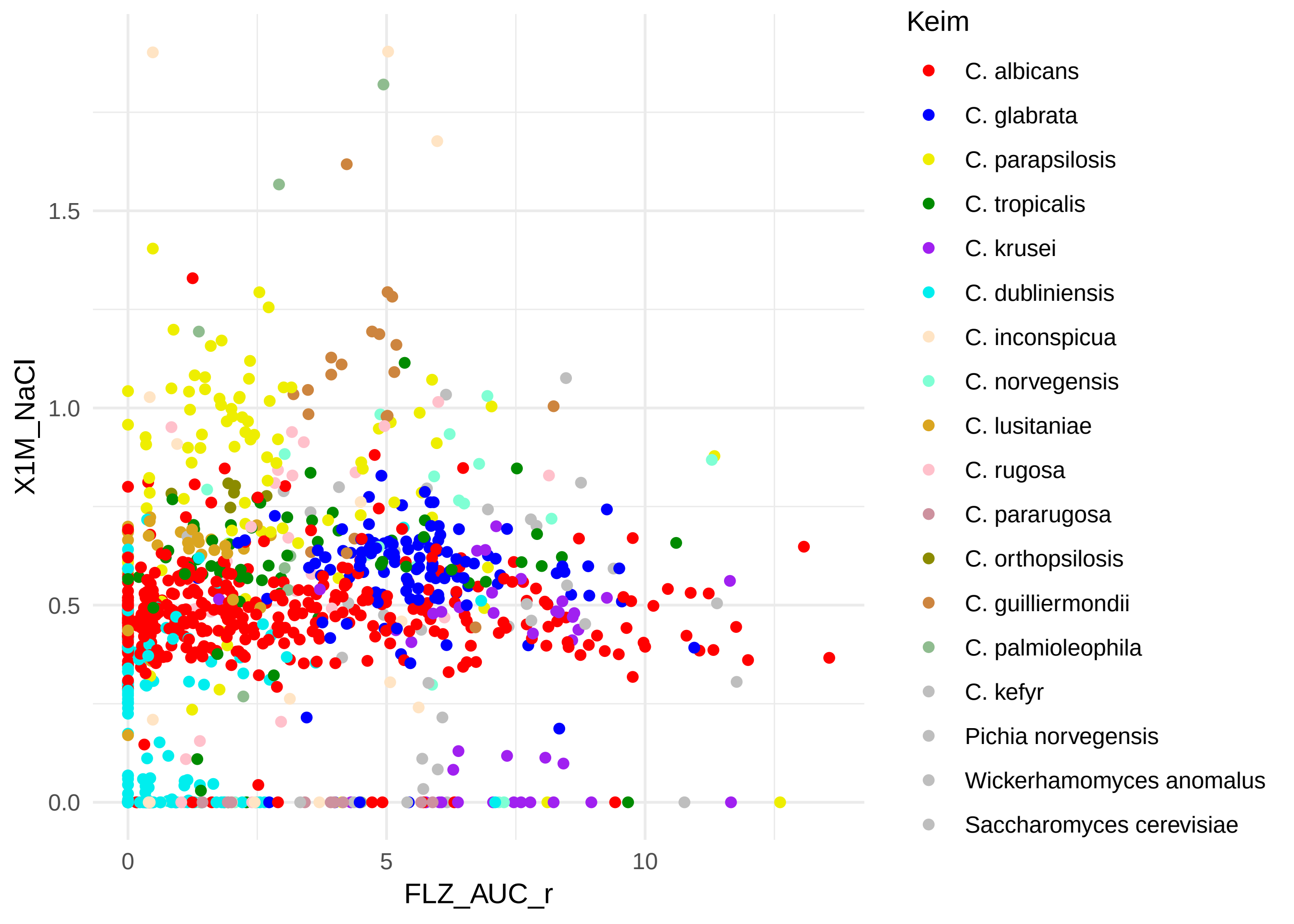

Keim
C. albicans
C. glabrata
C. parapsilosis
C. tropicalis
C. krusei
C. dubliniensis
C. inconspicua
C. norvegensis
C. lusitaniae
C. rugosa
C. pararugosa
C. orthopsilosis
C. guilliermondii
C. palmioleophila
C. kefyr
Pichia norvegensis
Wickerhamomyces anomalus
Saccharomyces cerevisiae
X1M_NaCl
1.5
1.0
0.5
0.0
0
5
10
FLZ_AUC_r

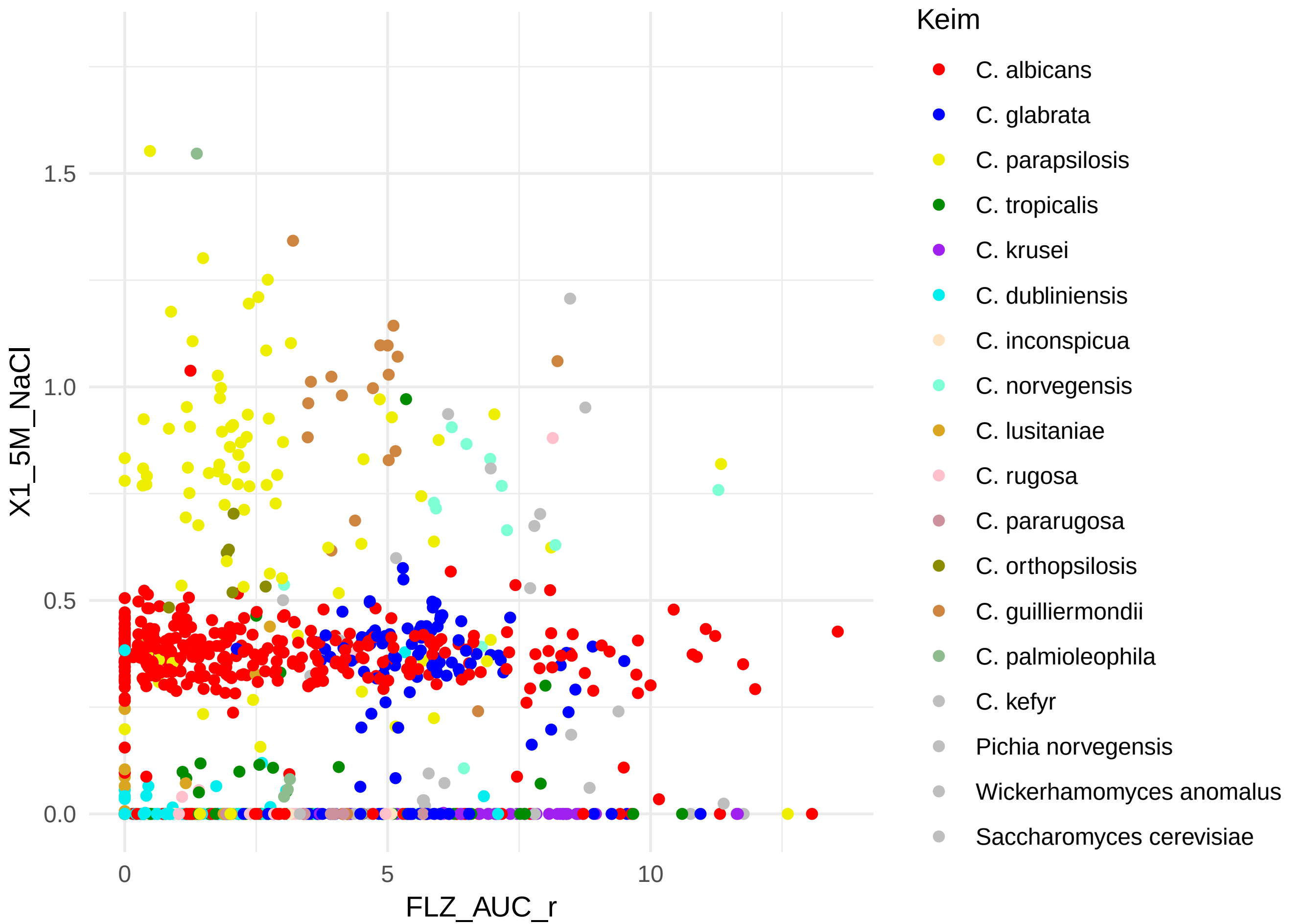
X1_5M_NaCl
FLZ_AUC_r
0.0
0.5
1.0
1.5
0
5
10
Keim
C. albicans
C. glabrata
C. parapsilosis
C. tropicalis
C. krusei
C. dubliniensis
C. inconspicua
C. norvegensis
C. lusitaniae
C. rugosa
C. pararugosa
C. orthopsilosis
C. guilliermondii
C. palmioleophila
C. kefyr
Pichia norvegensis
Wickerhamomyces anomalus
Saccharomyces cerevisiae

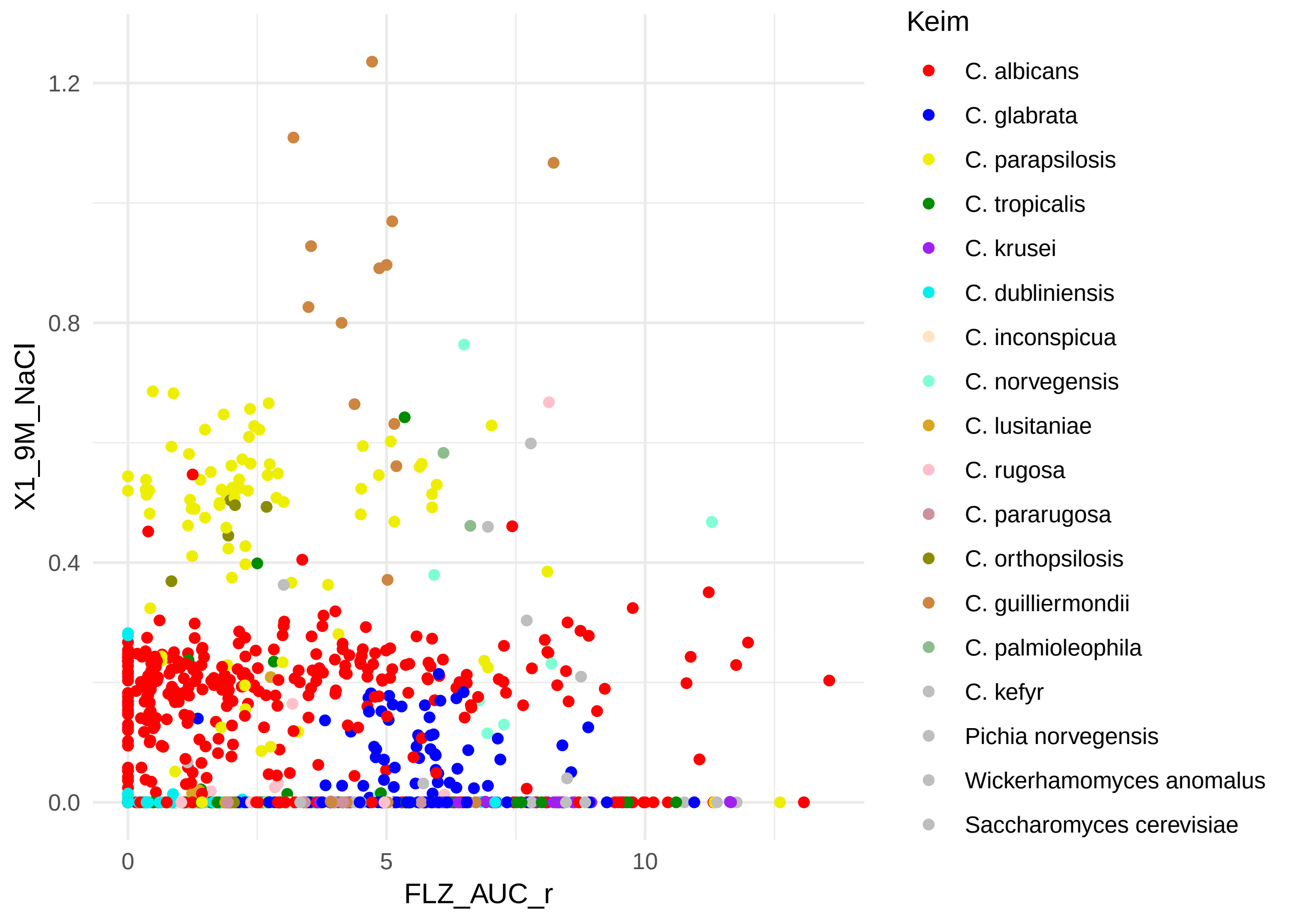
Keim
C. albicans
C. glabrata
C. parapsilosis
C. tropicalis
C. krusei
C. dubliniensis
C. inconspicua
C. norvegensis
C. lusitaniae
C. rugosa
C. pararugosa
C. orthopsilosis
C. guilliermondii
C. palmioleophila
C. kefyr
Pichia norvegensis
Wickerhamomyces anomalus
Saccharomyces cerevisiae
X1_9M_NaCl
1.2
0.8
0.4
0.0
0
5
10
FLZ_AUC_r

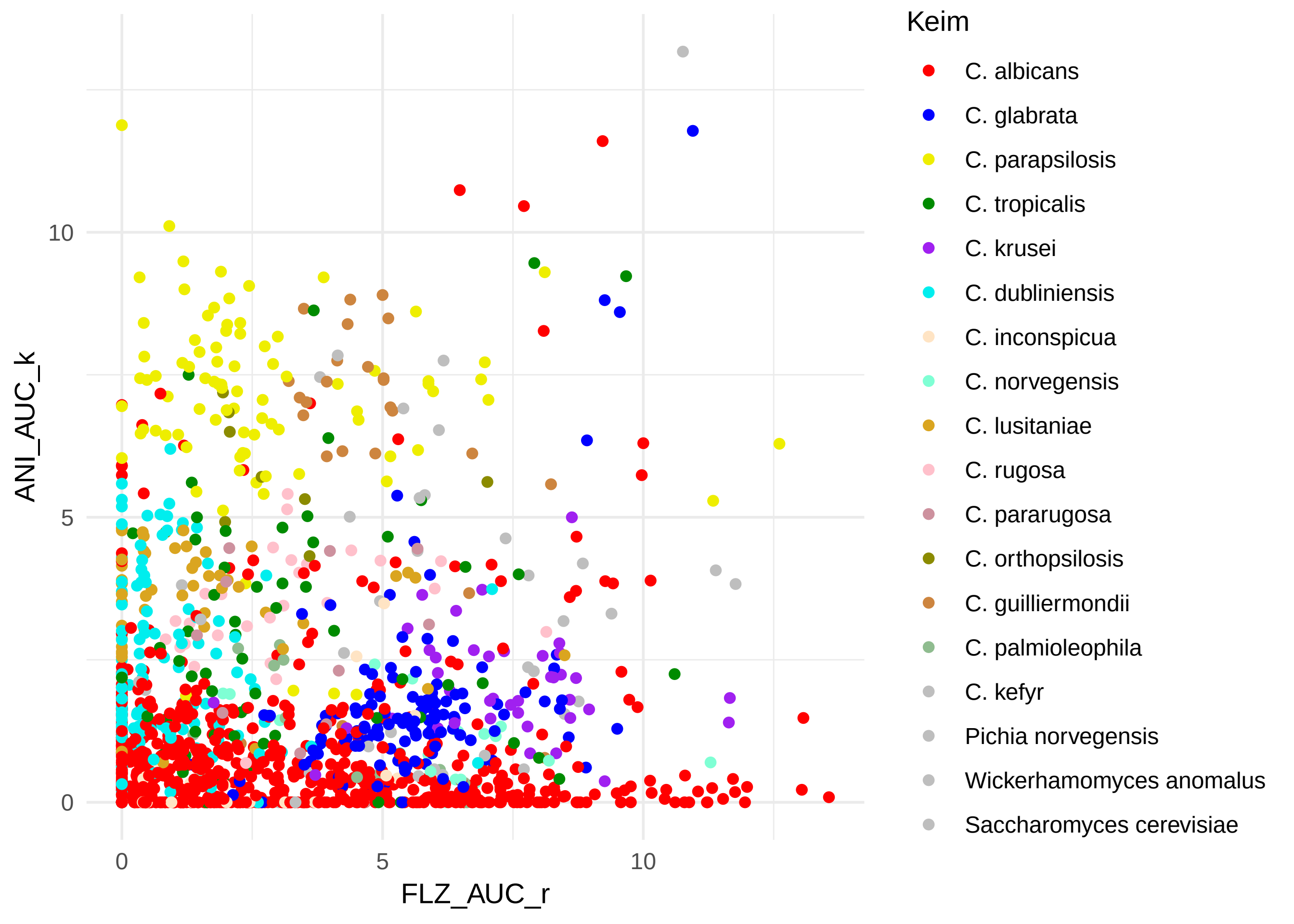

Keim
C. albicans
C. glabrata
C. parapsilosis
C. tropicalis
C. krusei
C. dubliniensis
C. inconspicua
C. norvegensis
C. lusitaniae
C. rugosa
C. pararugosa
C. orthopsilosis
C. guilliermondii
C. palmioleophila
C. kefyr
Pichia norvegensis
Wickerhamomyces anomalus
Saccharomyces cerevisiae
ANI_AUC_k
10
5
0
FLZ_AUC_r
0
5
10

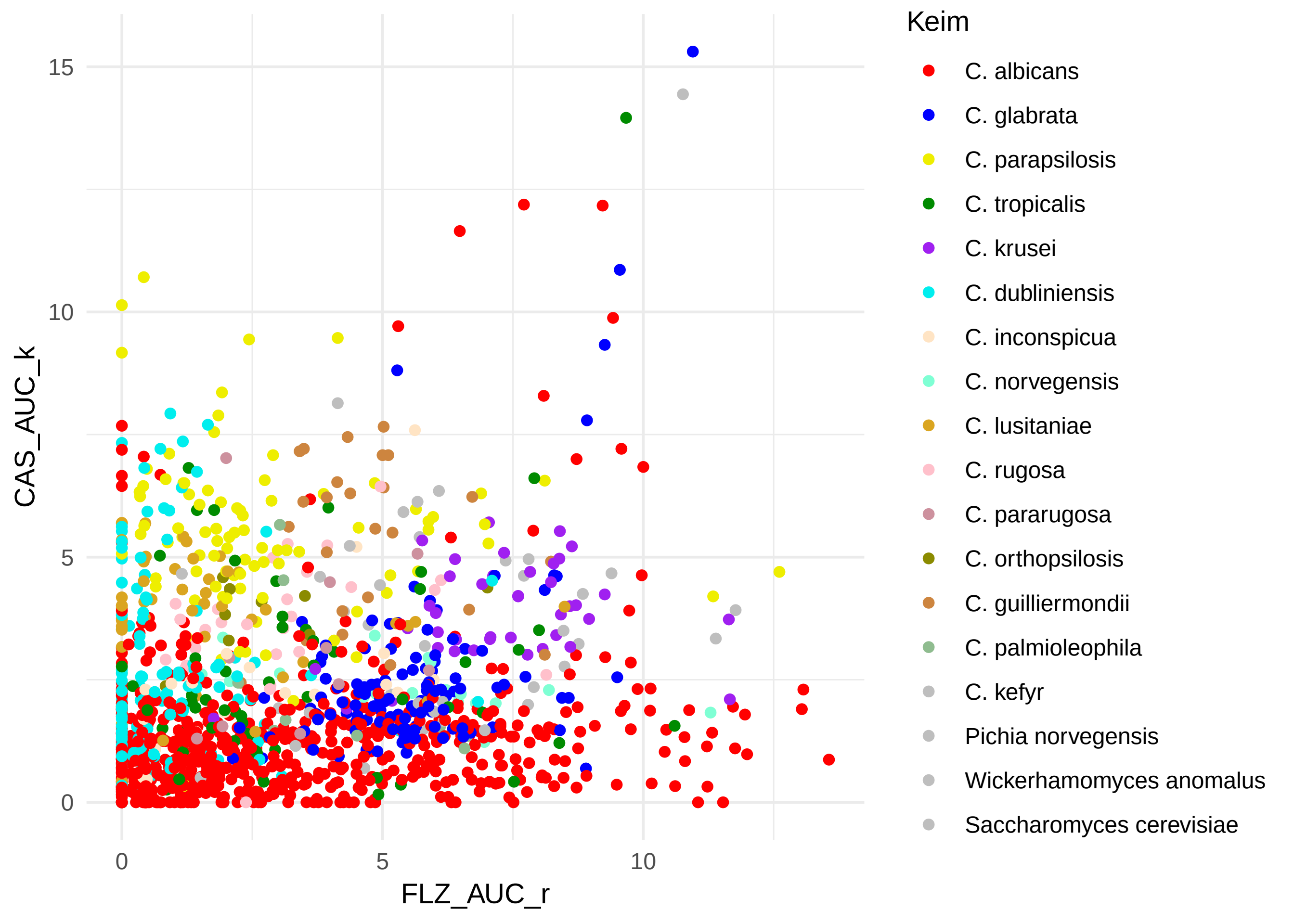

Keim
C. albicans
C. glabrata
C. parapsilosis
C. tropicalis
C. krusei
C. dubliniensis
C. inconspicua
C. norvegensis
C. lusitaniae
C. rugosa
C. pararugosa
C. orthopsilosis
C. guilliermondii
C. palmioleophila
C. kefyr
Pichia norvegensis
Wickerhamomyces anomalus
Saccharomyces cerevisiae
CAS_AUC_k
FLZ_AUC_r
0
5
10
15

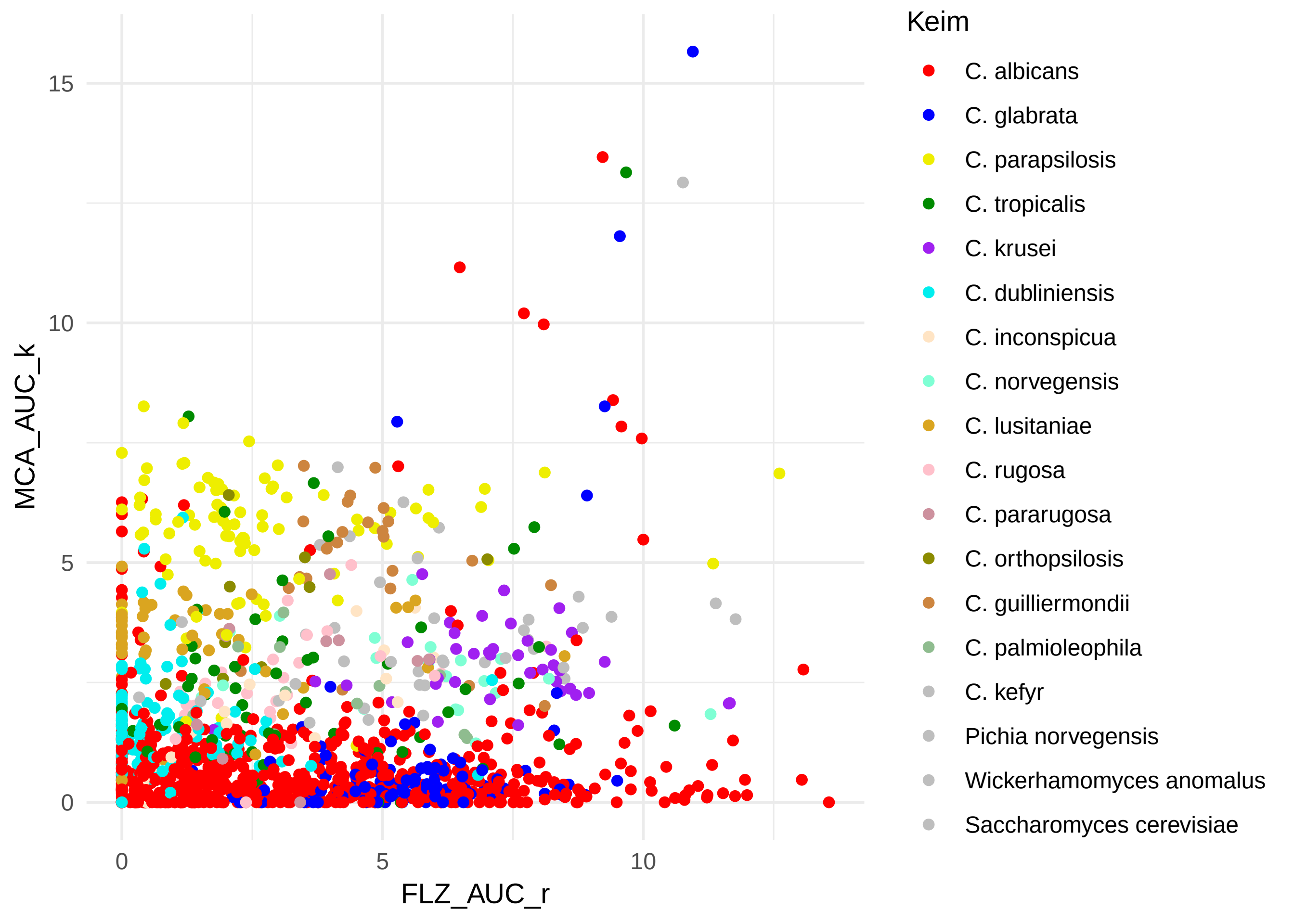

Keim
C. albicans
C. glabrata
C. parapsilosis
C. tropicalis
C. krusei
C. dubliniensis
C. inconspicua
C. norvegensis
C. lusitaniae
C. rugosa
C. pararugosa
C. orthopsilosis
C. guilliermondii
C. palmioleophila
C. kefyr
Pichia norvegensis
Wickerhamomyces anomalus
Saccharomyces cerevisiae
MCA_AUC_k
FLZ_AUC_r
0
5
10
15

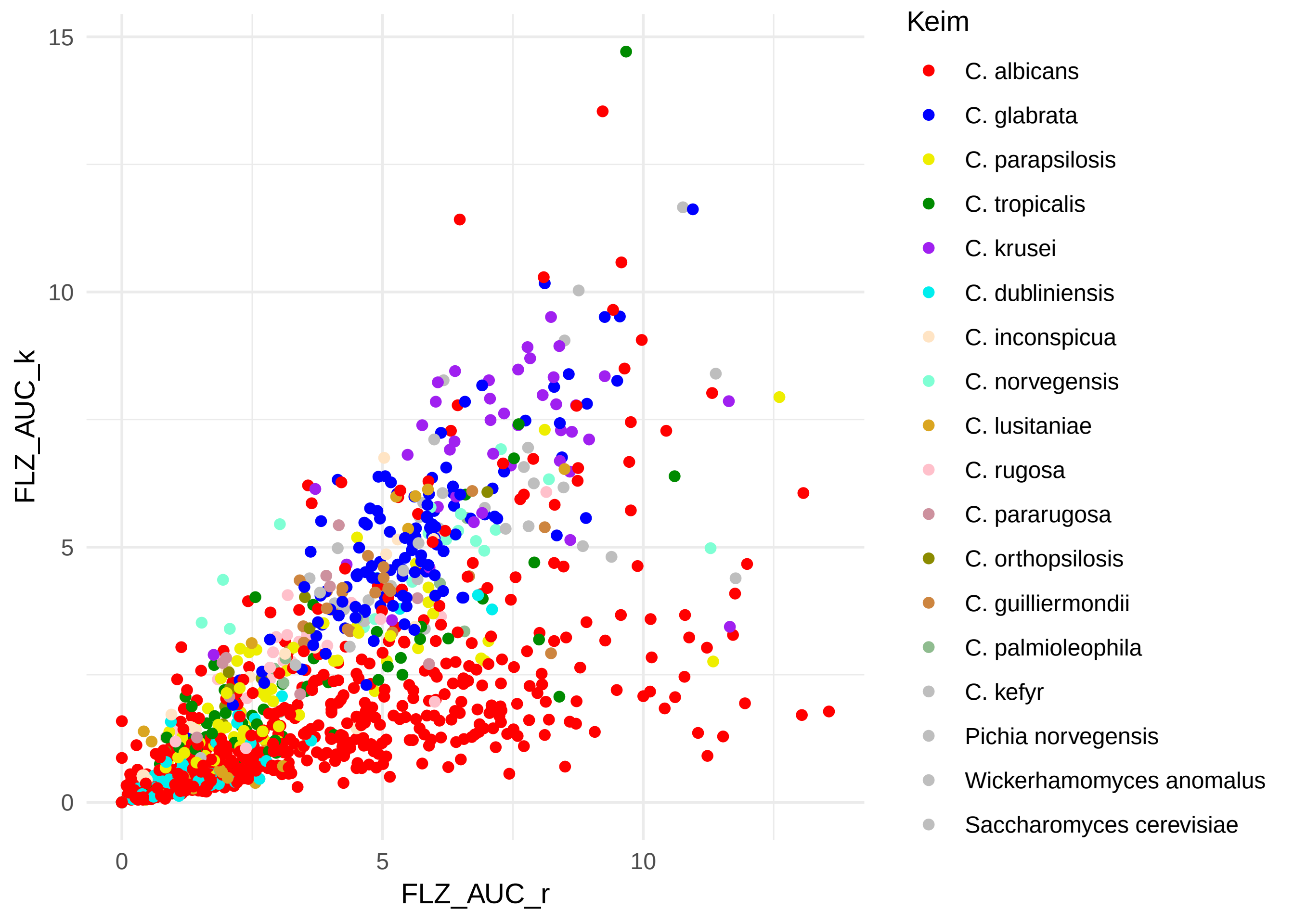

Keim
C. albicans
C. glabrata
C. parapsilosis
C. tropicalis
C. krusei
C. dubliniensis
C. inconspicua
C. norvegensis
C. lusitaniae
C. rugosa
C. pararugosa
C. orthopsilosis
C. guilliermondii
C. palmioleophila
C. kefyr
Pichia norvegensis
Wickerhamomyces anomalus
Saccharomyces cerevisiae
FLZ_AUC_k
FLZ_AUC_r
0
5
10
15

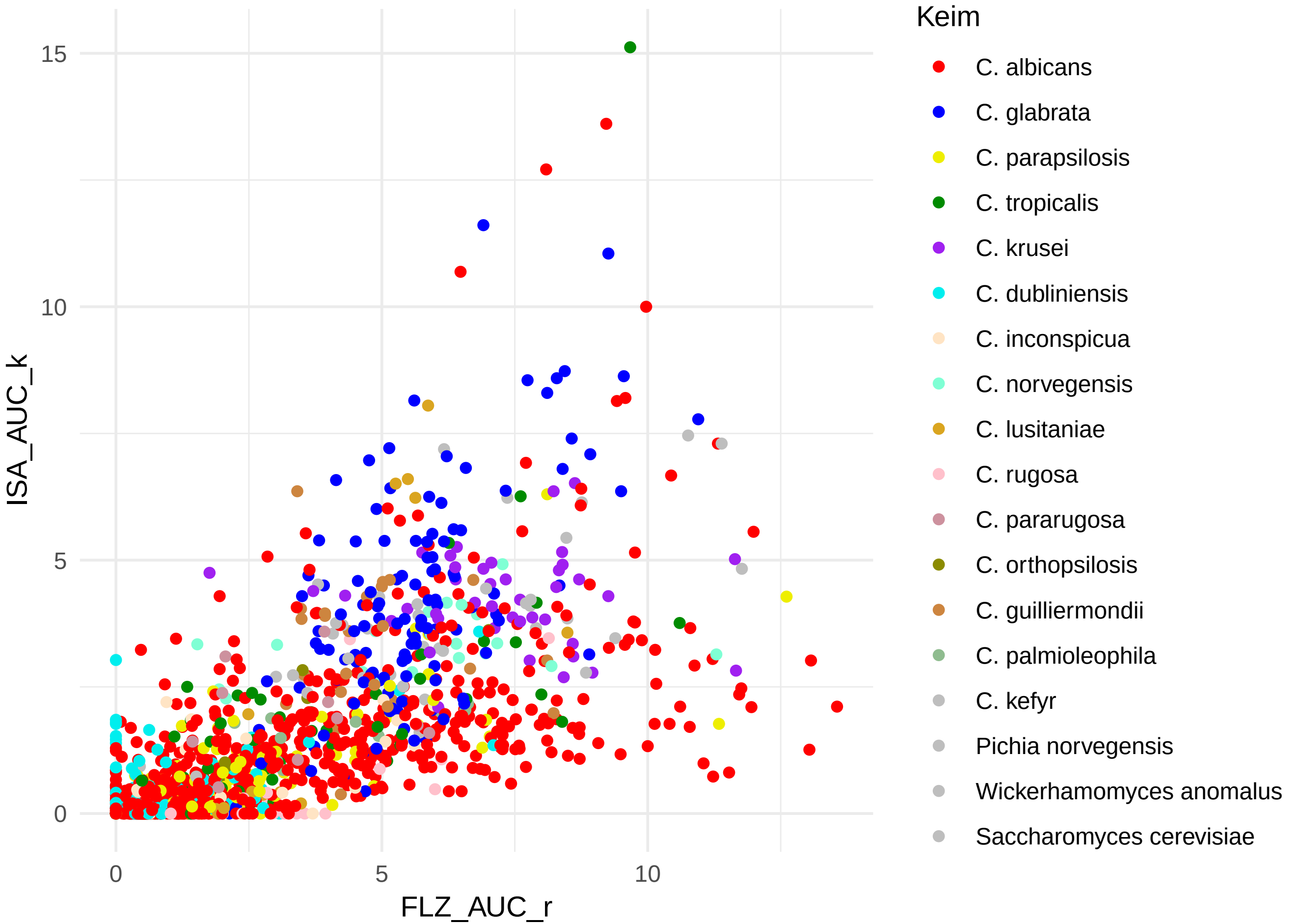
Keim
C. albicans
C. glabrata
C. parapsilosis
C. tropicalis
C. krusei
C. dubliniensis
C. inconspicua
C. norvegensis
C. lusitaniae
C. rugosa
C. pararugosa
C. orthopsilosis
C. guilliermondii
C. palmioleophila
C. kefyr
Pichia norvegensis
Wickerhamomyces anomalus
Saccharomyces cerevisiae
ISA_AUC_k
FLZ_AUC_r
15
10
5
0
0
5
10

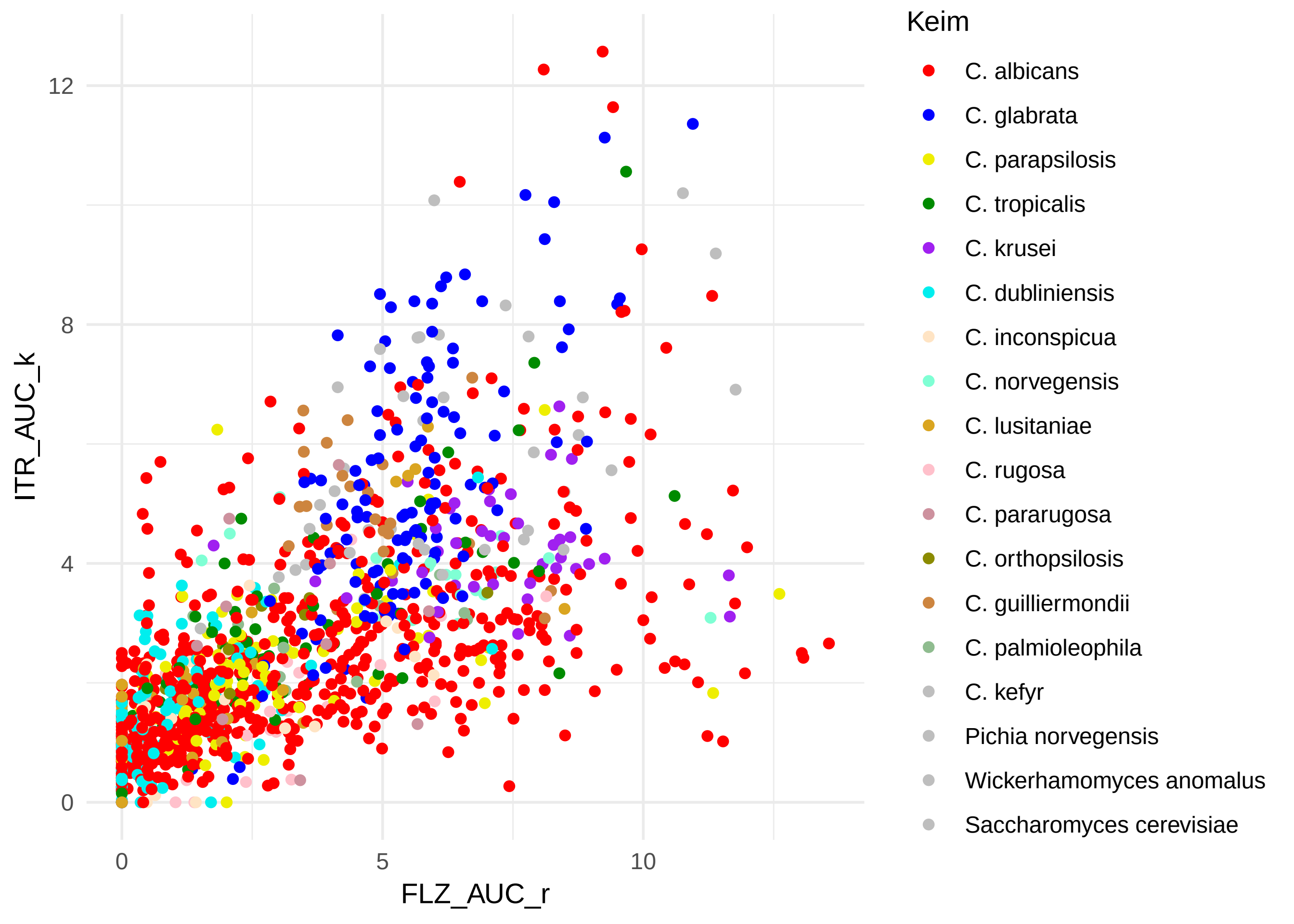

Keim
C. albicans
C. glabrata
C. parapsilosis
C. tropicalis
C. krusei
C. dubliniensis
C. inconspicua
C. norvegensis
C. lusitaniae
C. rugosa
C. pararugosa
C. orthopsilosis
C. guilliermondii
C. palmioleophila
C. kefyr
Pichia norvegensis
Wickerhamomyces anomalus
Saccharomyces cerevisiae
ITR_AUC_k
FLZ_AUC_r
0
4
8
12
0
5
10

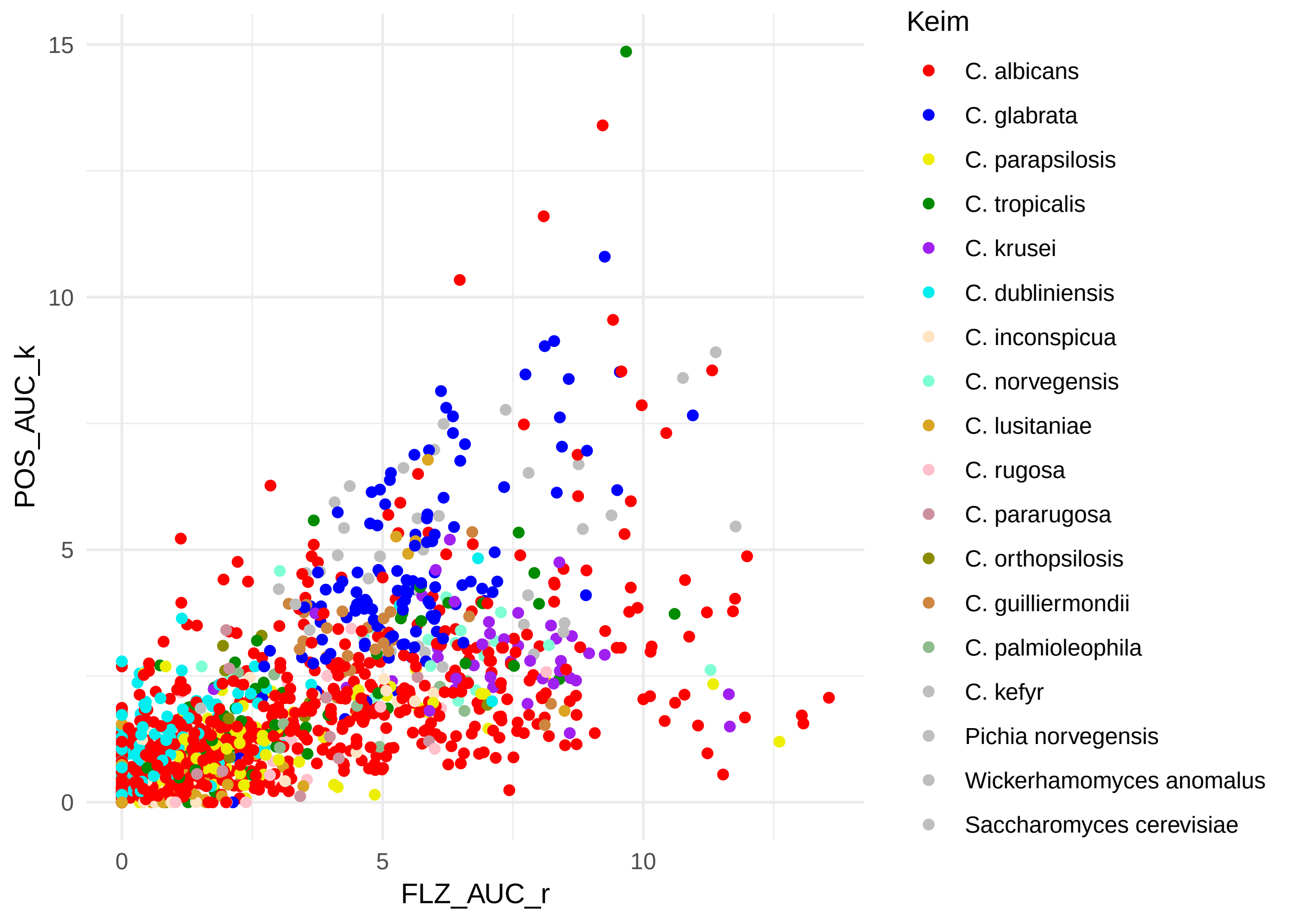

Keim
C. albicans
C. glabrata
C. parapsilosis
C. tropicalis
C. krusei
C. dubliniensis
C. inconspicua
C. norvegensis
C. lusitaniae
C. rugosa
C. pararugosa
C. orthopsilosis
C. guilliermondii
C. palmioleophila
C. kefyr
Pichia norvegensis
Wickerhamomyces anomalus
Saccharomyces cerevisiae
POS_AUC_k
FLZ_AUC_r
0
5
10
15
0
5
10

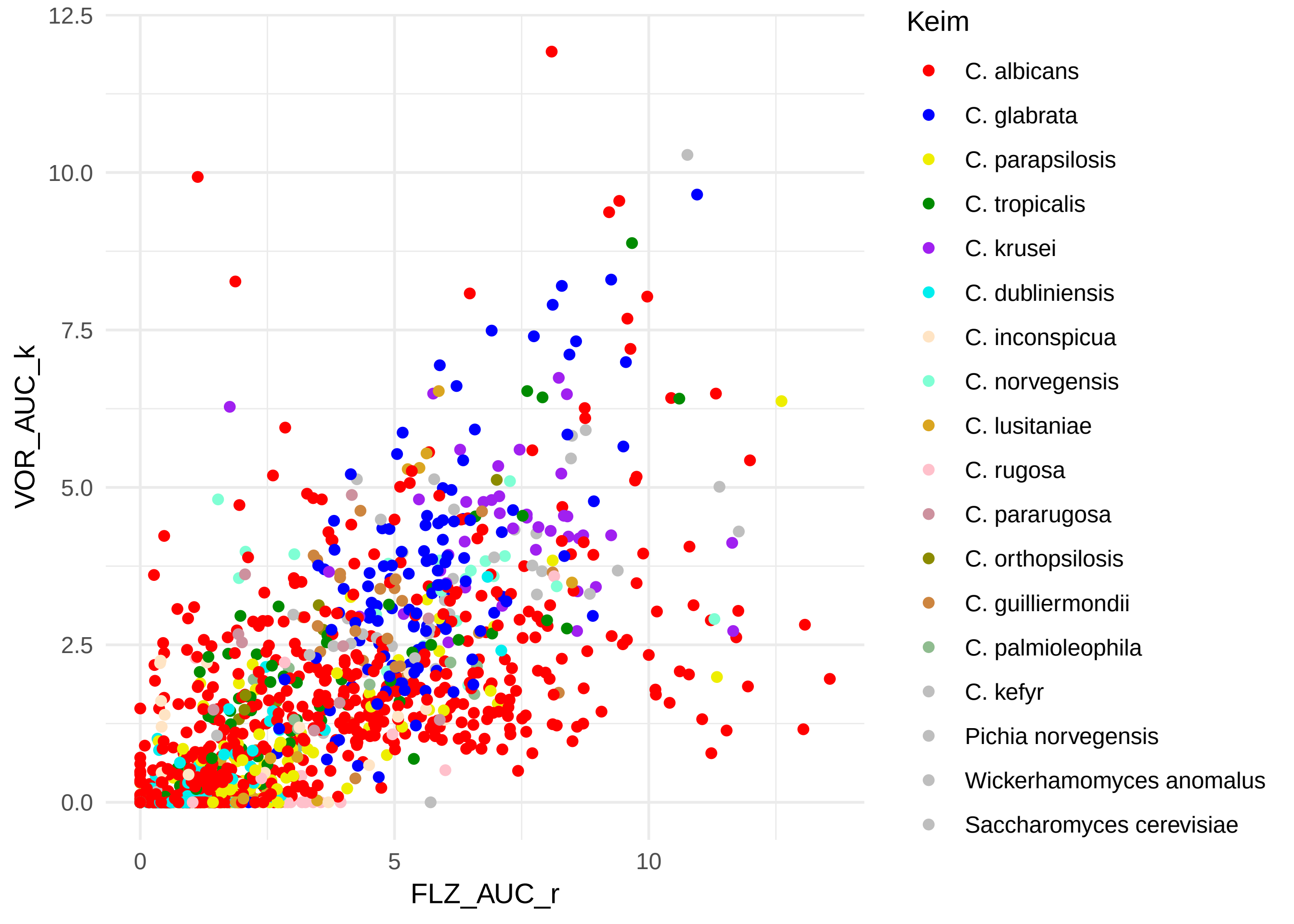

12.5
10.0
7.5
5.0
2.5
0.0
VOR_AUC_k
0
5
10
FLZ_AUC_r
Keim
C. albicans
C. glabrata
C. parapsilosis
C. tropicalis
C. krusei
C. dubliniensis
C. inconspicua
C. norvegensis
C. lusitaniae
C. rugosa
C. pararugosa
C. orthopsilosis
C. guilliermondii
C. palmioleophila
C. kefyr
Pichia norvegensis
Wickerhamomyces anomalus
Saccharomyces cerevisiae

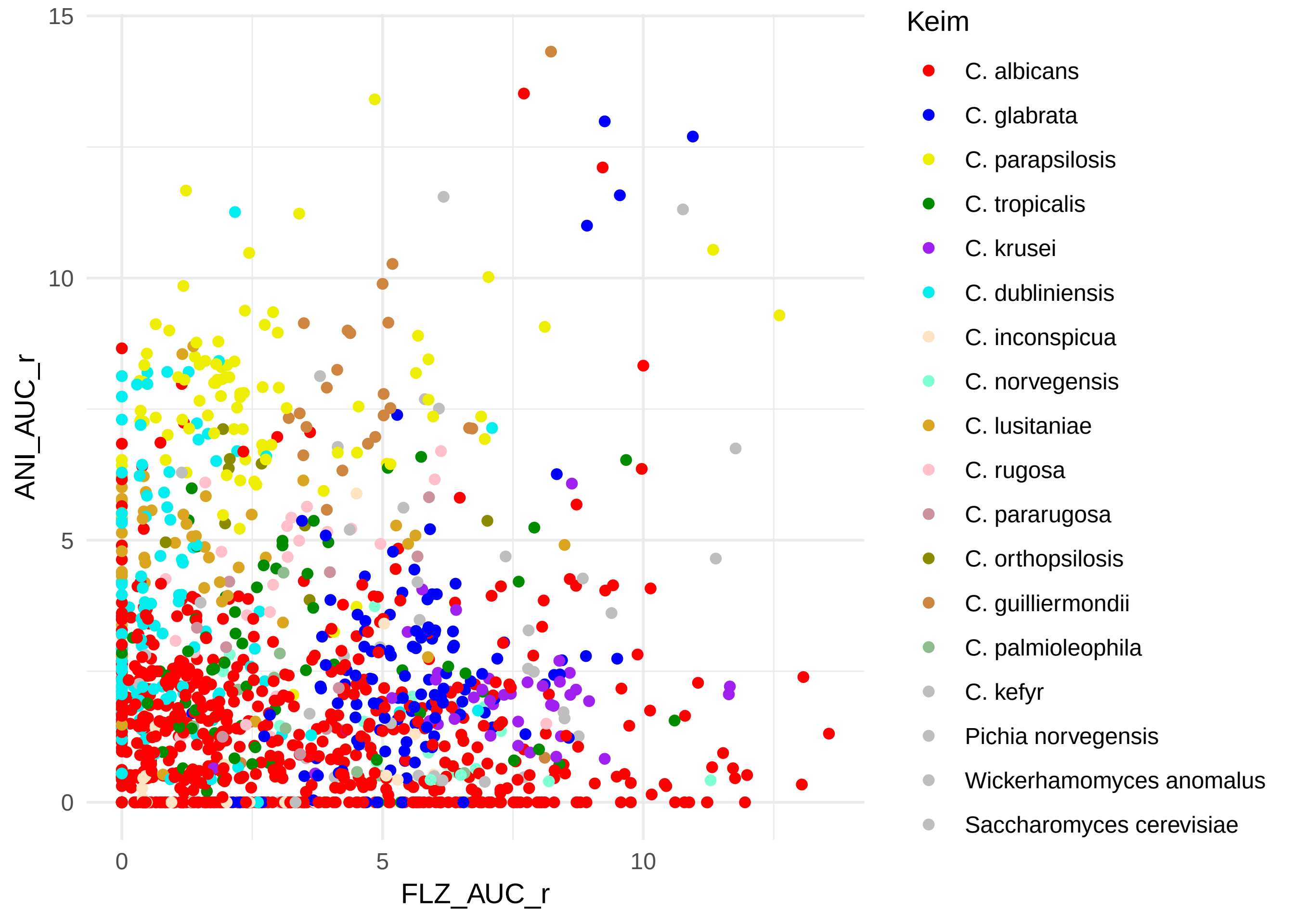
15
10
5
0
ANI_AUC_r
0
5
10
FLZ_AUC_r
Keim
C. albicans
C. glabrata
C. parapsilosis
C. tropicalis
C. krusei
C. dubliniensis
C. inconspicua
C. norvegensis
C. lusitaniae
C. rugosa
C. pararugosa
C. orthopsilosis
C. guilliermondii
C. palmioleophila
C. kefyr
Pichia norvegensis
Wickerhamomyces anomalus
Saccharomyces cerevisiae

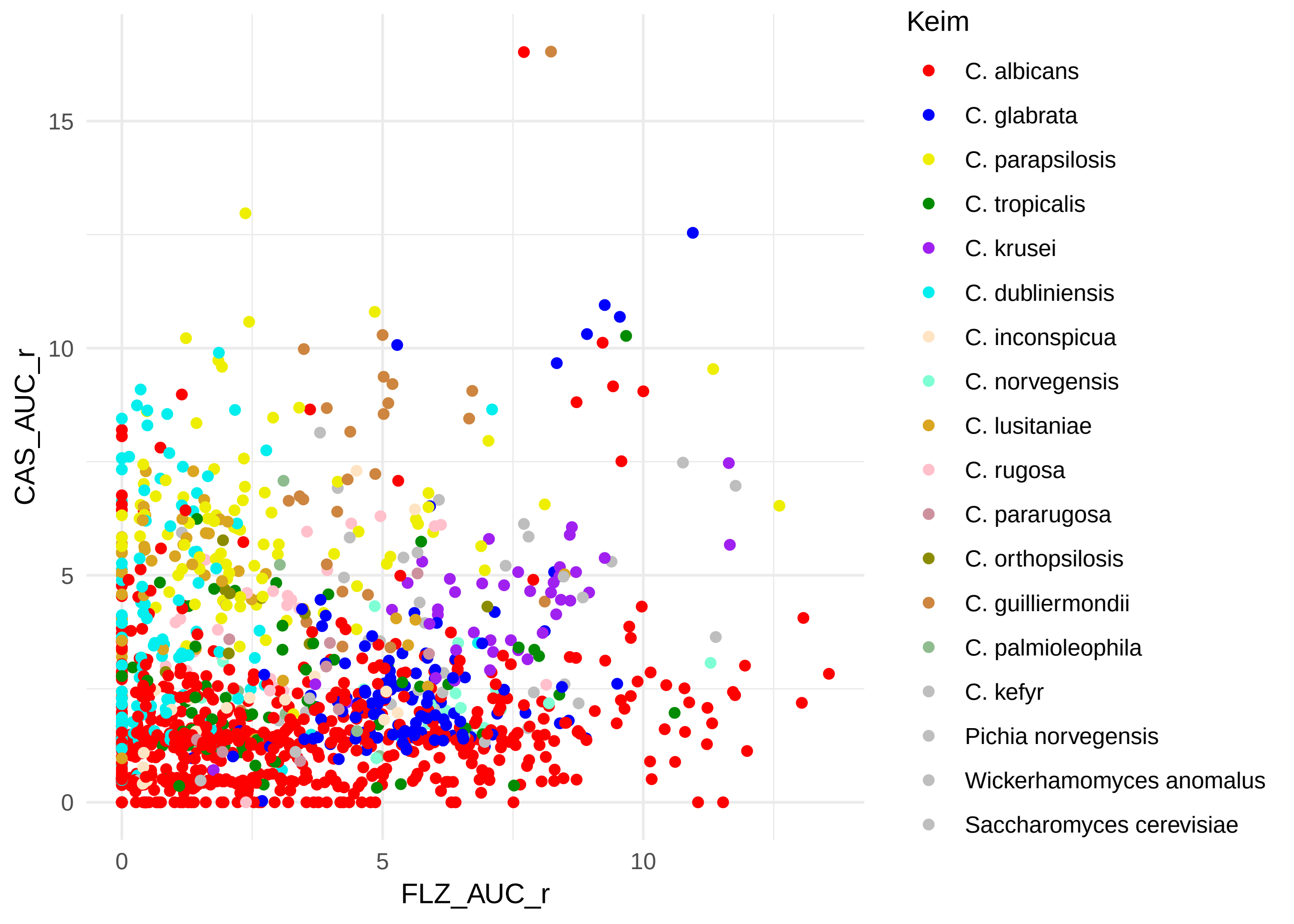
CAS_AUC_r
FLZ_AUC_r
0
5
10
15
0
5
10
Keim
C. albicans
C. glabrata
C. parapsilosis
C. tropicalis
C. krusei
C. dubliniensis
C. inconspicua
C. norvegensis
C. lusitaniae
C. rugosa
C. pararugosa
C. orthopsilosis
C. guilliermondii
C. palmioleophila
C. kefyr
Pichia norvegensis
Wickerhamomyces anomalus
Saccharomyces cerevisiae

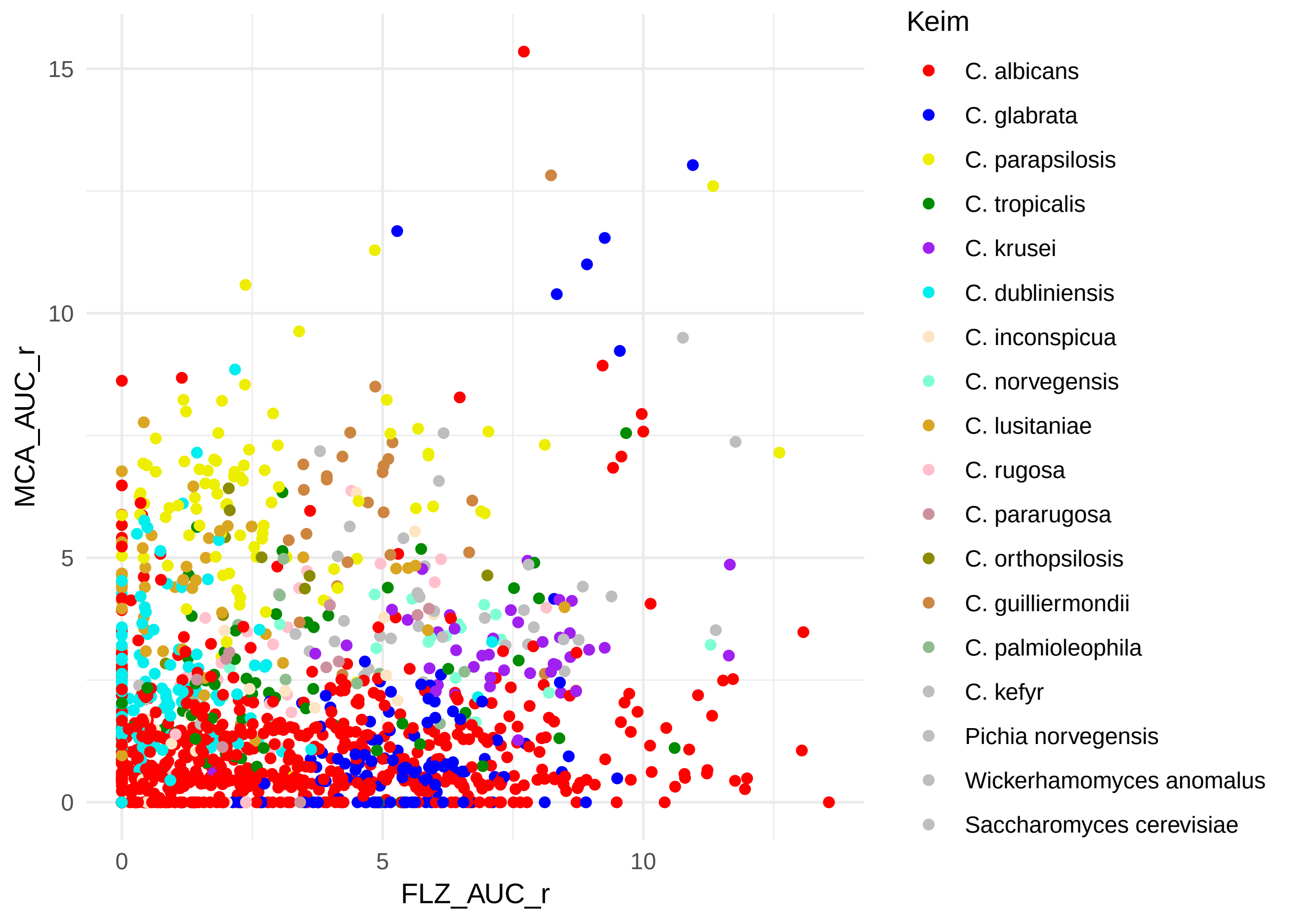

Keim
C. albicans
C. glabrata
C. parapsilosis
C. tropicalis
C. krusei
C. dubliniensis
C. inconspicua
C. norvegensis
C. lusitaniae
C. rugosa
C. pararugosa
C. orthopsilosis
C. guilliermondii
C. palmioleophila
C. kefyr
Pichia norvegensis
Wickerhamomyces anomalus
Saccharomyces cerevisiae
MCA_AUC_r
FLZ_AUC_r
0
5
10
15

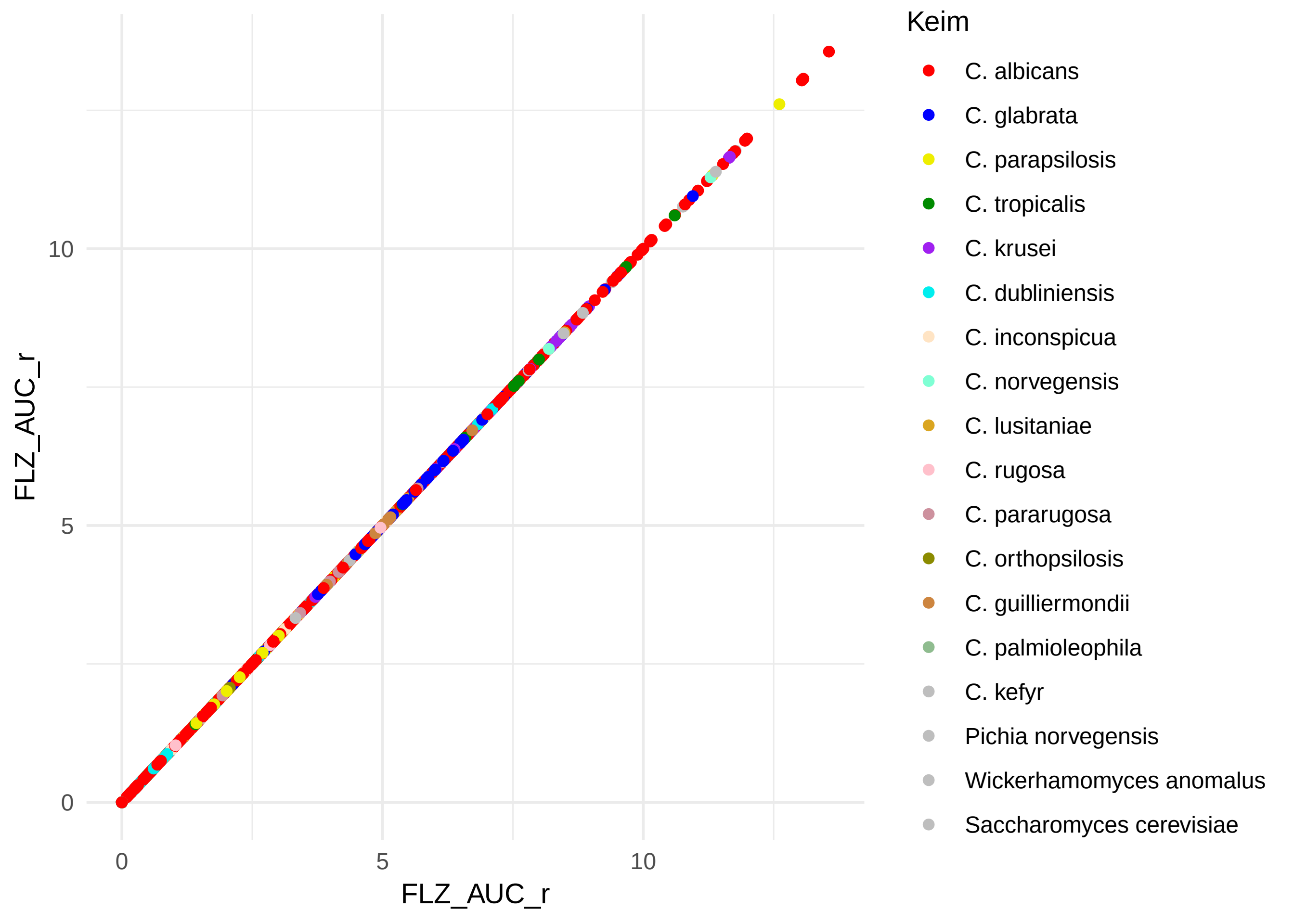
Keim
C. albicans
C. glabrata
C. parapsilosis
C. tropicalis
C. krusei
C. dubliniensis
C. inconspicua
C. norvegensis
C. lusitaniae
C. rugosa
C. pararugosa
C. orthopsilosis
C. guilliermondii
C. palmioleophila
C. kefyr
Pichia norvegensis
Wickerhamomyces anomalus
Saccharomyces cerevisiae
FLZ_AUC_r
FLZ_AUC_r
0
5
10
0
5
10

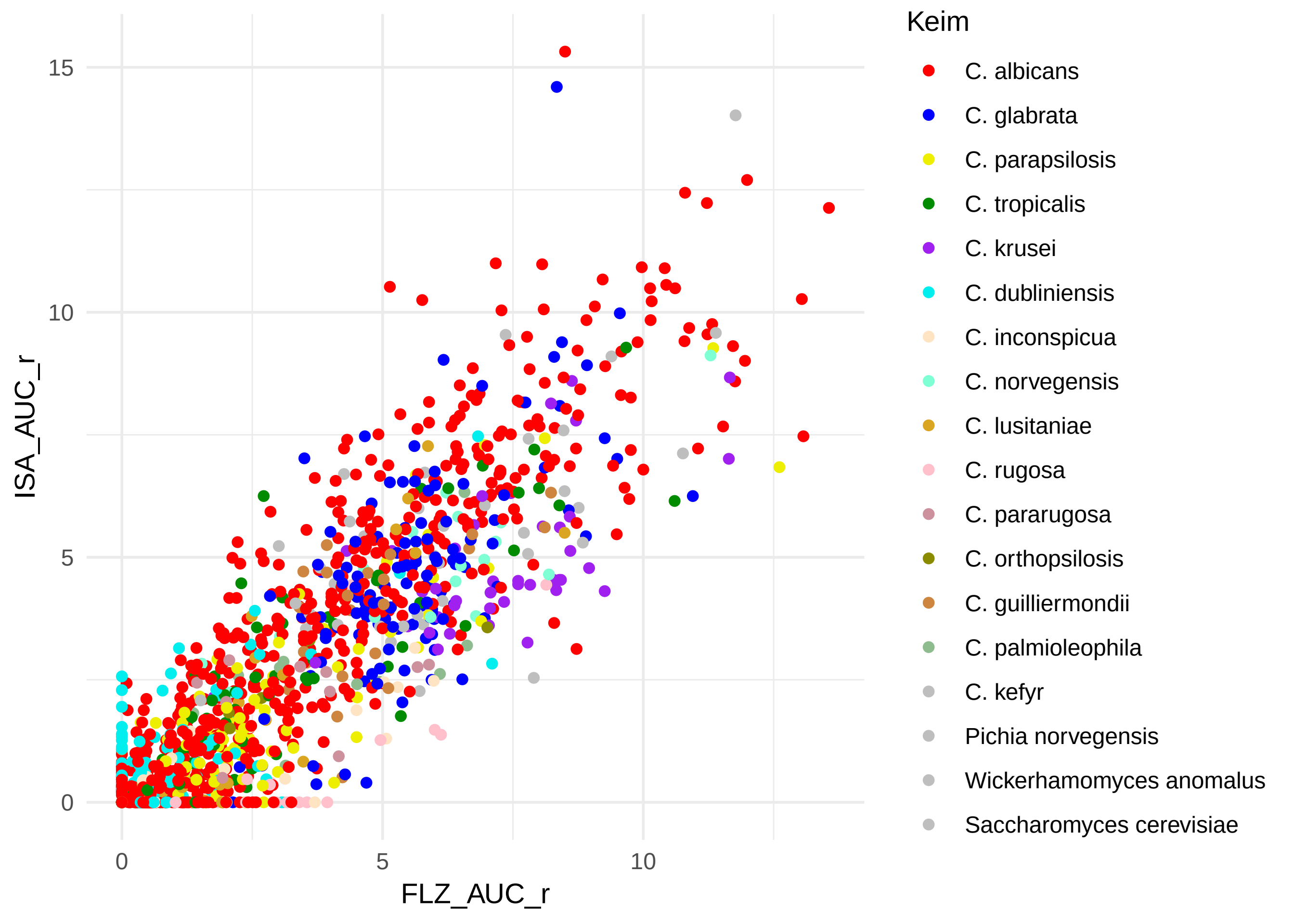

Keim
C. albicans
C. glabrata
C. parapsilosis
C. tropicalis
C. krusei
C. dubliniensis
C. inconspicua
C. norvegensis
C. lusitaniae
C. rugosa
C. pararugosa
C. orthopsilosis
C. guilliermondii
C. palmioleophila
C. kefyr
Pichia norvegensis
Wickerhamomyces anomalus
Saccharomyces cerevisiae
ISA_AUC_r
FLZ_AUC_r
15
10
5
0
0
5
10

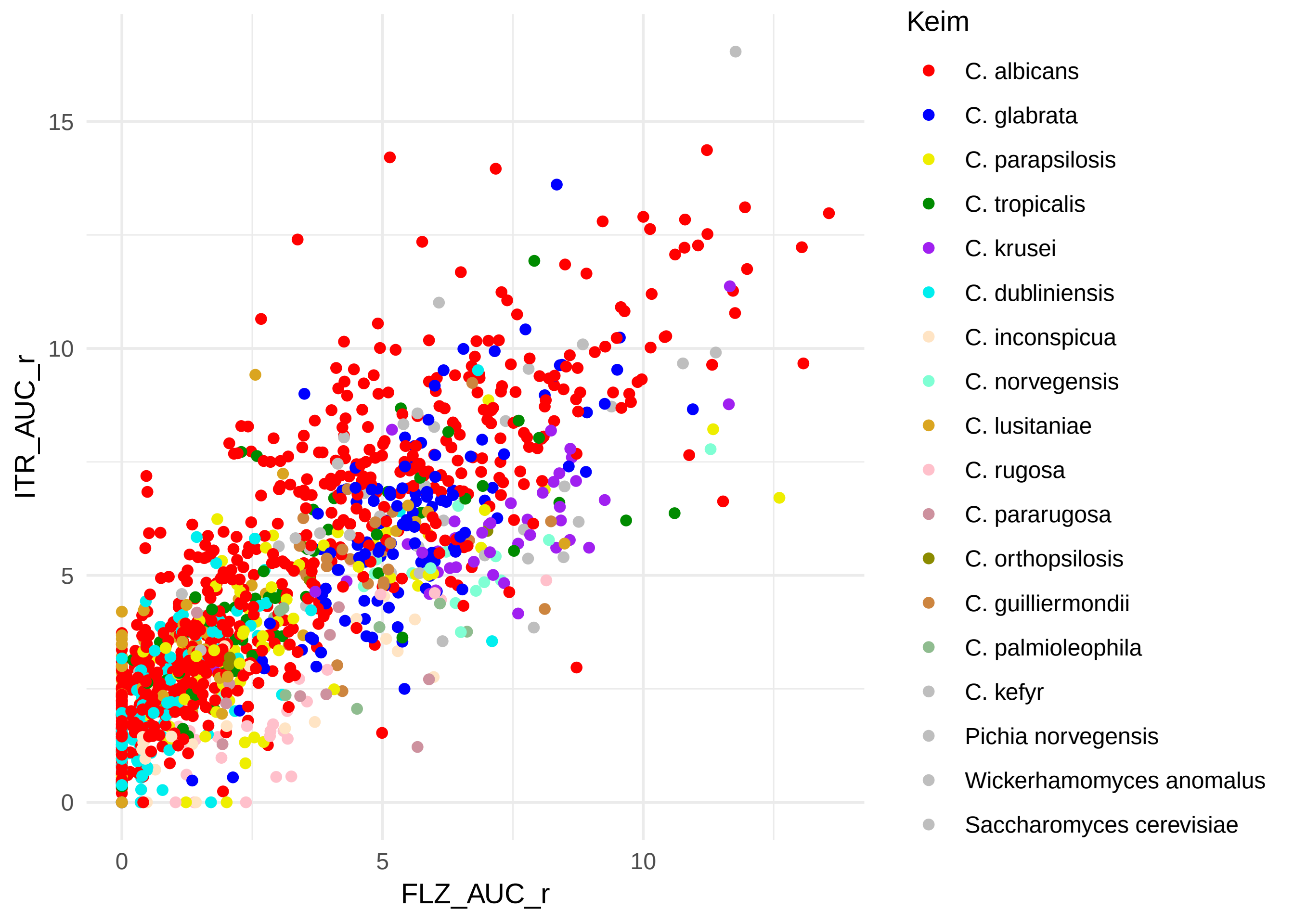

Keim
C. albicans
C. glabrata
C. parapsilosis
C. tropicalis
C. krusei
C. dubliniensis
C. inconspicua
C. norvegensis
C. lusitaniae
C. rugosa
C. pararugosa
C. orthopsilosis
C. guilliermondii
C. palmioleophila
C. kefyr
Pichia norvegensis
Wickerhamomyces anomalus
Saccharomyces cerevisiae
ITR_AUC_r
FLZ_AUC_r
0
5
10
15

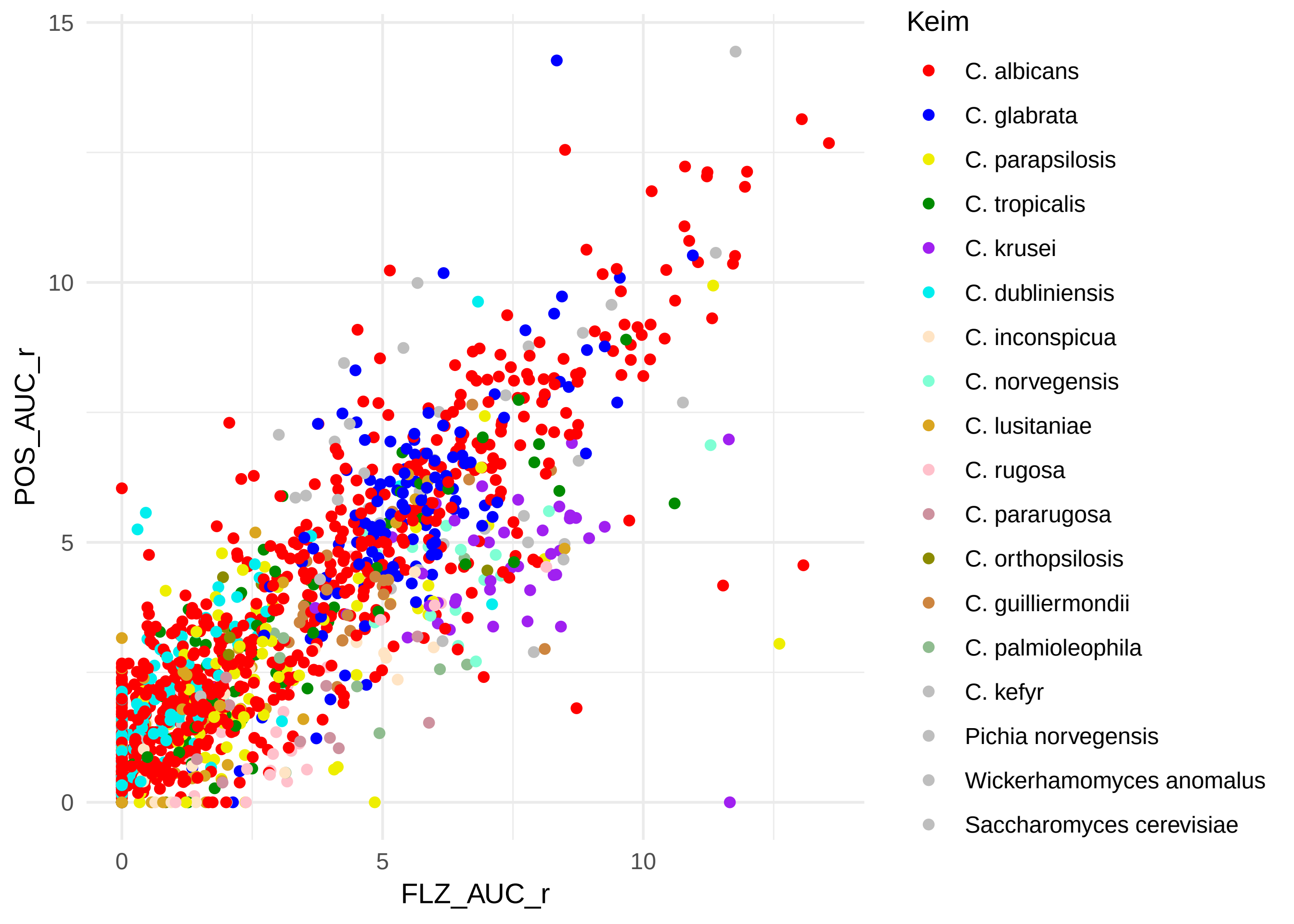

15
10
5
0
POS_AUC_r
0
5
10
FLZ_AUC_r
Keim
C. albicans
C. glabrata
C. parapsilosis
C. tropicalis
C. krusei
C. dubliniensis
C. inconspicua
C. norvegensis
C. lusitaniae
C. rugosa
C. pararugosa
C. orthopsilosis
C. guilliermondii
C. palmioleophila
C. kefyr
Pichia norvegensis
Wickerhamomyces anomalus
Saccharomyces cerevisiae

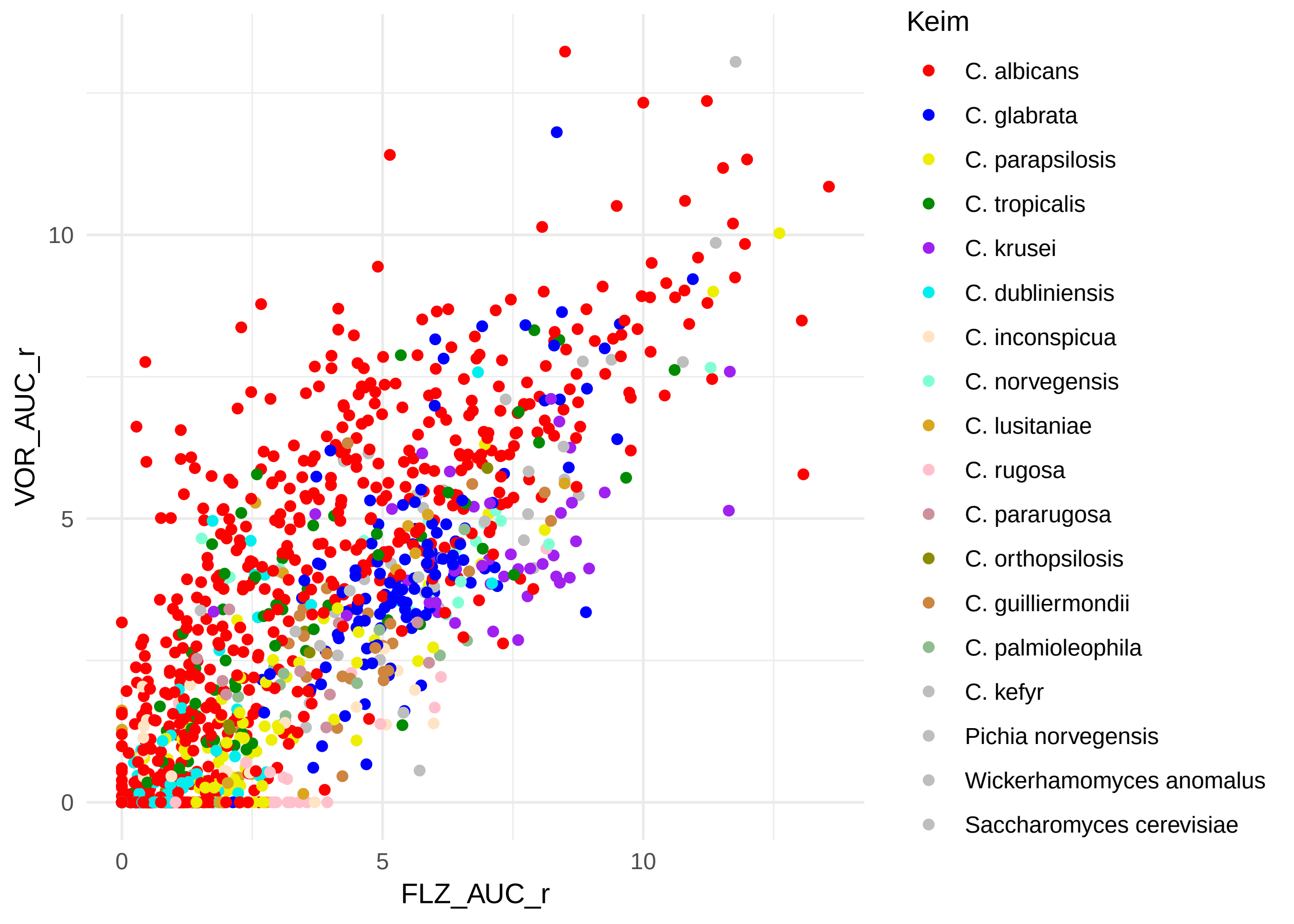

Keim
C. albicans
C. glabrata
C. parapsilosis
C. tropicalis
C. krusei
C. dubliniensis
C. inconspicua
C. norvegensis
C. lusitaniae
C. rugosa
C. pararugosa
C. orthopsilosis
C. guilliermondii
C. palmioleophila
C. kefyr
Pichia norvegensis
Wickerhamomyces anomalus
Saccharomyces cerevisiae
VOR_AUC_r
FLZ_AUC_r
0
5
10
0
5
10

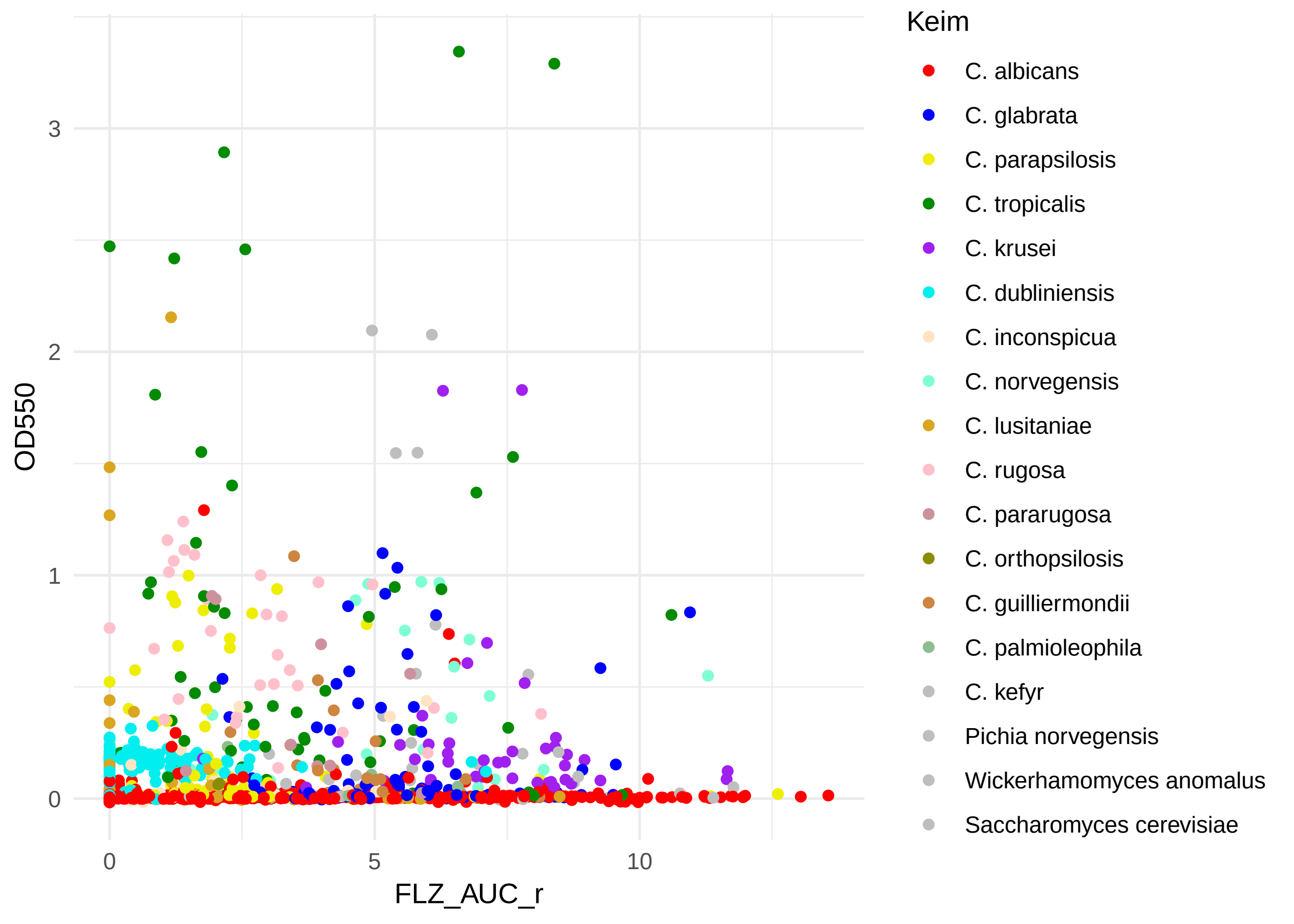

OD550
3
2
1
0
0
5
10
FLZ_AUC_r
Keim
C. albicans
C. glabrata
C. parapsilosis
C. tropicalis
C. krusei
C. dubliniensis
C. inconspicua
C. norvegensis
C. lusitaniae
C. rugosa
C. pararugosa
C. orthopsilosis
C. guilliermondii
C. palmioleophila
C. kefyr
Pichia norvegensis
Wickerhamomyces anomalus
Saccharomyces cerevisiae

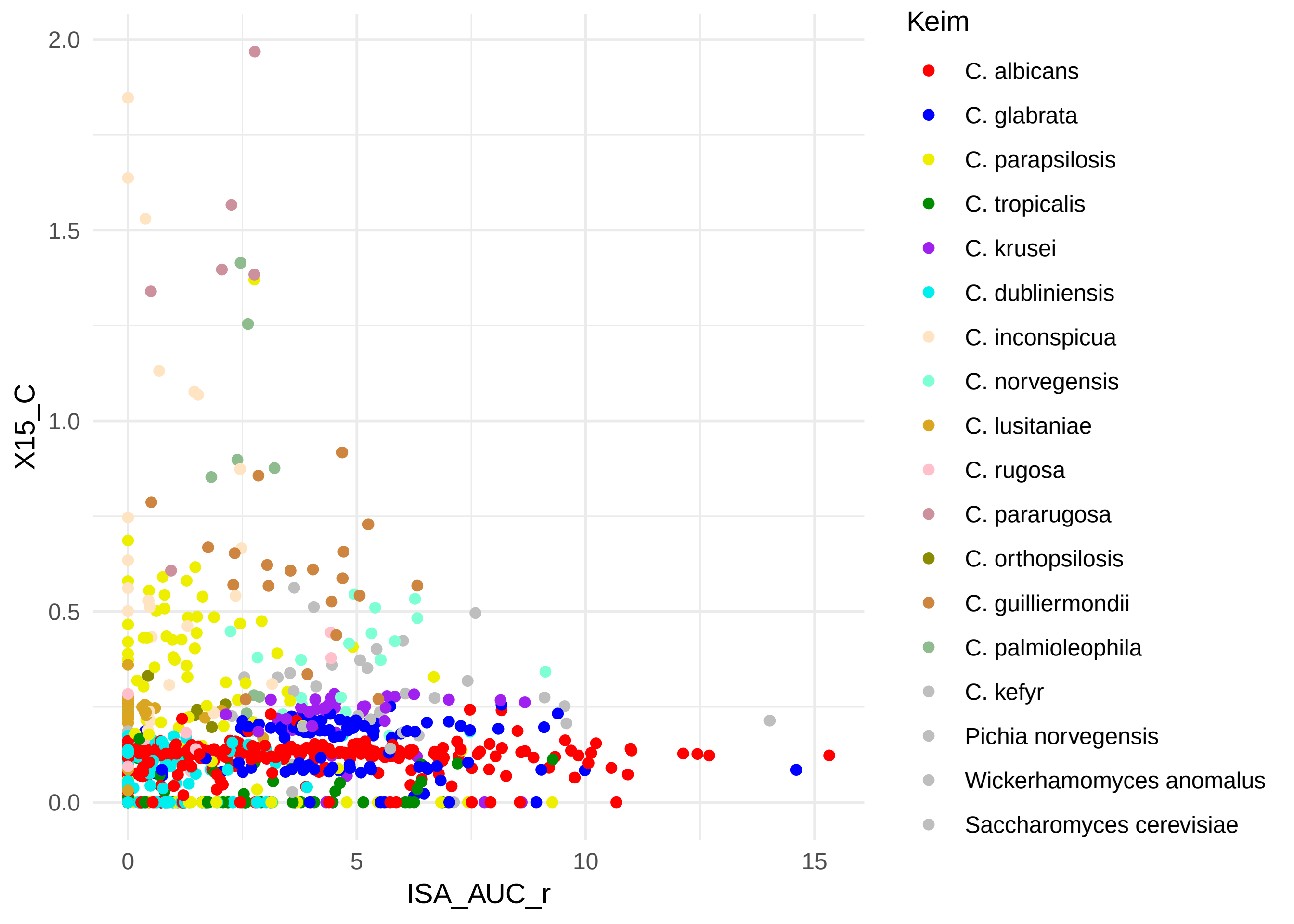

X15_C
0.0
0.5
1.0
1.5
2.0
0
5
10
15
ISA_AUC_r
Keim
C. albicans
C. glabrata
C. parapsilosis
C. tropicalis
C. krusei
C. dubliniensis
C. inconspicua
C. norvegensis
C. lusitaniae
C. rugosa
C. pararugosa
C. orthopsilosis
C. guilliermondii
C. palmioleophila
C. kefyr
Pichia norvegensis
Wickerhamomyces anomalus
Saccharomyces cerevisiae

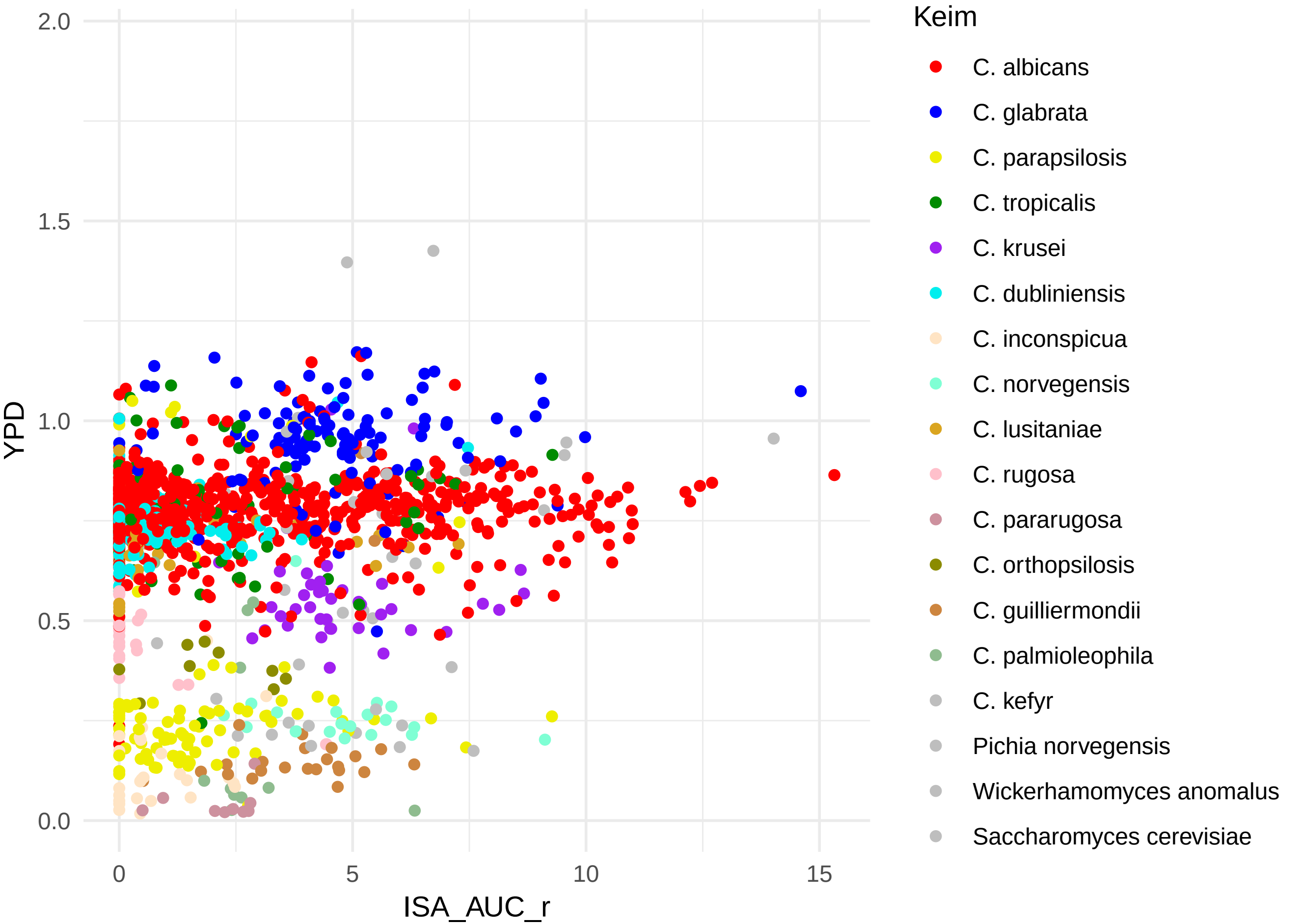
YPD
2.0
1.5
1.0
0.5
0.0
0
5
10
15
ISA_AUC_r
Keim
C. albicans
C. glabrata
C. parapsilosis
C. tropicalis
C. krusei
C. dubliniensis
C. inconspicua
C. norvegensis
C. lusitaniae
C. rugosa
C. pararugosa
C. orthopsilosis
C. guilliermondii
C. palmioleophila
C. kefyr
Pichia norvegensis
Wickerhamomyces anomalus
Saccharomyces cerevisiae

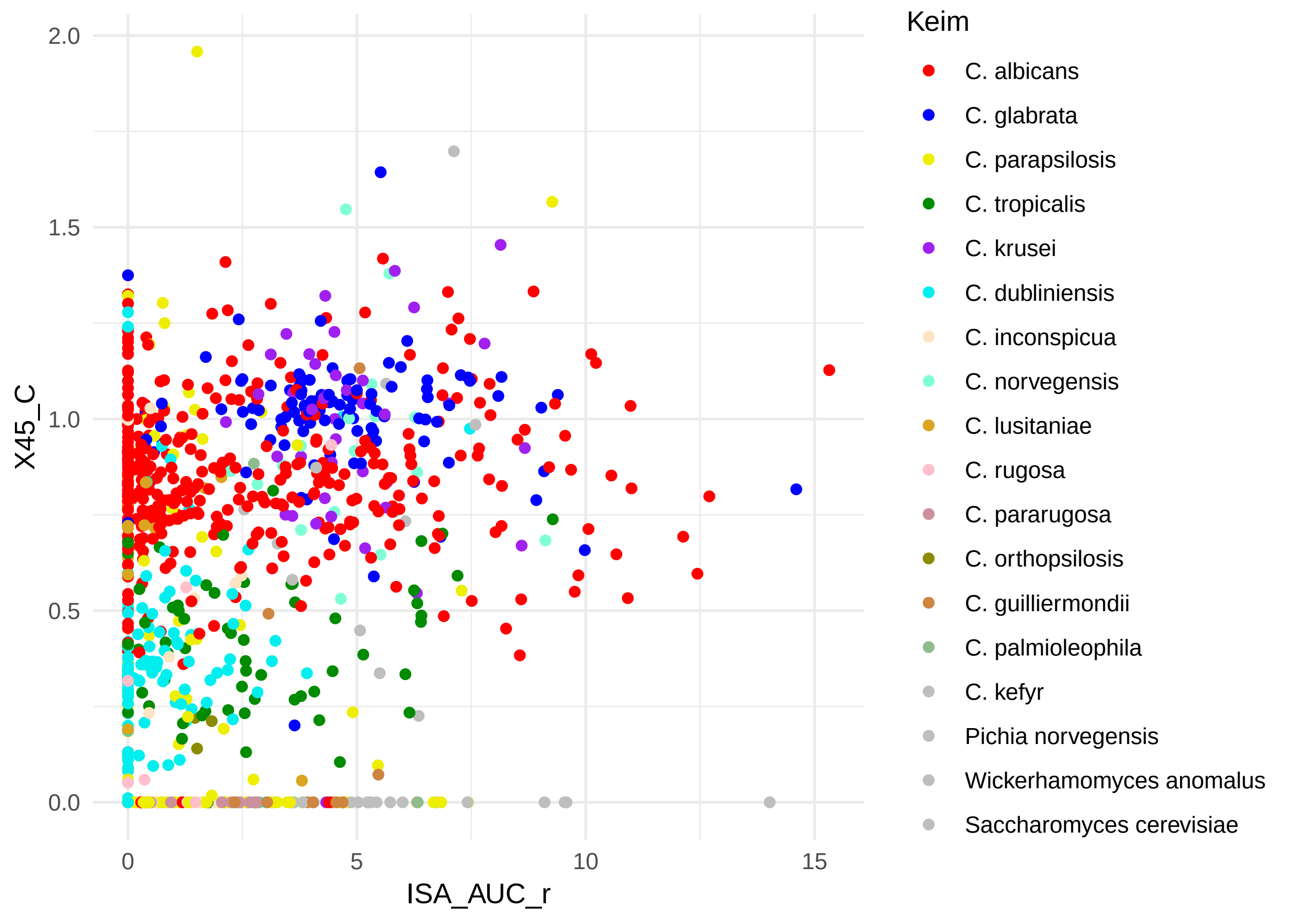

Keim
C. albicans
C. glabrata
C. parapsilosis
C. tropicalis
C. krusei
C. dubliniensis
C. inconspicua
C. norvegensis
C. lusitaniae
C. rugosa
C. pararugosa
C. orthopsilosis
C. guilliermondii
C. palmioleophila
C. kefyr
Pichia norvegensis
Wickerhamomyces anomalus
Saccharomyces cerevisiae
X45_C
ISA_AUC_r
2.0
1.5
1.0
0.5
0.0
0
5
10
15

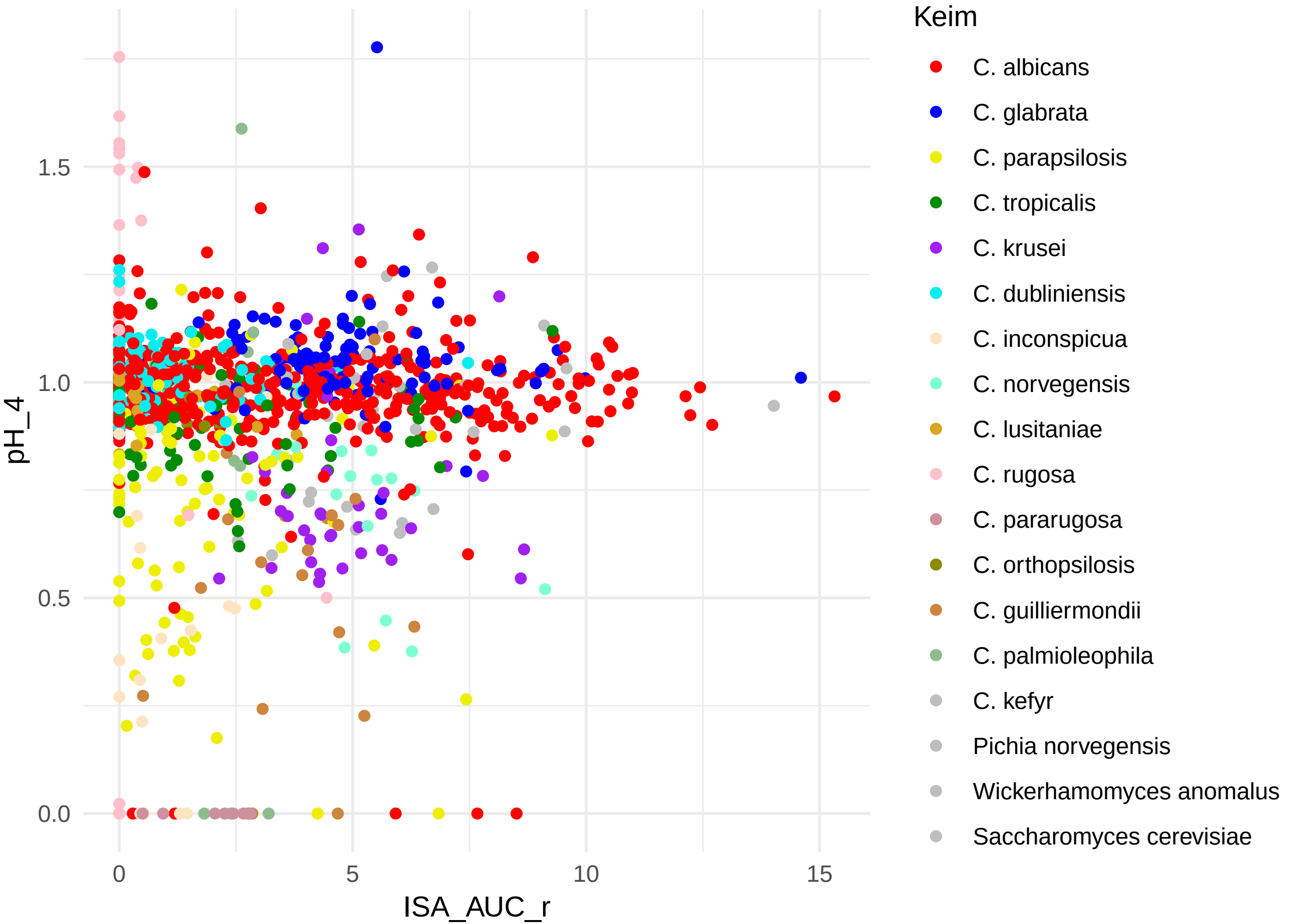

Keim
C. albicans
C. glabrata
C. parapsilosis
C. tropicalis
C. krusei
C. dubliniensis
C. inconspicua
C. norvegensis
C. lusitaniae
C. rugosa
C. pararugosa
C. orthopsilosis
C. guilliermondii
C. palmioleophila
C. kefyr
Pichia norvegensis
Wickerhamomyces anomalus
Saccharomyces cerevisiae
pH_4
ISA_AUC_r
0.0
0.5
1.0
1.5
0
5
10
15

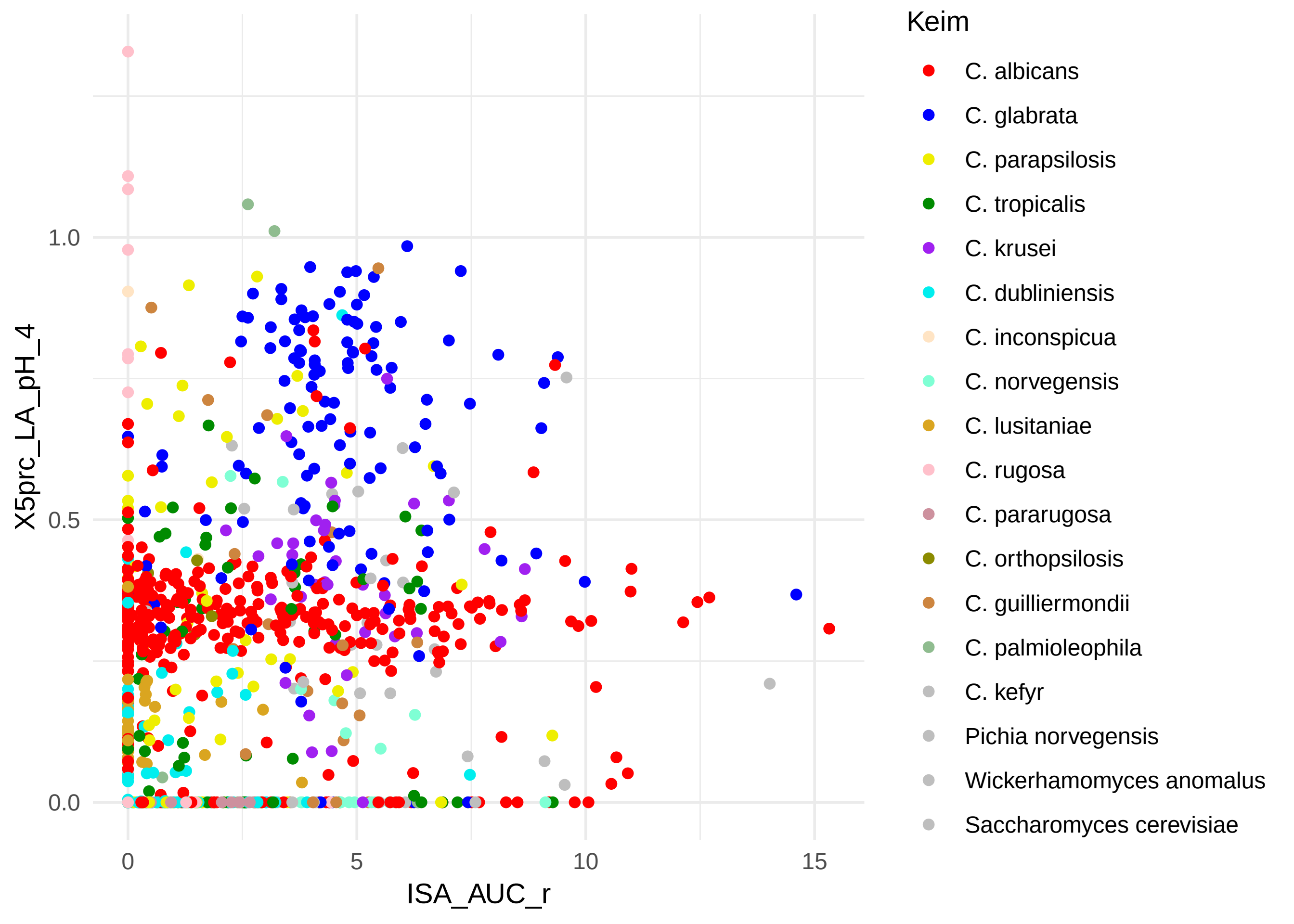

Keim
C. albicans
C. glabrata
C. parapsilosis
C. tropicalis
C. krusei
C. dubliniensis
C. inconspicua
C. norvegensis
C. lusitaniae
C. rugosa
C. pararugosa
C. orthopsilosis
C. guilliermondii
C. palmioleophila
C. kefyr
Pichia norvegensis
Wickerhamomyces anomalus
Saccharomyces cerevisiae
X5prc_LA_pH_4
1.0
0.5
0.0
0
5
10
15
ISA_AUC_r

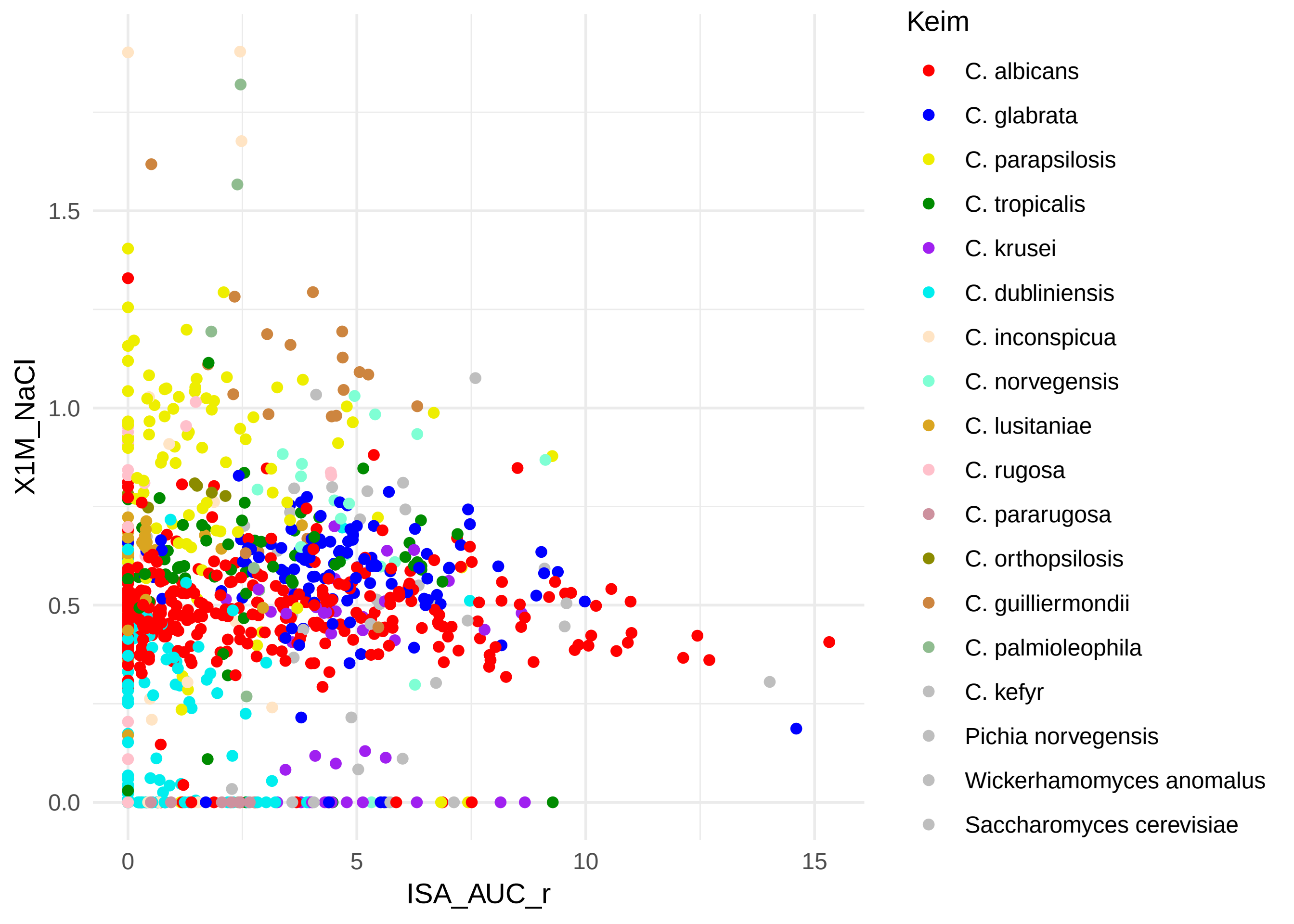
X1M_NaCl
ISA_AUC_r
0.0
0.5
1.0
1.5
0
5
10
15
Keim
C. albicans
C. glabrata
C. parapsilosis
C. tropicalis
C. krusei
C. dubliniensis
C. inconspicua
C. norvegensis
C. lusitaniae
C. rugosa
C. pararugosa
C. orthopsilosis
C. guilliermondii
C. palmioleophila
C. kefyr
Pichia norvegensis
Wickerhamomyces anomalus
Saccharomyces cerevisiae

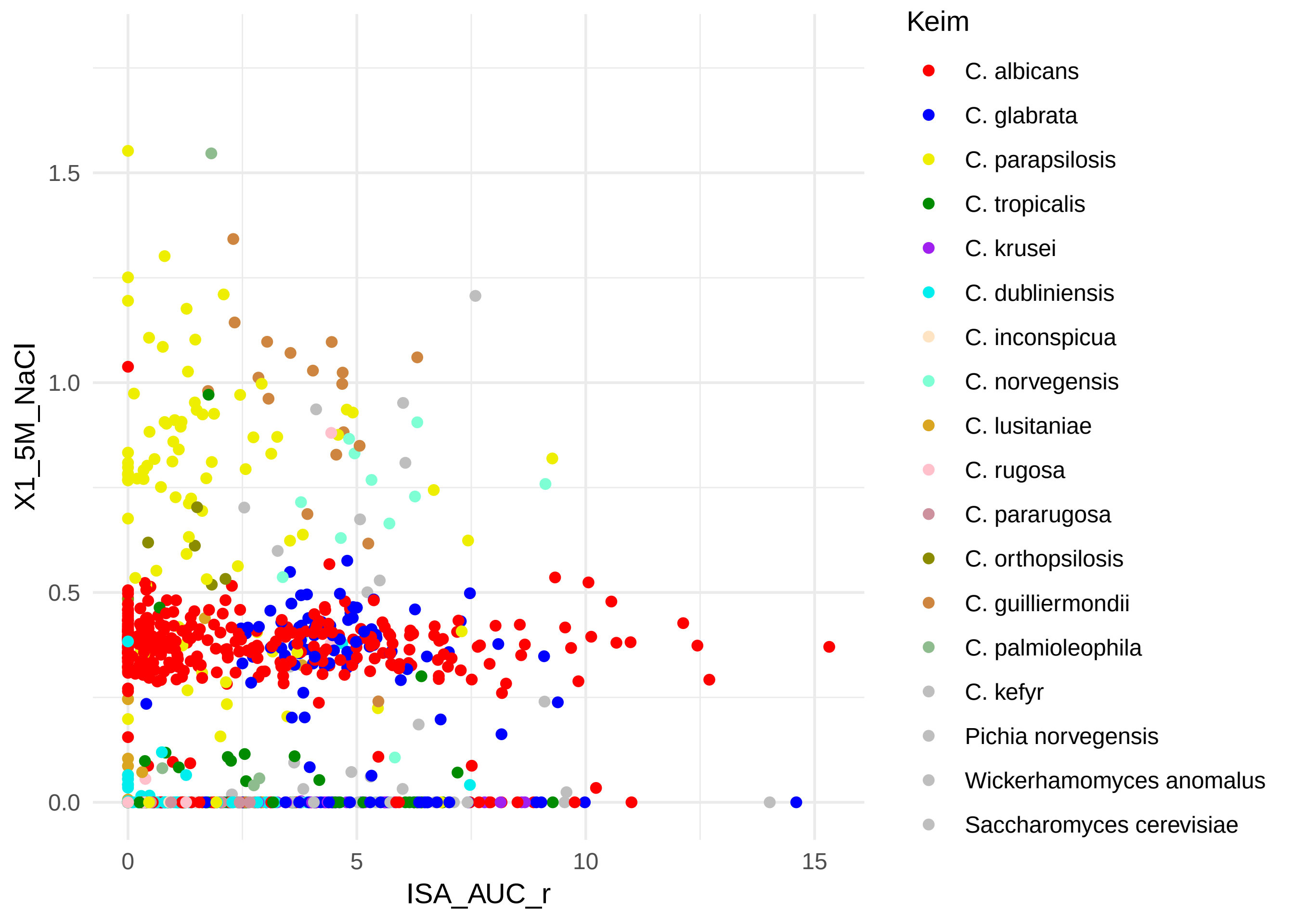

Keim
C. albicans
C. glabrata
C. parapsilosis
C. tropicalis
C. krusei
C. dubliniensis
C. inconspicua
C. norvegensis
C. lusitaniae
C. rugosa
C. pararugosa
C. orthopsilosis
C. guilliermondii
C. palmioleophila
C. kefyr
Pichia norvegensis
Wickerhamomyces anomalus
Saccharomyces cerevisiae
X1_5M_NaCl
1.5
1.0
0.5
0.0
ISA_AUC_r
0
5
10
15

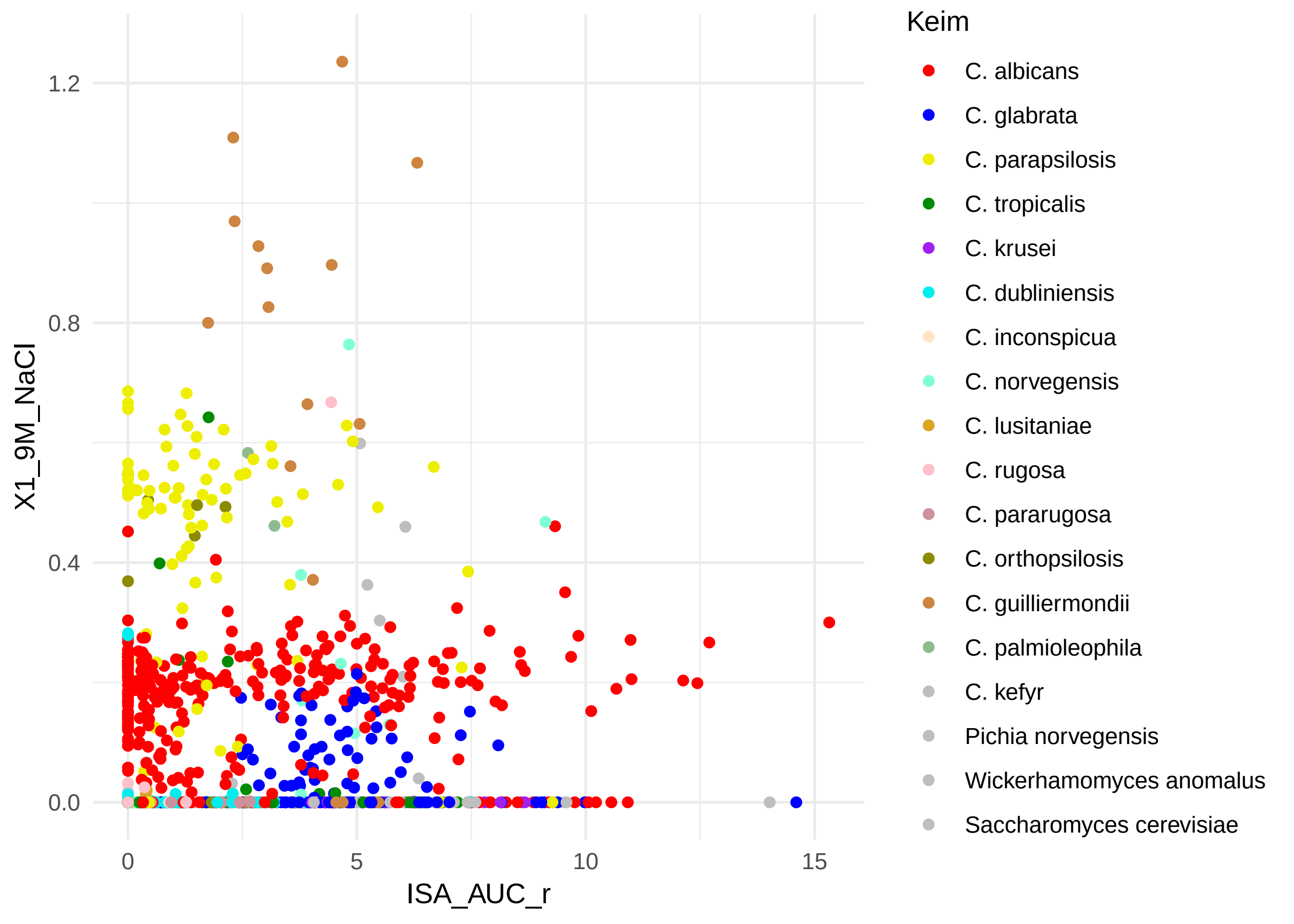

X1_9M_NaCl
1.2
0.8
0.4
0.0
0
5
10
15
ISA_AUC_r
Keim
C. albicans
C. glabrata
C. parapsilosis
C. tropicalis
C. krusei
C. dubliniensis
C. inconspicua
C. norvegensis
C. lusitaniae
C. rugosa
C. pararugosa
C. orthopsilosis
C. guilliermondii
C. palmioleophila
C. kefyr
Pichia norvegensis
Wickerhamomyces anomalus
Saccharomyces cerevisiae

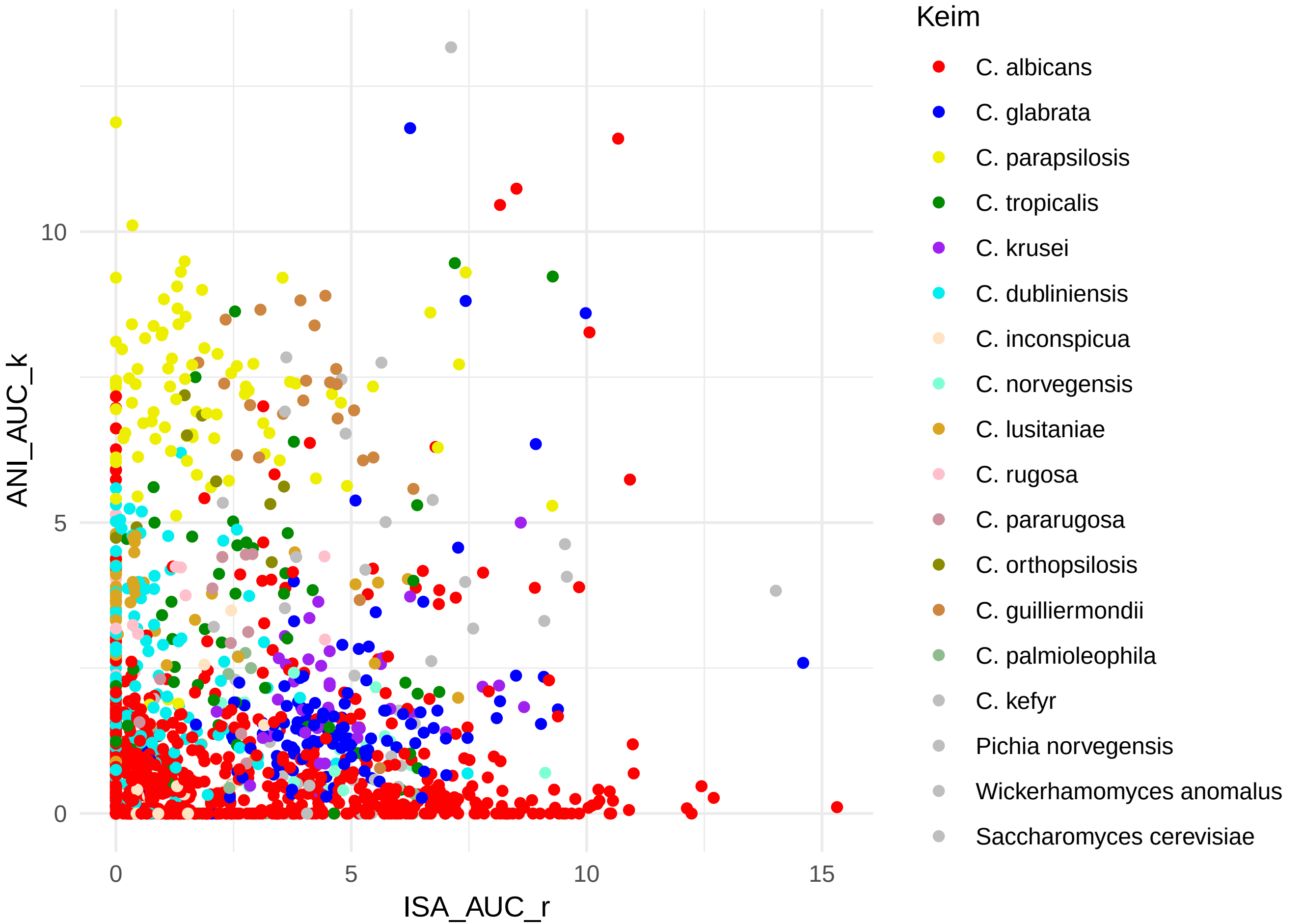

Keim
C. albicans
C. glabrata
C. parapsilosis
C. tropicalis
C. krusei
C. dubliniensis
C. inconspicua
C. norvegensis
C. lusitaniae
C. rugosa
C. pararugosa
C. orthopsilosis
C. guilliermondii
C. palmioleophila
C. kefyr
Pichia norvegensis
Wickerhamomyces anomalus
Saccharomyces cerevisiae
ANI_AUC_k
ISA_AUC_r
0
5
10
15

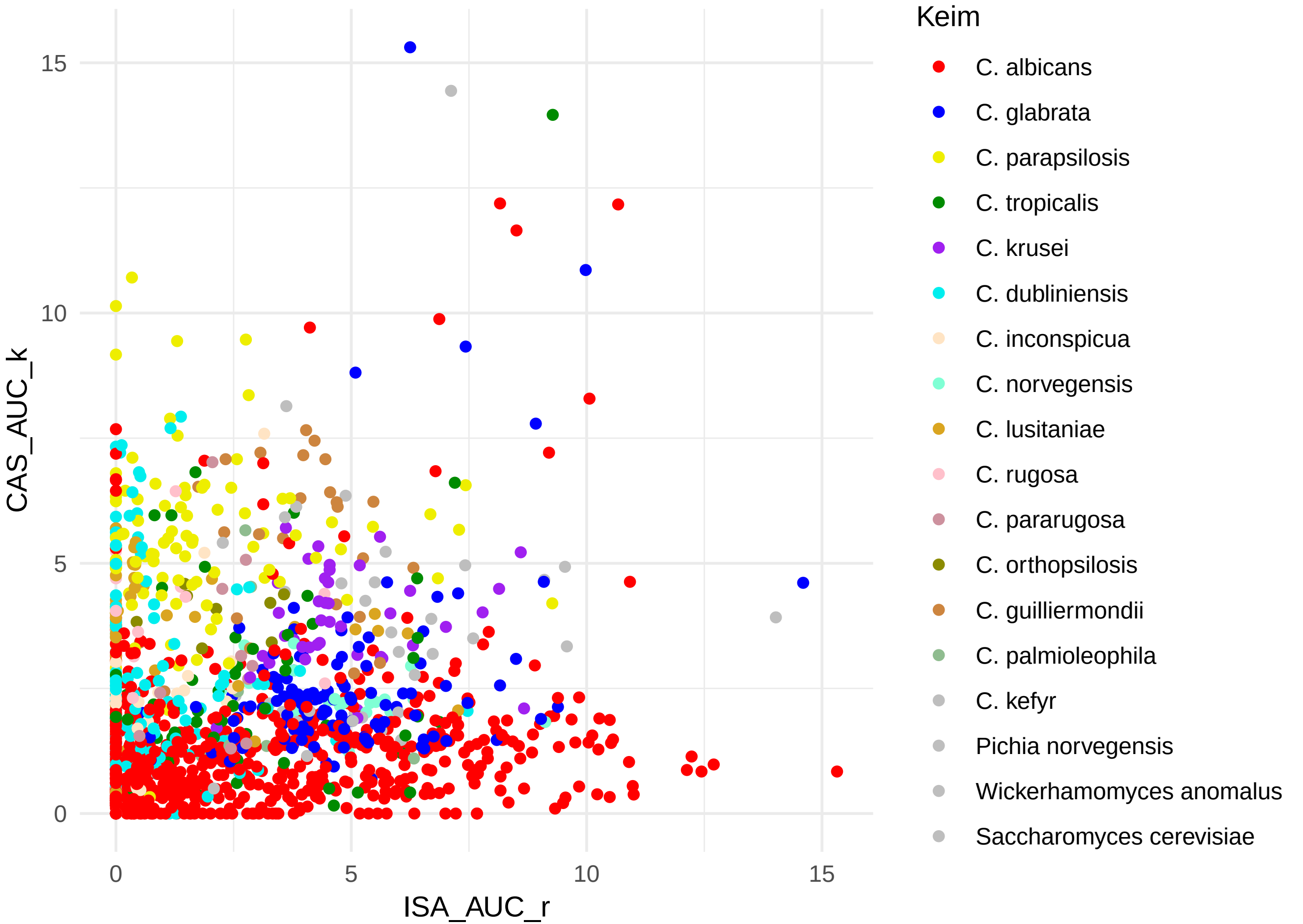
Keim
C. albicans
C. glabrata
C. parapsilosis
C. tropicalis
C. krusei
C. dubliniensis
C. inconspicua
C. norvegensis
C. lusitaniae
C. rugosa
C. pararugosa
C. orthopsilosis
C. guilliermondii
C. palmioleophila
C. kefyr
Pichia norvegensis
Wickerhamomyces anomalus
Saccharomyces cerevisiae
CAS_AUC_k
ISA_AUC_r
0
5
10
15
0
5
10
15

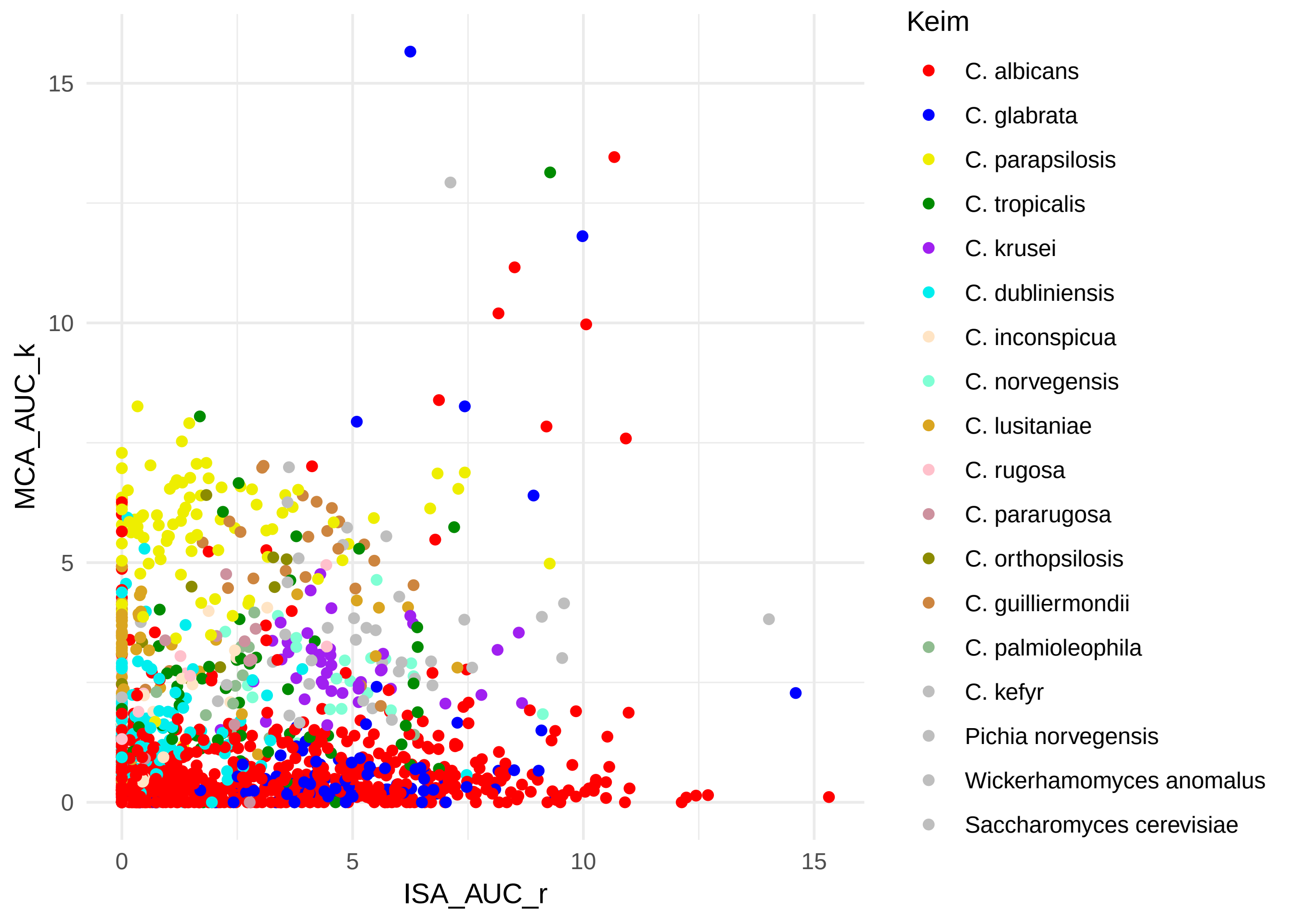

Keim
C. albicans
C. glabrata
C. parapsilosis
C. tropicalis
C. krusei
C. dubliniensis
C. inconspicua
C. norvegensis
C. lusitaniae
C. rugosa
C. pararugosa
C. orthopsilosis
C. guilliermondii
C. palmioleophila
C. kefyr
Pichia norvegensis
Wickerhamomyces anomalus
Saccharomyces cerevisiae
MCA_AUC_k
ISA_AUC_r
0
5
10
15

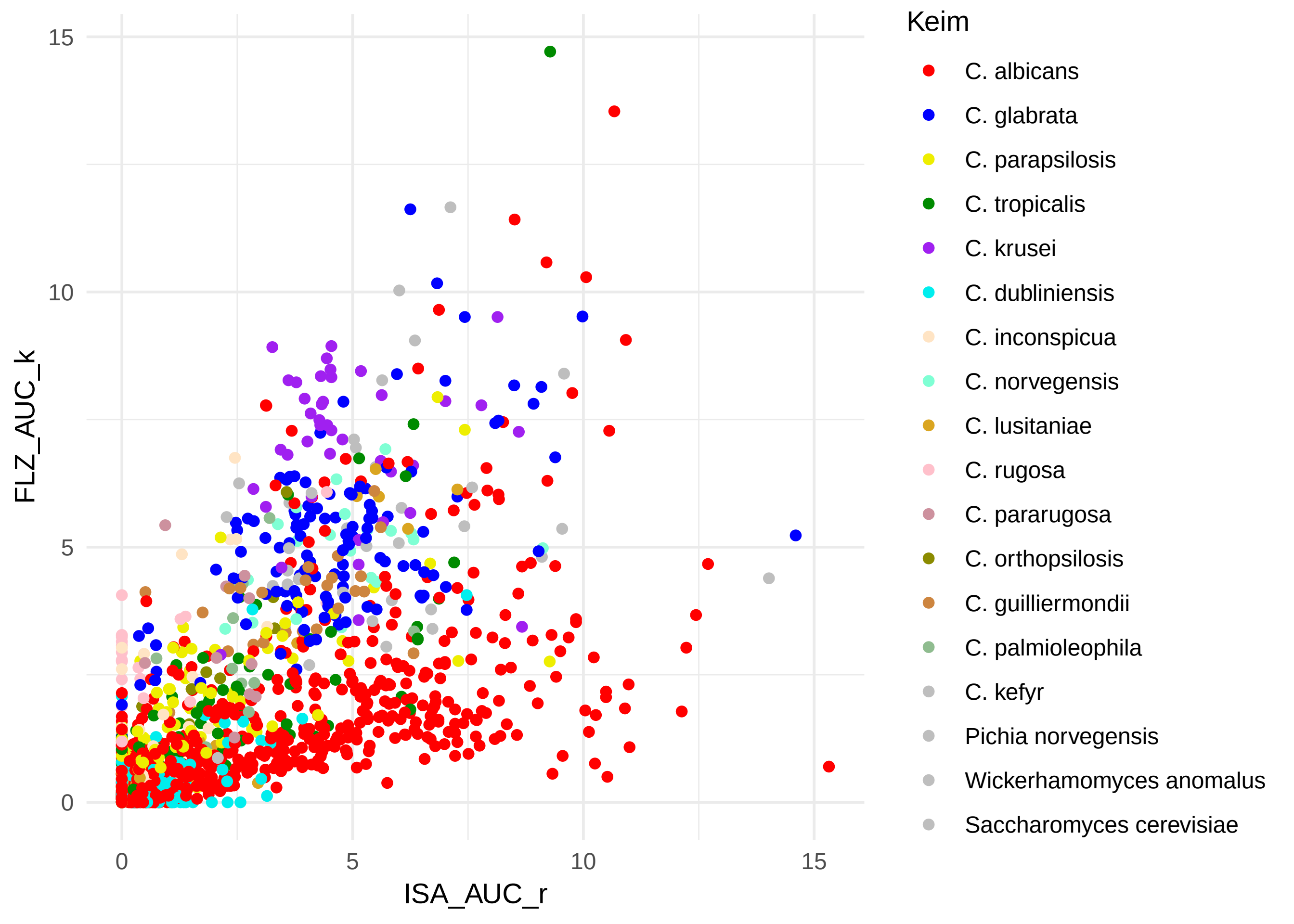

15
10
5
0
FLZ_AUC_k
0
5
10
15
ISA_AUC_r
Keim
C. albicans
C. glabrata
C. parapsilosis
C. tropicalis
C. krusei
C. dubliniensis
C. inconspicua
C. norvegensis
C. lusitaniae
C. rugosa
C. pararugosa
C. orthopsilosis
C. guilliermondii
C. palmioleophila
C. kefyr
Pichia norvegensis
Wickerhamomyces anomalus
Saccharomyces cerevisiae

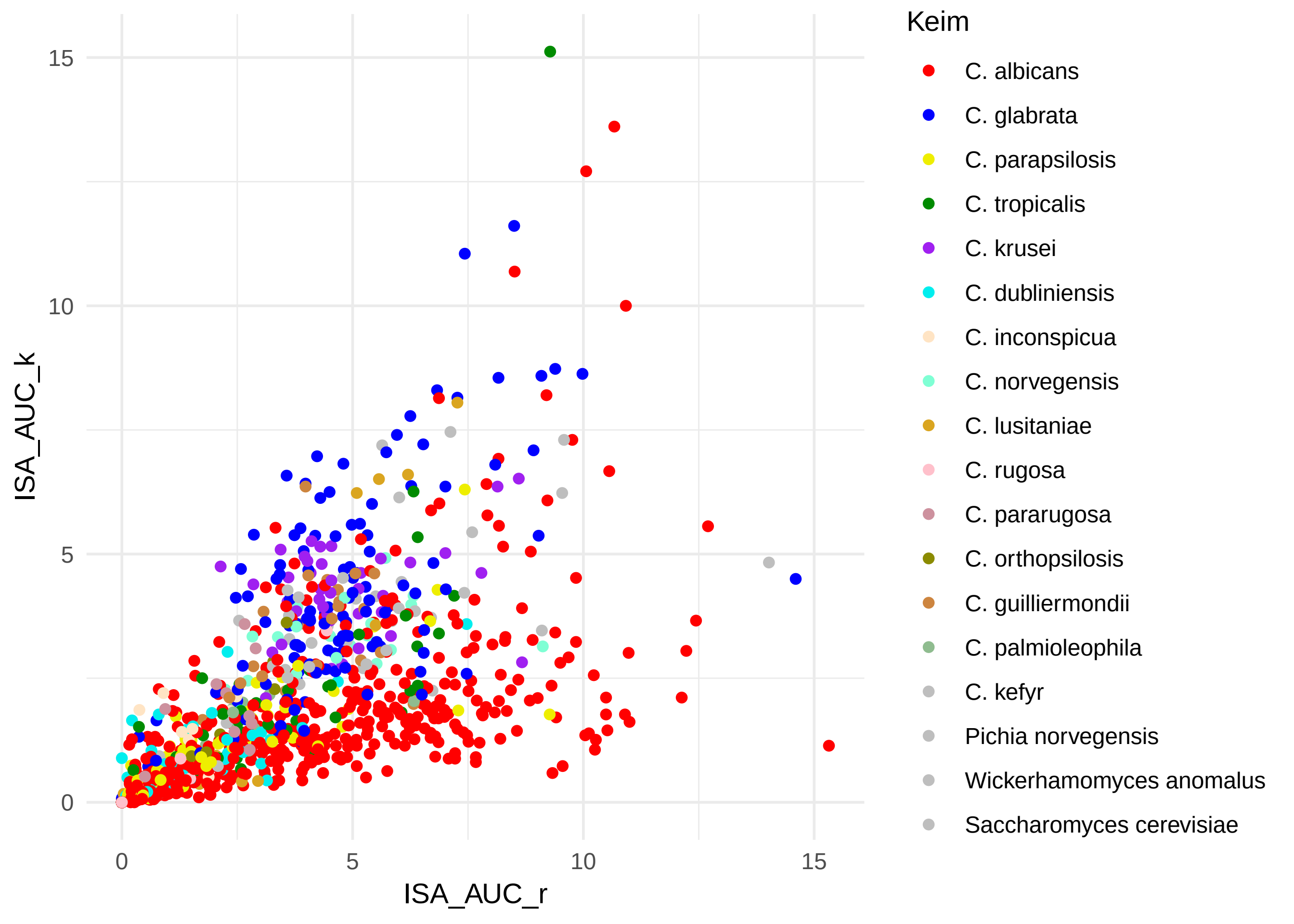
Keim
C. albicans
C. glabrata
C. parapsilosis
C. tropicalis
C. krusei
C. dubliniensis
C. inconspicua
C. norvegensis
C. lusitaniae
C. rugosa
C. pararugosa
C. orthopsilosis
C. guilliermondii
C. palmioleophila
C. kefyr
Pichia norvegensis
Wickerhamomyces anomalus
Saccharomyces cerevisiae
ISA_AUC_k
ISA_AUC_r
0
5
10
15

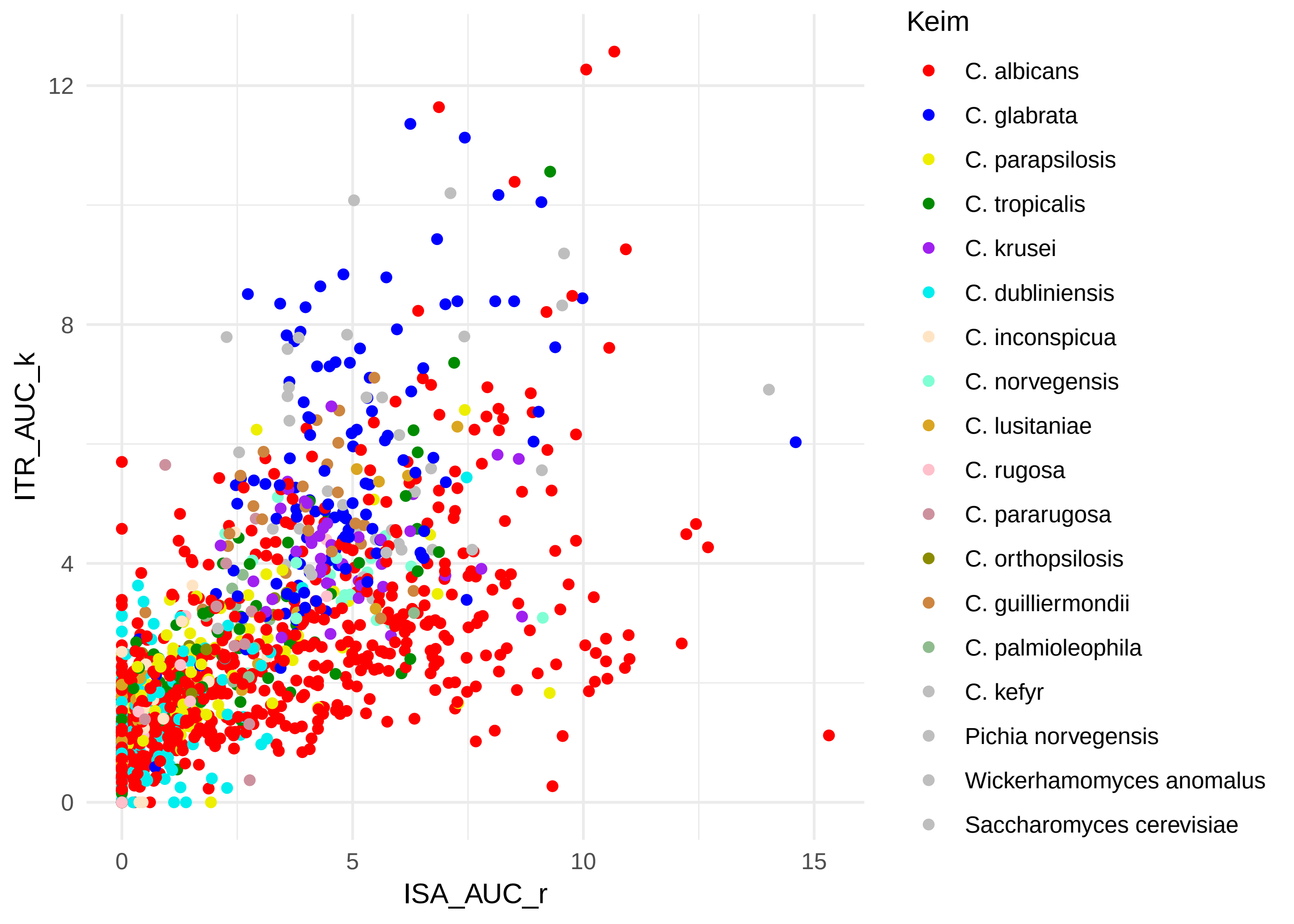
Keim
C. albicans
C. glabrata
C. parapsilosis
C. tropicalis
C. krusei
C. dubliniensis
C. inconspicua
C. norvegensis
C. lusitaniae
C. rugosa
C. pararugosa
C. orthopsilosis
C. guilliermondii
C. palmioleophila
C. kefyr
Pichia norvegensis
Wickerhamomyces anomalus
Saccharomyces cerevisiae
ITR_AUC_k
ISA_AUC_r
0
4
8
12
0
5
10
15

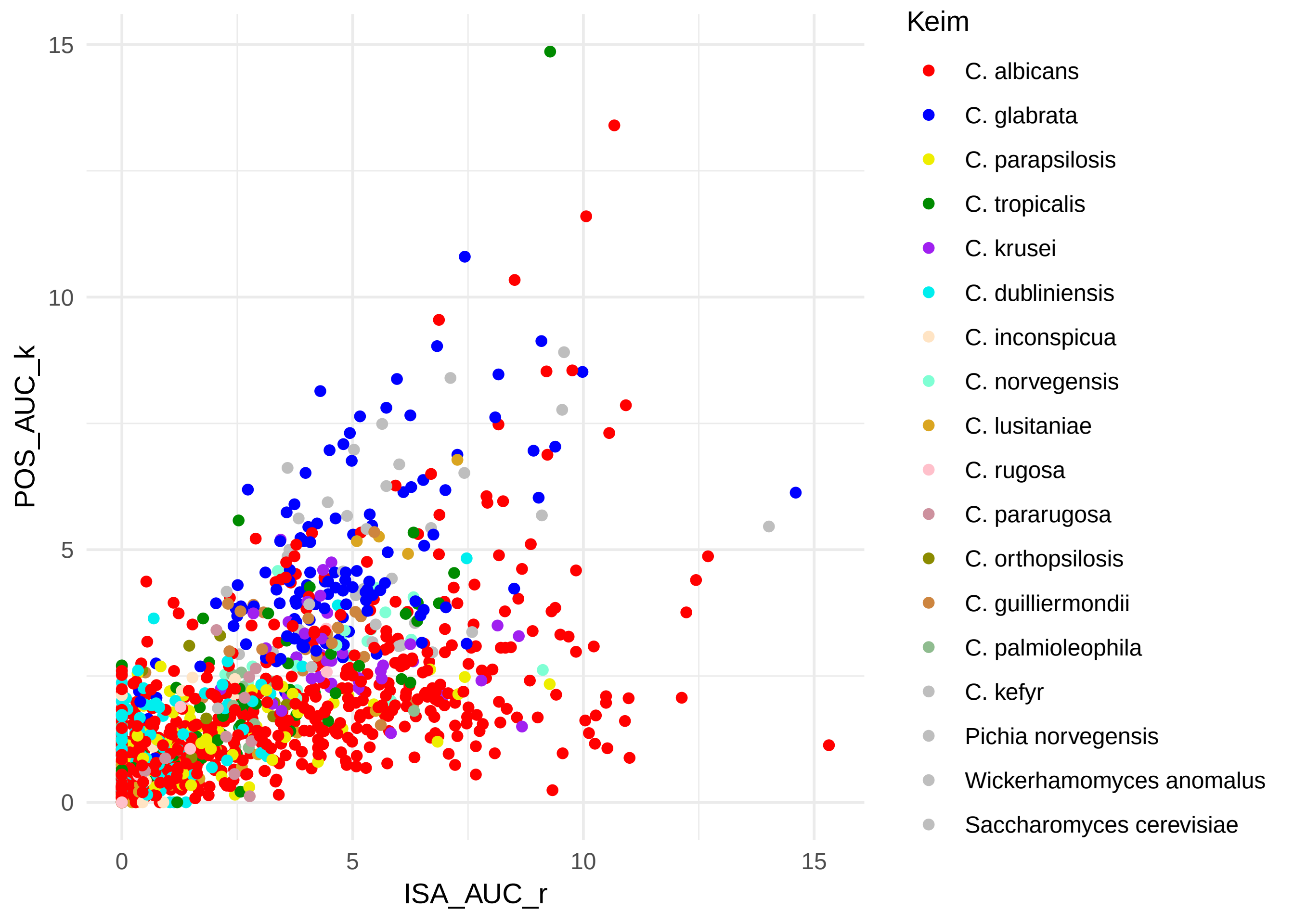
Keim
C. albicans
C. glabrata
C. parapsilosis
C. tropicalis
C. krusei
C. dubliniensis
C. inconspicua
C. norvegensis
C. lusitaniae
C. rugosa
C. pararugosa
C. orthopsilosis
C. guilliermondii
C. palmioleophila
C. kefyr
Pichia norvegensis
Wickerhamomyces anomalus
Saccharomyces cerevisiae
POS_AUC_k
ISA_AUC_r
0
5
10
15
0
5
10
15

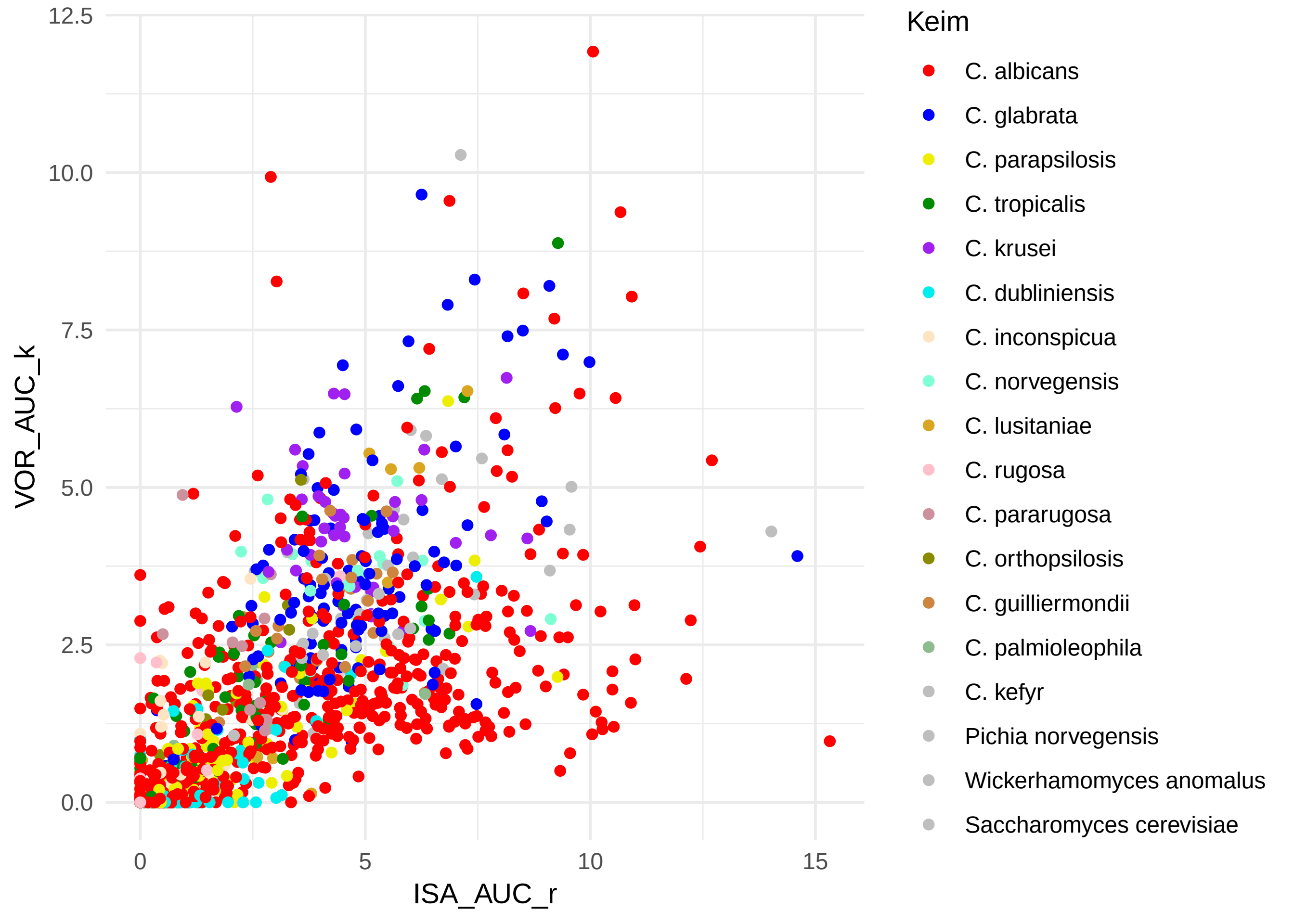

12.5
10.0
7.5
5.0
2.5
0.0
VOR_AUC_k
0
5
10
15
ISA_AUC_r
Keim
C. albicans
C. glabrata
C. parapsilosis
C. tropicalis
C. krusei
C. dubliniensis
C. inconspicua
C. norvegensis
C. lusitaniae
C. rugosa
C. pararugosa
C. orthopsilosis
C. guilliermondii
C. palmioleophila
C. kefyr
Pichia norvegensis
Wickerhamomyces anomalus
Saccharomyces cerevisiae

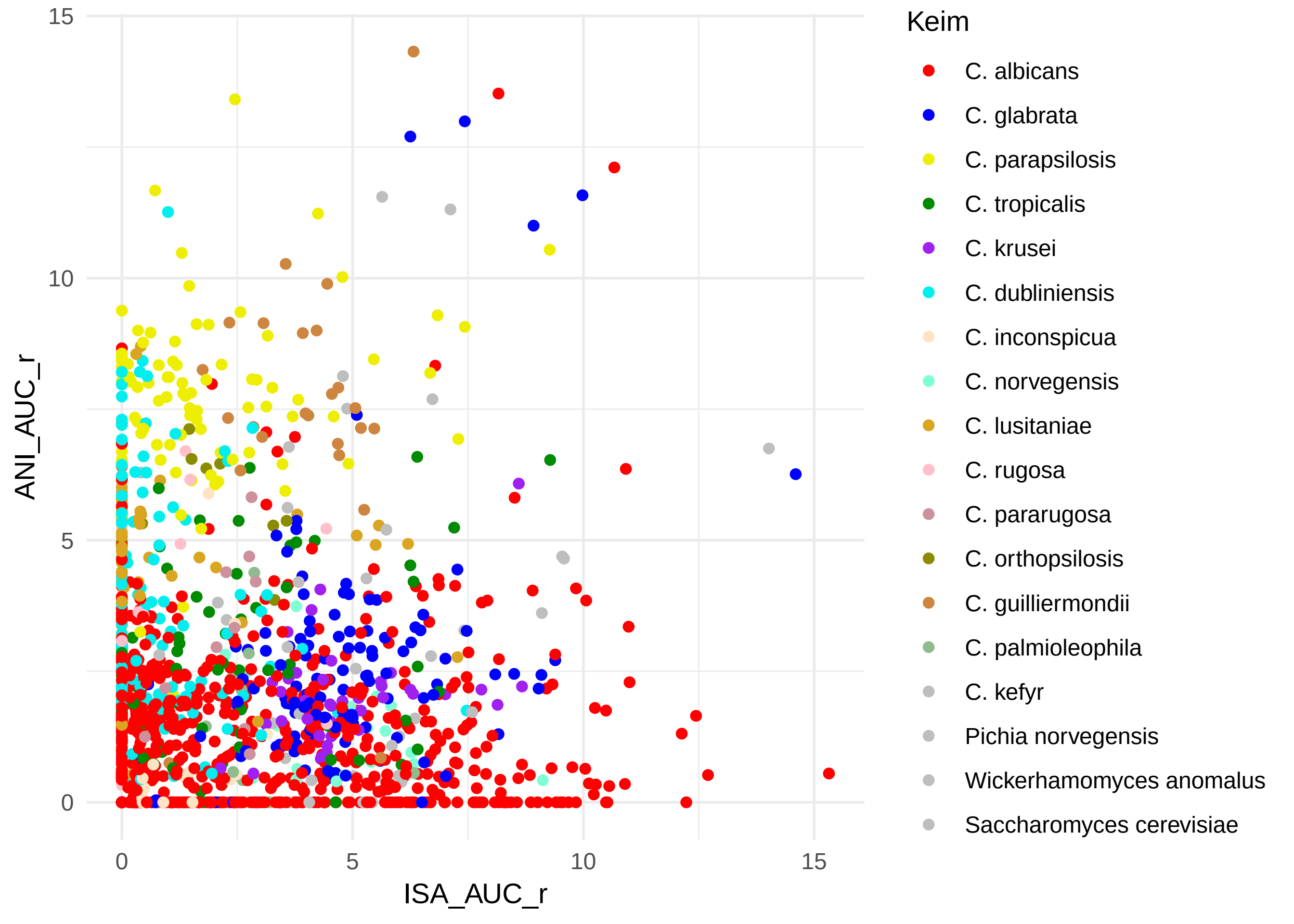
15
10
5
0
ANI_AUC_r
0
5
10
15
ISA_AUC_r
Keim
C. albicans
C. glabrata
C. parapsilosis
C. tropicalis
C. krusei
C. dubliniensis
C. inconspicua
C. norvegensis
C. lusitaniae
C. rugosa
C. pararugosa
C. orthopsilosis
C. guilliermondii
C. palmioleophila
C. kefyr
Pichia norvegensis
Wickerhamomyces anomalus
Saccharomyces cerevisiae

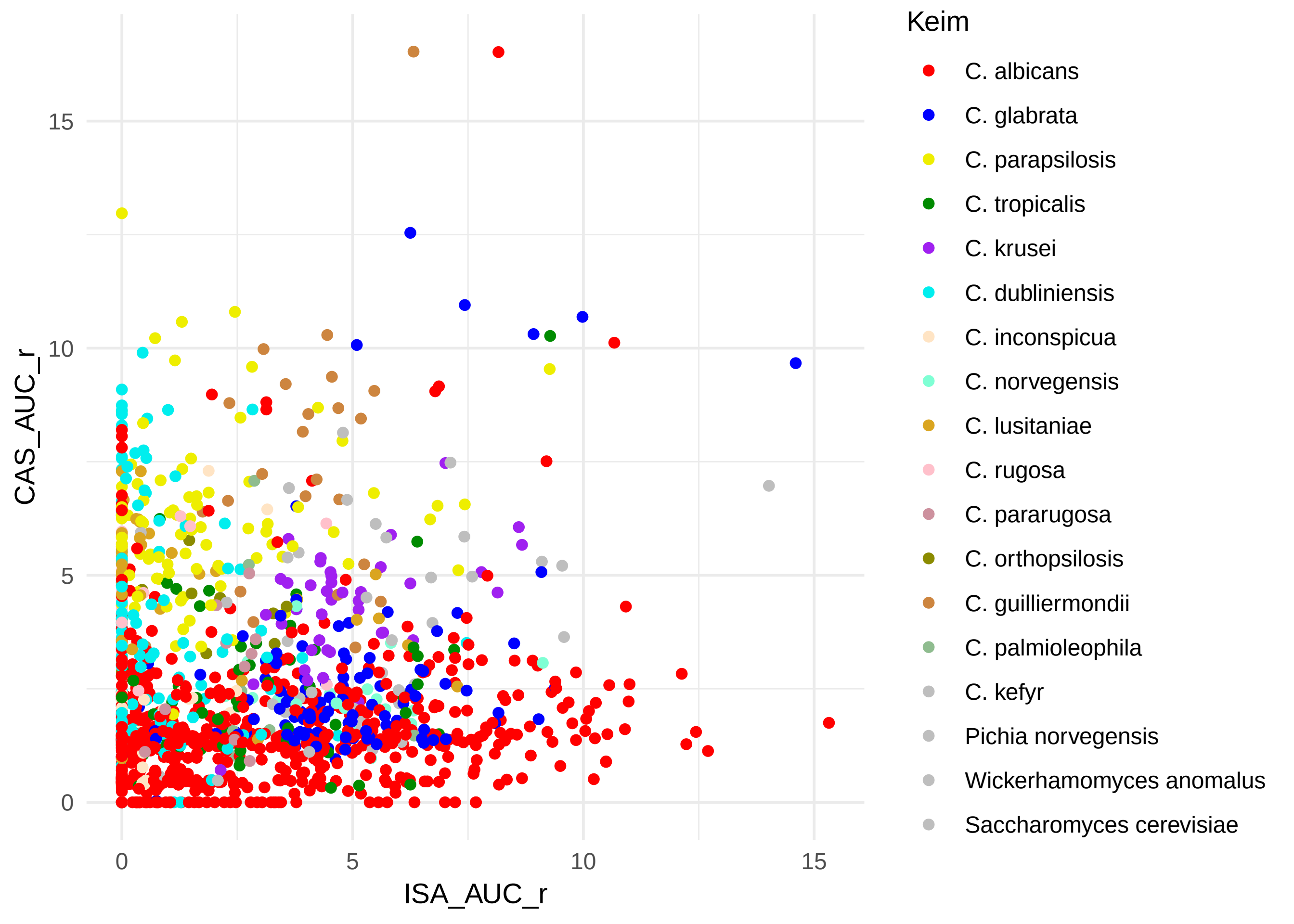
Keim
C. albicans
C. glabrata
C. parapsilosis
C. tropicalis
C. krusei
C. dubliniensis
C. inconspicua
C. norvegensis
C. lusitaniae
C. rugosa
C. pararugosa
C. orthopsilosis
C. guilliermondii
C. palmioleophila
C. kefyr
Pichia norvegensis
Wickerhamomyces anomalus
Saccharomyces cerevisiae
CAS_AUC_r
ISA_AUC_r
0
5
10
15
0
5
10
15

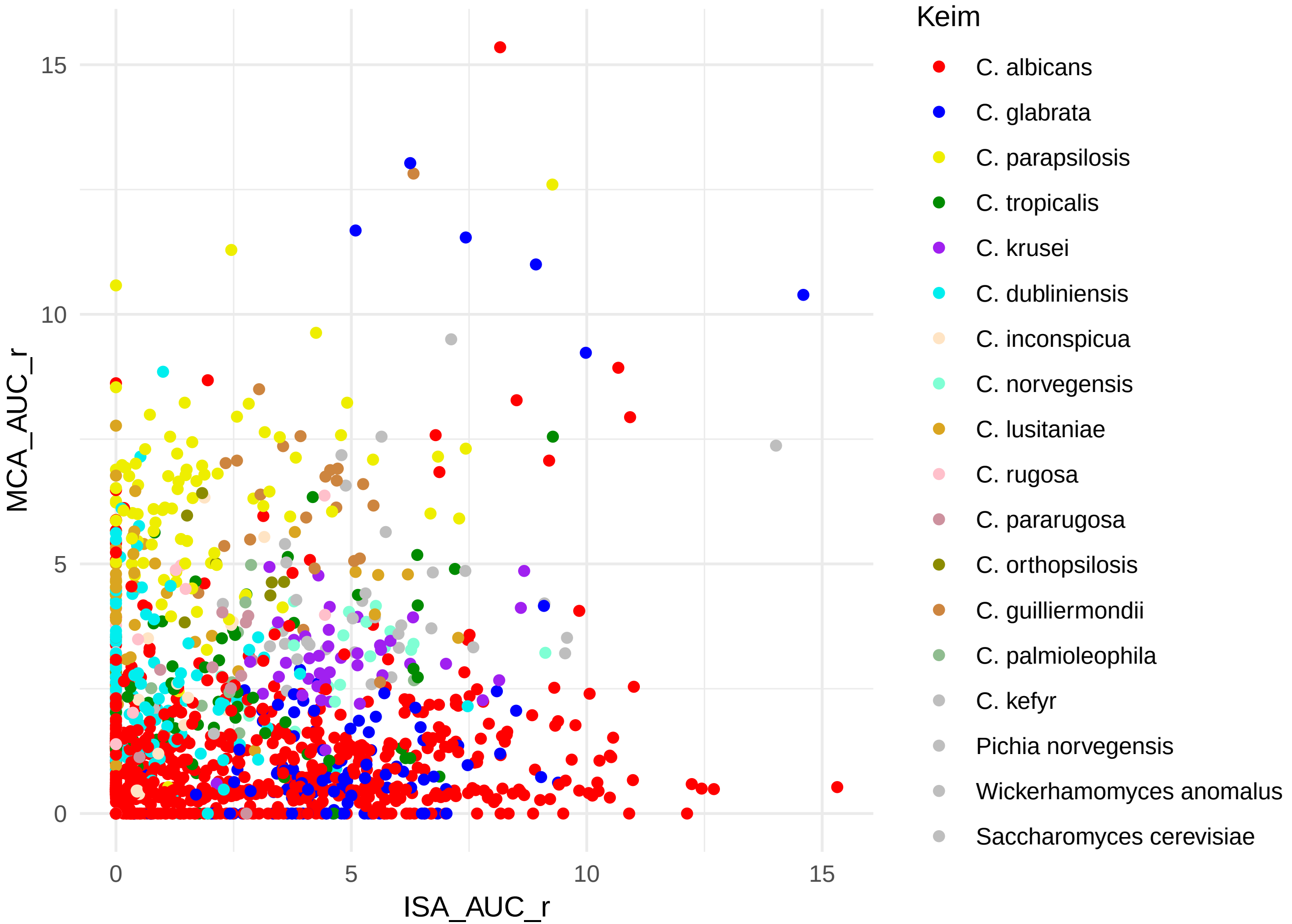

Keim
C. albicans
C. glabrata
C. parapsilosis
C. tropicalis
C. krusei
C. dubliniensis
C. inconspicua
C. norvegensis
C. lusitaniae
C. rugosa
C. pararugosa
C. orthopsilosis
C. guilliermondii
C. palmioleophila
C. kefyr
Pichia norvegensis
Wickerhamomyces anomalus
Saccharomyces cerevisiae
MCA_AUC_r
ISA_AUC_r
0
5
10
15

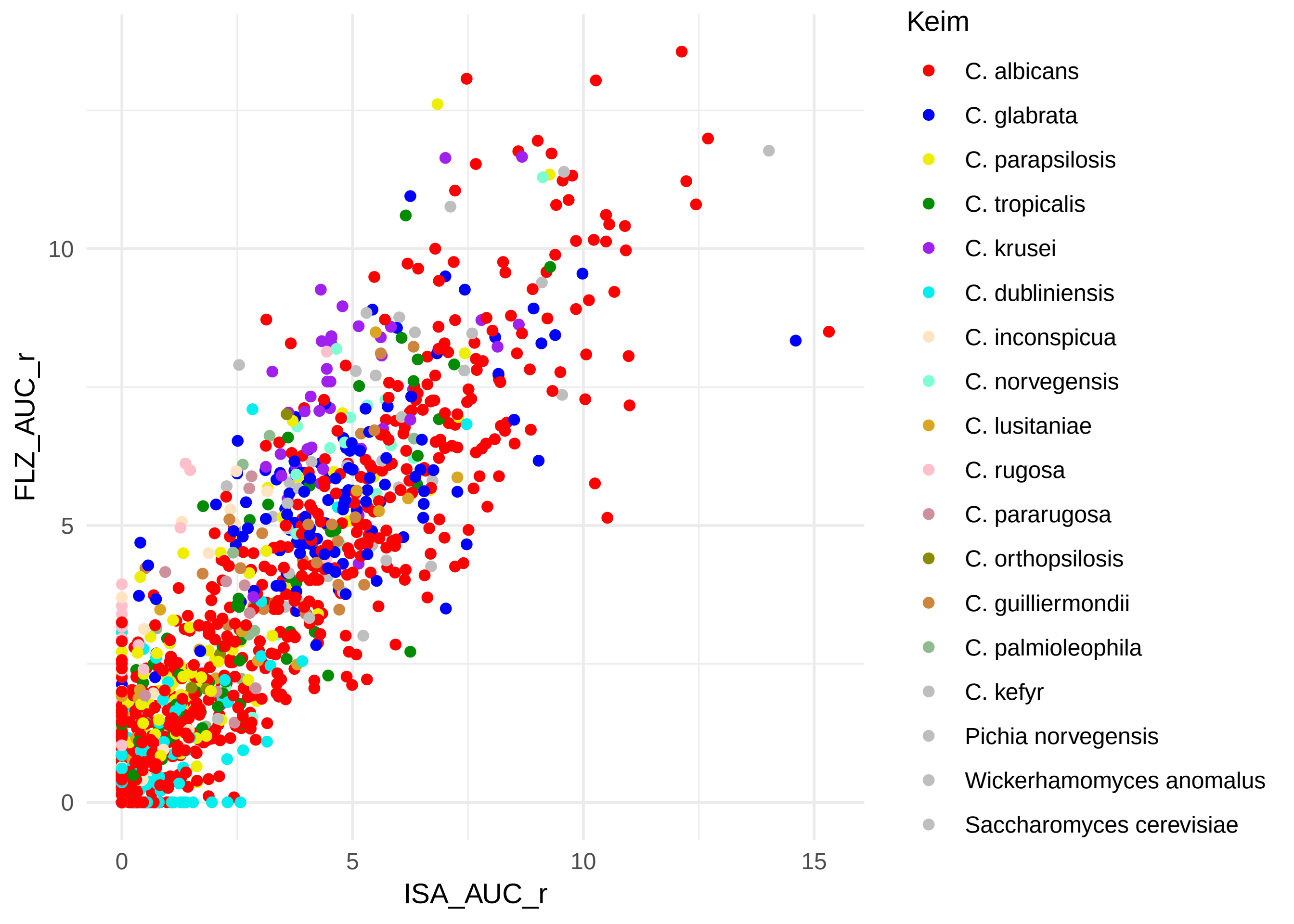

Keim
C. albicans
C. glabrata
C. parapsilosis
C. tropicalis
C. krusei
C. dubliniensis
C. inconspicua
C. norvegensis
C. lusitaniae
C. rugosa
C. pararugosa
C. orthopsilosis
C. guilliermondii
C. palmioleophila
C. kefyr
Pichia norvegensis
Wickerhamomyces anomalus
Saccharomyces cerevisiae
FLZ_AUC_r
10
5
0
0
5
10
15
ISA_AUC_r

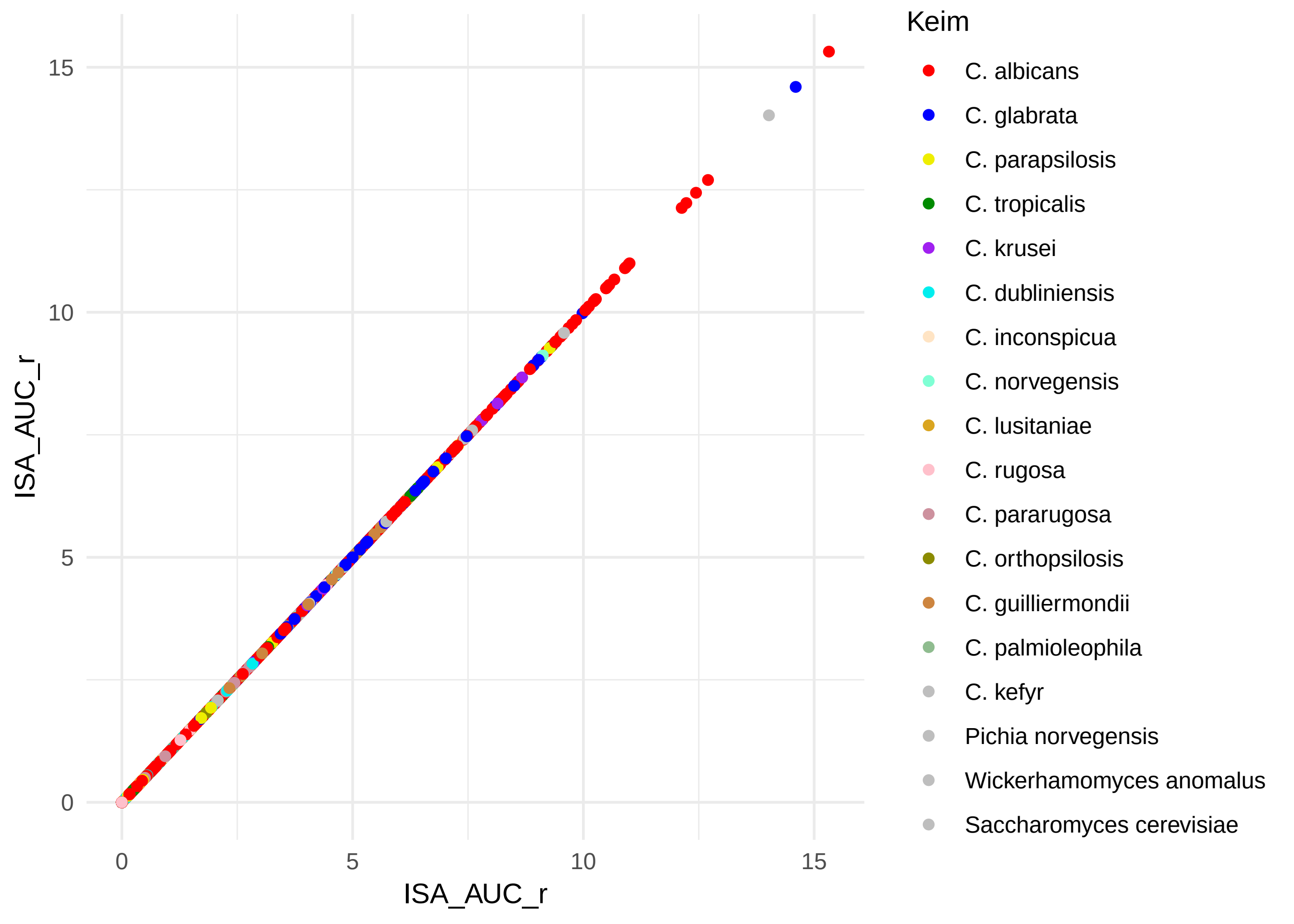
Keim
C. albicans
C. glabrata
C. parapsilosis
C. tropicalis
C. krusei
C. dubliniensis
C. inconspicua
C. norvegensis
C. lusitaniae
C. rugosa
C. pararugosa
C. orthopsilosis
C. guilliermondii
C. palmioleophila
C. kefyr
Pichia norvegensis
Wickerhamomyces anomalus
Saccharomyces cerevisiae
ISA_AUC_r
ISA_AUC_r
0
5
10
15
0
5
10
15

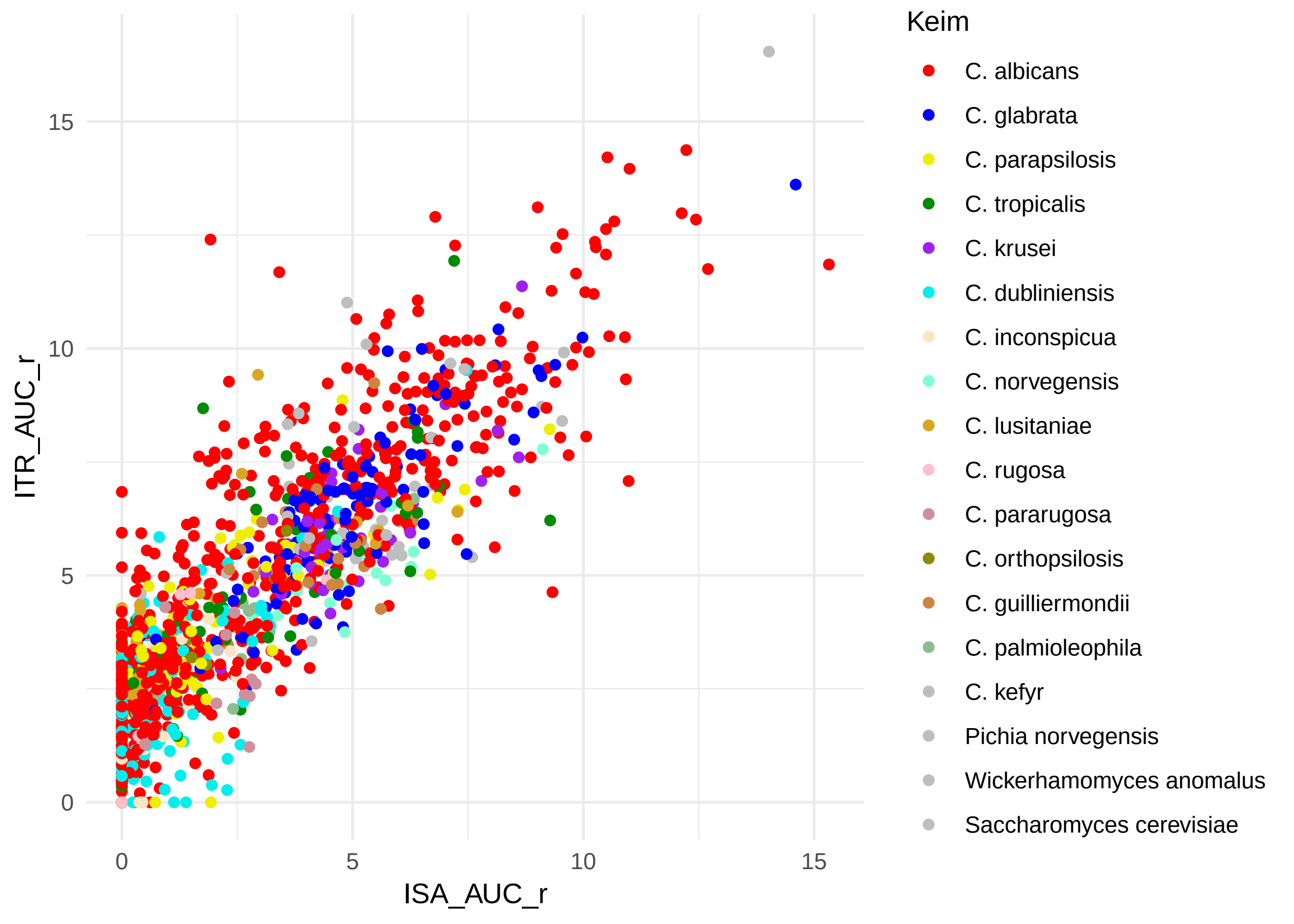

Keim
C. albicans
C. glabrata
C. parapsilosis
C. tropicalis
C. krusei
C. dubliniensis
C. inconspicua
C. norvegensis
C. lusitaniae
C. rugosa
C. pararugosa
C. orthopsilosis
C. guilliermondii
C. palmioleophila
C. kefyr
Pichia norvegensis
Wickerhamomyces anomalus
Saccharomyces cerevisiae
ITR_AUC_r
ISA_AUC_r
0
5
10
15

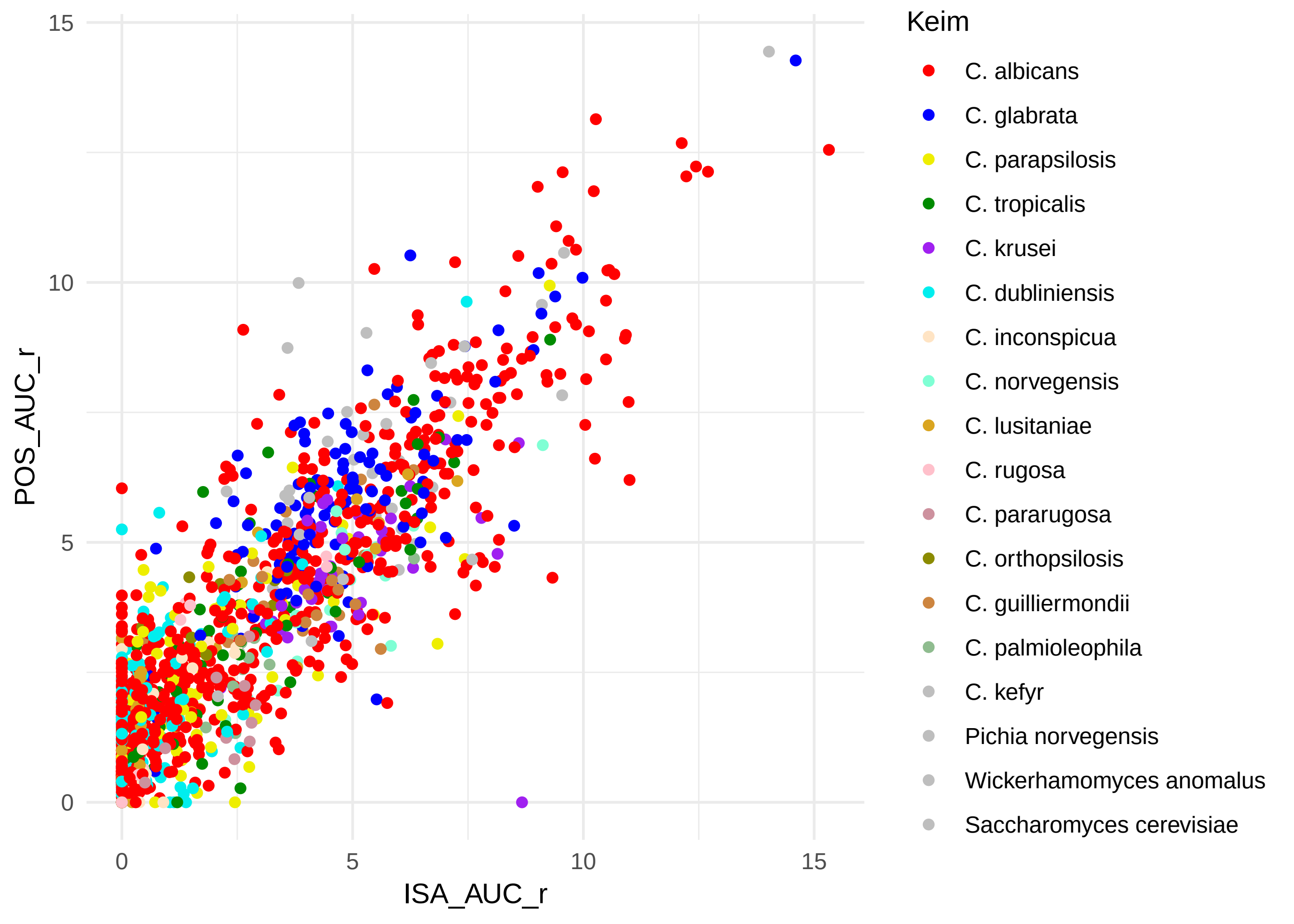

Keim
C. albicans
C. glabrata
C. parapsilosis
C. tropicalis
C. krusei
C. dubliniensis
C. inconspicua
C. norvegensis
C. lusitaniae
C. rugosa
C. pararugosa
C. orthopsilosis
C. guilliermondii
C. palmioleophila
C. kefyr
Pichia norvegensis
Wickerhamomyces anomalus
Saccharomyces cerevisiae
POS_AUC_r
ISA_AUC_r
0
5
10
15

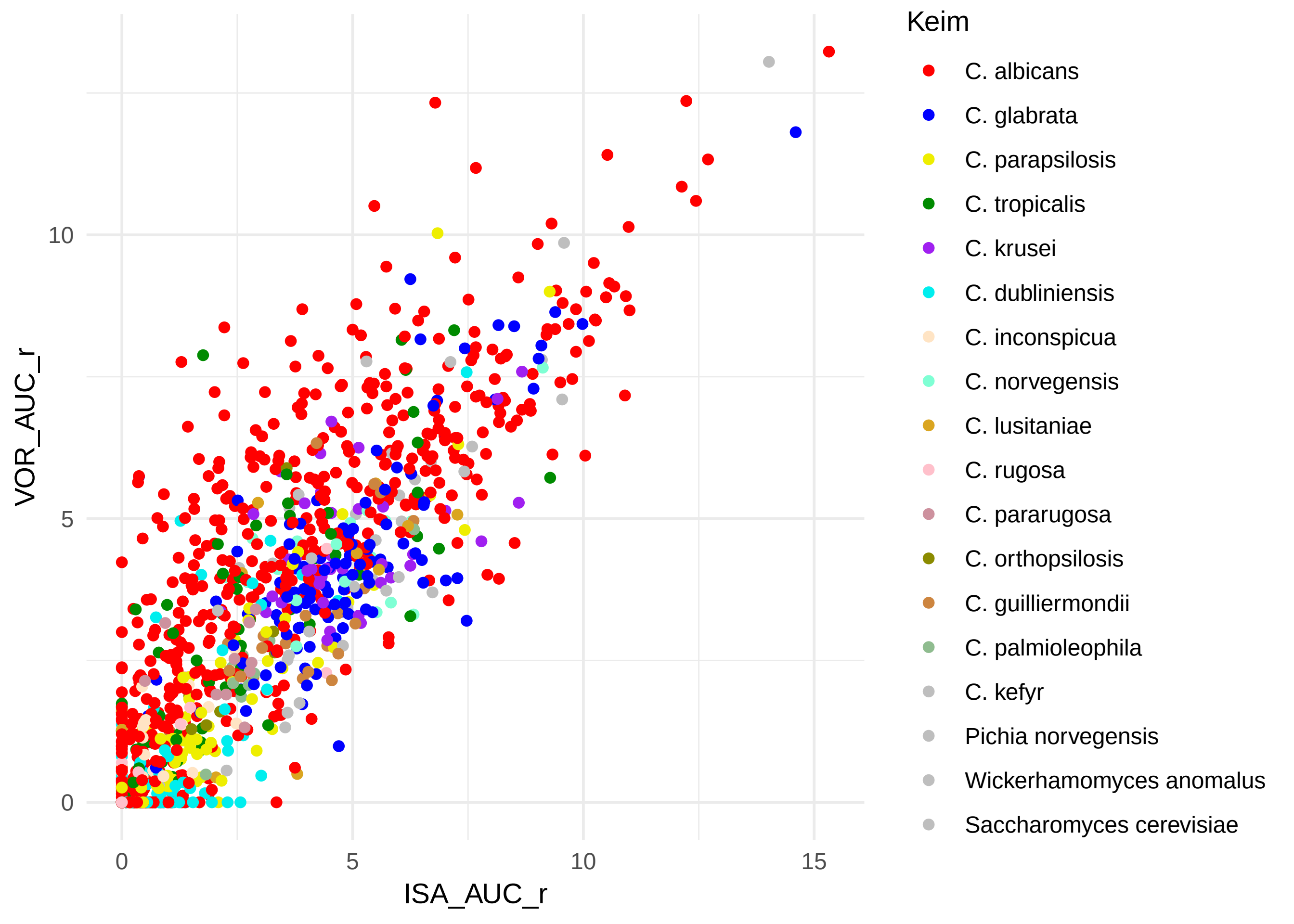

Keim
C. albicans
C. glabrata
C. parapsilosis
C. tropicalis
C. krusei
C. dubliniensis
C. inconspicua
C. norvegensis
C. lusitaniae
C. rugosa
C. pararugosa
C. orthopsilosis
C. guilliermondii
C. palmioleophila
C. kefyr
Pichia norvegensis
Wickerhamomyces anomalus
Saccharomyces cerevisiae
VOR_AUC_r
ISA_AUC_r
0
5
10
15

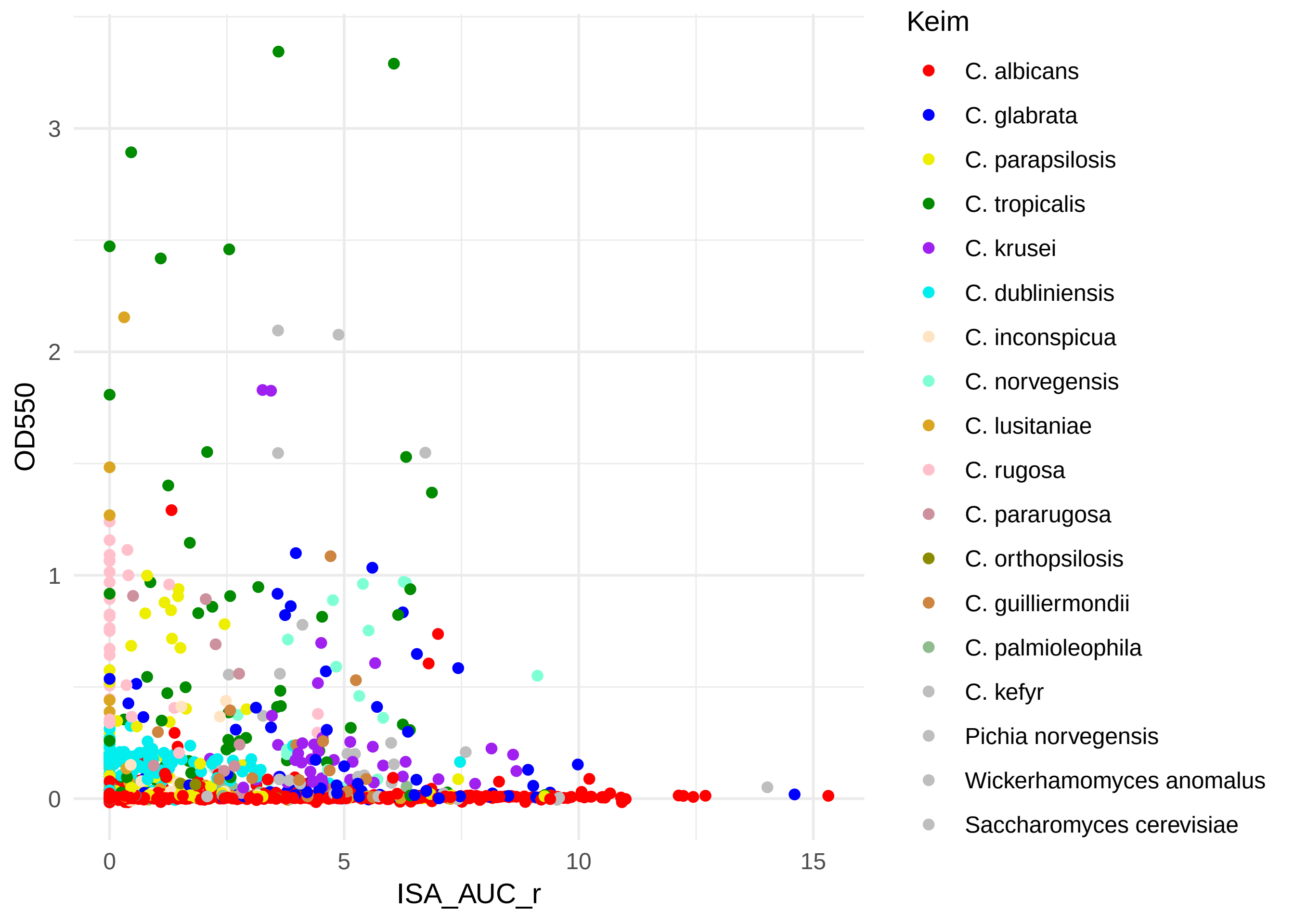
Keim
C. albicans
C. glabrata
C. parapsilosis
C. tropicalis
C. krusei
C. dubliniensis
C. inconspicua
C. norvegensis
C. lusitaniae
C. rugosa
C. pararugosa
C. orthopsilosis
C. guilliermondii
C. palmioleophila
C. kefyr
Pichia norvegensis
Wickerhamomyces anomalus
Saccharomyces cerevisiae
OD550
ISA_AUC_r
0
1
2
3
0
5
10
15

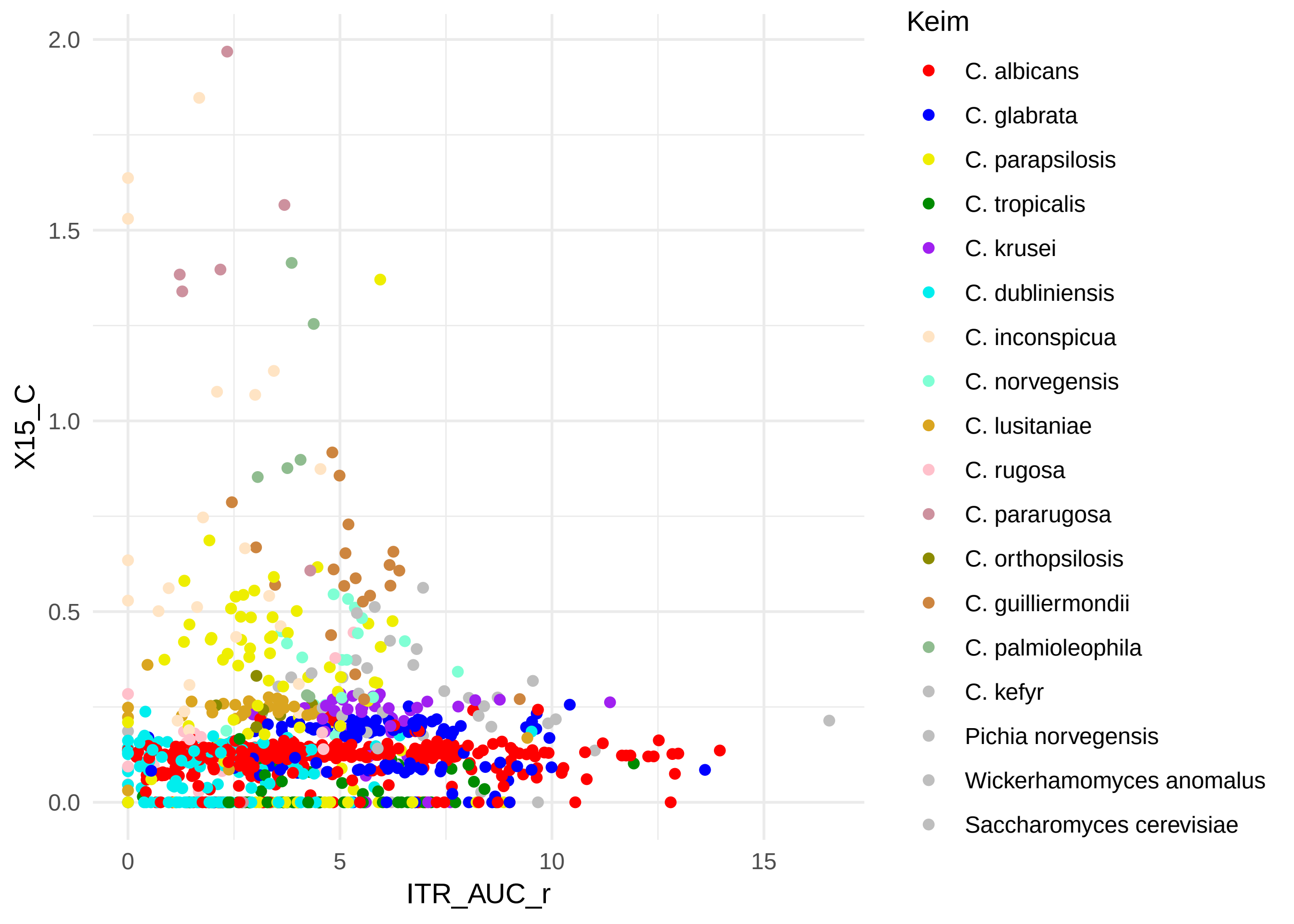
Keim
C. albicans
C. glabrata
C. parapsilosis
C. tropicalis
C. krusei
C. dubliniensis
C. inconspicua
C. norvegensis
C. lusitaniae
C. rugosa
C. pararugosa
C. orthopsilosis
C. guilliermondii
C. palmioleophila
C. kefyr
Pichia norvegensis
Wickerhamomyces anomalus
Saccharomyces cerevisiae
X15_C
2.0
1.5
1.0
0.5
0.0
0
5
10
15
ITR_AUC_r

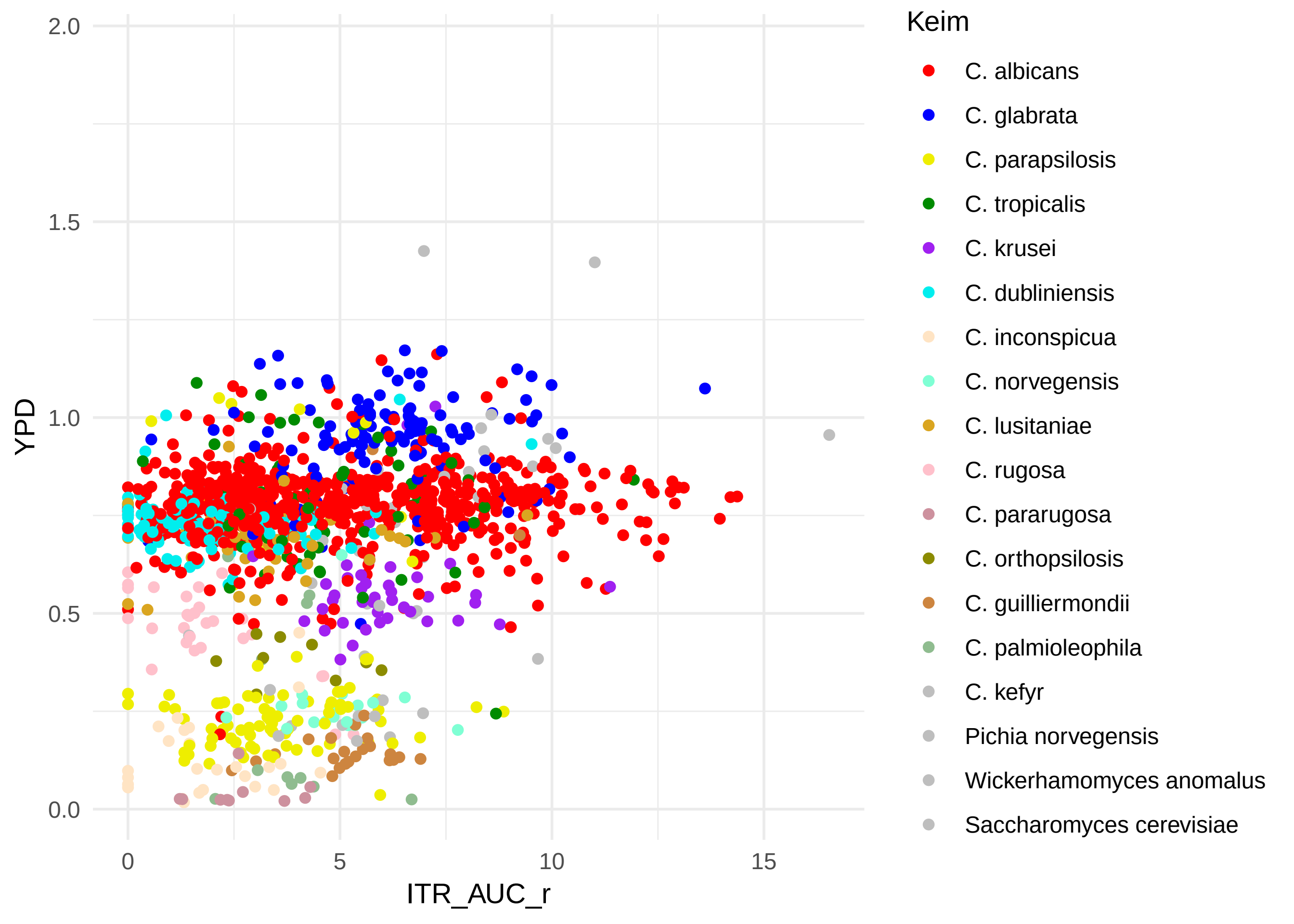

Keim
C. albicans
C. glabrata
C. parapsilosis
C. tropicalis
C. krusei
C. dubliniensis
C. inconspicua
C. norvegensis
C. lusitaniae
C. rugosa
C. pararugosa
C. orthopsilosis
C. guilliermondii
C. palmioleophila
C. kefyr
Pichia norvegensis
Wickerhamomyces anomalus
Saccharomyces cerevisiae
YPD
2.0
1.5
1.0
0.5
0.0
0
5
10
15
ITR_AUC_r

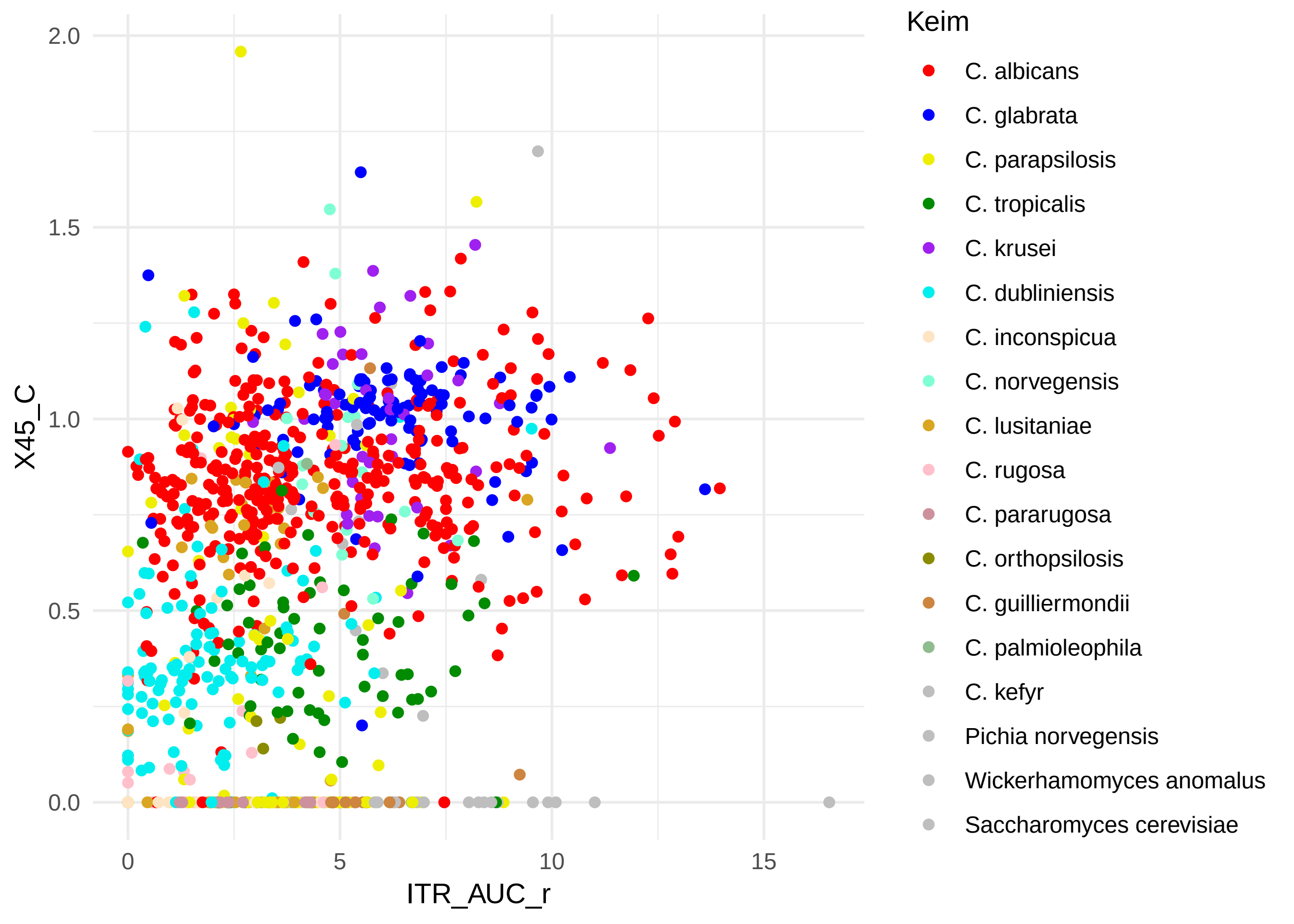

2.0
1.5
1.0
0.5
0.0
X45_C
0
5
10
15
ITR_AUC_r
Keim
C. albicans
C. glabrata
C. parapsilosis
C. tropicalis
C. krusei
C. dubliniensis
C. inconspicua
C. norvegensis
C. lusitaniae
C. rugosa
C. pararugosa
C. orthopsilosis
C. guilliermondii
C. palmioleophila
C. kefyr
Pichia norvegensis
Wickerhamomyces anomalus
Saccharomyces cerevisiae

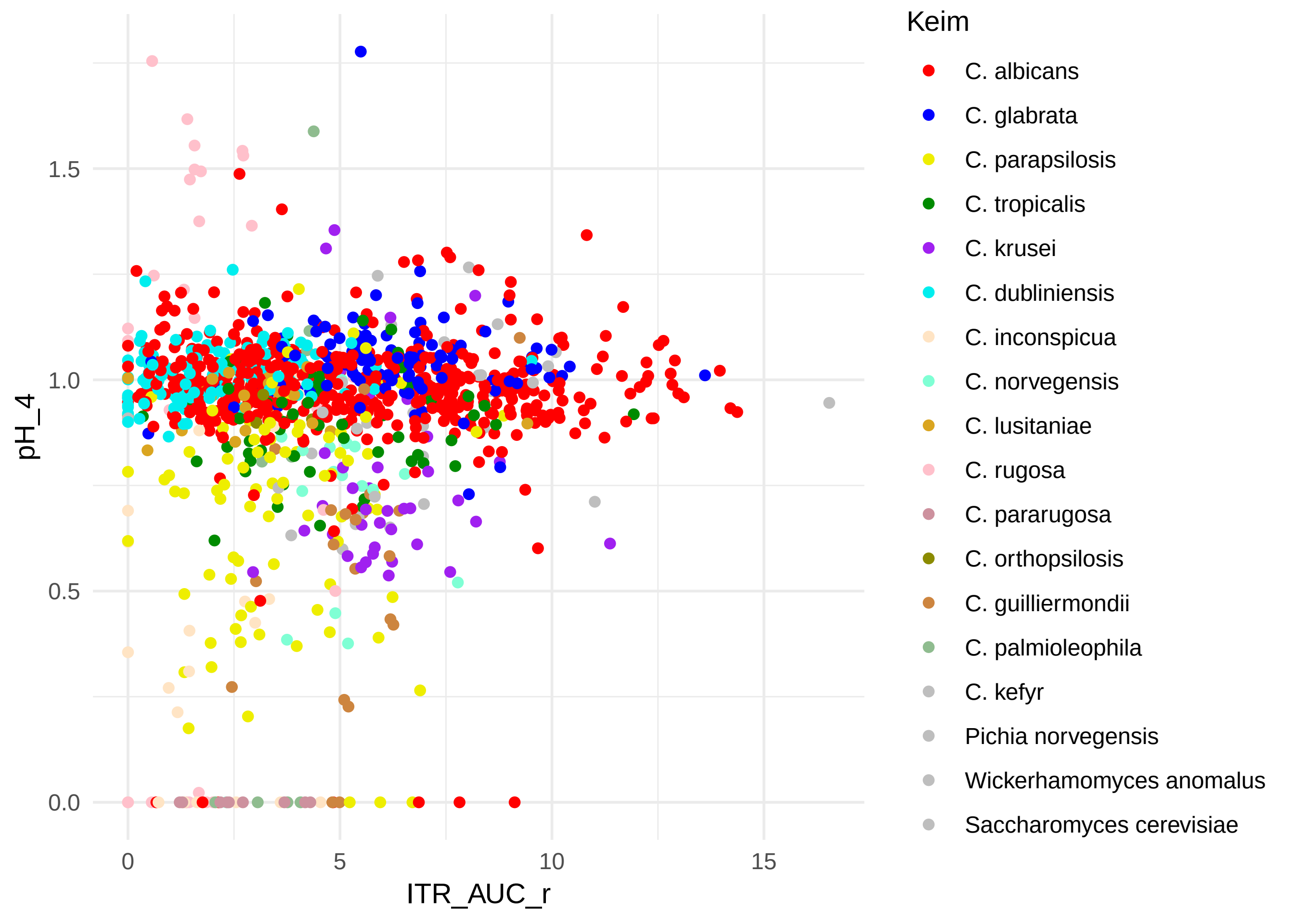

pH_4
ITR_AUC_r
0
5
10
15
0.0
0.5
1.0
1.5
Keim
C. albicans
C. glabrata
C. parapsilosis
C. tropicalis
C. krusei
C. dubliniensis
C. inconspicua
C. norvegensis
C. lusitaniae
C. rugosa
C. pararugosa
C. orthopsilosis
C. guilliermondii
C. palmioleophila
C. kefyr
Pichia norvegensis
Wickerhamomyces anomalus
Saccharomyces cerevisiae

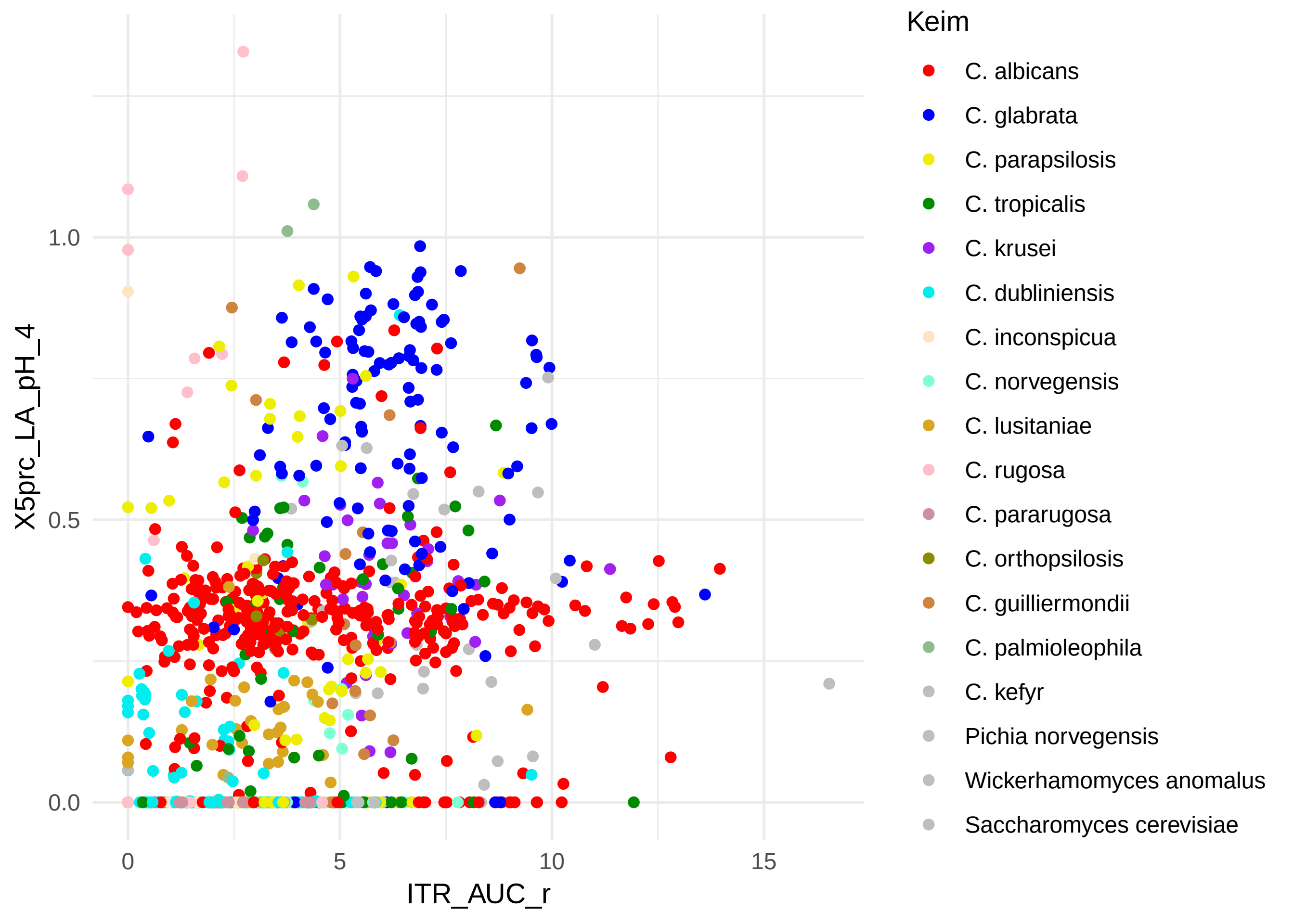

Keim
C. albicans
C. glabrata
C. parapsilosis
C. tropicalis
C. krusei
C. dubliniensis
C. inconspicua
C. norvegensis
C. lusitaniae
C. rugosa
C. pararugosa
C. orthopsilosis
C. guilliermondii
C. palmioleophila
C. kefyr
Pichia norvegensis
Wickerhamomyces anomalus
Saccharomyces cerevisiae
X5prc_LA_pH_4
1.0
0.5
0.0
0
5
10
15
ITR_AUC_r

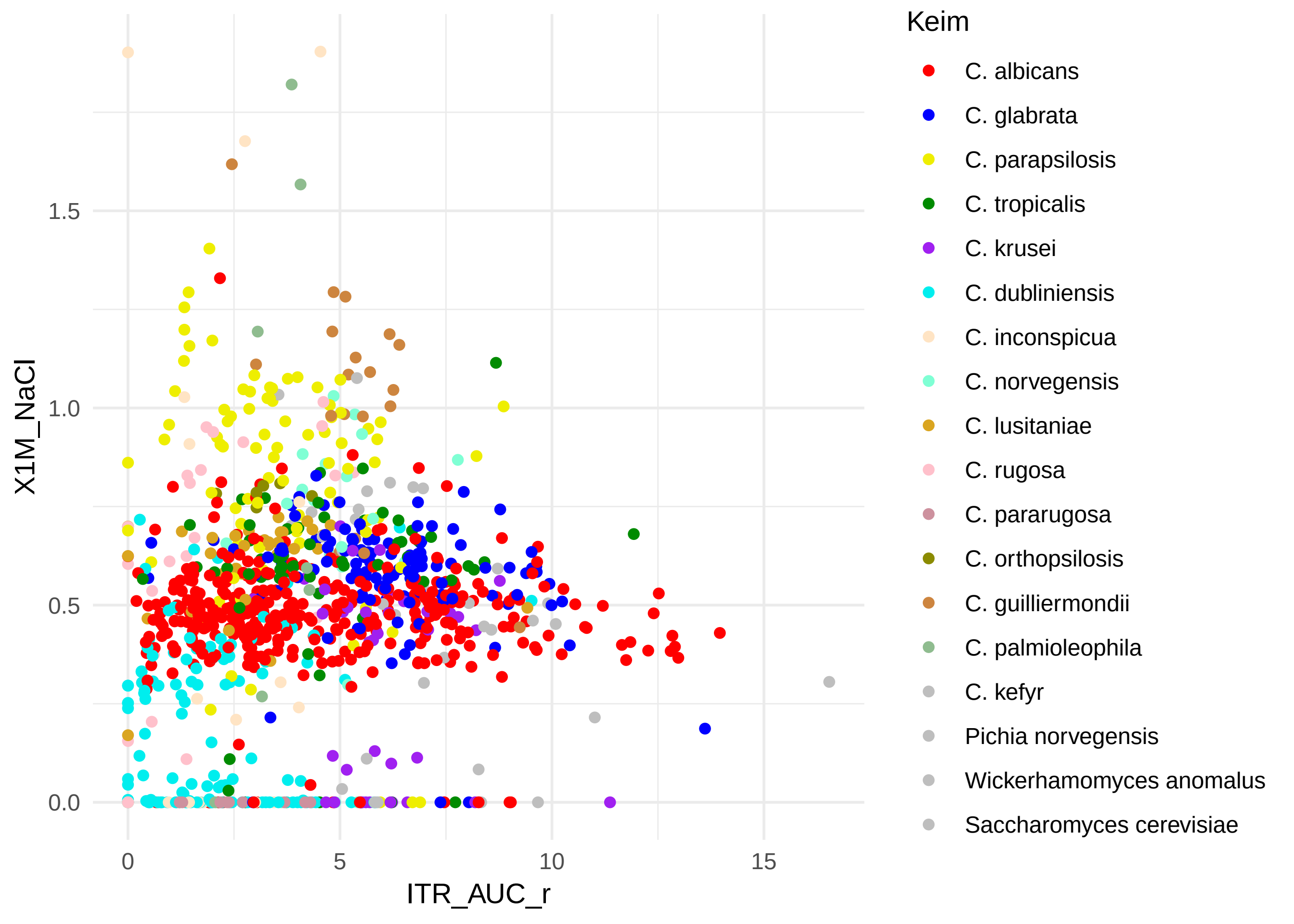

Keim
C. albicans
C. glabrata
C. parapsilosis
C. tropicalis
C. krusei
C. dubliniensis
C. inconspicua
C. norvegensis
C. lusitaniae
C. rugosa
C. pararugosa
C. orthopsilosis
C. guilliermondii
C. palmioleophila
C. kefyr
Pichia norvegensis
Wickerhamomyces anomalus
Saccharomyces cerevisiae
X1M_NaCl
ITR_AUC_r
0.0
0.5
1.0
1.5
0
5
10
15

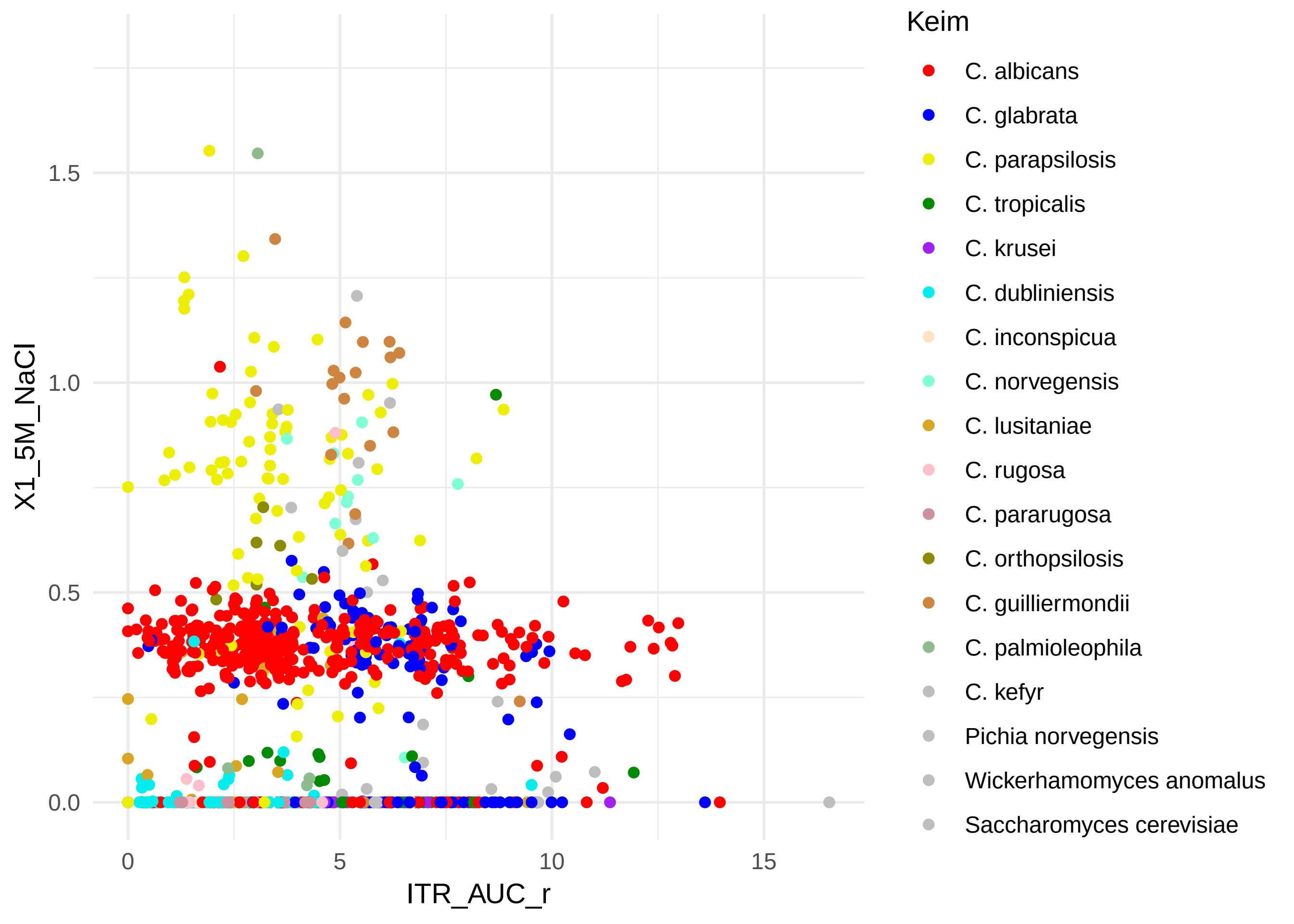
Keim
C. albicans
C. glabrata
C. parapsilosis
C. tropicalis
C. krusei
C. dubliniensis
C. inconspicua
C. norvegensis
C. lusitaniae
C. rugosa
C. pararugosa
C. orthopsilosis
C. guilliermondii
C. palmioleophila
C. kefyr
Pichia norvegensis
Wickerhamomyces anomalus
Saccharomyces cerevisiae
X1_5M_NaCl
1.5
1.0
0.5
0.0
0
5
10
15
ITR_AUC_r

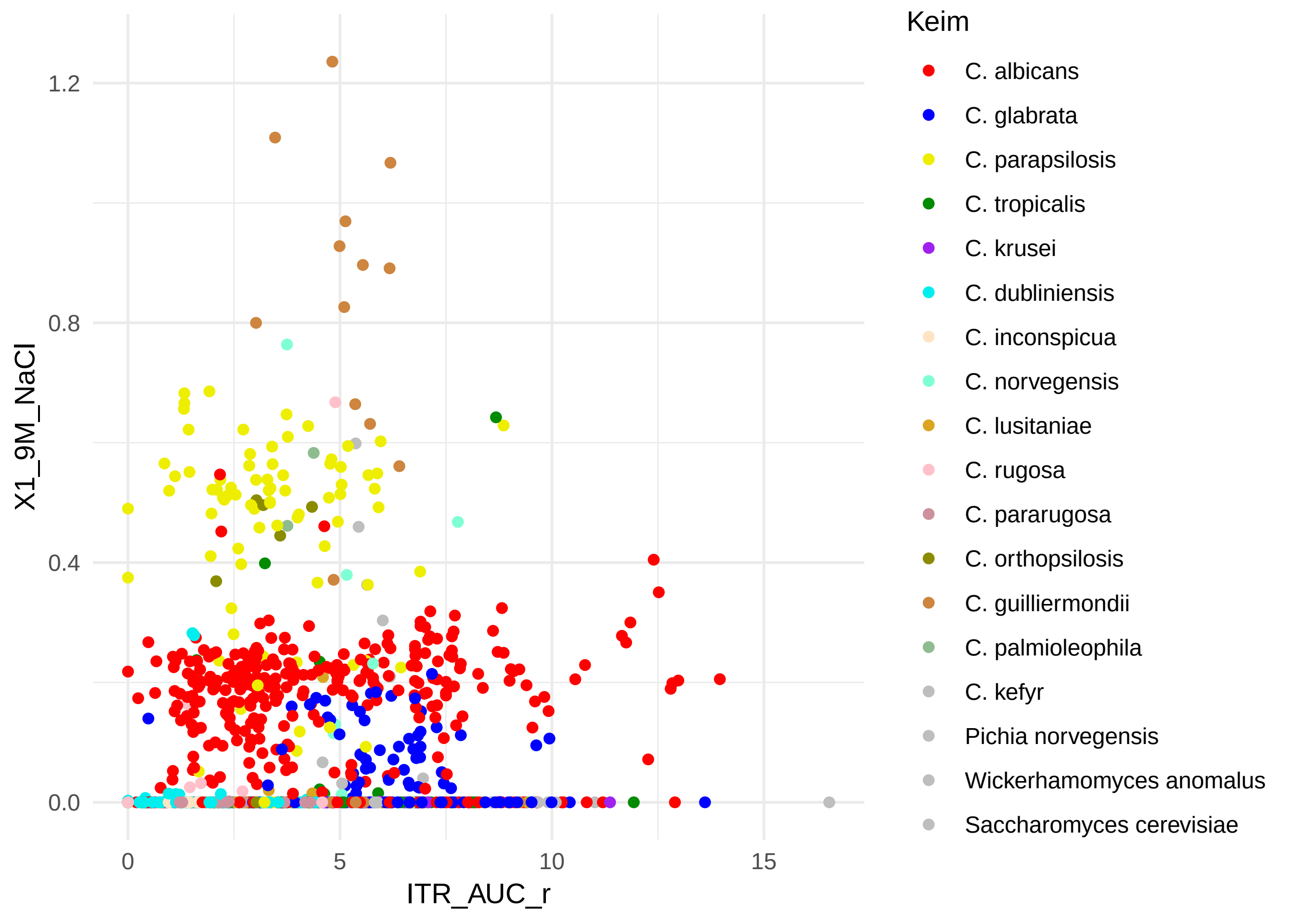

Keim
C. albicans
C. glabrata
C. parapsilosis
C. tropicalis
C. krusei
C. dubliniensis
C. inconspicua
C. norvegensis
C. lusitaniae
C. rugosa
C. pararugosa
C. orthopsilosis
C. guilliermondii
C. palmioleophila
C. kefyr
Pichia norvegensis
Wickerhamomyces anomalus
Saccharomyces cerevisiae
X1_9M_NaCl
1.2
0.8
0.4
0.0
0
5
10
15
ITR_AUC_r

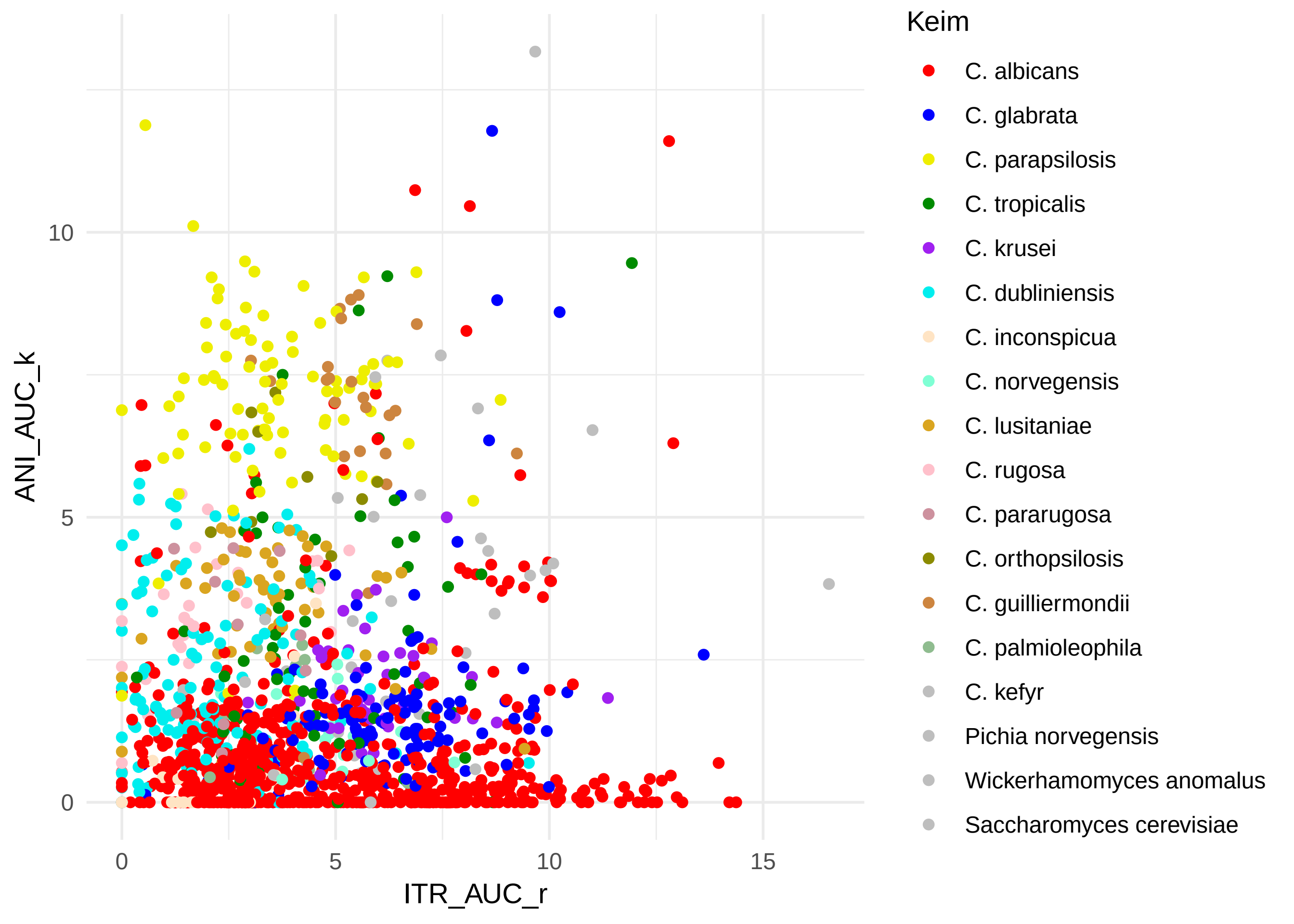
Keim
C. albicans
C. glabrata
C. parapsilosis
C. tropicalis
C. krusei
C. dubliniensis
C. inconspicua
C. norvegensis
C. lusitaniae
C. rugosa
C. pararugosa
C. orthopsilosis
C. guilliermondii
C. palmioleophila
C. kefyr
Pichia norvegensis
Wickerhamomyces anomalus
Saccharomyces cerevisiae
ANI_AUC_k
ITR_AUC_r
0
5
10
15

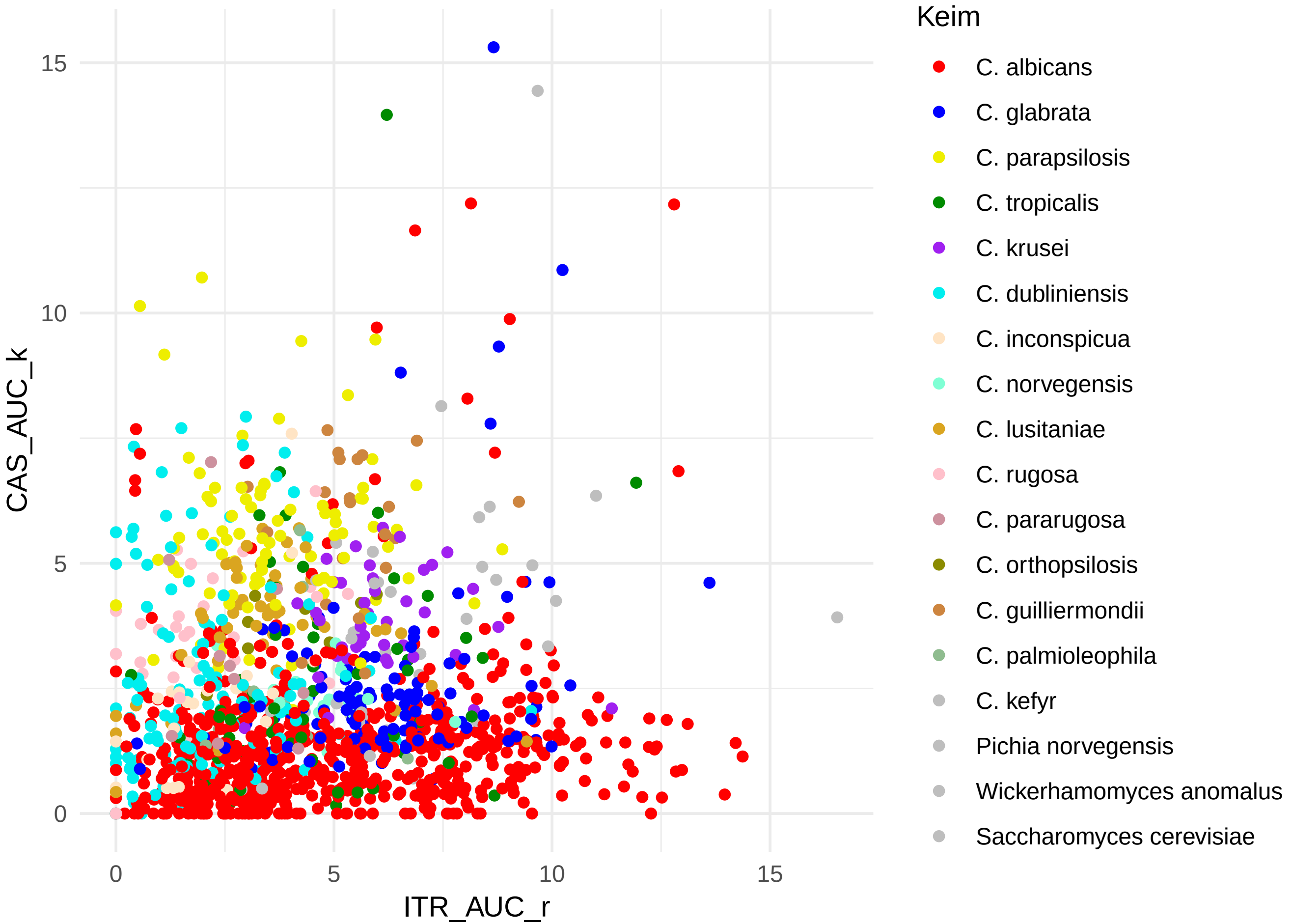
Keim
C. albicans
C. glabrata
C. parapsilosis
C. tropicalis
C. krusei
C. dubliniensis
C. inconspicua
C. norvegensis
C. lusitaniae
C. rugosa
C. pararugosa
C. orthopsilosis
C. guilliermondii
C. palmioleophila
C. kefyr
Pichia norvegensis
Wickerhamomyces anomalus
Saccharomyces cerevisiae
CAS_AUC_k
ITR_AUC_r
0
5
10
15

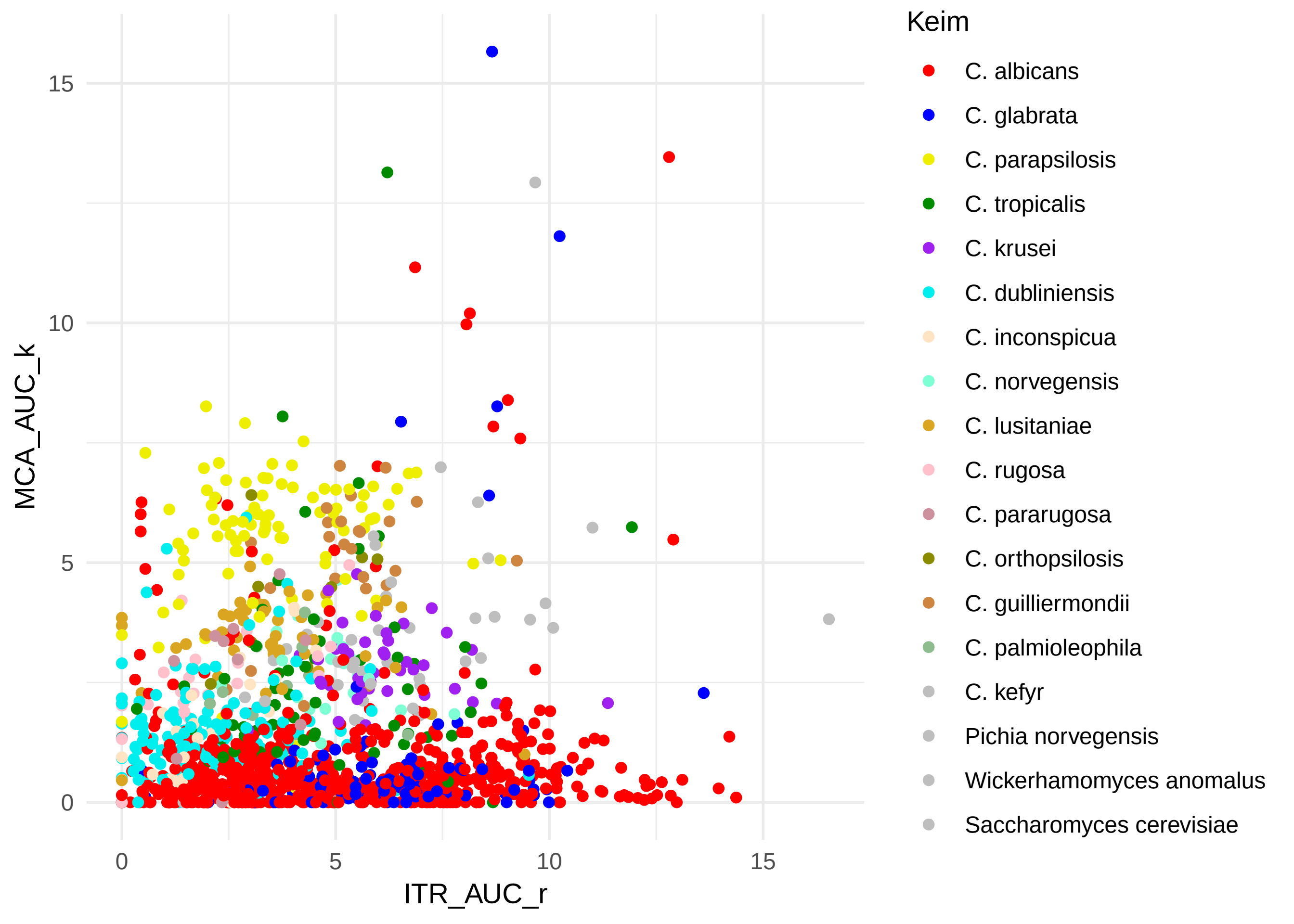
MCA_AUC_k
ITR_AUC_r
0
5
10
15
Keim
C. albicans
C. glabrata
C. parapsilosis
C. tropicalis
C. krusei
C. dubliniensis
C. inconspicua
C. norvegensis
C. lusitaniae
C. rugosa
C. pararugosa
C. orthopsilosis
C. guilliermondii
C. palmioleophila
C. kefyr
Pichia norvegensis
Wickerhamomyces anomalus
Saccharomyces cerevisiae

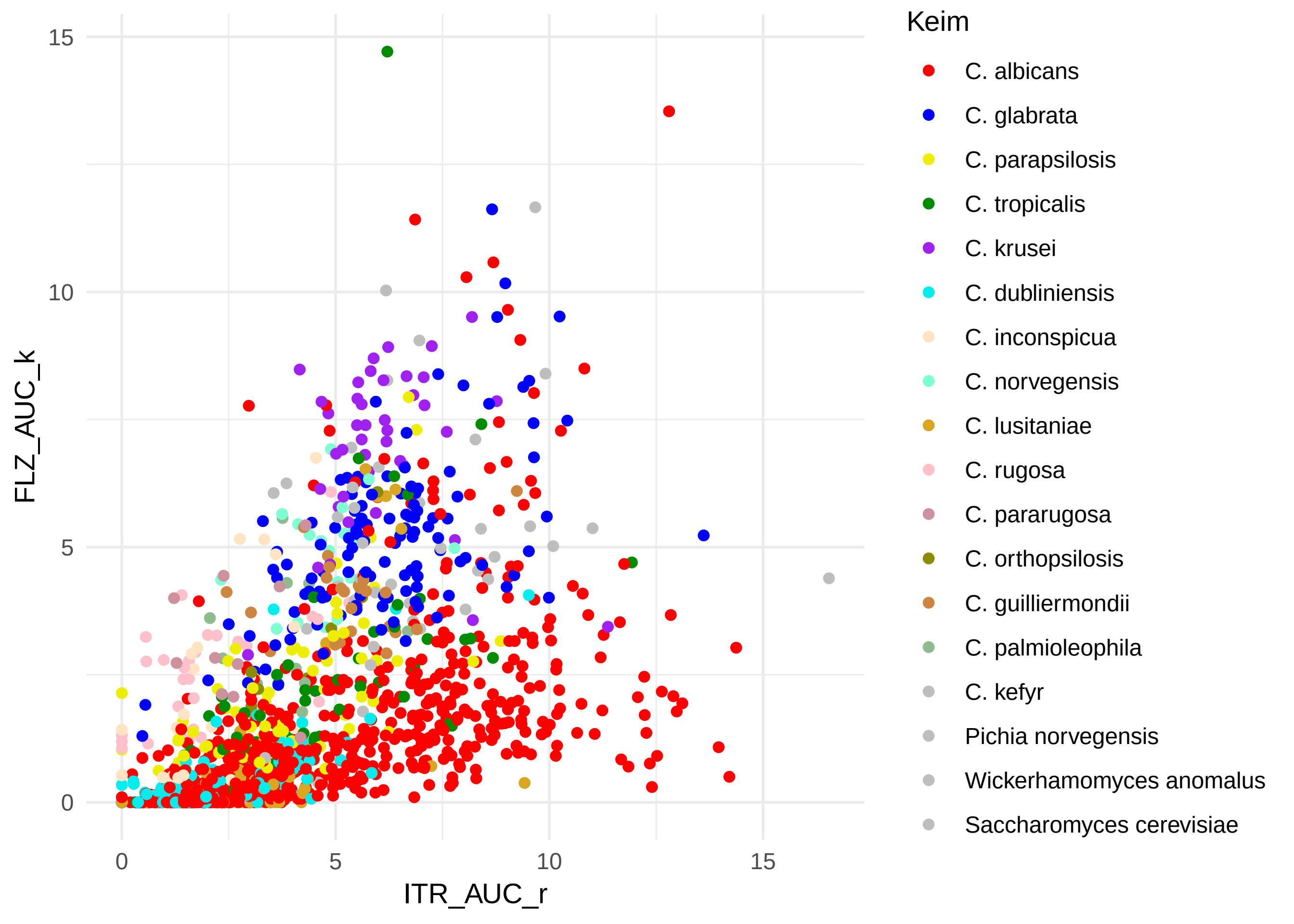
Keim
C. albicans
C. glabrata
C. parapsilosis
C. tropicalis
C. krusei
C. dubliniensis
C. inconspicua
C. norvegensis
C. lusitaniae
C. rugosa
C. pararugosa
C. orthopsilosis
C. guilliermondii
C. palmioleophila
C. kefyr
Pichia norvegensis
Wickerhamomyces anomalus
Saccharomyces cerevisiae
FLZ_AUC_k
ITR_AUC_r
0
5
10
15
0
5
10
15

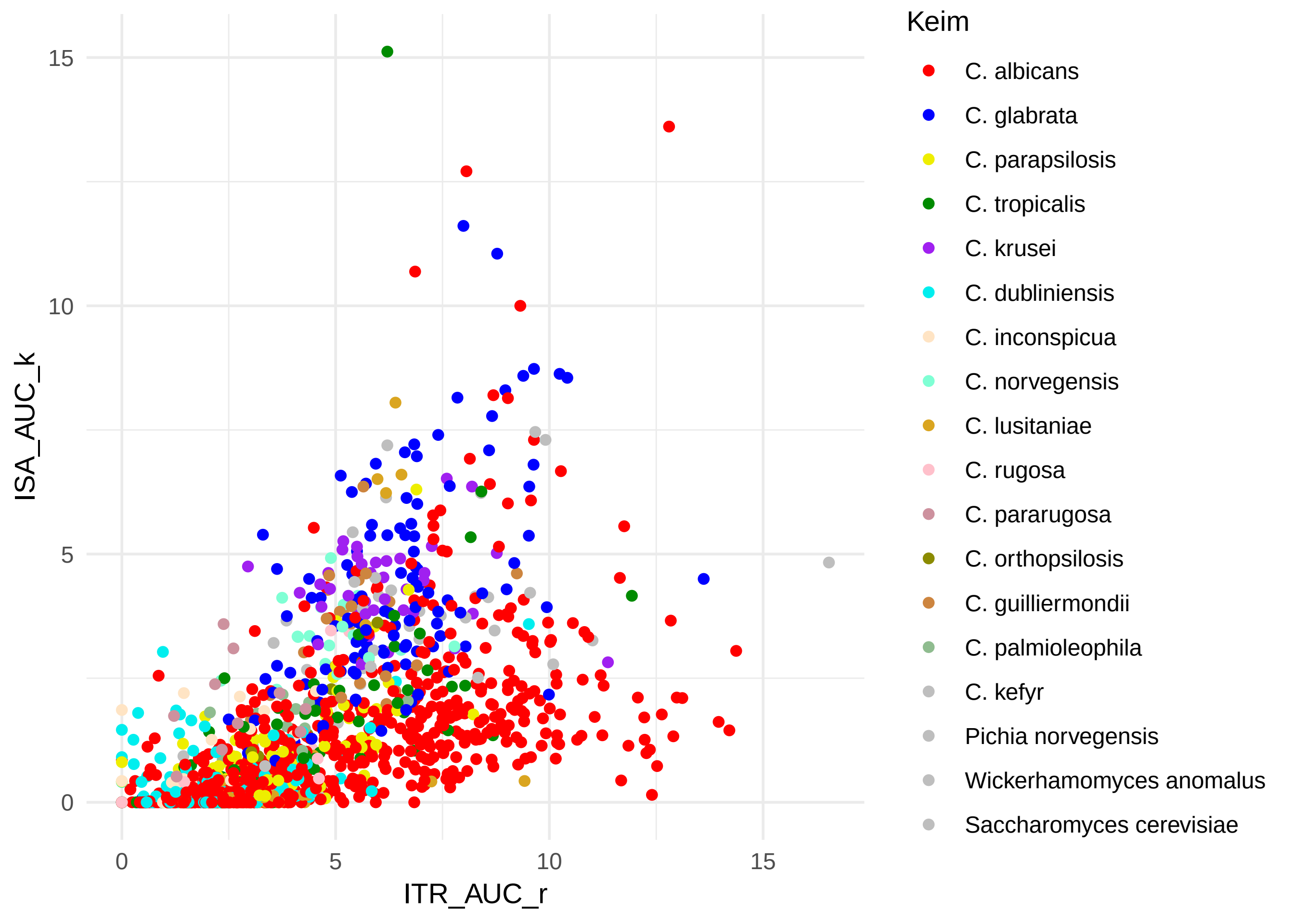
Keim
C. albicans
C. glabrata
C. parapsilosis
C. tropicalis
C. krusei
C. dubliniensis
C. inconspicua
C. norvegensis
C. lusitaniae
C. rugosa
C. pararugosa
C. orthopsilosis
C. guilliermondii
C. palmioleophila
C. kefyr
Pichia norvegensis
Wickerhamomyces anomalus
Saccharomyces cerevisiae
ISA_AUC_k
ITR_AUC_r
0
5
10
15
0
5
10
15

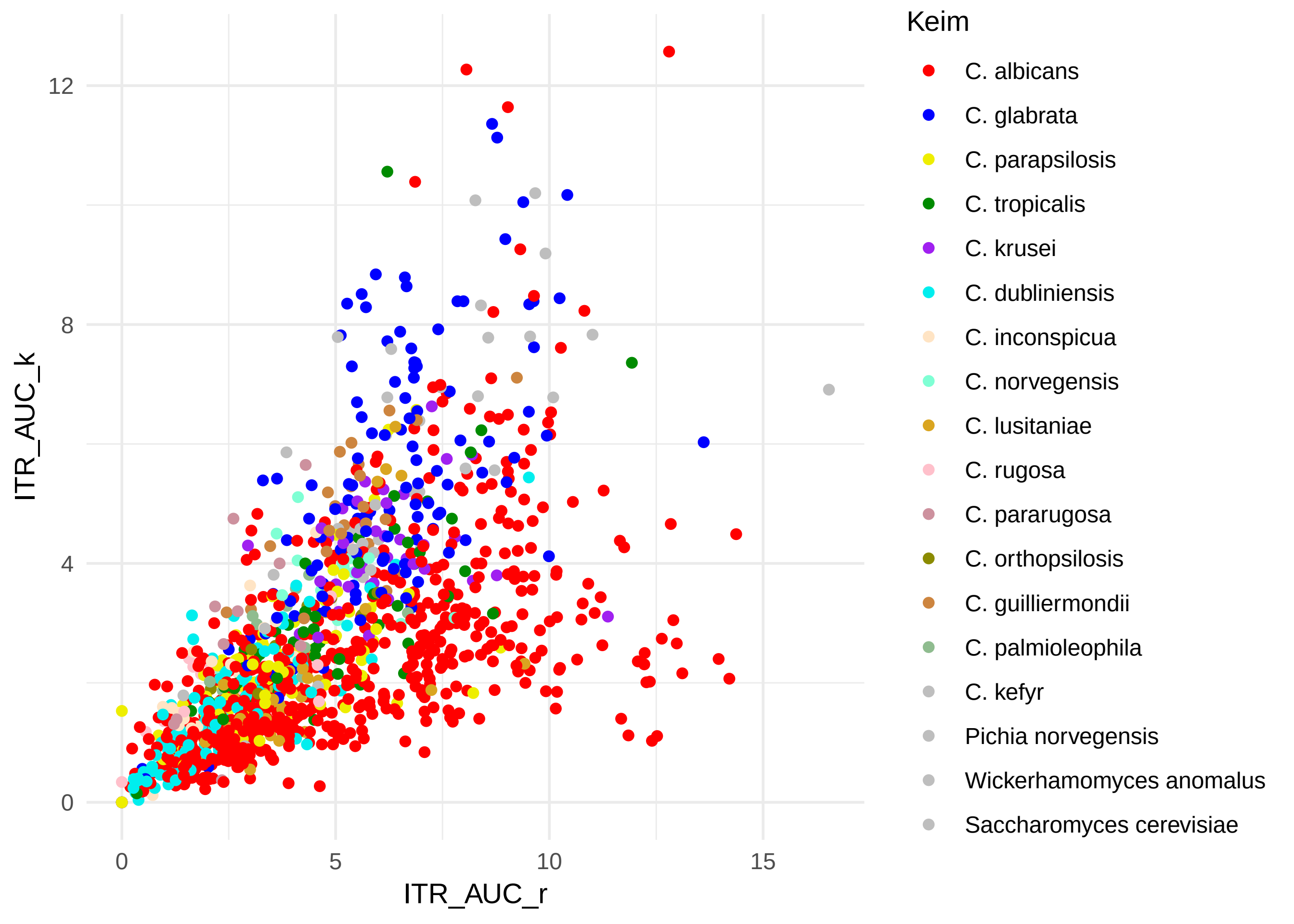

Keim
C. albicans
C. glabrata
C. parapsilosis
C. tropicalis
C. krusei
C. dubliniensis
C. inconspicua
C. norvegensis
C. lusitaniae
C. rugosa
C. pararugosa
C. orthopsilosis
C. guilliermondii
C. palmioleophila
C. kefyr
Pichia norvegensis
Wickerhamomyces anomalus
Saccharomyces cerevisiae
ITR_AUC_k
ITR_AUC_r
0
4
8
12
0
5
10
15

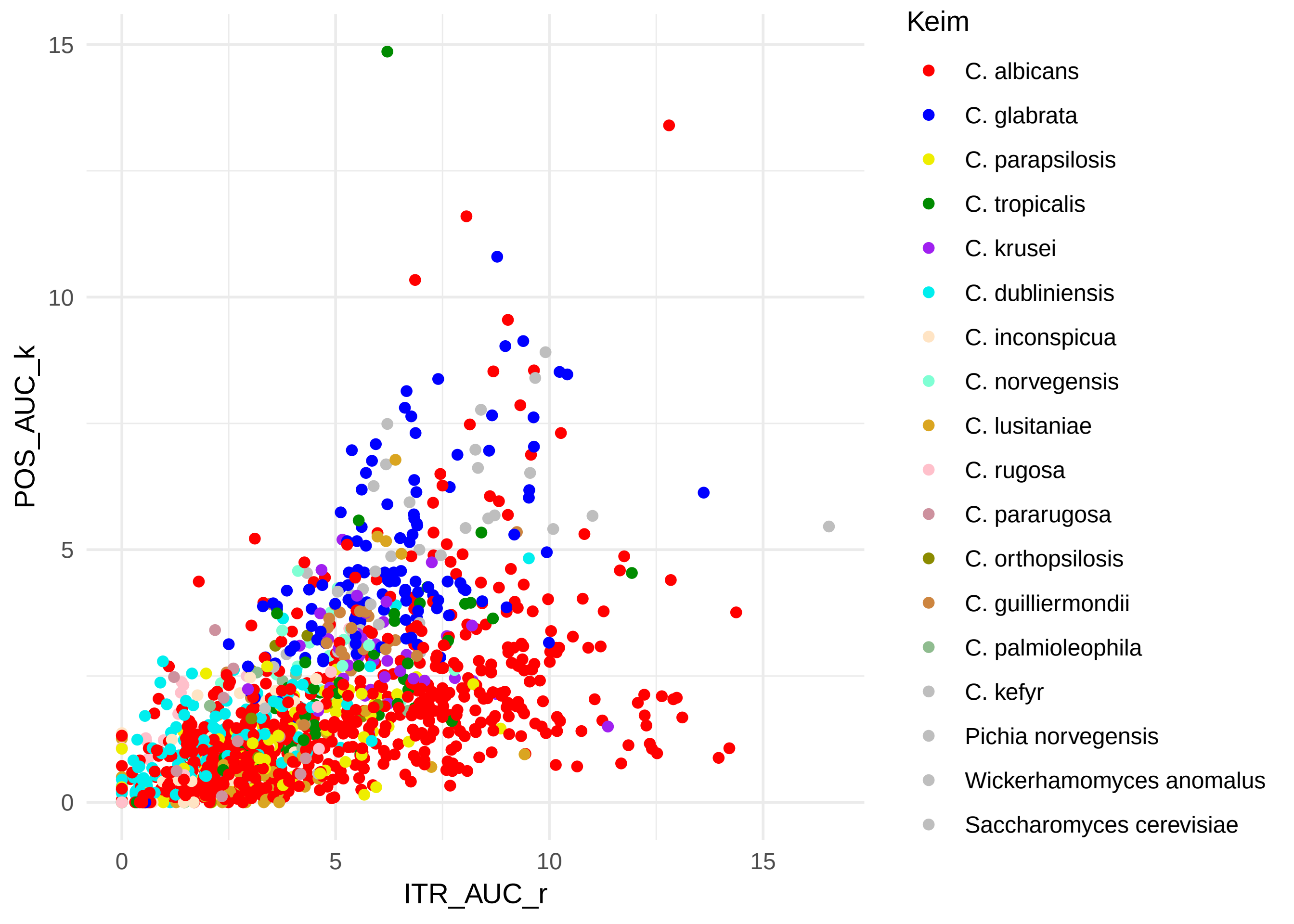

POS_AUC_k
ITR_AUC_r
0
5
10
15
Keim
C. albicans
C. glabrata
C. parapsilosis
C. tropicalis
C. krusei
C. dubliniensis
C. inconspicua
C. norvegensis
C. lusitaniae
C. rugosa
C. pararugosa
C. orthopsilosis
C. guilliermondii
C. palmioleophila
C. kefyr
Pichia norvegensis
Wickerhamomyces anomalus
Saccharomyces cerevisiae

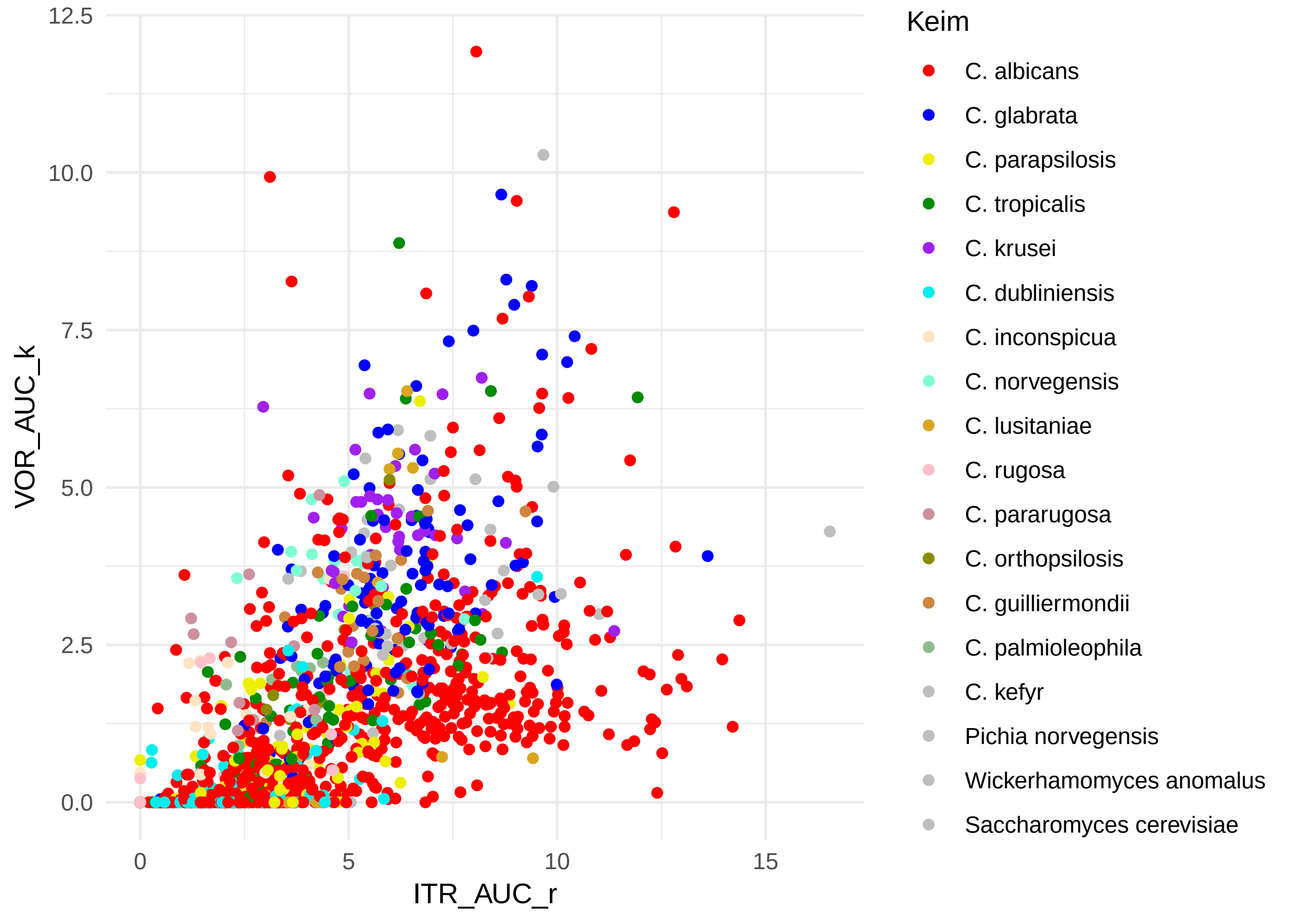

Keim
C. albicans
C. glabrata
C. parapsilosis
C. tropicalis
C. krusei
C. dubliniensis
C. inconspicua
C. norvegensis
C. lusitaniae
C. rugosa
C. pararugosa
C. orthopsilosis
C. guilliermondii
C. palmioleophila
C. kefyr
Pichia norvegensis
Wickerhamomyces anomalus
Saccharomyces cerevisiae
VOR_AUC_k
ITR_AUC_r
0.0
2.5
5.0
7.5
10.0
12.5
0
5
10
15

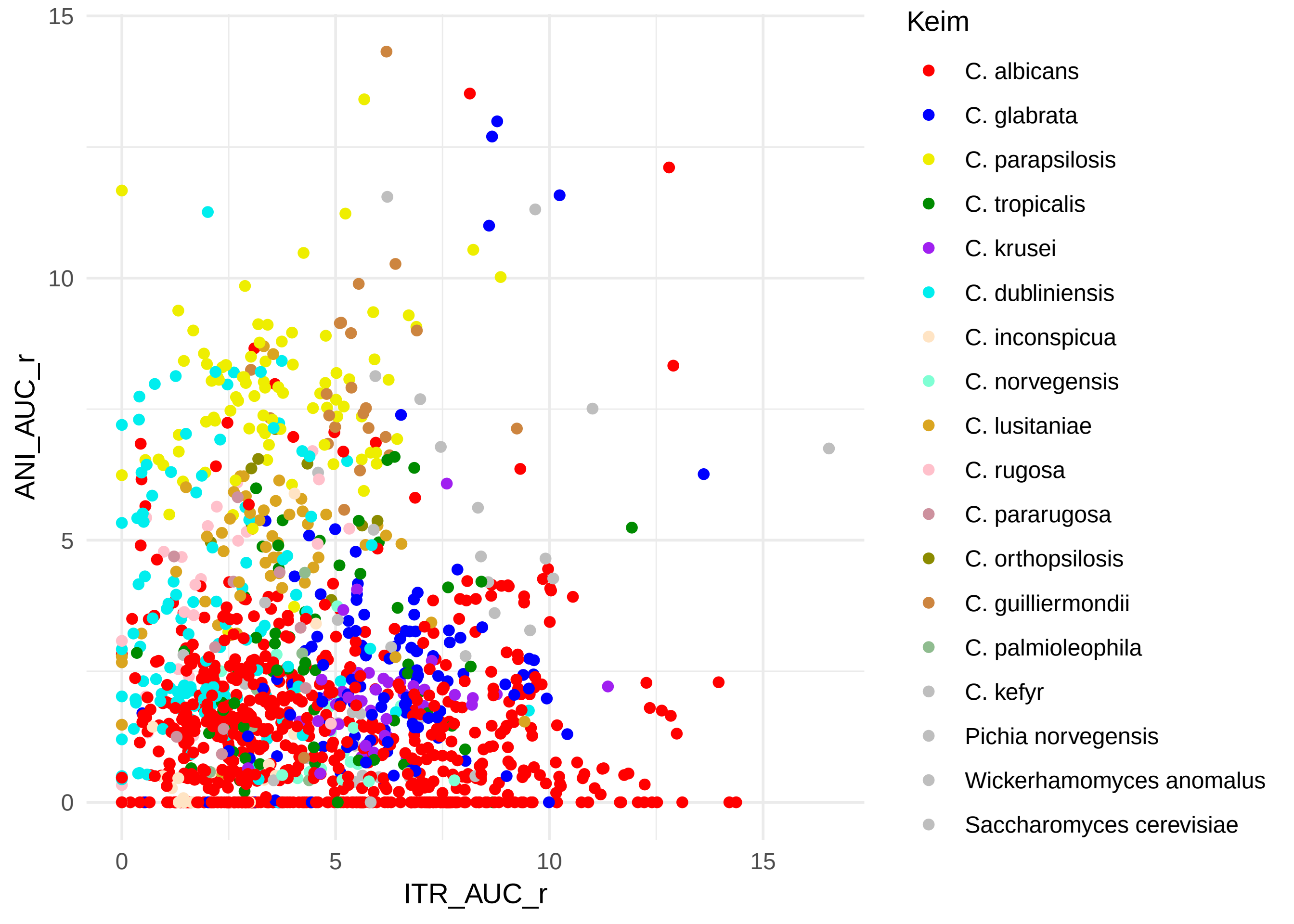

15
10
5
0
ANI_AUC_r
0
5
10
15
ITR_AUC_r
Keim
C. albicans
C. glabrata
C. parapsilosis
C. tropicalis
C. krusei
C. dubliniensis
C. inconspicua
C. norvegensis
C. lusitaniae
C. rugosa
C. pararugosa
C. orthopsilosis
C. guilliermondii
C. palmioleophila
C. kefyr
Pichia norvegensis
Wickerhamomyces anomalus
Saccharomyces cerevisiae

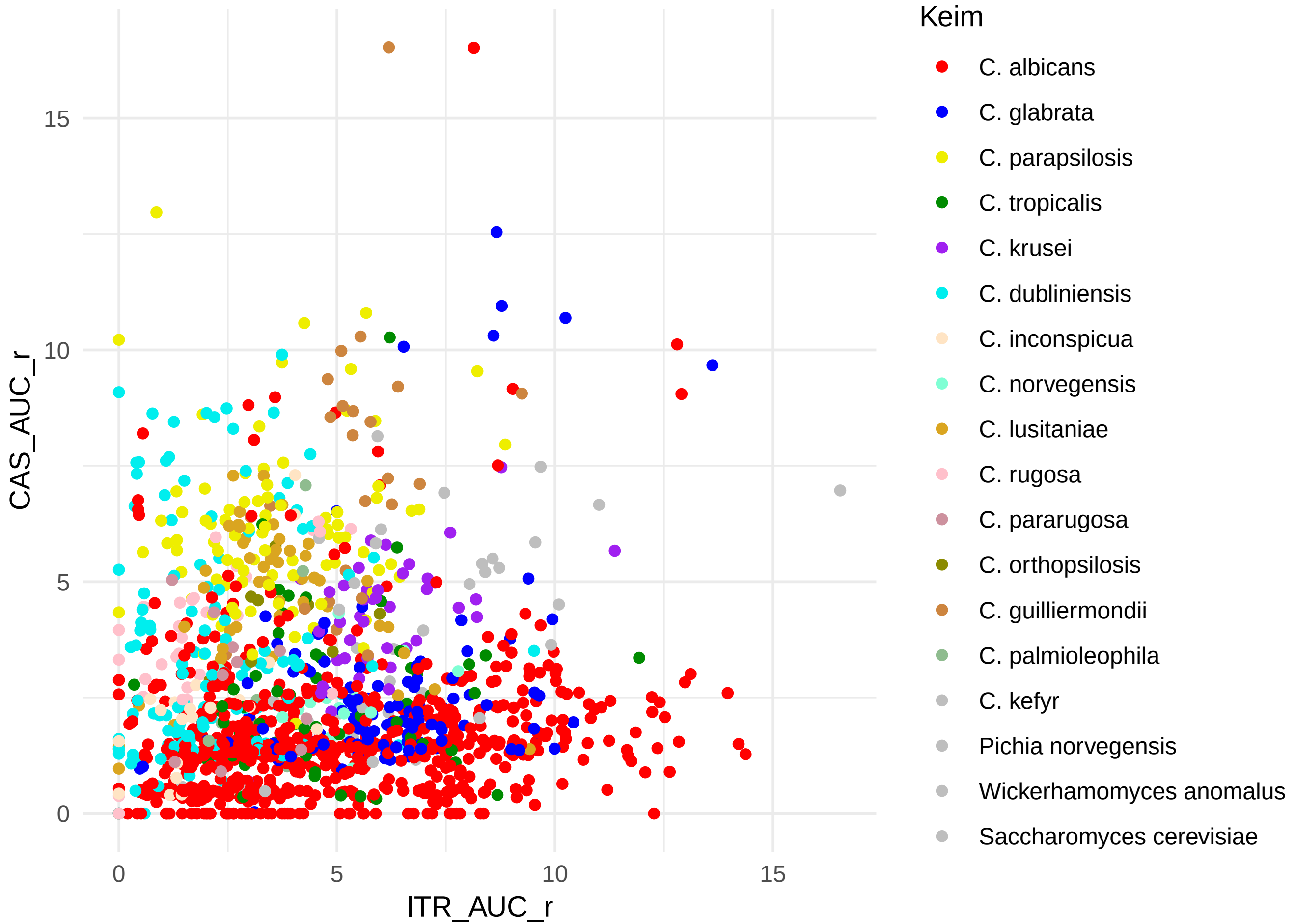
Keim
C. albicans
C. glabrata
C. parapsilosis
C. tropicalis
C. krusei
C. dubliniensis
C. inconspicua
C. norvegensis
C. lusitaniae
C. rugosa
C. pararugosa
C. orthopsilosis
C. guilliermondii
C. palmioleophila
C. kefyr
Pichia norvegensis
Wickerhamomyces anomalus
Saccharomyces cerevisiae
CAS_AUC_r
ITR_AUC_r
0
5
10
15
0
5
10
15

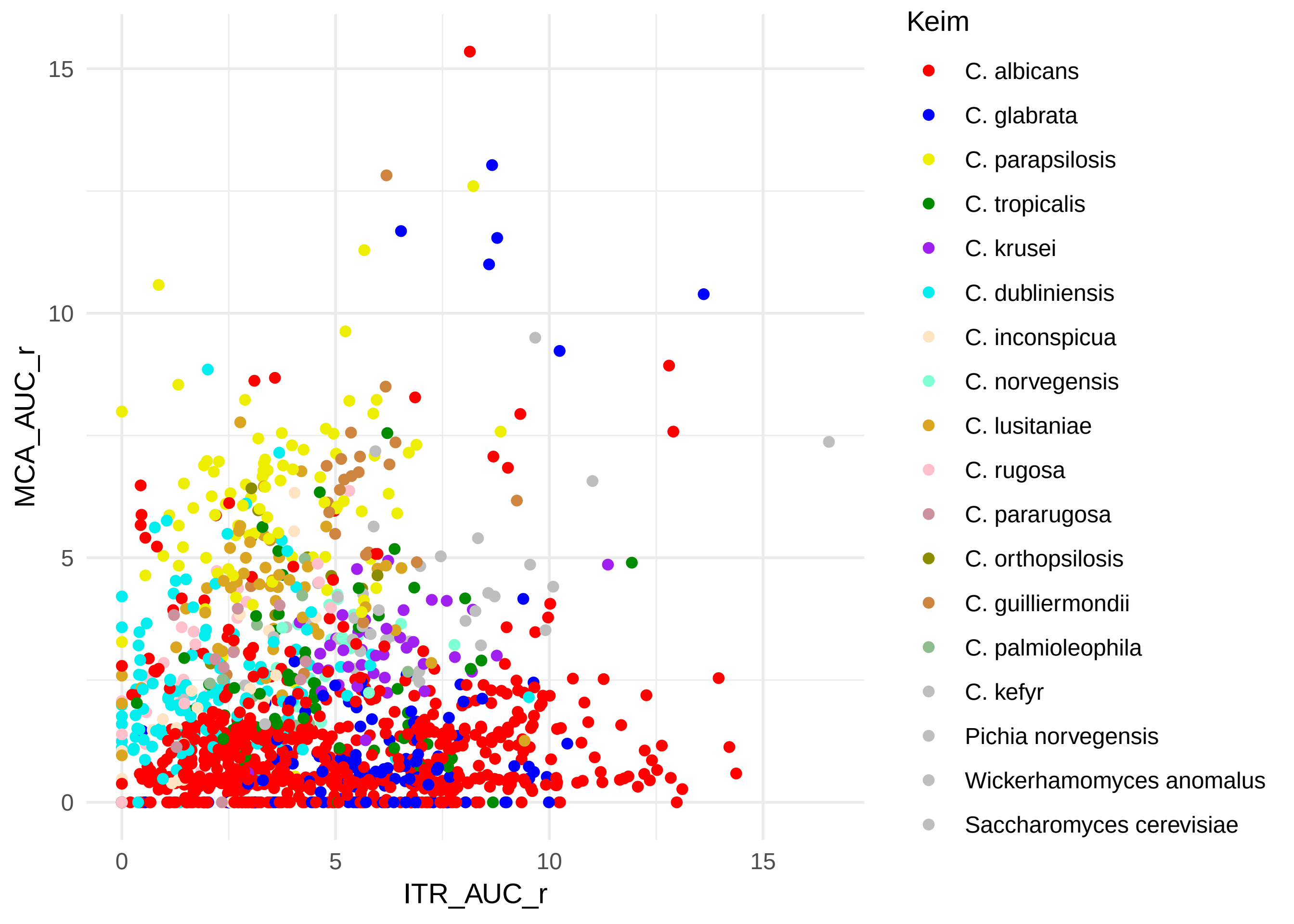

Keim
C. albicans
C. glabrata
C. parapsilosis
C. tropicalis
C. krusei
C. dubliniensis
C. inconspicua
C. norvegensis
C. lusitaniae
C. rugosa
C. pararugosa
C. orthopsilosis
C. guilliermondii
C. palmioleophila
C. kefyr
Pichia norvegensis
Wickerhamomyces anomalus
Saccharomyces cerevisiae
MCA_AUC_r
ITR_AUC_r
0
5
10
15

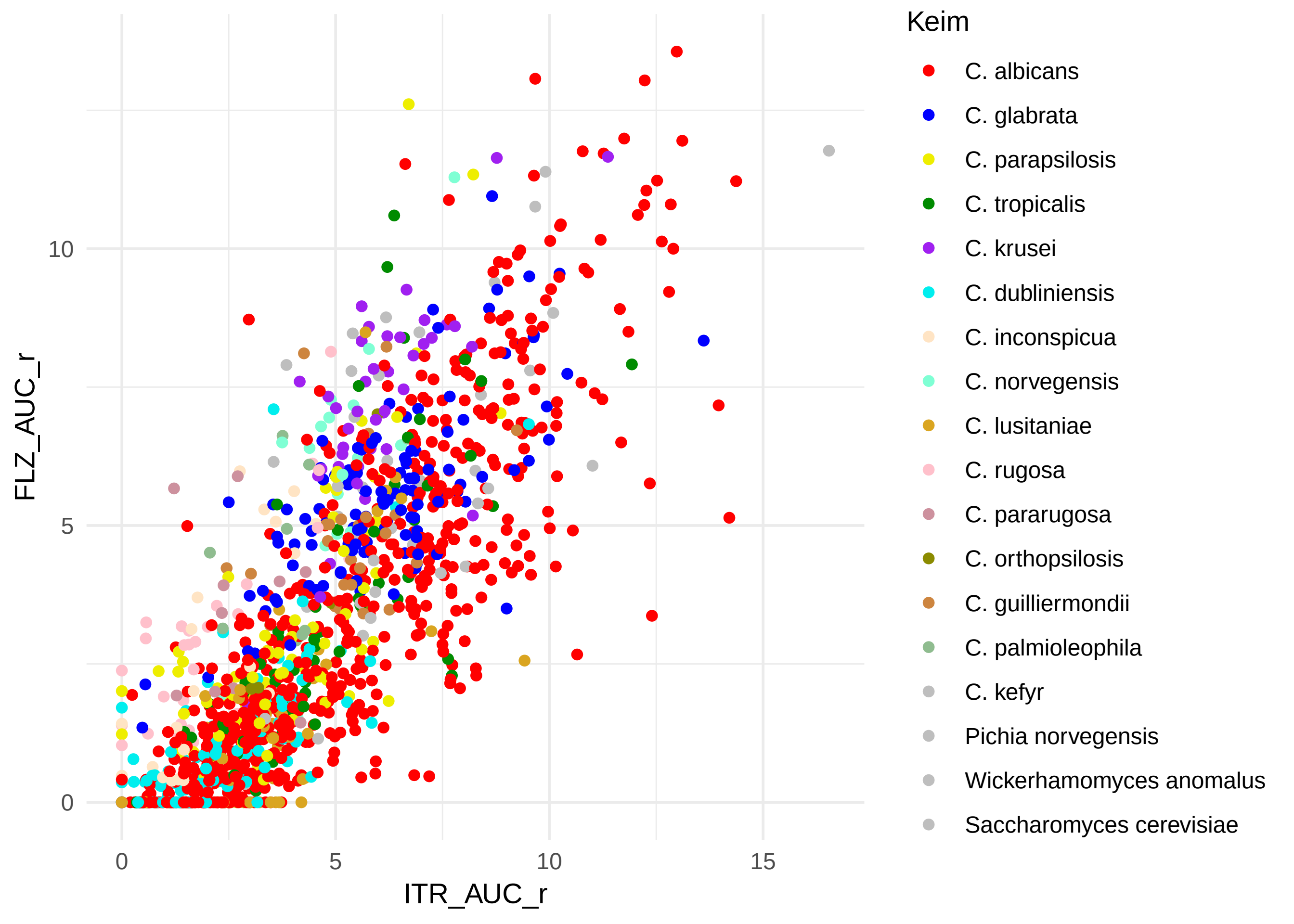
Keim
C. albicans
C. glabrata
C. parapsilosis
C. tropicalis
C. krusei
C. dubliniensis
C. inconspicua
C. norvegensis
C. lusitaniae
C. rugosa
C. pararugosa
C. orthopsilosis
C. guilliermondii
C. palmioleophila
C. kefyr
Pichia norvegensis
Wickerhamomyces anomalus
Saccharomyces cerevisiae
FLZ_AUC_r
10
5
0
0
5
10
15
ITR_AUC_r

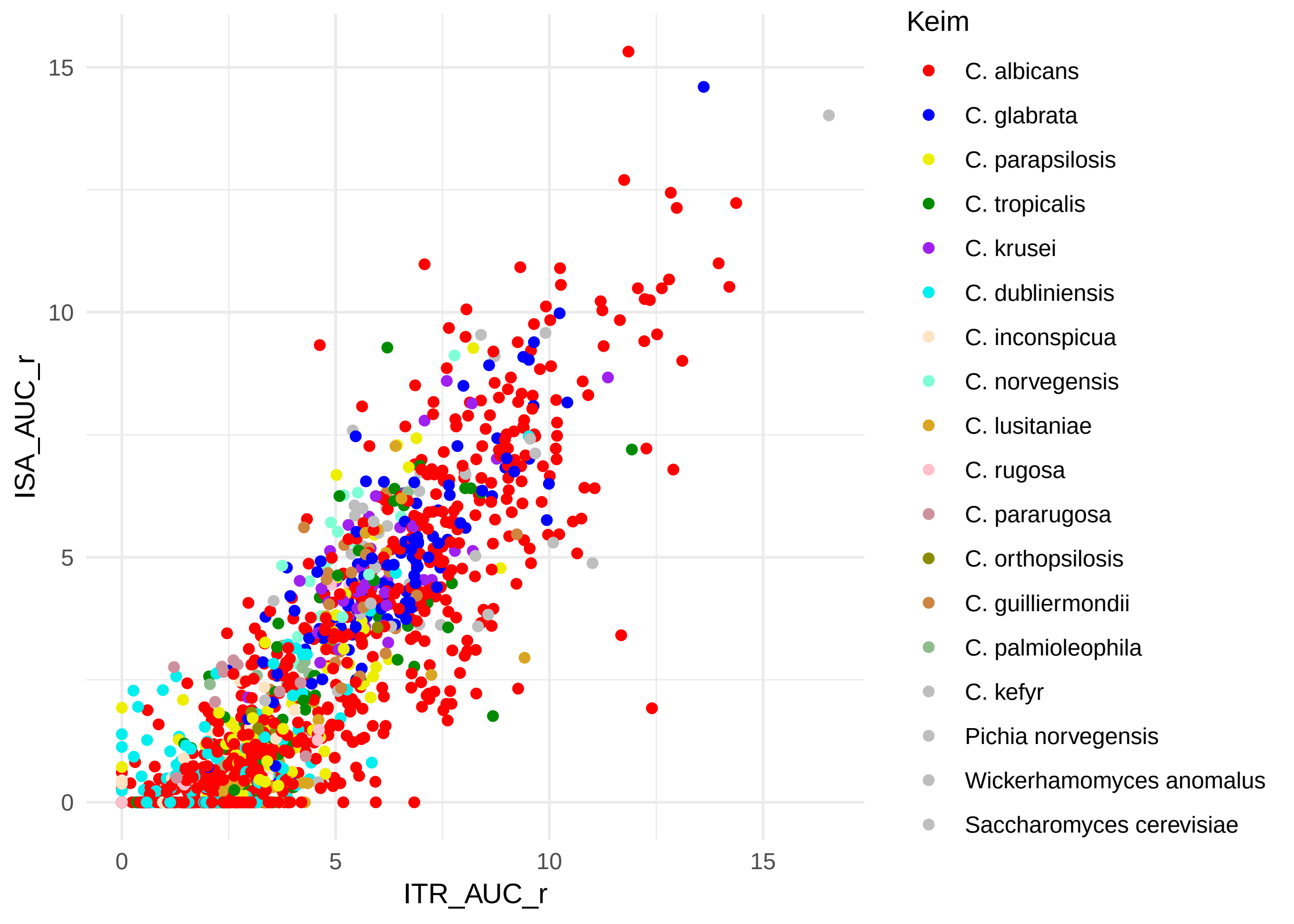
Keim
C. albicans
C. glabrata
C. parapsilosis
C. tropicalis
C. krusei
C. dubliniensis
C. inconspicua
C. norvegensis
C. lusitaniae
C. rugosa
C. pararugosa
C. orthopsilosis
C. guilliermondii
C. palmioleophila
C. kefyr
Pichia norvegensis
Wickerhamomyces anomalus
Saccharomyces cerevisiae
ISA_AUC_r
ITR_AUC_r
0
5
10
15
0
5
10
15

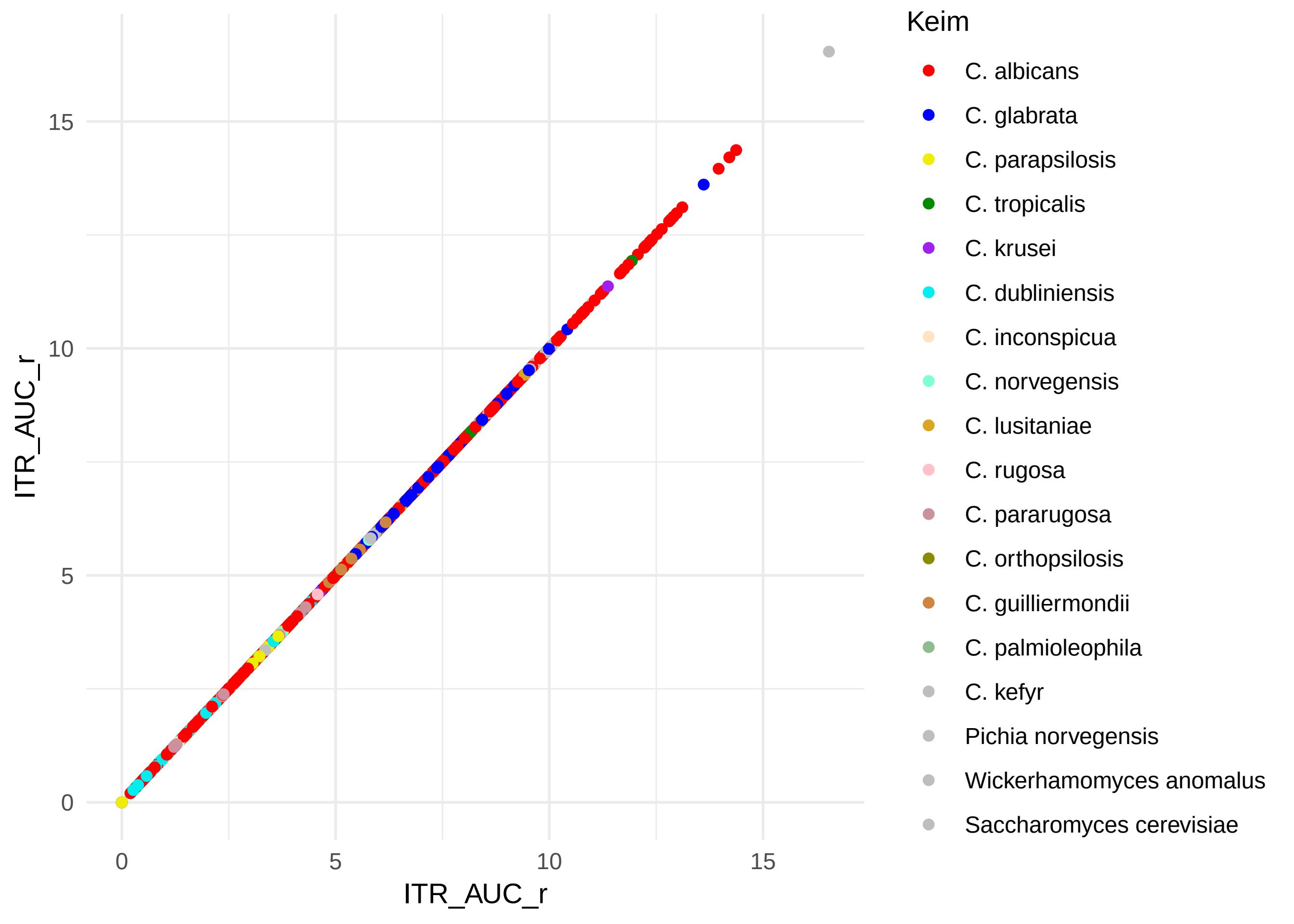
Keim
C. albicans
C. glabrata
C. parapsilosis
C. tropicalis
C. krusei
C. dubliniensis
C. inconspicua
C. norvegensis
C. lusitaniae
C. rugosa
C. pararugosa
C. orthopsilosis
C. guilliermondii
C. palmioleophila
C. kefyr
Pichia norvegensis
Wickerhamomyces anomalus
Saccharomyces cerevisiae
ITR_AUC_r
ITR_AUC_r
0
5
10
15
0
5
10
15

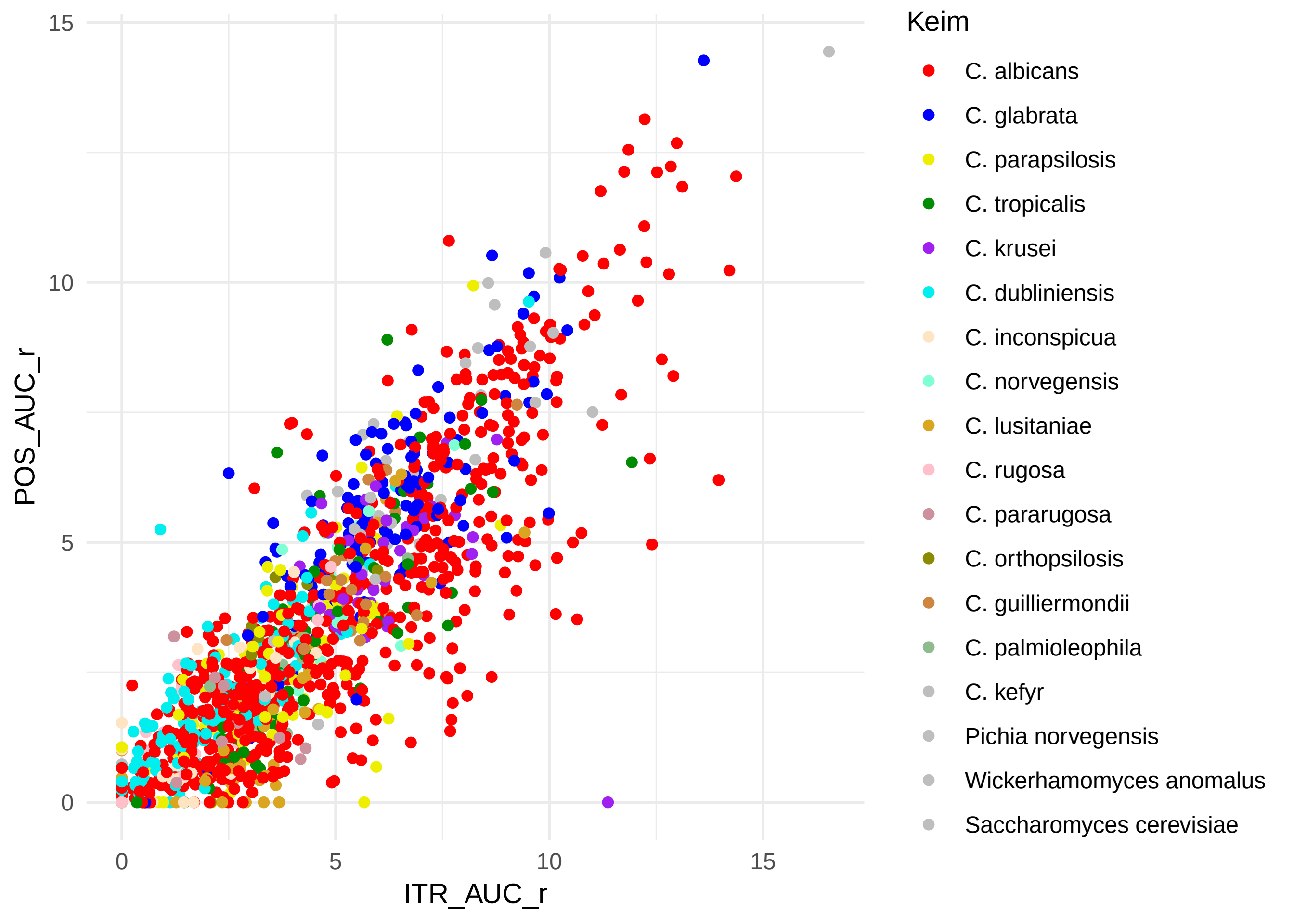
Keim
C. albicans
C. glabrata
C. parapsilosis
C. tropicalis
C. krusei
C. dubliniensis
C. inconspicua
C. norvegensis
C. lusitaniae
C. rugosa
C. pararugosa
C. orthopsilosis
C. guilliermondii
C. palmioleophila
C. kefyr
Pichia norvegensis
Wickerhamomyces anomalus
Saccharomyces cerevisiae
POS_AUC_r
ITR_AUC_r
0
5
10
15
0
5
10
15

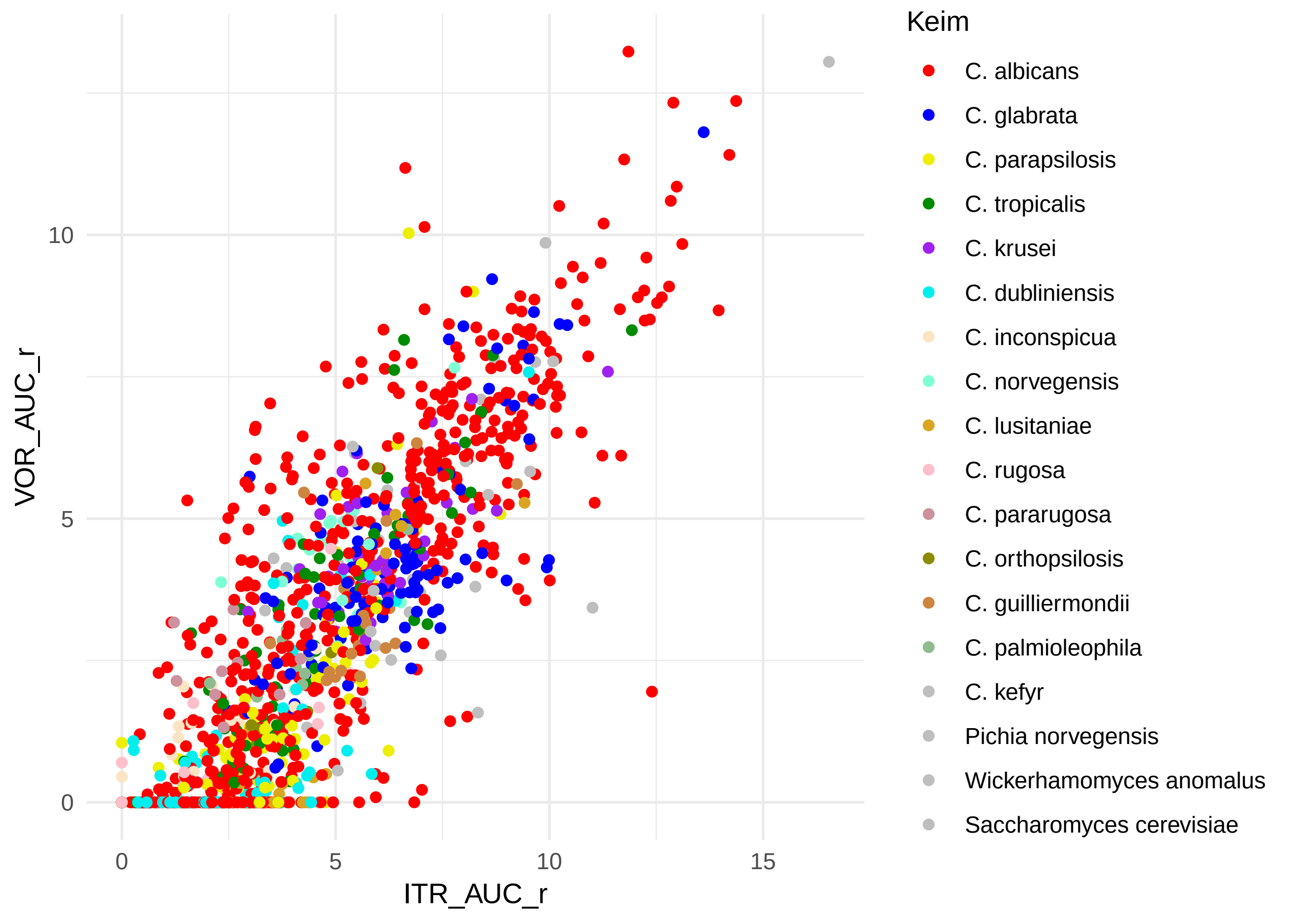
Keim
C. albicans
C. glabrata
C. parapsilosis
C. tropicalis
C. krusei
C. dubliniensis
C. inconspicua
C. norvegensis
C. lusitaniae
C. rugosa
C. pararugosa
C. orthopsilosis
C. guilliermondii
C. palmioleophila
C. kefyr
Pichia norvegensis
Wickerhamomyces anomalus
Saccharomyces cerevisiae
VOR_AUC_r
ITR_AUC_r
0
5
10
15

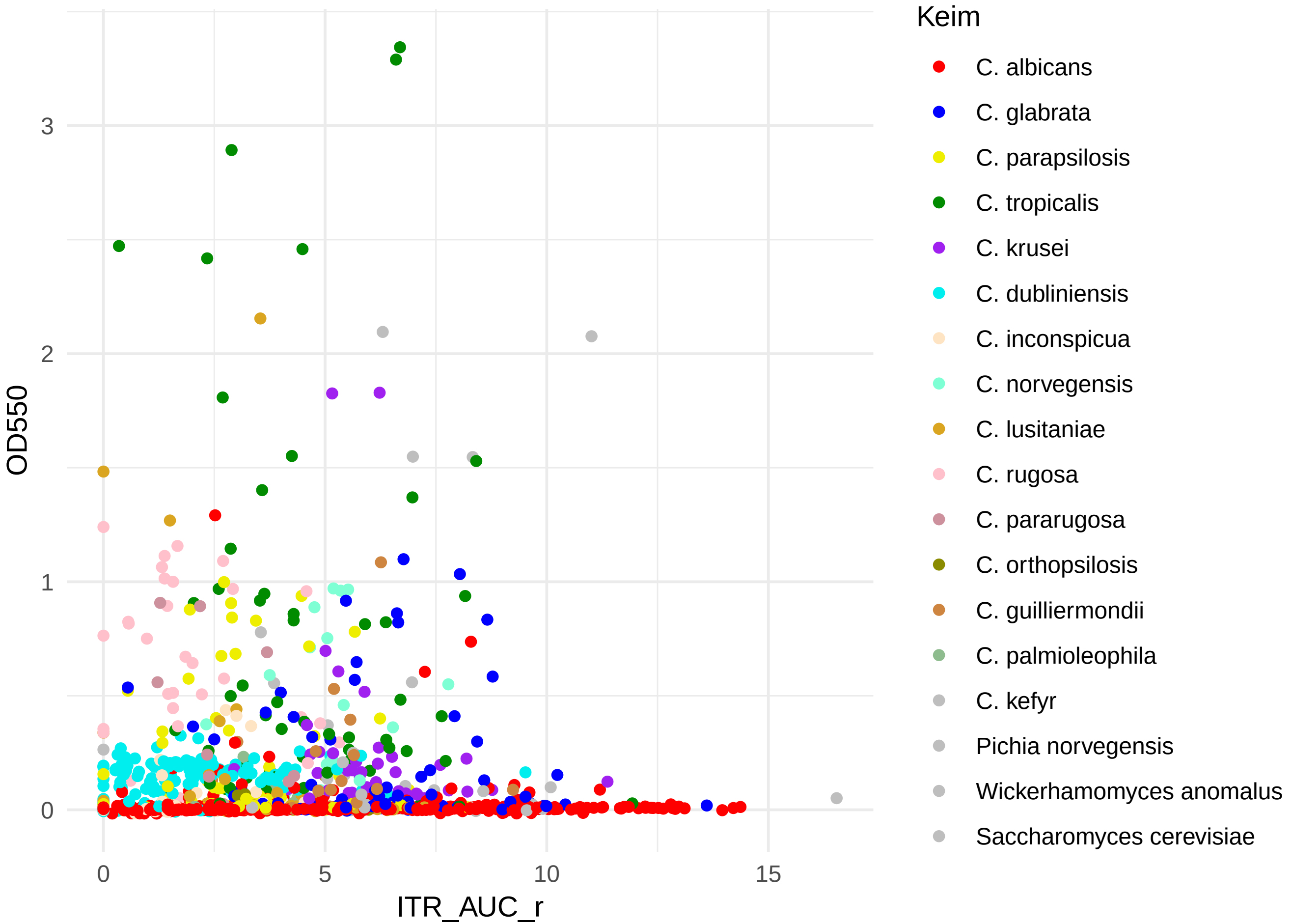
Keim
C. albicans
C. glabrata
C. parapsilosis
C. tropicalis
C. krusei
C. dubliniensis
C. inconspicua
C. norvegensis
C. lusitaniae
C. rugosa
C. pararugosa
C. orthopsilosis
C. guilliermondii
C. palmioleophila
C. kefyr
Pichia norvegensis
Wickerhamomyces anomalus
Saccharomyces cerevisiae
OD550
ITR_AUC_r
0
1
2
3
0
5
10
15

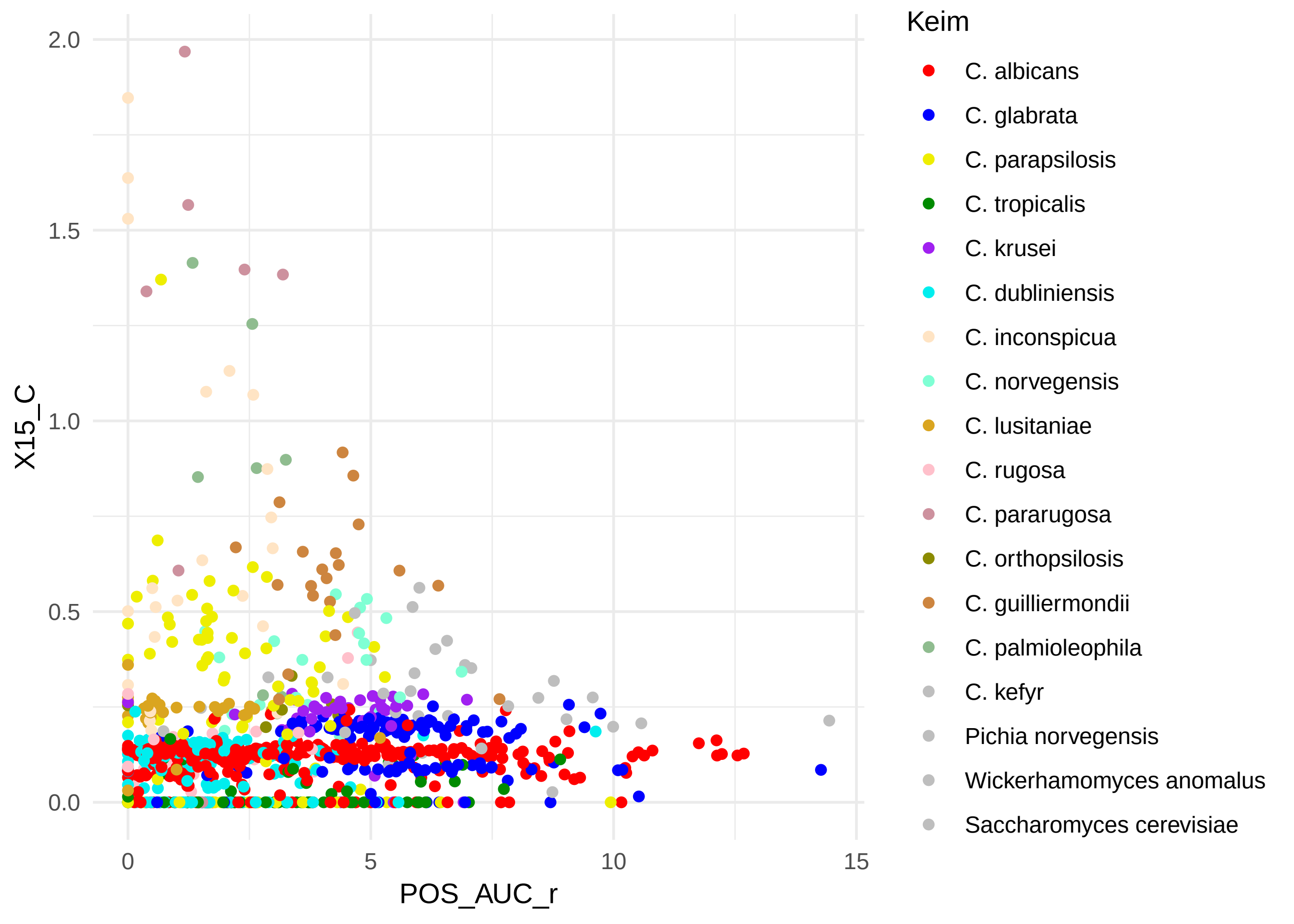

Keim
C. albicans
C. glabrata
C. parapsilosis
C. tropicalis
C. krusei
C. dubliniensis
C. inconspicua
C. norvegensis
C. lusitaniae
C. rugosa
C. pararugosa
C. orthopsilosis
C. guilliermondii
C. palmioleophila
C. kefyr
Pichia norvegensis
Wickerhamomyces anomalus
Saccharomyces cerevisiae
X15_C
2.0
1.5
1.0
0.5
0.0
0
5
10
15
POS_AUC_r

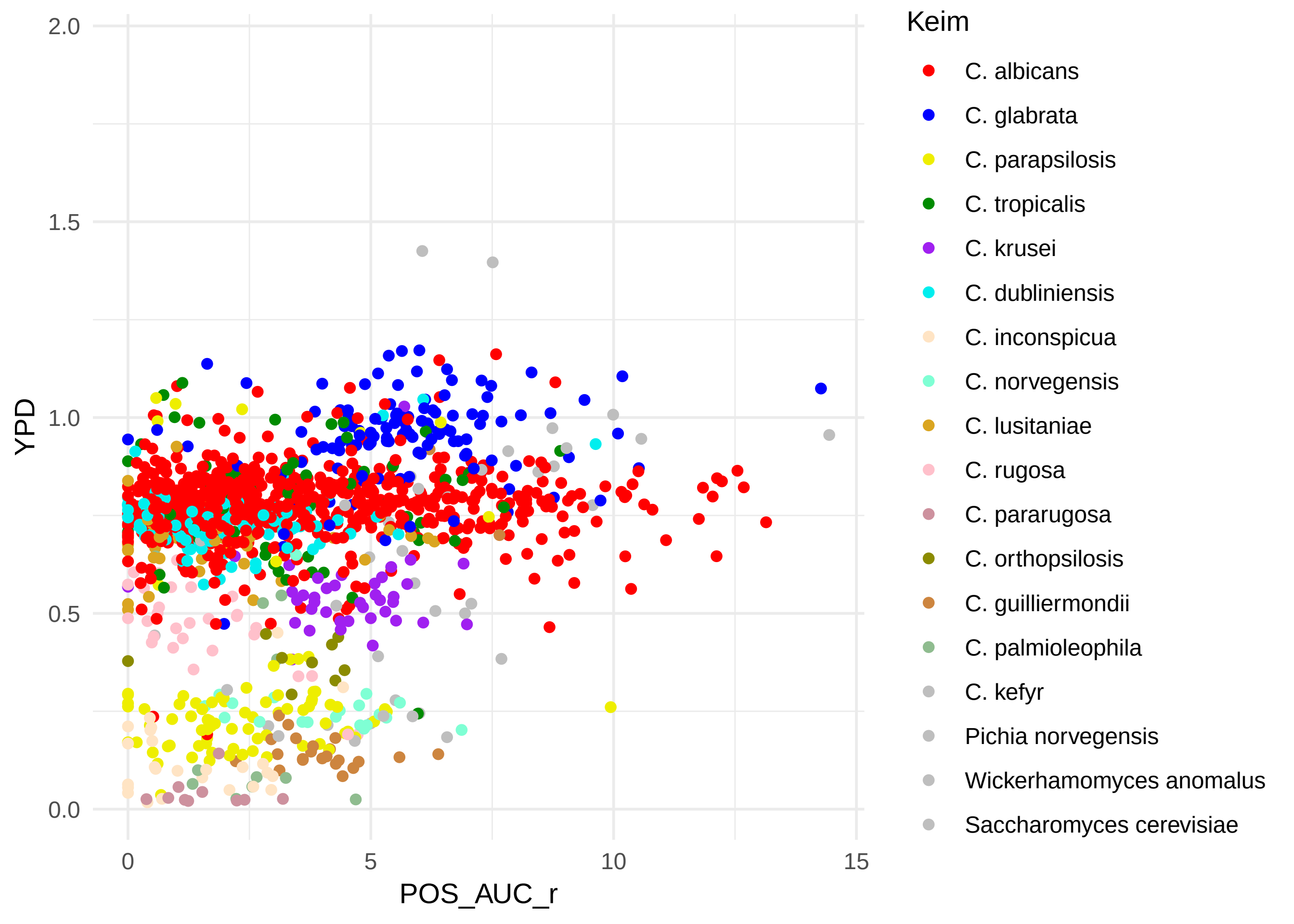

Keim
C. albicans
C. glabrata
C. parapsilosis
C. tropicalis
C. krusei
C. dubliniensis
C. inconspicua
C. norvegensis
C. lusitaniae
C. rugosa
C. pararugosa
C. orthopsilosis
C. guilliermondii
C. palmioleophila
C. kefyr
Pichia norvegensis
Wickerhamomyces anomalus
Saccharomyces cerevisiae
YPD
2.0
1.5
1.0
0.5
0.0
0
5
10
15
POS_AUC_r

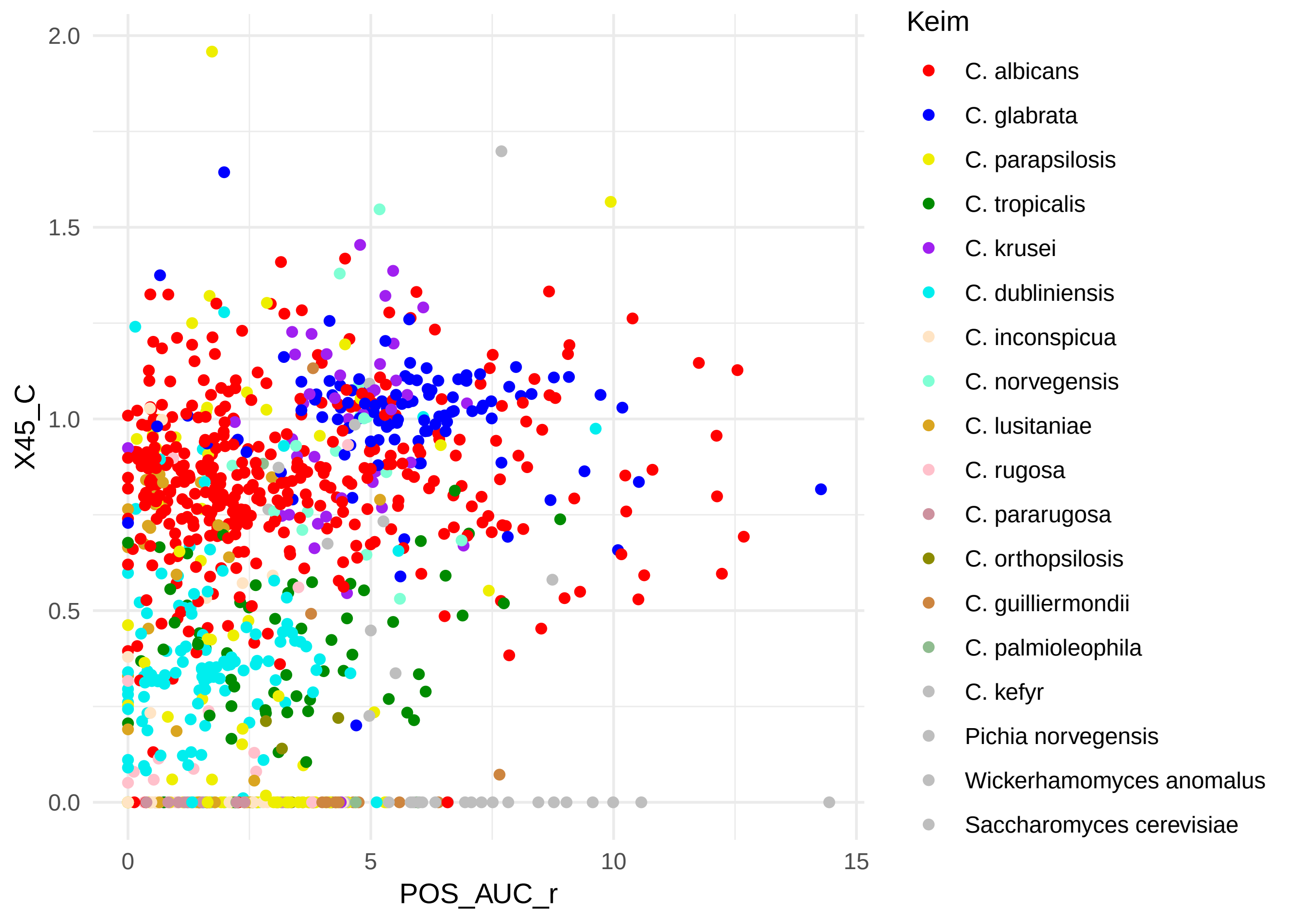

2.0
1.5
1.0
0.5
0.0
X45_C
0
5
10
15
POS_AUC_r
Keim
C. albicans
C. glabrata
C. parapsilosis
C. tropicalis
C. krusei
C. dubliniensis
C. inconspicua
C. norvegensis
C. lusitaniae
C. rugosa
C. pararugosa
C. orthopsilosis
C. guilliermondii
C. palmioleophila
C. kefyr
Pichia norvegensis
Wickerhamomyces anomalus
Saccharomyces cerevisiae

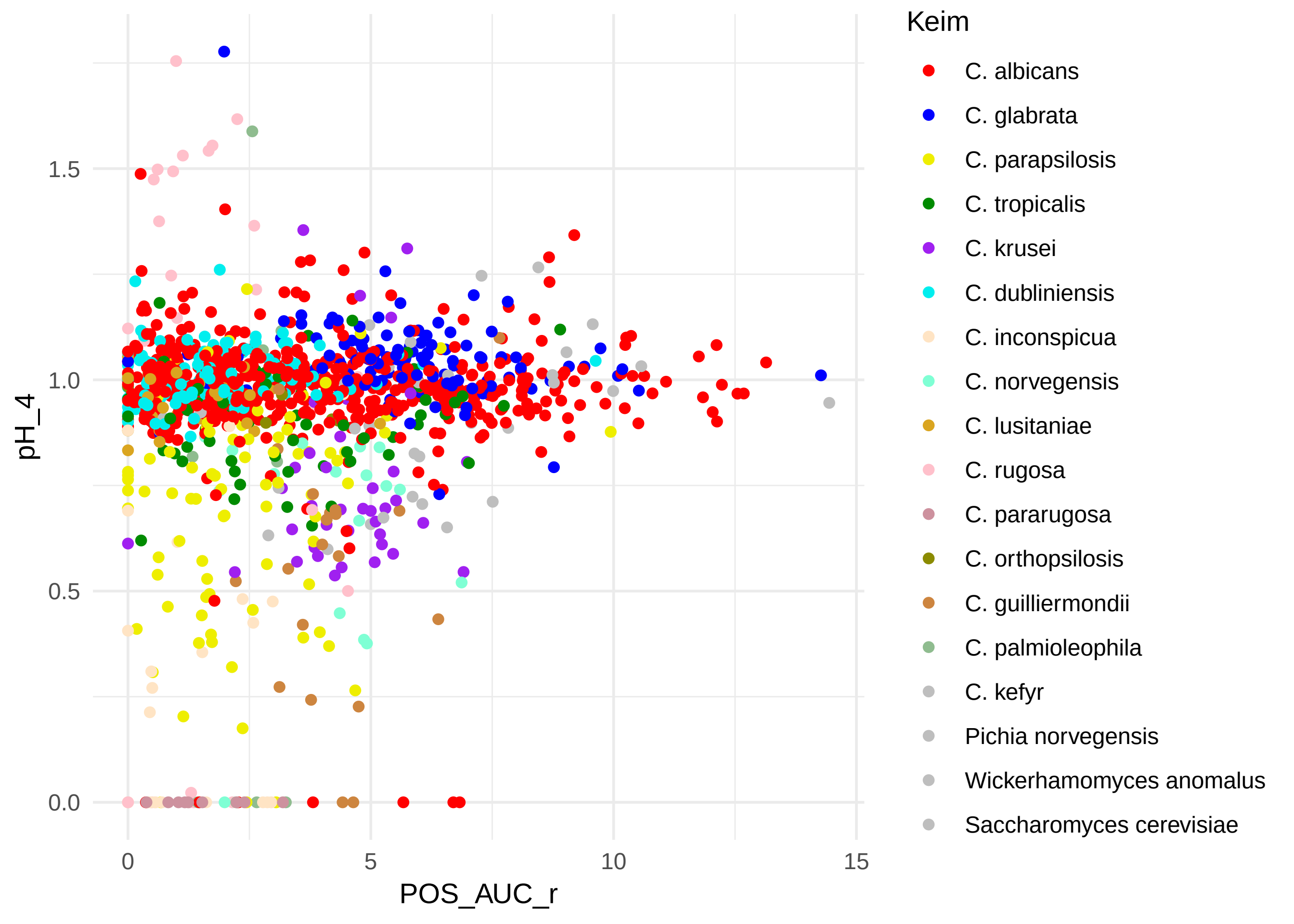

Keim
C. albicans
C. glabrata
C. parapsilosis
C. tropicalis
C. krusei
C. dubliniensis
C. inconspicua
C. norvegensis
C. lusitaniae
C. rugosa
C. pararugosa
C. orthopsilosis
C. guilliermondii
C. palmioleophila
C. kefyr
Pichia norvegensis
Wickerhamomyces anomalus
Saccharomyces cerevisiae
pH_4
POS_AUC_r
0.0
0.5
1.0
1.5
0
5
10
15

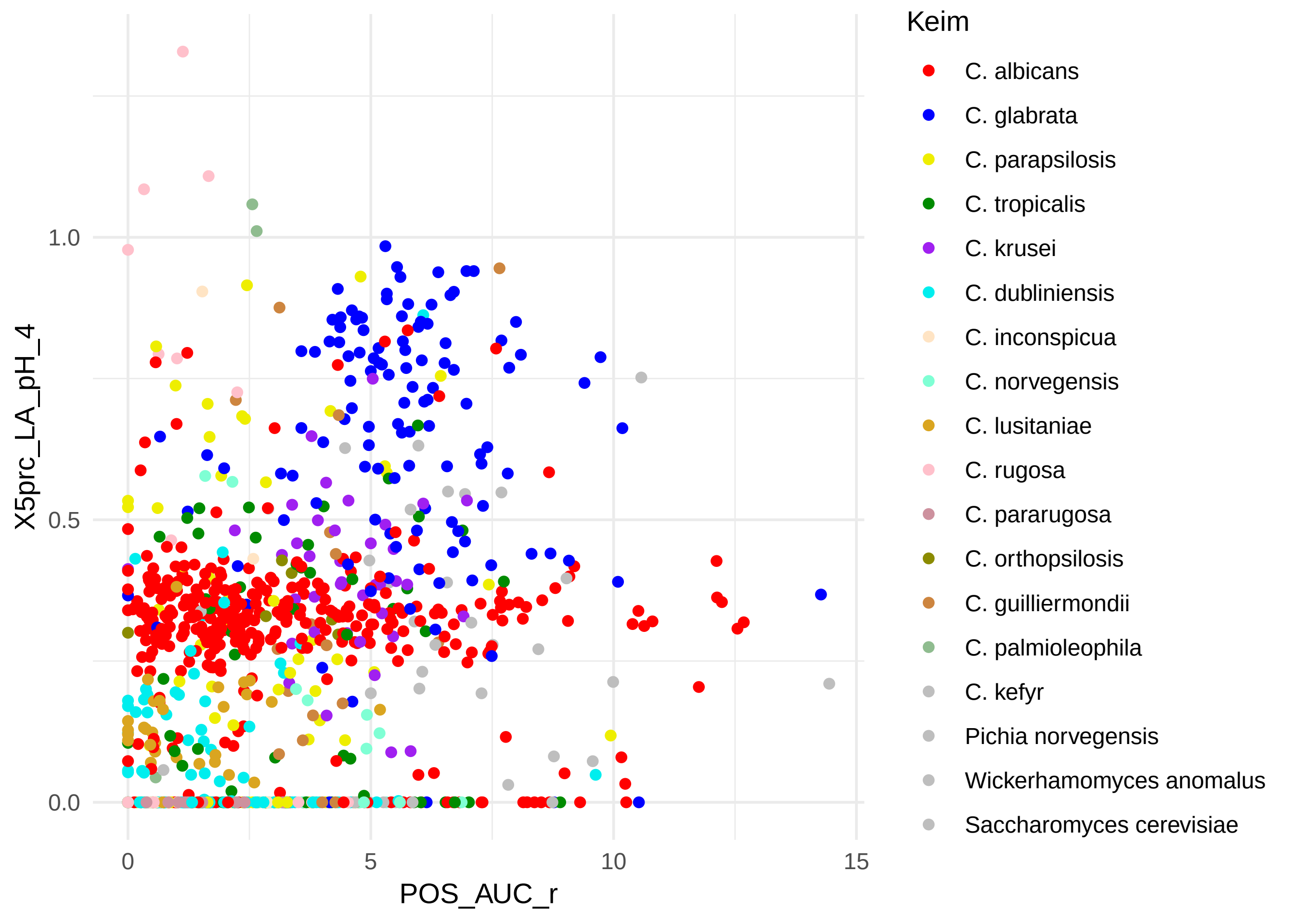
Keim
C. albicans
C. glabrata
C. parapsilosis
C. tropicalis
C. krusei
C. dubliniensis
C. inconspicua
C. norvegensis
C. lusitaniae
C. rugosa
C. pararugosa
C. orthopsilosis
C. guilliermondii
C. palmioleophila
C. kefyr
Pichia norvegensis
Wickerhamomyces anomalus
Saccharomyces cerevisiae
X5prc_LA_pH_4
1.0
0.5
0.0
0
5
10
15
POS_AUC_r

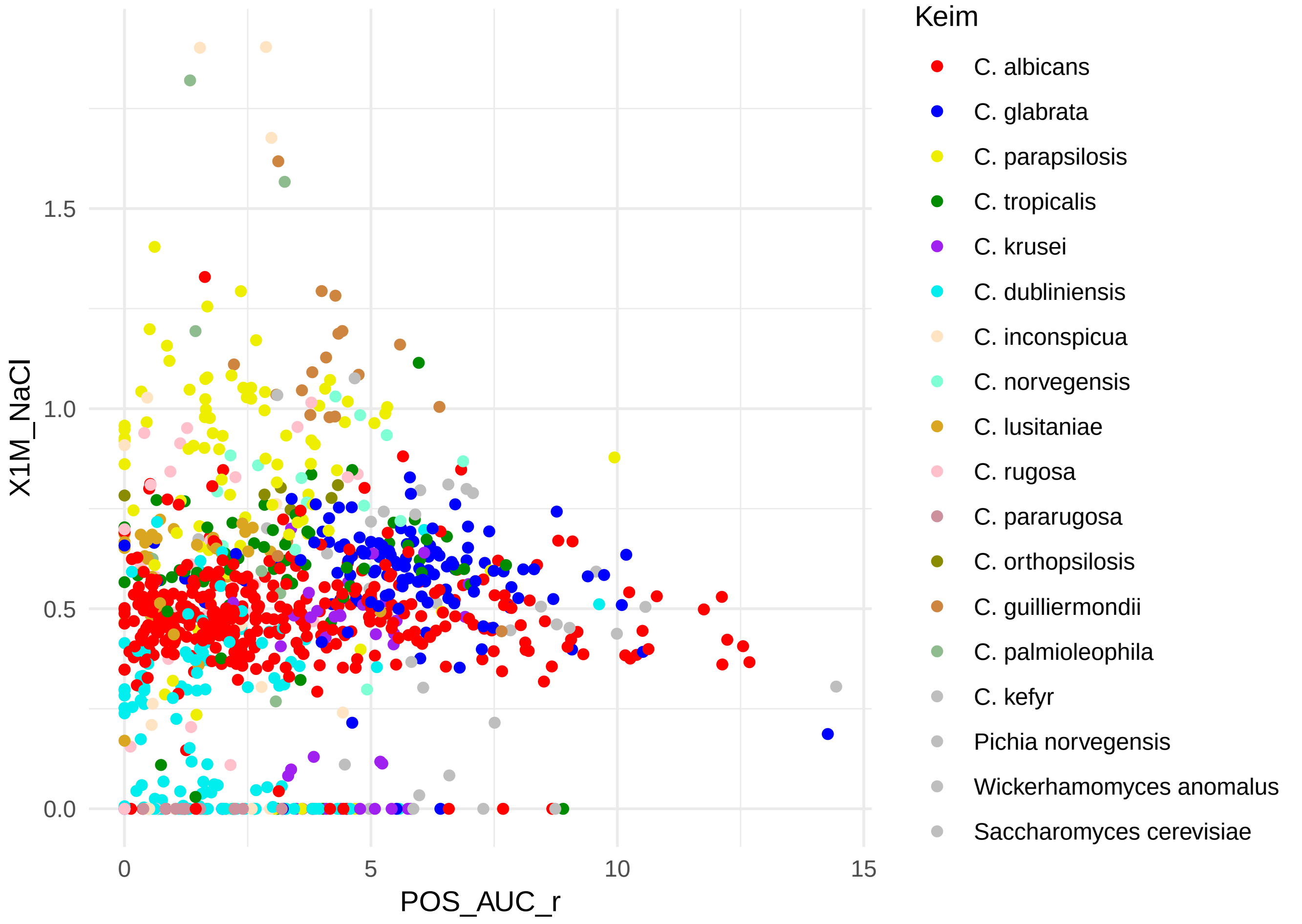
Keim
C. albicans
C. glabrata
C. parapsilosis
C. tropicalis
C. krusei
C. dubliniensis
C. inconspicua
C. norvegensis
C. lusitaniae
C. rugosa
C. pararugosa
C. orthopsilosis
C. guilliermondii
C. palmioleophila
C. kefyr
Pichia norvegensis
Wickerhamomyces anomalus
Saccharomyces cerevisiae
X1M_NaCl
1.5
1.0
0.5
0.0
0
5
10
15
POS_AUC_r

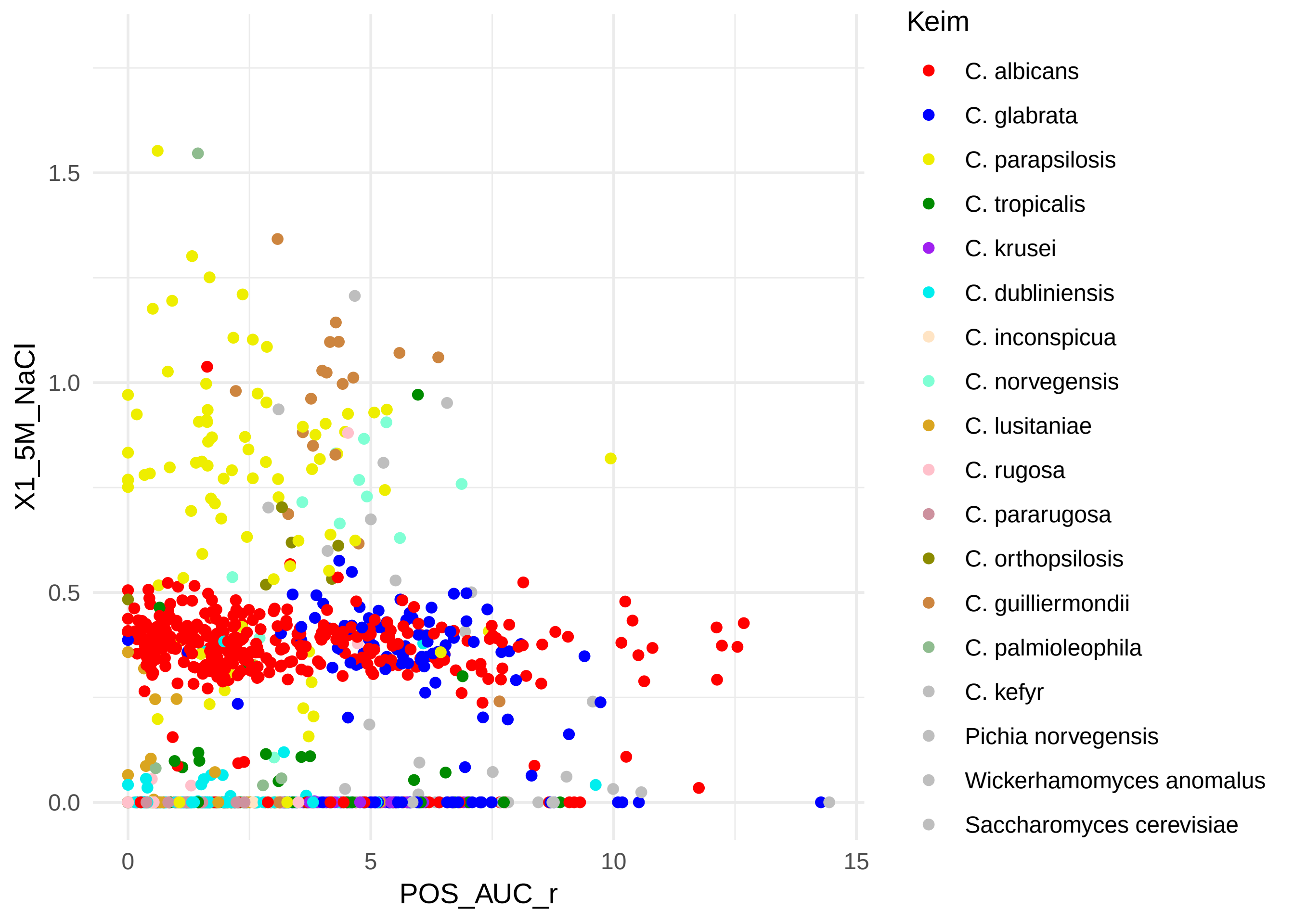

Keim
C. albicans
C. glabrata
C. parapsilosis
C. tropicalis
C. krusei
C. dubliniensis
C. inconspicua
C. norvegensis
C. lusitaniae
C. rugosa
C. pararugosa
C. orthopsilosis
C. guilliermondii
C. palmioleophila
C. kefyr
Pichia norvegensis
Wickerhamomyces anomalus
Saccharomyces cerevisiae
X1_5M_NaCl
1.5
1.0
0.5
0.0
0
5
10
15
POS_AUC_r

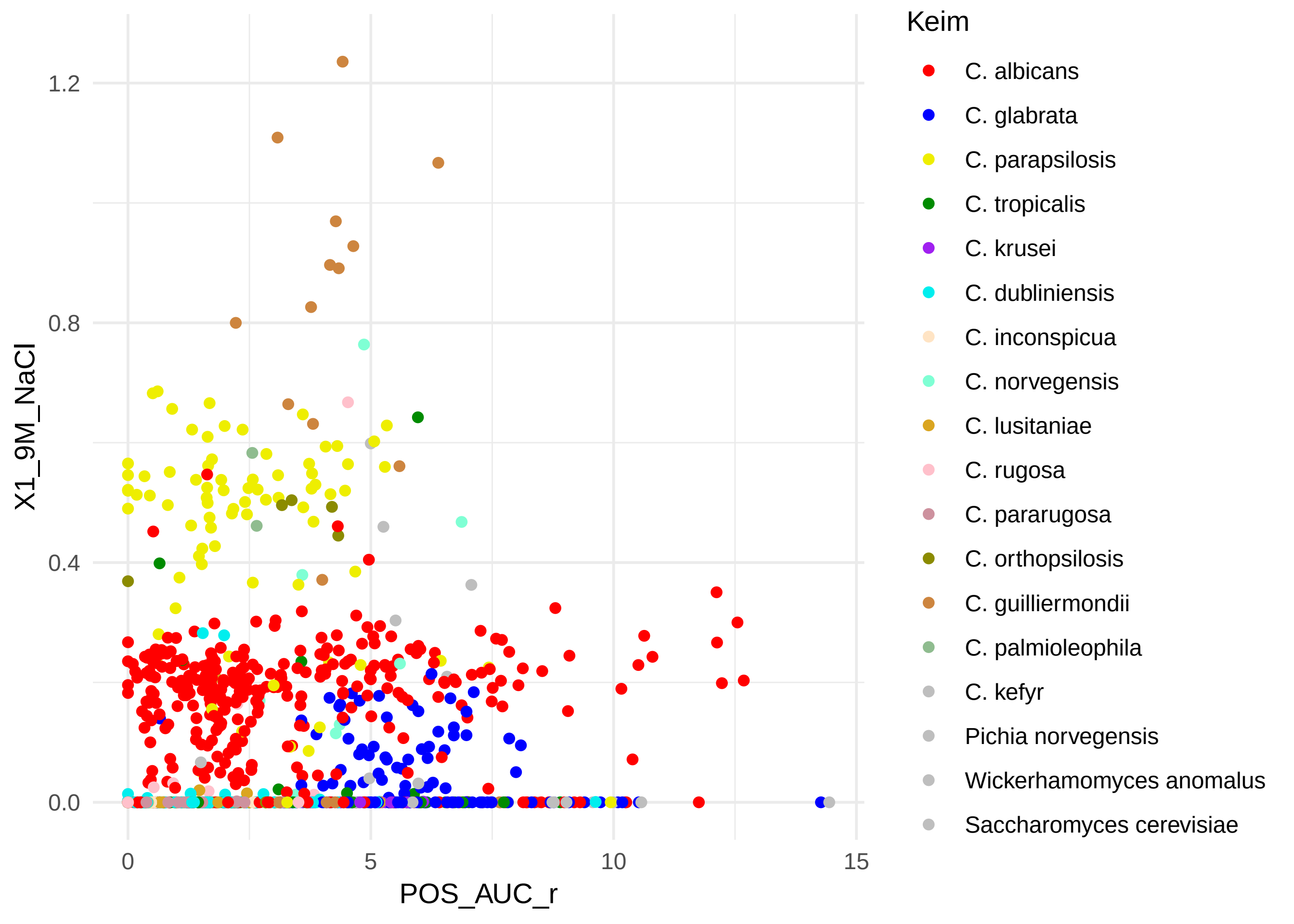
Keim
C. albicans
C. glabrata
C. parapsilosis
C. tropicalis
C. krusei
C. dubliniensis
C. inconspicua
C. norvegensis
C. lusitaniae
C. rugosa
C. pararugosa
C. orthopsilosis
C. guilliermondii
C. palmioleophila
C. kefyr
Pichia norvegensis
Wickerhamomyces anomalus
Saccharomyces cerevisiae
X1_9M_NaCl
POS_AUC_r
0.0
0.4
0.8
1.2
0
5
10
15

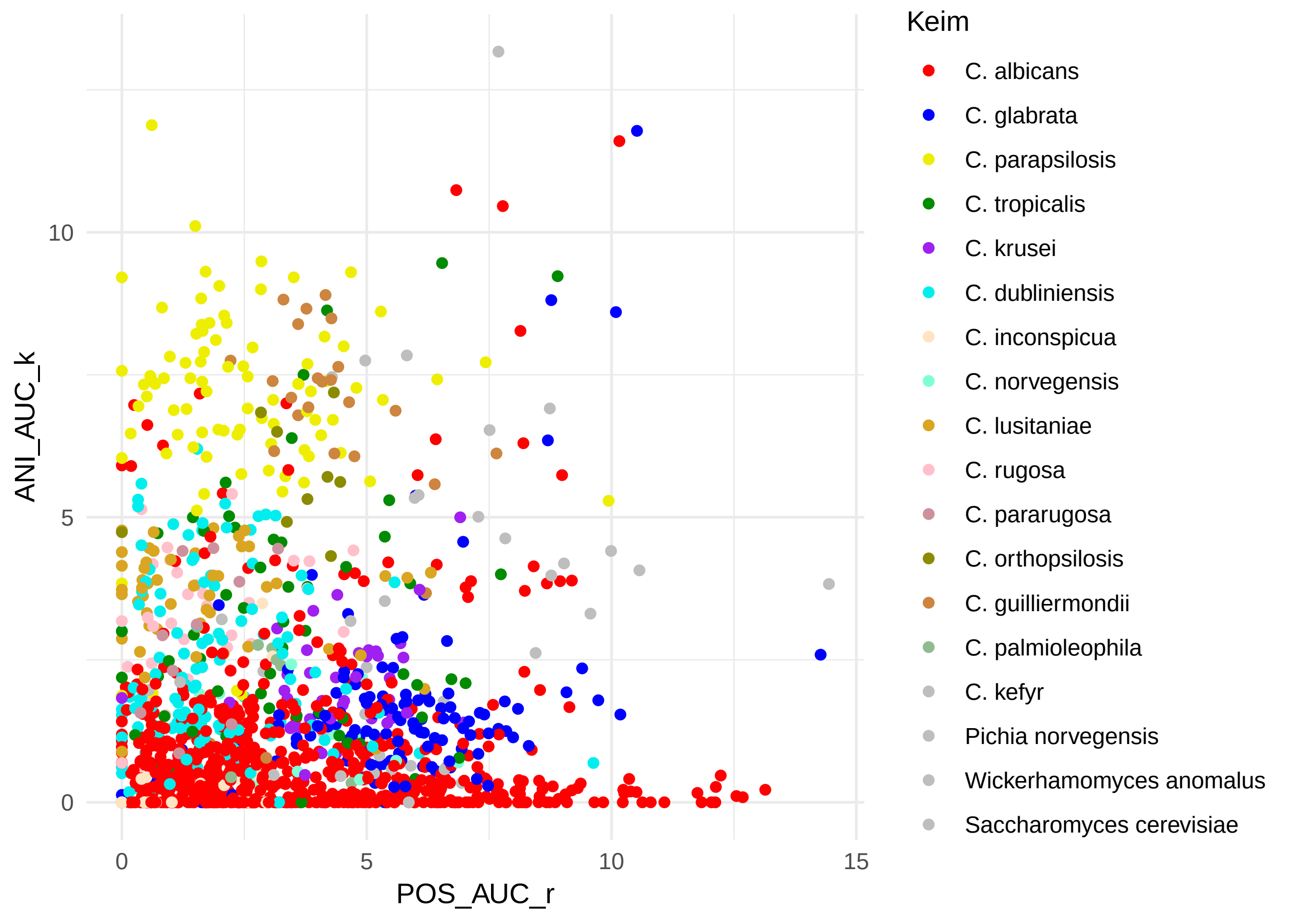

Keim
C. albicans
C. glabrata
C. parapsilosis
C. tropicalis
C. krusei
C. dubliniensis
C. inconspicua
C. norvegensis
C. lusitaniae
C. rugosa
C. pararugosa
C. orthopsilosis
C. guilliermondii
C. palmioleophila
C. kefyr
Pichia norvegensis
Wickerhamomyces anomalus
Saccharomyces cerevisiae
ANI_AUC_k
POS_AUC_r
0
5
10
15

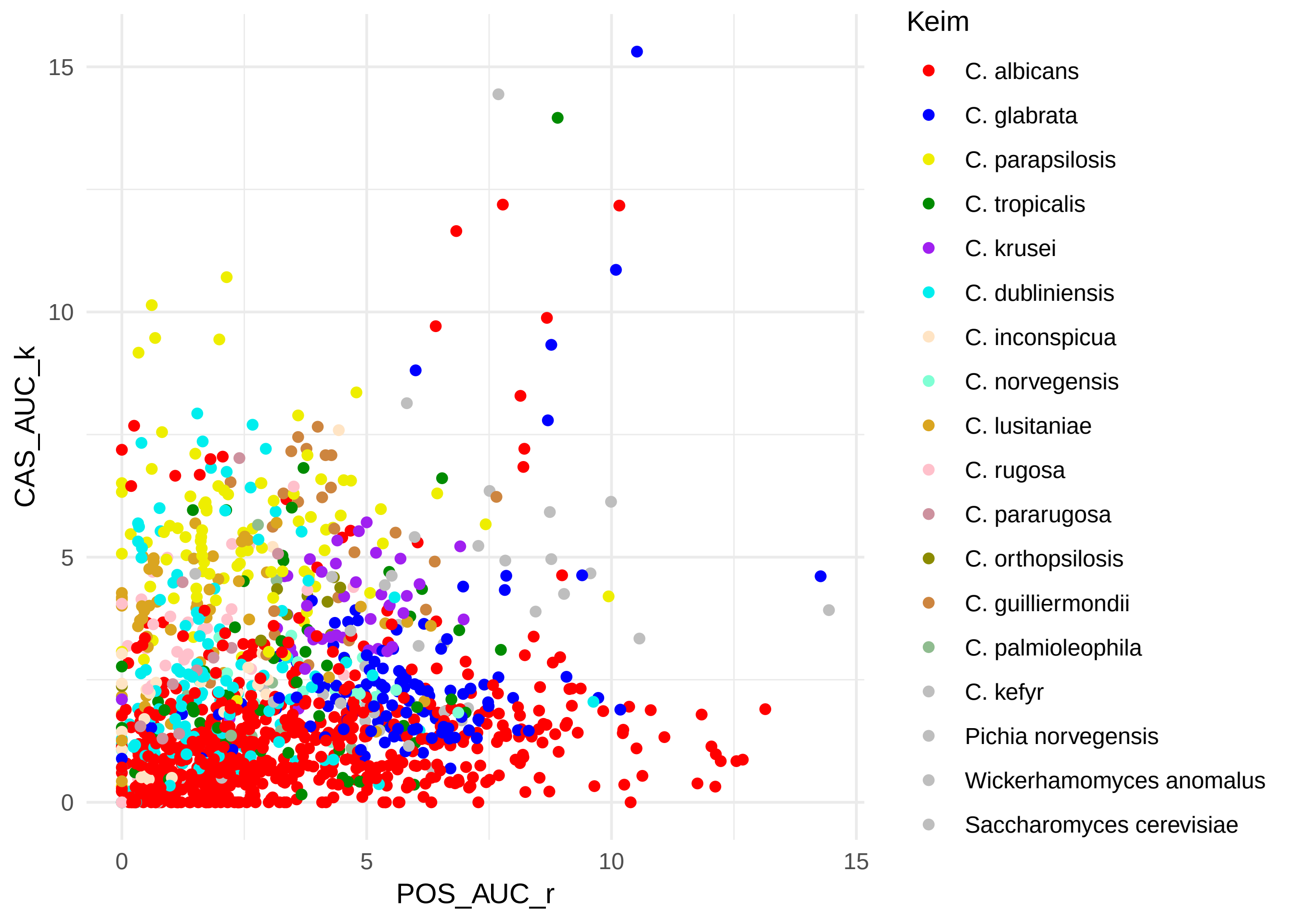

CAS_AUC_k
POS_AUC_r
0
5
10
15
Keim
C. albicans
C. glabrata
C. parapsilosis
C. tropicalis
C. krusei
C. dubliniensis
C. inconspicua
C. norvegensis
C. lusitaniae
C. rugosa
C. pararugosa
C. orthopsilosis
C. guilliermondii
C. palmioleophila
C. kefyr
Pichia norvegensis
Wickerhamomyces anomalus
Saccharomyces cerevisiae

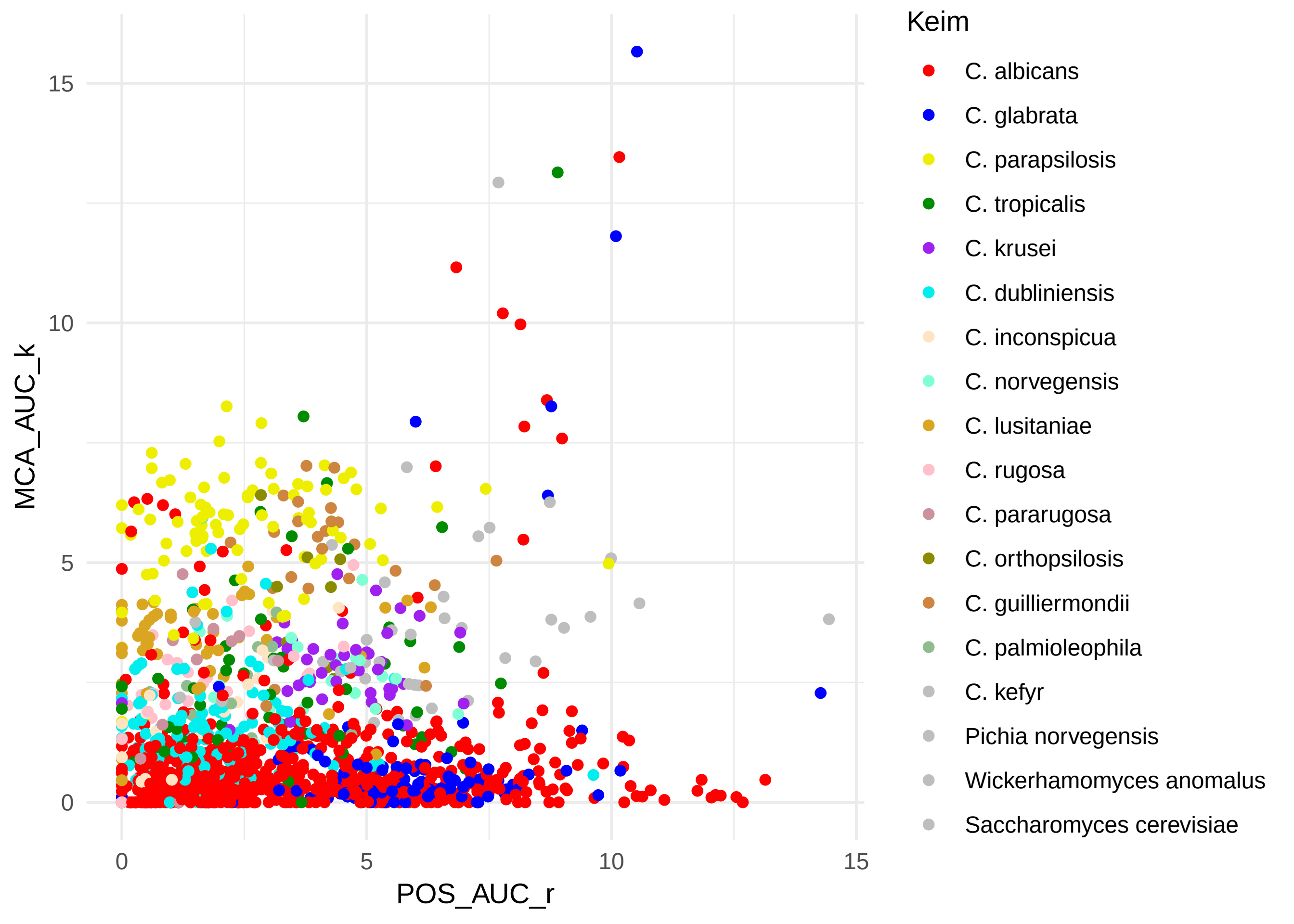

Keim
C. albicans
C. glabrata
C. parapsilosis
C. tropicalis
C. krusei
C. dubliniensis
C. inconspicua
C. norvegensis
C. lusitaniae
C. rugosa
C. pararugosa
C. orthopsilosis
C. guilliermondii
C. palmioleophila
C. kefyr
Pichia norvegensis
Wickerhamomyces anomalus
Saccharomyces cerevisiae
MCA_AUC_k
POS_AUC_r
0
5
10
15

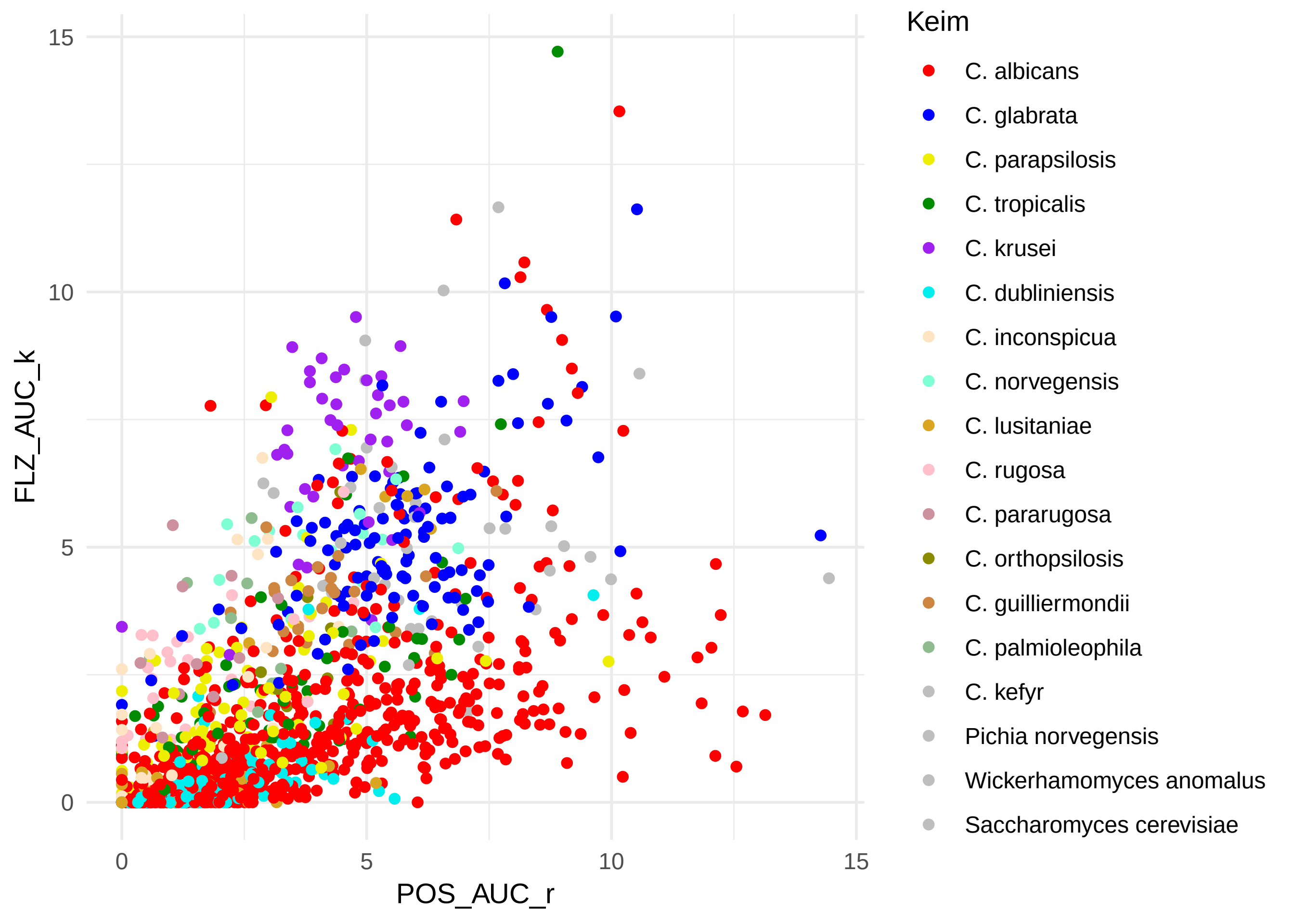

Keim
C. albicans
C. glabrata
C. parapsilosis
C. tropicalis
C. krusei
C. dubliniensis
C. inconspicua
C. norvegensis
C. lusitaniae
C. rugosa
C. pararugosa
C. orthopsilosis
C. guilliermondii
C. palmioleophila
C. kefyr
Pichia norvegensis
Wickerhamomyces anomalus
Saccharomyces cerevisiae
FLZ_AUC_k
POS_AUC_r
0
5
10
15
0
5
10
15

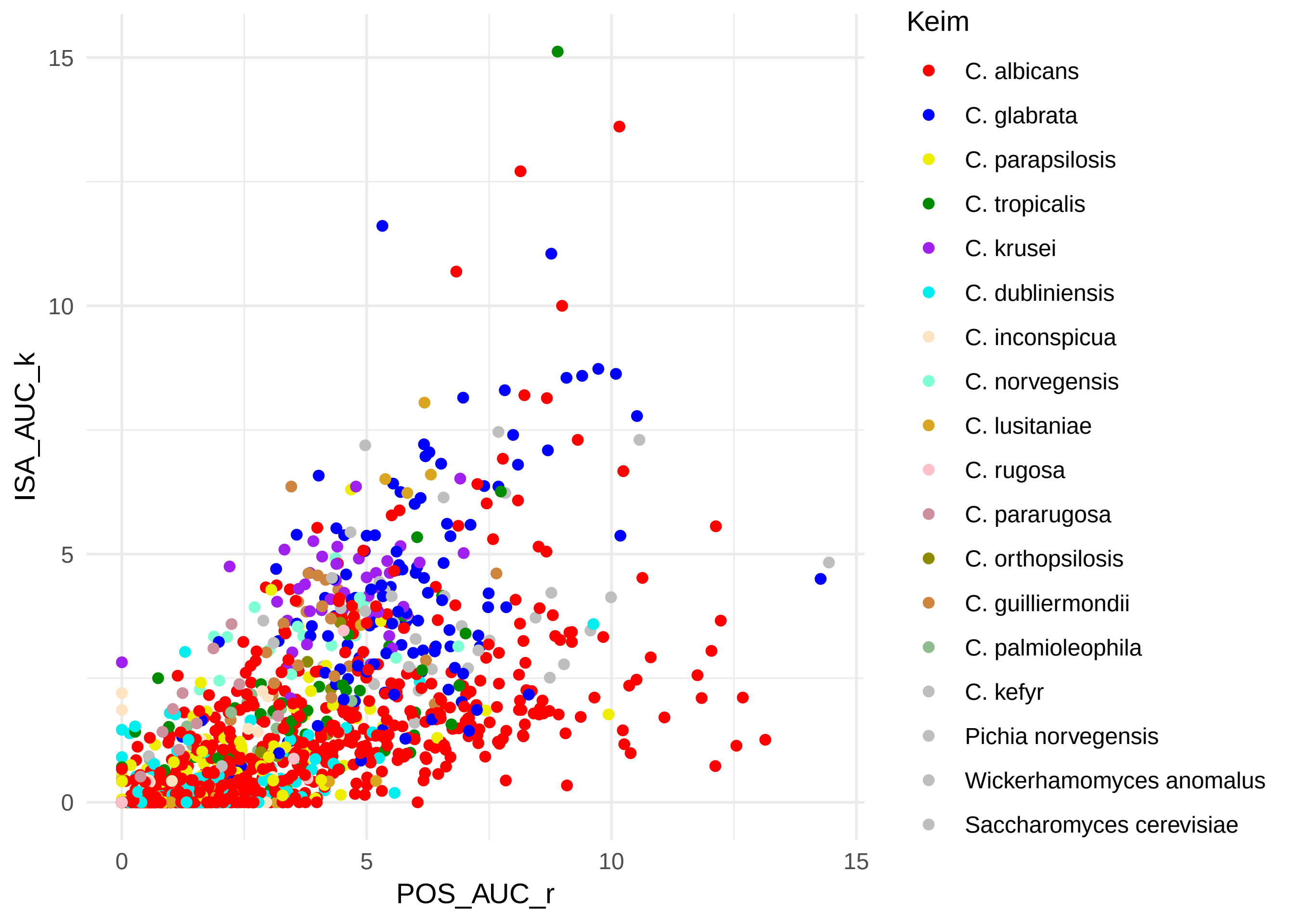

Keim
C. albicans
C. glabrata
C. parapsilosis
C. tropicalis
C. krusei
C. dubliniensis
C. inconspicua
C. norvegensis
C. lusitaniae
C. rugosa
C. pararugosa
C. orthopsilosis
C. guilliermondii
C. palmioleophila
C. kefyr
Pichia norvegensis
Wickerhamomyces anomalus
Saccharomyces cerevisiae
15
10
5
0
ISA_AUC_k
0
5
10
15
POS_AUC_r

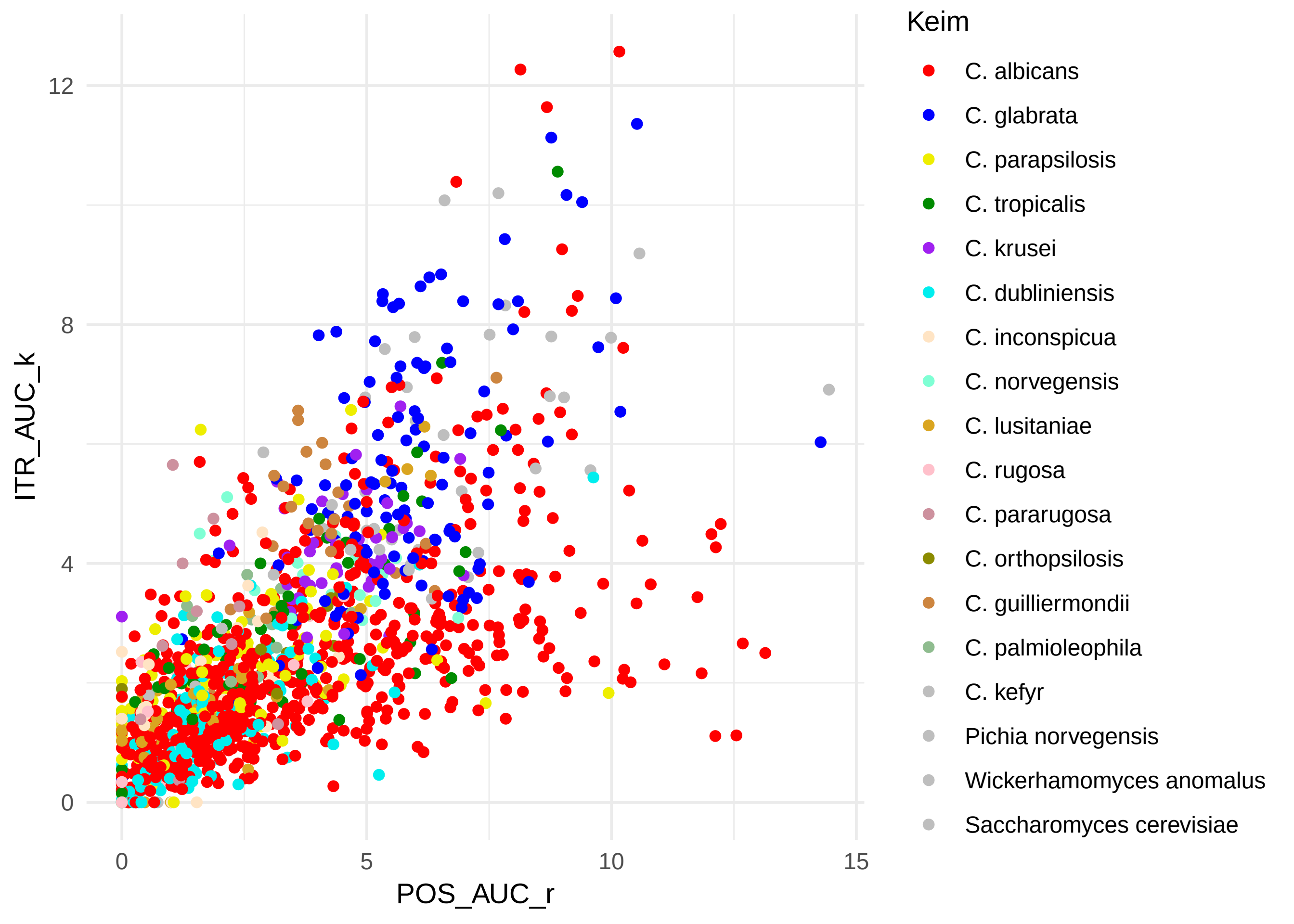

Keim
C. albicans
C. glabrata
C. parapsilosis
C. tropicalis
C. krusei
C. dubliniensis
C. inconspicua
C. norvegensis
C. lusitaniae
C. rugosa
C. pararugosa
C. orthopsilosis
C. guilliermondii
C. palmioleophila
C. kefyr
Pichia norvegensis
Wickerhamomyces anomalus
Saccharomyces cerevisiae
ITR_AUC_k
POS_AUC_r
0
4
8
12
0
5
10
15

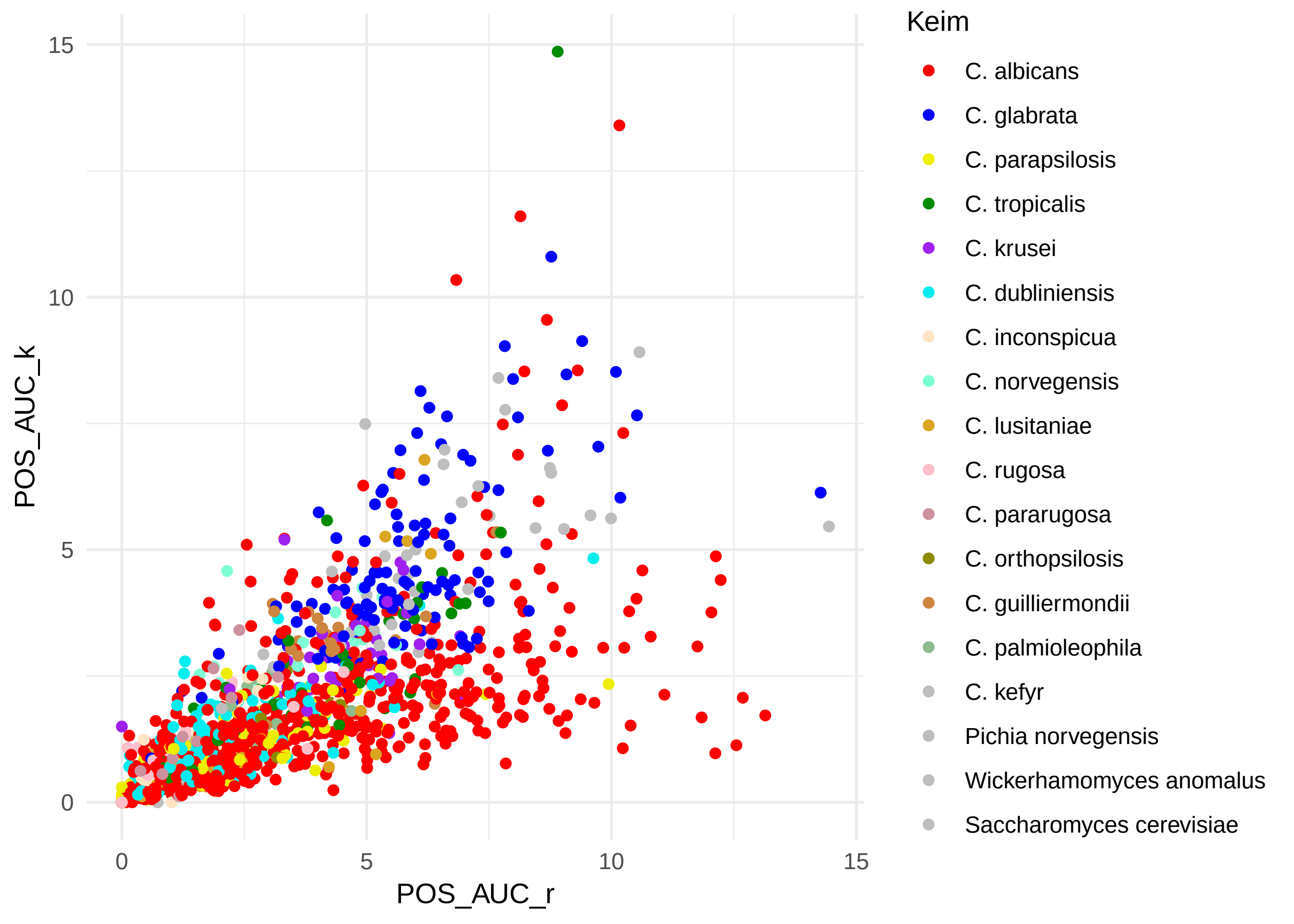

Keim
C. albicans
C. glabrata
C. parapsilosis
C. tropicalis
C. krusei
C. dubliniensis
C. inconspicua
C. norvegensis
C. lusitaniae
C. rugosa
C. pararugosa
C. orthopsilosis
C. guilliermondii
C. palmioleophila
C. kefyr
Pichia norvegensis
Wickerhamomyces anomalus
Saccharomyces cerevisiae
POS_AUC_k
POS_AUC_r
0
5
10
15

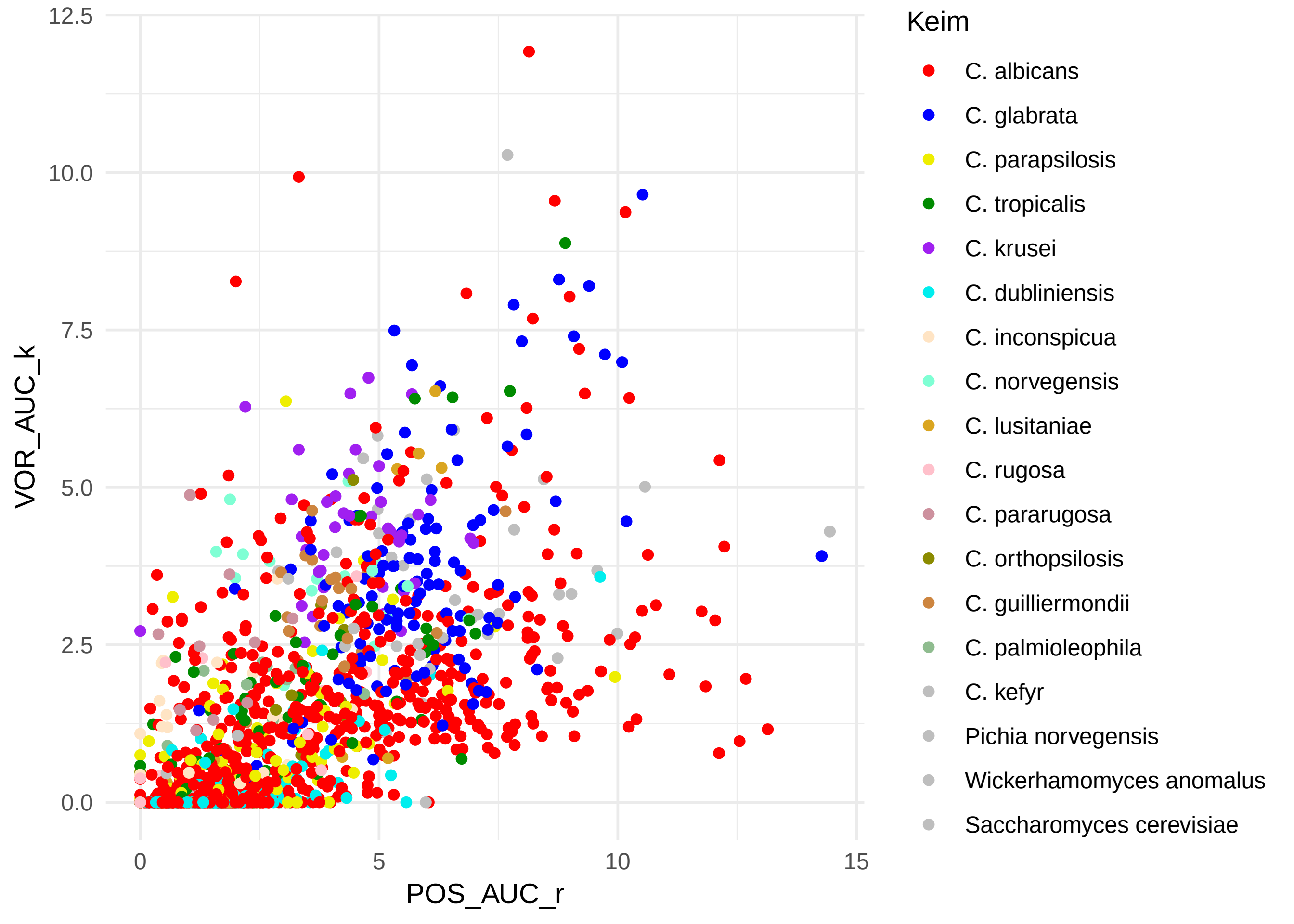

12.5
10.0
7.5
5.0
2.5
0.0
VOR_AUC_k
0
5
10
15
POS_AUC_r
Keim
C. albicans
C. glabrata
C. parapsilosis
C. tropicalis
C. krusei
C. dubliniensis
C. inconspicua
C. norvegensis
C. lusitaniae
C. rugosa
C. pararugosa
C. orthopsilosis
C. guilliermondii
C. palmioleophila
C. kefyr
Pichia norvegensis
Wickerhamomyces anomalus
Saccharomyces cerevisiae

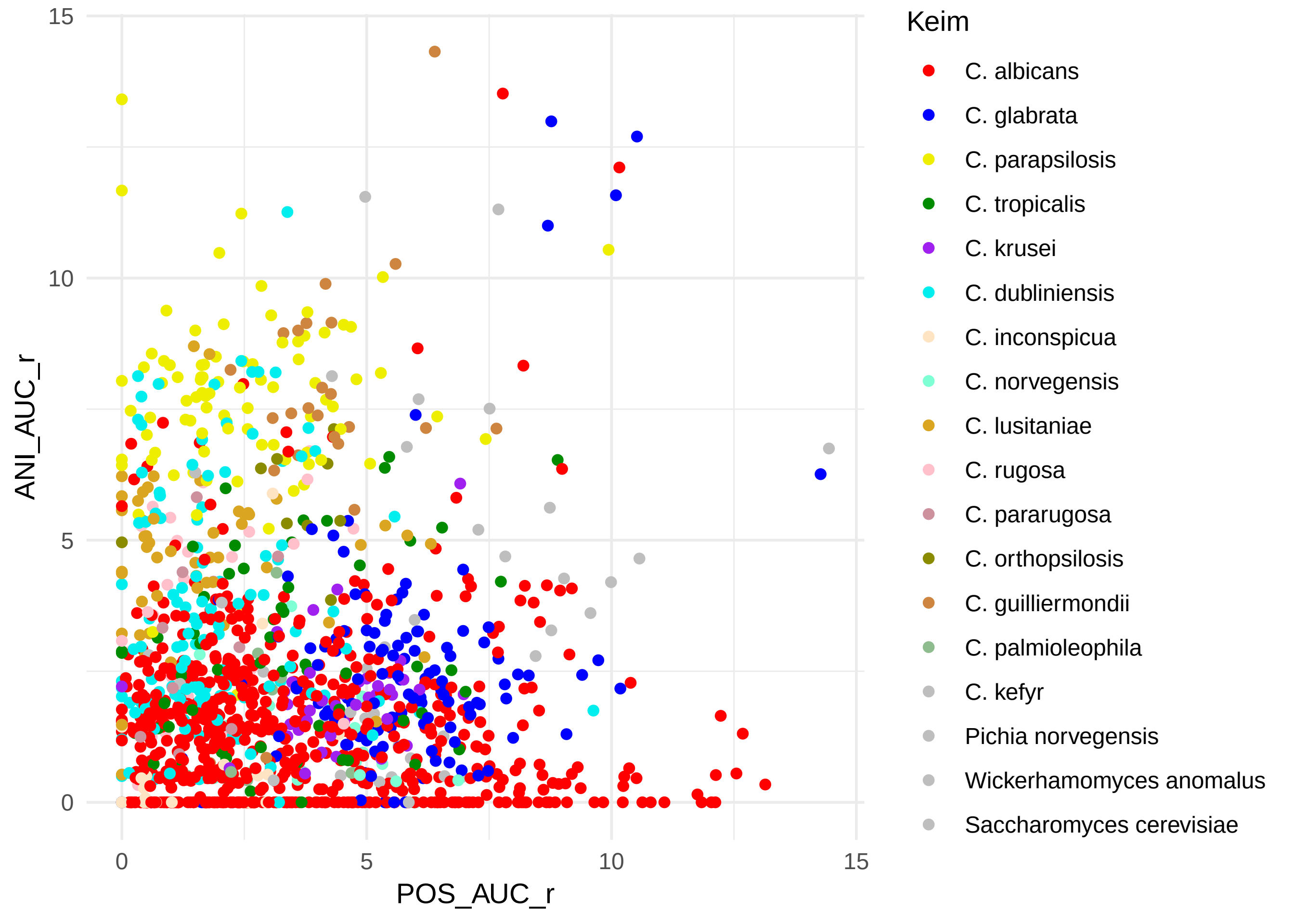

Keim
C. albicans
C. glabrata
C. parapsilosis
C. tropicalis
C. krusei
C. dubliniensis
C. inconspicua
C. norvegensis
C. lusitaniae
C. rugosa
C. pararugosa
C. orthopsilosis
C. guilliermondii
C. palmioleophila
C. kefyr
Pichia norvegensis
Wickerhamomyces anomalus
Saccharomyces cerevisiae
ANI_AUC_r
POS_AUC_r
0
5
10
15

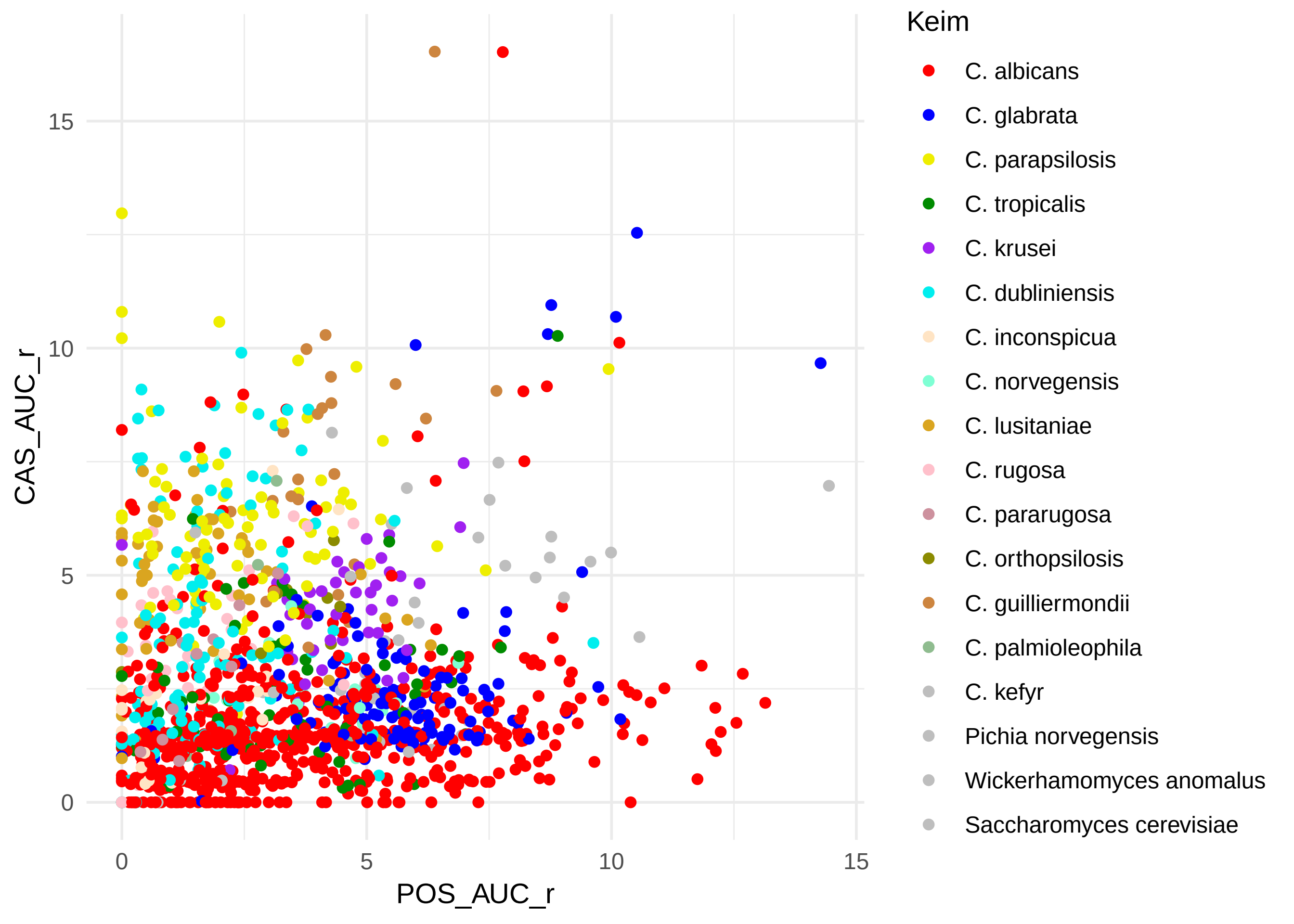

Keim
C. albicans
C. glabrata
C. parapsilosis
C. tropicalis
C. krusei
C. dubliniensis
C. inconspicua
C. norvegensis
C. lusitaniae
C. rugosa
C. pararugosa
C. orthopsilosis
C. guilliermondii
C. palmioleophila
C. kefyr
Pichia norvegensis
Wickerhamomyces anomalus
Saccharomyces cerevisiae
CAS_AUC_r
POS_AUC_r
0
5
10
15

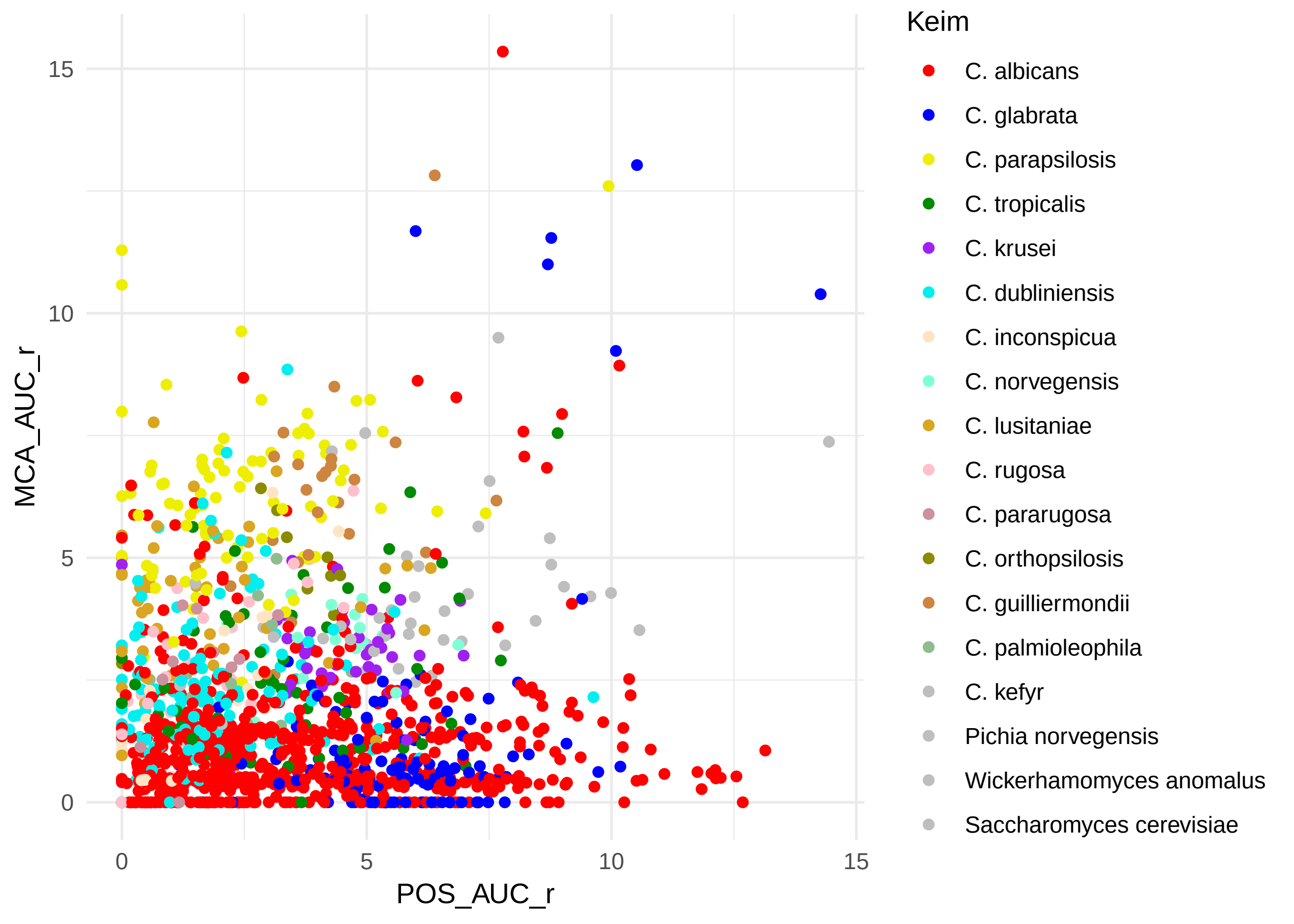

Keim
C. albicans
C. glabrata
C. parapsilosis
C. tropicalis
C. krusei
C. dubliniensis
C. inconspicua
C. norvegensis
C. lusitaniae
C. rugosa
C. pararugosa
C. orthopsilosis
C. guilliermondii
C. palmioleophila
C. kefyr
Pichia norvegensis
Wickerhamomyces anomalus
Saccharomyces cerevisiae
MCA_AUC_r
POS_AUC_r
0
5
10
15

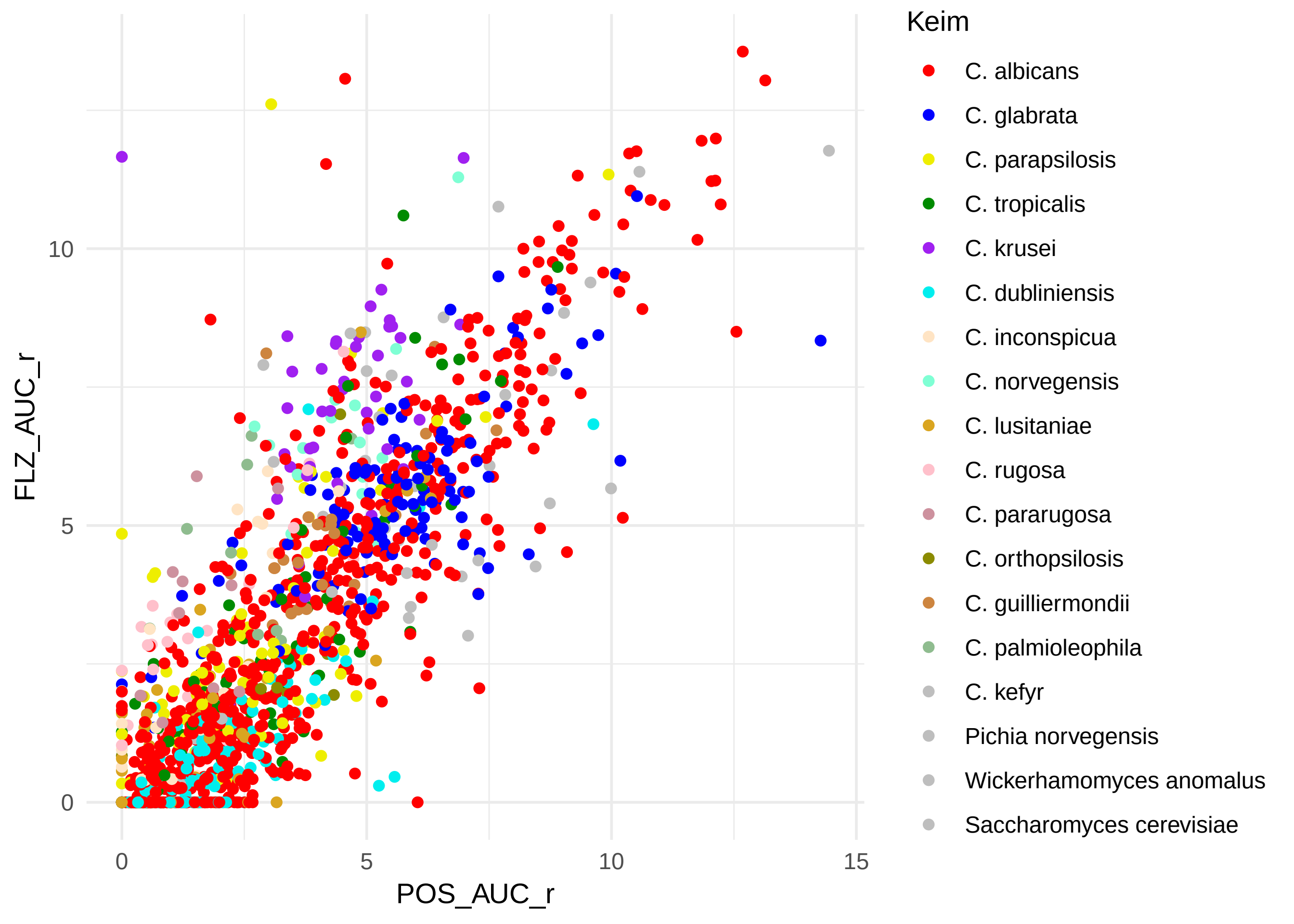

Keim
C. albicans
C. glabrata
C. parapsilosis
C. tropicalis
C. krusei
C. dubliniensis
C. inconspicua
C. norvegensis
C. lusitaniae
C. rugosa
C. pararugosa
C. orthopsilosis
C. guilliermondii
C. palmioleophila
C. kefyr
Pichia norvegensis
Wickerhamomyces anomalus
Saccharomyces cerevisiae
FLZ_AUC_r
POS_AUC_r
0
5
10
15

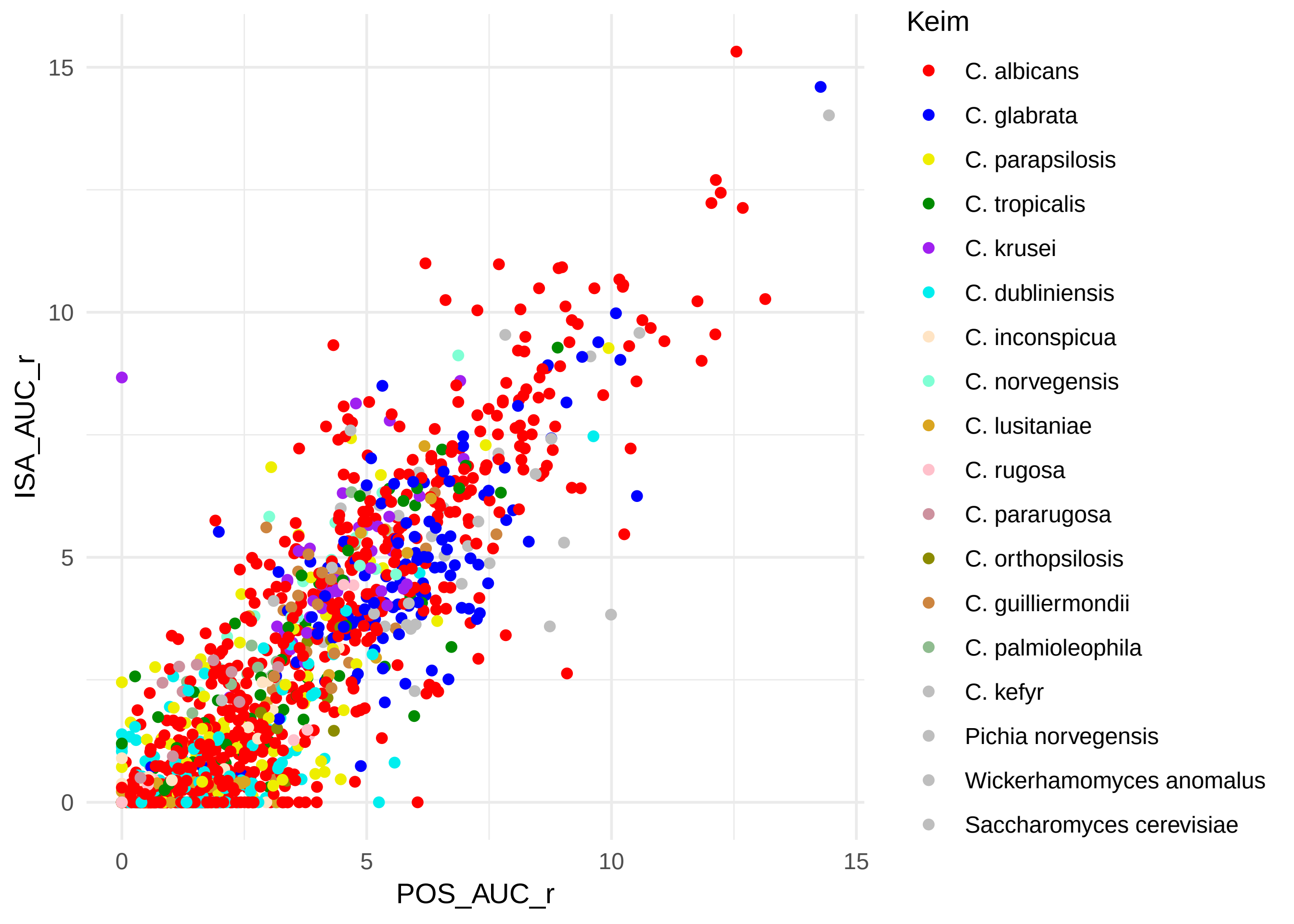
Keim
C. albicans
C. glabrata
C. parapsilosis
C. tropicalis
C. krusei
C. dubliniensis
C. inconspicua
C. norvegensis
C. lusitaniae
C. rugosa
C. pararugosa
C. orthopsilosis
C. guilliermondii
C. palmioleophila
C. kefyr
Pichia norvegensis
Wickerhamomyces anomalus
Saccharomyces cerevisiae
ISA_AUC_r
POS_AUC_r
0
5
10
15
0
5
10
15

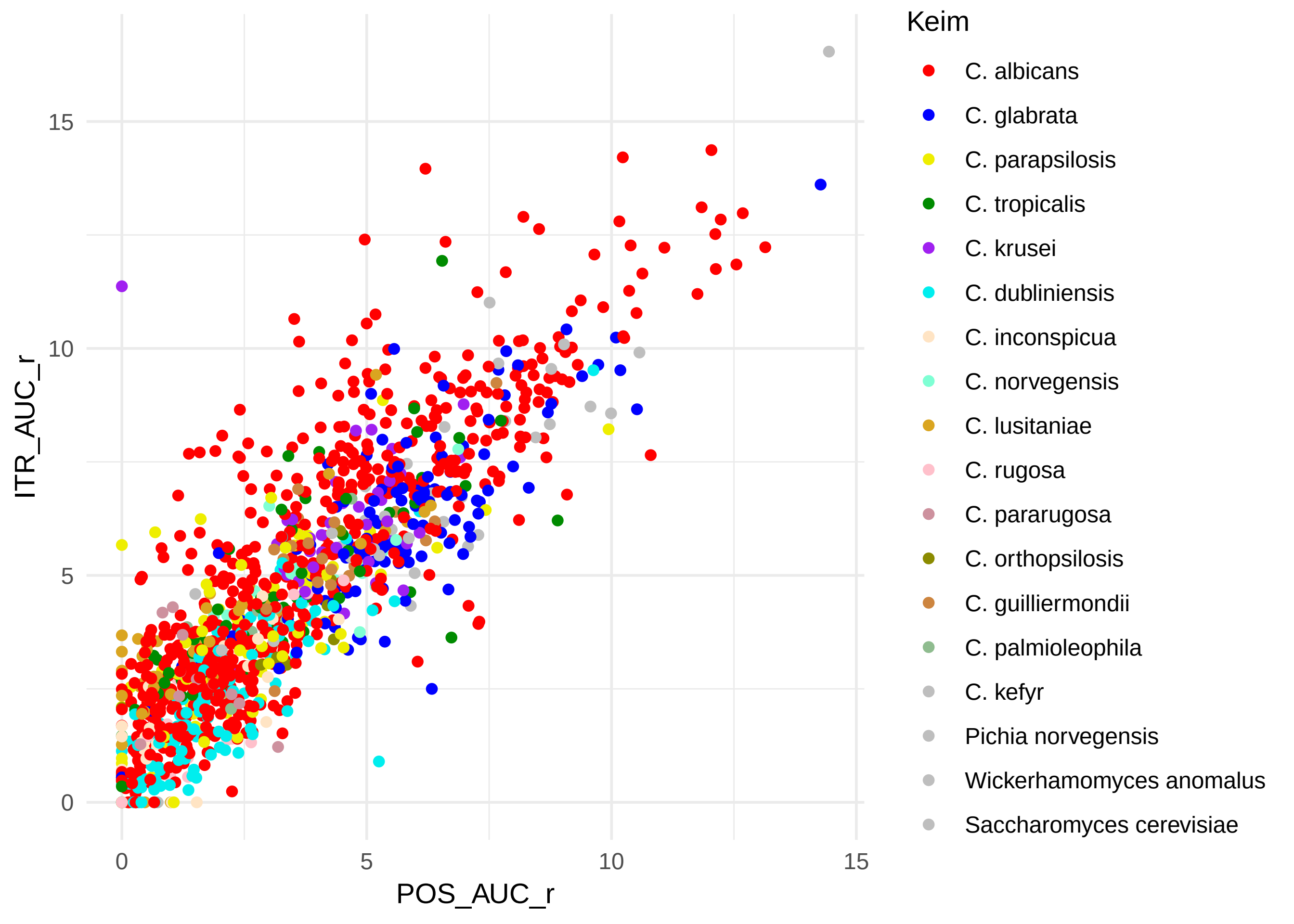

Keim
C. albicans
C. glabrata
C. parapsilosis
C. tropicalis
C. krusei
C. dubliniensis
C. inconspicua
C. norvegensis
C. lusitaniae
C. rugosa
C. pararugosa
C. orthopsilosis
C. guilliermondii
C. palmioleophila
C. kefyr
Pichia norvegensis
Wickerhamomyces anomalus
Saccharomyces cerevisiae
ITR_AUC_r
POS_AUC_r
0
5
10
15

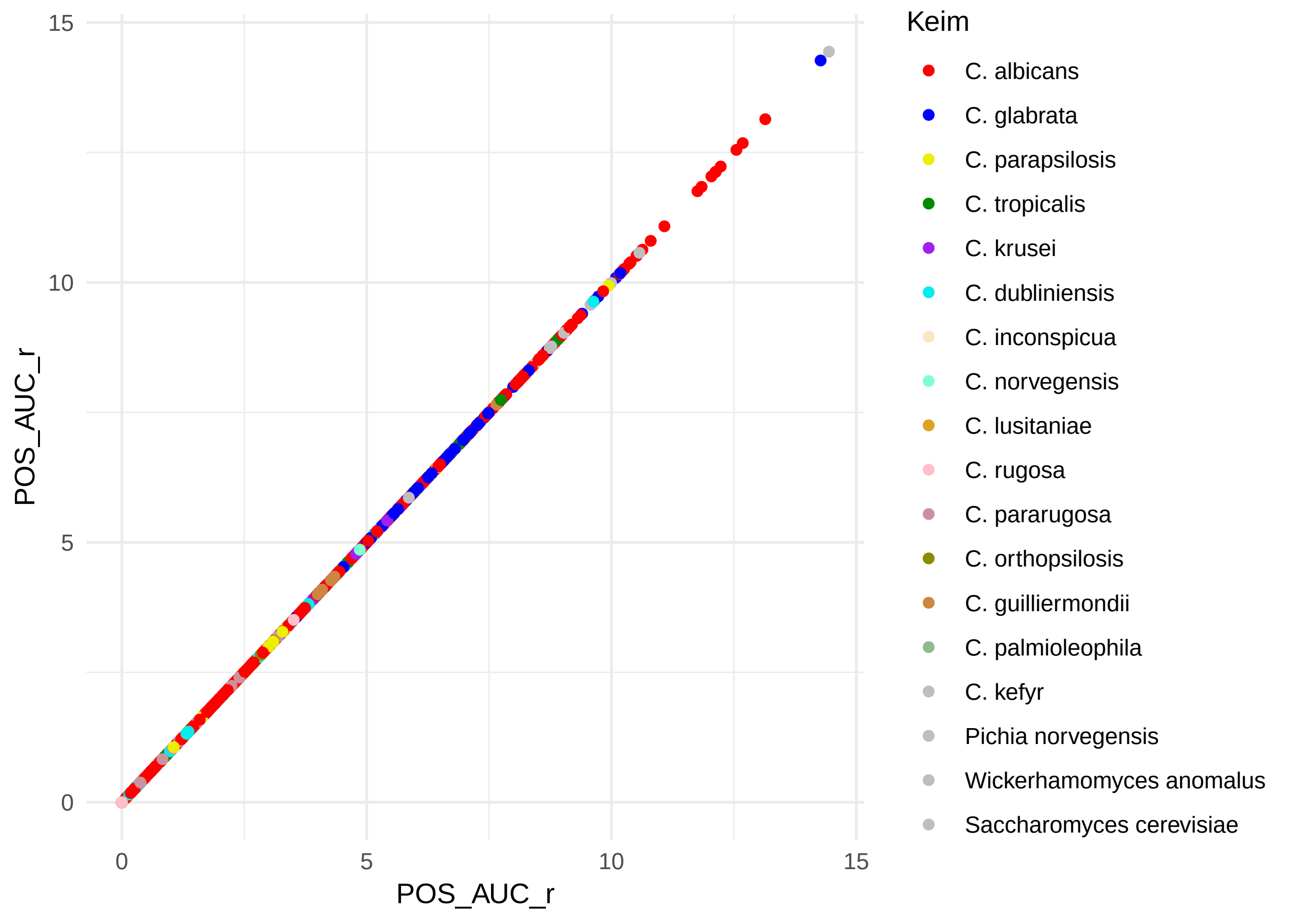

15
10
5
0
POS_AUC_r
0
5
10
15
POS_AUC_r
Keim
C. albicans
C. glabrata
C. parapsilosis
C. tropicalis
C. krusei
C. dubliniensis
C. inconspicua
C. norvegensis
C. lusitaniae
C. rugosa
C. pararugosa
C. orthopsilosis
C. guilliermondii
C. palmioleophila
C. kefyr
Pichia norvegensis
Wickerhamomyces anomalus
Saccharomyces cerevisiae

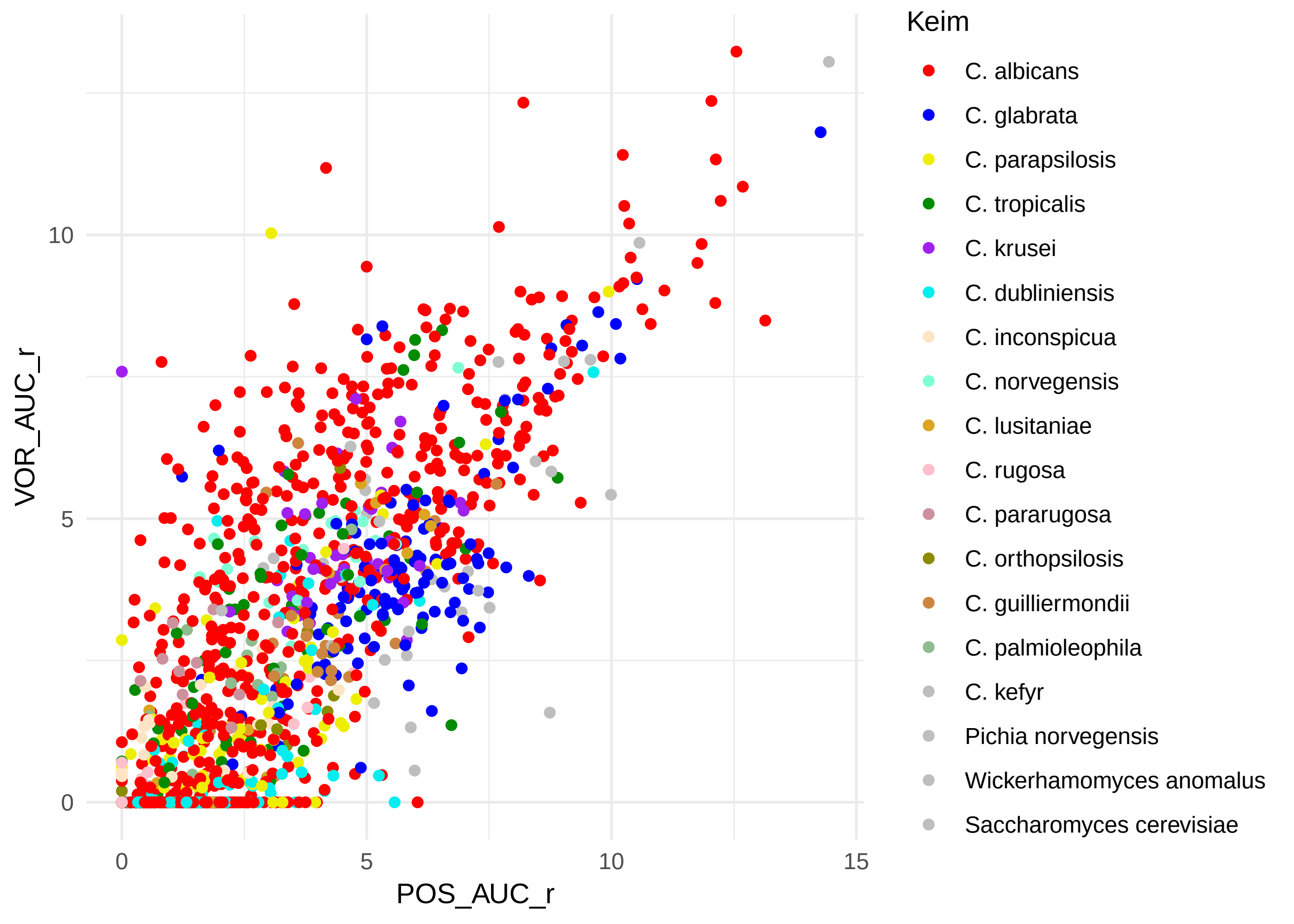
Keim
C. albicans
C. glabrata
C. parapsilosis
C. tropicalis
C. krusei
C. dubliniensis
C. inconspicua
C. norvegensis
C. lusitaniae
C. rugosa
C. pararugosa
C. orthopsilosis
C. guilliermondii
C. palmioleophila
C. kefyr
Pichia norvegensis
Wickerhamomyces anomalus
Saccharomyces cerevisiae
VOR_AUC_r
POS_AUC_r
0
5
10
15

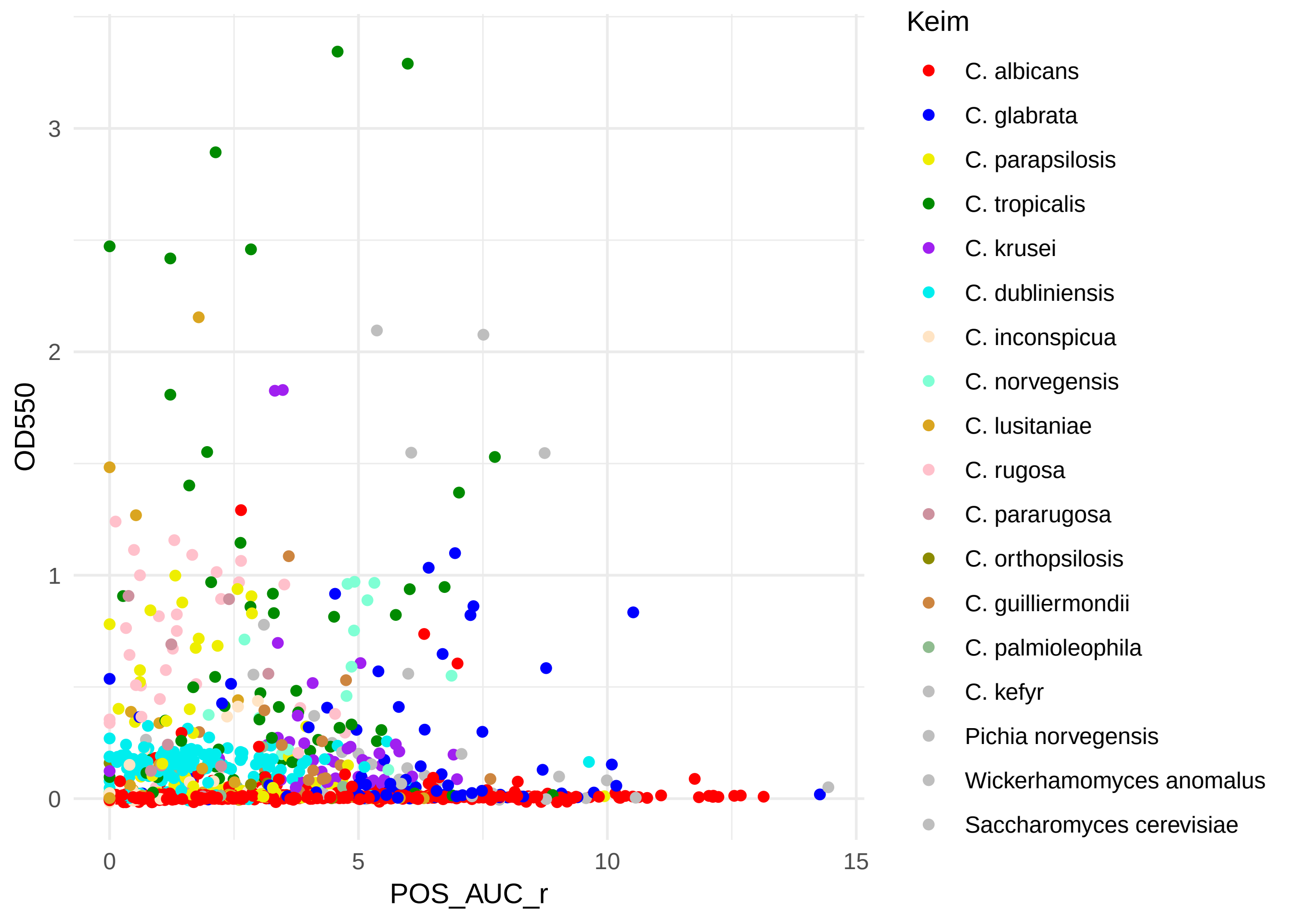
Keim
C. albicans
C. glabrata
C. parapsilosis
C. tropicalis
C. krusei
C. dubliniensis
C. inconspicua
C. norvegensis
C. lusitaniae
C. rugosa
C. pararugosa
C. orthopsilosis
C. guilliermondii
C. palmioleophila
C. kefyr
Pichia norvegensis
Wickerhamomyces anomalus
Saccharomyces cerevisiae
OD550
POS_AUC_r
0
1
2
3
0
5
10
15

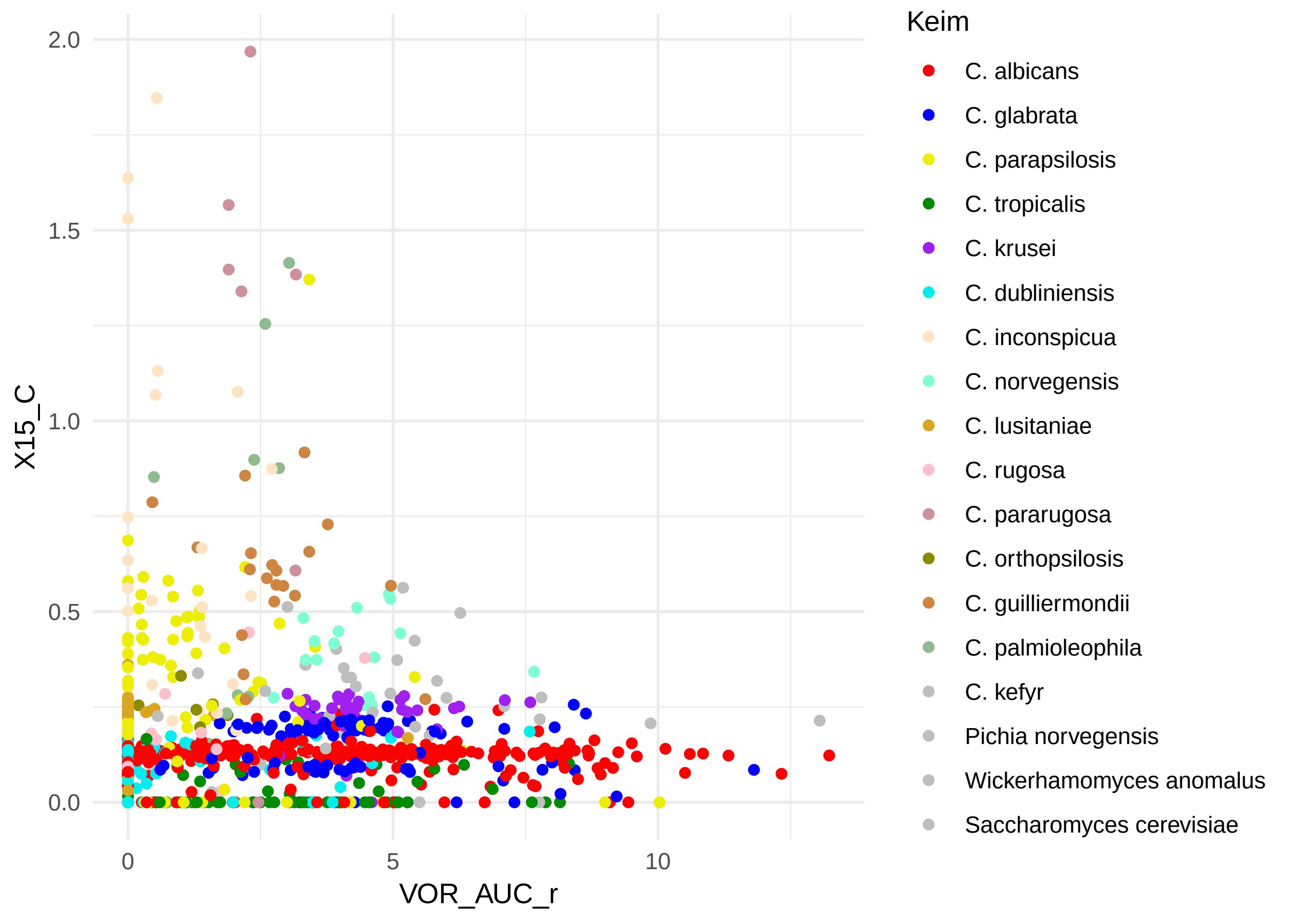

X15_C
2.0
1.5
1.0
0.5
0.0
0
5
10
VOR_AUC_r
Keim
C. albicans
C. glabrata
C. parapsilosis
C. tropicalis
C. krusei
C. dubliniensis
C. inconspicua
C. norvegensis
C. lusitaniae
C. rugosa
C. pararugosa
C. orthopsilosis
C. guilliermondii
C. palmioleophila
C. kefyr
Pichia norvegensis
Wickerhamomyces anomalus
Saccharomyces cerevisiae

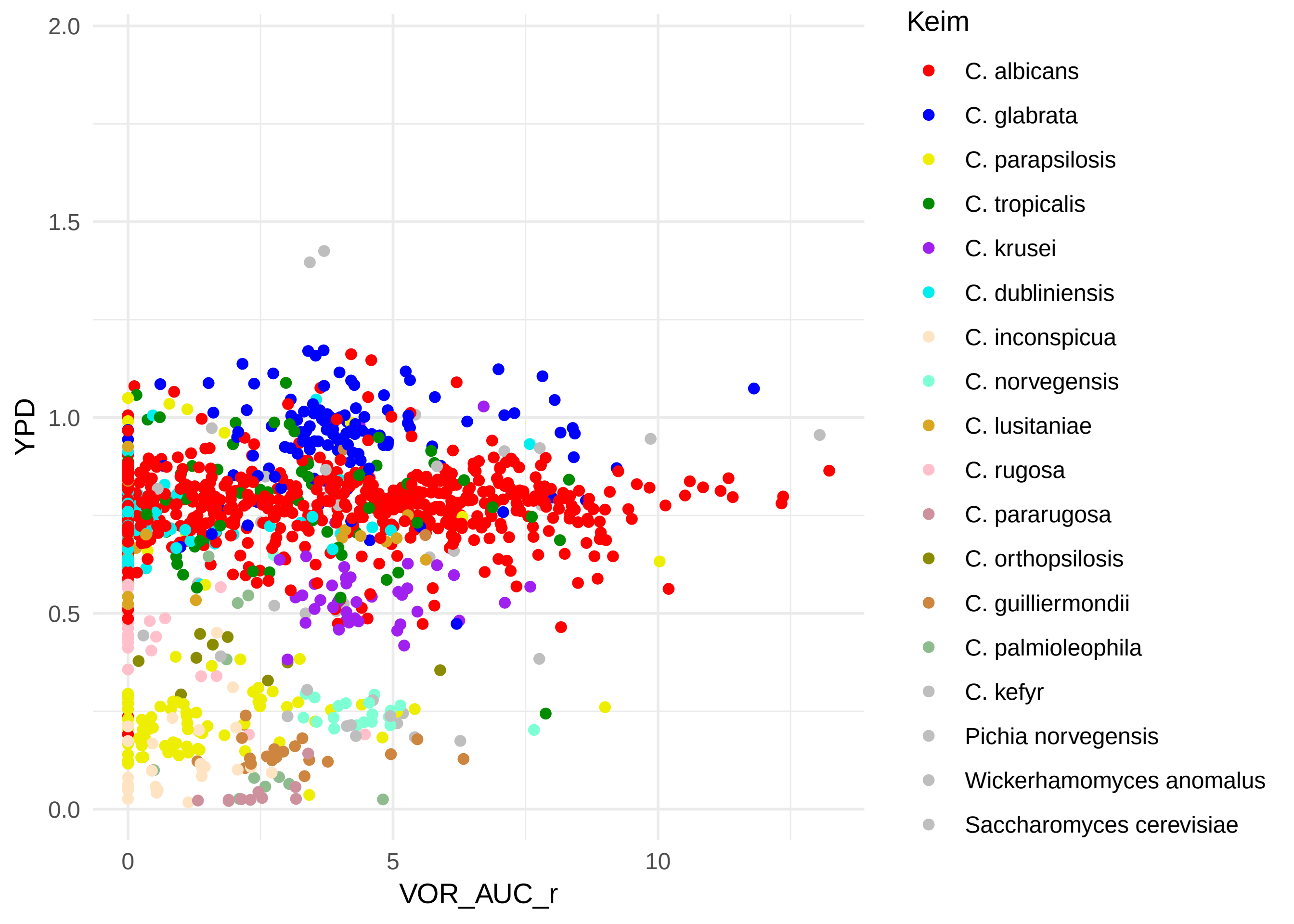

2.0
1.5
1.0
0.5
0.0
YPD
0
5
10
VOR_AUC_r
Keim
C. albicans
C. glabrata
C. parapsilosis
C. tropicalis
C. krusei
C. dubliniensis
C. inconspicua
C. norvegensis
C. lusitaniae
C. rugosa
C. pararugosa
C. orthopsilosis
C. guilliermondii
C. palmioleophila
C. kefyr
Pichia norvegensis
Wickerhamomyces anomalus
Saccharomyces cerevisiae

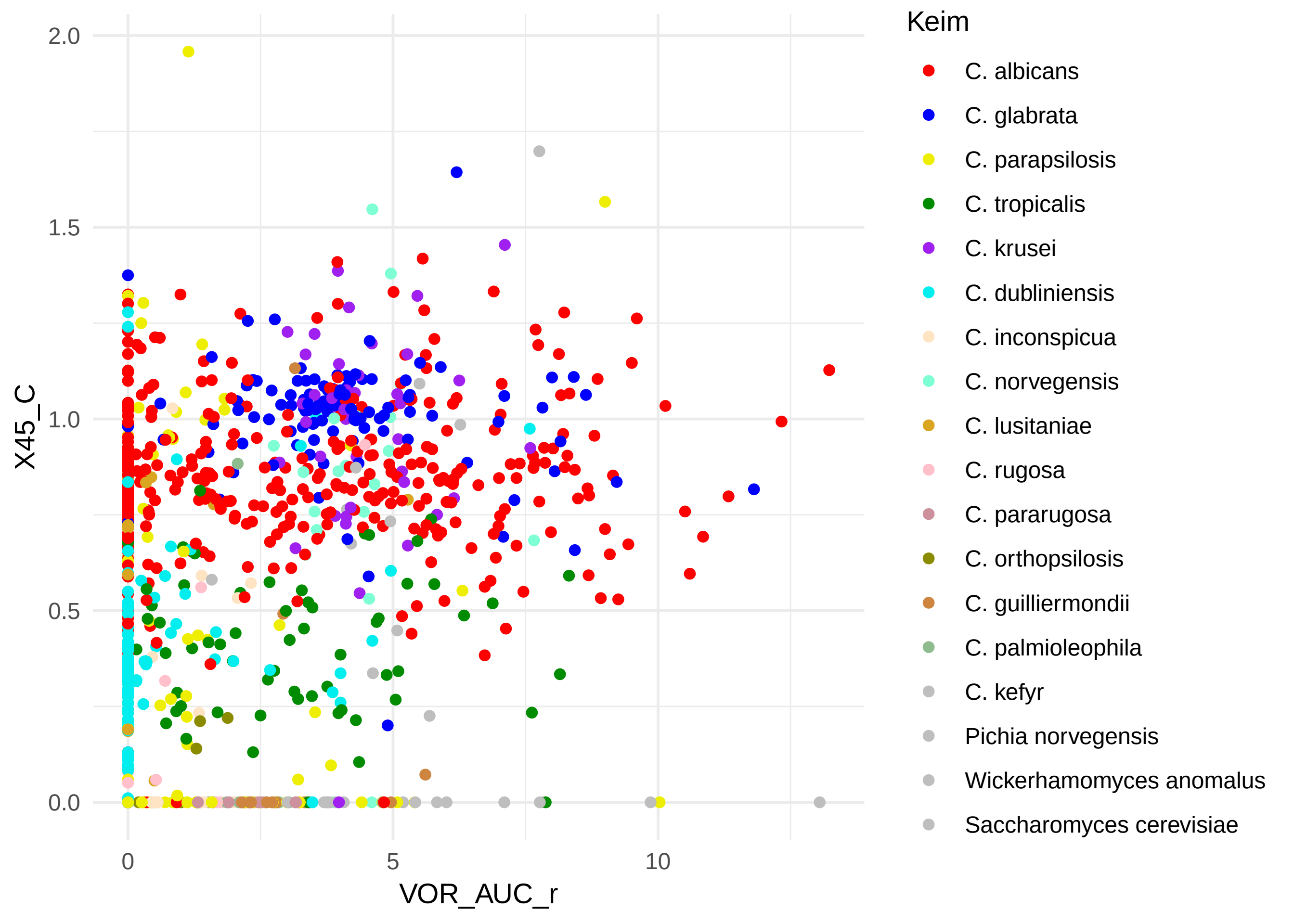

X45_C
VOR_AUC_r
2.0
1.5
1.0
0.5
0.0
0
5
10
Keim
C. albicans
C. glabrata
C. parapsilosis
C. tropicalis
C. krusei
C. dubliniensis
C. inconspicua
C. norvegensis
C. lusitaniae
C. rugosa
C. pararugosa
C. orthopsilosis
C. guilliermondii
C. palmioleophila
C. kefyr
Pichia norvegensis
Wickerhamomyces anomalus
Saccharomyces cerevisiae

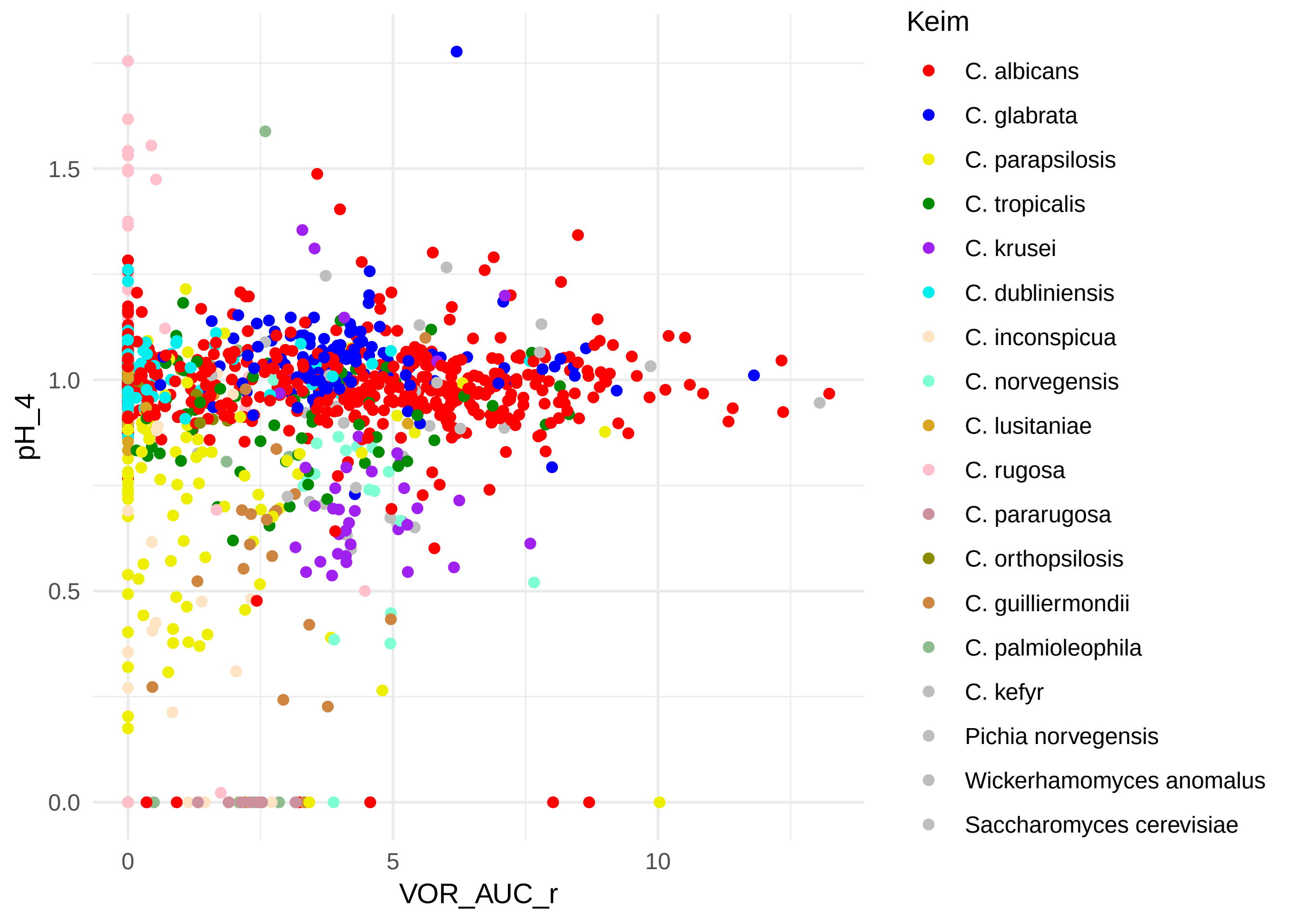
Keim
C. albicans
C. glabrata
C. parapsilosis
C. tropicalis
C. krusei
C. dubliniensis
C. inconspicua
C. norvegensis
C. lusitaniae
C. rugosa
C. pararugosa
C. orthopsilosis
C. guilliermondii
C. palmioleophila
C. kefyr
Pichia norvegensis
Wickerhamomyces anomalus
Saccharomyces cerevisiae
pH_4
VOR_AUC_r
0.0
0.5
1.0
1.5
0
5
10

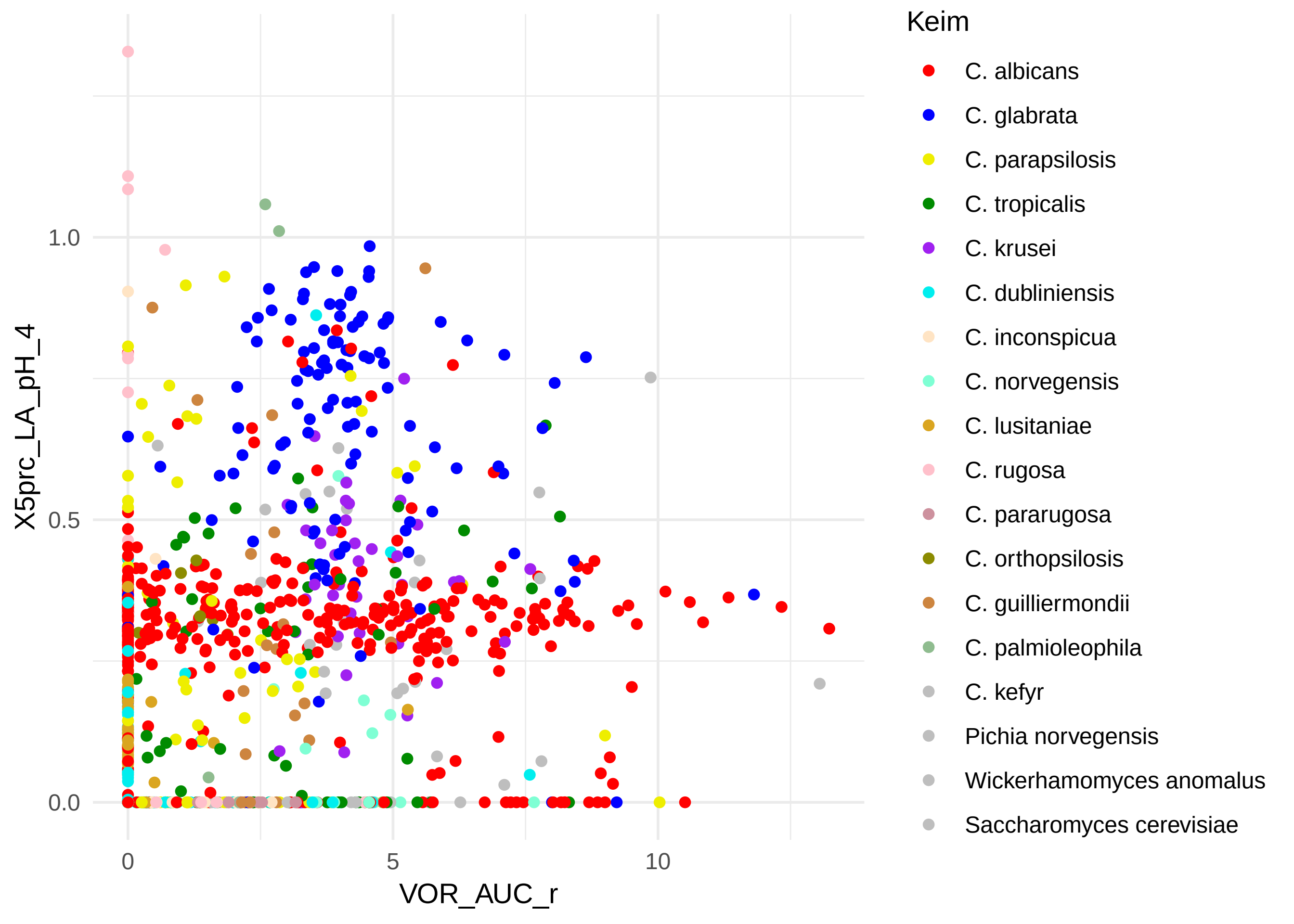
Keim
C. albicans
C. glabrata
C. parapsilosis
C. tropicalis
C. krusei
C. dubliniensis
C. inconspicua
C. norvegensis
C. lusitaniae
C. rugosa
C. pararugosa
C. orthopsilosis
C. guilliermondii
C. palmioleophila
C. kefyr
Pichia norvegensis
Wickerhamomyces anomalus
Saccharomyces cerevisiae
X5prc_LA_pH_4
1.0
0.5
0.0
0
5
10
VOR_AUC_r

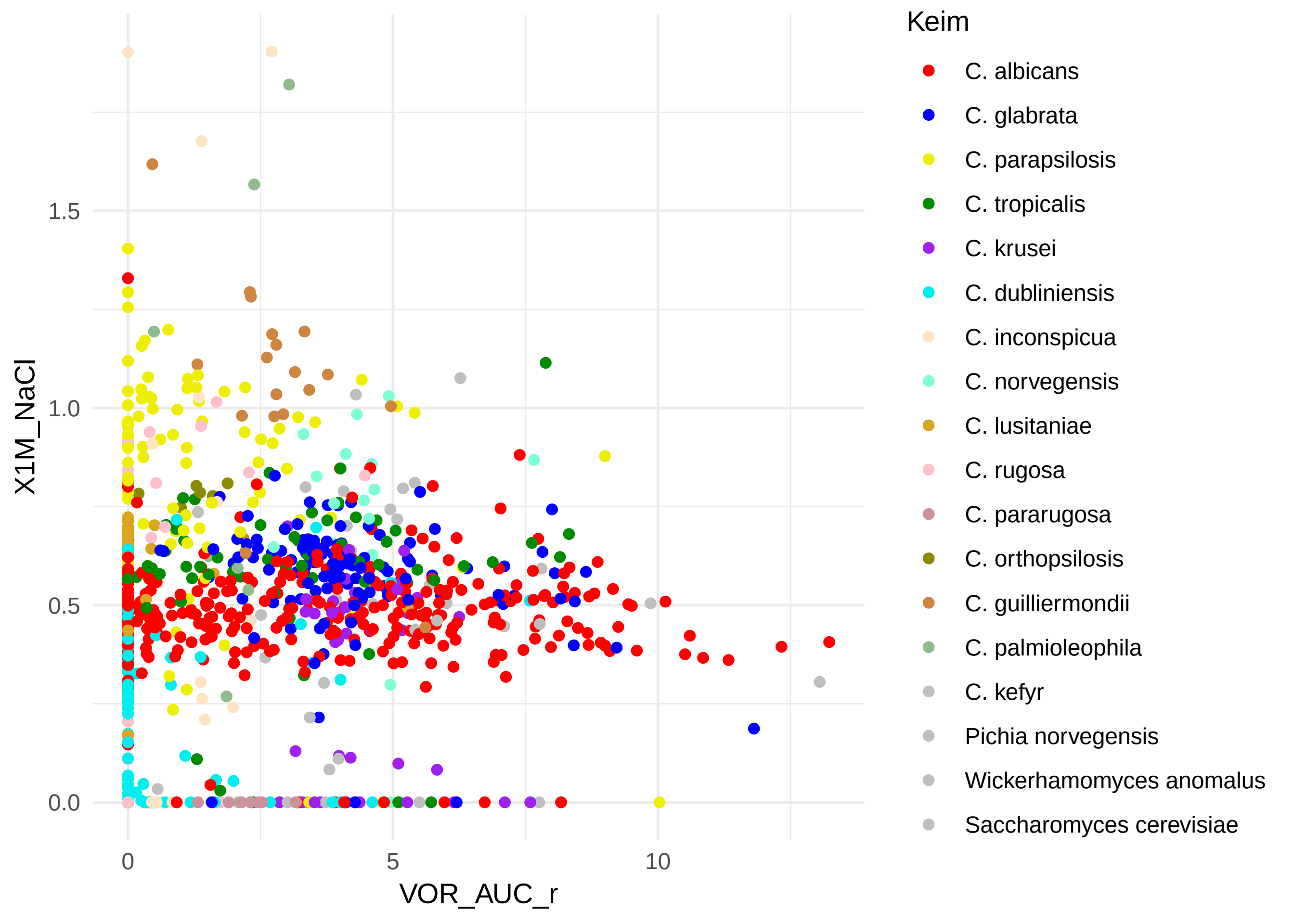

X1M_NaCl
VOR_AUC_r
0.0
0.5
1.0
1.5
0
5
10
Keim
C. albicans
C. glabrata
C. parapsilosis
C. tropicalis
C. krusei
C. dubliniensis
C. inconspicua
C. norvegensis
C. lusitaniae
C. rugosa
C. pararugosa
C. orthopsilosis
C. guilliermondii
C. palmioleophila
C. kefyr
Pichia norvegensis
Wickerhamomyces anomalus
Saccharomyces cerevisiae

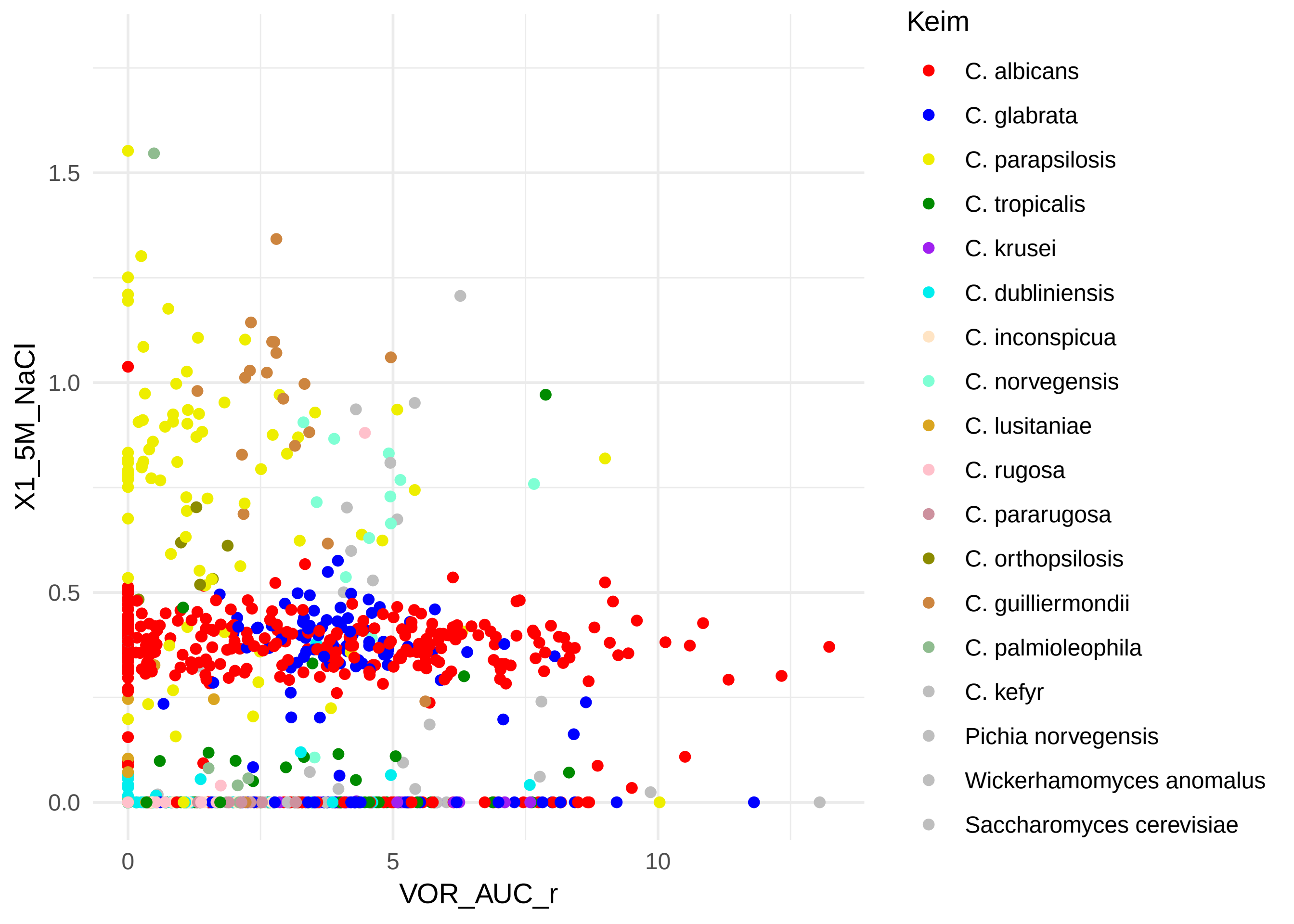

X1_5M_NaCl
0.0
0.5
1.0
1.5
0
5
10
VOR_AUC_r
Keim
C. albicans
C. glabrata
C. parapsilosis
C. tropicalis
C. krusei
C. dubliniensis
C. inconspicua
C. norvegensis
C. lusitaniae
C. rugosa
C. pararugosa
C. orthopsilosis
C. guilliermondii
C. palmioleophila
C. kefyr
Pichia norvegensis
Wickerhamomyces anomalus
Saccharomyces cerevisiae

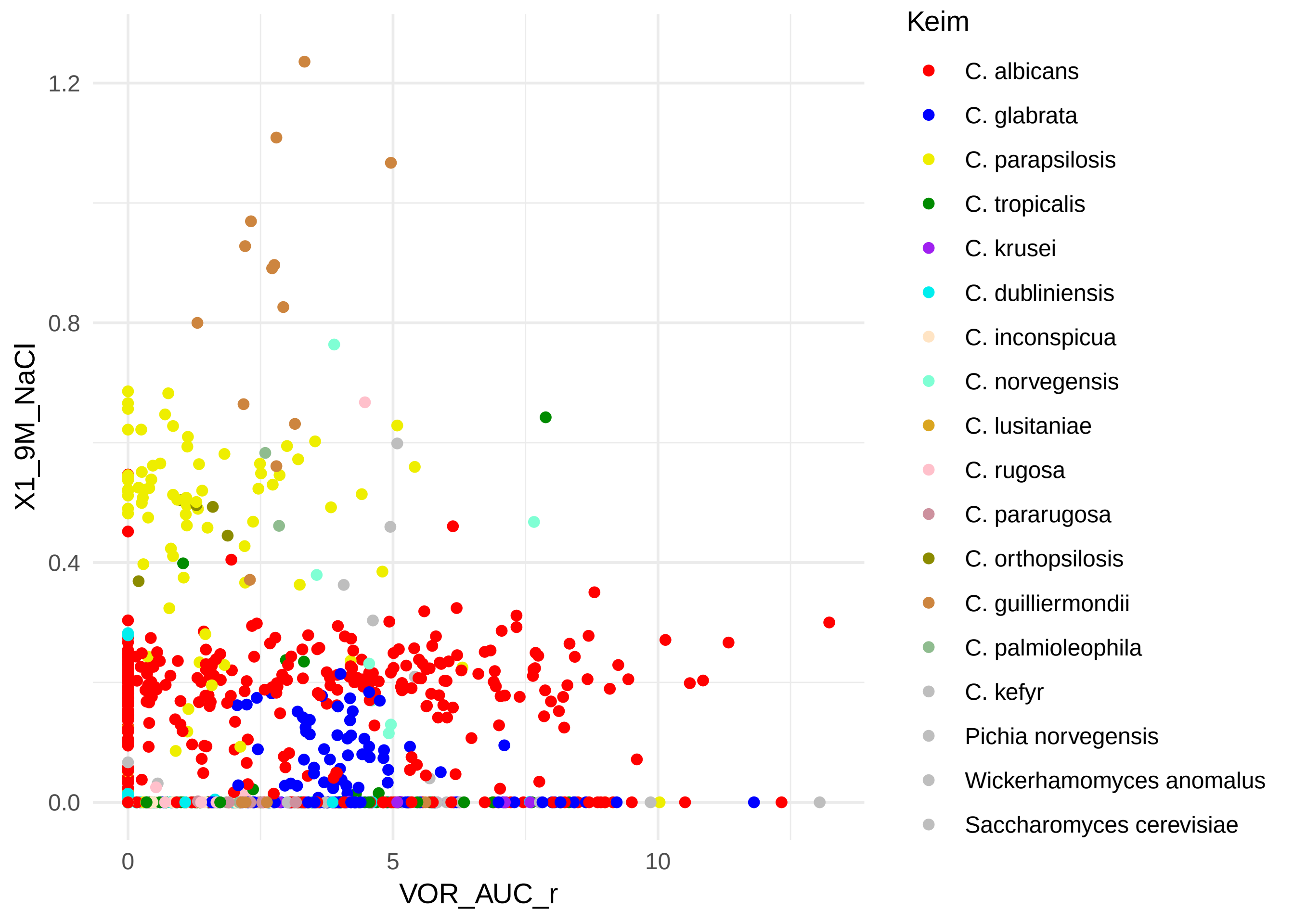

1.2
0.8
0.4
0.0
0
5
10
VOR_AUC_r
X1_9M_NaCl
Keim
C. albicans
C. glabrata
C. parapsilosis
C. tropicalis
C. krusei
C. dubliniensis
C. inconspicua
C. norvegensis
C. lusitaniae
C. rugosa
C. pararugosa
C. orthopsilosis
C. guilliermondii
C. palmioleophila
C. kefyr
Pichia norvegensis
Wickerhamomyces anomalus
Saccharomyces cerevisiae

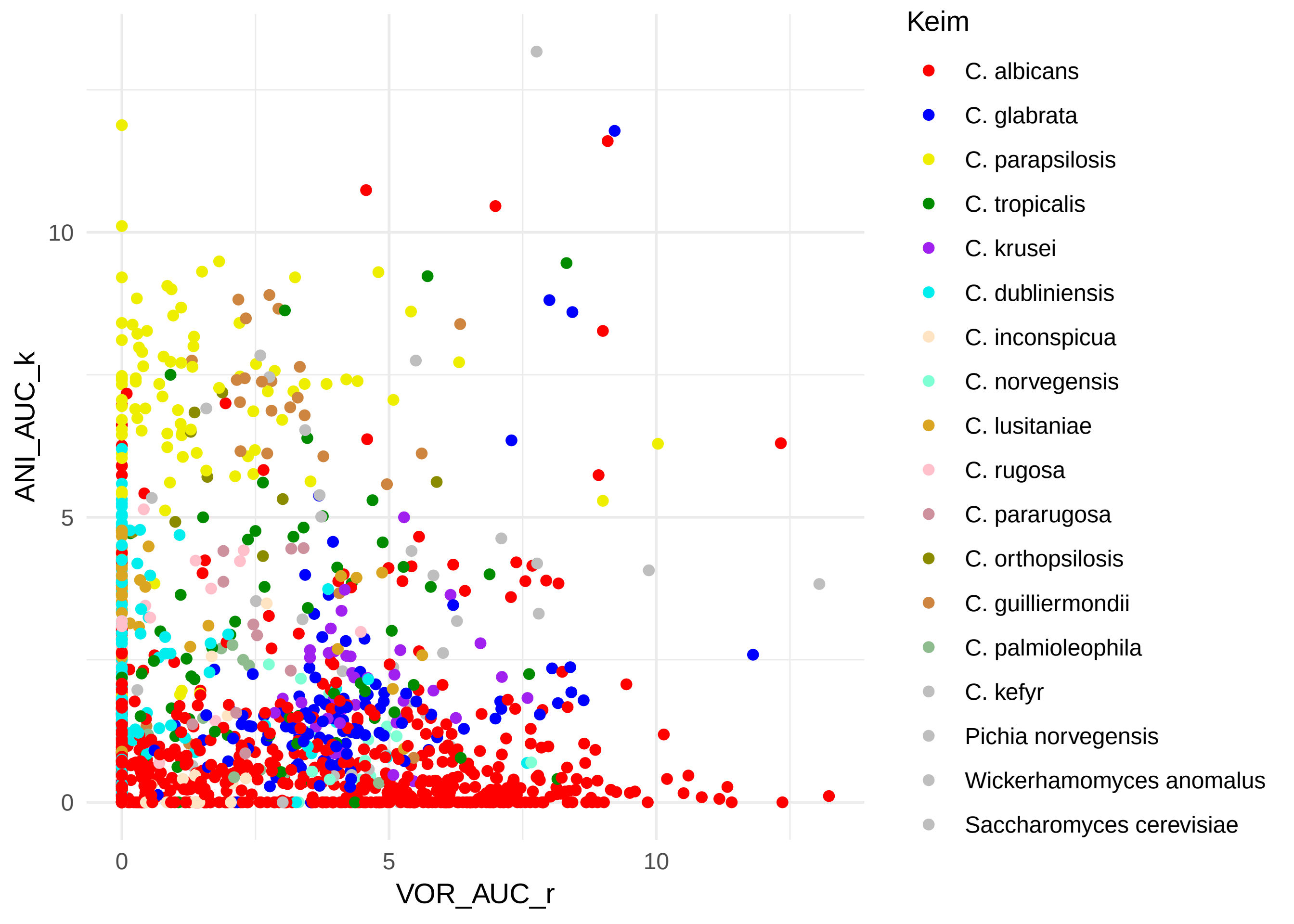

Keim
C. albicans
C. glabrata
C. parapsilosis
C. tropicalis
C. krusei
C. dubliniensis
C. inconspicua
C. norvegensis
C. lusitaniae
C. rugosa
C. pararugosa
C. orthopsilosis
C. guilliermondii
C. palmioleophila
C. kefyr
Pichia norvegensis
Wickerhamomyces anomalus
Saccharomyces cerevisiae
ANI_AUC_k
VOR_AUC_r
0
5
10
0
5
10

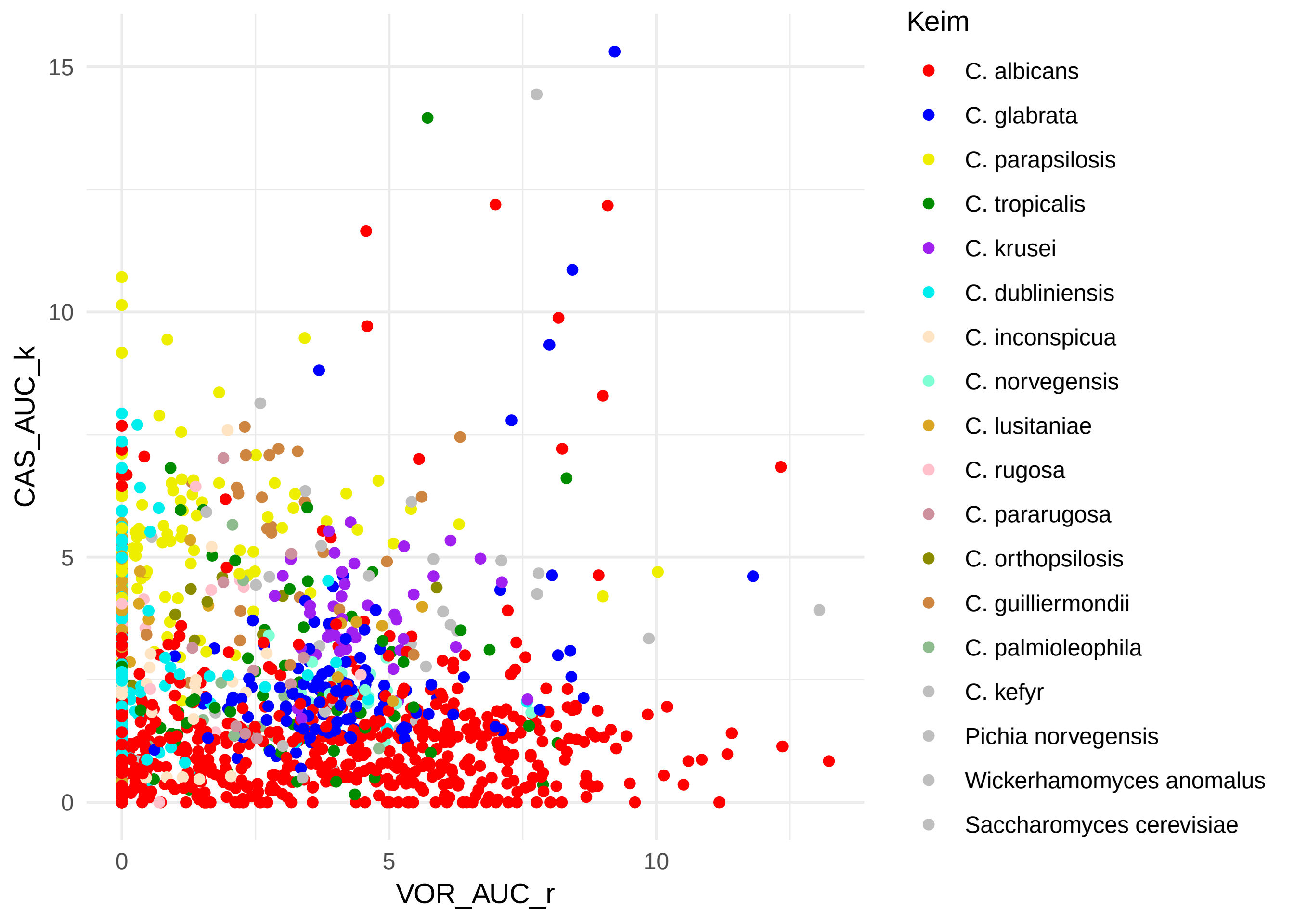

Keim
C. albicans
C. glabrata
C. parapsilosis
C. tropicalis
C. krusei
C. dubliniensis
C. inconspicua
C. norvegensis
C. lusitaniae
C. rugosa
C. pararugosa
C. orthopsilosis
C. guilliermondii
C. palmioleophila
C. kefyr
Pichia norvegensis
Wickerhamomyces anomalus
Saccharomyces cerevisiae
CAS_AUC_k
VOR_AUC_r
0
5
10
15
0
5
10

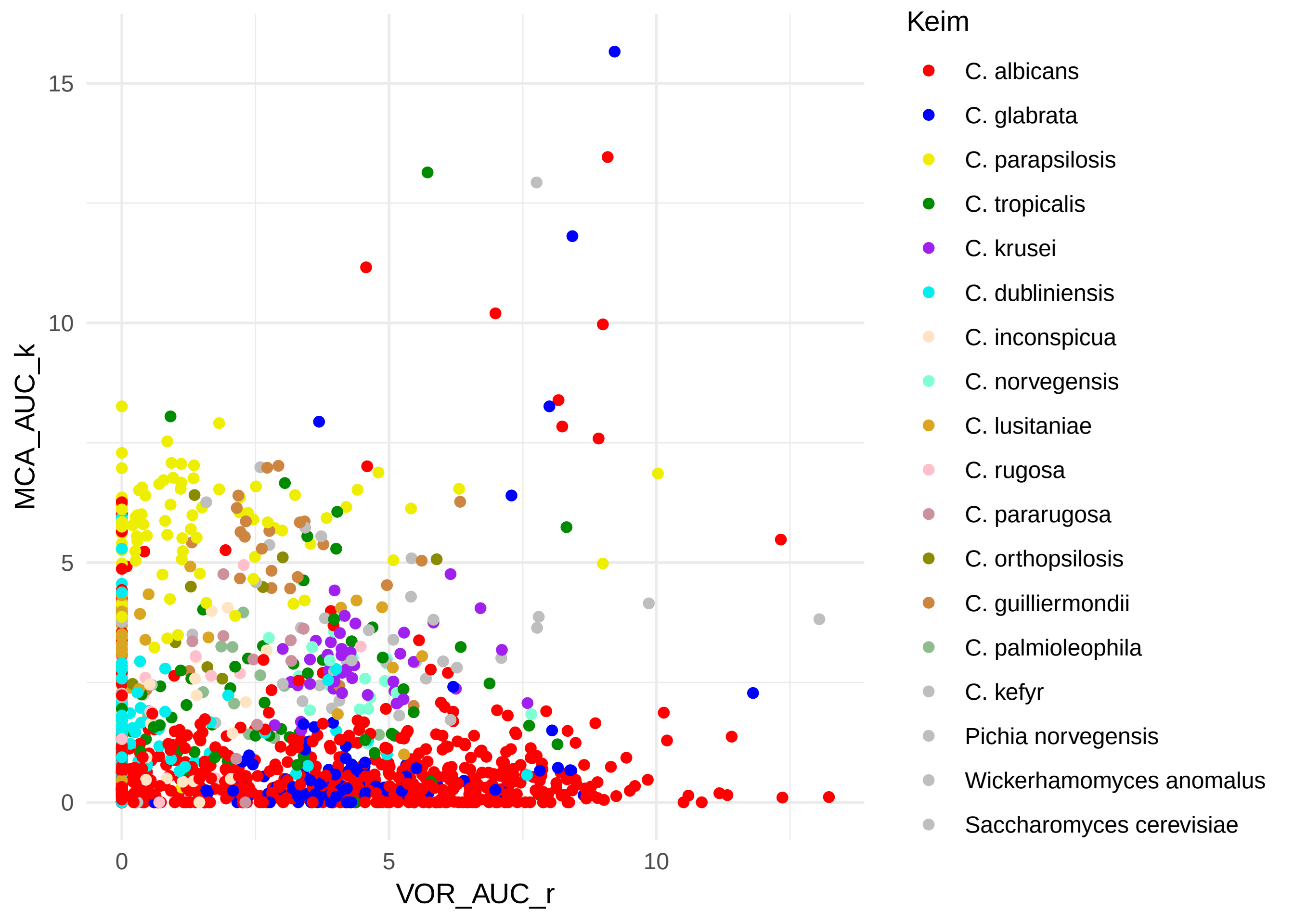

Keim
C. albicans
C. glabrata
C. parapsilosis
C. tropicalis
C. krusei
C. dubliniensis
C. inconspicua
C. norvegensis
C. lusitaniae
C. rugosa
C. pararugosa
C. orthopsilosis
C. guilliermondii
C. palmioleophila
C. kefyr
Pichia norvegensis
Wickerhamomyces anomalus
Saccharomyces cerevisiae
MCA_AUC_k
VOR_AUC_r
0
5
10
15

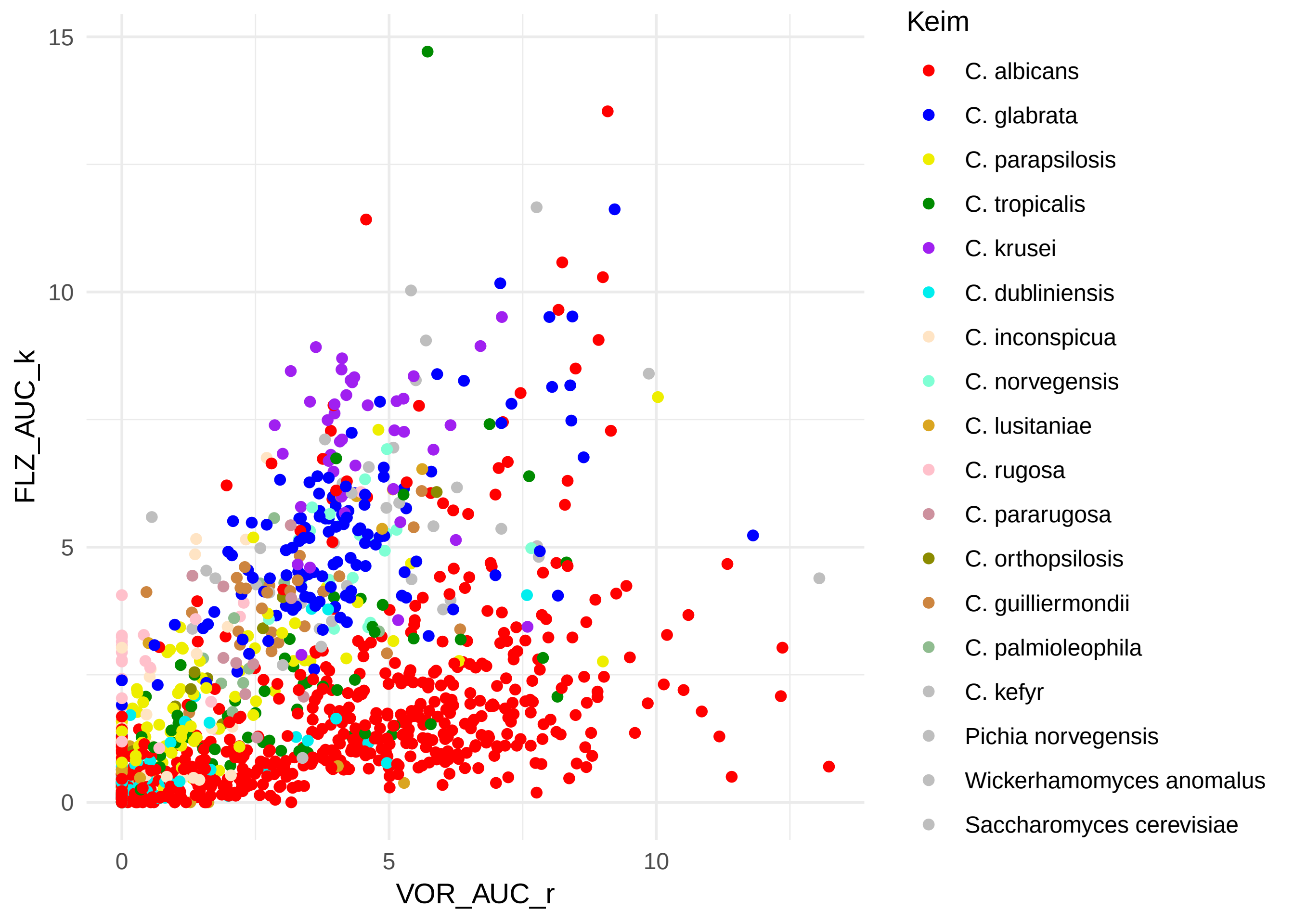

Keim
C. albicans
C. glabrata
C. parapsilosis
C. tropicalis
C. krusei
C. dubliniensis
C. inconspicua
C. norvegensis
C. lusitaniae
C. rugosa
C. pararugosa
C. orthopsilosis
C. guilliermondii
C. palmioleophila
C. kefyr
Pichia norvegensis
Wickerhamomyces anomalus
Saccharomyces cerevisiae
FLZ_AUC_k
VOR_AUC_r
15
10
5
0
0
5
10

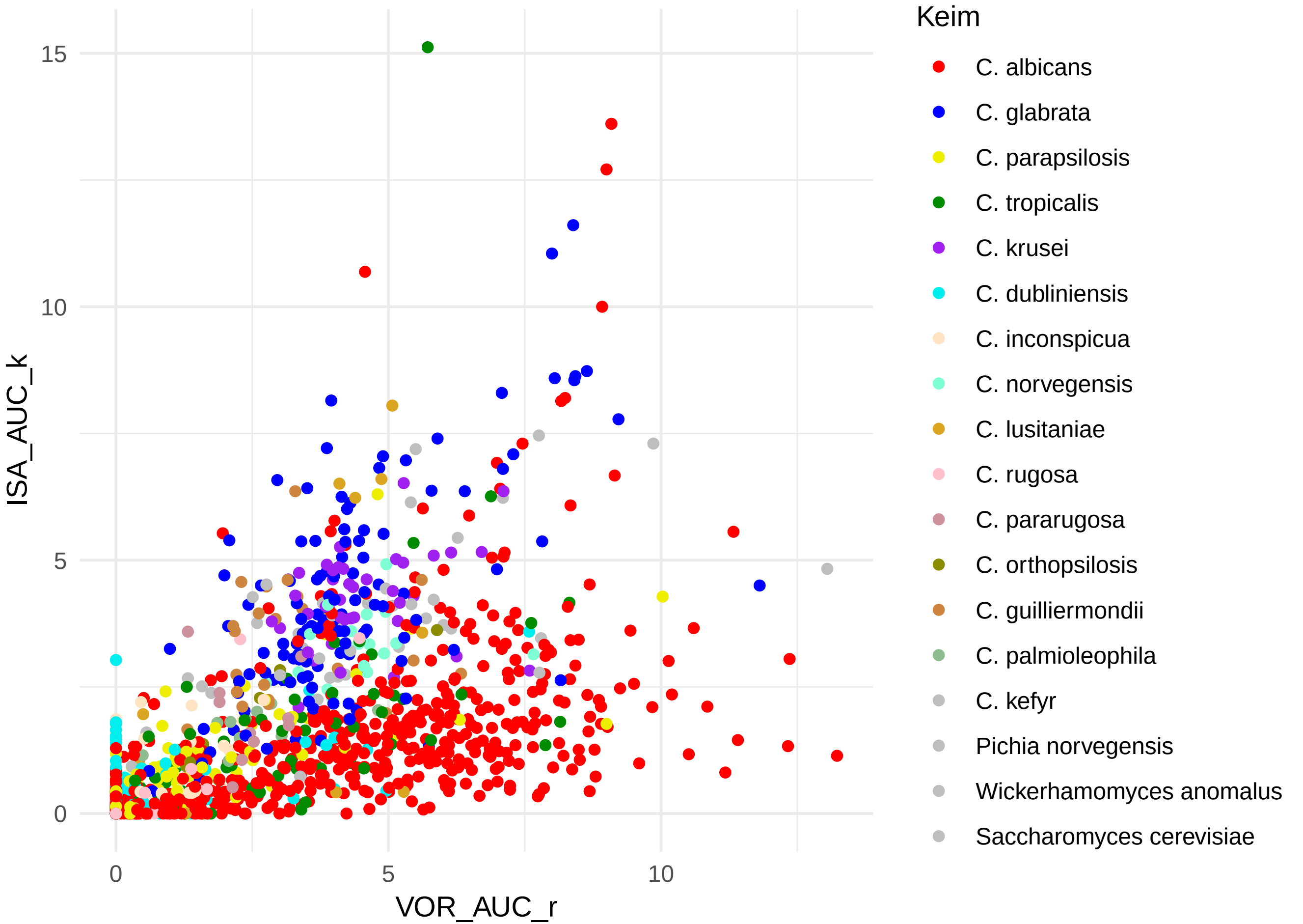

Keim
C. albicans
C. glabrata
C. parapsilosis
C. tropicalis
C. krusei
C. dubliniensis
C. inconspicua
C. norvegensis
C. lusitaniae
C. rugosa
C. pararugosa
C. orthopsilosis
C. guilliermondii
C. palmioleophila
C. kefyr
Pichia norvegensis
Wickerhamomyces anomalus
Saccharomyces cerevisiae
ISA_AUC_k
VOR_AUC_r
0
5
10
15

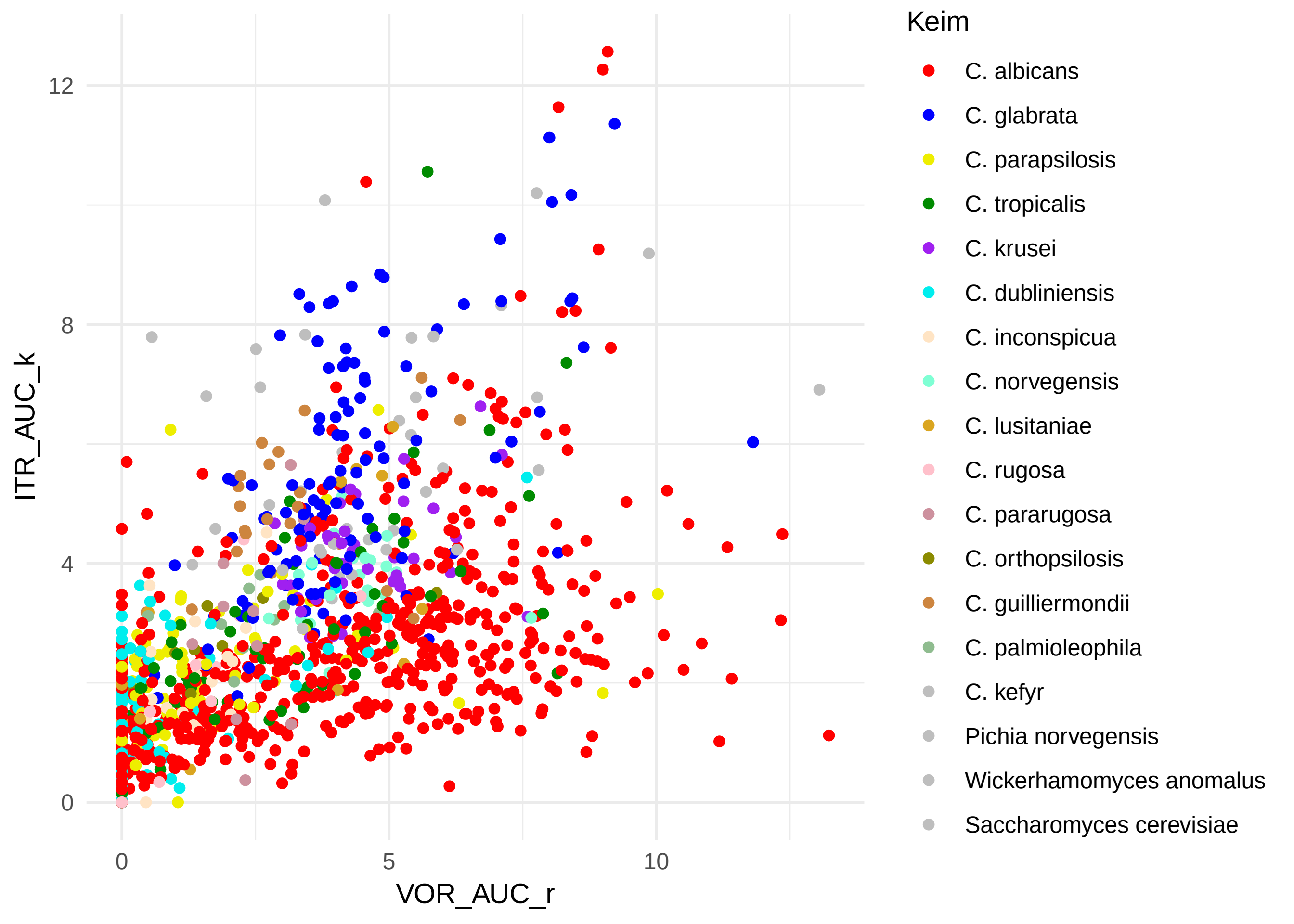
ITR_AUC_k
VOR_AUC_r
0
4
8
12
0
5
10
Keim
C. albicans
C. glabrata
C. parapsilosis
C. tropicalis
C. krusei
C. dubliniensis
C. inconspicua
C. norvegensis
C. lusitaniae
C. rugosa
C. pararugosa
C. orthopsilosis
C. guilliermondii
C. palmioleophila
C. kefyr
Pichia norvegensis
Wickerhamomyces anomalus
Saccharomyces cerevisiae

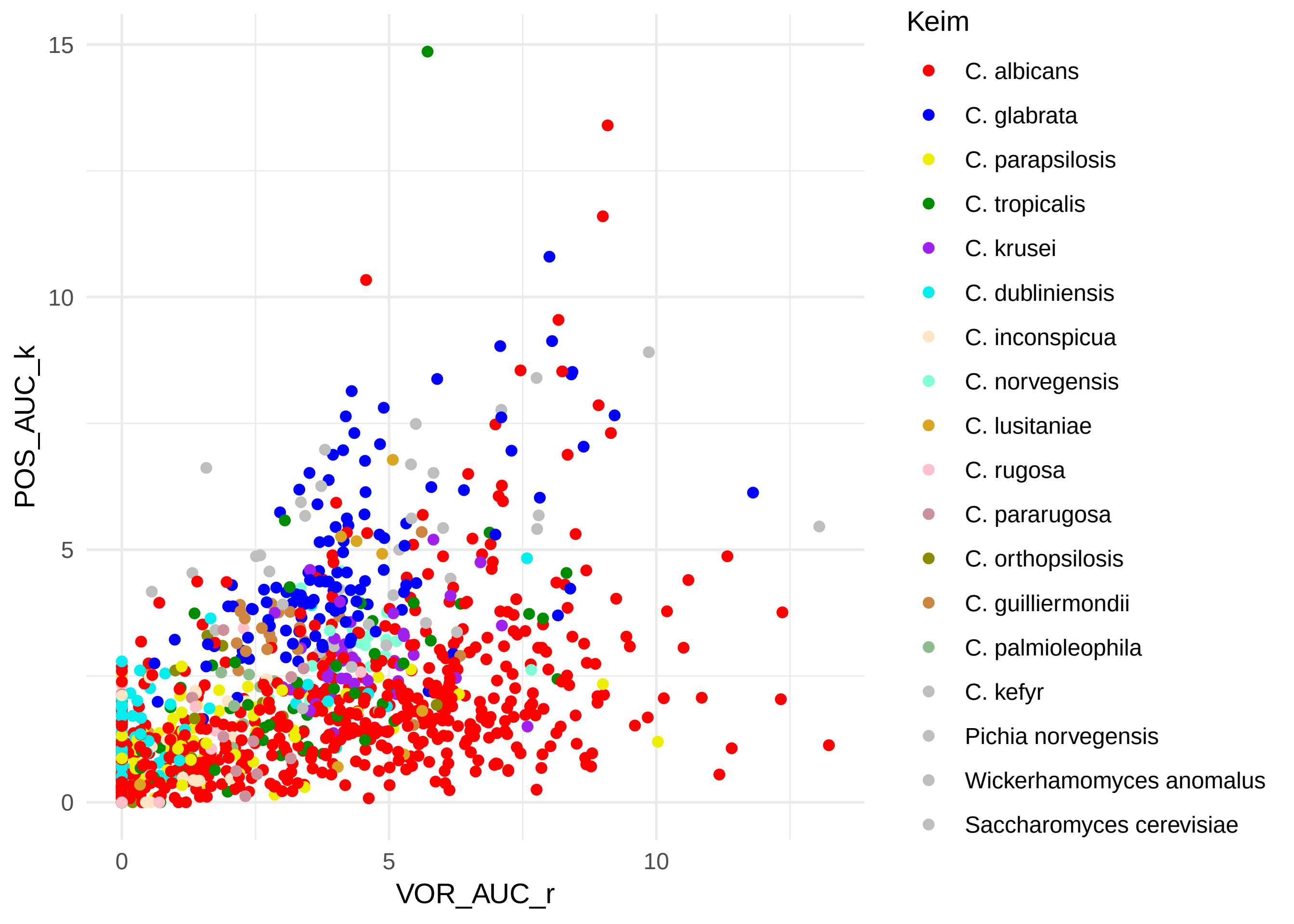

Keim
C. albicans
C. glabrata
C. parapsilosis
C. tropicalis
C. krusei
C. dubliniensis
C. inconspicua
C. norvegensis
C. lusitaniae
C. rugosa
C. pararugosa
C. orthopsilosis
C. guilliermondii
C. palmioleophila
C. kefyr
Pichia norvegensis
Wickerhamomyces anomalus
Saccharomyces cerevisiae
POS_AUC_k
VOR_AUC_r
0
5
10
15

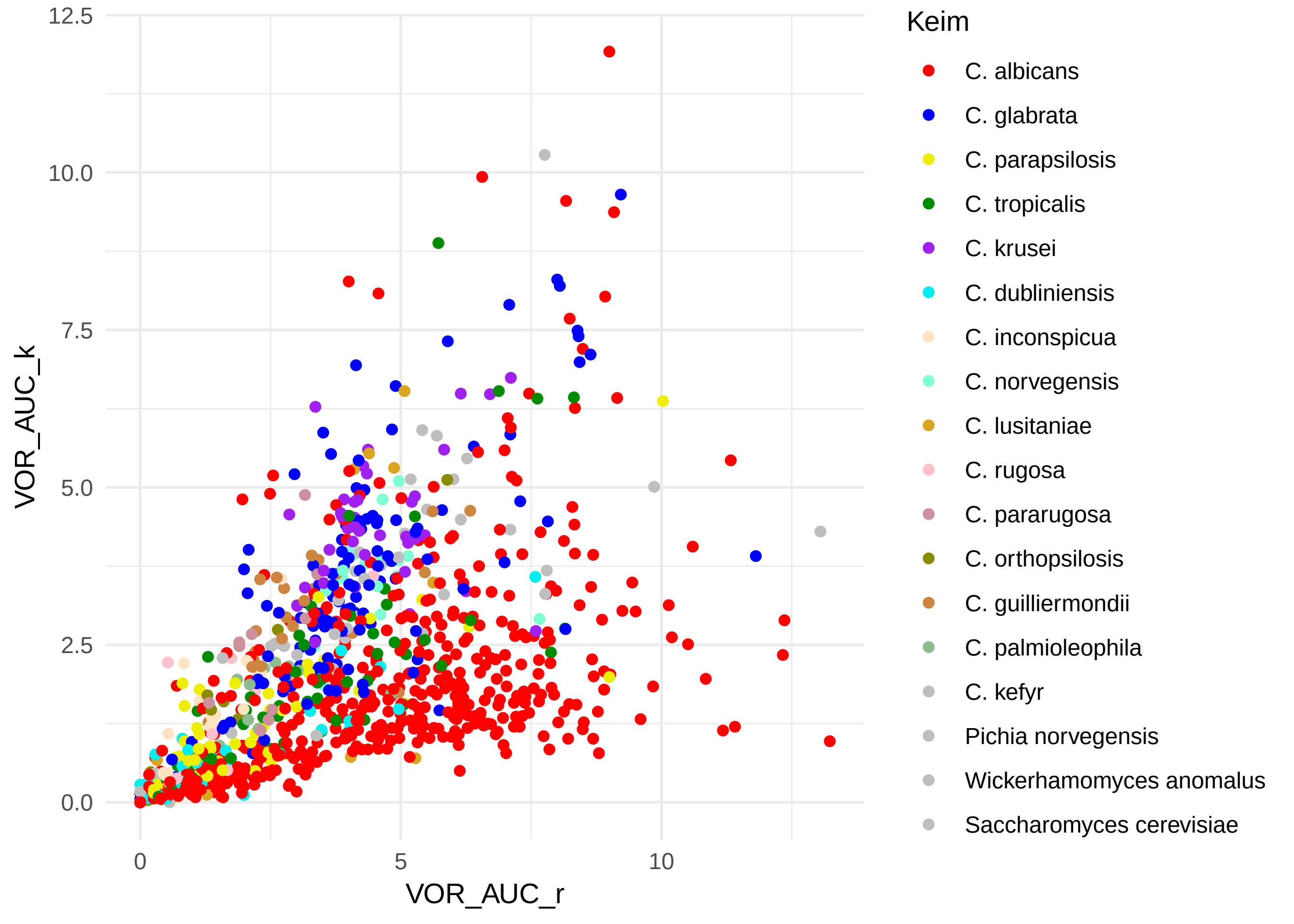
Keim
C. albicans
C. glabrata
C. parapsilosis
C. tropicalis
C. krusei
C. dubliniensis
C. inconspicua
C. norvegensis
C. lusitaniae
C. rugosa
C. pararugosa
C. orthopsilosis
C. guilliermondii
C. palmioleophila
C. kefyr
Pichia norvegensis
Wickerhamomyces anomalus
Saccharomyces cerevisiae
VOR_AUC_k
12.5
10.0
7.5
5.0
2.5
0.0
VOR_AUC_r
0
5
10

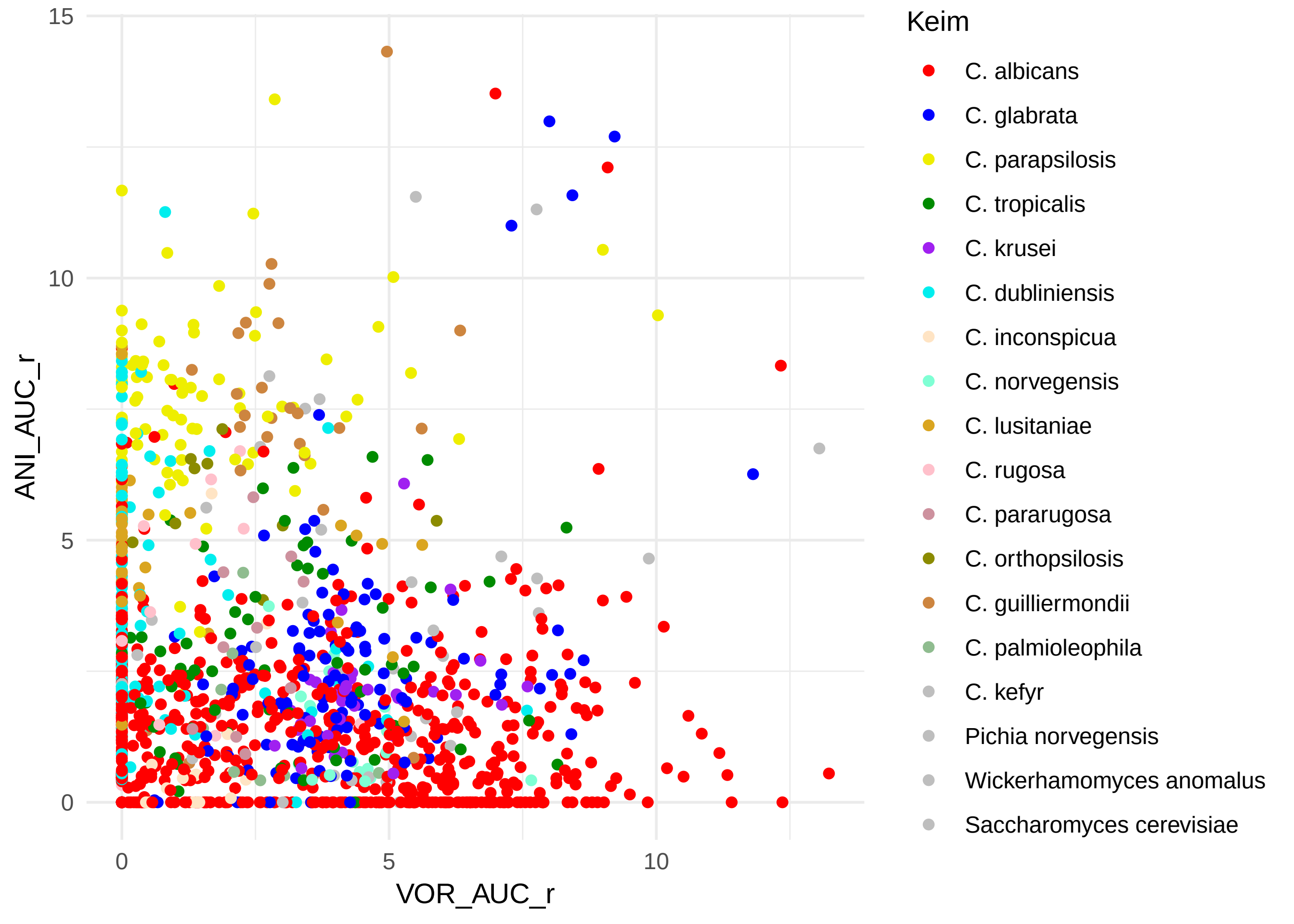

15
10
5
0
ANI_AUC_r
0
5
10
VOR_AUC_r
Keim
C. albicans
C. glabrata
C. parapsilosis
C. tropicalis
C. krusei
C. dubliniensis
C. inconspicua
C. norvegensis
C. lusitaniae
C. rugosa
C. pararugosa
C. orthopsilosis
C. guilliermondii
C. palmioleophila
C. kefyr
Pichia norvegensis
Wickerhamomyces anomalus
Saccharomyces cerevisiae

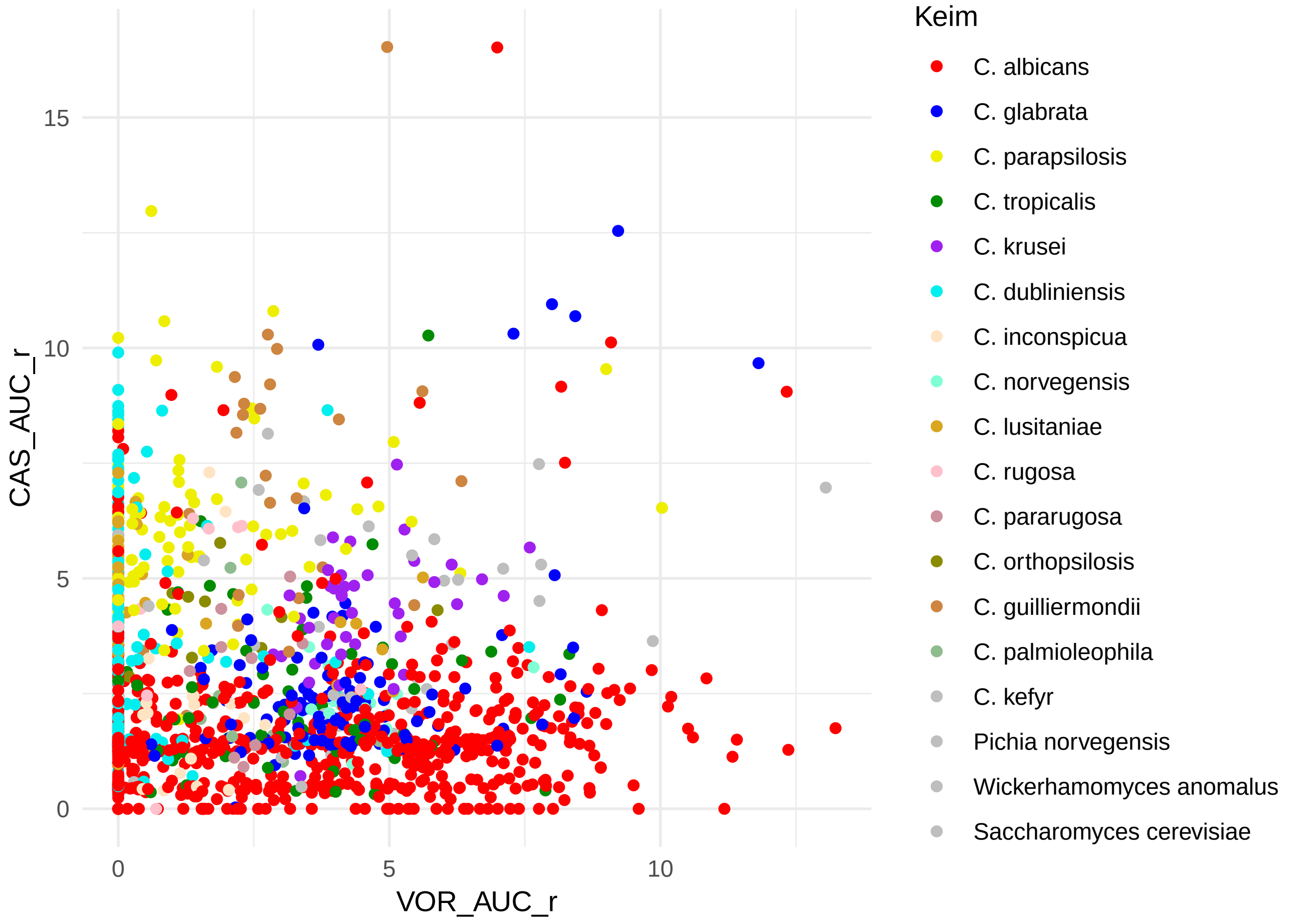

Keim
C. albicans
C. glabrata
C. parapsilosis
C. tropicalis
C. krusei
C. dubliniensis
C. inconspicua
C. norvegensis
C. lusitaniae
C. rugosa
C. pararugosa
C. orthopsilosis
C. guilliermondii
C. palmioleophila
C. kefyr
Pichia norvegensis
Wickerhamomyces anomalus
Saccharomyces cerevisiae
CAS_AUC_r
VOR_AUC_r
0
5
10
15

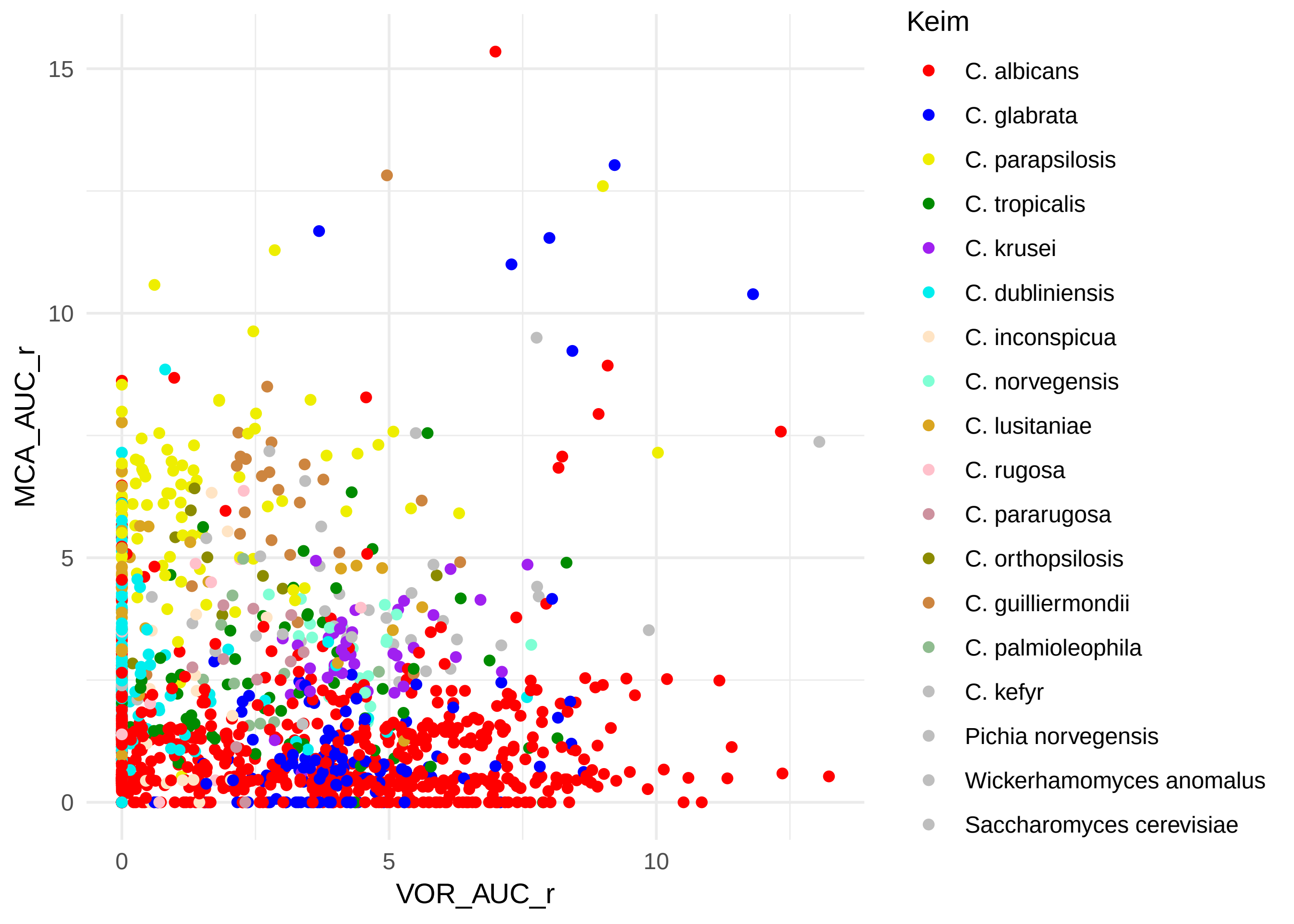
Keim
C. albicans
C. glabrata
C. parapsilosis
C. tropicalis
C. krusei
C. dubliniensis
C. inconspicua
C. norvegensis
C. lusitaniae
C. rugosa
C. pararugosa
C. orthopsilosis
C. guilliermondii
C. palmioleophila
C. kefyr
Pichia norvegensis
Wickerhamomyces anomalus
Saccharomyces cerevisiae
MCA_AUC_r
VOR_AUC_r
0
5
10
15
0
5
10

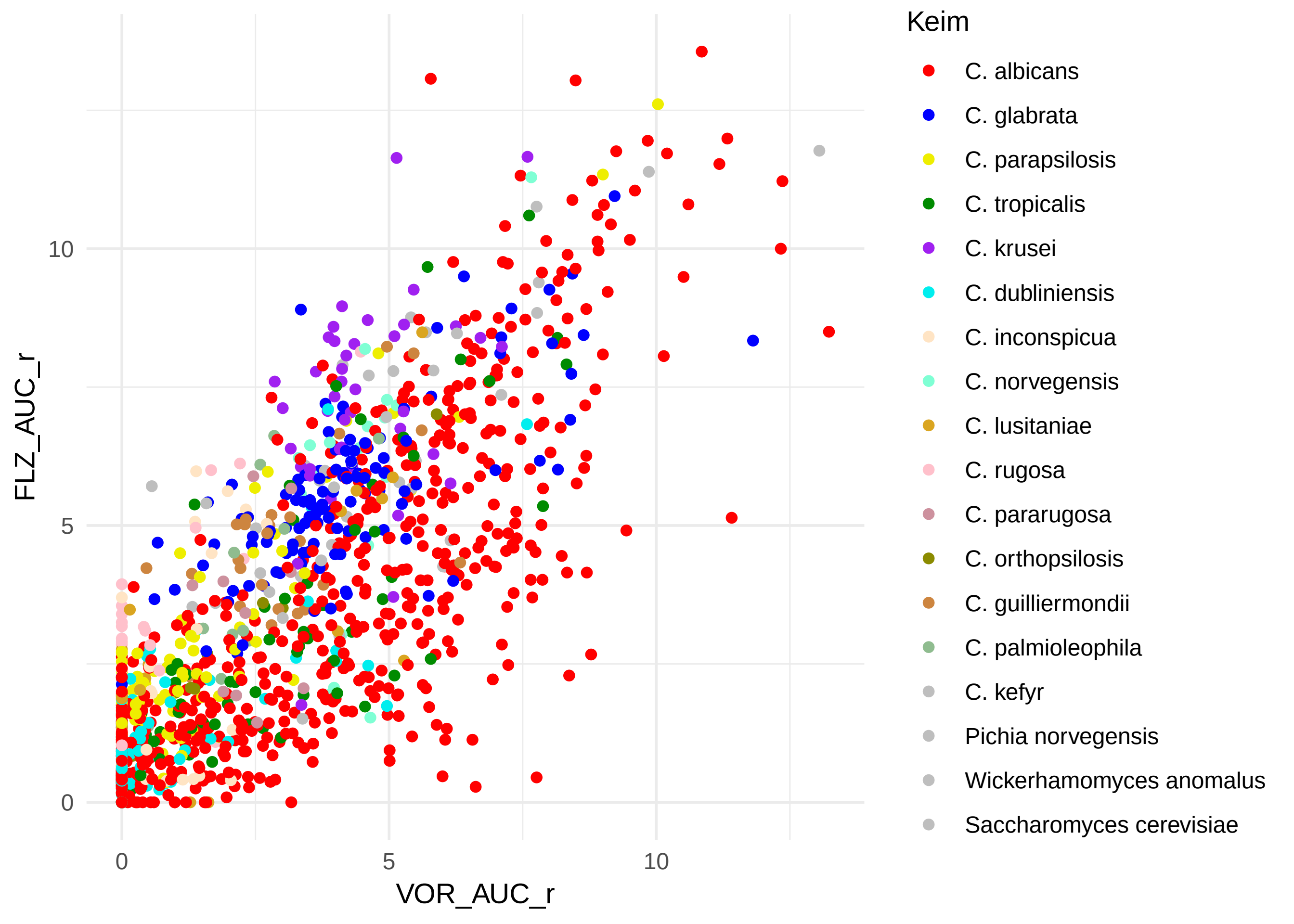

Keim
C. albicans
C. glabrata
C. parapsilosis
C. tropicalis
C. krusei
C. dubliniensis
C. inconspicua
C. norvegensis
C. lusitaniae
C. rugosa
C. pararugosa
C. orthopsilosis
C. guilliermondii
C. palmioleophila
C. kefyr
Pichia norvegensis
Wickerhamomyces anomalus
Saccharomyces cerevisiae
FLZ_AUC_r
VOR_AUC_r
0
5
10
0
5
10

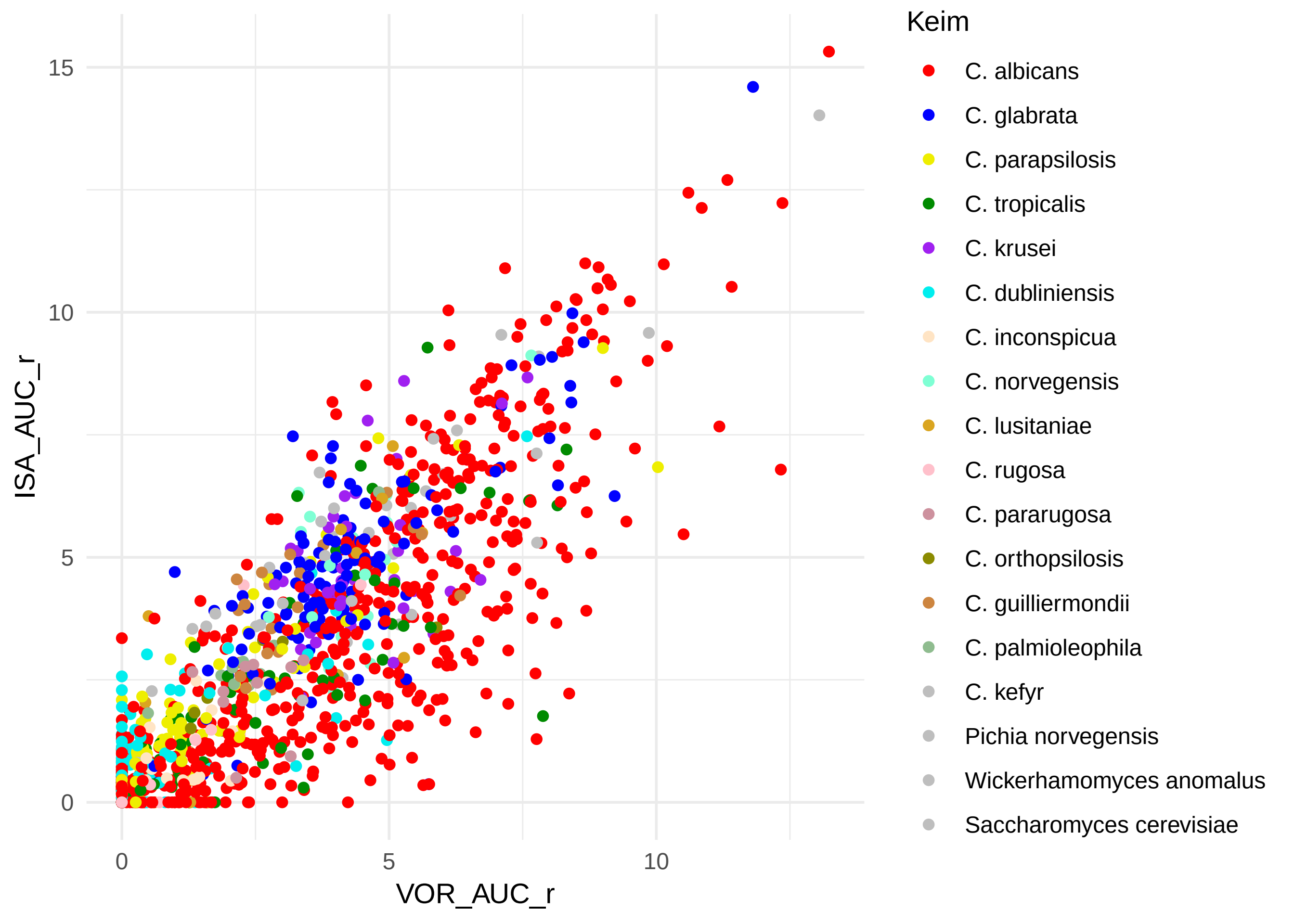

Keim
C. albicans
C. glabrata
C. parapsilosis
C. tropicalis
C. krusei
C. dubliniensis
C. inconspicua
C. norvegensis
C. lusitaniae
C. rugosa
C. pararugosa
C. orthopsilosis
C. guilliermondii
C. palmioleophila
C. kefyr
Pichia norvegensis
Wickerhamomyces anomalus
Saccharomyces cerevisiae
ISA_AUC_r
VOR_AUC_r
0
5
10
15
0
5
10

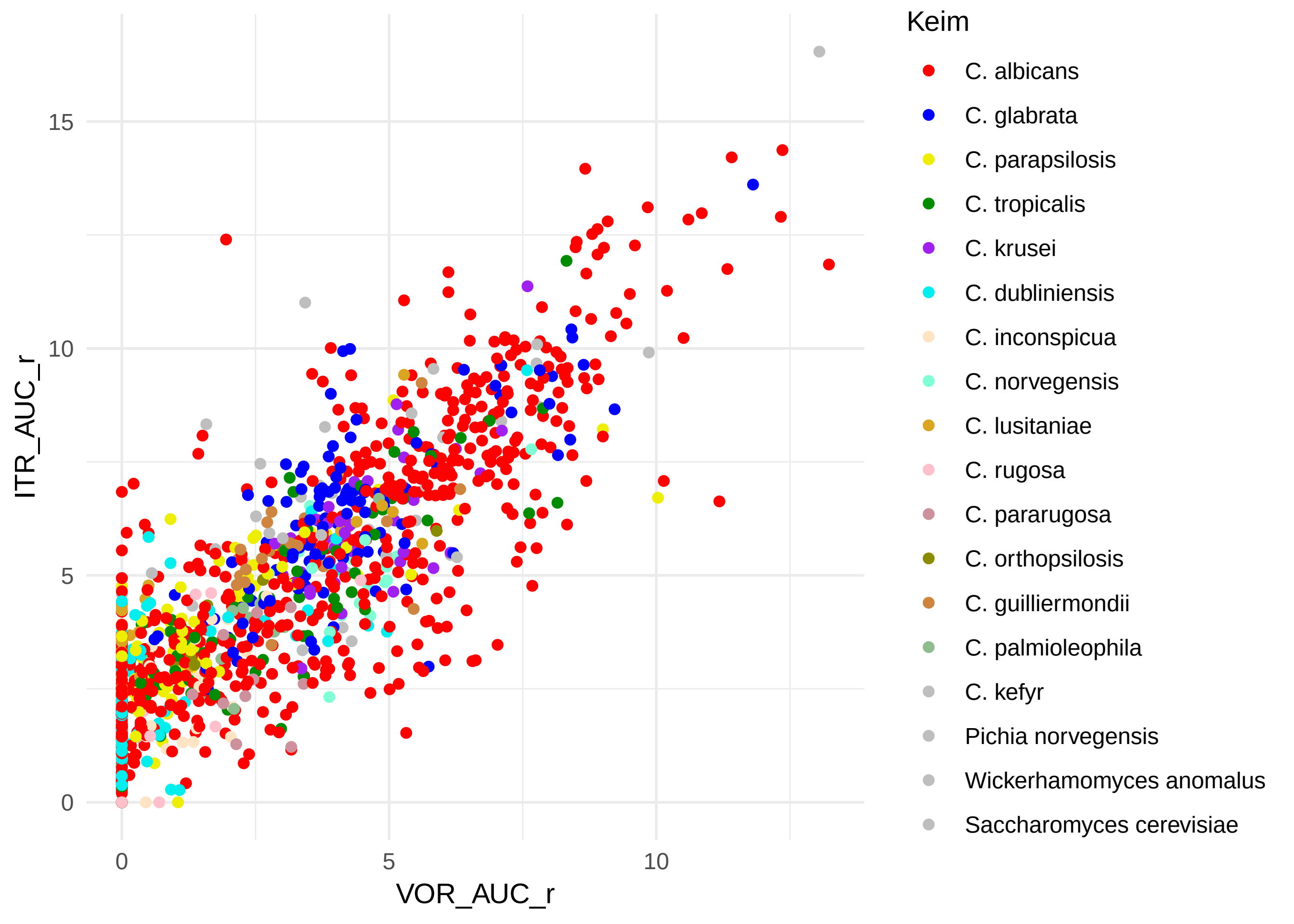

Keim
C. albicans
C. glabrata
C. parapsilosis
C. tropicalis
C. krusei
C. dubliniensis
C. inconspicua
C. norvegensis
C. lusitaniae
C. rugosa
C. pararugosa
C. orthopsilosis
C. guilliermondii
C. palmioleophila
C. kefyr
Pichia norvegensis
Wickerhamomyces anomalus
Saccharomyces cerevisiae
ITR_AUC_r
VOR_AUC_r
0
5
10
15
0
5
10

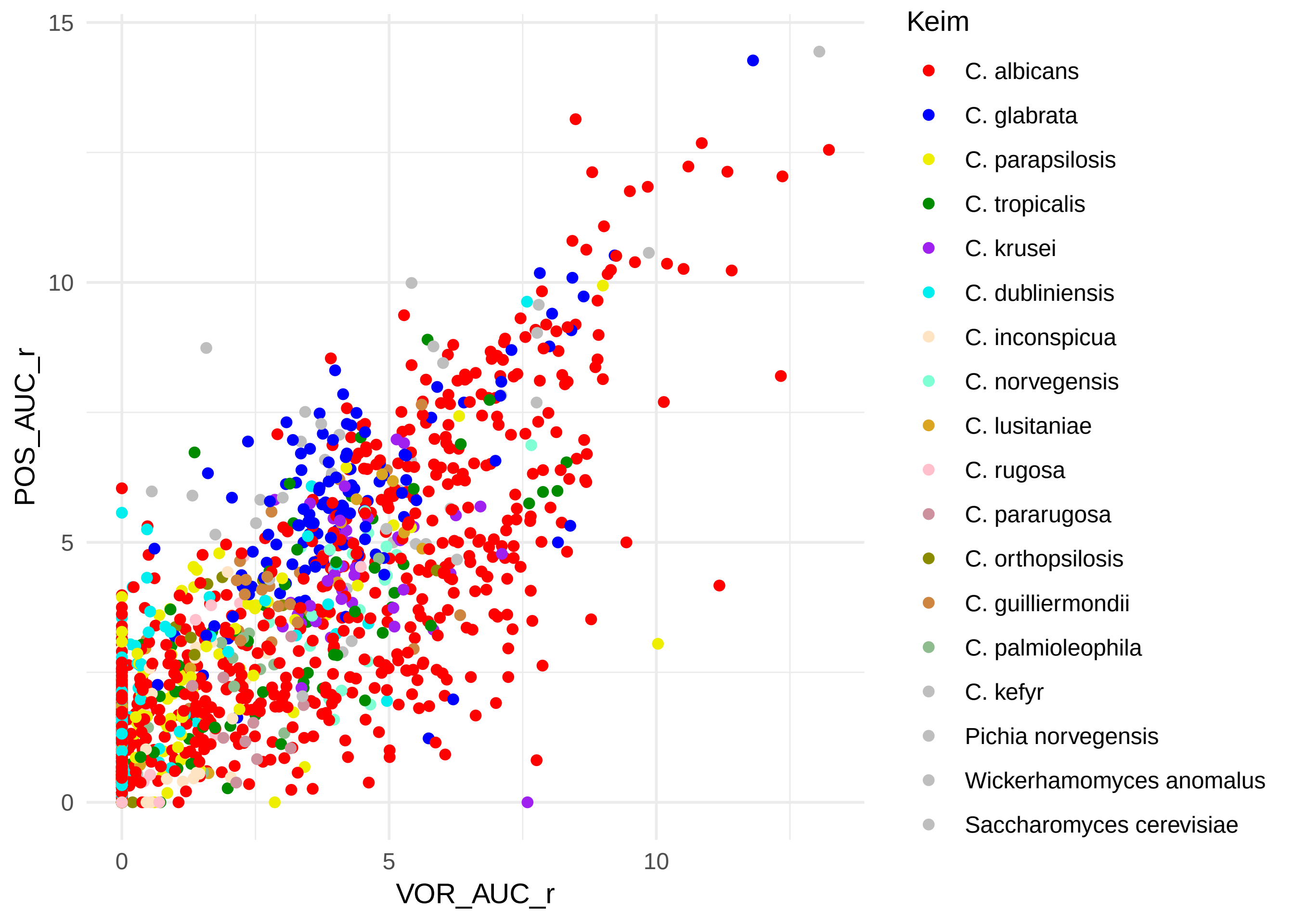
Keim
C. albicans
C. glabrata
C. parapsilosis
C. tropicalis
C. krusei
C. dubliniensis
C. inconspicua
C. norvegensis
C. lusitaniae
C. rugosa
C. pararugosa
C. orthopsilosis
C. guilliermondii
C. palmioleophila
C. kefyr
Pichia norvegensis
Wickerhamomyces anomalus
Saccharomyces cerevisiae
POS_AUC_r
VOR_AUC_r
0
5
10
15

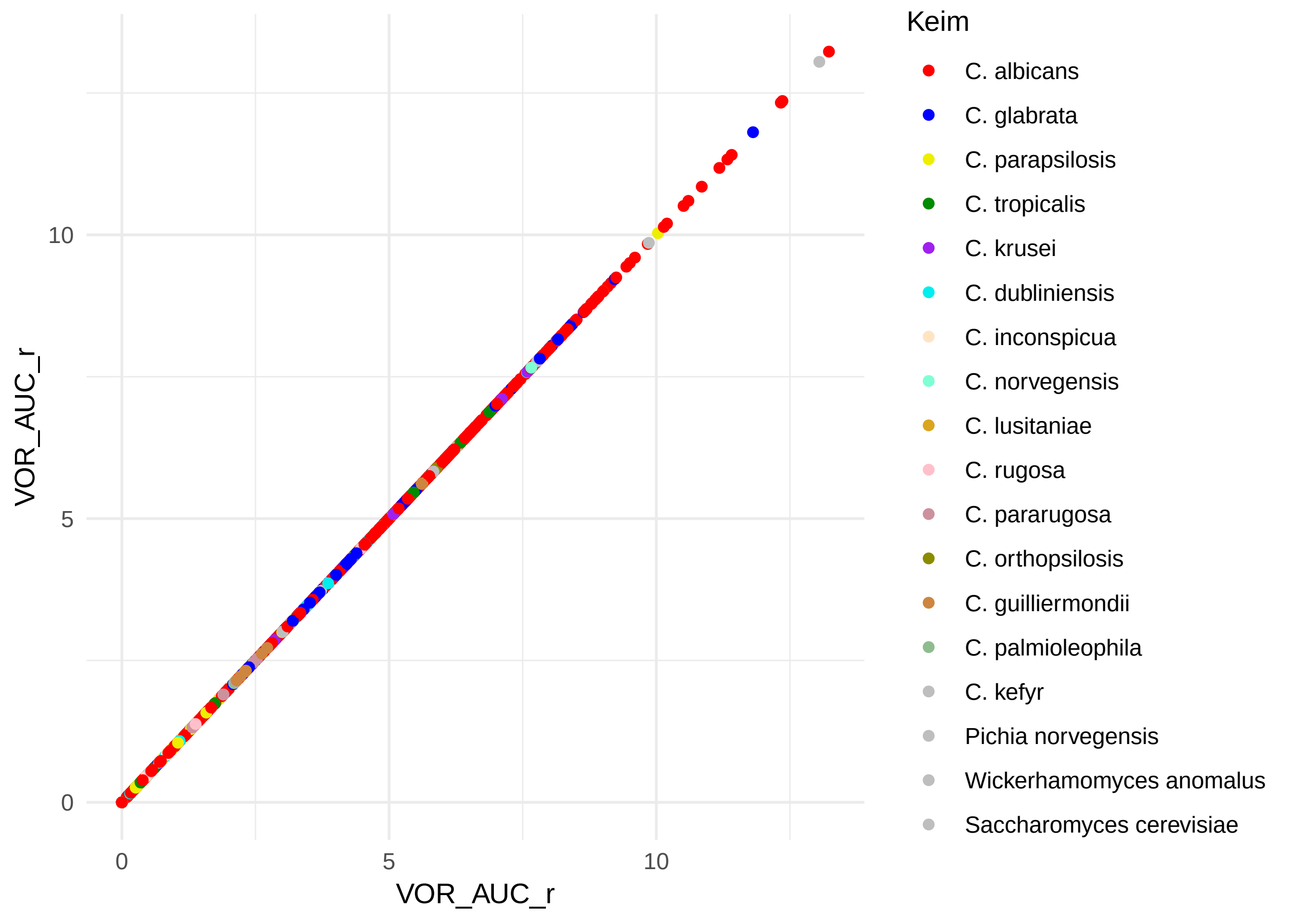

Keim
C. albicans
C. glabrata
C. parapsilosis
C. tropicalis
C. krusei
C. dubliniensis
C. inconspicua
C. norvegensis
C. lusitaniae
C. rugosa
C. pararugosa
C. orthopsilosis
C. guilliermondii
C. palmioleophila
C. kefyr
Pichia norvegensis
Wickerhamomyces anomalus
Saccharomyces cerevisiae
VOR_AUC_r
VOR_AUC_r
0
5
10
0
5
10

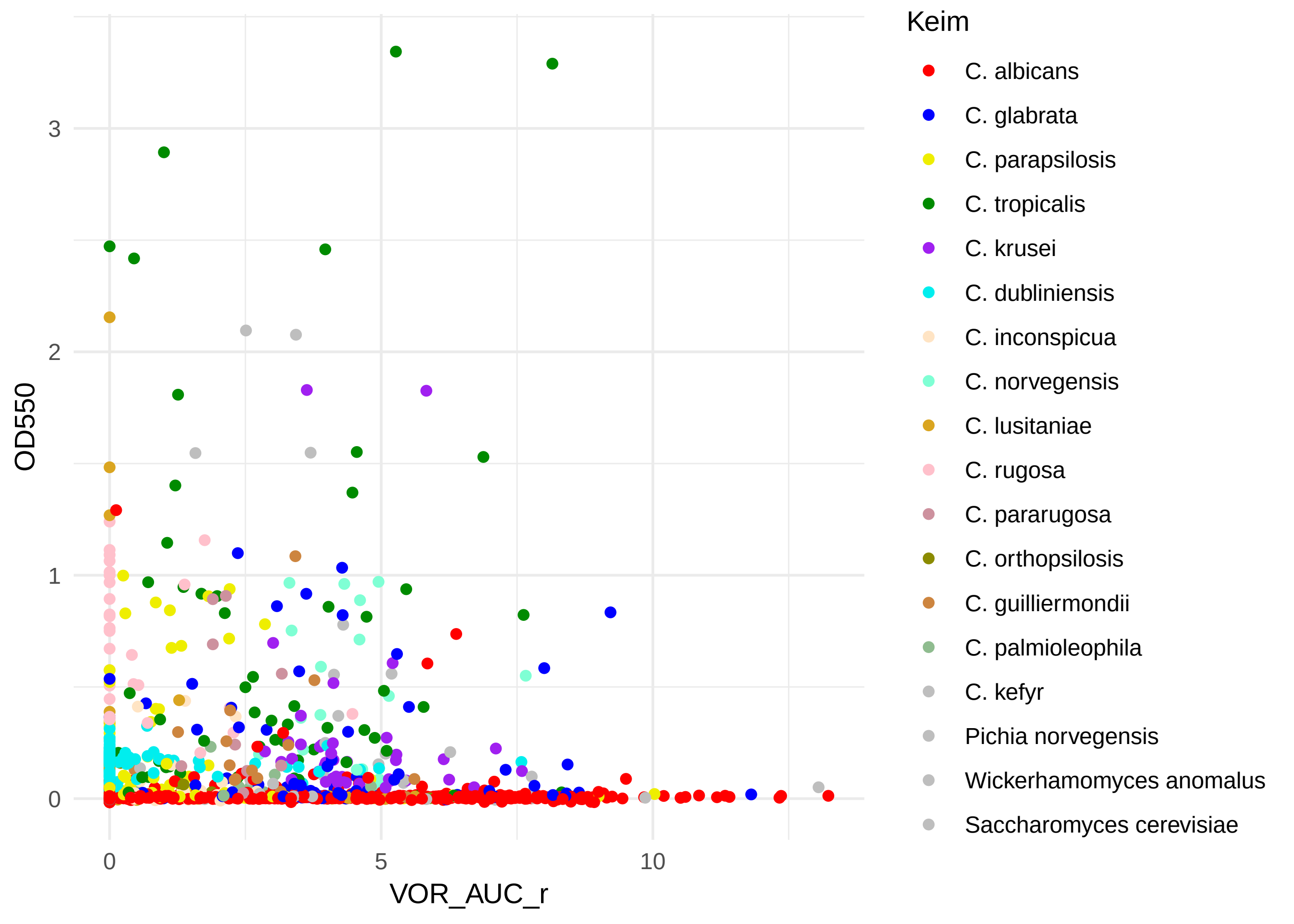
Keim
C. albicans
C. glabrata
C. parapsilosis
C. tropicalis
C. krusei
C. dubliniensis
C. inconspicua
C. norvegensis
C. lusitaniae
C. rugosa
C. pararugosa
C. orthopsilosis
C. guilliermondii
C. palmioleophila
C. kefyr
Pichia norvegensis
Wickerhamomyces anomalus
Saccharomyces cerevisiae
OD550
0
1
2
3
0
5
10
VOR_AUC_r

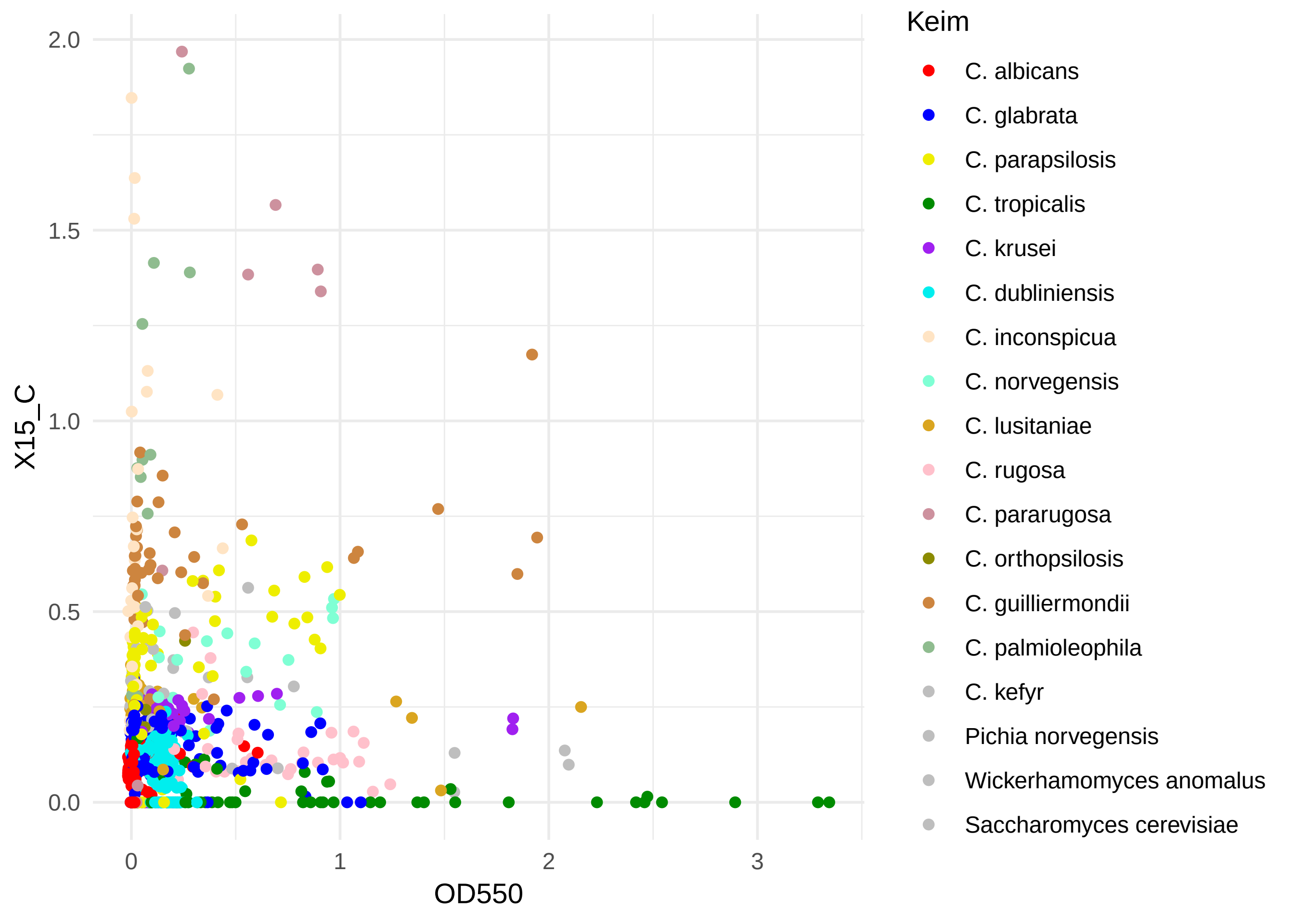

Keim
C. albicans
C. glabrata
C. parapsilosis
C. tropicalis
C. krusei
C. dubliniensis
C. inconspicua
C. norvegensis
C. lusitaniae
C. rugosa
C. pararugosa
C. orthopsilosis
C. guilliermondii
C. palmioleophila
C. kefyr
Pichia norvegensis
Wickerhamomyces anomalus
Saccharomyces cerevisiae
X15_C
OD550
2.0
1.5
1.0
0.5
0.0
0
1
2
3

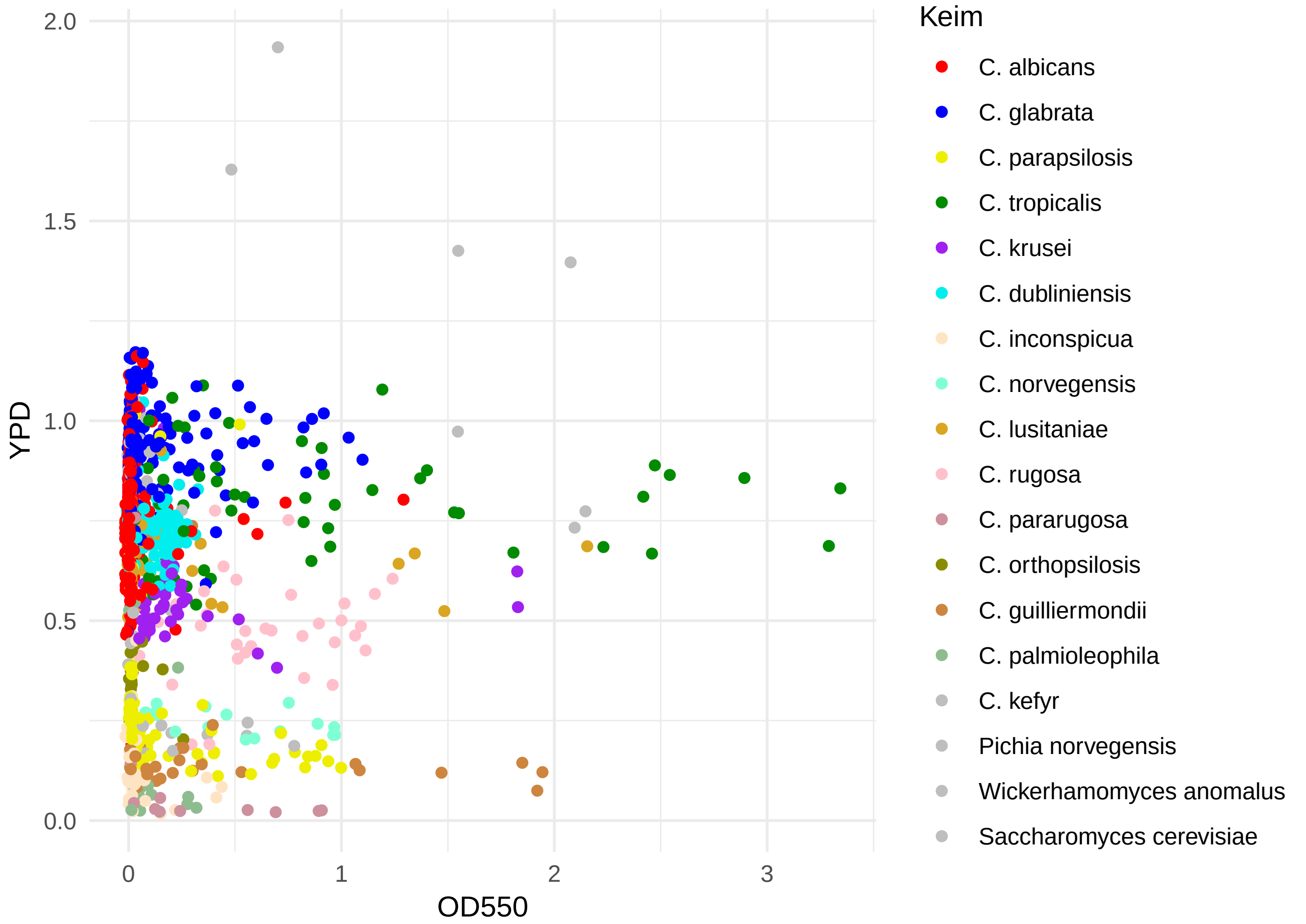
Keim
C. albicans
C. glabrata
C. parapsilosis
C. tropicalis
C. krusei
C. dubliniensis
C. inconspicua
C. norvegensis
C. lusitaniae
C. rugosa
C. pararugosa
C. orthopsilosis
C. guilliermondii
C. palmioleophila
C. kefyr
Pichia norvegensis
Wickerhamomyces anomalus
Saccharomyces cerevisiae
YPD
2.0
1.5
1.0
0.5
0.0
0
1
2
3
OD550

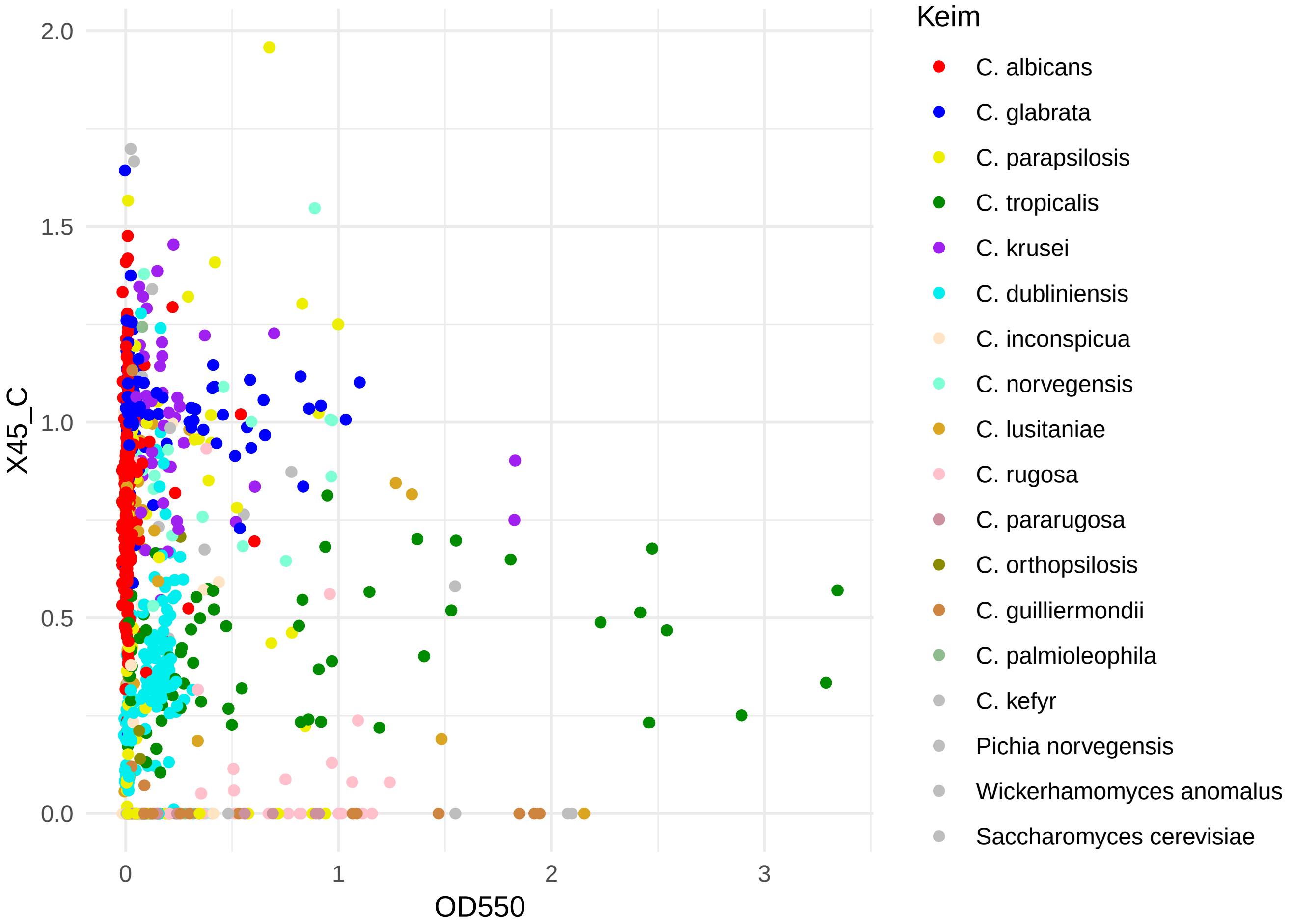

Keim
C. albicans
C. glabrata
C. parapsilosis
C. tropicalis
C. krusei
C. dubliniensis
C. inconspicua
C. norvegensis
C. lusitaniae
C. rugosa
C. pararugosa
C. orthopsilosis
C. guilliermondii
C. palmioleophila
C. kefyr
Pichia norvegensis
Wickerhamomyces anomalus
Saccharomyces cerevisiae
X45_C
OD550
2.0
1.5
1.0
0.5
0.0
0
1
2
3

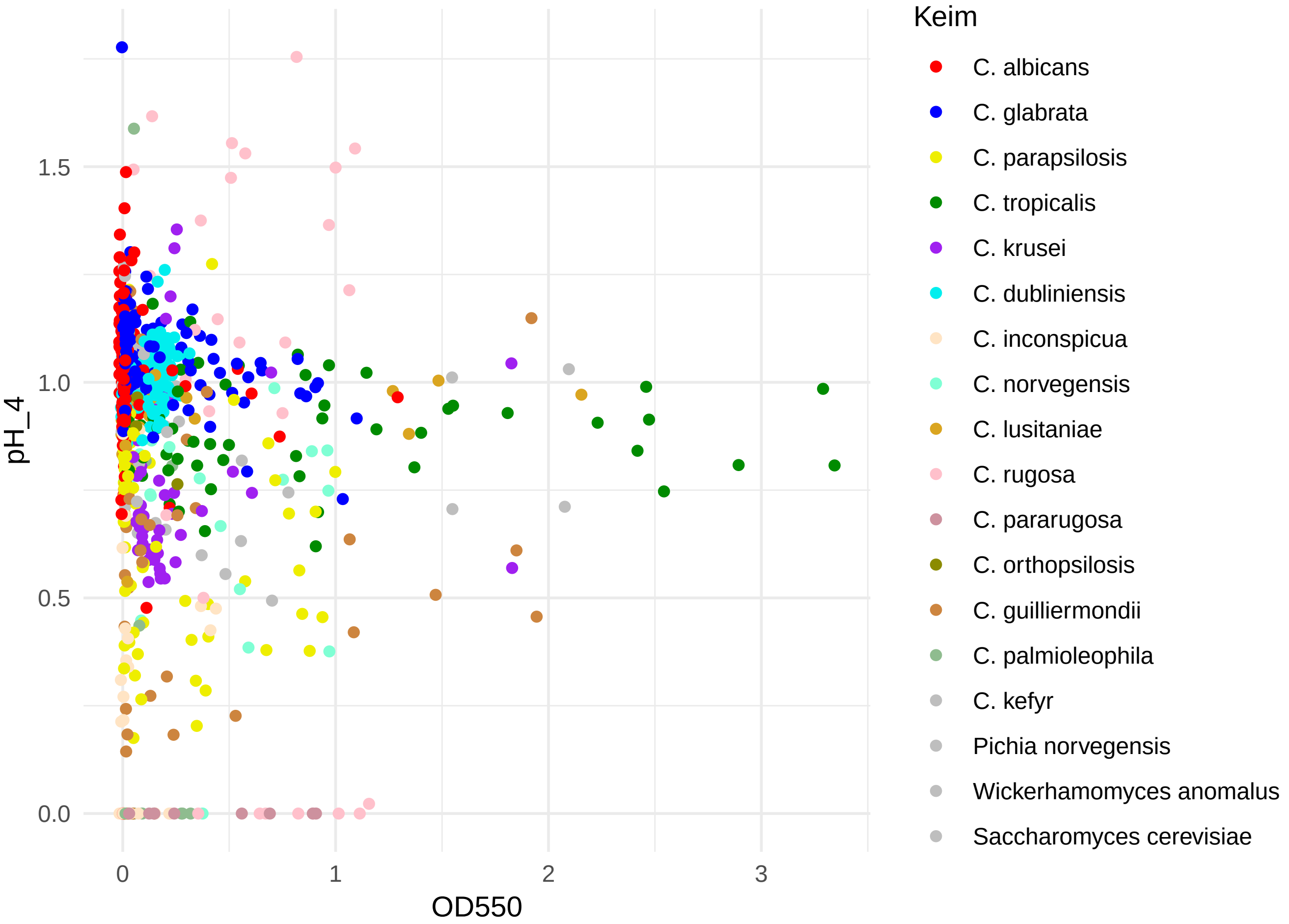
Keim
C. albicans
C. glabrata
C. parapsilosis
C. tropicalis
C. krusei
C. dubliniensis
C. inconspicua
C. norvegensis
C. lusitaniae
C. rugosa
C. pararugosa
C. orthopsilosis
C. guilliermondii
C. palmioleophila
C. kefyr
Pichia norvegensis
Wickerhamomyces anomalus
Saccharomyces cerevisiae
pH_4
1.5
1.0
0.5
0.0
0
1
2
3
OD550

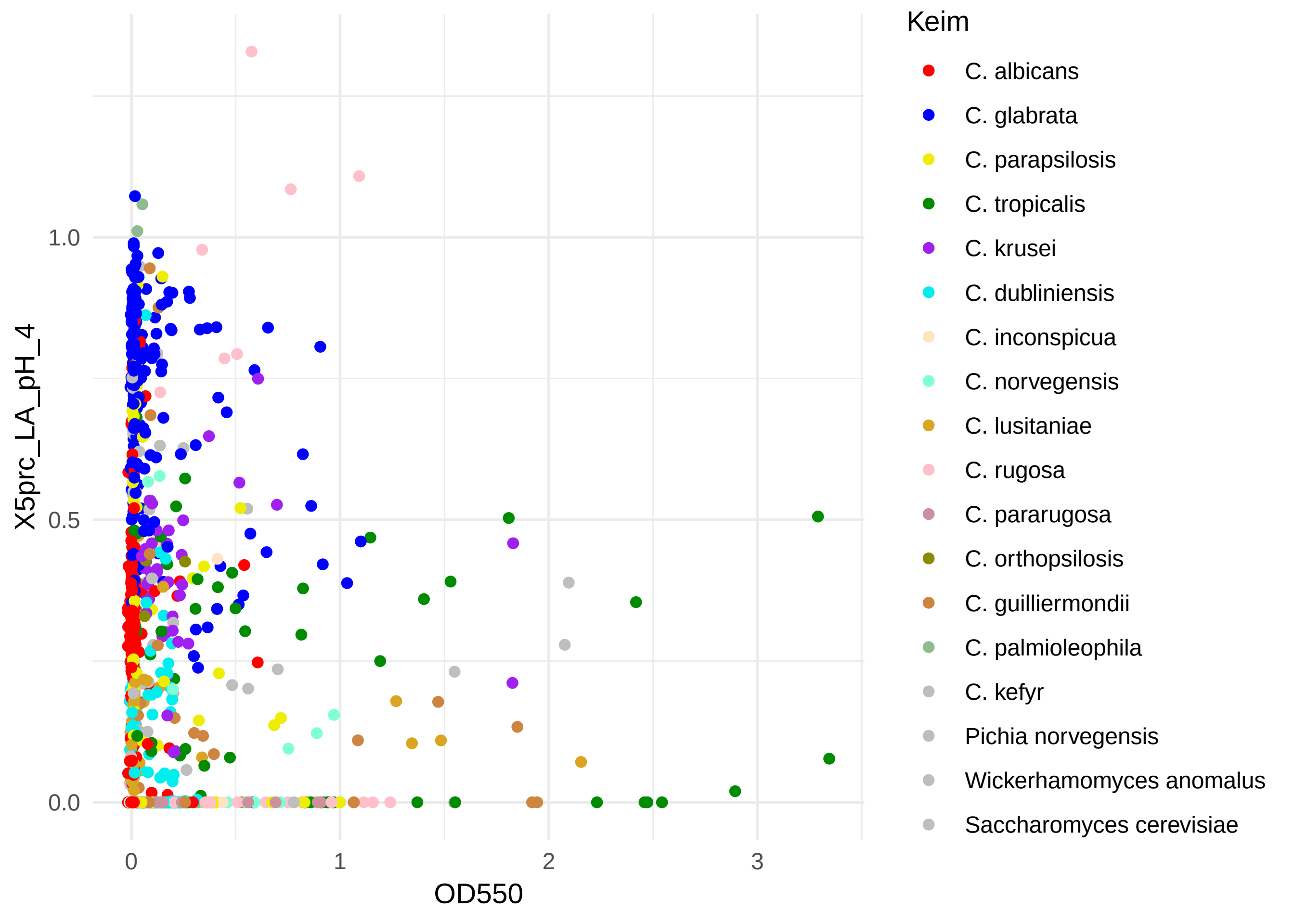
Keim
C. albicans
C. glabrata
C. parapsilosis
C. tropicalis
C. krusei
C. dubliniensis
C. inconspicua
C. norvegensis
C. lusitaniae
C. rugosa
C. pararugosa
C. orthopsilosis
C. guilliermondii
C. palmioleophila
C. kefyr
Pichia norvegensis
Wickerhamomyces anomalus
Saccharomyces cerevisiae
X5prc_LA_pH_4
1.0
0.5
0.0
0
1
2
3
OD550

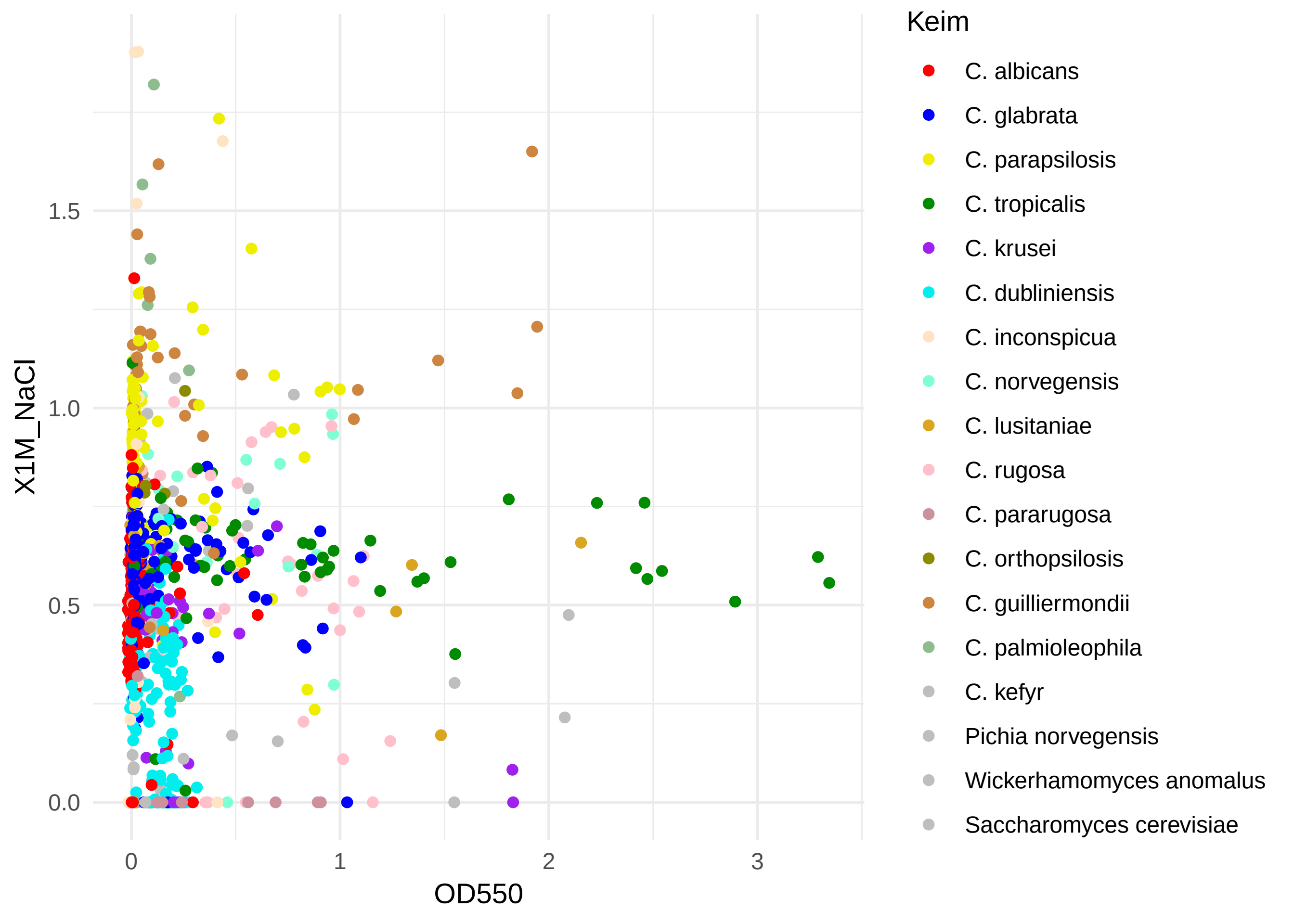

Keim
C. albicans
C. glabrata
C. parapsilosis
C. tropicalis
C. krusei
C. dubliniensis
C. inconspicua
C. norvegensis
C. lusitaniae
C. rugosa
C. pararugosa
C. orthopsilosis
C. guilliermondii
C. palmioleophila
C. kefyr
Pichia norvegensis
Wickerhamomyces anomalus
Saccharomyces cerevisiae
X1M_NaCl
1.5
1.0
0.5
0.0
0
1
2
3
OD550

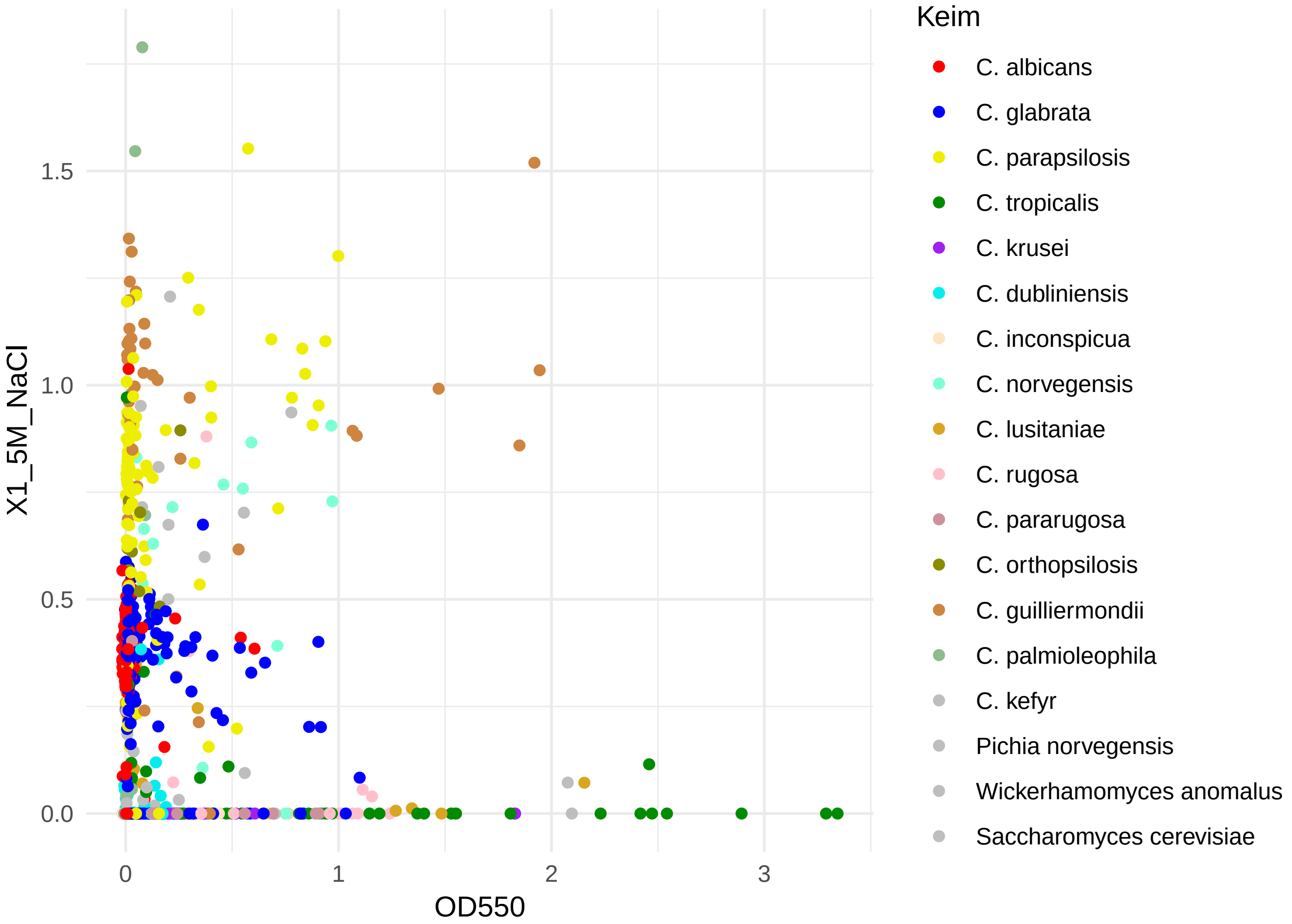
Keim
C. albicans
C. glabrata
C. parapsilosis
C. tropicalis
C. krusei
C. dubliniensis
C. inconspicua
C. norvegensis
C. lusitaniae
C. rugosa
C. pararugosa
C. orthopsilosis
C. guilliermondii
C. palmioleophila
C. kefyr
Pichia norvegensis
Wickerhamomyces anomalus
Saccharomyces cerevisiae
X1_5M_NaCl
OD550
0.0
0.5
1.0
1.5
0
1
2
3

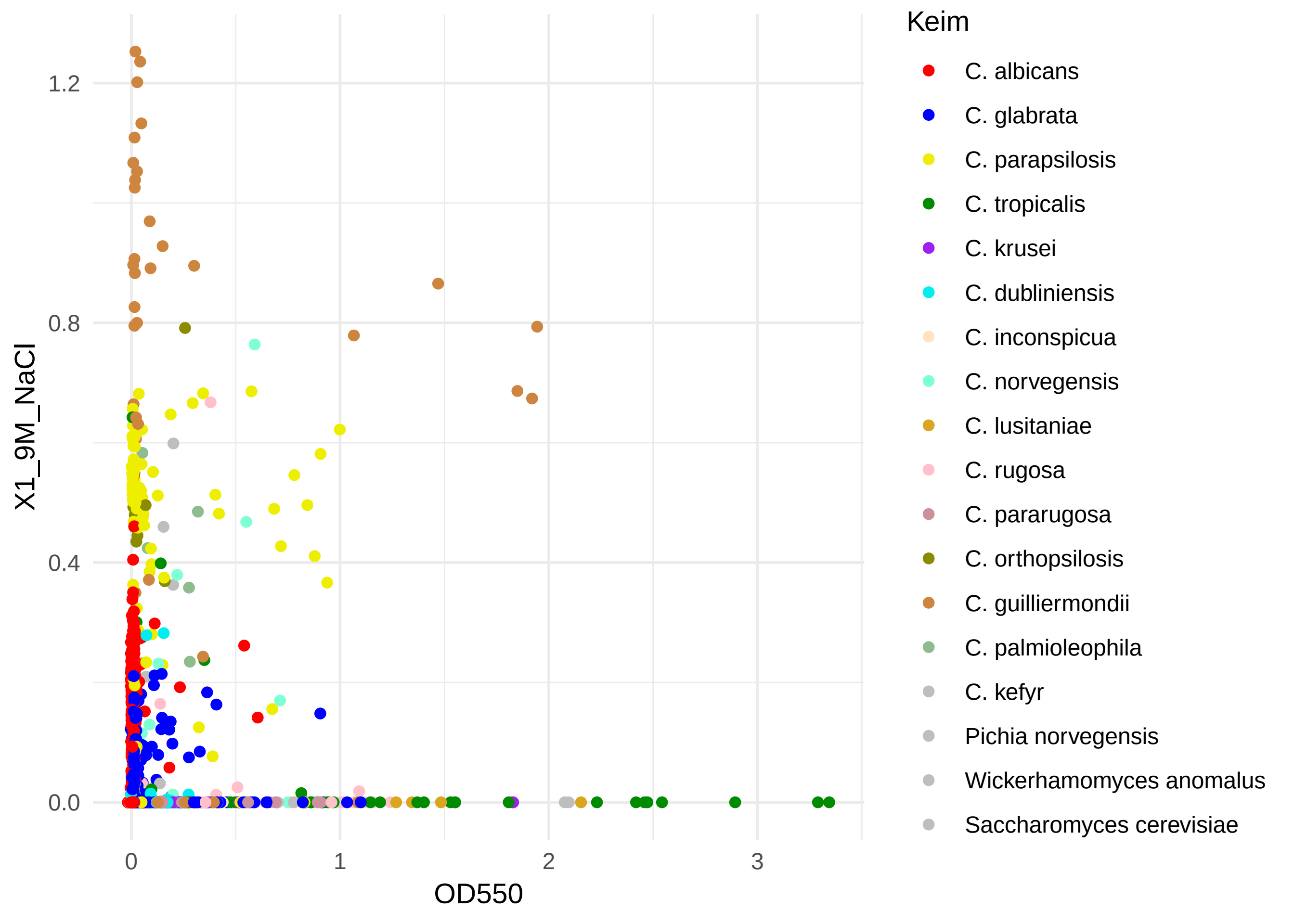

Keim
C. albicans
C. glabrata
C. parapsilosis
C. tropicalis
C. krusei
C. dubliniensis
C. inconspicua
C. norvegensis
C. lusitaniae
C. rugosa
C. pararugosa
C. orthopsilosis
C. guilliermondii
C. palmioleophila
C. kefyr
Pichia norvegensis
Wickerhamomyces anomalus
Saccharomyces cerevisiae
X1_9M_NaCl
1.2
0.8
0.4
0.0
0
1
2
3
OD550

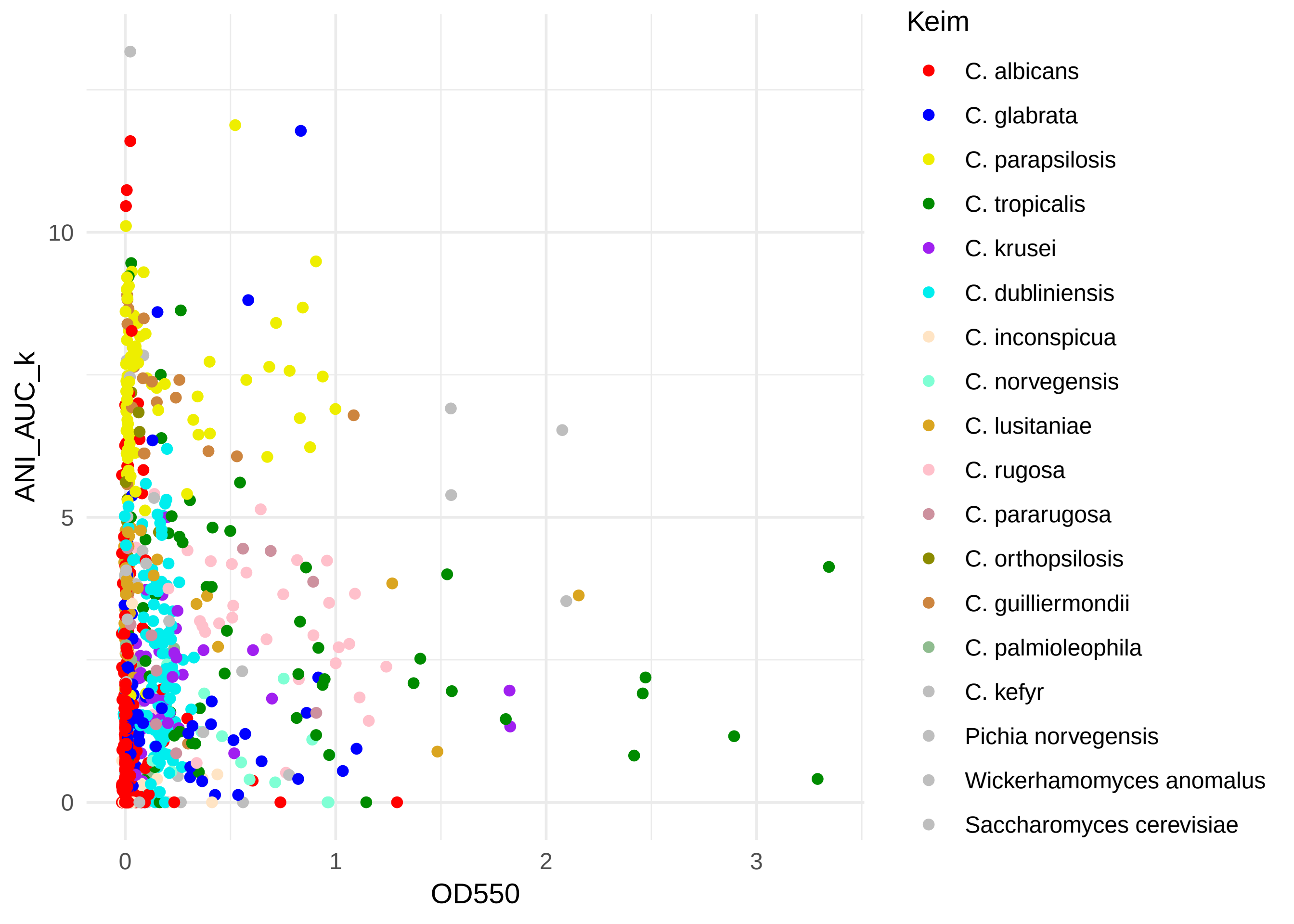

Keim
C. albicans
C. glabrata
C. parapsilosis
C. tropicalis
C. krusei
C. dubliniensis
C. inconspicua
C. norvegensis
C. lusitaniae
C. rugosa
C. pararugosa
C. orthopsilosis
C. guilliermondii
C. palmioleophila
C. kefyr
Pichia norvegensis
Wickerhamomyces anomalus
Saccharomyces cerevisiae
ANI_AUC_k
OD550
0
5
10
0
1
2
3

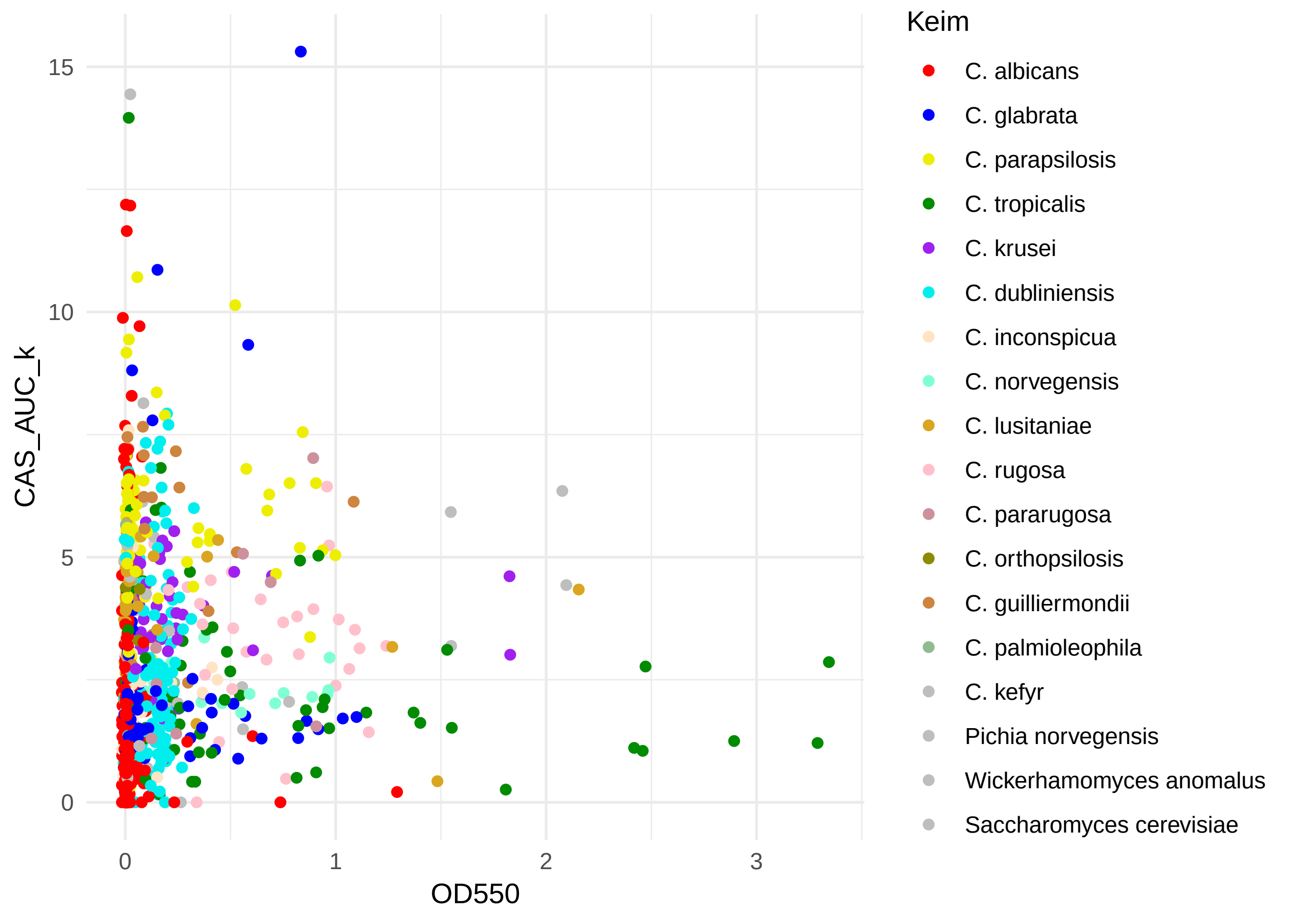

CAS_AUC_k
15
10
5
0
0
1
2
3
OD550
Keim
C. albicans
C. glabrata
C. parapsilosis
C. tropicalis
C. krusei
C. dubliniensis
C. inconspicua
C. norvegensis
C. lusitaniae
C. rugosa
C. pararugosa
C. orthopsilosis
C. guilliermondii
C. palmioleophila
C. kefyr
Pichia norvegensis
Wickerhamomyces anomalus
Saccharomyces cerevisiae

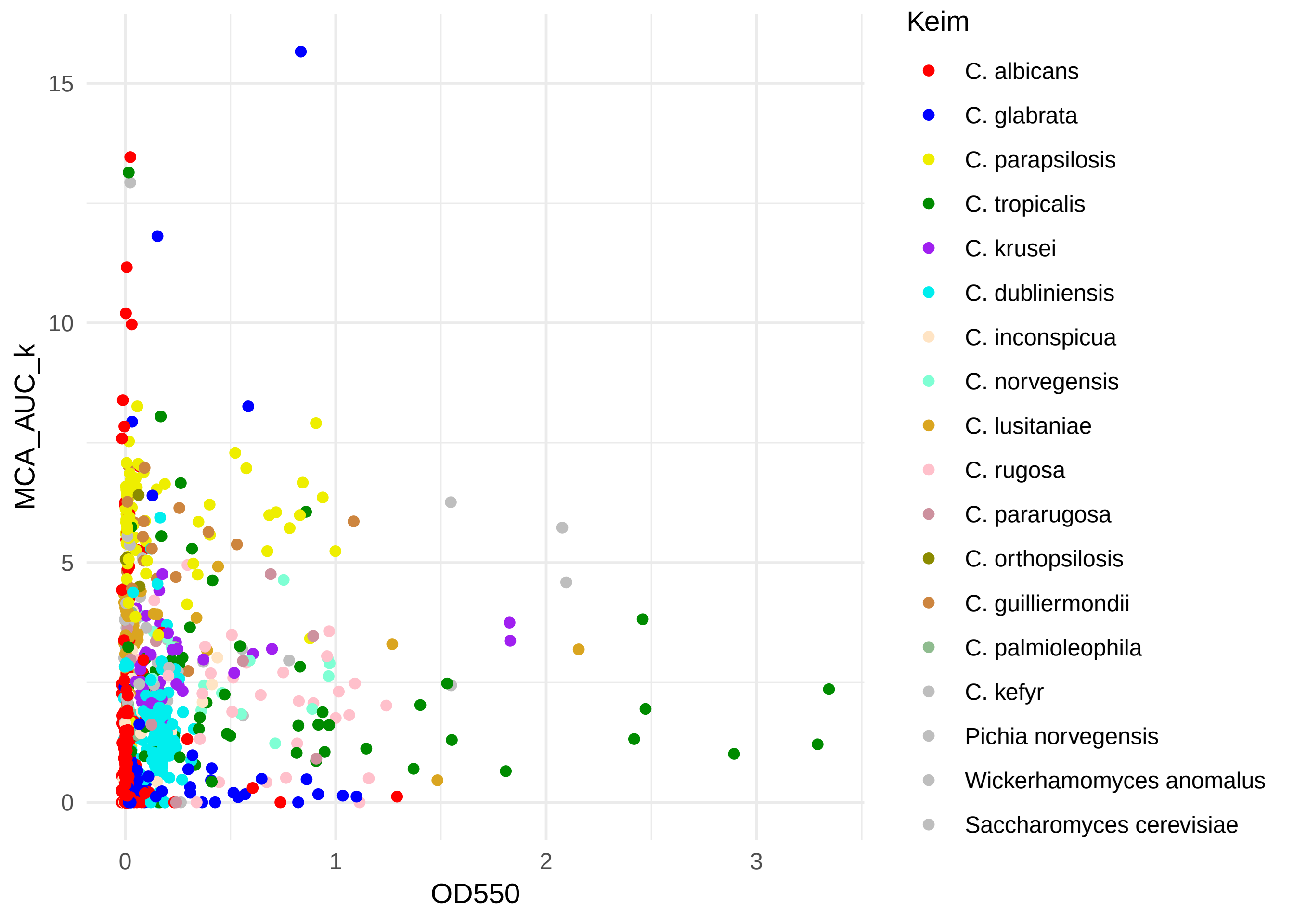
Keim
C. albicans
C. glabrata
C. parapsilosis
C. tropicalis
C. krusei
C. dubliniensis
C. inconspicua
C. norvegensis
C. lusitaniae
C. rugosa
C. pararugosa
C. orthopsilosis
C. guilliermondii
C. palmioleophila
C. kefyr
Pichia norvegensis
Wickerhamomyces anomalus
Saccharomyces cerevisiae
MCA_AUC_k
OD550
0
5
10
15
0
1
2
3

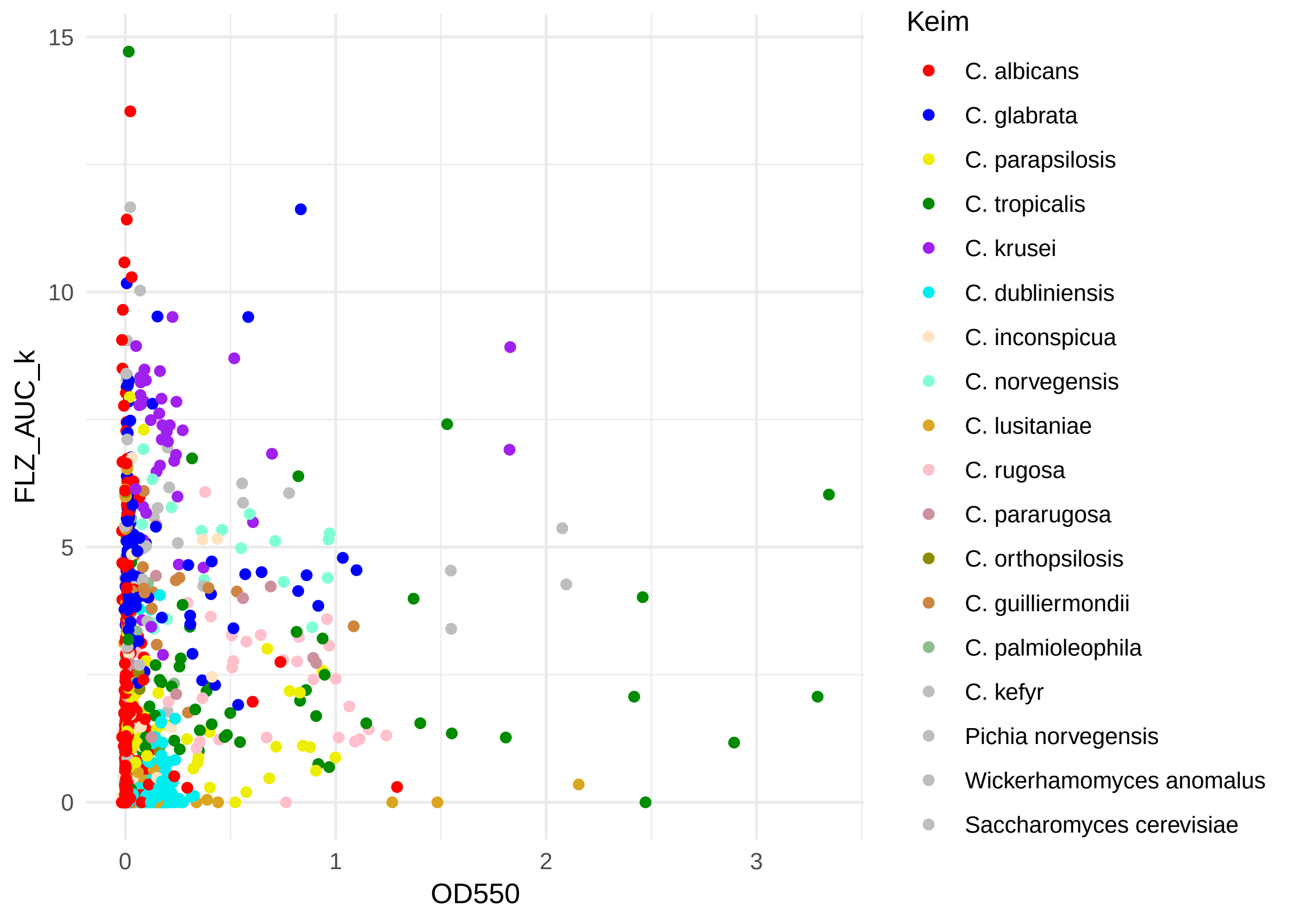
Keim
C. albicans
C. glabrata
C. parapsilosis
C. tropicalis
C. krusei
C. dubliniensis
C. inconspicua
C. norvegensis
C. lusitaniae
C. rugosa
C. pararugosa
C. orthopsilosis
C. guilliermondii
C. palmioleophila
C. kefyr
Pichia norvegensis
Wickerhamomyces anomalus
Saccharomyces cerevisiae
FLZ_AUC_k
OD550
0
5
10
15
0
1
2
3

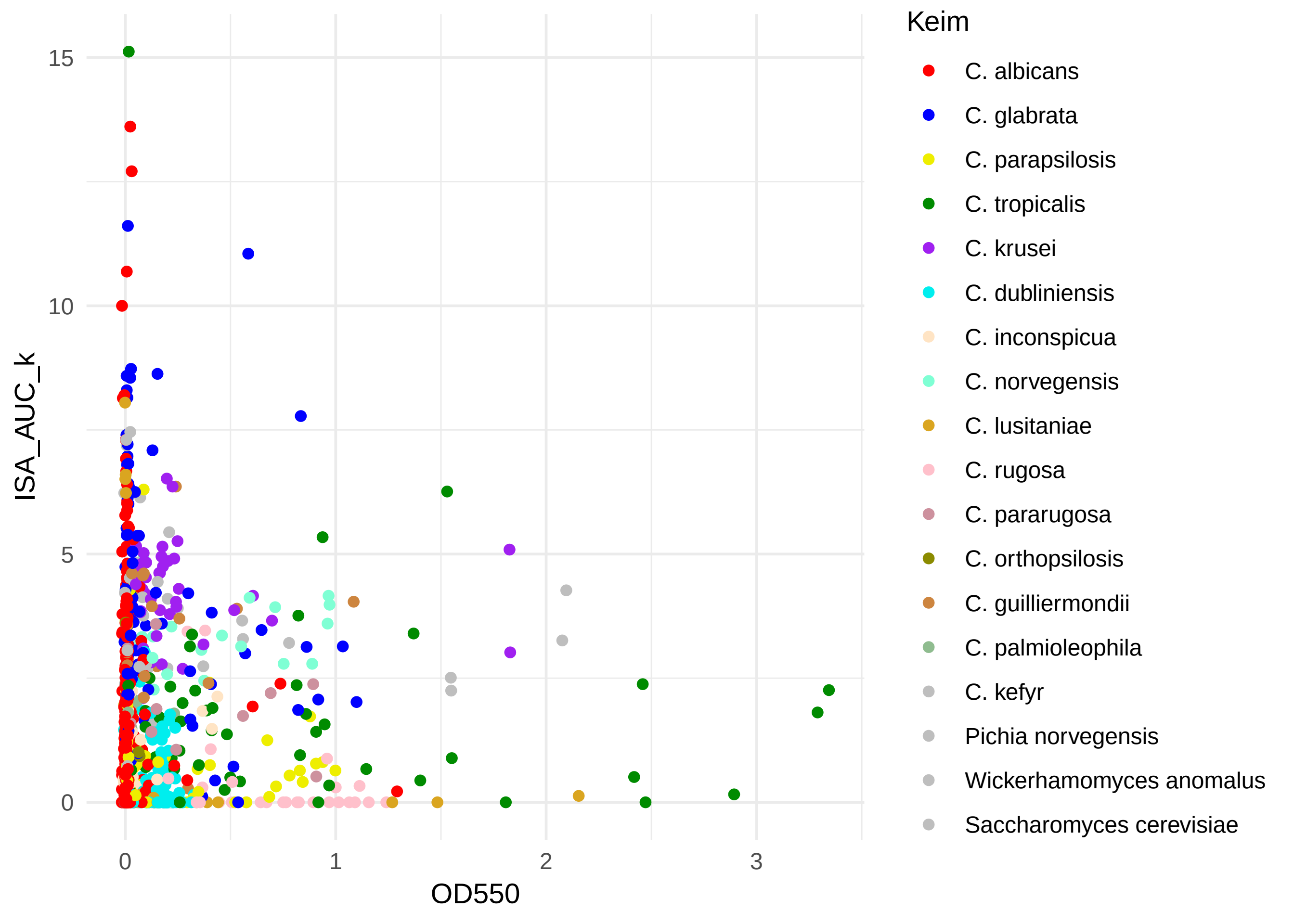
Keim
C. albicans
C. glabrata
C. parapsilosis
C. tropicalis
C. krusei
C. dubliniensis
C. inconspicua
C. norvegensis
C. lusitaniae
C. rugosa
C. pararugosa
C. orthopsilosis
C. guilliermondii
C. palmioleophila
C. kefyr
Pichia norvegensis
Wickerhamomyces anomalus
Saccharomyces cerevisiae
ISA_AUC_k
15
10
5
0
0
1
2
3
OD550

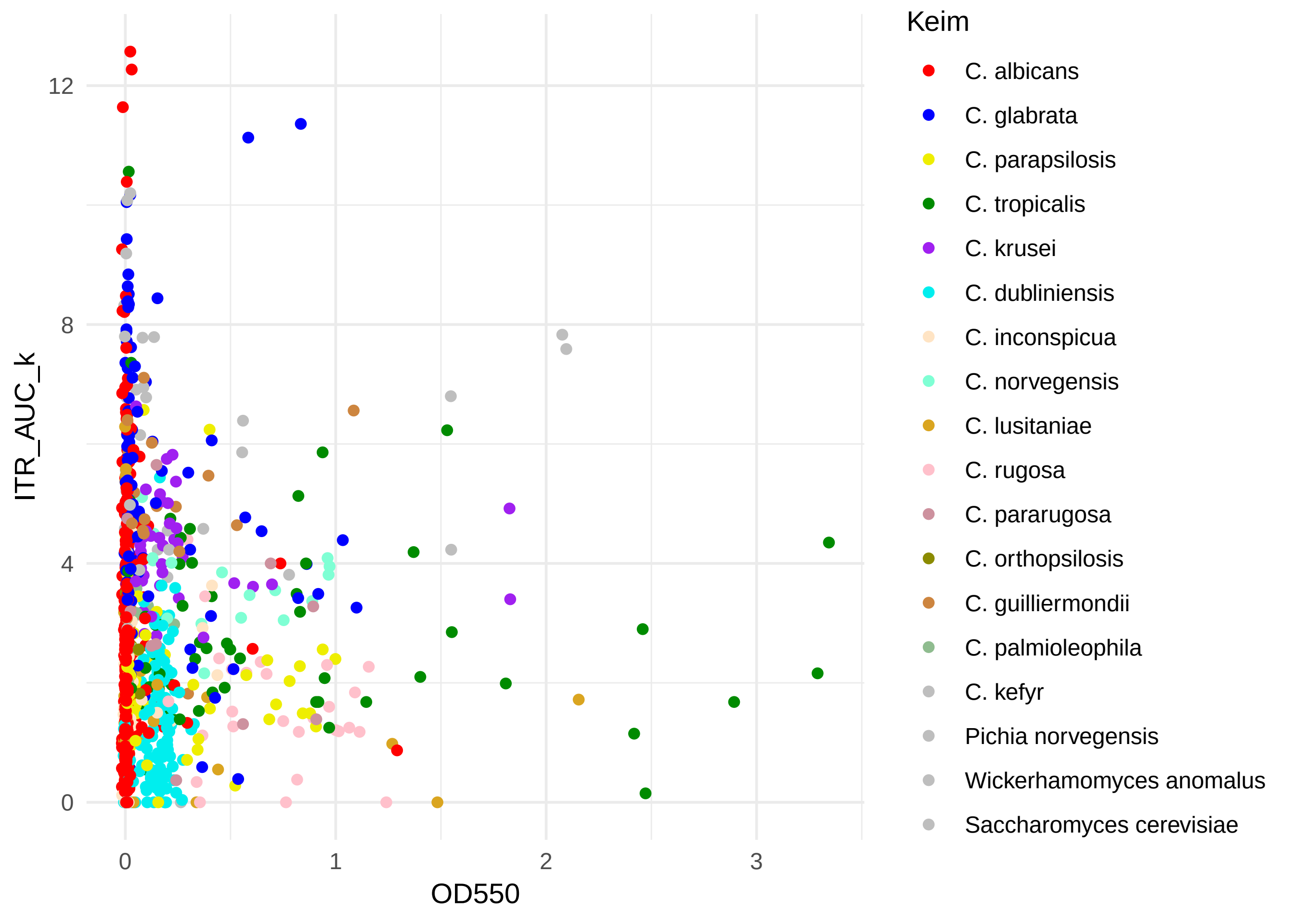
Keim
C. albicans
C. glabrata
C. parapsilosis
C. tropicalis
C. krusei
C. dubliniensis
C. inconspicua
C. norvegensis
C. lusitaniae
C. rugosa
C. pararugosa
C. orthopsilosis
C. guilliermondii
C. palmioleophila
C. kefyr
Pichia norvegensis
Wickerhamomyces anomalus
Saccharomyces cerevisiae
ITR_AUC_k
OD550
0
4
8
12
0
1
2
3

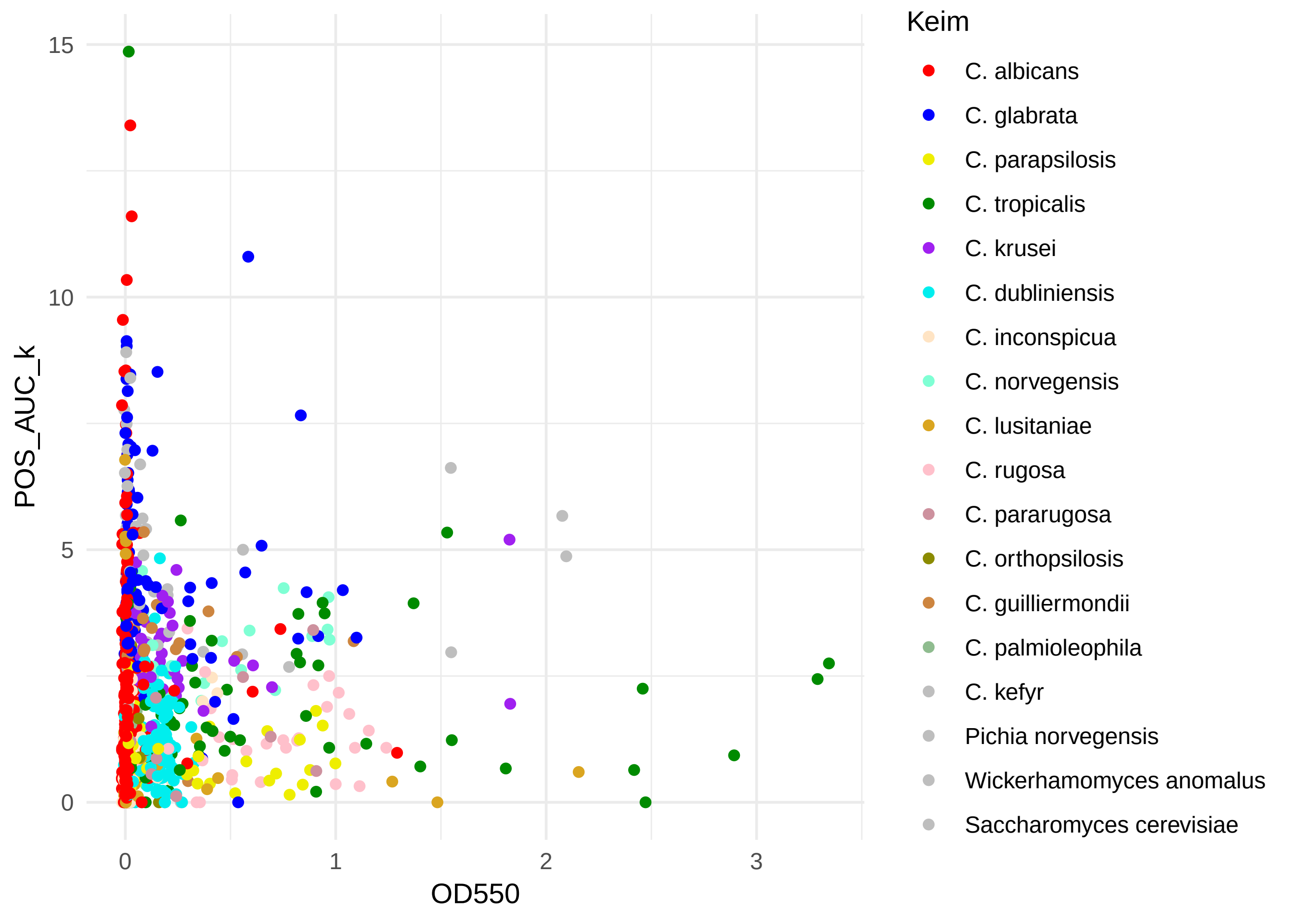

POS_AUC_k
OD550
15
10
5
0
0
1
2
3
Keim
C. albicans
C. glabrata
C. parapsilosis
C. tropicalis
C. krusei
C. dubliniensis
C. inconspicua
C. norvegensis
C. lusitaniae
C. rugosa
C. pararugosa
C. orthopsilosis
C. guilliermondii
C. palmioleophila
C. kefyr
Pichia norvegensis
Wickerhamomyces anomalus
Saccharomyces cerevisiae

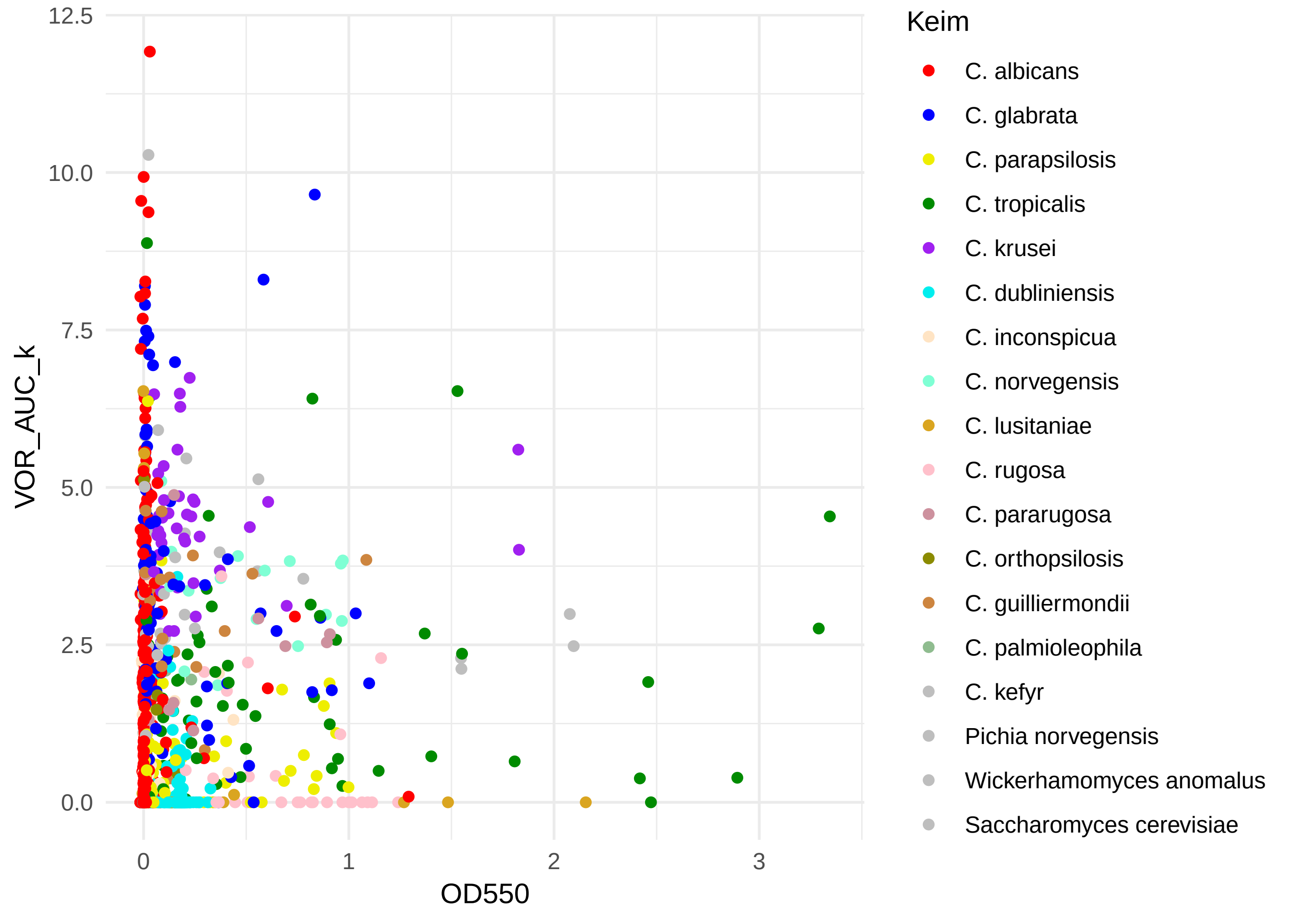
12.5
10.0
7.5
5.0
2.5
0.0
VOR_AUC_k
0
1
2
3
OD550
Keim
C. albicans
C. glabrata
C. parapsilosis
C. tropicalis
C. krusei
C. dubliniensis
C. inconspicua
C. norvegensis
C. lusitaniae
C. rugosa
C. pararugosa
C. orthopsilosis
C. guilliermondii
C. palmioleophila
C. kefyr
Pichia norvegensis
Wickerhamomyces anomalus
Saccharomyces cerevisiae

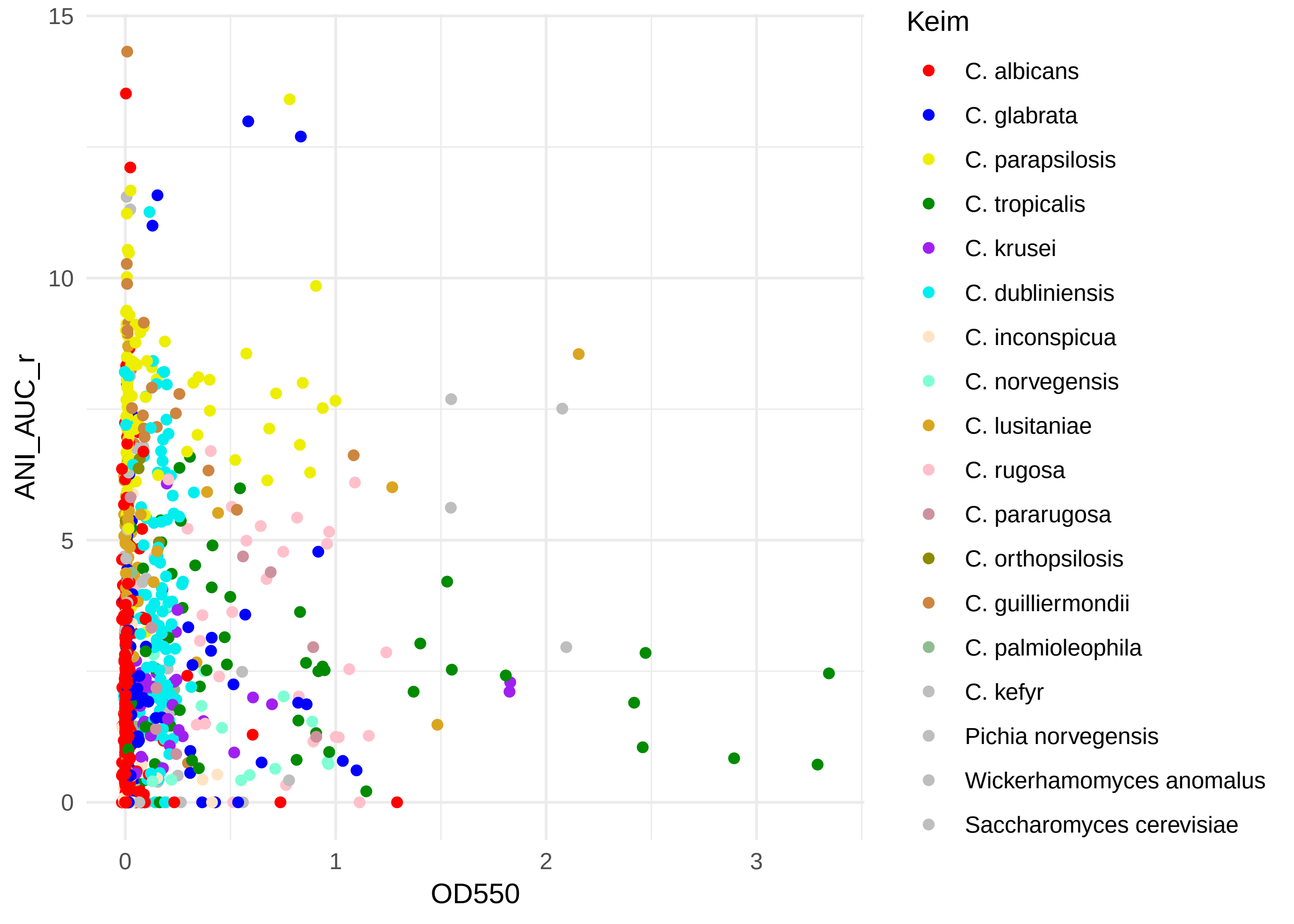

15
10
5
0
ANI_AUC_r
0
1
2
3
OD550
Keim
C. albicans
C. glabrata
C. parapsilosis
C. tropicalis
C. krusei
C. dubliniensis
C. inconspicua
C. norvegensis
C. lusitaniae
C. rugosa
C. pararugosa
C. orthopsilosis
C. guilliermondii
C. palmioleophila
C. kefyr
Pichia norvegensis
Wickerhamomyces anomalus
Saccharomyces cerevisiae

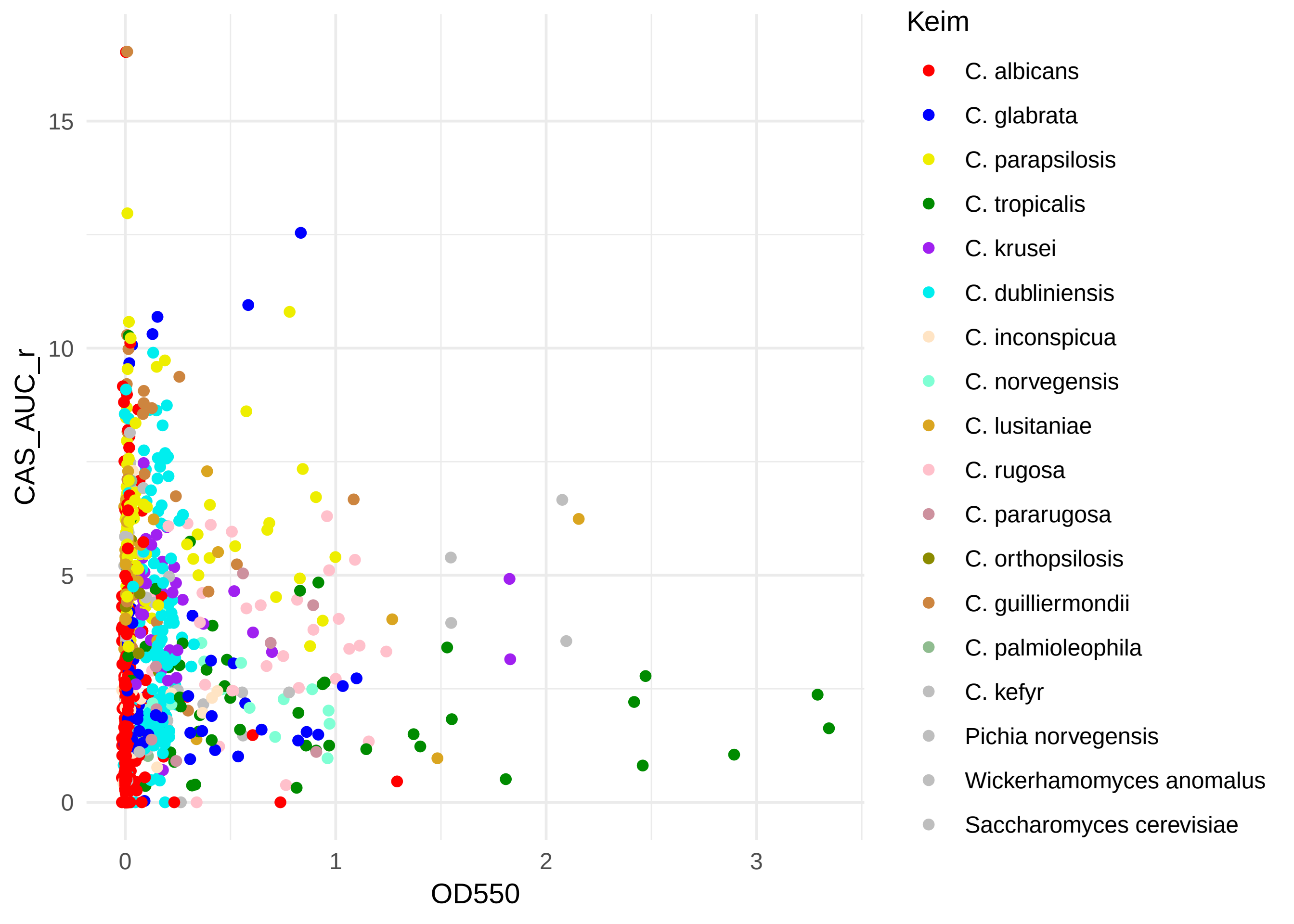

CAS_AUC_r
15
10
5
0
0
1
2
3
OD550
Keim
C. albicans
C. glabrata
C. parapsilosis
C. tropicalis
C. krusei
C. dubliniensis
C. inconspicua
C. norvegensis
C. lusitaniae
C. rugosa
C. pararugosa
C. orthopsilosis
C. guilliermondii
C. palmioleophila
C. kefyr
Pichia norvegensis
Wickerhamomyces anomalus
Saccharomyces cerevisiae

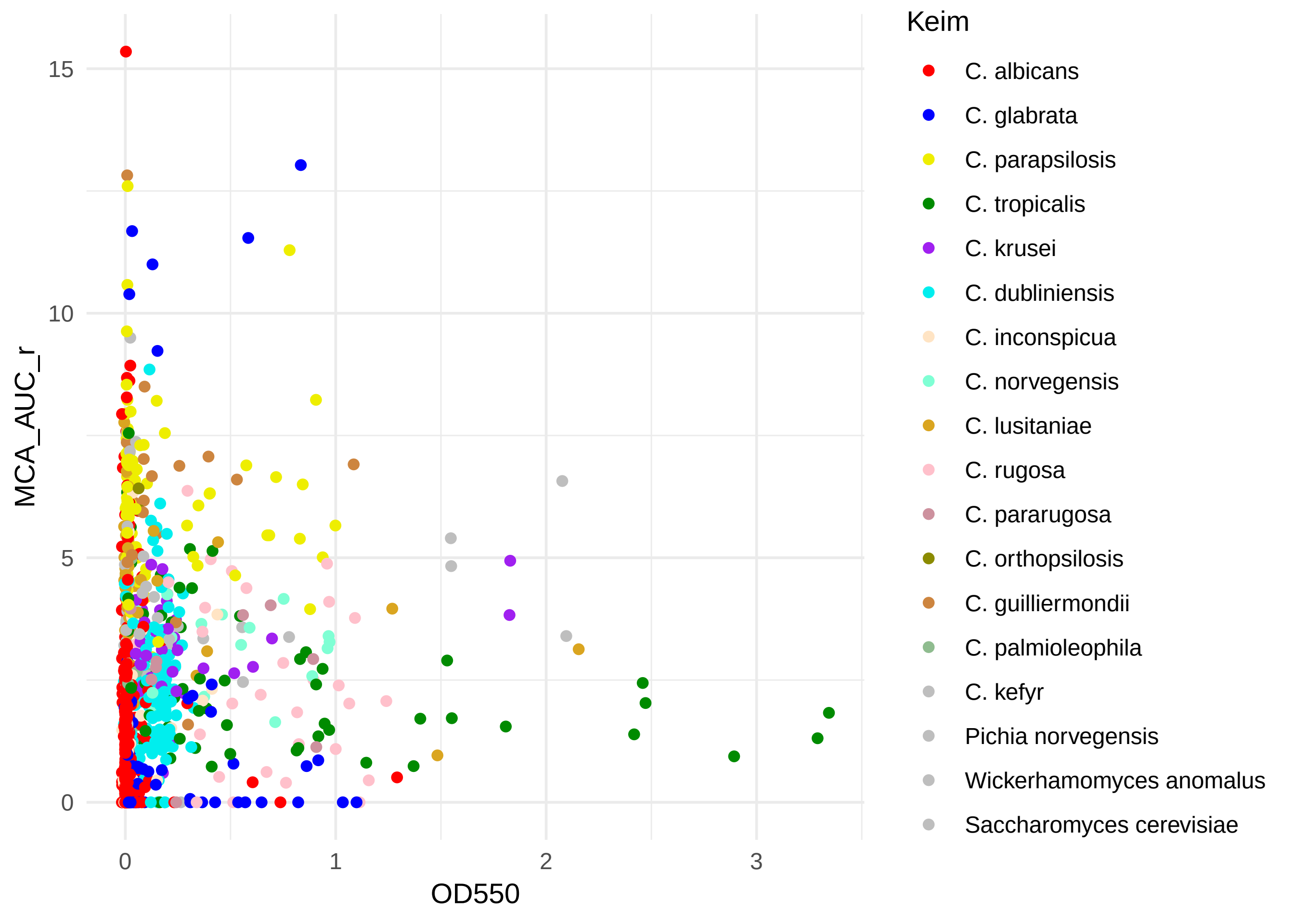

Keim
C. albicans
C. glabrata
C. parapsilosis
C. tropicalis
C. krusei
C. dubliniensis
C. inconspicua
C. norvegensis
C. lusitaniae
C. rugosa
C. pararugosa
C. orthopsilosis
C. guilliermondii
C. palmioleophila
C. kefyr
Pichia norvegensis
Wickerhamomyces anomalus
Saccharomyces cerevisiae
MCA_AUC_r
OD550
15
10
5
0
0
1
2
3

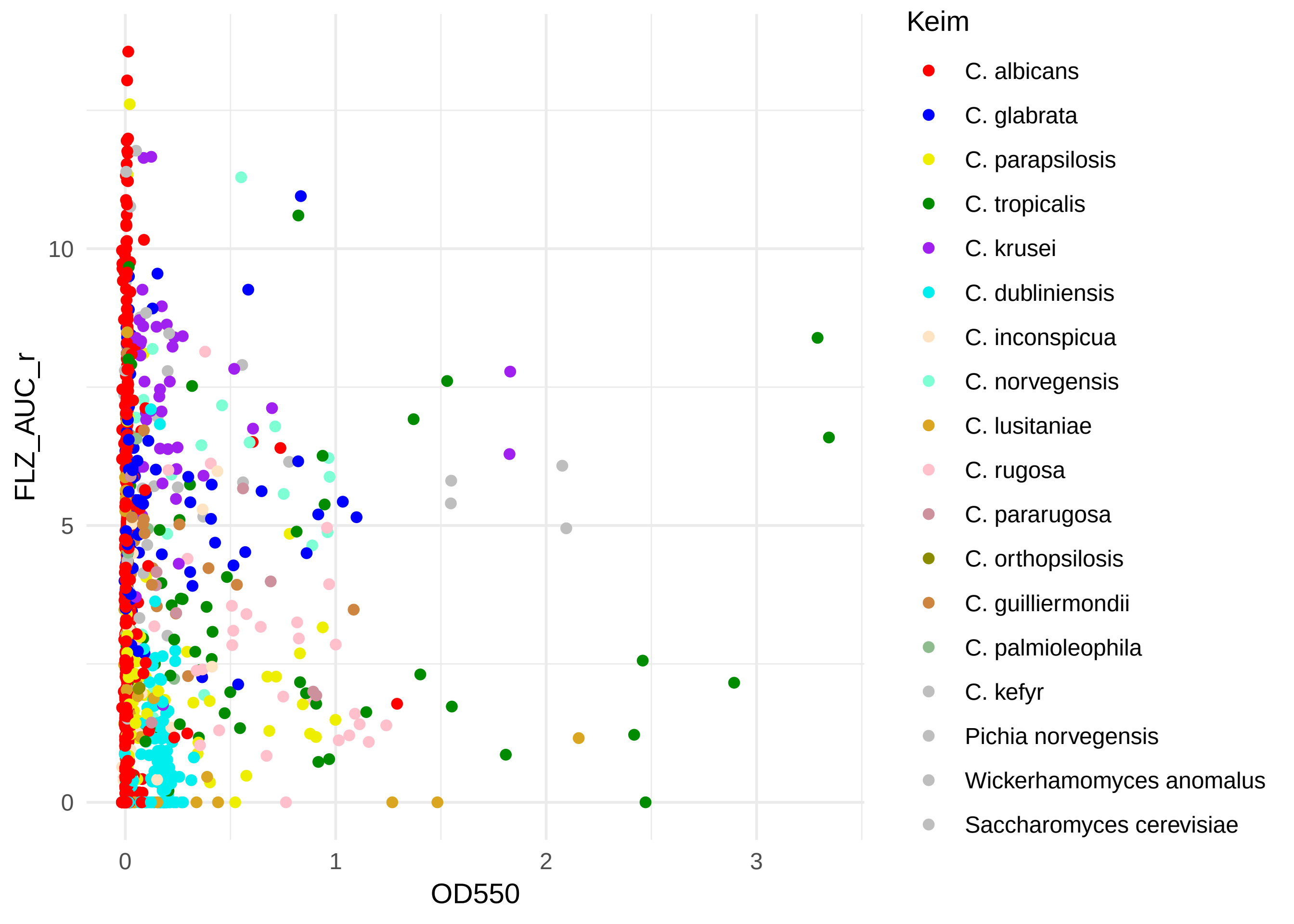
Keim
C. albicans
C. glabrata
C. parapsilosis
C. tropicalis
C. krusei
C. dubliniensis
C. inconspicua
C. norvegensis
C. lusitaniae
C. rugosa
C. pararugosa
C. orthopsilosis
C. guilliermondii
C. palmioleophila
C. kefyr
Pichia norvegensis
Wickerhamomyces anomalus
Saccharomyces cerevisiae
FLZ_AUC_r
OD550
0
5
10
0
1
2
3

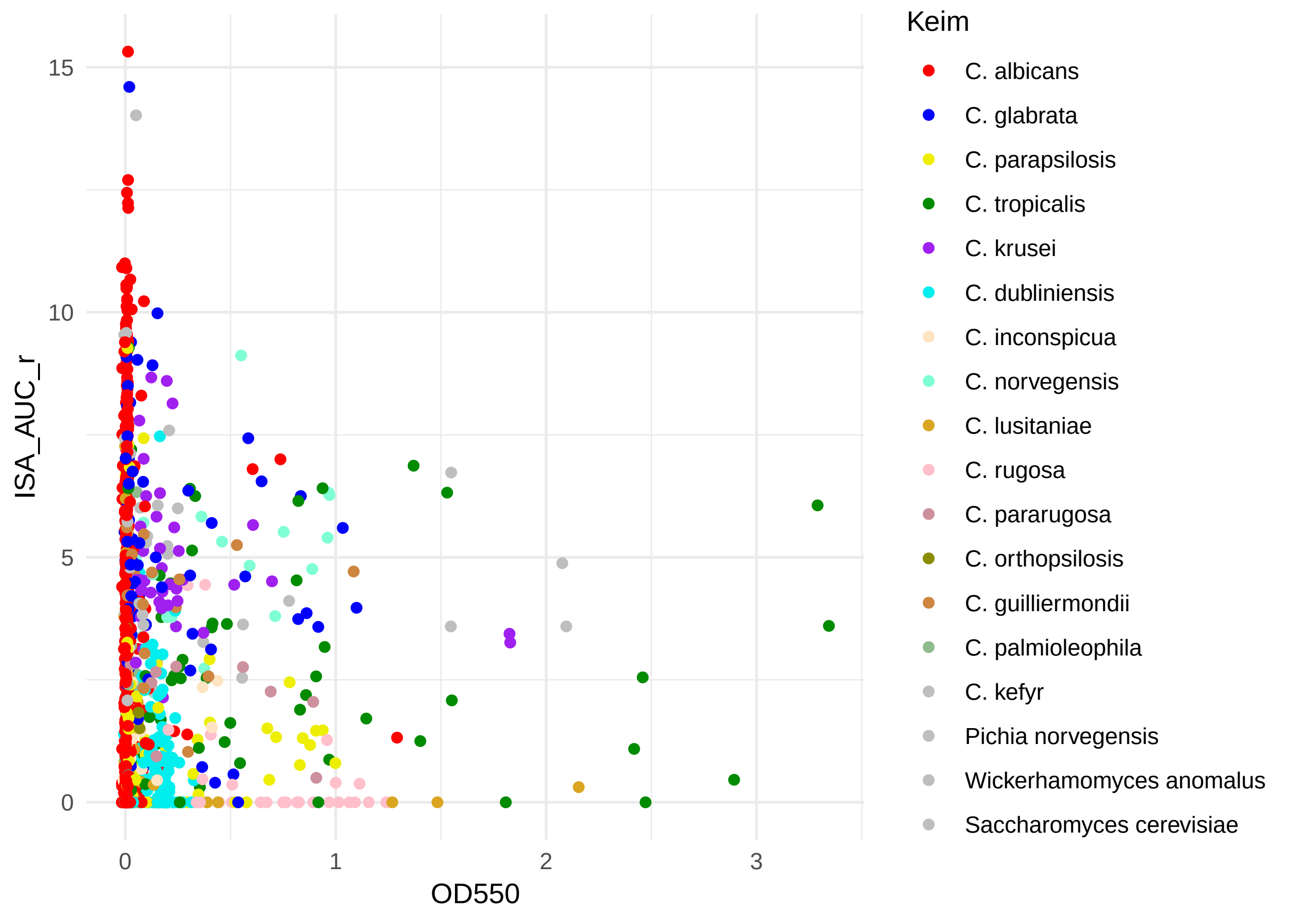

Keim
C. albicans
C. glabrata
C. parapsilosis
C. tropicalis
C. krusei
C. dubliniensis
C. inconspicua
C. norvegensis
C. lusitaniae
C. rugosa
C. pararugosa
C. orthopsilosis
C. guilliermondii
C. palmioleophila
C. kefyr
Pichia norvegensis
Wickerhamomyces anomalus
Saccharomyces cerevisiae
ISA_AUC_r
OD550
15
10
5
0
0
1
2
3

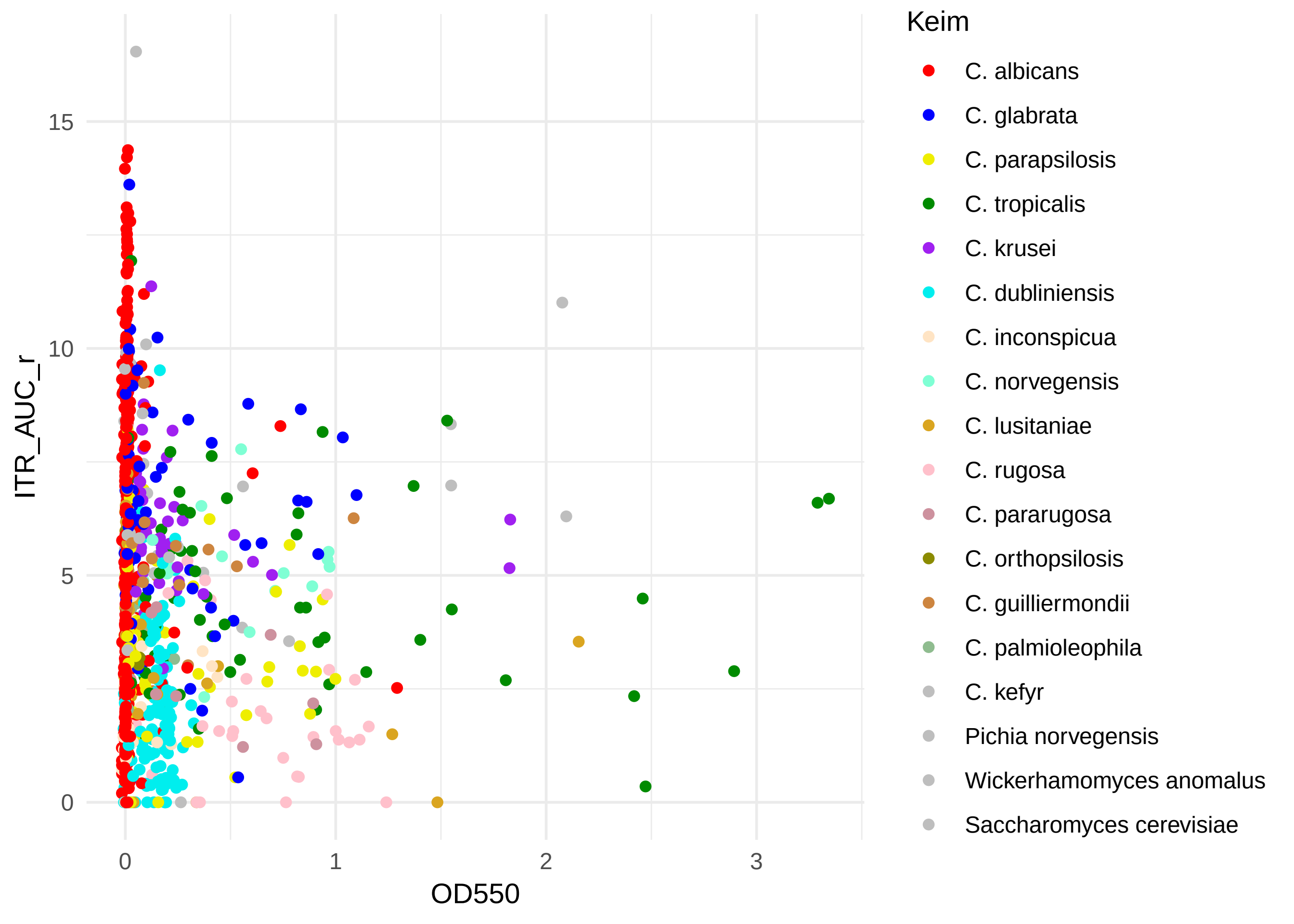

Keim
C. albicans
C. glabrata
C. parapsilosis
C. tropicalis
C. krusei
C. dubliniensis
C. inconspicua
C. norvegensis
C. lusitaniae
C. rugosa
C. pararugosa
C. orthopsilosis
C. guilliermondii
C. palmioleophila
C. kefyr
Pichia norvegensis
Wickerhamomyces anomalus
Saccharomyces cerevisiae
ITR_AUC_r
15
10
5
0
OD550
0
1
2
3

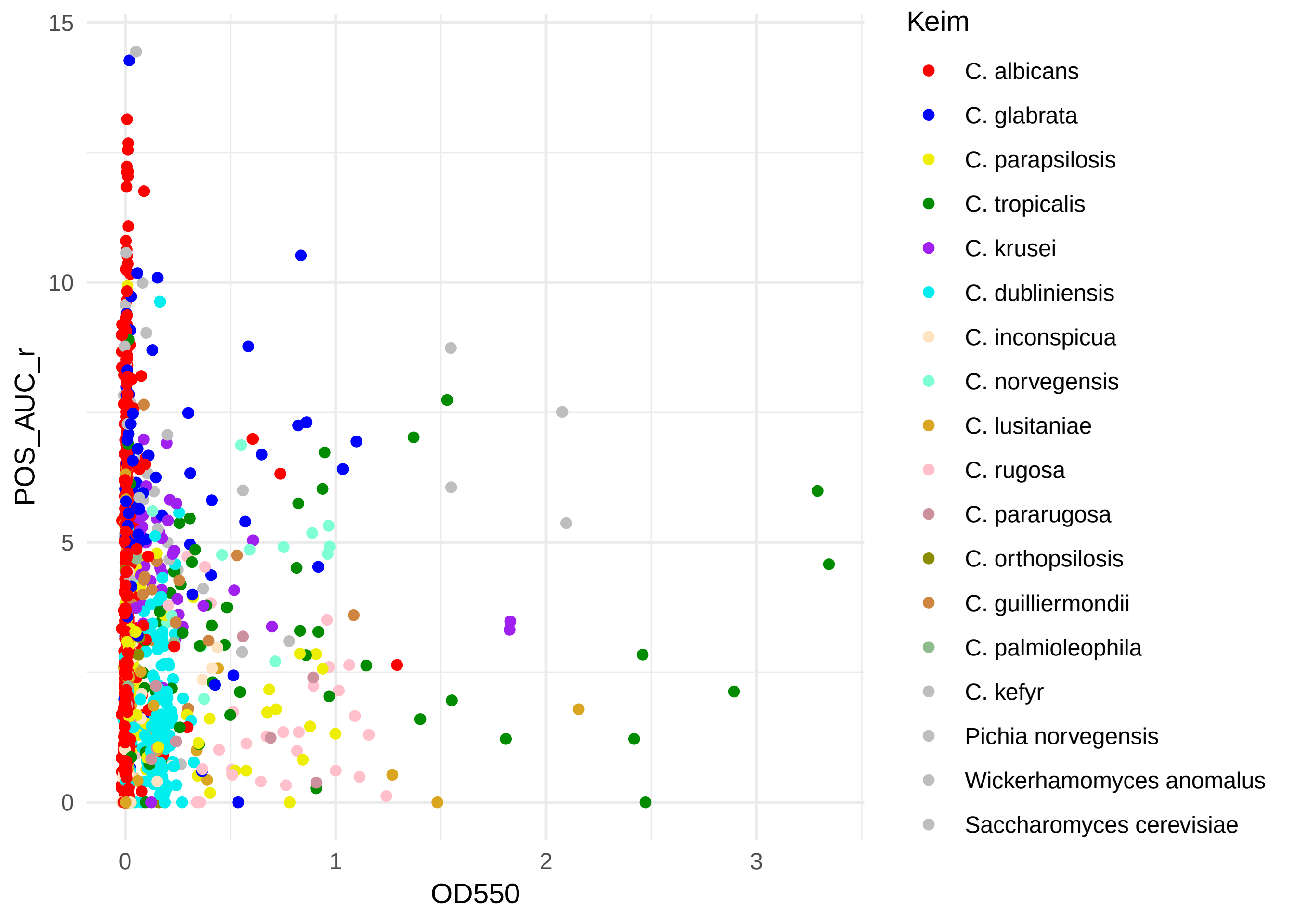

Keim
C. albicans
C. glabrata
C. parapsilosis
C. tropicalis
C. krusei
C. dubliniensis
C. inconspicua
C. norvegensis
C. lusitaniae
C. rugosa
C. pararugosa
C. orthopsilosis
C. guilliermondii
C. palmioleophila
C. kefyr
Pichia norvegensis
Wickerhamomyces anomalus
Saccharomyces cerevisiae
POS_AUC_r
OD550
15
10
5
0
0
1
2
3

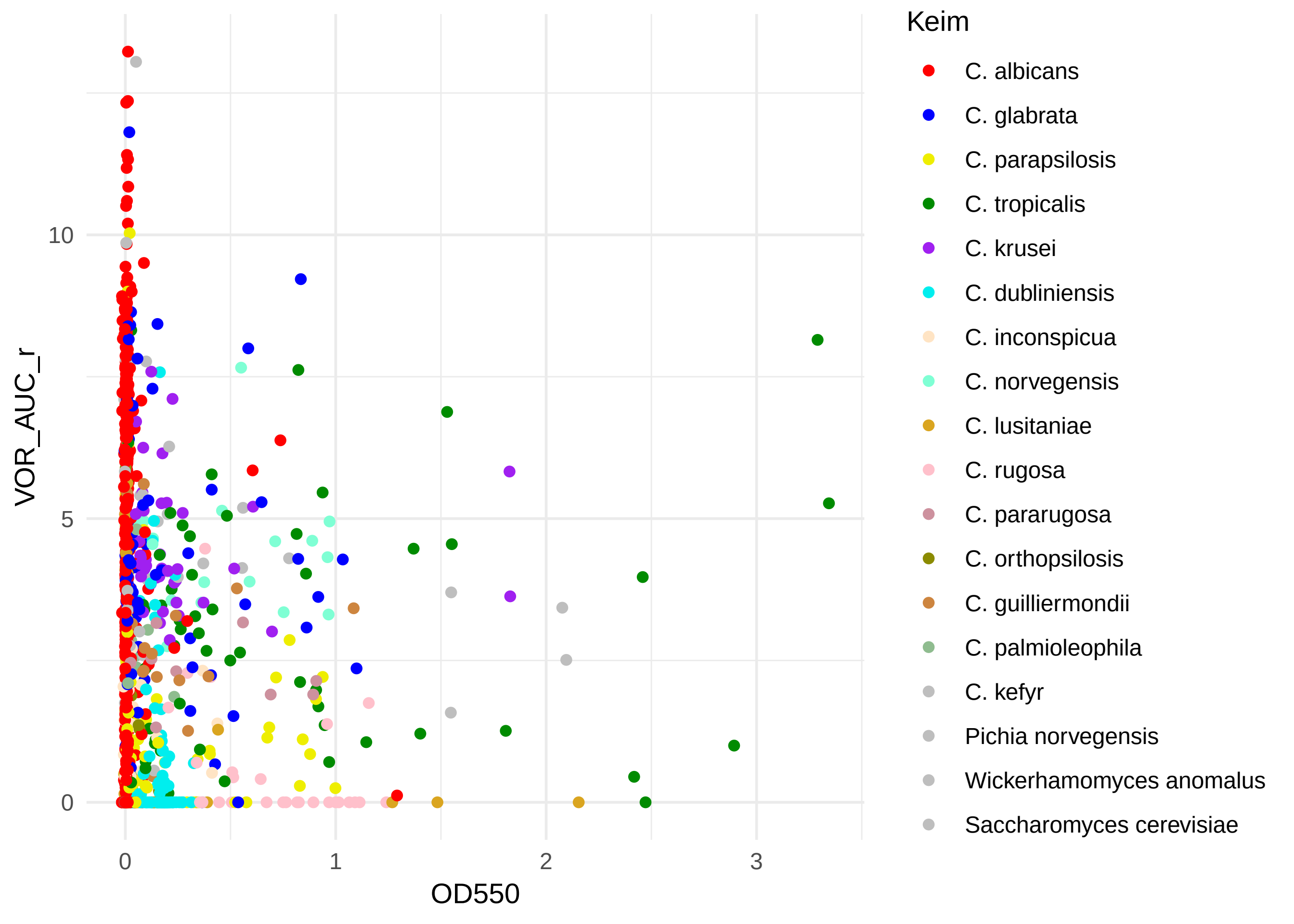

Keim
C. albicans
C. glabrata
C. parapsilosis
C. tropicalis
C. krusei
C. dubliniensis
C. inconspicua
C. norvegensis
C. lusitaniae
C. rugosa
C. pararugosa
C. orthopsilosis
C. guilliermondii
C. palmioleophila
C. kefyr
Pichia norvegensis
Wickerhamomyces anomalus
Saccharomyces cerevisiae
VOR_AUC_r
10
5
0
0
1
2
3
OD550

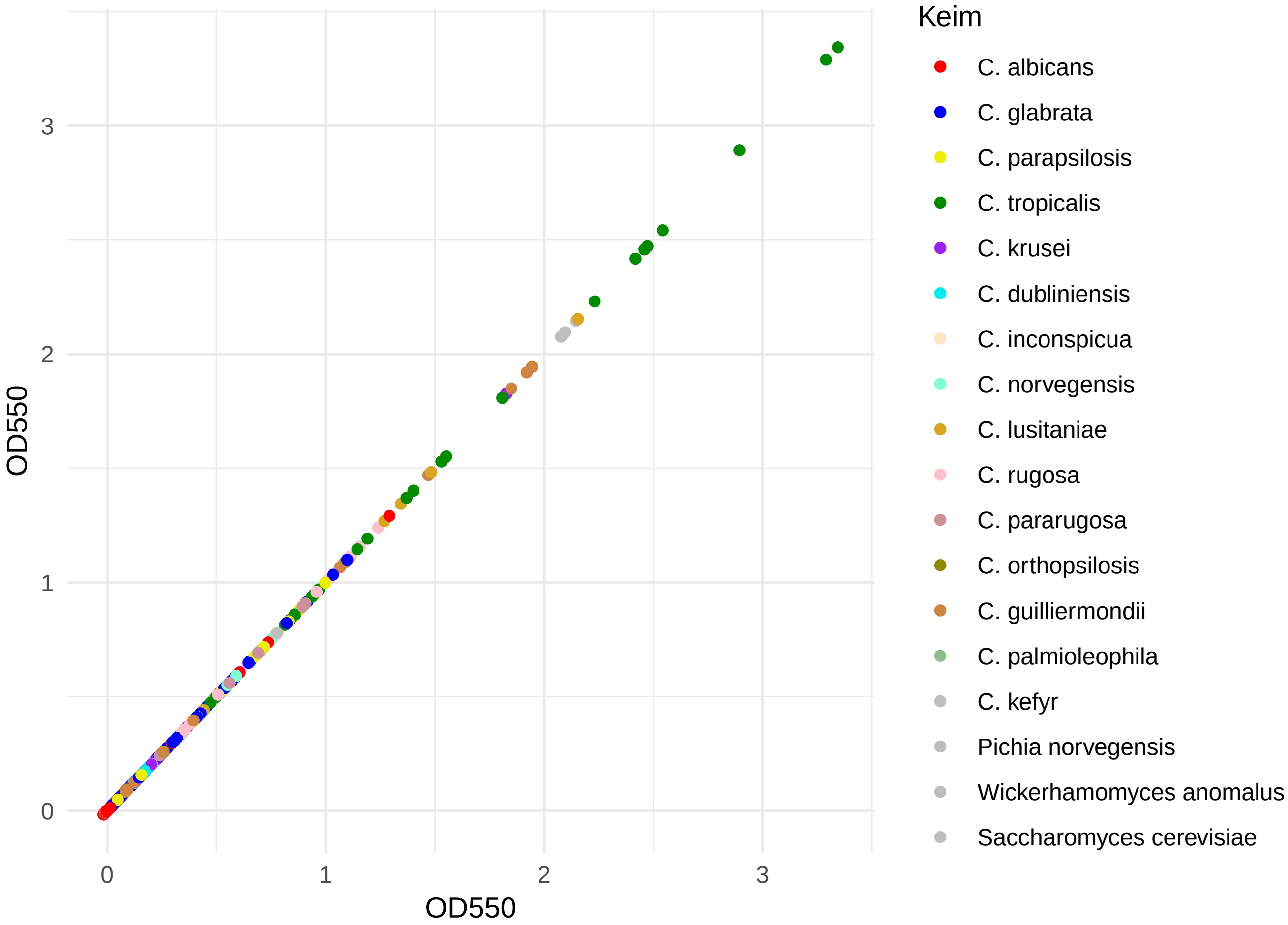

Keim
C. albicans
C. glabrata
C. parapsilosis
C. tropicalis
C. krusei
C. dubliniensis
C. inconspicua
C. norvegensis
C. lusitaniae
C. rugosa
C. pararugosa
C. orthopsilosis
C. guilliermondii
C. palmioleophila
C. kefyr
Pichia norvegensis
Wickerhamomyces anomalus
Saccharomyces cerevisiae
OD550
OD550
0
1
2
3
0
1
2
3
